# Chromosome 19 microRNA cluster enhances cell reprogramming by inhibiting epithelial-to-mesenchymal transition

**DOI:** 10.1038/s41598-020-59812-8

**Published:** 2020-02-20

**Authors:** Ezinne F. Mong, Ying Yang, Kemal M. Akat, John Canfield, Jeffrey VanWye, John Lockhart, John C. M. Tsibris, Frederick Schatz, Charles J. Lockwood, Thomas Tuschl, Umit A. Kayisli, Hana Totary-Jain

**Affiliations:** 1 Department of Molecular Pharmacology and Physiology, University of South Florida, Morsani College of Medicine, Tampa, Florida USA; 2 Howard Hughes Medical Institute and Laboratory for RNA Molecular Biology, The Rockefeller University, New York, New York USA; 3 Department of Obstetrics and Gynecology, University of South Florida, Morsani College of Medicine, Tampa, Florida USA; 4 Present Address: Vanderbilt Heart and Vascular Institute, Vanderbilt University Medical Center, Nashville, TN USA

**Keywords:** Differentiation, Reprogramming

## Abstract

During implantation, cytotrophoblasts undergo epithelial-to-mesenchymal transition (EMT) as they differentiate into invasive extravillous trophoblasts (EVTs). The primate-specific microRNA cluster on chromosome 19 (C19MC) is exclusively expressed in the placenta, embryonic stem cells and certain cancers however, its role in EMT gene regulation is unknown. *In situ* hybridization for miR-517a/c, a C19MC cistron microRNA, in first trimester human placentas displayed strong expression in villous trophoblasts and a gradual decrease from proximal to distal cell columns as cytotrophoblasts differentiate into invasive EVTs. To investigate the role of C19MC in the regulation of EMT genes, we employed the CRISPR/dCas9 Synergistic Activation Mediator (SAM) system, which induced robust transcriptional activation of the entire C19MC cistron and resulted in suppression of EMT associated genes. Exposure of human iPSCs to hypoxia or differentiation of iPSCs into either cytotrophoblast-stem-like cells or EVT-like cells under hypoxia reduced C19MC expression and increased EMT genes. Furthermore, transcriptional activation of the C19MC cistron induced the expression of OCT4 and FGF4 and accelerated cellular reprogramming. This study establishes the CRISPR/dCas9 SAM as a powerful tool that enables activation of the entire C19MC cistron and uncovers its novel role in suppressing EMT genes critical for maintaining the epithelial cytotrophoblasts stem cell phenotype.

## Introduction

Human embryonic implantation into the uterus requires extensive coordinated attachment and invasion of the maternal endometrium by fetal trophoblasts. While in the fallopian tube, the developing embryo differentiates into the blastocyst, which consists of an inner cell mass, destined to become the fetus, and the trophectoderm, an outer layer of epithelial cells that eventually develops into the placenta^1^. Shortly before implantation, the highly mitotic cells derived from the trophectoderm – the cytotrophoblast (CTs) – differentiate into either multinucleated syncytiotrophoblast (STs) or extravillous trophoblast (EVTs). STs form the outer villous layer of the placenta and regulate maternal-fetal gas exchange, nutrient uptake and waste elimination. The interstitial EVTs invade the decidua and inner myometrium to anchor the chorionic villi to the decidua and uterine wall. Concurrently, endovascular EVTs penetrate the maternal spiral arteries and participate in remodeling them into high-flow, low-resistance vessels that facilitate placental perfusion to accommodate increasing O_2_ and nutrient demands by the developing fetus^2,3^.

In humans, EVT differentiation and invasion are crucial steps involved in implantation and placentation. These cellular processes are regulated by several molecular mechanisms, which have not been completely elucidated. Inadequate EVT invasion, so called shallow placentation, can elicit placental hypoperfusion resulting in pregnancy complications including fetal loss, preeclampsia and/or fetal growth restriction^4,5^. In contrast increased EVT invasion can lead to placenta accreta requiring pregnancy termination^6^. EVT differentiation involves extensive changes during which cells lose both their intercellular junctions and apical-basal polarity, while acquire the capacity for migration and invasion. These changes represent a prototypical example of the trans-differentiation process known as epithelial-to-mesenchymal transition (EMT)^7,8^. EMT is facilitated by transcription factors such as SNAI1, SNAI2 and TWIST1, which down-regulate epithelial markers and induce the expression of mesenchymal cell markers such as N-cadherin (also known as CDH2) and vimentin^9^.

MicroRNAs (miRNA) play a major role in the regulation of EMT^10,11^. Human CTs highly express a unique cluster of miRNAs located on chromosome 19 (C19MC)^12^. This miRNA cluster spans approximately 100 kb and produces 56 mature miRNAs^12^. Expression of C19MC is epigenetically controlled by imprinting with only the paternal allele expressed in the placenta^12^. Laser capture microdissection microscopy of human first trimester and term placentas revealed higher C19MC expression in villi-containing regions compared to EVT regions^13^. Furthermore, overexpression of C19MC in the human EVT-derived cell line HTR8/SVneo attenuated migration by regulating genes involved in cell motility, implying a role for C19MC in the regulation of EVT invasion^13^.

Embryonic stem cells also highly express C19MC^14^. Reprogramming of somatic cells into induced pluripotent stem cells (iPSCs) by ectopic expression of the OSKM (OCT4, SOX2, KLF4 and MYC) or the OSLN (OCT4, SOX2, LIN28 and NANOG) transcription factors^15–17^, induced the expression of C19MC and other stem-cell enriched miRNA clusters including miR-302/367, miR-17/92, miR-200 and miR-371-373 as well as genes involved in the mesenchymal-to-epithelial transition (MET)^14,18^. Overexpression of miR-524-5p, a member of C19MC, enhanced reprogramming efficiency when co-expressed with OSKM transcription factors^19^. Currently, the regulatory effects of the entire C19MC cluster on EMT and somatic cell reprogramming are not fully understood. Therefore, we sought to investigate the role of the entire C19MC on EMT, which is critical for trophoblast cell differentiation and invasion and somatic cell reprogramming.

Until recently, studying the functions of C19MC miRNAs has been challenging because of its large size. Employing Bacterial Artificial Chromosome (BAC) technology, which carries the entire C19MC locus including the upstream CpG island^12^ resulted in only modest up-regulation of some miRNAs of the C19MC^13,20^. To overcome these hurdles, we employed a novel approach using the CRISPR/dCas9 synergistic activating mediator (SAM) technology^21^ to transcriptionally activate the entire C19MC cistron in a highly specific and efficient manner. Using this method, the current study demonstrates that overexpression of C19MC miRNAs decreased the expression of EMT genes and increased the expression of reprograming factors and accelerated cell reprograming.

## Results

### C19MC is expressed in human villous trophoblasts

Previously, laser capture microdissection and RT-qPCR demonstrated higher C19MC expression in villi-containing regions of human first trimester and term placentas than in EVT regions^13^. However, C19MC expression *in situ* in the proximal cell columns (proliferative trophoblasts) and in the distal cell columns (invasive trophoblasts) of the anchoring villi at the maternal decidua had not been studied. Thus, we performed *in situ* hybridizations (ISH) with probes for miR-517a/c, a C19MC miRNA, or scramble control on first trimester and term placental sections. To distinguish trophoblasts (fetal) from decidual cells (maternal), consecutive serial sections were subjected to immunohistochemical staining for cytokeratin and vimentin, respectively.

**Figure 1:**
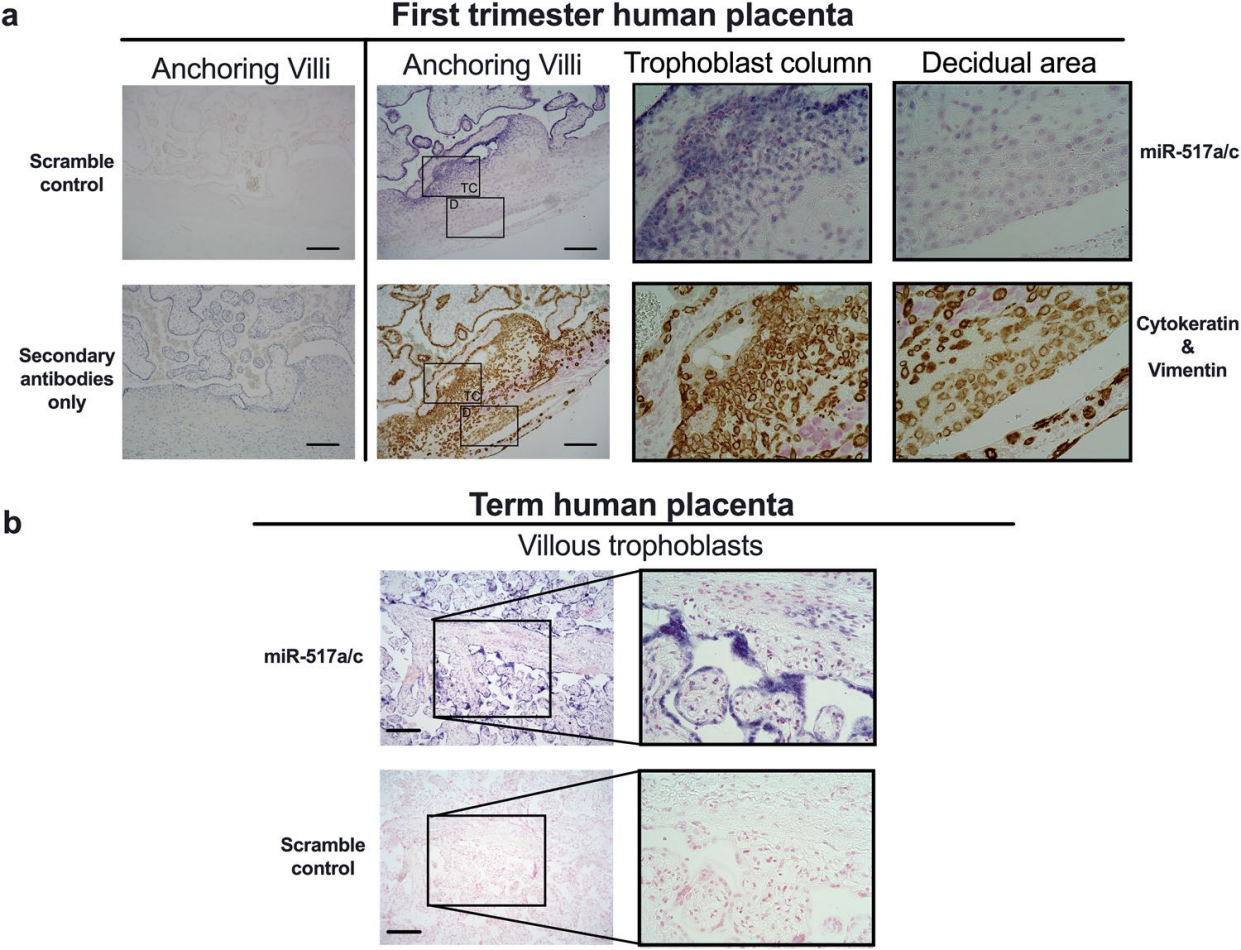
C19MC expression in human placenta. (**a**,**b**) Representative images of *in situ* hybridization for miR-517a/c (purple) or scramble control of first trimester human placentas (**a**) or term placentas (**b**). Nuclei were counterstained with nuclear fast red. To differentiate trophoblast from decidual cells, adjacent consecutively cut sections from first trimester human placentas were double-immunostained for trophoblast cell marker cytokeratin (brown), decidual cell marker vimentin (pink) or control secondary antibody and counterstained with hematoxylin. Scale bar represents 400 μm. Original magnification 10X, Black inserts 40X. TC, Trophoblast column; D, Decidual area.

We observed the strongest miR-517a/c expression in both CTs and STs in the villi, as well as in proximal CTs in the cell columns of the anchoring villi. This miR-517a/c expression gradually decreased in distal CTs with a further reduction in the interstitial EVTs (cytokeratin positive) in the maternal decidua and was undetectable in maternal decidual (vimentin positive) cells. No signal was detected in both ISH and immunohistochemical staining when scramble control or secondary antibody only were used, respectively (Fig. 1a). Term placental sections also displayed strong expression of miR-517a/c in villous trophoblasts (VTs, comprised of CTs and STs), whereas the scrambled control probe showed no staining (Fig. 1b). These results suggest that C19MC is required to maintaining the non-invasive epithelial phenotype of villous trophoblasts. Therefore, we sought to investigated whether C19MC regulates EMT.

### Transcriptional activation of C19MC

Investigation of the regulatory role of C19MC on EMT genes, requires an improved technique to efficiently overexpress the entire C19MC cistron *in vitro*. Hence, we employed the dCas9-based transcription activation system that combines a single-guide RNA (sgRNA) containing two MS2 RNA aptamers, a catalytically inactive Cas9 variant fused to the VP64 gene activator and MS2-p65-HSF1 activation helper proteins, designated as synergistic activation mediator (SAM) system^21^. This system facilitates robust and specific transcriptional activation by using a single targeted guide RNA (sgRNA) while overcoming the constraints of epigenetic regulation of C19MC. We designed two different sgRNAs targeted to the region immediately upstream of the first C19MC miRNAs, the 759-sgRNA binds at ~171 bp upstream of the first two miRNAs of the C19MC, whereas 620-sgRNA binds at ~585 bp upstream of the first miRNA of the C19MC (Supplementary Fig. s1).

**Figure 2:**
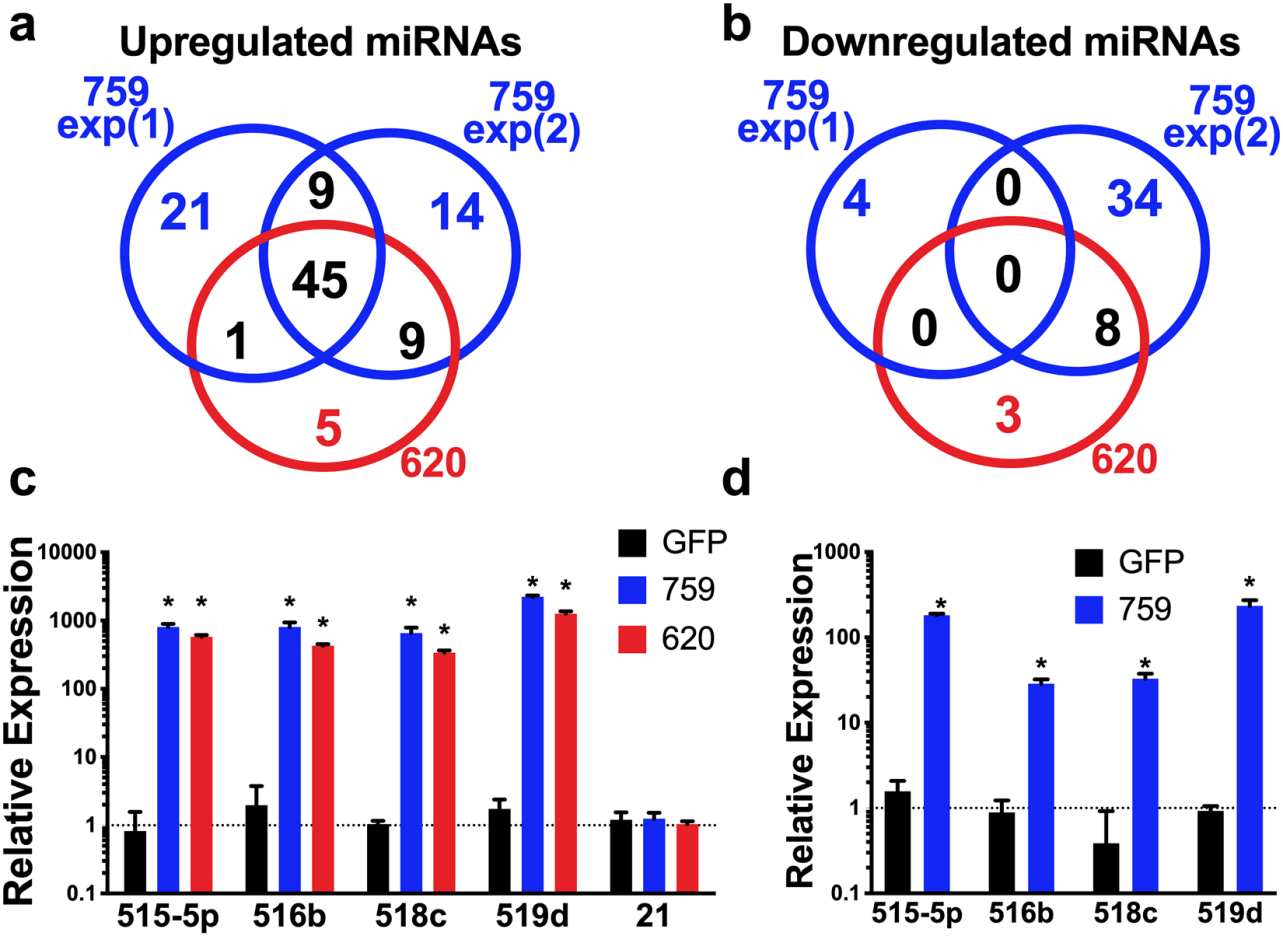
Transcriptional activation of C19MC. (**a**,**b**) Venn diagram of upregulated miRNAs (**a**) and downregulated miRNAs (b) from sRNAseq data from 3 independent experiments in HEK293 cells transfected with either 759-sgRNA/SAM (759) or 620-sgRNA/SAM (620) for 72 hr. (**c**,**d**) qRT-PCR analysis of four representative miRNAs of the C19MC cistron or miR-21 normalized to U18, 72 h after transfection of HEK293 cells with 759-sgRNA/SAM or 620-sgRNA/SAM (**c**) or 72 h after transfection of HTR8/SVneo cells with 759-sgRNA/SAM (**d**). Graphs represent means ± SEM of at least 3 independent experiments containing 3 replicates each. *p < 0.05 *vs*. GFP transfected control cells by 2-way ANOVA with Dunnett’s post-hoc (**c**) or by Multiple T-tests with Holm-Sidak correction (**d**).

To determine the specificity of our guide RNAs for C19MC and the extent of overexpression of the entire C19MC miRNA cistron using this system, we transiently transfected HEK293 cells with 759-sgRNA/SAM (2 independent experiments) or 620-sgRNA/SAM (1 independent experiment) for 72 hours. Remarkably, sRNAseq analysis displayed up-regulation of 45 miRNAs, ranging from 2- to 7984-fold, in all three independent experiments (threshold: ≥2- fold, FDR ≤ 0.20), 43 of which belong to the C19MC miRNA cluster (Fig. 2a, Supplementary Table s1). We also observed that activation of the C19MC cistron was ~2-fold higher with 759-sgRNA/SAM compared to 620-sgRNA/SAM (Supplementary Table s1). Importantly, miRNAs of the miR-371-373 cluster, located in close proximity to C19MC, were not induced by either 759-sgRNA/SAM or 620-sgRNA/SAM, indicating the specificity of the CRISPR/dCas9 SAM system for the C19MC cistron. Interestingly, other ESC-enriched miRNAs such as miR-302a-5p, miR-20b, miR-200a, miR-200b, miR-200c and miR-141 were also up-regulated (FDR ≤ 0.20) in HEK293 cells transfected with either 759-sgRNA/SAM or 620-sgRNA/SAM (Supplementary Table s1). On the other hand, no overlap was observed in the down-regulated miRNAs, although eight miRNAs that are known to promote cell differentiation including let-7a, miR-34a, miR-181b, miR-15a, miR-15b, miR-133a, miR-29b and miR-205, were down-regulated in 759-sgRNA/SAM exp2 and 620-sgRNA/SAM (Fig. 2b, Supplementary Table s1).

The expression of 4 randomly selected C19MC miRNAs was validated by RT-qPCR, which showed significant up-regulation of all 4 C19MC miRNAs (300- to 31,000-fold, *p* ≤ 0.05) in HEK293 cells, while the expression of miR-21, a non-C19MC miRNA, was unchanged further confirming the specificity of the CRISPR/dCas9 SAM system (Fig. 2c). Furthermore, transfection of two human tumor cell lines, MCF7 and PC3 cells with either 759-sgRNA/SAM or 620-sgRNA/SAM also induced C19MC expression (Supplementary Fig. s2). Lastly, we tested the reproducibility of this method in an immortalized invasive trophoblast cell line, HTR8/SVneo, that does not express C19MC. Since 759-sgRNA/SAM displayed higher C19MC induction efficiency in HEK293 cells, we used 759-sgRNA/SAM and observed 16- to 244-fold increased expression of 4 selected C19MC miRNAs compared to GFP transfected control, despite the low transfection efficiency (Fig. 2d). These results demonstrate that the CRISPR/dCas9 SAM system can be used to selectively and consistently activate the entire C19MC cistron, which spans over 100 kb.

### C19MC targets EMT and cell differentiation pathways

**Figure 3:**
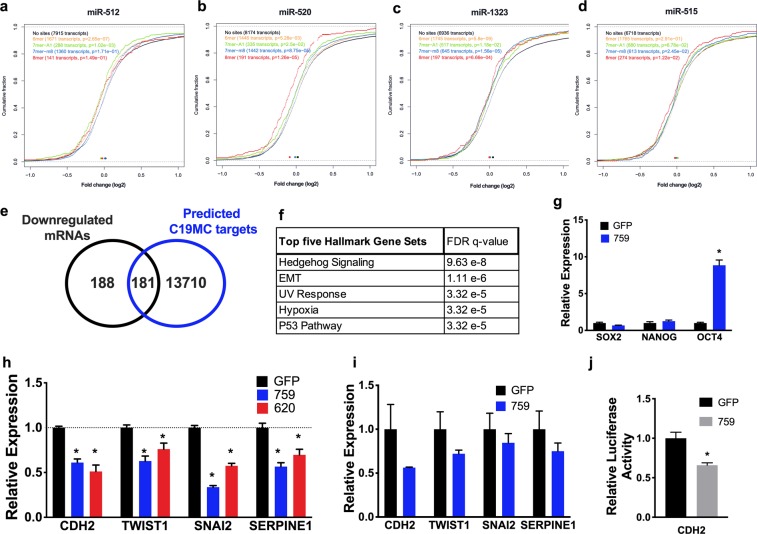
C19MC miRNAs increase OCT4 expression and decrease EMT gene expression. (**a**–**d**) Cumulative distribution of differentially expressed mRNAs targeted by miR-512, miR-520, miR-1323 and miR-515 in HEK293 cells transfected with 759-sgRNA/SAM (759) compared to GFP transfected cells. (**e**,**f**) Venn diagram (**e**) and Hallmark gene set analysis (**f**) of the 181 common downregulated mRNAs in HEK293 cells transfected with 759-sgRNA/SAM or 620-sgRNA/SAM (620) compared to GFP control and predicted C19MC miRNAs target genes. (**g**–**i**) qRT-PCR analysis of the indicated genes normalized to *GAPDH* in HEK293 cells (**g**,**h**) or in HTR8/SVneo cells (**i**) transfected with the indicated gRNA. (**j**) Luciferase reporter assay of HEK293 cells transfected with 759-sgRNA/SAM and PGL2 plasmid containing CDH2 3′ UTR normalized to GFP control. Graphs represent means ± SEM of at least 3 independent experiments containing 3 replicates each. *p < 0.05 *vs*. GFP transfected control cells. Multiple t-tests with Holm-Sidak correction (**g**,**I**,**j**); Two-way ANOVA with Dunnett’s post-hoc (**h**).

To identify potential C19MC miRNAs regulated genes in an unbiased manner, we performed RNAseq analysis of HEK293 cells transiently transfected for 72 hours with either 620-sgRNA/SAM or 759-sgRNA/SAM (Supplementary Table s2). RNAseq analysis revealed that induction of C19MC miRNAs by 759-sgRNA/SAM resulted in a higher number of differentially expressed genes than elicited by 620-sgRNA/SAM (Supplementary Table S2). Cumulative distribution of differentially expressed mRNAs (FDR ≤ 0.20) with predicted targets of miR-512, miR-520, miR-1323 and miR-515 (TargetScan, release 7.1) displayed a statistically significant (*p* ≤ 0.05) shift to the left, implying a dominant down-regulation of their predicted target genes consistent with activation of the C19MC cistron (Fig. 3a–d). Therefore, we focused on the 369 transcripts down-regulated (FDR ≤ 0.20, ≤−1.5 -fold) in both 759-sgRNA/SAM and 620-sgRNA/SAM transfected cells. Of these 369 mRNA transcripts, 181 were predicted targets of C19MC (MirWalk 2.0^22^) (Fig. 3e). The top five hallmark gene set analyses of these 181 genes were hedgehog signaling (HH), EMT, UV response, hypoxia and p53 pathways (FDR ≤ 3.32 × 10^−5^) (Fig. 3f). Furthermore, RNAseq revealed that *FGF4* expression was increased by 288- or 11-fold in HEK293 cells transiently transfected with 620-gRNA/SAM or 759-gRNA/SAM respectively, and *OCT4* expression was 13 -fold increased (FDR ≤ 0.2) only in 759-gRNA/SAM transfected cells (Supplementary Table S2). The expression of *OCT4* and other pluripotency transcription factors *SOX2* and *NANOG* were assessed by RT-qPCR and confirmed that *OCT4* expression was indeed increased (>8 -fold, *p* < 0.05) in HEK293 cells transiently transfected with 759-gRNA/SAM (Fig. 3g).

To further investigate the effect of C19MC overexpression on the EMT pathway, the EMT RT^2^ profiler PCR Array (Qiagen) was used to quantify changes in the expression of 84 EMT-associated genes and found 2- to 4.75-fold decrease in the expression of *CDH2*, *SERPINE1*, *TWIST1*, *SNAI2*, *CALD1*, *GSC*, *ITGAV*, *MMP3*, *TCF4* and *WNT5a* (p ≤ 0.05) in HEK293 cells transfected with 620-sgRNA/SAM compared to control, further confirming the hallmark gene set analysis (Supplementary Fig. s3). HEK293 cells transfected with 759-sgRNA/SAM or 620-sgRNA/SAM showed significant decrease in *CDH2*, *SERPINE1*, *TWIST1* and *SNAI2* expression compared to control (Fig. 3h). Importantly, HTR8/SVneo cells transfected with 759-sgRNA/SAM also showed decrease in the expression of EMT gene. However, due to their low transfection efficiency (Fig. 2d), the reduction in EMT gene expression did not reach statistical significance (Fig. 3i).

To determine if the decrease in the expression of one of the target mRNAs, specifically *CDH2*, resulted from direct targeting by C19MC miRNAs, we constructed a luciferase reporter containing the 3′ UTR of the *CDH2* gene. Accordingly, HEK293 cells transfected with 759-sgRNA/SAM and the *CDH2* 3′ UTR luciferase construct displayed a 35% decrease (p < 0.05) in luciferase activity (Fig. 3j). Taken together, these results indicate that overexpression of C19MC induces transcription of major pluripotent factors *OCT4* and *FGF4* and inhibits EMT by directly repressing expression of genes crucial for EMT.

### Hypoxia inhibits C19MC and increases expression of EMT markers

**Figure 4:**
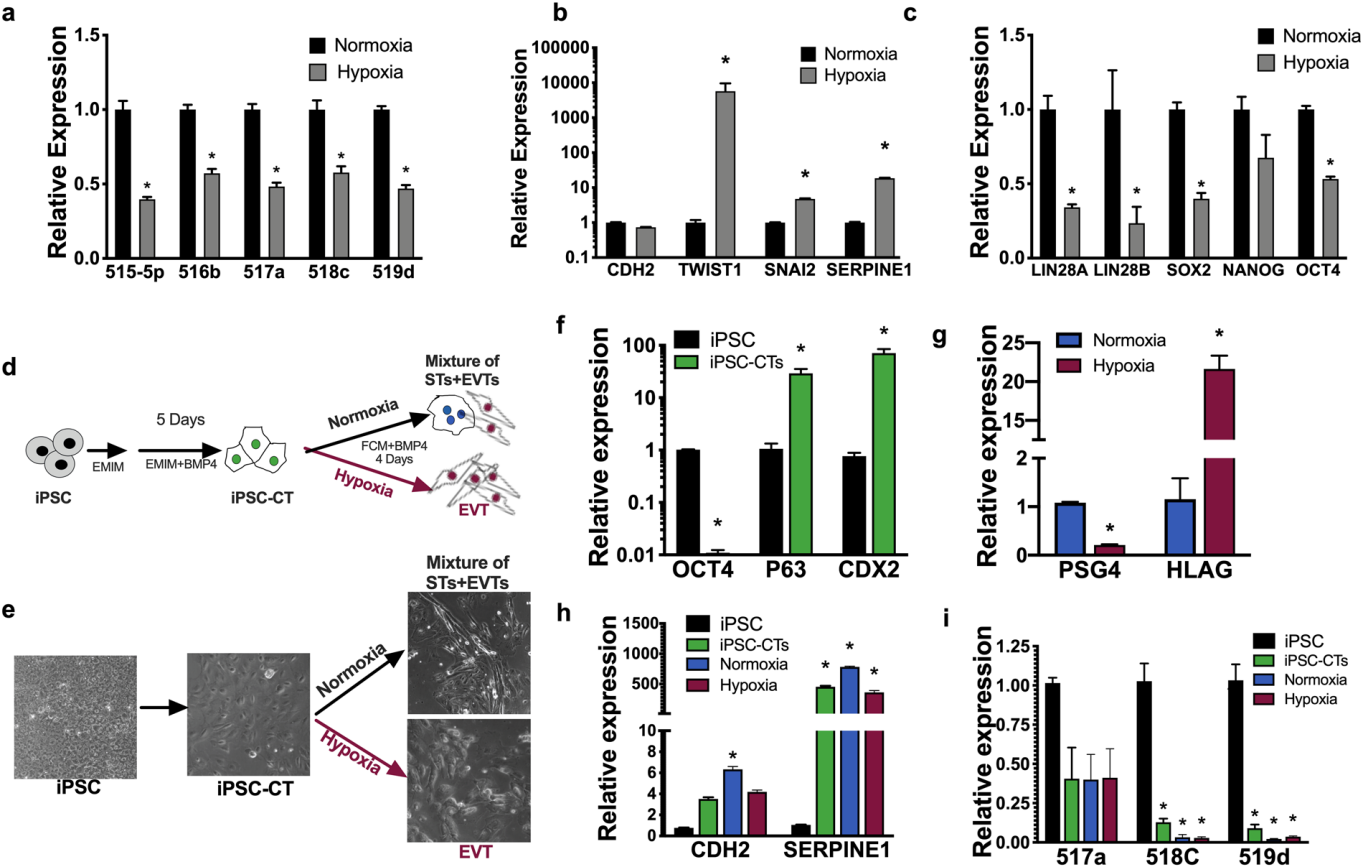
Hypoxia decreases the expression of C19MC miRNAs and upregulates EMT genes. (**a**–**c**) qRT-PCR analysis of the indicated miRNA normalized to U18 (**a**) or indicated genes normalized to *GAPDH* (**b**,**c**) of iPSCs cultured in hypoxia (1% O_2_) or normoxia (21% O_2_) for 24 hours. (**d**,**e**) Schematic (**d**) and 20x phase images (**e**) showing two-step iPSC differentiation. Generation of iPSC derived CTs (iPSC-CT) using EMIM medium supplemented with 10 ng/ml BMP4 (step1) and subsequently cultured in FCM medium supplemented with 10 ng/ml BMP4 under normoxia or hypoxia to terminally differentiate iPSC-CTs into STs and EVTs, respectively (step2). (**f**–**i**) qRT-PCR analysis of the indicated genes normalized to *GAPDH* (f-h) or the indicated miRNA normalized to U18 (**i**). Graphs represent means ± SEM of a minimum of 3 independent experiments each comprised of 3 replicates. *p < 0.05 *vs*. normoxia (**a**–**c**) or vs. iPSC (**f**,**h**,**i**), Multiple t-tests with Holm-Sidak correction (**a**–**c**,**f**,**g**); Two-way ANOVA with Dunnett’s post-hoc (**h**,**i**).

During the first trimester of human pregnancy, oxygen tension plays a key role in regulating trophoblast cell proliferation and differentiation^23,24^. To evaluate the *in vitro* effect of hypoxia and avoid the spontaneous differentiation that occurs during culturing of primary human trophoblast cells^25^, we used iPSCs, which share many stem-like characteristics with CTs and express similarly high levels of C19MC. sRNAseq analysis of iPSCs incubated in hypoxia (1% O_2_) for 24 hours displayed ~ 2-fold decrease in the expression of C19MC (mir-498 cistron), while the hypoxia-inducible miR-210^26^ was increased 13.9-fold (FDR ≤ 0.20) compared to normoxia (21% O_2_) (Table 1). Specifically, 12 miRNAs of the C19MC were decreased by 2.1- to 292.6-fold (Supplementary Table S3). Moreover, RT-qPCR analysis confirmed the reduction of five miRNAs of the C19MC by hypoxia (Fig. 4a).

**Table 1:** Hypoxic conditions decrease expression of the C19MC cistron in iPSC.

miRNA cistron	Normalized frequency (%)		Fold change	FDR
	Hypoxia	Normoxia		
mir-498(46)	0.342795	0.653611	−1.9	0.01078437
mir-30b(2)	0.683601	0.385398	1.8	0.12703533
mir-424(2)	0.108522	0.03614	3	0.00814663
mir-598(1)	0.0331	0.010923	3	0.10326634
mir-9-1(3)	0.381634	0.123784	3.1	0.01362189
mir-489(2)	0.012991	0.003859	3.4	0.16704579
mir-210(1)	0.412455	0.029741	13.9	1.34E-08

Data shows fold change of normalized frequency (>2- fold, FDR ≤ 0.2). C19MC.

To determine whether hypoxia regulates genes involved in EMT, we performed RNAseq analysis on iPSCs exposed to hypoxia (1% O_2_) or normoxia (21% O_2_) for 24 hours. Hallmark gene set analysis of up-regulated genes revealed that hypoxia induced the expression of genes involved in EMT and P53 pathways (FDR ≤ 0.2), of which *SERPINE1* and *TWIST1* exhibited 43.7- and 10-fold increased expression, respectively (FDR ≤ 0.20) (Supplementary Table s4). Increased expression of the EMT genes, *TWIST*, *SNAI2 and SERPINE1* was also confirmed by RT-qPCR (Fig. 4b). Furthermore, RT-qPCR analysis of iPSCs exposed to hypoxia displayed down-regulation of the stem cell associated genes *LIN28A*, *LIN28B*, *SOX2*, *NANOG*, and *OCT4* (Fig. 4c).

Hypoxia is known to preferentially promote the initial differentiation of CTs into EVTs^27^. To gain insight into the effect of hypoxia on C19MC and EMT genes during the CT-to-EVT differentiation process, we used a two-step method *in vitro* that utilizes a low concentration of BMP4 to differentiate iPSCs into CTs^27^ (Fig. 4d). After the first differentiation step, iPSC-derived CTs displayed a more flattened and elongated CT phenotype and expressed high levels of the CT associated genes *P63* and *CDX2* compared to undifferentiated iPSCs (Fig. 4e,f). Moreover, iPSC-derived CTs showed a reduction in *OCT4* expression compared to undifferentiated iPSC (Fig. 4f). In the second step, iPSC-derived CTs were cultured in either normoxia (20% oxygen) or hypoxia (1% oxygen) to further differentiate them into STs- and EVT-like cells, respectively^27^. Consistent with previous reports^25,28^, the expression levels of *PSG4* (an ST-associated transcript) was significantly reduced, whereas the expression of *HLAG* (an EVT marker) was significantly increased when the cells were incubated under hypoxia (Fig. 4g). Importantly, iPSC-derived CTs, or iPSC-derived CTs cultured in normoxia or hypoxia showed a significant increase in the EMT genes *CDH2* and *SERPINE1* and loss of miR-518c and miR-519d (C19MC miRNAs) compared to undifferentiated iPSCs (Fig. 4h,i). These findings indicate that EMT induced either by hypoxia or by BMP4 used for iPSC-to-CT differentiation is associated with a significant decrease in expression of C19MC.

### C19MC enhances cellular reprogramming of somatic cells

**Figure 5:**
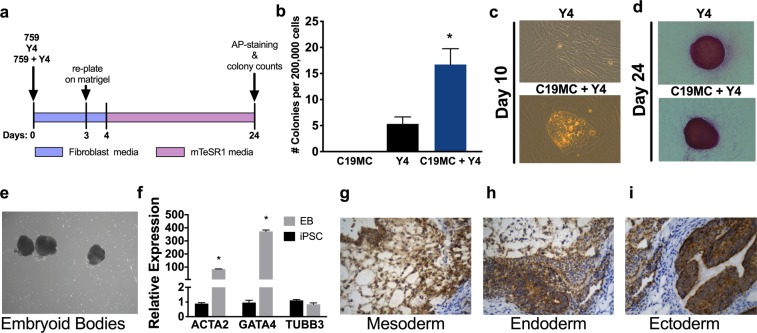
C19MC accelerates iPSC generation when co-transfected with Yamanaka factors. (**a**) Schematic of transfection protocol. 759, sgRNA-759/SAM; Y4, Yamanaka factors. (**b**) Colony counts from 759-, Y4- and 759+Y4-transfected NHDFs at day 24. (**c**,**d**) Colony morphology at 10 days post transfection (**c**) or AP staining of iPSC clones at day 24 (**d**) of Y4- or 759+Y4-transfected NHDFs. (**e**,**f**) Image (**e**) and qRT-PCR analysis of the indicated genes normalized to *GAPDH* (**f**) of embryoid bodies (EB) generated from 759+Y4 iPSC clone 3-CF-6 (passage 5) after 7 days culture in suspension compared to the parental non-differentiated iPSC clone. (**g**–**i**) Images of teratoma sections from SCID mice injected with iPSCs generated using 759-sgRNA/SAM +Y4, immunostained with human mitochondria specific antibody and hematoxylin showing ectoderm, mesoderm and endoderm like cellular structures. Graph represents means ± SEM. *p < 0.05 vs. Y4 (**b**) or non-differentiated iPSC 3-CF-6 clone (**f**). Two way ANOVA with Dunnett’s post-hoc (**b**); Multiple t-tests with Holm-Sidak correction (**f**).

Since C19MC miRNAs are highly expressed in embryonic stem cells and its overexpression increased the expression *of OCT4* and *FGF4*, we tested whether the robust activation of the C19MC by CRISPR/dCas9 SAM system induces cell reprogramming. Accordingly, normal human dermal fibroblast cells (NHDFs) were transfected with 759-sgRNA/SAM alone, or with Y4, which consists of three episomal plasmids that contain the Yamanaka reprogramming factors *OCT3/4*, *SOX2*, *KLF4*, and *LIN28* in combination with *L-MYC* and *p53* shRNA as previously described^29^, or with a combination of 759-sgRNA/SAM and Y4 (759-sgRNA/SAM +Y4) (Fig. 5a).

NHDFs transfected with 759-sgRNA/SAM alone exhibited a gradual increase in the expression of miR-515-5p (a representative miRNA of the C19MC cistron) that peaked at ~24-fold on day 4 and returned to baseline on day 7 (Supplement Fig. s4). NHDFs transfected with 759-sgRNA/SAM alone did not produce any reprogrammed iPSC colonies at day 24 post transfection (Fig. 5b). Intriguingly, NHDF cells transfected with 759-sgRNA/SAM +Y4 produced a significantly higher number of iPSC colonies (an average of 17 colonies/200,000 cells) compared to cells transfected with Y4 alone, which produced an average of 5 colonies/200,000 cells (p ≤ 0.05) on day 24 post transfection (Fig. 5b). Moreover, the colonies were observed earlier in NHDFs transfected with 759-sgRNA/SAM +Y4 at 7 to 10 days post transfection compared to 14 to 20 days post transfection in NHDFs transfected with Y4 alone (Fig. 5c). The undifferentiated stage of the colonies was confirmed by alkaline-phosphatase staining at day 24 post transfection (Fig. 5d). These results indicate that the combination of 759-sgRNA/SAM +Y4 increases both the speed and efficiency of cell reprogramming.

To test the differentiation potential of iPSCs generated by using 759-sgRNA/SAM +Y4 *in vitro*, we used a floating cultivation method for embryoid body formation. iPSCs generated using 759-sgRNA/SAM +Y4 formed spheroid shaped structures after 8 days in suspension culture (Fig. 5e). Embryoid bodies were transferred to gelatin-coated plates and cultured for an additional 7 days. Attached cells displayed various cell morphologies that included contracting cardiac-like cells and expressed higher levels of the germ layer markers *ACTA2* and *GATA4* (84- and 37- fold respectively, p ≤ 0.05) compared to undifferentiated control iPSCs (Fig. 5f).

Lastly, iPSCs generated using 759-sgRNA/SAM +Y4 transplanted subcutaneously into dorsal flanks of SCID immunodeficient mice also displayed tissue structures derived from endoderm, mesoderm and ectoderm, further confirming that iPSCs generated by 759-sgRNA/SAM +Y4 exhibit characteristics of pluripotent stem cells (Fig. 5g–i).

## Discussion

Trophoblast differentiation and subsequent invasion into the decidua is critical for implantation and placentation during early human pregnancy and maintenance of pregnancy thereafter. Villous trophoblasts display epithelial characteristics including apicobasal polarity, lateral cell junctions and contact with basement membrane^30^. Upon differentiation to EVTs, trophoblasts undergo EMT, resulting in loss of cell-cell junctions and apical basal polarity and acquisition of a migratory and invasive capacity^30^. The current study demonstrates that C19MC is primarily expressed in the non-invasive villous trophoblasts and is lost as trophoblasts differentiate into EVTs. Under hypoxia, the expression of C19MC is reduced, whereas EMT and cell differentiation are induced. Utilizing the CRISPR/dCas9 SAM system we were able to transcriptionally activate the entire C19MC and demonstrate its role in inhibition of EMT and enhancing the epithelial stem cell state. Moreover, co-expression of C19MC with Yamanaka factors in NHDFs significantly increased the efficiency of somatic cell reprogramming into iPSCs.

The current study establishes *in situ*, the localization of C19MC cistron in first trimester human placental tissues that is highly expressed in villous trophoblasts comprising of CTs, STs and proliferative proximal trophoblastic cell columns in the anchoring villi. However, as trophoblasts differentiate into EVTs in the distal trophoblastic cell columns and in the interstitial trophoblasts in the decidua C19MC expression appears to diminish. These results agree with a previous report that used laser-capture microdissection of the villi-containing regions and EVT regions of paraffin-embedded first trimester placental specimens followed by RT-qPCR^13^.

To transcriptionally activate the entire C19MC cistron, which spans over 100 kb, we employed the CRISPR/dCas9 SAM system^21^. Comparisons of sRNAseq analysis of three independent experiments using either 759-sgRNA/SAM or 620-sgRNA/SAM in HEK293 cells, revealed that a single guide RNA is sufficient to up-regulate the expression of the entire 100 kb C19MC locus. We observed that 759-sgRNA/SAM, which binds at two locations (~171 bp upstream of the first two C19MC miRNAs), resulted in ~2-fold higher expression of miRNA of the C19MC cistron and higher number of differentially expressed genes compared to the 620-sgRNA/SAM, which binds to one location (~585 bp upstream of the first C19MC miRNA). These findings are in agreement with previous studies showing that the efficiency of gene editing increases when multiple gRNAs that target the same gene are used^31,32^. The transcriptional activation that we achieved was specific to C19MC since the expression of the neighboring miR-371–373 cluster, which is located 20 kb downstream of the C19MC, was not increased. These results are consistent with a previous report showing that C19MC miRNAs are a product of a single transcript that is rapidly spliced and processed by the Drosha-DGCR8 complex^33^. In the current study, only two up-regulated miRNAs, miR-139 and miR-449, which are not members of the C19MC, overlapped in the 3 independent experiments. Interestingly, these two miRNAs are known to inhibit EMT and may be regulated by the C19MC^34,35^. A few other miRNAs that are known to stimulate cell reprogramming and do not belong to the C19MC were also up-regulated in HEK293 cells transfected with either 759-sgRNA/SAM or 620-sgRNA/SAM. Conversely, eight miRNAs that are known to promote cell differentiation were down-regulated in 620-sgRNA/SAM and in one of the 759-sgRNA/SAM transfected cells. Additional studies are required to investigate whether these non-C19MC miRNAs are directly or indirectly regulated by the C19MC.

In agreement with the physiological expression of C19MC miRNAs in villous trophoblasts and its gradual loss in EVTs, we found that transcriptional activation of the C19MC led to a significant decrease in the expression of numerous genes involved in Hedgehod signaling (HH) and EMT, several of which are predicted targets of C19MC. These findings are in agreement with a previous study, which showed that BAC mediated C19MC overexpression in HTR8/SVneo cells, decreases their migration^13^. Moreover, several studies have demonstrated a link between HH signaling and EMT^36^. In human trophoblasts, activation of HH signaling induced key EMT regulators including *SNAI1* and *TWIST1*^37^. Therefore, the current results place C19MC upstream of HH and EMT signaling.

The early placental environment is highly hypoxic because intervillous circulation is not established until after the first trimester^23,24^. EVT migration and invasion into the maternal decidua and subsequent remodeling of the maternal spiral arteries are critical steps for proper perfusion of the placenta, which is required for a successful pregnancy^30^. Changes in oxygen tension within the intervillous space are stringently controlled and play a role in trophoblast differentiation, migration and invasion that are essential for vascular remodeling during pregnancy^38^. Previously, primary human trophoblast (PHT) cells were used to test the effect of hypoxia (<1% oxygen) on C19MC expression^39^. However, PHT cells undergo spontaneous differentiation and lose C19MC expression when cultured for 72 hours^39^. To avoid the spontaneous differentiation of PHTs, in this study, we used undifferentiated iPSCs and found that 24 hours exposure to hypoxia (1% oxygen) significantly decreased the expression of numerous C19MC miRNAs and several reprogramming factors including *OCT4* and *LIN28B*, whereas the expression of EMT related genes was increased. The repression of *LIN28B* expression in iPSCs by hypoxia agrees with our previous study that showed that hypoxia decreased the expression of *LIN28B* in choriocarcinoma BeWo and JEG3 cells, and in placentas from preeclampsia-affected pregnancies^40^. In addition, repression of *OCT4* induces loss of pluripotency and differentiation of ESC cells derived from the inner cell mass toward the trophectoderm lineage^41^. These findings are also consistent with previous studies reporting that hypoxia is a major inducer of EMT^42–44^. Although the mechanisms by which hypoxia reduces the expression of C19MC miRNA are not yet known, potential mechanisms include *de novo* methylation or regulation of C19MC miRNA biogenesis and activity^45,46^. The current study also found significant reduction in the expression of C19MC miRNAs and increased expression of the EMT genes when iPSCs were initially differentiated to bipotential stem-like CTs and subsequently differentiated into STs and EVTs. Although the first step of iPSC differentiation to iPSC-derived CTs increased CT markers, C19MC miRNAs were unexpectedly reduced, perhaps due to the use of BMP4 that induce EMT^41,47–49^. These results indicate that trophoblasts generated using this two-step protocol are atypical since they do not fully recapitulate elevated C19MC expression found in normal trophoblasts. Although our results indicate a strong association between C19MC and EMT, C19MC knockdown experiments in stem cells are required to prove that C19MC regulates EMT.

Our results also reveal that transient activation of C19MC increased the expression of *FGF4* and *OCT4*, significantly increased the number of iPSC colonies when co-transfected with Y4. These data are consistent with a previous study that used a lentiviral vector encoding a C19MC miRNA (mir-524 precursor)^19^. Moreover, some C19MC miRNAs share seed sequences with the miR-302/-372 family, which also enhances reprogramming by promoting MET^50^. Although the mechanisms by which C19MC induces the expression of *OCT4* and *FGF4* are not yet clear, they may reflect either C19MC mediated inhibition of transcriptional suppressors or binding to the promoter regions and induction of gene expression^51,52^.

The results presented here establish the CRISPR/dCas9 SAM technique as a powerful tool to investigate the role of C19MC cistron in human placental physiology. Employing this robust technique enabled us to uncover the crucial role of C19MC in regulating EMT genes in villous trophoblasts and maintaining their stem-like epithelial cell phenotype. The hypoxic condition during early placentation reduce C19MC expression and releases the inhibition of EMT genes leading to the acquisition of migratory and invasive characteristics of EVTs. Therefore, maintaining optimal expression levels of C19MC is likely critical for EVT differentiation and invasion. Dysregulation of C19MC may result in impaired invasion associated with either the shallow placentation of preeclampsia or the exuberant invasion of placenta accreta.

## Materials and Methods

### Study approvals

Term placental sections were obtained from pregnancies following vaginal delivery after obtaining a written informed consent and approval by the institutional review board of the University of South Florida (Protocol 00015578). Placental sections from the first trimester (7- and 8-week gestation, n = 2) and early human pregnancies (20-weeks week gestation, n = 2) were obtained from a previously banked deidentified paraffin tissues, under approval by Yale University Human Investigation Committee and by the institutional review board of the University of South Florida. Animal study procedures were approved by the Institutional Animal Care and Use Committee (IACUC, IS00004309) at the Morsani College of Medicine, University of South Florida. All methods were performed in accordance with the relevant guidelines and regulations.

### *In situ* hybridization

*In situ* hybridization for hsa-miR-517a/c, a C19MC miRNA, was performed in paraffin embedded sections of early pregnancy placentas. After deparaffinization in xylene and rehydration by a series of graded alcohol washes, *in situ* hybridization was performed using 40 nm 5′,3′ digoxigenin-labeled locked nucleic acid probe for hsa-miR-517a/c (Exiqon, 611715-360) or scrambled (negative) control (Exiqon, 90005). Hybridization and post-hybridization graded SSC washes were performed at 55 °C. The sections were then blocked, and the probes were detected using alkaline phosphatase conjugated sheep anti-digoxigenin Fab fragments (Roche, 11093274910). The signal was developed using NBT/BCIP (Roche, 11697471001) as a substrate that produces dark-blue indigo precipitating dye followed by nuclear counterstaining with Nuclear Fast Red (Vector laboratories, H-3403). The sections were dried and covered with mounting medium for later image analysis.

### Immunohistochemistry

Cytokeratin and vimentin immunostaining of early human placental sections was performed as previously described^40,53^. Briefly, paraffin embedded sections were deparaffinized in xylene and rehydrated in a series of graded alcohol washes followed by antigen retrieval by boiling in citric acid (pH6.0). Endogenous peroxidase activity was quenched by incubation in 3% H_2_O_2_. The sections were then blocked using normal horse serum followed by incubation with the primary antibody (mouse-anti-cytokeratin, Dako M7018, 1:600, RRID:AB_2134589). The following day, cytokeratin was detected by incubation with a secondary antibody (biotinylated horse-anti-mouse, Vector Laboratories BA-2000, 3.75ug/ml, RRID:AB_2313581) together with the avidin-biotin-peroxidase complex (Vectastain ABC Kit, pk6200, Vector Laboratories). The signal was then developed using 3,3- diaminobezidine (sk-4100, Vector Laboratories) as a substrate. The sections were thoroughly washed and blocked using normal donkey serum, followed by overnight incubation with the primary antibody (chicken-anti-vimentin, Abcam ab39376, 1ug/ml, RRID:AB_778827). The following day, vimentin was detected by incubation with a secondary antibody (donkey-anti-chicken, Jackson ImmunoResearch 703-065-155, 1.2ug/ml, RRID:AB_2313596) together with avidin-biotin-alkaline phosphatase (Vectastain ABC-AP, Vector laboratories AK-5200). The signal was then developed using Vector Red AP substrate (Vector Red, Vector Laboratories SK-5100) followed by nuclear counterstaining with hematoxylin. The sections were then dried and covered using a mounting medium for later image analysis.

### Cell culture

HEK293 cells (Stratagene #240085) were maintained in DMEM supplemented with 10% heat-inactivated fetal bovine serum (FBS; Sigma-Aldrich). HTR8/SVneo cells (CRL-3271; ATCC) were cultured in RPMI 1640 supplemented with 5% FBS. MCF7 cells (ATCC HTB-22) were maintained in EMEM supplemented with 10% FBS and PC3 cells (ATCC CRL-1435) were maintained in F-12K medium supplemented with 10% FBS. Normal Human Dermal Fibroblasts (NHDF, Lonza CC-2511) were purchased and maintained in FGM-2 BulletKit (Lonza CC-3132). The iPSC cell line SCVI274, a gift from Dr. Joseph C. Wu at Stanford Cardiovascular Institute, Stanford University School of Medicine, was cultured on Matrigel coated 6-well plates and maintained in Essential 8 medium (A1517001, Life Technologies) and passaged every fourth day.

### Design and cloning of C19MC-specific sgRNAs

Single guide RNAs that target the upstream region of C19MC were designed using the ATUM CRISPR sgRNA design tool. Two guide sequence oligos, 759-sgRNA 5′-CAAATCCTAGGCCTGCCCTG and 620-sgRNA5′- GTGAGCTGATGATCGCTCCA, were cloned into the ssgRNA(MS2) lenti ssgRNA(MS2) zeo backbone (a gift from Dr. Feng Zhang^21^, Addgene #61427) using a Golden-Gate sgRNA cloning protocol and transformed into Stbl3 recombination deficient competent cells (Life Technologies, C7373-03). Ampicillin resistant clones were selected and verified by sequencing.

### Transient activation of C19MC miRNAs

HEK293 (10^5^ cells/well) were seeded in 12-well plates (Greiner Bio). The following day, the cells were transfected with 1.25 μg total DNA in a 1:1:1 mass ratio of sgRNA, MS2-P65-HSF1-Hygro and dCAS9-VP64-GFP (gift from Dr. Feng Zhang^21^ Addgene #61426 and #61422, respectively) using Lipofectamine 2000 (Life Technologies) according to the manufacturer’s instructions. Culture medium was replaced with fresh medium after 24 h. Total RNA was isolated after 72 h.

### iPSC generation

Three micrograms of 759-sgRNA, MS2-P65-HSF1-Hygro and dCAS9-VP64-GFP in a 1:1:1 mass ratio alone or together with three micrograms of pCXLE-hOCT3/4-shp53-F, pCXLE-hSK and pCXLE-hUL (gift from Dr. Shinya Yamanaka^29^ Addgene #27077, #27078 and #27080, respectively) plasmid mixture at 1:1:1 mass ratio were electroporated into 6 × 10^5^ NHDF cells using nucleofector (Lonza) and Amaxa Human Dermal Fibroblast Nucleofector Kit (Lonza, CC-2511) according to the manufacturer’s instructions. The cells were then seeded on 0.1% gelatin coated 6 well plates. After 3 days incubation, cells were trypsinized and 2 × 10^5^ cells were seeded onto 100-mm Matrigel-coated dishes. The next day, the culture medium was replaced with mTeSR1 medium (STEMCELL Technologies, 85857). Colonies were counted from 20 to 30 days after re-seeding, the resulting colonies similar to ESCs in morphology were harvested for further expansion and maintained in E8 media (Life Tech, A1517001) for at least 10 passages.

### *In vitro* differentiation of iPSCs

Human iPSCs were harvested by treating with collagenase IV for 30 minutes at 37 °C. Cell clumps were transferred to ultra-low attachment plates (Fisher Scientific, 07-200-601) and maintained in DMEM supplemented with 20% FBS. Media was replaced with fresh medium every other day. After 7 days in suspension culture, embryoid bodies were transferred to gelatin coated 8-well chamber slides and cultured for an additional 10 days. Expression of the different germ layer markers were quantified by RT-qPCR.

Differentiation of iPSCs into CTs, STs and EVTs was performed as previously described^27^. Briefly, iPSCs (cell line SCVI274) were treated for 2 days with EMIM medium containing KnockOut DMEM/F12 medium (Thermo Fisher Scientific, 12660012), 1% insulin-transferrin-selenium A (Thermo Fisher Scientific 41400045), 1x nonessential amino acid (Sigma-Aldrich M7145-100ML), 2% BSA (Thermo Fisher Scientific, AM2618) 2 mM L-glutamine (Life Technologies, 25030-081) and 100 ng/mL heparan sulfate proteoglycan (Sigma- Aldrich, H4777-.1MG). The medium was then replaced with EMIM supplemented with 10 ng/mL human BMP4 (R&D Systems, 314-BP-010) for an additional 5 days, at which point they were designated iPSC-derived CTs. For ST and EVT differentiation, iPSC-derived CTs were passaged using 0.25% trypsin and seeded on Matrigel (Corning, 354277) coated plates in FCM [KnockOut DMEM/F12 containing 20% (vol/vol) KnockOut serum replacement (Knockout SR; Thermo Fisher Scientific, 10828010), 1x GlutaMAX (Thermo Fisher Scientific, 35050061), and 1x nonessential amino acid, 0.1 mM 2-mercaptoethanol (Thermo Fisher Scientific, 21985023); conditioned on irradiated mouse embryonic fibroblasts for 24 h], supplemented with 10 ng/mL human BMP4 and incubated in normoxia (21% Oxygen) or hypoxia (1% Oxygen) respectively for the indicated number of days.

### Teratoma formation and immunohistochemistry

iPSCs were mixed with Matrigel and injected into each flank of NOD-SCID mice. Tumors were harvested at 4 weeks post-injection, fixed in 4% paraformaldehyde and embedded in paraffin. To differentiate implanted human cells from mouse cells, tumor sections were immunostained with a human anti-mitochondria specific antibody (Abcam cat# ab92824) at 1:1000 dilution followed by secondary antibody (biotinylated horse-anti-mouse, Vector Lab BA-2000, 3.75ug/ml, RRID:AB_2313581) according to the manufacturer’s protocol. Nuclei were subsequently counterstained with hematoxylin.

### Alkaline phosphatase staining

Alkaline phosphatase histochemical staining was performed using an Alkaline phosphatase detection kit (Millipore, SCR004) as described in the manufacturer’s instructions. Briefly, cells were fixed with 4% paraformaldehyde for 10 minutes and washed once with rinse buffer (20 mM Tris-HCl, pH 7.4 and 0.05% Tween 20). Staining solution was added to the wells and plates incubated in the dark for 25 min. Bright field images were then obtained using a light microscope.

### RNA isolation and quantitative PCR

Total RNA was isolated using the RNeasy Mini Kit (Qiagen) and stored at −80 °C in RNAse-free water. For qRT–PCR analysis, 1 µg total RNA was reverse transcribed using random hexamer or oligodT primers and M-MuLV reverse transcriptase (New England Biolabs) according to the manufacturer’s specifications. For miRNA cDNA, 0.5 ug of RNA was reverse transcribed using the TaqMan miRNA Reverse Transcription Kit (ThermoFisher Scientific, 4366596) as previously described^11,40^.

To assess relative mRNA and miRNA expression levels, quantitative PCR (RT-qPCR) of cDNA products was performed using the following ThermoFisher TaqMan RT-qPCR probes*: LIN28A* (Hs00702808_s1), *LIN28B* (Hs01013729_m1), *SOX2* (Hs01053049_s1), *NANOG* (Hs04399610_g1), *OCT4* (Hs00999632_g1), *CDH2* (Hs00983056_m1), *SERPINE1* (Hs00167155_m1), *TWIST1* (Hs01675818_s1), SNAI2 (Hs00161904_m1), *ACTA2* (Hs00426835_g1), *TUBB3* (Hs00801390_s1), *GATA4* (Hs00171403_m1), *CDX2* (Hs01078080_m1), *PSG4* (Hs01923769_s1), *p63* (Hs00978340_m1), *HLAG* (Hs00365950_g1), *GAPDH* (Hs02786624_g1), *miR-515-5p* (001112), miR-516b (001150), *miR-518c* (002401), *miR-519d* (002403), *miR-21* (000397), *U18* (001204). TaqMan probes were used according to manufacturer’s instructions with TaqMan Fast Advanced Master Mix and QuantStudio 3 instrument (Life Technologies). Data were analyzed by the 2^-ΔΔ*C*_t_ method; target *C*_t_ values were normalized to *GAPDH C*_t_ values for mRNA expression and U18 for miRNA expression. RT-qPCR for EMT associated genes was performed using RT² Profiler PCR Array Human Epithelial to Mesenchymal Transition (EMT) (Qiagen, PAHS-090Z) according to manufacturer’s instructions.

### Global miRNA- and mRNA-sequencing

Two micrograms of total RNA were converted into a small RNA (sRNA) cDNA library according to published protocol^54^. Briefly, the RNA input for each sample was ligated to a 3′ adaptor barcoded sequence, pooled, size selected, and gel purified, followed by 5′ adapter ligation and then subjected to size selection and gel purification. The cDNA library preparation was completed by second strand synthesis using SuperScript III, alkaline RNA hydrolysis, and PCR amplification for 10 cycles. mRNA libraries were prepared by utilizing the Illumina TruSeq Stranded mRNA LT protocol using 500 ng total RNA and NEB’s Protoscript II reverse transcriptase for the first-strand cDNA synthesis according to the manufacturer’s protocol. Individual RNAseq libraries were quality controlled on an Agilent TapeStation with a High Sensitivity D1000 ScreenTape. Indexed samples were quantified using the Qubit dsDNA HS assay and were pooled at equimolar concentration (10 nM). The libraries were sequenced on an Illumina NextSeq. 500 sequencer 75-bp paired-end in mid-output mode in the Genomics Core Facility of The Rockefeller University.

### Bioinformatics analysis

The miRNA read annotation was performed as previously described^54,55^. Multimapping reads were assigned to each mapping transcript divided by the number of mapping locations to create a count matrix (i.e. fractional counting). The mRNAseq data were aligned to the human genome build 38 using the STAR aligner^56^ (version 2.0.4j) allowing for two mismatches. Expression values (count matrices) were generated using featureCounts together with gene definitions from Ensembl release 82 (GTF file).

### Luciferase reporter plasmid, transfection and luciferase assay

The CDH2 3′ UTR was amplified by PCR using the following primer pairs that contain the NOT1 restriction site (F: 5′-ATGCGCGGCCGC TGTAGCAGTTAAAAAGAGGTAGGTG and R:5′-ATGCGCGGCCGC AACTTTGTAGTCTACTAGCACAGTG. PCR product and PGL2 basic (Promega, #E1641) were digested with the NOT1 restriction enzyme and ligated. The orientation of the CDH2 3′UTR was confirmed by DNA sequencing. 10^4^ HEK293 cells were plated and transfected after 24 hr with 1:1:1 mass ratio of 759 sgRNA, MS2-P65-HSF1-Hygro and dCAS9-VP64-GFP using Lipofectamine 2000 (Life Technologies) according to the manufacturer’s instructions. Culture medium was replaced with fresh medium after 24 hours. At 48 hours after transfection, pGL2-CDH2 3′ UTR and pGL4.74 (Promega cat #E6921) were co-transfected in a 4:1 mass ratio. Firefly and Renilla luciferase activity were measured after 24 hours using the Dual Glo Luciferase Reporter Assay System (Promega #E1910) and a luminometer (Cytation 3 cell imaging multi-mode Reader, BioTek Instruments).

### Statistical analysis

sRNAseq and mRNAseq data analysis were performed using the R statistical language. Differential analysis was performed using the Bioconductor package edgeR and considered FDR ≤ 0.2 throughout the manuscript as statistically significant. All RT-PCR results are reported as mean ± standard error of the mean (SEM). Two-tailed Student’s t tests, one-way ANOVA and two-way ANOVA with appropriate post hoc tests and corrections were performed using GraphPad Prism 7 software. *P* ≤ 0.05 was considered statistically significant as specified in the relevant tables and figure legends.

## Supplementary information

Supplementary Figures.

Supplementary table S1.

Supplementary table S2.

Supplementary table S3.

Supplementary table S4.

## Data Availability

All the RNAseq and sRNAseq datasets generated during the current study are available in the Sequence Read Archive (SRA) repository (http://www.ncbi.nlm.nih.gov/sra/), under the accession # PRJNA603843.
