# Supplementary material for: Chromosome 19 microRNA cluster enhances cell reprogramming by inhibiting epithelial-to-mesenchymal transition
Source: Sci Rep. 2020 Feb 20;10:3029. doi: 10.1038/s41598-020-59812-8 (PMC7033247; doi:10.1038/s41598-020-59812-8)
Supplement: Supplementary file 1 — Supplementary Figures. [file 41598_2020_59812_MOESM1_ESM.docx]

**Supplementary Material**

**Chromosome 19 microRNA cluster enhances cell reprogramming by inhibiting epithelial-to-mesenchymal transition**

Ezinne F. Mong^1^, Ying Yang^1^, Kemal M. Akat^2,3^, John Canfield^1^, Jeffrey VanWye^1^, John Lockhart^1^, John C. M. Tsibris^4^, Frederick Schatz^4^, Charles J. Lockwood^4^, Thomas Tuschl^2^, Umit A. Kayisli^4^ and Hana Totary-Jain^1*^

^1^ Department of Molecular Pharmacology & Physiology, ^4^ Department of Obstetrics and Gynecology, Morsani College of Medicine, Tampa, Florida, U.S.A.

^2^ Howard Hughes Medical Institute and Laboratory for RNA Molecular Biology, The Rockefeller University, New York, New York, USA

^3^ Vanderbilt Heart and Vascular Institute, Vanderbilt University Medical Center, Nashville, TN, USA

*Correspondence should be addressed to H.T.J. Tel: +1 813-974-6821; Fax: +1 813-974-3079; Email: [totaryjainh@usf.edu](mailto:totaryjainh@usf.edu)

**Supplementary Figure s1**

**
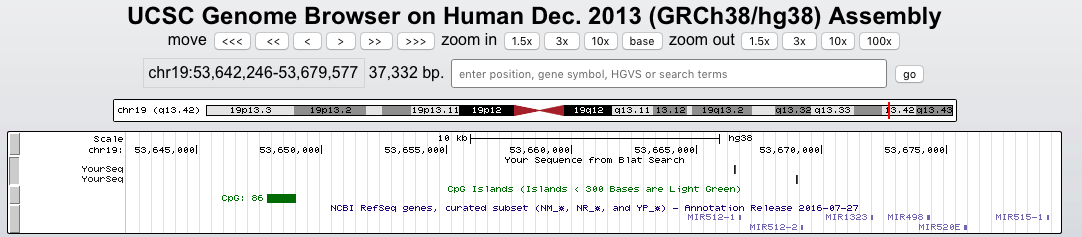
**

a

b

**
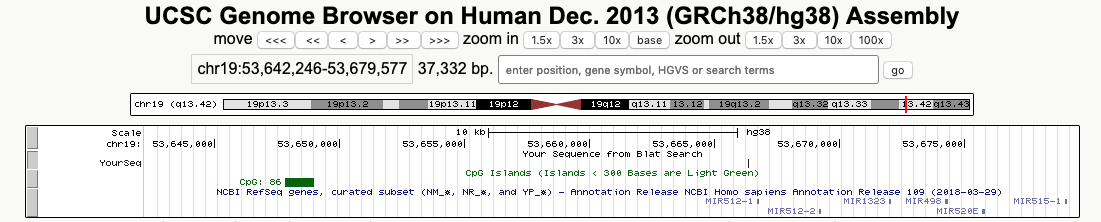
**

**Supplementary Figure s1.** Location of the gRNA#759 (a) and gRNA#620 (b) relative to the upstream CpG island (Green rectangle) and the first miRNAs of the C19MC cluster as viewed on the UCSC Genome Browser screen.

**Supplementary Figure s2**

**
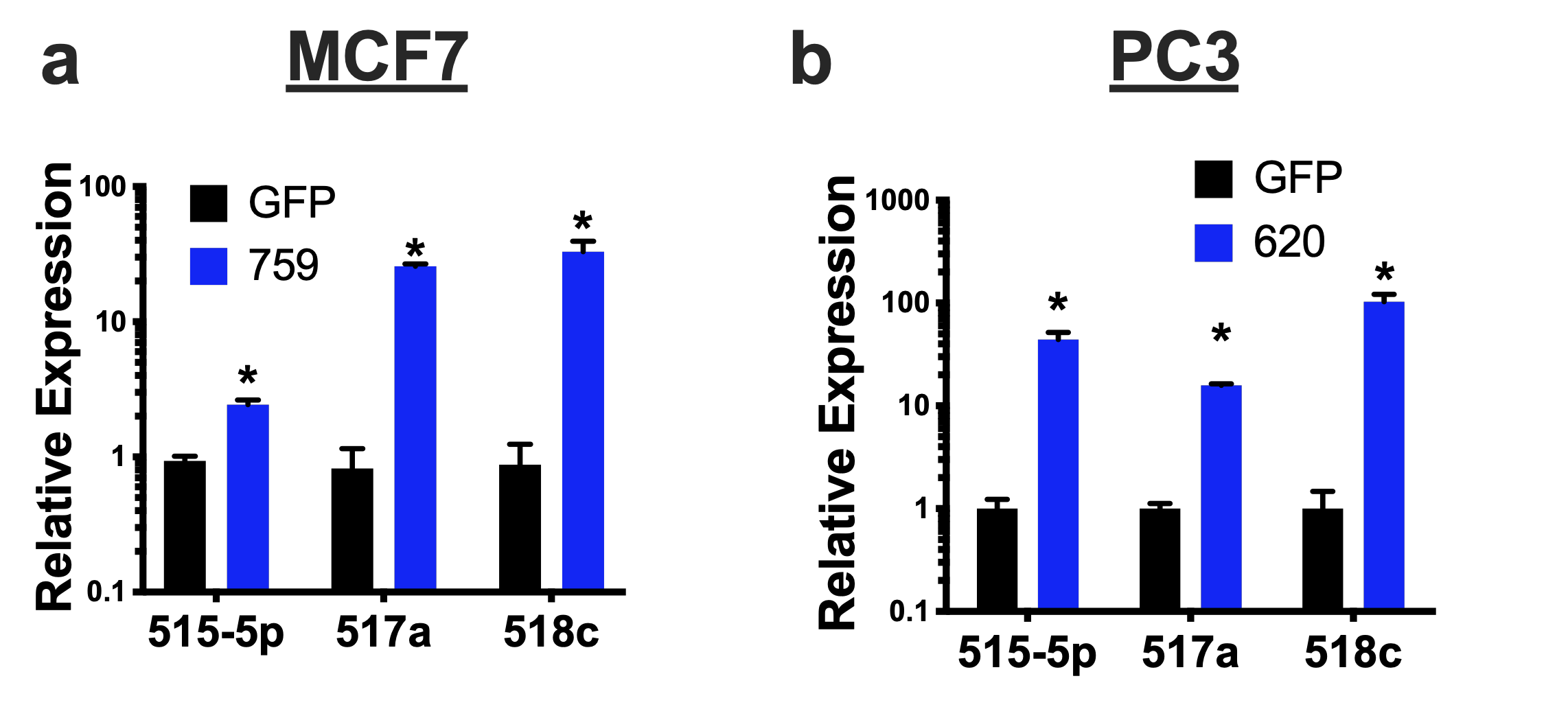
**

**Supplementary Figure s2. (a-b)** qRT-PCR analysis of 3 representative miRNAs of the C19MC cistron normalized to U18, 72h after transfection of MCF7 cells with 759-sgRNA/SAM (a) or PC3 cells with 620-sgRNA/SAM (b) compared to GFP transfected control cells. Graph represents means ± SEM. * *p* < 0.05 compared to GFP transfected cells by Multiple t-tests with Holm-Sidak correction.

**Supplementary Figure s3**

**
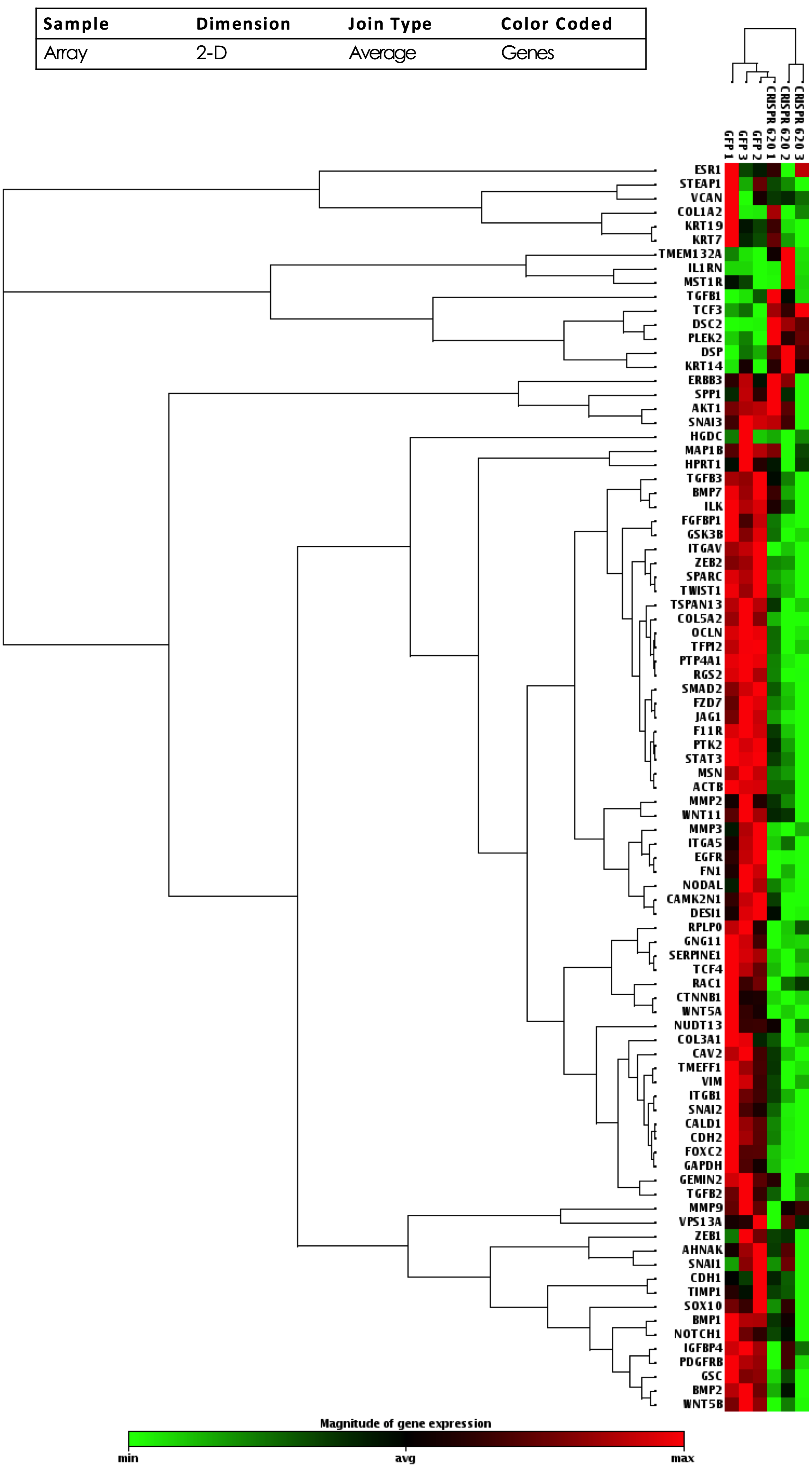
**

**Supplementary Figure s3.** Heatmap showing non-hierarchical clustering of differentially expressed EMT genes in 620-sgRNA/SAM compared to control GFP transfected cells.

**Supplementary Figure s4**

**
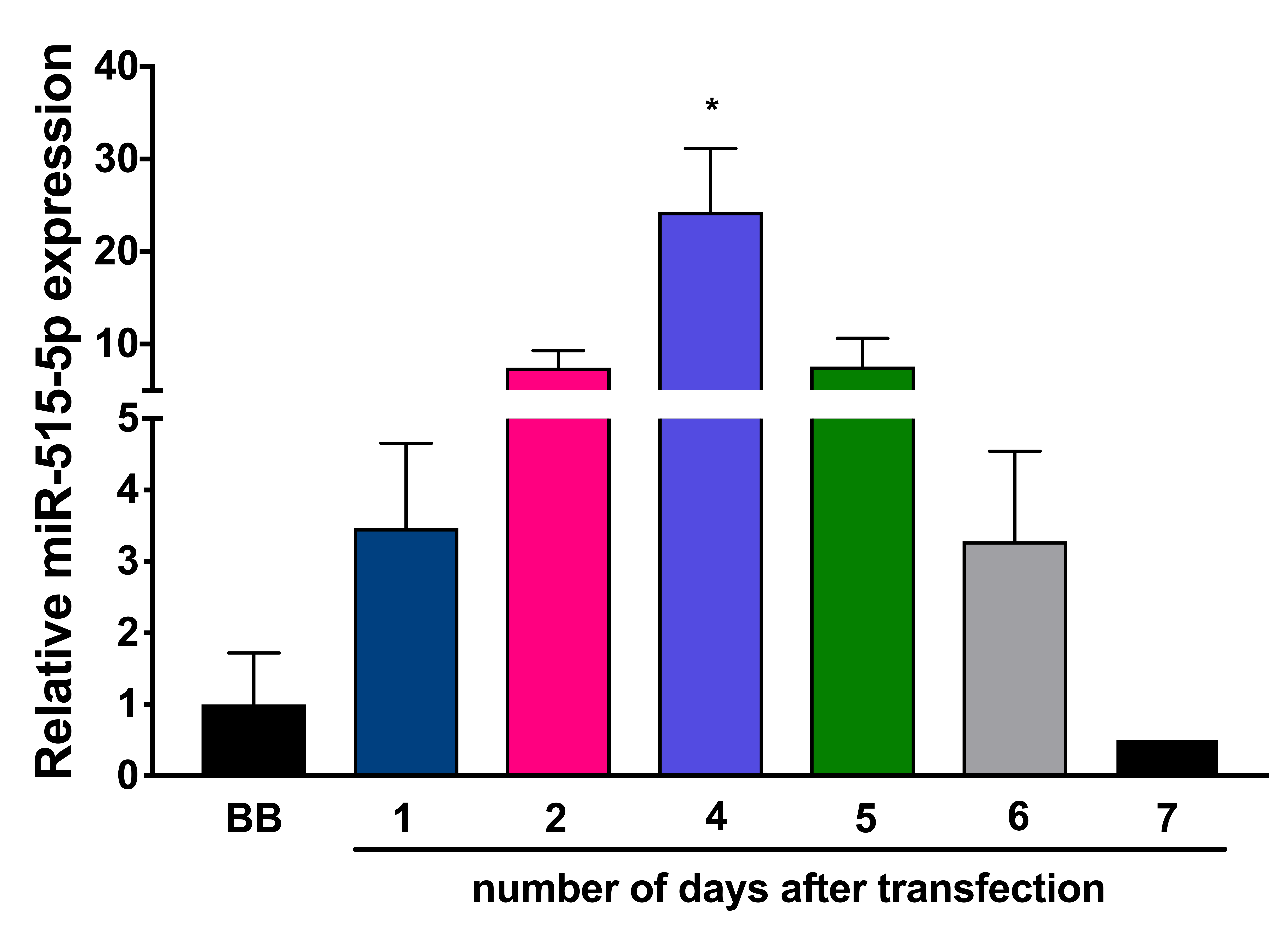
**

**Supplementary Figure s4.** qRT-PCR analysis of miR-515-5p in NHDFs transfected with 759-sgRNA/SAM 1, 2, 4, 5, 6, and 7 days post transfection. Graph represents means ± SEM. * *p* < 0.05 versus backbone (ANOVA with Dunnett’s post-hoc).
