# Supplementary material for: Chromosome 19 microRNA cluster enhances cell reprogramming by inhibiting epithelial-to-mesenchymal transition
Source: Sci Rep. 2020 Feb 20;10:3029. doi: 10.1038/s41598-020-59812-8 (PMC7033247; doi:10.1038/s41598-020-59812-8)
Supplement: Supplementary file 2 — Supplementary table S1. [file 41598_2020_59812_MOESM2_ESM.pdf]

Sheet "miRNAs 759-GFP exp1": Small RNA sequencing data analysis of miRNAs differentially expressed in HEK293 cells transfected with 759 sgRNA/SAM compared to GFP transfected control

Sheet "miRNAs 759-GFP exp2": Small RNA sequencing data analysis of miRNAs differentially expressed in HEK293 cells transfected with 759 sgRNA/SAM compared to GFP transfected control

Sheet "miRNAs 620-GFP": Small RNA sequencing data analysis of miRNAs differentially expressed in HEK293 cells transfected with 620 sgRNA/SAM compared to GFP transfected control

Sheet "Cistrons 759-GFP exp2": Small RNA sequencing data analysis of miRNA cistrons differentially expressed in HEK293 cells transfected with 759 sgRNA/SAM compared to GFP transfected control

Sheet "Cistrons 620-GFP": Small RNA sequencing data analysis of miRNA cistrons differentially expressed in HEK293 cells transfected with 620 sgRNA/SAM compared to GFP transfected control

	Normalized relative Frequency				
miRNA	CRISPR/Cas9	GFP	Fold change	PValue	FDR
525-3p(1)	0.004799%	0.000005%	900.5	6.0011E-04	9.1854E-03
520e-3p(1)	0.003716%	0.000005%	692.4	4.9271E-05	1.3198E-03
518c-3p(1)	0.024946%	0.000040%	631.2	1.1291E-06	2.3324E-04
520a-3p(1)	0.003212%	0.000005%	597.8	9.0259E-05	2.0759E-03
519e-5p(1)	0.003097%	0.000005%	568.3	5.9996E-04	9.1854E-03
526b-5p(1)	0.017139%	0.000040%	432.9	3.5435E-06	2.9529E-04
517STAR(3)	0.002118%	0.000006%	379.7	3.0914E-04	5.8157E-03
512-3p(2)	0.082218%	0.000245%	335.8	1.2440E-06	2.3324E-04
515(2)	0.046566%	0.000144%	322.4	2.6157E-05	8.5295E-04
515STAR(2)	0.017935%	0.000057%	315.1	1.2836E-04	2.6741E-03
520d-3p(1)	0.001744%	0.000006%	306.1	1.8208E-04	3.6907E-03
885(1)	0.001702%	0.000006%	301.8	4.7969E-04	7.8258E-03
519c-3p(1)	0.019011%	0.000073%	260.2	1.0356E-06	2.3324E-04
518b-3p(1)	0.014500%	0.000057%	254.5	6.3584E-06	3.9740E-04
512-5p(2)	0.019217%	0.000076%	254.1	2.0160E-06	2.9529E-04
518a-1-3p(1)	0.001476%	0.000006%	251.1	1.1268E-04	2.4146E-03
518a-2-3p(1)	0.001476%	0.000006%	251.1	1.1268E-04	2.4146E-03
519b-3p(1)	0.005728%	0.000023%	249.8	2.4825E-06	2.9529E-04
498(1)	0.021764%	0.000093%	234.1	3.3188E-06	2.9529E-04
520e-5p(1)	0.001279%	0.000006%	214.3	9.1338E-05	2.0759E-03
525-5p(1)	0.018235%	0.000090%	202.2	2.8213E-06	2.9529E-04
519e-3p(1)	0.001049%	0.000006%	168.5	3.1017E-04	5.8157E-03
517a(2)	0.037037%	0.000224%	165.6	1.7670E-05	6.9748E-04
518d-3p(1)	0.006366%	0.000039%	162.8	9.1901E-06	4.6112E-04
518c-5p(1)	0.000991%	0.000006%	161.5	3.4919E-04	6.2355E-03
518d-5p(5)	0.011081%	0.000071%	155.1	9.2224E-06	4.6112E-04
498STAR(1)	0.000943%	0.000006%	150.3	4.7998E-04	7.8258E-03
524STAR(1)	0.000936%	0.000006%	148.0	2.7901E-03	3.6984E-02
519d(1)	0.020602%	0.000141%	145.9	2.5891E-05	8.5295E-04
1323(1)	0.037557%	0.000260%	144.3	5.6153E-07	2.3324E-04
518b-5p(1)	0.000905%	0.000006%	142.9	3.2734E-04	5.9879E-03
526a-1-3p(1)	0.000849%	0.000006%	131.5	3.0958E-04	5.8157E-03
520f-5p(1)	0.000829%	0.000007%	126.2	4.0502E-04	6.9956E-03
1270(2)	0.000784%	0.000006%	123.3	3.4036E-03	4.4012E-02
520g-3p(2)	0.015097%	0.000127%	119.0	9.9301E-06	4.6547E-04
518e-5p(5)	0.013503%	0.000122%	110.8	5.5705E-06	3.7980E-04
524(1)	0.049101%	0.000467%	105.3	4.4260E-05	1.2767E-03
520f-3p(1)	0.063792%	0.000620%	102.9	1.0864E-05	4.7930E-04
516b(2)	0.020732%	0.000208%	99.9	2.3803E-05	8.5010E-04
520a-5p(1)	0.035758%	0.000377%	94.8	1.9058E-05	7.1466E-04
519dSTAR(1)	0.000657%	0.000007%	94.4	6.5445E-04	9.8168E-03
520b-3p(2)	0.013027%	0.000142%	91.8	4.8170E-05	1.3198E-03
526b-3p(1)	0.005214%	0.000058%	90.3	1.4756E-05	6.1482E-04

521-2STAR(1)	0.000635%	0.000007%	89.9	3.5900E-03	4.5636E-02
517b(1)	0.008088%	0.000090%	89.7	5.4469E-06	3.7980E-04
523-3p(1)	0.004434%	0.000057%	77.7	7.6582E-06	4.4182E-04
520g-5p(2)	0.001844%	0.000025%	73.2	7.4178E-05	1.7946E-03
518f-3p(1)	0.012531%	0.000187%	67.0	3.6687E-05	1.1006E-03
520d-5p(1)	0.025045%	0.000432%	58.0	8.8336E-04	1.2500E-02
516STAR(4)	0.000433%	0.000008%	53.1	5.0960E-03	5.9718E-02
518e-3p(1)	0.002287%	0.000061%	37.4	8.3481E-04	1.2277E-02
519a-1-5p(1)	0.003783%	0.000108%	35.1	6.9123E-05	1.7281E-03
1283-2-3p(1)	0.000763%	0.000029%	26.6	1.8499E-03	2.5624E-02
1283-5p(2)	0.007164%	0.000342%	20.9	5.3322E-05	1.3790E-03
519a-2-5p(2)	0.002844%	0.000137%	20.7	3.4599E-05	1.0812E-03
518a-5p(3)	0.007050%	0.000397%	17.7	4.1041E-04	6.9956E-03
133a(2)	0.002260%	0.000205%	11.0	6.6827E-03	7.5940E-02
519a-3p(2)	0.005371%	0.000487%	11.0	4.9883E-03	5.9384E-02
1323STAR(1)	0.000384%	0.000036%	10.5	1.8648E-02	1.8163E-01
522-3p(1)	0.002773%	0.000328%	8.5	2.8108E-03	3.6984E-02
124STAR(3)	0.000448%	0.000057%	7.9	2.0200E-02	1.8937E-01
489(1)	0.001916%	0.000281%	6.8	5.6026E-04	8.9403E-03
516a(2)	0.012263%	0.001935%	6.3	1.8791E-03	2.5624E-02
521(2)	0.005693%	0.000966%	5.9	1.2654E-02	1.3182E-01
539(1)	0.001254%	0.000216%	5.8	4.3859E-03	5.3055E-02
449c(1)	0.009596%	0.001836%	5.2	4.1839E-03	5.2298E-02
135bSTAR(1)	0.000513%	0.000100%	5.1	9.9998E-03	1.0869E-01
449b(1)	0.003243%	0.000639%	5.1	8.5672E-04	1.2357E-02
410(1)	0.001409%	0.000354%	4.0	1.8968E-02	1.8238E-01
377(1)	0.002802%	0.000709%	4.0	7.5486E-03	8.3257E-02
653-3p(1)	0.001785%	0.000486%	3.7	1.2314E-02	1.3008E-01
1185-1-3p(1)	0.001477%	0.000433%	3.4	1.5380E-02	1.5380E-01
708STAR(1)	0.013535%	0.004130%	3.3	4.3404E-03	5.3055E-02
139(1)	0.007370%	0.002283%	3.2	1.2065E-02	1.2927E-01
141(1)	0.213092%	0.066232%	3.2	1.3202E-02	1.3564E-01
449a(1)	0.014223%	0.005173%	2.7	1.5178E-02	1.5380E-01
7c(1)	0.060556%	0.122025%	-2.0	1.9358E-02	1.8377E-01
92a-1STAR(1)	0.005018%	0.011656%	-2.3	1.5845E-02	1.5637E-01
588-3p(1)	0.000037%	0.000430%	-11.7	7.4211E-03	8.3072E-02
2116STAR(1)	0.000025%	0.000343%	-13.7	5.9438E-03	6.8582E-02

miRNA	Normalized frequency (%) g759	GFP	Linear fold chan	P value	FDR
520f-3p(1)	0.000396	0.000000	7984.5	7.26E-11	2.18861E-08
520a-5p(1)	0.000239	0.000000	5979.6	1.48E-10	2.23688E-08
498(1)	0.000226	0.000000	4892.1	4.52E-11	2.18861E-08
516b(2)	0.000193	0.000000	4582	1.33E-10	2.23688E-08
520d-5p(1)	0.000179	0.000000	4166.6	1.00015E-09	7.53866E-08
518b-3p(1)	0.000147	0.000000	3693.9	5.90E-10	5.93238E-08
524(1)	0.000155	0.000000	3476	2.07E-10	2.49705E-08
520b-3p(2)	0.000152	0.000000	3408.9	2.54897E-09	1.70781E-07
515(2)	0.000143	0.000000	3329.4	1.14243E-07	3.62572E-06
512-5p(2)	0.000162	0.000000	3190.5	1.81385E-08	8.41349E-07
518c-3p(1)	0.000138	0.000000	3183.4	7.70E-10	6.63031E-08
519c-3p(1)	0.000126	0.000000	2905.9	3.9695E-09	2.39361E-07
526b-5p(1)	0.000099	0.000000	2694	2.15827E-08	9.29598E-07
518e-5p(5)	0.000102	0.000000	2626.8	7.31022E-09	4.00733E-07
520g-3p(2)	0.000142	0.000000	2419	7.32396E-08	2.45353E-06
518f-3p(1)	0.000096	0.000000	2383.2	1.59155E-08	7.99754E-07
520a-3p(1)	0.000081	0.000000	2280.1	4.81176E-08	1.70676E-06
1283-5p(2)	0.000075	0.000000	1847.2	3.20844E-07	9.67345E-06
518d-5p(5)	0.000083	0.000000	1790.4	2.37912E-08	9.56406E-07
519b-3p(1)	0.000058	0.000000	1594.4	7.00288E-05	0.00162413
517b(1)	0.000069	0.000000	1138.2	2.03371E-05	0.000490532
525-3p(1)	0.000055	0.000000	1099.2	3.57043E-06	9.36074E-05
512-3p(2)	0.001995	0.000002	913.1	1.60715E-06	4.61482E-05
515STAR(2)	0.000064	0.000000	837.2	0.000281939	0.004594851
139STAR(1)	0.000035	0.000000	837.2	0.000338303	0.005368338
526b-3p(1)	0.000028	0.000000	750.5	0.005200262	0.04072413
519a-1-5p(1)	0.000027	0.000000	704.4	0.000137822	0.00251838
518e-3p(1)	0.000036	0.000000	650	0.000496518	0.007485007
520d-3p(1)	0.000033	0.000000	642.6	0.000560511	0.008047341
523-3p(1)	0.000023	0.000000	628.4	0.009877933	0.065454874
654(1)	0.000022	0.000000	585.5	0.001040156	0.013066963
520e-5p(1)	0.000016	0.000000	489.9	0.007027216	0.051053145
514a(3)	0.000014	0.000000	434.3	0.007405478	0.053160751
518c-5p(1)	0.000016	0.000000	370.7	0.004817322	0.040345071
519a-2-5p(2)	0.000015	0.000000	338.3	0.012346611	0.077552152
518a-1-3p(1)	0.000018	0.000000	336.1	0.001098684	0.013250128
518a-2-3p(1)	0.000018	0.000000	336.1	0.001098684	0.013250128
524STAR(1)	0.000012	0.000000	302.9	0.00797213	0.056555226
3934(1)	0.000015	0.000000	301	0.013963396	0.085049775
525-5p(1)	0.000145	0.000001	278.7	2.00933E-06	5.5074E-05
518d-3p(1)	0.000014	0.000000	277.9	0.009307942	0.062363209
520e-3p(1)	0.000027	0.000000	264.3	0.001685811	0.018152572
1270(2)	0.000018	0.000000	230.2	0.002207887	0.021710557

1294(1)	0.000015	0.000000	212.1	0.010678477	0.069237866
498STAR(1)	0.000008	0.000000	174.6	0.028199487	0.132811174
1323(1)	0.000392	0.000002	160.2	0.000122742	0.002467106
526a-1-3p(1)	0.000006	0.000000	139.4	0.028666069	0.132966457
519e-5p(1)	0.000009	0.000000	111.9	0.035222489	0.152315518
519dSTAR(1)	0.000006	0.000000	111	0.031210316	0.141502411
20bSTAR(1)	0.000009	0.000000	109.5	0.025375577	0.125373646
517a(2)	0.000377	0.000005	80.6	0.000178041	0.003157606
516a(2)	0.000162	0.000003	57.7	0.000106173	0.002286509
518a-5p(3)	0.000077	0.000002	47.4	0.000782161	0.010253111
302a-3p(1)	0.000453	0.000010	46	0.010429265	0.068357028
519a-3p(2)	0.000042	0.000001	42.5	0.011125417	0.071368366
302a-5p(1)	0.000325	0.000008	42	0.014199521	0.085623114
519d(1)	0.000110	0.000003	37.2	0.002180385	0.021710557
381(1)	0.000037	0.000003	10.6	0.03718249	0.156790502
139(1)	0.000090	0.000009	9.8	0.001529668	0.016948117
29b-2STAR(1)	0.000057	0.000006	9.5	0.030270512	0.138465369
522-3p(1)	0.000066	0.000008	8.1	0.032752039	0.145216761
1(2)	0.000084	0.000012	6.9	0.006282099	0.047351323
135b(1)	0.000363	0.000054	6.7	0.000244558	0.004096346
146b(1)	0.001193	0.000261	4.6	3.31383E-08	1.2489E-06
708STAR(1)	0.000082	0.000018	4.5	0.004891279	0.040403305
215(1)	0.000059	0.000019	3.1	0.0189531	0.10506652
449c(1)	0.000064	0.000021	3.1	0.038884353	0.161053152
203(1)	0.000210	0.000070	3	0.000626416	0.008784397
708(1)	0.000317	0.000111	2.9	0.006100762	0.046566574
455-3p(1)	0.000135	0.000049	2.7	0.002115193	0.021617988
1292(1)	0.000063	0.000025	2.5	0.043775369	0.175976983
103(2)	0.032019	0.013164	2.4	0.000707122	0.009475432
134(1)	0.000127	0.000055	2.3	0.013842295	0.085049775
455-5p(1)	0.000261	0.000116	2.3	0.022553001	0.116234699
17STAR(1)	0.005708	0.002817	2	0.000194661	0.003353734
141(1)	0.000650	0.000328	2	0.003131305	0.029048874
200c(1)	0.000336	0.000165	2	0.005174341	0.04072413
33a(1)	0.000441	0.000902	-2	0.00384693	0.034113216
345(1)	0.000288	0.000579	-2	0.00414025	0.035665294
505(1)	0.000351	0.000689	-2	0.006905101	0.051053145
1296(1)	0.000193	0.000395	-2	0.016215563	0.094019084
1305(1)	0.000058	0.000117	-2	0.018576054	0.104685613
181b(2)	0.001079	0.002291	-2.1	0.000134802	0.00251838
301b(1)	0.000252	0.000555	-2.2	0.000395244	0.006111079
218(2)	0.003804	0.008706	-2.3	0.001009974	0.012957748
15bSTAR(1)	0.000093	0.000213	-2.3	0.001725033	0.018249033
30b(1)	0.000728	0.001694	-2.3	0.002232263	0.021710557
99a(1)	0.001553	0.003734	-2.4	0.000117082	0.002434503

188(1)	0.000080	0.000197	-2.5	0.021394675	0.113166572
577(1)	0.000027	0.000066	-2.5	0.025573729	0.125373646
34aSTAR(1)	0.000065	0.000169	-2.6	0.006947865	0.051053145
15a(1)	0.001584	0.004300	-2.7	0.000512216	0.007533317
25(1)	0.007010	0.020345	-2.9	1.53504E-05	0.000385678
491(1)	0.000029	0.000083	-2.9	0.035590065	0.152315518
32(1)	0.001033	0.003237	-3.1	0.001545848	0.016948117
29b(2)	0.000641	0.001967	-3.1	0.016654737	0.095645775
19aSTAR(1)	0.000018	0.000058	-3.3	0.025282484	0.125373646
365(2)	0.000152	0.000520	-3.4	0.021589755	0.113205409
500-5p(2)	0.000024	0.000086	-3.6	0.005101252	0.04072413
362-3p(1)	0.000039	0.000161	-4.1	0.001364972	0.015828421
1277-5p(1)	0.000032	0.000132	-4.2	0.004061608	0.035494922
450b(1)	0.000065	0.000277	-4.3	8.58201E-05	0.00191665
7a-1STAR(2)	0.000024	0.000111	-4.6	0.000133916	0.00251838
296-5p(1)	0.000052	0.000268	-5.1	0.00070522	0.009475432
31-3p(1)	0.000002	0.000045	-21.1	0.023101731	0.117715232
33b(1)	0.000007	0.000198	-28.3	0.004721154	0.040096561
133a(2)	0.000001	0.000025	-31	0.023712225	0.119153929
4473-5p(1)	0.000000	0.000007	-101.5	0.027815896	0.132811174
653-3p(1)	0.000000	0.000010	-137.8	0.020452653	0.10914115
210STAR(1)	0.000000	0.000009	-157.1	0.019969962	0.107516851
1269STAR(1)	0.000000	0.000017	-161.4	0.015524986	0.091780066
605-5p(1)	0.000000	0.000012	-168.8	0.018383858	0.104579869
26a-2STAR(1)	0.000000	0.000010	-183.8	0.035616066	0.152315518
1537(1)	0.000000	0.000009	-194.2	0.033240003	0.146304538
561STAR(1)	0.000000	0.000014	-230	0.008737491	0.059871674
942STAR(1)	0.000000	0.000012	-234	0.031753679	0.141833101
144(1)	0.000000	0.000019	-369.6	0.0084048	0.05825396
491STAR(1)	0.000000	0.000019	-502.6	0.005074749	0.04072413
205(1)	0.000000	0.000020	-549.9	0.002101957	0.021617988

miRNA	Normalized frequency (%) g620	GFP	Linear fold char	P value	FDR
520f-3p(1)	0.000396	0.000000	6039	1.00E-10	3.02644E-08
498(1)	0.000226	0.000000	3099.2	7.96E-11	3.02644E-08
520g-3p(2)	0.000142	0.000000	2587	6.74946E-08	2.71328E-06
512-5p(2)	0.000162	0.000000	2532.6	2.39758E-08	1.31431E-06
520a-5p(1)	0.000239	0.000000	2465	4.45E-10	5.37235E-08
516b(2)	0.000193	0.000000	2228.4	3.29E-10	5.37235E-08
520d-5p(1)	0.000179	0.000000	2141.2	2.29328E-09	1.72856E-07
524(1)	0.000155	0.000000	1992.2	4.20E-10	5.37235E-08
520b-3p(2)	0.000152	0.000000	1970.5	5.05537E-09	3.3871E-07
515(2)	0.000143	0.000000	1713.5	2.53311E-07	8.98508E-06
518c-3p(1)	0.000138	0.000000	1678.3	1.74351E-09	1.61023E-07
519c-3p(1)	0.000126	0.000000	1545.3	8.84844E-09	5.33561E-07
518b-3p(1)	0.000147	0.000000	1505.5	1.86926E-09	1.61023E-07
515STAR(2)	0.000064	0.000000	1384.8	0.000177616	0.00428409
517b(1)	0.000069	0.000000	1279.6	1.79433E-05	0.000470426
518d-5p(5)	0.000083	0.000000	1135.2	4.20203E-08	1.9491E-06
518f-3p(1)	0.000096	0.000000	982	4.91311E-08	2.11614E-06
518e-5p(5)	0.000102	0.000000	955.8	2.69621E-08	1.35485E-06
525-3p(1)	0.000055	0.000000	805.4	5.06652E-06	0.000145481
526b-5p(1)	0.000099	0.000000	773.2	1.07838E-07	4.06413E-06
1283-5p(2)	0.000075	0.000000	767.7	9.27683E-07	2.94417E-05
520e-3p(1)	0.000027	0.000000	666.2	0.000786173	0.01281249
518e-3p(1)	0.000036	0.000000	589.2	0.000540294	0.009872636
520a-3p(1)	0.000081	0.000000	556.5	2.95045E-07	9.884E-06
512-3p(2)	0.001994	0.000004	553.5	2.7574E-06	8.31355E-05
520d-3p(1)	0.000033	0.000000	483.5	0.000713306	0.011947876
519b-3p(1)	0.000058	0.000000	417.9	0.000271778	0.005911968
139STAR(1)	0.000035	0.000000	381.1	0.000677964	0.011680359
1294(1)	0.000015	0.000000	276.1	0.009053616	0.084206932
518a-1-3p(1)	0.000018	0.000000	275.4	0.0012918	0.019473882
518a-2-3p(1)	0.000018	0.000000	275.4	0.0012918	0.019473882
519a-1-5p(1)	0.000026	0.000000	197.8	0.000464161	0.008746529
3934(1)	0.000015	0.000000	194.9	0.017888272	0.139174728
526b-3p(1)	0.000028	0.000000	192.3	0.012702609	0.110428093
518d-3p(1)	0.000014	0.000000	189	0.011825443	0.10642899
20bSTAR(1)	0.000009	0.000000	184.5	0.01868913	0.142652471
654(1)	0.000022	0.000000	165.6	0.002895518	0.034919945
523-3p(1)	0.000023	0.000000	161.5	0.0220479	0.160179324
518c-5p(1)	0.000016	0.000000	159.7	0.008508845	0.084112022
519a-2-5p(2)	0.000015	0.000000	149.8	0.019814179	0.147505553
525-5p(1)	0.000145	0.000001	142.4	5.85391E-06	0.00016045
524STAR(1)	0.000012	0.000000	87	0.018002701	0.139174728
1323(1)	0.000390	0.000005	81.5	0.000302583	0.00629163

517a(2)	0.000375	0.000007	51.3	0.000354108	0.006887972
516a(2)	0.000161	0.000005	34.2	0.000274519	0.005911968
519d(1)	0.000109	0.000004	29.1	0.003187193	0.036959178
518a-5p(3)	0.000076	0.000003	23.4	0.002720933	0.034431258
184(1)	0.000046	0.000003	14.7	0.008697465	0.084206932
1(2)	0.000088	0.000008	11.7	0.001288371	0.019473882
135b(1)	0.000377	0.000040	9.5	5.59821E-05	0.001406551
522-3p(1)	0.000067	0.000007	9.4	0.024556504	0.174206729
338-3p(1)	0.000865	0.000111	7.8	0.002740797	0.034431258
449c(1)	0.000072	0.000013	5.6	0.004016519	0.045697377
139(1)	0.000083	0.000017	4.9	0.013902921	0.118076918
200a(1)	0.000062	0.000014	4.4	0.003160173	0.036959178
449a(1)	0.000166	0.000046	3.6	0.001911954	0.026388436
200b(1)	0.000099	0.000028	3.6	0.004406716	0.049208325
455-3p(1)	0.000143	0.000041	3.5	0.000354007	0.006887972
338-5p(1)	0.000162	0.000057	2.8	0.001925524	0.026388436
203(1)	0.000194	0.000086	2.2	0.006032226	0.063814601
25(1)	0.008910	0.018445	-2.1	0.000651094	0.01154735
450b(1)	0.000111	0.000231	-2.1	0.015469931	0.127837862
1296(1)	0.000183	0.000405	-2.2	0.009127112	0.084206932
7a-1STAR(2)	0.000042	0.000093	-2.2	0.016869372	0.135629752
99a(1)	0.001615	0.003673	-2.3	0.000218399	0.005065184
1305(1)	0.000054	0.000122	-2.3	0.007837624	0.078768119
24-2STAR(1)	0.000066	0.000173	-2.6	0.00204908	0.02739748
21STAR(1)	0.000171	0.000457	-2.7	0.001431262	0.021050028
577(1)	0.000023	0.000070	-3	0.009092982	0.084206932
296-5p(1)	0.000066	0.000254	-3.9	0.002836218	0.034902846
4473-3p(1)	0.000000	0.000013	-329	0.012819182	0.110428093

	Normalized frequency (%)				
**miRNA cistron**	**g620**	**GFP**	**near fold change**	**P value**	**FDR**
1270-1(1)	0.000959	0.000003	300.3	0.008046674	0.064610062
1270-2(1)	0.000959	0.000003	300.3	0.008046674	0.064610062
498(46)	0.693319	0.002436	284.6	7.20E-14	1.97E-11
1294(1)	0.001603	0.000007	217.7	0.007712637	0.064610062
937(1)	0.001019	0.000009	110.3	0.021666308	0.134429594
302a(5)	0.113243	0.008012	14.1	0.006357459	0.061985226
139(1)	0.012621	0.000915	13.8	0.000211924	0.009642563
135b(1)	0.037467	0.005622	6.7	0.000132641	0.009031184
146b(1)	0.123156	0.025508	4.8	3.67364E-09	5.01452E-07
708(1)	0.040645	0.012593	3.2	0.000317432	0.012379861
203(1)	0.021656	0.006905	3.1	0.000399672	0.013638817
134(43)	0.033642	0.011150	3	3.61278E-05	0.003287633
216a(3)	0.012922	0.004774	2.7	0.002320623	0.036251694
1292(1)	0.006497	0.002501	2.6	0.035569866	0.195311561
103-1(2)	3.259458	1.280146	2.5	0.000165406	0.009031184
455(1)	0.040154	0.016041	2.5	0.001699152	0.035682195
449a(3)	0.022143	0.009460	2.3	0.003834372	0.045210394
141(2)	0.100944	0.048612	2.1	0.000496621	0.01506418
33a(1)	0.046845	0.093700	-2	0.004140146	0.045210394
1305(1)	0.005938	0.011735	-2	0.019812334	0.127523804
218-1(3)	0.396363	0.852876	-2.2	0.000878749	0.022924477
31(1)	0.006206	0.014934	-2.4	0.003638362	0.045148759
577(1)	0.002711	0.006613	-2.4	0.028354078	0.16126382
579(1)	0.002299	0.006272	-2.7	0.016751396	0.115822664
32(1)	0.106061	0.332414	-3.1	0.001007669	0.022924477
491(1)	0.002903	0.010206	-3.5	0.01107445	0.08580957
1277(1)	0.005417	0.020835	-3.8	0.002077521	0.036251694
33b(1)	0.001224	0.021944	-17.9	0.00410529	0.045210394
605(1)	0.000008	0.001245	-161.3	0.0166515	0.115822664
1537(1)	0.000005	0.001003	-217.9	0.026689791	0.155027938
144(2)	0.000005	0.002482	-499.4	0.001849369	0.036062703
205(1)	0.000004	0.001936	-526.4	0.002230001	0.036251694

	Normalized frequency (%)				
**miRNA cistron**	**g620**	**GFP**	**Linear fold char**	**P value**	**FDR**
1294(1)	0.001605	0.000005	310.8	0.005949213	0.134927448
937(1)	0.001023	0.000005	211.2	0.013971577	0.181630498
**498(46)**	**0.691542**	**0.004213**	**164.1**	**2.32E-13**	**6.34E-11**
184(1)	0.004653	0.000314	14.8	0.006555121	0.134927448
135b(1)	0.038836	0.004253	9.1	3.07592E-05	0.00279909
139(1)	0.011755	0.001781	6.6	0.002666126	0.090981538
338(1)	0.101467	0.016376	6.2	0.000205292	0.011208917
449a(3)	0.025605	0.005998	4.3	3.0334E-05	0.00279909
200a(3)	0.018333	0.004706	3.9	0.000125802	0.008586004
1-1(4)	0.010001	0.002660	3.8	0.007729822	0.134927448
203(1)	0.019830	0.008730	2.3	0.005555966	0.134927448
143(2)	0.030517	0.014919	2	0.001713835	0.066839556
1305(1)	0.005516	0.012157	-2.2	0.008439253	0.134927448
1296(1)	0.018613	0.041084	-2.2	0.008758076	0.134927448
577(1)	0.002327	0.006997	-3	0.009145006	0.134927448
