# Supplementary material for: Chromosome 19 microRNA cluster enhances cell reprogramming by inhibiting epithelial-to-mesenchymal transition
Source: Sci Rep. 2020 Feb 20;10:3029. doi: 10.1038/s41598-020-59812-8 (PMC7033247; doi:10.1038/s41598-020-59812-8)
Supplement: Supplementary file 3 — Supplementary table S2. [file 41598_2020_59812_MOESM3_ESM.pdf]

Sheet "mRNA g759-GFP ": RNA sequencing data analysis of differentially expressed mRNAs in HEK293 cells transfected with 759 sgRNA/SAM compared to GFP transfected control

Sheet "mRNA 620-GFP ": RNA sequencing data analysis of differentially expressed mRNAs in HEK293 cells transfected with 620 sgRNA/SAM compared to GFP transfected control

Sheet "Differentially expressed mRNAs ": Differentially expressed mRNAs in HEK293 cells transfected with 759 sgRNA/SAM and 620 sgRNA/SAM compared to control.

Sheet "GSEA downregulated genes ": Gene set expression analysis of significantly downregulated mRNAs in HEK293 cells transfected with 759 sgRNA/SAM and 620 sgRNA/SAM compared to control.

Gene		Linear Fold Change	P Value	FDR	Transcripts per million (TPM) TestMean	TestStdDev	TestMin	TestMax	RefMean	RefStdDev	RefMin	RefMax
ENSG00000176371	ZSCAN2	-1.5	3.5E-08	4.4E-07	11.185	0.795	10.303	12.193	15.414	0.767	14.750	16.133
ENSG00000196812	ZSCAN16	-1.5	5.7E-06	4.1E-05	17.496	1.723	16.514	20.068	25.203	1.730	24.242	27.797
ENSG00000196605	ZNF846	-1.5	1.5E-05	9.5E-05	3.815	0.623	3.209	4.408	5.649	0.503	5.102	6.077
ENSG00000196152	ZNF79	-1.5	1.7E-06	1.4E-05	18.591	2.123	16.501	21.470	26.885	1.586	24.753	28.591
ENSG00000182903	ZNF721	-1.5	0.00087	0.00317	21.331	5.219	13.542	24.667	32.743	3.041	29.786	35.366
ENSG00000175105	ZNF654	-1.5	6.6E-06	4.7E-05	15.957	2.967	11.639	18.388	23.680	1.691	22.131	25.144
ENSG00000196357	ZNF565	-1.5	0.00018	0.00083	2.645	0.522	2.050	3.198	3.939	0.123	3.775	4.072
ENSG00000177599	ZNF491	-1.5	5.8E-05	0.00031	3.145	0.196	2.988	3.394	4.871	0.361	4.406	5.157
ENSG00000196263	ZNF471	-1.5	1.3E-05	8.7E-05	8.908	1.427	6.867	10.167	13.460	1.692	11.612	14.896
ENSG00000083817	ZNF416	-1.5	1.2E-06	1E-05	13.636	1.224	12.487	15.079	19.544	0.244	19.259	19.748
ENSG00000186496	ZNF396	-1.5	0.00036	0.00148	1.564	0.142	1.365	1.680	2.144	0.197	1.977	2.362
ENSG00000188994	ZNF292	-1.5	0.02339	0.04988	13.493	4.618	6.585	16.198	20.790	2.262	18.546	22.738
ENSG00000149050	ZNF214	-1.5	8.7E-06	6E-05	6.355	0.696	5.647	7.031	9.118	1.019	7.883	9.954
ENSG00000121417	ZNF211	-1.5	7.2E-09	1.1E-07	18.941	0.859	17.863	19.682	27.838	2.177	24.584	29.056
ENSG00000096654	ZNF184	-1.5	3.7E-07	3.6E-06	15.728	2.200	12.777	18.048	23.763	1.829	22.138	25.346
ENSG00000169946	ZFPM2	-1.5	3.9E-05	0.00022	2.320	0.339	2.044	2.786	3.409	0.213	3.230	3.717
ENSG00000205189	ZBTB10	-1.5	1.1E-05	7.3E-05	24.374	4.197	18.123	27.109	36.033	2.289	33.560	37.980
ENSG00000143816	WNT9A	-1.5	4.5E-05	0.00025	4.838	0.746	4.156	5.522	6.919	0.409	6.526	7.272
ENSG00000228775	WEE2-AS1	-1.5	0.03014	0.06125	0.468	0.134	0.304	0.605	0.738	0.154	0.551	0.865
ENSG00000178201	VN1R1	-1.5	0.0021	0.00668	3.455	0.481	2.769	3.819	5.379	0.278	5.062	5.741
ENSG00000189136	UBE2Q2P1	-1.5	0.00088	0.00317	6.490	1.270	4.591	7.257	10.242	1.123	9.132	11.209
ENSG00000100271	TTLL1	-1.5	8.3E-05	0.00042	8.918	1.602	6.705	10.297	12.537	0.954	11.320	13.651
ENSG00000182463	TSHZ2	-1.5	1.7E-06	1.4E-05	2.626	0.300	2.248	2.983	3.906	0.106	3.783	3.995
ENSG00000155428	TRIM74	-1.5	0.01194	0.02844	1.588	0.227	1.369	1.834	2.546	0.368	2.060	2.955
ENSG00000071575	TRIB2	-1.5	2.6E-08	3.4E-07	13.874	1.231	12.885	15.546	19.685	0.563	18.968	20.134
ENSG00000230359	TPI1P2	-1.5	0.02483	0.05234	2.736	1.198	1.024	3.813	4.254	0.392	3.831	4.588
ENSG00000271092	TMEM56-RW	-1.5	0.02865	0.05878	1.275	0.384	0.729	1.621	1.987	0.379	1.560	2.483
ENSG00000228158	TLE1P1	-1.5	0.0004	0.00163	12.609	1.810	9.983	14.132	19.977	1.084	19.096	21.316
ENSG00000251022	THAP9-AS1	-1.5	2.8E-07	2.9E-06	31.932	4.772	25.047	36.036	49.855	5.136	44.441	54.244
ENSG00000229950	TFAP2A-AS1	-1.5	0.00348	0.01013	2.924	0.768	2.250	3.832	4.448	0.416	4.085	4.809
ENSG00000137203	TFAP2A	-1.5	1E-07	1.2E-06	4.385	0.324	4.027	4.754	6.438	0.121	6.306	6.540
ENSG00000168769	TET2	-1.5	4E-05	0.00023	6.466	1.209	4.659	7.211	9.900	0.996	8.944	10.759
ENSG00000218336	TENM3	-1.5	2.8E-09	4.8E-08	24.557	2.702	20.530	26.244	37.294	1.636	35.059	38.517
ENSG00000148737	TCF7L2	-1.5	4.1E-08	5.1E-07	10.552	0.880	9.789	11.638	16.150	0.713	15.727	17.210

ENSG00000196628	TCF4	-1.5	4.6E-07	4.5E-06	2.838	0.464	2.176	3.209	4.148	0.241	3.933	4.357
ENSG00000176896	TCEANC	-1.5	0.00027	0.00117	2.035	0.234	1.690	2.213	3.099	0.549	2.522	3.568
ENSG00000177565	TBL1XR1	-1.5	0.00018	0.00084	18.476	3.944	12.710	21.084	28.339	2.119	26.191	30.160
ENSG00000101463	SYNDIG1	-1.5	0.00018	0.00084	4.763	0.664	4.187	5.694	6.815	0.547	6.124	7.463
ENSG00000254995	STX16-NPEPI	-1.5	0.00396	0.01128	1.185	0.237	1.026	1.529	1.962	0.516	1.384	2.398
ENSG00000242866	STRC	-1.5	0.02798	0.05766	0.246	0.027	0.208	0.267	0.377	0.107	0.236	0.460
ENSG00000260589	STAM-AS1	-1.5	0.00323	0.00952	2.279	0.506	1.531	2.612	3.410	0.499	2.663	3.689
ENSG00000263465	SRSF8	-1.5	8.35E-12	2.70E-10	41.999	1.956	39.099	43.245	62.406	0.375	62.144	62.939
ENSG00000110693	SOX6	-1.5	7.6E-08	9E-07	2.729	0.415	2.177	3.114	4.199	0.210	3.910	4.355
ENSG00000145147	SLIT2	-1.5	3.2E-09	5.4E-08	87.065	8.358	76.351	95.869	127.111	3.702	123.806	130.316
ENSG00000134294	SLC38A2	-1.5	3.6E-05	0.00021	156.761	27.425	116.843	179.325	227.095	7.191	216.333	231.021
ENSG00000253598	SLC10A5	-1.5	0.03098	0.06265	0.798	0.168	0.619	1.002	1.270	0.352	0.752	1.491
ENSG00000116991	SIPA1L2	-1.5	6.91E-10	1.4E-08	13.432	1.026	11.959	14.253	19.985	1.038	18.959	20.879
ENSG00000112246	SIM1	-1.5	0.00037	0.00152	3.501	0.839	2.279	4.112	5.344	0.515	4.669	5.746
ENSG00000168779	SHOX2	-1.5	1.2E-06	1E-05	5.536	0.566	5.141	6.360	8.197	0.800	7.019	8.696
ENSG00000137872	SEMA6D	-1.5	0.0008	0.00292	1.015	0.262	0.780	1.326	1.582	0.192	1.336	1.734
ENSG00000151967	SCHIP1	-1.5	0.0011	0.00385	0.918	0.185	0.728	1.109	1.234	0.118	1.148	1.397
ENSG00000124813	RUNX2	-1.5	8.1E-05	0.00042	3.264	0.256	2.910	3.510	4.906	0.381	4.480	5.407
ENSG00000108309	RUNDC3A	-1.5	0.00448	0.01252	0.986	0.119	0.833	1.118	1.455	0.321	1.281	1.936
ENSG00000230364	RPL4P3	-1.5	0.02396	0.05086	2.604	1.113	1.859	4.252	3.564	0.125	3.385	3.651
ENSG00000185483	ROR1	-1.5	1.8E-08	2.5E-07	6.891	0.683	6.059	7.640	10.323	0.332	9.924	10.596
ENSG00000115841	RMDN2	-1.5	0.00288	0.00864	1.736	0.652	0.927	2.523	2.537	0.177	2.330	2.764
ENSG00000141314	RHBDL3	-1.5	1.6E-06	1.3E-05	5.561	0.662	4.823	6.409	7.916	0.552	7.209	8.355
ENSG00000232104	RFX3-AS1	-1.5	0.08125	0.13907	1.332	0.689	0.712	2.228	2.016	0.522	1.408	2.451
ENSG00000248643	RBM14-RBM	-1.5	0.00112	0.00392	2.338	0.183	2.097	2.541	3.679	0.375	3.170	3.962
ENSG00000122257	RBBP6	-1.5	5.5E-06	4E-05	27.596	4.936	20.314	31.225	40.743	1.511	39.434	42.051
ENSG00000123094	RASSF8	-1.5	0.00035	0.00144	20.491	4.300	14.371	24.134	29.179	2.275	26.617	31.095
ENSG00000171522	PTGER4	-1.5	2.5E-05	0.00015	2.736	0.402	2.384	3.248	4.141	0.272	3.779	4.350
ENSG00000111215	PRR4	-1.5	0.01962	0.04315	1.201	0.516	0.630	1.850	1.988	0.451	1.505	2.373
ENSG00000185532	PRKG1	-1.5	3.1E-07	3.2E-06	5.064	0.701	4.086	5.599	7.282	0.165	7.141	7.449
ENSG00000106536	POU6F2	-1.5	0.00019	0.00089	1.126	0.094	1.055	1.255	1.647	0.241	1.439	1.877
ENSG00000214783	POLR2J4	-1.5	0.0004	0.00164	1.192	0.207	0.988	1.421	1.759	0.070	1.702	1.846
ENSG00000240694	PNMA2	-1.5	1.1E-08	1.6E-07	9.830	0.865	8.844	10.796	14.470	0.666	14.050	15.465
ENSG00000136040	PLXNC1	-1.5	0.01552	0.03552	0.558	0.248	0.257	0.811	0.903	0.118	0.763	1.000
ENSG00000162407	PLPP3	-1.5	4.2E-07	4.1E-06	7.456	0.541	7.014	8.196	10.972	0.394	10.719	11.560
ENSG00000182621	PLCB1	-1.5	8.2E-05	0.00042	7.697	1.526	5.458	8.718	11.316	0.582	10.788	11.820
ENSG00000105499	PLA2G4C	-1.5	6.7E-05	0.00035	2.292	0.488	1.805	2.932	3.446	0.421	2.874	3.764
ENSG00000171033	PKIA	-1.5	1.9E-06	1.5E-05	6.491	0.938	5.202	7.441	10.010	0.373	9.507	10.293

ENSG00000145675	PIK3R1	-1.5	0.00026	0.00113	7.654	1.660	5.240	9.020	11.633	1.067	10.563	12.550
ENSG00000134853	PDGFRA	-1.5	0.00633	0.01672	0.972	0.135	0.845	1.090	1.491	0.256	1.130	1.672
ENSG00000145431	PDGFC	-1.5	1.3E-09	2.5E-08	15.361	1.332	13.639	16.890	23.141	0.615	22.218	23.451
ENSG00000168300	PCMTD1	-1.5	0.00044	0.00177	13.185	2.857	9.364	16.047	19.661	2.541	17.255	21.857
ENSG00000240764	PCDHGC5	-1.5	0.06583	0.11692	0.261	0.071	0.182	0.350	0.456	0.099	0.315	0.548
ENSG00000253731	PCDHGA6	-1.5	0.00022	0.00098	1.502	0.288	1.276	1.881	2.352	0.291	2.089	2.604
ENSG00000113212	PCDHB7	-1.5	0.00023	0.00102	2.730	0.466	2.116	3.247	3.973	0.168	3.748	4.156
ENSG00000113205	PCDHB3	-1.5	0.00223	0.00704	1.520	0.189	1.344	1.788	2.525	0.611	1.915	3.051
ENSG00000187372	PCDHB13	-1.5	1.8E-06	1.5E-05	6.522	0.663	5.627	7.095	9.596	0.117	9.439	9.684
ENSG00000120324	PCDHB10	-1.5	8.1E-06	5.7E-05	5.429	0.412	4.947	5.954	7.907	0.593	7.070	8.469
ENSG00000204962	PCDHA8	-1.5	0.00357	0.01034	0.981	0.165	0.754	1.144	1.396	0.261	1.059	1.603
ENSG00000223891	OSER1-AS1	-1.5	0.00051	0.002	5.548	0.642	4.708	6.252	7.830	0.650	7.298	8.623
ENSG00000180530	NRIP1	-1.5	4.8E-05	0.00026	9.083	1.844	6.340	10.306	13.684	1.290	12.408	14.795
ENSG00000153234	NR4A2	-1.5	0.0022	0.00696	1.594	0.312	1.300	2.022	2.207	0.203	1.907	2.333
ENSG00000185551	NR2F2	-1.5	5.2E-09	8.3E-08	18.481	1.577	16.552	19.846	26.438	1.071	25.793	28.041
ENSG00000174738	NR1D2	-1.5	4.7E-07	4.5E-06	31.949	5.435	24.193	36.293	48.209	2.575	45.026	50.302
ENSG00000168743	NPNT	-1.5	3.1E-07	3.1E-06	17.742	2.570	14.419	20.386	26.715	2.578	24.051	28.924
ENSG00000255524	NPIPB8	-1.5	0.02907	0.05954	0.949	0.135	0.864	1.150	1.341	0.130	1.269	1.535
ENSG00000173614	NMNAT1	-1.5	2.9E-06	2.2E-05	6.105	0.690	5.253	6.895	8.838	0.592	8.340	9.507
ENSG00000177453	NIM1K	-1.5	0.04421	0.08421	0.683	0.186	0.571	0.962	0.924	0.110	0.838	1.067
ENSG00000140396	NCOA2	-1.5	0.00039	0.00159	17.087	3.629	11.666	19.306	24.369	0.507	23.616	24.723
ENSG00000186310	NAP1L3	-1.5	1.5E-05	9.6E-05	4.968	0.498	4.276	5.417	7.486	0.729	6.667	8.101
ENSG00000188613	NANOS1	-1.5	1.6E-07	1.8E-06	6.633	0.219	6.442	6.884	9.617	0.394	9.111	9.929
ENSG00000281026	N4BP2L2-IT2	-1.5	0.00229	0.00719	1.132	0.334	0.780	1.547	1.702	0.259	1.448	1.925
ENSG00000166866	MYO1A	-1.5	0.00832	0.02107	0.591	0.106	0.466	0.721	0.978	0.162	0.798	1.114
ENSG00000059728	MXD1	-1.5	2E-08	2.7E-07	7.012	0.771	5.880	7.611	10.383	0.195	10.190	10.551
ENSG00000262902	MTCO1P40	-1.5	0.00094	0.00335	9.640	0.839	8.920	10.807	14.479	1.919	12.838	17.255
ENSG00000262454	MIR193BHG	-1.5	0.02879	0.05903	1.701	0.652	0.766	2.283	2.547	0.194	2.257	2.660
ENSG00000221184	MIR1254-1	-1.5	0.0536	0.09862	23.572	10.497	7.850	29.490	39.060	8.819	25.910	44.804
ENSG00000233325	MIPEPP3	-1.5	0.05737	0.10433	2.788	0.868	1.962	3.864	4.004	0.642	3.620	4.957
ENSG00000203740	METTL11B	-1.5	0.01551	0.03552	2.342	0.062	2.263	2.403	3.208	0.499	2.783	3.743
ENSG00000134138	MEIS2	-1.5	4E-08	5E-07	4.388	0.480	3.738	4.831	6.368	0.072	6.309	6.455
ENSG00000102316	MAGED2	-1.5	5.34E-10	1.1E-08	275.826	12.229	267.236	293.947	387.932	7.261	379.853	397.511
ENSG00000188993	LRRC66	-1.5	0.00196	0.00632	1.561	0.248	1.196	1.739	2.456	0.356	1.936	2.685
ENSG00000185158	LRRC37B	-1.5	2E-07	2.1E-06	6.182	0.636	5.440	6.839	9.276	0.783	8.536	9.953
ENSG00000230069	LRRC37A15P	-1.5	0.0215	0.0465	2.337	0.452	1.675	2.698	3.288	0.471	2.613	3.710
ENSG00000163431	LMOD1	-1.5	6E-05	0.00032	2.963	0.354	2.646	3.468	4.412	0.371	4.098	4.824
ENSG00000239335	LLPH-AS1	-1.5	0.01358	0.03177	5.738	1.441	3.708	6.860	8.993	0.789	7.955	9.876

ENSG00000246876	LINC02466	-1.5	0.01213	0.0288	1.579	0.259	1.240	1.860	2.356	0.633	1.535	3.080
ENSG00000267767	LINC01801	-1.5	0.00124	0.00425	3.081	0.657	2.621	4.032	4.531	0.417	3.907	4.783
ENSG00000179935	LINC00652	-1.5	0.00011	0.00055	2.365	0.254	2.059	2.644	3.343	0.035	3.295	3.379
ENSG00000245526	LINC00461	-1.5	0.01418	0.03296	0.978	0.450	0.592	1.623	1.470	0.240	1.121	1.628
ENSG00000203441	LINC00449	-1.5	0.01191	0.02839	3.406	0.526	3.098	4.191	4.656	0.322	4.391	5.044
ENSG00000224843	LINC00240	-1.5	0.03001	0.06108	0.514	0.159	0.392	0.747	0.734	0.094	0.668	0.867
ENSG00000111052	LIN7A	-1.5	1.2E-05	7.8E-05	2.813	0.408	2.222	3.096	4.158	0.585	3.316	4.555
ENSG00000172086	KRCC1	-1.5	1.1E-08	1.6E-07	34.697	3.683	29.282	37.530	51.110	2.044	49.194	52.876
ENSG00000227268	KLLN	-1.5	1.7E-05	0.00011	4.258	0.413	3.641	4.494	6.316	0.950	5.142	7.087
ENSG00000109787	KLF3	-1.5	7.2E-09	1.1E-07	29.365	2.636	25.994	31.805	43.112	1.790	40.567	44.363
ENSG00000250305	KIAA1456	-1.5	0.00038	0.00155	0.976	0.194	0.715	1.184	1.499	0.058	1.414	1.534
ENSG00000115548	KDM3A	-1.5	9.8E-09	1.5E-07	16.347	1.858	13.769	18.062	23.071	1.030	22.094	23.961
ENSG00000120696	KBTBD7	-1.5	2.1E-08	2.8E-07	16.788	1.696	14.519	18.542	23.759	1.324	22.563	24.905
ENSG00000177606	JUN	-1.5	9.29E-10	1.8E-08	98.631	6.059	91.710	106.485	141.456	1.989	138.844	143.685
ENSG00000008083	JARID2	-1.5	2.28E-10	5.1E-09	28.882	1.611	26.685	30.552	43.356	1.796	41.091	44.798
ENSG00000176049	JAKMIP2	-1.5	2.3E-07	2.4E-06	5.308	0.777	4.215	5.993	7.678	0.244	7.341	7.926
ENSG00000077684	JADE1	-1.5	2.4E-09	4.3E-08	15.465	1.492	13.291	16.669	22.190	1.291	20.850	23.296
ENSG00000105855	ITGB8	-1.5	0.00013	0.00064	3.134	0.708	2.097	3.598	4.674	0.378	4.301	5.000
ENSG00000105655	ISYNA1	-1.5	1.3E-09	2.4E-08	109.259	4.606	102.670	112.996	154.203	1.426	152.288	155.742
ENSG00000185950	IRS2	-1.5	1.5E-09	2.8E-08	13.044	1.005	12.014	13.929	19.365	0.293	19.114	19.669
ENSG00000168264	IRF2BP2	-1.5	4.22E-11	1.1E-09	49.182	3.191	46.295	52.379	74.730	0.148	74.517	74.860
ENSG00000250492	INTS6P1	-1.5	0.00128	0.00438	1.644	0.171	1.427	1.836	2.504	0.405	2.018	2.838
ENSG00000168918	INPP5D	-1.5	8.91E-10	1.7E-08	9.968	0.331	9.491	10.254	15.237	0.198	15.001	15.401
ENSG00000099377	HSD3B7	-1.5	0.00031	0.00132	2.991	0.245	2.669	3.190	4.499	0.149	4.412	4.722
ENSG00000125430	HS3ST3B1	-1.5	1.2E-06	1.1E-05	6.125	0.545	5.448	6.753	8.777	0.147	8.695	8.997
ENSG00000135547	HEY2	-1.5	0.00018	0.00082	5.231	0.920	4.583	6.552	7.118	0.440	6.752	7.634
ENSG00000253203	GUSBP3	-1.5	0.00072	0.00268	3.718	0.488	3.212	4.381	5.607	0.451	5.017	5.959
ENSG00000123201	GUCY1B2	-1.5	0.01501	0.03453	1.186	0.299	0.744	1.388	1.696	0.378	1.189	1.984
ENSG00000196275	GTF2IRD2	-1.5	0.00139	0.0047	0.748	0.127	0.634	0.926	1.030	0.132	0.956	1.228
ENSG00000046653	GPM6B	-1.5	2E-05	0.00012	2.870	0.438	2.268	3.220	4.210	0.237	3.971	4.414
ENSG00000278175	GLIDR	-1.5	7E-06	5E-05	3.361	0.295	3.109	3.676	5.006	0.486	4.390	5.396
ENSG00000106571	GLI3	-1.5	1.7E-08	2.3E-07	11.589	0.562	10.879	12.253	17.970	0.810	16.900	18.598
ENSG00000164949	GEM	-1.5	1.5E-06	1.3E-05	9.583	1.226	8.682	11.373	13.755	0.525	13.130	14.415
ENSG00000164404	GDF9	-1.5	0.02164	0.04672	0.934	0.302	0.533	1.258	1.444	0.342	0.982	1.703
ENSG00000174804	FZD4	-1.5	2.9E-07	3E-06	13.816	1.646	11.602	15.529	19.403	1.016	18.757	20.893
ENSG00000106701	FSD1L	-1.5	0.00082	0.00301	5.177	1.034	3.636	5.804	8.002	1.773	6.111	9.514
ENSG00000053254	FOXN3	-1.5	4.30E-10	9E-09	15.517	1.344	13.864	17.098	22.293	0.435	21.648	22.555
ENSG00000118407	FILIP1	-1.5	6.5E-05	0.00034	1.493	0.247	1.178	1.761	2.052	0.043	2.024	2.115

ENSG00000176971	FIBIN	-1.5	0.00016	0.00077	5.240	0.980	4.182	6.169	7.189	0.889	6.421	8.024
ENSG00000168143	FAM83B	-1.5	1.9E-05	0.00012	7.530	1.264	5.771	8.704	10.776	0.476	10.428	11.436
ENSG00000145945	FAM50B	-1.5	5.7E-05	0.00031	6.232	0.442	5.640	6.581	9.116	0.936	7.724	9.748
ENSG00000112773	FAM46A	-1.5	6.1E-06	4.4E-05	5.097	0.724	4.227	5.736	7.215	0.260	6.834	7.380
ENSG00000235618	FAM21EP	-1.5	0.00662	0.01738	1.108	0.150	0.975	1.325	1.651	0.303	1.491	2.105
ENSG00000186973	FAM183A	-1.5	0.00768	0.0197	4.259	0.983	3.071	5.467	6.568	1.165	5.404	7.570
ENSG00000248019	FAM13A-AS1	-1.5	0.00677	0.01772	0.715	0.159	0.493	0.844	1.122	0.174	0.871	1.238
ENSG00000145242	EPHA5	-1.5	1.6E-05	0.0001	2.034	0.329	1.552	2.293	2.847	0.106	2.732	2.937
ENSG00000224032	EPB41L4A-A!	-1.5	5.7E-09	9E-08	18.339	0.418	17.852	18.872	25.747	0.633	24.849	26.192
ENSG00000184349	EFNA5	-1.5	2E-08	2.7E-07	9.975	0.742	9.307	10.907	14.421	0.455	13.744	14.733
ENSG00000186976	EFCAB6	-1.5	9.2E-05	0.00046	1.418	0.197	1.207	1.680	2.071	0.060	1.983	2.107
ENSG00000245205	EEF1A1P4	-1.5	0.00105	0.00368	5.663	0.951	4.386	6.673	7.892	0.723	6.811	8.330
ENSG00000179532	DNHD1	-1.5	4.9E-06	3.7E-05	1.495	0.174	1.279	1.681	2.378	0.363	2.017	2.690
ENSG00000176124	DLEU1	-1.5	7.8E-06	5.4E-05	1.399	0.151	1.194	1.558	2.003	0.046	1.971	2.068
ENSG00000204311	DFNB59	-1.5	0.00076	0.00282	2.214	0.430	1.876	2.835	3.164	0.336	2.686	3.474
ENSG00000134574	DDB2	-1.5	2.78E-11	7.71E-10	65.925	0.636	65.198	66.657	98.379	3.799	92.710	100.539
ENSG00000186529	CYP4F3	-1.5	0.00112	0.00391	1.113	0.207	0.868	1.372	1.724	0.119	1.546	1.793
ENSG00000138161	CUZD1	-1.5	7.2E-05	0.00038	4.276	0.622	3.624	5.063	5.789	0.733	5.250	6.802
ENSG00000077063	CTTNBP2	-1.5	1.2E-06	1.1E-05	3.317	0.321	3.076	3.767	4.719	0.175	4.599	4.979
ENSG00000178662	CSRNP3	-1.5	0.00021	0.00095	2.797	0.646	1.897	3.321	4.200	0.376	3.800	4.520
ENSG00000143578	CREB3L4	-1.5	2.9E-05	0.00017	9.229	0.594	8.496	9.850	13.509	0.767	13.020	14.633
ENSG00000106034	CPED1	-1.5	0.00732	0.01894	0.721	0.189	0.521	0.968	0.992	0.094	0.911	1.081
ENSG00000214575	CPEB1	-1.5	4.2E-05	0.00023	2.231	0.229	2.021	2.476	3.378	0.263	3.017	3.573
ENSG00000080573	COL5A3	-1.5	0.01405	0.0327	0.453	0.096	0.327	0.561	0.707	0.149	0.548	0.908
ENSG00000011021	CLCN6	-1.5	7.7E-07	7E-06	7.567	0.577	7.078	8.376	10.832	0.525	10.046	11.117
ENSG00000164442	CITED2	-1.5	2.4E-09	4.3E-08	45.435	2.354	42.413	48.146	66.056	1.195	65.263	67.790
ENSG00000183196	CHST6	-1.5	6.1E-05	0.00033	0.979	0.100	0.882	1.096	1.447	0.115	1.349	1.614
ENSG00000111860	CEP85L	-1.5	0.00019	0.00087	6.732	1.587	4.468	8.109	10.077	0.814	8.999	10.707
ENSG00000135315	CEP162	-1.5	0.02286	0.04893	8.661	3.106	4.006	10.312	12.903	1.519	11.019	14.135
ENSG00000117724	CENPF	-1.5	0.0646	0.11512	57.903	21.623	25.748	71.338	85.800	10.949	73.945	95.110
ENSG00000048740	CELF2	-1.5	7.7E-07	7.1E-06	10.506	1.824	7.962	12.228	15.683	0.673	14.912	16.248
ENSG00000147883	CDKN2B	-1.5	1.9E-09	3.4E-08	16.268	0.837	15.293	17.304	23.535	0.566	22.986	24.329
ENSG00000163171	CDC42EP3	-1.5	2.1E-09	3.7E-08	15.216	1.185	13.455	15.925	21.557	0.927	20.514	22.338
ENSG00000184305	CCSER1	-1.5	0.00116	0.00402	0.682	0.187	0.503	0.929	0.969	0.078	0.864	1.054
ENSG00000186409	CCDC30	-1.5	0.0002	0.00092	1.118	0.203	0.884	1.297	1.658	0.118	1.501	1.749
ENSG00000104957	CCDC130	-1.5	3.3E-06	2.6E-05	13.968	0.974	13.401	15.426	19.694	1.856	18.091	21.470
ENSG00000272168	CASC15	-1.5	0.00735	0.01899	0.295	0.014	0.275	0.307	0.381	0.046	0.331	0.420
ENSG00000204682	CASC10	-1.5	6.3E-05	0.00033	5.082	0.451	4.567	5.654	6.922	0.629	6.422	7.728

ENSG00000145349	CAMK2D	-1.5	2.3E-08	3.1E-07	17.053	2.174	14.159	19.341	24.534	1.168	23.083	25.481
ENSG00000183049	CAMK1D	-1.5	2.7E-07	2.8E-06	4.645	0.395	4.353	5.214	6.731	0.152	6.526	6.846
ENSG00000153956	CACNA2D1	-1.5	6.5E-06	4.7E-05	9.577	1.473	7.426	10.697	14.363	1.335	13.024	15.511
ENSG00000169239	CA5B	-1.5	7.1E-08	8.4E-07	5.678	0.310	5.265	6.004	8.370	0.400	8.060	8.899
ENSG00000164743	C8orf48	-1.5	0.00053	0.00208	4.292	0.753	3.629	5.375	6.369	0.565	5.544	6.735
ENSG00000164746	C7orf57	-1.5	0.0284	0.05839	1.100	0.238	0.908	1.425	1.381	0.635	0.842	2.071
ENSG00000182326	C1S	-1.5	3E-06	2.4E-05	3.866	0.389	3.535	4.287	5.726	0.215	5.518	6.028
ENSG00000143110	C1orf162	-1.5	0.00939	0.02333	1.222	0.252	0.919	1.476	1.810	0.265	1.512	2.156
ENSG00000010671	BTK	-1.5	0.06188	0.11102	0.401	0.146	0.258	0.562	0.574	0.063	0.523	0.652
ENSG00000183336	BOLA2	-1.5	0.00677	0.01772	1.672	0.189	1.532	1.948	2.450	0.373	2.143	2.993
ENSG00000138696	BMPR1B	-1.5	1.9E-05	0.00012	4.883	0.533	4.255	5.440	7.439	0.978	5.993	8.038
ENSG00000153094	BCL2L11	-1.5	1.1E-08	1.6E-07	14.123	1.201	12.627	15.116	21.028	0.240	20.762	21.230
ENSG00000176788	BASP1	-1.5	3.00E-10	6.5E-09	181.933	9.009	174.752	194.976	269.107	11.216	259.495	280.689
ENSG00000100852	ARHGAP5	-1.5	0.00082	0.00299	23.598	5.422	15.486	26.847	35.417	3.449	32.158	38.398
ENSG00000004777	ARHGAP33	-1.5	0.00039	0.00159	3.595	0.308	3.194	3.942	5.278	0.307	5.017	5.722
ENSG00000126016	AMOT	-1.5	2E-09	3.6E-08	16.013	1.811	13.832	17.957	24.517	0.885	23.609	25.276
ENSG00000279355	AGPAT4-IT1	-1.5	0.02115	0.0459	1.712	0.736	0.846	2.579	2.735	0.079	2.657	2.803
ENSG00000233435	AGGF1P2	-1.5	3.1E-05	0.00018	10.407	1.980	7.999	12.647	16.828	1.551	15.038	18.124
ENSG00000214273	AGGF1P1	-1.5	2.3E-05	0.00014	5.811	1.088	4.536	6.911	8.943	0.717	8.250	9.562
ENSG00000198221	AFDN-AS1	-1.5	0.00128	0.00438	1.820	0.262	1.538	2.128	2.764	0.028	2.726	2.793
ENSG00000142303	ADAMTS10	-1.5	6.5E-07	6.1E-06	4.928	0.452	4.513	5.401	7.233	0.063	7.141	7.273
ENSG00000241547	ACTG1P20	-1.5	0.01577	0.03599	5.226	1.128	3.735	6.298	7.362	2.735	5.113	10.662
ENSG00000087085	ACHE	-1.5	0.00072	0.0027	3.210	0.474	2.571	3.706	4.977	0.984	3.581	5.894
ENSG00000223764		-1.5	2E-08	2.7E-07	16.606	1.118	15.368	18.031	24.183	1.869	21.788	25.668
ENSG00000260293		-1.5	5.1E-07	5E-06	5.086	0.540	4.487	5.631	7.919	0.749	7.079	8.551
ENSG00000259943		-1.5	3.8E-06	2.9E-05	12.317	0.930	10.932	12.865	17.444	0.423	16.819	17.704
ENSG00000254815		-1.5	9.6E-06	6.5E-05	5.900	0.391	5.324	6.196	9.169	0.810	7.988	9.699
ENSG00000176593		-1.5	1.4E-05	9.1E-05	2.744	0.589	2.333	3.617	4.129	0.141	3.986	4.323
ENSG00000244627		-1.5	1.7E-05	0.0001	4.270	0.309	4.003	4.716	6.481	0.399	5.976	6.801
ENSG00000213976		-1.5	4.4E-05	0.00024	5.812	1.071	4.324	6.622	8.593	0.144	8.418	8.771
ENSG00000177822		-1.5	4.6E-05	0.00025	2.920	0.512	2.204	3.353	4.076	0.181	3.849	4.292
ENSG00000219665		-1.5	5.6E-05	0.0003	2.141	0.292	1.902	2.563	2.983	0.244	2.817	3.334
ENSG00000215158		-1.5	7.6E-05	0.00039	4.269	0.932	3.288	5.527	6.529	0.793	5.490	7.148
ENSG00000254701		-1.5	7.7E-05	0.0004	14.652	2.248	12.194	17.422	23.115	1.942	20.372	24.491
ENSG00000267506		-1.5	0.00011	0.00056	1.721	0.166	1.533	1.915	2.720	0.137	2.607	2.884
ENSG00000263745		-1.5	0.00014	0.00065	2.360	0.366	2.050	2.871	3.525	0.425	2.895	3.781
ENSG00000269399		-1.5	0.00014	0.00068	4.985	0.431	4.455	5.447	7.080	0.244	6.768	7.364
ENSG00000253366		-1.5	0.00018	0.00085	10.327	1.615	8.602	12.504	15.913	1.910	13.718	17.512

ENSG00000261116	-1.5	0.0002	0.0009	5.929	1.274	4.111	7.046	8.676	0.463	8.045	9.160
ENSG00000198237	-1.5	0.00024	0.00104	10.386	1.507	8.870	12.473	15.896	1.949	13.696	17.537
ENSG00000241288	-1.5	0.00027	0.00116	1.901	0.103	1.802	2.036	3.041	0.396	2.450	3.263
ENSG00000272301	-1.5	0.00032	0.00135	12.504	0.748	11.564	13.215	18.804	1.675	16.655	20.748
ENSG00000242622	-1.5	0.00045	0.00181	3.526	0.323	3.193	3.932	4.921	0.316	4.511	5.282
ENSG00000272870	-1.5	0.00046	0.00184	2.365	0.112	2.229	2.503	3.511	0.303	3.226	3.773
ENSG00000280239	-1.5	0.00056	0.00216	3.084	0.528	2.479	3.733	4.430	0.243	4.250	4.765
ENSG00000260966	-1.5	0.00064	0.00243	2.785	0.451	2.114	3.078	4.056	0.928	2.896	4.805
ENSG00000263412	-1.5	0.00071	0.00266	1.945	0.206	1.713	2.211	2.715	0.132	2.579	2.829
ENSG00000279520	-1.5	0.00171	0.00561	5.517	1.119	3.972	6.632	8.044	0.635	7.725	8.996
ENSG00000271109	-1.5	0.00181	0.0059	6.161	0.745	5.473	7.010	9.128	0.115	8.955	9.190
ENSG00000262877	-1.5	0.00188	0.00609	3.551	0.399	3.148	4.087	5.066	0.425	4.517	5.554
ENSG00000279369	-1.5	0.00224	0.00705	3.338	0.561	2.788	4.091	4.999	0.804	4.148	5.685
ENSG00000276728	-1.5	0.0023	0.0072	3.115	0.469	2.608	3.743	4.852	0.496	4.241	5.455
ENSG00000241764	-1.5	0.00248	0.00766	3.399	0.763	2.786	4.460	5.141	0.494	4.470	5.513
ENSG00000251432	-1.5	0.00305	0.00906	1.778	0.418	1.210	2.110	2.427	0.247	2.284	2.795
ENSG00000261578	-1.5	0.00356	0.01033	1.117	0.088	1.017	1.230	1.443	0.116	1.344	1.611
ENSG00000267284	-1.5	0.00415	0.01176	3.593	0.946	2.809	4.877	5.291	0.354	4.825	5.566
ENSG00000279673	-1.5	0.00432	0.01214	2.035	0.398	1.481	2.392	2.882	0.205	2.578	3.007
ENSG00000271918	-1.5	0.00445	0.01245	2.716	0.399	2.444	3.295	4.060	0.572	3.610	4.899
ENSG00000266490	-1.5	0.0053	0.0144	10.123	2.674	8.256	14.086	15.435	1.250	13.600	16.230
ENSG00000259366	-1.5	0.00542	0.01466	12.363	3.268	8.061	15.901	17.251	2.628	13.944	19.364
ENSG00000241170	-1.5	0.00598	0.0159	1.935	0.275	1.556	2.187	3.119	0.371	2.732	3.437
ENSG00000268670	-1.5	0.00671	0.01759	4.812	1.007	4.094	6.273	7.102	0.698	6.354	7.697
ENSG00000224046	-1.5	0.00697	0.01815	3.441	0.611	2.744	4.116	4.981	0.668	4.599	5.982
ENSG00000228395	-1.5	0.00723	0.01875	2.019	0.525	1.408	2.575	3.015	0.439	2.698	3.629
ENSG00000272195	-1.5	0.00728	0.01885	1.774	0.133	1.576	1.864	2.632	0.164	2.407	2.800
ENSG00000277383	-1.5	0.00751	0.01935	5.726	1.557	3.798	7.373	8.415	0.413	8.021	8.772
ENSG00000279873	-1.5	0.00762	0.01956	2.538	0.756	1.665	3.402	4.185	0.020	4.168	4.205
ENSG00000258472	-1.5	0.0086	0.02168	0.808	0.148	0.705	1.026	1.126	0.090	1.025	1.202
ENSG00000279511	-1.5	0.00883	0.02217	1.574	0.504	0.847	1.989	2.398	0.119	2.231	2.512
ENSG00000260461	-1.5	0.00907	0.02263	1.160	0.233	0.932	1.481	1.869	0.197	1.710	2.157
ENSG00000267546	-1.5	0.01019	0.0249	2.331	0.669	1.733	2.978	3.573	0.930	2.734	4.378
ENSG00000254165	-1.5	0.01141	0.02737	3.120	0.789	2.694	4.302	4.892	0.639	4.092	5.408
ENSG00000272630	-1.5	0.01166	0.02788	1.127	0.351	0.608	1.360	1.641	0.394	1.327	2.143
ENSG00000271971	-1.5	0.01338	0.03137	7.631	1.722	5.614	9.664	12.768	3.334	9.185	15.607
ENSG00000239763	-1.5	0.01431	0.03322	9.420	0.392	9.072	9.981	13.721	1.462	12.480	15.314
ENSG00000225420	-1.5	0.01734	0.03899	2.300	0.734	1.569	3.002	3.399	0.630	3.014	4.330

ENSG00000231728		-1.5	0.01857	0.04121	3.130	1.008	1.742	3.925	4.250	0.370	4.016	4.793
ENSG00000271895		-1.5	0.01933	0.04261	5.444	1.527	3.653	6.776	8.232	0.833	7.401	9.389
ENSG00000280061		-1.5	0.02041	0.04461	1.017	0.289	0.620	1.291	1.434	0.271	1.098	1.761
ENSG00000263884		-1.5	0.02195	0.04732	1.180	0.207	0.923	1.429	1.512	0.259	1.209	1.843
ENSG00000256092		-1.5	0.02208	0.04755	1.469	0.425	1.213	2.100	2.323	0.353	2.007	2.628
ENSG00000259349		-1.5	0.02294	0.04907	3.149	1.226	2.052	4.216	4.667	0.238	4.376	4.959
ENSG00000235946		-1.5	0.0232	0.04953	4.636	0.684	3.685	5.220	7.072	1.179	6.017	8.762
ENSG00000258357		-1.5	0.02421	0.05128	3.200	0.750	2.522	4.100	4.007	1.230	2.966	5.365
ENSG00000234996		-1.5	0.02488	0.05244	1.258	0.292	0.822	1.444	2.025	0.440	1.624	2.406
ENSG00000279347		-1.5	0.02639	0.05502	2.554	0.426	2.023	2.935	3.980	0.287	3.771	4.379
ENSG00000272812		-1.5	0.03034	0.0616	3.751	0.465	3.184	4.153	5.452	0.837	4.242	6.169
ENSG00000226853		-1.5	0.03542	0.07015	2.642	0.388	2.296	3.167	3.521	0.584	2.645	3.832
ENSG00000273007		-1.5	0.03551	0.07028	1.157	0.150	1.063	1.380	1.560	0.293	1.307	1.838
ENSG00000227885		-1.5	0.03604	0.07115	2.604	1.067	1.560	4.095	4.258	0.946	2.841	4.805
ENSG00000248015		-1.5	0.04176	0.08029	1.031	0.482	0.424	1.602	1.493	0.156	1.385	1.716
ENSG00000269974		-1.5	0.05091	0.09466	4.489	2.527	2.312	7.272	6.511	1.477	5.441	8.572
ENSG00000204584		-1.5	0.05514	0.10093	0.962	0.387	0.561	1.441	1.575	0.172	1.346	1.763
ENSG00000261251		-1.5	0.05652	0.10305	1.412	0.489	0.840	1.925	1.766	0.438	1.450	2.379
ENSG00000279199		-1.5	0.1086	0.17621	0.903	0.360	0.543	1.389	1.271	0.405	1.058	1.878
ENSG00000151612	ZNF827	-1.6	7.4E-07	6.8E-06	5.824	0.945	4.466	6.622	9.263	0.113	9.162	9.425
ENSG00000164684	ZNF704	-1.6	2.7E-08	3.5E-07	10.126	1.298	8.217	11.125	15.927	0.791	15.112	16.605
ENSG00000183779	ZNF703	-1.6	1.3E-08	1.8E-07	69.027	7.686	59.388	77.823	109.829	1.808	107.551	111.282
ENSG00000188295	ZNF669	-1.6	0.00034	0.00141	4.360	0.654	3.615	5.210	6.743	0.853	5.474	7.317
ENSG00000269834	ZNF528-AS1	-1.6	6E-08	7.2E-07	9.274	1.047	8.252	10.238	14.891	1.641	12.763	16.184
ENSG00000198795	ZNF521	-1.6	2.4E-06	1.9E-05	4.357	0.836	3.448	5.457	6.538	0.195	6.384	6.790
ENSG00000165244	ZNF367	-1.6	7.36E-11	1.8E-09	42.106	3.767	36.919	45.783	63.914	2.685	60.350	65.986
ENSG00000176293	ZNF135	-1.6	3.9E-06	3E-05	1.595	0.169	1.376	1.769	2.452	0.243	2.299	2.809
ENSG00000100027	YPEL1	-1.6	3E-08	3.8E-07	7.288	0.625	6.592	8.025	11.150	0.692	10.562	11.899
ENSG00000143184	XCL1	-1.6	0.01571	0.03586	2.047	0.483	1.448	2.533	3.508	0.764	2.473	4.085
ENSG00000255282	WTAPP1	-1.6	0.00248	0.00765	0.977	0.237	0.664	1.240	1.565	0.269	1.208	1.772
ENSG00000114251	WNT5A	-1.6	1.3E-08	1.9E-07	7.054	0.804	6.016	7.959	11.404	1.190	10.172	12.423
ENSG00000002745	WNT16	-1.6	0.00745	0.0192	1.051	0.065	0.975	1.121	1.857	0.114	1.686	1.925
ENSG00000187650	VMAC	-1.6	4E-05	0.00023	3.914	0.423	3.455	4.431	6.805	0.867	5.715	7.503
ENSG00000147852	VLDLR	-1.6	1.2E-09	2.3E-08	8.007	0.723	7.062	8.773	12.311	0.236	12.186	12.665
ENSG00000197355	UAP1L1	-1.6	2.53E-10	5.6E-09	24.957	1.351	23.739	26.381	37.210	2.424	35.151	39.853
ENSG00000164938	TP53INP1	-1.6	3.2E-08	4.1E-07	29.665	4.176	23.715	32.662	47.988	2.373	45.635	50.032
ENSG00000101470	TNNC2	-1.6	0.00941	0.02336	3.129	0.982	2.246	4.520	4.365	0.192	4.124	4.594
ENSG00000154310	TNIK	-1.6	7.6E-09	1.2E-07	13.994	1.888	11.505	15.777	21.207	0.298	20.907	21.619

ENSG00000147003	TMEM27	-1.6	0.00541	0.01463	1.727	0.508	1.108	2.335	2.825	0.277	2.448	3.033
ENSG00000274386	TMEM269	-1.6	0.00118	0.00408	1.327	0.164	1.211	1.563	2.341	0.415	1.976	2.700
ENSG00000261115	TMEM178B	-1.6	1E-07	1.2E-06	3.229	0.315	2.887	3.636	5.104	0.320	4.698	5.360
ENSG00000144339	TMEFF2	-1.6	0.01135	0.02728	0.309	0.062	0.251	0.378	0.481	0.069	0.379	0.534
ENSG00000239405	TMED10P2	-1.6	0.00184	0.00598	4.679	0.904	3.853	5.688	7.551	0.586	6.673	7.890
ENSG00000106829	TLE4	-1.6	1.5E-09	2.8E-08	10.201	0.657	9.673	11.160	16.170	0.914	14.822	16.849
ENSG00000187720	THSD4	-1.6	1.20E-10	2.8E-09	5.248	0.087	5.164	5.351	8.541	0.341	8.202	8.835
ENSG00000167074	TEF	-1.6	1.5E-09	2.8E-08	10.446	0.773	9.572	11.252	15.964	0.819	15.531	17.191
ENSG00000135111	TBX3	-1.6	2.27E-10	5.1E-09	12.354	0.837	11.592	13.150	19.236	1.331	17.873	20.378
ENSG00000198075	SULT1C4	-1.6	0.00299	0.00891	1.169	0.162	0.946	1.306	1.838	0.394	1.444	2.385
ENSG00000145687	SSBP2	-1.6	4.24E-12	1.50E-10	10.332	0.246	10.069	10.662	16.605	0.644	15.677	17.040
ENSG00000149634	SPATA25	-1.6	0.01661	0.03763	4.235	1.950	2.531	7.041	7.141	1.668	4.891	8.923
ENSG00000217236	SP9	-1.6	1.6E-05	0.0001	3.888	0.464	3.360	4.472	6.112	0.174	5.966	6.365
ENSG00000177732	SOX12	-1.6	6.48E-10	1.3E-08	23.775	1.991	21.248	25.692	37.154	0.787	36.200	37.800
ENSG00000120669	SOHLH2	-1.6	0.01714	0.03859	0.607	0.155	0.390	0.757	1.136	0.371	0.758	1.455
ENSG00000197989	SNHG12	-1.6	6.6E-09	1E-07	13.641	0.886	12.672	14.744	20.580	0.838	20.048	21.830
ENSG00000184347	SLIT3	-1.6	1.6E-05	0.0001	1.162	0.144	1.004	1.337	1.784	0.154	1.656	1.964
ENSG00000050438	SLC4A8	-1.6	1.8E-06	1.5E-05	1.408	0.233	1.069	1.598	2.325	0.291	1.934	2.547
ENSG00000137968	SLC44A5	-1.6	5.6E-05	0.0003	29.166	5.911	20.663	34.309	48.322	4.319	44.139	52.049
ENSG00000198879	SFMBT2	-1.6	2.7E-08	3.5E-07	6.725	0.652	5.764	7.214	10.578	0.133	10.432	10.755
ENSG00000082684	SEMA5B	-1.6	1.5E-06	1.3E-05	2.878	0.149	2.667	3.013	4.663	0.627	3.905	5.178
ENSG00000228451	SDAD1P1	-1.6	4E-05	0.00022	4.224	0.987	3.004	5.422	6.435	0.314	6.035	6.685
ENSG00000225953	SATB2-AS1	-1.6	0.00956	0.02364	0.665	0.113	0.560	0.818	1.103	0.265	0.892	1.441
ENSG00000004139	SARM1	-1.6	3.2E-08	4E-07	2.800	0.192	2.569	2.991	4.338	0.202	4.203	4.639
ENSG00000079102	RUNX1T1	-1.6	2.6E-06	2.1E-05	1.469	0.219	1.285	1.766	2.280	0.161	2.105	2.416
ENSG00000100784	RPS6KA5	-1.6	1.9E-07	2E-06	1.854	0.247	1.513	2.093	2.846	0.286	2.544	3.090
ENSG00000224892	RPS4XP16	-1.6	0.00334	0.00979	3.047	0.521	2.471	3.567	5.010	0.489	4.641	5.672
ENSG00000177519	RPRM	-1.6	3.2E-08	4E-07	19.923	1.798	18.033	21.931	30.660	1.075	29.873	32.249
ENSG00000140986	RPL3L	-1.6	0.01611	0.03664	0.853	0.223	0.663	1.176	1.475	0.419	0.911	1.793
ENSG00000170153	RNF150	-1.6	3.2E-08	4.1E-07	9.058	1.301	7.277	10.403	14.701	0.600	13.966	15.190
ENSG00000186479	RGS7BP	-1.6	0.00179	0.00585	0.867	0.203	0.653	1.113	1.365	0.218	1.040	1.507
ENSG00000223638	RFPL4A	-1.6	0.00202	0.00648	4.527	0.931	3.690	5.564	6.796	1.466	5.742	8.967
ENSG00000009413	REV3L	-1.6	0.00327	0.00962	14.409	4.051	8.339	16.699	22.975	1.910	20.961	24.606
ENSG00000151164	RAD9B	-1.6	0.00216	0.00683	2.115	0.492	1.700	2.714	3.537	0.799	2.422	4.117
ENSG00000050628	PTGER3	-1.6	0.02447	0.05174	0.243	0.153	0.115	0.458	0.388	0.030	0.358	0.413
ENSG00000244694	PTCHD4	-1.6	7.5E-07	7E-06	3.947	0.134	3.837	4.136	6.398	0.163	6.187	6.584
ENSG00000150687	PRSS23	-1.6	4.61E-12	1.61E-10	23.987	0.681	23.353	24.682	36.402	0.284	36.105	36.788
ENSG00000152784	PRDM8	-1.6	0.0077	0.01974	0.468	0.132	0.353	0.623	0.798	0.132	0.632	0.904

ENSG00000061455	PRDM6	-1.6	3.2E-08	4.1E-07	3.934	0.433	3.314	4.240	6.515	0.461	5.957	6.893
ENSG00000118557	PMFBP1	-1.6	0.00145	0.00486	1.020	0.260	0.726	1.330	1.721	0.315	1.386	1.990
ENSG00000125551	PLGLB2	-1.6	0.00889	0.02229	0.989	0.238	0.776	1.275	1.742	0.445	1.238	2.116
ENSG00000120278	PLEKHG1	-1.6	4.3E-09	7.1E-08	8.375	0.970	7.108	9.292	13.287	0.247	13.003	13.493
ENSG00000134627	PIWIL4	-1.6	0.00026	0.00112	1.276	0.296	1.002	1.639	2.028	0.266	1.689	2.240
ENSG00000139946	PELI2	-1.6	1.4E-07	1.6E-06	14.914	2.173	11.999	17.159	23.347	1.403	21.896	24.548
ENSG00000254122	PCDHGB7	-1.6	0.00306	0.00909	0.608	0.178	0.455	0.863	0.977	0.058	0.891	1.012
ENSG00000262209	PCDHGB3	-1.6	0.00115	0.004	1.028	0.268	0.635	1.241	1.567	0.220	1.288	1.827
ENSG00000081853	PCDHGA2	-1.6	0.04199	0.08065	0.263	0.078	0.181	0.368	0.379	0.088	0.286	0.499
ENSG00000120327	PCDHB14	-1.6	3.6E-07	3.6E-06	3.584	0.341	3.284	4.074	5.696	0.485	5.066	6.077
ENSG00000169851	PCDH7	-1.6	5.09E-10	1E-08	9.887	0.623	9.004	10.467	15.554	0.882	14.495	16.281
ENSG00000167081	PBX3	-1.6	1.3E-09	2.5E-08	14.441	0.398	14.122	15.018	22.104	0.193	21.851	22.320
ENSG00000267270	PARD6G-AS1	-1.6	0.00084	0.00305	0.902	0.221	0.623	1.121	1.457	0.130	1.263	1.530
ENSG00000116117	PARD3B	-1.6	1.9E-08	2.6E-07	3.480	0.212	3.291	3.703	5.543	0.281	5.220	5.778
ENSG00000180914	OXTR	-1.6	0.00732	0.01892	0.865	0.206	0.669	1.077	1.207	0.245	0.856	1.427
ENSG00000176046	NUPR1	-1.6	1.1E-06	1E-05	2.430	0.118	2.255	2.507	3.738	0.504	3.346	4.402
ENSG00000113580	NR3C1	-1.6	9.70E-11	2.4E-09	22.698	2.136	19.544	24.156	35.511	2.421	32.905	37.571
ENSG00000198156	NPIPB6	-1.6	0.00166	0.00547	1.323	0.111	1.216	1.476	1.988	0.381	1.660	2.354
ENSG00000162599	NFIA	-1.6	2.2E-08	3E-07	4.351	0.646	3.494	5.056	6.888	0.093	6.824	7.022
ENSG00000245532	NEAT1	-1.6	3E-07	3.1E-06	76.597	8.683	65.489	86.481	125.039	15.588	110.923	138.531
ENSG00000249437	NAIP	-1.6	5.8E-07	5.6E-06	1.711	0.183	1.483	1.914	2.786	0.232	2.571	2.987
ENSG00000255823	MTRNR2L8	-1.6	0.0014	0.00472	4.527	0.438	4.046	5.012	6.563	1.417	4.819	8.289
ENSG00000003987	MTMR7	-1.6	1.3E-05	8.7E-05	3.922	0.379	3.378	4.254	6.100	0.530	5.599	6.559
ENSG00000210151	MT-TS1	-1.6	0.036	0.0711	80.501	41.991	37.521	128.218	140.259	11.803	131.400	156.330
ENSG00000173531	MST1	-1.6	3.6E-05	0.00021	1.827	0.095	1.713	1.915	3.030	0.598	2.497	3.549
ENSG00000171843	MLLT3	-1.6	1.1E-08	1.6E-07	7.747	1.022	6.334	8.702	12.169	0.622	11.498	12.698
ENSG00000135679	MDM2	-1.6	2E-07	2.1E-06	121.517	17.121	96.607	134.808	201.327	21.664	178.536	219.843
ENSG00000277443	MARCKS	-1.6	7.91E-12	2.58E-10	134.649	4.359	130.760	138.940	203.288	11.154	192.025	212.875
ENSG00000251562	MALAT1	-1.6	7.9E-06	5.5E-05	69.186	7.618	58.195	74.518	107.023	16.914	85.852	120.671
ENSG00000150551	LYPD1	-1.6	1.6E-08	2.2E-07	12.147	0.751	11.609	13.259	18.389	0.994	17.528	19.286
ENSG00000176204	LRRTM4	-1.6	1.7E-05	0.00011	2.025	0.308	1.601	2.322	2.951	0.031	2.916	2.991
ENSG00000033122	LRRC7	-1.6	1.6E-07	1.8E-06	1.946	0.307	1.518	2.221	3.157	0.084	3.065	3.270
ENSG00000238083	LRRC37A2	-1.6	2.9E-08	3.7E-07	4.827	0.496	4.289	5.489	7.632	0.774	6.690	8.265
ENSG00000176681	LRRC37A	-1.6	1.5E-07	1.7E-06	2.866	0.294	2.611	3.232	4.632	0.502	4.189	5.067
ENSG00000275294	LINC02340	-1.6	0.01264	0.02985	2.314	0.466	1.882	2.953	3.192	0.878	2.470	4.251
ENSG00000253161	LINC01605	-1.6	0.01512	0.03475	1.551	0.258	1.356	1.901	2.150	0.622	1.445	2.961
ENSG00000272888	LINC01578	-1.6	2.77E-11	7.68E-10	20.361	1.554	18.676	21.866	31.259	0.516	30.731	31.969
ENSG00000250584	LINC01511	-1.6	0.02601	0.05441	1.102	0.292	0.845	1.440	1.554	0.517	1.131	2.188

ENSG00000248441	LINC01197	-1.6	0.00244	0.00755	0.475	0.066	0.414	0.541	0.695	0.187	0.570	0.967
ENSG00000260941	LINC00622	-1.6	0.01545	0.03541	1.117	0.477	0.610	1.552	1.886	0.115	1.751	1.982
ENSG00000225511	LINC00475	-1.6	0.01373	0.03208	0.688	0.181	0.549	0.955	1.024	0.309	0.733	1.460
ENSG00000196668	LINC00173	-1.6	0.00098	0.00349	2.700	0.704	1.686	3.309	4.047	0.406	3.786	4.640
ENSG00000005483	KMT2E	-1.6	7.4E-08	8.7E-07	25.846	3.982	20.160	29.313	39.371	0.589	38.611	39.837
ENSG00000118263	KLF7	-1.6	1.2E-06	1.1E-05	1.650	0.177	1.512	1.895	2.541	0.149	2.412	2.676
ENSG00000118922	KLF12	-1.6	1.9E-05	0.00012	12.804	2.365	9.442	14.603	19.967	1.166	18.853	20.974
ENSG00000172059	KLF11	-1.6	1.78E-10	4E-09	16.924	0.994	15.895	17.907	27.086	1.440	26.213	29.218
ENSG00000196196	HRCT1	-1.6	0.00392	0.01119	3.074	0.925	1.840	3.983	4.650	0.544	4.198	5.291
ENSG00000258900	HNRNPCP1	-1.6	0.00983	0.0242	4.016	1.366	1.971	4.810	5.857	1.346	4.272	7.565
ENSG00000215630	GUSBP9	-1.6	0.0025	0.00769	5.605	1.365	3.992	7.134	9.464	1.335	7.502	10.307
ENSG00000120370	GORAB	-1.6	4.3E-08	5.3E-07	8.147	1.226	6.613	9.586	12.897	0.307	12.532	13.150
ENSG00000124713	GNMT	-1.6	0.00537	0.01455	2.773	0.719	2.062	3.694	4.530	0.692	3.604	5.060
ENSG00000213453	FTH1P3	-1.6	0.00529	0.01438	4.845	0.483	4.372	5.519	7.067	1.937	5.527	9.546
ENSG00000265817	FSBP	-1.6	0.00529	0.01438	0.877	0.228	0.619	1.143	1.238	0.174	1.074	1.485
ENSG00000114861	FOXP1	-1.6	5E-08	6.1E-07	3.635	0.535	2.974	4.228	5.687	0.155	5.590	5.917
ENSG00000185070	FLRT2	-1.6	6.45E-10	1.3E-08	2.832	0.306	2.386	3.070	4.688	0.432	4.202	5.051
ENSG00000170271	FAXDC2	-1.6	3.6E-07	3.6E-06	5.081	0.231	4.820	5.303	7.834	0.812	7.135	8.648
ENSG00000219626	FAM228B	-1.6	2.5E-06	2E-05	3.945	0.547	3.427	4.593	6.275	1.007	4.857	6.994
ENSG00000148541	FAM13C	-1.6	9.3E-08	1.1E-06	3.989	0.602	3.130	4.533	6.115	0.105	6.048	6.269
ENSG00000206120	EGFEM1P	-1.6	0.00018	0.00081	1.490	0.287	1.126	1.772	2.531	0.520	2.039	2.980
ENSG00000143507	DUSP10	-1.6	0.00014	0.00067	2.992	0.521	2.529	3.708	4.426	0.333	4.161	4.852
ENSG00000259660	DNM1P47	-1.6	0.0001	0.0005	0.374	0.029	0.332	0.395	0.604	0.092	0.519	0.684
ENSG00000105516	DBP	-1.6	1.5E-08	2.1E-07	7.666	0.768	6.749	8.475	11.581	0.467	11.093	12.218
ENSG00000165182	CXorf58	-1.6	0.00386	0.01105	1.461	0.267	1.238	1.808	2.459	0.286	2.174	2.856
ENSG00000107562	CXCL12	-1.6	2.7E-07	2.8E-06	2.299	0.111	2.144	2.406	3.656	0.137	3.544	3.826
ENSG00000129749	CHRNA10	-1.6	0.02241	0.04816	0.845	0.236	0.620	1.176	1.194	0.133	1.046	1.370
ENSG00000116785	CFHR3	-1.6	0.00084	0.00305	1.569	0.364	1.305	2.105	2.504	0.394	2.043	2.832
ENSG00000111276	CDKN1B	-1.6	3.98E-12	1.42E-10	43.476	1.288	42.412	45.346	69.866	3.898	64.598	72.817
ENSG00000134256	CD101	-1.6	0.00112	0.00391	1.024	0.241	0.789	1.265	1.550	0.036	1.514	1.600
ENSG00000256304	CCDC150P1	-1.6	0.00112	0.0039	1.669	0.312	1.397	2.118	2.374	0.259	2.233	2.761
ENSG00000183287	CCBE1	-1.6	6.7E-09	1.1E-07	4.133	0.238	3.867	4.446	6.473	0.153	6.281	6.657
ENSG00000204387	C6orf48	-1.6	4.63E-11	1.2E-09	132.766	4.903	126.421	138.360	202.458	7.782	198.144	214.122
ENSG00000261652	C15orf65	-1.6	0.02357	0.0502	3.785	1.262	2.168	5.244	6.232	0.606	5.346	6.624
ENSG00000183346	C10orf107	-1.6	0.0007	0.00263	3.078	0.865	2.425	4.338	4.866	0.315	4.394	5.034
ENSG00000232260	BTF3L4P1	-1.6	0.00211	0.0067	12.465	1.527	10.180	13.342	17.349	3.357	13.872	20.225
ENSG00000112175	BMP5	-1.6	0.00449	0.01254	0.596	0.076	0.523	0.671	0.906	0.060	0.855	0.994
ENSG00000123095	BHLHE41	-1.6	2.9E-08	3.8E-07	7.561	0.440	7.015	8.091	11.284	0.357	10.764	11.517

ENSG00000113916	BCL6	-1.6	2.4E-08	3.2E-07	4.552	0.302	4.283	4.970	6.859	0.213	6.560	7.012
ENSG00000127152	BCL11B	-1.6	1.7E-05	0.00011	1.311	0.177	1.144	1.482	2.013	0.082	1.940	2.131
ENSG00000119866	BCL11A	-1.6	1.3E-07	1.4E-06	4.574	0.627	3.966	5.439	7.305	0.444	6.677	7.620
ENSG00000105327	BBC3	-1.6	9.7E-09	1.4E-07	22.421	2.251	20.688	25.537	35.281	0.488	34.977	36.010
ENSG00000158321	AUTS2	-1.6	2.4E-07	2.6E-06	8.362	0.226	8.175	8.671	13.267	0.672	12.889	14.274
ENSG00000124788	ATXN1	-1.6	1.8E-07	1.9E-06	3.580	0.560	2.757	4.001	5.550	0.186	5.340	5.706
ENSG00000105409	ATP1A3	-1.6	0.02704	0.05611	0.686	0.337	0.402	1.109	1.006	0.147	0.902	1.214
ENSG00000171681	ATF7IP	-1.6	6.2E-07	5.9E-06	34.721	5.506	26.509	38.268	53.529	2.646	50.990	55.813
ENSG00000221883	ARIH2OS	-1.6	0.00228	0.00717	1.800	0.337	1.476	2.226	2.731	0.313	2.261	2.895
ENSG00000178878	APOLD1	-1.6	1.2E-07	1.4E-06	4.939	0.776	4.213	5.997	8.100	0.426	7.807	8.734
ENSG00000155966	AFF2	-1.6	1.2E-06	1E-05	3.413	0.516	2.689	3.893	5.349	0.193	5.160	5.619
ENSG00000145536	ADAMTS16	-1.6	2.2E-06	1.8E-05	4.108	0.485	3.415	4.449	6.448	0.528	6.095	7.234
ENSG00000077522	ACTN2	-1.6	1.6E-07	1.7E-06	4.832	0.571	3.995	5.254	7.996	0.938	6.666	8.655
ENSG00000160179	ABCG1	-1.6	7.4E-07	6.9E-06	3.464	0.432	3.010	3.997	5.388	0.221	5.056	5.500
ENSG00000269994		-1.6	7E-08	8.3E-07	7.972	0.444	7.425	8.477	12.887	0.697	11.867	13.335
ENSG00000179978		-1.6	6.8E-07	6.4E-06	3.766	0.267	3.374	3.971	6.114	0.579	5.569	6.614
ENSG00000279208		-1.6	3.5E-06	2.7E-05	22.072	2.966	18.692	25.590	34.773	2.452	31.521	36.667
ENSG00000268575		-1.6	9.3E-06	6.3E-05	2.776	0.357	2.443	3.264	4.463	0.466	3.820	4.806
ENSG00000279696		-1.6	2.8E-05	0.00017	2.356	0.091	2.271	2.478	3.751	0.084	3.626	3.796
ENSG00000261754		-1.6	3E-05	0.00018	3.961	0.655	3.343	4.776	6.338	0.452	5.663	6.597
ENSG00000213073		-1.6	3.5E-05	0.0002	3.050	0.414	2.589	3.480	5.061	0.592	4.370	5.554
ENSG00000253816		-1.6	4.2E-05	0.00024	17.476	3.503	13.180	20.974	28.008	0.774	26.855	28.457
ENSG00000257511		-1.6	5.9E-05	0.00032	9.077	1.858	7.332	11.520	13.735	0.736	13.026	14.770
ENSG00000273373		-1.6	6.8E-05	0.00036	8.014	1.847	5.966	10.142	13.048	1.405	11.409	14.219
ENSG00000204802		-1.6	7.2E-05	0.00037	2.616	0.249	2.308	2.852	3.918	0.283	3.702	4.298
ENSG00000224886		-1.6	0.0002	0.00091	9.578	1.326	8.493	11.429	16.096	2.132	13.785	17.908
ENSG00000233045		-1.6	0.00025	0.00109	9.059	0.889	8.137	10.274	14.144	0.867	13.482	15.308
ENSG00000261136		-1.6	0.00076	0.00282	1.552	0.276	1.209	1.838	2.561	0.103	2.430	2.681
ENSG00000185065		-1.6	0.00105	0.0037	2.466	0.516	1.949	3.162	3.514	0.202	3.221	3.684
ENSG00000261879		-1.6	0.00108	0.00379	2.881	0.688	2.153	3.483	4.783	0.177	4.519	4.881
ENSG00000275481		-1.6	0.00142	0.00477	1.312	0.180	1.164	1.532	2.046	0.218	1.902	2.362
ENSG00000280367		-1.6	0.00164	0.00541	1.592	0.299	1.173	1.877	2.530	0.671	1.902	3.110
ENSG00000215022		-1.6	0.00237	0.00739	1.017	0.192	0.757	1.215	1.598	0.080	1.536	1.705
ENSG00000273448		-1.6	0.00281	0.00848	3.534	0.379	3.086	3.905	6.547	1.917	4.454	8.174
ENSG00000272669		-1.6	0.00476	0.01321	5.206	1.361	3.902	7.089	8.518	1.065	7.358	9.423
ENSG00000265218		-1.6	0.00477	0.01321	2.665	0.441	2.044	3.077	4.503	1.338	2.688	5.512
ENSG00000231551		-1.6	0.00491	0.01354	1.138	0.284	0.838	1.505	1.750	0.260	1.533	2.054
ENSG00000253414		-1.6	0.00493	0.01357	7.690	1.932	6.068	10.153	10.529	1.605	9.698	12.936

ENSG00000259275		-1.6	0.00519	0.01416	0.944	0.300	0.574	1.251	1.607	0.412	1.153	1.956
ENSG00000283757		-1.6	0.00523	0.01425	0.543	0.123	0.446	0.713	0.941	0.218	0.678	1.120
ENSG00000275560		-1.6	0.00551	0.01487	6.910	1.580	4.632	8.270	10.717	2.862	6.450	12.589
ENSG00000236028		-1.6	0.00564	0.01516	7.900	3.313	5.367	12.774	11.584	1.161	10.938	13.319
ENSG00000272142		-1.6	0.0086	0.02168	2.557	0.078	2.455	2.643	3.901	0.400	3.459	4.431
ENSG00000272328		-1.6	0.01185	0.02827	2.893	0.902	2.161	4.022	5.091	0.924	3.866	5.805
ENSG00000235100		-1.6	0.01337	0.03137	1.810	0.514	1.102	2.332	3.129	0.230	2.852	3.319
ENSG00000224505		-1.6	0.01391	0.03244	3.404	0.597	2.753	3.939	5.087	0.641	4.163	5.642
ENSG00000256591		-1.6	0.01684	0.03808	0.316	0.110	0.176	0.423	0.529	0.026	0.501	0.551
ENSG00000279456		-1.6	0.02482	0.05234	1.225	0.121	1.071	1.356	1.896	0.503	1.186	2.254
ENSG00000272720		-1.6	0.02505	0.0527	1.417	0.391	1.028	1.885	2.562	1.059	1.096	3.340
ENSG00000272562		-1.6	0.03727	0.07314	4.068	1.194	3.308	5.838	7.571	2.073	4.488	8.791
ENSG00000248774		-1.6	0.0414	0.07973	5.260	2.941	2.829	9.061	7.798	1.604	6.331	9.187
ENSG00000235109	ZSCAN31	-1.7	1E-06	8.9E-06	2.246	0.199	2.051	2.519	3.767	0.494	3.215	4.184
ENSG00000229956	ZRANB2-AS2	-1.7	0.00379	0.01087	0.318	0.062	0.266	0.406	0.470	0.086	0.389	0.591
ENSG00000127903	ZNF835	-1.7	0.00224	0.00705	0.982	0.247	0.679	1.281	1.454	0.298	1.286	1.900
ENSG00000258405	ZNF578	-1.7	7.2E-07	6.7E-06	2.321	0.368	1.791	2.600	3.856	0.414	3.318	4.181
ENSG00000198342	ZNF442	-1.7	3.3E-05	0.00019	1.060	0.071	0.987	1.137	1.709	0.153	1.579	1.875
ENSG00000196724	ZNF418	-1.7	0.00033	0.00139	1.243	0.166	1.095	1.479	2.301	0.372	1.967	2.623
ENSG00000159915	ZNF233	-1.7	3.2E-07	3.3E-06	4.019	0.265	3.648	4.259	6.232	0.380	5.907	6.636
ENSG00000188177	ZC3H6	-1.7	1.6E-08	2.3E-07	3.349	0.554	2.666	3.988	5.734	0.397	5.210	6.042
ENSG00000175564	UCP3	-1.7	0.00229	0.00717	0.714	0.104	0.575	0.827	1.199	0.161	0.961	1.299
ENSG00000179981	TSHZ1	-1.7	5.36E-10	1.1E-08	6.533	0.248	6.314	6.853	11.199	0.078	11.135	11.313
ENSG00000117586	TNFSF4	-1.7	0.00047	0.00188	0.813	0.163	0.673	1.043	1.515	0.220	1.266	1.700
ENSG00000139364	TMEM132B	-1.7	1.1E-05	7.4E-05	0.531	0.049	0.476	0.592	0.951	0.155	0.760	1.077
ENSG00000196781	TLE1	-1.7	1.87E-11	5.40E-10	20.100	1.202	19.002	21.222	33.812	0.803	33.262	34.964
ENSG00000125398	SOX9	-1.7	6.49E-10	1.3E-08	10.294	0.977	8.940	11.106	17.456	0.126	17.285	17.589
ENSG00000134595	SOX3	-1.7	0.00013	0.00062	2.481	0.180	2.311	2.697	4.074	0.631	3.242	4.565
ENSG00000084453	SLCO1A2	-1.7	0.00507	0.01389	0.309	0.105	0.221	0.461	0.516	0.099	0.391	0.634
ENSG00000147606	SLC26A7	-1.7	0.01202	0.0286	0.429	0.124	0.316	0.555	0.776	0.257	0.401	0.941
ENSG00000180592	SKIDA1	-1.7	2E-06	1.6E-05	1.172	0.059	1.100	1.242	1.957	0.217	1.641	2.138
ENSG00000185008	ROBO2	-1.7	3.4E-08	4.3E-07	1.337	0.135	1.154	1.468	2.241	0.088	2.154	2.317
ENSG00000207081	RNU6-616P	-1.7	0.00018	0.00082	48.009	4.586	43.072	54.084	79.633	8.086	70.704	86.479
ENSG00000229292	RFPL4AL1	-1.7	0.00373	0.01075	2.750	0.725	1.962	3.710	4.547	0.515	4.117	5.140
ENSG00000225465	RFPL1S	-1.7	0.00443	0.01243	0.359	0.117	0.212	0.486	0.571	0.020	0.560	0.601
ENSG00000102032	RENBP	-1.7	1.7E-08	2.4E-07	10.513	0.309	10.198	10.809	17.251	1.066	16.520	18.780
ENSG00000138670	RASGEF1B	-1.7	1.1E-08	1.6E-07	2.120	0.148	1.932	2.258	3.433	0.247	3.130	3.635
ENSG00000117569	PTBP2	-1.7	1.2E-09	2.4E-08	5.482	0.686	4.557	6.212	9.129	0.767	8.286	9.779

ENSG00000109819	PPARGC1A	-1.7	2.7E-07	2.8E-06	1.395	0.166	1.170	1.569	2.343	0.156	2.162	2.543
ENSG00000184486	POU3F2	-1.7	1.2E-06	1E-05	3.485	0.546	2.736	4.047	5.699	0.389	5.151	6.070
ENSG00000145632	PLK2	-1.7	4.38E-11	1.1E-09	18.035	1.516	16.474	19.931	30.219	1.628	28.366	31.587
ENSG00000186472	PCLO	-1.7	5.9E-06	4.3E-05	2.362	0.344	1.876	2.680	4.252	0.543	3.777	4.722
ENSG00000081818	PCDHB4	-1.7	0.04552	0.08619	0.550	0.178	0.335	0.764	0.995	0.338	0.708	1.484
ENSG00000146001	PCDHB18P	-1.7	1.6E-06	1.4E-05	2.821	0.396	2.380	3.334	5.010	0.187	4.731	5.120
ENSG00000113248	PCDHB15	-1.7	1.2E-05	8E-05	2.349	0.296	1.922	2.601	3.916	0.472	3.213	4.233
ENSG00000250120	PCDHA10	-1.7	1.1E-06	1E-05	2.165	0.286	1.743	2.376	3.713	0.523	3.106	4.150
ENSG00000188158	NHS	-1.7	3.9E-06	3E-05	1.631	0.287	1.219	1.875	2.644	0.116	2.580	2.818
ENSG00000147862	NFIB	-1.7	8.4E-09	1.3E-07	6.213	0.800	5.179	7.054	11.049	0.829	9.943	11.686
ENSG00000185432	METTL7A	-1.7	1.29E-11	3.93E-10	28.034	1.761	26.295	29.597	48.148	3.863	43.704	51.382
ENSG00000229619	MBNL1-AS1	-1.7	6.6E-06	4.7E-05	1.885	0.386	1.327	2.206	3.133	0.358	2.767	3.440
ENSG00000141639	MAPK4	-1.7	1.4E-07	1.5E-06	3.213	0.511	2.865	3.955	5.505	0.319	5.082	5.751
ENSG00000196782	MAML3	-1.7	2.3E-08	3.1E-07	4.214	0.558	3.536	4.813	6.707	0.074	6.666	6.818
ENSG00000173988	LRRC63	-1.7	0.00057	0.00221	1.375	0.268	1.009	1.639	2.576	0.427	1.936	2.801
ENSG00000168702	LRP1B	-1.7	0.00346	0.01009	0.147	0.039	0.093	0.178	0.255	0.030	0.227	0.281
ENSG00000226383	LINC01876	-1.7	0.00025	0.00109	5.219	1.422	3.218	6.575	8.993	1.830	6.845	10.515
ENSG00000261326	LINC01355	-1.7	0.00017	0.00079	5.231	0.724	4.583	6.199	9.374	1.459	7.762	10.609
ENSG00000258441	LINC00641	-1.7	5.1E-07	4.9E-06	9.809	1.704	7.341	11.069	16.444	1.394	14.674	17.559
ENSG00000250682	LINC00491	-1.7	1.4E-06	1.2E-05	9.140	1.445	7.059	10.383	15.044	0.877	13.731	15.516
ENSG00000116678	LEPR	-1.7	1.8E-07	1.9E-06	8.607	1.585	6.374	10.058	14.184	0.843	13.277	14.902
ENSG00000124743	KLHL31	-1.7	0.00097	0.00347	0.714	0.112	0.581	0.821	1.074	0.182	0.917	1.233
ENSG00000231160	KLF3-AS1	-1.7	0.01829	0.04076	0.563	0.133	0.427	0.691	0.956	0.098	0.833	1.073
ENSG00000049130	KITLG	-1.7	3.4E-05	0.0002	11.776	2.764	7.643	13.452	20.446	2.448	17.506	22.463
ENSG00000162849	KIF26B	-1.7	0.00011	0.00055	0.487	0.145	0.353	0.620	0.832	0.040	0.798	0.872
ENSG00000156650	KAT6B	-1.7	8.5E-09	1.3E-07	11.093	1.756	8.658	12.493	18.285	0.316	17.814	18.466
ENSG00000122641	INHBA	-1.7	3.6E-03	0.0104	0.362	0.055	0.306	0.437	0.606	0.181	0.359	0.795
ENSG00000002587	HS3ST1	-1.7	1.0E-02	0.02496	0.280	0.104	0.180	0.426	0.508	0.124	0.346	0.606
ENSG00000135116	HRK	-1.7	8.9E-08	1E-06	4.230	0.543	3.422	4.578	7.062	0.590	6.177	7.358
ENSG00000105996	HOXA2	-1.7	3.0E-03	0.00892	0.961	0.293	0.682	1.259	1.684	0.158	1.572	1.917
ENSG00000237125	HAND2-AS1	-1.7	5.2E-02	0.09594	0.117	0.053	0.062	0.185	0.174	0.077	0.115	0.288
ENSG00000183098	GPC6	-1.7	4.5E-09	7.3E-08	5.662	0.456	4.988	5.980	9.627	1.030	8.281	10.434
ENSG00000107485	GATA3	-1.7	1.3E-07	1.4E-06	7.623	0.615	6.886	8.193	12.452	1.557	11.378	14.680
ENSG00000141665	FBXO15	-1.7	1.7E-03	0.00553	0.404	0.116	0.256	0.541	0.649	0.024	0.623	0.669
ENSG00000010030	ETV7	-1.7	3.1E-05	0.00018	5.219	0.849	4.106	6.025	8.343	0.975	6.881	8.882
ENSG00000178568	ERBB4	-1.7	2.6E-06	2E-05	1.346	0.304	0.916	1.615	2.344	0.286	2.028	2.586
ENSG00000165617	DACT1	-1.7	1.4E-09	2.6E-08	6.691	0.653	6.088	7.348	11.473	0.434	10.827	11.726
ENSG00000106258	CYP3A5	-1.7	9.7E-08	1.1E-06	3.552	0.368	3.022	3.846	6.036	0.210	5.815	6.216

ENSG00000168772	CXXC4	-1.7	2.3E-06	1.8E-05	7.278	1.604	5.025	8.557	13.051	1.140	12.040	14.038
ENSG00000164463	CREBRF	-1.7	2.6E-05	0.00015	5.770	1.304	3.840	6.626	9.072	0.432	8.575	9.629
ENSG00000206561	COLQ	-1.7	2.4E-04	0.00105	1.248	0.203	1.058	1.522	2.232	0.295	1.919	2.484
ENSG00000124749	COL21A1	-1.7	2.0E-05	0.00012	0.972	0.126	0.786	1.069	1.660	0.196	1.443	1.826
ENSG00000158258	CLSTN2	-1.7	7.7E-06	5.4E-05	0.711	0.054	0.642	0.762	1.255	0.041	1.202	1.302
ENSG00000106069	CHN2	-1.7	1.8E-05	0.00011	1.566	0.331	1.118	1.858	2.449	0.325	2.260	2.933
ENSG00000008300	CELSR3	-1.7	8.1E-06	5.6E-05	0.853	0.185	0.648	1.087	1.363	0.115	1.265	1.483
ENSG00000185267	CDNF	-1.7	2.8E-04	0.00121	2.131	0.279	1.747	2.373	3.953	0.645	3.354	4.511
ENSG00000228409	CCT6P1	-1.7	6.2E-07	5.9E-06	14.375	2.625	11.229	17.428	24.681	1.192	24.010	26.462
ENSG00000174898	CATSPERD	-1.7	1.3E-03	0.00431	1.092	0.298	0.754	1.398	1.803	0.218	1.605	2.114
ENSG00000157445	CACNA2D3	-1.7	1.6E-07	1.8E-06	4.835	0.572	4.152	5.477	7.887	0.250	7.687	8.203
ENSG00000230185	C9orf147	-1.7	4.7E-03	0.01317	1.225	0.262	0.944	1.573	1.948	0.405	1.672	2.530
ENSG00000133639	BTG1	-1.7	6.3E-11	1.6E-09	17.568	0.877	16.556	18.695	28.357	1.059	27.086	29.679
ENSG00000104081	BMF	-1.7	1.3E-12	5.23E-11	18.206	0.228	17.883	18.421	30.903	1.488	28.694	31.945
ENSG00000066279	ASPM	-1.7	2.0E-02	0.04443	25.351	9.272	11.473	30.818	42.614	6.982	35.494	48.608
ENSG00000138639	ARHGAP24	-1.7	1.6E-06	1.4E-05	2.251	0.260	1.971	2.497	3.674	0.210	3.420	3.933
ENSG00000230002	ALMS1-IT1	-1.7	9.6E-05	0.00048	2.451	0.446	2.036	2.873	4.098	0.454	3.438	4.473
ENSG00000161912	ADCY10P1	-1.7	3.6E-08	4.5E-07	3.776	0.259	3.405	4.004	6.419	0.564	5.776	6.892
ENSG00000250546		-1.7	2.0E-05	0.00012	2.338	0.383	1.816	2.670	4.316	0.390	3.813	4.624
ENSG00000262251		-1.7	5.3E-05	0.00029	5.547	0.898	4.355	6.494	9.310	0.030	9.264	9.326
ENSG00000260077		-1.7	8.4E-05	0.00043	6.588	2.010	4.359	9.242	10.712	1.122	9.743	11.791
ENSG00000260103		-1.7	1.2E-04	0.00061	2.279	0.094	2.160	2.370	3.670	0.718	3.064	4.470
ENSG00000261557		-1.7	2.1E-04	0.00093	5.271	0.914	4.294	6.299	8.154	1.353	7.053	10.129
ENSG00000267419		-1.7	4.1E-04	0.00166	2.220	0.557	1.417	2.691	4.060	0.378	3.535	4.336
ENSG00000223855		-1.7	6.9E-04	0.0026	0.923	0.177	0.695	1.122	1.455	0.265	1.274	1.836
ENSG00000225793		-1.7	7.4E-04	0.00277	1.778	0.164	1.604	1.940	2.860	0.661	2.416	3.815
ENSG00000276900		-1.7	8.5E-04	0.00308	1.913	0.523	1.240	2.470	3.203	0.436	2.827	3.623
ENSG00000275620		-1.7	9.6E-04	0.00343	0.441	0.150	0.299	0.637	0.756	0.017	0.740	0.780
ENSG00000260265		-1.7	1.1E-03	0.00375	3.039	1.025	1.538	3.709	5.206	0.478	4.942	5.921
ENSG00000236829		-1.7	1.1E-03	0.00392	1.502	0.148	1.363	1.651	2.804	0.164	2.600	2.937
ENSG00000260526		-1.7	1.4E-03	0.0048	1.293	0.149	1.122	1.483	2.228	0.239	1.897	2.469
ENSG00000270558		-1.7	1.8E-03	0.00576	6.142	1.155	5.150	7.811	10.764	1.526	9.128	12.063
ENSG00000235609		-1.7	2.1E-03	0.00675	0.604	0.149	0.385	0.721	0.975	0.183	0.715	1.103
ENSG00000272070		-1.7	2.8E-03	0.00856	0.713	0.133	0.532	0.843	1.149	0.216	0.869	1.397
ENSG00000262728		-1.7	3.7E-03	0.01075	1.944	0.465	1.545	2.616	3.117	0.585	2.723	3.963
ENSG00000260572		-1.7	3.8E-03	0.01098	1.388	0.600	0.888	2.148	2.183	0.062	2.107	2.233
ENSG00000255471		-1.7	4.1E-03	0.01154	1.776	0.402	1.375	2.299	2.842	0.243	2.511	3.095
ENSG00000261732		-1.7	4.3E-03	0.01202	0.398	0.130	0.298	0.581	0.675	0.119	0.570	0.779

ENSG00000278000		-1.7	5.0E-03	0.01367	3.566	0.243	3.221	3.784	6.066	1.238	4.377	7.356
ENSG00000234345		-1.7	5.9E-03	0.01573	1.739	0.615	1.017	2.455	2.961	0.425	2.740	3.597
ENSG00000248027		-1.7	6.3E-03	0.01663	0.897	0.163	0.682	1.062	1.452	0.195	1.283	1.640
ENSG00000274422		-1.7	8.8E-03	0.02202	4.359	1.227	2.783	5.743	8.551	1.913	6.861	10.208
ENSG00000250222		-1.7	9.2E-03	0.02288	5.647	1.495	4.048	6.974	7.356	2.644	5.909	11.312
ENSG00000234329		-1.7	1.1E-02	0.02576	2.977	1.123	1.412	4.058	5.707	1.035	4.290	6.479
ENSG00000270933		-1.7	1.2E-02	0.02845	3.249	1.067	2.127	4.660	4.587	1.245	3.631	6.249
ENSG00000281195		-1.7	1.3E-02	0.03009	1.782	0.665	1.226	2.738	3.359	1.131	1.751	4.149
ENSG00000273038		-1.7	1.4E-02	0.03188	1.051	0.455	0.618	1.692	1.954	0.214	1.643	2.094
ENSG00000176723	ZNF843	-1.8	2.1E-03	0.00657	0.646	0.157	0.452	0.836	1.091	0.132	0.979	1.234
ENSG00000168916	ZNF608	-1.8	6.2E-10	1.3E-08	11.509	1.537	9.390	13.069	20.287	1.169	18.882	21.250
ENSG00000166432	ZMAT1	-1.8	1.2E-04	0.00057	2.134	0.525	1.417	2.638	4.055	0.761	3.387	4.714
ENSG00000169554	ZEB2	-1.8	4.8E-09	7.7E-08	1.728	0.298	1.324	2.043	3.053	0.268	2.806	3.284
ENSG00000166415	WDR72	-1.8	7.6E-04	0.00281	0.561	0.197	0.278	0.724	0.929	0.095	0.875	1.070
ENSG00000038427	VCAN	-1.8	1.1E-05	7.1E-05	33.986	6.775	23.981	38.980	61.723	4.517	56.914	65.576
ENSG00000171724	VAT1L	-1.8	5.6E-03	0.01502	0.517	0.191	0.293	0.730	0.972	0.173	0.778	1.118
ENSG00000121297	TSHZ3	-1.8	4.2E-08	5.2E-07	4.198	0.083	4.129	4.319	7.269	0.585	6.774	7.914
ENSG00000198846	TOX	-1.8	3.7E-03	0.01074	0.661	0.142	0.462	0.774	1.131	0.440	0.646	1.714
ENSG00000162814	SPATA17	-1.8	6.1E-04	0.00235	0.691	0.275	0.317	0.976	1.253	0.269	0.914	1.469
ENSG00000166922	SCG5	-1.8	6.4E-03	0.01686	0.779	0.162	0.579	0.965	1.377	0.137	1.173	1.453
ENSG00000213363	RPS3P6	-1.8	3.8E-03	0.01095	3.400	1.397	1.827	5.122	5.995	0.842	4.744	6.577
ENSG00000116574	RHOU	-1.8	1.7E-14	1.20E-12	38.177	1.437	36.059	39.254	67.865	2.004	65.350	69.479
ENSG00000261126	RBFADN	-1.8	1.0E-03	0.00356	0.486	0.188	0.264	0.713	0.878	0.034	0.826	0.901
ENSG00000077092	RARB	-1.8	3.3E-07	3.3E-06	3.240	0.450	2.708	3.807	5.414	0.456	4.876	5.991
ENSG00000166473	PKD1L2	-1.8	6.2E-04	0.00238	0.141	0.022	0.114	0.162	0.233	0.008	0.225	0.244
ENSG00000117461	PIK3R3	-1.8	9.6E-12	3.04E-10	13.513	1.467	11.581	14.856	23.739	1.056	22.157	24.315
ENSG00000253305	PCDHGB6	-1.8	2.7E-04	0.00119	0.583	0.086	0.494	0.677	1.161	0.237	0.866	1.353
ENSG00000116774	OLFML3	-1.8	1.2E-06	1.1E-05	5.027	0.802	3.995	5.851	8.397	1.083	7.465	9.472
ENSG00000149294	NCAM1	-1.8	6.7E-13	2.98E-11	7.392	0.496	7.025	8.122	13.454	0.648	12.688	13.991
ENSG00000101928	MOSPD1	-1.8	1.2E-12	4.88E-11	116.653	10.897	100.758	125.483	209.918	11.418	198.994	219.777
ENSG00000153714	LURAP1L	-1.8	5.4E-04	0.0021	1.484	0.165	1.349	1.714	2.578	0.095	2.505	2.718
ENSG00000254402	LRRC24	-1.8	2.5E-04	0.00111	1.976	0.131	1.879	2.162	3.255	0.535	2.453	3.546
ENSG00000247675	LRP4-AS1	-1.8	8.9E-03	0.02229	1.159	0.174	0.913	1.321	1.824	0.526	1.384	2.433
ENSG00000110031	LPXN	-1.8	8.0E-09	1.2E-07	7.240	0.785	6.120	7.885	13.155	1.014	12.266	14.033
ENSG00000226476	LINC01748	-1.8	1.8E-06	1.5E-05	0.839	0.086	0.721	0.928	1.436	0.145	1.242	1.547
ENSG00000236204	LINC01376	-1.8	5.4E-06	4E-05	2.921	0.754	1.931	3.677	5.203	0.466	4.559	5.546
ENSG00000250337	LINC01021	-1.8	6.4E-05	0.00034	36.590	8.207	24.760	43.371	69.712	9.357	58.891	77.532
ENSG00000185261	KIAA0825	-1.8	6.8E-04	0.00258	0.417	0.123	0.321	0.596	0.743	0.044	0.705	0.789

ENSG00000128052	KDR	-1.8	3.0E-03	0.00884	0.400	0.108	0.279	0.494	0.713	0.149	0.586	0.874
ENSG00000272767	JMJD1C-AS1	-1.8	9.8E-04	0.0035	2.170	0.520	1.587	2.852	3.756	0.361	3.255	4.118
ENSG00000144668	ITGA9	-1.8	7.8E-06	5.4E-05	0.752	0.119	0.644	0.922	1.323	0.095	1.186	1.388
ENSG00000236778	INTS6-AS1	-1.8	1.1E-05	7.1E-05	0.669	0.093	0.571	0.773	1.204	0.118	1.051	1.338
ENSG00000254289	IGHV3-32	-1.8	3.5E-05	0.0002	13.509	3.674	9.625	18.472	25.033	2.956	21.757	28.942
ENSG00000270276	HIST2H4B	-1.8	3.4E-11	9.15E-10	29.276	2.637	26.622	32.660	51.871	0.882	50.596	52.626
ENSG00000270882	HIST2H4A	-1.8	3.0E-11	8.32E-10	30.392	2.552	27.724	33.739	53.350	0.763	52.389	54.255
ENSG00000274750	HIST1H3E	-1.8	5.1E-08	6.2E-07	5.976	0.471	5.457	6.546	10.382	1.014	9.854	11.903
ENSG00000124635	HIST1H2BJ	-1.8	3.6E-08	4.5E-07	16.198	2.526	12.744	18.595	28.627	0.595	28.129	29.312
ENSG00000214544	GTF2IRD2P1	-1.8	2.6E-04	0.00113	0.651	0.082	0.592	0.773	1.237	0.162	0.994	1.323
ENSG00000180447	GAS1	-1.8	1.0E-10	2.4E-09	10.802	0.414	10.451	11.349	18.478	0.421	18.115	18.892
ENSG00000130222	GADD45G	-1.8	8.6E-06	5.9E-05	7.645	1.713	5.351	9.497	13.920	0.753	13.189	14.976
ENSG00000116717	GADD45A	-1.8	1.9E-11	5.47E-10	51.966	2.891	49.289	56.071	91.402	3.945	86.930	96.547
ENSG00000054598	FOXC1	-1.8	9.5E-12	3.01E-10	26.868	0.834	25.634	27.463	47.052	2.399	44.972	49.130
ENSG00000125848	FLRT3	-1.8	1.6E-06	1.3E-05	2.632	0.449	2.266	3.286	4.746	0.587	3.877	5.103
ENSG00000102678	FGF9	-1.8	3.3E-05	0.00019	1.684	0.234	1.376	1.916	3.027	0.210	2.769	3.199
ENSG00000165323	FAT3	-1.8	3.4E-07	3.4E-06	5.886	1.068	4.393	6.931	10.903	0.485	10.419	11.320
ENSG00000283709	FAM238C	-1.8	1.8E-04	0.00081	0.949	0.104	0.831	1.037	1.875	0.460	1.473	2.273
ENSG00000044524	EPHA3	-1.8	3.6E-08	4.5E-07	6.970	1.285	5.072	7.823	11.856	0.323	11.462	12.120
ENSG00000128512	DOCK4	-1.8	1.1E-07	1.3E-06	3.753	0.755	2.723	4.541	6.555	0.123	6.420	6.660
ENSG00000224165	DNAJC27-AS	-1.8	2.9E-03	0.00864	0.382	0.167	0.225	0.589	0.705	0.030	0.667	0.741
ENSG00000276644	DACH1	-1.8	4.0E-08	5E-07	4.445	0.647	3.674	5.223	8.239	0.814	7.437	8.941
ENSG00000259736	CRTC3-AS1	-1.8	7.7E-03	0.01964	1.967	0.760	1.232	2.989	3.330	0.253	3.167	3.700
ENSG00000215018	COL28A1	-1.8	1.4E-04	0.00065	0.450	0.037	0.423	0.504	0.782	0.152	0.651	0.926
ENSG00000254413	CHKB-CPT1B	-1.8	1.5E-02	0.03505	0.394	0.106	0.293	0.512	0.609	0.322	0.381	1.063
ENSG00000159409	CELF3	-1.8	2.8E-03	0.00833	0.365	0.102	0.223	0.465	0.709	0.014	0.689	0.719
ENSG00000123080	CDKN2C	-1.8	2.0E-13	1.06E-11	29.444	0.975	28.549	30.514	50.212	0.837	49.283	50.920
ENSG00000164076	CAMKV	-1.8	9.1E-03	0.02273	0.487	0.180	0.234	0.642	0.889	0.198	0.604	1.023
ENSG00000106804	C5	-1.8	8.4E-07	7.7E-06	2.085	0.267	1.800	2.340	3.672	0.566	2.909	4.103
ENSG00000163009	C2orf48	-1.8	2.4E-05	0.00015	2.966	0.411	2.513	3.510	5.305	0.666	4.837	6.293
ENSG00000214212	C19orf38	-1.8	1.2E-03	0.00417	1.917	0.793	1.320	3.017	3.665	0.307	3.466	4.114
ENSG00000143032	BARHL2	-1.8	1.3E-03	0.00435	1.076	0.150	0.883	1.231	1.811	0.230	1.612	2.033
ENSG00000150347	ARID5B	-1.8	6.5E-11	1.6E-09	12.826	1.694	10.356	14.170	22.090	0.466	21.665	22.493
ENSG00000103723	AP3B2	-1.8	1.7E-04	0.0008	0.848	0.049	0.798	0.914	1.650	0.185	1.435	1.888
ENSG00000163126	ANKRD23	-1.8	6.9E-04	0.00259	0.674	0.135	0.555	0.846	1.171	0.190	1.047	1.447
ENSG00000215559	ANKRD20A1	-1.8	1.0E-04	0.00052	1.119	0.366	0.831	1.651	2.020	0.166	1.784	2.136
ENSG00000101745	ANKRD12	-1.8	1.6E-02	0.03715	4.255	1.649	1.855	5.509	7.959	1.465	6.064	9.115
ENSG00000170214	ADRA1B	-1.8	9.9E-04	0.00352	2.796	0.854	1.916	3.932	4.749	0.841	3.615	5.649

ENSG00000150471	ADGRL3	-1.8	9.5E-10	1.8E-08	2.265	0.271	1.863	2.434	4.075	0.301	3.762	4.333
ENSG00000185736	ADARB2	-1.8	9.1E-05	0.00046	0.528	0.105	0.423	0.666	1.016	0.192	0.747	1.153
ENSG00000165029	ABCA1	-1.8	5.2E-07	5E-06	1.273	0.273	0.924	1.545	2.225	0.072	2.117	2.263
ENSG00000272341		-1.8	3.0E-07	3.1E-06	6.209	0.768	5.640	7.334	10.998	0.893	10.021	11.755
ENSG00000251634		-1.8	1.2E-06	1.1E-05	3.014	0.557	2.188	3.404	5.221	0.164	5.077	5.363
ENSG00000248583		-1.8	6.9E-06	4.9E-05	8.867	1.578	7.258	10.368	15.161	0.798	14.485	16.041
ENSG00000279522		-1.8	1.3E-05	8.5E-05	2.910	0.308	2.555	3.238	5.028	0.284	4.631	5.305
ENSG00000232611		-1.8	2.5E-05	0.00015	1.737	0.243	1.422	2.006	3.137	0.409	2.880	3.747
ENSG00000279900		-1.8	1.2E-04	0.0006	9.473	2.203	6.329	11.411	16.376	2.451	12.820	18.001
ENSG00000266680		-1.8	1.3E-04	0.00063	5.046	1.363	3.962	7.033	9.309	1.952	6.937	10.909
ENSG00000263080		-1.8	2.2E-04	0.001	4.068	0.304	3.639	4.297	7.834	1.692	6.095	9.285
ENSG00000251661		-1.8	4.0E-04	0.00161	2.042	0.613	1.484	2.865	3.654	0.123	3.551	3.796
ENSG00000214243		-1.8	4.1E-04	0.00167	4.445	0.715	3.463	5.157	7.600	0.529	6.849	7.971
ENSG00000240291		-1.8	7.1E-04	0.00267	2.646	0.622	1.917	3.368	4.952	0.892	3.679	5.571
ENSG00000271133		-1.8	7.2E-04	0.0027	2.082	0.185	1.858	2.303	3.556	0.180	3.331	3.773
ENSG00000258457		-1.8	7.3E-04	0.00272	3.286	0.470	2.588	3.587	6.370	0.383	5.945	6.876
ENSG00000229127		-1.8	9.1E-04	0.00327	2.032	0.744	1.156	2.839	3.911	1.129	2.635	4.861
ENSG00000204666		-1.8	1.2E-03	0.00415	1.481	0.390	1.098	1.980	2.715	0.396	2.217	3.034
ENSG00000267370		-1.8	1.4E-03	0.00482	3.103	0.657	2.139	3.613	5.713	1.321	3.751	6.498
ENSG00000263280		-1.8	1.6E-03	0.00526	4.471	1.422	2.496	5.718	7.885	0.719	6.830	8.446
ENSG00000249721		-1.8	1.7E-03	0.00555	6.884	3.076	2.451	9.471	12.994	0.773	12.414	14.133
ENSG00000255435		-1.8	2.6E-03	0.00795	1.589	0.251	1.357	1.867	2.718	0.390	2.138	2.981
ENSG00000237732		-1.8	2.9E-03	0.00859	0.745	0.161	0.574	0.929	1.242	0.164	1.040	1.442
ENSG00000249655		-1.8	4.0E-03	0.01131	3.586	1.365	2.219	4.832	7.045	1.458	4.864	7.846
ENSG00000259723		-1.8	5.2E-03	0.0141	5.075	1.890	2.304	6.526	7.891	1.631	6.159	10.097
ENSG00000240731		-1.8	5.6E-03	0.015	5.341	0.894	4.200	6.273	11.248	3.240	8.340	14.053
ENSG00000272030		-1.8	1.6E-02	0.03601	2.299	0.873	1.149	3.016	4.089	1.236	2.235	4.734
ENSG00000237149	ZNF503-AS2	-1.9	2.3E-06	1.9E-05	3.587	0.686	3.093	4.578	6.557	0.786	5.489	7.148
ENSG00000152763	WDR78	-1.9	3.3E-05	0.00019	1.407	0.374	1.114	1.941	2.512	0.524	2.070	3.097
ENSG00000182168	UNC5C	-1.9	6.7E-09	1E-07	2.374	0.353	2.067	2.883	4.386	0.321	4.037	4.659
ENSG00000133710	SPINK5	-1.9	2.4E-08	3.2E-07	3.113	0.506	2.394	3.558	6.032	0.642	5.070	6.373
ENSG00000134532	SOX5	-1.9	1.8E-07	2E-06	1.226	0.095	1.144	1.329	2.216	0.280	1.812	2.457
ENSG00000124766	SOX4	-1.9	8.6E-14	5.04E-12	68.681	5.271	62.731	74.516	125.366	4.712	121.005	129.442
ENSG00000176887	SOX11	-1.9	5.5E-11	1.4E-09	6.175	0.752	5.380	6.862	11.318	0.445	10.652	11.566
ENSG00000148942	SLC5A12	-1.9	9.0E-05	0.00045	0.583	0.106	0.442	0.684	1.108	0.091	1.003	1.184
ENSG00000139209	SLC38A4	-1.9	3.2E-05	0.00018	1.085	0.231	0.767	1.280	2.114	0.275	1.819	2.348
ENSG00000106483	SFRP4	-1.9	4.7E-04	0.00187	0.657	0.115	0.490	0.749	1.192	0.132	1.027	1.350
ENSG00000152217	SETBP1	-1.9	4.0E-09	6.7E-08	3.089	0.293	2.751	3.393	5.680	0.427	5.322	6.170

ENSG00000243819	RN7SL832P	-1.9	1.3E-04	0.00062	1.298	0.295	0.996	1.648	2.517	0.034	2.475	2.544
ENSG00000268006	PTOV1-AS1	-1.9	1.0E-06	9.2E-06	1.758	0.078	1.656	1.823	3.514	0.477	2.949	3.910
ENSG00000164708	PGAM2	-1.9	1.5E-04	0.00071	3.461	0.723	2.488	4.062	6.038	0.743	5.399	6.782
ENSG00000049246	PER3	-1.9	3.9E-10	8.2E-09	4.297	0.409	3.767	4.765	8.123	0.659	7.135	8.470
ENSG00000189184	PCDH18	-1.9	3.2E-09	5.5E-08	3.978	0.590	3.375	4.715	7.203	0.418	6.909	7.824
ENSG00000185630	PBX1	-1.9	1.8E-13	9.80E-12	9.139	0.810	7.958	9.776	17.534	1.021	16.455	18.406
ENSG00000085465	OVGP1	-1.9	4.4E-07	4.3E-06	3.367	0.297	2.966	3.674	6.235	0.312	5.966	6.548
ENSG00000099250	NRP1	-1.9	4.6E-13	2.11E-11	11.432	0.874	10.179	12.215	21.682	1.406	20.243	22.888
ENSG00000247809	NR2F2-AS1	-1.9	1.3E-05	8.6E-05	0.773	0.111	0.656	0.899	1.268	0.099	1.184	1.381
ENSG00000176771	NCKAP5	-1.9	1.8E-08	2.5E-07	1.335	0.195	1.215	1.626	2.483	0.153	2.405	2.713
ENSG00000143995	MEIS1	-1.9	4.9E-10	1E-08	2.710	0.310	2.326	3.019	5.140	0.484	4.446	5.472
ENSG00000130518	KIAA1683	-1.9	2.5E-08	3.4E-07	1.239	0.094	1.165	1.372	2.427	0.121	2.335	2.591
ENSG00000267365	KCNJ2-AS1	-1.9	2.0E-04	0.00092	1.373	0.333	1.123	1.859	2.414	0.077	2.316	2.505
ENSG00000090376	IRAK3	-1.9	1.1E-02	0.02563	0.209	0.104	0.061	0.303	0.426	0.067	0.330	0.483
ENSG00000196890	HIST3H2BB	-1.9	3.8E-06	2.9E-05	2.044	0.324	1.664	2.454	3.797	0.115	3.677	3.954
ENSG00000187837	HIST1H1C	-1.9	1.2E-11	3.76E-10	97.292	4.365	90.852	100.541	174.671	7.779	168.983	185.463
ENSG00000134363	FST	-1.9	5.2E-05	0.00028	1.899	0.552	1.390	2.651	3.594	0.119	3.416	3.654
ENSG00000168386	FILIP1L	-1.9	2.2E-05	0.00013	0.970	0.243	0.619	1.179	1.850	0.305	1.437	2.081
ENSG00000196159	FAT4	-1.9	3.3E-07	3.3E-06	6.756	1.225	5.085	7.996	13.033	0.653	12.083	13.461
ENSG00000116106	EPHA4	-1.9	1.4E-10	3.3E-09	6.137	0.543	5.344	6.572	11.240	0.925	10.356	12.038
ENSG00000242689	CNTF	-1.9	3.9E-05	0.00022	2.395	0.335	2.023	2.814	4.493	0.840	3.824	5.723
ENSG00000240498	CDKN2B-AS1	-1.9	6.8E-09	1.1E-07	2.775	0.307	2.559	3.224	5.193	0.414	4.800	5.551
ENSG00000129757	CDKN1C	-1.9	9.2E-10	1.8E-08	7.564	0.501	7.008	8.173	13.591	0.613	13.009	14.457
ENSG00000150394	CDH8	-1.9	9.2E-06	6.3E-05	0.395	0.079	0.289	0.475	0.742	0.124	0.627	0.849
ENSG00000272398	CD24	-1.9	8.3E-02	0.1415	2.469	0.144	2.335	2.665	4.201	2.674	2.850	8.212
ENSG00000234585	CCT6P3	-1.9	3.6E-09	6.1E-08	3.653	0.220	3.365	3.899	6.629	0.249	6.264	6.790
ENSG00000133101	CCNA1	-1.9	8.8E-08	1E-06	5.542	0.196	5.342	5.774	9.784	1.497	8.509	11.388
ENSG00000179397	CATSPERE	-1.9	4.5E-03	0.01248	0.342	0.097	0.220	0.422	0.607	0.152	0.479	0.780
ENSG00000167995	BEST1	-1.9	1.2E-07	1.4E-06	1.031	0.109	0.913	1.148	1.941	0.162	1.697	2.028
ENSG00000162373	BEND5	-1.9	3.4E-04	0.00142	1.212	0.198	0.920	1.353	2.106	0.434	1.791	2.710
ENSG00000258655	ARHGAP5-AS	-1.9	7.7E-06	5.4E-05	3.642	0.315	3.354	4.067	6.282	1.224	5.313	8.075
ENSG00000259456	ADNP-AS1	-1.9	7.2E-03	0.01866	1.975	0.417	1.479	2.400	3.144	1.132	1.525	4.167
ENSG00000270605		-1.9	5.5E-09	8.8E-08	10.756	1.023	9.474	11.905	19.675	0.961	18.817	20.506
ENSG00000279865		-1.9	2.5E-05	0.00015	1.893	0.116	1.794	2.050	3.425	0.148	3.306	3.612
ENSG00000255363		-1.9	5.6E-04	0.00219	1.284	0.369	0.854	1.755	2.101	0.296	1.850	2.426
ENSG00000236654		-1.9	5.8E-04	0.00224	12.764	3.701	8.773	16.890	25.574	1.346	23.717	26.939
ENSG00000250753		-1.9	1.1E-03	0.00384	6.997	2.677	4.527	9.884	14.196	1.009	12.716	14.992
ENSG00000273759		-1.9	1.8E-03	0.00582	2.805	0.515	2.113	3.353	4.826	1.373	3.771	6.657

ENSG00000214432		-1.9	2.0E-03	0.00632	0.579	0.091	0.485	0.695	1.056	0.316	0.791	1.420
ENSG00000279659		-1.9	1.6E-02	0.03704	0.851	0.236	0.645	1.189	2.045	0.496	1.362	2.411
ENSG00000272054		-1.9	1.9E-02	0.04229	0.478	0.209	0.209	0.716	0.954	0.154	0.867	1.185
ENSG00000280010		-1.9	2.1E-02	0.04511	1.497	0.865	0.553	2.645	2.591	1.138	1.723	4.267
ENSG00000026025	VIM	-2	1.7E-15	1.69E-13	171.678	6.813	165.429	181.380	340.579	7.338	330.942	346.298
ENSG00000181449	SOX2	-2	5.7E-12	1.93E-10	15.304	1.335	13.878	17.006	29.536	1.595	28.119	30.918
ENSG00000198732	SMOC1	-2	4.7E-09	7.7E-08	7.987	0.968	6.693	9.040	14.749	1.891	13.146	16.826
ENSG00000149212	SESN3	-2	6.9E-06	4.9E-05	38.510	8.203	26.514	44.128	77.638	6.782	70.716	83.459
ENSG00000257557	PPP1R12A-A	-2	1.0E-04	0.00052	0.685	0.129	0.501	0.801	1.269	0.074	1.166	1.344
ENSG00000170836	PPM1D	-2	1.3E-14	9.20E-13	36.154	1.711	34.437	38.011	69.966	2.472	66.568	71.799
ENSG00000151623	NR3C2	-2	5.1E-12	1.77E-10	6.437	0.644	5.508	6.915	12.452	0.722	11.986	13.528
ENSG00000237187	NR2F1-AS1	-2	1.1E-09	2.1E-08	1.882	0.189	1.647	2.111	3.683	0.097	3.561	3.761
ENSG00000165555	NOXRED1	-2	1.0E-03	0.00356	0.810	0.217	0.591	1.093	1.375	0.309	1.152	1.809
ENSG00000169184	MN1	-2	1.0E-10	2.5E-09	5.372	0.192	5.107	5.567	11.142	0.425	10.545	11.448
ENSG00000255248	MIR100HG	-2	3.6E-05	0.00021	0.356	0.057	0.289	0.429	0.705	0.073	0.628	0.768
ENSG00000183496	MEX3B	-2	3.6E-12	1.29E-10	9.284	0.788	8.445	10.300	17.605	0.913	16.306	18.242
ENSG00000233639	LINC01158	-2	1.4E-03	0.00481	2.302	0.674	1.624	3.228	4.339	0.235	4.159	4.653
ENSG00000123700	KCNJ2	-2	2.1E-07	2.3E-06	1.304	0.149	1.085	1.400	2.590	0.342	2.078	2.775
ENSG00000233822	HIST1H2BN	-2	5.1E-08	6.3E-07	1.585	0.144	1.474	1.796	3.298	0.043	3.262	3.350
ENSG00000236993	GAPDHP21	-2	3.0E-05	0.00018	2.935	0.465	2.413	3.356	5.833	0.260	5.687	6.221
ENSG00000128573	FOXP2	-2	1.7E-06	1.4E-05	2.756	0.700	1.712	3.176	5.641	0.128	5.495	5.749
ENSG00000228314	CYP4F29P	-2	3.7E-03	0.01075	0.954	0.491	0.463	1.631	2.088	0.202	1.967	2.391
ENSG00000183230	CTNNA3	-2	1.5E-03	0.00497	0.163	0.071	0.071	0.232	0.339	0.065	0.246	0.395
ENSG00000168542	COL3A1	-2	5.1E-13	2.34E-11	8.442	0.529	7.871	8.958	16.231	0.762	15.647	17.251
ENSG00000213760	ATP6V1G2	-2	4.7E-08	5.8E-07	3.826	0.423	3.257	4.229	7.397	0.345	7.122	7.902
ENSG00000261716		-2	1.4E-10	3.3E-09	8.430	1.314	6.494	9.394	17.171	0.930	16.274	17.973
ENSG00000251095		-2	1.8E-09	3.3E-08	11.599	2.214	8.353	13.341	22.492	0.847	21.244	23.139
ENSG00000220323		-2	2.5E-07	2.6E-06	9.514	0.883	8.704	10.385	19.325	1.551	17.100	20.387
ENSG00000203819		-2	2.8E-06	2.2E-05	9.613	2.199	6.713	11.947	18.318	2.453	16.229	20.953
ENSG00000270681		-2	6.6E-06	4.8E-05	4.001	0.260	3.807	4.369	7.149	0.934	5.785	7.903
ENSG00000257261		-2	7.7E-05	0.0004	1.267	0.132	1.155	1.427	2.591	0.334	2.096	2.794
ENSG00000230699		-2	2.8E-04	0.00122	2.009	0.523	1.451	2.712	4.268	1.192	3.097	5.295
ENSG00000234537		-2	5.8E-03	0.01541	0.692	0.313	0.341	1.063	1.153	0.458	0.771	1.686
ENSG00000056487	PHF21B	-2.1	7.6E-04	0.00282	0.499	0.111	0.424	0.663	0.954	0.367	0.638	1.311
ENSG00000262096	PCDHB19P	-2.1	2.0E-04	0.0009	0.745	0.220	0.535	1.028	1.463	0.206	1.258	1.641
ENSG00000181143	MUC16	-2.1	1.9E-04	0.00087	0.046	0.016	0.034	0.069	0.094	0.013	0.084	0.108
ENSG00000233930	KRTAP5-AS1	-2.1	1.7E-04	0.00078	0.678	0.106	0.553	0.813	1.564	0.229	1.233	1.757
ENSG00000099251	HSD17B7P2	-2.1	9.0E-10	1.8E-08	12.246	2.146	9.761	14.621	25.578	2.251	22.530	27.279

ENSG00000180596	HIST1H2BC	-2.1	1.5E-05	9.5E-05	2.926	0.246	2.722	3.239	6.057	0.999	4.632	6.969
ENSG00000265962	GACAT2	-2.1	4.8E-05	0.00026	3.389	0.455	2.914	3.963	6.875	1.584	4.801	8.658
ENSG00000107105	ELAVL2	-2.1	1.1E-04	0.00054	0.546	0.179	0.340	0.766	1.177	0.179	0.908	1.273
ENSG00000164309	CMYA5	-2.1	7.6E-06	5.3E-05	0.310	0.089	0.202	0.406	0.663	0.025	0.641	0.687
ENSG00000095585	BLNK	-2.1	3.1E-04	0.00133	0.702	0.201	0.580	1.000	1.722	0.341	1.332	2.008
ENSG00000141431	ASXL3	-2.1	1.1E-07	1.2E-06	0.870	0.165	0.669	1.058	1.899	0.165	1.748	2.041
ENSG00000154736	ADAMTS5	-2.1	1.1E-08	1.6E-07	0.793	0.091	0.723	0.928	1.601	0.090	1.509	1.724
ENSG00000272993		-2.1	1.5E-08	2.1E-07	12.159	2.372	9.305	14.886	25.099	0.279	24.948	25.517
ENSG00000229931		-2.1	1.6E-05	0.0001	4.947	1.228	3.871	6.123	11.004	2.355	7.841	12.800
ENSG00000267731		-2.1	6.2E-04	0.00239	0.660	0.140	0.518	0.835	1.625	0.415	1.130	1.968
ENSG00000248596		-2.1	6.9E-04	0.0026	0.361	0.098	0.253	0.455	0.719	0.043	0.683	0.765
ENSG00000165655	ZNF503	-2.2	7.9E-14	4.66E-12	30.763	2.663	27.710	33.759	66.814	0.551	66.174	67.521
ENSG00000265972	TXNIP	-2.2	9.7E-16	1.03E-13	84.604	3.313	80.157	88.074	181.912	3.734	176.321	184.098
ENSG00000092421	SEMA6A	-2.2	1.6E-11	4.62E-10	3.476	0.362	3.177	3.995	7.440	0.221	7.325	7.772
ENSG00000253846	PCDHGA10	-2.2	2.4E-03	0.00743	0.227	0.065	0.137	0.275	0.606	0.188	0.426	0.769
ENSG00000188729	OSTN	-2.2	1.1E-04	0.00053	0.821	0.353	0.296	1.053	1.733	0.294	1.415	1.982
ENSG00000134986	NREP	-2.2	1.4E-14	1.01E-12	9.829	0.841	9.171	11.033	21.011	0.427	20.525	21.566
ENSG00000015520	NPC1L1	-2.2	5.4E-06	4E-05	0.951	0.132	0.814	1.131	2.199	0.450	1.716	2.582
ENSG00000138347	MYPN	-2.2	1.4E-06	1.2E-05	0.582	0.067	0.518	0.665	1.320	0.098	1.201	1.401
ENSG00000144214	LYG1	-2.2	5.6E-04	0.00219	1.975	0.669	1.018	2.492	4.332	0.748	3.240	4.817
ENSG00000181016	LSMEM1	-2.2	1.7E-06	1.4E-05	1.450	0.338	1.042	1.868	3.286	0.395	2.732	3.570
ENSG00000148798	INA	-2.2	1.1E-03	0.0039	0.518	0.164	0.393	0.749	1.060	0.059	0.972	1.096
ENSG00000164330	EBF1	-2.2	1.4E-05	8.8E-05	0.633	0.121	0.518	0.739	1.412	0.144	1.220	1.524
ENSG00000158486	DNAH3	-2.2	4.6E-07	4.5E-06	0.431	0.108	0.308	0.542	1.008	0.038	0.968	1.061
ENSG00000082196	C1QTNF3	-2.2	2.6E-05	0.00016	1.017	0.300	0.667	1.354	2.406	0.725	1.757	3.034
ENSG00000213199	ASIC3	-2.2	1.2E-02	0.02846	0.506	0.335	0.173	0.965	1.159	0.208	0.987	1.406
ENSG00000274460		-2.2	8.5E-06	5.9E-05	2.242	0.423	1.941	2.866	4.698	0.417	4.387	5.268
ENSG00000271474		-2.2	4.6E-05	0.00025	0.537	0.111	0.412	0.682	1.257	0.111	1.132	1.402
ENSG00000225792		-2.2	5.3E-05	0.00029	2.326	0.604	1.887	3.175	4.854	0.450	4.522	5.475
ENSG00000255389		-2.2	1.1E-04	0.00054	0.958	0.119	0.788	1.063	2.106	0.316	1.752	2.372
ENSG00000272468		-2.2	2.5E-04	0.00109	4.155	1.575	2.426	5.999	8.655	2.060	7.342	11.677
ENSG00000139865	TTC6	-2.3	3.2E-05	0.00019	0.328	0.139	0.200	0.525	0.777	0.105	0.620	0.847
ENSG00000019549	SNAI2	-2.3	2.2E-07	2.3E-06	2.061	0.491	1.451	2.596	4.426	0.097	4.343	4.528
ENSG00000115896	PLCL1	-2.3	5.9E-07	5.6E-06	0.656	0.129	0.510	0.817	1.442	0.277	1.117	1.672
ENSG00000169116	PARM1	-2.3	4.2E-15	3.60E-13	13.322	0.214	13.196	13.642	30.164	1.155	29.411	31.847
ENSG00000175745	NR2F1	-2.3	5.4E-13	2.46E-11	10.255	0.526	9.860	11.015	23.274	1.040	21.837	24.039
ENSG00000078018	MAP2	-2.3	6.6E-11	1.7E-09	5.836	1.027	4.496	6.861	13.503	1.531	12.013	14.824
ENSG00000203814	HIST2H2BF	-2.3	9.2E-07	8.3E-06	1.792	0.487	1.160	2.236	4.127	0.244	3.794	4.309

ENSG00000184678	HIST2H2BE	-2.3	1.6E-09	2.9E-08	4.425	0.636	3.725	5.239	9.667	0.373	9.464	10.226
ENSG00000196787	HIST1H2AG	-2.3	6.6E-10	1.3E-08	4.508	0.611	3.752	5.240	10.018	0.371	9.495	10.281
ENSG00000174844	DNAH12	-2.3	1.6E-05	0.0001	0.167	0.029	0.137	0.206	0.360	0.044	0.337	0.426
ENSG00000135407	AVIL	-2.3	9.7E-07	8.7E-06	0.711	0.043	0.672	0.751	1.745	0.364	1.394	2.059
ENSG00000186517	ARHGAP30	-2.3	1.7E-08	2.4E-07	1.388	0.198	1.225	1.659	3.154	0.121	3.009	3.306
ENSG00000270876	ZNF30-AS1	-2.4	2.0E-04	0.00092	2.566	0.994	1.706	3.874	6.876	0.617	6.439	7.791
ENSG00000137573	SULF1	-2.4	3.8E-13	1.81E-11	4.265	0.591	3.397	4.701	10.015	0.154	9.788	10.111
ENSG00000070731	ST6GALNAC2	-2.4	1.1E-08	1.6E-07	2.359	0.231	2.103	2.655	5.640	0.444	4.988	5.922
ENSG00000178573	MAF	-2.4	1.4E-10	3.2E-09	1.392	0.129	1.201	1.479	3.162	0.052	3.092	3.202
ENSG00000180573	HIST1H2AC	-2.4	2.8E-13	1.42E-11	14.676	1.628	12.386	16.069	34.083	0.698	33.100	34.750
ENSG00000087589	CASS4	-2.4	2.3E-05	0.00014	0.520	0.140	0.320	0.631	1.202	0.152	1.070	1.334
ENSG00000224294		-2.4	5.4E-07	5.1E-06	1.839	0.563	1.213	2.581	4.772	0.536	4.190	5.227
ENSG00000278238		-2.4	9.3E-07	8.4E-06	6.005	0.973	5.162	7.408	13.647	0.936	12.715	14.947
ENSG00000250519		-2.4	1.7E-05	0.00011	2.562	0.757	1.465	3.077	6.125	0.780	5.121	7.024
ENSG00000284526		-2.4	6.0E-05	0.00032	0.365	0.142	0.268	0.573	0.916	0.057	0.861	0.996
ENSG00000283235		-2.4	2.4E-04	0.00107	0.858	0.258	0.500	1.089	2.102	0.156	1.870	2.214
ENSG00000272486		-2.4	3.3E-04	0.0014	1.278	0.392	0.724	1.646	3.165	0.441	2.530	3.549
ENSG00000260727	SLC7A5P1	-2.5	1.0E-04	0.00052	3.117	1.344	1.832	4.963	7.775	1.808	5.096	9.068
ENSG00000075223	SEMA3C	-2.5	5.1E-10	1E-08	10.806	2.131	7.784	12.700	26.051	1.381	24.473	27.209
ENSG00000113389	NPR3	-2.5	1.0E-05	7E-05	0.317	0.123	0.224	0.494	0.733	0.223	0.604	1.067
ENSG00000163121	NEURL3	-2.5	2.3E-04	0.00102	0.414	0.115	0.305	0.573	0.924	0.143	0.831	1.132
ENSG00000182308	DCAF4L1	-2.5	1.6E-05	9.9E-05	0.590	0.078	0.511	0.698	1.535	0.043	1.484	1.571
ENSG00000145087	STXBP5L	-2.6	5.0E-06	3.7E-05	0.284	0.087	0.158	0.357	0.690	0.142	0.604	0.900
ENSG00000188738	FSIP2	-2.6	3.2E-03	0.00941	0.084	0.043	0.027	0.122	0.272	0.117	0.140	0.370
ENSG00000164972	C9orf24	-2.6	2.7E-02	0.05675	0.212	0.007	0.205	0.221	0.692	0.061	0.655	0.783
ENSG00000261087		-2.6	3.1E-05	0.00018	0.752	0.245	0.506	1.078	2.057	0.194	1.766	2.169
ENSG00000143469	SYT14	-2.7	1.2E-09	2.3E-08	0.366	0.058	0.285	0.413	0.978	0.115	0.857	1.077
ENSG00000257335	MGAM	-2.7	1.2E-04	0.00059	0.085	0.019	0.059	0.099	0.207	0.036	0.164	0.237
ENSG00000158406	HIST1H4H	-2.7	6.2E-11	1.6E-09	4.282	0.652	3.527	5.110	11.767	0.493	11.039	12.130
ENSG00000158373	HIST1H2BD	-2.7	1.5E-12	5.85E-11	13.869	1.276	12.859	15.736	37.180	2.754	33.364	39.198
ENSG00000132837	DMGDH	-2.7	7.9E-08	9.3E-07	0.499	0.065	0.432	0.589	1.288	0.061	1.245	1.375
ENSG00000276966	HIST1H4E	-2.9	1.2E-07	1.4E-06	1.722	0.445	1.323	2.253	4.849	0.367	4.331	5.196
ENSG00000274290	HIST1H2BE	-2.9	3.6E-06	2.8E-05	1.025	0.325	0.676	1.347	2.543	0.341	2.345	3.051
ENSG00000188959	C9orf152	-2.9	5.4E-06	4E-05	0.415	0.069	0.350	0.485	1.054	0.092	0.974	1.147
ENSG00000268756		-2.9	2.3E-08	3.1E-07	5.942	1.161	4.843	7.581	17.527	2.380	16.067	21.045
ENSG00000169432	SCN9A	-3	2.9E-12	1.07E-10	2.080	0.476	1.388	2.475	6.452	0.597	5.850	6.966
ENSG00000237693	IRGM	-3	2.1E-05	0.00013	0.653	0.349	0.333	1.140	2.015	0.210	1.701	2.144
ENSG00000159208	CIART	-3	3.2E-10	6.8E-09	2.050	0.240	1.740	2.316	5.806	0.827	5.148	6.866

ENSG00000115461	IGFBP5	-3.1	5.4E-16	6.10E-14	6.063	0.501	5.568	6.726	18.532	0.628	18.065	19.458
ENSG00000171587	DSCAM	-3.2	9.7E-03	0.02399	0.022	0.017	0.000	0.036	0.065	0.019	0.037	0.075
ENSG00000187775	DNAH17	-3.3	1.2E-14	9.19E-13	1.243	0.091	1.118	1.314	4.031	0.242	3.823	4.267
ENSG00000113369	ARRDC3	-3.3	2.6E-16	3.11E-14	8.222	1.229	6.828	9.815	26.063	0.710	25.022	26.514
ENSG00000166793	YPEL4	-3.4	1.6E-09	2.9E-08	0.651	0.121	0.496	0.748	2.018	0.178	1.896	2.274
ENSG00000188580	NKAIN2	-3.4	1.4E-06	1.2E-05	0.370	0.131	0.227	0.541	1.130	0.240	0.950	1.457
ENSG00000273802	HIST1H2BG	-3.4	7.0E-11	1.8E-09	2.428	0.267	2.176	2.786	8.276	0.556	7.660	9.011
ENSG00000233030		-3.4	1.3E-05	8.7E-05	0.279	0.053	0.217	0.344	0.998	0.184	0.810	1.156
ENSG00000171951	SCG2	-3.5	5.9E-07	5.6E-06	0.710	0.445	0.325	1.344	2.402	0.142	2.250	2.523
ENSG00000126368	NR1D1	-3.6	6.3E-14	3.85E-12	7.707	0.630	7.087	8.542	26.913	3.452	24.345	31.647
ENSG00000139269	INHBE	-3.6	1.3E-06	1.1E-05	0.548	0.234	0.391	0.896	1.598	0.158	1.482	1.815
ENSG00000183813	CCR4	-3.7	2.8E-06	2.2E-05	0.283	0.162	0.098	0.489	1.017	0.035	0.964	1.040
ENSG00000234424		-3.9	2.0E-08	2.7E-07	1.048	0.337	0.685	1.335	3.988	0.570	3.173	4.383
ENSG00000015592	STMN4	-4.4	3.8E-08	4.8E-07	0.302	0.123	0.134	0.416	1.366	0.111	1.222	1.453
ENSG00000132639	SNAP25	-4.9	1.8E-05	0.00012	0.647	0.221	0.318	0.790	3.131	0.323	2.851	3.416
ENSG00000256779		-5.3	3.1E-09	5.3E-08	0.903	0.396	0.429	1.370	4.667	0.605	3.902	5.382
ENSG00000144188	TRIM43CP	-5.5	8.7E-13	3.76E-11	2.028	0.201	1.827	2.294	10.501	0.691	9.906	11.181
ENSG00000144010	TRIM43B	-5.8	3.3E-14	2.21E-12	3.919	0.922	2.906	5.051	21.859	2.419	20.404	25.446
ENSG00000204510	PRAMEF7	-5.8	1.7E-09	3.1E-08	0.563	0.064	0.488	0.642	2.864	0.628	2.355	3.643
ENSG00000284546		-5.8	8.2E-08	9.6E-07	1.097	0.569	0.668	1.932	6.185	1.287	5.214	7.930
ENSG00000144015	TRIM43	-5.9	1.2E-14	9.13E-13	4.239	0.927	3.199	5.428	23.274	2.683	21.373	27.064
ENSG00000284306		-5.9	4.5E-09	7.3E-08	1.700	0.333	1.227	1.978	9.227	2.293	7.342	11.994
ENSG00000250782		-6.1	2.0E-14	1.34E-12	12.211	1.145	11.338	13.845	69.314	12.385	58.686	81.978
ENSG00000182330	PRAMEF8	-6.2	1.0E-08	1.5E-07	0.472	0.116	0.342	0.590	2.533	0.800	2.100	3.730
ENSG00000277862	PRAMEF34P	-6.3	4.5E-13	2.09E-11	1.802	0.146	1.684	2.013	10.430	1.865	8.820	12.231
ENSG00000283873		-6.3	7.2E-11	1.8E-09	2.256	0.796	1.360	3.037	13.044	1.494	11.760	14.556
ENSG00000284018		-6.3	1.7E-08	2.4E-07	1.416	0.612	0.912	2.304	8.278	2.652	6.000	10.972
ENSG00000229978	PRAMEF36P	-6.4	6.1E-13	2.75E-11	1.728	0.140	1.573	1.897	10.308	1.690	8.845	11.846
ENSG00000283776		-6.6	5.7E-14	3.53E-12	8.881	1.240	7.668	10.573	55.437	9.323	48.525	68.250
ENSG00000235268	KDM4E	-6.8	2.6E-12	9.61E-11	0.674	0.207	0.521	0.971	4.543	0.309	4.095	4.802
ENSG00000250386		-6.9	3.9E-10	8.1E-09	9.266	2.181	6.217	11.170	59.215	18.192	44.468	81.805
ENSG00000283740		-7.1	3.6E-15	3.19E-13	16.036	2.342	14.327	19.473	106.075	18.013	90.894	126.328
ENSG00000236175	SSU72P6	-7.7	1.5E-10	3.5E-09	1.363	0.467	0.704	1.763	10.032	0.459	9.670	10.626
ENSG00000189325	C6orf222	-7.7	4.8E-09	7.8E-08	0.163	0.031	0.122	0.197	1.310	0.138	1.196	1.472
ENSG00000150244	TRIM48	-7.8	1.5E-11	4.5E-10	0.942	0.282	0.646	1.320	6.710	1.426	5.612	8.606
ENSG00000284438		-7.8	2.5E-11	6.9E-10	2.069	0.848	0.891	2.839	15.264	1.291	14.146	16.398
ENSG00000204449	TRIM49C	-8.1	3.5E-16	4.1E-14	1.772	0.256	1.469	2.082	13.557	1.737	12.121	15.625
ENSG00000168930	TRIM49	-8.4	1.4E-15	1.4E-13	2.825	0.488	2.180	3.204	22.602	3.013	20.010	25.625

ENSG00000157765	SLC34A2	-8.4	6.3E-12	2.1E-10	0.334	0.097	0.188	0.389	2.582	0.551	2.139	3.276
ENSG00000249620		-8.7	5.7E-11	1.4E-09	3.836	0.835	2.740	4.501	33.501	8.153	27.310	44.493
ENSG00000233802	TRIM49D2	-8.9	1.6E-15	1.5E-13	1.779	0.152	1.620	1.944	14.759	2.347	12.844	17.639
ENSG00000223417	TRIM49D1	-8.9	1.8E-15	1.7E-13	1.356	0.106	1.253	1.483	11.203	1.769	9.744	13.326
ENSG00000237706	TRIM51EP	-9	9.6E-13	4.1E-11	1.282	0.309	0.998	1.718	10.516	2.211	8.919	13.608
ENSG00000251258	RFPL4B	-9.4	2.5E-09	4.4E-08	0.456	0.233	0.251	0.776	4.084	0.765	3.443	4.960
ENSG00000204455	TRIM51BP	-9.5	4.3E-13	2.0E-11	1.391	0.404	1.114	1.988	12.098	2.392	10.301	15.353
ENSG00000269466		-9.6	3.4E-13	1.6E-11	23.282	5.069	19.932	30.732	218.205	24.844	197.802	248.265
ENSG00000249156		-9.7	2.1E-12	8.1E-11	6.055	2.023	3.929	8.804	56.141	10.221	47.843	68.797
ENSG00000257951		-10.1	1.4E-12	5.6E-11	1.894	0.500	1.200	2.363	18.124	4.011	15.793	24.130
ENSG00000189253	TRIM64B	-10.3	2.0E-12	7.7E-11	0.459	0.194	0.267	0.722	4.465	0.582	4.020	5.243
ENSG00000225581	TRIM53AP	-10.4	2.8E-12	1.0E-10	0.932	0.203	0.643	1.119	8.813	2.795	6.613	12.440
ENSG00000180532	ZSCAN4	-10.6	3.4E-13	1.7E-11	1.511	0.633	0.565	1.900	14.940	2.600	12.698	17.463
ENSG00000116726	PRAMEF12	-11.1	1.3E-11	4.1E-10	0.387	0.171	0.165	0.534	3.898	0.647	3.394	4.749
ENSG00000166013	TRIM53BP	-11.3	1.2E-12	5.1E-11	0.633	0.169	0.439	0.851	6.755	1.583	5.589	8.940
ENSG00000204450	TRIM64	-11.6	7.0E-09	1.1E-07	0.148	0.094	0.079	0.283	1.670	0.563	1.201	2.325
ENSG00000254764	TRIM53CP	-11.8	3.8E-11	1.0E-09	0.547	0.196	0.375	0.822	6.115	1.497	4.438	8.083
ENSG00000182053	TRIM49B	-12.1	2.4E-12	8.9E-11	0.519	0.146	0.392	0.676	5.631	1.486	4.577	7.729
ENSG00000268799		-12.9	9.3E-12	3.0E-10	3.824	1.207	2.035	4.654	48.173	13.156	36.991	62.445
ENSG00000106278	PTPRZ1	-13.9	7.0E-02	1.2E-01	0.008	0.009	0.000	0.015	0.126	0.252	0.000	0.503
ENSG00000236941		-18.4	2.9E-11	8.1E-10	0.307	0.167	0.117	0.448	5.833	1.162	5.162	7.565
ENSG00000255855	KDM4F	-19.2	2.6E-12	9.5E-11	0.248	0.120	0.079	0.346	4.648	0.841	4.078	5.859

					Transcripts per million (TPM)							
Gene		Linear	P Value	FDR	TestMea	TestStdD	TestMin	TestMax	RefMea	RefStdD	RefMin	RefMax
ENSG00000259456	ADNP-AS1	-1.5	5.1E-02	0.09682	2.572	0.598	2.016	3.419	3.144	1.132	1.525	4.167
ENSG00000279355	AGPAT4-IT1	-1.5	3.3E-02	0.06816	1.834	0.583	1.371	2.653	2.735	0.079	2.657	2.803
ENSG00000189060	H1F0	-1.5	5.2E-11	1.5E-09	301.195	11.230	286.121	310.652	432.005	12.983	420.765	443.633
ENSG00000176788	BASP1	-1.5	3.7E-10	8.3E-09	190.272	6.726	182.452	197.936	269.107	11.216	259.495	280.689
ENSG00000277443	MARCKS	-1.5	8.0E-11	2.2E-09	147.635	2.665	145.160	151.376	203.288	11.154	192.025	212.875
ENSG00000143032	BARHL2	-1.5	1.7E-02	0.0396	1.371	0.367	1.023	1.802	1.811	0.230	1.612	2.033
ENSG00000187837	HIST1H1C	-1.5	4.5E-08	5.8E-07	129.057	8.112	118.174	135.751	174.671	7.779	168.983	185.463
ENSG00000151725	CENPU	-1.5	4.0E-09	6.6E-08	101.855	5.016	98.944	109.339	146.155	13.334	134.582	157.703
ENSG00000148677	ANKRD1	-1.5	2.2E-04	0.00096	94.766	10.203	81.344	106.157	123.701	3.845	118.029	126.084
ENSG00000149201	CCDC81	-1.5	2.3E-02	0.04955	0.591	0.169	0.349	0.742	0.824	0.103	0.726	0.913
ENSG00000087074	PPP1R15A	-1.5	6.0E-08	7.4E-07	76.433	3.289	72.448	80.119	104.133	3.119	101.511	107.674
ENSG00000251562	MALAT1	-1.5	1.8E-05	0.00011	74.589	8.074	64.211	83.489	107.023	16.914	85.852	120.671
ENSG00000115738	ID2	-1.5	2.0E-10	4.8E-09	72.631	3.510	69.105	77.452	101.783	1.435	99.644	102.726
ENSG00000135269	TES	-1.5	3.0E-10	6.9E-09	44.751	2.985	40.819	48.064	62.171	1.028	61.281	63.089
ENSG00000174371	EXO1	-1.5	1.3E-08	1.8E-07	39.731	1.850	37.185	41.579	53.792	2.961	50.349	56.263
ENSG00000270882	HIST2H4A	-1.5	1.1E-08	1.6E-07	37.608	1.981	35.205	39.517	53.350	0.763	52.389	54.255
ENSG00000112742	TTK	-1.5	2.1E-04	0.00092	37.307	0.855	36.230	38.199	51.404	3.717	47.032	54.492
ENSG00000141665	FBXO15	-1.5	1.3E-02	0.03222	0.485	0.106	0.415	0.643	0.649	0.024	0.623	0.669
ENSG00000170558	CDH2	-1.5	5.5E-12	2.11E-10	36.633	0.937	35.357	37.457	52.665	1.078	51.726	53.599
ENSG00000149948	HMGA2	-1.5	1.2E-09	2.3E-08	36.373	1.746	34.639	37.925	49.437	1.989	47.564	51.157
ENSG00000270276	HIST2H4B	-1.5	1.2E-08	1.7E-07	36.309	2.019	34.075	38.351	51.871	0.882	50.596	52.626
ENSG00000105974	CAV1	-1.5	1.3E-09	2.5E-08	35.354	2.494	32.821	37.996	51.000	2.803	48.564	53.427
ENSG00000174738	NR1D2	-1.5	5.9E-07	5.5E-06	33.458	1.266	32.287	35.057	48.209	2.575	45.026	50.302
ENSG00000170017	ALCAM	-1.5	2.3E-08	3.2E-07	33.194	0.899	32.221	34.049	46.056	2.621	42.799	48.180
ENSG00000168564	CDKN2AIP	-1.5	9.8E-09	1.5E-07	31.861	0.349	31.516	32.237	43.937	1.507	42.308	45.219
ENSG00000174579	MSL2	-1.5	5.2E-08	6.5E-07	30.487	1.510	28.981	32.270	40.833	0.999	39.712	42.145
ENSG00000248873	SERBP1P6	-1.5	1.8E-07	2E-06	29.704	2.786	27.708	33.830	40.640	2.064	37.782	42.716
ENSG00000138814	PPP3CA	-1.5	1.9E-09	3.5E-08	29.164	1.757	27.393	31.585	40.292	0.377	40.000	40.789
ENSG00000236539	HNRNPA1P54	-1.5	2.1E-02	0.04726	0.258	0.051	0.205	0.322	0.340	0.021	0.326	0.371
ENSG00000171476	HOPX	-1.5	1.3E-02	0.03221	0.380	0.057	0.325	0.460	0.581	0.148	0.405	0.768
ENSG00000218336	TENM3	-1.5	2.7E-08	3.6E-07	27.000	2.096	24.277	29.386	37.294	1.636	35.059	38.517
ENSG00000143333	RGS16	-1.5	1.3E-06	1.1E-05	25.603	1.262	24.318	27.342	34.887	4.315	31.159	38.963
ENSG00000279208		-1.5	3.6E-05	0.0002	24.877	3.904	21.294	29.642	34.773	2.452	31.521	36.667
ENSG00000272767	JMJD1C-AS1	-1.5	1.8E-02	0.04034	2.733	0.784	2.226	3.901	3.756	0.361	3.255	4.118
ENSG00000205189	ZBTB10	-1.5	4.5E-06	3.2E-05	24.498	1.700	22.011	25.746	36.033	2.289	33.560	37.980
ENSG00000196781	TLE1	-1.5	1.6E-09	3E-08	23.701	0.978	22.885	25.101	33.812	0.803	33.262	34.964

ENSG00000128052	KDR	-1.5	3.0E-02	0.06305	0.504	0.106	0.367	0.597	0.713	0.149	0.586	0.874
ENSG00000137831	UACA	-1.5	8.2E-03	0.02129	23.306	1.671	21.026	25.049	32.677	3.630	28.309	35.665
ENSG00000116044	NFE2L2	-1.5	3.4E-09	5.8E-08	22.951	0.853	21.930	23.881	33.367	2.461	30.371	35.382
ENSG00000248971	KRT8P46	-1.5	5.2E-02	0.09851	1.605	0.707	0.859	2.327	2.250	0.447	1.625	2.573
ENSG00000233930	KRTAP5-AS1	-1.5	1.2E-02	0.02966	0.972	0.141	0.811	1.127	1.564	0.229	1.233	1.757
ENSG00000123094	RASSF8	-1.5	3.3E-04	0.00138	21.268	1.923	18.740	23.165	29.179	2.275	26.617	31.095
ENSG00000233639	LINC01158	-1.5	4.0E-02	0.08029	3.255	1.179	1.661	4.285	4.339	0.235	4.159	4.653
ENSG00000214900	LINC01588	-1.5	9.4E-02	0.159	0.118	0.032	0.075	0.148	0.183	0.035	0.153	0.234
ENSG00000138050	THUMPD2	-1.5	1.3E-08	1.9E-07	20.929	0.867	20.448	22.229	28.414	0.922	27.611	29.212
ENSG00000124635	HIST1H2BJ	-1.5	6.8E-06	4.7E-05	20.487	1.222	19.343	21.785	28.627	0.595	28.129	29.312
ENSG00000133639	BTG1	-1.5	1.3E-09	2.5E-08	19.839	0.988	18.498	20.650	28.357	1.059	27.086	29.679
ENSG00000165072	MAMDC2	-1.5	2.6E-02	0.05502	0.677	0.106	0.612	0.835	1.106	0.067	1.019	1.159
ENSG00000177565	TBL1XR1	-1.5	1.9E-04	0.00085	19.257	1.751	17.225	21.502	28.339	2.119	26.191	30.160
ENSG00000165480	SKA3	-1.5	1.1E-07	1.2E-06	19.199	2.403	16.354	21.902	26.910	0.561	26.246	27.375
ENSG00000172059	KLF11	-1.5	2.4E-09	4.2E-08	18.867	0.806	17.819	19.784	27.086	1.440	26.213	29.218
ENSG00000168556	ING2	-1.5	3.4E-07	3.4E-06	17.955	1.903	15.659	20.003	26.493	1.855	23.930	27.858
ENSG00000066468	FGFR2	-1.5	6.4E-10	1.3E-08	17.434	0.841	16.538	18.555	25.193	1.064	23.896	26.064
ENSG00000197050	ZNF420	-1.5	4.4E-06	3.2E-05	16.789	1.237	15.686	17.941	23.335	2.195	20.850	25.182
ENSG00000166866	MYO1A	-1.5	1.5E-02	0.03433	0.634	0.100	0.531	0.759	0.978	0.162	0.798	1.114
ENSG00000139946	PELI2	-1.5	1.4E-06	1.2E-05	16.587	1.473	14.753	18.331	23.347	1.403	21.896	24.548
ENSG00000077684	JADE1	-1.5	6.5E-10	1.3E-08	15.585	0.673	14.848	16.469	22.190	1.291	20.850	23.296
ENSG00000259485	LINC02253	-1.5	2.2E-05	0.00013	15.362	1.447	13.674	16.612	20.195	1.141	19.519	21.890
ENSG00000134986	NREP	-1.5	3.3E-09	5.6E-08	15.292	0.688	14.652	16.084	21.011	0.427	20.525	21.566
ENSG00000165555	NOXRED1	-1.5	2.8E-02	0.05979	1.139	0.331	0.676	1.431	1.375	0.309	1.152	1.809
ENSG00000123572	NRK	-1.5	4.0E-02	0.07911	0.293	0.078	0.181	0.358	0.392	0.122	0.213	0.488
ENSG00000118922	KLF12	-1.5	1.3E-04	0.00061	14.467	1.719	11.906	15.551	19.967	1.166	18.853	20.974
ENSG00000253846	PCDHGA10	-1.5	7.8E-02	0.13716	0.345	0.086	0.268	0.438	0.606	0.188	0.426	0.769
ENSG00000023608	SNAPC1	-1.5	4.1E-06	3E-05	13.148	0.956	12.430	14.532	17.671	1.448	15.850	19.393
ENSG00000121361	KCNJ8	-1.5	7.1E-07	6.5E-06	12.742	0.744	12.121	13.674	17.659	0.769	16.654	18.528
ENSG00000106571	GLI3	-1.5	7.8E-08	9.3E-07	12.558	1.197	11.046	13.786	17.970	0.810	16.900	18.598
ENSG00000164776	PHKG1	-1.5	3.0E-02	0.06198	1.333	0.416	0.789	1.793	2.076	0.066	2.022	2.156
ENSG00000102032	RENBP	-1.5	4.3E-07	4.2E-06	12.116	1.066	10.797	13.400	17.251	1.066	16.520	18.780
ENSG00000100142	POLR2F	-1.5	3.2E-02	0.06671	0.456	0.109	0.305	0.563	0.617	0.092	0.566	0.754
ENSG00000228169	PPIAP19	-1.5	3.4E-02	0.06887	4.704	1.549	2.586	6.178	6.219	1.060	4.735	7.253
ENSG00000198732	SMOC1	-1.5	3.9E-06	2.9E-05	10.744	1.108	9.934	12.305	14.749	1.891	13.146	16.826
ENSG00000229083	PSMA6P2	-1.5	1.1E-02	0.02652	5.708	0.671	5.043	6.353	7.372	0.901	6.628	8.447
ENSG00000050628	PTGER3	-1.5	7.2E-02	0.12916	0.284	0.055	0.244	0.365	0.388	0.030	0.358	0.413
ENSG00000239269	RPSAP4	-1.5	1.1E-04	0.00054	10.464	1.006	9.282	11.412	14.375	1.465	12.213	15.282

ENSG00000233045		-1.5	9.5E-04	0.00346	9.876	1.897	7.099	11.336	14.144	0.867	13.482	15.308
ENSG00000146707	POMZP3	-1.5	1.7E-05	0.00011	9.669	0.472	9.098	10.059	13.325	0.741	12.816	14.386
ENSG00000134438	RAX	-1.5	1.3E-02	0.03193	1.232	0.225	0.971	1.521	1.751	0.433	1.413	2.319
ENSG00000249465	RBMXP4	-1.5	1.3E-02	0.03109	2.830	0.567	2.009	3.286	4.228	0.749	3.106	4.631
ENSG00000168621	GDNF	-1.5	9.4E-08	1.1E-06	9.200	0.778	8.096	9.822	13.102	0.742	12.356	13.740
ENSG00000064042	LIMCH1	-1.5	2.3E-09	4E-08	9.189	0.297	8.903	9.534	13.309	0.387	12.784	13.601
ENSG00000196159	FAT4	-1.5	5.3E-05	0.00028	9.015	1.050	7.519	9.869	13.033	0.653	12.083	13.461
ENSG00000259802		-1.5	9.3E-04	0.0034	8.893	1.103	7.475	10.030	12.344	1.959	10.286	15.007
ENSG00000168772	CXXC4	-1.5	7.1E-05	0.00036	8.805	0.591	7.972	9.361	13.051	1.140	12.040	14.038
ENSG00000147852	VLDLR	-1.5	3.1E-09	5.3E-08	8.543	0.592	7.695	9.004	12.311	0.236	12.186	12.665
ENSG00000165617	DACT1	-1.5	2.0E-07	2.1E-06	8.121	0.683	7.283	8.956	11.473	0.434	10.827	11.726
ENSG00000224892	RPS4XP16	-1.5	1.7E-02	0.03842	3.586	1.016	2.129	4.470	5.010	0.489	4.641	5.672
ENSG00000237440	ZNF737	-1.5	1.1E-05	7E-05	7.875	0.689	7.117	8.788	10.754	0.692	9.780	11.416
ENSG00000272341		-1.5	5.2E-05	0.00028	7.868	1.565	6.691	10.139	10.998	0.893	10.021	11.755
ENSG00000147862	NFIB	-1.5	1.2E-06	1E-05	7.548	0.495	7.071	8.240	11.049	0.829	9.943	11.686
ENSG00000185483	ROR1	-1.5	6.9E-08	8.4E-07	7.411	0.612	6.791	8.212	10.323	0.332	9.924	10.596
ENSG00000203943	SAMD13	-1.5	4.5E-02	0.08835	0.895	0.108	0.745	1.001	1.462	0.367	0.932	1.709
ENSG00000196081	ZNF724	-1.5	4.3E-06	3.2E-05	7.232	0.339	6.927	7.607	11.021	1.979	8.750	12.679
ENSG00000220804	LINC01881	-1.5	3.1E-05	0.00018	7.123	0.457	6.607	7.631	9.605	0.911	8.238	10.076
ENSG00000188404	SELL	-1.5	2.1E-02	0.04584	0.818	0.228	0.635	1.124	1.082	0.068	0.980	1.122
ENSG00000226102	SEPT7P3	-1.5	3.2E-02	0.06656	3.989	0.865	2.807	4.866	4.894	1.145	3.949	6.265
ENSG00000240914		-1.5	5.9E-03	0.01617	6.836	1.359	5.047	8.353	9.562	0.507	9.230	10.301
ENSG00000163535	SGO2	-1.5	3.9E-02	0.07799	16.502	2.992	13.757	20.449	22.989	3.310	19.042	25.724
ENSG00000180376	CCDC66	-1.5	7.1E-05	0.00037	6.632	0.307	6.453	7.091	9.767	1.372	8.187	10.915
ENSG00000139174	PRICKLE1	-1.5	6.1E-09	9.7E-08	6.628	0.394	6.063	6.966	9.224	0.372	8.666	9.427
ENSG00000164463	CREBRF	-1.5	1.6E-04	0.00072	6.569	0.534	5.903	7.212	9.072	0.432	8.575	9.629
ENSG00000085276	MECOM	-1.5	6.7E-05	0.00035	6.473	0.628	5.752	7.151	8.782	0.419	8.240	9.114
ENSG00000261116		-1.5	4.8E-04	0.00192	6.373	0.902	5.288	7.448	8.676	0.463	8.045	9.160
ENSG00000004799	PDK4	-1.5	2.5E-06	1.9E-05	6.369	0.657	5.672	7.050	9.179	0.615	8.299	9.603
ENSG00000147606	SLC26A7	-1.5	3.3E-02	0.0685	0.487	0.124	0.411	0.670	0.776	0.257	0.401	0.941
ENSG00000158014	SLC30A2	-1.5	5.9E-02	0.10971	0.773	0.236	0.450	0.963	0.933	0.292	0.684	1.242
ENSG00000011201	ANOS1	-1.5	2.2E-06	1.8E-05	5.969	0.390	5.514	6.459	8.544	0.265	8.171	8.800
ENSG00000235245		-1.5	4.9E-03	0.01378	5.966	1.250	4.747	7.712	9.165	0.928	7.779	9.685
ENSG00000084453	SLCO1A2	-1.5	2.1E-02	0.0469	0.364	0.137	0.257	0.545	0.516	0.099	0.391	0.634
ENSG00000178878	APOLD1	-1.5	2.6E-06	2E-05	5.662	0.387	5.211	6.099	8.100	0.426	7.807	8.734
ENSG00000276644	DACH1	-1.5	7.1E-06	4.9E-05	5.579	0.603	4.844	6.175	8.239	0.814	7.437	8.941
ENSG00000176971	FIBIN	-1.5	2.8E-04	0.00119	5.556	0.398	5.058	6.026	7.189	0.889	6.421	8.024
ENSG00000049246	PER3	-1.5	2.1E-07	2.2E-06	5.497	0.440	4.912	5.976	8.123	0.659	7.135	8.470

ENSG00000077522	ACTN2	-1.5	2.6E-06	2.1E-05	5.478	0.313	5.051	5.772	7.996	0.938	6.666	8.655
ENSG00000204682	CASC10	-1.5	8.5E-05	0.00043	5.285	1.033	4.326	6.447	6.922	0.629	6.422	7.728
ENSG00000112182	BACH2	-1.5	4.7E-08	6E-07	5.280	0.415	4.805	5.791	7.738	0.481	7.089	8.103
ENSG00000113916	BCL6	-1.5	3.4E-07	3.4E-06	5.096	0.470	4.502	5.560	6.859	0.213	6.560	7.012
ENSG00000226763	SRRM5	-1.5	2.4E-02	0.05299	0.907	0.316	0.514	1.212	1.388	0.070	1.339	1.492
ENSG00000196782	MAML3	-1.5	2.1E-06	1.7E-05	5.016	0.424	4.508	5.419	6.707	0.074	6.666	6.818
ENSG00000119866	BCL11A	-1.5	4.9E-07	4.7E-06	4.960	0.534	4.187	5.345	7.305	0.444	6.677	7.620
ENSG00000263080		-1.5	2.8E-03	0.00852	4.890	1.154	3.570	6.348	7.834	1.692	6.095	9.285
ENSG00000198075	SULT1C4	-1.5	1.2E-02	0.02892	1.366	0.447	0.922	1.982	1.838	0.394	1.444	2.385
ENSG00000229124	VIM-AS1	-1.5	1.3E-03	0.00457	4.625	1.084	3.381	5.785	6.290	0.884	5.172	7.334
ENSG00000061455	PRDM6	-1.5	1.4E-06	1.1E-05	4.584	0.534	3.784	4.873	6.515	0.461	5.957	6.893
ENSG00000148541	FAM13C	-1.5	1.5E-06	1.3E-05	4.525	0.221	4.308	4.781	6.115	0.105	6.048	6.269
ENSG00000180596	HIST1H2BC	-1.5	6.3E-03	0.01706	4.407	0.452	3.800	4.855	6.057	0.999	4.632	6.969
ENSG00000242952	RPSAP3	-1.5	4.9E-03	0.01367	4.245	0.649	3.438	4.792	5.964	0.711	5.019	6.513
ENSG00000234911	TEX21P	-1.5	2.1E-02	0.04705	2.261	0.466	1.688	2.663	3.397	1.031	2.072	4.215
ENSG00000152217	SETBP1	-1.5	2.8E-06	2.1E-05	4.081	0.745	3.342	5.116	5.680	0.427	5.322	6.170
ENSG00000185339	TCN2	-1.5	4.0E-05	0.00022	4.057	0.590	3.426	4.735	6.006	0.130	5.826	6.138
ENSG00000180998	GPR137C	-1.5	4.6E-05	0.00025	3.972	0.029	3.936	4.007	6.089	0.898	5.120	6.852
ENSG00000267796	LIN37	-1.5	1.4E-04	0.00065	3.965	0.465	3.290	4.355	5.210	0.332	4.922	5.515
ENSG00000116117	PARD3B	-1.5	3.5E-07	3.4E-06	3.908	0.308	3.665	4.323	5.543	0.281	5.220	5.778
ENSG00000114423	CBLB	-1.5	4.5E-05	0.00025	3.756	0.727	3.108	4.735	5.465	0.287	5.207	5.713
ENSG00000251634		-1.5	9.6E-05	0.00047	3.749	0.292	3.503	4.171	5.221	0.164	5.077	5.363
ENSG00000275342	PRAG1	-1.5	5.8E-05	0.0003	3.729	0.353	3.207	3.969	4.986	0.852	4.294	6.039
ENSG00000146070	PLA2G7	-1.5	3.2E-03	0.00965	3.695	0.294	3.422	4.036	5.347	0.206	5.040	5.482
ENSG00000119865	CNRIP1	-1.5	1.6E-04	0.00073	3.634	0.474	3.014	4.170	4.655	0.344	4.407	5.136
ENSG00000218890		-1.5	6.8E-03	0.01805	3.565	0.582	2.948	4.085	4.890	1.227	3.558	5.933
ENSG00000249069	LINC01033	-1.5	2.2E-03	0.00702	3.529	0.322	3.087	3.770	5.052	0.307	4.723	5.314
ENSG00000077063	CTTNBP2	-1.5	1.7E-06	1.4E-05	3.481	0.200	3.219	3.697	4.719	0.175	4.599	4.979
ENSG00000185070	FLRT2	-1.5	6.7E-08	8.1E-07	3.332	0.148	3.199	3.474	4.688	0.432	4.202	5.051
ENSG00000138028	CGREF1	-1.5	5.3E-05	0.00028	3.271	0.461	2.768	3.701	4.449	0.541	4.161	5.261
ENSG00000279369		-1.5	7.9E-04	0.00294	3.247	0.529	2.606	3.863	4.999	0.804	4.148	5.685
ENSG00000277462	ZNF670	-1.5	5.0E-03	0.01391	3.147	0.530	2.655	3.775	4.718	0.793	3.545	5.201
ENSG00000182168	UNC5C	-1.5	3.2E-06	2.4E-05	3.082	0.390	2.561	3.498	4.386	0.321	4.037	4.659
ENSG00000250546		-1.5	1.0E-03	0.00367	2.951	0.566	2.454	3.754	4.316	0.390	3.813	4.624
ENSG00000218358	RAET1K	-1.5	1.9E-04	0.00084	2.910	0.133	2.733	3.039	4.428	0.509	3.920	4.866
ENSG00000221994	ZNF630	-1.5	1.7E-04	0.00076	2.900	0.247	2.669	3.250	3.724	0.487	3.342	4.359
ENSG00000271918		-1.5	4.2E-03	0.01218	2.839	0.448	2.442	3.445	4.060	0.572	3.610	4.899
ENSG00000235109	ZSCAN31	-1.5	4.5E-05	0.00025	2.728	0.333	2.410	3.197	3.767	0.494	3.215	4.184

ENSG00000258405	ZNF578	-1.5	1.1E-05	7.2E-05	2.664	0.248	2.384	2.914	3.856	0.414	3.318	4.181
ENSG00000214892	USP8P1	-1.5	2.1E-02	0.04662	0.688	0.134	0.560	0.842	0.926	0.188	0.691	1.078
ENSG00000153832	FBXO36	-1.5	6.4E-04	0.00243	2.586	0.351	2.109	2.950	3.918	0.349	3.424	4.165
ENSG00000106804	C5	-1.5	5.3E-05	0.00028	2.570	0.412	2.070	3.051	3.672	0.566	2.909	4.103
ENSG00000272870		-1.5	5.6E-04	0.00217	2.492	0.406	1.954	2.938	3.511	0.303	3.226	3.773
ENSG00000250120	PCDHA10	-1.5	1.4E-05	8.8E-05	2.452	0.319	2.127	2.762	3.713	0.523	3.106	4.150
ENSG00000270964		-1.5	5.8E-03	0.01591	2.426	0.379	2.098	2.946	3.119	0.606	2.706	3.990
ENSG00000260589	STAM-AS1	-1.5	2.7E-03	0.00828	2.346	0.575	1.757	3.069	3.410	0.499	2.663	3.689
ENSG00000233822	HIST1H2BN	-1.5	6.4E-05	0.00033	2.225	0.157	2.025	2.386	3.298	0.043	3.262	3.350
ENSG00000272711		-1.5	5.7E-03	0.01572	2.142	0.425	1.652	2.570	3.046	0.182	2.777	3.182
ENSG00000183671	GPR1	-1.5	2.3E-03	0.00727	2.128	0.325	1.838	2.586	2.992	0.257	2.613	3.153
ENSG00000166578	IQCD	-1.5	1.8E-03	0.006	2.046	0.167	1.918	2.279	3.142	0.326	2.682	3.372
ENSG00000270876	ZNF30-AS1	-1.5	2.8E-02	0.05994	4.307	0.931	3.019	5.071	6.876	0.617	6.439	7.791
ENSG00000187624	C17orf97	-1.5	8.8E-03	0.02253	1.798	0.393	1.462	2.299	2.645	0.201	2.473	2.851
ENSG00000176771	NCKAP5	-1.5	1.3E-05	8.5E-05	1.780	0.050	1.712	1.828	2.483	0.153	2.405	2.713
ENSG00000250492	INTS6P1	-1.5	2.1E-03	0.00676	1.744	0.149	1.544	1.877	2.504	0.405	2.018	2.838
ENSG00000137672	TRPC6	-1.5	8.0E-04	0.00298	1.697	0.359	1.193	1.968	2.333	0.116	2.224	2.497
ENSG00000130518	KIAA1683	-1.5	2.5E-05	0.00015	1.687	0.068	1.616	1.774	2.427	0.121	2.335	2.591
ENSG00000146674	IGFBP3	-1.5	9.4E-04	0.00343	1.658	0.182	1.431	1.858	2.300	0.050	2.234	2.338
ENSG00000178568	ERBB4	-1.5	1.2E-04	0.00059	1.640	0.199	1.353	1.783	2.344	0.286	2.028	2.586
ENSG00000270011	ZNF559-ZNF177	-1.5	6.5E-02	0.11928	0.583	0.146	0.371	0.699	0.781	0.301	0.339	0.970
ENSG00000049192	ADAMTS6	-1.5	1.5E-03	0.00498	1.581	0.268	1.318	1.891	2.082	0.206	1.841	2.253
ENSG00000134532	SOX5	-1.5	2.2E-05	0.00013	1.560	0.243	1.239	1.806	2.216	0.280	1.812	2.457
ENSG00000138653	NDST4	-1.5	1.7E-03	0.00561	1.550	0.118	1.438	1.717	2.086	0.221	1.904	2.408
ENSG00000150636	CCDC102B	-1.5	1.1E-03	0.00404	1.448	0.065	1.382	1.536	1.874	0.193	1.759	2.163
ENSG00000205592	MUC19	-1.5	5.8E-03	0.01576	1.441	0.215	1.136	1.639	2.230	0.474	1.726	2.635
ENSG00000196724	ZNF418	-1.5	2.7E-03	0.00847	1.435	0.247	1.144	1.695	2.301	0.372	1.967	2.623
ENSG00000180592	SKIDA1	-1.5	8.3E-05	0.00042	1.415	0.154	1.197	1.537	1.957	0.217	1.641	2.138
ENSG00000103460	TOX3	-1.5	6.7E-03	0.01783	1.373	0.453	0.938	1.981	1.866	0.301	1.681	2.310
ENSG00000105792	CFAP69	-1.5	7.6E-04	0.00284	1.359	0.139	1.187	1.517	1.860	0.029	1.832	1.885
ENSG00000157168	NRG1	-1.5	8.2E-05	0.00041	1.309	0.154	1.113	1.471	1.769	0.145	1.636	1.975
ENSG00000260339	HEXA-AS1	-1.5	6.2E-03	0.01669	1.243	0.133	1.058	1.374	1.787	0.352	1.409	2.087
ENSG00000141934	PLPP2	-1.5	3.0E-04	0.00127	1.214	0.127	1.063	1.368	1.641	0.149	1.514	1.801
ENSG00000235618	FAM21EP	-1.5	6.5E-03	0.0174	1.152	0.136	1.033	1.295	1.651	0.303	1.491	2.105
ENSG00000269906		-1.5	5.9E-02	0.10913	3.546	1.788	1.883	5.931	5.548	0.580	4.703	6.024
ENSG00000231458		-1.5	7.1E-02	0.12671	3.048	0.418	2.714	3.636	4.967	1.610	3.566	6.361
ENSG00000250790		-1.5	7.8E-02	0.1379	0.642	0.271	0.282	0.916	0.965	0.164	0.722	1.085
ENSG00000166262	FAM227B	-1.5	7.6E-03	0.02001	1.125	0.079	1.048	1.234	1.372	0.354	1.025	1.866

ENSG00000152402	GUCY1A2	-1.5	1.1E-04	0.00054	1.111	0.164	0.903	1.285	1.570	0.080	1.488	1.638
ENSG00000259275		-1.5	6.7E-03	0.01787	0.993	0.115	0.890	1.098	1.607	0.412	1.153	1.956
ENSG00000169031	COL4A3	-1.5	2.1E-04	0.00092	0.941	0.137	0.755	1.075	1.387	0.125	1.200	1.451
ENSG00000137090	DMRT1	-1.5	8.3E-03	0.02155	0.915	0.191	0.792	1.197	1.273	0.116	1.159	1.373
ENSG00000109906	ZBTB16	-1.5	6.9E-04	0.00261	0.886	0.171	0.666	1.057	1.249	0.137	1.177	1.454
ENSG00000106483	SFRP4	-1.5	8.9E-03	0.02278	0.849	0.232	0.510	1.036	1.192	0.132	1.027	1.350
ENSG00000176884	GRIN1	-1.5	2.7E-03	0.00837	0.727	0.071	0.648	0.818	1.006	0.032	0.980	1.053
ENSG00000258472		-1.5	1.2E-02	0.02923	0.853	0.092	0.777	0.980	1.126	0.090	1.025	1.202
ENSG00000247728		-1.5	1.3E-02	0.03117	3.168	0.586	2.346	3.654	4.624	0.295	4.232	4.852
ENSG00000240291		-1.5	1.3E-02	0.03147	3.290	0.854	2.012	3.794	4.952	0.892	3.679	5.571
ENSG00000246528		-1.5	1.5E-02	0.03603	1.755	0.273	1.359	1.979	2.446	0.451	2.128	3.114
ENSG00000254427		-1.5	1.6E-02	0.03816	1.922	0.187	1.723	2.144	3.205	0.510	2.504	3.584
ENSG00000283403		-1.5	1.7E-02	0.04008	1.260	0.531	0.891	2.021	1.919	0.154	1.758	2.050
ENSG00000262728		-1.5	1.8E-02	0.04057	2.331	0.656	1.866	3.303	3.117	0.585	2.723	3.963
ENSG00000226468		-1.5	1.8E-02	0.04173	3.867	0.984	2.935	5.195	5.946	1.269	4.044	6.614
ENSG00000226754		-1.5	2.0E-02	0.04465	2.283	0.520	1.686	2.769	3.273	0.461	2.926	3.953
ENSG00000271888		-1.5	2.1E-02	0.04713	2.799	0.862	1.750	3.859	3.764	0.505	3.434	4.517
ENSG00000226471		-1.5	2.9E-02	0.06177	1.684	0.321	1.348	2.119	2.164	0.549	1.736	2.885
ENSG00000251867		-1.5	3.1E-02	0.06397	2.074	0.339	1.618	2.432	2.969	0.163	2.736	3.115
ENSG00000272720		-1.5	4.4E-02	0.08549	1.584	0.434	1.283	2.212	2.562	1.059	1.096	3.340
ENSG00000260244		-1.5	4.6E-02	0.08883	0.915	0.112	0.774	1.022	1.464	0.586	0.781	1.952
ENSG00000279145		-1.5	4.6E-02	0.09005	0.370	0.139	0.216	0.535	0.568	0.050	0.498	0.603
ENSG00000120675	DNAJC15	-1.5	7.6E-03	0.01987	0.601	0.114	0.445	0.704	0.917	0.103	0.762	0.972
ENSG00000148848	ADAM12	-1.5	5.2E-03	0.01449	0.503	0.063	0.419	0.563	0.812	0.224	0.574	1.003
ENSG00000148053	NTRK2	-1.5	7.8E-04	0.00291	0.341	0.033	0.314	0.389	0.432	0.063	0.392	0.525
ENSG00000134294	SLC38A2	-1.6	1.3E-05	8.4E-05	156.295	12.379	138.171	166.136	227.095	7.191	216.333	231.021
ENSG00000271614	ATP2B1-AS1	-1.6	1.3E-02	0.03193	0.648	0.071	0.548	0.709	0.965	0.100	0.879	1.064
ENSG00000135679	MDM2	-1.6	1.9E-07	2.1E-06	126.284	6.528	120.470	135.597	201.327	21.664	178.536	219.843
ENSG00000177606	JUN	-1.6	2.4E-11	7.24E-10	93.578	5.503	86.620	98.780	141.456	1.989	138.844	143.685
ENSG00000135346	CGA	-1.6	1.8E-02	0.04135	1.066	0.284	0.826	1.474	1.518	0.223	1.307	1.832
ENSG00000230043	TMSB4XP6	-1.6	8.2E-05	0.00041	57.032	9.639	48.422	70.762	82.630	5.452	75.415	88.664
ENSG00000259051	HNRNPUP1	-1.6	1.5E-06	1.3E-05	52.348	7.685	45.333	59.105	77.852	2.078	76.654	80.965
ENSG00000149212	SESN3	-1.6	1.6E-04	0.00074	49.564	5.359	41.540	52.601	77.638	6.782	70.716	83.459
ENSG00000114315	HES1	-1.6	3.6E-11	1E-09	48.436	3.362	44.536	52.714	74.558	0.335	74.122	74.940
ENSG00000137193	PIM1	-1.6	9.3E-11	2.5E-09	45.417	2.945	41.254	48.055	67.591	3.549	64.845	72.290
ENSG00000116574	RHOU	-1.6	1.7E-12	7.85E-11	45.360	1.014	44.765	46.875	67.865	2.004	65.350	69.479
ENSG00000165655	ZNF503	-1.6	1.0E-09	2E-08	44.567	2.657	41.814	47.135	66.814	0.551	66.174	67.521
ENSG00000250337	LINC01021	-1.6	4.5E-04	0.00182	43.418	5.541	35.229	46.856	69.712	9.357	58.891	77.532

ENSG00000232956	SNHG15	-1.6	3.4E-11	9.92E-10	42.701	0.847	41.565	43.507	61.650	1.235	60.587	62.884
ENSG00000198734	F5	-1.6	1.6E-02	0.03785	0.306	0.058	0.249	0.385	0.495	0.056	0.417	0.535
ENSG00000173530	TNFRSF10D	-1.6	7.8E-12	2.80E-10	38.107	1.786	36.046	40.200	55.522	0.841	54.504	56.563
ENSG00000128610	FEZF1	-1.6	1.7E-02	0.03917	0.844	0.237	0.662	1.192	1.172	0.227	0.986	1.503
ENSG00000162772	ATF3	-1.6	3.1E-06	2.4E-05	35.504	2.759	31.675	37.582	51.476	2.729	47.397	53.150
ENSG00000115414	FN1	-1.6	1.7E-10	4.1E-09	31.975	2.550	28.162	33.535	48.593	0.861	48.109	49.883
ENSG00000054598	FOXC1	-1.6	2.2E-10	5.3E-09	30.799	2.467	29.113	34.371	47.052	2.399	44.972	49.130
ENSG00000115274	INO80B	-1.6	1.7E-02	0.03969	1.467	0.407	1.023	1.912	1.936	0.060	1.887	2.009
ENSG00000150687	PRSS23	-1.6	2.8E-12	1.19E-10	24.660	0.897	23.323	25.240	36.402	0.284	36.105	36.788
ENSG00000121621	KIF18A	-1.6	8.8E-03	0.02253	24.400	3.327	20.708	27.694	36.015	3.468	31.093	38.449
ENSG00000102554	KLF5	-1.6	1.3E-10	3.3E-09	23.482	1.164	21.933	24.708	35.003	0.508	34.257	35.326
ENSG00000138182	KIF20B	-1.6	7.5E-02	0.13368	28.277	7.151	20.673	36.105	47.132	13.566	30.151	58.078
ENSG00000115963	RND3	-1.6	7.2E-07	6.6E-06	23.085	0.895	22.157	23.951	33.330	0.968	31.949	34.003
ENSG00000231160	KLF3-AS1	-1.6	2.4E-02	0.05162	0.606	0.344	0.254	0.999	0.956	0.098	0.833	1.073
ENSG00000104081	BMF	-1.6	1.3E-11	4.35E-10	20.234	0.925	18.996	21.033	30.903	1.488	28.694	31.945
ENSG00000144214	LYG1	-1.6	2.5E-02	0.05328	2.883	1.303	1.450	4.204	4.332	0.748	3.240	4.817
ENSG00000163659	TIPARP	-1.6	3.0E-10	6.8E-09	18.313	1.124	17.430	19.895	26.728	0.718	25.672	27.180
ENSG00000196812	ZSCAN16	-1.6	1.3E-06	1.1E-05	17.157	2.017	15.361	19.301	25.203	1.730	24.242	27.797
ENSG00000168743	NPNT	-1.6	2.3E-08	3.1E-07	17.059	1.623	14.884	18.304	26.715	2.578	24.051	28.924
ENSG00000086967	MYBPC2	-1.6	2.1E-02	0.04619	0.575	0.132	0.424	0.744	0.837	0.101	0.695	0.932
ENSG00000101384	JAG1	-1.6	2.6E-10	6.1E-09	16.370	0.753	15.257	16.907	24.306	0.963	22.861	24.789
ENSG00000197168	NEK5	-1.6	1.0E-02	0.02527	0.394	0.124	0.286	0.569	0.586	0.123	0.474	0.692
ENSG00000251095		-1.6	1.3E-06	1.1E-05	15.768	0.893	14.587	16.758	22.492	0.847	21.244	23.139
ENSG00000213315		-1.6	2.0E-04	0.00088	15.427	2.120	13.875	18.557	22.993	1.526	21.898	25.134
ENSG00000116991	SIPA1L2	-1.6	2.0E-10	4.9E-09	13.554	0.887	12.656	14.673	19.985	1.038	18.959	20.879
ENSG00000261934	PCDHGA9	-1.6	1.4E-02	0.03236	0.631	0.224	0.334	0.853	1.116	0.228	0.794	1.277
ENSG00000049130	KITLG	-1.6	1.1E-04	0.00052	13.033	0.069	12.943	13.091	20.446	2.448	17.506	22.463
ENSG00000270605		-1.6	2.3E-07	2.4E-06	12.924	2.226	10.106	14.971	19.675	0.961	18.817	20.506
ENSG00000230847		-1.6	8.2E-05	0.00042	12.780	1.838	11.193	15.366	18.861	1.570	18.045	21.217
ENSG00000183496	MEX3B	-1.6	1.4E-09	2.7E-08	11.831	0.585	10.990	12.347	17.605	0.913	16.306	18.242
ENSG00000125398	SOX9	-1.6	1.4E-08	2E-07	11.735	0.443	11.189	12.183	17.456	0.126	17.285	17.589
ENSG00000145687	SSBP2	-1.6	1.6E-11	5.19E-10	11.129	0.365	10.730	11.496	16.605	0.644	15.677	17.040
ENSG00000266490		-1.6	3.2E-03	0.0096	10.312	2.554	7.957	13.292	15.435	1.250	13.600	16.230
ENSG00000169851	PCDH7	-1.6	2.4E-10	5.6E-09	10.047	0.797	9.359	10.908	15.554	0.882	14.495	16.281
ENSG00000184349	EFNA5	-1.6	1.1E-09	2.1E-08	9.571	0.259	9.239	9.871	14.421	0.455	13.744	14.733
ENSG00000129757	CDKN1C	-1.6	1.1E-07	1.2E-06	9.315	0.618	8.426	9.755	13.591	0.613	13.009	14.457
ENSG00000180530	NRIP1	-1.6	2.4E-05	0.00014	9.178	0.919	7.976	10.164	13.684	1.290	12.408	14.795
ENSG00000267178	PHF5CP	-1.6	7.6E-03	0.01995	9.156	0.891	7.906	9.897	14.760	1.830	12.382	16.199

ENSG00000146592	CREB5	-1.6	1.8E-07	1.9E-06	8.791	0.694	7.786	9.333	13.105	0.289	12.742	13.338
ENSG00000151623	NR3C2	-1.6	3.1E-09	5.3E-08	8.357	0.413	7.949	8.791	12.452	0.722	11.986	13.528
ENSG00000270067		-1.6	6.3E-04	0.00242	8.354	1.642	6.346	9.771	11.851	1.015	10.331	12.401
ENSG00000265254		-1.6	4.4E-03	0.01263	7.923	2.815	3.828	10.108	13.329	2.171	11.427	15.210
ENSG00000158406	HIST1H4H	-1.6	6.5E-06	4.5E-05	7.919	1.297	6.325	9.433	11.767	0.493	11.039	12.130
ENSG00000116106	EPHA4	-1.6	2.6E-08	3.5E-07	7.552	0.830	6.641	8.473	11.240	0.925	10.356	12.038
ENSG00000169184	MN1	-1.6	1.4E-07	1.5E-06	7.221	0.762	6.091	7.765	11.142	0.425	10.545	11.448
ENSG00000165323	FAT3	-1.6	5.7E-06	4E-05	7.006	0.853	5.881	7.921	10.903	0.485	10.419	11.320
ENSG00000106546	AHR	-1.6	2.4E-06	1.9E-05	6.227	0.617	5.634	7.048	9.522	0.815	8.579	10.203
ENSG00000183098	GPC6	-1.6	2.4E-08	3.3E-07	6.187	0.468	5.788	6.814	9.627	1.030	8.281	10.434
ENSG00000272468		-1.6	9.0E-03	0.02302	5.917	1.667	4.134	7.862	8.655	2.060	7.342	11.677
ENSG00000245205	EEF1A1P4	-1.6	3.2E-04	0.00136	5.464	1.091	4.677	7.012	7.892	0.723	6.811	8.330
ENSG00000157445	CACNA2D3	-1.6	9.0E-07	8E-06	5.340	0.710	4.310	5.938	7.887	0.250	7.687	8.203
ENSG00000232368	FTLP2	-1.6	8.9E-03	0.0227	5.135	1.217	3.939	6.799	8.529	0.772	7.469	9.321
ENSG00000270681		-1.6	2.7E-04	0.00115	5.110	0.587	4.239	5.517	7.149	0.934	5.785	7.903
ENSG00000213453	FTH1P3	-1.6	5.7E-03	0.01573	5.049	0.457	4.494	5.608	7.067	1.937	5.527	9.546
ENSG00000278974		-1.6	1.7E-03	0.00564	4.962	0.567	4.421	5.623	7.305	1.358	5.270	8.028
ENSG00000135547	HEY2	-1.6	1.2E-05	8E-05	4.894	0.553	4.415	5.397	7.118	0.440	6.752	7.634
ENSG00000189184	PCDH18	-1.6	1.6E-07	1.8E-06	4.776	0.483	4.402	5.475	7.203	0.418	6.909	7.824
ENSG00000254480		-1.6	7.3E-03	0.01916	4.692	1.062	3.404	6.003	6.374	0.545	5.575	6.800
ENSG00000162599	NFIA	-1.6	6.6E-08	8E-07	4.672	0.307	4.281	4.987	6.888	0.093	6.824	7.022
ENSG00000265962	GACAT2	-1.6	2.4E-03	0.00754	4.595	0.724	3.845	5.330	6.875	1.584	4.801	8.658
ENSG00000150630	VEGFC	-1.6	1.7E-04	0.00078	4.374	0.721	3.481	5.157	6.237	0.373	5.999	6.784
ENSG00000104313	EYA1	-1.6	7.5E-05	0.00038	4.330	0.593	3.924	5.211	6.568	0.770	5.604	7.190
ENSG00000118898	PPL	-1.6	1.8E-08	2.6E-07	4.300	0.385	4.030	4.871	6.271	0.332	5.968	6.743
ENSG00000228451	SDAD1P1	-1.6	2.7E-05	0.00015	4.289	0.320	3.869	4.636	6.435	0.314	6.035	6.685
ENSG00000151012	SLC7A11	-1.6	1.9E-04	0.00087	4.157	0.267	3.815	4.436	6.303	0.375	5.791	6.583
ENSG00000169432	SCN9A	-1.6	7.6E-07	6.9E-06	4.077	0.265	3.687	4.275	6.452	0.597	5.850	6.966
ENSG00000114861	FOXP1	-1.6	1.6E-07	1.7E-06	3.929	0.396	3.691	4.521	5.687	0.155	5.590	5.917
ENSG00000188177	ZC3H6	-1.6	2.7E-07	2.8E-06	3.815	0.295	3.397	4.078	5.734	0.397	5.210	6.042
ENSG00000077092	RARB	-1.6	4.1E-06	3E-05	3.715	0.343	3.483	4.226	5.414	0.456	4.876	5.991
ENSG00000240498	CDKN2B-AS1	-1.6	1.3E-06	1.1E-05	3.510	0.233	3.238	3.796	5.193	0.414	4.800	5.551
ENSG00000134533	RERG	-1.6	5.8E-06	4.1E-05	3.242	0.282	2.911	3.518	4.783	0.618	4.308	5.692
ENSG00000087085	ACHE	-1.6	1.2E-04	0.00057	3.039	0.285	2.867	3.464	4.977	0.984	3.581	5.894
ENSG00000124813	RUNX2	-1.6	3.0E-06	2.3E-05	2.991	0.379	2.757	3.552	4.906	0.381	4.480	5.407
ENSG00000176697	BDNF	-1.6	2.2E-08	3E-07	2.858	0.109	2.722	2.988	4.229	0.195	4.102	4.513
ENSG00000266709		-1.6	9.4E-04	0.00341	2.572	0.342	2.284	3.060	3.760	0.455	3.380	4.287
ENSG00000171724	VAT1L	-1.6	1.8E-02	0.04147	0.590	0.150	0.479	0.798	0.972	0.173	0.778	1.118

ENSG00000179546	HTR1D	-1.6	4.2E-04	0.00171	2.484	0.483	1.997	3.026	3.539	0.385	3.208	3.918
ENSG00000137727	ARHGAP20	-1.6	5.1E-06	3.7E-05	2.399	0.288	2.141	2.709	3.777	0.441	3.253	4.142
ENSG00000231154	MORF4L2-AS1	-1.6	6.9E-04	0.00261	2.327	0.299	2.111	2.754	3.797	0.720	2.855	4.359
ENSG00000230069	LRRC37A15P	-1.6	5.0E-03	0.01393	2.203	0.722	1.356	3.070	3.288	0.471	2.613	3.710
ENSG00000203740	METTL11B	-1.6	4.3E-03	0.01237	2.191	0.579	1.810	3.053	3.208	0.499	2.783	3.743
ENSG00000033122	LRRC7	-1.6	3.5E-07	3.5E-06	2.083	0.226	1.792	2.312	3.157	0.084	3.065	3.270
ENSG00000205323	SARNP	-1.6	1.4E-03	0.00482	2.079	0.499	1.488	2.683	2.826	0.047	2.775	2.866
ENSG00000259495		-1.6	1.2E-05	7.7E-05	2.023	0.303	1.753	2.349	3.059	0.136	2.860	3.146
ENSG00000229619	MBNL1-AS1	-1.6	1.4E-05	8.9E-05	2.012	0.225	1.887	2.349	3.133	0.358	2.767	3.440
ENSG00000198153	ZNF849P	-1.6	3.4E-03	0.01006	1.993	0.371	1.548	2.347	2.793	0.243	2.544	3.127
ENSG00000241170		-1.6	2.2E-03	0.00705	1.864	0.470	1.334	2.440	3.119	0.371	2.732	3.437
ENSG00000256304	CCDC150P1	-1.6	1.3E-03	0.00453	1.743	0.279	1.349	2.005	2.374	0.259	2.233	2.761
ENSG00000257261		-1.6	3.1E-03	0.00929	1.706	0.491	1.049	2.224	2.591	0.334	2.096	2.794
ENSG00000267365	KCNJ2-AS1	-1.6	2.7E-03	0.00845	1.705	0.495	1.020	2.195	2.414	0.077	2.316	2.505
ENSG00000183336	BOLA2	-1.6	2.5E-03	0.00779	1.638	0.392	1.111	2.032	2.450	0.373	2.143	2.993
ENSG00000188993	LRRC66	-1.6	1.5E-03	0.00514	1.581	0.366	1.134	1.888	2.456	0.356	1.936	2.685
ENSG00000165182	CXorf58	-1.6	4.7E-03	0.01338	1.552	0.475	0.980	2.022	2.459	0.286	2.174	2.856
ENSG00000079102	RUNX1T1	-1.6	2.0E-06	1.6E-05	1.512	0.205	1.231	1.675	2.280	0.161	2.105	2.416
ENSG00000246876	LINC02466	-1.6	3.3E-03	0.00979	1.500	0.245	1.268	1.729	2.356	0.633	1.535	3.080
ENSG00000006128	TAC1	-1.6	9.5E-03	0.02403	1.492	0.406	0.944	1.897	2.404	0.190	2.131	2.535
ENSG00000215386	MIR99AHG	-1.6	1.8E-04	0.00082	1.453	0.256	1.172	1.765	1.975	0.164	1.731	2.068
ENSG00000114200	BCHE	-1.6	6.7E-03	0.01787	1.436	0.473	0.900	2.041	2.184	0.410	1.776	2.536
ENSG00000172748	ZNF596	-1.6	7.1E-05	0.00036	1.368	0.185	1.109	1.503	2.079	0.147	1.922	2.277
ENSG00000178127	NDUFV2	-1.6	7.9E-03	0.02053	1.341	0.466	0.731	1.864	2.114	0.448	1.544	2.472
ENSG00000186976	EFCAB6	-1.6	3.6E-06	2.7E-05	1.298	0.150	1.121	1.441	2.071	0.060	1.983	2.107
ENSG00000259315	ACTG1P17	-1.6	6.2E-03	0.01673	1.282	0.137	1.096	1.418	1.940	0.355	1.649	2.457
ENSG00000138669	PRKG2	-1.6	2.7E-03	0.00828	1.190	0.337	0.883	1.665	1.571	0.321	1.310	1.965
ENSG00000141431	ASXL3	-1.6	2.4E-05	0.00014	1.185	0.251	0.962	1.543	1.899	0.165	1.748	2.041
ENSG00000002745	WNT16	-1.6	8.6E-03	0.02206	1.132	0.429	0.798	1.762	1.857	0.114	1.686	1.925
ENSG00000272345		-1.6	7.7E-02	0.13663	2.523	1.676	1.209	4.740	4.219	0.780	3.266	4.856
ENSG00000106536	POU6F2	-1.6	5.7E-05	0.0003	1.095	0.215	0.860	1.305	1.647	0.241	1.439	1.877
ENSG00000223855		-1.6	2.6E-03	0.008	1.028	0.198	0.791	1.267	1.455	0.265	1.274	1.836
ENSG00000156463	SH3RF2	-1.6	9.6E-05	0.00047	1.024	0.160	0.868	1.174	1.607	0.215	1.291	1.743
ENSG00000182308	DCAF4L1	-1.6	4.5E-03	0.0129	0.982	0.447	0.560	1.453	1.535	0.043	1.484	1.571
ENSG00000239389	PCDHA13	-1.6	3.5E-03	0.01026	0.974	0.363	0.764	1.517	1.453	0.203	1.159	1.589
ENSG00000226476	LINC01748	-1.6	2.5E-05	0.00015	0.969	0.148	0.829	1.158	1.436	0.145	1.242	1.547
ENSG00000008300	CELSR3	-1.6	5.8E-05	0.0003	0.964	0.109	0.816	1.071	1.363	0.115	1.265	1.483
ENSG00000166923	GREM1	-1.6	4.5E-05	0.00025	0.888	0.222	0.624	1.139	1.295	0.050	1.226	1.333

ENSG00000265817	FSBP	-1.6	3.9E-03	0.01145	0.886	0.179	0.699	1.126	1.238	0.174	1.074	1.485
ENSG00000259663		-1.6	2.7E-04	0.00117	0.881	0.034	0.831	0.901	1.312	0.234	1.103	1.647
ENSG00000138675	FGF5	-1.6	2.8E-03	0.00865	0.860	0.219	0.627	1.154	1.269	0.177	1.117	1.449
ENSG00000162814	SPATA17	-1.6	2.9E-03	0.00879	0.804	0.092	0.682	0.893	1.253	0.269	0.914	1.469
ENSG00000271474		-1.6	3.9E-03	0.01137	0.781	0.094	0.644	0.850	1.257	0.111	1.132	1.402
ENSG00000269113	TRABD2B	-1.6	1.6E-03	0.00523	0.752	0.099	0.664	0.894	1.173	0.242	0.826	1.338
ENSG00000113532	ST8SIA4	-1.6	4.9E-04	0.00195	0.712	0.138	0.605	0.915	1.068	0.069	0.991	1.126
ENSG00000235100		-1.6	1.0E-02	0.02534	1.847	0.481	1.333	2.360	3.129	0.230	2.852	3.319
ENSG00000260572		-1.6	1.1E-02	0.02765	1.600	0.244	1.453	1.964	2.183	0.062	2.107	2.233
ENSG00000267731		-1.6	1.5E-02	0.03452	0.895	0.271	0.543	1.185	1.625	0.415	1.130	1.968
ENSG00000280426		-1.6	1.5E-02	0.03625	1.662	0.492	1.255	2.278	2.212	0.246	1.884	2.482
ENSG00000255129		-1.6	2.7E-02	0.05723	1.397	0.509	0.821	1.861	2.326	0.319	1.848	2.498
ENSG00000261560		-1.6	2.9E-02	0.06027	2.085	0.681	1.473	2.812	3.298	0.981	1.830	3.842
ENSG00000248774		-1.6	3.6E-02	0.07229	5.280	1.242	3.802	6.338	7.798	1.604	6.331	9.187
ENSG00000112175	BMP5	-1.6	6.4E-03	0.01724	0.647	0.219	0.398	0.902	0.906	0.060	0.855	0.994
ENSG00000141433	ADCYAP1	-1.6	4.5E-03	0.01268	0.631	0.105	0.521	0.737	1.004	0.078	0.888	1.048
ENSG00000139364	TMEM132B	-1.6	1.1E-04	0.00053	0.611	0.103	0.461	0.694	0.951	0.155	0.760	1.077
ENSG00000251664	PCDHA12	-1.6	4.1E-03	0.01186	0.595	0.055	0.548	0.671	0.961	0.118	0.841	1.063
ENSG00000159450	TCHH	-1.6	4.9E-03	0.01368	0.550	0.096	0.427	0.657	0.934	0.009	0.921	0.943
ENSG00000099260	PALMD	-1.6	7.3E-04	0.00276	0.492	0.045	0.446	0.553	0.695	0.108	0.639	0.857
ENSG00000156475	PPP2R2B	-1.6	4.3E-04	0.00175	0.450	0.054	0.377	0.500	0.651	0.058	0.609	0.737
ENSG00000124766	SOX4	-1.7	4.8E-12	1.89E-10	80.790	4.325	75.534	85.457	125.366	4.712	121.005	129.442
ENSG00000245532	NEAT1	-1.7	4.6E-08	5.9E-07	74.286	8.061	63.288	82.604	125.039	15.588	110.923	138.531
ENSG00000277449	CEBPB-AS1	-1.7	1.0E-02	0.02535	0.882	0.326	0.475	1.273	1.351	0.286	1.205	1.780
ENSG00000116717	GADD45A	-1.7	1.1E-10	3E-09	57.125	3.140	53.288	60.422	91.402	3.945	86.930	96.547
ENSG00000251129		-1.7	3.9E-10	8.6E-09	52.291	2.565	50.024	55.925	80.411	3.122	75.972	82.595
ENSG00000170836	PPM1D	-1.7	3.5E-12	1.46E-10	44.981	1.528	42.807	46.339	69.966	2.472	66.568	71.799
ENSG00000164442	CITED2	-1.7	1.5E-10	3.7E-09	43.596	2.387	40.321	46.041	66.056	1.195	65.263	67.790
ENSG00000165244	ZNF367	-1.7	7.9E-12	2.83E-10	41.180	0.878	39.981	42.094	63.914	2.685	60.350	65.986
ENSG00000251022	THAP9-AS1	-1.7	3.5E-08	4.6E-07	30.992	2.483	29.006	34.554	49.855	5.136	44.441	54.244
ENSG00000002587	HS3ST1	-1.7	1.3E-02	0.03153	0.292	0.095	0.218	0.431	0.508	0.124	0.346	0.606
ENSG00000113580	NR3C1	-1.7	3.2E-11	9.52E-10	22.899	1.433	20.779	23.908	35.511	2.421	32.905	37.571
ENSG00000105327	BBC3	-1.7	1.4E-09	2.7E-08	21.802	1.946	19.435	24.191	35.281	0.488	34.977	36.010
ENSG00000128285	MCHR1	-1.7	7.3E-02	0.13	0.285	0.135	0.103	0.401	0.497	0.126	0.326	0.593
ENSG00000181449	SOX2	-1.7	6.0E-10	1.3E-08	18.719	1.378	16.770	20.016	29.536	1.595	28.119	30.918
ENSG00000067082	KLF6	-1.7	7.8E-13	4.08E-11	15.771	0.455	15.138	16.198	24.958	0.621	24.358	25.494
ENSG00000175745	NR2F1	-1.7	2.2E-09	3.9E-08	14.696	1.750	13.344	17.063	23.274	1.040	21.837	24.039
ENSG00000096654	ZNF184	-1.7	1.6E-08	2.2E-07	14.646	1.218	13.261	15.994	23.763	1.829	22.138	25.346

ENSG00000150347	ARID5B	-1.7	3.5E-10	7.8E-09	14.051	0.513	13.290	14.388	22.090	0.466	21.665	22.493
ENSG00000099250	NRP1	-1.7	1.1E-11	3.77E-10	13.178	0.483	12.550	13.719	21.682	1.406	20.243	22.888
ENSG00000168916	ZNF608	-1.7	2.8E-09	4.7E-08	12.589	1.296	11.018	13.736	20.287	1.169	18.882	21.250
ENSG00000115461	IGFBP5	-1.7	2.8E-10	6.4E-09	11.682	1.169	10.033	12.792	18.532	0.628	18.065	19.458
ENSG00000150551	LYPD1	-1.7	1.6E-09	2.9E-08	11.668	0.553	10.846	12.002	18.389	0.994	17.528	19.286
ENSG00000185630	PBX1	-1.7	1.9E-11	5.84E-10	11.051	0.748	9.953	11.609	17.534	1.021	16.455	18.406
ENSG00000246465		-1.7	4.3E-08	5.5E-07	9.237	0.412	8.828	9.603	14.371	1.025	13.296	15.766
ENSG00000164949	GEM	-1.7	1.5E-07	1.7E-06	9.140	0.710	8.154	9.798	13.755	0.525	13.130	14.415
ENSG00000120370	GORAB	-1.7	1.3E-08	1.8E-07	8.132	0.361	7.794	8.610	12.897	0.307	12.532	13.150
ENSG00000249859	PVT1	-1.7	1.8E-09	3.2E-08	7.760	0.337	7.399	8.114	12.792	0.489	12.073	13.099
ENSG00000044524	EPHA3	-1.7	1.8E-07	1.9E-06	7.685	0.794	6.691	8.629	11.856	0.323	11.462	12.120
ENSG00000176887	SOX11	-1.7	2.3E-09	4E-08	7.310	0.723	6.312	7.958	11.318	0.445	10.652	11.566
ENSG00000179981	TSHZ1	-1.7	1.5E-09	2.9E-08	6.988	0.796	6.124	7.785	11.199	0.078	11.135	11.313
ENSG00000261557		-1.7	2.1E-04	0.00092	5.368	1.099	3.761	6.247	8.154	1.353	7.053	10.129
ENSG00000277383		-1.7	1.9E-03	0.00616	5.315	1.083	4.425	6.689	8.415	0.413	8.021	8.772
ENSG00000239405	TMED10P2	-1.7	1.8E-03	0.00595	4.772	1.198	3.597	5.852	7.551	0.586	6.673	7.890
ENSG00000164708	PGAM2	-1.7	1.1E-03	0.00378	4.034	0.805	3.467	5.183	6.038	0.743	5.399	6.782
ENSG00000164743	C8orf48	-1.7	5.7E-05	0.0003	3.994	0.636	3.286	4.742	6.369	0.565	5.544	6.735
ENSG00000145536	ADAMTS16	-1.7	1.3E-07	1.5E-06	3.861	0.330	3.487	4.291	6.448	0.528	6.095	7.234
ENSG00000266983		-1.7	4.1E-03	0.01181	3.703	1.281	2.707	5.580	6.794	1.382	5.542	7.989
ENSG00000070731	ST6GALNAC2	-1.7	4.8E-06	3.5E-05	3.366	0.597	2.571	4.010	5.640	0.444	4.988	5.922
ENSG00000143995	MEIS1	-1.7	2.0E-08	2.8E-07	3.230	0.301	2.935	3.589	5.140	0.484	4.446	5.472
ENSG00000225792		-1.7	2.0E-03	0.00643	3.164	0.763	2.496	4.237	4.854	0.450	4.522	5.475
ENSG00000258357		-1.7	5.7E-03	0.01558	2.916	0.281	2.695	3.326	4.007	1.230	2.966	5.365
ENSG00000203814	HIST2H2BF	-1.7	9.8E-05	0.00048	2.451	0.524	1.820	2.964	4.127	0.244	3.794	4.309
ENSG00000230699		-1.7	1.9E-03	0.00618	2.425	0.677	1.433	2.946	4.268	1.192	3.097	5.295
ENSG00000260103		-1.7	1.3E-04	0.00061	2.401	0.558	1.819	3.160	3.670	0.718	3.064	4.470
ENSG00000217648		-1.7	2.3E-03	0.00737	2.380	0.659	1.642	3.163	3.782	0.639	3.435	4.738
ENSG00000140527	WDR93	-1.7	1.8E-02	0.0416	0.481	0.186	0.345	0.753	0.812	0.111	0.673	0.902
ENSG00000196890	HIST3H2BB	-1.7	2.5E-05	0.00015	2.316	0.201	2.095	2.528	3.797	0.115	3.677	3.954
ENSG00000232906		-1.7	2.1E-03	0.00681	2.242	0.761	1.629	3.347	3.743	0.663	3.108	4.676
ENSG00000229127		-1.7	2.4E-03	0.00747	2.242	0.457	1.621	2.691	3.911	1.129	2.635	4.861
ENSG00000138670	RASGEF1B	-1.7	1.9E-08	2.6E-07	2.240	0.065	2.146	2.295	3.433	0.247	3.130	3.635
ENSG00000107562	CXCL12	-1.7	5.4E-08	6.7E-07	2.229	0.171	1.988	2.369	3.656	0.137	3.544	3.826
ENSG00000279865		-1.7	2.3E-04	0.00099	2.223	0.441	1.834	2.851	3.425	0.148	3.306	3.612
ENSG00000263745		-1.7	1.2E-05	7.9E-05	2.215	0.232	1.946	2.419	3.525	0.425	2.895	3.781
ENSG00000225420		-1.7	4.3E-03	0.01221	2.157	0.660	1.604	2.948	3.399	0.630	3.014	4.330
ENSG00000169554	ZEB2	-1.7	1.6E-08	2.3E-07	1.877	0.117	1.757	2.023	3.053	0.268	2.806	3.284

ENSG00000123700	KCNJ2	-1.7	6.1E-06	4.3E-05	1.587	0.120	1.410	1.669	2.590	0.342	2.078	2.775
ENSG00000261136		-1.7	2.8E-04	0.00118	1.531	0.384	1.032	1.950	2.561	0.103	2.430	2.681
ENSG00000118407	FILIP1	-1.7	3.3E-06	2.5E-05	1.386	0.133	1.254	1.512	2.052	0.043	2.024	2.115
ENSG00000139209	SLC38A4	-1.7	2.0E-04	0.00089	1.240	0.127	1.099	1.404	2.114	0.275	1.819	2.348
ENSG00000186056	MATN1-AS1	-1.7	2.7E-04	0.00117	1.192	0.195	0.985	1.432	1.989	0.358	1.679	2.300
ENSG00000108932	SLC16A6	-1.7	1.7E-04	0.0008	1.174	0.240	0.833	1.390	1.784	0.086	1.665	1.846
ENSG00000234996		-1.7	4.8E-03	0.01354	1.145	0.392	0.604	1.491	2.025	0.440	1.624	2.406
ENSG00000143110	C1orf162	-1.7	1.4E-03	0.00491	1.126	0.265	0.885	1.360	1.810	0.265	1.512	2.156
ENSG00000154736	ADAMTS5	-1.7	1.1E-06	9.5E-06	1.019	0.060	0.955	1.099	1.601	0.090	1.509	1.724
ENSG00000102174	PHEX	-1.7	7.2E-06	4.9E-05	1.011	0.108	0.888	1.148	1.616	0.100	1.501	1.699
ENSG00000154479	CCDC173	-1.7	6.4E-03	0.01716	0.989	0.340	0.563	1.393	1.459	0.220	1.224	1.756
ENSG00000156453	PCDH1	-1.7	4.8E-04	0.00189	0.935	0.169	0.826	1.187	1.403	0.136	1.297	1.582
ENSG00000115896	PLCL1	-1.7	1.1E-04	0.00055	0.928	0.115	0.805	1.070	1.442	0.277	1.117	1.672
ENSG00000230873	STMND1	-1.7	4.0E-03	0.01163	0.923	0.304	0.709	1.375	1.408	0.232	1.218	1.690
ENSG00000170961	HAS2	-1.7	1.1E-03	0.00403	0.786	0.207	0.595	1.060	1.206	0.205	1.066	1.500
ENSG00000087589	CASS4	-1.7	1.6E-03	0.00525	0.742	0.235	0.434	0.983	1.202	0.152	1.070	1.334
ENSG00000249637		-1.7	1.5E-02	0.03563	3.485	0.755	2.790	4.255	5.154	1.537	4.068	7.330
ENSG00000214198	TTC41P	-1.7	3.5E-04	0.00146	0.658	0.035	0.613	0.695	0.955	0.204	0.835	1.258
ENSG00000253305	PCDHGB6	-1.7	5.5E-04	0.00215	0.636	0.137	0.505	0.754	1.161	0.237	0.866	1.353
ENSG00000284526		-1.7	3.9E-03	0.01124	0.545	0.142	0.389	0.701	0.916	0.057	0.861	0.996
ENSG00000136040	PLXNC1	-1.7	5.1E-03	0.01426	0.535	0.077	0.429	0.601	0.903	0.118	0.763	1.000
ENSG00000113389	NPR3	-1.7	2.6E-03	0.00823	0.507	0.040	0.466	0.562	0.733	0.223	0.604	1.067
ENSG00000248441	LINC01197	-1.7	1.3E-03	0.00445	0.466	0.080	0.397	0.579	0.695	0.187	0.570	0.967
ENSG00000164309	CMYA5	-1.7	4.8E-04	0.00191	0.421	0.094	0.335	0.545	0.663	0.025	0.641	0.687
ENSG00000169896	ITGAM	-1.7	5.3E-03	0.01474	0.390	0.091	0.302	0.490	0.573	0.198	0.422	0.838
ENSG00000134207	SYT6	-1.7	6.2E-03	0.01679	0.291	0.062	0.240	0.370	0.452	0.089	0.375	0.536
ENSG00000133169	BEX1	-1.8	3.2E-02	0.06562	4.760	4.002	1.820	10.661	7.382	0.596	6.893	8.104
ENSG00000138778	CENPE	-1.8	8.0E-02	0.14064	12.317	4.921	8.096	17.467	24.546	10.483	11.527	33.043
ENSG00000236824	BCYRN1	-1.8	1.0E-04	0.0005	55.441	10.695	44.901	68.215	92.569	20.031	63.806	106.253
ENSG00000228314	CYP4F29P	-1.8	1.1E-02	0.02779	1.166	0.504	0.773	1.884	2.088	0.202	1.967	2.391
ENSG00000155090	KLF10	-1.8	1.2E-14	1.19E-12	47.086	1.911	44.900	49.534	79.005	0.317	78.733	79.463
ENSG00000140450	ARRDC4	-1.8	9.5E-12	3.29E-10	40.418	2.873	36.609	42.881	67.499	5.755	62.416	72.482
ENSG00000158373	HIST1H2BD	-1.8	1.2E-08	1.7E-07	22.507	2.464	18.978	24.465	37.180	2.754	33.364	39.198
ENSG00000180573	HIST1H2AC	-1.8	3.5E-10	7.8E-09	20.719	0.761	19.833	21.671	34.083	0.698	33.100	34.750
ENSG00000169116	PARM1	-1.8	2.6E-12	1.12E-10	17.911	1.354	16.275	19.241	30.164	1.155	29.411	31.847
ENSG00000168542	COL3A1	-1.8	1.6E-11	5.10E-10	9.920	0.447	9.651	10.586	16.231	0.762	15.647	17.251
ENSG00000278238		-1.8	6.5E-05	0.00034	8.095	1.717	6.401	10.446	13.647	0.936	12.715	14.947
ENSG00000218069	RSL24D1P1	-1.8	1.3E-04	0.00063	6.459	0.953	5.748	7.815	10.736	0.979	10.032	12.108

ENSG00000260077		-1.8	1.5E-05	9.2E-05	6.234	0.267	5.944	6.553	10.712	1.122	9.743	11.791
ENSG00000137573	SULF1	-1.8	2.8E-10	6.5E-09	5.862	0.272	5.552	6.160	10.015	0.154	9.788	10.111
ENSG00000214243		-1.8	5.9E-04	0.00228	4.695	0.554	4.009	5.273	7.600	0.529	6.849	7.971
ENSG00000092421	SEMA6A	-1.8	2.4E-09	4.2E-08	4.444	0.549	3.646	4.827	7.440	0.221	7.325	7.772
ENSG00000135116	HRK	-1.8	1.6E-08	2.3E-07	4.107	0.254	3.844	4.454	7.062	0.590	6.177	7.358
ENSG00000234585	CCT6P3	-1.8	8.5E-09	1.3E-07	3.896	0.318	3.563	4.192	6.629	0.249	6.264	6.790
ENSG00000128573	FOXP2	-1.8	2.1E-05	0.00012	3.361	0.314	2.891	3.537	5.641	0.128	5.495	5.749
ENSG00000140465	CYP1A1	-1.8	1.6E-04	0.00073	2.342	0.329	1.995	2.675	3.482	0.965	2.926	4.920
ENSG00000176046	NUPR1	-1.8	1.6E-07	1.8E-06	2.327	0.203	2.051	2.510	3.738	0.504	3.346	4.402
ENSG00000237187	NR2F1-AS1	-1.8	3.5E-08	4.7E-07	2.265	0.321	1.813	2.500	3.683	0.097	3.561	3.761
ENSG00000138639	ARHGAP24	-1.8	3.8E-07	3.8E-06	2.203	0.355	1.674	2.426	3.674	0.210	3.420	3.933
ENSG00000178573	MAF	-1.8	9.1E-08	1.1E-06	1.984	0.243	1.623	2.130	3.162	0.052	3.092	3.202
ENSG00000274290	HIST1H2BE	-1.8	9.5E-04	0.00345	1.715	0.544	0.920	2.139	2.543	0.341	2.345	3.051
ENSG00000102678	FGF9	-1.8	2.0E-05	0.00012	1.687	0.474	1.143	2.230	3.027	0.210	2.769	3.199
ENSG00000152763	WDR78	-1.8	8.7E-05	0.00043	1.547	0.334	1.196	1.974	2.512	0.524	2.070	3.097
ENSG00000243819	RN7SL832P	-1.8	4.7E-04	0.00188	1.476	0.286	1.075	1.745	2.517	0.034	2.475	2.544
ENSG00000109819	PPARGC1A	-1.8	7.1E-08	8.6E-07	1.372	0.096	1.232	1.451	2.343	0.156	2.162	2.543
ENSG00000260774		-1.8	6.2E-04	0.00238	1.287	0.151	1.116	1.478	1.912	0.381	1.341	2.104
ENSG00000166793	YPEL4	-1.8	1.3E-05	8.3E-05	1.265	0.145	1.133	1.432	2.018	0.178	1.896	2.274
ENSG00000182752	PAPPA	-1.8	3.7E-07	3.6E-06	1.219	0.146	1.095	1.429	1.999	0.224	1.820	2.281
ENSG00000273038		-1.8	7.0E-03	0.01867	1.005	0.352	0.681	1.452	1.954	0.214	1.643	2.094
ENSG00000247809	NR2F2-AS1	-1.8	3.0E-05	0.00017	0.836	0.094	0.700	0.909	1.268	0.099	1.184	1.381
ENSG00000186479	RGS7BP	-1.8	2.7E-04	0.00117	0.808	0.128	0.711	0.995	1.365	0.218	1.040	1.507
ENSG00000121075	TBX4	-1.8	5.4E-04	0.00212	0.727	0.104	0.662	0.882	1.225	0.071	1.128	1.297
ENSG00000107105	ELAVL2	-1.8	1.2E-03	0.00425	0.701	0.254	0.536	1.079	1.177	0.179	0.908	1.273
ENSG00000269974		-1.8	1.3E-02	0.03153	3.998	1.049	2.925	5.311	6.511	1.477	5.441	8.572
ENSG00000185736	ADARB2	-1.8	1.3E-04	0.00062	0.556	0.109	0.481	0.718	1.016	0.192	0.747	1.153
ENSG00000231672	DIRC3	-1.8	1.1E-03	0.00381	0.496	0.121	0.361	0.634	0.818	0.187	0.587	1.044
ENSG00000215018	COL28A1	-1.8	3.2E-04	0.00135	0.492	0.079	0.423	0.579	0.782	0.152	0.651	0.926
ENSG00000150394	CDH8	-1.8	3.0E-05	0.00017	0.434	0.040	0.395	0.487	0.742	0.124	0.627	0.849
ENSG00000175197	DDIT3	-1.9	2.9E-10	6.7E-09	39.272	2.848	35.276	41.777	67.965	4.580	65.225	74.818
ENSG00000145632	PLK2	-1.9	2.7E-12	1.16E-10	17.036	0.919	16.494	18.407	30.219	1.628	28.366	31.587
ENSG00000228409	CCT6P1	-1.9	5.5E-08	6.8E-07	13.533	1.717	11.346	15.539	24.681	1.192	24.010	26.462
ENSG00000078018	MAP2	-1.9	2.7E-09	4.7E-08	7.288	0.606	6.802	8.174	13.503	1.531	12.013	14.824
ENSG00000260727	SLC7A5P1	-1.9	1.8E-03	0.0058	4.243	1.205	3.075	5.847	7.775	1.808	5.096	9.068
ENSG00000250519		-1.9	4.2E-04	0.00171	3.414	0.440	2.827	3.877	6.125	0.780	5.121	7.024
ENSG00000163009	C2orf48	-1.9	1.2E-05	7.7E-05	2.949	0.740	2.134	3.876	5.305	0.666	4.837	6.293
ENSG00000274460		-1.9	9.2E-05	0.00046	2.742	0.721	1.849	3.586	4.698	0.417	4.387	5.268

ENSG00000276966	HIST1H4E	-1.9	3.1E-05	0.00018	2.662	0.548	2.221	3.456	4.849	0.367	4.331	5.196
ENSG00000224596	ZMIZ1-AS1	-1.9	2.3E-02	0.04957	0.274	0.114	0.173	0.391	0.568	0.143	0.355	0.649
ENSG00000225793		-1.9	2.4E-04	0.00103	1.702	0.120	1.553	1.845	2.860	0.661	2.416	3.815
ENSG00000164440	TXLNB	-1.9	2.7E-06	2.1E-05	1.670	0.391	1.334	2.082	2.894	0.240	2.706	3.245
ENSG00000104783	KCNN4	-1.9	7.8E-06	5.3E-05	1.255	0.182	1.118	1.515	2.264	0.125	2.183	2.447
ENSG00000261087		-1.9	1.3E-03	0.00455	1.124	0.478	0.542	1.683	2.057	0.194	1.766	2.169
ENSG00000188959	C9orf152	-1.9	7.9E-04	0.00295	0.658	0.140	0.471	0.780	1.054	0.092	0.974	1.147
ENSG00000233030		-1.9	3.0E-03	0.00903	0.520	0.265	0.266	0.894	0.998	0.184	0.810	1.156
ENSG00000248596		-1.9	1.9E-03	0.00628	0.424	0.176	0.259	0.659	0.719	0.043	0.683	0.765
ENSG00000173406	DAB1	-1.9	1.1E-03	0.00402	0.298	0.089	0.187	0.371	0.496	0.125	0.423	0.682
ENSG00000174844	DNAH12	-1.9	2.5E-04	0.00107	0.209	0.045	0.152	0.249	0.360	0.044	0.337	0.426
ENSG00000026025	VIM	-2	3.5E-15	4.10E-13	183.403	5.370	178.925	191.207	340.579	7.338	330.942	346.298
ENSG00000256589	ENPP7P5	-2	1.0E-01	0.17299	0.274	0.134	0.153	0.453	0.657	0.083	0.609	0.781
ENSG00000188738	FSIP2	-2	2.1E-02	0.04644	0.114	0.048	0.046	0.156	0.272	0.117	0.140	0.370
ENSG00000075223	SEMA3C	-2	1.4E-08	2.1E-07	13.655	1.164	12.610	14.906	26.051	1.381	24.473	27.209
ENSG00000130222	GADD45G	-2	1.3E-06	1.1E-05	7.112	0.740	6.361	7.763	13.920	0.753	13.189	14.976
ENSG00000123095	BHLHE41	-2	4.0E-11	1.2E-09	6.177	0.388	5.616	6.469	11.284	0.357	10.764	11.517
ENSG00000010030	ETV7	-2	9.1E-07	8.1E-06	4.535	0.980	3.161	5.424	8.343	0.975	6.881	8.882
ENSG00000159208	CIART	-2	3.0E-07	3.1E-06	3.284	0.469	2.625	3.693	5.806	0.827	5.148	6.866
ENSG00000213363	RPS3P6	-2	1.2E-03	0.00407	3.097	0.484	2.619	3.769	5.995	0.842	4.744	6.577
ENSG00000170214	ADRA1B	-2	1.9E-04	0.00085	2.581	0.753	1.810	3.261	4.749	0.841	3.615	5.649
ENSG00000179046	TRIML2	-2	6.8E-06	4.7E-05	1.939	0.432	1.340	2.298	3.671	0.786	3.138	4.803
ENSG00000255471		-2	7.6E-04	0.00284	1.648	0.752	1.162	2.765	2.842	0.243	2.511	3.095
ENSG00000283235		-2	1.5E-03	0.00508	1.098	0.625	0.452	1.789	2.102	0.156	1.870	2.214
ENSG00000139269	INHBE	-2	6.0E-04	0.00232	1.026	0.219	0.854	1.343	1.598	0.158	1.482	1.815
ENSG00000108309	RUNDC3A	-2	7.7E-05	0.00039	0.794	0.285	0.593	1.212	1.455	0.321	1.281	1.936
ENSG00000249790		-2	7.2E-03	0.01906	0.478	0.118	0.319	0.583	0.960	0.355	0.598	1.448
ENSG00000273419		-2	3.5E-03	0.01028	0.464	0.104	0.339	0.561	0.844	0.333	0.663	1.343
ENSG00000038295	TLL1	-2	1.3E-03	0.00455	0.310	0.026	0.285	0.334	0.662	0.176	0.449	0.806
ENSG00000272398	CD24	-2.1	4.8E-02	0.09193	2.385	1.846	1.360	5.149	4.201	2.674	2.850	8.212
ENSG00000099251	HSD17B7P2	-2.1	1.1E-09	2.1E-08	12.832	1.553	11.168	14.797	25.578	2.251	22.530	27.279
ENSG00000273802	HIST1H2BG	-2.1	1.2E-07	1.4E-06	4.204	0.484	3.745	4.873	8.276	0.556	7.660	9.011
ENSG00000223638	RFPL4A	-2.1	1.8E-05	0.00011	3.489	0.605	2.899	4.195	6.796	1.466	5.742	8.967
ENSG00000236993	GAPDHP21	-2.1	1.8E-05	0.00011	2.934	0.564	2.467	3.640	5.833	0.260	5.687	6.221
ENSG00000224294		-2.1	7.4E-06	5E-05	2.273	0.302	1.957	2.681	4.772	0.536	4.190	5.227
ENSG00000229292	RFPL4AL1	-2.1	2.2E-04	0.00095	2.259	0.395	1.778	2.736	4.547	0.515	4.117	5.140
ENSG00000242689	CNTF	-2.1	9.1E-06	6.1E-05	2.240	0.275	1.987	2.631	4.493	0.840	3.824	5.723
ENSG00000151023	ENKUR	-2.1	2.2E-03	0.0069	0.373	0.146	0.188	0.535	0.664	0.074	0.553	0.705

ENSG00000080573	COL5A3	-2.1	3.3E-04	0.00138	0.335	0.119	0.235	0.508	0.707	0.149	0.548	0.908
ENSG00000165186	PTCHD1	-2.1	8.0E-05	0.0004	0.173	0.027	0.145	0.206	0.366	0.026	0.329	0.389
ENSG00000210151	MT-TS1	-2.2	3.9E-03	0.01129	61.773	9.410	54.546	75.605	140.259	11.803	131.400	156.330
ENSG00000196787	HIST1H2AG	-2.2	1.7E-09	3E-08	4.897	0.497	4.232	5.393	10.018	0.371	9.495	10.281
ENSG00000108924	HLF	-2.2	2.3E-08	3.2E-07	1.551	0.121	1.380	1.643	3.124	0.454	2.731	3.535
ENSG00000186517	ARHGAP30	-2.2	4.3E-08	5.6E-07	1.525	0.301	1.087	1.724	3.154	0.121	3.009	3.306
ENSG00000188580	NKAIN2	-2.2	1.5E-04	0.00068	0.624	0.237	0.408	0.944	1.130	0.240	0.950	1.457
ENSG00000122641	INHBA	-2.2	2.4E-04	0.00104	0.290	0.095	0.167	0.369	0.606	0.181	0.359	0.795
ENSG00000100867	DHRS2	-2.3	5.7E-10	1.2E-08	9.467	0.470	9.000	9.936	20.462	0.474	19.874	20.847
ENSG00000134363	FST	-2.3	3.1E-06	2.4E-05	1.606	0.437	1.201	2.167	3.594	0.119	3.416	3.654
ENSG00000181016	LSMEM1	-2.3	1.1E-06	9.8E-06	1.466	0.289	1.182	1.802	3.286	0.395	2.732	3.570
ENSG00000138347	MYPN	-2.3	6.6E-07	6.1E-06	0.567	0.149	0.434	0.780	1.320	0.098	1.201	1.401
ENSG00000145087	STXBP5L	-2.3	2.1E-05	0.00012	0.341	0.132	0.249	0.536	0.690	0.142	0.604	0.900
ENSG00000158486	DNAH3	-2.4	2.3E-07	2.4E-06	0.428	0.035	0.396	0.477	1.008	0.038	0.968	1.061
ENSG00000265972	TXNIP	-2.5	1.1E-16	2.23E-14	79.628	4.790	74.361	84.802	181.912	3.734	176.321	184.098
ENSG00000164746	C7orf57	-2.5	2.2E-04	0.00097	0.703	0.166	0.479	0.875	1.381	0.635	0.842	2.071
ENSG00000056487	PHF21B	-2.5	1.1E-04	0.00054	0.437	0.198	0.271	0.723	0.954	0.367	0.638	1.311
ENSG00000143469	SYT14	-2.5	3.8E-09	6.3E-08	0.412	0.065	0.339	0.498	0.978	0.115	0.857	1.077
ENSG00000187775	DNAH17	-2.6	5.2E-13	2.84E-11	1.652	0.179	1.502	1.905	4.031	0.242	3.823	4.267
ENSG00000111087	GLI1	-2.6	2.9E-09	5.1E-08	1.225	0.069	1.131	1.296	2.992	0.283	2.770	3.363
ENSG00000255389		-2.6	1.3E-05	8.1E-05	0.807	0.401	0.515	1.392	2.106	0.316	1.752	2.372
ENSG00000164484	TMEM200A	-2.6	6.7E-04	0.00256	0.167	0.022	0.152	0.200	0.377	0.199	0.234	0.656
ENSG00000234424		-3.1	1.7E-07	1.9E-06	1.365	0.253	1.016	1.601	3.988	0.570	3.173	4.383
ENSG00000132639	SNAP25	-3.1	2.4E-04	0.00105	1.159	0.961	0.593	2.596	3.131	0.323	2.851	3.416
ENSG00000268756		-3.2	1.2E-08	1.8E-07	5.762	0.389	5.412	6.300	17.527	2.380	16.067	21.045
ENSG00000277862	PRAMEF34P	-3.2	1.9E-10	4.7E-09	3.674	0.448	3.217	4.128	10.430	1.865	8.820	12.231
ENSG00000229978	PRAMEF36P	-3.2	3.2E-10	7.2E-09	3.620	0.635	2.933	4.290	10.308	1.690	8.845	11.846
ENSG00000167995	BEST1	-3.2	3.4E-11	1E-09	0.635	0.098	0.511	0.750	1.941	0.162	1.697	2.028
ENSG00000126368	NR1D1	-3.4	1.1E-13	7.24E-12	8.252	0.328	7.789	8.565	26.913	3.452	24.345	31.647
ENSG00000113369	ARRDC3	-3.5	1.7E-16	3.40E-14	8.254	0.579	7.854	9.114	26.063	0.710	25.022	26.514
ENSG00000144010	TRIM43B	-3.5	3.5E-12	1.46E-10	6.776	1.183	5.882	8.511	21.859	2.419	20.404	25.446
ENSG00000144015	TRIM43	-3.6	1.2E-12	6.09E-11	7.297	1.071	5.925	8.382	23.274	2.683	21.373	27.064
ENSG00000204510	PRAMEF7	-4	2.5E-08	3.5E-07	0.856	0.144	0.704	1.035	2.864	0.628	2.355	3.643
ENSG00000249620		-4.4	3.8E-09	6.4E-08	8.026	2.114	5.659	10.246	33.501	8.153	27.310	44.493
ENSG00000183571	PGPEP1L	-4.4	6.0E-04	0.00232	0.150	0.050	0.094	0.193	0.588	0.421	0.294	1.186
ENSG00000250386		-4.5	5.1E-09	8.2E-08	15.004	3.352	10.765	18.646	59.215	18.192	44.468	81.805
ENSG00000182330	PRAMEF8	-4.6	7.2E-08	8.7E-07	0.664	0.138	0.523	0.852	2.533	0.800	2.100	3.730
ENSG00000268799		-4.7	1.5E-09	2.8E-08	11.016	0.790	10.235	12.026	48.173	13.156	36.991	62.445

ENSG00000116726	PRAMEF12	-4.8	1.3E-09	2.5E-08	0.939	0.118	0.872	1.115	3.898	0.647	3.394	4.749
ENSG00000237693	IRGM	-4.8	7.2E-07	6.6E-06	0.413	0.186	0.270	0.686	2.015	0.210	1.701	2.144
ENSG00000144188	TRIM43CP	-4.9	2.3E-12	1.02E-10	2.346	0.146	2.152	2.499	10.501	0.691	9.906	11.181
ENSG00000283740		-5	4.7E-14	3.63E-12	23.906	0.540	23.236	24.356	106.075	18.013	90.894	126.328
ENSG00000171951	SCG2	-5.1	4.3E-08	5.6E-07	0.499	0.055	0.451	0.573	2.402	0.142	2.250	2.523
ENSG00000269466		-5.2	8.8E-12	3.10E-10	45.028	9.711	34.710	57.162	218.205	24.844	197.802	248.265
ENSG00000204531	POU5F1	-5.2	1.1E-01	0.1814	0.132	0.076	0.027	0.205	0.701	1.113	0.000	2.362
ENSG00000284438		-5.2	3.0E-10	6.8E-09	3.211	0.927	2.440	4.459	15.264	1.291	14.146	16.398
ENSG00000189325	C6orf222	-5.2	4.4E-08	5.7E-07	0.259	0.133	0.123	0.375	1.310	0.138	1.196	1.472
ENSG00000168930	TRIM49	-5.3	3.6E-14	2.89E-12	4.679	0.967	3.462	5.613	22.602	3.013	20.010	25.625
ENSG00000204449	TRIM49C	-5.3	7.2E-15	7.52E-13	2.854	0.128	2.692	3.002	13.557	1.737	12.121	15.625
ENSG00000150244	TRIM48	-5.4	1.6E-10	3.9E-09	1.434	0.305	1.185	1.827	6.710	1.426	5.612	8.606
ENSG00000250782		-5.7	3.6E-14	2.9E-12	13.526	2.439	9.962	15.471	69.314	12.385	58.686	81.978
ENSG00000283776		-5.7	1.8E-13	1.1E-11	10.874	2.888	8.237	14.978	55.437	9.323	48.525	68.250
ENSG00000180532	ZSCAN4	-5.7	9.3E-12	3.3E-10	2.984	0.545	2.587	3.787	14.940	2.600	12.698	17.463
ENSG00000284306		-5.7	6.2E-09	9.8E-08	1.802	0.717	0.980	2.728	9.227	2.293	7.342	11.994
ENSG00000284546		-5.7	9.9E-08	1.2E-06	1.137	0.390	0.804	1.688	6.185	1.287	5.214	7.930
ENSG00000235268	KDM4E	-5.7	9.0E-12	3.2E-10	0.845	0.195	0.605	1.059	4.543	0.309	4.095	4.802
ENSG00000204455	TRIM51BP	-6.1	5.5E-12	2.1E-10	2.234	0.553	1.749	2.930	12.098	2.392	10.301	15.353
ENSG00000157765	SLC34A2	-6.1	4.6E-11	1.3E-09	0.489	0.148	0.335	0.632	2.582	0.551	2.139	3.276
ENSG00000255855	KDM4F	-6.2	2.5E-10	5.9E-09	0.850	0.325	0.574	1.292	4.648	0.841	4.078	5.859
ENSG00000236175	SSU72P6	-6.3	5.0E-10	1.1E-08	1.742	0.309	1.476	2.135	10.032	0.459	9.670	10.626
ENSG00000283873		-6.5	7.1E-11	1.9E-09	2.276	0.517	1.645	2.757	13.044	1.494	11.760	14.556
ENSG00000237706	TRIM51EP	-6.5	6.7E-12	2.4E-10	1.845	0.540	1.331	2.526	10.516	2.211	8.919	13.608
ENSG00000236941		-6.8	1.3E-09	2.5E-08	0.929	0.459	0.573	1.602	5.833	1.162	5.162	7.565
ENSG00000249156		-6.9	1.1E-11	3.7E-10	8.935	0.735	8.384	9.973	56.141	10.221	47.843	68.797
ENSG00000254764	TRIM53CP	-6.9	4.6E-10	9.9E-09	0.965	0.298	0.731	1.395	6.115	1.497	4.438	8.083
ENSG00000284018		-7	1.3E-08	1.9E-07	1.326	0.394	0.948	1.868	8.278	2.652	6.000	10.972
ENSG00000182053	TRIM49B	-7	3.1E-11	9.3E-10	0.932	0.229	0.630	1.150	5.631	1.486	4.577	7.729
ENSG00000225581	TRIM53AP	-7.2	1.9E-11	5.8E-10	1.418	0.241	1.069	1.600	8.813	2.795	6.613	12.440
ENSG00000223417	TRIM49D1	-7.3	6.2E-15	6.6E-13	1.726	0.242	1.538	2.060	11.203	1.769	9.744	13.326
ENSG00000233802	TRIM49D2	-7.4	5.0E-15	5.4E-13	2.227	0.197	2.016	2.473	14.759	2.347	12.844	17.639
ENSG00000166013	TRIM53BP	-7.7	8.3E-12	2.9E-10	0.993	0.259	0.709	1.328	6.755	1.583	5.589	8.940
ENSG00000251258	RFPL4B	-8.3	4.9E-09	8.0E-08	0.559	0.421	0.083	0.938	4.084	0.765	3.443	4.960
ENSG00000189253	TRIM64B	-8.5	5.6E-12	2.1E-10	0.576	0.208	0.427	0.869	4.465	0.582	4.020	5.243
ENSG00000257951		-8.7	3.3E-12	1.4E-10	2.347	0.911	1.389	3.534	18.124	4.011	15.793	24.130
ENSG00000183813	CCR4	-8.8	3.0E-08	4.1E-07	0.120	0.025	0.099	0.151	1.017	0.035	0.964	1.040
ENSG00000204450	TRIM64	-9.8	1.5E-08	2.1E-07	0.179	0.105	0.061	0.311	1.670	0.563	1.201	2.325

ENSG00000256779	-9.9	1.5E-10	3.8E-09	0.484	0.207	0.356	0.792	4.667	0.605	3.902	5.382

downregulated 759	downregulated 620	downregulated in 759 & 620	All C19MC targets ensem	C19MC targets & downregulated in both expts
ENSG00000238083	ENSG00000002587	ENSG00000002587	ENSG00000000003	ENSG00000002587
ENSG00000186479	ENSG00000002745	ENSG00000002745	ENSG00000000938	ENSG00000002745
ENSG00000061455	ENSG00000008300	ENSG00000008300	ENSG00000000971	ENSG00000008300
ENSG00000250682	ENSG00000010030	ENSG00000010030	ENSG00000001036	ENSG00000010030
ENSG00000167074	ENSG00000033122	ENSG00000033122	ENSG00000001167	ENSG00000033122
ENSG00000124713	ENSG00000044524	ENSG00000044524	ENSG00000001460	ENSG00000044524
ENSG00000279873	ENSG00000049130	ENSG00000049130	ENSG00000001461	ENSG00000049130
ENSG00000267767	ENSG00000049246	ENSG00000049246	ENSG00000001561	ENSG00000049246
ENSG00000145675	ENSG00000050628	ENSG00000050628	ENSG00000001617	ENSG00000050628
ENSG00000263412	ENSG00000054598	ENSG00000054598	ENSG00000001626	ENSG00000054598
ENSG00000250546	ENSG00000056487	ENSG00000056487	ENSG00000001629	ENSG00000056487
ENSG00000120696	ENSG00000061455	ENSG00000061455	ENSG00000001630	ENSG00000061455
ENSG00000259660	ENSG00000070731	ENSG00000070731	ENSG00000001631	ENSG00000070731
ENSG00000226383	ENSG00000075223	ENSG00000075223	ENSG00000002016	ENSG00000075223
ENSG00000187650	ENSG00000077092	ENSG00000077092	ENSG00000002586	ENSG00000077092
ENSG00000179935	ENSG00000077522	ENSG00000077522	ENSG00000002587	ENSG00000077522
ENSG00000141314	ENSG00000077684	ENSG00000077684	ENSG00000002745	ENSG00000077684
ENSG00000102032	ENSG00000078018	ENSG00000078018	ENSG00000002746	ENSG00000078018
ENSG00000198879	ENSG00000079102	ENSG00000079102	ENSG00000002834	ENSG00000079102
ENSG00000206561	ENSG00000080573	ENSG00000080573	ENSG00000002919	ENSG00000080573
ENSG00000230069	ENSG00000084453	ENSG00000084453	ENSG00000003056	ENSG00000084453
ENSG00000164938	ENSG00000087085	ENSG00000087085	ENSG00000003096	ENSG00000087085
ENSG00000268575	ENSG00000087589	ENSG00000087589	ENSG00000003137	ENSG00000087589
ENSG00000272328	ENSG00000092421	ENSG00000092421	ENSG00000003147	ENSG00000092421
ENSG00000260941	ENSG00000096654	ENSG00000096654	ENSG00000003249	ENSG00000096654
ENSG00000196668	ENSG00000099250	ENSG00000099250	ENSG00000003393	ENSG00000099250
ENSG00000127903	ENSG00000102678	ENSG00000102678	ENSG00000003436	ENSG00000102678
ENSG00000261578	ENSG00000104081	ENSG00000104081	ENSG00000003509	ENSG00000104081
ENSG00000275294	ENSG00000105327	ENSG00000105327	ENSG00000003756	ENSG00000105327
ENSG00000143110	ENSG00000106483	ENSG00000106483	ENSG00000003987	ENSG00000106483
ENSG00000214783	ENSG00000106571	ENSG00000106571	ENSG00000004059	ENSG00000106571
ENSG00000186973	ENSG00000106804	ENSG00000106804	ENSG00000004139	ENSG00000106804
ENSG00000125398	ENSG00000107105	ENSG00000107105	ENSG00000004142	ENSG00000107105
ENSG00000232260	ENSG00000107562	ENSG00000107562	ENSG00000004399	ENSG00000107562
ENSG00000002587	ENSG00000108309	ENSG00000108309	ENSG00000004455	ENSG00000108309
ENSG00000196724	ENSG00000109819	ENSG00000109819	ENSG00000004468	ENSG00000109819
ENSG00000270558	ENSG00000113369	ENSG00000113369	ENSG00000004487	ENSG00000113369

ENSG00000273373	ENSG00000113389	ENSG00000113389	ENSG00000004660	ENSG00000113389
ENSG00000176681	ENSG00000113580	ENSG00000113580	ENSG00000004700	ENSG00000113580
ENSG00000114251	ENSG00000114861	ENSG00000114861	ENSG00000004776	ENSG00000114861
ENSG00000118263	ENSG00000115461	ENSG00000115461	ENSG00000004779	ENSG00000115461
ENSG00000269994	ENSG00000115896	ENSG00000115896	ENSG00000004799	ENSG00000115896
ENSG00000258357	ENSG00000116106	ENSG00000116106	ENSG00000004809	ENSG00000116106
ENSG00000250492	ENSG00000116117	ENSG00000116117	ENSG00000004838	ENSG00000116117
ENSG00000253366	ENSG00000116574	ENSG00000116574	ENSG00000004846	ENSG00000116574
ENSG00000135679	ENSG00000116717	ENSG00000116717	ENSG00000004864	ENSG00000116717
ENSG00000248027	ENSG00000116726	ENSG00000116726	ENSG00000004866	ENSG00000116726
ENSG00000272195	ENSG00000116991	ENSG00000116991	ENSG00000004897	ENSG00000116991
ENSG00000171522	ENSG00000118922	ENSG00000118922	ENSG00000004948	ENSG00000118922
ENSG00000227268	ENSG00000119866	ENSG00000119866	ENSG00000004961	ENSG00000119866
ENSG00000215630	ENSG00000120370	ENSG00000120370	ENSG00000004975	ENSG00000120370
ENSG00000174898	ENSG00000122641	ENSG00000122641	ENSG00000005007	ENSG00000122641
ENSG00000263465	ENSG00000123094	ENSG00000123094	ENSG00000005020	ENSG00000123094
ENSG00000258441	ENSG00000123095	ENSG00000123095	ENSG00000005022	ENSG00000123095
ENSG00000119866	ENSG00000123700	ENSG00000123700	ENSG00000005073	ENSG00000123700
ENSG00000210151	ENSG00000124766	ENSG00000124766	ENSG00000005100	ENSG00000124766
ENSG00000101470	ENSG00000124813	ENSG00000124813	ENSG00000005102	ENSG00000124813
ENSG00000267546	ENSG00000125398	ENSG00000125398	ENSG00000005108	ENSG00000125398
ENSG00000177565	ENSG00000128052	ENSG00000128052	ENSG00000005175	ENSG00000128052
ENSG00000256591	ENSG00000128573	ENSG00000128573	ENSG00000005194	ENSG00000128573
ENSG00000225420	ENSG00000129757	ENSG00000129757	ENSG00000005206	ENSG00000129757
ENSG00000178662	ENSG00000130518	ENSG00000130518	ENSG00000005249	ENSG00000130518
ENSG00000225793	ENSG00000132639	ENSG00000132639	ENSG00000005302	ENSG00000132639
ENSG00000116991	ENSG00000133639	ENSG00000133639	ENSG00000005339	ENSG00000133639
ENSG00000176593	ENSG00000134294	ENSG00000134294	ENSG00000005379	ENSG00000134294
ENSG00000011021	ENSG00000134532	ENSG00000134532	ENSG00000005436	ENSG00000134532
ENSG00000248643	ENSG00000134986	ENSG00000134986	ENSG00000005469	ENSG00000134986
ENSG00000235100	ENSG00000135116	ENSG00000135116	ENSG00000005483	ENSG00000135116
ENSG00000151612	ENSG00000135547	ENSG00000135547	ENSG00000005486	ENSG00000135547
ENSG00000213976	ENSG00000135679	ENSG00000135679	ENSG00000005513	ENSG00000135679
ENSG00000257511	ENSG00000136040	ENSG00000136040	ENSG00000005801	ENSG00000136040
ENSG00000236028	ENSG00000137573	ENSG00000137573	ENSG00000005810	ENSG00000137573
ENSG00000104081	ENSG00000138347	ENSG00000138347	ENSG00000005812	ENSG00000138347
ENSG00000248774	ENSG00000138639	ENSG00000138639	ENSG00000005844	ENSG00000138639
ENSG00000183346	ENSG00000138670	ENSG00000138670	ENSG00000005882	ENSG00000138670

ENSG00000124788	ENSG00000139209	ENSG00000139209	ENSG00000005884	ENSG00000139209
ENSG00000122641	ENSG00000139269	ENSG00000139269	ENSG00000005889	ENSG00000139269
ENSG00000162599	ENSG00000139364	ENSG00000139364	ENSG00000005893	ENSG00000139364
ENSG00000115841	ENSG00000139946	ENSG00000139946	ENSG00000005981	ENSG00000139946
ENSG00000170153	ENSG00000141431	ENSG00000141431	ENSG00000006007	ENSG00000141431
ENSG00000262251	ENSG00000143469	ENSG00000143469	ENSG00000006062	ENSG00000143469
ENSG00000105516	ENSG00000143995	ENSG00000143995	ENSG00000006116	ENSG00000143995
ENSG00000066279	ENSG00000145087	ENSG00000145087	ENSG00000006283	ENSG00000145087
ENSG00000206120	ENSG00000145536	ENSG00000145536	ENSG00000006327	ENSG00000145536
ENSG00000229292	ENSG00000145632	ENSG00000145632	ENSG00000006377	ENSG00000145632
ENSG00000254165	ENSG00000145687	ENSG00000145687	ENSG00000006432	ENSG00000145687
ENSG00000134138	ENSG00000147606	ENSG00000147606	ENSG00000006451	ENSG00000147606
ENSG00000223891	ENSG00000147852	ENSG00000147852	ENSG00000006453	ENSG00000147852
ENSG00000168743	ENSG00000147862	ENSG00000147862	ENSG00000006459	ENSG00000147862
ENSG00000177606	ENSG00000148541	ENSG00000148541	ENSG00000006468	ENSG00000148541
ENSG00000267506	ENSG00000149212	ENSG00000149212	ENSG00000006530	ENSG00000149212
ENSG00000166415	ENSG00000150244	ENSG00000150244	ENSG00000006534	ENSG00000150244
ENSG00000225511	ENSG00000150347	ENSG00000150347	ENSG00000006555	ENSG00000150347
ENSG00000185008	ENSG00000150394	ENSG00000150394	ENSG00000006576	ENSG00000150394
ENSG00000118922	ENSG00000150551	ENSG00000150551	ENSG00000006606	ENSG00000150551
ENSG00000271109	ENSG00000150687	ENSG00000150687	ENSG00000006607	ENSG00000150687
ENSG00000263884	ENSG00000151623	ENSG00000151623	ENSG00000006611	ENSG00000151623
ENSG00000105855	ENSG00000152217	ENSG00000152217	ENSG00000006625	ENSG00000152217
ENSG00000258405	ENSG00000152763	ENSG00000152763	ENSG00000006634	ENSG00000152763
ENSG00000155966	ENSG00000154736	ENSG00000154736	ENSG00000006638	ENSG00000154736
ENSG00000116574	ENSG00000157765	ENSG00000157765	ENSG00000006695	ENSG00000157765
ENSG00000188295	ENSG00000162599	ENSG00000162599	ENSG00000006747	ENSG00000162599
ENSG00000139946	ENSG00000162814	ENSG00000162814	ENSG00000006756	ENSG00000162814
ENSG00000262454	ENSG00000164463	ENSG00000164463	ENSG00000007001	ENSG00000164463
ENSG00000148541	ENSG00000164743	ENSG00000164743	ENSG00000007062	ENSG00000164743
ENSG00000161912	ENSG00000164746	ENSG00000164746	ENSG00000007129	ENSG00000164746
ENSG00000258900	ENSG00000164949	ENSG00000164949	ENSG00000007168	ENSG00000164949
ENSG00000176204	ENSG00000165244	ENSG00000165244	ENSG00000007202	ENSG00000165244
ENSG00000279347	ENSG00000165323	ENSG00000165323	ENSG00000007216	ENSG00000165323
ENSG00000180530	ENSG00000165617	ENSG00000165617	ENSG00000007255	ENSG00000165617
ENSG00000261116	ENSG00000165655	ENSG00000165655	ENSG00000007306	ENSG00000165655
ENSG00000188177	ENSG00000166793	ENSG00000166793	ENSG00000007314	ENSG00000166793
ENSG00000185483	ENSG00000166866	ENSG00000166866	ENSG00000007341	ENSG00000166866

ENSG00000236829	ENSG00000167995	ENSG00000167995	ENSG00000007350	ENSG00000167995
ENSG00000266490	ENSG00000168743	ENSG00000168743	ENSG00000007376	ENSG00000168743
ENSG00000204962	ENSG00000168772	ENSG00000168772	ENSG00000007392	ENSG00000168772
ENSG00000005483	ENSG00000168916	ENSG00000168916	ENSG00000007402	ENSG00000168916
ENSG00000231551	ENSG00000169116	ENSG00000169116	ENSG00000007541	ENSG00000169116
ENSG00000234996	ENSG00000169184	ENSG00000169184	ENSG00000007545	ENSG00000169184
ENSG00000255282	ENSG00000169432	ENSG00000169432	ENSG00000007866	ENSG00000169432
ENSG00000127152	ENSG00000169554	ENSG00000169554	ENSG00000007908	ENSG00000169554
ENSG00000271971	ENSG00000169851	ENSG00000169851	ENSG00000007923	ENSG00000169851
ENSG00000231160	ENSG00000170214	ENSG00000170214	ENSG00000007944	ENSG00000170214
ENSG00000251562	ENSG00000170836	ENSG00000170836	ENSG00000007952	ENSG00000170836
ENSG00000274386	ENSG00000171724	ENSG00000171724	ENSG00000007968	ENSG00000171724
ENSG00000151967	ENSG00000172059	ENSG00000172059	ENSG00000008083	ENSG00000172059
ENSG00000182463	ENSG00000174738	ENSG00000174738	ENSG00000008086	ENSG00000174738
ENSG00000141639	ENSG00000174844	ENSG00000174844	ENSG00000008118	ENSG00000174844
ENSG00000248441	ENSG00000175745	ENSG00000175745	ENSG00000008130	ENSG00000175745
ENSG00000272720	ENSG00000176046	ENSG00000176046	ENSG00000008196	ENSG00000176046
ENSG00000186529	ENSG00000176771	ENSG00000176771	ENSG00000008256	ENSG00000176771
ENSG00000196152	ENSG00000176788	ENSG00000176788	ENSG00000008277	ENSG00000176788
ENSG00000213453	ENSG00000176887	ENSG00000176887	ENSG00000008283	ENSG00000176887
ENSG00000246876	ENSG00000176971	ENSG00000176971	ENSG00000008294	ENSG00000176971
ENSG00000168779	ENSG00000177565	ENSG00000177565	ENSG00000008300	ENSG00000177565
ENSG00000177822	ENSG00000177606	ENSG00000177606	ENSG00000008311	ENSG00000177606
ENSG00000106034	ENSG00000178568	ENSG00000178568	ENSG00000008323	ENSG00000178568
ENSG00000235109	ENSG00000178573	ENSG00000178573	ENSG00000008324	ENSG00000178573
ENSG00000163431	ENSG00000178878	ENSG00000178878	ENSG00000008394	ENSG00000178878
ENSG00000265817	ENSG00000179981	ENSG00000179981	ENSG00000008441	ENSG00000179981
ENSG00000231728	ENSG00000180530	ENSG00000180530	ENSG00000008513	ENSG00000180530
ENSG00000117724	ENSG00000180592	ENSG00000180592	ENSG00000008710	ENSG00000180592
ENSG00000133639	ENSG00000181016	ENSG00000181016	ENSG00000008838	ENSG00000181016
ENSG00000272888	ENSG00000181449	ENSG00000181449	ENSG00000008853	ENSG00000181449
ENSG00000174804	ENSG00000182168	ENSG00000182168	ENSG00000009307	ENSG00000182168
ENSG00000244694	ENSG00000182308	ENSG00000182308	ENSG00000009335	ENSG00000182308
ENSG00000278000	ENSG00000182330	ENSG00000182330	ENSG00000009413	ENSG00000182330
ENSG00000226853	ENSG00000183098	ENSG00000183098	ENSG00000009694	ENSG00000183098
ENSG00000260461	ENSG00000183496	ENSG00000183496	ENSG00000009724	ENSG00000183496
ENSG00000239763	ENSG00000184349	ENSG00000184349	ENSG00000009780	ENSG00000184349
ENSG00000278175	ENSG00000185070	ENSG00000185070	ENSG00000009790	ENSG00000185070

ENSG00000255524	ENSG00000185483	ENSG00000185483	ENSG00000009830	ENSG00000185483
ENSG00000172059	ENSG00000185630	ENSG00000185630	ENSG00000009844	ENSG00000185630
ENSG00000084453	ENSG00000185736	ENSG00000185736	ENSG00000009950	ENSG00000185736
ENSG00000139364	ENSG00000186479	ENSG00000186479	ENSG00000009954	ENSG00000186479
ENSG00000185267	ENSG00000186517	ENSG00000186517	ENSG00000010017	ENSG00000186517
ENSG00000002745	ENSG00000188177	ENSG00000188177	ENSG00000010278	ENSG00000188177
ENSG00000228409	ENSG00000188580	ENSG00000188580	ENSG00000010295	ENSG00000188580
ENSG00000188158	ENSG00000188959	ENSG00000188959	ENSG00000010318	ENSG00000188959
ENSG00000050438	ENSG00000188993	ENSG00000188993	ENSG00000010319	ENSG00000188993
ENSG00000164684	ENSG00000189184	ENSG00000189184	ENSG00000010322	ENSG00000189184
ENSG00000168916	ENSG00000189325	ENSG00000189325	ENSG00000010404	ENSG00000189325
ENSG00000187372	ENSG00000196159	ENSG00000196159	ENSG00000010539	ENSG00000196159
ENSG00000135315	ENSG00000196724	ENSG00000196724	ENSG00000010610	ENSG00000196724
ENSG00000176896	ENSG00000196781	ENSG00000196781	ENSG00000010626	ENSG00000196781
ENSG00000172086	ENSG00000196782	ENSG00000196782	ENSG00000010671	ENSG00000196782
ENSG00000105499	ENSG00000198732	ENSG00000198732	ENSG00000010932	ENSG00000198732
ENSG00000245526	ENSG00000204682	ENSG00000204682	ENSG00000011105	ENSG00000204682
ENSG00000196628	ENSG00000205189	ENSG00000205189	ENSG00000011114	ENSG00000205189
ENSG00000137203	ENSG00000218336	ENSG00000218336	ENSG00000011295	ENSG00000218336
ENSG00000168143	ENSG00000235109	ENSG00000235109	ENSG00000013275	ENSG00000235109
ENSG00000168772	ENSG00000242689	ENSG00000242689	ENSG00000013588	ENSG00000242689
ENSG00000145242	ENSG00000250120	ENSG00000250120	ENSG00000015153	ENSG00000250120
ENSG00000236204	ENSG00000251258	ENSG00000251258	ENSG00000016082	ENSG00000251258
ENSG00000213073	ENSG00000253305	ENSG00000253305	ENSG00000016864	ENSG00000253305
ENSG00000111215	ENSG00000253846	ENSG00000253846	ENSG00000017260	ENSG00000253846
ENSG00000145945	ENSG00000258405	ENSG00000258405	ENSG00000018625	ENSG00000258405
ENSG00000125551	ENSG00000265817	ENSG00000265817	ENSG00000020633	ENSG00000265817
ENSG00000230002	ENSG00000265972	ENSG00000265972	ENSG00000021300	ENSG00000265972
ENSG00000239335	ENSG00000276644	ENSG00000276644	ENSG00000024862	ENSG00000276644
ENSG00000182903	ENSG00000277443	ENSG00000277443	ENSG00000025293	ENSG00000277443
ENSG00000196782	ENSG00000026025	ENSG00000026025	ENSG00000001084	
ENSG00000046653	ENSG00000077063	ENSG00000077063	ENSG00000002330	
ENSG00000107485	ENSG00000099251	ENSG00000099251	ENSG00000003400	
ENSG00000136040	ENSG00000102032	ENSG00000102032	ENSG00000003402	
ENSG00000009413	ENSG00000106536	ENSG00000106536	ENSG00000003989	
ENSG00000113580	ENSG00000112175	ENSG00000112175	ENSG00000004478	
ENSG00000224843	ENSG00000113916	ENSG00000113916	ENSG00000004766	
ENSG00000158321	ENSG00000118407	ENSG00000118407	ENSG00000004939	

ENSG00000253598	ENSG00000124635	ENSG00000124635	ENSG00000005075
ENSG00000260526	ENSG00000126368	ENSG00000126368	ENSG00000005156
ENSG00000261326	ENSG00000130222	ENSG00000130222	ENSG00000005238
ENSG00000230185	ENSG00000134363	ENSG00000134363	ENSG00000005381
ENSG00000143816	ENSG00000141665	ENSG00000141665	ENSG00000006025
ENSG00000175105	ENSG00000143032	ENSG00000143032	ENSG00000006042
ENSG00000178878	ENSG00000143110	ENSG00000143110	ENSG00000006047
ENSG00000235609	ENSG00000144010	ENSG00000144010	ENSG00000006125
ENSG00000272070	ENSG00000144015	ENSG00000144015	ENSG00000006128
ENSG00000250222	ENSG00000144188	ENSG00000144188	ENSG00000006194
ENSG00000267284	ENSG00000144214	ENSG00000144214	ENSG00000006210
ENSG00000248015	ENSG00000157445	ENSG00000157445	ENSG00000006652
ENSG00000272669	ENSG00000158373	ENSG00000158373	ENSG00000006704
ENSG00000235946	ENSG00000158406	ENSG00000158406	ENSG00000006715
ENSG00000138639	ENSG00000158486	ENSG00000158486	ENSG00000006740
ENSG00000116678	ENSG00000159208	ENSG00000159208	ENSG00000006744
ENSG00000109819	ENSG00000163009	ENSG00000163009	ENSG00000006757
ENSG00000224886	ENSG00000164309	ENSG00000164309	ENSG00000006831
ENSG00000150551	ENSG00000164442	ENSG00000164442	ENSG00000006837
ENSG00000219626	ENSG00000164708	ENSG00000164708	ENSG00000007047
ENSG00000137968	ENSG00000165182	ENSG00000165182	ENSG00000007171
ENSG00000122257	ENSG00000165555	ENSG00000165555	ENSG00000007237
ENSG00000226476	ENSG00000166013	ENSG00000166013	ENSG00000007312
ENSG00000253414	ENSG00000168542	ENSG00000168542	ENSG00000007372
ENSG00000166432	ENSG00000168930	ENSG00000168930	ENSG00000007516
ENSG00000270933	ENSG00000171951	ENSG00000171951	ENSG00000008056
ENSG00000121297	ENSG00000180532	ENSG00000180532	ENSG00000008516
ENSG00000265218	ENSG00000180573	ENSG00000180573	ENSG00000008517
ENSG00000008083	ENSG00000180596	ENSG00000180596	ENSG00000008735
ENSG00000153714	ENSG00000182053	ENSG00000182053	ENSG00000008952
ENSG00000184347	ENSG00000183336	ENSG00000183336	ENSG00000009709
ENSG00000128052	ENSG00000183813	ENSG00000183813	ENSG00000009765
ENSG00000224892	ENSG00000186976	ENSG00000186976	ENSG00000010030
ENSG00000260077	ENSG00000187775	ENSG00000187775	ENSG00000010072
ENSG00000196357	ENSG00000187837	ENSG00000187837	ENSG00000010256
ENSG00000168702	ENSG00000188738	ENSG00000188738	ENSG00000010310
ENSG00000250337	ENSG00000189253	ENSG00000189253	ENSG00000010379
ENSG00000106069	ENSG00000196787	ENSG00000196787	ENSG00000010704

ENSG00000271918	ENSG00000196812	ENSG00000196812	ENSG00000010803
ENSG00000183287	ENSG00000196890	ENSG00000196890	ENSG00000010810
ENSG00000250305	ENSG00000198075	ENSG00000198075	ENSG00000010818
ENSG00000187720	ENSG00000203740	ENSG00000203740	ENSG00000011007
ENSG00000129749	ENSG00000203814	ENSG00000203814	ENSG00000011009
ENSG00000169554	ENSG00000204449	ENSG00000204449	ENSG00000011021
ENSG00000272767	ENSG00000204450	ENSG00000204450	ENSG00000011028
ENSG00000213363	ENSG00000204455	ENSG00000204455	ENSG00000011052
ENSG00000262728	ENSG00000204510	ENSG00000204510	ENSG00000011083
ENSG00000158258	ENSG00000210151	ENSG00000210151	ENSG00000011143
ENSG00000124743	ENSG00000213363	ENSG00000213363	ENSG00000011198
ENSG00000261251	ENSG00000213453	ENSG00000213453	ENSG00000011201
ENSG00000272142	ENSG00000214243	ENSG00000214243	ENSG00000011258
ENSG00000180592	ENSG00000215018	ENSG00000215018	ENSG00000011275
ENSG00000247675	ENSG00000223417	ENSG00000223417	ENSG00000011304
ENSG00000010030	ENSG00000223638	ENSG00000223638	ENSG00000011332
ENSG00000151164	ENSG00000223855	ENSG00000223855	ENSG00000011347
ENSG00000165617	ENSG00000224294	ENSG00000224294	ENSG00000011376
ENSG00000153234	ENSG00000224892	ENSG00000224892	ENSG00000011405
ENSG00000113916	ENSG00000225420	ENSG00000225420	ENSG00000011422
ENSG00000254402	ENSG00000225581	ENSG00000225581	ENSG00000011426
ENSG00000165182	ENSG00000225792	ENSG00000225792	ENSG00000011451
ENSG00000081818	ENSG00000225793	ENSG00000225793	ENSG00000011454
ENSG00000152784	ENSG00000226476	ENSG00000226476	ENSG00000011465
ENSG00000280367	ENSG00000228314	ENSG00000228314	ENSG00000011478
ENSG00000225465	ENSG00000228409	ENSG00000228409	ENSG00000011485
ENSG00000100027	ENSG00000228451	ENSG00000228451	ENSG00000011523
ENSG00000261652	ENSG00000229127	ENSG00000229127	ENSG00000011600
ENSG00000106258	ENSG00000229292	ENSG00000229292	ENSG00000011638
ENSG00000145687	ENSG00000229619	ENSG00000229619	ENSG00000011677
ENSG00000260265	ENSG00000229978	ENSG00000229978	ENSG00000012048
ENSG00000106829	ENSG00000230069	ENSG00000230069	ENSG00000012061
ENSG00000116785	ENSG00000230699	ENSG00000230699	ENSG00000012124
ENSG00000251432	ENSG00000231160	ENSG00000231160	ENSG00000012171
ENSG00000147862	ENSG00000233030	ENSG00000233030	ENSG00000012174
ENSG00000145431	ENSG00000233045	ENSG00000233045	ENSG00000012211
ENSG00000110693	ENSG00000233639	ENSG00000233639	ENSG00000012232
ENSG00000110031	ENSG00000233802	ENSG00000233802	ENSG00000012504

ENSG00000186976	ENSG00000233822	ENSG00000233822	ENSG00000012660
ENSG00000260589	ENSG00000233930	ENSG00000233930	ENSG00000012817
ENSG00000223855	ENSG00000234424	ENSG00000234424	ENSG00000012822
ENSG00000224505	ENSG00000234585	ENSG00000234585	ENSG00000012963
ENSG00000221883	ENSG00000234996	ENSG00000234996	ENSG00000012983
ENSG00000120327	ENSG00000235100	ENSG00000235100	ENSG00000013016
ENSG00000166922	ENSG00000235268	ENSG00000235268	ENSG00000013288
ENSG00000104957	ENSG00000235618	ENSG00000235618	ENSG00000013293
ENSG00000077092	ENSG00000236175	ENSG00000236175	ENSG00000013297
ENSG00000253305	ENSG00000236941	ENSG00000236941	ENSG00000013306
ENSG00000178568	ENSG00000236993	ENSG00000236993	ENSG00000013374
ENSG00000272562	ENSG00000237187	ENSG00000237187	ENSG00000013375
ENSG00000274422	ENSG00000237693	ENSG00000237693	ENSG00000013392
ENSG00000117461	ENSG00000237706	ENSG00000237706	ENSG00000013523
ENSG00000170271	ENSG00000239405	ENSG00000239405	ENSG00000013561
ENSG00000153094	ENSG00000240291	ENSG00000240291	ENSG00000013563
ENSG00000147852	ENSG00000240498	ENSG00000240498	ENSG00000013573
ENSG00000217236	ENSG00000241170	ENSG00000241170	ENSG00000013583
ENSG00000198342	ENSG00000243819	ENSG00000243819	ENSG00000013619
ENSG00000227885	ENSG00000245205	ENSG00000245205	ENSG00000013725
ENSG00000173531	ENSG00000245532	ENSG00000245532	ENSG00000014123
ENSG00000082684	ENSG00000246876	ENSG00000246876	ENSG00000014138
ENSG00000117586	ENSG00000247809	ENSG00000247809	ENSG00000014216
ENSG00000204802	ENSG00000248441	ENSG00000248441	ENSG00000014257
ENSG00000185432	ENSG00000248596	ENSG00000248596	ENSG00000014824
ENSG00000176046	ENSG00000248774	ENSG00000248774	ENSG00000014914
ENSG00000116774	ENSG00000249156	ENSG00000249156	ENSG00000014919
ENSG00000260572	ENSG00000249620	ENSG00000249620	ENSG00000015133
ENSG00000198237	ENSG00000250337	ENSG00000250337	ENSG00000015171
ENSG00000261732	ENSG00000250386	ENSG00000250386	ENSG00000015413
ENSG00000138161	ENSG00000250492	ENSG00000250492	ENSG00000015475
ENSG00000117569	ENSG00000250519	ENSG00000250519	ENSG00000015520
ENSG00000106701	ENSG00000250546	ENSG00000250546	ENSG00000015532
ENSG00000038427	ENSG00000250782	ENSG00000250782	ENSG00000015568
ENSG00000033122	ENSG00000251022	ENSG00000251022	ENSG00000015592
ENSG00000224046	ENSG00000251095	ENSG00000251095	ENSG00000015676
ENSG00000188994	ENSG00000251562	ENSG00000251562	ENSG00000016391
ENSG00000099377	ENSG00000251634	ENSG00000251634	ENSG00000016402

ENSG00000149294	ENSG00000254764	ENSG00000254764	ENSG00000017427
ENSG00000261557	ENSG00000255389	ENSG00000255389	ENSG00000017483
ENSG00000162849	ENSG00000255471	ENSG00000255471	ENSG00000017797
ENSG00000261126	ENSG00000255855	ENSG00000255855	ENSG00000018189
ENSG00000177732	ENSG00000256304	ENSG00000256304	ENSG00000018236
ENSG00000144668	ENSG00000256779	ENSG00000256779	ENSG00000018280
ENSG00000120669	ENSG00000257261	ENSG00000257261	ENSG00000018408
ENSG00000185261	ENSG00000257951	ENSG00000257951	ENSG00000018510
ENSG00000107562	ENSG00000258357	ENSG00000258357	ENSG00000018610
ENSG00000183336	ENSG00000258472	ENSG00000258472	ENSG00000018699
ENSG00000169239	ENSG00000259275	ENSG00000259275	ENSG00000018869
ENSG00000176371	ENSG00000259456	ENSG00000259456	ENSG00000019144
ENSG00000275560	ENSG00000260077	ENSG00000260077	ENSG00000019169
ENSG00000111276	ENSG00000260103	ENSG00000260103	ENSG00000019186
ENSG00000169851	ENSG00000260572	ENSG00000260572	ENSG00000019485
ENSG00000184349	ENSG00000260589	ENSG00000260589	ENSG00000019505
ENSG00000138670	ENSG00000260727	ENSG00000260727	ENSG00000019549
ENSG00000250120	ENSG00000261087	ENSG00000261087	ENSG00000019582
ENSG00000233325	ENSG00000261116	ENSG00000261116	ENSG00000019991
ENSG00000105327	ENSG00000261136	ENSG00000261136	ENSG00000019995
ENSG00000114861	ENSG00000261557	ENSG00000261557	ENSG00000020129
ENSG00000126016	ENSG00000262728	ENSG00000262728	ENSG00000020181
ENSG00000109787	ENSG00000263080	ENSG00000263080	ENSG00000020256
ENSG00000123095	ENSG00000263745	ENSG00000263745	ENSG00000020577
ENSG00000269834	ENSG00000265962	ENSG00000265962	ENSG00000020922
ENSG00000272630	ENSG00000266490	ENSG00000266490	ENSG00000021574
ENSG00000197989	ENSG00000267365	ENSG00000267365	ENSG00000021645
ENSG00000167081	ENSG00000267731	ENSG00000267731	ENSG00000021762
ENSG00000276900	ENSG00000268756	ENSG00000268756	ENSG00000021852
ENSG00000234345	ENSG00000268799	ENSG00000268799	ENSG00000022267
ENSG00000281195	ENSG00000269466	ENSG00000269466	ENSG00000022277
ENSG00000134595	ENSG00000269974	ENSG00000269974	ENSG00000022355
ENSG00000189136	ENSG00000270276	ENSG00000270276	ENSG00000022567
ENSG00000258472	ENSG00000270605	ENSG00000270605	ENSG00000022840
ENSG00000204584	ENSG00000270681	ENSG00000270681	ENSG00000023041
ENSG00000204311	ENSG00000270876	ENSG00000270876	ENSG00000023171
ENSG00000169946	ENSG00000270882	ENSG00000270882	ENSG00000023287
ENSG00000254122	ENSG00000271474	ENSG00000271474	ENSG00000023318

ENSG00000176971	ENSG00000271918	ENSG00000271918	ENSG00000023445
ENSG00000174738	ENSG00000272341	ENSG00000272341	ENSG00000023516
ENSG00000183098	ENSG00000272398	ENSG00000272398	ENSG00000023608
ENSG00000273007	ENSG00000272468	ENSG00000272468	ENSG00000023697
ENSG00000241547	ENSG00000272720	ENSG00000272720	ENSG00000023734
ENSG00000148737	ENSG00000272767	ENSG00000272767	ENSG00000023892
ENSG00000248019	ENSG00000272870	ENSG00000272870	ENSG00000023902
ENSG00000179981	ENSG00000273038	ENSG00000273038	ENSG00000023909
ENSG00000141665	ENSG00000273802	ENSG00000273802	ENSG00000024048
ENSG00000229956	ENSG00000274290	ENSG00000274290	ENSG00000024422
ENSG00000184486	ENSG00000274460	ENSG00000274460	ENSG00000024526
ENSG00000145632	ENSG00000276966	ENSG00000276966	ENSG00000025039
ENSG00000185070	ENSG00000277383	ENSG00000277383	ENSG00000025156
ENSG00000275620	ENSG00000277862	ENSG00000277862	ENSG00000025770
ENSG00000259275	ENSG00000278238	ENSG00000278238	ENSG00000025772
ENSG00000218336	ENSG00000279208	ENSG00000279208	ENSG00000025796
ENSG00000196812	ENSG00000279355	ENSG00000279355	ENSG00000025800
ENSG00000164949	ENSG00000279369	ENSG00000279369	ENSG00000026103
ENSG00000112175	ENSG00000279865	ENSG00000279865	ENSG00000026508
ENSG00000156650	ENSG00000283235	ENSG00000283235	ENSG00000026559
ENSG00000234329	ENSG00000283740	ENSG00000283740	ENSG00000026652
ENSG00000171724	ENSG00000283776	ENSG00000283776	ENSG00000026751
ENSG00000166473	ENSG00000283873	ENSG00000283873	ENSG00000026950
ENSG00000101928	ENSG00000284018	ENSG00000284018	ENSG00000027075
ENSG00000198846	ENSG00000284306	ENSG00000284306	ENSG00000028116
ENSG00000176723	ENSG00000284438	ENSG00000284438	ENSG00000028137
ENSG00000253816	ENSG00000284526	ENSG00000284526	ENSG00000028203
ENSG00000162814	ENSG00000284546	ENSG00000284546	ENSG00000028277
ENSG00000196605	ENSG00000189060		ENSG00000028310
ENSG00000177599	ENSG00000151725		ENSG00000028528
ENSG00000196263	ENSG00000148677		ENSG00000029153
ENSG00000083817	ENSG00000149201		ENSG00000029363
ENSG00000186496	ENSG00000087074		ENSG00000029364
ENSG00000149050	ENSG00000115738		ENSG00000029534
ENSG00000121417	ENSG00000135269		ENSG00000029559
ENSG00000096654	ENSG00000174371		ENSG00000029639
ENSG00000205189	ENSG00000112742		ENSG00000029725
ENSG00000228775	ENSG00000170558		ENSG00000029993

ENSG00000178201	ENSG00000149948	ENSG00000030066
ENSG00000100271	ENSG00000105974	ENSG00000030110
ENSG00000155428	ENSG00000170017	ENSG00000030304
ENSG00000071575	ENSG00000168564	ENSG00000030419
ENSG00000230359	ENSG00000174579	ENSG00000030582
ENSG00000271092	ENSG00000248873	ENSG00000031003
ENSG00000228158	ENSG00000138814	ENSG00000031081
ENSG00000251022	ENSG00000236539	ENSG00000031691
ENSG00000229950	ENSG00000171476	ENSG00000031698
ENSG00000168769	ENSG00000143333	ENSG00000031823
ENSG00000101463	ENSG00000137831	ENSG00000032219
ENSG00000254995	ENSG00000116044	ENSG00000032389
ENSG00000242866	ENSG00000248971	ENSG00000033011
ENSG00000145147	ENSG00000214900	ENSG00000033030
ENSG00000134294	ENSG00000138050	ENSG00000033050
ENSG00000112246	ENSG00000165072	ENSG00000033122
ENSG00000137872	ENSG00000165480	ENSG00000033170
ENSG00000124813	ENSG00000168556	ENSG00000033178
ENSG00000108309	ENSG00000066468	ENSG00000033327
ENSG00000230364	ENSG00000197050	ENSG00000033627
ENSG00000232104	ENSG00000259485	ENSG00000033800
ENSG00000123094	ENSG00000123572	ENSG00000033867
ENSG00000185532	ENSG00000023608	ENSG00000034053
ENSG00000106536	ENSG00000121361	ENSG00000034152
ENSG00000240694	ENSG00000164776	ENSG00000034239
ENSG00000162407	ENSG00000100142	ENSG00000034533
ENSG00000182621	ENSG00000228169	ENSG00000034677
ENSG00000171033	ENSG00000229083	ENSG00000034713
ENSG00000134853	ENSG00000239269	ENSG00000034971
ENSG00000168300	ENSG00000146707	ENSG00000035115
ENSG00000240764	ENSG00000134438	ENSG00000035141
ENSG00000253731	ENSG00000249465	ENSG00000035403
ENSG00000113212	ENSG00000168621	ENSG00000035499
ENSG00000113205	ENSG00000064042	ENSG00000035664
ENSG00000120324	ENSG00000259802	ENSG00000035687
ENSG00000185551	ENSG00000237440	ENSG00000035720
ENSG00000173614	ENSG00000203943	ENSG00000035862
ENSG00000177453	ENSG00000196081	ENSG00000035928

ENSG00000140396	ENSG00000220804	ENSG00000036054
ENSG00000186310	ENSG00000188404	ENSG00000036257
ENSG00000188613	ENSG00000226102	ENSG00000036530
ENSG00000281026	ENSG00000240914	ENSG00000036549
ENSG00000166866	ENSG00000163535	ENSG00000036565
ENSG00000059728	ENSG00000180376	ENSG00000036672
ENSG00000262902	ENSG00000139174	ENSG00000036828
ENSG00000221184	ENSG00000085276	ENSG00000037241
ENSG00000203740	ENSG00000004799	ENSG00000037280
ENSG00000102316	ENSG00000158014	ENSG00000037474
ENSG00000188993	ENSG00000011201	ENSG00000037637
ENSG00000185158	ENSG00000235245	ENSG00000037749
ENSG00000203441	ENSG00000112182	ENSG00000037757
ENSG00000111052	ENSG00000226763	ENSG00000037897
ENSG00000115548	ENSG00000229124	ENSG00000037965
ENSG00000176049	ENSG00000242952	ENSG00000038002
ENSG00000077684	ENSG00000234911	ENSG00000038274
ENSG00000105655	ENSG00000185339	ENSG00000038295
ENSG00000185950	ENSG00000180998	ENSG00000038382
ENSG00000168264	ENSG00000267796	ENSG00000038427
ENSG00000168918	ENSG00000114423	ENSG00000038532
ENSG00000125430	ENSG00000275342	ENSG00000038945
ENSG00000135547	ENSG00000146070	ENSG00000039068
ENSG00000253203	ENSG00000119865	ENSG00000039123
ENSG00000123201	ENSG00000218890	ENSG00000039139
ENSG00000196275	ENSG00000249069	ENSG00000039319
ENSG00000106571	ENSG00000138028	ENSG00000039523
ENSG00000164404	ENSG00000277462	ENSG00000039537
ENSG00000053254	ENSG00000218358	ENSG00000039560
ENSG00000118407	ENSG00000221994	ENSG00000040199
ENSG00000112773	ENSG00000214892	ENSG00000040341
ENSG00000235618	ENSG00000153832	ENSG00000040531
ENSG00000224032	ENSG00000270964	ENSG00000040633
ENSG00000245205	ENSG00000272711	ENSG00000040933
ENSG00000179532	ENSG00000183671	ENSG00000041353
ENSG00000176124	ENSG00000166578	ENSG00000041515
ENSG00000134574	ENSG00000187624	ENSG00000041802
ENSG00000077063	ENSG00000137672	ENSG00000041982

ENSG00000143578	ENSG00000146674	ENSG00000041988
ENSG00000214575	ENSG00000270011	ENSG00000042062
ENSG00000080573	ENSG00000049192	ENSG00000042088
ENSG00000164442	ENSG00000138653	ENSG00000042286
ENSG00000183196	ENSG00000150636	ENSG00000042429
ENSG00000111860	ENSG00000205592	ENSG00000042445
ENSG00000048740	ENSG00000103460	ENSG00000042493
ENSG00000147883	ENSG00000105792	ENSG00000042753
ENSG00000163171	ENSG00000157168	ENSG00000042781
ENSG00000184305	ENSG00000260339	ENSG00000042980
ENSG00000186409	ENSG00000141934	ENSG00000043039
ENSG00000272168	ENSG00000269906	ENSG00000043093
ENSG00000204682	ENSG00000231458	ENSG00000043143
ENSG00000145349	ENSG00000250790	ENSG00000043355
ENSG00000183049	ENSG00000166262	ENSG00000043462
ENSG00000153956	ENSG00000152402	ENSG00000043514
ENSG00000164743	ENSG00000169031	ENSG00000043591
ENSG00000164746	ENSG00000137090	ENSG00000044012
ENSG00000182326	ENSG00000109906	ENSG00000044115
ENSG00000010671	ENSG00000176884	ENSG00000044446
ENSG00000138696	ENSG00000247728	ENSG00000044459
ENSG00000176788	ENSG00000246528	ENSG00000044524
ENSG00000100852	ENSG00000254427	ENSG00000044574
ENSG00000004777	ENSG00000283403	ENSG00000046604
ENSG00000279355	ENSG00000226468	ENSG00000046647
ENSG00000233435	ENSG00000226754	ENSG00000046653
ENSG00000214273	ENSG00000271888	ENSG00000046889
ENSG00000198221	ENSG00000226471	ENSG00000047056
ENSG00000142303	ENSG00000251867	ENSG00000047188
ENSG00000087085	ENSG00000260244	ENSG00000047230
ENSG00000223764	ENSG00000279145	ENSG00000047249
ENSG00000260293	ENSG00000120675	ENSG00000047365
ENSG00000259943	ENSG00000148848	ENSG00000047410
ENSG00000254815	ENSG00000148053	ENSG00000047457
ENSG00000244627	ENSG00000271614	ENSG00000047578
ENSG00000219665	ENSG00000135346	ENSG00000047579
ENSG00000215158	ENSG00000230043	ENSG00000047597
ENSG00000254701	ENSG00000259051	ENSG00000047617

ENSG00000263745	ENSG00000114315	ENSG00000047621
ENSG00000269399	ENSG00000137193	ENSG00000047634
ENSG00000241288	ENSG00000232956	ENSG00000047644
ENSG00000272301	ENSG00000198734	ENSG00000047648
ENSG00000242622	ENSG00000173530	ENSG00000047849
ENSG00000272870	ENSG00000128610	ENSG00000047932
ENSG00000280239	ENSG00000162772	ENSG00000048028
ENSG00000260966	ENSG00000115414	ENSG00000048052
ENSG00000279520	ENSG00000115274	ENSG00000048140
ENSG00000262877	ENSG00000121621	ENSG00000048162
ENSG00000279369	ENSG00000102554	ENSG00000048342
ENSG00000276728	ENSG00000138182	ENSG00000048392
ENSG00000241764	ENSG00000115963	ENSG00000048471
ENSG00000279673	ENSG00000163659	ENSG00000048540
ENSG00000259366	ENSG00000086967	ENSG00000048544
ENSG00000241170	ENSG00000101384	ENSG00000048545
ENSG00000268670	ENSG00000197168	ENSG00000048707
ENSG00000228395	ENSG00000213315	ENSG00000048740
ENSG00000277383	ENSG00000261934	ENSG00000048828
ENSG00000279511	ENSG00000230847	ENSG00000048991
ENSG00000271895	ENSG00000267178	ENSG00000049089
ENSG00000280061	ENSG00000146592	ENSG00000049130
ENSG00000256092	ENSG00000270067	ENSG00000049167
ENSG00000259349	ENSG00000265254	ENSG00000049192
ENSG00000272812	ENSG00000106546	ENSG00000049239
ENSG00000269974	ENSG00000232368	ENSG00000049245
ENSG00000279199	ENSG00000278974	ENSG00000049246
ENSG00000183779	ENSG00000254480	ENSG00000049247
ENSG00000198795	ENSG00000150630	ENSG00000049249
ENSG00000165244	ENSG00000104313	ENSG00000049283
ENSG00000176293	ENSG00000118898	ENSG00000049449
ENSG00000143184	ENSG00000151012	ENSG00000049540
ENSG00000197355	ENSG00000134533	ENSG00000049541
ENSG00000154310	ENSG00000176697	ENSG00000049618
ENSG00000147003	ENSG00000266709	ENSG00000049656
ENSG00000261115	ENSG00000179546	ENSG00000049759
ENSG00000144339	ENSG00000137727	ENSG00000049768
ENSG00000239405	ENSG00000231154	ENSG00000049769

ENSG00000135111	ENSG00000205323	ENSG00000049883
ENSG00000198075	ENSG00000259495	ENSG00000050030
ENSG00000149634	ENSG00000198153	ENSG00000050130
ENSG00000228451	ENSG00000006128	ENSG00000050165
ENSG00000225953	ENSG00000215386	ENSG00000050327
ENSG00000004139	ENSG00000114200	ENSG00000050344
ENSG00000079102	ENSG00000172748	ENSG00000050393
ENSG00000100784	ENSG00000178127	ENSG00000050405
ENSG00000177519	ENSG00000259315	ENSG00000050426
ENSG00000140986	ENSG00000138669	ENSG00000050438
ENSG00000223638	ENSG00000272345	ENSG00000050555
ENSG00000050628	ENSG00000156463	ENSG00000050628
ENSG00000150687	ENSG00000239389	ENSG00000050730
ENSG00000118557	ENSG00000166923	ENSG00000050748
ENSG00000120278	ENSG00000259663	ENSG00000050767
ENSG00000134627	ENSG00000138675	ENSG00000051009
ENSG00000262209	ENSG00000269113	ENSG00000051108
ENSG00000081853	ENSG00000113532	ENSG00000051180
ENSG00000267270	ENSG00000280426	ENSG00000051341
ENSG00000116117	ENSG00000255129	ENSG00000051382
ENSG00000180914	ENSG00000261560	ENSG00000051523
ENSG00000198156	ENSG00000141433	ENSG00000051596
ENSG00000245532	ENSG00000251664	ENSG00000052126
ENSG00000249437	ENSG00000159450	ENSG00000052344
ENSG00000255823	ENSG00000099260	ENSG00000052723
ENSG00000003987	ENSG00000156475	ENSG00000052749
ENSG00000171843	ENSG00000277449	ENSG00000052795
ENSG00000277443	ENSG00000251129	ENSG00000052802
ENSG00000253161	ENSG00000128285	ENSG00000052841
ENSG00000250584	ENSG00000067082	ENSG00000052850
ENSG00000196196	ENSG00000246465	ENSG00000053108
ENSG00000120370	ENSG00000249859	ENSG00000053254
ENSG00000143507	ENSG00000266983	ENSG00000053371
ENSG00000134256	ENSG00000217648	ENSG00000053372
ENSG00000256304	ENSG00000140527	ENSG00000053438
ENSG00000204387	ENSG00000232906	ENSG00000053524
ENSG00000105409	ENSG00000186056	ENSG00000053702
ENSG00000171681	ENSG00000108932	ENSG00000053770

ENSG00000145536	ENSG00000102174	ENSG00000053918
ENSG00000077522	ENSG00000154479	ENSG00000054116
ENSG00000160179	ENSG00000156453	ENSG00000054118
ENSG00000179978	ENSG00000230873	ENSG00000054179
ENSG00000279208	ENSG00000170961	ENSG00000054267
ENSG00000279696	ENSG00000249637	ENSG00000054356
ENSG00000261754	ENSG00000214198	ENSG00000054392
ENSG00000233045	ENSG00000169896	ENSG00000054523
ENSG00000261136	ENSG00000134207	ENSG00000054598
ENSG00000185065	ENSG00000133169	ENSG00000054611
ENSG00000261879	ENSG00000138778	ENSG00000054654
ENSG00000275481	ENSG00000236824	ENSG00000054690
ENSG00000215022	ENSG00000155090	ENSG00000054793
ENSG00000273448	ENSG00000140450	ENSG00000054796
ENSG00000283757	ENSG00000218069	ENSG00000054965
ENSG00000279456	ENSG00000140465	ENSG00000054967
ENSG00000159915	ENSG00000260774	ENSG00000054983
ENSG00000175564	ENSG00000182752	ENSG00000055070
ENSG00000196781	ENSG00000121075	ENSG00000055118
ENSG00000147606	ENSG00000231672	ENSG00000055147
ENSG00000207081	ENSG00000175197	ENSG00000055163
ENSG00000186472	ENSG00000224596	ENSG00000055332
ENSG00000146001	ENSG00000164440	ENSG00000055483
ENSG00000113248	ENSG00000104783	ENSG00000055609
ENSG00000229619	ENSG00000173406	ENSG00000055732
ENSG00000173988	ENSG00000256589	ENSG00000055917
ENSG00000049130	ENSG00000179046	ENSG00000055950
ENSG00000135116	ENSG00000249790	ENSG00000055955
ENSG00000105996	ENSG00000273419	ENSG00000056277
ENSG00000237125	ENSG00000038295	ENSG00000056291
ENSG00000164463	ENSG00000151023	ENSG00000056487
ENSG00000124749	ENSG00000165186	ENSG00000056558
ENSG00000008300	ENSG00000108924	ENSG00000056736
ENSG00000157445	ENSG00000100867	ENSG00000056972
ENSG00000260103	ENSG00000111087	ENSG00000056998
ENSG00000267419	ENSG00000164484	ENSG00000057019
ENSG00000255471	ENSG00000183571	ENSG00000057252
ENSG00000273038	ENSG00000204531	ENSG00000057294

ENSG00000236778
ENSG00000254289
ENSG00000270276
ENSG00000270882
ENSG00000274750
ENSG00000124635
ENSG00000214544
ENSG00000180447
ENSG00000130222
ENSG00000116717
ENSG00000054598
ENSG00000125848
ENSG00000102678
ENSG00000165323
ENSG00000283709
ENSG00000044524
ENSG00000128512
ENSG00000224165
ENSG00000276644
ENSG00000259736
ENSG00000215018
ENSG00000254413
ENSG00000159409
ENSG00000123080
ENSG00000164076
ENSG00000106804
ENSG00000163009
ENSG00000214212
ENSG00000143032
ENSG00000150347
ENSG00000103723
ENSG00000163126
ENSG00000215559
ENSG00000101745
ENSG00000170214
ENSG00000150471
ENSG00000185736
ENSG00000165029
ENSG00000057593
ENSG00000057608
ENSG00000057657
ENSG00000057663
ENSG00000057704
ENSG00000057757
ENSG00000057935
ENSG00000058056
ENSG00000058063
ENSG00000058085
ENSG00000058091
ENSG00000058262
ENSG00000058272
ENSG00000058335
ENSG00000058404
ENSG00000058453
ENSG00000058600
ENSG00000058668
ENSG00000058673
ENSG00000058729
ENSG00000058799
ENSG00000058804
ENSG00000058866
ENSG00000059122
ENSG00000059145
ENSG00000059377
ENSG00000059378
ENSG00000059728
ENSG00000059758
ENSG00000059769
ENSG00000059804
ENSG00000059915
ENSG00000060069
ENSG00000060138
ENSG00000060140
ENSG00000060237
ENSG00000060339
ENSG00000060491

ENSG00000272341
ENSG00000251634
ENSG00000248583
ENSG00000279522
ENSG00000232611
ENSG00000279900
ENSG00000266680
ENSG00000263080
ENSG00000251661
ENSG00000214243
ENSG00000240291
ENSG00000271133
ENSG00000258457
ENSG00000229127
ENSG00000204666
ENSG00000267370
ENSG00000263280
ENSG00000249721
ENSG00000255435
ENSG00000237732
ENSG00000249655
ENSG00000259723
ENSG00000240731
ENSG00000272030
ENSG00000237149
ENSG00000152763
ENSG00000182168
ENSG00000133710
ENSG00000134532
ENSG00000124766
ENSG00000176887
ENSG00000148942
ENSG00000139209
ENSG00000106483
ENSG00000152217
ENSG00000243819
ENSG00000268006
ENSG00000164708
ENSG00000060558
ENSG00000060566
ENSG00000060656
ENSG00000060709
ENSG00000060718
ENSG00000060749
ENSG00000060971
ENSG00000060982
ENSG00000061273
ENSG00000061337
ENSG00000061455
ENSG00000061492
ENSG00000061676
ENSG00000061794
ENSG00000061918
ENSG00000061938
ENSG00000061987
ENSG00000062038
ENSG00000062096
ENSG00000062194
ENSG00000062282
ENSG00000062370
ENSG00000062485
ENSG00000062582
ENSG00000062598
ENSG00000062650
ENSG00000062716
ENSG00000062725
ENSG00000063015
ENSG00000063046
ENSG00000063127
ENSG00000063169
ENSG00000063176
ENSG00000063241
ENSG00000063244
ENSG00000063245
ENSG00000063322
ENSG00000063438

ENSG00000049246
ENSG00000189184
ENSG00000185630
ENSG00000085465
ENSG00000099250
ENSG00000247809
ENSG00000176771
ENSG00000143995
ENSG00000130518
ENSG00000267365
ENSG00000090376
ENSG00000196890
ENSG00000187837
ENSG00000134363
ENSG00000168386
ENSG00000196159
ENSG00000116106
ENSG00000242689
ENSG00000240498
ENSG00000129757
ENSG00000150394
ENSG00000272398
ENSG00000234585
ENSG00000133101
ENSG00000179397
ENSG00000167995
ENSG00000162373
ENSG00000258655
ENSG00000259456
ENSG00000270605
ENSG00000279865
ENSG00000255363
ENSG00000236654
ENSG00000250753
ENSG00000273759
ENSG00000214432
ENSG00000279659
ENSG00000272054
ENSG00000063587
ENSG00000063601
ENSG00000063660
ENSG00000063761
ENSG00000063854
ENSG00000063978
ENSG00000064042
ENSG00000064102
ENSG00000064115
ENSG00000064195
ENSG00000064199
ENSG00000064201
ENSG00000064205
ENSG00000064218
ENSG00000064225
ENSG00000064300
ENSG00000064309
ENSG00000064313
ENSG00000064393
ENSG00000064419
ENSG00000064545
ENSG00000064547
ENSG00000064601
ENSG00000064607
ENSG00000064651
ENSG00000064652
ENSG00000064655
ENSG00000064666
ENSG00000064687
ENSG00000064692
ENSG00000064726
ENSG00000064763
ENSG00000064961
ENSG00000064989
ENSG00000064995
ENSG00000064999
ENSG00000065029
ENSG00000065054

ENSG00000280010
ENSG00000026025
ENSG00000181449
ENSG00000198732
ENSG00000149212
ENSG00000257557
ENSG00000170836
ENSG00000151623
ENSG00000237187
ENSG00000165555
ENSG00000169184
ENSG00000255248
ENSG00000183496
ENSG00000233639
ENSG00000123700
ENSG00000233822
ENSG00000236993
ENSG00000128573
ENSG00000228314
ENSG00000183230
ENSG00000168542
ENSG00000213760
ENSG00000261716
ENSG00000251095
ENSG00000220323
ENSG00000203819
ENSG00000270681
ENSG00000257261
ENSG00000230699
ENSG00000234537
ENSG00000056487
ENSG00000262096
ENSG00000181143
ENSG00000233930
ENSG00000099251
ENSG00000180596
ENSG00000265962
ENSG00000107105
ENSG00000065060
ENSG00000065135
ENSG00000065150
ENSG00000065183
ENSG00000065243
ENSG00000065308
ENSG00000065320
ENSG00000065325
ENSG00000065328
ENSG00000065357
ENSG00000065361
ENSG00000065457
ENSG00000065491
ENSG00000065518
ENSG00000065526
ENSG00000065534
ENSG00000065559
ENSG00000065600
ENSG00000065609
ENSG00000065613
ENSG00000065615
ENSG00000065618
ENSG00000065665
ENSG00000065675
ENSG00000065717
ENSG00000065802
ENSG00000065809
ENSG00000065833
ENSG00000065882
ENSG00000065883
ENSG00000065911
ENSG00000065923
ENSG00000065970
ENSG00000065978
ENSG00000065989
ENSG00000066027
ENSG00000066032
ENSG00000066044

ENSG00000164309
ENSG00000095585
ENSG00000141431
ENSG00000154736
ENSG00000272993
ENSG00000229931
ENSG00000267731
ENSG00000248596
ENSG00000165655
ENSG00000265972
ENSG00000092421
ENSG00000253846
ENSG00000188729
ENSG00000134986
ENSG00000015520
ENSG00000138347
ENSG00000144214
ENSG00000181016
ENSG00000148798
ENSG00000164330
ENSG00000158486
ENSG00000082196
ENSG00000213199
ENSG00000274460
ENSG00000271474
ENSG00000225792
ENSG00000255389
ENSG00000272468
ENSG00000139865
ENSG00000019549
ENSG00000115896
ENSG00000169116
ENSG00000175745
ENSG00000078018
ENSG00000203814
ENSG00000184678
ENSG00000196787
ENSG00000174844

ENSG00000066084
ENSG00000066117
ENSG00000066135
ENSG00000066136
ENSG00000066185
ENSG00000066230
ENSG00000066248
ENSG00000066294
ENSG00000066322
ENSG00000066382
ENSG00000066405
ENSG00000066422
ENSG00000066427
ENSG00000066468
ENSG00000066557
ENSG00000066583
ENSG00000066629
ENSG00000066654
ENSG00000066697
ENSG00000066735
ENSG00000066739
ENSG00000066777
ENSG00000066813
ENSG00000066827
ENSG00000066855
ENSG00000066923
ENSG00000066926
ENSG00000066933
ENSG00000067048
ENSG00000067057
ENSG00000067064
ENSG00000067066
ENSG00000067082
ENSG00000067113
ENSG00000067141
ENSG00000067167
ENSG00000067177
ENSG00000067182

ENSG00000135407
ENSG00000186517
ENSG00000270876
ENSG00000137573
ENSG00000070731
ENSG00000178573
ENSG00000180573
ENSG00000087589
ENSG00000224294
ENSG00000278238
ENSG00000250519
ENSG00000284526
ENSG00000283235
ENSG00000272486
ENSG00000260727
ENSG00000075223
ENSG00000113389
ENSG00000163121
ENSG00000182308
ENSG00000145087
ENSG00000188738
ENSG00000164972
ENSG00000261087
ENSG00000143469
ENSG00000257335
ENSG00000158406
ENSG00000158373
ENSG00000132837
ENSG00000276966
ENSG00000274290
ENSG00000188959
ENSG00000268756
ENSG00000169432
ENSG00000237693
ENSG00000159208
ENSG00000115461
ENSG00000171587
ENSG00000187775
ENSG00000067191
ENSG00000067208
ENSG00000067221
ENSG00000067225
ENSG00000067369
ENSG00000067445
ENSG00000067533
ENSG00000067560
ENSG00000067596
ENSG00000067606
ENSG00000067646
ENSG00000067715
ENSG00000067798
ENSG00000067836
ENSG00000067840
ENSG00000067842
ENSG00000067955
ENSG00000067992
ENSG00000068001
ENSG00000068024
ENSG00000068028
ENSG00000068078
ENSG00000068097
ENSG00000068305
ENSG00000068308
ENSG00000068323
ENSG00000068354
ENSG00000068366
ENSG00000068383
ENSG00000068394
ENSG00000068400
ENSG00000068489
ENSG00000068615
ENSG00000068650
ENSG00000068654
ENSG00000068697
ENSG00000068724
ENSG00000068745

ENSG00000113369
ENSG00000166793
ENSG00000188580
ENSG00000273802
ENSG00000233030
ENSG00000171951
ENSG00000126368
ENSG00000139269
ENSG00000183813
ENSG00000234424
ENSG00000015592
ENSG00000132639
ENSG00000256779
ENSG00000144188
ENSG00000144010
ENSG00000204510
ENSG00000284546
ENSG00000144015
ENSG00000284306
ENSG00000250782
ENSG00000182330
ENSG00000277862
ENSG00000283873
ENSG00000284018
ENSG00000229978
ENSG00000283776
ENSG00000235268
ENSG00000250386
ENSG00000283740
ENSG00000236175
ENSG00000189325
ENSG00000150244
ENSG00000284438
ENSG00000204449
ENSG00000168930
ENSG00000157765
ENSG00000249620
ENSG00000233802
ENSG00000068781
ENSG00000068796
ENSG00000068878
ENSG00000068885
ENSG00000068903
ENSG00000068912
ENSG00000068971
ENSG00000069020
ENSG00000069122
ENSG00000069188
ENSG00000069248
ENSG00000069275
ENSG00000069329
ENSG00000069345
ENSG00000069399
ENSG00000069424
ENSG00000069493
ENSG00000069667
ENSG00000069696
ENSG00000069702
ENSG00000069812
ENSG00000069869
ENSG00000069956
ENSG00000069966
ENSG00000069974
ENSG00000069998
ENSG00000070010
ENSG00000070018
ENSG00000070019
ENSG00000070061
ENSG00000070081
ENSG00000070087
ENSG00000070159
ENSG00000070182
ENSG00000070190
ENSG00000070214
ENSG00000070269
ENSG00000070366

ENSG00000223417
ENSG00000237706
ENSG00000251258
ENSG00000204455
ENSG00000269466
ENSG00000249156
ENSG00000257951
ENSG00000189253
ENSG00000225581
ENSG00000180532
ENSG00000116726
ENSG00000166013
ENSG00000204450
ENSG00000254764
ENSG00000182053
ENSG00000268799
ENSG00000106278
ENSG00000236941
ENSG00000255855

ENSG00000070367
ENSG00000070371
ENSG00000070404
ENSG00000070413
ENSG00000070423
ENSG00000070444
ENSG00000070476
ENSG00000070495
ENSG00000070540
ENSG00000070610
ENSG00000070614
ENSG00000070718
ENSG00000070729
ENSG00000070731
ENSG00000070759
ENSG00000070778
ENSG00000070785
ENSG00000070808
ENSG00000070814
ENSG00000070831
ENSG00000070882
ENSG00000070886
ENSG00000070915
ENSG00000070950
ENSG00000070961
ENSG00000070985
ENSG00000071051
ENSG00000071054
ENSG00000071073
ENSG00000071082
ENSG00000071127
ENSG00000071189
ENSG00000071205
ENSG00000071242
ENSG00000071243
ENSG00000071246
ENSG00000071282
ENSG00000071462

ENSG00000071537
ENSG00000071539
ENSG00000071553
ENSG00000071564
ENSG00000071575
ENSG00000071626
ENSG00000071655
ENSG00000071794
ENSG00000071859
ENSG00000071889
ENSG00000071909
ENSG00000071967
ENSG00000071991
ENSG00000071994
ENSG00000072041
ENSG00000072042
ENSG00000072062
ENSG00000072071
ENSG00000072110
ENSG00000072121
ENSG00000072133
ENSG00000072134
ENSG00000072135
ENSG00000072163
ENSG00000072182
ENSG00000072195
ENSG00000072201
ENSG00000072210
ENSG00000072274
ENSG00000072310
ENSG00000072315
ENSG00000072364
ENSG00000072401
ENSG00000072415
ENSG00000072422
ENSG00000072501
ENSG00000072518
ENSG00000072571

ENSG00000072609
ENSG00000072657
ENSG00000072682
ENSG00000072694
ENSG00000072736
ENSG00000072778
ENSG00000072786
ENSG00000072803
ENSG00000072832
ENSG00000072840
ENSG00000072849
ENSG00000072858
ENSG00000072864
ENSG00000072952
ENSG00000072954
ENSG00000072958
ENSG00000073008
ENSG00000073060
ENSG00000073067
ENSG00000073150
ENSG00000073282
ENSG00000073331
ENSG00000073350
ENSG00000073417
ENSG00000073464
ENSG00000073536
ENSG00000073578
ENSG00000073584
ENSG00000073605
ENSG00000073614
ENSG00000073670
ENSG00000073711
ENSG00000073734
ENSG00000073754
ENSG00000073792
ENSG00000073803
ENSG00000073849
ENSG00000073861

ENSG00000073910
ENSG00000073921
ENSG00000073969
ENSG00000074047
ENSG00000074054
ENSG00000074071
ENSG00000074181
ENSG00000074201
ENSG00000074211
ENSG00000074219
ENSG00000074266
ENSG00000074317
ENSG00000074356
ENSG00000074370
ENSG00000074410
ENSG00000074416
ENSG00000074527
ENSG00000074590
ENSG00000074603
ENSG00000074621
ENSG00000074657
ENSG00000074660
ENSG00000074695
ENSG00000074696
ENSG00000074706
ENSG00000074755
ENSG00000074803
ENSG00000074842
ENSG00000074964
ENSG00000074966
ENSG00000075035
ENSG00000075043
ENSG00000075073
ENSG00000075089
ENSG00000075131
ENSG00000075142
ENSG00000075151
ENSG00000075213

ENSG00000075223
ENSG00000075234
ENSG00000075239
ENSG00000075240
ENSG00000075275
ENSG00000075290
ENSG00000075292
ENSG00000075303
ENSG00000075336
ENSG00000075340
ENSG00000075388
ENSG00000075391
ENSG00000075399
ENSG00000075407
ENSG00000075420
ENSG00000075426
ENSG00000075461
ENSG00000075539
ENSG00000075618
ENSG00000075624
ENSG00000075651
ENSG00000075711
ENSG00000075785
ENSG00000075790
ENSG00000075826
ENSG00000075856
ENSG00000075891
ENSG00000075975
ENSG00000076053
ENSG00000076067
ENSG00000076108
ENSG00000076248
ENSG00000076258
ENSG00000076321
ENSG00000076344
ENSG00000076351
ENSG00000076356
ENSG00000076382

ENSG00000076513
ENSG00000076554
ENSG00000076555
ENSG00000076604
ENSG00000076641
ENSG00000076685
ENSG00000076706
ENSG00000076716
ENSG00000076770
ENSG00000076826
ENSG00000076864
ENSG00000076928
ENSG00000076984
ENSG00000077044
ENSG00000077092
ENSG00000077147
ENSG00000077150
ENSG00000077157
ENSG00000077232
ENSG00000077238
ENSG00000077254
ENSG00000077264
ENSG00000077274
ENSG00000077279
ENSG00000077312
ENSG00000077327
ENSG00000077348
ENSG00000077380
ENSG00000077420
ENSG00000077454
ENSG00000077458
ENSG00000077463
ENSG00000077514
ENSG00000077522
ENSG00000077585
ENSG00000077684
ENSG00000077713
ENSG00000077721

ENSG00000077782
ENSG00000077800
ENSG00000077935
ENSG00000077942
ENSG00000077943
ENSG00000077984
ENSG00000078018
ENSG00000078043
ENSG00000078053
ENSG00000078061
ENSG00000078070
ENSG00000078081
ENSG00000078098
ENSG00000078114
ENSG00000078124
ENSG00000078140
ENSG00000078177
ENSG00000078237
ENSG00000078246
ENSG00000078269
ENSG00000078295
ENSG00000078304
ENSG00000078328
ENSG00000078369
ENSG00000078399
ENSG00000078401
ENSG00000078403
ENSG00000078487
ENSG00000078549
ENSG00000078589
ENSG00000078668
ENSG00000078674
ENSG00000078687
ENSG00000078699
ENSG00000078725
ENSG00000078747
ENSG00000078795
ENSG00000078804

ENSG00000078808
ENSG00000078814
ENSG00000078900
ENSG00000078902
ENSG00000078967
ENSG00000079102
ENSG00000079112
ENSG00000079134
ENSG00000079150
ENSG00000079156
ENSG00000079215
ENSG00000079246
ENSG00000079263
ENSG00000079277
ENSG00000079308
ENSG00000079313
ENSG00000079332
ENSG00000079335
ENSG00000079337
ENSG00000079385
ENSG00000079387
ENSG00000079393
ENSG00000079432
ENSG00000079435
ENSG00000079459
ENSG00000079482
ENSG00000079557
ENSG00000079689
ENSG00000079691
ENSG00000079739
ENSG00000079805
ENSG00000079819
ENSG00000079841
ENSG00000079931
ENSG00000079950
ENSG00000079974
ENSG00000079999
ENSG00000080166

ENSG00000080189
ENSG00000080200
ENSG00000080224
ENSG00000080293
ENSG00000080298
ENSG00000080345
ENSG00000080371
ENSG00000080493
ENSG00000080503
ENSG00000080511
ENSG00000080546
ENSG00000080561
ENSG00000080572
ENSG00000080573
ENSG00000080603
ENSG00000080644
ENSG00000080802
ENSG00000080815
ENSG00000080819
ENSG00000080822
ENSG00000080824
ENSG00000080839
ENSG00000080845
ENSG00000080854
ENSG00000081014
ENSG00000081019
ENSG00000081026
ENSG00000081041
ENSG00000081052
ENSG00000081059
ENSG00000081087
ENSG00000081138
ENSG00000081148
ENSG00000081154
ENSG00000081177
ENSG00000081181
ENSG00000081189
ENSG00000081248

ENSG00000081277
ENSG00000081307
ENSG00000081377
ENSG00000081386
ENSG00000081479
ENSG00000081665
ENSG00000081692
ENSG00000081760
ENSG00000081791
ENSG00000081800
ENSG00000081803
ENSG00000081818
ENSG00000081842
ENSG00000081853
ENSG00000081913
ENSG00000081923
ENSG00000081985
ENSG00000082074
ENSG00000082146
ENSG00000082153
ENSG00000082175
ENSG00000082196
ENSG00000082212
ENSG00000082213
ENSG00000082258
ENSG00000082269
ENSG00000082293
ENSG00000082397
ENSG00000082438
ENSG00000082458
ENSG00000082482
ENSG00000082497
ENSG00000082512
ENSG00000082515
ENSG00000082516
ENSG00000082556
ENSG00000082641
ENSG00000082684

ENSG00000082701
ENSG00000082781
ENSG00000082805
ENSG00000082898
ENSG00000082996
ENSG00000083067
ENSG00000083099
ENSG00000083123
ENSG00000083168
ENSG00000083223
ENSG00000083290
ENSG00000083307
ENSG00000083312
ENSG00000083444
ENSG00000083454
ENSG00000083520
ENSG00000083635
ENSG00000083642
ENSG00000083720
ENSG00000083799
ENSG00000083812
ENSG00000083814
ENSG00000083817
ENSG00000083828
ENSG00000083838
ENSG00000083844
ENSG00000083857
ENSG00000083896
ENSG00000084070
ENSG00000084072
ENSG00000084073
ENSG00000084090
ENSG00000084093
ENSG00000084110
ENSG00000084112
ENSG00000084207
ENSG00000084234
ENSG00000084444

ENSG00000084453
ENSG00000084463
ENSG00000084623
ENSG00000084628
ENSG00000084652
ENSG00000084674
ENSG00000084676
ENSG00000084710
ENSG00000084731
ENSG00000084733
ENSG00000084754
ENSG00000084764
ENSG00000084774
ENSG00000085063
ENSG00000085117
ENSG00000085185
ENSG00000085224
ENSG00000085231
ENSG00000085274
ENSG00000085276
ENSG00000085365
ENSG00000085377
ENSG00000085382
ENSG00000085415
ENSG00000085433
ENSG00000085491
ENSG00000085511
ENSG00000085644
ENSG00000085662
ENSG00000085719
ENSG00000085721
ENSG00000085733
ENSG00000085741
ENSG00000085760
ENSG00000085831
ENSG00000085832
ENSG00000085871
ENSG00000085872

ENSG00000085978
ENSG00000085982
ENSG00000085998
ENSG00000085999
ENSG00000086061
ENSG00000086062
ENSG00000086065
ENSG00000086102
ENSG00000086159
ENSG00000086189
ENSG00000086200
ENSG00000086232
ENSG00000086289
ENSG00000086300
ENSG00000086475
ENSG00000086544
ENSG00000086548
ENSG00000086570
ENSG00000086589
ENSG00000086598
ENSG00000086619
ENSG00000086666
ENSG00000086712
ENSG00000086717
ENSG00000086730
ENSG00000086758
ENSG00000086827
ENSG00000086848
ENSG00000086967
ENSG00000086991
ENSG00000087008
ENSG00000087053
ENSG00000087076
ENSG00000087085
ENSG00000087086
ENSG00000087088
ENSG00000087095
ENSG00000087111

ENSG00000087116
ENSG00000087128
ENSG00000087152
ENSG00000087206
ENSG00000087245
ENSG00000087250
ENSG00000087253
ENSG00000087258
ENSG00000087263
ENSG00000087266
ENSG00000087269
ENSG00000087274
ENSG00000087299
ENSG00000087301
ENSG00000087302
ENSG00000087303
ENSG00000087338
ENSG00000087448
ENSG00000087460
ENSG00000087470
ENSG00000087494
ENSG00000087502
ENSG00000087510
ENSG00000087586
ENSG00000087589
ENSG00000087842
ENSG00000087903
ENSG00000087995
ENSG00000088035
ENSG00000088038
ENSG00000088053
ENSG00000088179
ENSG00000088205
ENSG00000088256
ENSG00000088280
ENSG00000088305
ENSG00000088356
ENSG00000088367

ENSG00000088386
ENSG00000088387
ENSG00000088448
ENSG00000088538
ENSG00000088543
ENSG00000088726
ENSG00000088756
ENSG00000088766
ENSG00000088782
ENSG00000088808
ENSG00000088812
ENSG00000088826
ENSG00000088827
ENSG00000088832
ENSG00000088833
ENSG00000088836
ENSG00000088854
ENSG00000088876
ENSG00000088881
ENSG00000088888
ENSG00000088899
ENSG00000088926
ENSG00000089006
ENSG00000089012
ENSG00000089041
ENSG00000089050
ENSG00000089057
ENSG00000089060
ENSG00000089063
ENSG00000089094
ENSG00000089101
ENSG00000089116
ENSG00000089123
ENSG00000089154
ENSG00000089159
ENSG00000089163
ENSG00000089169
ENSG00000089177

ENSG00000089220
ENSG00000089225
ENSG00000089234
ENSG00000089248
ENSG00000089250
ENSG00000089280
ENSG00000089327
ENSG00000089335
ENSG00000089351
ENSG00000089356
ENSG00000089472
ENSG00000089486
ENSG00000089558
ENSG00000089597
ENSG00000089639
ENSG00000089682
ENSG00000089685
ENSG00000089693
ENSG00000089723
ENSG00000089737
ENSG00000089775
ENSG00000089818
ENSG00000089820
ENSG00000089902
ENSG00000089916
ENSG00000090013
ENSG00000090020
ENSG00000090054
ENSG00000090060
ENSG00000090061
ENSG00000090097
ENSG00000090104
ENSG00000090238
ENSG00000090263
ENSG00000090273
ENSG00000090316
ENSG00000090339
ENSG00000090372

ENSG00000090376
ENSG00000090382
ENSG00000090432
ENSG00000090447
ENSG00000090470
ENSG00000090487
ENSG00000090512
ENSG00000090520
ENSG00000090530
ENSG00000090534
ENSG00000090554
ENSG00000090565
ENSG00000090581
ENSG00000090612
ENSG00000090615
ENSG00000090659
ENSG00000090661
ENSG00000090674
ENSG00000090686
ENSG00000090776
ENSG00000090857
ENSG00000090863
ENSG00000090889
ENSG00000090924
ENSG00000090932
ENSG00000090971
ENSG00000090975
ENSG00000090989
ENSG00000091073
ENSG00000091127
ENSG00000091129
ENSG00000091137
ENSG00000091138
ENSG00000091140
ENSG00000091164
ENSG00000091181
ENSG00000091262
ENSG00000091317

ENSG00000091409
ENSG00000091428
ENSG00000091436
ENSG00000091482
ENSG00000091490
ENSG00000091513
ENSG00000091527
ENSG00000091536
ENSG00000091542
ENSG00000091592
ENSG00000091622
ENSG00000091640
ENSG00000091651
ENSG00000091732
ENSG00000091831
ENSG00000091844
ENSG00000091879
ENSG00000091947
ENSG00000091972
ENSG00000091986
ENSG00000092009
ENSG00000092010
ENSG00000092036
ENSG00000092051
ENSG00000092054
ENSG00000092068
ENSG00000092140
ENSG00000092199
ENSG00000092201
ENSG00000092203
ENSG00000092208
ENSG00000092295
ENSG00000092345
ENSG00000092421
ENSG00000092439
ENSG00000092445
ENSG00000092470
ENSG00000092529

ENSG00000092531
ENSG00000092607
ENSG00000092820
ENSG00000092841
ENSG00000092847
ENSG00000092853
ENSG00000092871
ENSG00000092929
ENSG00000092931
ENSG00000092964
ENSG00000092969
ENSG00000092978
ENSG00000093000
ENSG00000093072
ENSG00000093144
ENSG00000093167
ENSG00000093183
ENSG00000093217
ENSG00000094631
ENSG00000094755
ENSG00000094841
ENSG00000094880
ENSG00000094916
ENSG00000094963
ENSG00000094975
ENSG00000095015
ENSG00000095066
ENSG00000095110
ENSG00000095139
ENSG00000095203
ENSG00000095209
ENSG00000095261
ENSG00000095303
ENSG00000095319
ENSG00000095321
ENSG00000095383
ENSG00000095397
ENSG00000095485

ENSG00000095539
ENSG00000095574
ENSG00000095587
ENSG00000095627
ENSG00000095637
ENSG00000095713
ENSG00000095739
ENSG00000095752
ENSG00000095777
ENSG00000095787
ENSG00000095794
ENSG00000095906
ENSG00000095981
ENSG00000096006
ENSG00000096060
ENSG00000096063
ENSG00000096070
ENSG00000096080
ENSG00000096088
ENSG00000096093
ENSG00000096384
ENSG00000096395
ENSG00000096401
ENSG00000096433
ENSG00000096654
ENSG00000096696
ENSG00000096717
ENSG00000096746
ENSG00000096968
ENSG00000096996
ENSG00000097007
ENSG00000097021
ENSG00000097033
ENSG00000097046
ENSG00000097096
ENSG00000099139
ENSG00000099194
ENSG00000099203

ENSG00000099204
ENSG00000099219
ENSG00000099246
ENSG00000099250
ENSG00000099256
ENSG00000099282
ENSG00000099284
ENSG00000099290
ENSG00000099308
ENSG00000099326
ENSG00000099331
ENSG00000099337
ENSG00000099364
ENSG00000099365
ENSG00000099377
ENSG00000099381
ENSG00000099385
ENSG00000099399
ENSG00000099617
ENSG00000099622
ENSG00000099624
ENSG00000099625
ENSG00000099715
ENSG00000099783
ENSG00000099785
ENSG00000099800
ENSG00000099804
ENSG00000099810
ENSG00000099812
ENSG00000099814
ENSG00000099817
ENSG00000099834
ENSG00000099840
ENSG00000099849
ENSG00000099860
ENSG00000099864
ENSG00000099866
ENSG00000099875

ENSG00000099889
ENSG00000099899
ENSG00000099901
ENSG00000099904
ENSG00000099910
ENSG00000099917
ENSG00000099937
ENSG00000099940
ENSG00000099942
ENSG00000099949
ENSG00000099953
ENSG00000099954
ENSG00000099957
ENSG00000099958
ENSG00000099960
ENSG00000099968
ENSG00000099974
ENSG00000099977
ENSG00000099985
ENSG00000099992
ENSG00000099995
ENSG00000099999
ENSG00000100003
ENSG00000100012
ENSG00000100014
ENSG00000100023
ENSG00000100024
ENSG00000100027
ENSG00000100028
ENSG00000100029
ENSG00000100030
ENSG00000100031
ENSG00000100033
ENSG00000100034
ENSG00000100036
ENSG00000100038
ENSG00000100053
ENSG00000100055

ENSG00000100056
ENSG00000100060
ENSG00000100065
ENSG00000100068
ENSG00000100075
ENSG00000100077
ENSG00000100078
ENSG00000100083
ENSG00000100084
ENSG00000100095
ENSG00000100099
ENSG00000100100
ENSG00000100105
ENSG00000100106
ENSG00000100109
ENSG00000100116
ENSG00000100121
ENSG00000100124
ENSG00000100129
ENSG00000100138
ENSG00000100139
ENSG00000100142
ENSG00000100146
ENSG00000100147
ENSG00000100150
ENSG00000100151
ENSG00000100154
ENSG00000100156
ENSG00000100162
ENSG00000100167
ENSG00000100170
ENSG00000100196
ENSG00000100197
ENSG00000100201
ENSG00000100206
ENSG00000100207
ENSG00000100209
ENSG00000100211

ENSG00000100216
ENSG00000100219
ENSG00000100220
ENSG00000100221
ENSG00000100225
ENSG00000100226
ENSG00000100227
ENSG00000100228
ENSG00000100234
ENSG00000100241
ENSG00000100242
ENSG00000100243
ENSG00000100246
ENSG00000100258
ENSG00000100263
ENSG00000100266
ENSG00000100276
ENSG00000100280
ENSG00000100281
ENSG00000100285
ENSG00000100290
ENSG00000100292
ENSG00000100294
ENSG00000100296
ENSG00000100297
ENSG00000100298
ENSG00000100299
ENSG00000100302
ENSG00000100304
ENSG00000100307
ENSG00000100311
ENSG00000100314
ENSG00000100320
ENSG00000100321
ENSG00000100324
ENSG00000100325
ENSG00000100330
ENSG00000100335

ENSG00000100336
ENSG00000100341
ENSG00000100342
ENSG00000100344
ENSG00000100345
ENSG00000100346
ENSG00000100348
ENSG00000100350
ENSG00000100351
ENSG00000100353
ENSG00000100354
ENSG00000100362
ENSG00000100364
ENSG00000100365
ENSG00000100368
ENSG00000100372
ENSG00000100373
ENSG00000100376
ENSG00000100379
ENSG00000100380
ENSG00000100385
ENSG00000100393
ENSG00000100395
ENSG00000100399
ENSG00000100401
ENSG00000100403
ENSG00000100410
ENSG00000100413
ENSG00000100417
ENSG00000100418
ENSG00000100422
ENSG00000100425
ENSG00000100427
ENSG00000100433
ENSG00000100439
ENSG00000100441
ENSG00000100442
ENSG00000100445

ENSG00000100450
ENSG00000100453
ENSG00000100461
ENSG00000100462
ENSG00000100478
ENSG00000100483
ENSG00000100485
ENSG00000100490
ENSG00000100503
ENSG00000100505
ENSG00000100522
ENSG00000100523
ENSG00000100528
ENSG00000100554
ENSG00000100557
ENSG00000100558
ENSG00000100564
ENSG00000100568
ENSG00000100577
ENSG00000100578
ENSG00000100592
ENSG00000100593
ENSG00000100596
ENSG00000100599
ENSG00000100600
ENSG00000100601
ENSG00000100603
ENSG00000100604
ENSG00000100605
ENSG00000100612
ENSG00000100614
ENSG00000100625
ENSG00000100626
ENSG00000100628
ENSG00000100629
ENSG00000100644
ENSG00000100647
ENSG00000100650

ENSG00000100664
ENSG00000100665
ENSG00000100678
ENSG00000100697
ENSG00000100711
ENSG00000100721
ENSG00000100722
ENSG00000100726
ENSG00000100731
ENSG00000100744
ENSG00000100767
ENSG00000100784
ENSG00000100796
ENSG00000100802
ENSG00000100804
ENSG00000100811
ENSG00000100813
ENSG00000100815
ENSG00000100836
ENSG00000100842
ENSG00000100852
ENSG00000100865
ENSG00000100867
ENSG00000100884
ENSG00000100888
ENSG00000100889
ENSG00000100890
ENSG00000100897
ENSG00000100906
ENSG00000100916
ENSG00000100926
ENSG00000100938
ENSG00000100941
ENSG00000100949
ENSG00000100968
ENSG00000100983
ENSG00000100987
ENSG00000100991

ENSG00000100994
ENSG00000100997
ENSG00000101003
ENSG00000101004
ENSG00000101017
ENSG00000101019
ENSG00000101040
ENSG00000101049
ENSG00000101057
ENSG00000101076
ENSG00000101079
ENSG00000101082
ENSG00000101084
ENSG00000101096
ENSG00000101098
ENSG00000101109
ENSG00000101115
ENSG00000101126
ENSG00000101132
ENSG00000101134
ENSG00000101138
ENSG00000101144
ENSG00000101146
ENSG00000101150
ENSG00000101152
ENSG00000101158
ENSG00000101162
ENSG00000101166
ENSG00000101180
ENSG00000101181
ENSG00000101187
ENSG00000101188
ENSG00000101189
ENSG00000101191
ENSG00000101193
ENSG00000101194
ENSG00000101199
ENSG00000101203

ENSG00000101204
ENSG00000101213
ENSG00000101216
ENSG00000101220
ENSG00000101224
ENSG00000101230
ENSG00000101236
ENSG00000101246
ENSG00000101247
ENSG00000101251
ENSG00000101255
ENSG00000101265
ENSG00000101266
ENSG00000101276
ENSG00000101280
ENSG00000101282
ENSG00000101290
ENSG00000101292
ENSG00000101294
ENSG00000101298
ENSG00000101306
ENSG00000101307
ENSG00000101310
ENSG00000101311
ENSG00000101323
ENSG00000101327
ENSG00000101331
ENSG00000101333
ENSG00000101335
ENSG00000101337
ENSG00000101342
ENSG00000101343
ENSG00000101346
ENSG00000101347
ENSG00000101350
ENSG00000101353
ENSG00000101361
ENSG00000101363

ENSG00000101365
ENSG00000101367
ENSG00000101384
ENSG00000101391
ENSG00000101412
ENSG00000101413
ENSG00000101417
ENSG00000101421
ENSG00000101438
ENSG00000101439
ENSG00000101440
ENSG00000101441
ENSG00000101442
ENSG00000101443
ENSG00000101444
ENSG00000101445
ENSG00000101446
ENSG00000101448
ENSG00000101452
ENSG00000101457
ENSG00000101460
ENSG00000101463
ENSG00000101464
ENSG00000101470
ENSG00000101474
ENSG00000101489
ENSG00000101493
ENSG00000101542
ENSG00000101544
ENSG00000101546
ENSG00000101557
ENSG00000101558
ENSG00000101574
ENSG00000101577
ENSG00000101624
ENSG00000101638
ENSG00000101654
ENSG00000101665

ENSG00000101670
ENSG00000101680
ENSG00000101695
ENSG00000101745
ENSG00000101746
ENSG00000101751
ENSG00000101752
ENSG00000101773
ENSG00000101782
ENSG00000101811
ENSG00000101812
ENSG00000101825
ENSG00000101842
ENSG00000101844
ENSG00000101846
ENSG00000101849
ENSG00000101850
ENSG00000101868
ENSG00000101871
ENSG00000101888
ENSG00000101892
ENSG00000101901
ENSG00000101911
ENSG00000101916
ENSG00000101928
ENSG00000101935
ENSG00000101938
ENSG00000101945
ENSG00000101951
ENSG00000101958
ENSG00000101966
ENSG00000101972
ENSG00000101974
ENSG00000101981
ENSG00000101986
ENSG00000102003
ENSG00000102007
ENSG00000102021

ENSG00000102024
ENSG00000102030
ENSG00000102034
ENSG00000102043
ENSG00000102048
ENSG00000102053
ENSG00000102054
ENSG00000102076
ENSG00000102078
ENSG00000102081
ENSG00000102096
ENSG00000102098
ENSG00000102100
ENSG00000102104
ENSG00000102119
ENSG00000102125
ENSG00000102144
ENSG00000102145
ENSG00000102158
ENSG00000102172
ENSG00000102174
ENSG00000102178
ENSG00000102181
ENSG00000102189
ENSG00000102218
ENSG00000102221
ENSG00000102225
ENSG00000102226
ENSG00000102230
ENSG00000102245
ENSG00000102271
ENSG00000102287
ENSG00000102290
ENSG00000102309
ENSG00000102312
ENSG00000102313
ENSG00000102316
ENSG00000102317

ENSG00000102349
ENSG00000102359
ENSG00000102362
ENSG00000102383
ENSG00000102384
ENSG00000102385
ENSG00000102387
ENSG00000102401
ENSG00000102409
ENSG00000102445
ENSG00000102452
ENSG00000102466
ENSG00000102468
ENSG00000102524
ENSG00000102531
ENSG00000102543
ENSG00000102547
ENSG00000102554
ENSG00000102572
ENSG00000102580
ENSG00000102606
ENSG00000102678
ENSG00000102683
ENSG00000102710
ENSG00000102743
ENSG00000102753
ENSG00000102755
ENSG00000102760
ENSG00000102763
ENSG00000102780
ENSG00000102781
ENSG00000102786
ENSG00000102794
ENSG00000102796
ENSG00000102802
ENSG00000102804
ENSG00000102837
ENSG00000102858

ENSG00000102870
ENSG00000102871
ENSG00000102878
ENSG00000102882
ENSG00000102893
ENSG00000102897
ENSG00000102898
ENSG00000102908
ENSG00000102910
ENSG00000102921
ENSG00000102924
ENSG00000102931
ENSG00000102934
ENSG00000102935
ENSG00000102962
ENSG00000102967
ENSG00000102970
ENSG00000102974
ENSG00000102977
ENSG00000102978
ENSG00000102984
ENSG00000102996
ENSG00000103005
ENSG00000103018
ENSG00000103021
ENSG00000103024
ENSG00000103034
ENSG00000103035
ENSG00000103042
ENSG00000103044
ENSG00000103047
ENSG00000103051
ENSG00000103056
ENSG00000103061
ENSG00000103064
ENSG00000103066
ENSG00000103067
ENSG00000103089

ENSG00000103091
ENSG00000103111
ENSG00000103121
ENSG00000103126
ENSG00000103145
ENSG00000103148
ENSG00000103150
ENSG00000103154
ENSG00000103160
ENSG00000103168
ENSG00000103174
ENSG00000103175
ENSG00000103184
ENSG00000103187
ENSG00000103194
ENSG00000103196
ENSG00000103199
ENSG00000103202
ENSG00000103222
ENSG00000103226
ENSG00000103241
ENSG00000103245
ENSG00000103248
ENSG00000103253
ENSG00000103257
ENSG00000103260
ENSG00000103264
ENSG00000103266
ENSG00000103269
ENSG00000103274
ENSG00000103275
ENSG00000103313
ENSG00000103319
ENSG00000103326
ENSG00000103342
ENSG00000103343
ENSG00000103351
ENSG00000103353

ENSG00000103355
ENSG00000103356
ENSG00000103363
ENSG00000103365
ENSG00000103375
ENSG00000103381
ENSG00000103404
ENSG00000103415
ENSG00000103423
ENSG00000103449
ENSG00000103460
ENSG00000103479
ENSG00000103485
ENSG00000103489
ENSG00000103494
ENSG00000103495
ENSG00000103496
ENSG00000103502
ENSG00000103507
ENSG00000103510
ENSG00000103512
ENSG00000103522
ENSG00000103528
ENSG00000103534
ENSG00000103540
ENSG00000103546
ENSG00000103549
ENSG00000103550
ENSG00000103569
ENSG00000103591
ENSG00000103599
ENSG00000103642
ENSG00000103647
ENSG00000103653
ENSG00000103657
ENSG00000103707
ENSG00000103710
ENSG00000103723

ENSG00000103740
ENSG00000103742
ENSG00000103769
ENSG00000103852
ENSG00000103855
ENSG00000103876
ENSG00000103888
ENSG00000103932
ENSG00000103942
ENSG00000103966
ENSG00000103978
ENSG00000103994
ENSG00000103995
ENSG00000104043
ENSG00000104044
ENSG00000104047
ENSG00000104055
ENSG00000104059
ENSG00000104064
ENSG00000104067
ENSG00000104081
ENSG00000104093
ENSG00000104112
ENSG00000104131
ENSG00000104140
ENSG00000104142
ENSG00000104147
ENSG00000104154
ENSG00000104164
ENSG00000104177
ENSG00000104205
ENSG00000104213
ENSG00000104218
ENSG00000104219
ENSG00000104221
ENSG00000104228
ENSG00000104231
ENSG00000104237

ENSG00000104267
ENSG00000104290
ENSG00000104299
ENSG00000104312
ENSG00000104313
ENSG00000104320
ENSG00000104321
ENSG00000104327
ENSG00000104331
ENSG00000104332
ENSG00000104341
ENSG00000104343
ENSG00000104356
ENSG00000104361
ENSG00000104365
ENSG00000104368
ENSG00000104369
ENSG00000104375
ENSG00000104381
ENSG00000104388
ENSG00000104413
ENSG00000104415
ENSG00000104419
ENSG00000104427
ENSG00000104432
ENSG00000104435
ENSG00000104442
ENSG00000104447
ENSG00000104450
ENSG00000104472
ENSG00000104490
ENSG00000104497
ENSG00000104499
ENSG00000104517
ENSG00000104524
ENSG00000104529
ENSG00000104611
ENSG00000104626

ENSG00000104635
ENSG00000104643
ENSG00000104660
ENSG00000104671
ENSG00000104687
ENSG00000104691
ENSG00000104695
ENSG00000104722
ENSG00000104723
ENSG00000104728
ENSG00000104731
ENSG00000104738
ENSG00000104760
ENSG00000104763
ENSG00000104765
ENSG00000104783
ENSG00000104805
ENSG00000104812
ENSG00000104814
ENSG00000104824
ENSG00000104833
ENSG00000104835
ENSG00000104848
ENSG00000104853
ENSG00000104856
ENSG00000104859
ENSG00000104866
ENSG00000104870
ENSG00000104880
ENSG00000104881
ENSG00000104884
ENSG00000104885
ENSG00000104886
ENSG00000104888
ENSG00000104889
ENSG00000104894
ENSG00000104897
ENSG00000104903

ENSG00000104915
ENSG00000104921
ENSG00000104936
ENSG00000104938
ENSG00000104941
ENSG00000104953
ENSG00000104957
ENSG00000104964
ENSG00000104967
ENSG00000104969
ENSG00000104972
ENSG00000104974
ENSG00000104976
ENSG00000104979
ENSG00000104998
ENSG00000105011
ENSG00000105053
ENSG00000105058
ENSG00000105063
ENSG00000105072
ENSG00000105085
ENSG00000105088
ENSG00000105122
ENSG00000105127
ENSG00000105131
ENSG00000105135
ENSG00000105136
ENSG00000105137
ENSG00000105141
ENSG00000105143
ENSG00000105146
ENSG00000105171
ENSG00000105173
ENSG00000105185
ENSG00000105186
ENSG00000105197
ENSG00000105204
ENSG00000105205

ENSG00000105219
ENSG00000105220
ENSG00000105221
ENSG00000105227
ENSG00000105229
ENSG00000105245
ENSG00000105278
ENSG00000105281
ENSG00000105287
ENSG00000105298
ENSG00000105321
ENSG00000105323
ENSG00000105325
ENSG00000105327
ENSG00000105329
ENSG00000105339
ENSG00000105341
ENSG00000105355
ENSG00000105357
ENSG00000105364
ENSG00000105366
ENSG00000105369
ENSG00000105370
ENSG00000105376
ENSG00000105388
ENSG00000105392
ENSG00000105397
ENSG00000105398
ENSG00000105401
ENSG00000105402
ENSG00000105409
ENSG00000105426
ENSG00000105428
ENSG00000105429
ENSG00000105438
ENSG00000105443
ENSG00000105447
ENSG00000105464

ENSG00000105467
ENSG00000105472
ENSG00000105483
ENSG00000105492
ENSG00000105497
ENSG00000105499
ENSG00000105507
ENSG00000105509
ENSG00000105514
ENSG00000105519
ENSG00000105520
ENSG00000105549
ENSG00000105556
ENSG00000105568
ENSG00000105576
ENSG00000105605
ENSG00000105607
ENSG00000105609
ENSG00000105610
ENSG00000105612
ENSG00000105618
ENSG00000105639
ENSG00000105641
ENSG00000105642
ENSG00000105643
ENSG00000105647
ENSG00000105649
ENSG00000105650
ENSG00000105656
ENSG00000105662
ENSG00000105664
ENSG00000105668
ENSG00000105669
ENSG00000105671
ENSG00000105675
ENSG00000105676
ENSG00000105695
ENSG00000105696

ENSG00000105698
ENSG00000105700
ENSG00000105701
ENSG00000105711
ENSG00000105717
ENSG00000105722
ENSG00000105723
ENSG00000105732
ENSG00000105737
ENSG00000105738
ENSG00000105750
ENSG00000105755
ENSG00000105767
ENSG00000105771
ENSG00000105778
ENSG00000105784
ENSG00000105792
ENSG00000105793
ENSG00000105808
ENSG00000105810
ENSG00000105819
ENSG00000105825
ENSG00000105835
ENSG00000105849
ENSG00000105851
ENSG00000105854
ENSG00000105855
ENSG00000105856
ENSG00000105865
ENSG00000105866
ENSG00000105875
ENSG00000105877
ENSG00000105879
ENSG00000105887
ENSG00000105889
ENSG00000105926
ENSG00000105928
ENSG00000105939

ENSG00000105948
ENSG00000105953
ENSG00000105963
ENSG00000105967
ENSG00000105968
ENSG00000105971
ENSG00000105974
ENSG00000105976
ENSG00000105982
ENSG00000105983
ENSG00000105989
ENSG00000105991
ENSG00000105993
ENSG00000105996
ENSG00000105997
ENSG00000106003
ENSG00000106004
ENSG00000106009
ENSG00000106012
ENSG00000106018
ENSG00000106025
ENSG00000106038
ENSG00000106052
ENSG00000106069
ENSG00000106070
ENSG00000106077
ENSG00000106078
ENSG00000106080
ENSG00000106086
ENSG00000106089
ENSG00000106100
ENSG00000106113
ENSG00000106123
ENSG00000106125
ENSG00000106144
ENSG00000106178
ENSG00000106236
ENSG00000106244

ENSG00000106245
ENSG00000106246
ENSG00000106261
ENSG00000106263
ENSG00000106266
ENSG00000106268
ENSG00000106290
ENSG00000106299
ENSG00000106302
ENSG00000106304
ENSG00000106327
ENSG00000106331
ENSG00000106341
ENSG00000106344
ENSG00000106348
ENSG00000106351
ENSG00000106355
ENSG00000106366
ENSG00000106367
ENSG00000106392
ENSG00000106397
ENSG00000106399
ENSG00000106400
ENSG00000106404
ENSG00000106410
ENSG00000106415
ENSG00000106436
ENSG00000106459
ENSG00000106460
ENSG00000106462
ENSG00000106477
ENSG00000106479
ENSG00000106483
ENSG00000106484
ENSG00000106511
ENSG00000106524
ENSG00000106526
ENSG00000106537

ENSG00000106546
ENSG00000106554
ENSG00000106571
ENSG00000106603
ENSG00000106608
ENSG00000106609
ENSG00000106615
ENSG00000106617
ENSG00000106624
ENSG00000106628
ENSG00000106631
ENSG00000106633
ENSG00000106635
ENSG00000106636
ENSG00000106638
ENSG00000106665
ENSG00000106682
ENSG00000106683
ENSG00000106686
ENSG00000106688
ENSG00000106692
ENSG00000106701
ENSG00000106714
ENSG00000106723
ENSG00000106733
ENSG00000106771
ENSG00000106772
ENSG00000106780
ENSG00000106785
ENSG00000106789
ENSG00000106799
ENSG00000106804
ENSG00000106809
ENSG00000106819
ENSG00000106823
ENSG00000106829
ENSG00000106852
ENSG00000106853

ENSG00000106868
ENSG00000106948
ENSG00000106952
ENSG00000106976
ENSG00000106991
ENSG00000106992
ENSG00000106993
ENSG00000107021
ENSG00000107099
ENSG00000107104
ENSG00000107105
ENSG00000107130
ENSG00000107140
ENSG00000107147
ENSG00000107159
ENSG00000107164
ENSG00000107185
ENSG00000107186
ENSG00000107187
ENSG00000107201
ENSG00000107223
ENSG00000107242
ENSG00000107249
ENSG00000107262
ENSG00000107263
ENSG00000107281
ENSG00000107282
ENSG00000107290
ENSG00000107295
ENSG00000107338
ENSG00000107341
ENSG00000107362
ENSG00000107371
ENSG00000107372
ENSG00000107404
ENSG00000107443
ENSG00000107447
ENSG00000107518

ENSG00000107521
ENSG00000107551
ENSG00000107554
ENSG00000107560
ENSG00000107562
ENSG00000107566
ENSG00000107581
ENSG00000107593
ENSG00000107611
ENSG00000107614
ENSG00000107643
ENSG00000107651
ENSG00000107669
ENSG00000107679
ENSG00000107719
ENSG00000107731
ENSG00000107736
ENSG00000107738
ENSG00000107742
ENSG00000107745
ENSG00000107758
ENSG00000107771
ENSG00000107779
ENSG00000107789
ENSG00000107798
ENSG00000107807
ENSG00000107815
ENSG00000107816
ENSG00000107819
ENSG00000107821
ENSG00000107829
ENSG00000107831
ENSG00000107833
ENSG00000107854
ENSG00000107862
ENSG00000107863
ENSG00000107864
ENSG00000107874

ENSG00000107882
ENSG00000107890
ENSG00000107897
ENSG00000107902
ENSG00000107929
ENSG00000107937
ENSG00000107938
ENSG00000107949
ENSG00000107951
ENSG00000107954
ENSG00000107957
ENSG00000107960
ENSG00000107984
ENSG00000108001
ENSG00000108018
ENSG00000108039
ENSG00000108061
ENSG00000108064
ENSG00000108091
ENSG00000108094
ENSG00000108100
ENSG00000108107
ENSG00000108175
ENSG00000108176
ENSG00000108179
ENSG00000108187
ENSG00000108219
ENSG00000108231
ENSG00000108239
ENSG00000108242
ENSG00000108256
ENSG00000108262
ENSG00000108306
ENSG00000108309
ENSG00000108312
ENSG00000108342
ENSG00000108344
ENSG00000108349

ENSG00000108352
ENSG00000108370
ENSG00000108375
ENSG00000108379
ENSG00000108381
ENSG00000108387
ENSG00000108389
ENSG00000108395
ENSG00000108405
ENSG00000108417
ENSG00000108423
ENSG00000108424
ENSG00000108439
ENSG00000108443
ENSG00000108448
ENSG00000108465
ENSG00000108468
ENSG00000108469
ENSG00000108474
ENSG00000108506
ENSG00000108509
ENSG00000108510
ENSG00000108511
ENSG00000108523
ENSG00000108528
ENSG00000108551
ENSG00000108556
ENSG00000108557
ENSG00000108559
ENSG00000108561
ENSG00000108576
ENSG00000108578
ENSG00000108582
ENSG00000108587
ENSG00000108588
ENSG00000108590
ENSG00000108591
ENSG00000108592

ENSG00000108599
ENSG00000108604
ENSG00000108622
ENSG00000108639
ENSG00000108641
ENSG00000108651
ENSG00000108654
ENSG00000108666
ENSG00000108669
ENSG00000108671
ENSG00000108679
ENSG00000108684
ENSG00000108691
ENSG00000108702
ENSG00000108733
ENSG00000108759
ENSG00000108773
ENSG00000108774
ENSG00000108784
ENSG00000108786
ENSG00000108788
ENSG00000108797
ENSG00000108798
ENSG00000108799
ENSG00000108813
ENSG00000108819
ENSG00000108821
ENSG00000108828
ENSG00000108829
ENSG00000108830
ENSG00000108840
ENSG00000108846
ENSG00000108848
ENSG00000108852
ENSG00000108854
ENSG00000108861
ENSG00000108878
ENSG00000108883

ENSG00000108924
ENSG00000108932
ENSG00000108946
ENSG00000108947
ENSG00000108950
ENSG00000108960
ENSG00000108961
ENSG00000108963
ENSG00000108984
ENSG00000109016
ENSG00000109046
ENSG00000109062
ENSG00000109065
ENSG00000109066
ENSG00000109079
ENSG00000109083
ENSG00000109084
ENSG00000109089
ENSG00000109099
ENSG00000109101
ENSG00000109103
ENSG00000109107
ENSG00000109111
ENSG00000109113
ENSG00000109118
ENSG00000109132
ENSG00000109133
ENSG00000109158
ENSG00000109163
ENSG00000109171
ENSG00000109180
ENSG00000109181
ENSG00000109184
ENSG00000109189
ENSG00000109193
ENSG00000109205
ENSG00000109220
ENSG00000109265

ENSG00000109270
ENSG00000109321
ENSG00000109323
ENSG00000109332
ENSG00000109339
ENSG00000109381
ENSG00000109390
ENSG00000109452
ENSG00000109458
ENSG00000109466
ENSG00000109471
ENSG00000109475
ENSG00000109501
ENSG00000109519
ENSG00000109534
ENSG00000109572
ENSG00000109576
ENSG00000109586
ENSG00000109610
ENSG00000109618
ENSG00000109654
ENSG00000109670
ENSG00000109680
ENSG00000109684
ENSG00000109685
ENSG00000109686
ENSG00000109689
ENSG00000109705
ENSG00000109736
ENSG00000109756
ENSG00000109771
ENSG00000109787
ENSG00000109790
ENSG00000109794
ENSG00000109805
ENSG00000109819
ENSG00000109832
ENSG00000109854

ENSG00000109861
ENSG00000109881
ENSG00000109917
ENSG00000109919
ENSG00000109920
ENSG00000109929
ENSG00000109943
ENSG00000109944
ENSG00000109956
ENSG00000110002
ENSG00000110013
ENSG00000110025
ENSG00000110042
ENSG00000110046
ENSG00000110047
ENSG00000110048
ENSG00000110057
ENSG00000110066
ENSG00000110074
ENSG00000110075
ENSG00000110076
ENSG00000110077
ENSG00000110079
ENSG00000110080
ENSG00000110090
ENSG00000110092
ENSG00000110104
ENSG00000110107
ENSG00000110108
ENSG00000110148
ENSG00000110171
ENSG00000110172
ENSG00000110237
ENSG00000110243
ENSG00000110245
ENSG00000110274
ENSG00000110315
ENSG00000110318

ENSG00000110324
ENSG00000110344
ENSG00000110367
ENSG00000110375
ENSG00000110395
ENSG00000110400
ENSG00000110422
ENSG00000110427
ENSG00000110429
ENSG00000110436
ENSG00000110442
ENSG00000110448
ENSG00000110492
ENSG00000110497
ENSG00000110514
ENSG00000110536
ENSG00000110583
ENSG00000110619
ENSG00000110660
ENSG00000110665
ENSG00000110675
ENSG00000110680
ENSG00000110693
ENSG00000110696
ENSG00000110697
ENSG00000110713
ENSG00000110719
ENSG00000110721
ENSG00000110723
ENSG00000110756
ENSG00000110768
ENSG00000110777
ENSG00000110786
ENSG00000110801
ENSG00000110841
ENSG00000110848
ENSG00000110851
ENSG00000110852

ENSG00000110871
ENSG00000110876
ENSG00000110880
ENSG00000110881
ENSG00000110888
ENSG00000110900
ENSG00000110906
ENSG00000110911
ENSG00000110917
ENSG00000110921
ENSG00000110925
ENSG00000110931
ENSG00000110934
ENSG00000110944
ENSG00000110958
ENSG00000110987
ENSG00000111011
ENSG00000111012
ENSG00000111049
ENSG00000111052
ENSG00000111057
ENSG00000111058
ENSG00000111110
ENSG00000111142
ENSG00000111144
ENSG00000111145
ENSG00000111181
ENSG00000111186
ENSG00000111196
ENSG00000111203
ENSG00000111206
ENSG00000111218
ENSG00000111224
ENSG00000111229
ENSG00000111231
ENSG00000111241
ENSG00000111247
ENSG00000111249

ENSG00000111252
ENSG00000111261
ENSG00000111262
ENSG00000111266
ENSG00000111269
ENSG00000111275
ENSG00000111276
ENSG00000111300
ENSG00000111305
ENSG00000111319
ENSG00000111321
ENSG00000111325
ENSG00000111328
ENSG00000111331
ENSG00000111335
ENSG00000111341
ENSG00000111344
ENSG00000111348
ENSG00000111358
ENSG00000111361
ENSG00000111364
ENSG00000111371
ENSG00000111405
ENSG00000111412
ENSG00000111424
ENSG00000111432
ENSG00000111445
ENSG00000111450
ENSG00000111452
ENSG00000111481
ENSG00000111490
ENSG00000111530
ENSG00000111537
ENSG00000111540
ENSG00000111554
ENSG00000111596
ENSG00000111602
ENSG00000111605

ENSG00000111639
ENSG00000111641
ENSG00000111642
ENSG00000111647
ENSG00000111652
ENSG00000111653
ENSG00000111664
ENSG00000111665
ENSG00000111667
ENSG00000111669
ENSG00000111670
ENSG00000111671
ENSG00000111674
ENSG00000111676
ENSG00000111679
ENSG00000111684
ENSG00000111696
ENSG00000111701
ENSG00000111704
ENSG00000111707
ENSG00000111711
ENSG00000111713
ENSG00000111725
ENSG00000111727
ENSG00000111728
ENSG00000111731
ENSG00000111732
ENSG00000111737
ENSG00000111752
ENSG00000111783
ENSG00000111785
ENSG00000111790
ENSG00000111799
ENSG00000111802
ENSG00000111832
ENSG00000111834
ENSG00000111837
ENSG00000111843

ENSG00000111846
ENSG00000111850
ENSG00000111859
ENSG00000111860
ENSG00000111863
ENSG00000111875
ENSG00000111877
ENSG00000111880
ENSG00000111885
ENSG00000111886
ENSG00000111906
ENSG00000111907
ENSG00000111911
ENSG00000111912
ENSG00000111913
ENSG00000111961
ENSG00000111962
ENSG00000111981
ENSG00000112031
ENSG00000112033
ENSG00000112038
ENSG00000112039
ENSG00000112041
ENSG00000112053
ENSG00000112062
ENSG00000112078
ENSG00000112079
ENSG00000112081
ENSG00000112096
ENSG00000112115
ENSG00000112118
ENSG00000112130
ENSG00000112137
ENSG00000112139
ENSG00000112144
ENSG00000112146
ENSG00000112149
ENSG00000112159

ENSG00000112164
ENSG00000112167
ENSG00000112182
ENSG00000112183
ENSG00000112195
ENSG00000112200
ENSG00000112210
ENSG00000112212
ENSG00000112214
ENSG00000112218
ENSG00000112232
ENSG00000112237
ENSG00000112242
ENSG00000112245
ENSG00000112246
ENSG00000112249
ENSG00000112276
ENSG00000112280
ENSG00000112282
ENSG00000112290
ENSG00000112293
ENSG00000112294
ENSG00000112297
ENSG00000112299
ENSG00000112303
ENSG00000112304
ENSG00000112305
ENSG00000112308
ENSG00000112319
ENSG00000112320
ENSG00000112333
ENSG00000112335
ENSG00000112337
ENSG00000112339
ENSG00000112343
ENSG00000112365
ENSG00000112367
ENSG00000112378

ENSG00000112379
ENSG00000112394
ENSG00000112406
ENSG00000112414
ENSG00000112419
ENSG00000112425
ENSG00000112462
ENSG00000112473
ENSG00000112486
ENSG00000112494
ENSG00000112499
ENSG00000112514
ENSG00000112531
ENSG00000112541
ENSG00000112559
ENSG00000112561
ENSG00000112562
ENSG00000112576
ENSG00000112584
ENSG00000112592
ENSG00000112599
ENSG00000112619
ENSG00000112624
ENSG00000112640
ENSG00000112651
ENSG00000112655
ENSG00000112658
ENSG00000112667
ENSG00000112679
ENSG00000112685
ENSG00000112695
ENSG00000112697
ENSG00000112699
ENSG00000112715
ENSG00000112739
ENSG00000112742
ENSG00000112759
ENSG00000112763

ENSG00000112769
ENSG00000112773
ENSG00000112782
ENSG00000112787
ENSG00000112796
ENSG00000112799
ENSG00000112812
ENSG00000112818
ENSG00000112837
ENSG00000112851
ENSG00000112855
ENSG00000112874
ENSG00000112893
ENSG00000112902
ENSG00000112936
ENSG00000112941
ENSG00000112964
ENSG00000112972
ENSG00000112977
ENSG00000112981
ENSG00000112983
ENSG00000112984
ENSG00000113048
ENSG00000113068
ENSG00000113070
ENSG00000113073
ENSG00000113083
ENSG00000113108
ENSG00000113119
ENSG00000113140
ENSG00000113161
ENSG00000113163
ENSG00000113194
ENSG00000113196
ENSG00000113212
ENSG00000113231
ENSG00000113240
ENSG00000113262

ENSG00000113263
ENSG00000113269
ENSG00000113272
ENSG00000113273
ENSG00000113300
ENSG00000113302
ENSG00000113303
ENSG00000113312
ENSG00000113318
ENSG00000113319
ENSG00000113327
ENSG00000113356
ENSG00000113360
ENSG00000113361
ENSG00000113368
ENSG00000113369
ENSG00000113384
ENSG00000113387
ENSG00000113389
ENSG00000113391
ENSG00000113396
ENSG00000113430
ENSG00000113441
ENSG00000113448
ENSG00000113456
ENSG00000113492
ENSG00000113494
ENSG00000113504
ENSG00000113522
ENSG00000113525
ENSG00000113532
ENSG00000113552
ENSG00000113555
ENSG00000113558
ENSG00000113578
ENSG00000113580
ENSG00000113583
ENSG00000113594

ENSG00000113595
ENSG00000113597
ENSG00000113600
ENSG00000113615
ENSG00000113621
ENSG00000113638
ENSG00000113645
ENSG00000113648
ENSG00000113657
ENSG00000113658
ENSG00000113712
ENSG00000113716
ENSG00000113719
ENSG00000113721
ENSG00000113722
ENSG00000113732
ENSG00000113739
ENSG00000113742
ENSG00000113749
ENSG00000113761
ENSG00000113763
ENSG00000113790
ENSG00000113805
ENSG00000113810
ENSG00000113811
ENSG00000113812
ENSG00000113838
ENSG00000113845
ENSG00000113851
ENSG00000113889
ENSG00000113905
ENSG00000113946
ENSG00000113966
ENSG00000114013
ENSG00000114019
ENSG00000114023
ENSG00000114026
ENSG00000114030

ENSG00000114062
ENSG00000114098
ENSG00000114107
ENSG00000114113
ENSG00000114115
ENSG00000114120
ENSG00000114125
ENSG00000114126
ENSG00000114127
ENSG00000114166
ENSG00000114209
ENSG00000114248
ENSG00000114251
ENSG00000114268
ENSG00000114270
ENSG00000114279
ENSG00000114302
ENSG00000114315
ENSG00000114316
ENSG00000114331
ENSG00000114349
ENSG00000114353
ENSG00000114354
ENSG00000114374
ENSG00000114378
ENSG00000114383
ENSG00000114395
ENSG00000114405
ENSG00000114416
ENSG00000114423
ENSG00000114439
ENSG00000114446
ENSG00000114450
ENSG00000114455
ENSG00000114473
ENSG00000114480
ENSG00000114491
ENSG00000114503

ENSG00000114529
ENSG00000114541
ENSG00000114544
ENSG00000114554
ENSG00000114573
ENSG00000114626
ENSG00000114638
ENSG00000114648
ENSG00000114654
ENSG00000114698
ENSG00000114735
ENSG00000114737
ENSG00000114738
ENSG00000114739
ENSG00000114742
ENSG00000114744
ENSG00000114745
ENSG00000114757
ENSG00000114770
ENSG00000114771
ENSG00000114779
ENSG00000114790
ENSG00000114796
ENSG00000114805
ENSG00000114812
ENSG00000114850
ENSG00000114853
ENSG00000114854
ENSG00000114857
ENSG00000114859
ENSG00000114861
ENSG00000114867
ENSG00000114904
ENSG00000114923
ENSG00000114933
ENSG00000114948
ENSG00000114956
ENSG00000114978

ENSG00000114982
ENSG00000114988
ENSG00000114999
ENSG00000115008
ENSG00000115009
ENSG00000115020
ENSG00000115041
ENSG00000115073
ENSG00000115084
ENSG00000115091
ENSG00000115107
ENSG00000115109
ENSG00000115112
ENSG00000115129
ENSG00000115137
ENSG00000115145
ENSG00000115159
ENSG00000115163
ENSG00000115165
ENSG00000115170
ENSG00000115194
ENSG00000115204
ENSG00000115207
ENSG00000115216
ENSG00000115221
ENSG00000115226
ENSG00000115232
ENSG00000115233
ENSG00000115234
ENSG00000115239
ENSG00000115241
ENSG00000115252
ENSG00000115263
ENSG00000115266
ENSG00000115271
ENSG00000115275
ENSG00000115282
ENSG00000115286

ENSG00000115289
ENSG00000115295
ENSG00000115297
ENSG00000115306
ENSG00000115310
ENSG00000115317
ENSG00000115318
ENSG00000115325
ENSG00000115339
ENSG00000115350
ENSG00000115353
ENSG00000115355
ENSG00000115363
ENSG00000115364
ENSG00000115365
ENSG00000115380
ENSG00000115392
ENSG00000115414
ENSG00000115421
ENSG00000115425
ENSG00000115457
ENSG00000115459
ENSG00000115461
ENSG00000115468
ENSG00000115474
ENSG00000115484
ENSG00000115486
ENSG00000115504
ENSG00000115507
ENSG00000115514
ENSG00000115520
ENSG00000115523
ENSG00000115525
ENSG00000115526
ENSG00000115540
ENSG00000115556
ENSG00000115561
ENSG00000115590

ENSG00000115592
ENSG00000115593
ENSG00000115594
ENSG00000115596
ENSG00000115598
ENSG00000115602
ENSG00000115604
ENSG00000115607
ENSG00000115616
ENSG00000115641
ENSG00000115648
ENSG00000115652
ENSG00000115661
ENSG00000115665
ENSG00000115677
ENSG00000115687
ENSG00000115694
ENSG00000115705
ENSG00000115718
ENSG00000115738
ENSG00000115756
ENSG00000115758
ENSG00000115760
ENSG00000115761
ENSG00000115762
ENSG00000115806
ENSG00000115808
ENSG00000115825
ENSG00000115827
ENSG00000115839
ENSG00000115844
ENSG00000115875
ENSG00000115884
ENSG00000115896
ENSG00000115902
ENSG00000115904
ENSG00000115935
ENSG00000115942

ENSG00000115944
ENSG00000115947
ENSG00000115956
ENSG00000115963
ENSG00000115966
ENSG00000115977
ENSG00000115993
ENSG00000115998
ENSG00000116001
ENSG00000116005
ENSG00000116014
ENSG00000116016
ENSG00000116017
ENSG00000116030
ENSG00000116031
ENSG00000116044
ENSG00000116095
ENSG00000116096
ENSG00000116106
ENSG00000116117
ENSG00000116120
ENSG00000116128
ENSG00000116132
ENSG00000116133
ENSG00000116138
ENSG00000116141
ENSG00000116147
ENSG00000116151
ENSG00000116157
ENSG00000116161
ENSG00000116171
ENSG00000116191
ENSG00000116194
ENSG00000116198
ENSG00000116199
ENSG00000116205
ENSG00000116209
ENSG00000116218

ENSG00000116237
ENSG00000116251
ENSG00000116254
ENSG00000116260
ENSG00000116266
ENSG00000116273
ENSG00000116285
ENSG00000116299
ENSG00000116329
ENSG00000116337
ENSG00000116350
ENSG00000116353
ENSG00000116396
ENSG00000116406
ENSG00000116455
ENSG00000116473
ENSG00000116478
ENSG00000116489
ENSG00000116497
ENSG00000116521
ENSG00000116525
ENSG00000116539
ENSG00000116574
ENSG00000116580
ENSG00000116584
ENSG00000116586
ENSG00000116604
ENSG00000116641
ENSG00000116649
ENSG00000116661
ENSG00000116663
ENSG00000116667
ENSG00000116668
ENSG00000116675
ENSG00000116678
ENSG00000116679
ENSG00000116685
ENSG00000116688

ENSG00000116691
ENSG00000116698
ENSG00000116701
ENSG00000116704
ENSG00000116717
ENSG00000116721
ENSG00000116726
ENSG00000116729
ENSG00000116731
ENSG00000116741
ENSG00000116747
ENSG00000116750
ENSG00000116754
ENSG00000116761
ENSG00000116771
ENSG00000116785
ENSG00000116786
ENSG00000116793
ENSG00000116809
ENSG00000116819
ENSG00000116830
ENSG00000116833
ENSG00000116852
ENSG00000116857
ENSG00000116863
ENSG00000116871
ENSG00000116874
ENSG00000116885
ENSG00000116903
ENSG00000116918
ENSG00000116922
ENSG00000116954
ENSG00000116962
ENSG00000116977
ENSG00000116983
ENSG00000116984
ENSG00000116985
ENSG00000116990

ENSG00000116991
ENSG00000117009
ENSG00000117010
ENSG00000117013
ENSG00000117016
ENSG00000117020
ENSG00000117036
ENSG00000117054
ENSG00000117069
ENSG00000117090
ENSG00000117091
ENSG00000117114
ENSG00000117115
ENSG00000117122
ENSG00000117139
ENSG00000117143
ENSG00000117148
ENSG00000117151
ENSG00000117152
ENSG00000117153
ENSG00000117155
ENSG00000117174
ENSG00000117215
ENSG00000117222
ENSG00000117226
ENSG00000117228
ENSG00000117245
ENSG00000117262
ENSG00000117266
ENSG00000117280
ENSG00000117281
ENSG00000117298
ENSG00000117305
ENSG00000117318
ENSG00000117322
ENSG00000117335
ENSG00000117362
ENSG00000117385

ENSG00000117394
ENSG00000117400
ENSG00000117407
ENSG00000117408
ENSG00000117410
ENSG00000117411
ENSG00000117419
ENSG00000117425
ENSG00000117450
ENSG00000117461
ENSG00000117472
ENSG00000117475
ENSG00000117479
ENSG00000117480
ENSG00000117481
ENSG00000117500
ENSG00000117501
ENSG00000117505
ENSG00000117519
ENSG00000117523
ENSG00000117525
ENSG00000117528
ENSG00000117533
ENSG00000117543
ENSG00000117560
ENSG00000117569
ENSG00000117586
ENSG00000117592
ENSG00000117595
ENSG00000117597
ENSG00000117598
ENSG00000117600
ENSG00000117602
ENSG00000117614
ENSG00000117620
ENSG00000117625
ENSG00000117632
ENSG00000117640

ENSG00000117643
ENSG00000117676
ENSG00000117682
ENSG00000117697
ENSG00000117707
ENSG00000117713
ENSG00000117724
ENSG00000117748
ENSG00000117751
ENSG00000117758
ENSG00000117859
ENSG00000117862
ENSG00000117868
ENSG00000117899
ENSG00000117971
ENSG00000117983
ENSG00000118004
ENSG00000118007
ENSG00000118017
ENSG00000118058
ENSG00000118096
ENSG00000118113
ENSG00000118160
ENSG00000118162
ENSG00000118193
ENSG00000118197
ENSG00000118200
ENSG00000118217
ENSG00000118242
ENSG00000118245
ENSG00000118246
ENSG00000118257
ENSG00000118260
ENSG00000118263
ENSG00000118276
ENSG00000118298
ENSG00000118307
ENSG00000118322

ENSG00000118363
ENSG00000118369
ENSG00000118412
ENSG00000118420
ENSG00000118432
ENSG00000118434
ENSG00000118454
ENSG00000118473
ENSG00000118482
ENSG00000118491
ENSG00000118492
ENSG00000118496
ENSG00000118503
ENSG00000118507
ENSG00000118508
ENSG00000118513
ENSG00000118514
ENSG00000118515
ENSG00000118518
ENSG00000118520
ENSG00000118523
ENSG00000118526
ENSG00000118564
ENSG00000118579
ENSG00000118596
ENSG00000118620
ENSG00000118640
ENSG00000118655
ENSG00000118689
ENSG00000118690
ENSG00000118707
ENSG00000118729
ENSG00000118733
ENSG00000118762
ENSG00000118777
ENSG00000118785
ENSG00000118804
ENSG00000118849

ENSG00000118873
ENSG00000118894
ENSG00000118898
ENSG00000118900
ENSG00000118922
ENSG00000118946
ENSG00000118960
ENSG00000118961
ENSG00000118965
ENSG00000118971
ENSG00000118972
ENSG00000118985
ENSG00000119004
ENSG00000119042
ENSG00000119048
ENSG00000119121
ENSG00000119125
ENSG00000119138
ENSG00000119203
ENSG00000119227
ENSG00000119231
ENSG00000119242
ENSG00000119280
ENSG00000119283
ENSG00000119285
ENSG00000119314
ENSG00000119318
ENSG00000119321
ENSG00000119328
ENSG00000119335
ENSG00000119383
ENSG00000119396
ENSG00000119401
ENSG00000119402
ENSG00000119403
ENSG00000119408
ENSG00000119411
ENSG00000119414

ENSG00000119421
ENSG00000119431
ENSG00000119446
ENSG00000119457
ENSG00000119471
ENSG00000119487
ENSG00000119508
ENSG00000119509
ENSG00000119514
ENSG00000119522
ENSG00000119537
ENSG00000119541
ENSG00000119547
ENSG00000119559
ENSG00000119596
ENSG00000119599
ENSG00000119608
ENSG00000119614
ENSG00000119616
ENSG00000119630
ENSG00000119636
ENSG00000119638
ENSG00000119650
ENSG00000119655
ENSG00000119661
ENSG00000119669
ENSG00000119681
ENSG00000119682
ENSG00000119684
ENSG00000119685
ENSG00000119686
ENSG00000119688
ENSG00000119689
ENSG00000119698
ENSG00000119699
ENSG00000119703
ENSG00000119707
ENSG00000119711

ENSG00000119714
ENSG00000119715
ENSG00000119720
ENSG00000119725
ENSG00000119729
ENSG00000119760
ENSG00000119771
ENSG00000119772
ENSG00000119777
ENSG00000119778
ENSG00000119782
ENSG00000119787
ENSG00000119801
ENSG00000119812
ENSG00000119820
ENSG00000119862
ENSG00000119865
ENSG00000119866
ENSG00000119878
ENSG00000119899
ENSG00000119900
ENSG00000119906
ENSG00000119912
ENSG00000119913
ENSG00000119915
ENSG00000119917
ENSG00000119919
ENSG00000119922
ENSG00000119927
ENSG00000119938
ENSG00000119943
ENSG00000119946
ENSG00000119950
ENSG00000119953
ENSG00000119965
ENSG00000119973
ENSG00000119977
ENSG00000119979

ENSG00000119986
ENSG00000120029
ENSG00000120049
ENSG00000120051
ENSG00000120054
ENSG00000120057
ENSG00000120063
ENSG00000120068
ENSG00000120071
ENSG00000120088
ENSG00000120093
ENSG00000120094
ENSG00000120129
ENSG00000120137
ENSG00000120149
ENSG00000120156
ENSG00000120158
ENSG00000120162
ENSG00000120215
ENSG00000120217
ENSG00000120242
ENSG00000120251
ENSG00000120253
ENSG00000120256
ENSG00000120262
ENSG00000120278
ENSG00000120279
ENSG00000120280
ENSG00000120289
ENSG00000120306
ENSG00000120314
ENSG00000120318
ENSG00000120324
ENSG00000120332
ENSG00000120333
ENSG00000120334
ENSG00000120341
ENSG00000120370

ENSG00000120438
ENSG00000120440
ENSG00000120451
ENSG00000120457
ENSG00000120471
ENSG00000120509
ENSG00000120519
ENSG00000120526
ENSG00000120533
ENSG00000120539
ENSG00000120549
ENSG00000120594
ENSG00000120616
ENSG00000120645
ENSG00000120647
ENSG00000120656
ENSG00000120658
ENSG00000120659
ENSG00000120662
ENSG00000120669
ENSG00000120675
ENSG00000120686
ENSG00000120690
ENSG00000120693
ENSG00000120694
ENSG00000120705
ENSG00000120708
ENSG00000120709
ENSG00000120725
ENSG00000120727
ENSG00000120733
ENSG00000120738
ENSG00000120742
ENSG00000120756
ENSG00000120784
ENSG00000120798
ENSG00000120800
ENSG00000120802

ENSG00000120805
ENSG00000120820
ENSG00000120832
ENSG00000120833
ENSG00000120837
ENSG00000120875
ENSG00000120885
ENSG00000120889
ENSG00000120896
ENSG00000120899
ENSG00000120903
ENSG00000120907
ENSG00000120913
ENSG00000120915
ENSG00000120925
ENSG00000120942
ENSG00000120948
ENSG00000120949
ENSG00000120963
ENSG00000121005
ENSG00000121022
ENSG00000121039
ENSG00000121058
ENSG00000121060
ENSG00000121064
ENSG00000121067
ENSG00000121068
ENSG00000121073
ENSG00000121075
ENSG00000121101
ENSG00000121104
ENSG00000121152
ENSG00000121207
ENSG00000121210
ENSG00000121236
ENSG00000121274
ENSG00000121281
ENSG00000121289

ENSG00000121297
ENSG00000121310
ENSG00000121350
ENSG00000121351
ENSG00000121361
ENSG00000121380
ENSG00000121381
ENSG00000121390
ENSG00000121406
ENSG00000121410
ENSG00000121413
ENSG00000121417
ENSG00000121446
ENSG00000121454
ENSG00000121481
ENSG00000121486
ENSG00000121542
ENSG00000121552
ENSG00000121570
ENSG00000121577
ENSG00000121578
ENSG00000121579
ENSG00000121594
ENSG00000121644
ENSG00000121653
ENSG00000121671
ENSG00000121680
ENSG00000121716
ENSG00000121741
ENSG00000121742
ENSG00000121743
ENSG00000121749
ENSG00000121753
ENSG00000121766
ENSG00000121769
ENSG00000121774
ENSG00000121807
ENSG00000121851

ENSG00000121858
ENSG00000121864
ENSG00000121871
ENSG00000121879
ENSG00000121892
ENSG00000121895
ENSG00000121897
ENSG00000121898
ENSG00000121903
ENSG00000121904
ENSG00000121905
ENSG00000121933
ENSG00000121940
ENSG00000121957
ENSG00000121964
ENSG00000121966
ENSG00000121988
ENSG00000121989
ENSG00000122008
ENSG00000122012
ENSG00000122025
ENSG00000122034
ENSG00000122042
ENSG00000122068
ENSG00000122085
ENSG00000122121
ENSG00000122122
ENSG00000122126
ENSG00000122133
ENSG00000122136
ENSG00000122140
ENSG00000122176
ENSG00000122180
ENSG00000122188
ENSG00000122194
ENSG00000122203
ENSG00000122223
ENSG00000122224

ENSG00000122254
ENSG00000122299
ENSG00000122304
ENSG00000122335
ENSG00000122359
ENSG00000122367
ENSG00000122375
ENSG00000122376
ENSG00000122378
ENSG00000122386
ENSG00000122390
ENSG00000122417
ENSG00000122420
ENSG00000122477
ENSG00000122481
ENSG00000122482
ENSG00000122484
ENSG00000122490
ENSG00000122507
ENSG00000122512
ENSG00000122515
ENSG00000122543
ENSG00000122545
ENSG00000122547
ENSG00000122550
ENSG00000122557
ENSG00000122565
ENSG00000122566
ENSG00000122574
ENSG00000122584
ENSG00000122585
ENSG00000122591
ENSG00000122592
ENSG00000122641
ENSG00000122642
ENSG00000122643
ENSG00000122644
ENSG00000122678

ENSG00000122679
ENSG00000122687
ENSG00000122691
ENSG00000122692
ENSG00000122694
ENSG00000122696
ENSG00000122705
ENSG00000122707
ENSG00000122728
ENSG00000122729
ENSG00000122733
ENSG00000122741
ENSG00000122756
ENSG00000122778
ENSG00000122779
ENSG00000122783
ENSG00000122786
ENSG00000122787
ENSG00000122824
ENSG00000122852
ENSG00000122859
ENSG00000122861
ENSG00000122862
ENSG00000122863
ENSG00000122870
ENSG00000122873
ENSG00000122877
ENSG00000122952
ENSG00000122958
ENSG00000122965
ENSG00000122966
ENSG00000122986
ENSG00000123064
ENSG00000123066
ENSG00000123080
ENSG00000123091
ENSG00000123094
ENSG00000123095

ENSG00000123096
ENSG00000123104
ENSG00000123106
ENSG00000123119
ENSG00000123124
ENSG00000123130
ENSG00000123144
ENSG00000123146
ENSG00000123154
ENSG00000123159
ENSG00000123165
ENSG00000123178
ENSG00000123179
ENSG00000123191
ENSG00000123200
ENSG00000123213
ENSG00000123219
ENSG00000123240
ENSG00000123243
ENSG00000123268
ENSG00000123297
ENSG00000123307
ENSG00000123329
ENSG00000123338
ENSG00000123342
ENSG00000123349
ENSG00000123352
ENSG00000123353
ENSG00000123360
ENSG00000123364
ENSG00000123374
ENSG00000123388
ENSG00000123405
ENSG00000123411
ENSG00000123415
ENSG00000123427
ENSG00000123444
ENSG00000123453

ENSG00000123454
ENSG00000123472
ENSG00000123473
ENSG00000123485
ENSG00000123500
ENSG00000123505
ENSG00000123545
ENSG00000123552
ENSG00000123560
ENSG00000123562
ENSG00000123569
ENSG00000123570
ENSG00000123572
ENSG00000123575
ENSG00000123576
ENSG00000123584
ENSG00000123600
ENSG00000123607
ENSG00000123612
ENSG00000123636
ENSG00000123643
ENSG00000123684
ENSG00000123685
ENSG00000123689
ENSG00000123700
ENSG00000123728
ENSG00000123739
ENSG00000123810
ENSG00000123815
ENSG00000123836
ENSG00000123892
ENSG00000123901
ENSG00000123908
ENSG00000123933
ENSG00000123983
ENSG00000123989
ENSG00000123992
ENSG00000124006

ENSG00000124019
ENSG00000124067
ENSG00000124092
ENSG00000124098
ENSG00000124104
ENSG00000124116
ENSG00000124120
ENSG00000124126
ENSG00000124134
ENSG00000124140
ENSG00000124143
ENSG00000124145
ENSG00000124151
ENSG00000124155
ENSG00000124160
ENSG00000124164
ENSG00000124171
ENSG00000124177
ENSG00000124181
ENSG00000124191
ENSG00000124193
ENSG00000124194
ENSG00000124198
ENSG00000124201
ENSG00000124203
ENSG00000124205
ENSG00000124208
ENSG00000124209
ENSG00000124212
ENSG00000124214
ENSG00000124215
ENSG00000124216
ENSG00000124217
ENSG00000124222
ENSG00000124225
ENSG00000124226
ENSG00000124227
ENSG00000124237

ENSG00000124243
ENSG00000124249
ENSG00000124253
ENSG00000124256
ENSG00000124260
ENSG00000124275
ENSG00000124299
ENSG00000124302
ENSG00000124313
ENSG00000124333
ENSG00000124334
ENSG00000124343
ENSG00000124356
ENSG00000124357
ENSG00000124370
ENSG00000124374
ENSG00000124406
ENSG00000124422
ENSG00000124429
ENSG00000124440
ENSG00000124444
ENSG00000124459
ENSG00000124467
ENSG00000124469
ENSG00000124479
ENSG00000124486
ENSG00000124491
ENSG00000124493
ENSG00000124496
ENSG00000124507
ENSG00000124508
ENSG00000124523
ENSG00000124532
ENSG00000124535
ENSG00000124557
ENSG00000124562
ENSG00000124568
ENSG00000124570

ENSG00000124571
ENSG00000124574
ENSG00000124593
ENSG00000124596
ENSG00000124602
ENSG00000124608
ENSG00000124613
ENSG00000124615
ENSG00000124641
ENSG00000124659
ENSG00000124664
ENSG00000124688
ENSG00000124701
ENSG00000124702
ENSG00000124713
ENSG00000124731
ENSG00000124743
ENSG00000124749
ENSG00000124762
ENSG00000124766
ENSG00000124767
ENSG00000124772
ENSG00000124782
ENSG00000124783
ENSG00000124784
ENSG00000124785
ENSG00000124786
ENSG00000124788
ENSG00000124795
ENSG00000124812
ENSG00000124813
ENSG00000124818
ENSG00000124827
ENSG00000124831
ENSG00000124839
ENSG00000124875
ENSG00000124882
ENSG00000124920

ENSG00000124942
ENSG00000125037
ENSG00000125046
ENSG00000125084
ENSG00000125107
ENSG00000125149
ENSG00000125166
ENSG00000125170
ENSG00000125246
ENSG00000125249
ENSG00000125255
ENSG00000125257
ENSG00000125266
ENSG00000125285
ENSG00000125304
ENSG00000125319
ENSG00000125347
ENSG00000125351
ENSG00000125354
ENSG00000125355
ENSG00000125375
ENSG00000125378
ENSG00000125384
ENSG00000125386
ENSG00000125398
ENSG00000125409
ENSG00000125430
ENSG00000125434
ENSG00000125445
ENSG00000125447
ENSG00000125449
ENSG00000125454
ENSG00000125457
ENSG00000125458
ENSG00000125459
ENSG00000125462
ENSG00000125482
ENSG00000125484

ENSG00000125485
ENSG00000125498
ENSG00000125503
ENSG00000125505
ENSG00000125508
ENSG00000125510
ENSG00000125520
ENSG00000125538
ENSG00000125551
ENSG00000125611
ENSG00000125618
ENSG00000125629
ENSG00000125630
ENSG00000125633
ENSG00000125637
ENSG00000125648
ENSG00000125651
ENSG00000125657
ENSG00000125675
ENSG00000125686
ENSG00000125726
ENSG00000125733
ENSG00000125735
ENSG00000125740
ENSG00000125741
ENSG00000125744
ENSG00000125746
ENSG00000125753
ENSG00000125772
ENSG00000125775
ENSG00000125779
ENSG00000125780
ENSG00000125788
ENSG00000125798
ENSG00000125810
ENSG00000125812
ENSG00000125813
ENSG00000125814

ENSG00000125816
ENSG00000125817
ENSG00000125818
ENSG00000125820
ENSG00000125821
ENSG00000125826
ENSG00000125827
ENSG00000125834
ENSG00000125841
ENSG00000125843
ENSG00000125848
ENSG00000125850
ENSG00000125851
ENSG00000125861
ENSG00000125863
ENSG00000125868
ENSG00000125869
ENSG00000125870
ENSG00000125871
ENSG00000125872
ENSG00000125875
ENSG00000125877
ENSG00000125878
ENSG00000125879
ENSG00000125885
ENSG00000125898
ENSG00000125900
ENSG00000125901
ENSG00000125903
ENSG00000125910
ENSG00000125912
ENSG00000125931
ENSG00000125944
ENSG00000125945
ENSG00000125952
ENSG00000125954
ENSG00000125965
ENSG00000125966

ENSG00000125967
ENSG00000125968
ENSG00000125970
ENSG00000125971
ENSG00000125975
ENSG00000125977
ENSG00000125991
ENSG00000125998
ENSG00000126001
ENSG00000126003
ENSG00000126012
ENSG00000126016
ENSG00000126062
ENSG00000126067
ENSG00000126070
ENSG00000126091
ENSG00000126106
ENSG00000126214
ENSG00000126215
ENSG00000126216
ENSG00000126217
ENSG00000126218
ENSG00000126226
ENSG00000126231
ENSG00000126233
ENSG00000126243
ENSG00000126247
ENSG00000126259
ENSG00000126261
ENSG00000126262
ENSG00000126337
ENSG00000126351
ENSG00000126353
ENSG00000126391
ENSG00000126453
ENSG00000126458
ENSG00000126460
ENSG00000126464

ENSG00000126500
ENSG00000126524
ENSG00000126545
ENSG00000126561
ENSG00000126562
ENSG00000126581
ENSG00000126603
ENSG00000126698
ENSG00000126705
ENSG00000126733
ENSG00000126746
ENSG00000126767
ENSG00000126768
ENSG00000126773
ENSG00000126775
ENSG00000126777
ENSG00000126778
ENSG00000126785
ENSG00000126787
ENSG00000126803
ENSG00000126804
ENSG00000126814
ENSG00000126821
ENSG00000126822
ENSG00000126856
ENSG00000126858
ENSG00000126860
ENSG00000126878
ENSG00000126882
ENSG00000126883
ENSG00000126890
ENSG00000126895
ENSG00000126903
ENSG00000126934
ENSG00000126945
ENSG00000126950
ENSG00000126953
ENSG00000126970

ENSG00000127022
ENSG00000127074
ENSG00000127080
ENSG00000127081
ENSG00000127083
ENSG00000127084
ENSG00000127124
ENSG00000127125
ENSG00000127129
ENSG00000127152
ENSG00000127184
ENSG00000127191
ENSG00000127241
ENSG00000127249
ENSG00000127311
ENSG00000127314
ENSG00000127318
ENSG00000127325
ENSG00000127328
ENSG00000127329
ENSG00000127334
ENSG00000127362
ENSG00000127366
ENSG00000127377
ENSG00000127418
ENSG00000127423
ENSG00000127445
ENSG00000127452
ENSG00000127463
ENSG00000127472
ENSG00000127483
ENSG00000127507
ENSG00000127511
ENSG00000127526
ENSG00000127527
ENSG00000127528
ENSG00000127533
ENSG00000127554

ENSG00000127561
ENSG00000127580
ENSG00000127585
ENSG00000127603
ENSG00000127663
ENSG00000127804
ENSG00000127837
ENSG00000127838
ENSG00000127863
ENSG00000127884
ENSG00000127903
ENSG00000127914
ENSG00000127922
ENSG00000127946
ENSG00000127947
ENSG00000127951
ENSG00000127952
ENSG00000127954
ENSG00000127955
ENSG00000127989
ENSG00000127993
ENSG00000128000
ENSG00000128011
ENSG00000128016
ENSG00000128039
ENSG00000128045
ENSG00000128050
ENSG00000128052
ENSG00000128059
ENSG00000128165
ENSG00000128185
ENSG00000128191
ENSG00000128203
ENSG00000128242
ENSG00000128245
ENSG00000128266
ENSG00000128268
ENSG00000128271

ENSG00000128274
ENSG00000128283
ENSG00000128284
ENSG00000128285
ENSG00000128309
ENSG00000128313
ENSG00000128335
ENSG00000128340
ENSG00000128342
ENSG00000128346
ENSG00000128383
ENSG00000128394
ENSG00000128463
ENSG00000128482
ENSG00000128487
ENSG00000128510
ENSG00000128512
ENSG00000128513
ENSG00000128524
ENSG00000128534
ENSG00000128536
ENSG00000128563
ENSG00000128564
ENSG00000128567
ENSG00000128573
ENSG00000128578
ENSG00000128581
ENSG00000128585
ENSG00000128590
ENSG00000128591
ENSG00000128594
ENSG00000128595
ENSG00000128596
ENSG00000128602
ENSG00000128604
ENSG00000128607
ENSG00000128609
ENSG00000128610

ENSG00000128626
ENSG00000128654
ENSG00000128655
ENSG00000128656
ENSG00000128683
ENSG00000128694
ENSG00000128699
ENSG00000128708
ENSG00000128709
ENSG00000128710
ENSG00000128713
ENSG00000128714
ENSG00000128731
ENSG00000128789
ENSG00000128791
ENSG00000128805
ENSG00000128815
ENSG00000128829
ENSG00000128833
ENSG00000128849
ENSG00000128872
ENSG00000128881
ENSG00000128891
ENSG00000128908
ENSG00000128915
ENSG00000128917
ENSG00000128918
ENSG00000128923
ENSG00000128928
ENSG00000128944
ENSG00000128951
ENSG00000128965
ENSG00000128973
ENSG00000128989
ENSG00000129003
ENSG00000129007
ENSG00000129009
ENSG00000129028

ENSG00000129055
ENSG00000129071
ENSG00000129103
ENSG00000129116
ENSG00000129128
ENSG00000129152
ENSG00000129159
ENSG00000129167
ENSG00000129170
ENSG00000129187
ENSG00000129195
ENSG00000129197
ENSG00000129204
ENSG00000129219
ENSG00000129221
ENSG00000129226
ENSG00000129235
ENSG00000129244
ENSG00000129245
ENSG00000129250
ENSG00000129255
ENSG00000129292
ENSG00000129295
ENSG00000129315
ENSG00000129347
ENSG00000129351
ENSG00000129353
ENSG00000129354
ENSG00000129355
ENSG00000129422
ENSG00000129451
ENSG00000129460
ENSG00000129465
ENSG00000129472
ENSG00000129473
ENSG00000129474
ENSG00000129493
ENSG00000129514

ENSG00000129515
ENSG00000129521
ENSG00000129534
ENSG00000129535
ENSG00000129538
ENSG00000129559
ENSG00000129595
ENSG00000129596
ENSG00000129625
ENSG00000129654
ENSG00000129657
ENSG00000129667
ENSG00000129675
ENSG00000129680
ENSG00000129682
ENSG00000129691
ENSG00000129696
ENSG00000129749
ENSG00000129757
ENSG00000129810
ENSG00000129910
ENSG00000129911
ENSG00000129925
ENSG00000129932
ENSG00000129933
ENSG00000129946
ENSG00000129988
ENSG00000129990
ENSG00000129991
ENSG00000129993
ENSG00000130005
ENSG00000130021
ENSG00000130032
ENSG00000130035
ENSG00000130037
ENSG00000130038
ENSG00000130045
ENSG00000130052

ENSG00000130054
ENSG00000130055
ENSG00000130119
ENSG00000130147
ENSG00000130150
ENSG00000130159
ENSG00000130164
ENSG00000130165
ENSG00000130167
ENSG00000130173
ENSG00000130175
ENSG00000130176
ENSG00000130193
ENSG00000130201
ENSG00000130202
ENSG00000130204
ENSG00000130224
ENSG00000130226
ENSG00000130227
ENSG00000130234
ENSG00000130270
ENSG00000130287
ENSG00000130294
ENSG00000130303
ENSG00000130304
ENSG00000130305
ENSG00000130309
ENSG00000130311
ENSG00000130312
ENSG00000130313
ENSG00000130332
ENSG00000130338
ENSG00000130340
ENSG00000130347
ENSG00000130348
ENSG00000130349
ENSG00000130368
ENSG00000130377

ENSG00000130382
ENSG00000130383
ENSG00000130396
ENSG00000130402
ENSG00000130413
ENSG00000130414
ENSG00000130427
ENSG00000130429
ENSG00000130449
ENSG00000130475
ENSG00000130477
ENSG00000130487
ENSG00000130508
ENSG00000130513
ENSG00000130517
ENSG00000130518
ENSG00000130520
ENSG00000130522
ENSG00000130529
ENSG00000130540
ENSG00000130544
ENSG00000130545
ENSG00000130558
ENSG00000130559
ENSG00000130560
ENSG00000130584
ENSG00000130589
ENSG00000130590
ENSG00000130592
ENSG00000130595
ENSG00000130598
ENSG00000130635
ENSG00000130638
ENSG00000130640
ENSG00000130653
ENSG00000130675
ENSG00000130684
ENSG00000130695

ENSG00000130700
ENSG00000130701
ENSG00000130703
ENSG00000130706
ENSG00000130707
ENSG00000130711
ENSG00000130713
ENSG00000130714
ENSG00000130717
ENSG00000130720
ENSG00000130723
ENSG00000130725
ENSG00000130731
ENSG00000130741
ENSG00000130749
ENSG00000130751
ENSG00000130758
ENSG00000130766
ENSG00000130768
ENSG00000130770
ENSG00000130772
ENSG00000130775
ENSG00000130779
ENSG00000130783
ENSG00000130787
ENSG00000130803
ENSG00000130810
ENSG00000130813
ENSG00000130818
ENSG00000130821
ENSG00000130822
ENSG00000130826
ENSG00000130827
ENSG00000130829
ENSG00000130830
ENSG00000130844
ENSG00000130856
ENSG00000130881

ENSG00000130921
ENSG00000130940
ENSG00000130943
ENSG00000130948
ENSG00000130950
ENSG00000130956
ENSG00000130957
ENSG00000130958
ENSG00000130962
ENSG00000130988
ENSG00000130997
ENSG00000131018
ENSG00000131019
ENSG00000131023
ENSG00000131037
ENSG00000131043
ENSG00000131044
ENSG00000131051
ENSG00000131059
ENSG00000131061
ENSG00000131067
ENSG00000131068
ENSG00000131069
ENSG00000131080
ENSG00000131089
ENSG00000131094
ENSG00000131095
ENSG00000131100
ENSG00000131116
ENSG00000131142
ENSG00000131143
ENSG00000131148
ENSG00000131149
ENSG00000131153
ENSG00000131165
ENSG00000131171
ENSG00000131183
ENSG00000131187

ENSG00000131188
ENSG00000131196
ENSG00000131233
ENSG00000131236
ENSG00000131238
ENSG00000131242
ENSG00000131263
ENSG00000131323
ENSG00000131368
ENSG00000131370
ENSG00000131374
ENSG00000131375
ENSG00000131378
ENSG00000131381
ENSG00000131386
ENSG00000131389
ENSG00000131398
ENSG00000131409
ENSG00000131435
ENSG00000131437
ENSG00000131446
ENSG00000131459
ENSG00000131467
ENSG00000131470
ENSG00000131471
ENSG00000131475
ENSG00000131482
ENSG00000131495
ENSG00000131503
ENSG00000131504
ENSG00000131507
ENSG00000131508
ENSG00000131558
ENSG00000131584
ENSG00000131591
ENSG00000131620
ENSG00000131626
ENSG00000131634

ENSG00000131650
ENSG00000131652
ENSG00000131653
ENSG00000131669
ENSG00000131697
ENSG00000131711
ENSG00000131724
ENSG00000131737
ENSG00000131738
ENSG00000131746
ENSG00000131747
ENSG00000131748
ENSG00000131759
ENSG00000131771
ENSG00000131773
ENSG00000131779
ENSG00000131781
ENSG00000131788
ENSG00000131791
ENSG00000131808
ENSG00000131828
ENSG00000131831
ENSG00000131844
ENSG00000131845
ENSG00000131848
ENSG00000131849
ENSG00000131871
ENSG00000131873
ENSG00000131899
ENSG00000131910
ENSG00000131914
ENSG00000131931
ENSG00000131941
ENSG00000131943
ENSG00000131944
ENSG00000131966
ENSG00000131979
ENSG00000132000

ENSG00000132002
ENSG00000132003
ENSG00000132004
ENSG00000132005
ENSG00000132010
ENSG00000132016
ENSG00000132017
ENSG00000132026
ENSG00000132109
ENSG00000132122
ENSG00000132128
ENSG00000132155
ENSG00000132164
ENSG00000132170
ENSG00000132182
ENSG00000132185
ENSG00000132196
ENSG00000132199
ENSG00000132205
ENSG00000132254
ENSG00000132274
ENSG00000132275
ENSG00000132286
ENSG00000132294
ENSG00000132297
ENSG00000132300
ENSG00000132305
ENSG00000132313
ENSG00000132321
ENSG00000132326
ENSG00000132329
ENSG00000132330
ENSG00000132334
ENSG00000132341
ENSG00000132356
ENSG00000132357
ENSG00000132359
ENSG00000132361

ENSG00000132376
ENSG00000132382
ENSG00000132383
ENSG00000132388
ENSG00000132394
ENSG00000132405
ENSG00000132406
ENSG00000132424
ENSG00000132434
ENSG00000132436
ENSG00000132437
ENSG00000132463
ENSG00000132464
ENSG00000132465
ENSG00000132466
ENSG00000132467
ENSG00000132471
ENSG00000132475
ENSG00000132478
ENSG00000132481
ENSG00000132485
ENSG00000132507
ENSG00000132510
ENSG00000132517
ENSG00000132518
ENSG00000132530
ENSG00000132535
ENSG00000132541
ENSG00000132549
ENSG00000132563
ENSG00000132570
ENSG00000132581
ENSG00000132589
ENSG00000132591
ENSG00000132603
ENSG00000132604
ENSG00000132612
ENSG00000132613

ENSG00000132622
ENSG00000132623
ENSG00000132631
ENSG00000132639
ENSG00000132640
ENSG00000132664
ENSG00000132669
ENSG00000132670
ENSG00000132676
ENSG00000132677
ENSG00000132680
ENSG00000132681
ENSG00000132692
ENSG00000132693
ENSG00000132694
ENSG00000132704
ENSG00000132716
ENSG00000132718
ENSG00000132740
ENSG00000132746
ENSG00000132749
ENSG00000132763
ENSG00000132768
ENSG00000132780
ENSG00000132793
ENSG00000132801
ENSG00000132819
ENSG00000132821
ENSG00000132823
ENSG00000132824
ENSG00000132825
ENSG00000132840
ENSG00000132842
ENSG00000132846
ENSG00000132849
ENSG00000132854
ENSG00000132872
ENSG00000132874

ENSG00000132879
ENSG00000132906
ENSG00000132911
ENSG00000132912
ENSG00000132915
ENSG00000132932
ENSG00000132938
ENSG00000132950
ENSG00000132952
ENSG00000132953
ENSG00000132964
ENSG00000132970
ENSG00000132975
ENSG00000133020
ENSG00000133026
ENSG00000133027
ENSG00000133030
ENSG00000133055
ENSG00000133056
ENSG00000133059
ENSG00000133063
ENSG00000133065
ENSG00000133067
ENSG00000133069
ENSG00000133083
ENSG00000133103
ENSG00000133104
ENSG00000133105
ENSG00000133106
ENSG00000133107
ENSG00000133110
ENSG00000133111
ENSG00000133112
ENSG00000133114
ENSG00000133116
ENSG00000133119
ENSG00000133121
ENSG00000133131

ENSG00000133134
ENSG00000133135
ENSG00000133138
ENSG00000133193
ENSG00000133195
ENSG00000133216
ENSG00000133226
ENSG00000133247
ENSG00000133250
ENSG00000133265
ENSG00000133313
ENSG00000133317
ENSG00000133318
ENSG00000133392
ENSG00000133393
ENSG00000133398
ENSG00000133401
ENSG00000133422
ENSG00000133424
ENSG00000133433
ENSG00000133454
ENSG00000133460
ENSG00000133466
ENSG00000133475
ENSG00000133477
ENSG00000133488
ENSG00000133561
ENSG00000133574
ENSG00000133606
ENSG00000133612
ENSG00000133619
ENSG00000133627
ENSG00000133639
ENSG00000133641
ENSG00000133657
ENSG00000133678
ENSG00000133687
ENSG00000133703

ENSG00000133704
ENSG00000133706
ENSG00000133740
ENSG00000133773
ENSG00000133789
ENSG00000133794
ENSG00000133800
ENSG00000133805
ENSG00000133808
ENSG00000133812
ENSG00000133816
ENSG00000133835
ENSG00000133872
ENSG00000133874
ENSG00000133878
ENSG00000133884
ENSG00000133935
ENSG00000133943
ENSG00000133961
ENSG00000133980
ENSG00000133983
ENSG00000133985
ENSG00000133997
ENSG00000134001
ENSG00000134013
ENSG00000134014
ENSG00000134020
ENSG00000134028
ENSG00000134030
ENSG00000134042
ENSG00000134046
ENSG00000134049
ENSG00000134057
ENSG00000134061
ENSG00000134070
ENSG00000134072
ENSG00000134077
ENSG00000134086

ENSG00000134107
ENSG00000134108
ENSG00000134109
ENSG00000134115
ENSG00000134121
ENSG00000134138
ENSG00000134146
ENSG00000134160
ENSG00000134186
ENSG00000134193
ENSG00000134198
ENSG00000134200
ENSG00000134201
ENSG00000134202
ENSG00000134207
ENSG00000134215
ENSG00000134240
ENSG00000134243
ENSG00000134245
ENSG00000134247
ENSG00000134249
ENSG00000134250
ENSG00000134253
ENSG00000134255
ENSG00000134256
ENSG00000134258
ENSG00000134265
ENSG00000134278
ENSG00000134283
ENSG00000134285
ENSG00000134287
ENSG00000134291
ENSG00000134294
ENSG00000134308
ENSG00000134317
ENSG00000134318
ENSG00000134321
ENSG00000134323

ENSG00000134324
ENSG00000134326
ENSG00000134333
ENSG00000134339
ENSG00000134343
ENSG00000134352
ENSG00000134369
ENSG00000134371
ENSG00000134375
ENSG00000134389
ENSG00000134398
ENSG00000134419
ENSG00000134438
ENSG00000134444
ENSG00000134452
ENSG00000134453
ENSG00000134460
ENSG00000134461
ENSG00000134470
ENSG00000134489
ENSG00000134490
ENSG00000134504
ENSG00000134508
ENSG00000134516
ENSG00000134531
ENSG00000134532
ENSG00000134533
ENSG00000134538
ENSG00000134539
ENSG00000134548
ENSG00000134551
ENSG00000134569
ENSG00000134574
ENSG00000134575
ENSG00000134590
ENSG00000134602
ENSG00000134627
ENSG00000134640

ENSG00000134644
ENSG00000134668
ENSG00000134684
ENSG00000134686
ENSG00000134690
ENSG00000134698
ENSG00000134709
ENSG00000134716
ENSG00000134717
ENSG00000134744
ENSG00000134748
ENSG00000134755
ENSG00000134757
ENSG00000134758
ENSG00000134760
ENSG00000134762
ENSG00000134765
ENSG00000134769
ENSG00000134775
ENSG00000134779
ENSG00000134780
ENSG00000134802
ENSG00000134809
ENSG00000134815
ENSG00000134817
ENSG00000134824
ENSG00000134851
ENSG00000134852
ENSG00000134853
ENSG00000134864
ENSG00000134873
ENSG00000134874
ENSG00000134882
ENSG00000134897
ENSG00000134899
ENSG00000134909
ENSG00000134910
ENSG00000134917

ENSG00000134954
ENSG00000134962
ENSG00000134970
ENSG00000134982
ENSG00000134986
ENSG00000134987
ENSG00000134996
ENSG00000135002
ENSG00000135018
ENSG00000135040
ENSG00000135045
ENSG00000135047
ENSG00000135048
ENSG00000135049
ENSG00000135052
ENSG00000135063
ENSG00000135069
ENSG00000135070
ENSG00000135074
ENSG00000135077
ENSG00000135083
ENSG00000135090
ENSG00000135093
ENSG00000135094
ENSG00000135097
ENSG00000135100
ENSG00000135108
ENSG00000135111
ENSG00000135114
ENSG00000135116
ENSG00000135119
ENSG00000135124
ENSG00000135127
ENSG00000135144
ENSG00000135148
ENSG00000135164
ENSG00000135185
ENSG00000135218

ENSG00000135220
ENSG00000135226
ENSG00000135241
ENSG00000135245
ENSG00000135248
ENSG00000135250
ENSG00000135272
ENSG00000135297
ENSG00000135299
ENSG00000135312
ENSG00000135315
ENSG00000135316
ENSG00000135318
ENSG00000135324
ENSG00000135334
ENSG00000135338
ENSG00000135341
ENSG00000135362
ENSG00000135363
ENSG00000135365
ENSG00000135372
ENSG00000135373
ENSG00000135374
ENSG00000135378
ENSG00000135387
ENSG00000135392
ENSG00000135404
ENSG00000135406
ENSG00000135409
ENSG00000135414
ENSG00000135423
ENSG00000135424
ENSG00000135426
ENSG00000135436
ENSG00000135439
ENSG00000135443
ENSG00000135446
ENSG00000135447

ENSG00000135452
ENSG00000135454
ENSG00000135457
ENSG00000135469
ENSG00000135472
ENSG00000135473
ENSG00000135482
ENSG00000135502
ENSG00000135503
ENSG00000135506
ENSG00000135517
ENSG00000135519
ENSG00000135521
ENSG00000135525
ENSG00000135535
ENSG00000135537
ENSG00000135540
ENSG00000135541
ENSG00000135547
ENSG00000135597
ENSG00000135604
ENSG00000135605
ENSG00000135622
ENSG00000135624
ENSG00000135625
ENSG00000135631
ENSG00000135632
ENSG00000135637
ENSG00000135638
ENSG00000135643
ENSG00000135655
ENSG00000135677
ENSG00000135678
ENSG00000135679
ENSG00000135686
ENSG00000135697
ENSG00000135702
ENSG00000135709

ENSG00000135720
ENSG00000135722
ENSG00000135736
ENSG00000135740
ENSG00000135744
ENSG00000135766
ENSG00000135775
ENSG00000135776
ENSG00000135778
ENSG00000135801
ENSG00000135821
ENSG00000135823
ENSG00000135824
ENSG00000135828
ENSG00000135837
ENSG00000135838
ENSG00000135842
ENSG00000135845
ENSG00000135862
ENSG00000135870
ENSG00000135898
ENSG00000135902
ENSG00000135903
ENSG00000135905
ENSG00000135912
ENSG00000135913
ENSG00000135916
ENSG00000135917
ENSG00000135919
ENSG00000135924
ENSG00000135925
ENSG00000135926
ENSG00000135930
ENSG00000135931
ENSG00000135932
ENSG00000135940
ENSG00000135951
ENSG00000135953

ENSG00000135956
ENSG00000135960
ENSG00000135966
ENSG00000135968
ENSG00000135973
ENSG00000135974
ENSG00000135999
ENSG00000136002
ENSG00000136003
ENSG00000136010
ENSG00000136014
ENSG00000136021
ENSG00000136026
ENSG00000136040
ENSG00000136044
ENSG00000136045
ENSG00000136048
ENSG00000136051
ENSG00000136068
ENSG00000136098
ENSG00000136099
ENSG00000136100
ENSG00000136104
ENSG00000136108
ENSG00000136111
ENSG00000136114
ENSG00000136122
ENSG00000136141
ENSG00000136143
ENSG00000136144
ENSG00000136146
ENSG00000136152
ENSG00000136153
ENSG00000136155
ENSG00000136158
ENSG00000136159
ENSG00000136160
ENSG00000136161

ENSG00000136167
ENSG00000136169
ENSG00000136193
ENSG00000136197
ENSG00000136205
ENSG00000136206
ENSG00000136213
ENSG00000136235
ENSG00000136237
ENSG00000136238
ENSG00000136240
ENSG00000136244
ENSG00000136250
ENSG00000136261
ENSG00000136267
ENSG00000136270
ENSG00000136271
ENSG00000136273
ENSG00000136279
ENSG00000136280
ENSG00000136295
ENSG00000136297
ENSG00000136319
ENSG00000136327
ENSG00000136352
ENSG00000136367
ENSG00000136371
ENSG00000136379
ENSG00000136381
ENSG00000136383
ENSG00000136404
ENSG00000136425
ENSG00000136436
ENSG00000136444
ENSG00000136448
ENSG00000136450
ENSG00000136451
ENSG00000136457

ENSG00000136478
ENSG00000136485
ENSG00000136487
ENSG00000136490
ENSG00000136492
ENSG00000136504
ENSG00000136518
ENSG00000136521
ENSG00000136527
ENSG00000136531
ENSG00000136535
ENSG00000136536
ENSG00000136541
ENSG00000136546
ENSG00000136574
ENSG00000136603
ENSG00000136634
ENSG00000136643
ENSG00000136688
ENSG00000136689
ENSG00000136695
ENSG00000136696
ENSG00000136697
ENSG00000136698
ENSG00000136699
ENSG00000136709
ENSG00000136710
ENSG00000136715
ENSG00000136718
ENSG00000136720
ENSG00000136731
ENSG00000136732
ENSG00000136738
ENSG00000136750
ENSG00000136754
ENSG00000136758
ENSG00000136783
ENSG00000136802

ENSG00000136811
ENSG00000136813
ENSG00000136816
ENSG00000136819
ENSG00000136824
ENSG00000136827
ENSG00000136828
ENSG00000136830
ENSG00000136840
ENSG00000136842
ENSG00000136848
ENSG00000136854
ENSG00000136856
ENSG00000136859
ENSG00000136861
ENSG00000136867
ENSG00000136868
ENSG00000136869
ENSG00000136870
ENSG00000136872
ENSG00000136874
ENSG00000136875
ENSG00000136877
ENSG00000136878
ENSG00000136881
ENSG00000136883
ENSG00000136888
ENSG00000136897
ENSG00000136908
ENSG00000136925
ENSG00000136928
ENSG00000136931
ENSG00000136932
ENSG00000136935
ENSG00000136936
ENSG00000136940
ENSG00000136943
ENSG00000136944

ENSG00000136950
ENSG00000136960
ENSG00000136986
ENSG00000136999
ENSG00000137033
ENSG00000137038
ENSG00000137040
ENSG00000137054
ENSG00000137055
ENSG00000137070
ENSG00000137073
ENSG00000137074
ENSG00000137075
ENSG00000137077
ENSG00000137094
ENSG00000137100
ENSG00000137101
ENSG00000137103
ENSG00000137106
ENSG00000137124
ENSG00000137135
ENSG00000137142
ENSG00000137145
ENSG00000137161
ENSG00000137166
ENSG00000137168
ENSG00000137171
ENSG00000137177
ENSG00000137193
ENSG00000137198
ENSG00000137200
ENSG00000137203
ENSG00000137204
ENSG00000137210
ENSG00000137216
ENSG00000137221
ENSG00000137225
ENSG00000137261

ENSG00000137265
ENSG00000137266
ENSG00000137269
ENSG00000137270
ENSG00000137273
ENSG00000137274
ENSG00000137275
ENSG00000137288
ENSG00000137309
ENSG00000137310
ENSG00000137331
ENSG00000137337
ENSG00000137343
ENSG00000137364
ENSG00000137393
ENSG00000137404
ENSG00000137409
ENSG00000137413
ENSG00000137414
ENSG00000137440
ENSG00000137441
ENSG00000137449
ENSG00000137460
ENSG00000137462
ENSG00000137473
ENSG00000137474
ENSG00000137478
ENSG00000137486
ENSG00000137491
ENSG00000137492
ENSG00000137494
ENSG00000137496
ENSG00000137500
ENSG00000137501
ENSG00000137502
ENSG00000137504
ENSG00000137507
ENSG00000137509

ENSG00000137513
ENSG00000137522
ENSG00000137547
ENSG00000137558
ENSG00000137561
ENSG00000137571
ENSG00000137573
ENSG00000137574
ENSG00000137575
ENSG00000137628
ENSG00000137642
ENSG00000137648
ENSG00000137656
ENSG00000137691
ENSG00000137692
ENSG00000137693
ENSG00000137699
ENSG00000137707
ENSG00000137709
ENSG00000137710
ENSG00000137713
ENSG00000137714
ENSG00000137720
ENSG00000137726
ENSG00000137727
ENSG00000137731
ENSG00000137745
ENSG00000137747
ENSG00000137752
ENSG00000137760
ENSG00000137764
ENSG00000137770
ENSG00000137776
ENSG00000137801
ENSG00000137802
ENSG00000137807
ENSG00000137809
ENSG00000137814

ENSG00000137815
ENSG00000137817
ENSG00000137819
ENSG00000137822
ENSG00000137824
ENSG00000137825
ENSG00000137831
ENSG00000137841
ENSG00000137842
ENSG00000137843
ENSG00000137845
ENSG00000137868
ENSG00000137869
ENSG00000137871
ENSG00000137872
ENSG00000137875
ENSG00000137876
ENSG00000137877
ENSG00000137936
ENSG00000137941
ENSG00000137942
ENSG00000137944
ENSG00000137947
ENSG00000137959
ENSG00000137960
ENSG00000137962
ENSG00000137965
ENSG00000137968
ENSG00000137975
ENSG00000137992
ENSG00000137996
ENSG00000138018
ENSG00000138028
ENSG00000138030
ENSG00000138031
ENSG00000138032
ENSG00000138036
ENSG00000138039

ENSG00000138050
ENSG00000138061
ENSG00000138069
ENSG00000138071
ENSG00000138073
ENSG00000138074
ENSG00000138075
ENSG00000138078
ENSG00000138079
ENSG00000138081
ENSG00000138083
ENSG00000138085
ENSG00000138092
ENSG00000138095
ENSG00000138100
ENSG00000138101
ENSG00000138107
ENSG00000138109
ENSG00000138111
ENSG00000138138
ENSG00000138152
ENSG00000138160
ENSG00000138166
ENSG00000138172
ENSG00000138175
ENSG00000138180
ENSG00000138185
ENSG00000138193
ENSG00000138246
ENSG00000138279
ENSG00000138286
ENSG00000138303
ENSG00000138308
ENSG00000138311
ENSG00000138316
ENSG00000138326
ENSG00000138336
ENSG00000138346

ENSG00000138347
ENSG00000138356
ENSG00000138376
ENSG00000138380
ENSG00000138395
ENSG00000138398
ENSG00000138399
ENSG00000138400
ENSG00000138413
ENSG00000138430
ENSG00000138434
ENSG00000138435
ENSG00000138439
ENSG00000138443
ENSG00000138448
ENSG00000138449
ENSG00000138463
ENSG00000138468
ENSG00000138472
ENSG00000138496
ENSG00000138592
ENSG00000138593
ENSG00000138594
ENSG00000138604
ENSG00000138606
ENSG00000138613
ENSG00000138614
ENSG00000138617
ENSG00000138621
ENSG00000138622
ENSG00000138623
ENSG00000138629
ENSG00000138639
ENSG00000138640
ENSG00000138641
ENSG00000138642
ENSG00000138650
ENSG00000138660

ENSG00000138663
ENSG00000138668
ENSG00000138669
ENSG00000138670
ENSG00000138675
ENSG00000138678
ENSG00000138684
ENSG00000138685
ENSG00000138696
ENSG00000138698
ENSG00000138709
ENSG00000138722
ENSG00000138735
ENSG00000138738
ENSG00000138741
ENSG00000138744
ENSG00000138750
ENSG00000138755
ENSG00000138756
ENSG00000138757
ENSG00000138758
ENSG00000138759
ENSG00000138760
ENSG00000138764
ENSG00000138767
ENSG00000138768
ENSG00000138769
ENSG00000138771
ENSG00000138777
ENSG00000138780
ENSG00000138792
ENSG00000138794
ENSG00000138795
ENSG00000138796
ENSG00000138801
ENSG00000138802
ENSG00000138814
ENSG00000138821

ENSG00000138823
ENSG00000138829
ENSG00000138834
ENSG00000138835
ENSG00000138867
ENSG00000138892
ENSG00000138942
ENSG00000138944
ENSG00000138964
ENSG00000139044
ENSG00000139055
ENSG00000139083
ENSG00000139112
ENSG00000139117
ENSG00000139131
ENSG00000139132
ENSG00000139133
ENSG00000139146
ENSG00000139151
ENSG00000139154
ENSG00000139155
ENSG00000139160
ENSG00000139163
ENSG00000139168
ENSG00000139173
ENSG00000139174
ENSG00000139178
ENSG00000139180
ENSG00000139182
ENSG00000139187
ENSG00000139190
ENSG00000139192
ENSG00000139193
ENSG00000139194
ENSG00000139197
ENSG00000139200
ENSG00000139209
ENSG00000139211

ENSG00000139218
ENSG00000139220
ENSG00000139233
ENSG00000139266
ENSG00000139269
ENSG00000139278
ENSG00000139287
ENSG00000139289
ENSG00000139291
ENSG00000139292
ENSG00000139304
ENSG00000139318
ENSG00000139323
ENSG00000139324
ENSG00000139329
ENSG00000139344
ENSG00000139350
ENSG00000139352
ENSG00000139354
ENSG00000139364
ENSG00000139370
ENSG00000139372
ENSG00000139405
ENSG00000139410
ENSG00000139428
ENSG00000139433
ENSG00000139436
ENSG00000139437
ENSG00000139438
ENSG00000139445
ENSG00000139496
ENSG00000139505
ENSG00000139508
ENSG00000139514
ENSG00000139515
ENSG00000139517
ENSG00000139531
ENSG00000139546

ENSG00000139547
ENSG00000139549
ENSG00000139567
ENSG00000139579
ENSG00000139597
ENSG00000139613
ENSG00000139618
ENSG00000139620
ENSG00000139624
ENSG00000139625
ENSG00000139626
ENSG00000139629
ENSG00000139631
ENSG00000139636
ENSG00000139641
ENSG00000139644
ENSG00000139645
ENSG00000139648
ENSG00000139651
ENSG00000139656
ENSG00000139668
ENSG00000139687
ENSG00000139697
ENSG00000139718
ENSG00000139719
ENSG00000139722
ENSG00000139725
ENSG00000139726
ENSG00000139734
ENSG00000139737
ENSG00000139746
ENSG00000139767
ENSG00000139793
ENSG00000139797
ENSG00000139800
ENSG00000139826
ENSG00000139835
ENSG00000139842

ENSG00000139874
ENSG00000139880
ENSG00000139890
ENSG00000139899
ENSG00000139908
ENSG00000139910
ENSG00000139915
ENSG00000139926
ENSG00000139946
ENSG00000139970
ENSG00000139974
ENSG00000139977
ENSG00000139985
ENSG00000139988
ENSG00000139990
ENSG00000139998
ENSG00000140006
ENSG00000140009
ENSG00000140015
ENSG00000140022
ENSG00000140025
ENSG00000140030
ENSG00000140043
ENSG00000140044
ENSG00000140067
ENSG00000140090
ENSG00000140092
ENSG00000140093
ENSG00000140104
ENSG00000140105
ENSG00000140107
ENSG00000140153
ENSG00000140157
ENSG00000140199
ENSG00000140254
ENSG00000140259
ENSG00000140262
ENSG00000140263

ENSG00000140264
ENSG00000140265
ENSG00000140279
ENSG00000140280
ENSG00000140285
ENSG00000140297
ENSG00000140299
ENSG00000140307
ENSG00000140319
ENSG00000140320
ENSG00000140323
ENSG00000140326
ENSG00000140332
ENSG00000140350
ENSG00000140365
ENSG00000140367
ENSG00000140368
ENSG00000140374
ENSG00000140382
ENSG00000140386
ENSG00000140391
ENSG00000140396
ENSG00000140400
ENSG00000140403
ENSG00000140406
ENSG00000140416
ENSG00000140443
ENSG00000140450
ENSG00000140451
ENSG00000140455
ENSG00000140463
ENSG00000140464
ENSG00000140465
ENSG00000140470
ENSG00000140474
ENSG00000140478
ENSG00000140479
ENSG00000140481

ENSG00000140488
ENSG00000140497
ENSG00000140511
ENSG00000140521
ENSG00000140525
ENSG00000140526
ENSG00000140527
ENSG00000140538
ENSG00000140543
ENSG00000140545
ENSG00000140548
ENSG00000140553
ENSG00000140557
ENSG00000140563
ENSG00000140564
ENSG00000140575
ENSG00000140577
ENSG00000140598
ENSG00000140600
ENSG00000140612
ENSG00000140632
ENSG00000140650
ENSG00000140678
ENSG00000140682
ENSG00000140688
ENSG00000140691
ENSG00000140694
ENSG00000140718
ENSG00000140743
ENSG00000140750
ENSG00000140795
ENSG00000140807
ENSG00000140829
ENSG00000140830
ENSG00000140832
ENSG00000140835
ENSG00000140836
ENSG00000140839

ENSG00000140848
ENSG00000140853
ENSG00000140859
ENSG00000140873
ENSG00000140905
ENSG00000140931
ENSG00000140932
ENSG00000140937
ENSG00000140939
ENSG00000140941
ENSG00000140943
ENSG00000140945
ENSG00000140948
ENSG00000140950
ENSG00000140955
ENSG00000140961
ENSG00000140968
ENSG00000140983
ENSG00000140987
ENSG00000140992
ENSG00000140995
ENSG00000141002
ENSG00000141012
ENSG00000141013
ENSG00000141026
ENSG00000141027
ENSG00000141030
ENSG00000141034
ENSG00000141040
ENSG00000141052
ENSG00000141068
ENSG00000141084
ENSG00000141086
ENSG00000141098
ENSG00000141101
ENSG00000141161
ENSG00000141179
ENSG00000141219

ENSG00000141252
ENSG00000141258
ENSG00000141293
ENSG00000141294
ENSG00000141295
ENSG00000141298
ENSG00000141314
ENSG00000141337
ENSG00000141349
ENSG00000141367
ENSG00000141376
ENSG00000141380
ENSG00000141384
ENSG00000141385
ENSG00000141391
ENSG00000141401
ENSG00000141404
ENSG00000141424
ENSG00000141425
ENSG00000141428
ENSG00000141429
ENSG00000141431
ENSG00000141433
ENSG00000141437
ENSG00000141441
ENSG00000141446
ENSG00000141447
ENSG00000141448
ENSG00000141458
ENSG00000141469
ENSG00000141480
ENSG00000141485
ENSG00000141497
ENSG00000141503
ENSG00000141504
ENSG00000141505
ENSG00000141506
ENSG00000141510

ENSG00000141519
ENSG00000141522
ENSG00000141524
ENSG00000141526
ENSG00000141527
ENSG00000141540
ENSG00000141542
ENSG00000141551
ENSG00000141552
ENSG00000141556
ENSG00000141562
ENSG00000141564
ENSG00000141568
ENSG00000141569
ENSG00000141570
ENSG00000141574
ENSG00000141576
ENSG00000141577
ENSG00000141579
ENSG00000141580
ENSG00000141622
ENSG00000141627
ENSG00000141639
ENSG00000141642
ENSG00000141644
ENSG00000141646
ENSG00000141655
ENSG00000141664
ENSG00000141668
ENSG00000141682
ENSG00000141696
ENSG00000141698
ENSG00000141699
ENSG00000141736
ENSG00000141738
ENSG00000141741
ENSG00000141750
ENSG00000141753

ENSG00000141756
ENSG00000141759
ENSG00000141837
ENSG00000141867
ENSG00000141873
ENSG00000141905
ENSG00000141946
ENSG00000141956
ENSG00000141959
ENSG00000141965
ENSG00000141971
ENSG00000141985
ENSG00000142002
ENSG00000142025
ENSG00000142039
ENSG00000142046
ENSG00000142065
ENSG00000142082
ENSG00000142089
ENSG00000142102
ENSG00000142149
ENSG00000142156
ENSG00000142166
ENSG00000142173
ENSG00000142178
ENSG00000142185
ENSG00000142188
ENSG00000142192
ENSG00000142207
ENSG00000142208
ENSG00000142224
ENSG00000142230
ENSG00000142235
ENSG00000142252
ENSG00000142273
ENSG00000142319
ENSG00000142405
ENSG00000142408

ENSG00000142409
ENSG00000142444
ENSG00000142449
ENSG00000142453
ENSG00000142459
ENSG00000142494
ENSG00000142512
ENSG00000142515
ENSG00000142530
ENSG00000142541
ENSG00000142544
ENSG00000142546
ENSG00000142549
ENSG00000142552
ENSG00000142556
ENSG00000142583
ENSG00000142599
ENSG00000142611
ENSG00000142619
ENSG00000142621
ENSG00000142623
ENSG00000142634
ENSG00000142657
ENSG00000142661
ENSG00000142675
ENSG00000142677
ENSG00000142684
ENSG00000142686
ENSG00000142687
ENSG00000142694
ENSG00000142700
ENSG00000142751
ENSG00000142784
ENSG00000142794
ENSG00000142856
ENSG00000142864
ENSG00000142867
ENSG00000142871

ENSG00000142875
ENSG00000142892
ENSG00000142920
ENSG00000142949
ENSG00000142959
ENSG00000142961
ENSG00000142973
ENSG00000143006
ENSG00000143013
ENSG00000143028
ENSG00000143036
ENSG00000143061
ENSG00000143067
ENSG00000143079
ENSG00000143093
ENSG00000143106
ENSG00000143107
ENSG00000143119
ENSG00000143126
ENSG00000143127
ENSG00000143147
ENSG00000143153
ENSG00000143155
ENSG00000143157
ENSG00000143158
ENSG00000143162
ENSG00000143164
ENSG00000143167
ENSG00000143171
ENSG00000143178
ENSG00000143179
ENSG00000143183
ENSG00000143184
ENSG00000143185
ENSG00000143190
ENSG00000143194
ENSG00000143195
ENSG00000143196

ENSG00000143199
ENSG00000143217
ENSG00000143222
ENSG00000143226
ENSG00000143252
ENSG00000143257
ENSG00000143258
ENSG00000143294
ENSG00000143297
ENSG00000143314
ENSG00000143315
ENSG00000143318
ENSG00000143319
ENSG00000143320
ENSG00000143321
ENSG00000143322
ENSG00000143324
ENSG00000143333
ENSG00000143337
ENSG00000143340
ENSG00000143344
ENSG00000143355
ENSG00000143363
ENSG00000143365
ENSG00000143367
ENSG00000143369
ENSG00000143374
ENSG00000143375
ENSG00000143376
ENSG00000143382
ENSG00000143384
ENSG00000143387
ENSG00000143390
ENSG00000143393
ENSG00000143398
ENSG00000143401
ENSG00000143409
ENSG00000143418

ENSG00000143420
ENSG00000143434
ENSG00000143437
ENSG00000143442
ENSG00000143443
ENSG00000143450
ENSG00000143452
ENSG00000143457
ENSG00000143458
ENSG00000143469
ENSG00000143473
ENSG00000143476
ENSG00000143493
ENSG00000143494
ENSG00000143498
ENSG00000143502
ENSG00000143512
ENSG00000143514
ENSG00000143515
ENSG00000143520
ENSG00000143536
ENSG00000143537
ENSG00000143545
ENSG00000143549
ENSG00000143553
ENSG00000143569
ENSG00000143570
ENSG00000143575
ENSG00000143578
ENSG00000143590
ENSG00000143595
ENSG00000143603
ENSG00000143612
ENSG00000143614
ENSG00000143622
ENSG00000143624
ENSG00000143627
ENSG00000143630

ENSG00000143633
ENSG00000143641
ENSG00000143643
ENSG00000143653
ENSG00000143669
ENSG00000143674
ENSG00000143702
ENSG00000143740
ENSG00000143742
ENSG00000143748
ENSG00000143751
ENSG00000143753
ENSG00000143756
ENSG00000143761
ENSG00000143768
ENSG00000143771
ENSG00000143772
ENSG00000143776
ENSG00000143786
ENSG00000143793
ENSG00000143797
ENSG00000143799
ENSG00000143801
ENSG00000143811
ENSG00000143815
ENSG00000143819
ENSG00000143842
ENSG00000143845
ENSG00000143847
ENSG00000143850
ENSG00000143851
ENSG00000143858
ENSG00000143862
ENSG00000143867
ENSG00000143870
ENSG00000143878
ENSG00000143882
ENSG00000143891

ENSG00000143919
ENSG00000143921
ENSG00000143924
ENSG00000143947
ENSG00000143952
ENSG00000143970
ENSG00000143977
ENSG00000143994
ENSG00000143995
ENSG00000144021
ENSG00000144026
ENSG00000144028
ENSG00000144029
ENSG00000144031
ENSG00000144035
ENSG00000144036
ENSG00000144040
ENSG00000144043
ENSG00000144048
ENSG00000144057
ENSG00000144063
ENSG00000144115
ENSG00000144118
ENSG00000144119
ENSG00000144130
ENSG00000144134
ENSG00000144136
ENSG00000144152
ENSG00000144161
ENSG00000144191
ENSG00000144218
ENSG00000144224
ENSG00000144227
ENSG00000144228
ENSG00000144229
ENSG00000144230
ENSG00000144231
ENSG00000144233

ENSG00000144278
ENSG00000144285
ENSG00000144290
ENSG00000144306
ENSG00000144320
ENSG00000144331
ENSG00000144354
ENSG00000144355
ENSG00000144357
ENSG00000144366
ENSG00000144369
ENSG00000144381
ENSG00000144401
ENSG00000144406
ENSG00000144407
ENSG00000144445
ENSG00000144451
ENSG00000144452
ENSG00000144455
ENSG00000144460
ENSG00000144468
ENSG00000144476
ENSG00000144481
ENSG00000144485
ENSG00000144488
ENSG00000144524
ENSG00000144535
ENSG00000144550
ENSG00000144554
ENSG00000144559
ENSG00000144560
ENSG00000144566
ENSG00000144567
ENSG00000144579
ENSG00000144580
ENSG00000144583
ENSG00000144591
ENSG00000144596

ENSG00000144597
ENSG00000144619
ENSG00000144642
ENSG00000144644
ENSG00000144645
ENSG00000144647
ENSG00000144648
ENSG00000144649
ENSG00000144655
ENSG00000144659
ENSG00000144674
ENSG00000144677
ENSG00000144681
ENSG00000144711
ENSG00000144712
ENSG00000144713
ENSG00000144724
ENSG00000144730
ENSG00000144736
ENSG00000144744
ENSG00000144746
ENSG00000144749
ENSG00000144791
ENSG00000144792
ENSG00000144815
ENSG00000144820
ENSG00000144821
ENSG00000144824
ENSG00000144827
ENSG00000144834
ENSG00000144837
ENSG00000144840
ENSG00000144843
ENSG00000144847
ENSG00000144848
ENSG00000144852
ENSG00000144857
ENSG00000144867

ENSG00000144868
ENSG00000144891
ENSG00000144895
ENSG00000144908
ENSG00000144909
ENSG00000144935
ENSG00000144959
ENSG00000145002
ENSG00000145012
ENSG00000145014
ENSG00000145016
ENSG00000145020
ENSG00000145022
ENSG00000145029
ENSG00000145040
ENSG00000145041
ENSG00000145050
ENSG00000145087
ENSG00000145088
ENSG00000145103
ENSG00000145113
ENSG00000145147
ENSG00000145191
ENSG00000145192
ENSG00000145194
ENSG00000145214
ENSG00000145216
ENSG00000145217
ENSG00000145242
ENSG00000145244
ENSG00000145246
ENSG00000145248
ENSG00000145283
ENSG00000145284
ENSG00000145287
ENSG00000145293
ENSG00000145331
ENSG00000145332

ENSG00000145335
ENSG00000145337
ENSG00000145348
ENSG00000145349
ENSG00000145354
ENSG00000145358
ENSG00000145362
ENSG00000145365
ENSG00000145375
ENSG00000145384
ENSG00000145386
ENSG00000145388
ENSG00000145390
ENSG00000145391
ENSG00000145416
ENSG00000145431
ENSG00000145439
ENSG00000145451
ENSG00000145495
ENSG00000145506
ENSG00000145526
ENSG00000145536
ENSG00000145545
ENSG00000145555
ENSG00000145569
ENSG00000145592
ENSG00000145604
ENSG00000145623
ENSG00000145626
ENSG00000145632
ENSG00000145675
ENSG00000145681
ENSG00000145685
ENSG00000145687
ENSG00000145692
ENSG00000145703
ENSG00000145715
ENSG00000145723

ENSG00000145725
ENSG00000145730
ENSG00000145734
ENSG00000145740
ENSG00000145743
ENSG00000145757
ENSG00000145777
ENSG00000145779
ENSG00000145780
ENSG00000145782
ENSG00000145794
ENSG00000145817
ENSG00000145819
ENSG00000145824
ENSG00000145832
ENSG00000145833
ENSG00000145860
ENSG00000145861
ENSG00000145863
ENSG00000145864
ENSG00000145879
ENSG00000145882
ENSG00000145888
ENSG00000145901
ENSG00000145907
ENSG00000145908
ENSG00000145911
ENSG00000145916
ENSG00000145919
ENSG00000145920
ENSG00000145945
ENSG00000145949
ENSG00000145979
ENSG00000145990
ENSG00000145996
ENSG00000146005
ENSG00000146006
ENSG00000146007

ENSG00000146021
ENSG00000146038
ENSG00000146039
ENSG00000146049
ENSG00000146054
ENSG00000146063
ENSG00000146066
ENSG00000146070
ENSG00000146072
ENSG00000146083
ENSG00000146085
ENSG00000146109
ENSG00000146112
ENSG00000146122
ENSG00000146147
ENSG00000146151
ENSG00000146166
ENSG00000146192
ENSG00000146197
ENSG00000146205
ENSG00000146215
ENSG00000146216
ENSG00000146223
ENSG00000146232
ENSG00000146233
ENSG00000146242
ENSG00000146243
ENSG00000146247
ENSG00000146250
ENSG00000146263
ENSG00000146267
ENSG00000146276
ENSG00000146278
ENSG00000146281
ENSG00000146282
ENSG00000146285
ENSG00000146350
ENSG00000146352

ENSG00000146360
ENSG00000146373
ENSG00000146374
ENSG00000146409
ENSG00000146410
ENSG00000146411
ENSG00000146414
ENSG00000146416
ENSG00000146426
ENSG00000146433
ENSG00000146453
ENSG00000146457
ENSG00000146463
ENSG00000146469
ENSG00000146476
ENSG00000146477
ENSG00000146535
ENSG00000146540
ENSG00000146555
ENSG00000146576
ENSG00000146587
ENSG00000146592
ENSG00000146648
ENSG00000146670
ENSG00000146674
ENSG00000146676
ENSG00000146678
ENSG00000146700
ENSG00000146701
ENSG00000146707
ENSG00000146731
ENSG00000146733
ENSG00000146755
ENSG00000146776
ENSG00000146802
ENSG00000146809
ENSG00000146826
ENSG00000146828

ENSG00000146830
ENSG00000146833
ENSG00000146834
ENSG00000146842
ENSG00000146856
ENSG00000146858
ENSG00000146859
ENSG00000146872
ENSG00000146904
ENSG00000146909
ENSG00000146910
ENSG00000146918
ENSG00000146926
ENSG00000146938
ENSG00000146950
ENSG00000146955
ENSG00000146963
ENSG00000146966
ENSG00000147003
ENSG00000147010
ENSG00000147027
ENSG00000147036
ENSG00000147041
ENSG00000147044
ENSG00000147059
ENSG00000147065
ENSG00000147099
ENSG00000147100
ENSG00000147113
ENSG00000147117
ENSG00000147118
ENSG00000147121
ENSG00000147124
ENSG00000147130
ENSG00000147133
ENSG00000147138
ENSG00000147140
ENSG00000147144

ENSG00000147145
ENSG00000147155
ENSG00000147160
ENSG00000147162
ENSG00000147164
ENSG00000147168
ENSG00000147183
ENSG00000147202
ENSG00000147223
ENSG00000147234
ENSG00000147246
ENSG00000147251
ENSG00000147255
ENSG00000147256
ENSG00000147257
ENSG00000147262
ENSG00000147274
ENSG00000147316
ENSG00000147324
ENSG00000147364
ENSG00000147378
ENSG00000147383
ENSG00000147394
ENSG00000147400
ENSG00000147403
ENSG00000147408
ENSG00000147416
ENSG00000147419
ENSG00000147421
ENSG00000147434
ENSG00000147439
ENSG00000147443
ENSG00000147454
ENSG00000147457
ENSG00000147459
ENSG00000147465
ENSG00000147471
ENSG00000147475

ENSG00000147481
ENSG00000147488
ENSG00000147509
ENSG00000147526
ENSG00000147533
ENSG00000147535
ENSG00000147536
ENSG00000147548
ENSG00000147570
ENSG00000147588
ENSG00000147596
ENSG00000147601
ENSG00000147606
ENSG00000147614
ENSG00000147647
ENSG00000147649
ENSG00000147650
ENSG00000147655
ENSG00000147669
ENSG00000147676
ENSG00000147679
ENSG00000147689
ENSG00000147697
ENSG00000147724
ENSG00000147789
ENSG00000147799
ENSG00000147852
ENSG00000147853
ENSG00000147854
ENSG00000147862
ENSG00000147872
ENSG00000147874
ENSG00000147883
ENSG00000147889
ENSG00000147894
ENSG00000147905
ENSG00000147912
ENSG00000147955

ENSG00000147996
ENSG00000148019
ENSG00000148053
ENSG00000148082
ENSG00000148090
ENSG00000148110
ENSG00000148120
ENSG00000148123
ENSG00000148143
ENSG00000148154
ENSG00000148158
ENSG00000148175
ENSG00000148180
ENSG00000148187
ENSG00000148200
ENSG00000148204
ENSG00000148218
ENSG00000148219
ENSG00000148225
ENSG00000148229
ENSG00000148248
ENSG00000148288
ENSG00000148296
ENSG00000148297
ENSG00000148300
ENSG00000148308
ENSG00000148331
ENSG00000148334
ENSG00000148335
ENSG00000148339
ENSG00000148341
ENSG00000148343
ENSG00000148344
ENSG00000148356
ENSG00000148358
ENSG00000148362
ENSG00000148377
ENSG00000148384

ENSG00000148396
ENSG00000148399
ENSG00000148400
ENSG00000148408
ENSG00000148411
ENSG00000148426
ENSG00000148429
ENSG00000148444
ENSG00000148450
ENSG00000148468
ENSG00000148483
ENSG00000148484
ENSG00000148488
ENSG00000148498
ENSG00000148513
ENSG00000148516
ENSG00000148541
ENSG00000148572
ENSG00000148584
ENSG00000148600
ENSG00000148604
ENSG00000148606
ENSG00000148634
ENSG00000148660
ENSG00000148672
ENSG00000148677
ENSG00000148680
ENSG00000148688
ENSG00000148690
ENSG00000148700
ENSG00000148702
ENSG00000148704
ENSG00000148719
ENSG00000148730
ENSG00000148735
ENSG00000148737
ENSG00000148773
ENSG00000148798

ENSG00000148803
ENSG00000148814
ENSG00000148824
ENSG00000148826
ENSG00000148832
ENSG00000148835
ENSG00000148840
ENSG00000148841
ENSG00000148843
ENSG00000148848
ENSG00000148908
ENSG00000148925
ENSG00000148926
ENSG00000148935
ENSG00000148942
ENSG00000148943
ENSG00000148948
ENSG00000148950
ENSG00000148985
ENSG00000149054
ENSG00000149090
ENSG00000149091
ENSG00000149115
ENSG00000149124
ENSG00000149150
ENSG00000149177
ENSG00000149179
ENSG00000149182
ENSG00000149187
ENSG00000149196
ENSG00000149212
ENSG00000149218
ENSG00000149256
ENSG00000149257
ENSG00000149260
ENSG00000149269
ENSG00000149273
ENSG00000149289

ENSG00000149294
ENSG00000149295
ENSG00000149300
ENSG00000149305
ENSG00000149308
ENSG00000149311
ENSG00000149313
ENSG00000149328
ENSG00000149346
ENSG00000149357
ENSG00000149380
ENSG00000149403
ENSG00000149418
ENSG00000149428
ENSG00000149451
ENSG00000149452
ENSG00000149474
ENSG00000149476
ENSG00000149480
ENSG00000149483
ENSG00000149485
ENSG00000149503
ENSG00000149516
ENSG00000149527
ENSG00000149532
ENSG00000149534
ENSG00000149541
ENSG00000149547
ENSG00000149548
ENSG00000149554
ENSG00000149557
ENSG00000149571
ENSG00000149573
ENSG00000149575
ENSG00000149577
ENSG00000149582
ENSG00000149591
ENSG00000149596

ENSG00000149599
ENSG00000149600
ENSG00000149633
ENSG00000149635
ENSG00000149636
ENSG00000149639
ENSG00000149646
ENSG00000149654
ENSG00000149657
ENSG00000149658
ENSG00000149679
ENSG00000149716
ENSG00000149742
ENSG00000149781
ENSG00000149782
ENSG00000149792
ENSG00000149798
ENSG00000149823
ENSG00000149922
ENSG00000149923
ENSG00000149925
ENSG00000149926
ENSG00000149927
ENSG00000149929
ENSG00000149930
ENSG00000149948
ENSG00000149968
ENSG00000149970
ENSG00000149972
ENSG00000150048
ENSG00000150054
ENSG00000150076
ENSG00000150093
ENSG00000150201
ENSG00000150244
ENSG00000150275
ENSG00000150281
ENSG00000150316

ENSG00000150337
ENSG00000150347
ENSG00000150394
ENSG00000150401
ENSG00000150403
ENSG00000150433
ENSG00000150455
ENSG00000150457
ENSG00000150459
ENSG00000150471
ENSG00000150477
ENSG00000150510
ENSG00000150526
ENSG00000150540
ENSG00000150551
ENSG00000150556
ENSG00000150593
ENSG00000150594
ENSG00000150625
ENSG00000150627
ENSG00000150636
ENSG00000150637
ENSG00000150656
ENSG00000150667
ENSG00000150672
ENSG00000150676
ENSG00000150681
ENSG00000150687
ENSG00000150712
ENSG00000150722
ENSG00000150753
ENSG00000150756
ENSG00000150760
ENSG00000150764
ENSG00000150768
ENSG00000150776
ENSG00000150779
ENSG00000150782

ENSG00000150787
ENSG00000150867
ENSG00000150873
ENSG00000150893
ENSG00000150907
ENSG00000150938
ENSG00000150967
ENSG00000150977
ENSG00000150990
ENSG00000150995
ENSG00000151005
ENSG00000151012
ENSG00000151014
ENSG00000151023
ENSG00000151025
ENSG00000151062
ENSG00000151065
ENSG00000151067
ENSG00000151090
ENSG00000151092
ENSG00000151116
ENSG00000151117
ENSG00000151135
ENSG00000151136
ENSG00000151148
ENSG00000151150
ENSG00000151151
ENSG00000151164
ENSG00000151176
ENSG00000151208
ENSG00000151224
ENSG00000151229
ENSG00000151233
ENSG00000151239
ENSG00000151240
ENSG00000151247
ENSG00000151276
ENSG00000151292

ENSG00000151304
ENSG00000151320
ENSG00000151322
ENSG00000151327
ENSG00000151332
ENSG00000151338
ENSG00000151348
ENSG00000151353
ENSG00000151364
ENSG00000151365
ENSG00000151366
ENSG00000151413
ENSG00000151414
ENSG00000151422
ENSG00000151445
ENSG00000151458
ENSG00000151461
ENSG00000151465
ENSG00000151466
ENSG00000151468
ENSG00000151470
ENSG00000151474
ENSG00000151490
ENSG00000151491
ENSG00000151498
ENSG00000151500
ENSG00000151502
ENSG00000151503
ENSG00000151532
ENSG00000151552
ENSG00000151553
ENSG00000151572
ENSG00000151575
ENSG00000151576
ENSG00000151577
ENSG00000151611
ENSG00000151612
ENSG00000151615

ENSG00000151617
ENSG00000151623
ENSG00000151632
ENSG00000151640
ENSG00000151650
ENSG00000151651
ENSG00000151657
ENSG00000151665
ENSG00000151687
ENSG00000151692
ENSG00000151693
ENSG00000151694
ENSG00000151702
ENSG00000151704
ENSG00000151715
ENSG00000151718
ENSG00000151726
ENSG00000151729
ENSG00000151743
ENSG00000151746
ENSG00000151748
ENSG00000151778
ENSG00000151789
ENSG00000151806
ENSG00000151834
ENSG00000151849
ENSG00000151876
ENSG00000151881
ENSG00000151882
ENSG00000151883
ENSG00000151892
ENSG00000151893
ENSG00000151914
ENSG00000151917
ENSG00000151923
ENSG00000151929
ENSG00000151948
ENSG00000151952

ENSG00000151962
ENSG00000152034
ENSG00000152049
ENSG00000152056
ENSG00000152061
ENSG00000152078
ENSG00000152086
ENSG00000152092
ENSG00000152102
ENSG00000152104
ENSG00000152127
ENSG00000152128
ENSG00000152133
ENSG00000152137
ENSG00000152154
ENSG00000152192
ENSG00000152193
ENSG00000152207
ENSG00000152208
ENSG00000152213
ENSG00000152217
ENSG00000152219
ENSG00000152223
ENSG00000152229
ENSG00000152242
ENSG00000152254
ENSG00000152256
ENSG00000152270
ENSG00000152284
ENSG00000152291
ENSG00000152315
ENSG00000152332
ENSG00000152348
ENSG00000152359
ENSG00000152377
ENSG00000152380
ENSG00000152382
ENSG00000152402

ENSG00000152404
ENSG00000152409
ENSG00000152413
ENSG00000152430
ENSG00000152433
ENSG00000152439
ENSG00000152443
ENSG00000152455
ENSG00000152457
ENSG00000152463
ENSG00000152465
ENSG00000152467
ENSG00000152475
ENSG00000152484
ENSG00000152492
ENSG00000152495
ENSG00000152503
ENSG00000152518
ENSG00000152520
ENSG00000152527
ENSG00000152556
ENSG00000152558
ENSG00000152578
ENSG00000152582
ENSG00000152592
ENSG00000152601
ENSG00000152620
ENSG00000152642
ENSG00000152661
ENSG00000152669
ENSG00000152670
ENSG00000152683
ENSG00000152684
ENSG00000152689
ENSG00000152700
ENSG00000152749
ENSG00000152760
ENSG00000152763

ENSG00000152766
ENSG00000152767
ENSG00000152778
ENSG00000152779
ENSG00000152782
ENSG00000152784
ENSG00000152785
ENSG00000152795
ENSG00000152804
ENSG00000152818
ENSG00000152822
ENSG00000152894
ENSG00000152904
ENSG00000152910
ENSG00000152926
ENSG00000152932
ENSG00000152936
ENSG00000152944
ENSG00000152952
ENSG00000152953
ENSG00000152954
ENSG00000152969
ENSG00000152977
ENSG00000152990
ENSG00000153006
ENSG00000153012
ENSG00000153015
ENSG00000153029
ENSG00000153037
ENSG00000153044
ENSG00000153046
ENSG00000153048
ENSG00000153064
ENSG00000153071
ENSG00000153093
ENSG00000153094
ENSG00000153107
ENSG00000153113

ENSG00000153130
ENSG00000153147
ENSG00000153162
ENSG00000153165
ENSG00000153179
ENSG00000153187
ENSG00000153201
ENSG00000153207
ENSG00000153214
ENSG00000153233
ENSG00000153234
ENSG00000153246
ENSG00000153250
ENSG00000153253
ENSG00000153266
ENSG00000153283
ENSG00000153291
ENSG00000153292
ENSG00000153294
ENSG00000153303
ENSG00000153310
ENSG00000153317
ENSG00000153339
ENSG00000153391
ENSG00000153395
ENSG00000153404
ENSG00000153406
ENSG00000153443
ENSG00000153446
ENSG00000153485
ENSG00000153487
ENSG00000153531
ENSG00000153551
ENSG00000153558
ENSG00000153560
ENSG00000153561
ENSG00000153563
ENSG00000153574

ENSG00000153707
ENSG00000153714
ENSG00000153721
ENSG00000153767
ENSG00000153786
ENSG00000153789
ENSG00000153790
ENSG00000153802
ENSG00000153814
ENSG00000153815
ENSG00000153822
ENSG00000153823
ENSG00000153827
ENSG00000153832
ENSG00000153879
ENSG00000153885
ENSG00000153896
ENSG00000153898
ENSG00000153902
ENSG00000153904
ENSG00000153914
ENSG00000153930
ENSG00000153933
ENSG00000153936
ENSG00000153944
ENSG00000153956
ENSG00000153982
ENSG00000153989
ENSG00000153993
ENSG00000154001
ENSG00000154016
ENSG00000154025
ENSG00000154027
ENSG00000154040
ENSG00000154059
ENSG00000154065
ENSG00000154080
ENSG00000154096

ENSG00000154102
ENSG00000154114
ENSG00000154118
ENSG00000154122
ENSG00000154124
ENSG00000154127
ENSG00000154133
ENSG00000154134
ENSG00000154143
ENSG00000154144
ENSG00000154146
ENSG00000154153
ENSG00000154162
ENSG00000154165
ENSG00000154174
ENSG00000154175
ENSG00000154188
ENSG00000154217
ENSG00000154222
ENSG00000154227
ENSG00000154229
ENSG00000154237
ENSG00000154265
ENSG00000154269
ENSG00000154274
ENSG00000154277
ENSG00000154310
ENSG00000154319
ENSG00000154328
ENSG00000154330
ENSG00000154359
ENSG00000154370
ENSG00000154380
ENSG00000154415
ENSG00000154429
ENSG00000154447
ENSG00000154451
ENSG00000154473

ENSG00000154478
ENSG00000154479
ENSG00000154493
ENSG00000154511
ENSG00000154518
ENSG00000154529
ENSG00000154545
ENSG00000154553
ENSG00000154556
ENSG00000154582
ENSG00000154611
ENSG00000154639
ENSG00000154642
ENSG00000154645
ENSG00000154646
ENSG00000154654
ENSG00000154655
ENSG00000154678
ENSG00000154710
ENSG00000154721
ENSG00000154727
ENSG00000154734
ENSG00000154736
ENSG00000154743
ENSG00000154760
ENSG00000154764
ENSG00000154767
ENSG00000154781
ENSG00000154783
ENSG00000154803
ENSG00000154813
ENSG00000154814
ENSG00000154839
ENSG00000154845
ENSG00000154856
ENSG00000154864
ENSG00000154889
ENSG00000154917

ENSG00000154928
ENSG00000154930
ENSG00000154945
ENSG00000154957
ENSG00000154975
ENSG00000154978
ENSG00000154997
ENSG00000155008
ENSG00000155011
ENSG00000155016
ENSG00000155034
ENSG00000155052
ENSG00000155066
ENSG00000155085
ENSG00000155090
ENSG00000155093
ENSG00000155096
ENSG00000155097
ENSG00000155099
ENSG00000155100
ENSG00000155111
ENSG00000155158
ENSG00000155189
ENSG00000155252
ENSG00000155254
ENSG00000155256
ENSG00000155265
ENSG00000155269
ENSG00000155287
ENSG00000155324
ENSG00000155329
ENSG00000155330
ENSG00000155366
ENSG00000155380
ENSG00000155438
ENSG00000155465
ENSG00000155495
ENSG00000155506

ENSG00000155508
ENSG00000155511
ENSG00000155542
ENSG00000155545
ENSG00000155592
ENSG00000155621
ENSG00000155622
ENSG00000155629
ENSG00000155636
ENSG00000155659
ENSG00000155660
ENSG00000155666
ENSG00000155719
ENSG00000155729
ENSG00000155744
ENSG00000155749
ENSG00000155754
ENSG00000155755
ENSG00000155760
ENSG00000155761
ENSG00000155792
ENSG00000155827
ENSG00000155833
ENSG00000155846
ENSG00000155849
ENSG00000155850
ENSG00000155858
ENSG00000155875
ENSG00000155876
ENSG00000155886
ENSG00000155890
ENSG00000155893
ENSG00000155903
ENSG00000155906
ENSG00000155918
ENSG00000155926
ENSG00000155959
ENSG00000155961

ENSG00000155962
ENSG00000155966
ENSG00000155970
ENSG00000155974
ENSG00000155975
ENSG00000156006
ENSG00000156009
ENSG00000156011
ENSG00000156017
ENSG00000156026
ENSG00000156030
ENSG00000156049
ENSG00000156050
ENSG00000156052
ENSG00000156097
ENSG00000156103
ENSG00000156113
ENSG00000156127
ENSG00000156136
ENSG00000156140
ENSG00000156150
ENSG00000156162
ENSG00000156170
ENSG00000156171
ENSG00000156172
ENSG00000156206
ENSG00000156218
ENSG00000156222
ENSG00000156232
ENSG00000156234
ENSG00000156253
ENSG00000156265
ENSG00000156269
ENSG00000156273
ENSG00000156304
ENSG00000156313
ENSG00000156345
ENSG00000156374

ENSG00000156381
ENSG00000156384
ENSG00000156395
ENSG00000156398
ENSG00000156411
ENSG00000156413
ENSG00000156414
ENSG00000156427
ENSG00000156453
ENSG00000156463
ENSG00000156466
ENSG00000156467
ENSG00000156471
ENSG00000156475
ENSG00000156486
ENSG00000156500
ENSG00000156504
ENSG00000156508
ENSG00000156510
ENSG00000156515
ENSG00000156521
ENSG00000156531
ENSG00000156535
ENSG00000156574
ENSG00000156587
ENSG00000156599
ENSG00000156603
ENSG00000156639
ENSG00000156642
ENSG00000156650
ENSG00000156671
ENSG00000156675
ENSG00000156687
ENSG00000156689
ENSG00000156709
ENSG00000156711
ENSG00000156735
ENSG00000156738

ENSG00000156795
ENSG00000156802
ENSG00000156804
ENSG00000156853
ENSG00000156858
ENSG00000156860
ENSG00000156869
ENSG00000156873
ENSG00000156876
ENSG00000156886
ENSG00000156925
ENSG00000156958
ENSG00000156959
ENSG00000156966
ENSG00000156968
ENSG00000156973
ENSG00000156976
ENSG00000156983
ENSG00000157017
ENSG00000157036
ENSG00000157045
ENSG00000157064
ENSG00000157087
ENSG00000157093
ENSG00000157103
ENSG00000157106
ENSG00000157107
ENSG00000157110
ENSG00000157119
ENSG00000157150
ENSG00000157152
ENSG00000157168
ENSG00000157181
ENSG00000157191
ENSG00000157193
ENSG00000157211
ENSG00000157212
ENSG00000157214

ENSG00000157216
ENSG00000157219
ENSG00000157224
ENSG00000157227
ENSG00000157240
ENSG00000157259
ENSG00000157303
ENSG00000157315
ENSG00000157322
ENSG00000157326
ENSG00000157335
ENSG00000157349
ENSG00000157350
ENSG00000157368
ENSG00000157379
ENSG00000157388
ENSG00000157399
ENSG00000157404
ENSG00000157423
ENSG00000157429
ENSG00000157450
ENSG00000157470
ENSG00000157483
ENSG00000157500
ENSG00000157502
ENSG00000157510
ENSG00000157514
ENSG00000157538
ENSG00000157540
ENSG00000157542
ENSG00000157551
ENSG00000157554
ENSG00000157557
ENSG00000157570
ENSG00000157593
ENSG00000157600
ENSG00000157613
ENSG00000157617

ENSG00000157625
ENSG00000157637
ENSG00000157653
ENSG00000157654
ENSG00000157657
ENSG00000157680
ENSG00000157693
ENSG00000157703
ENSG00000157734
ENSG00000157741
ENSG00000157765
ENSG00000157766
ENSG00000157778
ENSG00000157782
ENSG00000157796
ENSG00000157800
ENSG00000157823
ENSG00000157827
ENSG00000157833
ENSG00000157837
ENSG00000157851
ENSG00000157869
ENSG00000157870
ENSG00000157873
ENSG00000157895
ENSG00000157911
ENSG00000157916
ENSG00000157933
ENSG00000157954
ENSG00000157978
ENSG00000157985
ENSG00000157999
ENSG00000158006
ENSG00000158014
ENSG00000158023
ENSG00000158042
ENSG00000158050
ENSG00000158055

ENSG00000158077
ENSG00000158079
ENSG00000158089
ENSG00000158092
ENSG00000158104
ENSG00000158109
ENSG00000158122
ENSG00000158156
ENSG00000158158
ENSG00000158161
ENSG00000158163
ENSG00000158164
ENSG00000158169
ENSG00000158186
ENSG00000158195
ENSG00000158220
ENSG00000158246
ENSG00000158258
ENSG00000158270
ENSG00000158286
ENSG00000158290
ENSG00000158292
ENSG00000158296
ENSG00000158301
ENSG00000158315
ENSG00000158321
ENSG00000158352
ENSG00000158402
ENSG00000158417
ENSG00000158423
ENSG00000158427
ENSG00000158435
ENSG00000158445
ENSG00000158457
ENSG00000158458
ENSG00000158467
ENSG00000158470
ENSG00000158473

ENSG00000158480
ENSG00000158481
ENSG00000158483
ENSG00000158485
ENSG00000158488
ENSG00000158526
ENSG00000158528
ENSG00000158545
ENSG00000158552
ENSG00000158555
ENSG00000158571
ENSG00000158604
ENSG00000158615
ENSG00000158623
ENSG00000158636
ENSG00000158669
ENSG00000158691
ENSG00000158710
ENSG00000158711
ENSG00000158714
ENSG00000158715
ENSG00000158716
ENSG00000158717
ENSG00000158747
ENSG00000158748
ENSG00000158769
ENSG00000158773
ENSG00000158786
ENSG00000158792
ENSG00000158793
ENSG00000158796
ENSG00000158805
ENSG00000158806
ENSG00000158813
ENSG00000158815
ENSG00000158816
ENSG00000158828
ENSG00000158850

ENSG00000158856
ENSG00000158859
ENSG00000158863
ENSG00000158864
ENSG00000158869
ENSG00000158874
ENSG00000158882
ENSG00000158887
ENSG00000158941
ENSG00000158955
ENSG00000158966
ENSG00000158985
ENSG00000159023
ENSG00000159063
ENSG00000159069
ENSG00000159082
ENSG00000159110
ENSG00000159111
ENSG00000159128
ENSG00000159140
ENSG00000159147
ENSG00000159164
ENSG00000159166
ENSG00000159167
ENSG00000159173
ENSG00000159176
ENSG00000159184
ENSG00000159200
ENSG00000159202
ENSG00000159212
ENSG00000159216
ENSG00000159217
ENSG00000159224
ENSG00000159228
ENSG00000159251
ENSG00000159256
ENSG00000159259
ENSG00000159261

ENSG00000159263
ENSG00000159267
ENSG00000159289
ENSG00000159307
ENSG00000159314
ENSG00000159322
ENSG00000159335
ENSG00000159339
ENSG00000159346
ENSG00000159348
ENSG00000159352
ENSG00000159363
ENSG00000159374
ENSG00000159387
ENSG00000159388
ENSG00000159399
ENSG00000159409
ENSG00000159423
ENSG00000159433
ENSG00000159445
ENSG00000159450
ENSG00000159455
ENSG00000159459
ENSG00000159461
ENSG00000159479
ENSG00000159516
ENSG00000159579
ENSG00000159592
ENSG00000159593
ENSG00000159596
ENSG00000159618
ENSG00000159648
ENSG00000159658
ENSG00000159674
ENSG00000159685
ENSG00000159692
ENSG00000159720
ENSG00000159733

ENSG00000159763
ENSG00000159784
ENSG00000159788
ENSG00000159792
ENSG00000159840
ENSG00000159842
ENSG00000159871
ENSG00000159873
ENSG00000159882
ENSG00000159884
ENSG00000159899
ENSG00000159905
ENSG00000159915
ENSG00000159917
ENSG00000159921
ENSG00000159958
ENSG00000160007
ENSG00000160013
ENSG00000160014
ENSG00000160049
ENSG00000160050
ENSG00000160051
ENSG00000160055
ENSG00000160058
ENSG00000160062
ENSG00000160075
ENSG00000160087
ENSG00000160094
ENSG00000160097
ENSG00000160113
ENSG00000160117
ENSG00000160131
ENSG00000160145
ENSG00000160161
ENSG00000160179
ENSG00000160180
ENSG00000160183
ENSG00000160185

ENSG00000160190
ENSG00000160191
ENSG00000160193
ENSG00000160199
ENSG00000160200
ENSG00000160202
ENSG00000160207
ENSG00000160209
ENSG00000160211
ENSG00000160213
ENSG00000160214
ENSG00000160216
ENSG00000160218
ENSG00000160219
ENSG00000160221
ENSG00000160223
ENSG00000160224
ENSG00000160226
ENSG00000160233
ENSG00000160255
ENSG00000160256
ENSG00000160271
ENSG00000160282
ENSG00000160284
ENSG00000160285
ENSG00000160293
ENSG00000160298
ENSG00000160305
ENSG00000160307
ENSG00000160310
ENSG00000160321
ENSG00000160323
ENSG00000160325
ENSG00000160326
ENSG00000160336
ENSG00000160345
ENSG00000160352
ENSG00000160360

ENSG00000160392
ENSG00000160396
ENSG00000160401
ENSG00000160404
ENSG00000160408
ENSG00000160439
ENSG00000160445
ENSG00000160460
ENSG00000160471
ENSG00000160539
ENSG00000160551
ENSG00000160563
ENSG00000160570
ENSG00000160584
ENSG00000160588
ENSG00000160593
ENSG00000160602
ENSG00000160613
ENSG00000160654
ENSG00000160679
ENSG00000160683
ENSG00000160685
ENSG00000160691
ENSG00000160703
ENSG00000160710
ENSG00000160712
ENSG00000160714
ENSG00000160716
ENSG00000160741
ENSG00000160746
ENSG00000160753
ENSG00000160767
ENSG00000160781
ENSG00000160783
ENSG00000160785
ENSG00000160789
ENSG00000160791
ENSG00000160803

ENSG00000160808
ENSG00000160818
ENSG00000160856
ENSG00000160868
ENSG00000160870
ENSG00000160877
ENSG00000160882
ENSG00000160886
ENSG00000160888
ENSG00000160908
ENSG00000160917
ENSG00000160948
ENSG00000160953
ENSG00000160959
ENSG00000160961
ENSG00000160963
ENSG00000160973
ENSG00000160991
ENSG00000160993
ENSG00000161010
ENSG00000161011
ENSG00000161021
ENSG00000161040
ENSG00000161048
ENSG00000161055
ENSG00000161082
ENSG00000161091
ENSG00000161179
ENSG00000161180
ENSG00000161202
ENSG00000161203
ENSG00000161204
ENSG00000161217
ENSG00000161243
ENSG00000161249
ENSG00000161267
ENSG00000161270
ENSG00000161277

ENSG00000161298
ENSG00000161328
ENSG00000161381
ENSG00000161395
ENSG00000161405
ENSG00000161513
ENSG00000161526
ENSG00000161533
ENSG00000161542
ENSG00000161544
ENSG00000161547
ENSG00000161551
ENSG00000161609
ENSG00000161618
ENSG00000161638
ENSG00000161640
ENSG00000161642
ENSG00000161647
ENSG00000161649
ENSG00000161653
ENSG00000161654
ENSG00000161664
ENSG00000161671
ENSG00000161681
ENSG00000161692
ENSG00000161714
ENSG00000161791
ENSG00000161798
ENSG00000161800
ENSG00000161813
ENSG00000161835
ENSG00000161847
ENSG00000161849
ENSG00000161850
ENSG00000161888
ENSG00000161896
ENSG00000161904
ENSG00000161905

ENSG00000161911
ENSG00000161921
ENSG00000161929
ENSG00000161940
ENSG00000161944
ENSG00000161955
ENSG00000161958
ENSG00000161960
ENSG00000161973
ENSG00000161980
ENSG00000161981
ENSG00000161999
ENSG00000162009
ENSG00000162032
ENSG00000162062
ENSG00000162063
ENSG00000162065
ENSG00000162066
ENSG00000162073
ENSG00000162086
ENSG00000162104
ENSG00000162105
ENSG00000162129
ENSG00000162139
ENSG00000162144
ENSG00000162174
ENSG00000162188
ENSG00000162194
ENSG00000162231
ENSG00000162236
ENSG00000162241
ENSG00000162267
ENSG00000162298
ENSG00000162300
ENSG00000162302
ENSG00000162341
ENSG00000162344
ENSG00000162365

ENSG00000162366
ENSG00000162367
ENSG00000162368
ENSG00000162373
ENSG00000162374
ENSG00000162377
ENSG00000162378
ENSG00000162383
ENSG00000162390
ENSG00000162391
ENSG00000162396
ENSG00000162398
ENSG00000162407
ENSG00000162408
ENSG00000162409
ENSG00000162413
ENSG00000162415
ENSG00000162419
ENSG00000162426
ENSG00000162430
ENSG00000162433
ENSG00000162434
ENSG00000162441
ENSG00000162456
ENSG00000162458
ENSG00000162490
ENSG00000162493
ENSG00000162494
ENSG00000162496
ENSG00000162510
ENSG00000162511
ENSG00000162512
ENSG00000162517
ENSG00000162520
ENSG00000162521
ENSG00000162522
ENSG00000162542
ENSG00000162543

ENSG00000162545
ENSG00000162551
ENSG00000162552
ENSG00000162571
ENSG00000162576
ENSG00000162585
ENSG00000162591
ENSG00000162594
ENSG00000162595
ENSG00000162598
ENSG00000162599
ENSG00000162601
ENSG00000162607
ENSG00000162613
ENSG00000162614
ENSG00000162618
ENSG00000162621
ENSG00000162623
ENSG00000162627
ENSG00000162630
ENSG00000162631
ENSG00000162636
ENSG00000162639
ENSG00000162642
ENSG00000162645
ENSG00000162650
ENSG00000162654
ENSG00000162664
ENSG00000162670
ENSG00000162676
ENSG00000162687
ENSG00000162688
ENSG00000162692
ENSG00000162694
ENSG00000162695
ENSG00000162702
ENSG00000162704
ENSG00000162706

ENSG00000162714
ENSG00000162722
ENSG00000162723
ENSG00000162728
ENSG00000162729
ENSG00000162734
ENSG00000162735
ENSG00000162736
ENSG00000162738
ENSG00000162739
ENSG00000162745
ENSG00000162746
ENSG00000162747
ENSG00000162757
ENSG00000162761
ENSG00000162769
ENSG00000162772
ENSG00000162777
ENSG00000162779
ENSG00000162783
ENSG00000162813
ENSG00000162814
ENSG00000162817
ENSG00000162819
ENSG00000162825
ENSG00000162852
ENSG00000162869
ENSG00000162873
ENSG00000162877
ENSG00000162878
ENSG00000162885
ENSG00000162889
ENSG00000162892
ENSG00000162894
ENSG00000162896
ENSG00000162897
ENSG00000162909
ENSG00000162923

ENSG00000162924
ENSG00000162927
ENSG00000162928
ENSG00000162929
ENSG00000162931
ENSG00000162944
ENSG00000162946
ENSG00000162949
ENSG00000162959
ENSG00000162971
ENSG00000162972
ENSG00000162975
ENSG00000162976
ENSG00000162981
ENSG00000162989
ENSG00000162992
ENSG00000162994
ENSG00000162998
ENSG00000162999
ENSG00000163002
ENSG00000163006
ENSG00000163013
ENSG00000163026
ENSG00000163029
ENSG00000163032
ENSG00000163040
ENSG00000163050
ENSG00000163053
ENSG00000163060
ENSG00000163064
ENSG00000163069
ENSG00000163071
ENSG00000163072
ENSG00000163082
ENSG00000163083
ENSG00000163092
ENSG00000163093
ENSG00000163104

ENSG00000163106
ENSG00000163110
ENSG00000163121
ENSG00000163125
ENSG00000163126
ENSG00000163131
ENSG00000163132
ENSG00000163138
ENSG00000163141
ENSG00000163145
ENSG00000163154
ENSG00000163155
ENSG00000163156
ENSG00000163157
ENSG00000163161
ENSG00000163162
ENSG00000163170
ENSG00000163171
ENSG00000163202
ENSG00000163206
ENSG00000163214
ENSG00000163216
ENSG00000163219
ENSG00000163235
ENSG00000163239
ENSG00000163249
ENSG00000163251
ENSG00000163257
ENSG00000163263
ENSG00000163281
ENSG00000163285
ENSG00000163288
ENSG00000163291
ENSG00000163293
ENSG00000163295
ENSG00000163297
ENSG00000163319
ENSG00000163320

ENSG00000163322
ENSG00000163328
ENSG00000163331
ENSG00000163344
ENSG00000163346
ENSG00000163347
ENSG00000163348
ENSG00000163349
ENSG00000163359
ENSG00000163362
ENSG00000163374
ENSG00000163376
ENSG00000163377
ENSG00000163378
ENSG00000163380
ENSG00000163389
ENSG00000163393
ENSG00000163394
ENSG00000163399
ENSG00000163406
ENSG00000163412
ENSG00000163421
ENSG00000163428
ENSG00000163430
ENSG00000163431
ENSG00000163435
ENSG00000163444
ENSG00000163449
ENSG00000163462
ENSG00000163464
ENSG00000163466
ENSG00000163472
ENSG00000163479
ENSG00000163481
ENSG00000163482
ENSG00000163485
ENSG00000163491
ENSG00000163492

ENSG00000163497
ENSG00000163501
ENSG00000163507
ENSG00000163508
ENSG00000163510
ENSG00000163512
ENSG00000163513
ENSG00000163516
ENSG00000163517
ENSG00000163518
ENSG00000163521
ENSG00000163527
ENSG00000163528
ENSG00000163530
ENSG00000163531
ENSG00000163534
ENSG00000163539
ENSG00000163558
ENSG00000163564
ENSG00000163565
ENSG00000163577
ENSG00000163581
ENSG00000163584
ENSG00000163590
ENSG00000163596
ENSG00000163599
ENSG00000163600
ENSG00000163605
ENSG00000163606
ENSG00000163607
ENSG00000163608
ENSG00000163611
ENSG00000163617
ENSG00000163618
ENSG00000163624
ENSG00000163625
ENSG00000163626
ENSG00000163630

ENSG00000163633
ENSG00000163635
ENSG00000163637
ENSG00000163638
ENSG00000163644
ENSG00000163646
ENSG00000163659
ENSG00000163673
ENSG00000163681
ENSG00000163683
ENSG00000163684
ENSG00000163689
ENSG00000163694
ENSG00000163697
ENSG00000163701
ENSG00000163703
ENSG00000163704
ENSG00000163705
ENSG00000163710
ENSG00000163714
ENSG00000163719
ENSG00000163728
ENSG00000163734
ENSG00000163735
ENSG00000163736
ENSG00000163737
ENSG00000163738
ENSG00000163743
ENSG00000163751
ENSG00000163754
ENSG00000163755
ENSG00000163762
ENSG00000163788
ENSG00000163793
ENSG00000163795
ENSG00000163798
ENSG00000163803
ENSG00000163806

ENSG00000163807
ENSG00000163810
ENSG00000163811
ENSG00000163812
ENSG00000163814
ENSG00000163815
ENSG00000163817
ENSG00000163818
ENSG00000163820
ENSG00000163823
ENSG00000163827
ENSG00000163832
ENSG00000163833
ENSG00000163840
ENSG00000163848
ENSG00000163864
ENSG00000163866
ENSG00000163867
ENSG00000163870
ENSG00000163872
ENSG00000163873
ENSG00000163874
ENSG00000163875
ENSG00000163877
ENSG00000163879
ENSG00000163884
ENSG00000163888
ENSG00000163898
ENSG00000163900
ENSG00000163902
ENSG00000163904
ENSG00000163909
ENSG00000163913
ENSG00000163914
ENSG00000163930
ENSG00000163931
ENSG00000163932
ENSG00000163933

ENSG00000163939
ENSG00000163945
ENSG00000163946
ENSG00000163947
ENSG00000163950
ENSG00000163956
ENSG00000163958
ENSG00000163959
ENSG00000163960
ENSG00000163961
ENSG00000163964
ENSG00000163975
ENSG00000163995
ENSG00000164002
ENSG00000164007
ENSG00000164008
ENSG00000164010
ENSG00000164011
ENSG00000164022
ENSG00000164023
ENSG00000164024
ENSG00000164031
ENSG00000164032
ENSG00000164035
ENSG00000164038
ENSG00000164039
ENSG00000164040
ENSG00000164045
ENSG00000164048
ENSG00000164051
ENSG00000164054
ENSG00000164056
ENSG00000164061
ENSG00000164066
ENSG00000164070
ENSG00000164073
ENSG00000164074
ENSG00000164076

ENSG00000164077
ENSG00000164078
ENSG00000164080
ENSG00000164081
ENSG00000164082
ENSG00000164086
ENSG00000164087
ENSG00000164088
ENSG00000164089
ENSG00000164091
ENSG00000164093
ENSG00000164096
ENSG00000164100
ENSG00000164104
ENSG00000164107
ENSG00000164111
ENSG00000164112
ENSG00000164114
ENSG00000164116
ENSG00000164117
ENSG00000164118
ENSG00000164120
ENSG00000164122
ENSG00000164124
ENSG00000164125
ENSG00000164128
ENSG00000164129
ENSG00000164134
ENSG00000164142
ENSG00000164144
ENSG00000164151
ENSG00000164162
ENSG00000164163
ENSG00000164164
ENSG00000164168
ENSG00000164169
ENSG00000164171
ENSG00000164172

ENSG00000164175
ENSG00000164176
ENSG00000164180
ENSG00000164181
ENSG00000164188
ENSG00000164190
ENSG00000164197
ENSG00000164209
ENSG00000164211
ENSG00000164219
ENSG00000164220
ENSG00000164236
ENSG00000164237
ENSG00000164241
ENSG00000164244
ENSG00000164251
ENSG00000164252
ENSG00000164253
ENSG00000164256
ENSG00000164270
ENSG00000164283
ENSG00000164284
ENSG00000164287
ENSG00000164291
ENSG00000164292
ENSG00000164294
ENSG00000164296
ENSG00000164300
ENSG00000164303
ENSG00000164305
ENSG00000164306
ENSG00000164307
ENSG00000164308
ENSG00000164318
ENSG00000164323
ENSG00000164325
ENSG00000164326
ENSG00000164327

ENSG00000164329
ENSG00000164330
ENSG00000164332
ENSG00000164334
ENSG00000164338
ENSG00000164342
ENSG00000164347
ENSG00000164362
ENSG00000164366
ENSG00000164393
ENSG00000164399
ENSG00000164400
ENSG00000164402
ENSG00000164404
ENSG00000164405
ENSG00000164406
ENSG00000164411
ENSG00000164418
ENSG00000164434
ENSG00000164438
ENSG00000164440
ENSG00000164451
ENSG00000164458
ENSG00000164463
ENSG00000164465
ENSG00000164466
ENSG00000164485
ENSG00000164488
ENSG00000164494
ENSG00000164500
ENSG00000164506
ENSG00000164509
ENSG00000164520
ENSG00000164530
ENSG00000164535
ENSG00000164542
ENSG00000164543
ENSG00000164574

ENSG00000164576
ENSG00000164588
ENSG00000164591
ENSG00000164597
ENSG00000164600
ENSG00000164603
ENSG00000164604
ENSG00000164609
ENSG00000164610
ENSG00000164615
ENSG00000164619
ENSG00000164620
ENSG00000164626
ENSG00000164627
ENSG00000164631
ENSG00000164638
ENSG00000164649
ENSG00000164651
ENSG00000164659
ENSG00000164663
ENSG00000164675
ENSG00000164683
ENSG00000164684
ENSG00000164687
ENSG00000164690
ENSG00000164695
ENSG00000164713
ENSG00000164715
ENSG00000164729
ENSG00000164733
ENSG00000164741
ENSG00000164742
ENSG00000164743
ENSG00000164746
ENSG00000164749
ENSG00000164751
ENSG00000164756
ENSG00000164758

ENSG00000164761
ENSG00000164764
ENSG00000164776
ENSG00000164778
ENSG00000164794
ENSG00000164796
ENSG00000164808
ENSG00000164815
ENSG00000164818
ENSG00000164823
ENSG00000164828
ENSG00000164830
ENSG00000164841
ENSG00000164849
ENSG00000164850
ENSG00000164855
ENSG00000164867
ENSG00000164871
ENSG00000164877
ENSG00000164879
ENSG00000164885
ENSG00000164889
ENSG00000164897
ENSG00000164898
ENSG00000164902
ENSG00000164904
ENSG00000164916
ENSG00000164920
ENSG00000164924
ENSG00000164929
ENSG00000164930
ENSG00000164933
ENSG00000164935
ENSG00000164938
ENSG00000164944
ENSG00000164946
ENSG00000164949
ENSG00000164951

ENSG00000164953
ENSG00000164967
ENSG00000164970
ENSG00000164975
ENSG00000164976
ENSG00000164978
ENSG00000164983
ENSG00000164985
ENSG00000165006
ENSG00000165023
ENSG00000165025
ENSG00000165028
ENSG00000165029
ENSG00000165030
ENSG00000165046
ENSG00000165055
ENSG00000165059
ENSG00000165060
ENSG00000165061
ENSG00000165066
ENSG00000165071
ENSG00000165091
ENSG00000165092
ENSG00000165097
ENSG00000165102
ENSG00000165105
ENSG00000165118
ENSG00000165119
ENSG00000165124
ENSG00000165125
ENSG00000165138
ENSG00000165152
ENSG00000165168
ENSG00000165169
ENSG00000165175
ENSG00000165185
ENSG00000165186
ENSG00000165188

ENSG00000165192
ENSG00000165194
ENSG00000165195
ENSG00000165209
ENSG00000165215
ENSG00000165219
ENSG00000165233
ENSG00000165238
ENSG00000165240
ENSG00000165244
ENSG00000165246
ENSG00000165259
ENSG00000165269
ENSG00000165271
ENSG00000165272
ENSG00000165275
ENSG00000165280
ENSG00000165282
ENSG00000165283
ENSG00000165288
ENSG00000165309
ENSG00000165312
ENSG00000165322
ENSG00000165323
ENSG00000165325
ENSG00000165338
ENSG00000165355
ENSG00000165359
ENSG00000165376
ENSG00000165383
ENSG00000165389
ENSG00000165406
ENSG00000165409
ENSG00000165410
ENSG00000165417
ENSG00000165424
ENSG00000165434
ENSG00000165443

ENSG00000165449
ENSG00000165457
ENSG00000165458
ENSG00000165462
ENSG00000165471
ENSG00000165474
ENSG00000165475
ENSG00000165476
ENSG00000165478
ENSG00000165480
ENSG00000165490
ENSG00000165495
ENSG00000165501
ENSG00000165506
ENSG00000165507
ENSG00000165512
ENSG00000165516
ENSG00000165521
ENSG00000165526
ENSG00000165527
ENSG00000165533
ENSG00000165548
ENSG00000165553
ENSG00000165556
ENSG00000165566
ENSG00000165568
ENSG00000165572
ENSG00000165584
ENSG00000165588
ENSG00000165609
ENSG00000165617
ENSG00000165621
ENSG00000165623
ENSG00000165626
ENSG00000165632
ENSG00000165633
ENSG00000165643
ENSG00000165646

ENSG00000165650
ENSG00000165655
ENSG00000165660
ENSG00000165661
ENSG00000165669
ENSG00000165671
ENSG00000165675
ENSG00000165678
ENSG00000165682
ENSG00000165685
ENSG00000165688
ENSG00000165689
ENSG00000165694
ENSG00000165695
ENSG00000165698
ENSG00000165699
ENSG00000165702
ENSG00000165704
ENSG00000165714
ENSG00000165716
ENSG00000165724
ENSG00000165731
ENSG00000165732
ENSG00000165733
ENSG00000165752
ENSG00000165757
ENSG00000165775
ENSG00000165782
ENSG00000165792
ENSG00000165794
ENSG00000165795
ENSG00000165799
ENSG00000165801
ENSG00000165802
ENSG00000165804
ENSG00000165806
ENSG00000165810
ENSG00000165813

ENSG00000165816
ENSG00000165821
ENSG00000165832
ENSG00000165861
ENSG00000165863
ENSG00000165868
ENSG00000165879
ENSG00000165891
ENSG00000165895
ENSG00000165898
ENSG00000165899
ENSG00000165905
ENSG00000165912
ENSG00000165914
ENSG00000165915
ENSG00000165917
ENSG00000165923
ENSG00000165929
ENSG00000165934
ENSG00000165943
ENSG00000165948
ENSG00000165949
ENSG00000165959
ENSG00000165966
ENSG00000165973
ENSG00000165983
ENSG00000165985
ENSG00000165995
ENSG00000165996
ENSG00000165997
ENSG00000166002
ENSG00000166006
ENSG00000166016
ENSG00000166024
ENSG00000166025
ENSG00000166037
ENSG00000166049
ENSG00000166068

ENSG00000166069
ENSG00000166073
ENSG00000166086
ENSG00000166090
ENSG00000166106
ENSG00000166111
ENSG00000166118
ENSG00000166123
ENSG00000166128
ENSG00000166130
ENSG00000166133
ENSG00000166135
ENSG00000166140
ENSG00000166143
ENSG00000166145
ENSG00000166148
ENSG00000166152
ENSG00000166159
ENSG00000166160
ENSG00000166164
ENSG00000166166
ENSG00000166167
ENSG00000166169
ENSG00000166170
ENSG00000166171
ENSG00000166173
ENSG00000166181
ENSG00000166188
ENSG00000166192
ENSG00000166197
ENSG00000166200
ENSG00000166206
ENSG00000166220
ENSG00000166224
ENSG00000166225
ENSG00000166233
ENSG00000166246
ENSG00000166250

ENSG00000166257
ENSG00000166260
ENSG00000166261
ENSG00000166263
ENSG00000166266
ENSG00000166272
ENSG00000166275
ENSG00000166278
ENSG00000166289
ENSG00000166292
ENSG00000166295
ENSG00000166313
ENSG00000166317
ENSG00000166321
ENSG00000166323
ENSG00000166326
ENSG00000166329
ENSG00000166340
ENSG00000166342
ENSG00000166343
ENSG00000166348
ENSG00000166349
ENSG00000166351
ENSG00000166359
ENSG00000166387
ENSG00000166391
ENSG00000166396
ENSG00000166398
ENSG00000166402
ENSG00000166405
ENSG00000166411
ENSG00000166415
ENSG00000166426
ENSG00000166432
ENSG00000166435
ENSG00000166436
ENSG00000166439
ENSG00000166441

ENSG00000166444
ENSG00000166446
ENSG00000166448
ENSG00000166450
ENSG00000166451
ENSG00000166452
ENSG00000166454
ENSG00000166455
ENSG00000166471
ENSG00000166478
ENSG00000166479
ENSG00000166482
ENSG00000166483
ENSG00000166484
ENSG00000166501
ENSG00000166503
ENSG00000166508
ENSG00000166509
ENSG00000166510
ENSG00000166523
ENSG00000166526
ENSG00000166527
ENSG00000166529
ENSG00000166532
ENSG00000166535
ENSG00000166546
ENSG00000166548
ENSG00000166557
ENSG00000166562
ENSG00000166569
ENSG00000166573
ENSG00000166575
ENSG00000166578
ENSG00000166579
ENSG00000166592
ENSG00000166619
ENSG00000166634
ENSG00000166664

ENSG00000166669
ENSG00000166676
ENSG00000166682
ENSG00000166689
ENSG00000166704
ENSG00000166707
ENSG00000166710
ENSG00000166716
ENSG00000166734
ENSG00000166736
ENSG00000166747
ENSG00000166750
ENSG00000166762
ENSG00000166780
ENSG00000166783
ENSG00000166793
ENSG00000166796
ENSG00000166797
ENSG00000166800
ENSG00000166801
ENSG00000166813
ENSG00000166819
ENSG00000166821
ENSG00000166822
ENSG00000166823
ENSG00000166828
ENSG00000166831
ENSG00000166833
ENSG00000166839
ENSG00000166840
ENSG00000166845
ENSG00000166847
ENSG00000166848
ENSG00000166851
ENSG00000166855
ENSG00000166856
ENSG00000166860
ENSG00000166862

ENSG00000166866
ENSG00000166869
ENSG00000166881
ENSG00000166886
ENSG00000166887
ENSG00000166888
ENSG00000166889
ENSG00000166900
ENSG00000166902
ENSG00000166908
ENSG00000166912
ENSG00000166913
ENSG00000166922
ENSG00000166923
ENSG00000166924
ENSG00000166925
ENSG00000166927
ENSG00000166946
ENSG00000166949
ENSG00000166959
ENSG00000166961
ENSG00000166963
ENSG00000166974
ENSG00000166979
ENSG00000166986
ENSG00000166987
ENSG00000166997
ENSG00000167004
ENSG00000167005
ENSG00000167011
ENSG00000167034
ENSG00000167037
ENSG00000167065
ENSG00000167074
ENSG00000167080
ENSG00000167081
ENSG00000167083
ENSG00000167085

ENSG00000167088
ENSG00000167100
ENSG00000167103
ENSG00000167105
ENSG00000167106
ENSG00000167110
ENSG00000167112
ENSG00000167113
ENSG00000167114
ENSG00000167118
ENSG00000167123
ENSG00000167130
ENSG00000167131
ENSG00000167157
ENSG00000167165
ENSG00000167173
ENSG00000167178
ENSG00000167182
ENSG00000167183
ENSG00000167186
ENSG00000167191
ENSG00000167193
ENSG00000167195
ENSG00000167196
ENSG00000167202
ENSG00000167207
ENSG00000167208
ENSG00000167210
ENSG00000167216
ENSG00000167220
ENSG00000167232
ENSG00000167244
ENSG00000167257
ENSG00000167258
ENSG00000167272
ENSG00000167280
ENSG00000167281
ENSG00000167283

ENSG00000167291
ENSG00000167302
ENSG00000167306
ENSG00000167323
ENSG00000167332
ENSG00000167333
ENSG00000167371
ENSG00000167378
ENSG00000167384
ENSG00000167395
ENSG00000167397
ENSG00000167419
ENSG00000167460
ENSG00000167461
ENSG00000167470
ENSG00000167483
ENSG00000167487
ENSG00000167491
ENSG00000167508
ENSG00000167513
ENSG00000167515
ENSG00000167523
ENSG00000167524
ENSG00000167525
ENSG00000167526
ENSG00000167528
ENSG00000167531
ENSG00000167535
ENSG00000167536
ENSG00000167543
ENSG00000167548
ENSG00000167549
ENSG00000167552
ENSG00000167562
ENSG00000167565
ENSG00000167580
ENSG00000167588
ENSG00000167595

ENSG00000167600
ENSG00000167601
ENSG00000167604
ENSG00000167612
ENSG00000167613
ENSG00000167614
ENSG00000167615
ENSG00000167619
ENSG00000167625
ENSG00000167632
ENSG00000167633
ENSG00000167635
ENSG00000167642
ENSG00000167645
ENSG00000167653
ENSG00000167654
ENSG00000167656
ENSG00000167658
ENSG00000167670
ENSG00000167671
ENSG00000167676
ENSG00000167680
ENSG00000167693
ENSG00000167695
ENSG00000167701
ENSG00000167702
ENSG00000167703
ENSG00000167705
ENSG00000167711
ENSG00000167716
ENSG00000167720
ENSG00000167721
ENSG00000167723
ENSG00000167733
ENSG00000167740
ENSG00000167741
ENSG00000167747
ENSG00000167751

ENSG00000167754
ENSG00000167755
ENSG00000167759
ENSG00000167767
ENSG00000167768
ENSG00000167769
ENSG00000167770
ENSG00000167771
ENSG00000167772
ENSG00000167778
ENSG00000167779
ENSG00000167780
ENSG00000167785
ENSG00000167797
ENSG00000167799
ENSG00000167800
ENSG00000167815
ENSG00000167842
ENSG00000167850
ENSG00000167851
ENSG00000167861
ENSG00000167862
ENSG00000167880
ENSG00000167889
ENSG00000167895
ENSG00000167900
ENSG00000167904
ENSG00000167914
ENSG00000167916
ENSG00000167920
ENSG00000167925
ENSG00000167930
ENSG00000167941
ENSG00000167962
ENSG00000167964
ENSG00000167965
ENSG00000167967
ENSG00000167969

ENSG00000167971
ENSG00000167972
ENSG00000167977
ENSG00000167978
ENSG00000167981
ENSG00000167984
ENSG00000167985
ENSG00000167986
ENSG00000167987
ENSG00000167994
ENSG00000167995
ENSG00000167996
ENSG00000168000
ENSG00000168002
ENSG00000168003
ENSG00000168004
ENSG00000168005
ENSG00000168014
ENSG00000168016
ENSG00000168032
ENSG00000168036
ENSG00000168040
ENSG00000168056
ENSG00000168060
ENSG00000168062
ENSG00000168065
ENSG00000168066
ENSG00000168067
ENSG00000168077
ENSG00000168079
ENSG00000168090
ENSG00000168092
ENSG00000168096
ENSG00000168101
ENSG00000168118
ENSG00000168135
ENSG00000168137
ENSG00000168140

ENSG00000168143
ENSG00000168148
ENSG00000168152
ENSG00000168159
ENSG00000168172
ENSG00000168175
ENSG00000168209
ENSG00000168214
ENSG00000168216
ENSG00000168228
ENSG00000168229
ENSG00000168234
ENSG00000168237
ENSG00000168243
ENSG00000168246
ENSG00000168255
ENSG00000168256
ENSG00000168259
ENSG00000168263
ENSG00000168264
ENSG00000168269
ENSG00000168280
ENSG00000168282
ENSG00000168283
ENSG00000168286
ENSG00000168291
ENSG00000168297
ENSG00000168300
ENSG00000168301
ENSG00000168303
ENSG00000168309
ENSG00000168310
ENSG00000168314
ENSG00000168329
ENSG00000168333
ENSG00000168334
ENSG00000168348
ENSG00000168350

ENSG00000168356
ENSG00000168374
ENSG00000168385
ENSG00000168389
ENSG00000168393
ENSG00000168394
ENSG00000168395
ENSG00000168397
ENSG00000168398
ENSG00000168411
ENSG00000168418
ENSG00000168421
ENSG00000168438
ENSG00000168439
ENSG00000168447
ENSG00000168453
ENSG00000168461
ENSG00000168476
ENSG00000168477
ENSG00000168481
ENSG00000168484
ENSG00000168487
ENSG00000168488
ENSG00000168490
ENSG00000168491
ENSG00000168495
ENSG00000168496
ENSG00000168505
ENSG00000168509
ENSG00000168522
ENSG00000168530
ENSG00000168538
ENSG00000168539
ENSG00000168546
ENSG00000168556
ENSG00000168564
ENSG00000168566
ENSG00000168569

ENSG00000168575
ENSG00000168591
ENSG00000168610
ENSG00000168612
ENSG00000168615
ENSG00000168619
ENSG00000168621
ENSG00000168631
ENSG00000168634
ENSG00000168661
ENSG00000168672
ENSG00000168675
ENSG00000168679
ENSG00000168685
ENSG00000168701
ENSG00000168702
ENSG00000168703
ENSG00000168710
ENSG00000168724
ENSG00000168734
ENSG00000168743
ENSG00000168748
ENSG00000168754
ENSG00000168758
ENSG00000168763
ENSG00000168765
ENSG00000168769
ENSG00000168772
ENSG00000168778
ENSG00000168779
ENSG00000168781
ENSG00000168785
ENSG00000168792
ENSG00000168795
ENSG00000168802
ENSG00000168803
ENSG00000168806
ENSG00000168807

ENSG00000168813
ENSG00000168818
ENSG00000168824
ENSG00000168826
ENSG00000168827
ENSG00000168843
ENSG00000168872
ENSG00000168874
ENSG00000168875
ENSG00000168876
ENSG00000168878
ENSG00000168883
ENSG00000168884
ENSG00000168887
ENSG00000168890
ENSG00000168894
ENSG00000168903
ENSG00000168904
ENSG00000168906
ENSG00000168907
ENSG00000168913
ENSG00000168916
ENSG00000168918
ENSG00000168924
ENSG00000168936
ENSG00000168938
ENSG00000168939
ENSG00000168944
ENSG00000168952
ENSG00000168958
ENSG00000168959
ENSG00000168961
ENSG00000168993
ENSG00000168994
ENSG00000168995
ENSG00000169006
ENSG00000169016
ENSG00000169018

ENSG00000169019
ENSG00000169021
ENSG00000169031
ENSG00000169032
ENSG00000169035
ENSG00000169045
ENSG00000169047
ENSG00000169057
ENSG00000169067
ENSG00000169071
ENSG00000169083
ENSG00000169084
ENSG00000169085
ENSG00000169100
ENSG00000169105
ENSG00000169116
ENSG00000169118
ENSG00000169122
ENSG00000169129
ENSG00000169131
ENSG00000169136
ENSG00000169139
ENSG00000169155
ENSG00000169174
ENSG00000169180
ENSG00000169181
ENSG00000169184
ENSG00000169188
ENSG00000169193
ENSG00000169194
ENSG00000169213
ENSG00000169217
ENSG00000169218
ENSG00000169221
ENSG00000169223
ENSG00000169224
ENSG00000169228
ENSG00000169230

ENSG00000169239
ENSG00000169241
ENSG00000169242
ENSG00000169245
ENSG00000169247
ENSG00000169248
ENSG00000169251
ENSG00000169252
ENSG00000169255
ENSG00000169282
ENSG00000169291
ENSG00000169302
ENSG00000169313
ENSG00000169314
ENSG00000169330
ENSG00000169340
ENSG00000169347
ENSG00000169359
ENSG00000169372
ENSG00000169375
ENSG00000169379
ENSG00000169398
ENSG00000169403
ENSG00000169410
ENSG00000169413
ENSG00000169418
ENSG00000169427
ENSG00000169429
ENSG00000169432
ENSG00000169435
ENSG00000169439
ENSG00000169446
ENSG00000169474
ENSG00000169490
ENSG00000169495
ENSG00000169499
ENSG00000169504
ENSG00000169507

ENSG00000169508
ENSG00000169515
ENSG00000169519
ENSG00000169548
ENSG00000169550
ENSG00000169554
ENSG00000169562
ENSG00000169567
ENSG00000169570
ENSG00000169575
ENSG00000169592
ENSG00000169594
ENSG00000169598
ENSG00000169604
ENSG00000169607
ENSG00000169609
ENSG00000169612
ENSG00000169618
ENSG00000169629
ENSG00000169635
ENSG00000169641
ENSG00000169660
ENSG00000169679
ENSG00000169682
ENSG00000169683
ENSG00000169684
ENSG00000169689
ENSG00000169692
ENSG00000169696
ENSG00000169714
ENSG00000169715
ENSG00000169718
ENSG00000169727
ENSG00000169733
ENSG00000169740
ENSG00000169744
ENSG00000169750
ENSG00000169752

ENSG00000169756
ENSG00000169758
ENSG00000169760
ENSG00000169762
ENSG00000169764
ENSG00000169777
ENSG00000169789
ENSG00000169807
ENSG00000169813
ENSG00000169814
ENSG00000169826
ENSG00000169836
ENSG00000169851
ENSG00000169855
ENSG00000169856
ENSG00000169857
ENSG00000169860
ENSG00000169862
ENSG00000169871
ENSG00000169877
ENSG00000169884
ENSG00000169891
ENSG00000169895
ENSG00000169896
ENSG00000169900
ENSG00000169903
ENSG00000169905
ENSG00000169908
ENSG00000169914
ENSG00000169919
ENSG00000169925
ENSG00000169926
ENSG00000169933
ENSG00000169951
ENSG00000169953
ENSG00000169957
ENSG00000169964
ENSG00000169967

ENSG00000169976
ENSG00000169981
ENSG00000169991
ENSG00000169992
ENSG00000170004
ENSG00000170006
ENSG00000170011
ENSG00000170017
ENSG00000170027
ENSG00000170035
ENSG00000170043
ENSG00000170044
ENSG00000170049
ENSG00000170054
ENSG00000170074
ENSG00000170075
ENSG00000170085
ENSG00000170088
ENSG00000170091
ENSG00000170092
ENSG00000170099
ENSG00000170100
ENSG00000170113
ENSG00000170144
ENSG00000170145
ENSG00000170153
ENSG00000170166
ENSG00000170175
ENSG00000170180
ENSG00000170185
ENSG00000170191
ENSG00000170209
ENSG00000170214
ENSG00000170222
ENSG00000170234
ENSG00000170242
ENSG00000170248
ENSG00000170260

ENSG00000170262
ENSG00000170264
ENSG00000170265
ENSG00000170271
ENSG00000170275
ENSG00000170276
ENSG00000170279
ENSG00000170289
ENSG00000170290
ENSG00000170291
ENSG00000170296
ENSG00000170312
ENSG00000170324
ENSG00000170325
ENSG00000170340
ENSG00000170345
ENSG00000170348
ENSG00000170364
ENSG00000170365
ENSG00000170367
ENSG00000170370
ENSG00000170373
ENSG00000170374
ENSG00000170379
ENSG00000170381
ENSG00000170382
ENSG00000170390
ENSG00000170396
ENSG00000170412
ENSG00000170417
ENSG00000170419
ENSG00000170423
ENSG00000170425
ENSG00000170426
ENSG00000170439
ENSG00000170442
ENSG00000170454
ENSG00000170456

ENSG00000170464
ENSG00000170465
ENSG00000170471
ENSG00000170473
ENSG00000170476
ENSG00000170477
ENSG00000170482
ENSG00000170484
ENSG00000170485
ENSG00000170486
ENSG00000170500
ENSG00000170502
ENSG00000170509
ENSG00000170515
ENSG00000170522
ENSG00000170525
ENSG00000170537
ENSG00000170540
ENSG00000170542
ENSG00000170545
ENSG00000170549
ENSG00000170558
ENSG00000170561
ENSG00000170571
ENSG00000170577
ENSG00000170579
ENSG00000170581
ENSG00000170584
ENSG00000170604
ENSG00000170606
ENSG00000170608
ENSG00000170613
ENSG00000170615
ENSG00000170619
ENSG00000170624
ENSG00000170632
ENSG00000170633
ENSG00000170638

ENSG00000170653
ENSG00000170677
ENSG00000170681
ENSG00000170689
ENSG00000170703
ENSG00000170734
ENSG00000170743
ENSG00000170745
ENSG00000170759
ENSG00000170776
ENSG00000170777
ENSG00000170779
ENSG00000170786
ENSG00000170788
ENSG00000170801
ENSG00000170802
ENSG00000170807
ENSG00000170820
ENSG00000170832
ENSG00000170836
ENSG00000170852
ENSG00000170854
ENSG00000170855
ENSG00000170871
ENSG00000170873
ENSG00000170876
ENSG00000170889
ENSG00000170891
ENSG00000170892
ENSG00000170893
ENSG00000170899
ENSG00000170903
ENSG00000170909
ENSG00000170915
ENSG00000170921
ENSG00000170925
ENSG00000170927
ENSG00000170946

ENSG00000170948
ENSG00000170949
ENSG00000170954
ENSG00000170955
ENSG00000170956
ENSG00000170959
ENSG00000170961
ENSG00000170962
ENSG00000170965
ENSG00000170967
ENSG00000170989
ENSG00000171004
ENSG00000171016
ENSG00000171017
ENSG00000171033
ENSG00000171044
ENSG00000171045
ENSG00000171049
ENSG00000171053
ENSG00000171055
ENSG00000171056
ENSG00000171067
ENSG00000171097
ENSG00000171100
ENSG00000171102
ENSG00000171103
ENSG00000171105
ENSG00000171109
ENSG00000171115
ENSG00000171121
ENSG00000171124
ENSG00000171130
ENSG00000171132
ENSG00000171133
ENSG00000171135
ENSG00000171148
ENSG00000171150
ENSG00000171155

ENSG00000171160
ENSG00000171161
ENSG00000171163
ENSG00000171169
ENSG00000171189
ENSG00000171195
ENSG00000171201
ENSG00000171204
ENSG00000171206
ENSG00000171208
ENSG00000171209
ENSG00000171224
ENSG00000171227
ENSG00000171234
ENSG00000171236
ENSG00000171241
ENSG00000171243
ENSG00000171246
ENSG00000171262
ENSG00000171291
ENSG00000171295
ENSG00000171298
ENSG00000171302
ENSG00000171303
ENSG00000171310
ENSG00000171314
ENSG00000171316
ENSG00000171320
ENSG00000171345
ENSG00000171346
ENSG00000171357
ENSG00000171360
ENSG00000171365
ENSG00000171368
ENSG00000171385
ENSG00000171388
ENSG00000171396
ENSG00000171401

ENSG00000171403
ENSG00000171408
ENSG00000171425
ENSG00000171431
ENSG00000171435
ENSG00000171443
ENSG00000171444
ENSG00000171450
ENSG00000171451
ENSG00000171456
ENSG00000171466
ENSG00000171467
ENSG00000171469
ENSG00000171475
ENSG00000171476
ENSG00000171487
ENSG00000171488
ENSG00000171490
ENSG00000171492
ENSG00000171502
ENSG00000171509
ENSG00000171522
ENSG00000171530
ENSG00000171532
ENSG00000171533
ENSG00000171540
ENSG00000171551
ENSG00000171552
ENSG00000171560
ENSG00000171564
ENSG00000171566
ENSG00000171574
ENSG00000171587
ENSG00000171595
ENSG00000171596
ENSG00000171603
ENSG00000171604
ENSG00000171606

ENSG00000171608
ENSG00000171612
ENSG00000171617
ENSG00000171621
ENSG00000171631
ENSG00000171634
ENSG00000171643
ENSG00000171649
ENSG00000171657
ENSG00000171680
ENSG00000171681
ENSG00000171700
ENSG00000171703
ENSG00000171711
ENSG00000171714
ENSG00000171720
ENSG00000171722
ENSG00000171724
ENSG00000171729
ENSG00000171735
ENSG00000171766
ENSG00000171772
ENSG00000171773
ENSG00000171777
ENSG00000171786
ENSG00000171790
ENSG00000171791
ENSG00000171792
ENSG00000171798
ENSG00000171812
ENSG00000171817
ENSG00000171823
ENSG00000171827
ENSG00000171840
ENSG00000171843
ENSG00000171847
ENSG00000171848
ENSG00000171855

ENSG00000171861
ENSG00000171862
ENSG00000171864
ENSG00000171867
ENSG00000171872
ENSG00000171873
ENSG00000171877
ENSG00000171885
ENSG00000171903
ENSG00000171914
ENSG00000171916
ENSG00000171928
ENSG00000171940
ENSG00000171943
ENSG00000171953
ENSG00000171960
ENSG00000171962
ENSG00000171984
ENSG00000171988
ENSG00000171989
ENSG00000171992
ENSG00000172005
ENSG00000172006
ENSG00000172007
ENSG00000172014
ENSG00000172016
ENSG00000172046
ENSG00000172053
ENSG00000172057
ENSG00000172058
ENSG00000172059
ENSG00000172061
ENSG00000172062
ENSG00000172071
ENSG00000172081
ENSG00000172086
ENSG00000172113
ENSG00000172115

ENSG00000172116
ENSG00000172123
ENSG00000172137
ENSG00000172139
ENSG00000172156
ENSG00000172159
ENSG00000172164
ENSG00000172175
ENSG00000172183
ENSG00000172197
ENSG00000172201
ENSG00000172215
ENSG00000172232
ENSG00000172239
ENSG00000172243
ENSG00000172260
ENSG00000172262
ENSG00000172264
ENSG00000172269
ENSG00000172270
ENSG00000172273
ENSG00000172292
ENSG00000172296
ENSG00000172301
ENSG00000172315
ENSG00000172318
ENSG00000172322
ENSG00000172336
ENSG00000172339
ENSG00000172340
ENSG00000172345
ENSG00000172346
ENSG00000172348
ENSG00000172349
ENSG00000172350
ENSG00000172354
ENSG00000172361
ENSG00000172365

ENSG00000172366
ENSG00000172367
ENSG00000172375
ENSG00000172379
ENSG00000172380
ENSG00000172382
ENSG00000172399
ENSG00000172403
ENSG00000172404
ENSG00000172409
ENSG00000172432
ENSG00000172458
ENSG00000172461
ENSG00000172466
ENSG00000172468
ENSG00000172469
ENSG00000172478
ENSG00000172482
ENSG00000172493
ENSG00000172508
ENSG00000172530
ENSG00000172531
ENSG00000172534
ENSG00000172548
ENSG00000172568
ENSG00000172572
ENSG00000172575
ENSG00000172578
ENSG00000172590
ENSG00000172602
ENSG00000172613
ENSG00000172638
ENSG00000172663
ENSG00000172667
ENSG00000172671
ENSG00000172673
ENSG00000172689
ENSG00000172716

ENSG00000172724
ENSG00000172725
ENSG00000172728
ENSG00000172731
ENSG00000172752
ENSG00000172757
ENSG00000172765
ENSG00000172766
ENSG00000172775
ENSG00000172780
ENSG00000172782
ENSG00000172785
ENSG00000172794
ENSG00000172795
ENSG00000172817
ENSG00000172818
ENSG00000172819
ENSG00000172828
ENSG00000172830
ENSG00000172831
ENSG00000172840
ENSG00000172845
ENSG00000172867
ENSG00000172869
ENSG00000172878
ENSG00000172888
ENSG00000172889
ENSG00000172890
ENSG00000172893
ENSG00000172901
ENSG00000172915
ENSG00000172922
ENSG00000172927
ENSG00000172932
ENSG00000172935
ENSG00000172936
ENSG00000172939
ENSG00000172940

ENSG00000172943
ENSG00000172954
ENSG00000172955
ENSG00000172967
ENSG00000172969
ENSG00000172977
ENSG00000172985
ENSG00000172987
ENSG00000172992
ENSG00000172995
ENSG00000173011
ENSG00000173020
ENSG00000173039
ENSG00000173064
ENSG00000173065
ENSG00000173068
ENSG00000173077
ENSG00000173083
ENSG00000173110
ENSG00000173113
ENSG00000173120
ENSG00000173145
ENSG00000173153
ENSG00000173156
ENSG00000173166
ENSG00000173175
ENSG00000173193
ENSG00000173200
ENSG00000173208
ENSG00000173210
ENSG00000173212
ENSG00000173218
ENSG00000173221
ENSG00000173226
ENSG00000173227
ENSG00000173230
ENSG00000173237
ENSG00000173253

ENSG00000173258
ENSG00000173262
ENSG00000173264
ENSG00000173267
ENSG00000173269
ENSG00000173273
ENSG00000173275
ENSG00000173276
ENSG00000173281
ENSG00000173320
ENSG00000173327
ENSG00000173334
ENSG00000173335
ENSG00000173338
ENSG00000173349
ENSG00000173372
ENSG00000173391
ENSG00000173402
ENSG00000173404
ENSG00000173406
ENSG00000173418
ENSG00000173421
ENSG00000173432
ENSG00000173436
ENSG00000173451
ENSG00000173452
ENSG00000173456
ENSG00000173457
ENSG00000173464
ENSG00000173467
ENSG00000173473
ENSG00000173480
ENSG00000173482
ENSG00000173511
ENSG00000173517
ENSG00000173530
ENSG00000173535
ENSG00000173540

ENSG00000173542
ENSG00000173546
ENSG00000173548
ENSG00000173567
ENSG00000173572
ENSG00000173575
ENSG00000173585
ENSG00000173598
ENSG00000173610
ENSG00000173611
ENSG00000173614
ENSG00000173621
ENSG00000173626
ENSG00000173627
ENSG00000173638
ENSG00000173641
ENSG00000173653
ENSG00000173674
ENSG00000173678
ENSG00000173681
ENSG00000173692
ENSG00000173698
ENSG00000173699
ENSG00000173702
ENSG00000173705
ENSG00000173706
ENSG00000173714
ENSG00000173726
ENSG00000173744
ENSG00000173757
ENSG00000173762
ENSG00000173786
ENSG00000173801
ENSG00000173805
ENSG00000173809
ENSG00000173812
ENSG00000173818
ENSG00000173821

ENSG00000173825
ENSG00000173826
ENSG00000173838
ENSG00000173846
ENSG00000173848
ENSG00000173852
ENSG00000173868
ENSG00000173889
ENSG00000173890
ENSG00000173894
ENSG00000173898
ENSG00000173905
ENSG00000173915
ENSG00000173917
ENSG00000173918
ENSG00000173926
ENSG00000173928
ENSG00000173930
ENSG00000173933
ENSG00000173947
ENSG00000173950
ENSG00000173960
ENSG00000173976
ENSG00000173991
ENSG00000173992
ENSG00000174007
ENSG00000174010
ENSG00000174013
ENSG00000174016
ENSG00000174032
ENSG00000174038
ENSG00000174059
ENSG00000174080
ENSG00000174099
ENSG00000174106
ENSG00000174109
ENSG00000174123
ENSG00000174130

ENSG00000174132
ENSG00000174136
ENSG00000174137
ENSG00000174145
ENSG00000174151
ENSG00000174156
ENSG00000174165
ENSG00000174173
ENSG00000174175
ENSG00000174197
ENSG00000174206
ENSG00000174226
ENSG00000174227
ENSG00000174233
ENSG00000174236
ENSG00000174238
ENSG00000174243
ENSG00000174255
ENSG00000174282
ENSG00000174292
ENSG00000174306
ENSG00000174307
ENSG00000174325
ENSG00000174348
ENSG00000174358
ENSG00000174370
ENSG00000174371
ENSG00000174373
ENSG00000174429
ENSG00000174437
ENSG00000174442
ENSG00000174450
ENSG00000174456
ENSG00000174469
ENSG00000174473
ENSG00000174483
ENSG00000174485
ENSG00000174498

ENSG00000174500
ENSG00000174501
ENSG00000174502
ENSG00000174514
ENSG00000174516
ENSG00000174527
ENSG00000174529
ENSG00000174547
ENSG00000174562
ENSG00000174567
ENSG00000174574
ENSG00000174576
ENSG00000174579
ENSG00000174586
ENSG00000174599
ENSG00000174600
ENSG00000174606
ENSG00000174607
ENSG00000174611
ENSG00000174628
ENSG00000174640
ENSG00000174652
ENSG00000174667
ENSG00000174669
ENSG00000174672
ENSG00000174684
ENSG00000174695
ENSG00000174697
ENSG00000174705
ENSG00000174721
ENSG00000174738
ENSG00000174744
ENSG00000174748
ENSG00000174749
ENSG00000174775
ENSG00000174780
ENSG00000174792
ENSG00000174796

ENSG00000174799
ENSG00000174804
ENSG00000174807
ENSG00000174808
ENSG00000174827
ENSG00000174837
ENSG00000174839
ENSG00000174840
ENSG00000174844
ENSG00000174886
ENSG00000174899
ENSG00000174903
ENSG00000174915
ENSG00000174928
ENSG00000174938
ENSG00000174939
ENSG00000174944
ENSG00000174945
ENSG00000174946
ENSG00000174950
ENSG00000174951
ENSG00000174953
ENSG00000174963
ENSG00000174989
ENSG00000174990
ENSG00000174996
ENSG00000175018
ENSG00000175029
ENSG00000175040
ENSG00000175048
ENSG00000175066
ENSG00000175073
ENSG00000175077
ENSG00000175084
ENSG00000175087
ENSG00000175093
ENSG00000175104
ENSG00000175106

ENSG00000175115
ENSG00000175121
ENSG00000175130
ENSG00000175137
ENSG00000175155
ENSG00000175161
ENSG00000175166
ENSG00000175175
ENSG00000175182
ENSG00000175189
ENSG00000175193
ENSG00000175197
ENSG00000175198
ENSG00000175203
ENSG00000175206
ENSG00000175215
ENSG00000175216
ENSG00000175220
ENSG00000175221
ENSG00000175224
ENSG00000175229
ENSG00000175262
ENSG00000175264
ENSG00000175265
ENSG00000175267
ENSG00000175274
ENSG00000175279
ENSG00000175287
ENSG00000175294
ENSG00000175305
ENSG00000175311
ENSG00000175318
ENSG00000175322
ENSG00000175324
ENSG00000175329
ENSG00000175334
ENSG00000175336
ENSG00000175344

ENSG00000175348
ENSG00000175352
ENSG00000175354
ENSG00000175356
ENSG00000175376
ENSG00000175387
ENSG00000175395
ENSG00000175414
ENSG00000175416
ENSG00000175426
ENSG00000175445
ENSG00000175449
ENSG00000175455
ENSG00000175463
ENSG00000175467
ENSG00000175470
ENSG00000175471
ENSG00000175482
ENSG00000175489
ENSG00000175497
ENSG00000175505
ENSG00000175518
ENSG00000175536
ENSG00000175538
ENSG00000175544
ENSG00000175548
ENSG00000175550
ENSG00000175556
ENSG00000175564
ENSG00000175573
ENSG00000175575
ENSG00000175581
ENSG00000175582
ENSG00000175591
ENSG00000175592
ENSG00000175595
ENSG00000175600
ENSG00000175606

ENSG00000175634
ENSG00000175643
ENSG00000175662
ENSG00000175697
ENSG00000175707
ENSG00000175711
ENSG00000175727
ENSG00000175745
ENSG00000175764
ENSG00000175766
ENSG00000175779
ENSG00000175782
ENSG00000175785
ENSG00000175787
ENSG00000175793
ENSG00000175806
ENSG00000175809
ENSG00000175820
ENSG00000175826
ENSG00000175832
ENSG00000175854
ENSG00000175857
ENSG00000175866
ENSG00000175868
ENSG00000175874
ENSG00000175879
ENSG00000175893
ENSG00000175894
ENSG00000175895
ENSG00000175906
ENSG00000175928
ENSG00000175931
ENSG00000175938
ENSG00000175970
ENSG00000175984
ENSG00000176014
ENSG00000176018
ENSG00000176020

ENSG00000176022
ENSG00000176029
ENSG00000176046
ENSG00000176049
ENSG00000176055
ENSG00000176058
ENSG00000176076
ENSG00000176087
ENSG00000176095
ENSG00000176101
ENSG00000176105
ENSG00000176108
ENSG00000176125
ENSG00000176136
ENSG00000176142
ENSG00000176148
ENSG00000176153
ENSG00000176155
ENSG00000176160
ENSG00000176165
ENSG00000176170
ENSG00000176177
ENSG00000176182
ENSG00000176204
ENSG00000176209
ENSG00000176244
ENSG00000176261
ENSG00000176273
ENSG00000176293
ENSG00000176358
ENSG00000176371
ENSG00000176381
ENSG00000176383
ENSG00000176386
ENSG00000176387
ENSG00000176390
ENSG00000176396
ENSG00000176401

ENSG00000176402
ENSG00000176406
ENSG00000176407
ENSG00000176410
ENSG00000176422
ENSG00000176428
ENSG00000176435
ENSG00000176438
ENSG00000176444
ENSG00000176454
ENSG00000176463
ENSG00000176472
ENSG00000176473
ENSG00000176485
ENSG00000176490
ENSG00000176532
ENSG00000176533
ENSG00000176542
ENSG00000176563
ENSG00000176566
ENSG00000176595
ENSG00000176597
ENSG00000176601
ENSG00000176619
ENSG00000176623
ENSG00000176624
ENSG00000176635
ENSG00000176641
ENSG00000176658
ENSG00000176678
ENSG00000176681
ENSG00000176697
ENSG00000176714
ENSG00000176715
ENSG00000176720
ENSG00000176731
ENSG00000176732
ENSG00000176749

ENSG00000176771
ENSG00000176774
ENSG00000176782
ENSG00000176783
ENSG00000176788
ENSG00000176834
ENSG00000176842
ENSG00000176853
ENSG00000176871
ENSG00000176884
ENSG00000176887
ENSG00000176890
ENSG00000176896
ENSG00000176907
ENSG00000176909
ENSG00000176915
ENSG00000176920
ENSG00000176927
ENSG00000176928
ENSG00000176945
ENSG00000176953
ENSG00000176956
ENSG00000176971
ENSG00000176973
ENSG00000176974
ENSG00000176986
ENSG00000176994
ENSG00000177000
ENSG00000177023
ENSG00000177030
ENSG00000177034
ENSG00000177042
ENSG00000177051
ENSG00000177058
ENSG00000177076
ENSG00000177082
ENSG00000177084
ENSG00000177096

ENSG00000177098
ENSG00000177105
ENSG00000177106
ENSG00000177108
ENSG00000177119
ENSG00000177125
ENSG00000177138
ENSG00000177143
ENSG00000177150
ENSG00000177169
ENSG00000177181
ENSG00000177182
ENSG00000177189
ENSG00000177200
ENSG00000177202
ENSG00000177225
ENSG00000177238
ENSG00000177239
ENSG00000177257
ENSG00000177272
ENSG00000177283
ENSG00000177291
ENSG00000177294
ENSG00000177300
ENSG00000177301
ENSG00000177302
ENSG00000177303
ENSG00000177324
ENSG00000177352
ENSG00000177354
ENSG00000177363
ENSG00000177370
ENSG00000177380
ENSG00000177383
ENSG00000177398
ENSG00000177409
ENSG00000177414
ENSG00000177427

ENSG00000177455
ENSG00000177463
ENSG00000177464
ENSG00000177468
ENSG00000177469
ENSG00000177479
ENSG00000177483
ENSG00000177485
ENSG00000177494
ENSG00000177511
ENSG00000177519
ENSG00000177542
ENSG00000177551
ENSG00000177565
ENSG00000177570
ENSG00000177576
ENSG00000177599
ENSG00000177602
ENSG00000177606
ENSG00000177613
ENSG00000177614
ENSG00000177627
ENSG00000177628
ENSG00000177646
ENSG00000177663
ENSG00000177669
ENSG00000177674
ENSG00000177679
ENSG00000177683
ENSG00000177685
ENSG00000177688
ENSG00000177689
ENSG00000177694
ENSG00000177697
ENSG00000177700
ENSG00000177706
ENSG00000177707
ENSG00000177721

ENSG00000177728
ENSG00000177731
ENSG00000177732
ENSG00000177733
ENSG00000177807
ENSG00000177830
ENSG00000177839
ENSG00000177842
ENSG00000177853
ENSG00000177854
ENSG00000177868
ENSG00000177875
ENSG00000177879
ENSG00000177885
ENSG00000177888
ENSG00000177889
ENSG00000177917
ENSG00000177932
ENSG00000177946
ENSG00000177951
ENSG00000177963
ENSG00000177971
ENSG00000177981
ENSG00000177984
ENSG00000177990
ENSG00000177994
ENSG00000178021
ENSG00000178026
ENSG00000178031
ENSG00000178033
ENSG00000178038
ENSG00000178053
ENSG00000178057
ENSG00000178074
ENSG00000178075
ENSG00000178093
ENSG00000178096
ENSG00000178104

ENSG00000178105
ENSG00000178149
ENSG00000178150
ENSG00000178163
ENSG00000178171
ENSG00000178172
ENSG00000178175
ENSG00000178177
ENSG00000178184
ENSG00000178187
ENSG00000178188
ENSG00000178199
ENSG00000178202
ENSG00000178209
ENSG00000178217
ENSG00000178222
ENSG00000178233
ENSG00000178234
ENSG00000178235
ENSG00000178279
ENSG00000178295
ENSG00000178301
ENSG00000178307
ENSG00000178338
ENSG00000178343
ENSG00000178363
ENSG00000178372
ENSG00000178381
ENSG00000178385
ENSG00000178397
ENSG00000178401
ENSG00000178403
ENSG00000178409
ENSG00000178425
ENSG00000178445
ENSG00000178449
ENSG00000178460
ENSG00000178462

ENSG00000178502
ENSG00000178522
ENSG00000178531
ENSG00000178537
ENSG00000178538
ENSG00000178562
ENSG00000178567
ENSG00000178568
ENSG00000178573
ENSG00000178585
ENSG00000178597
ENSG00000178607
ENSG00000178662
ENSG00000178665
ENSG00000178690
ENSG00000178691
ENSG00000178695
ENSG00000178700
ENSG00000178718
ENSG00000178719
ENSG00000178726
ENSG00000178732
ENSG00000178734
ENSG00000178752
ENSG00000178761
ENSG00000178764
ENSG00000178772
ENSG00000178773
ENSG00000178789
ENSG00000178796
ENSG00000178802
ENSG00000178821
ENSG00000178826
ENSG00000178828
ENSG00000178852
ENSG00000178860
ENSG00000178878
ENSG00000178882

ENSG00000178904
ENSG00000178919
ENSG00000178921
ENSG00000178927
ENSG00000178928
ENSG00000178934
ENSG00000178935
ENSG00000178951
ENSG00000178952
ENSG00000178965
ENSG00000178966
ENSG00000178971
ENSG00000178974
ENSG00000178980
ENSG00000178988
ENSG00000178996
ENSG00000179010
ENSG00000179021
ENSG00000179023
ENSG00000179029
ENSG00000179041
ENSG00000179051
ENSG00000179057
ENSG00000179059
ENSG00000179083
ENSG00000179091
ENSG00000179094
ENSG00000179097
ENSG00000179104
ENSG00000179111
ENSG00000179115
ENSG00000179119
ENSG00000179133
ENSG00000179134
ENSG00000179142
ENSG00000179148
ENSG00000179151
ENSG00000179152

ENSG00000179163
ENSG00000179165
ENSG00000179178
ENSG00000179195
ENSG00000179213
ENSG00000179240
ENSG00000179241
ENSG00000179242
ENSG00000179256
ENSG00000179262
ENSG00000179270
ENSG00000179284
ENSG00000179292
ENSG00000179295
ENSG00000179299
ENSG00000179300
ENSG00000179304
ENSG00000179314
ENSG00000179335
ENSG00000179344
ENSG00000179348
ENSG00000179361
ENSG00000179364
ENSG00000179387
ENSG00000179388
ENSG00000179399
ENSG00000179403
ENSG00000179409
ENSG00000179431
ENSG00000179454
ENSG00000179455
ENSG00000179456
ENSG00000179476
ENSG00000179520
ENSG00000179526
ENSG00000179528
ENSG00000179532
ENSG00000179542

ENSG00000179546
ENSG00000179562
ENSG00000179564
ENSG00000179593
ENSG00000179598
ENSG00000179603
ENSG00000179604
ENSG00000179627
ENSG00000179630
ENSG00000179632
ENSG00000179709
ENSG00000179761
ENSG00000179772
ENSG00000179776
ENSG00000179813
ENSG00000179820
ENSG00000179826
ENSG00000179832
ENSG00000179833
ENSG00000179841
ENSG00000179855
ENSG00000179889
ENSG00000179909
ENSG00000179912
ENSG00000179913
ENSG00000179915
ENSG00000179922
ENSG00000179930
ENSG00000179933
ENSG00000179938
ENSG00000179941
ENSG00000179943
ENSG00000179950
ENSG00000179958
ENSG00000179981
ENSG00000180008
ENSG00000180011
ENSG00000180035

ENSG00000180043
ENSG00000180044
ENSG00000180071
ENSG00000180089
ENSG00000180090
ENSG00000180113
ENSG00000180116
ENSG00000180138
ENSG00000180155
ENSG00000180182
ENSG00000180185
ENSG00000180190
ENSG00000180198
ENSG00000180205
ENSG00000180219
ENSG00000180228
ENSG00000180233
ENSG00000180251
ENSG00000180263
ENSG00000180264
ENSG00000180269
ENSG00000180287
ENSG00000180304
ENSG00000180316
ENSG00000180329
ENSG00000180332
ENSG00000180336
ENSG00000180340
ENSG00000180347
ENSG00000180353
ENSG00000180354
ENSG00000180357
ENSG00000180370
ENSG00000180398
ENSG00000180423
ENSG00000180424
ENSG00000180425
ENSG00000180432

ENSG00000180440
ENSG00000180447
ENSG00000180448
ENSG00000180481
ENSG00000180488
ENSG00000180509
ENSG00000180530
ENSG00000180537
ENSG00000180543
ENSG00000180549
ENSG00000180592
ENSG00000180611
ENSG00000180616
ENSG00000180626
ENSG00000180628
ENSG00000180644
ENSG00000180658
ENSG00000180660
ENSG00000180667
ENSG00000180694
ENSG00000180697
ENSG00000180720
ENSG00000180730
ENSG00000180739
ENSG00000180772
ENSG00000180773
ENSG00000180776
ENSG00000180785
ENSG00000180787
ENSG00000180801
ENSG00000180806
ENSG00000180817
ENSG00000180818
ENSG00000180822
ENSG00000180828
ENSG00000180834
ENSG00000180855
ENSG00000180871

ENSG00000180875
ENSG00000180881
ENSG00000180884
ENSG00000180891
ENSG00000180901
ENSG00000180902
ENSG00000180914
ENSG00000180917
ENSG00000180921
ENSG00000180929
ENSG00000180938
ENSG00000180957
ENSG00000180979
ENSG00000180998
ENSG00000180999
ENSG00000181004
ENSG00000181007
ENSG00000181013
ENSG00000181016
ENSG00000181019
ENSG00000181026
ENSG00000181027
ENSG00000181029
ENSG00000181031
ENSG00000181035
ENSG00000181036
ENSG00000181038
ENSG00000181045
ENSG00000181061
ENSG00000181072
ENSG00000181092
ENSG00000181104
ENSG00000181163
ENSG00000181192
ENSG00000181195
ENSG00000181215
ENSG00000181220
ENSG00000181234

ENSG00000181240
ENSG00000181264
ENSG00000181274
ENSG00000181284
ENSG00000181291
ENSG00000181315
ENSG00000181322
ENSG00000181333
ENSG00000181350
ENSG00000181374
ENSG00000181378
ENSG00000181381
ENSG00000181396
ENSG00000181404
ENSG00000181418
ENSG00000181433
ENSG00000181449
ENSG00000181458
ENSG00000181467
ENSG00000181472
ENSG00000181481
ENSG00000181513
ENSG00000181523
ENSG00000181541
ENSG00000181544
ENSG00000181555
ENSG00000181562
ENSG00000181577
ENSG00000181585
ENSG00000181588
ENSG00000181610
ENSG00000181619
ENSG00000181626
ENSG00000181631
ENSG00000181634
ENSG00000181638
ENSG00000181649
ENSG00000181652

ENSG00000181656
ENSG00000181666
ENSG00000181690
ENSG00000181704
ENSG00000181722
ENSG00000181744
ENSG00000181751
ENSG00000181754
ENSG00000181773
ENSG00000181778
ENSG00000181788
ENSG00000181789
ENSG00000181790
ENSG00000181804
ENSG00000181826
ENSG00000181827
ENSG00000181830
ENSG00000181847
ENSG00000181852
ENSG00000181856
ENSG00000181873
ENSG00000181885
ENSG00000181894
ENSG00000181896
ENSG00000181904
ENSG00000181915
ENSG00000181924
ENSG00000181929
ENSG00000181938
ENSG00000181965
ENSG00000181982
ENSG00000181991
ENSG00000182004
ENSG00000182010
ENSG00000182013
ENSG00000182022
ENSG00000182035
ENSG00000182040

ENSG00000182050
ENSG00000182054
ENSG00000182087
ENSG00000182093
ENSG00000182095
ENSG00000182103
ENSG00000182107
ENSG00000182108
ENSG00000182111
ENSG00000182118
ENSG00000182132
ENSG00000182134
ENSG00000182141
ENSG00000182149
ENSG00000182150
ENSG00000182156
ENSG00000182158
ENSG00000182162
ENSG00000182168
ENSG00000182173
ENSG00000182175
ENSG00000182180
ENSG00000182185
ENSG00000182195
ENSG00000182196
ENSG00000182197
ENSG00000182199
ENSG00000182208
ENSG00000182218
ENSG00000182223
ENSG00000182224
ENSG00000182230
ENSG00000182240
ENSG00000182253
ENSG00000182256
ENSG00000182257
ENSG00000182263
ENSG00000182271

ENSG00000182287
ENSG00000182307
ENSG00000182308
ENSG00000182318
ENSG00000182324
ENSG00000182326
ENSG00000182330
ENSG00000182333
ENSG00000182346
ENSG00000182359
ENSG00000182372
ENSG00000182378
ENSG00000182379
ENSG00000182389
ENSG00000182400
ENSG00000182405
ENSG00000182446
ENSG00000182450
ENSG00000182459
ENSG00000182463
ENSG00000182472
ENSG00000182473
ENSG00000182489
ENSG00000182492
ENSG00000182504
ENSG00000182508
ENSG00000182512
ENSG00000182518
ENSG00000182533
ENSG00000182534
ENSG00000182541
ENSG00000182544
ENSG00000182551
ENSG00000182552
ENSG00000182557
ENSG00000182566
ENSG00000182568
ENSG00000182575

ENSG00000182578
ENSG00000182580
ENSG00000182584
ENSG00000182585
ENSG00000182591
ENSG00000182601
ENSG00000182606
ENSG00000182621
ENSG00000182628
ENSG00000182631
ENSG00000182636
ENSG00000182667
ENSG00000182670
ENSG00000182676
ENSG00000182687
ENSG00000182704
ENSG00000182718
ENSG00000182732
ENSG00000182742
ENSG00000182747
ENSG00000182749
ENSG00000182752
ENSG00000182759
ENSG00000182771
ENSG00000182782
ENSG00000182795
ENSG00000182798
ENSG00000182809
ENSG00000182827
ENSG00000182831
ENSG00000182836
ENSG00000182853
ENSG00000182858
ENSG00000182870
ENSG00000182871
ENSG00000182872
ENSG00000182885
ENSG00000182890

ENSG00000182896
ENSG00000182901
ENSG00000182902
ENSG00000182903
ENSG00000182916
ENSG00000182919
ENSG00000182923
ENSG00000182931
ENSG00000182934
ENSG00000182938
ENSG00000182952
ENSG00000182957
ENSG00000182963
ENSG00000182968
ENSG00000182983
ENSG00000182985
ENSG00000183010
ENSG00000183011
ENSG00000183018
ENSG00000183020
ENSG00000183023
ENSG00000183032
ENSG00000183034
ENSG00000183036
ENSG00000183044
ENSG00000183048
ENSG00000183049
ENSG00000183054
ENSG00000183060
ENSG00000183066
ENSG00000183072
ENSG00000183077
ENSG00000183087
ENSG00000183092
ENSG00000183098
ENSG00000183111
ENSG00000183114
ENSG00000183117

ENSG00000183128
ENSG00000183134
ENSG00000183137
ENSG00000183145
ENSG00000183146
ENSG00000183148
ENSG00000183153
ENSG00000183155
ENSG00000183160
ENSG00000183161
ENSG00000183166
ENSG00000183172
ENSG00000183186
ENSG00000183196
ENSG00000183207
ENSG00000183230
ENSG00000183246
ENSG00000183255
ENSG00000183273
ENSG00000183281
ENSG00000183283
ENSG00000183287
ENSG00000183305
ENSG00000183307
ENSG00000183309
ENSG00000183313
ENSG00000183317
ENSG00000183323
ENSG00000183337
ENSG00000183340
ENSG00000183347
ENSG00000183354
ENSG00000183379
ENSG00000183401
ENSG00000183421
ENSG00000183423
ENSG00000183431
ENSG00000183434

ENSG00000183454
ENSG00000183475
ENSG00000183476
ENSG00000183484
ENSG00000183486
ENSG00000183495
ENSG00000183496
ENSG00000183508
ENSG00000183513
ENSG00000183520
ENSG00000183527
ENSG00000183530
ENSG00000183549
ENSG00000183569
ENSG00000183570
ENSG00000183571
ENSG00000183576
ENSG00000183578
ENSG00000183579
ENSG00000183580
ENSG00000183597
ENSG00000183605
ENSG00000183615
ENSG00000183624
ENSG00000183625
ENSG00000183628
ENSG00000183632
ENSG00000183640
ENSG00000183647
ENSG00000183655
ENSG00000183662
ENSG00000183665
ENSG00000183668
ENSG00000183671
ENSG00000183682
ENSG00000183684
ENSG00000183688
ENSG00000183690

ENSG00000183695
ENSG00000183706
ENSG00000183715
ENSG00000183718
ENSG00000183722
ENSG00000183723
ENSG00000183726
ENSG00000183734
ENSG00000183735
ENSG00000183741
ENSG00000183742
ENSG00000183747
ENSG00000183760
ENSG00000183762
ENSG00000183763
ENSG00000183765
ENSG00000183770
ENSG00000183773
ENSG00000183775
ENSG00000183778
ENSG00000183779
ENSG00000183780
ENSG00000183784
ENSG00000183785
ENSG00000183798
ENSG00000183801
ENSG00000183808
ENSG00000183814
ENSG00000183826
ENSG00000183831
ENSG00000183833
ENSG00000183837
ENSG00000183840
ENSG00000183844
ENSG00000183853
ENSG00000183856
ENSG00000183862
ENSG00000183864

ENSG00000183873
ENSG00000183876
ENSG00000183878
ENSG00000183908
ENSG00000183918
ENSG00000183943
ENSG00000183955
ENSG00000183960
ENSG00000183963
ENSG00000183978
ENSG00000184005
ENSG00000184007
ENSG00000184009
ENSG00000184012
ENSG00000184014
ENSG00000184047
ENSG00000184056
ENSG00000184058
ENSG00000184060
ENSG00000184076
ENSG00000184083
ENSG00000184113
ENSG00000184117
ENSG00000184144
ENSG00000184148
ENSG00000184154
ENSG00000184156
ENSG00000184164
ENSG00000184178
ENSG00000184182
ENSG00000184185
ENSG00000184194
ENSG00000184203
ENSG00000184205
ENSG00000184206
ENSG00000184207
ENSG00000184208
ENSG00000184210

ENSG00000184216
ENSG00000184221
ENSG00000184226
ENSG00000184232
ENSG00000184254
ENSG00000184271
ENSG00000184277
ENSG00000184281
ENSG00000184292
ENSG00000184293
ENSG00000184305
ENSG00000184307
ENSG00000184330
ENSG00000184343
ENSG00000184347
ENSG00000184349
ENSG00000184363
ENSG00000184368
ENSG00000184371
ENSG00000184374
ENSG00000184378
ENSG00000184384
ENSG00000184388
ENSG00000184402
ENSG00000184408
ENSG00000184432
ENSG00000184434
ENSG00000184451
ENSG00000184454
ENSG00000184459
ENSG00000184465
ENSG00000184470
ENSG00000184471
ENSG00000184481
ENSG00000184486
ENSG00000184489
ENSG00000184500
ENSG00000184507

ENSG00000184508
ENSG00000184517
ENSG00000184524
ENSG00000184545
ENSG00000184557
ENSG00000184564
ENSG00000184571
ENSG00000184574
ENSG00000184584
ENSG00000184588
ENSG00000184601
ENSG00000184602
ENSG00000184613
ENSG00000184635
ENSG00000184640
ENSG00000184650
ENSG00000184675
ENSG00000184677
ENSG00000184678
ENSG00000184697
ENSG00000184702
ENSG00000184708
ENSG00000184716
ENSG00000184719
ENSG00000184731
ENSG00000184743
ENSG00000184785
ENSG00000184787
ENSG00000184788
ENSG00000184792
ENSG00000184811
ENSG00000184828
ENSG00000184840
ENSG00000184845
ENSG00000184857
ENSG00000184860
ENSG00000184863
ENSG00000184887

ENSG00000184898
ENSG00000184900
ENSG00000184911
ENSG00000184916
ENSG00000184923
ENSG00000184924
ENSG00000184937
ENSG00000184939
ENSG00000184949
ENSG00000184956
ENSG00000184979
ENSG00000184983
ENSG00000184984
ENSG00000184985
ENSG00000184986
ENSG00000184988
ENSG00000184990
ENSG00000185000
ENSG00000185002
ENSG00000185008
ENSG00000185009
ENSG00000185010
ENSG00000185013
ENSG00000185015
ENSG00000185019
ENSG00000185022
ENSG00000185024
ENSG00000185033
ENSG00000185040
ENSG00000185046
ENSG00000185049
ENSG00000185052
ENSG00000185053
ENSG00000185056
ENSG00000185069
ENSG00000185070
ENSG00000185085
ENSG00000185087

ENSG00000185088
ENSG00000185090
ENSG00000185100
ENSG00000185101
ENSG00000185104
ENSG00000185105
ENSG00000185112
ENSG00000185122
ENSG00000185127
ENSG00000185129
ENSG00000185149
ENSG00000185155
ENSG00000185163
ENSG00000185164
ENSG00000185189
ENSG00000185201
ENSG00000185215
ENSG00000185219
ENSG00000185222
ENSG00000185231
ENSG00000185236
ENSG00000185238
ENSG00000185245
ENSG00000185247
ENSG00000185250
ENSG00000185252
ENSG00000185262
ENSG00000185267
ENSG00000185269
ENSG00000185274
ENSG00000185278
ENSG00000185298
ENSG00000185303
ENSG00000185305
ENSG00000185332
ENSG00000185338
ENSG00000185339
ENSG00000185340

ENSG00000185344
ENSG00000185345
ENSG00000185347
ENSG00000185352
ENSG00000185359
ENSG00000185361
ENSG00000185379
ENSG00000185386
ENSG00000185420
ENSG00000185432
ENSG00000185436
ENSG00000185437
ENSG00000185442
ENSG00000185453
ENSG00000185475
ENSG00000185477
ENSG00000185480
ENSG00000185483
ENSG00000185499
ENSG00000185504
ENSG00000185515
ENSG00000185518
ENSG00000185519
ENSG00000185527
ENSG00000185532
ENSG00000185551
ENSG00000185559
ENSG00000185561
ENSG00000185565
ENSG00000185585
ENSG00000185591
ENSG00000185594
ENSG00000185610
ENSG00000185614
ENSG00000185619
ENSG00000185621
ENSG00000185624
ENSG00000185627

ENSG00000185630
ENSG00000185633
ENSG00000185634
ENSG00000185640
ENSG00000185650
ENSG00000185651
ENSG00000185652
ENSG00000185658
ENSG00000185666
ENSG00000185668
ENSG00000185669
ENSG00000185670
ENSG00000185686
ENSG00000185689
ENSG00000185697
ENSG00000185716
ENSG00000185722
ENSG00000185728
ENSG00000185730
ENSG00000185736
ENSG00000185737
ENSG00000185739
ENSG00000185742
ENSG00000185745
ENSG00000185753
ENSG00000185760
ENSG00000185761
ENSG00000185774
ENSG00000185787
ENSG00000185792
ENSG00000185800
ENSG00000185811
ENSG00000185813
ENSG00000185818
ENSG00000185823
ENSG00000185825
ENSG00000185829
ENSG00000185860

ENSG00000185869
ENSG00000185873
ENSG00000185875
ENSG00000185883
ENSG00000185888
ENSG00000185896
ENSG00000185897
ENSG00000185900
ENSG00000185905
ENSG00000185909
ENSG00000185915
ENSG00000185917
ENSG00000185920
ENSG00000185924
ENSG00000185933
ENSG00000185946
ENSG00000185950
ENSG00000185958
ENSG00000185960
ENSG00000185963
ENSG00000185966
ENSG00000185973
ENSG00000185985
ENSG00000185988
ENSG00000185989
ENSG00000186001
ENSG00000186007
ENSG00000186009
ENSG00000186017
ENSG00000186020
ENSG00000186038
ENSG00000186047
ENSG00000186049
ENSG00000186063
ENSG00000186073
ENSG00000186074
ENSG00000186075
ENSG00000186081

ENSG00000186088
ENSG00000186094
ENSG00000186103
ENSG00000186106
ENSG00000186111
ENSG00000186115
ENSG00000186130
ENSG00000186132
ENSG00000186141
ENSG00000186143
ENSG00000186150
ENSG00000186153
ENSG00000186174
ENSG00000186184
ENSG00000186185
ENSG00000186187
ENSG00000186188
ENSG00000186193
ENSG00000186197
ENSG00000186198
ENSG00000186205
ENSG00000186207
ENSG00000186212
ENSG00000186230
ENSG00000186231
ENSG00000186260
ENSG00000186265
ENSG00000186280
ENSG00000186283
ENSG00000186288
ENSG00000186297
ENSG00000186298
ENSG00000186300
ENSG00000186314
ENSG00000186318
ENSG00000186326
ENSG00000186334
ENSG00000186335

ENSG00000186340
ENSG00000186350
ENSG00000186352
ENSG00000186354
ENSG00000186376
ENSG00000186399
ENSG00000186407
ENSG00000186409
ENSG00000186416
ENSG00000186417
ENSG00000186431
ENSG00000186432
ENSG00000186439
ENSG00000186442
ENSG00000186446
ENSG00000186448
ENSG00000186451
ENSG00000186458
ENSG00000186468
ENSG00000186469
ENSG00000186470
ENSG00000186472
ENSG00000186474
ENSG00000186479
ENSG00000186480
ENSG00000186493
ENSG00000186496
ENSG00000186501
ENSG00000186509
ENSG00000186513
ENSG00000186517
ENSG00000186522
ENSG00000186523
ENSG00000186526
ENSG00000186529
ENSG00000186532
ENSG00000186564
ENSG00000186566

ENSG00000186567
ENSG00000186575
ENSG00000186577
ENSG00000186579
ENSG00000186591
ENSG00000186603
ENSG00000186625
ENSG00000186628
ENSG00000186635
ENSG00000186638
ENSG00000186642
ENSG00000186648
ENSG00000186652
ENSG00000186654
ENSG00000186660
ENSG00000186665
ENSG00000186666
ENSG00000186675
ENSG00000186684
ENSG00000186687
ENSG00000186710
ENSG00000186716
ENSG00000186732
ENSG00000186766
ENSG00000186767
ENSG00000186788
ENSG00000186790
ENSG00000186792
ENSG00000186806
ENSG00000186810
ENSG00000186812
ENSG00000186814
ENSG00000186815
ENSG00000186818
ENSG00000186834
ENSG00000186838
ENSG00000186854
ENSG00000186860

ENSG00000186862
ENSG00000186866
ENSG00000186867
ENSG00000186868
ENSG00000186871
ENSG00000186889
ENSG00000186895
ENSG00000186897
ENSG00000186907
ENSG00000186908
ENSG00000186912
ENSG00000186918
ENSG00000186925
ENSG00000186951
ENSG00000186952
ENSG00000186998
ENSG00000187010
ENSG00000187024
ENSG00000187033
ENSG00000187037
ENSG00000187045
ENSG00000187048
ENSG00000187049
ENSG00000187051
ENSG00000187054
ENSG00000187066
ENSG00000187068
ENSG00000187079
ENSG00000187082
ENSG00000187094
ENSG00000187097
ENSG00000187098
ENSG00000187105
ENSG00000187109
ENSG00000187116
ENSG00000187122
ENSG00000187123
ENSG00000187134

ENSG00000187140
ENSG00000187144
ENSG00000187147
ENSG00000187151
ENSG00000187164
ENSG00000187186
ENSG00000187187
ENSG00000187189
ENSG00000187191
ENSG00000187210
ENSG00000187231
ENSG00000187239
ENSG00000187243
ENSG00000187244
ENSG00000187258
ENSG00000187260
ENSG00000187266
ENSG00000187268
ENSG00000187272
ENSG00000187288
ENSG00000187323
ENSG00000187325
ENSG00000187398
ENSG00000187416
ENSG00000187446
ENSG00000187474
ENSG00000187479
ENSG00000187486
ENSG00000187498
ENSG00000187513
ENSG00000187522
ENSG00000187531
ENSG00000187533
ENSG00000187535
ENSG00000187546
ENSG00000187550
ENSG00000187554
ENSG00000187555

ENSG00000187566
ENSG00000187601
ENSG00000187605
ENSG00000187607
ENSG00000187624
ENSG00000187626
ENSG00000187627
ENSG00000187630
ENSG00000187642
ENSG00000187650
ENSG00000187658
ENSG00000187664
ENSG00000187672
ENSG00000187676
ENSG00000187678
ENSG00000187690
ENSG00000187695
ENSG00000187713
ENSG00000187714
ENSG00000187715
ENSG00000187720
ENSG00000187736
ENSG00000187741
ENSG00000187742
ENSG00000187753
ENSG00000187764
ENSG00000187772
ENSG00000187773
ENSG00000187778
ENSG00000187783
ENSG00000187791
ENSG00000187792
ENSG00000187800
ENSG00000187801
ENSG00000187806
ENSG00000187808
ENSG00000187823
ENSG00000187824

ENSG00000187838
ENSG00000187840
ENSG00000187848
ENSG00000187855
ENSG00000187860
ENSG00000187866
ENSG00000187871
ENSG00000187902
ENSG00000187905
ENSG00000187912
ENSG00000187922
ENSG00000187942
ENSG00000187954
ENSG00000187955
ENSG00000187957
ENSG00000187961
ENSG00000187987
ENSG00000187994
ENSG00000188001
ENSG00000188015
ENSG00000188021
ENSG00000188026
ENSG00000188033
ENSG00000188039
ENSG00000188042
ENSG00000188051
ENSG00000188056
ENSG00000188060
ENSG00000188064
ENSG00000188070
ENSG00000188086
ENSG00000188089
ENSG00000188092
ENSG00000188095
ENSG00000188107
ENSG00000188112
ENSG00000188120
ENSG00000188133

ENSG00000188152
ENSG00000188153
ENSG00000188155
ENSG00000188158
ENSG00000188167
ENSG00000188171
ENSG00000188175
ENSG00000188176
ENSG00000188177
ENSG00000188186
ENSG00000188191
ENSG00000188199
ENSG00000188211
ENSG00000188215
ENSG00000188227
ENSG00000188229
ENSG00000188243
ENSG00000188257
ENSG00000188263
ENSG00000188266
ENSG00000188269
ENSG00000188277
ENSG00000188283
ENSG00000188293
ENSG00000188305
ENSG00000188312
ENSG00000188313
ENSG00000188315
ENSG00000188316
ENSG00000188321
ENSG00000188322
ENSG00000188338
ENSG00000188342
ENSG00000188352
ENSG00000188373
ENSG00000188375
ENSG00000188385
ENSG00000188386

ENSG00000188393
ENSG00000188396
ENSG00000188404
ENSG00000188419
ENSG00000188425
ENSG00000188428
ENSG00000188452
ENSG00000188483
ENSG00000188486
ENSG00000188487
ENSG00000188488
ENSG00000188493
ENSG00000188501
ENSG00000188505
ENSG00000188508
ENSG00000188517
ENSG00000188523
ENSG00000188529
ENSG00000188542
ENSG00000188549
ENSG00000188554
ENSG00000188559
ENSG00000188566
ENSG00000188580
ENSG00000188582
ENSG00000188603
ENSG00000188610
ENSG00000188611
ENSG00000188613
ENSG00000188624
ENSG00000188626
ENSG00000188629
ENSG00000188636
ENSG00000188641
ENSG00000188643
ENSG00000188647
ENSG00000188649
ENSG00000188659

ENSG00000188674
ENSG00000188690
ENSG00000188694
ENSG00000188706
ENSG00000188707
ENSG00000188730
ENSG00000188735
ENSG00000188739
ENSG00000188760
ENSG00000188761
ENSG00000188763
ENSG00000188766
ENSG00000188770
ENSG00000188771
ENSG00000188778
ENSG00000188779
ENSG00000188783
ENSG00000188785
ENSG00000188786
ENSG00000188800
ENSG00000188803
ENSG00000188807
ENSG00000188811
ENSG00000188816
ENSG00000188817
ENSG00000188818
ENSG00000188822
ENSG00000188833
ENSG00000188846
ENSG00000188848
ENSG00000188859
ENSG00000188868
ENSG00000188883
ENSG00000188895
ENSG00000188897
ENSG00000188910
ENSG00000188916
ENSG00000188917

ENSG00000188921
ENSG00000188931
ENSG00000188937
ENSG00000188938
ENSG00000188958
ENSG00000188959
ENSG00000188976
ENSG00000188981
ENSG00000188984
ENSG00000188991
ENSG00000188993
ENSG00000188997
ENSG00000189001
ENSG00000189007
ENSG00000189013
ENSG00000189037
ENSG00000189042
ENSG00000189043
ENSG00000189050
ENSG00000189051
ENSG00000189057
ENSG00000189060
ENSG00000189067
ENSG00000189077
ENSG00000189079
ENSG00000189091
ENSG00000189099
ENSG00000189114
ENSG00000189120
ENSG00000189127
ENSG00000189139
ENSG00000189143
ENSG00000189152
ENSG00000189159
ENSG00000189164
ENSG00000189180
ENSG00000189182
ENSG00000189184

ENSG00000189221
ENSG00000189227
ENSG00000189233
ENSG00000189241
ENSG00000189266
ENSG00000189269
ENSG00000189280
ENSG00000189283
ENSG00000189292
ENSG00000189298
ENSG00000189299
ENSG00000189306
ENSG00000189308
ENSG00000189319
ENSG00000189320
ENSG00000189325
ENSG00000189334
ENSG00000189337
ENSG00000189339
ENSG00000189357
ENSG00000189367
ENSG00000189375
ENSG00000189377
ENSG00000189401
ENSG00000189403
ENSG00000189410
ENSG00000189430
ENSG00000189431
ENSG00000189433
ENSG00000196072
ENSG00000196074
ENSG00000196083
ENSG00000196090
ENSG00000196092
ENSG00000196110
ENSG00000196118
ENSG00000196123
ENSG00000196126

ENSG00000196132
ENSG00000196141
ENSG00000196150
ENSG00000196155
ENSG00000196156
ENSG00000196159
ENSG00000196166
ENSG00000196169
ENSG00000196172
ENSG00000196177
ENSG00000196182
ENSG00000196187
ENSG00000196189
ENSG00000196196
ENSG00000196199
ENSG00000196208
ENSG00000196209
ENSG00000196214
ENSG00000196220
ENSG00000196227
ENSG00000196228
ENSG00000196230
ENSG00000196233
ENSG00000196236
ENSG00000196242
ENSG00000196247
ENSG00000196262
ENSG00000196263
ENSG00000196268
ENSG00000196277
ENSG00000196284
ENSG00000196290
ENSG00000196296
ENSG00000196313
ENSG00000196323
ENSG00000196329
ENSG00000196338
ENSG00000196344

ENSG00000196345
ENSG00000196353
ENSG00000196358
ENSG00000196361
ENSG00000196363
ENSG00000196367
ENSG00000196368
ENSG00000196369
ENSG00000196371
ENSG00000196372
ENSG00000196376
ENSG00000196378
ENSG00000196381
ENSG00000196387
ENSG00000196388
ENSG00000196396
ENSG00000196405
ENSG00000196408
ENSG00000196411
ENSG00000196415
ENSG00000196417
ENSG00000196418
ENSG00000196431
ENSG00000196437
ENSG00000196440
ENSG00000196449
ENSG00000196453
ENSG00000196456
ENSG00000196458
ENSG00000196459
ENSG00000196470
ENSG00000196476
ENSG00000196482
ENSG00000196498
ENSG00000196502
ENSG00000196504
ENSG00000196505
ENSG00000196510

ENSG00000196511
ENSG00000196517
ENSG00000196526
ENSG00000196535
ENSG00000196542
ENSG00000196544
ENSG00000196547
ENSG00000196549
ENSG00000196550
ENSG00000196557
ENSG00000196576
ENSG00000196581
ENSG00000196586
ENSG00000196588
ENSG00000196591
ENSG00000196597
ENSG00000196604
ENSG00000196611
ENSG00000196616
ENSG00000196628
ENSG00000196632
ENSG00000196639
ENSG00000196642
ENSG00000196646
ENSG00000196652
ENSG00000196653
ENSG00000196660
ENSG00000196663
ENSG00000196664
ENSG00000196666
ENSG00000196670
ENSG00000196678
ENSG00000196683
ENSG00000196684
ENSG00000196689
ENSG00000196693
ENSG00000196700
ENSG00000196704

ENSG00000196705
ENSG00000196712
ENSG00000196715
ENSG00000196724
ENSG00000196730
ENSG00000196739
ENSG00000196743
ENSG00000196747
ENSG00000196748
ENSG00000196754
ENSG00000196757
ENSG00000196776
ENSG00000196781
ENSG00000196782
ENSG00000196792
ENSG00000196793
ENSG00000196800
ENSG00000196811
ENSG00000196814
ENSG00000196821
ENSG00000196844
ENSG00000196850
ENSG00000196859
ENSG00000196862
ENSG00000196865
ENSG00000196866
ENSG00000196867
ENSG00000196872
ENSG00000196876
ENSG00000196878
ENSG00000196911
ENSG00000196912
ENSG00000196914
ENSG00000196917
ENSG00000196924
ENSG00000196932
ENSG00000196935
ENSG00000196937

ENSG00000196943
ENSG00000196946
ENSG00000196967
ENSG00000196968
ENSG00000196975
ENSG00000196976
ENSG00000196981
ENSG00000196990
ENSG00000196993
ENSG00000196998
ENSG00000197006
ENSG00000197013
ENSG00000197016
ENSG00000197019
ENSG00000197021
ENSG00000197024
ENSG00000197037
ENSG00000197043
ENSG00000197044
ENSG00000197045
ENSG00000197046
ENSG00000197050
ENSG00000197054
ENSG00000197056
ENSG00000197057
ENSG00000197062
ENSG00000197063
ENSG00000197070
ENSG00000197077
ENSG00000197079
ENSG00000197081
ENSG00000197093
ENSG00000197106
ENSG00000197111
ENSG00000197119
ENSG00000197122
ENSG00000197128
ENSG00000197134

ENSG00000197142
ENSG00000197147
ENSG00000197150
ENSG00000197157
ENSG00000197162
ENSG00000197165
ENSG00000197168
ENSG00000197170
ENSG00000197172
ENSG00000197177
ENSG00000197181
ENSG00000197183
ENSG00000197217
ENSG00000197226
ENSG00000197245
ENSG00000197249
ENSG00000197256
ENSG00000197261
ENSG00000197265
ENSG00000197272
ENSG00000197275
ENSG00000197283
ENSG00000197302
ENSG00000197312
ENSG00000197323
ENSG00000197324
ENSG00000197343
ENSG00000197353
ENSG00000197355
ENSG00000197361
ENSG00000197362
ENSG00000197363
ENSG00000197364
ENSG00000197372
ENSG00000197375
ENSG00000197380
ENSG00000197381
ENSG00000197386

ENSG00000197405
ENSG00000197406
ENSG00000197408
ENSG00000197409
ENSG00000197415
ENSG00000197417
ENSG00000197429
ENSG00000197430
ENSG00000197444
ENSG00000197446
ENSG00000197448
ENSG00000197457
ENSG00000197461
ENSG00000197465
ENSG00000197467
ENSG00000197471
ENSG00000197472
ENSG00000197479
ENSG00000197487
ENSG00000197496
ENSG00000197498
ENSG00000197506
ENSG00000197520
ENSG00000197535
ENSG00000197540
ENSG00000197548
ENSG00000197555
ENSG00000197557
ENSG00000197562
ENSG00000197563
ENSG00000197565
ENSG00000197566
ENSG00000197576
ENSG00000197579
ENSG00000197580
ENSG00000197586
ENSG00000197587
ENSG00000197594

ENSG00000197601
ENSG00000197603
ENSG00000197608
ENSG00000197614
ENSG00000197620
ENSG00000197622
ENSG00000197629
ENSG00000197635
ENSG00000197641
ENSG00000197646
ENSG00000197647
ENSG00000197651
ENSG00000197658
ENSG00000197683
ENSG00000197696
ENSG00000197702
ENSG00000197705
ENSG00000197712
ENSG00000197713
ENSG00000197714
ENSG00000197724
ENSG00000197728
ENSG00000197745
ENSG00000197746
ENSG00000197747
ENSG00000197753
ENSG00000197763
ENSG00000197766
ENSG00000197769
ENSG00000197771
ENSG00000197776
ENSG00000197779
ENSG00000197780
ENSG00000197782
ENSG00000197808
ENSG00000197816
ENSG00000197818
ENSG00000197822

ENSG00000197826
ENSG00000197838
ENSG00000197841
ENSG00000197852
ENSG00000197858
ENSG00000197859
ENSG00000197860
ENSG00000197872
ENSG00000197879
ENSG00000197885
ENSG00000197891
ENSG00000197892
ENSG00000197893
ENSG00000197894
ENSG00000197901
ENSG00000197912
ENSG00000197915
ENSG00000197921
ENSG00000197928
ENSG00000197930
ENSG00000197933
ENSG00000197937
ENSG00000197943
ENSG00000197948
ENSG00000197951
ENSG00000197956
ENSG00000197959
ENSG00000197965
ENSG00000197969
ENSG00000197971
ENSG00000197976
ENSG00000197977
ENSG00000197978
ENSG00000197980
ENSG00000197982
ENSG00000197991
ENSG00000198000
ENSG00000198001

ENSG00000198015
ENSG00000198018
ENSG00000198019
ENSG00000198028
ENSG00000198039
ENSG00000198040
ENSG00000198042
ENSG00000198046
ENSG00000198049
ENSG00000198053
ENSG00000198055
ENSG00000198060
ENSG00000198074
ENSG00000198077
ENSG00000198081
ENSG00000198083
ENSG00000198087
ENSG00000198088
ENSG00000198090
ENSG00000198092
ENSG00000198093
ENSG00000198099
ENSG00000198105
ENSG00000198108
ENSG00000198113
ENSG00000198121
ENSG00000198125
ENSG00000198131
ENSG00000198133
ENSG00000198142
ENSG00000198146
ENSG00000198157
ENSG00000198160
ENSG00000198162
ENSG00000198168
ENSG00000198169
ENSG00000198171
ENSG00000198178

ENSG00000198182
ENSG00000198183
ENSG00000198185
ENSG00000198189
ENSG00000198198
ENSG00000198203
ENSG00000198205
ENSG00000198208
ENSG00000198216
ENSG00000198218
ENSG00000198223
ENSG00000198231
ENSG00000198242
ENSG00000198246
ENSG00000198252
ENSG00000198265
ENSG00000198270
ENSG00000198298
ENSG00000198301
ENSG00000198315
ENSG00000198324
ENSG00000198326
ENSG00000198331
ENSG00000198336
ENSG00000198346
ENSG00000198353
ENSG00000198354
ENSG00000198355
ENSG00000198356
ENSG00000198363
ENSG00000198369
ENSG00000198373
ENSG00000198380
ENSG00000198382
ENSG00000198390
ENSG00000198393
ENSG00000198398
ENSG00000198399

ENSG00000198408
ENSG00000198420
ENSG00000198429
ENSG00000198431
ENSG00000198435
ENSG00000198453
ENSG00000198455
ENSG00000198466
ENSG00000198467
ENSG00000198478
ENSG00000198482
ENSG00000198483
ENSG00000198488
ENSG00000198492
ENSG00000198498
ENSG00000198502
ENSG00000198513
ENSG00000198515
ENSG00000198517
ENSG00000198520
ENSG00000198521
ENSG00000198522
ENSG00000198523
ENSG00000198535
ENSG00000198542
ENSG00000198551
ENSG00000198553
ENSG00000198554
ENSG00000198561
ENSG00000198570
ENSG00000198574
ENSG00000198576
ENSG00000198585
ENSG00000198586
ENSG00000198589
ENSG00000198590
ENSG00000198598
ENSG00000198610

ENSG00000198612
ENSG00000198624
ENSG00000198625
ENSG00000198626
ENSG00000198633
ENSG00000198642
ENSG00000198643
ENSG00000198648
ENSG00000198650
ENSG00000198663
ENSG00000198668
ENSG00000198673
ENSG00000198680
ENSG00000198681
ENSG00000198682
ENSG00000198689
ENSG00000198690
ENSG00000198700
ENSG00000198704
ENSG00000198715
ENSG00000198720
ENSG00000198721
ENSG00000198722
ENSG00000198728
ENSG00000198729
ENSG00000198730
ENSG00000198732
ENSG00000198734
ENSG00000198736
ENSG00000198739
ENSG00000198740
ENSG00000198742
ENSG00000198743
ENSG00000198746
ENSG00000198754
ENSG00000198756
ENSG00000198759
ENSG00000198765

ENSG00000198768
ENSG00000198771
ENSG00000198780
ENSG00000198783
ENSG00000198785
ENSG00000198791
ENSG00000198792
ENSG00000198793
ENSG00000198794
ENSG00000198795
ENSG00000198796
ENSG00000198797
ENSG00000198798
ENSG00000198799
ENSG00000198805
ENSG00000198807
ENSG00000198812
ENSG00000198814
ENSG00000198815
ENSG00000198821
ENSG00000198824
ENSG00000198825
ENSG00000198829
ENSG00000198830
ENSG00000198833
ENSG00000198836
ENSG00000198837
ENSG00000198839
ENSG00000198841
ENSG00000198842
ENSG00000198843
ENSG00000198844
ENSG00000198846
ENSG00000198851
ENSG00000198853
ENSG00000198854
ENSG00000198858
ENSG00000198862

ENSG00000198863
ENSG00000198865
ENSG00000198870
ENSG00000198876
ENSG00000198879
ENSG00000198883
ENSG00000198887
ENSG00000198889
ENSG00000198890
ENSG00000198892
ENSG00000198894
ENSG00000198898
ENSG00000198900
ENSG00000198901
ENSG00000198908
ENSG00000198909
ENSG00000198910
ENSG00000198911
ENSG00000198912
ENSG00000198914
ENSG00000198915
ENSG00000198917
ENSG00000198918
ENSG00000198920
ENSG00000198924
ENSG00000198925
ENSG00000198929
ENSG00000198930
ENSG00000198931
ENSG00000198933
ENSG00000198937
ENSG00000198939
ENSG00000198944
ENSG00000198945
ENSG00000198947
ENSG00000198948
ENSG00000198951
ENSG00000198952

ENSG00000198954
ENSG00000198959
ENSG00000198960
ENSG00000198961
ENSG00000198963
ENSG00000198964
ENSG00000203485
ENSG00000203618
ENSG00000203666
ENSG00000203667
ENSG00000203668
ENSG00000203685
ENSG00000203697
ENSG00000203705
ENSG00000203710
ENSG00000203727
ENSG00000203730
ENSG00000203734
ENSG00000203737
ENSG00000203747
ENSG00000203760
ENSG00000203772
ENSG00000203778
ENSG00000203783
ENSG00000203784
ENSG00000203785
ENSG00000203786
ENSG00000203791
ENSG00000203797
ENSG00000203805
ENSG00000203812
ENSG00000203837
ENSG00000203867
ENSG00000203870
ENSG00000203879
ENSG00000203880
ENSG00000203896
ENSG00000203923

ENSG00000203933
ENSG00000203942
ENSG00000203943
ENSG00000203950
ENSG00000203963
ENSG00000203970
ENSG00000203972
ENSG00000203985
ENSG00000203995
ENSG00000204001
ENSG00000204006
ENSG00000204010
ENSG00000204025
ENSG00000204033
ENSG00000204052
ENSG00000204065
ENSG00000204070
ENSG00000204084
ENSG00000204099
ENSG00000204103
ENSG00000204104
ENSG00000204116
ENSG00000204120
ENSG00000204128
ENSG00000204130
ENSG00000204131
ENSG00000204138
ENSG00000204147
ENSG00000204152
ENSG00000204160
ENSG00000204161
ENSG00000204174
ENSG00000204175
ENSG00000204176
ENSG00000204178
ENSG00000204179
ENSG00000204186
ENSG00000204193

ENSG00000204195
ENSG00000204209
ENSG00000204217
ENSG00000204219
ENSG00000204227
ENSG00000204231
ENSG00000204237
ENSG00000204248
ENSG00000204252
ENSG00000204256
ENSG00000204262
ENSG00000204267
ENSG00000204271
ENSG00000204278
ENSG00000204287
ENSG00000204296
ENSG00000204301
ENSG00000204304
ENSG00000204305
ENSG00000204308
ENSG00000204310
ENSG00000204314
ENSG00000204323
ENSG00000204335
ENSG00000204344
ENSG00000204347
ENSG00000204348
ENSG00000204356
ENSG00000204366
ENSG00000204370
ENSG00000204371
ENSG00000204381
ENSG00000204386
ENSG00000204392
ENSG00000204397
ENSG00000204406
ENSG00000204414
ENSG00000204420

ENSG00000204421
ENSG00000204428
ENSG00000204438
ENSG00000204439
ENSG00000204442
ENSG00000204475
ENSG00000204480
ENSG00000204481
ENSG00000204482
ENSG00000204498
ENSG00000204511
ENSG00000204514
ENSG00000204516
ENSG00000204519
ENSG00000204520
ENSG00000204524
ENSG00000204531
ENSG00000204536
ENSG00000204539
ENSG00000204540
ENSG00000204544
ENSG00000204564
ENSG00000204568
ENSG00000204569
ENSG00000204571
ENSG00000204572
ENSG00000204574
ENSG00000204576
ENSG00000204577
ENSG00000204580
ENSG00000204590
ENSG00000204592
ENSG00000204599
ENSG00000204604
ENSG00000204611
ENSG00000204613
ENSG00000204614
ENSG00000204616

ENSG00000204618
ENSG00000204619
ENSG00000204642
ENSG00000204644
ENSG00000204655
ENSG00000204661
ENSG00000204669
ENSG00000204671
ENSG00000204673
ENSG00000204681
ENSG00000204682
ENSG00000204683
ENSG00000204688
ENSG00000204694
ENSG00000204713
ENSG00000204764
ENSG00000204767
ENSG00000204822
ENSG00000204842
ENSG00000204843
ENSG00000204851
ENSG00000204869
ENSG00000204889
ENSG00000204899
ENSG00000204909
ENSG00000204918
ENSG00000204919
ENSG00000204920
ENSG00000204922
ENSG00000204923
ENSG00000204930
ENSG00000204936
ENSG00000204946
ENSG00000204947
ENSG00000204950
ENSG00000204952
ENSG00000204954
ENSG00000204956

ENSG00000204961
ENSG00000204962
ENSG00000204963
ENSG00000204965
ENSG00000204967
ENSG00000204969
ENSG00000204970
ENSG00000204977
ENSG00000204978
ENSG00000204991
ENSG00000205002
ENSG00000205022
ENSG00000205038
ENSG00000205057
ENSG00000205060
ENSG00000205084
ENSG00000205085
ENSG00000205086
ENSG00000205089
ENSG00000205090
ENSG00000205097
ENSG00000205111
ENSG00000205133
ENSG00000205138
ENSG00000205155
ENSG00000205177
ENSG00000205189
ENSG00000205208
ENSG00000205209
ENSG00000205212
ENSG00000205213
ENSG00000205221
ENSG00000205238
ENSG00000205250
ENSG00000205268
ENSG00000205269
ENSG00000205277
ENSG00000205279

ENSG00000205302
ENSG00000205309
ENSG00000205323
ENSG00000205336
ENSG00000205339
ENSG00000205352
ENSG00000205356
ENSG00000205363
ENSG00000205403
ENSG00000205413
ENSG00000205420
ENSG00000205423
ENSG00000205436
ENSG00000205456
ENSG00000205457
ENSG00000205476
ENSG00000205502
ENSG00000205531
ENSG00000205560
ENSG00000205571
ENSG00000205572
ENSG00000205581
ENSG00000205639
ENSG00000205649
ENSG00000205659
ENSG00000205678
ENSG00000205683
ENSG00000205710
ENSG00000205726
ENSG00000205730
ENSG00000205765
ENSG00000205777
ENSG00000205784
ENSG00000205795
ENSG00000205808
ENSG00000205810
ENSG00000205835
ENSG00000205838

ENSG00000205857
ENSG00000205867
ENSG00000205903
ENSG00000205916
ENSG00000205923
ENSG00000205927
ENSG00000205929
ENSG00000205937
ENSG00000205944
ENSG00000205978
ENSG00000205981
ENSG00000206013
ENSG00000206026
ENSG00000206043
ENSG00000206052
ENSG00000206053
ENSG00000206072
ENSG00000206075
ENSG00000206102
ENSG00000206127
ENSG00000206140
ENSG00000206150
ENSG00000206172
ENSG00000206190
ENSG00000206199
ENSG00000206260
ENSG00000206262
ENSG00000206384
ENSG00000206418
ENSG00000206432
ENSG00000206503
ENSG00000206527
ENSG00000206530
ENSG00000206531
ENSG00000206538
ENSG00000206549
ENSG00000206557
ENSG00000206559

ENSG00000206560
ENSG00000206561
ENSG00000206562
ENSG00000206579
ENSG00000211445
ENSG00000211448
ENSG00000211450
ENSG00000211452
ENSG00000211455
ENSG00000211456
ENSG00000211460
ENSG00000211584
ENSG00000212124
ENSG00000212128
ENSG00000212710
ENSG00000212719
ENSG00000212722
ENSG00000212724
ENSG00000212747
ENSG00000212899
ENSG00000212916
ENSG00000212993
ENSG00000213015
ENSG00000213020
ENSG00000213022
ENSG00000213024
ENSG00000213047
ENSG00000213064
ENSG00000213066
ENSG00000213096
ENSG00000213160
ENSG00000213171
ENSG00000213185
ENSG00000213186
ENSG00000213203
ENSG00000213213
ENSG00000213215
ENSG00000213218

ENSG00000213221
ENSG00000213231
ENSG00000213246
ENSG00000213281
ENSG00000213316
ENSG00000213339
ENSG00000213341
ENSG00000213347
ENSG00000213390
ENSG00000213397
ENSG00000213416
ENSG00000213420
ENSG00000213462
ENSG00000213463
ENSG00000213465
ENSG00000213516
ENSG00000213533
ENSG00000213551
ENSG00000213563
ENSG00000213578
ENSG00000213588
ENSG00000213593
ENSG00000213614
ENSG00000213625
ENSG00000213626
ENSG00000213638
ENSG00000213639
ENSG00000213648
ENSG00000213654
ENSG00000213658
ENSG00000213672
ENSG00000213676
ENSG00000213694
ENSG00000213699
ENSG00000213741
ENSG00000213760
ENSG00000213762
ENSG00000213780

ENSG00000213782
ENSG00000213799
ENSG00000213809
ENSG00000213853
ENSG00000213859
ENSG00000213865
ENSG00000213889
ENSG00000213892
ENSG00000213903
ENSG00000213906
ENSG00000213918
ENSG00000213923
ENSG00000213937
ENSG00000213965
ENSG00000213973
ENSG00000213977
ENSG00000213983
ENSG00000213988
ENSG00000213995
ENSG00000213996
ENSG00000214013
ENSG00000214021
ENSG00000214022
ENSG00000214026
ENSG00000214046
ENSG00000214063
ENSG00000214078
ENSG00000214097
ENSG00000214102
ENSG00000214113
ENSG00000214114
ENSG00000214128
ENSG00000214140
ENSG00000214212
ENSG00000214215
ENSG00000214216
ENSG00000214253
ENSG00000214300

ENSG00000214309
ENSG00000214338
ENSG00000214357
ENSG00000214413
ENSG00000214456
ENSG00000214517
ENSG00000214518
ENSG00000214575
ENSG00000214595
ENSG00000214655
ENSG00000214688
ENSG00000214694
ENSG00000214706
ENSG00000214711
ENSG00000214717
ENSG00000214753
ENSG00000214756
ENSG00000214819
ENSG00000214827
ENSG00000214860
ENSG00000214872
ENSG00000214929
ENSG00000214941
ENSG00000214944
ENSG00000214946
ENSG00000214960
ENSG00000215012
ENSG00000215021
ENSG00000215029
ENSG00000215041
ENSG00000215045
ENSG00000215114
ENSG00000215131
ENSG00000215174
ENSG00000215182
ENSG00000215183
ENSG00000215186
ENSG00000215187

ENSG00000215193
ENSG00000215217
ENSG00000215218
ENSG00000215252
ENSG00000215271
ENSG00000215277
ENSG00000215301
ENSG00000215343
ENSG00000215372
ENSG00000215397
ENSG00000215421
ENSG00000215440
ENSG00000215454
ENSG00000215472
ENSG00000215474
ENSG00000215475
ENSG00000215529
ENSG00000215568
ENSG00000215595
ENSG00000215612
ENSG00000215695
ENSG00000215712
ENSG00000215717
ENSG00000215784
ENSG00000215788
ENSG00000215790
ENSG00000215845
ENSG00000215853
ENSG00000215883
ENSG00000215915
ENSG00000216490
ENSG00000216588
ENSG00000217128
ENSG00000217555
ENSG00000218336
ENSG00000218739
ENSG00000218819
ENSG00000219200

ENSG00000219607
ENSG00000219626
ENSG00000220201
ENSG00000220205
ENSG00000221818
ENSG00000221821
ENSG00000221823
ENSG00000221829
ENSG00000221838
ENSG00000221843
ENSG00000221852
ENSG00000221859
ENSG00000221864
ENSG00000221866
ENSG00000221867
ENSG00000221874
ENSG00000221880
ENSG00000221890
ENSG00000221900
ENSG00000221909
ENSG00000221914
ENSG00000221916
ENSG00000221932
ENSG00000221946
ENSG00000221947
ENSG00000221955
ENSG00000221957
ENSG00000221963
ENSG00000221978
ENSG00000221983
ENSG00000221986
ENSG00000221988
ENSG00000222011
ENSG00000222014
ENSG00000222018
ENSG00000222028
ENSG00000222036
ENSG00000222046

ENSG00000222047
ENSG00000223496
ENSG00000223547
ENSG00000223658
ENSG00000223802
ENSG00000223865
ENSG00000223953
ENSG00000224051
ENSG00000224089
ENSG00000224383
ENSG00000224420
ENSG00000224470
ENSG00000224531
ENSG00000224586
ENSG00000224940
ENSG00000224982
ENSG00000225110
ENSG00000225190
ENSG00000225362
ENSG00000225526
ENSG00000225663
ENSG00000225830
ENSG00000225921
ENSG00000225968
ENSG00000225973
ENSG00000226023
ENSG00000226174
ENSG00000226321
ENSG00000226479
ENSG00000226600
ENSG00000226650
ENSG00000226685
ENSG00000226742
ENSG00000226763
ENSG00000226784
ENSG00000226807
ENSG00000226887
ENSG00000226929

ENSG00000227051
ENSG00000227057
ENSG00000227059
ENSG00000227124
ENSG00000227151
ENSG00000227268
ENSG00000227345
ENSG00000227471
ENSG00000227500
ENSG00000227507
ENSG00000228049
ENSG00000228075
ENSG00000228300
ENSG00000228408
ENSG00000228439
ENSG00000228517
ENSG00000228716
ENSG00000228727
ENSG00000228836
ENSG00000229117
ENSG00000229183
ENSG00000229571
ENSG00000229665
ENSG00000229676
ENSG00000229809
ENSG00000229859
ENSG00000230062
ENSG00000230347
ENSG00000230510
ENSG00000230594
ENSG00000230778
ENSG00000230797
ENSG00000230873
ENSG00000230989
ENSG00000231256
ENSG00000231274
ENSG00000231389
ENSG00000231824

ENSG00000231852
ENSG00000231924
ENSG00000231925
ENSG00000232070
ENSG00000232119
ENSG00000232196
ENSG00000232237
ENSG00000232258
ENSG00000232423
ENSG00000232653
ENSG00000232810
ENSG00000232838
ENSG00000232859
ENSG00000232995
ENSG00000233087
ENSG00000233198
ENSG00000233276
ENSG00000233436
ENSG00000233608
ENSG00000233670
ENSG00000233932
ENSG00000233954
ENSG00000234127
ENSG00000234186
ENSG00000234278
ENSG00000234409
ENSG00000234438
ENSG00000234444
ENSG00000234545
ENSG00000234602
ENSG00000234616
ENSG00000234829
ENSG00000234965
ENSG00000235034
ENSG00000235098
ENSG00000235109
ENSG00000235173
ENSG00000235194

ENSG00000235376
ENSG00000235568
ENSG00000235711
ENSG00000235718
ENSG00000235750
ENSG00000235961
ENSG00000236027
ENSG00000236104
ENSG00000236126
ENSG00000236334
ENSG00000236371
ENSG00000236446
ENSG00000236543
ENSG00000236609
ENSG00000236699
ENSG00000237172
ENSG00000237353
ENSG00000237440
ENSG00000237441
ENSG00000237515
ENSG00000237649
ENSG00000237765
ENSG00000237957
ENSG00000238205
ENSG00000238227
ENSG00000239264
ENSG00000239282
ENSG00000239306
ENSG00000239382
ENSG00000239388
ENSG00000239389
ENSG00000239474
ENSG00000239521
ENSG00000239605
ENSG00000239642
ENSG00000239672
ENSG00000239697
ENSG00000239704

ENSG00000239789
ENSG00000239810
ENSG00000239857
ENSG00000239886
ENSG00000239887
ENSG00000239900
ENSG00000239961
ENSG00000239998
ENSG00000240050
ENSG00000240065
ENSG00000240184
ENSG00000240204
ENSG00000240224
ENSG00000240230
ENSG00000240303
ENSG00000240344
ENSG00000240505
ENSG00000240583
ENSG00000240654
ENSG00000240682
ENSG00000240694
ENSG00000240764
ENSG00000240771
ENSG00000240849
ENSG00000240871
ENSG00000240891
ENSG00000241058
ENSG00000241106
ENSG00000241119
ENSG00000241123
ENSG00000241127
ENSG00000241186
ENSG00000241233
ENSG00000241258
ENSG00000241322
ENSG00000241360
ENSG00000241399
ENSG00000241468

ENSG00000241476
ENSG00000241484
ENSG00000241553
ENSG00000241595
ENSG00000241635
ENSG00000241644
ENSG00000241685
ENSG00000241794
ENSG00000241839
ENSG00000241852
ENSG00000241878
ENSG00000241935
ENSG00000241962
ENSG00000241973
ENSG00000241978
ENSG00000242019
ENSG00000242028
ENSG00000242110
ENSG00000242114
ENSG00000242120
ENSG00000242173
ENSG00000242220
ENSG00000242247
ENSG00000242259
ENSG00000242265
ENSG00000242362
ENSG00000242366
ENSG00000242372
ENSG00000242498
ENSG00000242515
ENSG00000242550
ENSG00000242616
ENSG00000242689
ENSG00000242715
ENSG00000242732
ENSG00000242802
ENSG00000242852
ENSG00000242866

ENSG00000242950
ENSG00000243056
ENSG00000243073
ENSG00000243130
ENSG00000243135
ENSG00000243147
ENSG00000243156
ENSG00000243207
ENSG00000243232
ENSG00000243244
ENSG00000243279
ENSG00000243284
ENSG00000243317
ENSG00000243335
ENSG00000243364
ENSG00000243414
ENSG00000243440
ENSG00000243477
ENSG00000243543
ENSG00000243566
ENSG00000243627
ENSG00000243646
ENSG00000243667
ENSG00000243678
ENSG00000243709
ENSG00000243725
ENSG00000243749
ENSG00000243772
ENSG00000243789
ENSG00000243811
ENSG00000243902
ENSG00000243943
ENSG00000243978
ENSG00000244045
ENSG00000244094
ENSG00000244115
ENSG00000244122
ENSG00000244165

ENSG00000244187
ENSG00000244242
ENSG00000244274
ENSG00000244405
ENSG00000244462
ENSG00000244474
ENSG00000244476
ENSG00000244486
ENSG00000244537
ENSG00000244607
ENSG00000244617
ENSG00000244687
ENSG00000244694
ENSG00000244754
ENSG00000245680
ENSG00000245848
ENSG00000246705
ENSG00000247077
ENSG00000247315
ENSG00000247596
ENSG00000247746
ENSG00000248098
ENSG00000248099
ENSG00000248144
ENSG00000248329
ENSG00000248383
ENSG00000248385
ENSG00000248485
ENSG00000248592
ENSG00000248643
ENSG00000248672
ENSG00000248713
ENSG00000248746
ENSG00000248871
ENSG00000248905
ENSG00000248919
ENSG00000249115
ENSG00000249139

ENSG00000249158
ENSG00000249242
ENSG00000249437
ENSG00000249459
ENSG00000249471
ENSG00000249709
ENSG00000249853
ENSG00000249884
ENSG00000249915
ENSG00000249931
ENSG00000249992
ENSG00000250021
ENSG00000250120
ENSG00000250151
ENSG00000250305
ENSG00000250312
ENSG00000250317
ENSG00000250423
ENSG00000250486
ENSG00000250510
ENSG00000250565
ENSG00000250709
ENSG00000250741
ENSG00000251192
ENSG00000251201
ENSG00000251247
ENSG00000251258
ENSG00000251287
ENSG00000251369
ENSG00000251380
ENSG00000251493
ENSG00000251503
ENSG00000251664
ENSG00000253148
ENSG00000253159
ENSG00000253250
ENSG00000253276
ENSG00000253293

ENSG00000253304
ENSG00000253305
ENSG00000253313
ENSG00000253368
ENSG00000253485
ENSG00000253537
ENSG00000253626
ENSG00000253710
ENSG00000253719
ENSG00000253729
ENSG00000253731
ENSG00000253767
ENSG00000253797
ENSG00000253831
ENSG00000253846
ENSG00000253873
ENSG00000253910
ENSG00000253953
ENSG00000253958
ENSG00000254004
ENSG00000254087
ENSG00000254093
ENSG00000254122
ENSG00000254221
ENSG00000254245
ENSG00000254415
ENSG00000254440
ENSG00000254470
ENSG00000254505
ENSG00000254535
ENSG00000254585
ENSG00000254636
ENSG00000254685
ENSG00000254726
ENSG00000254827
ENSG00000254858
ENSG00000254901
ENSG00000254986

ENSG00000254996
ENSG00000254997
ENSG00000254999
ENSG00000255072
ENSG00000255112
ENSG00000255245
ENSG00000255251
ENSG00000255302
ENSG00000255346
ENSG00000255378
ENSG00000255398
ENSG00000255408
ENSG00000255423
ENSG00000255501
ENSG00000255529
ENSG00000255582
ENSG00000255587
ENSG00000255690
ENSG00000255823
ENSG00000255833
ENSG00000255837
ENSG00000255974
ENSG00000256043
ENSG00000256045
ENSG00000256053
ENSG00000256087
ENSG00000256162
ENSG00000256222
ENSG00000256229
ENSG00000256235
ENSG00000256294
ENSG00000256374
ENSG00000256537
ENSG00000256574
ENSG00000256646
ENSG00000256660
ENSG00000256671
ENSG00000256683

ENSG00000256713
ENSG00000256771
ENSG00000256870
ENSG00000256892
ENSG00000256977
ENSG00000257017
ENSG00000257093
ENSG00000257103
ENSG00000257108
ENSG00000257218
ENSG00000257335
ENSG00000257365
ENSG00000257594
ENSG00000257704
ENSG00000257923
ENSG00000257949
ENSG00000258102
ENSG00000258227
ENSG00000258289
ENSG00000258315
ENSG00000258366
ENSG00000258405
ENSG00000258429
ENSG00000258436
ENSG00000258555
ENSG00000258588
ENSG00000258643
ENSG00000258644
ENSG00000258659
ENSG00000258818
ENSG00000258839
ENSG00000258873
ENSG00000258986
ENSG00000259024
ENSG00000259075
ENSG00000259112
ENSG00000259120
ENSG00000259224

ENSG00000259288
ENSG00000259305
ENSG00000259316
ENSG00000259332
ENSG00000259399
ENSG00000259431
ENSG00000259753
ENSG00000259803
ENSG00000259823
ENSG00000259916
ENSG00000259956
ENSG00000260027
ENSG00000260097
ENSG00000260220
ENSG00000260238
ENSG00000260286
ENSG00000260314
ENSG00000260428
ENSG00000260458
ENSG00000260596
ENSG00000260691
ENSG00000260807
ENSG00000260903
ENSG00000260916
ENSG00000261052
ENSG00000261115
ENSG00000261221
ENSG00000261272
ENSG00000261371
ENSG00000261456
ENSG00000261459
ENSG00000261509
ENSG00000261594
ENSG00000261609
ENSG00000261667
ENSG00000261678
ENSG00000261701
ENSG00000261740

ENSG00000261787
ENSG00000261794
ENSG00000261796
ENSG00000261934
ENSG00000262179
ENSG00000262209
ENSG00000262246
ENSG00000262484
ENSG00000262576
ENSG00000262633
ENSG00000262655
ENSG00000262664
ENSG00000262814
ENSG00000262874
ENSG00000262919
ENSG00000263001
ENSG00000263002
ENSG00000263155
ENSG00000263201
ENSG00000263353
ENSG00000263464
ENSG00000263465
ENSG00000263513
ENSG00000263528
ENSG00000263715
ENSG00000263761
ENSG00000263956
ENSG00000263961
ENSG00000264058
ENSG00000264230
ENSG00000264343
ENSG00000264364
ENSG00000264522
ENSG00000264717
ENSG00000264813
ENSG00000265107
ENSG00000265190
ENSG00000265241

ENSG00000265354
ENSG00000265763
ENSG00000265808
ENSG00000265817
ENSG00000265972
ENSG00000266028
ENSG00000266074
ENSG00000266094
ENSG00000266173
ENSG00000266265
ENSG00000266302
ENSG00000266338
ENSG00000266412
ENSG00000266472
ENSG00000266524
ENSG00000266964
ENSG00000267041
ENSG00000267059
ENSG00000267060
ENSG00000267221
ENSG00000267368
ENSG00000267467
ENSG00000267508
ENSG00000267534
ENSG00000267680
ENSG00000267855
ENSG00000267909
ENSG00000267978
ENSG00000268009
ENSG00000268043
ENSG00000268104
ENSG00000268182
ENSG00000268221
ENSG00000268223
ENSG00000268350
ENSG00000268447
ENSG00000268500
ENSG00000268606

ENSG00000268916
ENSG00000268940
ENSG00000269096
ENSG00000269113
ENSG00000269190
ENSG00000269313
ENSG00000269335
ENSG00000269343
ENSG00000269404
ENSG00000269433
ENSG00000269502
ENSG00000269526
ENSG00000269556
ENSG00000269586
ENSG00000269713
ENSG00000269743
ENSG00000269791
ENSG00000269858
ENSG00000269897
ENSG00000269955
ENSG00000269964
ENSG00000270024
ENSG00000270136
ENSG00000270181
ENSG00000270339
ENSG00000270379
ENSG00000270601
ENSG00000270617
ENSG00000270629
ENSG00000270647
ENSG00000270672
ENSG00000270757
ENSG00000270885
ENSG00000270946
ENSG00000271092
ENSG00000271254
ENSG00000271303
ENSG00000271383

ENSG00000271447
ENSG00000271503
ENSG00000271567
ENSG00000271601
ENSG00000271605
ENSG00000272031
ENSG00000272047
ENSG00000272196
ENSG00000272325
ENSG00000272391
ENSG00000272410
ENSG00000272414
ENSG00000272573
ENSG00000272636
ENSG00000272674
ENSG00000272886
ENSG00000272899
ENSG00000272916
ENSG00000272968
ENSG00000273045
ENSG00000273046
ENSG00000273079
ENSG00000273136
ENSG00000273173
ENSG00000273217
ENSG00000273259
ENSG00000273274
ENSG00000273559
ENSG00000273590
ENSG00000273604
ENSG00000273611
ENSG00000273706
ENSG00000273749
ENSG00000273820
ENSG00000273899
ENSG00000273976
ENSG00000274070
ENSG00000274180

ENSG00000274211
ENSG00000274286
ENSG00000274523
ENSG00000274588
ENSG00000274600
ENSG00000274749
ENSG00000274997
ENSG00000275004
ENSG00000275023
ENSG00000275034
ENSG00000275052
ENSG00000275066
ENSG00000275074
ENSG00000275111
ENSG00000275152
ENSG00000275183
ENSG00000275302
ENSG00000275356
ENSG00000275410
ENSG00000275553
ENSG00000275591
ENSG00000275700
ENSG00000275722
ENSG00000275793
ENSG00000275832
ENSG00000275896
ENSG00000275993
ENSG00000276043
ENSG00000276070
ENSG00000276076
ENSG00000276085
ENSG00000276203
ENSG00000276231
ENSG00000276256
ENSG00000276289
ENSG00000276293
ENSG00000276345
ENSG00000276409

ENSG00000276547
ENSG00000276600
ENSG00000276644
ENSG00000276760
ENSG00000277117
ENSG00000277196
ENSG00000277203
ENSG00000277258
ENSG00000277288
ENSG00000277322
ENSG00000277363
ENSG00000277399
ENSG00000277443
ENSG00000277494
ENSG00000277586
ENSG00000277632
ENSG00000277758
ENSG00000277893
ENSG00000277957
ENSG00000277972
ENSG00000278023
ENSG00000278053
ENSG00000278057
ENSG00000278129
ENSG00000278195
ENSG00000278259
ENSG00000278318
ENSG00000278463
ENSG00000278505
ENSG00000278522
ENSG00000278535
ENSG00000278540
ENSG00000278558
ENSG00000278570
ENSG00000278615
ENSG00000278619
ENSG00000278662
ENSG00000278685

ENSG00000278817
ENSG00000278845
ENSG00000278848
ENSG00000278961
ENSG00000279362
ENSG00000279782
ENSG00000279804
ENSG00000280071
ENSG00000280267
ENSG00000280433
ENSG00000280755
ENSG00000280789
ENSG00000280987
ENSG00000281028
ENSG00000281766
ENSG00000281887
ENSG00000281938
ENSG00000281991
ENSG00000282301
ENSG00000282608
ENSG00000282960
ENSG00000283088

Overlap Results

Collection(s):	H
# overlaps shown:	10
# genesets in collections:	50
# genes in comparison (n):	277
# genes in universe (N):	45956

Gene Set Name	# Genes in Gen	Description	# Genes in Ove	k/K	p-value	FDR q-value
HALLMARK_HEDGEHOG_SIGNALING	36	Genes up-regulated by activation of hedgehog signaling.	7	0.1944	1.93E-09	9.63E-08
HALLMARK_EPITHELIAL_MESENCHYMAL_TRANSITION	200	Genes defining epithelial-mesenchymal transition as in wound healing, fibrosis and metastasis.	11	0.055	4.44E-08	1.11E-06
HALLMARK_UV_RESPONSE_DN	144	Genes down-regulated in response to ultraviolet (UV) radiation.	8	0.0556	2.92E-06	3.32E-05
HALLMARK_HYPOXIA	200	Genes up-regulated in response to low oxygen levels (hypoxia).	9	0.045	3.98E-06	3.32E-05
HALLMARK_P53_PATHWAY	200	Genes involved in p53 pathways and networks.	9	0.045	3.98E-06	3.32E-05
HALLMARK_TNFA_SIGNALING_VIA_NFKB	200	Genes regulated by NF-kB in response to TNF [GeneID=7124].	9	0.045	3.98E-06	3.32E-05
HALLMARK_ESTROGEN_RESPONSE_LATE	200	Genes defining late response to estrogen.	6	0.03	1.41E-03	8.83E-03
HALLMARK_KRAS_SIGNALING_UP	200	Genes up-regulated by KRAS activation.	6	0.03	1.41E-03	8.83E-03
HALLMARK_ANDROGEN_RESPONSE	101	Genes defining response to androgens.	4	0.0396	3.34E-03	1.85E-02
HALLMARK_MYOGENESIS	200	Genes involved in development of skeletal muscle (myogenesis).	5	0.025	7.51E-03	3.76E-02

Gene/Gene Set Overlap Matrix

Entrez Gene Id	Gene Symbol	Gene Description	HALLMARK_HE	HALLMARK_EPI	HALLMARK_UV	HALLMARK_HY
7857	SCG2	secretogranin II	HALLMARK_HE	HALLMARK_EPITHELIAL_MESENCHYMAL_TRAN		
7436	VLDLR	very low density lipoprotein receptor	HALLMARK_HEDGEHOG_SIGNA		HALLMARK_UV	HALLMARK_HY

8829	NRP1	neuropilin 1	HALLMARK_HEDGEHOG_SIGNA		HALLMARK_UV_RESPONSE_DN	
43	ACHE	acetylcholinesterase	HALLMARK_HEDGEHOG_SIGNALING			
23493	HEY2	hairy/enhancer-of-split related with YRPW motif 2	HALLMARK_HEDGEHOG_SIGNALING			
7088	TLE1	transducin-like enhancer of split 1 (E(sp1) homolo	HALLMARK_HEDGEHOG_SIGNALING			
8633	UNC5C	unc-5 homolog C (C. elegans)	HALLMARK_HEDGEHOG_SIGNALING			
1281	COL3A1	collagen, type III, alpha 1		HALLMARK_EPI	HALLMARK_UV_RESPONSE_DN	
3725	JUN	jun proto-oncogene		HALLMARK_EPITHELIAL_MESEN		HALLMARK_HY
1647	GADD45A	growth arrest and DNA-damage-inducible, alpha		HALLMARK_EPITHELIAL_MESENCHYMAL_TRAN		
3624	INHBA	inhibin, beta A		HALLMARK_EPITHELIAL_MESENCHYMAL_TRAN		
2669	GEM	GTP binding protein overexpressed in skeletal muscle		HALLMARK_EPITHELIAL_MESENCHYMAL_TRAN		
6387	CXCL12	chemokine (C-X-C motif) ligand 12		HALLMARK_EPITHELIAL_MESENCHYMAL_TRAN		
10409	BASP1	brain abundant, membrane attached signal protein 1		HALLMARK_EPITHELIAL_MESENCHYMAL_TRAN		
50509	COL5A3	collagen, type V, alpha 3		HALLMARK_EPITHELIAL_MESENCHYMAL_TRAN		
6424	SFRP4	secreted frizzled-related protein 4		HALLMARK_EPITHELIAL_MESENCHYMAL_TRAN		
7431	VIM	vimentin		HALLMARK_EPITHELIAL_MESENCHYMAL_TRAN		
10370	CITED2	Cbp/p300-interacting transactivator, with Glu/Asp-rich carboxy-terminal domain, 2			HALLMARK_UV	HALLMARK_HYI
2908	NR3C1	nuclear receptor subfamily 3, group C, member 1 (glucocorticoid receptor)			HALLMARK_UV	HALLMARK_HYI
3488	IGFBP5	insulin-like growth factor binding protein 5			HALLMARK_UV_RESPONSE_DN	
4781	NFIB	nuclear factor I/B			HALLMARK_UV_RESPONSE_DN	
9975	NR1D2	nuclear receptor subfamily 1, group D, member 2			HALLMARK_UV_RESPONSE_DN	
694	BTG1	B-cell translocation gene 1, anti-proliferative				HALLMARK_HY
5224	PGAM2	phosphoglycerate mutase 2 (muscle)				HALLMARK_HYI
1028	CDKN1C	cyclin-dependent kinase inhibitor 1C (p57, Kip2)				HALLMARK_HYI
10891	PPARGC1A	peroxisome proliferator-activated receptor gamma, coactivator 1 alpha				HALLMARK_HYI
9957	HS3ST1	heparan sulfate (glucosamine) 3-O-sulfotransferase 1				HALLMARK_HYI
10769	PLK2	polo-like kinase 2				
10628	TXNIP	thioredoxin interacting protein				
26471	NUPR1	nuclear protein, transcriptional regulator, 1				
8493	PPM1D	protein phosphatase, Mg2+/Mn2+ dependent, 1D				
3006	HIST1H1C	histone cluster 1, H1c				
4193	MDM2	Mdm2 p53 binding protein homolog (mouse)				
65986	ZBTB10	zinc finger and BTB domain containing 10				
604	BCL6	B-cell CLL/lymphoma 6				
4082	MARCKS	myristoylated alanine-rich protein kinase C substrate				
11098	PRSS23	protease, serine, 23				
2296	FOXC1	forkhead box C1				
8204	NRIP1	nuclear receptor interacting protein 1				
5733	PTGER3	prostaglandin E receptor 3 (subtype EP3)				

10610	ST6GALNAC2	ST6 (alpha-N-acetyl-neuraminyl-2,3-beta-galactosyl-1,3)-N-acetylgalactosaminide alpha-2,6-sialyltransferase 2
6662	SOX9	SRY (sex determining region Y)-box 9
10912	GADD45G	growth arrest and DNA-damage-inducible, gamma
2254	FGF9	fibroblast growth factor 9 (glia-activating factor)
6616	SNAP25	synaptosomal-associated protein, 25kDa
84159	ARID5B	AT rich interactive domain 5B (MRF1-like)
4094	MAF	v-maf musculoaponeurotic fibrosarcoma oncogene homolog (avian)
54407	SLC38A2	solute carrier family 38, member 2
88	ACTN2	actinin, alpha 2
10468	FST	follistatin
90427	BMF	Bcl2 modifying factor
1006	CDH8	cadherin 8, type 2
11096	ADAMTS5	ADAM metallopeptidase with thrombospondin type 1 motif, 5
147	ADRA1B	adrenergic, alpha-1B-, receptor
1233	CCR4	chemokine (C-C motif) receptor 4
6335	SCN9A	sodium channel, voltage-gated, type IX, alpha subunit
3759	KCNJ2	potassium inwardly-rectifying channel, subfamily J, member 2
5099	PCDH7	protocadherin 7
7439	BEST1	bestrophin 1
5334	PLCL1	phospholipase C-like 1
83729	INHBE	inhibin, beta E
1946	EFNA5	ephrin-A5
4211	MEIS1	Meis homeobox 1
4306	NR3C2	nuclear receptor subfamily 3, group C, member 2
6579	SLCO1A2	solute carrier organic anion transporter family, member 1A2
51384	WNT16	wingless-type MMTV integration site family, member 16
727	C5	complement component 5
552900	BOLA2	bolA homolog 2 (E. coli)
10512	SEMA3C	sema domain, immunoglobulin domain (Ig), short basic domain, secreted, (semaphorin) 3C
100048912	CDKN2B-AS	CDKN2B antisense RNA (non-protein coding)
100133941	CD24	CD24 molecule
100287082	LOC100287082	uncharacterized LOC100287082
100421577	LOC100421577	family with sequence similarity 21, member A pseudogene
100506421	LOC100506421	uncharacterized LOC100506421
10082	GPC6	glypican 6
10154	PLXNC1	plexin C1
101927282		
101927345		

101927501		
101927631		
101928371		
101928978		
101929333		
10194	TSHZ1	teashirt zinc finger homeobox 1
102723166		
105	ADARB2	adenosine deaminase, RNA-specific, B2
105371083		
105378763		
10568	SLC34A2	solute carrier family 34 (sodium phosphate), member 2
10900	RUNDC3A	RUN domain containing 3A
11228	RASSF8	Ras association (RalGDS/AF-6) domain family (N-terminal) member 8
11278	KLF12	Kruppel-like factor 12
11281	POU6F2	POU class 6 homeobox 2
112885	PHF21B	PHD finger protein 21B
114795	TMEM132B	transmembrane protein 132B
115111	SLC26A7	solute carrier family 26, member 7
116372	LYPD1	LY6/PLAUR domain containing 1
117583	PARD3B	par-3 partitioning defective 3 homolog B (C. elegans)
120114	FAT3	FAT tumor suppressor homolog 3 (Drosophila)
120146	TRIM64	tripartite motif containing 64
122945	NOXRED1	NADP-dependent oxidoreductase domain containing 1
1270	CNTF	ciliary neurotrophic factor
128153	SPATA17	spermatogenesis associated 17
128312	HIST3H2BB	histone cluster 3, H2bb
128346	C1orf162	chromosome 1 open reading frame 162
129530	LYG1	lysozyme G-like 1
129868	TRIM43	tripartite motif containing 43
136288	C7orf57	chromosome 7 open reading frame 57
143686	SESN3	sestrin 3
147660	ZNF578	zinc finger protein 578
147686	ZNF418	zinc finger protein 418
148523	C1orf51	chromosome 1 open reading frame 51
148709	LOC148709	actin pseudogene
149281	METTL11B	methyltransferase like 11B
153020	RASGEF1B	RasGEF domain family, member 1B
153222	C5orf41	chromosome 5 open reading frame 41

154215	NKAIN2	Na+/K+ transporting ATPase interacting 2
157489	SDAD1P1	SDA1 domain containing 1 pseudogene 1
157773	C8orf48	chromosome 8 open reading frame 48
158160	HSD17B7P2	hydroxysteroid (17-beta) dehydrogenase 7 pseudogene 2
1602	DACH1	dachshund homolog 1 (Drosophila)
170690	ADAMTS16	ADAM metallopeptidase with thrombospondin type 1 motif, 16
1951	CELSR3	cadherin, EGF LAG seven-pass G-type receptor 3 (flamingo homolog, Drosophila)
195828	ZNF367	zinc finger protein 367
1993	ELAVL2	ELAV (embryonic lethal, abnormal vision, Drosophila)-like 2 (Hu antigen B)
201456	FBXO15	F-box protein 15
201516	ZSCAN4	zinc finger and SCAN domain containing 4
201625	DNAH12	dynein, axonemal, heavy chain 12
202333	CMYA5	cardiomyopathy associated 5
2042	EPHA3	EPH receptor A3
2043	EPHA4	EPH receptor A4
2066	ERBB4	v-erb-a erythroblastic leukemia viral oncogene homolog 4 (avian)
219539	YPEL4	yippee-like 4 (Drosophila)
220965	FAM13C	family with sequence similarity 13, member C
23213	SULF1	sulfatase 1
23635	SSBP2	single-stranded DNA binding protein 2
23768	FLRT2	fibronectin leucine rich transmembrane protein 2
254158	CXorf58	chromosome X open reading frame 58
255743	NPNT	nephronectin
255928	SYT14	synaptotagmin XIV
257106	ARHGAP30	Rho GTPase activating protein 30
25788	RAD54B	RAD54 homolog B (S. cerevisiae)
25849	PARM1	prostate androgen-regulated mucin-like protein 1
26040	SETBP1	SET binding protein 1
27086	FOXP1	forkhead box P1
27113	BBC3	BCL2 binding component 3
27145	FILIP1	filamin A interacting protein 1
27233	SULT1C4	sulfotransferase family, cytosolic, 1C, member 4
2737	GLI3	GLI family zinc finger 3
283116	LOC283116	tripartite motif-containing protein LOC642612-like
283131	NEAT1	nuclear paraspeckle assembly transcript 1 (non-protein coding)
285429	DCAF4L1	DDB1 and CUL4 associated factor 4-like 1
286006	C7orf53	chromosome 7 open reading frame 53
3017	HIST1H2BD	histone cluster 1, H2bd

338651	LOC338651	uncharacterized LOC338651
339977	LRRC66	leucine rich repeat containing 66
340267	COL28A1	collagen, type XXVIII, alpha 1
342931	RFPL4A	ret finger protein-like 4A
343472	BARHL2	BarH-like homeobox 2
344148	NCKAP5	NCK-associated protein 5
345611	IRGM	immunity-related GTPase family, M
376940	ZC3H6	zinc finger CCCH-type containing 6
378938	MALAT1	metastasis associated lung adenocarcinoma transcript 1 (non-protein coding)
3791	KDR	kinase insert domain receptor (a type III receptor tyrosine kinase)
387640	C10orf140	chromosome 10 open reading frame 140
387758	FIBIN	fin bud initiation factor homolog (zebrafish)
389384	C6orf222	chromosome 6 open reading frame 222
390245	KDM4DL	lysine (K)-specific demethylase 4D-like
390999	PRAMEF12	PRAME family member 12
391002	PRAMEF8	PRAME family member 8
399726	C10orf114	chromosome 10 open reading frame 114
399939	LOC399939	ring finger protein 18-like
400456	LOC400456	uncharacterized LOC400456
400617	FLJ36644	uncharacterized LOC400617
401024	FSIP2	fibrous sheath interacting protein 2
401093	LOC401093	uncharacterized LOC401093
401190	RGS7BP	regulator of G-protein signaling 7 binding protein
401546	C9orf152	chromosome 9 open reading frame 152
4133	MAP2	microtubule-associated protein 2
4254	KITLG	KIT ligand
4330	MN1	meningioma (disrupted in balanced translocation) 1
440689	HIST2H2BF	histone cluster 2, H2bf
441094	FLJ42709	uncharacterized LOC441094
441307	FLJ44511	uncharacterized LOC441307
441871	PRAMEF7	PRAME family member 7
442247	RFPL4B	ret finger protein-like 4B
4640	MYO1A	myosin IA
4774	NFIA	nuclear factor I/A
4883	NPR3	natriuretic peptide receptor C/guanylate cyclase C (atrionatriuretic peptide receptor C)
4919	ROR1	receptor tyrosine kinase-like orphan receptor 1
5087	PBX1	pre-B-cell leukemia homeobox 1
51339	DACT1	dapper, antagonist of beta-catenin, homolog 1 (Xenopus laevis)

51513	ETV7	ets variant 7
53335	BCL11A	B-cell CLL/lymphoma 11A (zinc finger protein)
54055	CYP4F29P	cytochrome P450, family 4, subfamily F, polypeptide 29, pseudogene
54510	PCDH18	protocadherin 18
55089	SLC38A4	solute carrier family 38, member 4
554313	HIST2H4B	histone cluster 2, H4b
55534	MAML3	mastermind-like 3 (Drosophila)
55567	DNAH3	dynein, axonemal, heavy chain 3
55714	ODZ3	odz, odd Oz/ten-m homolog 3 (Drosophila)
55799	CACNA2D3	calcium channel, voltage-dependent, alpha 2/delta subunit 3
56100	PCDHGB6	protocadherin gamma subfamily B, 6
56106	PCDHGA10	protocadherin gamma subfamily A, 10
56139	PCDHA10	protocadherin alpha 10
57091	CASS4	Cas scaffolding protein family member 4
57093	TRIM49	tripartite motif containing 49
57161	PELI2	pellino homolog 2 (Drosophila)
57507	ZNF608	zinc finger protein 608
57554	LRRC7	leucine rich repeat containing 7
57556	SEMA6A	sema domain, transmembrane domain (TM), and cytoplasmic domain, (semaphorin) 6A
57561	ARRDC3	arrestin domain containing 3
57568	SIPA1L2	signal-induced proliferation-associated 1 like 2
57687	VAT1L	vesicle amine transport protein 1 homolog (T. californica)-like
58480	RHOU	ras homolog gene family, member U
5915	RARB	retinoic acid receptor, beta
5973	RENBP	renin binding protein
64093	SMOC1	SPARC related modular calcium binding 1
642446	TRIM64B	tripartite motif containing 64B
642612	TRIM49L2	tripartite motif containing 49-like 2
64288	ZNF323	zinc finger protein 323
643180	CCT6P3	chaperonin containing TCP1, subunit 6 (zeta) pseudogene 3
643401	LOC643401	uncharacterized LOC643401
644192	LOC644192	uncharacterized LOC644192
64800	EFCAB6	EF-hand calcium binding domain 6
653	BMP5	bone morphogenetic protein 5
653192	TRIM43B	tripartite motif containing 43B
6657	SOX2	SRY (sex determining region Y)-box 2
6659	SOX4	SRY (sex determining region Y)-box 4
6660	SOX5	SRY (sex determining region Y)-box 5

6664	SOX11	SRY (sex determining region Y)-box 11
7025	NR2F1	nuclear receptor subfamily 2, group F, member 1
729384	TRIM49L1	tripartite motif containing 49-like 1
729974	LOC729974	ret finger protein-like 4A-like
7738	ZNF184	zinc finger protein 184
79097	TRIM48	tripartite motif containing 48
79365	BHLHE41	basic helix-loop-helix family, member e41
79633	FAT4	FAT tumor suppressor homolog 4 (Drosophila)
79667	FLJ13197	uncharacterized FLJ13197
79718	TBL1XR1	transducin (beta)-like 1 X-linked receptor 1
79819	WDR78	WD repeat domain 78
79960	PHF17	PHD finger protein 17
79992	AGPAT4-IT1	AGPAT4 intronic transcript 1 (non-protein coding)
80319	CXXC4	CXXC finger protein 4
80345	ZSCAN16	zinc finger and SCAN domain containing 16
80726	KIAA1683	KIAA1683
80816	ASXL3	additional sex combs like 3 (Drosophila)
81575	APOLD1	apolipoprotein L domain containing 1
8334	HIST1H2AC	histone cluster 1, H2ac
8339	HIST1H2BG	histone cluster 1, H2bg
8341	HIST1H2BN	histone cluster 1, H2bn
8344	HIST1H2BE	histone cluster 1, H2be
8347	HIST1H2BC	histone cluster 1, H2bc
83478	ARHGAP24	Rho GTPase activating protein 24
8365	HIST1H4H	histone cluster 1, H4h
8367	HIST1H4E	histone cluster 1, H4e
8370	HIST2H4A	histone cluster 2, H4a
83992	CTTNBP2	cortactin binding protein 2
84206	MEX3B	mex-3 homolog B (C. elegans)
8462	KLF11	Kruppel-like factor 11
84665	MYPN	myopalladin
84858	ZNF503	zinc finger protein 503
84989	LOC84989	uncharacterized LOC84989
860	RUNX2	runt-related transcription factor 2
862	RUNX1T1	runt-related transcription factor 1; translocated to, 1 (cyclin D-related)
8632	DNAH17	dynein, axonemal, heavy chain 17
8739	HRK	harakiri, BCL2 interacting protein (contains only BH3 domain)
8863	PER3	period homolog 3 (Drosophila)

8969	HIST1H2AG	histone cluster 1, H2ag
8970	HIST1H2BJ	histone cluster 1, H2bj
92344	GORAB	golgin, RAB6-interacting
9315	C5orf13	chromosome 5 open reading frame 13
93166	PRDM6	PR domain containing 6
93986	FOXP2	forkhead box P2
9515	STXBP5L	syntaxin binding protein 5-like
9572	NR1D1	nuclear receptor subfamily 1, group D, member 1
9839	ZEB2	zinc finger E-box binding homeobox 2

	HALLMARK_P53	HALLMARK_TN	HALLMARK_EST	HALLMARK_KR	HALLMARK_AN	HALLMARK_MYOGENESIS
SITION						
POXIA						

			HALLMARK_KRAS_SIGNALING_UP		
					HALLMARK_MYOGENESIS
					HALLMARK_MYOGENESIS
HALLMARK_P5	HALLMARK_TNFA_SIGNALING_VIA_NFKB				
HALLMARK_P5	HALLMARK_TNFA_SIGNALING_VIA_NFKB				
SITION	HALLMARK_TNFA_SIGNALING_		HALLMARK_KRAS_SIGNALING_UP		
SITION	HALLMARK_TNFA_SIGNALING_VIA_NFKB				
SITION		HALLMARK_ESTROGEN_RESPONSE_LATE			
SITION					
SITION					
SITION					
SITION					
POXIA					
POXIA					
HALLMARK_P5	HALLMARK_TNFA_SIGNALING_VIA_NFKB				
POXIA					HALLMARK_MYOGENESIS
POXIA					
POXIA					
POXIA					
HALLMARK_P5	HALLMARK_TNFA_SIGNALING_VIA_NFKB				
HALLMARK_P53_PATHWAY					
HALLMARK_P53_PATHWAY					
HALLMARK_P53_PATHWAY					
HALLMARK_P53_PATHWAY					
HALLMARK_P53_PATHWAY					
	HALLMARK_TNFA_SIGNALING_VIA_NFKB			HALLMARK_ANDROGEN_RESPONSE	
	HALLMARK_TNFA_SIGNALING_VIA_NFKB				
	HALLMARK_TNFA_SIGNALING_VIA_NFKB				
		HALLMARK_ESTROGEN_RESPONSE_LATE			
		HALLMARK_ESTROGEN_RESPONSE_LATE			
		HALLMARK_ESTROGEN_RESPONSE_LATE			
		HALLMARK_ESTROGEN_RESPONSE_LATE			

HALLMARK_ESTROGEN_RESPONSE_LATE			
	HALLMARK_KRAS_SIGNALING_UP		
	HALLMARK_KRAS_SIGNALING_UP		
	HALLMARK_KRAS_SIGNALING_UP		
	HALLMARK_KRAS_SIGNALING_UP		
		HALLMARK_ANDROGEN_RESPONSE	
		HALLMARK_ANDROGEN_RESPONSE	
		HALLMARK_ANDROGEN_RESPONSE	
			HALLMARK_MYOGENESIS
			HALLMARK_MYOGENESIS
