# Supplementary material for: Chromosome 19 microRNA cluster enhances cell reprogramming by inhibiting epithelial-to-mesenchymal transition
Source: Sci Rep. 2020 Feb 20;10:3029. doi: 10.1038/s41598-020-59812-8 (PMC7033247; doi:10.1038/s41598-020-59812-8)
Supplement: Supplementary file 4 — Supplementary table S3. [file 41598_2020_59812_MOESM4_ESM.pdf]

Sheet "miRNAs Hypoxia-Normoxia": small miRNA sequencing data analysis of miRNAs differentially exppressed in iPSCs cultured in hypoxia compared to normoxia.

Sheet "Cistrons Hypoxia-Normoxia": small miRNA sequencing data analysis of miRNA cistrons differentially exppressed in iPSCs cultured in hypoxia compared to normoxia.

miRNA	Normalized frequency (%) Hypoxia	Normoxia	Linear fold chan	P value	FDR
210(1)	0.004146	0.000298	13.9	4.19569E-09	2.45867E-06
9STAR(3)	0.000173	0.000029	5.9	0.001021447	0.099761302
598(1)	0.000326	0.000104	3.1	0.001931411	0.141475871
9(3)	0.003652	0.001202	3	0.000457373	0.072964065
503(1)	0.000532	0.000191	2.8	0.000396417	0.072964065
21STAR(1)	0.000622	0.000219	2.8	0.000814109	0.095413552
30d(1)	0.006211	0.003261	1.9	0.002748052	0.178928731
498(1)	0.000073	0.000219	-3	0.001257955	0.105308777
518c-3p(1)	0.000048	0.000194	-4	0.000498048	0.072964065

	Normalized frequency (%)				
miRNA cistron	Hypoxia	Normoxia	Linear fold chan	P value	FDR
210(1)	0.412455	0.029741	13.9	4.96E-11	1.33956E-08
489(2)	0.012991	0.003859	3.4	0.004330817	0.167045789
9-1(3)	0.381634	0.123784	3.1	0.000201806	0.013621888
424(2)	0.108522	0.036140	3	6.03454E-05	0.008146628
598(1)	0.033100	0.010923	3	0.00191234	0.103266344
30b(2)	0.683601	0.385398	1.8	0.002823007	0.127035326
498(46)	0.342795	0.653611	-1.9	0.000119826	0.010784374
