# Supplementary material for: Chromosome 19 microRNA cluster enhances cell reprogramming by inhibiting epithelial-to-mesenchymal transition
Source: Sci Rep. 2020 Feb 20;10:3029. doi: 10.1038/s41598-020-59812-8 (PMC7033247; doi:10.1038/s41598-020-59812-8)
Supplement: Supplementary file 5 — Supplementary table S4. [file 41598_2020_59812_MOESM5_ESM.pdf]

Sheet "mRNAs Hypoxia-Normoxia ": RNA sequencing data analysis of differentially expressed mRNAs in iPSC
cultured in hypoxia for 24 h compared to normoxia control.

Sheet "GSEA upregulated genes ": Gene set expression analysis of significantly upregulated mRNAs in iPSCs
cultured in cultured in hypoxia for 24 h compared to normoxia control.

Gene		Linear Fold Change	P Value	FDR	Transcripts per million (TPM)							
					TestMean	TestStdDev	TestMin	TestMax	RefMean	RefStdDev	RefMin	RefMax
ENSG00000198695	MT-ND6	-24.9	8.3E-08	4.4E-07	47.592	16.122	33.701	65.271	1205.047	303.617	958.432	1544.153
ENSG00000210196	MT-TP	-19.7	1.3E-17	4.5E-16	11.127	3.976	6.810	14.637	227.196	49.426	171.455	265.679
ENSG00000173404	INSM1	-16.3	2.8E-05	9.3E-05	0.017	0.029	0.000	0.050	0.392	0.044	0.346	0.434
ENSG00000210194	MT-TE	-15.9	5.0E-11	5.2E-10	4.298	3.731	0.000	6.711	74.604	22.412	55.335	99.199
ENSG00000210107	MT-TQ	-12.2	1.6E-14	3.4E-13	9.437	5.537	3.216	13.824	119.623	5.412	113.635	124.167
ENSG00000224039	CDYLP1	-11	4.5E-02	7.3E-02	0.000	0.000	0.000	0.000	0.125	0.143	0.000	0.281
ENSG00000130222	GADD45G	-8.9	1.3E-13	2.3E-12	0.648	0.139	0.491	0.753	5.948	0.433	5.530	6.395
ENSG00000129990	SYT5	-8.4	1.2E-05	4.2E-05	0.037	0.014	0.029	0.053	0.345	0.111	0.237	0.459
ENSG00000210140	MT-TC	-8.3	6.5E-09	4.4E-08	7.877	4.678	3.689	12.926	69.622	18.893	50.619	88.403
ENSG00000139174	PRICKLE1	-7.8	5.6E-29	1.2E-26	1.558	0.136	1.414	1.684	12.219	0.276	12.019	12.534
ENSG00000209082	MT-TL1	-7.8	8.0E-19	3.4E-17	42.086	3.191	39.102	45.449	330.531	35.873	292.414	363.633
ENSG00000138795	LEF1	-7	7.2E-05	2.2E-04	0.035	0.061	0.000	0.106	0.285	0.069	0.208	0.341
ENSG00000127084	FGD3	-6.6	7.8E-11	7.6E-10	0.140	0.049	0.097	0.193	0.935	0.113	0.806	1.019
ENSG00000134817	APLNR	-6.6	1.5E-06	6.4E-06	0.095	0.017	0.083	0.114	0.653	0.261	0.451	0.948
ENSG00000124092	CTCFL	-5.8	5.3E-17	1.6E-15	0.453	0.067	0.398	0.527	2.614	0.123	2.535	2.756
ENSG00000075388	FGF4	-5.7	1.4E-07	7.0E-07	0.206	0.104	0.095	0.301	1.192	0.406	0.886	1.652
ENSG00000161798	AQP5	-5.7	7.2E-06	2.7E-05	0.243	0.141	0.082	0.344	1.424	0.721	0.820	2.223
ENSG00000214106	PAXIP1-AS2	-5.5	2.1E-06	8.5E-06	0.175	0.112	0.048	0.259	0.963	0.354	0.564	1.238
ENSG00000198712	MT-CO2	-5.5	3.1E-28	6.0E-26	4137.258	386.701	3825.173	4569.869	22650.007	143.121	22487.227	22756.108
ENSG00000236154		-5.5	7.2E-03	1.4E-02	0.188	0.326	0.000	0.565	1.283	0.551	0.833	1.898
ENSG00000229344	MTCO2P12	-5.4	5.8E-25	6.9E-23	19.536	2.251	17.239	21.737	106.399	5.551	100.548	111.583
ENSG00000118004	COLEC11	-5.3	3.1E-06	1.2E-05	0.183	0.159	0.000	0.292	1.000	0.063	0.933	1.056
ENSG00000101638	ST8SIA5	-5.2	5.0E-11	5.2E-10	0.089	0.018	0.072	0.108	0.475	0.108	0.389	0.597
ENSG00000137078	SIT1	-5.2	5.6E-12	7.1E-11	1.220	0.360	0.814	1.500	6.494	0.580	5.857	6.993
ENSG00000228253	MT-ATP8	-5.2	6.3E-11	6.4E-10	3113.449	431.383	2683.029	3555.016	16252.870	939.475	15645.167	17334.948
ENSG00000256463	SALL3	-5.1	2.6E-22	2.0E-20	1.402	0.025	1.377	1.426	7.228	0.150	7.101	7.394
ENSG00000229271		-5.1	1.0E-04	3.0E-04	0.741	0.646	0.000	1.185	3.947	1.401	2.719	5.473
ENSG00000164120	HPGD	-4.9	1.9E-05	6.4E-05	0.088	0.106	0.000	0.206	0.461	0.070	0.395	0.534
ENSG00000092345	DAZL	-4.9	5.5E-12	7.0E-11	0.596	0.243	0.340	0.822	2.976	0.633	2.291	3.539
ENSG00000147381	MAGEA4	-4.8	1.3E-07	6.9E-07	0.294	0.065	0.250	0.369	1.443	0.125	1.369	1.587
ENSG00000118513	MYB	-4.7	2.4E-14	4.7E-13	0.638	0.208	0.473	0.871	3.041	0.105	2.924	3.128
ENSG00000143297	FCRL5	-4.6	5.8E-11	6.0E-10	0.140	0.029	0.112	0.170	0.645	0.024	0.623	0.670
ENSG00000110090	CPT1A	-4.6	5.3E-20	2.7E-18	1.473	0.314	1.213	1.822	6.846	0.625	6.403	7.561
ENSG00000101180	HRH3	-4.5	1.7E-10	1.5E-09	0.646	0.197	0.486	0.867	2.959	0.400	2.720	3.421
ENSG00000168505	GBX2	-4.5	1.9E-06	7.8E-06	0.404	0.330	0.133	0.771	1.802	0.261	1.531	2.052
ENSG00000154319	FAM167A	-4.5	5.5E-07	2.5E-06	0.163	0.027	0.141	0.194	0.736	0.037	0.704	0.777
ENSG00000254377	MIR124-2HG	-4.4	7.8E-03	1.5E-02	0.045	0.047	0.000	0.093	0.212	0.080	0.121	0.274
ENSG00000197705	KLHL14	-4.4	1.1E-07	5.9E-07	0.226	0.088	0.155	0.325	1.011	0.125	0.901	1.147
ENSG00000163884	KLF15	-4.4	6.5E-20	3.2E-18	2.858	0.350	2.565	3.245	12.604	0.963	11.624	13.550
ENSG00000112541	PDE10A	-4.2	3.0E-09	2.1E-08	0.127	0.046	0.075	0.161	0.539	0.130	0.396	0.652
ENSG00000160179	ABCG1	-4.2	6.8E-17	2.0E-15	1.142	0.285	0.849	1.417	4.859	0.523	4.268	5.258
ENSG00000282164	PEG13	-4.1	2.7E-05	8.9E-05	0.107	0.020	0.086	0.125	0.457	0.148	0.323	0.616
ENSG00000225489		-4	1.7E-06	7.0E-06	0.266	0.083	0.172	0.326	1.093	0.090	1.009	1.189
ENSG00000240409	MTATP8P1	-3.9	2.1E-05	7.0E-05	2.982	1.379	1.737	4.465	12.029	1.652	10.188	13.382
ENSG00000210195	MT-TT	-3.9	4.3E-08	2.5E-07	19.955	5.189	14.032	23.698	79.200	7.776	70.296	84.660
ENSG00000173673	HES3	-3.9	2.7E-08	1.6E-07	6.417	1.645	4.891	8.160	25.519	4.410	22.188	30.520
ENSG00000129538	RNASE1	-3.8	5.2E-03	1.1E-02	0.166	0.148	0.000	0.283	0.679	0.306	0.453	1.027
ENSG00000164708	PGAM2	-3.8	2.1E-08	1.3E-07	1.704	0.705	0.947	2.341	6.536	1.001	5.742	7.661
ENSG00000185811	IKZF1	-3.8	4.4E-10	3.7E-09	0.223	0.021	0.199	0.237	0.860	0.080	0.790	0.948
ENSG00000215186	GOLGA6B	-3.8	2.6E-07	1.2E-06	0.539	0.210	0.368	0.773	2.080	0.204	1.894	2.298
ENSG00000272636	DOC2B	-3.8	1.2E-04	3.4E-04	0.102	0.030	0.072	0.132	0.396	0.107	0.275	0.477
ENSG00000177359		-3.8	1.9E-11	2.1E-10	0.532	0.172	0.368	0.712	1.998	0.033	1.968	2.033
ENSG00000127578	WFIKKN1	-3.7	3.9E-11	4.1E-10	0.696	0.199	0.466	0.823	2.563	0.176	2.460	2.767
ENSG00000184012	TMPRSS2	-3.7	9.4E-15	2.0E-13	0.892	0.153	0.716	1.000	3.343	0.266	3.037	3.522
ENSG00000227199	ST7-AS1	-3.7	1.5E-06	6.3E-06	0.610	0.413	0.163	0.979	2.327	0.119	2.218	2.454
ENSG00000225630	MTND2P28	-3.7	1.4E-18	5.7E-17	329.531	38.197	300.444	372.787	1221.526	54.161	1175.841	1281.356
ENSG00000248527	MTATP6P1	-3.7	2.4E-12	3.2E-11	675.469	89.291	601.002	774.459	2486.823	129.919	2377.806	2630.580
ENSG00000179546	HTR1D	-3.7	1.7E-07	8.8E-07	0.451	0.097	0.351	0.544	1.662	0.353	1.429	2.068
ENSG00000159289	GOLGA6A	-3.7	2.0E-10	1.8E-09	0.747	0.016	0.732	0.763	2.770	0.221	2.517	2.925
ENSG00000145365	TIFA	-3.6	1.2E-11	1.4E-10	0.755	0.131	0.617	0.878	2.743	0.178	2.564	2.920
ENSG00000198899	MT-ATP6	-3.6	7.8E-15	1.7E-13	3807.096	449.159	3501.482	4322.800	13919.328	377.667	13681.242	14355.264
ENSG00000173269	MMRN2	-3.6	3.6E-08	2.1E-07	0.284	0.058	0.231	0.347	1.049	0.135	0.949	1.202
ENSG00000168646	AXIN2	-3.6	2.0E-18	8.1E-17	2.103	0.399	1.739	2.530	7.667	0.298	7.484	8.011
ENSG00000249406		-3.6	4.3E-03	8.9E-03	0.305	0.214	0.169	0.552	1.162	0.302	0.813	1.341
ENSG00000125285	SOX21	-3.5	2.3E-03	5.0E-03	0.147	0.065	0.102	0.222	0.534	0.207	0.352	0.759
ENSG00000174004	NRROS	-3.5	1.0E-12	1.5E-11	1.348	0.168	1.222	1.538	4.750	0.609	4.272	5.436
ENSG00000198938	MT-CO3	-3.5	1.2E-18	4.8E-17	7589.342	771.555	7110.579	8479.406	26475.074	372.922	26062.612	26788.434
ENSG00000170561	IRX2	-3.5	1.9E-08	1.2E-07	0.892	0.493	0.384	1.368	3.202	0.172	3.022	3.365
ENSG00000180447	GAS1	-3.5	1.5E-08	9.1E-08	0.707	0.368	0.382	1.107	2.504	0.089	2.437	2.605
ENSG00000198719	DLL1	-3.5	3.0E-07	1.5E-06	0.378	0.037	0.335	0.403	1.348	0.134	1.212	1.479
ENSG00000227640	SOX21-AS1	-3.4	4.7E-04	1.2E-03	0.179	0.197	0.000	0.389	0.648	0.071	0.581	0.722
ENSG00000163704	PRRT3	-3.4	6.9E-13	1.0E-11	1.357	0.366	1.083	1.772	4.613	0.472	4.326	5.158
ENSG00000115267	IFIH1	-3.4	2.7E-06	1.1E-05	0.291	0.035	0.251	0.314	1.013	0.138	0.873	1.149
ENSG00000168509	HFE2	-3.4	2.1E-03	4.6E-03	0.172	0.102	0.059	0.255	0.594	0.149	0.462	0.756
ENSG00000163873	GRIK3	-3.4	1.2E-10	1.1E-09	0.311	0.045	0.260	0.338	1.057	0.101	0.959	1.160
ENSG00000140478	GOLGA6D	-3.4	1.8E-09	1.4E-08	0.781	0.098	0.689	0.885	2.692	0.137	2.569	2.840
ENSG00000129654	FOXJ1	-3.4	1.5E-11	1.8E-10	1.701	0.264	1.475	1.992	5.780	0.785	4.888	6.400
ENSG00000109846	CRYAB	-3.4	1.3E-09	1.0E-08	0.812	0.240	0.610	1.078	2.774	0.232	2.549	3.011
ENSG00000159212	CLIC6	-3.4	8.7E-10	6.9E-09	0.664	0.130	0.516	0.757	2.241	0.109	2.171	2.367
ENSG00000138311	ZNF365	-3.3	2.0E-04	5.5E-04	0.068	0.035	0.031	0.102	0.230	0.026	0.200	0.248
ENSG00000006555	TTC22	-3.3	3.6E-06	1.4E-05	0.227	0.113	0.102	0.322	0.759	0.207	0.540	0.952
ENSG00000074803	SLC12A1	-3.3	9.8E-06	3.5E-05	0.077	0.027	0.049	0.103	0.263	0.057	0.203	0.317
ENSG00000171759	PAH	-3.3	5.2E-10	4.3E-09	0.476	0.096	0.378	0.570	1.583	0.315	1.346	1.940
ENSG00000198840	MT-ND3	-3.3	4.3E-32	1.9E-29	1359.624	102.991	1288.703	1477.758	4558.510	173.980	4376.203	4722.753
ENSG00000188257	PLA2G2A	-3.3	3.2E-02	5.4E-02	0.087	0.084	0.000	0.169	0.330	0.005	0.324	0.334
ENSG00000198763	MT-ND2	-3.3	8.0E-14	1.4E-12	1648.415	192.778	1496.655	1865.327	5375.894	185.820	5168.980	5528.532
ENSG00000197993	KEL	-3.3	1.4E-08	8.7E-08	0.598	0.047	0.544	0.630	2.008	0.288	1.773	2.329
ENSG00000143119	CD53	-3.3	9.6E-05	2.8E-04	0.417	0.244	0.148	0.624	1.401	0.333	1.081	1.746
ENSG00000250421		-3.3	2.5E-05	8.2E-05	1.258	0.291	0.925	1.466	4.235	1.150	3.433	5.553
ENSG00000075290	WNT8B	-3.2	1.7E-05	5.8E-05	0.548	0.188	0.365	0.741	1.791	0.608	1.257	2.453
ENSG00000136842	TMOD1	-3.2	6.1E-04	1.5E-03	0.162	0.114	0.042	0.268	0.537	0.051	0.478	0.569
ENSG00000258986	TMEM179	-3.2	8.0E-12	9.8E-11	1.026	0.121	0.950	1.166	3.280	0.595	2.741	3.918
ENSG00000103710	RASL12	-3.2	7.3E-19	3.2E-17	5.678	0.505	5.125	6.114	18.183	0.627	17.461	18.595
ENSG00000167195	GOLGA6C	-3.2	5.3E-07	2.4E-06	0.751	0.125	0.676	0.895	2.407	0.246	2.237	2.689
ENSG00000198744	MTCO3P12	-3.1	1.3E-13	2.2E-12	106.680	13.645	97.383	122.346	328.324	11.678	321.169	341.799
ENSG00000198727	MT-CYB	-3.1	4.8E-14	9.1E-13	1377.484	146.556	1252.309	1538.697	4305.913	150.051	4214.825	4479.099
ENSG00000267327		-3.1	2.0E-03	4.5E-03	0.535	0.247	0.342	0.813	1.708	0.375	1.325	2.074
ENSG00000112619	PRPH2	-3	2.9E-06	1.2E-05	0.490	0.179	0.311	0.669	1.492	0.156	1.314	1.604
ENSG00000131203	IDO1	-3	2.9E-18	1.1E-16	9.158	0.162	9.009	9.331	27.540	2.795	25.090	30.585
ENSG00000180767	CHST13	-3	1.9E-07	9.5E-07	1.477	0.900	0.817	2.502	4.398	0.101	4.294	4.497
ENSG00000156966	B3GNT7	-3	9.0E-24	8.4E-22	12.026	0.957	11.202	13.076	36.619	1.555	34.830	37.649
ENSG00000105929	ATP6V0A4	-3	6.7E-06	2.5E-05	0.288	0.076	0.201	0.344	0.895	0.046	0.845	0.935
ENSG00000196767	POU3F4	-2.9	4.1E-05	1.3E-04	0.278	0.131	0.159	0.419	0.825	0.106	0.703	0.901
ENSG00000237973	MTCO1P12	-2.9	1.0E-32	4.8E-30	787.956	42.960	756.429	836.887	2320.023	59.847	2250.952	2356.447
ENSG00000198804	MT-CO1	-2.9	5.4E-33	2.8E-30	6567.687	397.223	6278.296	7020.564	18934.001	507.600	18441.236	19455.239
ENSG00000166963	MAP1A	-2.9	6.4E-10	5.3E-09	0.402	0.136	0.244	0.482	1.202	0.033	1.164	1.222
ENSG00000188828	GLRA4	-2.9	7.9E-03	1.5E-02	0.185	0.058	0.119	0.227	0.561	0.042	0.517	0.601
ENSG00000160097	FNDC5	-2.9	4.8E-04	1.2E-03	0.261	0.044	0.226	0.310	0.751	0.227	0.490	0.896
ENSG00000164488	DACT2	-2.9	6.9E-10	5.6E-09	0.979	0.196	0.804	1.192	2.811	0.435	2.325	3.163
ENSG00000256542		-2.9	1.7E-04	4.7E-04	0.336	0.087	0.254	0.427	0.976	0.243	0.763	1.241
ENSG00000111186	WNT5B	-2.8	2.0E-05	6.7E-05	0.377	0.083	0.302	0.466	1.065	0.167	0.909	1.241
ENSG00000165816	VWA2	-2.8	3.6E-08	2.1E-07	0.815	0.075	0.738	0.888	2.286	0.037	2.257	2.327
ENSG00000182095	TNRC18	-2.8	1.8E-12	2.5E-11	0.673	0.072	0.602	0.746	1.899	0.404	1.444	2.218
ENSG00000172572	PDE3A	-2.8	5.3E-12	6.7E-11	1.095	0.122	0.956	1.179	3.081	0.026	3.064	3.110
ENSG00000142347	MYO1F	-2.8	2.6E-09	1.9E-08	0.512	0.089	0.449	0.614	1.434	0.237	1.160	1.579
ENSG00000212907	MT-ND4L	-2.8	2.2E-25	2.7E-23	4575.667	400.548	4196.002	4994.256	12757.251	480.073	12333.896	13278.845
ENSG00000170382	LRRN2	-2.8	4.9E-11	5.1E-10	1.179	0.084	1.087	1.251	3.297	0.454	2.779	3.624
ENSG00000244342	LINC00688	-2.8	4.1E-13	6.4E-12	2.350	0.040	2.306	2.383	6.606	0.407	6.259	7.054
ENSG00000187715	KBTBD12	-2.8	4.8E-05	1.5E-04	0.190	0.053	0.139	0.244	0.548	0.073	0.504	0.633
ENSG00000157470	FAM81A	-2.8	2.2E-06	8.9E-06	0.330	0.081	0.255	0.415	0.913	0.102	0.832	1.027
ENSG00000183844	FAM3B	-2.8	1.2E-06	4.9E-06	0.540	0.126	0.429	0.677	1.544	0.210	1.406	1.785
ENSG00000177096	FAM109B	-2.8	1.5E-13	2.5E-12	3.150	0.221	2.897	3.307	8.834	0.671	8.093	9.402
ENSG00000215018	COL28A1	-2.8	1.2E-04	3.4E-04	0.144	0.043	0.117	0.193	0.419	0.107	0.295	0.489
ENSG00000187068	C3orf70	-2.8	1.5E-09	1.1E-08	0.705	0.039	0.660	0.732	1.991	0.199	1.763	2.130
ENSG00000154930	ACSS1	-2.8	2.7E-05	9.0E-05	0.147	0.058	0.112	0.213	0.410	0.019	0.388	0.424
ENSG00000260776		-2.8	8.3E-10	6.6E-09	1.409	0.394	0.958	1.688	4.050	0.283	3.758	4.323
ENSG00000162543	UBXN10	-2.7	7.9E-05	2.4E-04	0.267	0.146	0.117	0.408	0.737	0.202	0.555	0.955
ENSG00000187325	TAF8B	-2.7	1.1E-20	5.9E-19	13.525	0.483	13.093	14.047	36.171	2.184	33.650	37.487
ENSG00000126561	STAT5A	-2.7	1.4E-14	3.0E-13	1.883	0.189	1.757	2.100	5.142	0.648	4.540	5.827
ENSG00000177511	ST8SIA3	-2.7	1.6E-15	3.9E-14	1.308	0.137	1.197	1.461	3.524	0.403	3.146	3.947
ENSG00000148942	SLC5A12	-2.7	3.5E-07	1.7E-06	0.345	0.135	0.205	0.473	0.940	0.129	0.795	1.044
ENSG00000140090	SLC24A4	-2.7	3.2E-03	6.7E-03	0.047	0.029	0.021	0.079	0.127	0.032	0.098	0.162
ENSG00000179673	RPRML	-2.7	1.7E-05	6.0E-05	1.438	0.390	0.989	1.691	3.895	0.164	3.706	3.992
ENSG00000125637	PSD4	-2.7	3.0E-06	1.2E-05	0.164	0.028	0.138	0.193	0.437	0.064	0.364	0.477
ENSG00000005421	PON1	-2.7	1.1E-05	3.8E-05	0.572	0.169	0.382	0.703	1.582	0.306	1.340	1.926
ENSG00000154822	PLCL2	-2.7	1.1E-14	2.2E-13	2.812	0.518	2.460	3.407	7.480	0.535	6.863	7.818
ENSG00000229544	NKX1-2	-2.7	7.1E-12	8.8E-11	7.369	0.445	6.997	7.862	19.661	2.065	17.349	21.323
ENSG00000232790	LINC01162	-2.7	6.2E-04	1.5E-03	1.838	0.619	1.381	2.542	5.063	1.183	3.774	6.101
ENSG00000135253	KCP	-2.7	5.1E-07	2.3E-06	0.287	0.050	0.255	0.344	0.776	0.086	0.677	0.826
ENSG00000140968	IRF8	-2.7	3.8E-04	1.0E-03	0.208	0.038	0.179	0.252	0.565	0.116	0.433	0.654
ENSG00000176884	GRIN1	-2.7	8.6E-04	2.1E-03	0.114	0.008	0.106	0.121	0.309	0.016	0.293	0.324
ENSG00000186281	GPAT2	-2.7	2.5E-05	8.2E-05	0.287	0.099	0.228	0.400	0.792	0.248	0.528	1.020
ENSG00000144488	ESPNL	-2.7	7.4E-07	3.3E-06	0.500	0.161	0.345	0.666	1.364	0.091	1.303	1.468
ENSG00000082397	EPB41L3	-2.7	3.8E-20	2.0E-18	2.421	0.094	2.365	2.530	6.472	0.221	6.337	6.727
ENSG00000129173	E2F8	-2.7	8.0E-16	2.0E-14	3.774	0.446	3.333	4.224	10.177	0.518	9.783	10.763
ENSG00000276644	DACH1	-2.7	9.3E-05	2.8E-04	0.229	0.062	0.158	0.271	0.629	0.181	0.430	0.783
ENSG00000130303	BST2	-2.7	3.2E-17	1.0E-15	16.244	0.894	15.587	17.262	43.548	0.896	42.683	44.479
ENSG00000145692	BHMT	-2.7	2.4E-06	9.6E-06	0.681	0.223	0.438	0.877	1.878	0.383	1.492	2.257
ENSG00000228496		-2.7	2.7E-07	1.3E-06	1.999	0.324	1.802	2.373	5.374	0.317	5.010	5.590
ENSG00000162542	TMCO4	-2.6	1.3E-11	1.5E-10	2.128	0.513	1.719	2.703	5.557	0.322	5.306	5.920
ENSG00000110975	SYT10	-2.6	1.9E-06	7.7E-06	0.565	0.152	0.425	0.727	1.473	0.276	1.162	1.690
ENSG00000111671	SPSB2	-2.6	3.5E-15	8.0E-14	5.339	0.267	5.031	5.509	13.983	0.980	12.901	14.812
ENSG00000063176	SPHK2	-2.6	2.9E-17	9.4E-16	6.317	0.637	5.600	6.815	16.620	0.278	16.308	16.838
ENSG00000101276	SLC52A3	-2.6	3.3E-09	2.3E-08	1.317	0.178	1.144	1.499	3.476	0.564	2.888	4.012
ENSG00000152193	RNF219	-2.6	2.6E-18	1.0E-16	6.775	0.441	6.301	7.173	17.953	1.155	16.693	18.960
ENSG00000170854	RIOX2	-2.6	3.8E-17	1.2E-15	4.018	0.727	3.232	4.666	10.520	0.471	10.013	10.944
ENSG00000141314	RHBDL3	-2.6	2.5E-11	2.7E-10	1.911	0.128	1.815	2.056	4.965	0.211	4.787	5.198
ENSG00000186951	PPARA	-2.6	1.6E-13	2.7E-12	0.726	0.062	0.656	0.774	1.889	0.174	1.691	2.015
ENSG00000180287	PLD5	-2.6	1.1E-08	7.0E-08	0.520	0.157	0.340	0.631	1.375	0.336	1.083	1.742
ENSG00000111331	OAS3	-2.6	4.5E-07	2.1E-06	0.344	0.079	0.275	0.430	0.897	0.131	0.750	0.999
ENSG00000111321	LTBR	-2.6	4.2E-09	3.0E-08	0.635	0.117	0.565	0.770	1.659	0.097	1.585	1.769
ENSG00000183908	LRRC55	-2.6	1.6E-09	1.2E-08	0.814	0.088	0.720	0.894	2.175	0.182	1.966	2.298
ENSG00000177508	IRX3	-2.6	1.2E-03	2.7E-03	0.313	0.102	0.223	0.424	0.821	0.277	0.515	1.056
ENSG00000188816	HMX2	-2.6	1.2E-08	7.5E-08	2.843	0.424	2.370	3.191	7.445	0.604	6.778	7.957
ENSG00000152380	FAM151B	-2.6	6.3E-04	1.6E-03	0.310	0.011	0.298	0.320	0.818	0.125	0.678	0.921
ENSG00000134516	DOCK2	-2.6	4.8E-17	1.5E-15	1.334	0.035	1.305	1.373	3.438	0.160	3.258	3.567
ENSG00000214688	C10orf105	-2.6	1.3E-08	8.1E-08	0.747	0.154	0.576	0.876	1.969	0.214	1.756	2.184
ENSG00000204934	ATP6V0E2-AS1	-2.6	4.7E-04	1.2E-03	0.262	0.018	0.247	0.281	0.689	0.088	0.594	0.769
ENSG00000165092	ALDH1A1	-2.6	2.6E-07	1.3E-06	1.034	0.098	0.921	1.098	2.695	0.285	2.369	2.893
ENSG00000253838		-2.6	2.5E-05	8.2E-05	14.622	4.066	11.152	19.096	38.539	3.638	34.459	41.443
ENSG00000276043	UHRF1	-2.5	4.6E-21	2.7E-19	11.810	1.199	10.650	13.044	29.751	1.176	28.446	30.730
ENSG00000152760	TCTEX1D1	-2.5	8.2E-05	2.5E-04	0.580	0.140	0.459	0.733	1.437	0.379	1.056	1.814
ENSG00000108950	FAM20A	-2.5	1.4E-01	2.0E-01	0.023	0.040	0.000	0.069	0.067	0.037	0.045	0.110
ENSG00000143858	SYT2	-2.5	6.2E-21	3.6E-19	6.152	0.059	6.097	6.214	15.669	0.469	15.331	16.204
ENSG00000137872	SEMA6D	-2.5	7.9E-05	2.4E-04	0.193	0.121	0.057	0.289	0.503	0.020	0.490	0.526
ENSG00000115884	SDC1	-2.5	5.4E-22	3.9E-20	18.962	0.637	18.557	19.697	46.697	1.770	44.661	47.869
ENSG00000153246	PLA2R1	-2.5	4.0E-08	2.3E-07	0.239	0.067	0.190	0.315	0.612	0.054	0.551	0.646
ENSG00000165495	PKNOX2	-2.5	4.7E-05	1.5E-04	0.251	0.078	0.174	0.331	0.631	0.114	0.501	0.710
ENSG00000140538	NTRK3	-2.5	1.0E-08	6.6E-08	0.150	0.026	0.127	0.179	0.379	0.037	0.336	0.402
ENSG00000167034	NKX3-1	-2.5	3.2E-03	6.7E-03	0.400	0.270	0.094	0.609	1.009	0.450	0.625	1.504
ENSG00000187566	NHLRC1	-2.5	2.0E-05	6.7E-05	0.783	0.199	0.650	1.012	2.016	0.156	1.876	2.184
ENSG00000123405	NFE2	-2.5	7.2E-04	1.8E-03	0.450	0.063	0.394	0.518	1.141	0.464	0.626	1.528
ENSG00000198786	MT-ND5	-2.5	7.4E-21	4.3E-19	1046.892	104.283	943.385	1151.934	2593.274	40.921	2549.714	2630.910
ENSG00000198888	MT-ND1	-2.5	4.0E-29	8.9E-27	1272.820	102.470	1184.740	1385.281	3240.128	58.098	3174.352	3284.437
ENSG00000135324	MRAP2	-2.5	5.8E-13	8.7E-12	4.204	0.298	3.871	4.448	10.755	0.623	10.197	11.428
ENSG00000066382	MPPED2	-2.5	2.3E-08	1.4E-07	0.592	0.120	0.471	0.711	1.506	0.245	1.244	1.730
ENSG00000142961	MOB3C	-2.5	3.3E-11	3.6E-10	1.227	0.182	1.018	1.346	3.067	0.061	3.022	3.137
ENSG00000145794	MEGF10	-2.5	4.7E-21	2.8E-19	4.801	0.479	4.250	5.118	12.204	0.407	11.763	12.565
ENSG00000154655	L3MBTL4	-2.5	1.6E-05	5.5E-05	0.295	0.028	0.272	0.326	0.751	0.179	0.579	0.936
ENSG00000049283	EPN3	-2.5	1.6E-07	8.2E-07	0.699	0.102	0.594	0.798	1.763	0.189	1.595	1.968
ENSG00000138161	CUZD1	-2.5	9.2E-15	1.9E-13	3.907	0.213	3.698	4.124	9.621	0.422	9.254	10.081
ENSG00000214290	COLCA2	-2.5	1.4E-04	3.9E-04	0.745	0.395	0.483	1.199	1.890	0.619	1.289	2.526
ENSG00000226530		-2.5	1.8E-04	5.0E-04	1.632	0.144	1.468	1.737	4.130	0.228	3.891	4.347
ENSG00000249574		-2.5	5.0E-03	1.0E-02	0.385	0.163	0.199	0.503	0.954	0.501	0.381	1.310
ENSG00000253706		-2.5	1.7E-03	3.9E-03	0.288	0.220	0.128	0.539	0.726	0.223	0.472	0.890
ENSG00000275807		-2.5	1.6E-02	2.9E-02	0.303	0.119	0.185	0.422	0.752	0.061	0.709	0.822
ENSG00000256663		-2.5	1.1E-16	3.2E-15	18.929	2.214	16.975	21.333	46.735	1.862	44.682	48.313
ENSG00000196881	WDR5B	-2.4	5.1E-11	5.3E-10	2.208	0.427	1.784	2.637	5.246	0.179	5.040	5.367
ENSG00000088992	TESC	-2.4	6.7E-08	3.7E-07	1.994	0.465	1.683	2.528	4.875	0.339	4.569	5.239
ENSG00000149256	TENM4	-2.4	1.7E-10	1.6E-09	0.771	0.145	0.626	0.914	1.829	0.221	1.617	2.058
ENSG00000182747	SLC35D3	-2.4	3.6E-03	7.5E-03	0.339	0.138	0.206	0.482	0.843	0.218	0.592	0.983
ENSG00000268883	PNMA6B	-2.4	4.9E-04	1.2E-03	0.998	0.251	0.829	1.286	2.376	0.342	1.989	2.636
ENSG00000101198	NKAIN4	-2.4	5.0E-06	1.9E-05	0.914	0.066	0.852	0.983	2.184	0.250	1.897	2.351
ENSG00000198886	MT-ND4	-2.4	1.0E-17	3.7E-16	3956.438	415.056	3646.762	4428.052	9695.282	318.342	9499.879	10062.505
ENSG00000134042	MRO	-2.4	9.5E-07	4.2E-06	0.691	0.079	0.643	0.782	1.639	0.362	1.221	1.851
ENSG00000225077	LINC00337	-2.4	3.6E-06	1.4E-05	1.906	0.417	1.603	2.382	4.532	0.326	4.255	4.891
ENSG00000166816	LDHD	-2.4	4.4E-05	1.4E-04	0.749	0.116	0.619	0.844	1.826	0.083	1.734	1.895
ENSG00000176387	HSD11B2	-2.4	8.6E-16	2.2E-14	8.250	0.772	7.446	8.986	19.685	1.303	18.702	21.163
ENSG00000103044	HAS3	-2.4	6.8E-22	4.8E-20	19.832	1.079	18.823	20.969	47.048	0.720	46.589	47.878
ENSG00000185614	FAM212A	-2.4	5.0E-05	1.6E-04	1.801	0.538	1.187	2.187	4.322	0.465	3.802	4.701
ENSG00000007968	E2F2	-2.4	6.0E-12	7.6E-11	2.130	0.290	1.844	2.423	5.071	0.247	4.787	5.230
ENSG00000164733	CTSB	-2.4	8.8E-19	3.7E-17	4.773	0.382	4.468	5.201	11.369	0.530	11.039	11.981
ENSG00000158270	COLEC12	-2.4	5.1E-08	2.8E-07	0.720	0.154	0.542	0.820	1.709	0.303	1.370	1.954
ENSG00000204936	CD177	-2.4	1.5E-05	5.1E-05	0.807	0.145	0.710	0.974	1.972	0.372	1.745	2.402
ENSG00000234511	C5orf58	-2.4	5.7E-04	1.4E-03	0.331	0.104	0.242	0.446	0.811	0.121	0.700	0.940
ENSG00000183186	C2CD4C	-2.4	2.6E-08	1.5E-07	1.492	0.310	1.135	1.681	3.534	0.179	3.348	3.704
ENSG00000159403	C1R	-2.4	7.8E-11	7.7E-10	1.325	0.034	1.288	1.354	3.133	0.274	2.851	3.399
ENSG00000141431	ASXL3	-2.4	6.3E-05	1.9E-04	0.171	0.059	0.116	0.233	0.419	0.124	0.313	0.555
ENSG00000272734	ADIRF-AS1	-2.4	4.0E-04	1.0E-03	0.276	0.159	0.107	0.424	0.652	0.153	0.538	0.826
ENSG00000226816		-2.4	2.3E-06	9.4E-06	1.359	0.212	1.189	1.597	3.258	0.511	2.951	3.848
ENSG00000273489		-2.4	4.0E-03	8.3E-03	0.251	0.179	0.131	0.457	0.633	0.172	0.490	0.824
ENSG00000275155		-2.4	1.9E-03	4.2E-03	1.534	1.196	0.694	2.903	3.692	0.398	3.326	4.115
ENSG00000165156	ZHX1	-2.3	6.9E-10	5.6E-09	1.497	0.252	1.276	1.772	3.444	0.552	2.953	4.041
ENSG00000070388	FGF22	-2.3	1.3E-02	2.4E-02	0.222	0.063	0.154	0.278	0.510	0.190	0.295	0.654
ENSG00000093217	XYLB	-2.3	1.3E-06	5.6E-06	0.701	0.190	0.516	0.896	1.639	0.160	1.455	1.737
ENSG00000092470	WDR76	-2.3	6.0E-17	1.8E-15	7.967	0.298	7.721	8.298	18.611	0.394	18.169	18.926
ENSG00000174599	TRAM1L1	-2.3	3.5E-06	1.4E-05	2.008	0.723	1.444	2.823	4.563	1.120	3.370	5.593
ENSG00000136205	TNS3	-2.3	8.3E-15	1.8E-13	3.441	0.436	3.041	3.905	7.826	0.153	7.661	7.964
ENSG00000137842	TMEM62	-2.3	2.8E-06	1.1E-05	0.633	0.023	0.607	0.650	1.480	0.168	1.365	1.673
ENSG00000249242	TMEM150C	-2.3	3.4E-09	2.4E-08	1.604	0.120	1.472	1.704	3.653	0.391	3.217	3.975
ENSG00000101463	SYNDIG1	-2.3	8.7E-06	3.1E-05	1.120	0.276	0.813	1.348	2.546	0.557	2.074	3.161
ENSG00000165025	SYK	-2.3	4.5E-09	3.1E-08	1.528	0.379	1.090	1.748	3.557	0.425	3.288	4.048
ENSG00000173705	SUSD5	-2.3	1.9E-09	1.4E-08	1.813	0.338	1.533	2.188	4.165	0.328	3.874	4.520
ENSG00000107821	KAZALD1	-2.3	7.8E-02	1.2E-01	0.085	0.046	0.043	0.135	0.205	0.110	0.077	0.272
ENSG00000269404	SPIB	-2.3	1.3E-08	8.3E-08	1.499	0.248	1.213	1.661	3.417	0.206	3.281	3.655
ENSG00000080493	SLC4A4	-2.3	9.9E-07	4.3E-06	0.334	0.035	0.294	0.361	0.780	0.056	0.737	0.844
ENSG00000160190	SLC37A1	-2.3	2.1E-17	7.1E-16	4.422	0.329	4.100	4.758	10.319	0.116	10.186	10.399
ENSG00000258545	RHOXF1-AS1	-2.3	1.7E-04	4.7E-04	0.715	0.311	0.371	0.976	1.651	0.146	1.512	1.803
ENSG00000162407	PLPP3	-2.3	6.8E-20	3.4E-18	9.522	1.055	8.614	10.679	22.194	0.673	21.669	22.953
ENSG00000081277	PKP1	-2.3	5.9E-06	2.2E-05	0.441	0.043	0.392	0.472	1.034	0.172	0.905	1.229
ENSG00000169026	MFSD7	-2.3	1.9E-06	8.0E-06	1.102	0.220	0.934	1.351	2.538	0.101	2.430	2.630
ENSG00000076770	MBNL3	-2.3	6.4E-12	8.0E-11	1.091	0.222	0.918	1.341	2.525	0.174	2.336	2.679
ENSG00000280734	LINC01232	-2.3	6.9E-07	3.1E-06	0.496	0.028	0.473	0.527	1.171	0.101	1.073	1.276
ENSG00000135480	KRT7	-2.3	1.8E-08	1.1E-07	1.491	0.119	1.358	1.586	3.412	0.338	3.174	3.798
ENSG00000066735	KIF26A	-2.3	2.2E-20	1.2E-18	15.114	0.567	14.512	15.637	34.270	2.263	32.151	36.654
ENSG00000177807	KCNJ10	-2.3	5.2E-05	1.6E-04	0.345	0.103	0.238	0.444	0.796	0.122	0.706	0.935
ENSG00000174498	IGDCC3	-2.3	1.5E-21	1.0E-19	21.281	1.838	19.208	22.713	48.700	3.641	44.609	51.583
ENSG00000134317	GRHL1	-2.3	6.5E-11	6.5E-10	1.137	0.161	1.041	1.323	2.693	0.193	2.564	2.915
ENSG00000227811	FAM212B-AS1	-2.3	2.8E-05	9.1E-05	1.502	0.645	0.762	1.935	3.454	0.439	3.015	3.893
ENSG00000165475	CRYL1	-2.3	5.7E-05	1.8E-04	0.933	0.208	0.712	1.125	2.169	0.454	1.665	2.544
ENSG00000105205	CLC	-2.3	1.5E-04	4.2E-04	3.240	1.053	2.045	4.030	7.519	1.135	6.228	8.359
ENSG00000101204	CHRNA4	-2.3	5.1E-05	1.6E-04	0.210	0.053	0.154	0.257	0.490	0.077	0.405	0.555
ENSG00000105173	CCNE1	-2.3	2.4E-20	1.3E-18	25.351	2.190	22.852	26.932	59.325	4.047	55.054	63.101
ENSG00000185306	C12orf56	-2.3	2.8E-08	1.7E-07	1.090	0.147	0.925	1.209	2.491	0.095	2.433	2.601
ENSG00000164061	BSN	-2.3	1.0E-12	1.4E-11	0.771	0.101	0.660	0.856	1.773	0.097	1.681	1.874
ENSG00000161267	BDH1	-2.3	6.6E-13	9.8E-12	1.790	0.389	1.407	2.185	4.115	0.178	3.950	4.304
ENSG00000248966	BCLAF1P1	-2.3	7.4E-05	2.2E-04	0.742	0.167	0.629	0.934	1.680	0.303	1.338	1.918
ENSG00000254876		-2.3	5.6E-04	1.4E-03	0.353	0.094	0.292	0.461	0.838	0.117	0.717	0.950
ENSG00000272604		-2.3	4.6E-04	1.2E-03	0.506	0.144	0.378	0.662	1.204	0.139	1.111	1.363
ENSG00000277639		-2.3	2.1E-05	7.1E-05	1.795	0.976	0.778	2.725	4.132	0.209	3.970	4.367
ENSG00000156925	ZIC3	-2.2	6.1E-11	6.2E-10	16.974	2.007	15.039	19.045	37.754	2.240	35.508	39.988
ENSG00000136367	ZFHX2	-2.2	6.6E-17	2.0E-15	3.953	0.504	3.430	4.436	8.939	0.566	8.427	9.546
ENSG00000255690	TRIL	-2.2	9.1E-11	8.9E-10	2.306	0.055	2.252	2.363	4.983	0.616	4.559	5.689
ENSG00000135048	TMEM2	-2.2	7.8E-23	6.4E-21	16.782	1.107	15.635	17.845	37.181	1.033	36.425	38.358
ENSG00000137462	TLR2	-2.2	4.3E-05	1.4E-04	0.558	0.122	0.474	0.698	1.213	0.048	1.169	1.264
ENSG00000198959	TGM2	-2.2	2.7E-05	9.0E-05	0.328	0.025	0.303	0.354	0.732	0.166	0.611	0.921
ENSG00000143502	SUSD4	-2.2	7.2E-08	3.9E-07	1.174	0.093	1.068	1.245	2.637	0.303	2.287	2.817
ENSG00000197471	SPN	-2.2	4.0E-07	1.9E-06	0.531	0.139	0.396	0.674	1.210	0.110	1.105	1.325
ENSG00000164651	SP8	-2.2	2.6E-10	2.3E-09	2.110	0.245	1.868	2.358	4.727	0.492	4.160	5.034
ENSG00000185404	SP140L	-2.2	2.2E-03	4.9E-03	0.196	0.027	0.172	0.225	0.443	0.119	0.346	0.577
ENSG00000080503	SMARCA2	-2.2	6.6E-13	9.8E-12	1.046	0.091	0.943	1.116	2.284	0.113	2.200	2.413
ENSG00000110436	SLC1A2	-2.2	2.3E-04	6.3E-04	0.199	0.110	0.127	0.325	0.447	0.152	0.353	0.623
ENSG00000188488	SERPINA5	-2.2	8.3E-09	5.5E-08	1.535	0.051	1.505	1.595	3.386	0.295	3.179	3.724
ENSG00000235663	SAPCD1-AS1	-2.2	2.4E-03	5.2E-03	2.628	0.875	1.674	3.393	5.833	0.953	4.733	6.404
ENSG00000178222	RNF212	-2.2	3.6E-04	9.4E-04	0.406	0.105	0.324	0.524	0.889	0.112	0.763	0.977
ENSG00000197291	RAMP2-AS1	-2.2	1.9E-07	9.7E-07	0.964	0.102	0.852	1.051	2.166	0.095	2.061	2.245
ENSG00000235961	PNMA6A	-2.2	1.6E-05	5.6E-05	1.438	0.396	1.139	1.887	3.230	0.501	2.840	3.794
ENSG00000258429	PDF	-2.2	3.2E-05	1.1E-04	6.285	1.516	4.626	7.597	13.689	1.161	12.666	14.955
ENSG00000188389	PDCD1	-2.2	4.4E-04	1.1E-03	0.798	0.178	0.657	0.998	1.773	0.040	1.729	1.806
ENSG00000137843	PAK6	-2.2	5.4E-06	2.0E-05	0.505	0.060	0.462	0.574	1.095	0.176	0.914	1.267
ENSG00000162881	OXER1	-2.2	6.3E-03	1.3E-02	0.480	0.180	0.273	0.607	1.064	0.185	0.863	1.225
ENSG00000115507	OTX1	-2.2	3.3E-03	7.0E-03	0.252	0.053	0.192	0.294	0.557	0.117	0.423	0.640
ENSG00000144852	NR1I2	-2.2	1.1E-07	5.6E-07	0.884	0.112	0.754	0.951	2.004	0.088	1.933	2.103
ENSG00000183486	MX2	-2.2	3.3E-08	1.9E-07	0.919	0.305	0.592	1.194	2.003	0.168	1.852	2.184
ENSG00000151224	MAT1A	-2.2	2.3E-11	2.6E-10	2.547	0.086	2.463	2.635	5.620	0.230	5.413	5.868
ENSG00000061337	LZTS1	-2.2	1.2E-14	2.4E-13	3.631	0.079	3.556	3.713	8.113	0.674	7.565	8.866
ENSG00000150556	LYPD6B	-2.2	2.2E-06	6.1E-06	0.547	0.175	0.344	0.655	1.204	0.161	1.049	1.371
ENSG00000128594	LRRC4	-2.2	9.0E-11	8.8E-10	3.445	0.702	2.936	4.245	7.547	0.736	6.926	8.360
ENSG00000183722	LHFP	-2.2	3.5E-09	2.5E-08	3.293	0.199	3.069	3.449	7.126	0.533	6.563	7.624
ENSG00000129455	KLK8	-2.2	2.7E-04	7.3E-04	0.907	0.317	0.610	1.241	1.961	0.068	1.901	2.035
ENSG00000115041	KCNIP3	-2.2	6.9E-04	1.7E-03	0.227	0.075	0.181	0.314	0.500	0.067	0.433	0.568
ENSG00000182132	KCNIP1	-2.2	2.6E-03	5.7E-03	0.581	0.298	0.276	0.872	1.290	0.496	0.773	1.762
ENSG00000158764	ITLN2	-2.2	1.2E-08	7.7E-08	4.668	0.505	4.151	5.159	10.349	1.492	8.851	11.836
ENSG00000145681	HAPLN1	-2.2	2.5E-05	8.3E-05	0.426	0.147	0.257	0.511	0.972	0.107	0.884	1.091
ENSG00000185652	NTF3	-2.2	1.4E-02	2.5E-02	0.283	0.200	0.106	0.500	0.620	0.177	0.467	0.814
ENSG00000149557	FEZ1	-2.2	4.8E-14	9.1E-13	2.845	0.108	2.754	2.964	6.361	0.415	5.882	6.631
ENSG00000183690	EFHC2	-2.2	1.5E-08	9.5E-08	1.949	0.264	1.674	2.201	4.268	0.083	4.209	4.363
ENSG00000146122	DAAM2	-2.2	1.8E-10	1.7E-09	0.726	0.032	0.702	0.763	1.584	0.124	1.499	1.726
ENSG00000111012	CYP27B1	-2.2	6.8E-03	1.3E-02	0.465	0.315	0.179	0.802	0.997	0.154	0.832	1.137
ENSG00000184371	CSF1	-2.2	4.0E-07	1.9E-06	0.804	0.177	0.604	0.940	1.749	0.230	1.588	2.013
ENSG00000184887	BTBD6	-2.2	1.5E-11	1.8E-10	5.759	0.296	5.420	5.972	12.565	1.151	11.638	13.853
ENSG00000163141	BNIPL	-2.2	9.4E-06	3.4E-05	1.014	0.116	0.898	1.131	2.242	0.517	1.739	2.771
ENSG00000122870	BICC1	-2.2	2.5E-05	8.2E-05	0.413	0.130	0.329	0.563	0.937	0.082	0.856	1.021
ENSG00000198768	APCDD1L	-2.2	1.8E-07	9.1E-07	1.333	0.283	1.057	1.623	3.014	0.391	2.729	3.459
ENSG00000184378	ACTRT3	-2.2	8.7E-09	5.7E-08	3.404	0.505	3.002	3.971	7.606	1.188	6.897	8.977
ENSG00000256802		-2.2	2.9E-03	6.3E-03	0.554	0.276	0.297	0.846	1.213	0.463	0.771	1.695
ENSG00000259969		-2.2	4.3E-04	1.1E-03	1.460	0.287	1.160	1.731	3.253	0.461	2.723	3.560
ENSG00000283638		-2.2	7.7E-06	2.8E-05	0.794	0.205	0.599	1.008	1.789	0.371	1.377	2.097
ENSG00000284048		-2.2	4.7E-09	3.2E-08	4.377	0.631	3.942	5.101	9.637	0.249	9.356	9.830
ENSG00000180787	ZFP3	-2.1	1.6E-03	3.6E-03	0.305	0.060	0.267	0.374	0.631	0.042	0.590	0.675
ENSG00000233608	TWIST2	-2.1	5.0E-03	1.0E-02	0.724	0.162	0.605	0.909	1.577	0.187	1.422	1.785
ENSG00000235890	TSPEAR-AS1	-2.1	3.3E-04	8.6E-04	0.710	0.067	0.655	0.785	1.534	0.120	1.448	1.672
ENSG00000171227	TMEM37	-2.1	5.7E-12	7.1E-11	6.581	0.690	5.807	7.134	13.808	0.796	12.921	14.457
ENSG00000196832	TMEM26	-2.1	2.1E-05	7.1E-05	0.773	0.143	0.682	0.939	1.669	0.173	1.476	1.812
ENSG00000179886	TIGD5	-2.1	1.3E-08	8.1E-08	1.597	0.120	1.477	1.718	3.298	0.388	2.903	3.678
ENSG00000197165	SULT1A2	-2.1	1.2E-03	2.8E-03	0.639	0.018	0.624	0.659	1.342	0.211	1.195	1.584
ENSG00000185482	STAC3	-2.1	6.9E-04	1.7E-03	0.825	0.023	0.810	0.852	1.721	0.405	1.274	2.066
ENSG00000147059	SPIN2A	-2.1	1.4E-07	7.4E-07	1.717	0.191	1.500	1.862	3.713	0.207	3.538	3.942
ENSG00000113504	SLC12A7	-2.1	1.6E-17	5.4E-16	10.124	1.198	9.196	11.477	21.506	0.420	21.239	21.990
ENSG00000102032	RENBP	-2.1	1.7E-08	1.0E-07	3.274	0.773	2.401	3.871	6.883	0.199	6.688	7.093
ENSG00000155846	PPARGC1B	-2.1	8.7E-16	2.2E-14	1.984	0.106	1.864	2.060	4.266	0.091	4.162	4.327
ENSG00000100413	POLR3H	-2.1	2.8E-16	7.7E-15	5.719	0.140	5.563	5.834	11.959	0.313	11.599	12.160
ENSG00000183837	PNMA3	-2.1	1.1E-09	8.9E-09	3.006	0.380	2.677	3.421	6.258	0.420	5.777	6.549
ENSG00000141934	PLPP2	-2.1	1.2E-15	3.1E-14	4.612	0.157	4.438	4.745	9.555	0.817	8.667	10.277
ENSG00000161270	NPHS1	-2.1	2.1E-03	4.7E-03	0.251	0.012	0.238	0.260	0.535	0.171	0.384	0.721
ENSG00000109255	NMU	-2.1	2.0E-11	2.2E-10	21.460	0.714	20.738	22.167	45.617	4.125	41.258	49.460
ENSG00000162576	MXRA8	-2.1	1.9E-07	9.4E-07	1.728	0.353	1.335	2.017	3.549	0.396	3.162	3.953
ENSG00000199102	MIR302C	-2.1	7.6E-05	2.3E-04	33.641	0.353	33.419	34.048	72.764	17.543	53.756	88.332
ENSG00000141639	MAPK4	-2.1	9.8E-08	5.2E-07	0.952	0.167	0.769	1.097	1.888	0.323	1.727	2.350
ENSG00000162931	TRIM17	-2.1	3.0E-02	5.1E-02	0.244	0.029	0.218	0.274	0.507	0.320	0.159	0.787
ENSG00000163286	ALPPL2	-2.1	7.4E-02	1.1E-01	0.165	0.055	0.124	0.228	0.352	0.061	0.282	0.392
ENSG00000263146	LINC01896	-2.1	7.6E-06	2.8E-05	1.903	0.137	1.782	2.052	3.883	0.460	3.549	4.465
ENSG00000232229	LINC00865	-2.1	6.0E-05	1.8E-04	0.714	0.013	0.699	0.723	1.542	0.364	1.181	1.909
ENSG00000132321	IQCA1	-2.1	1.9E-07	9.3E-07	1.465	0.347	1.114	1.809	3.103	0.573	2.447	3.507
ENSG00000110944	IL23A	-2.1	9.1E-03	1.7E-02	0.892	0.317	0.547	1.170	1.876	0.865	1.177	2.843
ENSG00000186603	HPDL	-2.1	1.0E-14	2.2E-13	26.584	1.999	24.527	28.520	54.582	5.374	50.058	60.522
ENSG00000064393	HIPK2	-2.1	4.9E-19	2.2E-17	6.140	0.199	6.013	6.370	12.697	0.758	11.885	13.387
ENSG00000182218	HHIPL1	-2.1	9.4E-08	5.0E-07	0.636	0.039	0.591	0.663	1.371	0.150	1.266	1.543
ENSG00000163909	HEYL	-2.1	3.1E-08	1.8E-07	1.572	0.247	1.398	1.855	3.377	0.086	3.278	3.434
ENSG00000175697	GPR156	-2.1	7.3E-06	2.7E-05	0.645	0.217	0.482	0.892	1.382	0.201	1.153	1.531
ENSG00000181938	GINS3	-2.1	7.7E-14	1.4E-12	4.931	0.224	4.747	5.181	10.423	0.841	9.723	11.356
ENSG00000135414	GDF11	-2.1	1.4E-14	2.9E-13	4.671	0.336	4.463	5.059	9.800	0.769	9.310	10.686
ENSG00000147234	FRMPD3	-2.1	2.1E-05	7.2E-05	0.349	0.011	0.337	0.357	0.743	0.037	0.702	0.774
ENSG00000188001	TPRG1	-2.1	1.5E-02	2.7E-02	0.080	0.005	0.075	0.085	0.170	0.046	0.129	0.220
ENSG00000100350	FOXRED2	-2.1	1.6E-11	1.9E-10	6.494	0.403	6.130	6.927	13.863	0.908	13.096	14.866
ENSG00000151876	FBXO4	-2.1	2.7E-03	5.9E-03	0.425	0.083	0.330	0.485	0.875	0.091	0.793	0.973
ENSG00000151617	EDNRA	-2.1	2.3E-07	1.1E-06	1.613	0.200	1.457	1.838	3.336	0.596	2.886	4.014
ENSG00000163840	DTX3L	-2.1	4.9E-11	5.1E-10	2.596	0.403	2.183	2.987	5.359	0.343	4.996	5.678
ENSG00000110148	CCKBR	-2.1	1.5E-07	7.9E-07	3.612	0.612	2.935	4.124	7.782	0.668	7.139	8.473
ENSG00000205363	C15orf59	-2.1	6.8E-10	5.6E-09	1.672	0.332	1.479	2.055	3.558	0.233	3.340	3.804
ENSG00000206190	ATP10A	-2.1	4.6E-05	1.4E-04	0.345	0.019	0.330	0.367	0.714	0.133	0.588	0.859
ENSG00000172379	ARNT2	-2.1	1.3E-12	1.9E-11	1.877	0.058	1.814	1.929	3.880	0.306	3.645	4.245
ENSG00000127220	ABHD8	-2.1	2.0E-11	2.2E-10	7.367	0.238	7.194	7.639	15.176	1.426	13.590	16.351
ENSG00000242193		-2.1	1.3E-07	6.6E-07	1.019	0.135	0.865	1.115	2.190	0.185	2.018	2.386
ENSG00000260793		-2.1	6.1E-02	9.5E-02	0.202	0.045	0.153	0.242	0.437	0.033	0.417	0.474
ENSG00000260136		-2.1	6.4E-04	1.6E-03	2.154	0.384	1.773	2.542	4.550	1.046	3.630	5.687
ENSG00000261884		-2.1	5.3E-03	1.1E-02	0.254	0.124	0.114	0.350	0.550	0.049	0.504	0.602
ENSG00000283692		-2.1	3.3E-02	5.5E-02	0.281	0.167	0.178	0.473	0.601	0.348	0.341	0.996
ENSG00000144331	ZNF385B	-2	1.3E-04	3.7E-04	0.728	0.005	0.723	0.734	1.430	0.299	1.105	1.695
ENSG00000075702	WDR62	-2	7.7E-16	2.0E-14	7.361	0.579	6.808	7.964	14.570	1.022	13.404	15.317
ENSG00000261373	VPS9D1-AS1	-2	1.2E-08	7.5E-08	6.568	1.546	5.019	8.111	13.158	0.818	12.213	13.634
ENSG00000092445	TYRO3	-2	8.2E-19	3.5E-17	15.349	0.147	15.180	15.446	31.342	1.729	29.744	33.178
ENSG00000005379	TSPOAP1	-2	3.0E-08	1.8E-07	0.672	0.102	0.558	0.751	1.329	0.090	1.251	1.427
ENSG00000175274	TP53I11	-2	4.5E-15	1.0E-13	7.561	0.288	7.240	7.796	15.186	0.772	14.492	16.021
ENSG00000157111	TMEM171	-2	4.9E-03	9.9E-03	0.863	0.384	0.634	1.307	1.719	0.028	1.688	1.740
ENSG00000005108	THSD7A	-2	7.2E-04	1.8E-03	0.204	0.015	0.193	0.221	0.407	0.072	0.324	0.450
ENSG00000165730	STOX1	-2	8.3E-08	4.5E-07	2.126	0.244	1.911	2.391	4.238	0.387	3.822	4.586
ENSG00000137877	SPTBN5	-2	3.0E-05	9.8E-05	0.286	0.051	0.242	0.342	0.578	0.061	0.508	0.615
ENSG00000238062	SPATA3-AS1	-2	2.0E-03	4.4E-03	0.388	0.138	0.251	0.527	0.790	0.050	0.738	0.838
ENSG00000168077	SCARA3	-2	2.8E-12	3.8E-11	5.241	0.413	4.953	5.714	10.322	0.306	9.980	10.568
ENSG00000101347	SAMHD1	-2	1.4E-17	4.9E-16	12.004	0.629	11.341	12.592	24.205	0.639	23.629	24.893
ENSG00000123570	RAB9B	-2	3.7E-04	9.6E-04	0.666	0.025	0.645	0.693	1.373	0.372	1.118	1.801
ENSG00000196090	PTPRT	-2	7.6E-08	4.1E-07	0.581	0.035	0.552	0.620	1.143	0.108	1.058	1.264
ENSG00000143801	PSEN2	-2	2.5E-13	4.2E-12	6.749	0.566	6.316	7.390	13.700	0.537	13.377	14.319
ENSG00000146070	PLA2G7	-2	4.0E-08	2.3E-07	3.716	0.333	3.352	4.006	7.587	0.307	7.336	7.929
ENSG00000174516	PELI3	-2	1.6E-08	1.0E-07	2.253	0.197	2.109	2.477	4.576	0.182	4.376	4.732
ENSG00000138496	PARP9	-2	2.6E-07	1.3E-06	0.733	0.052	0.673	0.764	1.488	0.126	1.343	1.573
ENSG00000173193	PARP14	-2	1.7E-17	5.7E-16	5.060	0.213	4.918	5.305	10.393	0.320	10.036	10.654
ENSG00000171246	NPTX1	-2	2.2E-09	1.6E-08	2.568	0.172	2.397	2.740	5.234	0.126	5.139	5.377
ENSG00000089250	NOS1	-2	2.8E-07	1.3E-06	0.540	0.039	0.506	0.582	1.075	0.180	0.894	1.254
ENSG00000100503	NIN	-2	1.3E-16	3.9E-15	3.249	0.053	3.211	3.310	6.374	0.380	5.940	6.648
ENSG00000183091	NEB	-2	1.0E-11	1.2E-10	0.546	0.031	0.526	0.581	1.098	0.025	1.080	1.127
ENSG00000007944	MYLIP	-2	1.3E-17	4.6E-16	20.274	2.079	17.964	21.996	41.451	1.777	39.401	42.556
ENSG00000225972	MTND1P23	-2	1.2E-14	2.5E-13	73.779	4.102	69.234	77.209	150.836	11.560	137.935	160.251
ENSG00000157227	MMP14	-2	1.5E-22	1.2E-20	43.408	1.767	41.743	45.262	88.214	4.473	84.739	93.260
ENSG00000168404	MLKL	-2	3.0E-07	1.5E-06	1.293	0.328	1.003	1.649	2.579	0.256	2.396	2.871
ENSG00000199145	MIR302D	-2	1.7E-06	7.1E-06	74.607	4.577	70.365	79.457	150.712	5.367	145.761	156.416
ENSG00000128011	LRFN1	-2	3.1E-07	1.5E-06	2.362	0.372	2.054	2.776	4.686	0.905	4.090	5.727
ENSG00000048540	LMO3	-2	1.1E-11	1.3E-10	2.238	0.176	2.039	2.375	4.494	0.388	4.130	4.902
ENSG00000215241	LINC02449	-2	8.5E-04	2.1E-03	1.384	0.217	1.215	1.628	2.782	0.568	2.127	3.120
ENSG00000261455	LINC01003	-2	6.7E-03	1.3E-02	0.760	0.299	0.564	1.104	1.521	0.567	0.871	1.913
ENSG00000258609	LINC-ROR	-2	1.7E-03	3.7E-03	0.784	0.171	0.642	0.974	1.565	0.239	1.383	1.835
ENSG00000138030	KHK	-2	5.5E-11	5.6E-10	5.129	0.457	4.721	5.622	10.068	0.588	9.649	10.740
ENSG00000106012	IQCE	-2	4.2E-09	2.9E-08	1.088	0.164	0.984	1.277	2.234	0.076	2.161	2.313
ENSG00000148357	HMCN2	-2	3.0E-11	3.2E-10	0.927	0.133	0.775	1.020	1.872	0.088	1.777	1.949
ENSG00000162399	BSND	-2	2.1E-02	3.7E-02	0.278	0.086	0.178	0.328	0.573	0.113	0.443	0.648
ENSG00000185477	GPRIN3	-2	9.2E-07	4.0E-06	0.450	0.111	0.327	0.544	0.887	0.124	0.744	0.965
ENSG00000169258	GPRIN1	-2	3.9E-12	5.1E-11	6.080	0.715	5.301	6.707	12.403	0.621	11.975	13.115
ENSG00000176873	FAM89B	-2	2.5E-04	6.7E-04	1.598	0.238	1.336	1.800	3.240	0.430	2.989	3.737
ENSG00000142530	FAM71E1	-2	8.7E-06	3.1E-05	2.337	0.062	2.267	2.384	4.636	0.518	4.103	5.138
ENSG00000139438	FAM222A	-2	1.0E-03	2.4E-03	0.464	0.043	0.415	0.497	0.920	0.054	0.869	0.976
ENSG00000197852	FAM212B	-2	2.1E-15	5.0E-14	2.747	0.189	2.554	2.932	5.535	0.123	5.396	5.632
ENSG00000144649	FAM198A	-2	1.8E-06	7.5E-06	1.320	0.222	1.096	1.540	2.590	0.311	2.231	2.787
ENSG00000168309	FAM107A	-2	5.3E-03	1.1E-02	0.330	0.167	0.203	0.519	0.663	0.225	0.410	0.840
ENSG00000141736	ERBB2	-2	2.6E-18	1.0E-16	50.449	3.851	47.354	54.761	98.872	5.765	94.822	105.472
ENSG00000139080	EMILIN1	-2	2.7E-08	1.6E-07	2.494	0.050	2.443	2.543	5.075	0.249	4.831	5.329
ENSG00000186197	EDARADD	-2	6.6E-07	3.0E-06	1.454	0.206	1.241	1.653	2.944	0.266	2.638	3.107
ENSG00000011332	DPF1	-2	2.1E-08	1.3E-07	1.581	0.221	1.351	1.792	3.228	0.299	2.910	3.494
ENSG00000088305	DNMT3B	-2	5.0E-10	4.2E-09	457.561	18.955	443.381	479.090	936.978	26.483	914.731	966.272
ENSG00000179097	HTR1F	-2	4.8E-02	7.7E-02	0.227	0.113	0.098	0.310	0.457	0.200	0.269	0.667
ENSG00000181291	TMEM132E	-2	1.3E-01	1.8E-01	0.073	0.037	0.032	0.100	0.152	0.041	0.115	0.197
ENSG00000186047	DLEU7	-2	8.4E-06	3.0E-05	1.657	0.078	1.575	1.731	3.252	0.571	2.835	3.903
ENSG00000077279	DCX	-2	2.4E-13	3.9E-12	2.295	0.176	2.163	2.495	4.685	0.401	4.384	5.140
ENSG00000163734	CXCL3	-2	3.2E-07	1.5E-06	2.287	0.088	2.194	2.371	4.689	0.373	4.372	5.101
ENSG00000225932	CTAGE4	-2	1.2E-04	3.5E-04	1.062	0.236	0.789	1.205	2.192	0.276	1.991	2.507
ENSG00000163359	COL6A3	-2	2.8E-13	4.5E-12	1.119	0.078	1.057	1.207	2.230	0.033	2.199	2.264
ENSG00000143126	CELSR2	-2	5.9E-16	1.5E-14	5.988	0.522	5.387	6.321	11.730	0.937	10.938	12.765
ENSG00000138794	CASP6	-2	1.6E-17	5.4E-16	26.220	2.390	24.785	28.979	53.912	1.127	53.256	55.214
ENSG00000118292	C1orf54	-2	1.4E-10	1.3E-09	10.924	0.673	10.397	11.682	21.479	1.229	20.293	22.747
ENSG00000262874	C19orf84	-2	4.1E-05	1.3E-04	2.236	0.583	1.572	2.667	4.539	0.755	3.668	4.995
ENSG00000168283	BMI1	-2	4.3E-07	2.0E-06	1.240	0.189	1.023	1.369	2.539	0.107	2.429	2.642
ENSG00000279800	BCLAF1P2	-2	3.1E-18	1.2E-16	35.538	2.034	33.208	36.959	69.989	1.614	68.794	71.825
ENSG00000152092	ASTN1	-2	4.0E-10	3.4E-09	1.500	0.069	1.422	1.552	3.038	0.348	2.689	3.386
ENSG00000246100	LINC00900	-2	1.5E-02	2.7E-02	0.189	0.071	0.130	0.269	0.398	0.054	0.354	0.458
ENSG00000251085	LINC01969	-2	1.2E-01	1.7E-01	1.029	1.218	0.000	2.375	2.040	0.664	1.488	2.781
ENSG00000125843	AP5S1	-2	5.9E-05	1.8E-04	0.668	0.152	0.570	0.843	1.337	0.331	1.094	1.714
ENSG00000145362	ANK2	-2	3.9E-19	1.8E-17	5.100	0.409	4.718	5.532	10.100	0.474	9.802	10.647
ENSG00000181754	AMIGO1	-2	3.6E-08	2.1E-07	1.595	0.121	1.520	1.736	3.136	0.361	2.897	3.552
ENSG00000159618	ADGRG5	-2	2.9E-07	1.4E-06	1.144	0.131	0.996	1.248	2.278	0.221	2.029	2.451
ENSG00000149451	ADAM33	-2	6.0E-06	2.3E-05	0.690	0.058	0.635	0.751	1.406	0.176	1.239	1.591
ENSG00000259495		-2	1.0E-04	3.0E-04	0.490	0.168	0.298	0.604	0.973	0.241	0.696	1.137
ENSG00000259692		-2	4.4E-03	9.0E-03	1.770	0.605	1.207	2.410	3.645	1.172	2.610	4.918
ENSG00000274021		-2	1.7E-03	3.9E-03	0.768	0.067	0.693	0.821	1.536	0.161	1.370	1.692
ENSG00000283567		-2	1.1E-02	2.1E-02	1.064	0.212	0.836	1.256	2.109	0.510	1.622	2.639
ENSG00000008735	MAPK8IP2	-1.9	1.1E-03	2.7E-03	0.432	0.031	0.410	0.468	0.816	0.236	0.586	1.065
ENSG00000010610	CD4	-1.9	3.6E-05	1.2E-04	1.005	0.152	0.838	1.133	1.860	0.182	1.686	2.055
ENSG00000011677	GABRA3	-1.9	6.4E-11	6.5E-10	4.270	0.436	3.792	4.645	8.229	0.493	7.773	8.751
ENSG00000016391	CHDH	-1.9	7.7E-13	1.1E-11	3.203	0.203	3.044	3.431	6.127	0.068	6.086	6.180
ENSG00000050628	PTGER3	-1.9	1.5E-03	3.4E-03	0.261	0.089	0.176	0.354	0.495	0.101	0.434	0.612
ENSG00000064692	SNCAIP	-1.9	2.9E-08	1.7E-07	1.816	0.488	1.391	2.349	3.382	0.213	3.239	3.628
ENSG00000083812	ZNF324	-1.9	1.1E-06	4.8E-06	1.210	0.112	1.098	1.322	2.327	0.282	2.106	2.644
ENSG00000084073	ZMPSTE24	-1.9	5.1E-17	1.6E-15	33.648	2.918	30.377	35.985	62.581	3.402	60.220	66.481
ENSG00000088386	SLC15A1	-1.9	1.0E-04	3.0E-04	0.821	0.125	0.700	0.950	1.545	0.080	1.466	1.626
ENSG00000099617	EFNA2	-1.9	1.2E-03	2.8E-03	2.462	0.442	1.952	2.737	4.680	0.611	3.974	5.037
ENSG00000100292	HMOX1	-1.9	4.9E-15	1.1E-13	18.798	1.376	17.278	19.959	35.551	1.544	33.906	36.968
ENSG00000100362	PVALB	-1.9	5.0E-03	1.0E-02	1.410	0.267	1.176	1.700	2.725	0.900	1.741	3.506
ENSG00000103024	NME3	-1.9	5.7E-13	8.7E-12	29.567	2.746	26.666	32.127	55.478	3.035	52.832	58.791
ENSG00000103742	IGDCC4	-1.9	1.6E-11	1.8E-10	2.412	0.086	2.321	2.491	4.690	0.430	4.237	5.092
ENSG00000104524	PYCR3	-1.9	4.4E-09	3.0E-08	4.752	0.833	3.953	5.614	8.991	0.321	8.784	9.361
ENSG00000105939	ZC3HAV1	-1.9	2.6E-20	1.4E-18	18.410	0.862	17.529	19.252	35.144	0.914	34.095	35.767
ENSG00000107551	RASSF4	-1.9	1.5E-10	1.4E-09	1.708	0.078	1.620	1.763	3.321	0.436	2.858	3.725
ENSG00000108312	UBTF	-1.9	9.2E-20	4.5E-18	25.502	0.487	24.941	25.823	47.806	0.972	46.922	48.847
ENSG00000108984	MAP2K6	-1.9	5.3E-16	1.4E-14	9.634	0.545	9.007	9.991	17.861	0.770	17.264	18.730
ENSG00000110092	CCND1	-1.9	4.5E-14	8.4E-13	41.579	2.302	38.923	42.983	80.173	0.802	79.291	80.857
ENSG00000112320	SOBP	-1.9	2.4E-06	9.8E-06	1.034	0.104	0.961	1.153	2.013	0.267	1.857	2.321
ENSG00000112584	FAM120B	-1.9	4.5E-15	1.0E-13	5.850	0.218	5.613	6.042	11.122	0.325	10.750	11.354
ENSG00000113749	HRH2	-1.9	6.6E-12	8.2E-11	3.751	0.095	3.687	3.859	7.077	0.410	6.723	7.527
ENSG00000115107	STEAP3	-1.9	1.6E-14	3.3E-13	10.696	0.785	10.141	11.594	20.153	1.793	18.678	22.149
ENSG00000115594	IL1R1	-1.9	1.8E-02	3.3E-02	0.202	0.107	0.087	0.298	0.392	0.039	0.366	0.437
ENSG00000115648	MLPH	-1.9	3.0E-12	4.0E-11	3.040	0.289	2.835	3.370	5.891	0.271	5.662	6.191
ENSG00000116544	DLGAP3	-1.9	6.6E-10	5.4E-09	3.787	0.222	3.611	4.036	7.094	0.645	6.428	7.715
ENSG00000119812	FAM98A	-1.9	1.1E-20	6.0E-19	39.512	1.607	37.669	40.625	74.083	2.964	70.938	76.825
ENSG00000120659	TNFSF11	-1.9	3.2E-09	2.3E-08	4.456	0.647	4.066	5.203	8.733	0.606	8.178	9.380
ENSG00000128805	ARHGAP22	-1.9	1.2E-09	9.4E-09	2.193	0.261	1.934	2.417	4.204	0.395	3.917	4.654
ENSG00000129195	PIMREG	-1.9	1.9E-19	8.8E-18	48.576	2.188	46.086	50.192	90.277	4.573	86.133	95.183
ENSG00000130528	HRC	-1.9	9.2E-08	4.9E-07	3.171	0.379	2.807	3.564	5.953	0.538	5.333	6.295
ENSG00000131409	LRRC4B	-1.9	7.5E-04	1.8E-03	0.925	0.208	0.692	1.093	1.733	0.175	1.577	1.922
ENSG00000131620	ANO1	-1.9	4.5E-07	2.1E-06	1.221	0.138	1.063	1.307	2.331	0.188	2.197	2.545
ENSG00000131849	ZNF132	-1.9	2.6E-04	7.0E-04	0.906	0.142	0.744	1.008	1.706	0.033	1.674	1.740
ENSG00000132016	C19orf57	-1.9	1.4E-04	3.9E-04	1.042	0.095	0.965	1.148	1.952	0.103	1.855	2.060
ENSG00000134363	FST	-1.9	8.0E-09	5.3E-08	2.873	0.158	2.703	3.017	5.625	0.402	5.162	5.879
ENSG00000134369	NAV1	-1.9	3.4E-17	1.1E-15	5.005	0.131	4.858	5.110	9.485	0.467	8.990	9.918
ENSG00000135363	LMO2	-1.9	3.6E-04	9.5E-04	0.953	0.284	0.783	1.281	1.879	0.378	1.491	2.247
ENSG00000135638	EMX1	-1.9	2.5E-07	1.2E-06	1.886	0.310	1.679	2.243	3.707	0.405	3.241	3.980
ENSG00000137841	PLCB2	-1.9	3.3E-12	4.4E-11	3.255	0.217	3.092	3.501	6.363	0.541	5.911	6.963
ENSG00000141655	TNFRSF11A	-1.9	1.1E-09	8.8E-09	1.313	0.081	1.263	1.406	2.573	0.207	2.440	2.812
ENSG00000143375	CGN	-1.9	2.0E-21	1.3E-19	27.507	1.462	25.889	28.906	51.247	1.259	49.807	52.146
ENSG00000145113	MUC4	-1.9	1.3E-04	3.7E-04	0.197	0.055	0.152	0.259	0.380	0.061	0.316	0.439
ENSG00000145982	FARS2	-1.9	3.6E-11	3.8E-10	9.680	1.273	8.630	11.095	18.751	1.736	17.517	20.736
ENSG00000151572	ANO4	-1.9	1.0E-04	3.0E-04	0.834	0.190	0.618	0.975	1.558	0.211	1.426	1.801
ENSG00000153093	ACOXL	-1.9	8.5E-11	8.3E-10	2.879	0.347	2.485	3.142	5.637	0.441	5.129	5.920
ENSG00000153721	CNKSR3	-1.9	6.1E-10	5.0E-09	0.699	0.035	0.659	0.723	1.333	0.042	1.296	1.379
ENSG00000154479	CCDC173	-1.9	4.4E-03	9.0E-03	0.808	0.237	0.653	1.081	1.534	0.223	1.281	1.698
ENSG00000157601	MX1	-1.9	1.6E-07	8.3E-07	0.891	0.135	0.754	1.023	1.687	0.055	1.637	1.746
ENSG00000157851	DPYSL5	-1.9	1.3E-09	9.7E-09	2.361	0.232	2.159	2.614	4.405	0.220	4.183	4.623
ENSG00000158560	DYNC1I1	-1.9	5.9E-04	1.5E-03	0.513	0.055	0.467	0.574	0.992	0.186	0.795	1.164
ENSG00000158966	CACHD1	-1.9	1.2E-17	4.4E-16	16.822	0.922	15.763	17.447	31.342	0.877	30.337	31.958
ENSG00000159166	LAD1	-1.9	5.8E-14	1.1E-12	12.665	0.987	11.525	13.239	24.463	2.361	22.546	27.100
ENSG00000159231	CBR3	-1.9	1.9E-03	4.2E-03	1.860	0.435	1.389	2.245	3.518	0.847	2.629	4.316
ENSG00000162782	TDRD5	-1.9	4.1E-05	1.3E-04	0.926	0.125	0.839	1.069	1.741	0.140	1.602	1.883
ENSG00000164292	RHOBTB3	-1.9	1.9E-09	1.4E-08	4.953	0.092	4.851	5.033	9.611	0.775	8.877	10.422
ENSG00000164411	GJB7	-1.9	3.2E-03	6.7E-03	0.750	0.252	0.509	1.011	1.392	0.175	1.249	1.587
ENSG00000164707	SLC13A4	-1.9	1.6E-06	6.7E-06	0.761	0.137	0.603	0.851	1.463	0.152	1.310	1.614
ENSG00000165588	OTX2	-1.9	5.9E-10	4.9E-09	9.871	0.909	9.078	10.864	19.318	0.876	18.337	20.023
ENSG00000165633	VSTM4	-1.9	7.3E-05	2.2E-04	0.576	0.241	0.301	0.751	1.096	0.166	0.995	1.287
ENSG00000166669	ATF7IP2	-1.9	6.8E-12	8.4E-11	4.299	0.346	3.916	4.589	8.067	0.641	7.361	8.612
ENSG00000167191	GPRC5B	-1.9	4.8E-22	3.5E-20	29.110	0.710	28.607	29.922	56.376	2.688	53.281	58.128
ENSG00000167912		-1.9	2.4E-08	1.4E-07	5.890	0.845	4.976	6.642	11.334	0.507	10.899	11.892
ENSG00000168546	GFRA2	-1.9	1.4E-05	5.0E-05	0.952	0.210	0.824	1.194	1.790	0.048	1.760	1.845
ENSG00000168661	ZNF30	-1.9	3.6E-09	2.5E-08	4.568	0.554	4.021	5.128	8.568	0.351	8.295	8.963
ENSG00000169418	NPR1	-1.9	5.0E-13	7.8E-12	6.584	0.296	6.325	6.906	12.384	0.369	12.056	12.783
ENSG00000169683	LRRC45	-1.9	4.0E-13	6.3E-12	9.113	0.207	8.946	9.345	17.271	0.881	16.309	18.038
ENSG00000169783	LINGO1	-1.9	4.3E-12	5.6E-11	15.971	1.001	14.835	16.720	31.242	2.661	28.183	33.020
ENSG00000171224	C10orf35	-1.9	3.8E-12	5.0E-11	14.399	0.140	14.302	14.559	27.824	1.330	26.302	28.765
ENSG00000171813	PWWP2B	-1.9	9.7E-10	7.7E-09	6.220	0.589	5.578	6.735	11.639	0.721	11.065	12.448
ENSG00000173231	TERF1P1	-1.9	2.8E-09	2.0E-08	15.007	0.984	13.892	15.754	27.876	2.205	25.366	29.503
ENSG00000173809	TDRD12	-1.9	3.5E-05	1.1E-04	0.551	0.098	0.440	0.625	1.070	0.085	0.972	1.123
ENSG00000175591	P2RY2	-1.9	7.9E-04	1.9E-03	0.872	0.032	0.845	0.908	1.681	0.113	1.611	1.811
ENSG00000175766	EIF4E1B	-1.9	5.9E-02	9.2E-02	0.223	0.181	0.098	0.431	0.436	0.100	0.372	0.551
ENSG00000177706	FAM20C	-1.9	3.5E-12	4.7E-11	5.392	0.184	5.260	5.602	10.147	0.533	9.536	10.517
ENSG00000178896	EXOSC4	-1.9	7.2E-13	1.1E-11	22.240	1.291	20.953	23.535	41.686	2.867	39.315	44.873
ENSG00000179913	B3GNT3	-1.9	1.7E-03	3.9E-03	0.862	0.168	0.671	0.988	1.630	0.081	1.541	1.700
ENSG00000184060	ADAP2	-1.9	1.0E-01	1.5E-01	0.175	0.081	0.082	0.227	0.344	0.162	0.186	0.509
ENSG00000185220	PGBD2	-1.9	5.9E-07	2.7E-06	2.547	0.735	2.029	3.388	4.872	0.378	4.482	5.237
ENSG00000185669	SNAI3	-1.9	5.1E-08	2.9E-07	6.674	0.445	6.239	7.129	12.569	1.751	11.291	14.565
ENSG00000188886	ASTL	-1.9	7.2E-02	1.1E-01	0.400	0.301	0.196	0.747	0.754	0.156	0.595	0.907
ENSG00000189366	ALG1L	-1.9	1.1E-02	2.0E-02	1.246	0.050	1.190	1.284	2.447	0.453	2.126	2.965
ENSG00000196155	PLEKHG4	-1.9	2.2E-09	1.6E-08	1.820	0.089	1.750	1.921	3.546	0.279	3.238	3.780
ENSG00000197046	SIGLEC15	-1.9	1.1E-01	1.6E-01	0.126	0.034	0.090	0.158	0.244	0.078	0.155	0.303
ENSG00000198732	SMOC1	-1.9	3.4E-03	7.1E-03	0.443	0.143	0.283	0.557	0.836	0.089	0.735	0.905
ENSG00000199169	MIR367	-1.9	1.9E-06	7.9E-06	139.586	21.253	121.740	163.097	262.242	56.768	200.533	312.244
ENSG00000203485	INF2	-1.9	1.6E-16	4.4E-15	7.143	0.445	6.804	7.647	13.766	1.134	12.779	15.004

ENSG00000203995	ZYG11A	-1.9	2.6E-03	5.7E-03	0.513	0.370	0.242	0.934	0.978	0.208	0.784	1.198
ENSG00000221955	SLC12A8	-1.9	2.9E-10	2.6E-09	2.823	0.183	2.680	3.029	5.506	0.235	5.251	5.713
ENSG00000224167		-1.9	1.5E-03	3.5E-03	4.019	0.793	3.149	4.701	7.660	2.602	5.473	10.538
ENSG00000225705		-1.9	4.5E-03	9.1E-03	1.127	0.451	0.650	1.546	2.122	0.379	1.725	2.480
ENSG00000228034	HMGN2P21	-1.9	3.4E-03	7.2E-03	6.860	1.564	5.513	8.575	13.236	1.942	10.994	14.361
ENSG00000228863		-1.9	9.4E-02	1.4E-01	0.108	0.076	0.052	0.195	0.214	0.073	0.132	0.271
ENSG00000230555		-1.9	3.1E-03	6.6E-03	2.767	0.596	2.245	3.417	5.111	1.564	3.308	6.105
ENSG00000240137	ERICH6-AS1	-1.9	5.7E-03	1.1E-02	1.856	0.531	1.375	2.426	3.581	0.631	2.871	4.080
ENSG00000241399	CD302	-1.9	2.6E-05	8.6E-05	1.085	0.012	1.075	1.098	2.079	0.202	1.888	2.297
ENSG00000244144		-1.9	5.6E-03	1.1E-02	3.163	1.115	1.877	3.866	6.189	1.351	4.710	7.357
ENSG00000248785	HIGD1AP14	-1.9	1.4E-04	4.1E-04	13.659	2.051	11.512	15.599	26.270	4.271	23.018	31.107
ENSG00000258376		-1.9	6.2E-05	1.9E-04	1.203	0.075	1.119	1.265	2.260	0.292	1.924	2.443
ENSG00000265107	GJA5	-1.9	5.1E-05	1.6E-04	1.487	0.099	1.382	1.578	2.759	0.274	2.444	2.947
ENSG00000266208		-1.9	1.1E-02	2.1E-02	0.551	0.170	0.442	0.747	1.082	0.135	1.001	1.237
ENSG00000273018		-1.9	4.5E-02	7.2E-02	0.103	0.026	0.078	0.129	0.198	0.011	0.187	0.210
ENSG00000273314		-1.9	4.0E-05	1.3E-04	2.242	0.647	1.756	2.976	4.308	0.961	3.432	5.335
ENSG00000279369		-1.9	2.4E-05	8.0E-05	2.211	0.247	2.027	2.492	4.262	0.486	3.777	4.748
ENSG00000280219		-1.9	9.9E-03	1.9E-02	0.164	0.021	0.141	0.181	0.315	0.115	0.190	0.417
ENSG00000282012		-1.9	4.3E-04	1.1E-03	4.367	0.070	4.287	4.418	8.369	0.601	7.721	8.908
ENSG00000005187	ACSM3	-1.8	2.2E-06	8.9E-06	1.178	0.181	0.975	1.322	2.092	0.272	1.778	2.268
ENSG00000010319	SEMA3G	-1.8	3.4E-06	1.3E-05	1.314	0.258	1.020	1.504	2.419	0.224	2.160	2.565
ENSG00000035664	DAPK2	-1.8	4.7E-04	1.2E-03	0.257	0.037	0.215	0.283	0.462	0.103	0.349	0.551
ENSG00000037757	MRI1	-1.8	6.6E-12	8.2E-11	7.316	0.835	6.476	8.146	13.515	0.573	12.975	14.116
ENSG00000042286	AIFM2	-1.8	1.3E-07	6.8E-07	2.026	0.176	1.828	2.164	3.659	0.141	3.505	3.781
ENSG00000050327	ARHGEF5	-1.8	5.8E-14	1.1E-12	7.373	0.593	6.691	7.760	13.606	0.267	13.299	13.769
ENSG00000057704	TMCC3	-1.8	5.6E-07	2.5E-06	1.360	0.119	1.246	1.484	2.445	0.126	2.359	2.590
ENSG00000059378	PARP12	-1.8	8.4E-08	4.5E-07	1.622	0.132	1.472	1.719	2.887	0.145	2.756	3.042
ENSG00000079435	LIPE	-1.8	8.0E-08	4.3E-07	2.641	0.186	2.432	2.787	4.650	0.303	4.320	4.915
ENSG00000083067	TRPM3	-1.8	2.5E-08	1.5E-07	0.723	0.128	0.600	0.856	1.271	0.070	1.193	1.327
ENSG00000099377	HSD3B7	-1.8	5.8E-05	1.8E-04	2.342	0.922	1.485	3.318	4.190	0.311	3.832	4.372
ENSG00000100479	POLE2	-1.8	5.6E-13	8.5E-12	15.813	0.649	15.096	16.360	28.271	1.305	26.765	29.070
ENSG00000100604	CHGA	-1.8	1.1E-15	2.7E-14	21.757	1.439	20.102	22.710	38.908	0.661	38.207	39.520
ENSG00000101265	RASSF2	-1.8	6.9E-13	1.0E-11	7.367	0.381	6.938	7.665	13.172	1.445	11.650	14.526
ENSG00000102195	GPR50	-1.8	7.9E-02	1.2E-01	0.467	0.048	0.416	0.510	0.851	0.294	0.644	1.188
ENSG00000102924	CBLN1	-1.8	1.8E-06	7.4E-06	2.674	0.206	2.467	2.878	4.907	0.543	4.308	5.366
ENSG00000104833	TUBB4A	-1.8	1.7E-10	1.5E-09	8.399	0.744	7.802	9.233	14.980	0.897	13.958	15.636
ENSG00000108839	ALOX12	-1.8	2.3E-03	4.9E-03	1.091	0.380	0.807	1.522	2.018	0.118	1.922	2.150
ENSG00000109610	SOD3	-1.8	3.1E-03	6.7E-03	1.662	0.484	1.144	2.103	3.006	0.449	2.627	3.502
ENSG00000110446	SLC15A3	-1.8	1.7E-08	1.0E-07	2.339	0.176	2.149	2.498	4.267	0.200	4.136	4.498
ENSG00000112562	SMOC2	-1.8	9.5E-05	2.8E-04	1.365	0.263	1.148	1.657	2.435	0.126	2.356	2.580
ENSG00000114698	PLSCR4	-1.8	2.1E-05	7.1E-05	1.213	0.028	1.190	1.245	2.203	0.272	1.936	2.478
ENSG00000114812	VIPR1	-1.8	3.4E-06	1.3E-05	1.015	0.150	0.857	1.155	1.808	0.257	1.533	2.043
ENSG00000114853	ZBTB47	-1.8	2.1E-04	5.8E-04	1.194	0.348	0.902	1.579	2.129	0.102	2.032	2.236
ENSG00000115363	EVA1A	-1.8	3.7E-03	7.6E-03	0.800	0.144	0.674	0.956	1.420	0.283	1.093	1.588
ENSG00000115840	SLC25A12	-1.8	2.4E-11	2.6E-10	5.649	0.406	5.181	5.914	9.880	0.427	9.497	10.308
ENSG00000116711	PLA2G4A	-1.8	6.1E-05	1.9E-04	1.918	0.246	1.772	2.202	3.429	0.463	2.915	3.814
ENSG00000118894	EEF2KMT	-1.8	5.8E-14	1.1E-12	15.046	0.473	14.617	15.554	26.655	0.707	25.924	27.335
ENSG00000120332	TNN	-1.8	1.3E-02	2.4E-02	0.308	0.102	0.199	0.401	0.553	0.120	0.421	0.653
ENSG00000123243	ITIH5	-1.8	1.7E-09	1.2E-08	1.116	0.092	1.011	1.182	2.004	0.097	1.902	2.095
ENSG00000123609	NMI	-1.8	1.4E-05	4.9E-05	2.479	0.824	1.597	3.228	4.601	0.448	4.161	5.058
ENSG00000124357	NAGK	-1.8	6.0E-13	9.0E-12	3.804	0.345	3.520	4.189	7.005	0.453	6.738	7.528
ENSG00000125850	OVOL2	-1.8	2.8E-08	1.7E-07	6.266	0.689	5.491	6.813	11.241	0.888	10.596	12.267
ENSG00000125968	ID1	-1.8	3.5E-11	3.8E-10	19.920	1.915	17.776	21.458	36.008	2.818	34.321	39.261
ENSG00000126882	FAM78A	-1.8	4.9E-06	1.9E-05	1.472	0.263	1.207	1.732	2.713	0.353	2.480	3.119
ENSG00000128228	SDF2L1	-1.8	3.1E-09	2.2E-08	20.719	0.807	20.087	21.628	38.394	2.115	36.225	40.450
ENSG00000130347	RTN4IP1	-1.8	2.1E-12	2.8E-11	17.071	1.774	15.541	19.016	30.756	1.749	29.290	32.691
ENSG00000131196	NFATC1	-1.8	3.9E-05	1.2E-04	0.560	0.162	0.392	0.715	1.005	0.073	0.924	1.068
ENSG00000131831	RAI2	-1.8	1.0E-02	1.9E-02	0.674	0.122	0.589	0.813	1.198	0.307	0.858	1.456
ENSG00000132182	NUP210	-1.8	4.9E-20	2.5E-18	30.332	1.070	29.599	31.560	55.659	2.329	53.830	58.281
ENSG00000132361	CLUH	-1.8	1.4E-18	5.7E-17	21.556	0.359	21.161	21.864	39.474	3.022	37.129	42.885
ENSG00000132436	FIGNL1	-1.8	4.4E-14	8.3E-13	8.814	0.574	8.271	9.462	15.822	0.358	15.417	16.094
ENSG00000132613	MTSS1L	-1.8	3.0E-15	6.9E-14	11.299	0.256	11.023	11.527	20.837	0.978	19.728	21.577
ENSG00000132622	HSPA12B	-1.8	1.0E-07	5.4E-07	3.946	0.149	3.812	4.106	7.283	0.855	6.295	7.790
ENSG00000132773	TOE1	-1.8	1.6E-09	1.2E-08	5.860	0.485	5.445	6.393	10.587	0.355	10.245	10.953
ENSG00000133216	EPHB2	-1.8	2.4E-13	4.0E-12	3.341	0.133	3.229	3.488	6.060	0.423	5.661	6.504
ENSG00000134575	ACP2	-1.8	2.9E-09	2.1E-08	6.556	0.270	6.335	6.856	11.629	1.029	10.625	12.681
ENSG00000135636	DYSF	-1.8	1.6E-11	1.9E-10	4.687	0.564	4.196	5.304	8.222	0.824	7.429	9.073
ENSG00000136867	SLC31A2	-1.8	1.3E-03	3.1E-03	1.182	0.412	0.863	1.647	2.181	0.574	1.562	2.695
ENSG00000136960	ENPP2	-1.8	9.7E-06	3.5E-05	0.997	0.015	0.986	1.014	1.787	0.174	1.588	1.910
ENSG00000137261	KIAA0319	-1.8	1.5E-03	3.5E-03	0.455	0.123	0.362	0.595	0.820	0.145	0.686	0.974
ENSG00000137968	SLC44A5	-1.8	8.6E-06	3.1E-05	1.228	0.299	1.029	1.572	2.190	0.085	2.120	2.284
ENSG00000139531	SUOX	-1.8	2.1E-11	2.4E-10	7.149	0.720	6.380	7.808	12.836	0.538	12.317	13.195
ENSG00000139718	SETD1B	-1.8	3.6E-11	3.8E-10	3.719	0.111	3.592	3.797	6.822	0.810	6.351	7.758
ENSG00000140511	HAPLN3	-1.8	6.0E-16	1.6E-14	18.193	0.126	18.089	18.332	33.298	0.989	32.675	34.439
ENSG00000141096	DPEP3	-1.8	2.7E-04	7.1E-04	1.858	0.530	1.268	2.295	3.348	0.303	3.065	3.669
ENSG00000141485	SLC13A5	-1.8	1.7E-02	3.0E-02	0.184	0.092	0.086	0.268	0.334	0.089	0.279	0.436
ENSG00000141540	TTYH2	-1.8	2.1E-05	7.0E-05	1.257	0.119	1.120	1.329	2.220	0.226	1.962	2.385
ENSG00000142686	C1orf216	-1.8	1.4E-11	1.6E-10	7.456	0.255	7.248	7.741	13.127	0.392	12.776	13.549
ENSG00000143028	SYPL2	-1.8	1.0E-03	2.4E-03	0.770	0.398	0.389	1.184	1.449	0.094	1.342	1.516
ENSG00000143318	CASQ1	-1.8	2.5E-03	5.5E-03	1.135	0.269	0.922	1.438	2.066	0.395	1.797	2.520
ENSG00000144063	MALL	-1.8	1.6E-06	6.8E-06	2.341	0.119	2.222	2.460	4.234	0.421	3.773	4.600
ENSG00000144218	AFF3	-1.8	3.4E-07	1.6E-06	0.722	0.017	0.703	0.737	1.342	0.086	1.253	1.424
ENSG00000144227	NXPH2	-1.8	1.9E-03	4.3E-03	3.056	0.809	2.314	3.920	5.714	1.387	4.116	6.611
ENSG00000144229	THSD7B	-1.8	7.3E-04	1.8E-03	0.412	0.029	0.384	0.442	0.759	0.050	0.707	0.807
ENSG00000144712	CAND2	-1.8	2.8E-10	2.5E-09	4.477	0.519	3.971	5.008	8.039	0.584	7.446	8.613
ENSG00000144847	IGSF11	-1.8	1.1E-03	2.6E-03	0.571	0.234	0.431	0.842	1.059	0.194	0.946	1.283
ENSG00000145284	SCD5	-1.8	1.4E-03	3.3E-03	0.580	0.160	0.396	0.681	1.058	0.146	0.921	1.212
ENSG00000145476	CYP4V2	-1.8	3.5E-05	1.1E-04	0.532	0.167	0.425	0.725	0.981	0.080	0.897	1.057
ENSG00000151388	ADAMTS12	-1.8	2.3E-13	3.9E-12	5.779	0.257	5.620	6.076	10.311	0.580	9.637	10.729
ENSG00000156398	SFXN2	-1.8	1.9E-09	1.4E-08	2.517	0.228	2.318	2.767	4.483	0.279	4.270	4.799
ENSG00000156697	UTP14A	-1.8	3.3E-13	5.3E-12	12.578	0.754	11.855	13.359	22.848	1.678	21.380	24.677
ENSG00000156968	MPV17L	-1.8	1.4E-09	1.1E-08	2.649	0.152	2.485	2.787	4.744	0.127	4.598	4.824
ENSG00000157399	ARSE	-1.8	5.3E-04	1.3E-03	1.170	0.292	0.902	1.482	2.155	0.106	2.039	2.247
ENSG00000158186	MRAS	-1.8	2.4E-09	1.8E-08	3.022	0.295	2.703	3.284	5.334	0.352	4.949	5.640
ENSG00000158882	TOMM40L	-1.8	7.3E-14	1.3E-12	12.607	0.470	12.091	13.013	23.207	1.476	21.618	24.536
ENSG00000160094	ZNF362	-1.8	7.2E-12	8.9E-11	10.220	0.435	9.892	10.714	18.155	1.126	16.900	19.077
ENSG00000160216	AGPAT3	-1.8	1.3E-04	3.8E-04	0.491	0.048	0.437	0.532	0.868	0.131	0.730	0.990
ENSG00000160219	GAB3	-1.8	2.4E-02	4.1E-02	0.192	0.070	0.123	0.263	0.360	0.039	0.320	0.397
ENSG00000162669	HFM1	-1.8	4.1E-05	1.3E-04	0.976	0.066	0.901	1.025	1.756	0.208	1.592	1.990
ENSG00000162722	TRIM58	-1.8	3.8E-04	1.0E-03	1.251	0.226	1.057	1.499	2.292	0.558	1.657	2.706
ENSG00000162878	PKDCC	-1.8	6.1E-14	1.1E-12	15.782	0.582	15.142	16.280	28.197	1.166	26.977	29.301
ENSG00000163377	FAM19A4	-1.8	5.7E-08	3.1E-07	3.807	0.216	3.558	3.939	6.822	0.043	6.780	6.866
ENSG00000164129	NPY5R	-1.8	2.6E-03	5.7E-03	0.668	0.210	0.469	0.888	1.224	0.091	1.146	1.324
ENSG00000164591	MYOZ3	-1.8	1.8E-12	2.5E-11	4.654	0.242	4.428	4.909	8.527	0.392	8.267	8.978
ENSG00000164932	CTHRC1	-1.8	1.4E-04	4.0E-04	2.707	1.048	1.651	3.747	4.871	0.452	4.354	5.191
ENSG00000165125	TRPV6	-1.8	7.3E-03	1.4E-02	0.146	0.061	0.095	0.215	0.271	0.072	0.199	0.343
ENSG00000165511	C10orf25	-1.8	2.8E-05	9.1E-05	1.675	0.172	1.478	1.793	3.025	0.243	2.747	3.197
ENSG00000166289	PLEKHF1	-1.8	4.2E-07	2.0E-06	4.894	0.299	4.720	5.239	8.640	0.837	7.731	9.379
ENSG00000169083	AR	-1.8	1.2E-13	2.1E-12	3.132	0.170	3.024	3.328	5.693	0.258	5.500	5.987
ENSG00000169255	B3GALNT1	-1.8	1.3E-09	1.0E-08	4.368	0.321	4.022	4.657	7.754	0.874	6.746	8.302
ENSG00000169896	ITGAM	-1.8	2.8E-02	4.8E-02	0.219	0.084	0.125	0.287	0.392	0.030	0.361	0.422
ENSG00000169955	ZNF747	-1.8	8.1E-06	2.9E-05	1.828	0.282	1.553	2.118	3.264	0.423	2.815	3.655
ENSG00000172554	SNTG2	-1.8	2.1E-06	8.6E-06	2.158	0.430	1.780	2.625	3.873	0.518	3.461	4.455
ENSG00000174945	AMZ1	-1.8	4.3E-03	8.9E-03	0.322	0.068	0.243	0.374	0.594	0.138	0.461	0.737
ENSG00000175084	DES	-1.8	1.3E-03	2.9E-03	1.177	0.020	1.154	1.190	2.125	0.295	1.882	2.454
ENSG00000175087	PDIK1L	-1.8	1.4E-07	7.4E-07	1.969	0.131	1.838	2.101	3.605	0.301	3.368	3.943
ENSG00000176809	MAMSTR	-1.8	5.6E-03	1.1E-02	0.829	0.119	0.717	0.954	1.532	0.305	1.241	1.850
ENSG00000176920	FUT2	-1.8	2.1E-05	7.1E-05	1.437	0.185	1.279	1.641	2.603	0.107	2.482	2.683
ENSG00000177842	ZNF620	-1.8	4.9E-11	5.1E-10	6.861	0.682	6.206	7.568	12.205	0.572	11.799	12.859
ENSG00000178146		-1.8	8.5E-08	4.5E-07	8.065	0.832	7.220	8.883	14.333	1.330	12.916	15.128
ENSG00000179598	PLD6	-1.8	7.6E-04	1.8E-03	1.142	0.228	0.889	1.332	2.009	0.218	1.758	2.142
ENSG00000179965	ZNF771	-1.8	1.2E-06	5.0E-06	1.545	0.117	1.438	1.670	2.852	0.556	2.369	3.459
ENSG00000181143	MUC16	-1.8	5.4E-04	1.4E-03	0.077	0.012	0.067	0.091	0.138	0.022	0.118	0.162
ENSG00000182489	XKRX	-1.8	2.2E-03	4.9E-03	0.664	0.060	0.626	0.732	1.196	0.127	1.051	1.293
ENSG00000182700	IGIP	-1.8	9.3E-02	1.4E-01	0.176	0.037	0.134	0.206	0.321	0.108	0.244	0.444
ENSG00000183044	ABAT	-1.8	6.3E-09	4.3E-08	2.297	0.452	1.985	2.815	4.085	0.392	3.723	4.501
ENSG00000183798	EMILIN3	-1.8	2.1E-08	1.3E-07	3.501	0.145	3.334	3.592	6.221	0.706	5.557	6.963
ENSG00000183935	HTR7P1	-1.8	3.4E-06	1.3E-05	1.884	0.255	1.656	2.160	3.390	0.247	3.219	3.674
ENSG00000184164	CRELD2	-1.8	2.4E-10	2.1E-09	4.901	0.439	4.463	5.341	8.715	0.537	8.168	9.240
ENSG00000184185	KCNJ12	-1.8	3.0E-04	8.0E-04	0.605	0.131	0.481	0.742	1.115	0.095	1.030	1.210
ENSG00000185056	C5orf67	-1.8	3.6E-02	5.9E-02	0.386	0.030	0.353	0.412	0.689	0.188	0.557	0.918
ENSG00000185909	KLHDC8B	-1.8	5.0E-10	4.2E-09	10.609	0.862	10.004	11.596	18.825	1.548	17.377	20.457
ENSG00000186283	TOR3A	-1.8	4.4E-15	9.8E-14	12.263	0.655	11.797	13.012	22.389	0.869	21.433	23.116
ENSG00000186638	KIF24	-1.8	7.7E-10	6.2E-09	2.722	0.157	2.542	2.820	5.017	0.388	4.586	5.338
ENSG00000187554	TLR5	-1.8	9.8E-03	1.9E-02	0.447	0.265	0.216	0.737	0.793	0.105	0.671	0.858
ENSG00000188060	RAB42	-1.8	3.3E-07	1.6E-06	5.309	0.901	4.783	6.349	9.585	1.227	8.318	10.768
ENSG00000188130	MAPK12	-1.8	1.6E-09	1.2E-08	1.746	0.043	1.720	1.796	3.087	0.247	2.939	3.371
ENSG00000188770	OPTC	-1.8	1.4E-02	2.6E-02	0.895	0.054	0.836	0.942	1.635	0.101	1.552	1.747
ENSG00000196597	ZNF782	-1.8	3.3E-07	1.6E-06	2.213	0.263	1.970	2.492	3.988	0.288	3.717	4.290
[illegible]	[illegible]	[illegible]	[illegible]	[illegible]	[illegible]	[illegible]	[illegible]	[illegible]	[illegible]	[illegible]	[illegible]	[illegible]

ENSG00000167994	RAB3IL1	-1.6	5.1E-06	1.9E-05	3.602	0.381	3.169	3.888	5.741	0.261	5.530	6.033
ENSG00000169302	STK32A	-1.6	8.2E-03	1.6E-02	0.455	0.087	0.377	0.549	0.740	0.175	0.544	0.880
ENSG00000170604	IRF2BP1	-1.6	3.1E-08	1.8E-07	12.416	0.348	12.028	12.699	19.391	2.004	17.325	21.327
ENSG00000170835	CEL	-1.6	6.1E-02	9.5E-02	0.662	0.174	0.465	0.795	1.088	0.495	0.576	1.563
ENSG00000171130	ATP6V0E2	-1.6	1.7E-07	8.8E-07	5.835	0.486	5.372	6.341	9.100	0.667	8.377	9.691
ENSG00000171357	LURAP1	-1.6	3.1E-04	8.2E-04	3.072	0.152	2.977	3.247	4.819	0.170	4.671	5.005
ENSG00000171368	TPPP	-1.6	6.4E-03	1.3E-02	0.428	0.109	0.308	0.519	0.699	0.026	0.673	0.724
ENSG00000171956	FOXB1	-1.6	2.3E-03	5.0E-03	1.417	0.241	1.180	1.661	2.251	0.285	2.009	2.565
ENSG00000172602	RND1	-1.6	6.3E-06	2.4E-05	3.400	0.485	2.889	3.854	5.495	0.095	5.399	5.588
ENSG00000173546	CSPG4	-1.6	4.2E-07	1.9E-06	2.118	0.151	2.007	2.290	3.309	0.194	3.159	3.528
ENSG00000174607	UGT8	-1.6	1.4E-15	3.4E-14	20.248	0.436	19.820	20.691	33.421	0.205	33.211	33.621
ENSG00000179841	AKAP5	-1.6	1.1E-05	3.9E-05	2.242	0.378	1.808	2.498	3.538	0.662	3.069	4.296
ENSG00000179958	DCTPP1	-1.6	6.5E-15	1.4E-13	65.842	3.095	63.944	69.413	107.010	0.679	106.347	107.704
ENSG00000180336	MEIOC	-1.6	6.6E-08	3.6E-07	1.984	0.251	1.775	2.263	3.237	0.167	3.103	3.424
ENSG00000180938	ZNF572	-1.6	2.2E-04	6.0E-04	3.467	0.615	2.776	3.951	5.433	0.832	4.550	6.204
ENSG00000181019	NQO1	-1.6	8.2E-11	8.1E-10	43.153	1.848	41.742	45.244	67.180	3.515	63.297	70.145
ENSG00000181350	LRRC75A	-1.6	1.4E-07	7.1E-07	5.345	0.475	4.804	5.695	8.447	0.455	8.045	8.942
ENSG00000181381	DDX60L	-1.6	2.8E-03	6.1E-03	0.270	0.026	0.246	0.298	0.444	0.108	0.347	0.561
ENSG00000182118	FAM89A	-1.6	6.6E-03	1.3E-02	1.562	0.213	1.356	1.782	2.510	0.616	1.809	2.962
ENSG00000182326	C1S	-1.6	2.6E-08	1.5E-07	3.826	0.330	3.477	4.132	5.977	0.329	5.634	6.291
ENSG00000182912	TSPEAR-AS2	-1.6	2.2E-03	4.8E-03	0.989	0.067	0.912	1.034	1.636	0.182	1.440	1.800
ENSG00000183048	SLC25A10	-1.6	2.7E-05	9.0E-05	4.085	0.575	3.580	4.711	6.633	0.665	5.954	7.284
ENSG00000183092	BEGAIN	-1.6	2.8E-03	6.1E-03	0.556	0.185	0.343	0.666	0.875	0.109	0.750	0.952
ENSG00000183208	GDPGP1	-1.6	3.1E-02	5.3E-02	0.344	0.050	0.294	0.393	0.537	0.061	0.487	0.604
ENSG00000183323	CCDC125	-1.6	2.7E-07	1.3E-06	2.498	0.352	2.140	2.844	4.051	0.564	3.704	4.702
ENSG00000183605	SFXN4	-1.6	3.0E-08	1.8E-07	16.580	1.867	14.625	18.344	26.248	1.641	24.686	27.959
ENSG00000183655	KLHL25	-1.6	2.0E-10	1.8E-09	6.614	0.532	6.273	7.227	10.790	0.720	10.217	11.598
ENSG00000183831	ANKRD45	-1.6	1.1E-02	2.1E-02	1.420	0.295	1.220	1.758	2.337	0.158	2.159	2.463
ENSG00000183876	ARSI	-1.6	1.6E-04	4.6E-04	2.552	0.419	2.172	3.001	4.177	0.371	3.760	4.473
ENSG00000184408	KCND2	-1.6	3.2E-10	2.8E-09	5.791	0.169	5.640	5.974	9.346	0.352	8.959	9.647
ENSG00000184507	NUTM1	-1.6	2.7E-02	4.7E-02	0.447	0.025	0.426	0.474	0.738	0.126	0.633	0.877
ENSG00000184675	AMER1	-1.6	5.3E-13	8.1E-12	7.146	0.066	7.094	7.221	11.747	0.149	11.581	11.871
ENSG00000184992	BRI3BP	-1.6	4.4E-12	5.7E-11	10.094	0.444	9.589	10.426	15.750	0.927	14.809	16.663
ENSG00000185085	INTS5	-1.6	1.9E-09	1.4E-08	11.423	0.444	11.037	11.908	17.883	0.986	16.754	18.576
ENSG00000185915	KLHL34	-1.6	3.1E-02	5.2E-02	0.430	0.068	0.382	0.508	0.712	0.022	0.691	0.734
ENSG00000186193	SAPCD2	-1.6	2.0E-12	2.7E-11	24.275	0.631	23.559	24.749	38.146	2.437	35.757	40.629
ENSG00000186205	MARC1	-1.6	1.4E-13	2.5E-12	9.161	0.374	8.837	9.570	14.345	0.446	13.911	14.803
ENSG00000186310	NAP1L3	-1.6	1.6E-15	3.8E-14	81.945	6.012	76.736	88.523	129.170	2.784	126.080	131.483
ENSG00000188997	KCTD21	-1.6	3.0E-04	7.9E-04	1.684	0.047	1.631	1.715	2.621	0.227	2.465	2.882
ENSG00000189046	ALKBH2	-1.6	2.5E-08	1.5E-07	11.842	1.098	10.784	12.977	18.764	0.977	18.162	19.892
ENSG00000189127	ANKRD34B	-1.6	1.3E-04	3.7E-04	2.356	0.242	2.148	2.622	3.708	0.514	3.152	4.166
ENSG00000196814	MVB12B	-1.6	5.3E-07	2.4E-06	2.101	0.312	1.840	2.447	3.356	0.251	3.085	3.580
ENSG00000197496	SLC2A10	-1.6	3.2E-10	2.8E-09	7.509	0.323	7.272	7.877	11.726	0.271	11.500	12.027
ENSG00000197635	DPP4	-1.6	5.2E-04	1.3E-03	1.482	0.387	1.107	1.880	2.438	0.218	2.189	2.594
ENSG00000197785	ATAD3A	-1.6	3.6E-13	5.7E-12	35.528	1.524	33.786	36.614	55.682	5.132	52.010	61.547
ENSG00000197982	C1orf122	-1.6	1.8E-09	1.3E-08	19.302	1.939	17.909	21.516	30.844	3.434	27.362	34.228
ENSG00000198176	TFDP1	-1.6	1.5E-16	4.2E-15	53.330	2.862	51.351	56.613	85.311	3.061	82.635	88.648
ENSG00000198324	FAM109A	-1.6	7.0E-08	3.8E-07	6.704	0.193	6.521	6.906	10.568	0.599	10.143	11.252
ENSG00000198440	ZNF583	-1.6	2.9E-04	7.6E-04	0.820	0.158	0.644	0.953	1.314	0.078	1.265	1.404
ENSG00000198483	ANKRD35	-1.6	1.0E-05	3.6E-05	4.064	0.339	3.678	4.314	6.322	0.465	5.798	6.687
ENSG00000198846	TOX	-1.6	1.4E-09	1.0E-08	6.934	0.732	6.483	7.779	11.150	0.220	10.990	11.401
ENSG00000198917	SPOUT1	-1.6	6.7E-15	1.5E-13	23.797	0.530	23.356	24.385	38.606	1.947	37.406	40.852
ENSG00000204065	TCEAL5	-1.6	3.4E-08	2.0E-07	21.795	3.207	18.765	25.154	34.882	1.581	33.624	36.656
ENSG00000204071	TCEAL6	-1.6	6.2E-02	9.6E-02	1.220	0.281	0.898	1.417	1.937	0.088	1.835	1.994
ENSG00000204287	HLA-DRA	-1.6	1.4E-03	3.1E-03	4.462	0.418	4.220	4.946	7.145	2.091	4.911	9.055
ENSG00000204388	HSPA1B	-1.6	3.4E-13	5.4E-12	36.975	3.484	34.718	40.988	60.327	3.800	57.458	64.637
ENSG00000204442	FAM155A	-1.6	2.4E-07	1.2E-06	2.085	0.234	1.815	2.233	3.355	0.184	3.143	3.472
ENSG00000207927	MIR302A	-1.6	2.9E-02	4.9E-02	29.089	9.492	22.667	39.992	47.613	9.844	36.440	55.014
ENSG00000213185	FAM24B	-1.6	3.8E-02	6.2E-02	1.201	0.217	1.049	1.450	1.865	0.254	1.672	2.136
ENSG00000213965	NUDT19	-1.6	1.2E-03	2.9E-03	1.954	0.214	1.717	2.134	3.112	0.724	2.390	3.838
ENSG00000214336	FOXI3	-1.6	4.2E-07	2.0E-06	4.110	0.348	3.763	4.459	6.772	0.444	6.273	7.124
ENSG00000214717	ZBED1	-1.6	4.0E-07	1.9E-06	17.471	1.482	16.165	19.082	28.067	1.065	27.039	29.165
ENSG00000215910	C1orf167	-1.6	3.2E-02	5.3E-02	0.317	0.104	0.197	0.380	0.499	0.112	0.420	0.627
ENSG00000223573	TINCR	-1.6	6.1E-02	9.5E-02	0.375	0.121	0.236	0.455	0.600	0.209	0.358	0.730
ENSG00000223658	C1GALT1C1L	-1.6	1.2E-01	1.7E-01	0.736	0.181	0.527	0.849	1.188	0.328	0.854	1.430
ENSG00000225366	TDGF1P3	-1.6	3.7E-17	1.2E-15	84.010	2.991	80.858	86.807	136.487	4.075	132.146	140.228
ENSG00000225882	LINC01456	-1.6	3.5E-02	5.8E-02	2.522	0.312	2.209	2.834	3.978	1.219	2.762	5.200
ENSG00000227036	LINC00511	-1.6	9.4E-02	1.4E-01	0.186	0.141	0.066	0.340	0.290	0.083	0.195	0.348
ENSG00000227733		-1.6	7.1E-05	2.2E-04	1.071	0.244	0.801	1.277	1.693	0.168	1.504	1.825
ENSG00000228038	VN1R51P	-1.6	4.6E-03	9.3E-03	3.644	0.647	2.940	4.213	5.877	0.065	5.802	5.916
ENSG00000228962	HCG23	-1.6	1.2E-02	2.2E-02	4.158	1.946	2.797	6.386	6.760	0.628	6.036	7.145
ENSG00000230091	TMEM254-AS1	-1.6	3.2E-03	6.9E-03	1.061	0.175	0.861	1.190	1.682	0.088	1.613	1.781
ENSG00000230453	ANKRD18B	-1.6	7.2E-06	2.7E-05	2.313	0.206	2.076	2.451	3.686	0.245	3.445	3.935
ENSG00000230487	PSMG3-AS1	-1.6	2.4E-04	6.5E-04	1.077	0.124	0.945	1.190	1.719	0.260	1.451	1.969
ENSG00000230530	LIMD1-AS1	-1.6	3.1E-02	5.1E-02	1.210	0.365	0.861	1.589	1.911	0.455	1.404	2.284
ENSG00000230606		-1.6	9.4E-04	2.2E-03	0.986	0.095	0.877	1.056	1.587	0.293	1.361	1.919
ENSG00000234373	SNX18P7	-1.6	3.4E-02	5.6E-02	3.046	0.126	2.911	3.160	4.887	0.913	4.295	5.939
ENSG00000235217	TSPY26P	-1.6	7.5E-08	4.1E-07	4.497	0.474	3.966	4.877	7.395	0.686	6.759	8.121
ENSG00000237082	COX5BP6	-1.6	1.3E-01	1.8E-01	4.394	1.531	3.369	6.154	7.029	1.430	5.856	8.606
ENSG00000241634		-1.6	4.0E-03	8.2E-03	6.576	1.497	4.852	7.542	10.935	3.140	8.037	14.271
ENSG00000242474		-1.6	2.9E-03	6.2E-03	2.665	0.405	2.206	2.972	4.266	0.801	3.344	4.797
ENSG00000244198		-1.6	3.4E-08	2.0E-07	12.973	0.275	12.665	13.196	20.391	0.693	19.618	20.959
ENSG00000246985	SOCS2-AS1	-1.6	5.9E-04	1.5E-03	1.339	0.217	1.133	1.565	2.183	0.144	2.063	2.343
ENSG00000248103		-1.6	3.5E-02	5.8E-02	3.372	0.954	2.590	4.435	5.221	0.754	4.354	5.713
ENSG00000250746		-1.6	7.1E-04	1.7E-03	2.327	0.190	2.108	2.446	3.833	0.569	3.178	4.210
ENSG00000253958	CLDN23	-1.6	3.4E-03	7.2E-03	1.975	0.526	1.423	2.470	3.202	0.917	2.254	4.084
ENSG00000255198	SNHG9	-1.6	3.8E-04	9.9E-04	10.430	2.705	8.774	13.551	16.873	1.425	15.465	18.314
ENSG00000257167	TMPO-AS1	-1.6	1.6E-06	6.8E-06	5.474	0.307	5.150	5.760	8.643	1.729	7.115	10.521
ENSG00000258056		-1.6	3.0E-05	9.8E-05	6.472	1.220	5.419	7.808	10.416	0.804	9.874	11.340
ENSG00000258366	RTEL1	-1.6	7.5E-03	1.5E-02	0.488	0.128	0.360	0.617	0.809	0.213	0.563	0.933
ENSG00000259845	HERC2P10	-1.6	4.9E-02	7.8E-02	0.718	0.098	0.610	0.802	1.145	0.071	1.064	1.197
ENSG00000260128	ULK4P2	-1.6	2.1E-02	3.7E-02	1.317	0.124	1.184	1.430	2.070	0.131	1.963	2.216
ENSG00000260265		-1.6	3.5E-03	7.3E-03	4.062	0.658	3.303	4.483	6.364	0.699	5.679	7.077
ENSG00000262251		-1.6	4.1E-03	8.4E-03	3.657	1.078	2.685	4.817	5.781	1.382	4.299	7.035
ENSG00000265933	LINC00668	-1.6	6.0E-02	9.3E-02	0.240	0.012	0.234	0.254	0.390	0.040	0.344	0.420
ENSG00000266094	RASSF5	-1.6	1.0E-02	2.0E-02	0.592	0.287	0.372	0.917	0.956	0.214	0.741	1.169
ENSG00000266876		-1.6	1.3E-02	2.3E-02	2.936	0.290	2.759	3.270	4.628	0.406	4.218	5.029
ENSG00000267194		-1.6	6.4E-07	2.9E-06	7.673	0.029	7.649	7.705	12.415	1.319	11.000	13.612
ENSG00000269837	IPO5P1	-1.6	1.2E-06	5.1E-06	10.248	1.220	9.035	11.475	16.429	1.128	15.209	17.434
ENSG00000271369		-1.6	1.1E-01	1.6E-01	2.030	1.012	1.265	3.177	3.201	1.436	1.740	4.610
ENSG00000272077		-1.6	1.3E-03	3.1E-03	2.680	0.018	2.660	2.694	4.420	1.505	2.861	5.865
ENSG00000272293		-1.6	7.5E-04	1.8E-03	8.775	0.697	7.987	9.310	14.212	3.834	11.604	18.615
ENSG00000272821		-1.6	1.0E-02	1.9E-02	2.959	0.428	2.468	3.249	4.878	0.706	4.147	5.555
ENSG00000274114		-1.6	2.5E-02	4.3E-02	0.512	0.153	0.390	0.684	0.797	0.047	0.745	0.838
ENSG00000274286	ADRA2B	-1.6	1.3E-06	5.6E-06	4.010	0.689	3.419	4.766	6.547	0.272	6.256	6.796
ENSG00000275832	ARHGAP23	-1.6	9.2E-14	1.6E-12	15.583	0.146	15.442	15.734	24.371	0.594	23.897	25.037
ENSG00000276855		-1.6	2.5E-04	6.8E-04	8.410	0.785	7.606	9.175	13.432	0.727	12.598	13.933
ENSG00000279159		-1.6	1.9E-02	3.4E-02	0.045	0.019	0.025	0.063	0.073	0.014	0.061	0.088
ENSG00000279348		-1.6	7.7E-05	2.3E-04	4.671	0.775	3.897	5.446	7.478	0.378	7.043	7.724
ENSG00000280069		-1.6	9.3E-02	1.4E-01	0.637	0.154	0.543	0.815	1.004	0.207	0.772	1.172
ENSG00000280079		-1.6	2.4E-09	1.7E-08	24.186	0.364	23.787	24.501	37.631	2.132	35.248	39.359
ENSG00000282304		-1.6	2.7E-02	4.6E-02	1.570	0.176	1.373	1.711	2.599	0.556	2.062	3.173
ENSG00000001626	CFTR	-1.5	1.2E-01	1.7E-01	0.208	0.072	0.127	0.267	0.314	0.143	0.164	0.448
ENSG00000003249	DBNDD1	-1.5	2.9E-11	3.2E-10	18.052	0.632	17.685	18.782	26.754	1.809	24.666	27.836
ENSG00000003989	SLC7A2	-1.5	3.1E-11	3.4E-10	13.611	0.681	12.854	14.174	19.865	0.889	19.155	20.862
ENSG00000005469	CROT	-1.5	1.7E-04	4.9E-04	2.127	0.241	1.858	2.325	3.179	0.130	3.050	3.311
ENSG00000006118	TMEM132A	-1.5	6.5E-11	6.6E-10	8.678	0.366	8.291	9.019	13.374	0.300	13.045	13.632
ENSG00000007312	CD79B	-1.5	1.1E-02	2.0E-02	2.344	0.591	1.873	3.008	3.446	0.525	2.839	3.761
ENSG00000008853	RHOBTB2	-1.5	9.1E-07	4.0E-06	3.488	0.616	2.788	3.947	5.339	0.029	5.307	5.363
ENSG00000010072	SPRTN	-1.5	4.3E-07	2.0E-06	4.744	0.356	4.425	5.128	7.042	0.364	6.626	7.308
ENSG00000011376	LARS2	-1.5	4.3E-14	8.2E-13	27.335	1.036	26.516	28.501	41.298	1.516	39.773	42.806
ENSG00000012048	BRCA1	-1.5	6.5E-14	1.2E-12	14.175	0.678	13.399	14.646	21.208	0.557	20.648	21.762
ENSG00000012963	UBR7	-1.5	3.8E-14	7.3E-13	33.909	1.978	32.033	35.976	52.319	0.709	51.570	52.979
ENSG00000013288	MAN2B2	-1.5	9.8E-04	2.3E-03	1.478	0.330	1.232	1.854	2.184	0.162	2.007	2.323
ENSG00000013503	POLR3B	-1.5	4.3E-09	3.0E-08	10.034	1.017	8.921	10.917	15.225	0.565	14.607	15.715
ENSG00000014138	POLA2	-1.5	1.7E-11	1.9E-10	15.376	0.783	14.475	15.892	23.131	1.131	22.395	24.434
ENSG00000014216	CAPN1	-1.5	3.2E-14	6.2E-13	28.293	0.529	27.915	28.897	41.751	1.196	40.392	42.644
ENSG00000015413	DPEP1	-1.5	6.3E-06	2.4E-05	5.771	0.634	5.041	6.182	8.697	0.415	8.221	8.983
ENSG00000015475	BID	-1.5	3.3E-11	3.6E-10	14.415	0.189	14.199	14.551	21.796	0.807	21.009	22.622
ENSG00000016402	IL20RA	-1.5	7.6E-03	1.5E-02	1.172	0.084	1.077	1.235	1.716	0.355	1.326	2.021
ENSG00000017483	SLC38A5	-1.5	1.1E-10	1.0E-09	16.634	0.412	16.256	17.074	24.823	0.759	23.984	25.464
ENSG00000018625	ATP1A2	-1.5	5.3E-14	9.8E-13	26.877	1.031	25.978	28.003	39.266	1.402	38.371	40.882
ENSG00000019485	PRDM11	-1.5	2.1E-07	1.0E-06	1.818	0.037	1.782	1.856	2.767	0.280	2.475	3.032
ENSG00000021762	OSBPL5	-1.5	3.4E-05	1.1E-04	1.604	0.179	1.447	1.799	2.393	0.193	2.170	2.523
ENSG00000025423	HSD17B6	-1.5	2.6E-03	5.7E-03	2.347	0.100	2.242	2.441	3.462	0.212	3.218	3.601
ENSG00000035862	TIMP2	-1.5	4.3E-11	4.6E-10	14.241	0.121	14.111	14.350	20.763	0.776	20.165	21.639
ENSG00000038358	EDC4	-1.5	2.0E-12	2.7E-11	17.021	0.303	16.678	17.250	24.763	0.454	24.258	25.139
ENSG00000041802	LSG1	-1.5	5.9E-13	9.0E-12	30.998	0.532	30.389	31.371	45.524	0.633	44.794	45.907
ENSG00000046889	PREX2	-1.5	2.3E-15	5.5E-14	13.579	0.197	13.364	13.749	20.581	0.107	20.486	20.696
ENSG00000047621	C12orf4	-1.5	1.5E-07	7.6E-07	5.442	0.569	5.063	6.096	8.254	0.431	7.786	8.636
ENSG00000049323	LTBP1	-1.5	2.2E-13	3.7E-12	15.346	0.559	14.700	15.680	23.713	0.873	22.929	24.653
ENSG00000051128	HOMER3	-1.5	3.8E-07	1.8E-06	7.011	0.549	6.482	7.578	10.821	1.211	9.901	12.194
ENSG00000051180	RAD51	-1.5	1.8E-10	1.6E-09	25.768	0.240	25.627	26.046	37.915	1.181	36.734	39.095
ENSG00000054118	THRAP3	-1.5	3.6E-16	9.8E-15	128.033	6.262	121.253	133.600	187.242	3.311	183.685	190.233
ENSG00000062582	MRPS24	-1.5	1.1E-02	2.1E-02	1.728	0.128	1.595	1.850	2.637	0.410	2.192	3.001
ENSG00000063015	SEZ6	-1.5	8.6E-03	1.7E-02	1.060	0.280	0.815	1.365	1.611	0.132	1.484	1.748
ENSG00000064270	ATP2C2	-1.5	5.4E-03	1.1E-02	0.707	0.062	0.651	0.773	1.053	0.172	0.909	1.243
ENSG00000066032	CTNNA2	-1.5	3.5E-07	1.7E-06	2.971	0.143	2.827	3.114	4.394	0.141	4.253	4.534
ENSG00000066926	FECH	-1.5	2.4E-12	3.3E-11	19.450	0.729	18.616	19.963	29.177	0.405	28.856	29.632
ENSG00000068654	POLR1A	-1.5	4.9E-15	1.1E-13	16.416	0.488	15.861	16.776	24.206	0.693	23.462	24.833
ENSG00000070614	NDST1	-1.5	6.0E-14	1.1E-12	23.665	0.574	23.167	24.292	35.215	2.191	33.335	37.622
ENSG00000070882	OSBPL3	-1.5	3.9E-09	2.7E-08	6.038	0.168	5.845	6.152	8.867	0.378	8.518	9.269
ENSG00000072422	RHOBTB1	-1.5	7.0E-09	4.7E-08	9.810	0.568	9.310	10.428	14.332	0.190	14.173	14.543
ENSG00000072849	DERL2	-1.5	6.7E-10	5.5E-09	9.503	0.524	8.919	9.930	14.226	0.367	13.826	14.546
ENSG00000073464	CLCN4	-1.5	6.8E-09	4.6E-08	5.827	0.483	5.284	6.207	8.650	0.472	8.107	8.958
ENSG00000074370	ATP2A3	-1.5	1.5E-09	1.1E-08	8.419	0.601	7.818	9.019	12.688	1.235	11.267	13.502
ENSG00000074706	IPCEF1	-1.5	4.0E-02	6.6E-02	0.272	0.072	0.201	0.346	0.404	0.024	0.382	0.429
ENSG00000075643	MOCOS	-1.5	7.4E-08	4.0E-07	5.826	0.239	5.652	6.099	8.591	0.876	8.076	9.603
ENSG00000077514	POLD3	-1.5	7.0E-13	1.1E-11	20.010	0.786	19.362	20.884	30.015	0.800	29.213	30.814
ENSG00000078269	SYNJ2	-1.5	3.6E-08	2.1E-07	3.797	0.230	3.583	4.041	5.886	0.511	5.546	6.473
ENSG00000079385	CEACAM1	-1.5	3.6E-02	6.0E-02	0.451	0.069	0.411	0.531	0.663	0.139	0.506	0.769
ENSG00000081692	JMJD4	-1.5	2.1E-07	1.0E-06	5.573	0.544	5.009	6.094	8.366	0.118	8.287	8.501
ENSG00000085511	MAP3K4	-1.5	9.7E-16	2.5E-14	20.132	0.605	19.508	20.716	31.023	1.044	29.824	31.737
ENSG00000085552	IGSF9	-1.5	9.9E-09	6.4E-08	6.490	0.516	5.962	6.994	9.798	1.026	8.662	10.657
ENSG00000087087	SRRT	-1.5	8.8E-13	1.3E-11	48.998	3.623	46.829	53.181	74.475	2.581	72.319	77.335
ENSG00000087152	ATXN7L3	-1.5	3.6E-14	7.0E-13	32.909	1.174	32.026	34.242	48.860	0.743	48.052	49.514
ENSG00000088280	ASAP3	-1.5	3.7E-13	5.9E-12	28.107	0.498	27.540	28.474	42.076	1.988	40.019	43.987
ENSG00000088888	MAVS	-1.5	7.2E-15	1.6E-13	14.951	0.500	14.423	15.417	22.374	0.371	22.108	22.798
ENSG00000089091	DZANK1	-1.5	4.7E-05	1.5E-04	2.551	0.154	2.449	2.728	3.735	0.183	3.626	3.946
ENSG00000089692	LAG3	-1.5	3.1E-02	5.2E-02	0.755	0.179	0.558	0.909	1.169	0.358	0.848	1.554
ENSG00000089820	ARHGAP4	-1.5	2.9E-09	2.1E-08	8.320	0.630	7.912	9.045	12.610	0.756	11.916	13.416
ENSG00000090376	IRAK3	-1.5	6.5E-04	1.6E-03	0.940	0.130	0.796	1.049	1.369	0.078	1.321	1.458
ENSG00000090565	RAB11FIP3	-1.5	5.3E-10	4.4E-09	11.338	0.567	10.827	11.948	16.836	1.043	15.899	17.960
ENSG00000090674	MCOLN1	-1.5	3.0E-05	9.7E-05	3.514	0.151	3.365	3.666	5.161	0.175	4.964	5.297
ENSG00000091622	PITPNM3	-1.5	2.6E-05	8.5E-05	1.203	0.165	1.091	1.392	1.833	0.118	1.712	1.948
ENSG00000092439	TRPM7	-1.5	1.2E-12	1.7E-11	13.857	0.067	13.779	13.898	20.479	1.409	18.918	21.656
ENSG00000092929	UNC13D	-1.5	1.1E-04	3.2E-04	1.603	0.237	1.361	1.834	2.451	0.300	2.128	2.720
ENSG00000099866	MADCAM1	-1.5	1.4E-01	2.0E-01	0.405	0.075	0.322	0.466	0.612	0.217	0.422	0.849
ENSG00000099875	MKNK2	-1.5	1.3E-13	2.3E-12	40.854	1.349	39.325	41.878	60.614	4.570	56.640	65.608
ENSG00000099899	TRMT2A	-1.5	2.3E-09	1.7E-08	10.269	0.459	9.759	10.649	15.311	0.096	15.227	15.415
ENSG00000100211	CBY1	-1.5	1.8E-08	1.1E-07	16.410	1.661	14.517	17.622	24.710	0.930	23.637	25.268
ENSG00000100263	RHBDD3	-1.5	2.2E-09	1.6E-08	21.909	0.444	21.560	22.408	32.127	1.660	30.589	33.887
ENSG00000100304	TTLL12	-1.5	2.7E-14	5.4E-13	57.147	2.389	54.566	59.280	83.341	3.796	80.392	87.624
ENSG00000100385	IL2RB	-1.5	1.8E-02	3.2E-02	0.875	0.261	0.696	1.174	1.338	0.097	1.278	1.451
ENSG00000100395	L3MBTL2	-1.5	1.2E-10	1.1E-09	8.913	0.414	8.446	9.234	13.724	0.846	13.120	14.691
ENSG00000100422	CERK	-1.5	1.9E-14	3.9E-13	30.015	0.127	29.880	30.132	45.446	0.538	45.120	46.067
ENSG00000100478	AP4S1	-1.5	5.8E-07	2.6E-06	3.139	0.164	2.955	3.268	4.665	0.030	4.633	4.691
ENSG00000100626	GALNT16	-1.5	8.3E-05	2.5E-04	1.393	0.069	1.315	1.443	2.027	0.073	1.966	2.108
ENSG00000100842	EFS	-1.5	2.2E-09	1.6E-08	12.466	0.717	11.640	12.922	19.018	1.529	17.607	20.642
ENSG00000101331	CCM2L	-1.5	1.6E-04	4.5E-04	5.223	0.815	4.415	6.046	7.698	1.381	6.534	9.224
ENSG00000101850	GPR143	-1.5	6.4E-07	2.9E-06	11.962	1.799	10.865	14.038	17.401	1.117	16.183	18.375
ENSG00000101938	CHRDL1	-1.5	8.4E-12	1.0E-10	17.651	0.542	17.049	18.100	26.929	0.936	26.156	27.899
ENSG00000102383	ZDHHC15	-1.5	2.0E-04	5.6E-04	2.093	0.339	1.743	2.418	3.076	0.424	2.799	3.564
ENSG00000102935	ZNF423	-1.5	1.1E-10	1.0E-09	9.431	0.577	8.784	9.893	14.183	0.923	13.151	14.929
ENSG00000102996	MMP15	-1.5	1.3E-08	8.2E-08	29.176	0.707	28.362	29.634	42.555	3.859	38.249	45.701
ENSG00000103064	SLC7A6	-1.5	6.9E-13	1.0E-11	11.879	0.289	11.554	12.105	18.003	0.300	17.773	18.343
ENSG00000103326	CAPN15	-1.5	3.7E-12	4.8E-11	15.377	0.502	14.958	15.933	23.422	2.019	21.739	25.661
ENSG00000103647	CORO2B	-1.5	2.3E-04	6.3E-04	1.997	0.076	1.949	2.085	3.077	0.283	2.813	3.377
ENSG00000103811	CTSH	-1.5	3.2E-06	1.3E-05	3.464	0.114	3.333	3.537	5.112	0.347	4.718	5.369
ENSG00000104112	SCG3	-1.5	1.3E-14	2.8E-13	56.266	2.955	52.886	58.719	83.992	1.372	82.928	85.541
ENSG00000104129	DNAJC17	-1.5	6.5E-08	3.6E-07	5.608	0.402	5.233	6.032	8.488	0.194	8.326	8.703
ENSG00000104142	VPS18	-1.5	1.1E-10	1.1E-09	17.010	0.883	16.356	18.015	25.431	1.591	24.150	27.212
ENSG00000104356	POP1	-1.5	4.3E-10	3.7E-09	9.231	0.371	8.844	9.583	14.132	0.517	13.793	14.727
ENSG00000104611	SH2D4A	-1.5	1.2E-05	4.1E-05	3.631	0.395	3.200	3.976	5.531	0.387	5.118	5.884
ENSG00000104888	SLC17A7	-1.5	8.1E-03	1.6E-02	0.635	0.073	0.591	0.719	0.963	0.071	0.884	1.022
ENSG00000105011	ASF1B	-1.5	2.3E-10	2.1E-09	25.287	0.949	24.519	26.349	37.709	2.706	34.801	40.152
ENSG00000105251	SHD	-1.5	3.1E-02	5.2E-02	1.132	0.183	0.924	1.265	1.664	0.180	1.491	1.851
ENSG00000105464	GRIN2D	-1.5	9.4E-09	6.1E-08	8.500	0.439	8.180	9.001	12.809	0.880	11.821	13.548
ENSG00000105928	DFNA5	-1.5	9.6E-05	2.8E-04	1.495	0.242	1.249	1.733	2.182	0.247	2.038	2.468
ENSG00000106246	PTCD1	-1.5	2.7E-05	8.9E-05	1.969	0.178	1.764	2.074	3.024	0.181	2.866	3.221
ENSG00000107816	LZTS2	-1.5	1.6E-15	3.9E-14	23.784	0.551	23.245	24.347	36.123	1.165	34.778	36.829
ENSG00000107957	SH3PXD2A	-1.5	5.0E-12	6.3E-11	7.895	0.049	7.838	7.925	11.508	0.405	11.077	11.882
ENSG00000108798	ABI3	-1.5	1.1E-02	2.1E-02	1.465	0.151	1.320	1.621	2.262	0.150	2.104	2.404
ENSG00000108819	PPP1R9B	-1.5	3.5E-06	1.4E-05	5.561	0.513	4.992	5.988	8.167	0.716	7.540	8.947
ENSG00000108963	DPH1	-1.5	7.4E-07	3.3E-06	3.926	0.292	3.646	4.229	5.848	0.506	5.397	6.395
ENSG00000110057	UNC93B1	-1.5	7.6E-07	3.4E-06	7.771	0.350	7.375	8.038	11.427	0.600	10.863	12.058
ENSG00000110108	TMEM109	-1.5	4.4E-10	3.7E-09	22.811	2.105	20.717	24.928	34.862	1.529	33.177	36.160
ENSG00000110497	AMBRA1	-1.5	3.1E-11	3.4E-10	9.769	0.363	9.435	10.155	14.471	0.601	14.054	15.159
ENSG00000110721	CHKA	-1.5	7.4E-14	1.3E-12	33.230	1.285	31.929	34.499	51.300	1.288	49.902	52.437
ENSG00000110931	CAMKK2	-1.5	4.9E-11	5.1E-10	15.540	0.407	15.099	15.900	23.052	1.400	21.968	24.632
ENSG00000111452	ADGRD1	-1.5	3.8E-02	6.3E-02	0.128	0.036	0.099	0.168	0.198	0.023	0.172	0.213
ENSG00000111554	MDM1	-1.5	7.4E-07	3.3E-06	4.570	0.312	4.212	4.784	6.714	0.051	6.655	6.746
ENSG00000111664	GNB3	-1.5	3.1E-02	5.2E-02	0.644	0.159	0.472	0.786	1.007	0.234	0.818	1.270
ENSG00000111676	ATN1	-1.5	1.0E-10	9.7E-10	19.356	0.572	18.702	19.766	28.150	1.844	26.753	30.239
ENSG00000111879	FAM184A	-1.5	2.4E-07	1.2E-06	4.616	0.677	4.104	5.384	6.890	0.489	6.336	7.261
ENSG00000112081	TULP1	-1.5	4.7E-03	9.6E-03	1.889	0.356	1.538	2.250	2.782	0.355	2.503	3.181
ENSG00000112164	GLP1R	-1.5	2.7E-03	5.7E-03	1.655	0.367	1.243	1.946	2.522	0.012	2.512	2.536
ENSG00000112280	COL9A1	-1.5	8.5E-09	5.6E-08	6.082	0.398	5.657	6.446	9.262	0.399	8.803	9.524
ENSG00000113296	THBS4	-1.5	1.7E-08	1.1E-07	7.157	0.249	6.905	7.402	10.928	0.503	10.496	11.479
ENSG00000113396	SLC27A6	-1.5	1.3E-05	4.6E-05	4.394	1.058	3.535	5.576	6.727	0.522	6.158	7.183
ENSG00000113761	ZNF346	-1.5	1.5E-07	7.6E-07	7.376	0.765	6.706	8.209	10.749	0.669	10.141	11.465
ENSG00000114491	UMPS	-1.5	1.2E-14	2.4E-13	19.739	0.833	18.779	20.284	29.981	0.436	29.718	30.485
ENSG00000114790	ARHGEF26	-1.5	1.3E-09	1.0E-08	8.628	0.739	8.183	9.481	13.137	0.156	12.960	13.259
ENSG00000115380	EFEMP1	-1.5	1.4E-08	8.8E-08	9.841	1.162	8.972	11.161	15.145	0.566	14.593	15.725
ENSG00000116096	SPR	-1.5	4.3E-06	1.7E-05	10.189	0.526	9.703	10.748	15.761	1.182	14.441	16.719
ENSG00000118096	IFT46	-1.5	9.9E-07	4.3E-06	6.011	0.471	5.544	6.487	8.942	0.554	8.331	9.413
ENSG00000118246	FASTKD2	-1.5	5.1E-14	9.6E-13	24.018	0.226	23.764	24.197	35.973	0.384	35.532	36.238
ENSG00000118298	CA14	-1.5	3.8E-13	6.0E-12	20.834	0.924	19.861	21.701	32.356	0.570	31.698	32.690
ENSG00000118495	PLAGL1	-1.5	3.5E-02	5.9E-02	0.326	0.104	0.242	0.443	0.494	0.058	0.454	0.561
ENSG00000118655	DCLRE1B	-1.5	1.3E-07	6.9E-07	6.147	0.224	5.891	6.307	9.545	0.567	9.076	10.175
ENSG00000119431	HDHD3	-1.5	4.8E-06	1.8E-05	8.682	0.258	8.405	8.916	12.794	1.318	11.281	13.692
ENSG00000119574	ZBTB45	-1.5	3.1E-05	1.0E-04	4.957	0.441	4.534	5.414	7.267	1.001	6.130	8.019
ENSG00000119596	YLPM1	-1.5	9.2E-15	1.9E-13	17.300	0.654	16.549	17.749	25.206	0.299	24.887	25.480
ENSG00000120278	PLEKHG1	-1.5	7.5E-03	1.5E-02	0.670	0.145	0.578	0.837	0.885	0.038	0.961	1.029
ENSG00000120694	HSPH1	-1.5	7.8E-18	2.9E-16	55.911	2.947	53.146	59.010	86.312	1.431	85.263	87.942
ENSG00000120942	UBIAD1	-1.5	2.6E-10	2.3E-09	11.154	0.394	10.744	11.530	16.635	0.979	15.668	17.624
ENSG00000121281	ADCY7	-1.5	6.9E-05	2.1E-04	1.316	0.208	1.147	1.549	2.016	0.112	1.890	2.104
ENSG00000121310	ECHDC2	-1.5	2.1E-04	5.8E-04	0.907	0.120	0.774	1.008	1.339	0.085	1.288	1.438
ENSG00000121871	SLITRK3	-1.5	5.2E-04	1.3E-03	1.292	0.120	1.199	1.427	1.899	0.248	1.784	2.270
ENSG00000121898	CPXM2	-1.5	4.7E-05	1.5E-04	3.024	0.307	2.672	3.240	4.506	0.203	4.312	4.716
ENSG00000122547	EEPD1	-1.5	9.2E-06	3.3E-05	3.664	0.409	3.416	4.136	5.449	0.327	5.216	5.824
ENSG00000122779	TRIM24	-1.5	3.8E-17	1.2E-15	42.350	2.037	40.282	44.353	65.687	2.371	63.960	68.398
ENSG00000124608	AARS2	-1.5	1.6E-09	1.2E-08	14.950	0.115	14.880	15.083	22.293	1.888	20.804	24.430
ENSG00000125170	DOK4	-1.5	4.2E-10	3.6E-09	11.293	0.414	10.869	11.696	16.767	0.613	16.221	17.430
ENSG00000126351	THRA	-1.5	7.1E-07	3.2E-06	3.342	0.228	3.083	3.514	5.080	0.436	4.577	5.334
ENSG00000126464	PRR12	-1.5	1.5E-08	9.6E-08	13.670	0.570	13.227	14.313	20.739	1.418	19.398	22.224
ENSG00000127399	LRRC61	-1.5	9.3E-10	7.4E-09	21.659	0.988	21.069	22.800	31.754	0.805	31.062	32.636
ENSG00000127419	TMEM175	-1.5	3.0E-03	6.4E-03	1.423	0.165	1.321	1.613	2.158	0.166	2.005	2.334
ENSG00000127831	VIL1	-1.5	2.0E-05	6.7E-05	2.109	0.349	1.851	2.507	3.282	0.182	3.088	3.448
ENSG00000128309	MPST	-1.5	4.5E-11	4.7E-10	13.799	0.703	13.125	14.527	20.077	0.680	19.362	20.717
ENSG00000128578	STRIP2	-1.5	2.7E-07	1.3E-06	4.070	0.366	3.651	4.331	6.224	0.659	5.695	6.962
ENSG00000128602	SMO	-1.5	1.0E-12	1.4E-11	32.934	0.816	32.042	33.642	49.987	2.661	47.600	52.856
ENSG00000129691	ASH2L	-1.5	6.3E-14	1.2E-12	17.775	0.555	17.303	18.386	27.113	1.308	26.283	28.499
ENSG00000130182	ZSCAN10	-1.5	1.8E-16	5.1E-15	87.368	2.996	83.987	89.694	134.910	4.995	129.240	138.663
ENSG00000130511	SSBP4	-1.5	5.8E-07	2.6E-06	8.293	0.316	7.936	8.537	12.759	1.899	10.639	14.306
ENSG00000130584	ZBTB46	-1.5	5.3E-10	4.4E-09	10.096	0.573	9.738	10.757	15.497	0.251	15.222	15.714
ENSG00000130748	TMEM160	-1.5	1.3E-03	3.0E-03	63.691	2.422	62.159	66.483	96.144	9.058	86.173	103.866
ENSG00000130830	MPP1	-1.5	1.1E-06	4.7E-06	4.581	0.493	4.272	5.149	6.734	0.380	6.317	7.061
ENSG00000130881	LRP3	-1.5	1.4E-10	1.3E-09	12.904	0.845	12.251	13.858	19.867	1.721	18.543	21.813
ENSG00000131323	TRAF3	-1.5	2.6E-07	1.3E-06	3.734	0.560	3.320	4.371	5.617	0.417	5.149	5.951
ENSG00000131477	RAMP2	-1.5	3.3E-05	1.1E-04	10.259	0.886	9.289	11.025	15.480	0.747	14.696	16.183
ENSG00000131584	ACAP3	-1.5	4.5E-07	2.1E-06	2.968	0.307	2.628	3.225	4.449	0.171	4.325	4.644
ENSG00000132768	DPH2	-1.5	5.9E-13	8.9E-12	38.476	0.294	38.139	38.677	58.316	1.275	57.544	59.788
ENSG00000132906	CASP9	-1.5	1.5E-08	9.4E-08	6.327	0.531	5.730	6.746	9.796	0.379	9.438	10.193
ENSG00000133119	RFC3	-1.5	4.1E-13	6.4E-12	56.714	0.742	55.857	57.142	83.614	1.442	82.036	84.863
ENSG00000133302	SLF1	-1.5	4.5E-11	4.7E-10	11.245	0.272	10.934	11.438	16.397	0.871	15.830	17.400
ENSG00000133433	GSTT2B	-1.5	1.5E-03	3.5E-03	4.837	0.861	4.283	5.828	7.443	1.321	6.204	8.834
ENSG00000133980	VRTN	-1.5	4.8E-14	9.0E-13	100.642	1.881	98.495	102.004	147.071	5.741	142.399	153.481
ENSG00000134262	AP4B1	-1.5	4.6E-08	2.6E-07	4.427	0.146	4.341	4.595	6.709	0.161	6.527	6.835
ENSG00000134853	PDGFRA	-1.5	2.0E-02	3.5E-02	0.310	0.037	0.278	0.350	0.466	0.063	0.404	0.531
ENSG00000134873	CLDN10	-1.5	6.5E-10	5.3E-09	21.205	1.560	19.408	22.211	32.210	0.767	31.451	32.985
ENSG00000135097	MSI1	-1.5	3.9E-06	1.5E-05	9.573	0.549	9.141	10.191	14.138	1.571	12.387	15.423
ENSG00000136371	MTHFS	-1.5	1.2E-03	2.9E-03	3.914	0.527	3.309	4.274	5.835	0.629	5.132	6.346
ENSG00000136492	BRIP1	-1.5	2.5E-12	3.4E-11	14.673	0.480	14.146	15.085	21.500	1.159	20.699	22.830
ENSG00000136859	ANGPTL2	-1.5	4.2E-06	1.6E-05	6.154	0.543	5.594	6.678	8.970	0.605	8.297	9.467
ENSG00000137275	RIPK1	-1.5	1.0E-08	6.6E-08	11.313	0.237	11.163	11.586	16.674	0.657	16.282	17.432
ENSG00000137628	DDX60	-1.5	9.2E-05	2.7E-04	1.871	0.034	1.833	1.898	2.758	0.473	2.338	3.270
ENSG00000138386	NAB1	-1.5	1.8E-09	1.3E-08	20.947	1.092	20.228	22.203	30.560	2.599	28.395	33.443
ENSG00000138642	HERC6	-1.5	1.0E-05	3.7E-05	2.026	0.239	1.765	2.234	3.155	0.164	2.980	3.319
ENSG00000139737	SLAIN1	-1.5	8.7E-11	8.5E-10	13.832	0.814	12.959	14.571	21.107	0.858	20.357	22.043
ENSG00000139767	SRRM4	-1.5	1.7E-03	3.8E-03	0.711	0.089	0.629	0.805	1.053	0.036	1.018	1.091
ENSG00000139971	C14orf37	-1.5	4.1E-09	2.9E-08	4.976	0.117	4.849	5.080	7.553	0.338	7.220	7.895
ENSG00000140526	ABHD2	-1.5	9.6E-13	1.4E-11	13.103	1.023	12.175	14.200	19.627	0.362	19.337	20.032
ENSG00000140832	MARVELD3	-1.5	4.1E-08	2.4E-07	10.185	1.115	9.468	11.470	14.818	1.653	13.196	16.501
ENSG00000141401	IMPA2	-1.5	7.7E-14	1.4E-12	30.375	0.417	30.087	30.854	46.389	2.131	43.960	47.950
ENSG00000141404	GNAL	-1.5	2.3E-11	2.6E-10	7.302	0.256	7.112	7.593	11.249	0.363	10.932	11.645
ENSG00000141642	ELAC1	-1.5	1.1E-03	2.5E-03	1.916	0.323	1.612	2.255	2.859	0.273	2.610	3.152
ENSG00000141873	SLC39A3	-1.5	4.3E-08	2.4E-07	7.396	0.321	7.184	7.765	11.219	1.140	10.403	12.522
ENSG00000142619	PADI3	-1.5	1.4E-02	2.6E-02	0.808	0.116	0.713	0.936	1.248	0.072	1.183	1.326
ENSG00000142621	FHAD1	-1.5	2.1E-02	3.7E-02	0.190	0.011	0.177	0.200	0.291	0.023	0.266	0.311
ENSG00000142632	ARHGEF19	-1.5	6.7E-12	8.3E-11	28.600	1.078	27.564	29.716	42.775	1.719	40.993	44.422
ENSG00000142634	EFHD2	-1.5	5.9E-08	3.3E-07	17.699	0.413	17.415	18.173	26.845	1.844	24.781	28.330
ENSG00000143147	GPR161	-1.5	3.3E-10	2.9E-09	4.515	0.121	4.383	4.621	6.840	0.416	6.476	7.293
ENSG00000143393	PI4KB	-1.5	2.1E-12	2.8E-11	12.800	0.281	12.509	13.070	19.574	0.468	19.202	20.099
ENSG00000143416	SELENBP1	-1.5	3.0E-07	1.5E-06	7.804	0.472	7.444	8.338	11.501	0.440	11.034	11.908
ENSG00000143469	SYT14	-1.5	6.6E-10	5.4E-09	4.221	0.298	3.992	4.557	6.413	0.493	5.885	6.861
ENSG00000143845	ETNK2	-1.5	1.4E-07	7.4E-07	6.832	0.999	6.247	7.985	10.467	0.608	10.027	11.161
ENSG00000144559	TAMM41	-1.5	2.6E-06	1.0E-05	3.737	0.484	3.205	4.152	5.557	0.505	5.149	6.122
ENSG00000146197	SCUBE3	-1.5	8.3E-02	1.2E-01	0.322	0.156	0.147	0.445	0.478	0.121	0.342	0.573
ENSG00000146352	CLVS2	-1.5	1.1E-02	2.0E-02	0.297	0.050	0.242	0.339	0.456	0.096	0.377	0.563
ENSG00000146707	POM121C	-1.5	2.9E-05	9.4E-05	7.810	1.283	6.862	9.270	11.721	0.435	11.222	12.017
ENSG00000146828	SLC12A9	-1.5	6.2E-10	5.1E-09	7.418	0.217	7.172	7.584	11.013	0.670	10.272	11.576
ENSG00000147255	IGSF1	-1.5	3.8E-11	4.1E-10	11.261	0.385	10.967	11.696	16.529	0.568	15.897	16.998
ENSG00000148426	PROSER2	-1.5	6.2E-06	2.3E-05	3.659	0.222	3.413	3.843	5.659	0.202	5.489	5.882
ENSG00000148719	DNAJB12	-1.5	2.1E-08	1.3E-07	7.255	0.169	7.069	7.400	10.586	0.285	10.262	10.801
ENSG00000149782	PLCB3	-1.5	3.1E-15	7.2E-14	41.534	1.176	40.328	42.679	62.981	2.233	61.458	65.544
ENSG00000150054	MPP7	-1.5	9.1E-08	4.8E-07	5.593	0.322	5.290	5.932	8.351	0.500	7.805	8.789
ENSG00000150455	TIRAP	-1.5	6.3E-04	1.6E-03	1.836	0.256	1.544	2.020	2.831	0.412	2.545	3.304
ENSG00000150527	CTAGE5	-1.5	8.7E-03	1.7E-02	0.617	0.136	0.463	0.720	0.899	0.092	0.825	1.002
ENSG00000150625	GPM6A	-1.5	2.1E-04	5.7E-04	1.410	0.209	1.176	1.579	2.166	0.327	1.918	2.536
ENSG00000150867	PIP4K2A	-1.5	4.8E-13	7.5E-12	19.684	0.333	19.324	19.981	29.859	0.440	29.586	30.368
ENSG00000150893	FREM2	-1.5	3.9E-16	1.1E-14	13.112	0.487	12.587	13.550	20.238	0.442	19.759	20.631
ENSG00000150961	SEC24D	-1.5	3.6E-14	7.0E-13	23.439	0.724	22.630	24.023	34.528	0.888	33.527	35.219
ENSG00000151117	TMEM86A	-1.5	1.2E-01	1.7E-01	0.410	0.184	0.268	0.618	0.601	0.136	0.514	0.757
ENSG00000151576	QTRT2	-1.5	1.9E-13	3.2E-12	17.225	0.336	17.010	17.612	26.125	1.017	25.015	27.013
ENSG00000151962	RBM46	-1.5	9.0E-03	1.7E-02	0.982	0.221	0.728	1.130	1.505	0.047	1.466	1.558
ENSG00000152932	RAB3C	-1.5	2.7E-08	1.6E-07	3.050	0.317	2.723	3.357	4.743	0.294	4.547	5.081
ENSG00000155287	SLC25A28	-1.5	2.9E-07	1.4E-06	10.495	0.372	10.202	10.914	16.256	0.983	15.151	17.033
ENSG00000156453	PCDH1	-1.5	1.2E-14	2.4E-13	52.334	0.179	52.164	52.521	78.444	1.270	77.586	79.905
ENSG00000157617	C2CD2	-1.5	1.2E-11	1.4E-10	6.683	0.136	6.562	6.831	10.285	0.201	10.066	10.460
ENSG00000157657	ZNF618	-1.5	2.6E-12	3.5E-11	11.866	0.517	11.502	12.458	18.256	0.553	17.838	18.884
ENSG00000157890	MEGF11	-1.5	1.8E-02	3.1E-02	0.318	0.076	0.231	0.376	0.489	0.107	0.412	0.610
ENSG00000158234	FAIM	-1.5	1.1E-05	3.9E-05	4.039	0.155	3.876	4.186	5.945	0.196	5.729	6.111
ENSG00000158480	SPATA2	-1.5	1.3E-08	8.0E-08	8.627	0.465	8.092	8.933	12.964	0.418	12.497	13.302
ENSG00000159079	C21orf59	-1.5	4.7E-09	3.2E-08	13.964	0.526	13.373	14.378	20.501	0.165	20.334	20.664
ENSG00000159445	THEM4	-1.5	1.6E-06	6.7E-06	6.085	0.793	5.546	6.996	8.915	0.440	8.589	9.416
ENSG00000159917	ZNF235	-1.5	2.9E-03	6.2E-03	1.192	0.092	1.092	1.274	1.756	0.178	1.571	1.925
ENSG00000160072	ATAD3B	-1.5	2.2E-12	2.9E-11	11.694	0.483	11.148	12.069	17.807	0.728	17.041	18.490
ENSG00000160392	C19orf47	-1.5	2.5E-06	1.0E-05	4.582	0.209	4.404	4.813	6.675	0.188	6.504	6.893
ENSG00000160688	FLAD1	-1.5	8.5E-14	1.5E-12	29.515	0.554	28.877	29.879	45.615	0.801	44.787	46.386
ENSG00000160766	GBAP1	-1.5	9.7E-04	2.3E-03	1.906	0.531	1.390	2.450	2.921	0.282	2.615	3.171
ENSG00000160818	GPATCH4	-1.5	2.5E-14	5.0E-13	34.757	1.546	33.291	36.372	52.103	0.357	51.789	52.492
ENSG00000161048	NAPEPLD	-1.5	2.4E-07	1.2E-06	3.240	0.066	3.164	3.285	4.994	0.765	4.508	5.876
ENSG00000161547	SRSF2	-1.5	2.0E-17	6.9E-16	260.521	9.261	252.320	270.565	383.496	8.279	378.307	393.044
ENSG00000161682	FAM171A2	-1.5	8.5E-08	4.6E-07	10.111	0.879	9.539	11.123	15.023	1.467	13.844	16.666
ENSG00000162062	C16orf59	-1.5	1.2E-09	9.4E-09	19.577	1.174	18.820	20.930	28.640	1.237	27.262	29.655
ENSG00000162066	AMDHD2	-1.5	4.6E-08	2.6E-07	6.374	0.303	6.045	6.644	9.824	0.311	9.489	10.119
ENSG00000162174	ASRGL1	-1.5	5.1E-08	2.9E-07	8.627	0.324	8.278	8.919	12.620	0.799	11.788	13.381
ENSG00000162344	FGF19	-1.5	1.1E-06	4.7E-06	9.245	1.652	7.429	10.660	14.132	0.273	13.820	14.328
ENSG00000162408	NOL9	-1.5	1.2E-08	7.7E-08	7.130	0.549	6.610	7.704	10.564	1.121	9.408	11.645
ENSG00000162882	HAAO	-1.5	3.6E-02	6.0E-02	1.077	0.407	0.626	1.416	1.599	0.446	1.251	2.101
ENSG00000163006	CCDC138	-1.5	2.4E-09	1.8E-08	11.948	1.086	11.105	13.174	17.688	1.601	16.563	19.522
ENSG00000163319	MRPS18C	-1.5	2.3E-10	2.1E-09	12.882	0.611	12.376	13.560	18.888	0.614	18.524	19.607
ENSG00000163482	STK36	-1.5	1.4E-09	1.1E-08	8.090	1.167	6.764	8.961	12.481	0.562	11.875	12.986
ENSG00000163701	IL17RE	-1.5	2.5E-03	5.4E-03	1.911	0.472	1.452	2.395	2.821	0.068	2.766	2.898
ENSG00000163864	NMNAT3	-1.5	6.3E-03	1.3E-02	1.212	0.239	1.035	1.483	1.848	0.273	1.589	2.134
ENSG00000164007	CLDN19	-1.5	1.8E-06	7.6E-06	6.614	0.566	5.961	6.942	9.684	1.045	9.048	10.890
ENSG00000164045	CDC25A	-1.5	1.4E-13	2.4E-12	50.756	1.355	49.263	51.908	76.300	1.061	75.584	77.519
ENSG00000164128	NPY1R	-1.5	1.2E-05	4.3E-05	3.476	0.316	3.145	3.776	5.203	0.104	5.112	5.317
ENSG00000164344	KLKB1	-1.5	7.1E-07	3.2E-06	5.811	0.557	5.175	6.211	8.923	0.439	8.450	9.319
ENSG00000164627	KIF6	-1.5	4.8E-02	7.7E-02	0.229	0.055	0.178	0.288	0.347	0.070	0.272	0.412
ENSG00000165118	C9orf64	-1.5	1.9E-07	9.6E-07	14.611	0.705	13.918	15.327	21.284	1.358	19.758	22.359
ENSG00000165186	PTCHD1	-1.5	1.5E-05	5.2E-05	1.505	0.085	1.407	1.562	2.213	0.213	2.018	2.440
ENSG00000165238	WNK2	-1.5	3.1E-08	1.8E-07	8.945	0.068	8.867	8.993	13.030	1.225	12.288	14.444
ENSG00000165379	LRFN5	-1.5	8.5E-02	1.3E-01	0.237	0.076	0.185	0.324	0.362	0.085	0.296	0.458
ENSG00000165449	SLC16A9	-1.5	8.0E-08	4.3E-07	11.809	0.250	11.618	12.093	17.200	0.515	16.665	17.694
ENSG00000165458	INPPL1	-1.5	1.3E-13	2.2E-12	14.392	0.114	14.267	14.490	22.192	0.793	21.278	22.696
ENSG00000165506	DNAAF2	-1.5	4.8E-12	6.1E-11	36.295	1.172	35.463	37.635	55.075	2.420	52.281	56.479
ENSG00000165671	NSD1	-1.5	2.1E-17	7.1E-16	34.113	1.773	32.167	35.635	51.676	0.559	51.228	52.303
ENSG00000165806	CASP7	-1.5	4.6E-05	1.4E-04	3.700	0.428	3.412	4.191	5.582	0.700	4.902	6.300
ENSG00000166105	GLB1L3	-1.5	1.1E-08	6.9E-08	4.749	0.142	4.658	4.913	7.075	0.700	6.311	7.686
ENSG00000166167	BTRC	-1.5	8.6E-12	1.0E-10	14.689	0.916	13.696	15.502	21.493	0.538	20.950	22.025
ENSG00000166664	CHRFAM7A	-1.5	9.8E-04	2.4E-03	0.771	0.047	0.720	0.813	1.186	0.020	1.165	1.205
ENSG00000166736	HTR3A	-1.5	1.6E-06	6.6E-06	7.164	0.390	6.904	7.612	10.890	0.326	10.527	11.158
ENSG00000167100	SAMD14	-1.5	1.5E-03	3.4E-03	1.081	0.159	0.946	1.256	1.606	0.377	1.374	2.041
ENSG00000167173	C15orf39	-1.5	1.3E-11	1.5E-10	11.139	0.534	10.557	11.605	17.230	0.446	16.715	17.503
ENSG00000167703	SLC43A2	-1.5	1.5E-06	6.5E-06	2.091	0.045	2.051	2.139	3.115	0.110	3.036	3.240
ENSG00000167748	KLK1	-1.5	5.4E-02	8.6E-02	0.874	0.197	0.649	1.016	1.341	0.232	1.175	1.606
ENSG00000168016	TRANK1	-1.5	1.8E-10	1.7E-09	4.785	0.323	4.557	5.156	7.226	0.361	6.824	7.522
ENSG00000168395	ING5	-1.5	5.8E-13	8.8E-12	9.911	0.164	9.731	10.050	14.674	0.277	14.488	14.993
ENSG00000168824		-1.5	5.4E-10	4.5E-09	15.732	0.645	15.027	16.292	23.291	1.109	22.607	24.570
ENSG00000169330	KIAA1024	-1.5	2.0E-02	3.5E-02	0.551	0.048	0.522	0.606	0.825	0.111	0.742	0.951
ENSG00000169598	DFFB	-1.5	1.4E-06	6.0E-06	5.537	0.183	5.327	5.663	8.348	0.322	8.065	8.699
ENSG00000170925	TEX13B	-1.5	1.4E-01	2.0E-01	0.903	0.233	0.742	1.171	1.371	0.615	0.789	2.015
ENSG00000170989	S1PR1	-1.5	3.9E-02	6.3E-02	0.933	0.154	0.833	1.110	1.357	0.256	1.092	1.602
ENSG00000171848	RRM2	-1.5	6.2E-17	1.9E-15	98.682	1.872	96.628	100.291	144.530	1.765	142.508	145.760
ENSG00000171853	TRAPPC12	-1.5	3.3E-10	2.9E-09	7.368	0.175	7.169	7.501	10.943	0.288	10.743	11.273
ENSG00000172081	MOB3A	-1.5	1.4E-04	4.0E-04	3.073	0.412	2.739	3.534	4.494	0.196	4.309	4.700
ENSG00000172339	ALG14	-1.5	1.4E-03	3.3E-03	0.742	0.059	0.674	0.782	1.114	0.054	1.073	1.175
ENSG00000173068	BNC2	-1.5	1.7E-08	1.1E-07	3.143	0.055	3.083	3.192	4.671	0.206	4.540	4.909
ENSG00000173409	ARV1	-1.5	1.6E-08	1.0E-07	15.398	1.363	14.448	16.959	23.772	1.305	22.372	24.957
ENSG00000173621	LRFN4	-1.5	1.6E-10	1.5E-09	23.638	0.536	23.032	24.049	35.673	2.287	34.033	38.286
ENSG00000173638	SLC19A1	-1.5	5.6E-09	3.8E-08	5.730	0.523	5.134	6.113	8.572	0.140	8.423	8.702
ENSG00000173826	KCNH6	-1.5	1.9E-02	3.3E-02	0.804	0.163	0.617	0.916	1.209	0.129	1.062	1.302
ENSG00000173898	SPTBN2	-1.5	1.4E-10	1.3E-09	11.023	0.594	10.338	11.387	16.087	0.771	15.380	16.909
ENSG00000174640	SLCO2A1	-1.5	2.9E-02	4.9E-02	0.629	0.204	0.399	0.789	0.937	0.125	0.793	1.011
ENSG00000174705	SH3PXD2B	-1.5	2.1E-14	4.2E-13	30.410	0.862	29.420	30.999	44.904	2.263	43.252	47.484
ENSG00000174744	BRMS1	-1.5	1.5E-11	1.7E-10	28.284	2.052	26.339	30.428	43.209	0.949	42.118	43.844
ENSG00000174807	CD248	-1.5	7.4E-04	1.8E-03	3.326	0.334	3.017	3.680	4.976	0.373	4.587	5.331
ENSG00000175048	ZDHHC14	-1.5	7.4E-05	2.2E-04	1.540	0.137	1.394	1.665	2.247	0.227	1.985	2.391
ENSG00000175344	CHRNA7	-1.5	3.6E-04	9.4E-04	0.332	0.017	0.313	0.347	0.508	0.042	0.459	0.535
ENSG00000175785	PRIMA1	-1.5	6.6E-04	1.6E-03	2.004	0.534	1.644	2.617	3.054	0.239	2.890	3.328
ENSG00000176108	CHMP6	-1.5	4.5E-07	2.1E-06	12.448	1.193	11.113	13.409	19.194	1.413	18.122	20.796
ENSG00000176402	GJC3	-1.5	6.4E-02	9.9E-02	2.571	0.467	2.039	2.909	3.739	1.326	2.810	5.257
ENSG00000176533	GNG7	-1.5	2.4E-06	9.6E-06	4.213	0.245	3.959	4.449	6.511	0.538	5.890	6.843
ENSG00000176542	USF3	-1.5	3.0E-09	2.2E-08	5.051	0.623	4.404	5.648	7.385	0.357	7.008	7.718
ENSG00000176771	NCKAP5	-1.5	3.4E-02	5.7E-02	0.295	0.067	0.223	0.355	0.437	0.058	0.371	0.483
ENSG00000177058	SLC38A9	-1.5	2.4E-11	2.7E-10	10.306	0.475	9.954	10.846	15.518	0.247	15.316	15.793
ENSG00000177082	WDR73	-1.5	2.2E-09	1.6E-08	4.651	0.361	4.249	4.948	6.913	0.259	6.631	7.140
ENSG00000177084	POLE	-1.5	2.1E-14	4.2E-13	10.769	0.053	10.721	10.826	16.391	0.852	15.838	17.373
ENSG00000177108	ZDHHC22	-1.5	2.9E-12	3.9E-11	24.828	1.399	23.620	26.361	38.576	2.447	35.922	40.499
ENSG00000177192	PUS1	-1.5	2.4E-12	3.2E-11	18.530	1.133	17.449	19.709	28.174	0.032	28.143	28.207
ENSG00000177238	TRIM72	-1.5	1.7E-02	3.1E-02	0.305	0.028	0.272	0.325	0.473	0.129	0.329	0.578
ENSG00000177352	CCDC71	-1.5	1.3E-08	8.2E-08	23.398	1.484	21.782	24.699	34.958	0.489	34.667	35.523
ENSG00000177406		-1.5	4.8E-04	1.2E-03	1.710	0.182	1.517	1.880	2.650	0.257	2.483	2.947
ENSG00000177628	GBA	-1.5	5.9E-07	2.7E-06	5.896	1.099	5.012	7.126	9.224	0.478	8.687	9.630
ENSG00000178184	PARD6G	-1.5	2.0E-09	1.5E-08	9.899	0.261	9.645	10.166	15.117	0.886	14.121	15.819
ENSG00000178343	SHISA3	-1.5	3.7E-03	7.7E-03	2.862	0.172	2.663	2.965	4.276	0.252	4.111	4.565
ENSG00000178685	PARP10	-1.5	2.4E-02	4.1E-02	0.528	0.078	0.461	0.613	0.782	0.185	0.583	0.949
ENSG00000179041	RRS1	-1.5	2.4E-11	2.6E-10	57.074	4.448	53.108	61.884	84.598	4.004	80.021	87.448
ENSG00000179059	ZFP42	-1.5	1.9E-17	6.4E-16	178.753	8.387	172.892	188.360	273.022	3.779	268.669	275.458
ENSG00000179603	GRM8	-1.5	4.5E-03	9.3E-03	0.627	0.134	0.528	0.779	0.967	0.183	0.757	1.098
ENSG00000180011	ZADH2	-1.5	1.0E-05	3.7E-05	1.573	0.087	1.514	1.673	2.385	0.237	2.209	2.654
ENSG00000180543	TSPYL5	-1.5	3.1E-10	2.7E-09	11.975	0.826	11.168	12.819	18.214	0.737	17.543	19.002
ENSG00000181274	FRAT2	-1.5	5.5E-13	8.4E-12	41.095	1.706	39.895	43.048	63.778	1.571	62.728	65.583
ENSG00000181396	OGFOD3	-1.5	7.8E-08	4.2E-07	8.113	0.182	7.943	8.305	11.847	1.568	10.076	13.058
ENSG00000182173	TSEN54	-1.5	4.6E-09	3.2E-08	16.128	0.665	15.417	16.736	23.882	1.947	21.847	25.726
ENSG00000182541	LIMK2	-1.5	2.4E-12	3.3E-11	15.699	0.452	15.322	16.201	23.491	1.098	22.490	24.666
ENSG00000182810	DDX28	-1.5	1.5E-07	7.8E-07	13.656	1.314	12.198	14.750	20.488	1.154	19.321	21.627
ENSG00000183715	OPCML	-1.5	1.2E-04	3.6E-04	1.406	0.188	1.252	1.616	2.118	0.214	1.947	2.358
ENSG00000183741	CBX6	-1.5	2.9E-11	3.2E-10	7.751	0.080	7.703	7.843	11.928	0.393	11.673	12.380
ENSG00000183853	KIRREL	-1.5	1.5E-14	3.0E-13	19.838	0.820	19.235	20.772	30.655	0.441	30.193	31.071
ENSG00000184178	SCFD2	-1.5	6.4E-08	3.5E-07	8.426	0.079	8.335	8.482	12.772	0.259	12.500	13.015
ENSG00000184363	PKP3	-1.5	3.2E-04	8.6E-04	3.675	0.103	3.558	3.750	5.365	0.336	5.049	5.717
ENSG00000184497	TMEM255B	-1.5	7.8E-02	1.2E-01	0.248	0.067	0.178	0.312	0.381	0.095	0.289	0.479
ENSG00000184867	ARMCX2	-1.5	3.3E-16	9.0E-15	77.026	1.846	75.529	79.088	115.459	1.853	113.602	117.309
ENSG00000185090	MANEAL	-1.5	2.4E-03	5.2E-03	2.177	0.140	2.026	2.301	3.176	0.227	2.982	3.425
ENSG00000185275	CD24P4	-1.5	2.3E-13	3.8E-12	658.243	29.561	630.126	689.063	1018.611	6.024	1012.055	1023.903
ENSG00000185347	C14orf80	-1.5	6.9E-05	2.1E-04	1.741	0.210	1.521	1.939	2.563	0.111	2.435	2.628
ENSG00000185527	PDE6G	-1.5	1.4E-02	2.6E-02	2.613	0.560	2.145	3.234	3.880	0.934	2.842	4.652
ENSG00000186162	CIDECP	-1.5	3.4E-03	7.2E-03	2.033	0.450	1.516	2.333	3.042	0.328	2.687	3.334
ENSG00000186350	RXRA	-1.5	7.0E-11	7.0E-10	6.902	0.440	6.523	7.385	10.418	0.432	10.088	10.907
ENSG00000186684	CYP27C1	-1.5	5.3E-04	1.4E-03	1.904	0.241	1.626	2.058	2.920	0.607	2.459	3.608
ENSG00000187051	RPS19BP1	-1.5	9.2E-12	1.1E-10	28.653	1.096	27.848	29.902	42.571	2.395	41.094	45.333
ENSG00000187164	SHTN1	-1.5	1.6E-10	1.5E-09	7.339	0.229	7.121	7.578	10.778	0.199	10.549	10.911
ENSG00000187189	TSPYL4	-1.5	6.6E-11	6.7E-10	21.576	0.718	20.800	22.329	31.443	2.207	30.036	33.986
ENSG00000188033	ZNF490	-1.5	7.5E-02	1.1E-01	0.423	0.068	0.345	0.462	0.622	0.228	0.483	0.886
ENSG00000188549	C15orf52	-1.5	5.1E-02	8.2E-02	0.291	0.180	0.144	0.492	0.461	0.122	0.337	0.581
ENSG00000188636	RTL6	-1.5	8.2E-13	1.2E-11	18.990	1.283	17.930	20.416	29.257	0.777	28.599	30.114
ENSG00000188807	TMEM201	-1.5	5.8E-09	3.9E-08	6.736	0.337	6.524	7.124	10.048	0.565	9.565	10.669
ENSG00000189227	C15orf61	-1.5	1.7E-02	3.1E-02	0.781	0.133	0.675	0.930	1.183	0.215	0.964	1.394
ENSG00000189306	RRP7A	-1.5	9.2E-13	1.3E-11	31.139	1.564	29.356	32.282	46.377	2.293	44.398	48.889
ENSG00000189319	FAM53B	-1.5	1.5E-08	9.1E-08	5.873	0.275	5.710	6.191	8.959	1.039	7.763	9.637
ENSG00000196132	MYT1	-1.5	1.4E-02	2.5E-02	0.411	0.077	0.363	0.499	0.643	0.033	0.606	0.669
ENSG00000196139	AKR1C3	-1.5	3.0E-05	9.7E-05	3.531	0.212	3.319	3.744	5.219	0.138	5.134	5.379
ENSG00000196154	S100A4	-1.5	8.2E-05	2.4E-04	12.358	1.586	10.610	13.703	18.294	1.322	17.171	19.752
ENSG00000196218	RYR1	-1.5	1.1E-10	1.1E-09	3.985	0.151	3.887	4.158	6.010	0.299	5.671	6.235
ENSG00000196350	ZNF729	-1.5	9.9E-08	5.2E-07	8.780	1.024	7.604	9.472	13.345	1.407	12.261	14.934
ENSG00000196502	SULT1A1	-1.5	5.8E-05	1.8E-04	1.212	0.134	1.067	1.332	1.870	0.150	1.699	1.981
ENSG00000196730	DAPK1	-1.5	2.3E-16	6.5E-15	41.410	1.153	40.082	42.144	60.769	0.845	59.815	61.422
ENSG00000196922	ZNF252P	-1.5	3.6E-08	2.1E-07	4.652	0.102	4.539	4.738	6.861	0.605	6.347	7.528
ENSG00000197136	PCNX3	-1.5	1.8E-10	1.7E-09	11.235	0.141	11.097	11.379	17.461	1.499	16.313	19.158
ENSG00000197168	NEK5	-1.5	6.4E-02	9.9E-02	0.250	0.076	0.184	0.333	0.385	0.092	0.321	0.490
ENSG00000197299	BLM	-1.5	1.8E-13	3.1E-12	27.138	0.756	26.365	27.875	39.969	0.874	38.974	40.611
ENSG00000197362	ZNF786	-1.5	4.0E-07	1.9E-06	9.094	1.127	7.824	9.974	13.601	0.393	13.367	14.055
ENSG00000197375	SLC22A5	-1.5	1.4E-05	4.8E-05	1.140	0.073	1.085	1.223	1.742	0.118	1.610	1.836
ENSG00000197444	OGDHL	-1.5	7.3E-09	4.9E-08	4.628	0.167	4.509	4.819	7.184	0.391	6.766	7.541
ENSG00000197779	ZNF81	-1.5	1.1E-07	5.7E-07	3.299	0.294	2.962	3.499	4.909	0.290	4.603	5.179
ENSG00000197816	CCDC180	-1.5	1.3E-02	2.3E-02	0.214	0.054	0.174	0.275	0.324	0.026	0.296	0.348
ENSG00000197818	SLC9A8	-1.5	3.1E-07	1.5E-06	4.205	0.259	4.044	4.503	6.141	0.299	5.910	6.478
ENSG00000197859	ADAMTSL2	-1.5	6.2E-03	1.2E-02	0.878	0.101	0.764	0.957	1.305	0.128	1.172	1.429
ENSG00000197937	ZNF347	-1.5	7.6E-09	5.0E-08	5.125	0.589	4.498	5.668	7.633	0.414	7.188	8.022
ENSG00000198464	ZNF480	-1.5	2.4E-09	1.7E-08	18.584	2.467	16.182	21.110	27.458	1.847	26.195	29.578
ENSG00000198646	NCOA6	-1.5	8.8E-08	4.7E-07	2.171	0.170	1.998	2.338	3.365	0.179	3.213	3.562
ENSG00000198890	PRMT6	-1.5	3.8E-12	5.0E-11	39.433	0.638	38.917	40.145	57.883	2.411	55.150	59.709
ENSG00000203952	CCDC160	-1.5	9.2E-04	2.2E-03	4.562	0.893	3.535	5.163	6.883	1.080	6.009	8.090
ENSG00000204072	ARMCX7P	-1.5	4.5E-04	1.1E-03	7.140	1.381	5.977	8.667	10.701	0.704	10.151	11.494
ENSG00000204227	RING1	-1.5	1.6E-08	1.0E-07	17.257	1.460	15.961	18.839	26.233	1.868	24.827	28.353
ENSG00000204264	PSMB8	-1.5	1.7E-07	8.8E-07	10.102	0.208	9.872	10.278	15.566	0.888	14.972	16.586
ENSG00000204282	TNRC6C-AS1	-1.5	2.1E-02	3.6E-02	0.667	0.235	0.442	0.912	1.031	0.179	0.857	1.214
ENSG00000204366	ZBTB12	-1.5	8.1E-12	1.0E-10	31.988	0.863	30.992	32.516	49.300	0.727	48.872	50.139
ENSG00000204389	HSPA1A	-1.5	1.2E-10	1.1E-09	33.081	1.284	32.032	34.512	48.608	2.675	46.693	51.664
ENSG00000204439	C6orf47	-1.5	4.9E-09	3.3E-08	14.232	0.059	14.166	14.281	21.785	0.392	21.334	22.038
ENSG00000204967	PCDHA4	-1.5	7.2E-04	1.8E-03	0.566	0.031	0.531	0.592	0.845	0.048	0.805	0.898
ENSG00000205885	C1RL-AS1	-1.5	1.3E-02	2.4E-02	0.738	0.095	0.630	0.810	1.093	0.191	0.877	1.238
ENSG00000205978	NYNRIN	-1.5	3.7E-12	4.9E-11	10.990	0.645	10.283	11.547	16.804	0.888	15.839	17.587
ENSG00000206077	ZDHHC11B	-1.5	1.2E-01	1.7E-01	0.295	0.056	0.240	0.351	0.429	0.104	0.315	0.518
ENSG00000206337	HCP5	-1.5	5.3E-04	1.3E-03	2.348	0.444	2.017	2.853	3.643	0.079	3.561	3.719
ENSG00000211456	SACM1L	-1.5	1.3E-13	2.3E-12	26.390	1.427	25.235	27.985	38.741	2.138	37.156	41.173
ENSG00000213160	KLHL23	-1.5	9.7E-15	2.0E-13	39.649	3.048	36.143	41.663	60.180	1.366	59.208	61.743
ENSG00000213694	S1PR3	-1.5	3.0E-06	1.2E-05	2.438	0.324	2.188	2.803	3.763	0.349	3.458	4.144
ENSG00000214022	REPIN1	-1.5	5.2E-13	8.0E-12	35.404	2.265	32.902	37.313	51.966	2.166	49.487	53.497
ENSG00000214425	LRRC37A4P	-1.5	1.4E-08	8.8E-08	5.739	0.222	5.483	5.874	8.489	0.283	8.254	8.803
ENSG00000214776		-1.5	8.9E-02	1.3E-01	0.136	0.030	0.101	0.155	0.202	0.031	0.179	0.237
ENSG00000215014		-1.5	1.0E-01	1.5E-01	0.744	0.131	0.618	0.880	1.126	0.252	0.844	1.330
ENSG00000216895		-1.5	1.2E-02	2.2E-02	2.465	0.276	2.170	2.717	3.645	0.115	3.530	3.760
ENSG00000217648		-1.5	4.1E-02	6.6E-02	1.898	0.768	1.153	2.686	2.946	0.359	2.578	3.295
ENSG00000221990	EXOC3-AS1	-1.5	5.9E-02	9.2E-02	1.809	0.167	1.625	1.949	2.729	0.130	2.624	2.874
ENSG00000223547	ZNF844	-1.5	1.0E-07	5.3E-07	4.537	0.490	4.125	5.080	6.692	0.083	6.602	6.764
ENSG00000223984	HNRNPRP1	-1.5	1.6E-04	4.6E-04	6.914	0.846	5.954	7.550	10.236	0.809	9.313	10.820
ENSG00000224051	CPTP	-1.5	1.6E-04	4.6E-04	5.181	0.248	4.895	5.334	7.574	1.425	6.449	9.176
ENSG00000224597	SVIL-AS1	-1.5	2.0E-04	5.5E-04	2.013	0.305	1.708	2.318	3.037	0.283	2.741	3.303
ENSG00000225131	PSME2P2	-1.5	4.3E-03	8.9E-03	8.011	0.972	7.109	9.040	11.887	1.680	10.209	13.570
ENSG00000225548	LINC01980	-1.5	7.8E-03	1.5E-02	3.965	0.389	3.689	4.410	5.930	0.212	5.789	6.173
ENSG00000226465		-1.5	8.4E-02	1.3E-01	5.561	1.775	3.551	6.912	8.456	1.310	7.030	9.604
ENSG00000227057	WDR46	-1.5	5.3E-12	6.7E-11	44.085	2.788	41.218	46.787	64.505	2.502	61.660	66.364
ENSG00000227879	PSPC1P1	-1.5	4.7E-03	9.6E-03	7.413	1.299	6.575	8.910	11.247	1.457	9.751	12.662
ENSG00000229191		-1.5	2.0E-10	1.8E-09	18.486	0.573	17.952	19.091	27.060	0.332	26.679	27.288
ENSG00000230707		-1.5	7.1E-02	1.1E-01	2.260	0.940	1.189	2.951	3.502	0.522	2.900	3.829
ENSG00000231595		-1.5	3.4E-02	5.7E-02	1.496	0.658	0.803	2.112	2.322	0.478	1.863	2.817
ENSG00000232063		-1.5	4.7E-03	9.6E-03	1.874	0.274	1.644	2.177	2.895	0.489	2.334	3.231
ENSG00000232098		-1.5	8.7E-04	2.1E-03	2.141	0.286	1.855	2.426	3.184	0.255	2.976	3.468
ENSG00000232301	LNCPRESS1	-1.5	1.0E-12	1.5E-11	190.641	11.551	177.317	197.826	282.830	11.584	275.606	296.191
ENSG00000233058	LINC00884	-1.5	2.0E-03	4.4E-03	1.909	0.154	1.752	2.060	2.835	0.167	2.646	2.964
ENSG00000233757		-1.5	2.4E-03	5.1E-03	3.416	0.564	2.888	4.011	5.088	0.561	4.557	5.675
ENSG00000234337		-1.5	2.8E-06	1.1E-05	35.436	5.170	30.807	41.015	52.393	5.195	46.972	57.327
ENSG00000234383	CTBP2P8	-1.5	2.3E-02	4.0E-02	2.905	0.821	1.978	3.540	4.223	1.090	3.153	5.331
ENSG00000234996		-1.5	3.5E-03	7.3E-03	2.780	0.621	2.394	3.497	4.031	0.246	3.819	4.301
ENSG00000235559	NOP56P1	-1.5	9.6E-03	1.8E-02	8.671	1.879	7.200	10.788	13.522	2.160	11.364	15.684
ENSG00000235859		-1.5	7.1E-04	1.8E-03	6.111	0.736	5.262	6.568	9.188	0.228	8.943	9.393
ENSG00000236809	SNX25P1	-1.5	4.6E-02	7.4E-02	2.435	0.122	2.313	2.557	3.723	0.506	3.352	4.299
ENSG00000237594		-1.5	2.5E-02	4.2E-02	3.508	0.473	2.962	3.805	5.302	0.869	4.313	5.948
ENSG00000237940	LINC01238	-1.5	5.3E-02	8.4E-02	0.927	0.198	0.703	1.081	1.394	0.292	1.084	1.664
ENSG00000237978	KCNMB2-AS1	-1.5	2.1E-02	3.6E-02	2.096	0.175	1.920	2.270	3.109	0.409	2.809	3.575
ENSG00000239264	TXNDC5	-1.5	6.2E-06	2.3E-05	3.991	0.028	3.959	4.011	6.114	0.855	5.158	6.807
ENSG00000244462	RBM12	-1.5	9.5E-16	2.4E-14	58.948	3.261	57.046	62.713	89.624	2.335	87.288	91.958

ENSG00000249014	HMGN2P4	-1.5	3.4E-04	8.9E-04	32.245	6.938	24.339	37.319	47.044	3.091	43.630	49.652
ENSG00000249176		-1.5	3.9E-02	6.4E-02	2.804	1.099	2.101	4.070	4.310	0.471	3.802	4.733
ENSG00000250579		-1.5	2.6E-03	5.6E-03	5.574	0.896	4.638	6.424	8.444	1.268	7.209	9.742
ENSG00000250853	RNF138P1	-1.5	1.4E-03	3.2E-03	11.969	2.446	10.026	14.716	17.880	0.692	17.123	18.481
ENSG00000251602		-1.5	3.0E-02	5.1E-02	0.985	0.210	0.811	1.219	1.521	0.303	1.172	1.716
ENSG00000253251		-1.5	1.2E-02	2.3E-02	2.348	0.830	1.405	2.968	3.510	0.159	3.364	3.680
ENSG00000253477		-1.5	4.4E-02	7.1E-02	2.257	0.582	1.854	2.925	3.388	0.730	2.866	4.222
ENSG00000253636		-1.5	1.1E-04	3.3E-04	5.434	0.316	5.132	5.763	7.888	1.809	6.933	10.076
ENSG00000258670		-1.5	2.2E-02	3.9E-02	4.072	1.028	3.023	5.078	6.091	1.680	4.556	7.886
ENSG00000259408		-1.5	8.5E-02	1.3E-01	1.893	0.156	1.761	2.065	2.841	0.750	1.980	3.354
ENSG00000259456	ADNP-AS1	-1.5	8.3E-02	1.2E-01	1.933	0.652	1.332	2.627	2.893	0.692	2.123	3.466
ENSG00000259485	LINC02253	-1.5	8.6E-03	1.7E-02	4.812	1.618	3.019	6.165	7.140	0.277	6.825	7.345
ENSG00000260401		-1.5	5.7E-02	9.0E-02	0.664	0.062	0.597	0.719	1.031	0.363	0.627	1.330
ENSG00000260577		-1.5	6.5E-02	1.0E-01	0.938	0.025	0.918	0.965	1.456	0.312	1.163	1.784
ENSG00000260725		-1.5	3.4E-02	5.7E-02	2.113	0.539	1.551	2.626	3.127	0.530	2.779	3.737
ENSG00000260805		-1.5	9.6E-02	1.4E-01	0.795	0.188	0.598	0.973	1.215	0.380	0.962	1.652
ENSG00000261251		-1.5	1.4E-01	1.9E-01	0.685	0.185	0.492	0.861	1.025	0.313	0.776	1.377
ENSG00000261335		-1.5	1.4E-01	2.0E-01	0.829	0.021	0.809	0.851	1.239	0.528	0.737	1.790
ENSG00000261754		-1.5	2.8E-02	4.8E-02	0.981	0.358	0.589	1.289	1.511	0.123	1.368	1.583
ENSG00000263843		-1.5	6.2E-03	1.2E-02	2.006	0.181	1.832	2.194	2.964	0.352	2.653	3.346
ENSG00000267127		-1.5	1.1E-01	1.6E-01	0.397	0.199	0.237	0.619	0.617	0.055	0.576	0.680
ENSG00000267296	CEBPA-AS1	-1.5	7.9E-03	1.5E-02	2.165	0.282	1.896	2.458	3.270	0.700	2.544	3.940
ENSG00000271851		-1.5	4.4E-02	7.2E-02	0.934	0.218	0.684	1.078	1.402	0.301	1.066	1.648
ENSG00000272398	CD24	-1.5	9.0E-19	3.8E-17	1244.824	27.901	1226.144	1276.897	1884.971	23.003	1869.196	1911.365
ENSG00000273079	GRIN2B	-1.5	2.4E-05	8.1E-05	0.462	0.060	0.399	0.519	0.697	0.036	0.657	0.722
ENSG00000273274	ZBTB8B	-1.5	4.4E-07	2.0E-06	2.030	0.129	1.883	2.125	2.948	0.185	2.805	3.156
ENSG00000273983	HIST1H3G	-1.5	2.1E-02	3.7E-02	1.249	0.312	1.003	1.600	1.921	0.257	1.697	2.202
ENSG00000274864		-1.5	4.1E-03	8.5E-03	2.915	0.210	2.730	3.143	4.507	0.431	4.020	4.838
ENSG00000276875	HYDIN2	-1.5	1.5E-02	2.8E-02	0.181	0.033	0.159	0.219	0.281	0.020	0.258	0.295
ENSG00000277159		-1.5	2.5E-02	4.4E-02	0.672	0.197	0.452	0.832	1.059	0.127	0.912	1.136
ENSG00000277701		-1.5	1.3E-02	2.4E-02	1.021	0.063	0.981	1.094	1.566	0.247	1.306	1.799
ENSG00000282221		-1.5	5.5E-02	8.7E-02	3.736	2.087	2.302	6.131	5.573	0.670	4.804	6.030
ENSG00000001036	FUCA2	-1.4	5.3E-11	5.5E-10	49.952	3.028	46.476	52.014	69.849	3.112	66.301	72.120
ENSG00000004139	SARM1	-1.4	9.9E-04	2.4E-03	1.184	0.048	1.150	1.239	1.604	0.068	1.543	1.677
ENSG00000004866	ST7	-1.4	1.5E-07	7.8E-07	5.980	0.211	5.800	6.212	8.572	0.399	8.112	8.826
ENSG00000005007	UPF1	-1.4	1.2E-12	1.7E-11	28.540	0.122	28.411	28.652	40.058	2.586	37.570	42.732
ENSG00000005059	MCUB	-1.4	3.7E-06	1.4E-05	7.943	0.729	7.101	8.373	11.439	0.772	10.812	12.301
ENSG00000005100	DHX33	-1.4	8.8E-13	1.3E-11	51.860	2.735	48.873	54.240	72.888	1.312	71.374	73.698
ENSG00000006042	TMEM98	-1.4	8.0E-11	7.9E-10	27.696	1.140	26.412	28.587	38.290	0.823	37.344	38.841
ENSG00000007062	PROM1	-1.4	7.6E-14	1.4E-12	29.934	0.451	29.570	30.438	42.582	0.728	41.996	43.397
ENSG00000007520	TSR3	-1.4	2.0E-07	1.0E-06	55.685	3.266	52.162	58.610	75.914	6.135	69.788	82.059
ENSG00000010295	IFFO1	-1.4	4.6E-05	1.4E-04	4.473	0.206	4.236	4.598	6.152	0.454	5.713	6.620
ENSG00000011009	LYPLA2	-1.4	2.6E-11	2.8E-10	97.897	2.172	96.596	100.404	134.539	4.744	129.065	137.455
ENSG00000014164	ZC3H3	-1.4	2.3E-07	1.1E-06	10.583	0.531	10.137	11.170	15.062	1.302	14.302	16.566
ENSG00000015153	YAF2	-1.4	8.2E-02	1.2E-01	0.236	0.037	0.197	0.270	0.324	0.036	0.288	0.365
ENSG00000019505	SYT13	-1.4	7.8E-08	4.2E-07	10.270	0.484	9.883	10.814	14.089	0.571	13.469	14.593
ENSG00000027847	B4GALT7	-1.4	2.5E-05	8.3E-05	5.553	0.948	4.909	6.641	7.794	0.359	7.448	8.165
ENSG00000031081	ARHGAP31	-1.4	4.9E-06	1.9E-05	2.809	0.123	2.697	2.941	3.896	0.100	3.790	3.989
ENSG00000033011	ALG1	-1.4	6.5E-08	3.5E-07	12.875	0.997	12.287	14.026	17.719	0.478	17.217	18.168
ENSG00000038427	VCAN	-1.4	1.6E-15	3.9E-14	82.570	5.988	76.815	88.767	119.478	3.395	116.249	123.017
ENSG00000040487	PQLC2	-1.4	8.1E-05	2.4E-04	8.354	0.719	7.554	8.946	11.591	0.938	10.557	12.387
ENSG00000040531	CTNS	-1.4	1.1E-04	3.3E-04	7.219	0.810	6.544	8.118	9.885	0.614	9.332	10.551
ENSG00000040633	PHF23	-1.4	1.1E-09	8.6E-09	33.824	0.602	33.129	34.180	47.260	2.483	44.557	49.439
ENSG00000047617	ANO2	-1.4	1.0E-02	1.9E-02	1.043	0.127	0.934	1.182	1.436	0.023	1.417	1.461
ENSG00000048162	NOP16	-1.4	2.7E-10	2.4E-09	36.271	0.863	35.423	37.149	50.996	1.021	50.126	52.120
ENSG00000049192	ADAMTS6	-1.4	5.1E-03	1.0E-02	0.774	0.112	0.667	0.891	1.098	0.196	0.929	1.313
ENSG00000055118	KCNH2	-1.4	1.7E-04	4.7E-04	2.278	0.320	1.961	2.600	3.299	0.058	3.253	3.364
ENSG00000055130	CUL1	-1.4	7.5E-15	1.6E-13	80.825	2.936	78.480	84.118	117.193	1.683	115.262	118.349
ENSG00000056558	TRAF1	-1.4	2.0E-03	4.4E-03	1.204	0.062	1.133	1.245	1.728	0.288	1.408	1.966
ENSG00000056998	GYG2	-1.4	2.5E-13	4.0E-12	52.666	2.070	50.456	54.561	75.357	3.152	72.730	78.851
ENSG00000057757	PITHD1	-1.4	1.6E-10	1.5E-09	51.934	2.335	49.694	54.354	71.678	3.029	68.247	73.982
ENSG00000058262	SEC61A1	-1.4	2.1E-14	4.1E-13	101.033	4.163	97.350	105.550	143.180	3.057	141.161	146.698
ENSG00000058729	RIOK2	-1.4	6.2E-10	5.1E-09	18.115	1.746	16.355	19.847	25.934	1.732	24.301	27.750
ENSG00000059122	FLYWCH1	-1.4	8.8E-07	3.9E-06	5.043	0.428	4.748	5.533	7.105	0.723	6.413	7.856
ENSG00000059145	UNKL	-1.4	5.6E-09	3.8E-08	8.215	0.408	7.971	8.686	11.802	0.054	11.762	11.864
ENSG00000064490	RFXANK	-1.4	4.6E-08	2.6E-07	17.830	0.678	17.056	18.315	25.327	0.704	24.715	26.096
ENSG00000065320	NTN1	-1.4	8.7E-03	1.7E-02	0.914	0.101	0.814	1.016	1.276	0.077	1.189	1.335
ENSG00000065361	ERBB3	-1.4	3.6E-11	3.8E-10	22.936	1.517	21.232	24.141	31.163	0.592	30.542	31.720
ENSG00000066468	FGFR2	-1.4	6.6E-13	9.8E-12	23.860	0.592	23.342	24.505	33.086	0.190	32.867	33.209
ENSG00000067533	RRP15	-1.4	4.1E-11	4.3E-10	16.199	0.386	15.762	16.493	22.968	1.549	21.190	24.029
ENSG00000067596	DHX8	-1.4	3.2E-11	3.5E-10	20.722	0.859	19.945	21.644	28.114	0.910	27.076	28.777
ENSG00000068001	HYAL2	-1.4	3.8E-08	2.2E-07	17.421	2.324	14.807	19.250	24.244	1.468	22.559	25.242
ENSG00000068650	ATP11A	-1.4	2.2E-12	3.0E-11	14.170	0.300	13.903	14.495	19.621	0.709	18.869	20.276
ENSG00000069188	SDK2	-1.4	6.3E-03	1.3E-02	1.222	0.141	1.130	1.384	1.655	0.090	1.552	1.720
ENSG00000070413	DGCR2	-1.4	6.5E-11	6.5E-10	28.865	1.122	28.061	30.147	40.349	2.276	37.882	42.366
ENSG00000071073	MGAT4A	-1.4	2.9E-05	9.4E-05	1.926	0.255	1.635	2.107	2.735	0.144	2.648	2.901
ENSG00000072310	SREBF1	-1.4	1.2E-03	2.9E-03	16.380	0.551	15.839	16.939	22.334	1.137	21.194	23.469
ENSG00000073111	MCM2	-1.4	3.9E-14	7.5E-13	85.903	0.357	85.550	86.264	120.296	3.364	118.250	124.178
ENSG00000074071	MRPS34	-1.4	1.7E-11	2.0E-10	214.281	8.956	204.438	221.950	310.457	23.840	296.564	337.984
ENSG00000075131	TIPIN	-1.4	5.5E-08	3.1E-07	20.691	0.778	19.796	21.212	29.127	1.152	27.863	30.118
ENSG00000076003	MCM6	-1.4	2.8E-12	3.8E-11	139.121	7.226	130.865	144.293	198.751	1.997	196.927	200.886
ENSG00000077063	CTTNBP2	-1.4	6.4E-09	4.3E-08	13.350	0.994	12.440	14.411	18.418	0.307	18.182	18.766
ENSG00000078053	AMPH	-1.4	3.8E-05	1.2E-04	3.477	0.530	2.977	4.032	4.879	0.104	4.784	4.989
ENSG00000078549	ADCYAP1R1	-1.4	4.7E-08	2.7E-07	8.606	0.378	8.200	8.946	11.814	0.292	11.503	12.084
ENSG00000079215	SLC1A3	-1.4	6.1E-07	2.7E-06	9.259	0.261	8.965	9.460	12.610	0.136	12.455	12.709
ENSG00000079332	SAR1A	-1.4	5.2E-13	8.0E-12	46.162	1.319	44.677	47.197	63.053	2.271	60.430	64.389
ENSG00000080839	RBL1	-1.4	2.3E-06	9.2E-06	7.230	0.413	6.816	7.642	9.831	0.375	9.398	10.054
ENSG00000081052	COL4A4	-1.4	5.4E-03	1.1E-02	0.529	0.016	0.515	0.546	0.768	0.111	0.689	0.895
ENSG00000084674	APOB	-1.4	1.5E-02	2.8E-02	0.404	0.079	0.312	0.451	0.562	0.027	0.531	0.579
ENSG00000084710	EFR3B	-1.4	1.7E-06	6.9E-06	3.786	0.202	3.598	4.000	5.229	0.201	5.008	5.403
ENSG00000085491	SLC25A24	-1.4	2.5E-11	2.7E-10	20.816	0.087	20.716	20.872	29.104	0.914	28.051	29.695
ENSG00000085999	RAD54L	-1.4	7.4E-11	7.3E-10	34.184	0.873	33.205	34.883	47.465	0.997	46.316	48.104
ENSG00000086827	ZW10	-1.4	1.4E-11	1.6E-10	35.326	0.797	34.667	36.212	50.736	1.924	49.255	52.910
ENSG00000088035	ALG6	-1.4	1.3E-09	1.0E-08	18.121	1.030	17.412	19.303	25.870	2.084	23.939	28.079
ENSG00000088808	PPP1R13B	-1.4	1.5E-04	4.2E-04	2.057	0.148	1.897	2.188	2.940	0.243	2.662	3.110
ENSG00000088970	KIZ	-1.4	1.2E-10	1.1E-09	14.117	0.684	13.373	14.718	20.381	0.944	19.746	21.466
ENSG00000089050	RBBP9	-1.4	4.6E-09	3.2E-08	15.845	0.338	15.471	16.130	22.880	1.776	20.943	24.433
ENSG00000089057	SLC23A2	-1.4	8.3E-10	6.7E-09	9.424	0.362	9.007	9.653	13.394	0.396	12.946	13.699
ENSG00000089177	KIF16B	-1.4	5.7E-06	2.1E-05	4.555	0.381	4.118	4.814	6.220	0.248	5.954	6.446
ENSG00000090621	PABPC4	-1.4	1.7E-16	4.8E-15	74.429	0.505	73.884	74.881	106.988	1.551	105.755	108.729
ENSG00000093009	CDC45	-1.4	3.6E-08	2.1E-07	20.566	0.385	20.234	20.988	29.505	1.197	28.454	30.808
ENSG00000099783	HNRNPM	-1.4	5.3E-14	9.9E-13	196.234	5.760	189.585	199.680	274.394	4.480	269.365	277.958
ENSG00000099984	GSTT2	-1.4	2.9E-02	5.0E-02	2.618	0.500	2.042	2.933	3.740	0.650	2.994	4.188
ENSG00000100056	DGCR14	-1.4	3.5E-06	1.4E-05	5.772	0.266	5.489	6.016	7.930	0.457	7.493	8.405
ENSG00000100060	MFNG	-1.4	3.2E-02	5.4E-02	2.153	0.400	1.916	2.615	2.908	0.283	2.734	3.235
ENSG00000100065	CARD10	-1.4	2.3E-07	1.1E-06	8.946	0.137	8.793	9.056	12.449	0.810	11.715	13.318
ENSG00000100105	PATZ1	-1.4	7.7E-11	7.6E-10	27.051	1.063	26.217	28.248	37.668	2.297	35.210	39.761
ENSG00000100121	GGTLC2	-1.4	9.6E-02	1.4E-01	1.405	0.124	1.273	1.519	2.042	0.337	1.705	2.378
ENSG00000100162	CENPM	-1.4	4.1E-07	1.9E-06	20.276	0.247	20.053	20.541	28.524	1.844	26.398	29.681
ENSG00000100246	DNAL4	-1.4	2.8E-05	9.3E-05	11.170	0.503	10.591	11.497	15.205	1.002	14.371	16.316
ENSG00000100433	KCNK10	-1.4	5.5E-02	8.7E-02	0.338	0.031	0.314	0.374	0.482	0.049	0.429	0.524
ENSG00000100605	ITPK1	-1.4	2.3E-11	2.6E-10	31.122	0.788	30.224	31.698	43.995	1.561	42.197	44.999
ENSG00000100612	DHRS7	-1.4	2.6E-07	1.3E-06	16.988	0.547	16.542	17.598	23.289	1.522	21.805	24.846
ENSG00000100664	EIF5	-1.4	5.6E-04	1.4E-03	92.585	6.348	88.632	99.907	126.630	2.994	123.175	128.470
ENSG00000100888	CHD8	-1.4	9.6E-16	2.4E-14	38.506	1.650	37.373	40.398	55.959	0.933	54.999	56.864
ENSG00000100994	PYGB	-1.4	1.5E-11	1.8E-10	35.350	1.108	34.104	36.224	49.773	1.821	48.676	51.875
ENSG00000101160	CTSZ	-1.4	9.5E-08	5.0E-07	29.077	1.999	27.265	31.221	39.887	2.524	36.976	41.468
ENSG00000101188	NTSR1	-1.4	4.9E-02	7.8E-02	1.053	0.215	0.805	1.188	1.418	0.246	1.154	1.641
ENSG00000101191	DIDO1	-1.4	2.2E-12	3.0E-11	21.751	0.481	21.221	22.162	29.610	1.264	28.851	31.069
ENSG00000101194	SLC17A9	-1.4	5.3E-07	2.4E-06	6.718	0.565	6.262	7.350	9.715	0.450	9.218	10.095
ENSG00000101210	EEF1A2	-1.4	4.9E-05	1.5E-04	10.948	0.594	10.278	11.409	14.834	0.269	14.554	15.091
ENSG00000101311	FERMT1	-1.4	3.9E-07	1.9E-06	8.425	0.502	7.978	8.968	11.975	0.601	11.425	12.616
ENSG00000101346	POFUT1	-1.4	7.1E-10	5.8E-09	16.214	0.417	15.733	16.469	22.082	0.973	21.303	23.173
ENSG00000101353	MROH8	-1.4	5.4E-02	8.5E-02	0.685	0.088	0.587	0.756	0.990	0.058	0.943	1.055
ENSG00000101407	TTI1	-1.4	6.0E-11	6.1E-10	40.322	2.126	38.699	42.729	54.791	0.475	54.297	55.245
ENSG00000101811	CSTF2	-1.4	6.7E-09	4.5E-08	38.040	0.937	37.318	39.099	51.513	0.677	50.914	52.247
ENSG00000101825	MXRA5	-1.4	1.9E-03	4.2E-03	1.071	0.218	0.867	1.301	1.476	0.062	1.426	1.546
ENSG00000102043	MTMR8	-1.4	4.5E-03	9.2E-03	2.448	0.249	2.164	2.626	3.566	0.230	3.333	3.790
ENSG00000102743	SLC25A15	-1.4	5.7E-08	3.2E-07	27.819	2.357	25.291	29.954	38.975	1.190	37.858	40.226
ENSG00000102870	ZNF629	-1.4	8.5E-10	6.8E-09	12.479	0.325	12.273	12.853	17.714	1.094	17.028	18.975
ENSG00000102934	PLLP	-1.4	1.2E-02	2.2E-02	0.600	0.178	0.460	0.800	0.854	0.074	0.769	0.903
ENSG00000102978	POLR2C	-1.4	8.8E-13	1.3E-11	45.295	1.899	43.406	47.203	63.903	0.688	63.374	64.597
ENSG00000103042	SLC38A7	-1.4	1.4E-07	7.3E-07	8.329	0.406	8.010	8.786	11.387	0.661	10.819	12.113
ENSG00000103066	PLA2G15	-1.4	8.6E-05	2.6E-04	5.196	0.466	4.910	5.733	7.055	0.719	6.321	7.759
ENSG00000103460	TOX3	-1.4	1.1E-04	3.3E-04	4.361	0.377	3.988	4.743	5.887	0.667	5.258	6.567
ENSG00000103490	PYCARD	-1.4	1.6E-06	6.6E-06	15.511	0.372	15.147	15.890	22.466	2.936	19.427	25.286
ENSG00000103494	RPGRIP1L	-1.4	6.6E-07	3.0E-06	4.160	0.687	3.662	4.943	5.926	0.366	5.547	6.277
ENSG00000103657	HERC1	-1.4	8.6E-12	1.0E-10	12.452	0.544	12.040	13.069	16.925	0.185	16.728	17.095
ENSG00000103994	ZNF106	-1.4	6.2E-15	1.4E-13	24.711	0.925	23.649	25.342	35.501	0.925	34.616	36.461
ENSG00000104081	BMF	-1.4	4.2E-10	3.6E-09	15.348	0.139	15.198	15.472	22.186	1.219	21.414	23.591
ENSG00000104299	INTS9	-1.4	1.9E-06	7.8E-06	11.176	0.479	10.623	11.454	15.208	0.464	14.752	15.680
ENSG00000104327	CALB1	-1.4	4.9E-08	2.8E-07	9.479	0.414	9.023	9.831	13.015	0.467	12.665	13.546
ENSG00000104825	NFKBIB	-1.4	9.2E-08	4.9E-07	17.508	0.844	16.926	18.477	25.322	2.480	22.480	27.049
ENSG00000104880	ARHGEF18	-1.4	4.1E-04	1.1E-03	2.155	0.073	2.104	2.239	3.044	0.270	2.756	3.292
ENSG00000104969	SGTA	-1.4	4.0E-12	5.2E-11	40.010	0.518	39.416	40.364	57.115	2.212	55.162	59.517
[illegible]												

ENSG00000182158	CREB3L2	-1.4	2.1E-09	1.6E-08	12.308	1.100	11.276	13.465	17.101	0.137	16.958	17.231
ENSG00000182318	ZSCAN22	-1.4	2.3E-02	4.0E-02	1.366	0.157	1.227	1.536	1.883	0.334	1.502	2.126
ENSG00000182473	EXOC7	-1.4	1.4E-11	1.7E-10	23.760	0.646	23.246	24.028	32.185	0.821	31.425	33.056
ENSG00000182511	FES	-1.4	1.1E-03	2.6E-03	2.987	0.331	2.605	3.188	4.085	0.210	3.849	4.253
ENSG00000182544	MFSD5	-1.4	4.8E-06	1.8E-05	12.001	0.904	11.243	13.001	16.588	0.137	16.451	16.724
ENSG00000182809	CRIP2	-1.4	1.7E-03	3.9E-03	2.225	0.255	2.042	2.517	3.133	0.569	2.628	3.749
ENSG00000183171		-1.4	1.8E-02	3.3E-02	3.779	1.091	2.521	4.469	5.134	0.866	4.432	6.101
ENSG00000183248	PRR36	-1.4	9.0E-03	1.7E-02	8.990	0.751	8.378	9.828	12.674	2.285	10.502	15.056
ENSG00000183337	BCOR	-1.4	1.5E-11	1.8E-10	13.183	0.305	12.843	13.430	18.796	0.737	18.364	19.647
ENSG00000183833	MAATS1	-1.4	4.5E-03	9.2E-03	1.137	0.204	0.999	1.371	1.611	0.233	1.343	1.761
ENSG00000183873	SCN5A	-1.4	1.8E-05	6.2E-05	3.653	0.484	3.289	4.202	4.980	0.329	4.683	5.333
ENSG00000184162	NR2C2AP	-1.4	4.8E-05	1.5E-04	13.484	1.637	11.730	14.970	18.393	0.271	18.181	18.699
ENSG00000184436	THAP7	-1.4	7.1E-06	2.6E-05	7.792	0.311	7.507	8.119	11.061	0.851	10.141	11.818
ENSG00000184669	OR7E14P	-1.4	1.1E-01	1.6E-01	1.361	0.378	1.014	1.764	1.924	0.144	1.835	2.090
ENSG00000184708	EIF4ENIF1	-1.4	1.6E-07	7.9E-07	8.517	0.609	7.839	9.018	11.699	0.610	11.050	12.261
ENSG00000184908	CLCNKB	-1.4	9.5E-02	1.4E-01	0.981	0.284	0.653	1.160	1.329	0.459	1.022	1.856
ENSG00000184967	NOC4L	-1.4	3.1E-06	1.2E-05	20.621	0.617	19.972	21.200	28.632	2.011	27.125	30.915
ENSG00000185052	SLC24A3	-1.4	1.1E-04	3.1E-04	4.191	0.599	3.591	4.788	6.006	0.692	5.505	6.796
ENSG00000185561	TLCD2	-1.4	1.1E-02	2.1E-02	0.804	0.080	0.714	0.870	1.157	0.157	0.982	1.287
ENSG00000185664	PMEL	-1.4	7.8E-13	1.1E-11	40.204	1.121	39.401	41.485	56.785	1.077	55.580	57.651
ENSG00000185730	ZNF696	-1.4	3.0E-05	9.7E-05	6.079	0.531	5.624	6.663	8.384	0.463	7.850	8.667
ENSG00000185736	ADARB2	-1.4	1.5E-03	3.5E-03	1.145	0.062	1.081	1.204	1.562	0.089	1.488	1.660
ENSG00000185745	IFIT1	-1.4	3.2E-02	5.4E-02	1.311	0.255	1.017	1.472	1.773	0.457	1.250	2.089
ENSG00000185803	SLC52A2	-1.4	1.6E-08	1.0E-07	23.363	1.252	22.134	24.637	33.220	1.418	32.097	34.813
ENSG00000185818	NAT8L	-1.4	1.1E-06	4.8E-06	6.015	0.322	5.667	6.304	8.351	0.474	7.923	8.860
ENSG00000185917	SETD4	-1.4	1.9E-07	9.4E-07	6.986	0.202	6.755	7.126	9.799	0.817	8.989	10.622
ENSG00000186141	POLR3C	-1.4	1.5E-07	7.5E-07	19.370	1.623	17.818	21.055	27.403	1.167	26.608	28.743
ENSG00000186272	ZNF17	-1.4	5.6E-04	1.4E-03	3.512	0.333	3.137	3.775	4.968	0.355	4.559	5.198
ENSG00000186767	SPIN4	-1.4	1.1E-06	4.6E-06	10.421	0.655	9.898	11.155	14.280	1.161	13.455	15.608
ENSG00000186787	SPIN2B	-1.4	1.6E-03	3.6E-03	6.167	0.506	5.584	6.484	8.805	0.644	8.075	9.292
ENSG00000186838	SELENOV	-1.4	5.2E-04	1.3E-03	5.816	0.293	5.626	6.153	8.288	0.500	7.797	8.796
ENSG00000187231	SESTD1	-1.4	3.6E-11	3.9E-10	9.917	0.358	9.525	10.227	13.927	0.368	13.553	14.290
ENSG00000187624	C17orf97	-1.4	4.7E-02	7.6E-02	1.519	0.134	1.367	1.617	2.121	0.378	1.811	2.542
ENSG00000187792	ZNF70	-1.4	3.5E-03	7.4E-03	1.337	0.088	1.250	1.427	1.843	0.179	1.689	2.043
ENSG00000187952	HS6ST1P1	-1.4	6.4E-04	1.6E-03	10.691	0.205	10.457	10.841	14.751	0.924	13.729	15.526
ENSG00000188112	C6orf132	-1.4	1.2E-09	9.0E-09	14.563	0.632	14.011	15.252	20.900	0.653	20.239	21.544
ENSG00000188162	OTOG	-1.4	3.4E-02	5.6E-02	0.275	0.063	0.202	0.319	0.397	0.052	0.341	0.443
ENSG00000188191	PRKAR1B	-1.4	4.9E-06	1.9E-05	14.000	0.599	13.404	14.602	19.559	2.614	17.076	22.287
ENSG00000188315	C3orf62	-1.4	8.8E-03	1.7E-02	1.485	0.139	1.347	1.625	2.056	0.159	1.945	2.238
ENSG00000188372	ZP3	-1.4	1.0E-02	2.0E-02	2.490	0.250	2.201	2.643	3.399	0.227	3.137	3.540
ENSG00000188486	H2AFX	-1.4	8.6E-10	6.9E-09	138.355	5.338	135.227	144.519	190.602	18.691	177.337	211.978
ENSG00000188566	NDOR1	-1.4	1.1E-06	4.9E-06	11.625	1.373	10.360	13.086	16.705	1.055	15.565	17.646
ENSG00000188582	PAQR9	-1.4	4.4E-02	7.2E-02	1.230	0.161	1.090	1.407	1.673	0.290	1.383	1.963
ENSG00000188707	ZBED6CL	-1.4	3.1E-04	8.3E-04	5.142	0.275	4.841	5.381	7.429	0.770	6.567	8.049
[illegible]												

ENSG00000129596	CDO1	-1.3	4.9E-04	1.2E-03	15.859	2.876	14.160	19.179	20.411	1.148	19.527	21.709
ENSG00000129625	REEP5	-1.3	1.8E-08	1.1E-07	45.753	0.900	44.782	46.560	57.787	1.431	56.318	59.178
ENSG00000129675	ARHGEF6	-1.3	5.2E-05	1.6E-04	5.432	0.105	5.348	5.550	7.004	0.271	6.752	7.291
ENSG00000129951	PLPPR3	-1.3	3.8E-04	9.8E-04	25.072	0.569	24.485	25.622	31.847	3.853	28.335	35.969
ENSG00000130055	GDPD2	-1.3	2.6E-04	7.1E-04	12.452	0.759	11.752	13.259	16.553	1.060	15.674	17.730
ENSG00000130349	C6orf203	-1.3	1.2E-03	2.8E-03	15.628	0.803	14.707	16.184	20.292	0.872	19.749	21.298
ENSG00000130413	STK33	-1.3	8.0E-05	2.4E-04	5.930	0.685	5.276	6.642	7.589	0.093	7.482	7.644
ENSG00000130477	UNC13A	-1.3	6.5E-10	5.3E-09	22.051	0.787	21.191	22.735	28.805	1.447	27.543	30.385
ENSG00000130544	ZNF557	-1.3	1.7E-02	3.0E-02	1.898	0.178	1.795	2.104	2.400	0.137	2.289	2.553
ENSG00000130699	TAF4	-1.3	3.8E-07	1.8E-06	6.287	0.416	5.809	6.567	8.408	0.412	7.933	8.651
ENSG00000131149	GSE1	-1.3	9.5E-09	6.2E-08	14.376	0.401	13.928	14.700	18.575	0.941	17.854	19.639
ENSG00000131153	GINS2	-1.3	3.5E-06	1.4E-05	49.721	1.316	48.801	51.229	62.453	2.684	60.472	65.507
ENSG00000131165	CHMP1A	-1.3	4.0E-10	3.4E-09	44.854	0.754	44.012	45.467	60.413	2.250	57.870	62.145
ENSG00000131370	SH3BP5	-1.3	4.6E-04	1.2E-03	7.648	0.664	7.103	8.387	9.711	0.714	9.148	10.513
ENSG00000131375	CAPN7	-1.3	1.7E-05	5.8E-05	10.890	0.424	10.422	11.250	13.745	0.890	13.210	14.772
ENSG00000131504	DIAPH1	-1.3	1.5E-11	1.8E-10	62.740	1.019	61.999	63.902	81.368	1.870	79.213	82.564
ENSG00000131591	C1orf159	-1.3	1.6E-03	3.6E-03	2.431	0.065	2.362	2.491	3.149	0.075	3.063	3.202
ENSG00000131844	MCCC2	-1.3	1.6E-09	1.2E-08	47.667	0.554	47.323	48.307	60.349	1.224	58.940	61.153
ENSG00000132153	DHX30	-1.3	1.1E-09	8.9E-09	33.028	0.819	32.205	33.844	42.042	0.844	41.127	42.789
ENSG00000132330	SCLY	-1.3	3.8E-04	9.9E-04	1.955	0.119	1.878	2.092	2.593	0.202	2.394	2.798
ENSG00000132382	MYBBP1A	-1.3	1.1E-09	8.7E-09	42.093	0.311	41.738	42.315	54.373	3.391	50.759	57.486
ENSG00000132386	SERPINF1	-1.3	6.1E-05	1.9E-04	9.264	0.291	8.938	9.498	12.416	1.060	11.544	13.596
ENSG00000132429	POPDC3	-1.3	7.8E-02	1.2E-01	1.991	0.191	1.812	2.192	2.628	0.489	2.302	3.190
ENSG00000132434	LANCL2	-1.3	2.9E-04	7.8E-04	6.330	0.223	6.074	6.480	8.154	0.820	7.627	9.099
ENSG00000132591	ERAL1	-1.3	2.0E-07	1.0E-06	72.022	3.357	68.905	75.576	91.039	4.772	87.380	96.438
ENSG00000132600	PRMT7	-1.3	6.2E-08	3.4E-07	8.058	0.247	7.774	8.223	10.887	0.150	10.782	11.058
ENSG00000132603	NIP7	-1.3	9.6E-10	7.6E-09	43.264	1.219	42.340	44.645	57.154	1.294	55.825	58.410
ENSG00000132623	ANKEF1	-1.3	9.7E-07	4.2E-06	8.519	0.758	7.644	8.888	11.530	0.500	11.194	12.104
ENSG00000132801	ZSWIM3	-1.3	6.2E-02	9.6E-02	3.381	0.251	3.102	3.589	4.281	1.385	2.687	5.189
ENSG00000133028	SCO1	-1.3	2.3E-11	2.6E-10	25.128	1.028	24.216	26.241	33.534	0.650	32.949	34.234
ENSG00000133943	C14orf159	-1.3	2.2E-05	7.3E-05	5.674	0.232	5.522	5.942	7.366	0.235	7.096	7.525
ENSG00000134202	GSTM3	-1.3	3.6E-02	6.0E-02	1.781	0.223	1.560	2.006	2.250	0.155	2.072	2.354
ENSG00000134247	PTGFRN	-1.3	4.7E-08	2.7E-07	21.555	1.188	20.188	22.340	27.569	0.713	26.961	28.353
ENSG00000134330	IAH1	-1.3	3.0E-04	8.0E-04	4.065	0.142	3.901	4.154	5.362	0.372	4.962	5.698
ENSG00000134444	KIAA1468	-1.3	1.5E-08	9.5E-08	15.242	0.223	15.060	15.491	19.829	0.852	19.109	20.770
ENSG00000134452	FBXO18	-1.3	3.4E-09	2.4E-08	18.183	0.864	17.294	19.019	24.326	1.017	23.393	25.410
ENSG00000134809	TIMM10	-1.3	9.2E-07	4.0E-06	83.345	6.278	76.459	88.752	109.472	2.302	106.937	110.915
ENSG00000134851	TMEM165	-1.3	1.4E-07	7.2E-07	28.016	0.882	27.101	28.861	35.186	0.708	34.647	35.988
ENSG00000134982	APC	-1.3	6.2E-07	2.8E-06	10.431	0.891	9.405	11.007	13.158	0.399	12.779	13.573
ENSG00000135093	USP30	-1.3	8.2E-04	2.0E-03	3.477	0.071	3.406	3.547	4.602	0.363	4.353	5.019
ENSG00000135315	CEP162	-1.3	2.0E-05	6.9E-05	7.148	0.875	6.147	7.767	9.410	0.177	9.238	9.591
ENSG00000135378	PRRG4	-1.3	1.5E-02	2.7E-02	1.129	0.247	0.969	1.414	1.541	0.086	1.455	1.627
ENSG00000135451	TROAP	-1.3	5.2E-07	2.4E-06	15.156	0.871	14.170	15.823	19.958	0.184	19.770	20.137
ENSG00000135521	LTV1	-1.3	3.3E-08	1.9E-07	55.371	1.936	53.483	57.352	73.078	2.117	71.476	75.479
ENSG00000135723	FHOD1	-1.3	4.6E-07	2.1E-06	16.188	0.105	16.091	16.300	21.070	0.832	20.332	21.972
ENSG00000135776	ABCB10	-1.3	1.4E-04	3.9E-04	8.942	0.848	8.105	9.802	11.535	0.859	10.552	12.144
ENSG00000135801	TAF5L	-1.3	3.2E-07	1.5E-06	12.598	1.041	11.721	13.749	16.940	1.163	15.610	17.760
ENSG00000135899	SP110	-1.3	1.3E-02	2.4E-02	2.351	0.088	2.270	2.444	3.025	0.186	2.889	3.238
ENSG00000135931	ARMC9	-1.3	2.9E-06	1.1E-05	6.740	0.406	6.279	7.040	8.828	0.646	8.193	9.485
ENSG00000136026	CKAP4	-1.3	1.3E-10	1.2E-09	72.388	3.319	68.604	74.806	95.148	5.385	89.119	99.479
ENSG00000136059	VILL	-1.3	1.4E-02	2.6E-02	1.768	0.085	1.691	1.859	2.223	0.092	2.169	2.330
ENSG00000136111	TBC1D4	-1.3	1.1E-08	6.9E-08	36.787	0.840	36.301	37.757	46.750	0.874	45.780	47.475
ENSG00000136213	CHST12	-1.3	2.5E-04	6.7E-04	1.601	0.126	1.463	1.709	2.087	0.035	2.051	2.120
ENSG00000136231	IGF2BP3	-1.3	4.8E-09	3.3E-08	104.153	3.755	101.830	108.485	132.309	4.603	127.136	135.954
ENSG00000136271	DDX56	-1.3	1.5E-06	6.4E-06	28.519	0.914	27.508	29.288	35.841	2.689	33.171	38.549
ENSG00000136305	CIDEB	-1.3	4.0E-03	8.2E-03	6.499	0.773	5.939	7.381	8.227	0.870	7.557	9.210
ENSG00000136448	NMT1	-1.3	7.0E-11	6.9E-10	57.212	3.183	55.080	60.870	74.027	1.922	71.970	75.777
ENSG00000136463	TACO1	-1.3	3.0E-05	9.7E-05	18.088	0.558	17.668	18.721	23.842	2.869	22.145	27.155
ENSG00000136478	TEX2	-1.3	1.2E-06	5.1E-06	6.181	0.404	5.879	6.640	8.160	0.331	7.865	8.518
ENSG00000136709	WDR33	-1.3	6.4E-10	5.2E-09	21.244	0.399	20.783	21.474	26.754	0.368	26.473	27.171
ENSG00000136715	SAP130	-1.3	1.5E-07	7.8E-07	29.798	1.385	28.210	30.759	37.931	1.260	36.619	39.131
ENSG00000136718	IMP4	-1.3	1.3E-09	1.0E-08	39.682	0.637	38.977	40.214	52.346	1.532	50.579	53.295
ENSG00000136720	HS6ST1	-1.3	4.2E-09	2.9E-08	37.446	1.294	35.953	38.249	48.136	1.100	47.453	49.405
ENSG00000136731	UGGT1	-1.3	9.4E-11	9.1E-10	31.613	0.304	31.271	31.852	41.463	1.913	40.074	43.646
ENSG00000136819	C9orf78	-1.3	1.9E-07	9.3E-07	23.147	1.788	21.583	25.097	29.859	0.696	29.176	30.567
ENSG00000136861	CDK5RAP2	-1.3	3.1E-09	2.2E-08	18.143	0.625	17.689	18.857	23.239	0.882	22.242	23.918
ENSG00000136875	PRPF4	-1.3	3.4E-09	2.4E-08	38.662	1.065	37.469	39.516	50.198	0.868	49.372	51.103
ENSG00000136933	RABEPK	-1.3	6.1E-06	2.3E-05	18.634	1.667	17.659	20.559	24.250	0.391	23.811	24.561
ENSG00000136982	DSCC1	-1.3	1.6E-09	1.2E-08	68.199	3.144	65.108	71.394	90.095	3.725	87.931	94.396
ENSG00000137124	ALDH1B1	-1.3	1.8E-09	1.3E-08	70.480	0.255	70.252	70.755	90.669	3.582	86.779	93.833
ENSG00000137168	PPIL1	-1.3	2.0E-11	2.2E-10	139.099	2.228	137.464	141.637	183.647	6.663	176.038	188.441
ENSG00000137338	PGBD1	-1.3	5.5E-05	1.7E-04	8.051	0.162	7.867	8.168	10.885	1.200	9.588	11.973
ENSG00000137474	MYO7A	-1.3	3.7E-02	6.2E-02	0.632	0.046	0.603	0.685	0.813	0.077	0.757	0.901
ENSG00000137726	FXYD6	-1.3	7.2E-06	2.6E-05	10.979	0.672	10.246	11.565	14.678	1.407	13.107	15.821
ENSG00000137819	PAQR5	-1.3	6.9E-06	2.6E-05	7.884	0.557	7.489	8.521	10.509	0.768	9.684	11.204
ENSG00000137936	BCAR3	-1.3	8.0E-04	1.9E-03	4.980	0.438	4.484	5.312	6.339	0.482	5.788	6.677
ENSG00000137960	GIPC2	-1.3	4.2E-02	6.8E-02	1.153	0.249	0.899	1.396	1.562	0.171	1.442	1.758
ENSG00000138031	ADCY3	-1.3	9.6E-07	4.2E-06	12.364	0.657	11.665	12.969	16.167	0.846	15.380	17.062
ENSG00000138614	INTS14	-1.3	2.2E-06	9.0E-06	23.029	1.016	22.015	24.047	29.001	1.634	27.174	30.323
ENSG00000139173	TMEM117	-1.3	6.5E-02	1.0E-01	1.513	0.333	1.240	1.884	1.979	0.230	1.784	2.233
ENSG00000139364	TMEM132B	-1.3	7.3E-08	4.0E-07	23.924	1.181	22.706	25.064	30.017	0.505	29.542	30.548
ENSG00000139405	RITA1	-1.3	1.8E-05	6.2E-05	20.412	0.510	19.824	20.722	26.783	1.717	25.391	28.702
ENSG00000139618	BRCA2	-1.3	2.6E-06	1.0E-05	9.501	0.719	8.741	10.171	12.046	0.527	11.650	12.645
ENSG00000139719	VPS33A	-1.3	6.8E-06	2.5E-05	7.157	0.529	6.822	7.767	9.270	0.337	9.040	9.656
ENSG00000139910	NOVA1	-1.3	3.9E-03	8.1E-03	2.180	0.182	1.971	2.305	2.765	0.288	2.533	3.087
ENSG00000139946	PELI2	-1.3	8.6E-06	3.1E-05	12.239	1.173	11.113	13.453	15.674	0.220	15.420	15.811
ENSG00000140153	WDR20	-1.3	6.7E-05	2.1E-04	6.546	0.212	6.361	6.778	8.319	0.612	7.835	9.006
ENSG00000140157	NIPA2	-1.3	1.1E-09	8.9E-09	69.562	1.104	68.565	70.749	89.178	4.258	84.463	92.740
ENSG00000140259	MFAP1	-1.3	7.1E-08	3.9E-07	59.702	2.523	58.211	62.615	76.188	2.865	73.052	78.668
ENSG00000140326	CDAN1	-1.3	2.0E-05	6.9E-05	5.442	0.139	5.289	5.562	7.291	0.243	7.051	7.537
ENSG00000140365	COMMD4	-1.3	9.9E-08	5.2E-07	17.837	0.458	17.311	18.147	22.945	1.004	21.984	23.988
ENSG00000140463	BBS4	-1.3	2.4E-07	1.2E-06	19.459	0.850	18.564	20.254	25.292	1.304	23.992	26.600
ENSG00000140750	ARHGAP17	-1.3	4.4E-07	2.1E-06	12.375	0.384	11.937	12.652	15.531	0.510	15.101	16.095
ENSG00000140854	KATNB1	-1.3	7.5E-08	4.1E-07	24.722	0.970	23.693	25.618	32.163	2.004	30.335	34.306
ENSG00000140931	CMTM3	-1.3	2.0E-04	5.5E-04	13.880	2.025	11.733	15.755	18.230	1.936	16.473	20.306
ENSG00000141013	GAS8	-1.3	9.9E-05	2.9E-04	3.891	0.234	3.700	4.153	5.232	0.379	4.801	5.513
ENSG00000141030	COPS3	-1.3	1.2E-09	9.1E-09	87.650	2.100	86.254	90.065	111.075	1.116	109.951	112.181
ENSG00000141098	GFOD2	-1.3	5.8E-07	2.6E-06	9.703	0.195	9.525	9.911	12.708	0.256	12.529	13.001
ENSG00000141219	C17orf80	-1.3	9.1E-08	4.8E-07	24.519	0.917	23.671	25.492	31.351	1.309	30.589	32.863
ENSG00000141499	WRAP53	-1.3	1.5E-05	5.1E-05	11.756	0.663	11.040	12.349	14.860	0.160	14.690	15.007
ENSG00000141577	CEP131	-1.3	8.6E-06	3.1E-05	13.217	0.783	12.727	14.120	17.427	1.953	15.917	19.633
ENSG00000141582	CBX4	-1.3	9.1E-02	1.3E-01	1.674	0.341	1.284	1.916	2.154	0.500	1.757	2.716
ENSG00000142230	SAE1	-1.3	2.3E-11	2.6E-10	208.124	1.423	206.707	209.554	263.234	5.686	257.041	268.248
ENSG00000142396		-1.3	1.2E-04	3.5E-04	4.714	0.098	4.604	4.793	6.189	0.095	6.079	6.245
ENSG00000142409	ZNF787	-1.3	1.2E-05	4.4E-05	23.173	0.309	22.891	23.503	29.579	2.377	27.804	32.280
ENSG00000142544	CTU1	-1.3	3.7E-02	6.0E-02	4.003	0.639	3.543	4.733	5.210	0.804	4.284	5.726
ENSG00000142875	PRKACB	-1.3	9.2E-08	4.9E-07	15.684	0.523	15.114	16.141	20.371	1.288	18.884	21.141
ENSG00000143013	LMO4	-1.3	3.8E-08	2.2E-07	30.635	1.098	29.637	31.811	38.827	1.549	37.054	39.915
ENSG00000143476	DTL	-1.3	9.0E-10	7.2E-09	50.768	2.343	48.141	52.640	66.377	2.135	64.462	68.679
ENSG00000143543	JTB	-1.3	4.6E-09	3.2E-08	83.195	6.274	75.952	86.966	112.402	6.084	105.513	117.038
ENSG00000143569	UBAP2L	-1.3	2.4E-10	2.2E-09	60.572	3.174	58.341	64.205	78.096	3.097	74.564	80.349
ENSG00000143643	TTC13	-1.3	4.7E-04	1.2E-03	6.399	0.628	5.781	7.036	8.439	0.699	7.661	9.012
ENSG00000143891	GALM	-1.3	5.0E-06	1.9E-05	15.583	1.022	14.952	16.763	20.751	1.406	19.501	22.273
ENSG00000143924	EML4	-1.3	1.1E-10	1.1E-09	66.941	0.187	66.780	67.146	85.393	2.203	82.907	87.102
ENSG00000144115	THNSL2	-1.3	4.5E-03	9.3E-03	1.532	0.273	1.282	1.824	2.057	0.276	1.749	2.281
ENSG00000144120	TMEM177	-1.3	2.3E-07	1.1E-06	26.288	1.038	25.287	27.361	35.385	0.907	34.442	36.251
ENSG00000144357	UBR3	-1.3	3.0E-07	1.5E-06	8.818	0.567	8.421	9.467	11.501	0.631	10.773	11.876
ENSG00000144369	FAM171B	-1.3	1.8E-06	7.3E-06	10.746	0.796	9.829	11.256	14.438	1.087	13.224	15.321
ENSG00000144406	UNC80	-1.3	1.3E-01	1.8E-01	0.211	0.059	0.170	0.278	0.267	0.048	0.218	0.314
ENSG00000144659	SLC25A38	-1.3	2.1E-06	8.5E-06	32.005	1.624	30.138	33.090	41.620	3.099	39.062	45.067
ENSG00000144736	SHQ1	-1.3	4.2E-06	1.6E-05	13.821	0.784	13.267	14.719	17.502	0.496	17.042	18.028
ENSG00000144749	LRIG1	-1.3	5.8E-12	7.3E-11	61.722	1.497	60.822	63.450	81.287	2.056	79.002	82.812
ENSG00000144848	ATG3	-1.3	4.6E-09	3.2E-08	17.873	0.444	17.557	18.381	23.743	0.704	23.051	24.459
ENSG00000145428	RNF175	-1.3	2.2E-03	4.9E-03	4.613	0.248	4.405	4.887	5.908	0.366	5.591	6.309
ENSG00000145555	MYO10	-1.3	1.7E-09	1.3E-08	33.766	1.674	32.228	35.549	42.929	0.971	42.343	44.049
ENSG00000145604	SKP2	-1.3	1.3E-10	1.2E-09	54.752	0.890	53.853	55.633	71.756	2.486	69.496	74.418
ENSG00000145911	N4BP3	-1.3	1.3E-09	9.8E-09	32.430	0.934	31.504	33.372	43.221	2.047	40.873	44.629
ENSG00000145916	RMND5B	-1.3	1.5E-09	1.1E-08	15.788	0.916	14.733	16.375	21.017	0.231	20.751	21.159
ENSG00000146072	TNFRSF21	-1.3	5.5E-09	3.8E-08	35.680	1.388	34.251	37.024	47.455	2.017	45.130	48.741
ENSG00000146233	CYP39A1	-1.3	6.2E-02	9.6E-02	2.272	0.367	1.878	2.605	2.873	0.258	2.630	3.143
ENSG00000146263	MMS22L	-1.3	5.3E-09	3.6E-08	6.291	0.175	6.176	6.492	8.184	0.140	8.065	8.339
ENSG00000146776	ATXN7L1	-1.3	3.0E-07	1.4E-06	8.023	0.822	7.127	8.742	10.870	0.444	10.475	11.350
ENSG00000146830	GIGYF1	-1.3	8.1E-10	6.6E-09	32.124	0.716	31.477	32.892	40.842	0.680	40.424	41.626
ENSG00000146834	MEPCE	-1.3	1.8E-07	9.0E-07	23.979	0.751	23.114	24.461	31.770	2.046	30.511	34.130
ENSG00000147100	SLC16A2	-1.3	1.3E-03	2.9E-03	19.892	1.038	19.032	21.045	25.256	1.017	24.132	26.112
ENSG00000147804	SLC39A4	-1.3	3.8E-04	9.9E-04	4.797	0.469	4.289	5.213	6.244	0.111	6.118	6.328
ENSG00000148335	NTMT1	-1.3	2.8E-06	1.1E-05	31.600	1.578	30.231	33.326	39.885	1.219	39.035	41.282
ENSG00000148343	MIGA2	-1.3	4.2E-04	1.1E-03	5.972	1.031	4.972	7.032	7.588	0.380	7.204	7.964
ENSG00000148344	PTGES	-1.3	3.7E-02	6.0E-02	5.606	1.485	4.350	7.245	7.104	0.387	6.748	7.515
ENSG00000148843	PDCD11	-1.3	9.3E-13	1.3E-11	53.324	1.060	52.147	54.202	71.790	0.653	71.036	72.171
ENSG00000149054	ZNF215	-1.3	2.2E-06	8.8E-06	10.935	0.396	10.579	11.362	14.145	0.175	13.989	14.334
ENSG00000149328	GLB1L2	-1.3	8.0E-04	1.9E-03	2.728	0.210	2.516	2.936	3.563	0.130	3.422	3.676
ENSG00000149428	HYOU1	-1.3	9.1E-13	1.3E-11	82.993	4.702	78.198	87.596	111.206	1.326	110.424	112.737
ENSG00000149480	MTA2	-1.3	1.1E-10	1.1E-09	68.454	1.640	66.608	69.741	88.505	1.625	86.827	90.071
ENSG00000149658	YTHDF1	-1.3	5.4E-09	3.7E-08	48.319	4.227	44.561	52.895	63.791	0.600	63.121	64.279
ENSG00000150394	CDH8	-1.3	5.7E-02	8.9E-02	0.399	0.074	0.342	0.483	0.525	0.088	0.423	0.578
ENSG00000150540	HNMT	-1.3	3.5E-03	7.3E-03	3.271	0.225	3.106	3.528	4.320	0.215	4.079	4.494
ENSG00000151090	THRB	-1.3	1.0E-01	1.5E-01	0.514	0.077	0.466	0.603	0.648	0.090	0.545	0.710
ENSG00000151093	OXSM	-1.3	3.5E-02	5.8E-02	2.996	0.231	2.745	3.199	3.871	0.668	3.167	4.495
ENSG00000151116	UEVLD	-1.3	7.9E-04	1.9E-03	5.344	0.409	4.873	5.597	6.826	0.683	6.379	7.624
ENSG00000151348	EXT2	-1.3	5.8E-08	3.2E-07	29.789	0.504	29.462	30.370	37.765	1.059	36.571	38.592
ENSG00000151468	CCDC3	-1.3	1.1E-01	1.6E-01	1.013	0.244	0.796	1.278	1.318	0.210	1.100	1.518
ENSG00000151502	VPS26B	-1.3	1.1E-07	5.9E-07	14.038	0.288	13.771	14.344	18.395	0.412	18.110	18.867
ENSG00000151657	KIN	-1.3	6.6E-05	2.0E-04	7.855	0.299	7.510	8.040	9.861	0.338	9.614	10.246
ENSG00000151689	INPP1	-1.3	3.1E-02	5.2E-02	1.485	0.282	1.218	1.779	1.889	0.242	1.710	2.142
ENSG00000151693	ASAP2	-1.3	5.0E-06	1.9E-05	12.367	0.775	11.914	13.261	15.580	1.022	14.524	16.565
ENSG00000151746	BICD1	-1.3	7.9E-07	3.5E-06	6.300	0.484	5.745	6.635	8.142	0.181	7.980	8.336
ENSG00000151948	GLT1D1	-1.3	2.0E-03	4.5E-03	4.391	0.689	3.905	5.180	5.787	0.373	5.382	6.116
ENSG00000152520	PAN3	-1.3	1.3E-08	8.2E-08	19.155	0.784	18.527	20.034	25.011	0.225	24.752	25.166
ENSG00000153446	C16orf89	-1.3	1.5E-02	2.7E-02	4.328	0.522	3.734	4.712	5.735	0.335	5.542	6.122
ENSG00000153767	GTF2E1	-1.3	5.9E-09	4.0E-08	45.517	2.049	44.072	47.862	60.723	1.956	58.684	62.597
ENSG00000153786	ZDHHC7	-1.3	1.3E-07	6.9E-07	29.735	1.916	27.726	31.543	38.893	1.631	37.621	40.732
ENSG00000153827	TRIP12	-1.3	9.7E-11	9.4E-10	40.579	0.856	39.607	41.220	51.658	1.190	50.760	53.007
ENSG00000153885	KCTD15	-1.3	6.5E-07	2.9E-06	11.546	0.581	10.970	12.131	15.110	0.275	14.793	15.274
ENSG00000153896	ZNF599	-1.3	1.8E-02	3.2E-02	1.396	0.096	1.290	1.476	1.819	0.089	1.727	1.903
ENSG00000153922	CHD1	-1.3	4.4E-09	3.1E-08	27.124	0.986	26.146	28.119	34.272	1.256	33.528	35.722
ENSG00000154080	CHST9	-1.3	3.3E-05	1.1E-04	3.409	0.247	3.148	3.641	4.584	0.105	4.510	4.704
ENSG00000154309	DISP1	-1.3	1.9E-03	4.3E-03	4.879	0.343	4.494	5.152	6.146	0.325	5.777	6.391
ENSG00000154545	MAGED4	-1.3	6.5E-08	3.6E-07	18.440	0.915	17.607	19.419	24.684	1.803	22.777	26.361
ENSG00000154743	TSEN2	-1.3	6.5E-05	2.0E-04	8.242	0.354	7.899	8.606	10.505	0.555	10.122	11.142
ENSG00000154783	FGD5	-1.3	5.9E-06	2.2E-05	9.054	0.576	8.398	9.479	11.555	0.517	10.995	12.014
ENSG00000154813	DPH3	-1.3	2.0E-07	1.0E-06	31.209	1.004	30.072	31.974	40.546	3.006	37.172	42.942
ENSG00000156469	MTERF3	-1.3	2.3E-06	9.2E-06	49.201	2.542	46.416	51.396	63.111	5.761	57.619	69.107
ENSG00000156471	PTDSS1	-1.3	1.8E-09	1.4E-08	32.058	0.554	31.432	32.488	41.515	1.978	39.233	42.725
ENSG00000156535	CD109	-1.3	2.9E-05	9.6E-05	5.545	0.137	5.412	5.686	7.177	0.752	6.574	8.019
ENSG00000156587	UBE2L6	-1.3	1.7E-07	8.8E-07	43.056	2.043	41.310	45.303	55.985	3.325	53.535	59.771
ENSG00000156603	MED19	-1.3	9.4E-04	2.2E-03	10.485	0.749	9.621	10.937	13.871	1.137	13.173	15.184
ENSG00000156802	ATAD2	-1.3	5.9E-08	3.2E-07	22.575	0.632	22.097	23.292	28.809	0.850	27.931	29.628
ENSG00000157150	TIMP4	-1.3	4.7E-02	7.6E-02	5.272	1.137	4.033	6.267	6.791	0.945	6.135	7.873
ENSG00000157191	NECAP2	-1.3	7.5E-06	2.8E-05	12.373	0.343	12.106	12.760	15.599	0.724	14.801	16.214
ENSG00000157212	PAXIP1	-1.3	1.7E-06	7.0E-06	13.854	1.048	12.645	14.510	17.418	0.582	16.854	18.016
ENSG00000157216	SSBP3	-1.3	3.3E-10	2.9E-09	29.625	0.970	28.530	30.378	39.370	0.733	38.629	40.095
ENSG00000157600	TMEM164	-1.3	9.1E-07	4.0E-06	15.285	0.568	14.654	15.756	19.540	0.578	19.008	20.155
ENSG00000157778	PSMG3	-1.3	3.5E-07	1.7E-06	30.815	2.423	28.365	33.209	41.164	0.889	40.339	42.106
ENSG00000157823	AP3S2	-1.3	5.4E-05	1.7E-04	6.933	0.576	6.273	7.336	8.764	0.680	8.105	9.482
ENSG00000158089	GALNT14	-1.3	2.6E-04	7.0E-04	5.548	0.563	4.905	5.948	7.406	0.364	7.035	7.763
ENSG00000158106	RHPN1	-1.3	5.5E-04	1.4E-03	5.430	0.410	4.958	5.692	7.217	0.641	6.506	7.746
ENSG00000158109	TPRG1L	-1.3	1.5E-06	6.3E-06	27.833	3.003	25.753	31.276	36.783	1.685	34.928	37.844
ENSG00000158296	SLC13A3	-1.3	7.7E-05	2.3E-04	5.912	0.200	5.736	6.129	7.644	0.230	7.407	7.867
ENSG00000158321	AUTS2	-1.3	8.9E-08	4.8E-07	12.740	0.937	11.658	13.304	16.506	0.487	16.178	17.065
ENSG00000158457	TSPAN33	-1.3	1.2E-06	5.3E-06	19.813	1.286	18.769	21.249	26.743	1.411	25.144	27.812
ENSG00000158669	GPAT4	-1.3	6.1E-10	5.0E-09	23.787	0.479	23.247	24.160	31.296	0.810	30.814	32.231
ENSG00000159110	IFNAR2	-1.3	2.1E-04	5.8E-04	6.245	0.441	5.855	6.724	8.035	0.539	7.669	8.654
ENSG00000159247	TUBBP5	-1.3	1.5E-02	2.8E-02	4.063	0.448	3.729	4.572	5.251	0.891	4.232	5.879
ENSG00000159579	RSPRY1	-1.3	5.8E-07	2.6E-06	18.617	0.591	18.046	19.227	23.859	0.816	23.092	24.717
ENSG00000159712	ANKRD18CP	-1.3	7.7E-06	2.8E-05	18.422	0.940	17.368	19.173	23.972	1.435	22.316	24.844
ENSG00000159784	FAM131B	-1.3	1.8E-06	7.4E-06	16.756	0.740	15.921	17.331	21.153	0.636	20.769	21.887
ENSG00000159788	RGS12	-1.3	7.3E-06	2.7E-05	3.444	0.206	3.220	3.624	4.579	0.282	4.331	4.886
ENSG00000159792	PSKH1	-1.3	8.4E-07	3.7E-06	16.072	1.304	14.593	17.054	21.208	0.360	20.847	21.566
ENSG00000159842	ABR	-1.3	1.5E-08	9.2E-08	12.483	0.701	11.684	12.998	16.706	0.372	16.284	16.990
ENSG00000159899	NPR2	-1.3	5.0E-05	1.6E-04	6.323	0.133	6.174	6.430	8.472	0.676	7.987	9.244
ENSG00000160051	IQCC	-1.3	7.1E-03	1.4E-02	4.734	0.346	4.361	5.044	6.091	0.133	5.943	6.201
ENSG00000160055	TMEM234	-1.3	2.2E-02	3.9E-02	3.250	0.109	3.146	3.364	4.123	0.571	3.701	4.772
ENSG00000160113	NR2F6	-1.3	3.9E-06	1.5E-05	69.462	1.626	68.234	71.306	93.867	8.782	84.095	101.100
ENSG00000160294	MCM3AP	-1.3	1.2E-09	9.1E-09	36.398	0.834	35.620	37.278	46.107	0.342	45.768	46.452
ENSG00000160345	C9orf116	-1.3	4.7E-03	9.7E-03	5.675	0.115	5.568	5.796	7.519	0.582	6.864	7.974
ENSG00000160360	GPSM1	-1.3	4.9E-04	1.2E-03	8.646	1.599	7.610	10.489	11.037	1.185	9.733	12.049
ENSG00000160439	RDH13	-1.3	2.1E-05	7.2E-05	3.425	0.036	3.400	3.466	4.593	0.210	4.352	4.730
ENSG00000160716	CHRNB2	-1.3	1.8E-02	3.2E-02	2.189	0.231	2.017	2.452	2.769	0.346	2.434	3.124
ENSG00000160785	SLC25A44	-1.3	8.2E-06	3.0E-05	9.356	0.820	8.423	9.963	11.827	0.381	11.462	12.222
ENSG00000160932	LY6E	-1.3	2.8E-08	1.6E-07	48.044	1.099	46.974	49.169	64.047	5.966	58.415	70.299
ENSG00000160953	MUM1	-1.3	5.2E-08	2.9E-07	10.583	0.623	9.877	11.057	13.692	0.224	13.522	13.946
ENSG00000160991	ORAI2	-1.3	3.4E-07	1.6E-06	4.448	0.170	4.254	4.572	6.029	0.352	5.712	6.407
ENSG00000160993	ALKBH4	-1.3	1.4E-03	3.2E-03	12.676	1.178	11.483	13.837	16.100	1.870	14.319	18.048
ENSG00000160999	SH2B2	-1.3	8.8E-03	1.7E-02	7.710	0.274	7.446	7.992	10.177	1.990	8.203	12.183
ENSG00000161057	PSMC2	-1.3	2.1E-10	1.9E-09	73.018	3.391	69.124	75.323	95.932	3.392	92.922	99.608
ENSG00000161202	DVL3	-1.3	4.3E-08	2.4E-07	26.244	0.306	25.902	26.494	33.032	1.746	31.241	34.729
ENSG00000161618	ALDH16A1	-1.3	2.6E-05	8.5E-05	18.995	0.755	18.214	19.722	24.080	2.869	21.361	27.079
ENSG00000161642	ZNF385A	-1.3	3.3E-04	8.7E-04	6.525	0.334	6.229	6.888	8.701	0.613	8.049	9.265
ENSG00000161835	GRASP	-1.3	1.2E-01	1.7E-01	1.728	0.488	1.434	2.291	2.209	0.505	1.679	2.684
ENSG00000162063	CCNF	-1.3	1.1E-07	5.8E-07	20.770	0.827	19.888	21.636	27.019	1.504	26.006	28.747
ENSG00000162105	SHANK2	-1.3	9.6E-07	4.2E-06	6.959	0.407	6.704	7.429	8.739	0.324	8.515	9.111
ENSG00000162337	LRP5	-1.3	6.7E-07	3.0E-06	17.335	0.244	17.125	17.603	22.430	1.486	21.302	24.113
ENSG00000162378	ZYG11B	-1.3	1.2E-08	7.7E-08	13.041	0.757	12.407	13.880	17.433	0.929	16.850	18.505
ENSG00000162437	RAVER2	-1.3	4.7E-08	2.6E-07	19.051	1.497	17.381	20.270	24.777	0.676	24.164	25.501
ENSG00000162444	RBP7	-1.3	1.1E-01	1.6E-01	4.737	1.223	3.765	6.110	6.183	1.669	4.380	7.675
ENSG00000162493	PDPN	-1.3	8.3E-10	6.7E-09	52.600	1.270	51.422	53.946	70.233	1.358	68.732	71.377
ENSG00000162600	OMA1	-1.3	9.3E-06	3.3E-05	9.977	0.249	9.790	10.260	13.430	1.409	12.063	14.878
ENSG00000162607	USP1	-1.3	2.8E-11	3.0E-10	61.811	2.248	59.234	63.372	81.798	3.175	78.305	84.508
ENSG00000162694	EXTL2	-1.3	1.5E-10	1.4E-09	39.469	0.977	38.475	40.429	53.262	3.186	50.156	56.523
ENSG00000162702	ZNF281	-1.3	5.9E-10	4.9E-09	67.712	2.818	65.956	70.963	86.844	4.234	82.820	91.261
ENSG00000162729	IGSF8	-1.3	1.0E-09	8.1E-09	55.513	0.940	54.546	56.424	75.146	3.035	73.334	78.650
ENSG00000162755	KLHDC9	-1.3	3.1E-02	5.2E-02	4.531	0.573	3.869	4.877	5.814	0.305	5.523	6.130
ENSG00000162777	DENND2D	-1.3	1.1E-01	1.6E-01	0.789	0.097	0.677	0.851	0.999	0.118	0.887	1.122
ENSG00000162817	C1orf115	-1.3	2.3E-05	7.7E-05	9.313	0.771	8.540	10.083	12.389	0.620	12.028	13.105
ENSG00000162851	TFB2M	-1.3	2.9E-07	1.4E-06	33.041	0.792	32.148	33.659	43.875	1.172	42.982	45.202
ENSG00000163002	NUP35	-1.3	5.7E-08	3.2E-07	31.432	0.980	30.443	32.401	40.254	1.533	38.745	41.810
ENSG00000163297	ANTXR2	-1.3	1.1E-03	2.5E-03	2.589	0.269	2.332	2.869	3.270	0.048	3.221	3.318
ENSG00000163406	SLC15A2	-1.3	3.9E-02	6.4E-02	1.187	0.150	1.019	1.307	1.520	0.109	1.404	1.620
ENSG00000163507	KIAA1524	-1.3	8.2E-08	4.4E-07	41.405	2.706	38.461	43.783	52.881	1.395	51.311	53.977
ENSG00000163520	FBLN2	-1.3	8.6E-05	2.6E-04	17.570	1.304	16.429	18.991	22.203	0.969	21.191	23.124
ENSG00000163530	DPPA2	-1.3	4.4E-04	1.1E-03	13.606	1.515	12.441	15.319	18.363	1.651	16.469	19.494
ENSG00000163618	CADPS	-1.3	7.9E-05	2.4E-04	2.154	0.155	2.046	2.331	2.878	0.163	2.720	3.045
ENSG00000163904	SENP2	-1.3	4.2E-10	3.6E-09	20.267	0.833	19.784	21.229	27.022	0.788	26.173	27.730
ENSG00000163932	PRKCD	-1.3	2.9E-05	9.4E-05	17.328	0.640	16.589	17.711	22.040	1.268	21.142	23.490
ENSG00000163950	SLBP	-1.3	5.2E-08	2.9E-07	53.625	2.580	51.500	56.496	69.947	3.734	66.162	73.627
ENSG00000163964	PIGX	-1.3	2.9E-04	7.8E-04	7.776	0.332	7.557	8.158	9.900	1.060	8.981	11.059
ENSG00000164002	EXO5	-1.3	3.2E-02	5.4E-02	3.316	0.860	2.746	4.305	4.222	0.300	4.001	4.563
ENSG00000164011	ZNF691	-1.3	1.9E-04	5.4E-04	20.982	1.114	19.711	21.789	26.370	2.562	24.446	29.279
ENSG00000164032	H2AFZ	-1.3	5.6E-12	7.1E-11	662.171	7.819	656.619	671.112	839.448	20.659	825.485	863.180
ENSG00000164080	RAD54L2	-1.3	2.8E-08	1.6E-07	11.145	0.291	10.834	11.412	14.258	0.321	13.913	14.547
ENSG00000164134	NAA15	-1.3	2.0E-08	1.2E-07	62.025	4.305	58.834	66.921	81.134	3.211	78.922	84.817
ENSG00000164169	PRMT9	-1.3	1.3E-05	4.7E-05	13.046	0.468	12.750	13.585	16.661	0.523	16.057	16.965
ENSG00000164307	ERAP1	-1.3	7.3E-06	2.7E-05	6.495	0.461	6.022	6.944	8.392	0.242	8.153	8.636
ENSG00000164405	UQCRQ	-1.3	5.3E-09	3.6E-08	92.334	4.595	87.997	97.149	120.553	2.106	118.147	122.063
ENSG00000164535	DAGLB	-1.3	1.4E-04	4.0E-04	7.888	0.822	6.966	8.545	9.882	0.792	9.246	10.820
ENSG00000164548	TRA2A	-1.3	1.7E-07	8.4E-07	69.356	5.029	64.312	74.369	88.516	6.094	81.484	92.249
ENSG00000164654	MIOS	-1.3	3.7E-08	2.1E-07	25.222	0.641	24.713	25.941	32.031	0.882	31.054	32.769
ENSG00000164692	COL1A2	-1.3	6.9E-09	4.6E-08	21.109	0.428	20.673	21.528	26.661	0.479	26.245	27.184
ENSG00000164889	SLC4A2	-1.3	3.6E-09	2.6E-08	13.141	0.432	12.795	13.625	17.602	0.586	17.098	18.246
ENSG00000164930	FZD6	-1.3	8.4E-05	2.5E-04	10.084	0.887	9.304	11.049	12.888	0.947	11.909	13.800
ENSG00000165209	STRBP	-1.3	5.5E-12	6.9E-11	42.678	1.328	41.327	43.983	56.713	1.775	54.700	58.052
ENSG00000165490	DDIAS	-1.3	3.4E-06	1.3E-05	22.577	2.074	20.439	24.581	28.369	1.243	26.991	29.408
ENSG00000165501	LRR1	-1.3	2.0E-06	8.3E-06	26.779	0.575	26.182	27.328	34.969	0.662	34.384	35.687
ENSG00000165548	TMEM63C	-1.3	3.8E-03	7.8E-03	1.773	0.254	1.582	2.062	2.385	0.080	2.305	2.465
ENSG00000165572	KBTBD6	-1.3	2.0E-05	6.7E-05	9.590	0.559	9.013	10.128	12.500	1.313	11.416	13.961
ENSG00000165684	SNAPC4	-1.3	7.2E-04	1.8E-03	9.158	0.633	8.430	9.576	11.661	0.981	10.590	12.515
ENSG00000165724	ZMYND19	-1.3	2.5E-05	8.3E-05	29.087	1.071	28.054	30.193	36.974	3.105	33.803	40.012
ENSG00000165795	NDRG2	-1.3	2.0E-05	6.9E-05	3.901	0.134	3.785	4.048	5.218	0.150	5.093	5.384
ENSG00000165905	LARGE2	-1.3	1.9E-07	9.7E-07	39.239	1.387	38.092	40.780	50.159	3.385	46.368	52.881
ENSG00000165912	PACSIN3	-1.3	2.3E-05	7.8E-05	25.589	2.072	23.298	27.332	32.220	2.097	30.861	34.636
ENSG00000166188	ZNF319	-1.3	7.9E-03	1.5E-02	3.506	0.286	3.194	3.756	4.394	0.313	4.183	4.754
ENSG00000166206	GABRB3	-1.3	8.4E-10	6.7E-09	36.880	1.359	35.336	37.897	47.002	0.938	45.919	47.546
ENSG00000166250	CLMP	-1.3	8.3E-06	3.0E-05	16.886	1.129	16.178	18.188	21.301	0.466	20.801	21.724
ENSG00000166263	STXBP4	-1.3	8.1E-05	2.4E-04	2.894	0.276	2.599	3.147	3.678	0.161	3.506	3.822
ENSG00000166347	CYB5A	-1.3	2.7E-09	2.0E-08	22.983	0.655	22.229	23.408	29.629	0.985	28.679	30.646
ENSG00000166402	TUB	-1.3	8.6E-08	4.6E-07	22.417	0.721	21.607	22.988	28.439	0.819	27.751	29.344
ENSG00000166501	PRKCB	-1.3	1.3E-04	3.7E-04	5.385	0.271	5.072	5.547	6.962	0.462	6.657	7.494
ENSG00000166578	IQCD	-1.3	1.1E-01	1.6E-01	1.765	0.183	1.561	1.914	2.282	0.704	1.774	3.086
ENSG00000166707	ZCCHC18	-1.3	8.5E-02	1.3E-01	1.922	0.346	1.640	2.308	2.435	0.402	1.972	2.679
ENSG00000166801	FAM111A	-1.3	2.5E-07	1.2E-06	9.855	0.655	9.255	10.554	13.111	0.332	12.822	13.473
ENSG00000166803	PCLAF	-1.3	1.3E-03	2.9E-03	12.682	0.470	12.139	12.959	15.958	2.989	13.914	19.388
ENSG00000166840	GLYATL1	-1.3	1.7E-03	3.9E-03	3.074	0.215	2.864	3.293	4.020	0.429	3.585	4.442
ENSG00000166925	TSC22D4	-1.3	1.7E-04	4.8E-04	10.961	1.091	9.913	12.090	13.986	1.005	12.860	14.792
ENSG00000167106	FAM102A	-1.3	5.2E-06	2.0E-05	11.096	0.339	10.804	11.468	14.307	1.258	13.190	15.670
ENSG00000167202	TBC1D2B	-1.3	3.1E-06	1.2E-05	8.582	0.313	8.340	8.935	10.787	0.319	10.570	11.153
ENSG00000167306	MYO5B	-1.3	1.9E-04	5.4E-04	3.828	0.230	3.664	4.091	5.104	0.447	4.795	5.617
ENSG00000167447	SMG8	-1.3	2.0E-06	8.1E-06	17.016	0.808	16.208	17.824	21.980	0.818	21.043	22.558
ENSG00000167491	GATAD2A	-1.3	1.3E-09	9.8E-09	40.678	0.688	39.962	41.335	52.272	3.620	49.186	56.257
ENSG00000167566	NCKAP5L	-1.3	1.2E-05	4.3E-05	8.313	1.097	7.097	9.230	10.884	0.357	10.671	11.296
ENSG00000167600	CYP2S1	-1.3	8.2E-10	6.6E-09	117.783	6.983	111.150	125.069	153.795	5.328	150.527	159.943
ENSG00000167604	NFKBID	-1.3	1.4E-02	2.5E-02	2.505	0.151	2.335	2.623	3.384	0.550	2.752	3.752
ENSG00000167711	SERPINF2	-1.3	1.2E-01	1.7E-01	1.685	0.122	1.578	1.817	2.150	0.162	2.033	2.335
ENSG00000167716	WDR81	-1.3	1.5E-03	3.5E-03	3.247	0.212	3.023	3.446	4.074	0.271	3.820	4.360
ENSG00000167799	NUDT8	-1.3	5.9E-02	9.2E-02	4.182	0.499	3.742	4.723	5.433	0.578	5.085	6.100
ENSG00000167977	KCTD5	-1.3	2.9E-06	1.2E-05	20.557	0.653	19.813	21.037	26.004	0.748	25.316	26.801
ENSG00000167985	SDHAF2	-1.3	4.4E-03	9.0E-03	5.314	0.567	4.850	5.946	7.062	0.419	6.621	7.455
ENSG00000168056	LTBP3	-1.3	5.0E-03	1.0E-02	1.378	0.147	1.216	1.497	1.784	0.078	1.696	1.843
ENSG00000168060	NAALADL1	-1.3	1.7E-02	3.0E-02	2.232	0.138	2.137	2.391	2.918	0.268	2.642	3.178
ENSG00000168061	SAC3D1	-1.3	4.8E-03	9.8E-03	13.855	2.183	11.585	15.939	17.548	0.726	16.791	18.238
ENSG00000168066	SF1	-1.3	7.3E-09	4.9E-08	69.203	1.495	67.767	70.750	88.985	5.550	83.387	94.485
ENSG00000168096	ANKS3	-1.3	4.6E-04	1.2E-03	3.332	0.466	2.801	3.668	4.303	0.276	4.074	4.610
ENSG00000168116	KIAA1586	-1.3	2.9E-05	9.6E-05	20.055	1.582	18.245	21.171	26.580	3.361	23.188	29.909
ENSG00000168234	TTC39C	-1.3	2.1E-05	7.2E-05	10.594	0.531	9.985	10.959	13.423	0.344	13.027	13.623
ENSG00000168411	RFWD3	-1.3	1.3E-10	1.2E-09	71.034	2.490	69.379	73.898	90.549	1.823	89.301	92.641
ENSG00000168421	RHOH	-1.3	5.7E-02	9.0E-02	1.001	0.144	0.884	1.162	1.281	0.127	1.184	1.424
ENSG00000168758	SEMA4C	-1.3	8.7E-09	5.7E-08	24.840	0.498	24.317	25.309	32.328	2.586	30.827	35.325
ENSG00000168806	LCMT2	-1.3	1.8E-04	5.0E-04	3.434	0.429	2.940	3.705	4.643	0.369	4.217	4.870
ENSG00000168827	GFM1	-1.3	1.4E-10	1.3E-09	16.348	0.260	16.057	16.559	21.975	0.149	21.822	22.119
ENSG00000168906	MAT2A	-1.3	3.9E-11	4.1E-10	134.292	7.386	126.107	140.460	174.135	4.151	169.372	176.982
ENSG00000168938	PPIC	-1.3	8.9E-04	2.1E-03	9.126	0.728	8.460	9.903	11.897	0.674	11.179	12.517
ENSG00000169093	ASMTL	-1.3	2.0E-07	1.0E-06	43.446	0.196	43.273	43.659	55.864	1.809	54.744	57.952
ENSG00000169217	CD2BP2	-1.3	1.8E-07	9.2E-07	35.795	1.536	34.036	36.869	46.059	0.603	45.423	46.623
ENSG00000169635	HIC2	-1.3	1.4E-07	7.3E-07	15.445	0.504	15.088	16.009	19.716	0.809	18.783	20.186
ENSG00000169813	HNRNPF	-1.3	1.4E-13	2.4E-12	255.789	7.613	248.693	263.830	338.783	1.508	337.154	340.130
ENSG00000169972	PUSL1	-1.3	7.0E-05	2.1E-04	20.382	0.777	19.825	21.270	26.350	2.124	24.469	28.653
ENSG00000169981	ZNF35	-1.3	6.8E-05	2.1E-04	14.286	0.336	13.897	14.490	18.480	0.810	17.922	19.408
ENSG00000170145	SIK2	-1.3	1.2E-06	5.1E-06	5.747	0.535	5.170	6.227	7.646	0.104	7.573	7.764
ENSG00000170153	RNF150	-1.3	5.6E-05	1.7E-04	3.237	0.116	3.169	3.372	4.235	0.161	4.086	4.407
ENSG00000170365	SMAD1	-1.3	1.8E-05	6.3E-05	8.012	0.209	7.796	8.215	10.246	0.387	9.823	10.583
ENSG00000170385	SLC30A1	-1.3	1.3E-06	5.4E-06	17.324	0.813	16.398	17.923	22.437	1.414	20.984	23.809
ENSG00000170445	HARS	-1.3	1.0E-08	6.6E-08	16.533	0.426	16.130	16.979	21.317	0.530	20.814	21.870
ENSG00000170579	DLGAP1	-1.3	1.2E-03	2.8E-03	2.264	0.154	2.102	2.410	2.863	0.125	2.729	2.977
ENSG00000170581	STAT2	-1.3	6.0E-06	2.3E-05	10.575	0.534	9.985	11.027	13.503	0.608	12.988	14.174
ENSG00000170606	HSPA4	-1.3	3.8E-14	7.3E-13	343.366	3.577	340.483	347.369	450.309	8.580	442.641	459.583
ENSG00000170677	SOCS6	-1.3	1.0E-05	3.6E-05	12.963	1.421	11.788	14.542	16.305	0.230	16.060	16.517
ENSG00000170837	GPR27	-1.3	5.3E-05	1.6E-04	14.927	0.405	14.634	15.389	19.267	0.756	18.673	20.117
ENSG00000170871	KIAA0232	-1.3	2.7E-08	1.6E-07	21.945	0.658	21.257	22.569	27.652	0.358	27.292	28.007
ENSG00000170903	MSANTD4	-1.3	2.6E-05	8.7E-05	15.689	0.910	14.755	16.572	19.863	1.611	18.404	21.591
ENSG00000171161	ZNF672	-1.3	2.1E-07	1.0E-06	16.468	0.928	15.664	17.484	22.297	1.300	20.985	23.584
ENSG00000171204	TMEM126B	-1.3	1.1E-07	6.0E-07	57.914	2.298	56.411	60.559	73.921	2.575	70.947	75.915
ENSG00000171310	CHST11	-1.3	4.0E-04	1.0E-03	4.705	0.319	4.422	5.051	5.956	0.294	5.671	6.259
ENSG00000171488	LRRC8C	-1.3	7.5E-04	1.8E-03	1.383	0.186	1.205	1.577	1.872	0.124	1.791	2.015
ENSG00000171517	LPAR3	-1.3	5.9E-05	1.8E-04	12.600	1.113	11.678	13.836	16.354	0.419	15.889	16.704
ENSG00000171551	ECEL1	-1.3	2.3E-02	4.1E-02	2.394	0.184	2.181	2.512	3.147	0.184	3.028	3.359
ENSG00000171634	BPTF	-1.3	4.4E-10	3.7E-09	39.575	1.568	38.260	41.310	49.696	1.355	48.675	51.233
ENSG00000171681	ATF7IP	-1.3	2.3E-11	2.6E-10	38.786	0.673	38.011	39.223	49.464	1.460	47.891	50.775
ENSG00000171954	CYP4F22	-1.3	9.7E-02	1.4E-01	1.305	0.122	1.168	1.398	1.749	0.509	1.162	2.074
ENSG00000172366	MCRIP2	-1.3	1.6E-04	4.6E-04	5.503	0.732	4.906	6.320	7.335	0.803	6.410	7.848
ENSG00000172534	HCFC1	-1.3	3.7E-10	3.2E-09	40.315	1.832	38.467	42.130	54.096	3.333	51.879	57.928
ENSG00000172671	ZFAND4	-1.3	1.4E-03	3.2E-03	4.974	0.379	4.558	5.300	6.285	0.549	5.738	6.836
ENSG00000172766	NAA16	-1.3	1.8E-07	9.2E-07	17.883	0.656	17.205	18.516	23.179	1.083	22.150	24.309
ENSG00000172830	SSH3	-1.3	7.5E-05	2.3E-04	8.523	0.695	7.810	9.199	11.306	0.914	10.474	12.284
ENSG00000173064	HECTD4	-1.3	5.4E-07	2.5E-06	4.952	0.103	4.891	5.072	6.276	0.240	6.100	6.549
ENSG00000173141	MRPL57	-1.3	1.9E-06	8.0E-06	34.405	1.177	33.046	35.117	44.268	3.696	40.876	48.206
ENSG00000173402	DAG1	-1.3	1.8E-09	1.3E-08	89.679	4.550	86.193	94.826	112.649	2.755	110.451	115.740
ENSG00000173465	SSSCA1	-1.3	1.5E-05	5.3E-05	31.758	3.105	28.395	34.517	40.593	2.960	37.496	43.394
ENSG00000173540	GMPPB	-1.3	2.7E-06	1.1E-05	6.520	0.325	6.204	6.854	8.729	0.340	8.529	9.122
ENSG00000173545	ZNF622	-1.3	8.3E-07	3.7E-06	41.187	1.267	39.752	42.153	53.769	1.742	51.768	54.946
ENSG00000173581	CCDC106	-1.3	1.4E-03	3.2E-03	9.191	1.035	8.089	10.142	11.615	0.774	10.725	12.131
ENSG00000173599	PC	-1.3	2.6E-04	6.9E-04	4.821	0.097	4.714	4.902	6.254	0.274	5.939	6.442
ENSG00000173698	ADGRG2	-1.3	1.6E-05	5.6E-05	4.121	0.162	3.934	4.220	5.418	0.209	5.178	5.557
ENSG00000173926	MARCH3	-1.3	7.2E-03	1.4E-02	1.847	0.184	1.724	2.059	2.467	0.129	2.389	2.616
ENSG00000174109	C16orf91	-1.3	7.3E-04	1.8E-03	21.647	1.235	20.885	23.072	28.211	1.143	26.963	29.206
ENSG00000174151	CYB561D1	-1.3	1.5E-03	3.4E-03	2.589	0.360	2.199	2.910	3.449	0.038	3.410	3.486
ENSG00000174173	TRMT10C	-1.3	1.2E-07	6.1E-07	75.670	3.092	72.106	77.635	95.670	5.207	91.149	101.363
ENSG00000174233	ADCY6	-1.3	2.1E-04	5.9E-04	2.641	0.037	2.606	2.680	3.480	0.403	3.106	3.908
ENSG00000174243	DDX23	-1.3	4.2E-12	5.4E-11	50.394	1.104	49.529	51.638	67.521	0.988	66.407	68.336
ENSG00000174469	CNTNAP2	-1.3	6.7E-08	3.7E-07	14.477	0.965	13.362	15.046	18.580	0.728	17.933	19.368
ENSG00000174842	GLMN	-1.3	5.7E-07	2.6E-06	37.751	2.462	35.030	39.822	48.436	2.679	46.016	51.315
ENSG00000175029	CTBP2	-1.3	1.6E-11	1.8E-10	50.432	1.334	49.130	51.795	66.346	0.591	65.665	66.696
ENSG00000175213	ZNF408	-1.3	5.9E-04	1.5E-03	6.519	0.285	6.190	6.697	8.423	0.185	8.285	8.633
ENSG00000175229	GAL3ST3	-1.3	7.5E-04	1.8E-03	10.953	0.651	10.337	11.634	14.270	1.984	12.049	15.867
ENSG00000175348	TMEM9B	-1.3	4.6E-05	1.5E-04	8.484	0.562	8.028	9.112	11.354	0.682	10.600	11.927
ENSG00000175730	BAK1P1	-1.3	1.6E-02	2.8E-02	21.970	1.614	20.121	23.097	27.639	1.274	26.306	28.845
ENSG00000175756	AURKAIP1	-1.3	1.2E-08	7.4E-08	101.518	3.531	97.887	104.939	131.228	8.413	125.293	140.856
ENSG00000176022	B3GALT6	-1.3	8.9E-04	2.1E-03	10.465	0.403	10.005	10.752	13.484	1.927	11.620	15.468
ENSG00000176058	TPRN	-1.3	7.1E-05	2.2E-04	5.959	0.580	5.487	6.606	7.937	0.680	7.249	8.609
ENSG00000176248	ANAPC2	-1.3	2.7E-06	1.1E-05	8.572	0.303	8.274	8.880	11.435	0.909	10.386	12.002
ENSG00000176597	B3GNT5	-1.3	2.1E-05	7.2E-05	4.709	0.318	4.456	5.066	6.353	0.769	5.511	7.019
ENSG00000177144	NUDT4P1	-1.3	1.9E-05	6.3E-05	27.421	3.010	24.936	30.767	36.756	0.579	36.257	37.390
ENSG00000177169	ULK1	-1.3	2.2E-07	1.1E-06	16.015	0.734	15.277	16.745	20.357	0.665	19.738	21.060
ENSG00000177303	CASKIN2	-1.3	4.8E-05	1.5E-04	8.841	0.528	8.302	9.357	11.301	0.482	10.768	11.707
ENSG00000177370	TIMM22	-1.3	9.0E-04	2.2E-03	18.901	1.235	17.635	20.103	23.750	3.115	20.512	26.726
ENSG00000177663	IL17RA	-1.3	1.2E-04	3.6E-04	3.219	0.118	3.126	3.352	4.219	0.210	4.045	4.452
ENSG00000177733	HNRNPA0	-1.3	2.7E-13	4.5E-12	103.187	2.859	100.417	106.127	135.189	2.224	133.133	137.550
ENSG00000178078	STAP2	-1.3	2.0E-03	4.4E-03	8.313	0.878	7.310	8.945	10.494	0.202	10.353	10.725
ENSG00000178202	KDELC2	-1.3	1.1E-07	5.8E-07	18.702	0.474	18.378	19.246	24.012	0.608	23.512	24.689
ENSG00000178773	CPNE7	-1.3	5.6E-02	8.8E-02	2.350	0.123	2.237	2.481	2.958	0.538	2.629	3.579
ENSG00000178921	PFAS	-1.3	5.3E-10	4.4E-09	51.501	1.493	49.842	52.735	69.207	2.123	67.610	71.616
ENSG00000178935	ZNF552	-1.3	1.7E-03	3.8E-03	8.610	1.205	7.531	9.910	11.108	1.522	9.760	12.758
ENSG00000178950	GAK	-1.3	1.2E-08	7.8E-08	8.298	0.062	8.238	8.357	10.750	0.695	9.955	11.238
ENSG00000178951	ZBTB7A	-1.3	3.9E-04	1.0E-03	5.409	0.580	4.826	5.986	7.030	0.611	6.368	7.574
ENSG00000179051	RCC2	-1.3	2.2E-13	3.7E-12	354.533	7.846	348.713	363.456	468.526	8.526	462.713	478.322
ENSG00000179134	SAMD4B	-1.3	7.4E-09	4.9E-08	31.741	1.888	29.617	33.230	40.442	2.120	38.524	42.718
ENSG00000179331	RAB39A	-1.3	1.3E-04	3.7E-04	15.763	0.501	15.351	16.321	20.681	1.986	18.629	22.594
ENSG00000179409	GEMIN4	-1.3	4.2E-08	2.4E-07	24.762	1.727	23.484	26.726	32.016	1.455	30.337	32.927
ENSG00000179542	SLITRK4	-1.3	2.4E-04	6.4E-04	3.945	0.621	3.567	4.662	5.128	0.289	4.803	5.359
ENSG00000179750	APOBEC3B	-1.3	6.2E-03	1.2E-02	9.567	0.719	8.917	10.339	12.063	0.609	11.455	12.674
ENSG00000179862	CITED4	-1.3	1.9E-02	3.3E-02	9.553	0.746	8.914	10.372	12.518	1.959	10.296	13.995
ENSG00000179943	FIZ1	-1.3	1.1E-03	2.5E-03	3.351	0.294	3.120	3.683	4.352	0.419	3.971	4.800
ENSG00000179950	PUF60	-1.3	8.0E-09	5.3E-08	49.309	1.251	47.867	50.112	63.282	3.997	59.671	67.578
ENSG00000180035	ZNF48	-1.3	1.3E-06	5.4E-06	20.938	0.985	19.813	21.647	27.549	0.714	27.112	28.372
ENSG00000180104	EXOC3	-1.3	7.2E-06	2.6E-05	7.876	0.457	7.479	8.376	10.200	1.017	9.416	11.350
ENSG00000180376	CCDC66	-1.3	5.6E-06	2.1E-05	9.019	0.808	8.195	9.809	11.496	0.741	10.977	12.345
ENSG00000180423	HARBI1	-1.3	5.2E-02	8.2E-02	3.041	0.242	2.831	3.305	3.892	0.872	2.886	4.436
ENSG00000180573	HIST1H2AC	-1.3	2.4E-04	6.6E-04	24.043	1.599	23.016	25.885	30.113	2.062	28.574	32.456
ENSG00000180626	ZNF594	-1.3	2.4E-05	8.0E-05	9.916	0.631	9.411	10.623	12.690	0.144	12.541	12.828
ENSG00000180900	SCRIB	-1.3	4.5E-07	2.1E-06	28.538	0.278	28.222	28.741	35.849	2.484	33.749	38.605
ENSG00000180921	FAM83H	-1.3	1.1E-06	4.9E-06	21.289	0.753	20.420	21.730	28.599	2.004	27.201	30.895
ENSG00000181027	FKRP	-1.3	8.2E-05	2.5E-04	8.269	0.344	7.891	8.563	10.556	0.162	10.420	10.735
ENSG00000181135	ZNF707	-1.3	4.3E-04	1.1E-03	3.086	0.253	2.796	3.269	4.168	0.508	3.644	4.658
ENSG00000181472	ZBTB2	-1.3	4.8E-07	2.2E-06	20.850	0.686	20.330	21.627	28.066	0.849	27.124	28.773
ENSG00000181555	SETD2	-1.3	4.4E-09	3.0E-08	22.595	1.223	21.183	23.329	28.652	0.871	27.648	29.193
ENSG00000182154	MRPL41	-1.3	2.5E-05	8.2E-05	45.254	1.196	43.907	46.194	60.366	4.186	55.676	63.723
ENSG00000182240	BACE2	-1.3	3.9E-03	8.1E-03	2.398	0.236	2.129	2.567	3.005	0.330	2.643	3.289
ENSG00000182272	B4GALNT4	-1.3	4.3E-05	1.4E-04	20.366	2.247	19.054	22.960	25.746	2.289	24.410	28.389
ENSG00000182325	FBXL6	-1.3	2.5E-03	5.5E-03	9.297	0.616	8.872	10.004	11.771	1.252	10.471	12.970
ENSG00000182446	NPLOC4	-1.3	3.5E-11	3.8E-10	24.488	0.478	23.939	24.811	32.645	0.081	32.558	32.718
ENSG00000182621	PLCB1	-1.3	1.9E-04	5.1E-04	1.395	0.057	1.330	1.437	1.850	0.113	1.752	1.974
ENSG00000182704	TSKU	-1.3	2.2E-05	7.3E-05	11.904	0.977	11.002	12.942	15.336	0.460	14.805	15.613
ENSG00000182768	NGRN	-1.3	4.2E-07	1.9E-06	16.322	0.828	15.696	17.261	20.921	1.416	19.499	22.331
ENSG00000182866	LCK	-1.3	7.7E-10	6.2E-09	54.931	0.543	54.350	55.425	72.010	2.935	69.686	75.308
ENSG00000183287	CCBE1	-1.3	1.6E-03	3.6E-03	2.164	0.171	1.971	2.295	2.929	0.150	2.758	3.037
ENSG00000183527	PSMG1	-1.3	3.3E-07	1.6E-06	55.546	3.302	51.797	58.022	69.891	1.365	68.479	71.205
ENSG00000183580	FBXL7	-1.3	1.1E-05	3.9E-05	10.460	0.269	10.271	10.768	13.462	0.921	12.403	14.077
ENSG00000183621	ZNF438	-1.3	3.2E-02	5.4E-02	1.766	0.257	1.599	2.062	2.301	0.277	2.109	2.619
ENSG00000183665	TRMT12	-1.3	7.1E-05	2.2E-04	22.662	0.617	21.984	23.188	28.793	2.236	26.325	30.682
ENSG00000183688	RFLNB	-1.3	3.7E-08	2.1E-07	26.272	1.767	24.924	28.272	35.344	1.342	33.871	36.497
ENSG00000183751	TBL3	-1.3	1.2E-08	7.8E-08	18.668	0.966	18.076	19.783	24.568	0.384	24.243	24.991
ENSG00000183864	TOB2	-1.3	4.3E-08	2.4E-07	33.554	1.789	31.495	34.729	42.601	0.662	41.865	43.146
ENSG00000183978	COA3	-1.3	8.8E-07	3.9E-06	33.610	2.683	30.741	36.058	44.406	0.700	43.607	44.914
ENSG00000184156	KCNQ3	-1.3	1.1E-02	2.0E-02	1.116	0.042	1.074	1.158	1.458	0.223	1.262	1.701
ENSG00000184281	TSSC4	-1.3	3.1E-05	1.0E-04	15.187	0.942	14.106	15.828	19.981	0.271	19.669	20.154
ENSG00000184602	SNN	-1.3	3.4E-09	2.4E-08	69.243	4.444	64.871	73.755	89.428	2.799	87.052	92.514
ENSG00000184860	SDR42E1	-1.3	2.6E-04	7.0E-04	1.985	0.045	1.946	2.034	2.626	0.278	2.347	2.904
ENSG00000184922	FMNL1	-1.3	4.4E-03	9.1E-03	2.284	0.215	2.057	2.486	2.926	0.126	2.784	3.024
ENSG00000184990	SIVA1	-1.3	1.2E-07	6.5E-07	26.322	2.683	23.380	28.633	35.491	1.952	33.752	37.602
ENSG00000185024	BRF1	-1.3	1.3E-03	3.1E-03	2.110	0.135	1.965	2.231	2.641	0.284	2.376	2.941
ENSG00000185033	SEMA4B	-1.3	4.9E-07	2.3E-06	14.477	0.739	13.703	15.175	18.899	1.002	17.984	19.970
ENSG00000185163	DDX51	-1.3	5.1E-06	1.9E-05	9.341	0.397	8.883	9.579	12.222	0.900	11.697	13.261
ENSG00000185274	WBSCR17	-1.3	8.8E-07	3.9E-06	17.295	0.598	16.703	17.898	21.957	0.870	21.111	22.848
ENSG00000185298	CCDC137	-1.3	1.9E-07	9.3E-07	28.768	1.221	27.915	30.167	38.474	2.104	36.566	40.731
ENSG00000185338	SOCS1	-1.3	5.2E-02	8.2E-02	41.542	1.995	39.487	43.470	54.539	11.415	43.560	66.344
ENSG00000185339	TCN2	-1.3	6.9E-08	3.8E-07	25.222	0.849	24.275	25.914	33.441	0.508	33.099	34.025
ENSG00000185379	RAD51D	-1.3	3.8E-05	1.2E-04	2.955	0.114	2.826	3.044	3.863	0.159	3.679	3.967
ENSG00000185630	PBX1	-1.3	1.5E-09	1.1E-08	22.554	0.900	21.733	23.517	28.484	1.041	27.360	29.415
ENSG00000185684	EP400NL	-1.3	4.0E-05	1.3E-04	2.446	0.295	2.252	2.786	3.284	0.191	3.071	3.441
ENSG00000185838	GNB1L	-1.3	1.1E-03	2.6E-03	3.277	0.265	3.092	3.581	4.215	0.456	3.838	4.722
ENSG00000186007	LEMD1	-1.3	1.8E-02	3.3E-02	4.369	0.537	3.986	4.984	5.715	1.044	4.589	6.649
ENSG00000186111	PIP5K1C	-1.3	1.7E-06	7.2E-06	9.868	0.279	9.557	10.094	13.013	1.586	11.816	14.825
ENSG00000186340	THBS2	-1.3	2.8E-04	7.5E-04	6.966	0.455	6.699	7.491	9.261	0.718	8.756	10.083
ENSG00000186462	NAP1L2	-1.3	8.5E-07	3.8E-06	30.668	0.353	30.386	31.063	39.771	2.566	37.272	42.399
ENSG00000186470	BTN3A2	-1.3	7.0E-03	1.4E-02	1.916	0.148	1.757	2.049	2.464	0.159	2.296	2.613
ENSG00000186479	RGS7BP	-1.3	2.0E-02	3.5E-02	2.501	0.154	2.338	2.644	3.162	0.359	2.871	3.564
ENSG00000186532	SMYD4	-1.3	2.3E-07	1.1E-06	8.335	0.468	7.808	8.700	11.285	0.617	10.580	11.731
ENSG00000186998	EMID1	-1.3	1.1E-02	2.1E-02	1.757	0.320	1.418	2.054	2.361	0.132	2.210	2.452
ENSG00000187134	AKR1C1	-1.3	8.3E-02	1.2E-01	0.566	0.067	0.505	0.638	0.725	0.062	0.688	0.797
ENSG00000187140	FOXD3	-1.3	3.1E-04	8.2E-04	13.989	0.205	13.763	14.162	18.042	1.976	16.005	19.950
ENSG00000187243	MAGED4B	-1.3	1.7E-07	8.8E-07	18.165	0.747	17.446	18.938	23.849	1.521	22.236	25.258
ENSG00000187607	ZNF286A	-1.3	8.3E-07	3.7E-06	15.297	0.189	15.106	15.484	19.326	0.641	18.591	19.775
ENSG00000187626	ZKSCAN4	-1.3	3.7E-03	7.7E-03	7.475	0.809	6.954	8.406	9.628	0.666	9.116	10.381
ENSG00000187676	B3GLCT	-1.3	1.4E-03	3.3E-03	5.202	0.584	4.665	5.823	6.627	0.424	6.149	6.958
ENSG00000187713	TMEM203	-1.3	1.7E-07	8.6E-07	46.312	2.234	44.411	48.773	61.582	1.418	60.379	63.145
ENSG00000187730	GABRD	-1.3	1.0E-01	1.5E-01	0.557	0.031	0.526	0.589	0.734	0.040	0.687	0.759
ENSG00000187824	TMEM220	-1.3	2.5E-02	4.3E-02	1.908	0.102	1.848	2.027	2.507	0.253	2.230	2.725
ENSG00000187867	PALM3	-1.3	3.0E-06	1.2E-05	31.294	2.834	29.310	34.540	40.202	1.815	38.115	41.406
ENSG00000187957	DNER	-1.3	4.1E-03	8.5E-03	3.462	0.113	3.334	3.548	4.538	0.313	4.180	4.757
ENSG00000187994	RINL	-1.3	3.9E-04	1.0E-03	5.612	0.260	5.449	5.912	7.443	0.738	6.593	7.914
ENSG00000188092	GPR89B	-1.3	9.5E-06	3.4E-05	9.403	0.567	8.806	9.934	12.196	0.292	11.905	12.488
ENSG00000188227	ZNF793	-1.3	1.4E-04	4.1E-04	3.214	0.210	2.974	3.363	4.113	0.385	3.786	4.537
ENSG00000188322	SBK1	-1.3	1.9E-07	9.6E-07	45.500	2.215	43.279	47.709	60.681	4.053	56.580	64.685
ENSG00000188505	NCCRP1	-1.3	1.4E-01	1.9E-01	1.660	0.343	1.327	2.013	2.149	0.365	1.836	2.551
ENSG00000188517	COL25A1	-1.3	1.5E-03	3.3E-03	3.848	0.483	3.291	4.153	4.847	0.205	4.712	5.083
ENSG00000188848	BEND4	-1.3	6.7E-10	5.5E-09	33.593	0.202	33.390	33.793	43.998	2.344	41.414	45.987
ENSG00000189221	MAOA	-1.3	1.1E-02	2.1E-02	3.091	0.815	2.232	3.853	3.986	0.100	3.897	4.095
ENSG00000196208	GREB1	-1.3	6.3E-07	2.8E-06	8.628	0.200	8.416	8.813	11.136	0.692	10.459	11.842
ENSG00000196235	SUPT5H	-1.3	3.1E-11	3.4E-10	26.179	0.846	25.365	27.053	34.939	1.091	33.748	35.889
ENSG00000196363	WDR5	-1.3	9.3E-10	7.4E-09	59.597	3.392	55.993	62.728	77.581	2.392	74.829	79.153
ENSG00000196466	ZNF799	-1.3	1.7E-02	3.0E-02	2.047	0.203	1.895	2.278	2.662	0.373	2.239	2.943
ENSG00000196517	SLC6A9	-1.3	5.0E-04	1.3E-03	7.703	0.552	7.330	8.338	9.821	1.219	8.612	11.049
ENSG00000196655	TRAPPC4	-1.3	2.4E-07	1.2E-06	17.029	0.908	16.137	17.953	22.257	0.703	21.472	22.828
ENSG00000196663	TECPR2	-1.3	1.6E-05	5.6E-05	6.477	0.452	6.015	6.919	8.370	0.284	8.053	8.600
ENSG00000196678	ERI2	-1.3	6.4E-05	2.0E-04	6.034	0.241	5.858	6.309	7.665	0.213	7.500	7.906
ENSG00000196787	HIST1H2AG	-1.3	9.5E-02	1.4E-01	3.439	0.829	2.538	4.171	4.361	1.310	3.187	5.774
ENSG00000196812	ZSCAN16	-1.3	5.2E-03	1.1E-02	12.348	1.324	11.042	13.690	16.111	1.020	15.300	17.256
ENSG00000196843	ARID5A	-1.3	3.0E-03	6.4E-03	3.830	0.370	3.507	4.234	5.110	0.549	4.614	5.699
ENSG00000196975	ANXA4	-1.3	1.4E-03	3.2E-03	2.080	0.104	1.969	2.174	2.636	0.132	2.506	2.769
ENSG00000197024	ZNF398	-1.3	1.9E-10	1.7E-09	21.541	0.630	20.823	22.007	28.888	1.137	27.956	30.162
ENSG00000197124	ZNF682	-1.3	5.3E-05	1.6E-04	9.407	1.462	8.202	11.033	12.535	0.634	12.022	13.243
ENSG00000197128	ZNF772	-1.3	4.2E-05	1.3E-04	7.178	0.588	6.684	7.828	9.188	0.295	8.910	9.498
ENSG00000197134	ZNF257	-1.3	4.3E-05	1.4E-04	7.315	0.819	6.602	8.209	9.893	1.239	9.086	11.319
ENSG00000197170	PSMD12	-1.3	2.3E-11	2.6E-10	54.192	2.078	51.795	55.498	72.319	3.010	70.329	75.782
ENSG00000197256	KANK2	-1.3	8.1E-06	2.9E-05	13.324	0.186	13.199	13.537	16.765	1.196	15.388	17.550
ENSG00000197451	HNRNPAB	-1.3	4.7E-12	6.0E-11	623.604	13.054	608.540	631.618	789.571	20.441	771.608	811.813
ENSG00000197619	ZNF615	-1.3	1.7E-04	4.6E-04	6.430	0.320	6.147	6.778	8.280	0.205	8.046	8.425
ENSG00000197780	TAF13	-1.3	1.1E-05	3.7E-05	46.883	1.447	45.449	48.344	60.000	3.111	56.940	63.159
ENSG00000197798	FAM118B	-1.3	3.3E-08	1.9E-07	26.213	0.874	25.238	26.929	34.899	1.769	32.863	36.058
ENSG00000197857	ZNF44	-1.3	1.0E-02	1.9E-02	2.152	0.344	1.900	2.544	2.740	0.265	2.452	2.973
ENSG00000197935	ZNF311	-1.3	2.0E-03	4.4E-03	5.118	0.638	4.387	5.571	6.692	0.302	6.483	7.038
ENSG00000197977	ELOVL2	-1.3	7.8E-02	1.2E-01	1.136	0.107	1.067	1.259	1.511	0.560	0.955	2.075
ENSG00000198055	GRK6	-1.3	4.2E-07	2.0E-06	22.170	0.370	21.810	22.550	28.806	2.103	26.462	30.529
ENSG00000198093	ZNF649	-1.3	3.0E-09	2.2E-08	37.699	1.787	35.736	39.233	49.935	0.836	49.006	50.627
ENSG00000198134		-1.3	8.7E-04	2.1E-03	64.200	1.430	63.052	65.802	83.743	7.409	77.941	92.088
ENSG00000198231	DDX42	-1.3	1.9E-10	1.7E-09	55.344	2.134	53.273	57.536	69.733	0.879	68.951	70.684
ENSG00000198301	SDAD1	-1.3	3.6E-10	3.1E-09	54.825	0.939	54.029	55.861	71.310	1.251	70.447	72.745
ENSG00000198429	ZNF69	-1.3	2.2E-04	6.1E-04	9.375	1.043	8.689	10.575	12.720	0.899	11.896	13.678
ENSG00000198431	TXNRD1	-1.3	8.1E-14	1.5E-12	94.794	0.794	94.130	95.674	126.434	1.090	125.405	127.577
ENSG00000198435	NRARP	-1.3	7.3E-05	2.2E-04	16.830	1.939	15.407	19.039	22.328	0.955	21.229	22.958
ENSG00000198492	YTHDF2	-1.3	3.0E-12	4.0E-11	84.532	0.908	83.534	85.310	111.498	1.648	110.295	113.376
ENSG00000198580		-1.3	3.9E-02	6.3E-02	7.695	1.593	6.402	9.475	10.368	1.571	8.798	11.940
ENSG00000198585	NUDT16	-1.3	3.8E-08	2.2E-07	19.419	0.667	18.994	20.188	24.870	0.162	24.702	25.026
ENSG00000198743	SLC5A3	-1.3	1.3E-06	5.5E-06	18.096	0.661	17.640	18.854	22.943	1.354	21.486	24.163
ENSG00000198793	MTOR	-1.3	5.6E-11	5.8E-10	32.174	1.521	31.127	33.919	41.572	0.415	41.103	41.891
ENSG00000198816	ZNF358	-1.3	1.5E-03	3.5E-03	19.458	2.619	16.636	21.810	26.066	2.716	23.158	28.538
ENSG00000198824	CHAMP1	-1.3	4.2E-07	1.9E-06	25.377	0.367	24.953	25.607	32.002	1.430	30.376	33.067
ENSG00000198924	DCLRE1A	-1.3	3.8E-06	1.5E-05	16.394	0.673	15.632	16.908	21.181	0.568	20.692	21.804
ENSG00000198933	TBKBP1	-1.3	1.4E-04	3.9E-04	6.722	0.397	6.312	7.104	8.777	0.807	7.866	9.399
ENSG00000198934	MAGEE1	-1.3	2.2E-05	7.4E-05	10.256	0.273	9.992	10.537	13.423	0.365	13.014	13.711
ENSG00000198945	L3MBTL3	-1.3	5.7E-08	3.2E-07	17.290	0.741	16.445	17.826	22.714	0.212	22.536	22.949
ENSG00000198952	SMG5	-1.3	1.9E-11	2.1E-10	64.985	0.582	64.328	65.432	86.889	4.436	82.143	90.932
ENSG00000200087	SNORA73B	-1.3	1.1E-01	1.5E-01	18.241	5.549	13.619	24.395	24.332	5.592	18.048	28.759
ENSG00000203499	IQANK1	-1.3	4.8E-02	7.6E-02	1.024	0.153	0.912	1.199	1.347	0.042	1.298	1.374
ENSG00000204252	HLA-DOA	-1.3	4.2E-07	2.0E-06	26.562	0.783	25.699	27.229	33.977	1.167	32.629	34.678
ENSG00000204310	AGPAT1	-1.3	9.7E-07	4.2E-06	42.393	2.290	39.786	44.081	53.428	2.162	50.936	54.793
ENSG00000204344	STK19	-1.3	3.6E-03	7.5E-03	4.815	0.449	4.471	5.323	6.070	0.374	5.742	6.477
ENSG00000204370	SDHD	-1.3	1.2E-07	6.4E-07	57.952	2.755	55.236	60.745	72.701	2.092	70.455	74.594
ENSG00000204371	EHMT2	-1.3	7.3E-13	1.1E-11	42.694	0.664	41.933	43.156	57.564	1.309	56.075	58.530
ENSG00000204427	ABHD16A	-1.3	4.7E-02	7.5E-02	1.482	0.295	1.290	1.821	1.926	0.328	1.581	2.233
ENSG00000204472	AIF1	-1.3	4.3E-03	8.9E-03	9.072	0.251	8.905	9.360	11.969	0.620	11.349	12.589
ENSG00000204590	GNL1	-1.3	8.9E-10	7.1E-09	18.641	0.414	18.281	19.093	25.187	0.367	24.847	25.576
ENSG00000204611	ZNF616	-1.3	1.7E-07	8.7E-07	21.134	1.229	19.944	22.399	27.213	1.211	25.970	28.389
ENSG00000204624	DISP3	-1.3	1.6E-02	2.8E-02	2.249	0.331	1.968	2.614	2.875	0.319	2.655	3.241
ENSG00000204652	RPS26P8	-1.3	5.0E-04	1.3E-03	109.384	12.874	94.570	117.860	137.973	8.825	127.797	143.522
ENSG00000204991	SPIRE2	-1.3	6.8E-04	1.7E-03	2.460	0.030	2.439	2.478	3.185	0.144	3.048	3.334
ENSG00000205918	PDPK2P	-1.3	6.1E-02	9.4E-02	1.359	0.224	1.168	1.606	1.728	0.060	1.664	1.783

ENSG00000206562	METTL6	-1.3	3.2E-04	8.4E-04	6.543	0.292	6.221	6.789	8.198	0.554	7.775	8.825
ENSG00000210082	MT-RNR2	-1.3	6.0E-12	7.6E-11	6773.892	183.227	6585.129	6961.279	8625.137	271.157	8355.706	8897.988
ENSG00000211460	TSN	-1.3	4.0E-11	4.3E-10	86.888	1.565	85.598	88.629	111.319	2.003	109.102	112.997
ENSG00000213047	DENND1B	-1.3	1.3E-03	2.9E-03	1.134	0.066	1.064	1.195	1.502	0.103	1.384	1.576
ENSG00000213341	CHUK	-1.3	1.3E-05	4.6E-05	12.840	1.748	11.830	14.859	17.439	1.495	15.943	18.934
ENSG00000213551	DNAJC9	-1.3	2.5E-08	1.5E-07	26.648	0.634	26.219	27.377	34.626	0.884	34.067	35.645
ENSG00000213638	ADAT3	-1.3	6.2E-02	9.6E-02	3.155	0.588	2.685	3.814	4.008	0.688	3.372	4.755
ENSG00000213676	ATF6B	-1.3	2.6E-06	1.0E-05	15.277	1.615	13.571	16.783	20.144	0.835	19.322	20.991
ENSG00000213760	ATP6V1G2	-1.3	4.9E-02	7.8E-02	2.112	0.431	1.739	2.583	2.821	0.310	2.602	3.176
ENSG00000213888	ZNF90	-1.3	1.9E-08	1.2E-07	26.039	0.734	25.279	26.745	34.290	2.154	32.813	36.762
ENSG00000214077	GNAQP1	-1.3	1.8E-02	3.2E-02	9.446	1.109	8.752	10.725	12.042	1.457	10.670	13.572
ENSG00000214189	ZNF788	-1.3	8.6E-07	3.8E-06	15.936	0.441	15.524	16.401	20.031	0.980	18.969	20.901
ENSG00000214435	AS3MT	-1.3	9.0E-02	1.3E-01	2.059	0.788	1.485	2.957	2.718	0.301	2.382	2.963
ENSG00000214892	USP8P1	-1.3	1.1E-01	1.6E-01	0.745	0.085	0.679	0.841	1.004	0.088	0.942	1.104
ENSG00000215039	CD27-AS1	-1.3	1.8E-02	3.1E-02	3.448	0.216	3.236	3.668	4.347	0.423	3.878	4.699
ENSG00000215041	NEURL4	-1.3	1.6E-06	6.6E-06	10.202	0.210	9.971	10.382	13.256	0.680	12.646	13.990
ENSG00000215319	EIF5P1	-1.3	1.0E-01	1.5E-01	3.705	0.938	2.696	4.549	4.790	0.531	4.255	5.317
ENSG00000215883	CYB5RL	-1.3	5.3E-05	1.6E-04	4.123	0.276	3.887	4.427	5.426	0.384	5.077	5.837
ENSG00000219438	FAM19A5	-1.3	5.4E-05	1.7E-04	14.028	0.482	13.556	14.519	18.219	1.619	16.905	20.027
ENSG00000224081	SLC44A3-AS1	-1.3	1.3E-01	1.9E-01	0.509	0.206	0.289	0.697	0.646	0.069	0.586	0.721
ENSG00000224183	SDHDP6	-1.3	1.2E-02	2.3E-02	19.053	1.219	18.176	20.445	24.868	2.906	21.542	26.919
ENSG00000224805		-1.3	4.6E-02	7.4E-02	4.051	0.289	3.760	4.338	5.158	0.613	4.468	5.640
ENSG00000224914	LINC00863	-1.3	1.8E-02	3.2E-02	2.973	0.522	2.664	3.576	3.830	0.478	3.304	4.240
ENSG00000225968	ELFN1	-1.3	4.3E-02	7.0E-02	1.724	0.118	1.590	1.815	2.203	0.056	2.163	2.267
ENSG00000225973	PIGBOS1	-1.3	5.0E-03	1.0E-02	9.085	0.909	8.173	9.991	12.307	1.173	11.526	13.656
ENSG00000226085	UQCRFS1P1	-1.3	1.0E-04	3.0E-04	52.291	1.806	50.741	54.274	66.579	3.703	62.349	69.234
ENSG00000226650	KIF4B	-1.3	2.0E-02	3.5E-02	1.819	0.169	1.706	2.014	2.443	0.115	2.345	2.569
ENSG00000226752	PSMD5-AS1	-1.3	4.1E-04	1.1E-03	3.838	0.531	3.483	4.449	5.067	0.615	4.437	5.666
ENSG00000227398	KIF9-AS1	-1.3	1.0E-01	1.5E-01	0.750	0.123	0.639	0.882	0.973	0.153	0.799	1.086
ENSG00000227825	SLC9A7P1	-1.3	1.3E-01	1.9E-01	1.413	0.341	1.026	1.667	1.788	0.200	1.561	1.936
ENSG00000228223	HCG11	-1.3	2.5E-05	8.3E-05	9.275	0.845	8.679	10.242	12.447	1.028	11.850	13.634
ENSG00000228285	LYPLA2P1	-1.3	9.8E-04	2.3E-03	35.878	3.029	34.041	39.374	46.068	6.194	41.545	53.128
ENSG00000228716	DHFR	-1.3	4.8E-11	5.0E-10	64.889	1.124	63.974	66.143	87.594	2.343	85.424	90.079
ENSG00000228766	RPL7L1P8	-1.3	1.6E-02	2.8E-02	16.029	4.750	10.727	19.897	21.342	3.294	19.257	25.140
ENSG00000229589	ACVR2B-AS1	-1.3	3.8E-02	6.3E-02	3.730	0.288	3.418	3.986	4.685	0.402	4.413	5.147
ENSG00000229676	ZNF492	-1.3	8.2E-03	1.6E-02	3.101	0.092	3.023	3.202	3.887	0.150	3.865	4.154
ENSG00000230568	SF3A3P1	-1.3	4.0E-02	6.6E-02	5.779	1.145	4.494	6.691	7.323	0.871	6.809	8.329
ENSG00000230793	SMARCE1P5	-1.3	4.1E-02	6.6E-02	7.644	1.472	6.741	9.342	9.633	1.401	8.039	10.674
ENSG00000231711	LINC00899	-1.3	1.1E-01	1.6E-01	0.688	0.135	0.591	0.842	0.905	0.257	0.682	1.185
ENSG00000231831	MTHFD1P1	-1.3	1.6E-03	3.6E-03	5.310	0.105	5.196	5.403	7.128	0.495	6.791	7.696
ENSG00000232702		-1.3	5.8E-03	1.2E-02	35.619	5.825	29.480	41.069	45.604	3.030	42.793	48.814
ENSG00000232931	LINC00342	-1.3	2.1E-02	3.7E-02	2.314	0.545	1.710	2.770	3.116	0.113	3.041	3.247
ENSG00000233922	LINC01694	-1.3	1.8E-02	3.2E-02	1.500	0.184	1.382	1.712	1.956	0.174	1.755	2.062
ENSG00000234284	ZNF879	-1.3	2.3E-02	4.0E-02	2.018	0.249	1.868	2.305	2.659	0.577	2.209	3.310
ENSG00000234420	ZNF37BP	-1.3	2.4E-04	6.6E-04	3.769	0.401	3.386	4.187	4.803	0.115	4.680	4.908
ENSG00000234616	JRK	-1.3	9.1E-08	4.8E-07	11.075	0.260	10.801	11.319	14.087	0.204	13.892	14.298
ENSG00000234857	HNRNPUL2-B	-1.3	4.6E-02	7.4E-02	1.438	0.248	1.177	1.670	1.906	0.245	1.711	2.181
ENSG00000235008		-1.3	5.5E-05	1.7E-04	20.135	0.607	19.740	20.834	26.736	0.965	25.735	27.660
ENSG00000235489	DBF4P1	-1.3	3.9E-06	1.5E-05	20.110	0.858	19.294	21.005	27.158	1.913	24.973	28.536
ENSG00000237440	ZNF737	-1.3	7.3E-05	2.2E-04	9.966	0.396	9.515	10.254	12.888	0.610	12.341	13.545
ENSG00000237923		-1.3	5.6E-04	1.4E-03	32.514	2.104	30.136	34.130	41.753	2.761	38.571	43.506
ENSG00000238084		-1.3	5.5E-04	1.4E-03	50.290	4.651	45.042	53.899	64.664	3.389	60.764	66.890
ENSG00000239306	RBM14	-1.3	1.2E-05	4.1E-05	41.295	1.324	40.185	42.761	55.261	2.377	52.530	56.872
ENSG00000239857	GET4	-1.3	1.2E-03	2.8E-03	3.463	0.386	3.033	3.778	4.581	0.714	4.028	5.387
ENSG00000241258	CRCP	-1.3	7.9E-07	3.5E-06	18.447	1.634	16.569	19.533	24.315	0.332	24.037	24.682
ENSG00000241697	TMEFF1	-1.3	7.7E-02	1.2E-01	6.270	1.088	5.555	7.522	8.143	1.630	6.420	9.662
ENSG00000242485	MRPL20	-1.3	1.2E-09	9.4E-09	61.674	2.201	59.502	63.903	81.290	4.607	77.109	86.229
ENSG00000242616	GNG10	-1.3	3.9E-02	6.5E-02	4.461	0.383	4.193	4.899	5.848	1.483	4.390	7.354
ENSG00000243232	PCDHAC2	-1.3	2.7E-03	5.7E-03	2.030	0.135	1.927	2.183	2.730	0.289	2.426	3.000
ENSG00000243725	TTC4	-1.3	8.8E-03	1.7E-02	3.308	0.173	3.195	3.507	4.287	0.386	3.906	4.678
ENSG00000244486	SCARF2	-1.3	2.5E-03	5.4E-03	3.848	0.172	3.650	3.958	5.189	0.597	4.808	5.877
ENSG00000244625	MIATNB	-1.3	9.2E-02	1.4E-01	1.375	0.031	1.348	1.410	1.786	0.357	1.383	2.061
ENSG00000247595	SPTY2D1-AS1	-1.3	1.4E-01	2.0E-01	1.281	0.548	0.676	1.744	1.723	0.348	1.346	2.032
ENSG00000247626	MARS2	-1.3	5.8E-07	2.6E-06	24.776	2.051	23.211	27.098	32.408	0.309	32.086	32.702
ENSG00000248329	APELA	-1.3	5.3E-02	8.4E-02	33.840	0.921	32.828	34.629	43.575	3.478	39.862	46.759
ENSG00000248546	ANP32C	-1.3	9.1E-02	1.3E-01	5.543	0.726	4.950	6.353	7.413	1.196	6.576	8.784
ENSG00000249129	SUDS3P1	-1.3	1.5E-04	4.4E-04	25.326	2.470	22.600	27.414	34.077	2.882	31.379	37.113
ENSG00000249459	ZNF286B	-1.3	1.6E-05	5.5E-05	8.942	0.135	8.788	9.040	11.548	0.805	10.721	12.329
ENSG00000249485	RBBP4P1	-1.3	2.0E-05	6.8E-05	37.279	1.316	36.128	38.714	49.624	3.861	46.993	54.057
ENSG00000249532		-1.3	2.5E-08	1.5E-07	1293.106	7.218	1286.102	1300.521	1685.990	131.527	1534.672	1772.893
ENSG00000249550	LINC01234	-1.3	8.1E-03	1.6E-02	3.198	0.362	2.788	3.475	4.324	0.179	4.143	4.501
ENSG00000250046		-1.3	1.6E-02	3.0E-02	10.792	2.099	8.454	12.515	14.391	0.700	13.969	15.199
ENSG00000250056	LINC01018	-1.3	1.2E-01	1.7E-01	0.900	0.105	0.808	1.015	1.186	0.219	1.047	1.439
ENSG00000250312	ZNF718	-1.3	3.4E-05	1.1E-04	13.746	1.904	11.957	15.747	17.667	0.823	16.722	18.231
ENSG00000250479	CHCHD10	-1.3	5.9E-05	1.8E-04	21.454	1.483	19.813	22.698	28.689	3.378	26.602	32.587
ENSG00000250848		-1.3	2.7E-02	4.6E-02	6.789	1.284	6.002	8.271	8.778	0.201	8.561	8.958
ENSG00000250899		-1.3	3.3E-08	1.9E-07	28.833	2.043	26.750	30.833	39.042	1.557	37.249	40.047
ENSG00000251102	CTBP2P4	-1.3	5.6E-04	1.4E-03	20.647	2.299	19.294	23.302	27.325	1.787	25.331	28.780
ENSG00000253368	TRNP1	-1.3	3.7E-05	1.2E-04	35.106	3.222	32.612	38.743	44.113	3.302	40.719	47.315
ENSG00000253846	PCDHGA10	-1.3	9.5E-04	2.3E-03	2.748	0.125	2.676	2.893	3.605	0.410	3.217	4.034
ENSG00000254602		-1.3	3.3E-02	5.5E-02	2.774	0.102	2.669	2.873	3.593	0.755	3.131	4.464
ENSG00000254701		-1.3	1.3E-01	1.8E-01	6.007	0.336	5.690	6.359	7.639	0.403	7.201	7.994
ENSG00000254858	MPV17L2	-1.3	2.8E-05	9.2E-05	22.411	0.690	21.977	23.207	29.388	2.405	27.153	31.935
ENSG00000255441		-1.3	3.9E-02	6.4E-02	5.049	0.222	4.915	5.305	6.697	0.874	5.699	7.327
ENSG00000256060		-1.3	1.0E-02	1.9E-02	7.245	0.735	6.442	7.883	9.221	0.844	8.565	10.174
ENSG00000256210		-1.3	1.2E-04	3.3E-04	188.762	5.176	183.658	194.008	240.637	21.753	217.398	260.512
ENSG00000256238	SUPT16HP1	-1.3	1.3E-04	3.7E-04	12.582	0.434	12.299	13.082	15.959	0.576	15.364	16.514
ENSG00000256616		-1.3	8.8E-03	1.7E-02	4.176	0.267	3.988	4.481	5.344	0.481	4.814	5.750
ENSG00000256737	RBBP4P5	-1.3	8.8E-02	1.3E-01	2.603	0.632	2.012	3.269	3.448	0.473	2.905	3.776
ENSG00000257027		-1.3	2.5E-02	4.3E-02	2.341	0.272	2.154	2.654	3.083	0.177	2.971	3.286
ENSG00000257307		-1.3	3.9E-02	6.4E-02	11.508	2.167	9.106	13.318	14.515	1.016	13.782	15.675
ENSG00000257446	ZNF878	-1.3	3.7E-02	6.0E-02	3.952	0.184	3.744	4.095	5.124	0.751	4.378	5.881
ENSG00000258016	HIGD1AP1	-1.3	3.3E-04	8.7E-04	82.837	6.001	77.174	89.128	110.357	13.665	94.625	119.284
ENSG00000258441	LINC00641	-1.3	5.6E-07	2.5E-06	11.241	1.078	10.083	12.216	15.137	0.527	14.687	15.716
ENSG00000258738		-1.3	8.2E-02	1.2E-01	2.289	0.469	1.823	2.760	2.959	0.699	2.254	3.653
ENSG00000259479	SORD2P	-1.3	1.7E-06	7.1E-06	19.992	0.738	19.542	20.844	26.117	0.172	25.948	26.292
ENSG00000260290		-1.3	4.1E-06	1.6E-05	53.762	0.683	53.340	54.551	67.929	1.671	66.207	69.544
ENSG00000260469	C15orf59-AS1	-1.3	1.7E-02	3.0E-02	5.186	0.451	4.704	5.597	6.825	0.672	6.061	7.320
ENSG00000260912		-1.3	1.4E-03	3.3E-03	9.174	0.696	8.508	9.897	12.044	0.883	11.173	12.938
ENSG00000260924	LINC01311	-1.3	2.2E-02	3.9E-02	6.415	1.124	5.117	7.077	8.167	0.121	8.076	8.304
ENSG00000260947		-1.3	2.4E-02	4.1E-02	2.960	0.411	2.537	3.359	3.739	0.147	3.587	3.880
ENSG00000261150	EPPK1	-1.3	9.2E-06	3.3E-05	3.101	0.326	2.836	3.465	4.186	0.331	3.815	4.448
ENSG00000261167		-1.3	2.6E-02	4.4E-02	2.293	0.613	1.733	2.948	3.056	0.450	2.552	3.419
ENSG00000261236	BOP1	-1.3	8.1E-09	5.3E-08	52.863	1.866	51.048	54.776	69.243	3.678	66.386	73.392
ENSG00000261613		-1.3	1.0E-01	1.5E-01	1.479	0.148	1.338	1.634	1.965	0.099	1.897	2.078
ENSG00000261620		-1.3	6.6E-02	1.0E-01	12.487	0.918	11.752	13.517	16.131	1.720	14.206	17.516
ENSG00000262580		-1.3	1.0E-01	1.5E-01	0.872	0.367	0.542	1.268	1.120	0.075	1.036	1.180
ENSG00000263013		-1.3	1.1E-01	1.6E-01	3.547	0.950	2.822	4.622	4.514	0.199	4.339	4.731
ENSG00000263874	LINC00672	-1.3	4.6E-02	7.4E-02	1.192	0.265	1.003	1.495	1.591	0.149	1.438	1.736
ENSG00000265354	TIMM23	-1.3	1.7E-09	1.2E-08	162.037	12.101	148.704	172.323	216.971	14.216	200.559	225.466
ENSG00000265579		-1.3	2.3E-02	4.1E-02	3.395	0.824	2.836	4.341	4.513	0.180	4.327	4.686
ENSG00000266066		-1.3	9.3E-04	2.2E-03	7.754	0.523	7.152	8.100	9.845	0.985	8.912	10.874
ENSG00000266884	POLR3GP2	-1.3	5.7E-02	8.9E-02	7.622	0.704	7.047	8.408	10.079	1.327	9.165	11.601
ENSG00000267796	LIN37	-1.3	1.3E-01	1.8E-01	1.432	0.176	1.261	1.612	1.825	0.331	1.451	2.080
ENSG00000268295	POLR3GP1	-1.3	6.0E-02	9.3E-02	22.256	1.751	20.994	24.255	28.137	0.137	28.007	28.280
ENSG00000269028	MTRNR2L12	-1.3	3.7E-02	6.0E-02	5.996	0.468	5.529	6.466	7.751	0.690	7.054	8.390
ENSG00000269113	TRABD2B	-1.3	2.9E-02	4.9E-02	0.986	0.116	0.854	1.071	1.295	0.129	1.179	1.435
ENSG00000270157		-1.3	1.8E-02	3.2E-02	7.430	1.182	6.508	8.762	9.807	0.895	8.910	10.639
ENSG00000270503	YTHDF2P1	-1.3	1.9E-02	3.4E-02	6.340	0.873	5.798	7.347	8.446	0.843	7.748	9.384
ENSG00000270977		-1.3	8.0E-02	1.2E-01	0.589	0.146	0.430	0.717	0.800	0.082	0.729	0.889
ENSG00000271133		-1.3	5.9E-02	9.2E-02	5.064	0.934	4.167	6.030	6.714	0.683	5.924	7.218
ENSG00000271836		-1.3	1.3E-01	1.8E-01	4.157	0.842	3.622	5.127	5.266	0.572	4.923	5.925
ENSG00000272655	POLR2J4	-1.3	1.2E-01	1.7E-01	3.317	1.034	2.495	4.477	4.187	0.210	4.013	4.420
ENSG00000272841		-1.3	1.1E-01	1.6E-01	2.370	0.337	2.004	2.668	3.069	0.561	2.424	3.449
ENSG00000273024	INTS6P2	-1.3	1.4E-01	2.0E-01	1.598	0.192	1.377	1.732	2.123	0.144	1.957	2.215
ENSG00000274605		-1.3	1.3E-03	3.1E-03	22.138	3.801	18.134	25.697	29.259	1.066	28.611	30.490
ENSG00000275215	RNA5-8SN	-1.3	1.2E-01	1.7E-01	75.908	27.419	48.929	103.747	96.176	16.456	78.677	111.340
ENSG00000275700	AATF	-1.3	8.7E-09	5.7E-08	29.943	1.764	27.929	31.218	38.986	0.438	38.671	39.485
ENSG00000275757	RNA5-8SN	-1.3	1.3E-01	1.9E-01	65.625	26.576	38.558	91.681	83.763	14.948	66.997	95.700
ENSG00000276700	RNA5-8SN	-1.3	1.2E-01	1.7E-01	64.826	26.041	38.028	90.037	82.941	14.342	66.722	93.947
ENSG00000278233	RNA5-8SN	-1.3	1.2E-01	1.7E-01	75.908	27.419	48.929	103.747	96.219	16.288	78.906	111.239
ENSG00000278291		-1.3	3.8E-02	6.2E-02	1.564	0.071	1.508	1.644	2.006	0.198	1.793	2.185
ENSG00000278864		-1.3	7.8E-02	1.2E-01	3.267	0.373	2.869	3.608	4.148	0.603	3.576	4.778
ENSG00000279393		-1.3	1.1E-01	1.6E-01	2.460	0.356	2.077	2.780	3.268	1.505	1.978	4.921
ENSG00000280383		-1.3	9.6E-02	1.4E-01	0.138	0.017	0.120	0.154	0.182	0.026	0.152	0.199
ENSG00000281398	SNHG4	-1.3	3.5E-07	1.6E-06	55.785	2.553	53.293	58.396	73.177	4.386	69.785	78.130
ENSG00000281406	BLACAT1	-1.3	4.2E-04	1.1E-03	7.958	0.575	7.424	8.566	10.345	0.424	10.020	10.824
ENSG00000282718		-1.3	9.8E-02	1.4E-01	7.762	1.155	6.687	8.983	10.172	1.909	8.628	12.307
ENSG00000283757		-1.3	1.4E-01	2.0E-01	0.600	0.117	0.466	0.684	0.802	0.070	0.757	0.883
ENSG00000000457	SCYL3	-1.2	4.7E-02	7.6E-02	2.992	0.166	2.870	3.181	3.487	0.286	3.248	3.804
ENSG00000001461	NIPAL3	-1.2	1.9E-03	4.2E-03	3.829	0.214	3.599	4.021	4.639	0.251	4.425	4.916
ENSG00000002822	MAD1L1	-1.2	1.0E-02	2.0E-02	8.110	0.277	7.792	8.299	9.409	0.789	8.791	10.298
ENSG00000003096	KLHL13	-1.2	7.5E-04	1.8E-03	7.301	0.352	6.927	7.626	8.789	0.273	8.516	9.062
ENSG00000003402	CFLAR	-1.2	3.6E-03	7.5E-03	1.699	0.096	1.611	1.801	2.023	0.116	1.947	2.156
ENSG00000004478	FKBP4	-1.2	2.1E-07	1.0E-06	170.879	3.358	167.004	172.930	203.965	3.857	199.757	207.330
ENSG00000004487	KDM1A	-1.2	4.1E-09	2.9E-08	188.874	3.343	185.137	191.580	227.881	7.718	218.978	232.681
ENSG00000005156	LIG3	-1.2	5.6E-08	3.1E-07	21.090	0.923	20.040	21.771	26.169	0.785	25.475	27.021
ENSG00000005189	REXO5	-1.2	2.1E-02	3.7E-02	3.481	0.358	3.156	3.864	4.296	0.285	3.973	4.508
ENSG00000005436	GCFC2	-1.2	2.3E-03	5.0E-03	10.783	0.550	10.168	11.227	12.577	0.250	12.374	12.857
ENSG00000006015	C19orf60	-1.2	1.7E-02	3.0E-02	20.411	1.520	19.374	22.156	24.065	1.268	22.964	25.452
ENSG00000006468	ETV1	-1.2	8.3E-06	3.0E-05	29.463	1.421	27.961	30.785	34.651	0.886	33.637	35.271
ENSG00000006625	GGCT	-1.2	4.5E-05	1.4E-04	92.729	5.804	88.010	99.210	110.442	3.148	107.051	113.273
ENSG00000006715	VPS41	-1.2	1.9E-07	9.7E-07	21.657	1.303	20.162	22.546	26.750	0.270	26.447	26.967
ENSG00000008056	SYN1	-1.2	2.4E-02	4.2E-02	4.299	0.301	4.092	4.644	5.289	0.356	4.971	5.673
ENSG00000008086	CDKL5	-1.2	6.4E-04	1.6E-03	5.156	0.094	5.048	5.221	6.008	0.388	5.614	6.389
ENSG00000008323	PLEKHG6	-1.2	5.2E-03	1.0E-02	8.661	0.615	7.977	9.167	10.371	1.129	9.509	11.650
ENSG00000009413	REV3L	-1.2	1.1E-02	2.0E-02	3.516	0.164	3.329	3.639	4.115	0.357	3.734	4.441
ENSG00000010244	ZNF207	-1.2	2.5E-08	1.5E-07	41.850	1.137	41.031	43.148	50.271	1.750	48.395	51.859
ENSG00000010539	ZNF200	-1.2	6.3E-03	1.3E-02	4.557	0.454	4.286	5.081	5.461	0.201	5.328	5.692
ENSG00000011028	MRC2	-1.2	4.8E-03	9.8E-03	9.956	0.949	9.037	10.932	11.753	0.741	10.985	12.463
ENSG00000011114	BTBD7	-1.2	1.2E-04	3.6E-04	12.924	0.608	12.557	13.625	15.124	0.644	14.589	15.839
ENSG00000011143	MKS1	-1.2	1.9E-02	3.4E-02	10.429	0.515	9.916	10.946	12.073	1.218	11.068	13.428
ENSG00000011451	WIZ	-1.2	7.5E-04	1.8E-03	13.585	0.233	13.352	13.817	16.460	1.529	14.732	17.634
ENSG00000011523	CEP68	-1.2	2.5E-04	6.7E-04	12.730	0.364	12.351	13.076	14.982	0.736	14.283	15.751
ENSG00000013016	EHD3	-1.2	2.6E-04	7.0E-04	10.992	0.376	10.679	11.409	13.563	0.834	12.883	14.493
ENSG00000013810	TACC3	-1.2	8.0E-06	2.9E-05	69.096	0.092	69.024	69.200	83.576	4.563	80.757	88.840
ENSG00000018510	AGPS	-1.2	1.4E-07	7.3E-07	31.784	0.982	30.834	32.795	38.978	2.329	36.379	40.876
ENSG00000018610	CXorf56	-1.2	4.2E-04	1.1E-03	25.028	1.736	23.027	26.123	30.154	0.531	29.545	30.516
ENSG00000021355	SERPINB1	-1.2	1.8E-02	3.3E-02	12.052	0.589	11.673	12.730	14.008	1.646	12.506	15.768
ENSG00000021574	SPAST	-1.2	1.6E-05	5.5E-05	25.269	1.003	24.365	26.348	31.060	1.517	29.323	32.130
ENSG00000021826	CPS1	-1.2	2.8E-03	6.1E-03	6.208	0.177	6.004	6.325	7.265	0.308	7.022	7.612
ENSG00000023228	NDUFS1	-1.2	1.7E-08	1.0E-07	27.510	0.656	26.767	28.006	33.801	1.061	32.579	34.489
ENSG00000023892	DEF6	-1.2	3.8E-02	6.3E-02	8.229	0.905	7.207	8.929	9.835	1.628	7.959	10.883
ENSG00000023902	PLEKHO1	-1.2	5.7E-04	1.4E-03	9.016	0.382	8.580	9.287	11.012	0.555	10.384	11.436
ENSG00000023909	GCLM	-1.2	1.6E-03	3.6E-03	11.831	0.529	11.228	12.219	14.189	0.718	13.636	15.000
ENSG00000026950	BTN3A1	-1.2	4.8E-02	7.7E-02	3.066	0.311	2.716	3.311	3.622	0.261	3.383	3.901
ENSG00000027001	MIPEP	-1.2	4.1E-03	8.6E-03	19.079	1.930	17.342	21.157	22.204	0.607	21.533	22.714
ENSG00000027075	PRKCH	-1.2	8.3E-02	1.2E-01	1.471	0.185	1.320	1.678	1.780	0.302	1.448	2.040
ENSG00000029153	ARNTL2	-1.2	1.2E-04	3.4E-04	10.441	0.389	10.079	10.853	12.903	0.444	12.400	13.243
ENSG00000029993	HMGB3	-1.2	5.9E-10	4.9E-09	217.954	5.842	211.213	221.547	267.388	0.777	266.755	268.255
ENSG00000030419	IKZF2	-1.2	1.3E-02	2.4E-02	1.694	0.136	1.564	1.835	2.106	0.335	1.727	2.362
ENSG00000031823	RANBP3	-1.2	1.5E-05	5.3E-05	21.290	0.416	21.018	21.768	25.419	1.718	23.453	26.631
ENSG00000032219	ARID4A	-1.2	4.6E-03	9.3E-03	8.087	0.321	7.743	8.379	9.429	0.571	8.883	10.023
ENSG00000032444	PNPLA6	-1.2	9.3E-04	2.2E-03	18.962	0.602	18.320	19.515	22.289	1.263	21.107	23.619
ENSG00000035141	FAM136A	-1.2	6.0E-06	2.3E-05	89.725	4.998	84.605	94.592	106.192	1.720	104.857	108.132
ENSG00000035681	NSMAF	-1.2	2.4E-05	8.1E-05	12.802	0.258	12.638	13.100	15.702	0.888	14.886	16.648
ENSG00000037474	NSUN2	-1.2	4.2E-07	1.9E-06	62.646	2.107	61.336	65.076	76.184	0.164	76.082	76.373
ENSG00000037749	MFAP3	-1.2	1.3E-04	3.8E-04	15.113	0.369	14.688	15.340	18.516	1.004	17.614	19.598
ENSG00000040199	PHLPP2	-1.2	4.1E-06	1.6E-05	6.281	0.287	5.980	6.550	7.868	0.184	7.743	8.079
ENSG00000040933	INPP4A	-1.2	1.1E-05	4.1E-05	9.351	0.103	9.269	9.467	11.125	0.249	10.940	11.408
ENSG00000042429	MED17	-1.2	5.1E-02	8.1E-02	0.940	0.148	0.818	1.104	1.140	0.149	1.051	1.312
ENSG00000043093	DCUN1D1	-1.2	1.6E-04	4.4E-04	20.606	1.833	18.523	21.974	24.512	1.866	22.881	26.547
ENSG00000044446	PHKA2	-1.2	2.0E-04	5.5E-04	10.885	0.183	10.709	11.074	12.793	0.719	12.346	13.623
ENSG00000046653	GPM6B	-1.2	4.8E-09	3.3E-08	99.551	2.479	97.287	102.199	121.959	3.486	118.028	124.673
ENSG00000047932	GOPC	-1.2	5.4E-05	1.7E-04	22.258	1.403	21.325	23.872	26.887	1.393	25.340	28.040
ENSG00000048140	TSPAN17	-1.2	5.0E-07	2.3E-06	30.555	0.871	29.828	31.521	38.284	1.368	37.306	39.824
ENSG00000048405	ZNF800	-1.2	4.4E-02	7.1E-02	2.617	0.352	2.298	2.994	3.268	0.441	2.912	3.762
ENSG00000048544	MRPS10	-1.2	3.5E-05	1.1E-04	55.764	2.159	54.465	58.256	66.672	2.910	63.851	69.663
ENSG00000049167	ERCC8	-1.2	7.2E-03	1.4E-02	12.694	1.277	11.719	14.139	14.804	1.310	13.757	16.272
ENSG00000049541	RFC2	-1.2	1.0E-06	4.4E-06	85.985	2.956	83.839	89.356	105.575	1.650	104.030	107.314
ENSG00000049656	CLPTM1L	-1.2	5.7E-06	2.1E-05	34.926	1.307	33.879	36.390	41.349	1.413	40.062	42.861
ENSG00000051825	MPHOSPH9	-1.2	2.0E-05	6.7E-05	8.976	0.712	8.179	9.546	11.135	0.494	10.565	11.421
ENSG00000052126	PLEKHA5	-1.2	1.9E-06	7.6E-06	19.925	0.440	19.518	20.392	23.603	0.226	23.348	23.779
ENSG00000054277	OPN3	-1.2	1.1E-03	2.6E-03	24.379	0.591	23.717	24.855	28.864	2.331	27.441	31.554
ENSG00000055332	EIF2AK2	-1.2	2.6E-05	8.6E-05	24.944	1.290	24.198	26.433	28.981	0.819	28.051	29.593
ENSG00000058056	USP13	-1.2	9.3E-07	4.1E-06	16.760	0.250	16.476	16.944	20.584	1.030	19.563	21.622
ENSG00000058600	POLR3E	-1.2	3.0E-06	1.2E-05	19.416	0.642	18.692	19.916	23.323	0.926	22.275	24.030
ENSG00000060237	WNK1	-1.2	3.9E-06	1.5E-05	42.852	0.166	42.715	43.037	49.437	1.279	48.050	50.569
ENSG00000060971	ACAA1	-1.2	1.7E-03	3.8E-03	7.532	0.943	6.495	8.338	9.058	0.611	8.355	9.461
ENSG00000062038	CDH3	-1.2	3.3E-06	1.3E-05	77.520	3.849	73.635	81.331	90.564	3.622	86.387	92.825
ENSG00000062485	CS	-1.2	8.4E-06	3.0E-05	56.735	1.351	55.290	57.967	65.790	1.254	64.498	67.002
ENSG00000062650	WAPL	-1.2	1.3E-05	4.7E-05	47.623	0.894	46.601	48.262	54.989	0.946	54.226	56.048
ENSG00000062725	APPBP2	-1.2	1.0E-04	3.0E-04	7.435	0.279	7.191	7.739	9.083	0.600	8.505	9.703
ENSG00000064012	CASP8	-1.2	1.6E-02	2.8E-02	1.805	0.167	1.614	1.918	2.265	0.261	1.977	2.485
ENSG00000064419	TNPO3	-1.2	1.3E-07	6.9E-07	111.716	0.861	110.840	112.560	132.390	2.491	129.626	134.459
ENSG00000064652	SNX24	-1.2	1.7E-03	3.9E-03	10.959	0.856	10.151	11.856	13.305	0.637	12.714	13.980
ENSG00000064999	ANKS1A	-1.2	1.2E-06	5.2E-06	25.672	0.893	24.643	26.251	31.270	0.709	30.630	32.031
ENSG00000065000	AP3D1	-1.2	2.9E-07	1.4E-06	53.827	1.292	52.419	54.956	64.852	4.014	60.286	67.824
ENSG00000065057	NTHL1	-1.2	5.5E-06	2.1E-05	38.398	1.506	37.031	40.012	47.983	0.847	47.138	48.831
ENSG00000065183	WDR3	-1.2	3.5E-05	1.1E-04	31.570	0.996	30.505	32.478	36.548	0.252	36.326	36.821
ENSG00000065457	ADAT1	-1.2	1.3E-03	3.0E-03	12.137	0.154	11.997	12.302	14.439	1.119	13.172	15.290
ENSG00000065613	SLK	-1.2	8.3E-05	2.5E-04	24.779	0.983	23.692	25.606	28.779	1.238	27.943	30.201
ENSG00000065809	FAM107B	-1.2	2.1E-06	8.5E-06	16.961	0.738	16.449	17.806	21.155	0.857	20.263	21.971
ENSG00000065989	PDE4A	-1.2	3.8E-02	6.2E-02	1.812	0.147	1.647	1.929	2.226	0.258	2.060	2.524
ENSG00000066056	TIE1	-1.2	4.1E-02	6.6E-02	1.878	0.046	1.827	1.916	2.267	0.123	2.129	2.363
ENSG00000066379	ZNRD1	-1.2	1.2E-02	2.2E-02	14.255	1.518	12.856	15.869	16.401	0.655	16.004	17.157
ENSG00000067334	DNTTIP2	-1.2	7.9E-06	2.9E-05	35.746	3.080	32.455	38.558	43.286	2.587	41.342	46.222
ENSG00000067606	PRKCZ	-1.2	7.2E-04	1.8E-03	8.843	0.310	8.570	9.180	10.370	0.150	10.219	10.518
ENSG00000069345	DNAJA2	-1.2	8.1E-09	5.4E-08	61.424	0.978	60.412	62.364	75.935	0.547	75.383	76.477
ENSG00000069849	ATP1B3	-1.2	1.1E-08	6.9E-08	190.333	7.081	184.452	198.193	235.749	4.575	231.156	240.306
ENSG00000070047	PHRF1	-1.2	1.7E-04	4.6E-04	26.389	1.375	24.802	27.185	30.770	0.658	30.097	31.413
ENSG00000070061	IKBKAP	-1.2	1.8E-06	7.5E-06	33.256	1.428	31.901	34.747	39.931	0.262	39.635	40.133
ENSG00000070081	NUCB2	-1.2	5.0E-03	1.0E-02	5.207	0.724	4.710	6.038	6.171	0.137	6.018	6.283
ENSG00000070501	POLB	-1.2	4.7E-05	1.5E-04	22.457	0.545	21.861	22.931	27.322	2.044	24.962	28.509
ENSG00000070961	ATP2B1	-1.2	4.8E-07	2.2E-06	17.438	0.293	17.167	17.749	21.580	1.371	20.326	23.043
ENSG00000071051	NCK2	-1.2	3.2E-04	8.5E-04	52.855	1.674	51.121	54.461	61.036	2.001	59.192	63.164
ENSG00000071994	PDCD2	-1.2	1.5E-05	5.1E-05	36.720	0.497	36.228	37.221	42.915	2.430	41.501	45.721
ENSG00000072133	RPS6KA6	-1.2	2.3E-05	7.5E-05	8.495	0.477	7.988	8.935	10.552	0.674	9.864	11.212
ENSG00000072135	PTPN18	-1.2	1.3E-03	3.0E-03	4.488	0.037	4.464	4.531	5.607	0.434	5.243	6.087
ENSG00000072756	TRNT1	-1.2	3.5E-06	1.4E-05	19.704	0.740	19.121	20.537	24.645	1.394	23.225	26.011
ENSG00000072803	FBXW11	-1.2	1.6E-04	4.4E-04	48.200	2.629	45.579	50.837	55.805	3.954	51.640	59.507
ENSG00000072864	NDE1	-1.2	8.9E-05	2.6E-04	10.382	0.814	9.441	10.866	12.855	0.111	12.728	12.925
ENSG00000073050	XRCC1	-1.2	8.2E-05	2.5E-04	21.851	2.016	19.545	23.276	26.841	1.720	25.240	28.660
ENSG00000073670	ADAM11	-1.2	1.4E-01	1.9E-01	1.380	0.503	0.841	1.838	1.716	0.163	1.562	1.887
ENSG00000073711	PPP2R3A	-1.2	1.5E-05	5.2E-05	9.813	0.562	9.335	10.432	12.123	0.218	11.966	12.371
ENSG00000074181	NOTCH3	-1.2	6.7E-06	2.5E-05	31.051	1.174	29.837	32.277	37.999	2.315	35.903	40.485
ENSG00000074219	TEAD2	-1.2	8.8E-08	4.7E-07	98.802	3.065	95.540	101.622	123.455	5.161	118.219	128.537
ENSG00000074582	BCS1L	-1.2	1.8E-03	4.1E-03	10.256	0.328	9.944	10.597	12.660	1.025	12.025	13.842
ENSG00000074696	HACD3	-1.2	3.0E-06	1.2E-05	96.908	2.476	94.963	99.695	112.063	1.938	110.481	114.225
ENSG00000074964	ARHGEF10L	-1.2	1.3E-03	3.1E-03	11.596	0.611	10.868	12.189	13.526	0.458	13.045	13.955
ENSG00000075213	SEMA3A	-1.2	2.0E-05	6.7E-05	18.795	0.526	18.239	19.285	22.360	0.488	21.798	22.669
ENSG00000075240	GRAMD4	-1.2	2.7E-04	7.3E-04	10.459	0.537	10.042	11.065	13.070	0.793	12.239	13.818
ENSG00000079134	THOC1	-1.2	6.4E-06	2.4E-05	20.685	1.123	19.489	21.717	24.762	0.556	24.301	25.379
ENSG00000079150	FKBP7	-1.2	3.2E-02	5.3E-02	4.196	0.125	4.062	4.310	5.156	0.171	4.959	5.261
ENSG00000079313	REXO1	-1.2	9.7E-04	2.3E-03	16.051	0.359	15.705	16.422	19.186	2.023	17.303	21.324
ENSG00000079335	CDC14A	-1.2	4.6E-03	9.4E-03	6.083	0.735	5.644	6.932	7.398	0.540	6.795	7.837
ENSG00000079337	RAPGEF3	-1.2	3.1E-02	5.1E-02	2.351	0.191	2.213	2.569	2.762	0.102	2.649	2.845
ENSG00000079387	SENP1	-1.2	1.3E-04	3.7E-04	18.698	0.640	18.070	19.348	22.025	0.839	21.079	22.678
ENSG00000079462	PAFAH1B3	-1.2	1.6E-06	6.8E-06	82.145	4.849	77.442	87.127	102.628	5.173	97.989	108.206
ENSG00000079691	CARMIL1	-1.2	7.6E-05	2.3E-04	23.767	1.129	22.475	24.566	27.716	0.798	26.795	28.209
ENSG00000081803	CADPS2	-1.2	1.7E-03	3.9E-03	15.654	0.665	15.165	16.410	18.638	1.101	17.778	19.879
ENSG00000082438	COBLL1	-1.2	5.0E-02	8.0E-02	1.051	0.051	1.011	1.109	1.225	0.111	1.097	1.294
ENSG00000082805	ERC1	-1.2	6.7E-05	2.1E-04	8.417	0.129	8.270	8.510	10.086	0.531	9.769	10.699
ENSG00000083093	PALB2	-1.2	9.1E-04	2.2E-03	20.084	2.032	17.818	21.746	23.456	0.600	22.780	23.924
ENSG00000083520	DIS3	-1.2	2.1E-05	7.2E-05	24.161	1.522	22.436	25.314	28.194	0.983	27.222	29.188
ENSG00000083857	FAT1	-1.2	1.5E-06	6.2E-06	39.608	1.631	37.822	41.019	46.072	0.824	45.221	46.867
ENSG00000084090	STARD7	-1.2	1.3E-08	8.5E-08	134.961	5.335	129.186	139.707	164.569	4.423	159.656	168.233
ENSG00000084112	SSH1	-1.2	6.2E-05	1.9E-04	6.661	0.354	6.254	6.886	8.045	0.501	7.543	8.546
ENSG00000084453	SLCO1A2	-1.2	9.5E-03	1.8E-02	1.420	0.091	1.345	1.522	1.769	0.148	1.598	1.855
ENSG00000084628	NKAIN1	-1.2	1.4E-04	3.9E-04	39.922	0.215	39.724	40.150	47.144	2.391	45.596	49.898
ENSG00000084774	CAD	-1.2	1.4E-06	6.0E-06	65.452	1.396	63.851	66.417	78.091	3.442	74.273	80.956
ENSG00000085274	MYNN	-1.2	6.0E-04	1.5E-03	13.730	0.159	13.549	13.846	16.099	0.732	15.254	16.562
ENSG00000085644	ZNF213	-1.2	5.5E-02	8.7E-02	2.463	0.224	2.207	2.620	2.936	0.176	2.793	3.132
ENSG00000085733	CTTN	-1.2	3.1E-06	1.2E-05	63.681	1.297	62.215	64.680	74.024	2.192	72.148	76.433
ENSG00000085788	DDHD2	-1.2	4.9E-04	1.2E-03	5.500	0.435	5.214	6.001	6.655	0.141	6.495	6.763
ENSG00000085831	TTC39A	-1.2	2.0E-02	3.5E-02	2.076	0.088	1.977	2.141	2.483	0.084	2.389	2.551
ENSG00000085840	ORC1	-1.2	3.8E-05	1.2E-04	59.791	0.636	59.091	60.331	70.223	1.043	69.072	71.105
ENSG00000085871	MGST2	-1.2	8.0E-02	1.2E-01	4.708	0.501	4.269	5.254	5.502	0.378	5.185	5.921
ENSG00000085872	CHERP	-1.2	9.8E-05	2.9E-04	26.596	0.941	25.518	27.244	31.621	2.576	29.044	34.197
ENSG00000085998	POMGNT1	-1.2	1.6E-03	3.6E-03	13.643	0.373	13.298	14.040	15.845	1.097	14.988	17.081
ENSG00000086062	B4GALT1	-1.2	1.2E-04	3.4E-04	17.612	0.792	16.786	18.364	20.972	0.529	20.379	21.396
ENSG00000087116	ADAMTS2	-1.2	3.0E-02	5.1E-02	1.601	0.200	1.433	1.823	1.985	0.352	1.756	2.391
ENSG00000087157	PGS1	-1.2	1.8E-03	4.1E-03	17.970	1.086	16.725	18.715	20.765	0.769	20.172	21.634
ENSG00000087263	OGFOD1	-1.2	5.2E-08	2.9E-07	38.076	1.087	37.116	39.257	46.693	1.003	45.916	47.825
ENSG00000087448	KLHL42	-1.2	6.3E-04	1.6E-03	16.515	0.531	16.038	17.088	19.083	1.177	18.058	20.368
ENSG00000087995	METTL2A	-1.2	3.5E-05	1.1E-04	25.310	1.539	23.877	26.937	30.602	0.649	30.048	31.316
ENSG00000088356	PDRG1	-1.2	1.6E-03	3.6E-03	24.614	0.738	23.871	25.347	29.382	1.600	27.724	30.917
ENSG00000088833	NSFL1C	-1.2	7.0E-07	3.1E-06	29.781	1.501	28.085	30.937	36.546	1.918	34.692	38.522
ENSG00000088886	DYNLL1	-1.2	2.2E-06	9.0E-06	152.182	6.407	145.952	158.752	182.510	3.328	180.318	186.340
ENSG00000089154	GCN1	-1.2	4.6E-06	1.8E-05	77.037	0.834	76.087	77.648	89.518	3.691	86.064	93.408
ENSG00000089195	TRMT6	-1.2	2.0E-04	5.4E-04	16.335	1.009	15.451	17.434	19.912	0.922	19.237	20.962
ENSG00000089234	BRAP	-1.2	3.7E-05	1.2E-04	17.909	1.610	16.107	19.207	22.189	0.443	21.679	22.477
ENSG00000089335	ZNF302	-1.2	3.4E-04	8.9E-04	16.384	0.976	15.572	17.467	19.544	1.081	18.557	20.700
ENSG00000089472	HEPH	-1.2	1.9E-05	6.6E-05	28.014	0.337	27.645	28.305	33.145	1.178	31.912	34.258
ENSG00000089639	GMIP	-1.2	2.7E-03	5.9E-03	10.480	1.220	9.368	11.786	12.787	1.123	11.728	13.964
ENSG00000089685	BIRC5	-1.2	5.8E-07	2.6E-06	95.618	2.673	92.650	97.835	113.420	3.608	109.334	116.167
ENSG00000089723	OTUB2	-1.2	8.2E-02	1.2E-01	2.880	0.326	2.505	3.092	3.414	0.418	3.075	3.880
ENSG00000090006	LTBP4	-1.2	3.5E-03	7.4E-03	9.733	1.211	8.575	10.991	11.477	0.962	10.613	12.513
ENSG00000090020	SLC9A1	-1.2	1.7E-03	3.9E-03	10.095	0.895	9.184	10.972	12.014	0.378	11.579	12.253
ENSG00000090273	NUDC	-1.2	2.7E-08	1.6E-07	126.060	6.272	122.308	133.300	156.082	7.399	148.614	163.410
ENSG00000090612	ZNF268	-1.2	7.0E-06	2.6E-05	7.584	0.618	6.932	8.161	9.471	0.485	9.097	10.019
ENSG00000090661	CERS4	-1.2	3.5E-05	1.1E-04	24.943	0.564	24.375	25.502	29.842	0.572	29.218	30.342
ENSG00000091157	WDR7	-1.2	5.9E-05	1.8E-04	3.824	0.152	3.649	3.914	4.768	0.451	4.349	5.065
ENSG00000091490	SEL1L3	-1.2	2.0E-06	8.1E-06	31.827	0.677	31.183	32.533	38.134	0.688	37.345	38.611
ENSG00000091732	ZC3HC1	-1.2	2.1E-05	7.0E-05	23.657	1.039	22.696	24.759	29.674	1.710	28.068	31.471
ENSG00000092094	OSGEP	-1.2	1.6E-04	4.6E-04	15.069	0.260	14.792	15.307	17.974	0.249	17.693	18.171
ENSG00000092096	SLC22A17	-1.2	3.8E-04	9.8E-04	14.136	0.870	13.594	15.139	16.971	0.765	16.130	17.627
ENSG00000092201	SUPT16H	-1.2	1.0E-09	8.2E-09	191.432	4.453	187.049	195.952	235.123	2.864	232.127	237.834
ENSG00000092531	SNAP23	-1.2	1.5E-07	7.5E-07	32.229	0.706	31.759	33.040	40.213	1.926	38.343	42.191
ENSG00000093000	NUP50	-1.2	2.1E-06	8.7E-06	44.147	3.258	40.505	46.786	52.736	1.035	51.793	53.843
ENSG00000095319	NUP188	-1.2	4.0E-06	1.5E-05	48.362	1.111	47.337	49.543	57.022	1.115	55.825	58.032
ENSG00000095485	CWF19L1	-1.2	6.3E-05	1.9E-04	21.097	1.286	19.816	22.387	25.762	1.088	24.672	26.848
ENSG00000095906	NUBP2	-1.2	4.8E-05	1.5E-04	19.889	2.020	17.820	21.855	24.541	1.647	22.679	25.806
ENSG00000096060	FKBP5	-1.2	7.5E-07	3.3E-06	22.892	0.734	22.146	23.613	27.353	0.371	26.957	27.693
ENSG00000096070	BRPF3	-1.2	5.1E-05	1.6E-04	15.226	0.785	14.685	16.126	18.435	0.531	17.828	18.815
ENSG00000096080	MRPS18A	-1.2	2.6E-04	7.0E-04	64.406	3.492	62.268	68.436	76.901	1.782	74.849	78.058
ENSG00000096401	CDC5L	-1.2	3.3E-05	1.1E-04	43.003	0.403	42.654	43.444	49.624	1.484	48.702	51.336
ENSG00000099308	MAST3	-1.2	2.8E-02	4.7E-02	2.903	0.149	2.756	3.054	3.491	0.255	3.197	3.651
ENSG00000099364	FBXL19	-1.2	9.4E-05	2.8E-04	43.827	1.501	42.108	44.881	52.037	0.979	51.047	53.005
ENSG00000099804	CDC34	-1.2	1.9E-05	6.5E-05	85.652	1.315	84.199	86.760	103.073	8.793	93.437	110.663
ENSG00000099810	MTAP	-1.2	4.4E-06	1.7E-05	21.044	0.941	19.958	21.624	25.180	1.086	24.335	26.419
ENSG00000099889	ARVCF	-1.2	6.7E-03	1.3E-02	13.763	1.311	12.961	15.276	15.913	0.504	15.332	16.230
ENSG00000099904	ZDHHC8	-1.2	1.4E-03	3.2E-03	8.179	0.473	7.671	8.606	10.117	1.070	8.897	10.900
ENSG00000099949	LZTR1	-1.2	8.3E-04	2.0E-03	6.741	0.190	6.624	6.960	8.109	0.684	7.666	8.897
ENSG00000099953	MMP11	-1.2	2.7E-02	4.6E-02	3.535	0.508	2.952	3.889	4.263	0.422	3.805	4.637
ENSG00000099995	SF3A1	-1.2	2.7E-06	1.1E-05	53.204	0.789	52.435	54.012	63.852	3.838	59.548	66.921
ENSG00000100084	HIRA	-1.2	2.8E-05	9.1E-05	14.120	0.364	13.858	14.537	17.578	0.543	17.026	18.112
ENSG00000100151	PICK1	-1.2	6.1E-03	1.2E-02	9.638	0.266	9.395	9.923	11.253	0.355	10.872	11.574
ENSG00000100154	TTC28	-1.2	1.4E-03	3.2E-03	5.102	0.335	4.760	5.430	6.128	0.023	6.104	6.148
ENSG00000100347	SAMM50	-1.2	7.5E-07	3.3E-06	27.285	0.203	27.090	27.495	33.265	1.512	31.867	34.870
ENSG00000100372	SLC25A17	-1.2	1.3E-05	4.4E-05	28.160	1.482	26.734	29.692	34.875	1.755	32.851	35.985
ENSG00000100403	ZC3H7B	-1.2	1.3E-05	4.6E-05	36.155	1.137	35.313	37.448	41.717	0.416	41.356	42.172
ENSG00000100485	SOS2	-1.2	6.4E-03	1.3E-02	7.242	0.510	6.947	7.831	8.388	0.179	8.196	8.551
ENSG00000100526	CDKN3	-1.2	1.8E-04	5.1E-04	41.836	1.406	40.226	42.826	50.602	2.012	49.073	52.881
ENSG00000100578	KIAA0586	-1.2	1.9E-04	5.3E-04	9.168	0.234	9.005	9.436	10.909	0.528	10.364	11.417
ENSG00000100591	AHSA1	-1.2	1.9E-07	9.3E-07	133.310	2.243	131.045	135.531	158.675	4.200	155.988	163.514
ENSG00000100629	CEP128	-1.2	8.8E-03	1.7E-02	3.723	0.376	3.315	4.056	4.420	0.255	4.161	4.671
ENSG00000100722	ZC3H14	-1.2	1.4E-06	6.0E-06	12.358	0.354	11.957	12.628	14.693	0.169	14.503	14.825
ENSG00000100804	PSMB5	-1.2	2.9E-06	1.1E-05	264.883	6.045	258.031	269.459	306.535	5.211	302.565	312.436
ENSG00000100811	YY1	-1.2	1.2E-07	6.4E-07	57.150	1.146	56.477	58.473	68.463	1.276	67.571	69.924
ENSG00000100890	KIAA0391	-1.2	2.2E-02	3.9E-02	3.238	0.239	2.962	3.381	3.818	0.049	3.761	3.848
ENSG00000100926	TM9SF1	-1.2	1.2E-01	1.7E-01	3.009	0.578	2.619	3.672	3.542	0.553	2.982	4.088
ENSG00000100991	TRPC4AP	-1.2	2.7E-06	1.1E-05	77.574	1.123	76.294	78.392	91.981	2.588	89.113	94.177
ENSG00000101098	RIMS4	-1.2	1.0E-05	3.7E-05	28.765	0.885	27.749	29.371	35.736	2.778	33.004	38.557
ENSG00000101146	RAE1	-1.2	2.7E-06	1.1E-05	18.180	0.755	17.372	18.869	22.427	0.770	21.543	22.951
ENSG00000101161	PRPF6	-1.2	9.3E-08	4.9E-07	80.186	1.512	78.594	81.603	98.298	2.185	96.552	100.749
ENSG00000101199	ARFGAP1	-1.2	4.9E-04	1.2E-03	21.920	1.374	20.633	23.367	25.539	1.165	24.194	26.214
ENSG00000101290	CDS2	-1.2	2.9E-05	9.5E-05	20.555	0.203	20.360	20.765	23.784	0.883	23.091	24.778
ENSG00000101294	HM13	-1.2	1.8E-05	6.1E-05	17.358	0.344	16.994	17.677	20.348	0.101	20.231	20.417
ENSG00000101298	SNPH	-1.2	3.1E-02	5.2E-02	2.204	0.303	1.855	2.398	2.749	0.050	2.716	2.807
ENSG00000101335	MYL9	-1.2	1.6E-04	4.6E-04	52.711	4.135	50.151	57.482	62.555	4.106	57.935	65.788
ENSG00000101343	CRNKL1	-1.2	1.4E-06	5.8E-06	28.862	0.818	27.936	29.483	35.380	0.904	34.337	35.939
ENSG00000101350	KIF3B	-1.2	6.8E-06	2.5E-05	18.208	0.447	17.779	18.671	22.114	0.627	21.401	22.576
ENSG00000101361	NOP56	-1.2	6.5E-09	4.4E-08	127.662	2.709	124.540	129.385	157.686	5.105	152.443	162.642
ENSG00000101417	PXMP4	-1.2	2.4E-02	4.2E-02	3.096	0.337	2.708	3.317	3.698	0.202	3.574	3.931
ENSG00000101447	FAM83D	-1.2	2.1E-05	7.0E-05	90.812	4.292	87.161	95.540	106.415	1.153	105.674	107.743
ENSG00000101474	APMAP	-1.2	9.8E-06	3.5E-05	74.887	3.531	71.370	78.432	88.549	1.809	86.466	89.718
ENSG00000101639	CEP192	-1.2	8.8E-07	3.8E-06	17.733	1.182	16.902	19.086	21.722	0.243	21.499	21.981
ENSG00000101680	LAMA1	-1.2	5.4E-06	2.0E-05	18.809	0.218	18.632	19.052	22.009	0.501	21.703	22.587
ENSG00000101773	RBBP8	-1.2	2.6E-05	8.6E-05	30.998	0.727	30.348	31.783	36.413	0.720	35.873	37.231
ENSG00000101849	TBL1X	-1.2	2.8E-05	9.2E-05	20.394	0.753	19.678	21.179	24.402	1.140	23.099	25.214
ENSG00000101868	POLA1	-1.2	4.0E-07	1.9E-06	48.727	1.238	47.990	50.156	58.628	1.757	56.732	60.200
ENSG00000101935	AMMECR1	-1.2	1.8E-06	7.4E-06	28.155	1.984	26.879	30.441	34.650	1.920	32.437	35.882
ENSG00000101986	ABCD1	-1.2	4.0E-02	6.5E-02	3.477	0.357	3.068	3.729	4.199	0.369	3.795	4.517
ENSG00000102054	RBBP7	-1.2	3.8E-09	2.7E-08	89.438	1.216	88.575	90.829	109.017	4.268	106.202	113.927
ENSG00000102181	CD99L2	-1.2	1.1E-02	2.1E-02	4.285	0.607	3.662	4.875	5.216	0.549	4.776	5.832
ENSG00000102221	JADE3	-1.2	2.7E-04	7.3E-04	23.745	0.860	22.752	24.278	27.504	0.808	26.594	28.138
ENSG00000102287	GABRE	-1.2	1.1E-02	2.0E-02	1.412	0.031	1.385	1.445	1.768	0.114	1.660	1.887
ENSG00000102390	PBDC1	-1.2	2.8E-04	7.4E-04	77.853	0.908	76.882	78.794	93.190	5.331	88.734	99.096
ENSG00000102393	GLA	-1.2	4.1E-04	1.1E-03	34.114	1.278	32.700	35.185	40.792	0.451	40.305	41.196
ENSG00000102445	RUBCNL	-1.2	4.0E-02	6.6E-02	1.925	0.236	1.694	2.166	2.380	0.129	2.234	2.479
ENSG00000102755	FLT1	-1.2	6.7E-06	2.5E-05	34.084	1.119	32.916	35.145	39.646	0.793	38.765	40.303
ENSG00000102763	VWA8	-1.2	1.4E-05	4.7E-05	13.906	0.366	13.589	14.306	16.938	0.977	16.282	18.061
ENSG00000102786	INTS6	-1.2	1.0E-06	4.4E-06	5.111	0.089	5.034	5.209	6.283	0.268	5.979	6.487
ENSG00000102858	MGRN1	-1.2	2.4E-04	6.6E-04	10.112	0.297	9.800	10.392	12.055	0.757	11.182	12.520
ENSG00000102974	CTCF	-1.2	2.9E-05	9.3E-05	62.364	2.156	59.895	63.870	72.203	1.813	70.345	73.967
ENSG00000102977	ACD	-1.2	7.6E-04	1.9E-03	14.617	1.025	13.792	15.764	17.908	1.454	16.773	19.547
ENSG00000103005	USB1	-1.2	8.1E-06	3.0E-05	18.786	0.436	18.459	19.282	22.419	0.464	21.910	22.818
ENSG00000103168	TAF1C	-1.2	3.0E-04	7.9E-04	15.853	0.866	15.332	16.852	18.707	0.958	17.834	19.732
ENSG00000103194	USP10	-1.2	2.0E-06	8.2E-06	86.424	2.194	84.472	88.798	100.338	1.416	99.226	101.932
ENSG00000103197	TSC2	-1.2	6.2E-03	1.2E-02	9.686	0.219	9.474	9.911	11.503	1.128	10.350	12.605
ENSG00000103249	CLCN7	-1.2	3.8E-04	9.8E-04	9.350	0.345	8.967	9.636	11.265	0.642	10.563	11.824
ENSG00000103274	NUBP1	-1.2	1.4E-02	2.5E-02	23.712	2.678	21.753	26.764	27.476	1.333	26.223	28.877
ENSG00000103423	DNAJA3	-1.2	8.3E-05	2.5E-04	22.958	0.579	22.300	23.392	26.736	0.562	26.086	27.064
ENSG00000103472	RRN3P2	-1.2	1.0E-01	1.5E-01	2.719	0.398	2.364	3.149	3.313	0.141	3.194	3.468
ENSG00000103496	STX4	-1.2	2.9E-03	6.2E-03	14.167	1.592	13.175	16.004	16.844	0.970	16.169	17.956
ENSG00000103549	RNF40	-1.2	1.1E-05	3.8E-05	24.045	0.801	23.466	24.958	28.772	1.532	27.668	30.521
ENSG00000103876	FAH	-1.2	2.3E-03	5.1E-03	6.964	0.274	6.711	7.255	8.243	0.127	8.129	8.379
ENSG00000104059	FAM189A1	-1.2	1.8E-02	3.3E-02	5.048	0.268	4.801	5.334	5.923	0.433	5.542	6.394
ENSG00000104131	EIF3J	-1.2	3.5E-06	1.4E-05	90.181	3.704	87.993	94.458	106.775	4.614	102.346	111.554
ENSG00000104218	CSPP1	-1.2	6.9E-03	1.4E-02	6.116	0.743	5.655	6.973	7.283	0.422	6.796	7.552
ENSG00000104221	BRF2	-1.2	9.0E-03	1.7E-02	9.568	1.766	7.822	11.352	11.834	1.366	10.869	13.397
ENSG00000104228	TRIM35	-1.2	1.9E-03	4.3E-03	14.527	0.931	13.848	15.588	17.036	0.800	16.361	17.920
ENSG00000104365	IKBKB	-1.2	1.1E-04	3.3E-04	5.659	0.013	5.644	5.668	6.898	0.091	6.794	6.962
ENSG00000104381	GDAP1	-1.2	1.9E-04	5.2E-04	30.100	1.006	29.043	31.045	35.252	0.554	34.871	35.887
ENSG00000104522	TSTA3	-1.2	1.1E-03	2.5E-03	25.140	1.511	23.395	26.037	29.812	1.627	28.269	31.512
ENSG00000104626	ERI1	-1.2	5.2E-06	2.0E-05	13.154	0.466	12.812	13.684	16.450	0.818	15.505	16.944
ENSG00000104635	SLC39A14	-1.2	3.2E-08	1.9E-07	129.594	2.572	126.919	132.048	161.332	2.046	159.659	163.613
ENSG00000104643	MTMR9	-1.2	2.9E-06	1.1E-05	21.085	0.543	20.557	21.641	25.177	0.484	24.704	25.672
ENSG00000104738	MCM4	-1.2	4.3E-07	2.0E-06	161.654	2.577	159.596	164.544	188.673	4.034	184.395	192.408
ENSG00000104853	CLPTM1	-1.2	1.1E-05	3.9E-05	25.494	1.015	24.399	26.403	31.255	2.932	28.638	34.423
ENSG00000104870	FCGRT	-1.2	2.5E-03	5.3E-03	8.360	0.312	8.013	8.618	10.153	0.394	9.705	10.444
ENSG00000104886	PLEKHJ1	-1.2	1.6E-06	6.7E-06	34.071	0.994	32.958	34.870	41.412	2.114	39.881	43.823
ENSG00000104973	MED25	-1.2	1.2E-04	3.4E-04	14.100	0.818	13.155	14.579	17.108	0.726	16.582	17.937
ENSG00000105058	FAM32A	-1.2	7.9E-06	2.9E-05	97.323	2.022	95.534	99.516	116.808	6.391	110.311	123.088
ENSG00000105131	EPHX3	-1.2	2.1E-02	3.7E-02	18.854	2.509	16.008	20.749	22.045	2.028	19.710	23.363
ENSG00000105171	POP4	-1.2	5.2E-04	1.3E-03	8.576	0.276	8.264	8.790	10.396	0.401	9.998	10.800
ENSG00000105229	PIAS4	-1.2	1.4E-04	4.0E-04	19.325	1.450	17.780	20.657	23.800	0.452	23.332	24.236
ENSG00000105287	PRKD2	-1.2	3.4E-06	1.3E-05	18.116	1.067	17.062	19.195	22.692	1.327	21.238	23.837
ENSG00000105518	TMEM205	-1.2	3.2E-03	6.7E-03	49.754	4.162	46.695	54.493	57.471	1.901	55.362	59.054
ENSG00000105552	BCAT2	-1.2	1.6E-03	3.6E-03	10.806	0.516	10.306	11.337	12.938	0.666	12.190	13.463
ENSG00000105556	MIER2	-1.2	3.4E-04	9.0E-04	7.115	0.125	6.972	7.200	8.547	0.242	8.287	8.766
ENSG00000105618	PRPF31	-1.2	2.5E-05	8.3E-05	67.533	1.774	66.405	69.577	79.420	3.851	75.253	82.848
ENSG00000105671	DDX49	-1.2	1.8E-03	4.1E-03	23.672	1.494	22.012	24.908	27.992	1.549	26.430	29.529
ENSG00000105699	LSR	-1.2	6.4E-05	1.9E-04	82.946	3.881	78.889	86.746	99.640	7.239	94.563	107.930
ENSG00000105705	SUGP1	-1.2	9.9E-06	3.5E-05	13.007	0.849	12.371	13.971	16.266	0.252	16.040	16.537
ENSG00000105737	GRIK5	-1.2	9.1E-06	3.3E-05	17.854	0.502	17.393	18.388	22.057	1.107	20.811	22.926
ENSG00000105793	GTPBP10	-1.2	4.4E-03	9.1E-03	5.949	0.476	5.576	6.485	6.978	0.570	6.324	7.369
ENSG00000105866	SP4	-1.2	4.5E-05	1.4E-04	15.766	0.818	15.154	16.696	19.348	1.185	18.418	20.682
ENSG00000105875	WDR91	-1.2	1.8E-03	4.0E-03	6.122	0.215	5.893	6.320	7.461	0.510	7.132	8.048
ENSG00000105926	MPP6	-1.2	1.0E-06	4.4E-06	27.807	0.716	27.100	28.531	33.525	1.479	32.623	35.231
ENSG00000106069	CHN2	-1.2	6.5E-02	1.0E-01	0.727	0.064	0.685	0.800	0.913	0.178	0.760	1.108
ENSG00000106078	COBL	-1.2	1.9E-06	7.8E-06	9.726	0.227	9.502	9.955	11.850	0.222	11.699	12.106
ENSG00000106100	NOD1	-1.2	1.7E-02	3.0E-02	2.313	0.175	2.127	2.475	2.824	0.184	2.680	3.031
ENSG00000106299	WASL	-1.2	5.1E-04	1.3E-03	44.715	1.998	42.748	46.744	51.727	2.299	49.072	53.099
ENSG00000106346	USP42	-1.2	3.5E-03	7.3E-03	8.911	0.544	8.565	9.538	10.448	0.582	9.781	10.856
ENSG00000106462	EZH2	-1.2	1.1E-05	4.0E-05	39.771	2.045	37.597	41.657	47.937	1.585	46.481	49.626
ENSG00000106524	ANKMY2	-1.2	4.9E-04	1.2E-03	15.069	0.490	14.503	15.359	18.809	2.025	17.424	21.133
ENSG00000106537	TSPAN13	-1.2	5.7E-06	2.1E-05	49.336	2.244	47.513	51.843	61.671	4.580	56.409	64.757
ENSG00000106692	FKTN	-1.2	3.8E-05	1.2E-04	10.067	0.104	9.949	10.145	12.213	0.470	11.774	12.708
ENSG00000106829	TLE4	-1.2	2.4E-05	8.0E-05	8.855	0.464	8.545	9.389	10.908	0.237	10.693	11.162
ENSG00000106868	SUSD1	-1.2	6.1E-02	9.5E-02	2.272	0.308	1.919	2.483	2.825	0.383	2.543	3.260
ENSG00000107104	KANK1	-1.2	1.1E-04	3.3E-04	8.094	0.332	7.712	8.311	9.654	0.480	9.232	10.192
ENSG00000107201	DDX58	-1.2	1.1E-03	2.7E-03	8.053	0.683	7.309	8.652	9.998	0.328	9.680	10.335
ENSG00000107262	BAG1	-1.2	7.6E-04	1.8E-03	11.772	0.755	10.911	12.324	14.078	0.392	13.627	14.344
ENSG00000107263	RAPGEF1	-1.2	1.7E-05	5.7E-05	35.673	0.663	34.907	36.063	43.014	1.629	41.136	44.052
ENSG00000107290	SETX	-1.2	1.5E-06	6.5E-06	28.315	0.315	27.971	28.590	34.119	0.834	33.365	35.014
ENSG00000107362	ABHD17B	-1.2	5.2E-05	1.6E-04	44.745	3.721	40.530	47.572	54.182	1.639	52.628	55.894
ENSG00000107672	NSMCE4A	-1.2	1.2E-06	5.1E-06	41.032	2.416	39.392	43.807	50.331	0.888	49.320	50.988
ENSG00000107862	GBF1	-1.2	3.4E-04	9.1E-04	19.442	1.096	18.798	20.708	22.560	0.975	21.581	23.531
ENSG00000108064	TFAM	-1.2	2.7E-06	1.1E-05	75.723	2.778	72.852	78.398	88.545	3.344	85.160	91.846
ENSG00000108349	CASC3	-1.2	8.6E-06	3.1E-05	46.169	0.642	45.776	46.909	54.097	2.767	51.299	56.832
ENSG00000108509	CAMTA2	-1.2	6.3E-02	9.8E-02	2.204	0.368	1.813	2.547	2.637	0.118	2.555	2.773
ENSG00000108528	SLC25A11	-1.2	5.4E-04	1.4E-03	41.519	2.186	39.267	43.631	48.039	2.269	45.638	50.148
ENSG00000108578	BLMH	-1.2	7.3E-07	3.2E-06	44.446	0.250	44.290	44.735	53.164	1.849	51.985	55.295
ENSG00000108591	DRG2	-1.2	2.5E-05	8.3E-05	8.887	0.217	8.643	9.060	10.968	0.129	10.893	11.117
ENSG00000108592	FTSJ3	-1.2	2.5E-05	8.3E-05	58.445	1.613	56.588	59.495	67.442	2.577	65.181	70.248
ENSG00000108639	SYNGR2	-1.2	1.8E-03	4.0E-03	17.114	1.522	15.356	17.996	20.252	1.357	18.893	21.606
ENSG00000108651	UTP6	-1.2	2.1E-05	7.1E-05	36.422	1.167	35.232	37.564	42.421	0.299	42.093	42.679
ENSG00000108671	PSMD11	-1.2	5.2E-07	2.4E-06	79.518	0.301	79.221	79.822	92.683	1.563	91.327	94.393
ENSG00000108826	MRPL27	-1.2	2.2E-05	7.5E-05	27.564	1.636	25.788	29.010	34.502	2.544	32.791	37.426
ENSG00000108830	RND2	-1.2	4.8E-03	9.7E-03	40.981	1.808	39.826	43.069	47.491	2.375	44.890	49.545
ENSG00000109066	TMEM104	-1.2	6.2E-03	1.2E-02	12.355	0.527	11.899	12.932	14.313	0.923	13.764	15.379
ENSG00000109111	SUPT6H	-1.2	1.4E-06	6.0E-06	27.413	0.429	27.033	27.879	32.690	0.979	31.891	33.782
ENSG00000109133	TMEM33	-1.2	1.9E-06	7.7E-06	29.685	1.799	28.150	31.664	35.902	2.124	33.611	37.804
ENSG00000109265	KIAA1211	-1.2	7.7E-06	2.8E-05	11.441	0.313	11.081	11.657	14.270	0.626	13.562	14.752
ENSG00000109320	NFKB1	-1.2	2.4E-02	4.2E-02	4.858	0.477	4.391	5.344	5.636	0.242	5.358	5.797
ENSG00000109472	CPE	-1.2	7.4E-03	1.4E-02	12.364	0.993	11.219	12.979	14.682	1.603	12.833	15.678
ENSG00000109686	SH3D19	-1.2	8.5E-09	5.6E-08	31.103	0.890	30.530	32.129	38.992	1.463	37.357	40.179
ENSG00000109917	ZPR1	-1.2	9.1E-05	2.7E-04	21.513	0.452	21.200	22.031	25.006	0.773	24.246	25.791
ENSG00000110171	TRIM3	-1.2	4.8E-02	7.7E-02	3.509	0.092	3.407	3.583	4.108	0.327	3.807	4.457
ENSG00000110367	DDX6	-1.2	1.5E-07	7.5E-07	51.008	0.399	50.556	51.310	61.511	1.669	59.679	62.948
ENSG00000110400	NECTIN1	-1.2	2.1E-06	8.6E-06	39.867	0.887	38.973	40.747	47.995	2.119	45.565	49.456
ENSG00000110442	COMMD9	-1.2	2.3E-03	5.0E-03	7.322	0.424	6.902	7.750	8.668	0.355	8.336	9.043
ENSG00000110844	PRPF40B	-1.2	2.2E-03	4.8E-03	4.117	0.410	3.775	4.572	4.940	0.060	4.881	5.002
ENSG00000110911	SLC11A2	-1.2	9.4E-05	2.8E-04	18.644	0.488	18.086	18.991	21.654	1.116	20.393	22.514
ENSG00000111087	GLI1	-1.2	1.1E-02	2.0E-02	5.110	0.408	4.865	5.581	6.269	0.401	5.806	6.513
ENSG00000111196	MAGOHB	-1.2	2.3E-03	5.1E-03	18.025	0.724	17.323	18.769	20.944	1.310	19.470	21.977
ENSG00000111249	CUX2	-1.2	4.9E-02	7.8E-02	2.453	0.026	2.435	2.482	2.859	0.304	2.666	3.209
ENSG00000111361	EIF2B1	-1.2	5.7E-05	1.8E-04	30.508	0.328	30.129	30.701	36.075	1.303	35.119	37.559
ENSG00000111371	SLC38A1	-1.2	2.6E-09	1.9E-08	141.963	6.322	134.868	147.000	174.826	5.600	169.231	180.430
ENSG00000111641	NOP2	-1.2	1.4E-05	5.0E-05	34.365	1.045	33.162	35.047	40.916	1.735	39.085	42.535
ENSG00000111679	PTPN6	-1.2	9.2E-04	2.2E-03	7.560	0.717	6.805	8.232	9.339	0.518	8.757	9.746
ENSG00000111707	SUDS3	-1.2	1.3E-08	8.4E-08	64.544	2.176	62.381	66.733	79.221	1.911	77.116	80.845
ENSG00000111725	PRKAB1	-1.2	5.3E-05	1.7E-04	11.588	0.649	10.848	12.065	14.155	0.603	13.732	14.846
ENSG00000111731	C2CD5	-1.2	1.4E-07	7.4E-07	39.533	1.202	38.184	40.488	47.850	0.956	46.887	48.799
ENSG00000111785	RIC8B	-1.2	6.0E-04	1.5E-03	9.155	0.411	8.850	9.623	10.846	0.435	10.549	11.345
ENSG00000111832	RWDD1	-1.2	5.5E-06	2.1E-05	22.419	0.227	22.246	22.676	27.011	0.516	26.470	27.496
ENSG00000111859	NEDD9	-1.2	6.8E-02	1.0E-01	2.554	0.060	2.507	2.622	2.979	0.092	2.899	3.080
ENSG00000111877	MCM9	-1.2	6.9E-04	1.7E-03	3.177	0.281	2.897	3.459	3.916	0.201	3.787	4.147
ENSG00000112110	MRPL18	-1.2	5.3E-05	1.6E-04	96.955	1.343	95.653	98.336	114.985	6.845	110.634	122.875
ENSG00000112118	MCM3	-1.2	2.2E-07	1.1E-06	337.140	1.960	335.172	339.092	393.768	2.466	391.640	396.471
ENSG00000112146	FBXO9	-1.2	1.0E-04	2.9E-04	8.880	0.825	8.291	9.823	10.990	0.249	10.773	11.263
ENSG00000112242	E2F3	-1.2	5.4E-08	3.0E-07	39.653	1.610	38.505	41.494	49.564	0.826	48.706	50.354
ENSG00000112294	ALDH5A1	-1.2	5.1E-02	8.2E-02	3.660	0.298	3.371	3.967	4.241	0.220	3.998	4.428
ENSG00000112297	CRYBG1	-1.2	1.6E-04	4.6E-04	8.257	0.484	7.698	8.540	10.016	0.117	9.907	10.139
ENSG00000112308	C6orf62	-1.2	3.5E-06	1.4E-05	97.409	2.741	95.171	100.466	114.027	4.488	108.852	116.854
ENSG00000112578	BYSL	-1.2	1.8E-03	4.1E-03	38.992	1.689	37.042	39.974	45.309	1.600	44.367	47.156
ENSG00000112812	PRSS16	-1.2	9.0E-03	1.7E-02	4.834	0.214	4.603	5.026	5.858	0.570	5.465	6.511
ENSG00000112851	ERBIN	-1.2	1.5E-06	6.5E-06	13.552	0.721	12.721	14.002	16.614	0.718	15.977	17.393
ENSG00000113273	ARSB	-1.2	2.2E-03	4.8E-03	6.183	0.392	5.733	6.448	7.501	0.890	6.737	8.479
ENSG00000113312	TTC1	-1.2	2.9E-06	1.2E-05	90.315	2.550	87.441	92.307	109.401	5.526	106.177	115.782
ENSG00000113356	POLR3G	-1.2	3.3E-02	5.5E-02	170.935	16.289	156.903	188.798	197.891	13.802	186.962	213.402
ENSG00000113360	DROSHA	-1.2	4.0E-06	1.5E-05	37.642	0.474	37.278	38.178	43.810	0.859	42.858	44.527
ENSG00000113575	PPP2CA	-1.2	1.1E-06	4.7E-06	91.249	2.202	89.346	93.661	106.389	0.942	105.513	107.384
ENSG00000113593	PPWD1	-1.2	3.3E-05	1.1E-04	32.416	1.573	30.690	33.768	38.623	1.689	37.331	40.534
ENSG00000113621	TXNDC15	-1.2	7.4E-04	1.8E-03	14.655	0.638	13.925	15.107	17.105	0.338	16.716	17.327
ENSG00000113643	RARS	-1.2	8.0E-09	5.3E-08	96.176	2.790	93.140	98.628	119.354	3.386	115.475	121.722
ENSG00000113645	WWC1	-1.2	2.1E-04	5.9E-04	12.070	0.360	11.840	12.485	14.435	0.405	13.988	14.778
ENSG00000113763	UNC5A	-1.2	1.4E-01	2.0E-01	2.594	0.424	2.348	3.083	3.043	0.667	2.501	3.788
ENSG00000114030	KPNA1	-1.2	1.2E-04	3.4E-04	21.878	1.198	20.628	23.016	25.428	0.631	25.037	26.156
ENSG00000114279	FGF12	-1.2	1.6E-02	2.8E-02	4.708	0.249	4.546	4.995	5.579	0.388	5.210	5.983
ENSG00000114302	PRKAR2A	-1.2	2.0E-05	6.8E-05	26.005	1.412	24.911	27.599	30.801	0.679	30.107	31.463
ENSG00000114316	USP4	-1.2	3.1E-06	1.2E-05	18.006	0.296	17.732	18.321	22.136	0.421	21.662	22.467
ENSG00000114383	TUSC2	-1.2	1.2E-04	3.4E-04	28.971	1.269	27.539	29.959	35.129	1.688	33.325	36.697
ENSG00000114423	CBLB	-1.2	1.6E-04	4.5E-04	7.063	0.102	6.948	7.143	8.401	0.399	7.947	8.692
ENSG00000114503	NCBP2	-1.2	5.4E-06	2.0E-05	57.008	0.225	56.802	57.247	66.367	1.382	65.228	67.905
ENSG00000114554	PLXNA1	-1.2	2.8E-05	9.1E-05	34.009	1.389	32.948	35.582	39.368	1.681	37.424	40.495
ENSG00000114648	KLHL18	-1.2	6.2E-04	1.5E-03	11.075	0.421	10.707	11.534	13.129	0.953	12.360	14.195
ENSG00000114859	CLCN2	-1.2	2.9E-03	6.3E-03	2.529	0.075	2.445	2.590	3.129	0.181	2.983	3.332
ENSG00000114867	EIF4G1	-1.2	2.8E-09	2.0E-08	177.940	6.025	173.996	184.876	216.109	9.398	209.135	226.797
ENSG00000114904	NEK4	-1.2	4.0E-05	1.3E-04	16.358	0.708	15.553	16.886	19.769	1.258	18.349	20.742
ENSG00000114923	SLC4A3	-1.2	7.6E-02	1.2E-01	3.493	0.470	3.001	3.938	4.102	0.741	3.622	4.955
ENSG00000114933	INO80D	-1.2	6.3E-04	1.6E-03	7.989	0.088	7.914	8.086	9.415	0.399	9.155	9.874
ENSG00000114948	ADAM23	-1.2	2.4E-04	6.6E-04	10.560	0.158	10.386	10.693	12.806	0.630	12.296	13.510
ENSG00000114993	RTKN	-1.2	3.0E-03	6.4E-03	12.879	0.912	11.896	13.698	14.906	0.712	14.184	15.607
ENSG00000115112	TFCP2L1	-1.2	2.9E-02	4.9E-02	3.528	0.420	3.063	3.880	4.070	0.153	3.965	4.245
ENSG00000115233	PSMD14	-1.2	1.8E-05	6.0E-05	56.527	0.839	55.582	57.184	65.444	2.209	63.540	67.866
ENSG00000115241	PPM1G	-1.2	3.8E-08	2.2E-07	160.705	5.382	154.915	165.555	191.249	3.379	189.140	195.146
ENSG00000115275	MOGS	-1.2	2.6E-04	7.0E-04	33.634	1.691	32.623	35.586	39.971	1.800	38.442	41.954
ENSG00000115317	HTRA2	-1.2	8.6E-04	2.1E-03	15.274	1.034	14.214	16.281	18.823	0.874	18.153	19.811
ENSG00000115365	LANCL1	-1.2	1.7E-08	1.1E-07	56.691	0.783	56.140	57.587	69.437	2.388	66.728	71.286
ENSG00000115504	EHBP1	-1.2	3.4E-06	1.3E-05	24.129	0.341	23.737	24.358	29.130	1.026	28.006	30.015
ENSG00000115526	CHST10	-1.2	1.9E-03	4.2E-03	15.446	0.371	15.020	15.700	18.035	0.760	17.171	18.599
ENSG00000115641	FHL2	-1.2	5.0E-03	1.0E-02	10.697	0.227	10.472	10.926	12.492	0.900	11.490	13.233
ENSG00000115649	CNPPD1	-1.2	2.2E-03	4.8E-03	23.901	1.123	23.040	25.171	28.025	1.409	27.077	29.644

ENSG00000115816	CEBPZ	-1.2	5.6E-09	3.8E-08	120.655	3.956	116.283	123.987	148.866	3.226	145.861	152.275
ENSG00000115875	SRSF7	-1.2	1.6E-02	2.8E-02	190.958	5.766	185.829	197.199	230.318	2.171	228.424	232.687
ENSG00000115942	ORC2	-1.2	5.5E-07	2.5E-06	43.102	1.869	41.109	44.815	52.193	1.301	50.712	53.153
ENSG00000116095	PLEKHA3	-1.2	1.1E-02	2.1E-02	2.669	0.154	2.493	2.779	3.118	0.123	3.022	3.257
ENSG00000116138	DNAJC16	-1.2	3.3E-03	6.9E-03	7.005	0.498	6.464	7.444	8.207	0.187	8.090	8.423
ENSG00000116171	SCP2	-1.2	1.2E-05	4.3E-05	16.481	0.269	16.225	16.761	19.997	1.388	19.112	21.597
ENSG00000116406	EDEM3	-1.2	9.7E-04	2.3E-03	9.634	0.304	9.443	9.984	11.255	0.448	10.781	11.671
ENSG00000116525	TRIM62	-1.2	1.9E-02	3.3E-02	6.330	0.315	6.099	6.689	7.668	0.372	7.279	8.021
ENSG00000116679	IVNS1ABP	-1.2	5.0E-09	3.4E-08	118.084	3.731	115.091	122.264	142.796	3.843	138.392	145.467
ENSG00000116698	SMG7	-1.2	5.8E-06	2.2E-05	30.991	1.716	29.452	32.841	36.900	1.775	35.702	38.940
ENSG00000116752	BCAS2	-1.2	2.8E-04	7.5E-04	104.463	8.187	99.154	113.892	127.890	9.495	121.760	138.828
ENSG00000116786	PLEKHM2	-1.2	8.9E-04	2.1E-03	35.543	1.068	34.529	36.659	40.994	2.658	37.989	43.039
ENSG00000117054	ACADM	-1.2	7.1E-06	2.6E-05	32.223	0.915	31.173	32.849	38.668	1.753	37.490	40.683
ENSG00000117155	SSX2IP	-1.2	4.1E-05	1.3E-04	14.674	0.734	14.222	15.521	17.809	1.011	16.653	18.530
ENSG00000117222	RBBP5	-1.2	3.3E-05	1.1E-04	22.295	0.444	21.783	22.568	27.082	1.570	25.274	28.103
ENSG00000117245	KIF17	-1.2	3.0E-02	5.1E-02	5.849	0.527	5.535	6.457	6.885	0.475	6.344	7.234
ENSG00000117298	ECE1	-1.2	1.1E-06	4.6E-06	19.947	0.185	19.818	20.159	24.683	1.958	23.554	26.953
ENSG00000117408	IPO13	-1.2	3.6E-04	9.5E-04	11.410	0.814	10.506	12.086	13.994	1.037	13.166	15.157
ENSG00000117419	ERI3	-1.2	1.3E-04	3.8E-04	55.754	1.369	54.188	56.727	65.705	1.282	64.345	66.891
ENSG00000117592	PRDX6	-1.2	1.8E-09	1.3E-08	382.355	6.613	375.080	388.003	463.388	2.923	460.936	466.622
ENSG00000117597	DIEXF	-1.2	3.3E-03	7.0E-03	6.422	0.092	6.345	6.523	7.407	0.156	7.228	7.520
ENSG00000117625	RCOR3	-1.2	8.4E-03	1.6E-02	4.867	0.221	4.685	5.114	5.638	0.349	5.279	5.977
ENSG00000117632	STMN1	-1.2	1.8E-09	1.3E-08	220.497	4.987	215.778	225.714	271.100	3.653	266.882	273.258
ENSG00000117676	RPS6KA1	-1.2	2.1E-06	8.7E-06	23.996	0.433	23.537	24.396	29.680	1.370	28.109	30.522
ENSG00000117713	ARID1A	-1.2	8.1E-08	4.4E-07	22.874	0.268	22.571	23.082	28.142	1.098	27.171	29.333
ENSG00000117751	PPP1R8	-1.2	3.7E-04	9.7E-04	56.032	1.154	54.889	57.272	64.826	3.481	62.042	68.729
ENSG00000117877	CD3EAP	-1.2	1.8E-04	5.0E-04	29.766	1.867	28.332	31.877	35.319	1.932	33.091	36.515
ENSG00000118420	UBE3D	-1.2	1.1E-03	2.6E-03	5.228	0.100	5.142	5.337	6.498	0.686	5.761	7.144
ENSG00000118640	VAMP8	-1.2	1.3E-02	2.3E-02	47.940	3.523	44.269	51.294	55.670	3.015	52.207	57.713
ENSG00000118900	UBN1	-1.2	1.6E-06	6.5E-06	16.922	0.326	16.686	17.293	20.825	0.421	20.469	21.289
ENSG00000119328	FAM206A	-1.2	1.4E-02	2.5E-02	13.073	0.415	12.648	13.478	15.401	0.834	14.485	16.118
ENSG00000119402	FBXW2	-1.2	7.3E-06	2.7E-05	24.474	0.508	23.968	24.983	28.630	1.348	27.628	30.163
ENSG00000119599	DCAF4	-1.2	6.7E-03	1.3E-02	6.188	0.227	6.013	6.444	7.515	0.750	6.858	8.332
ENSG00000119632	IFI27L2	-1.2	5.2E-02	8.2E-02	5.965	1.117	5.176	7.244	7.362	1.002	6.236	8.153
ENSG00000119698	PPP4R4	-1.2	3.1E-03	6.6E-03	8.386	0.091	8.322	8.490	9.895	0.309	9.540	10.110
ENSG00000119720	NRDE2	-1.2	5.9E-04	1.5E-03	2.064	0.134	1.973	2.218	2.556	0.075	2.469	2.601
ENSG00000119772	DNMT3A	-1.2	2.2E-07	1.1E-06	27.215	0.898	26.557	28.237	33.426	1.736	31.616	35.078
ENSG00000119777	TMEM214	-1.2	3.7E-06	1.4E-05	20.206	0.825	19.710	21.158	25.228	0.838	24.381	26.057
ENSG00000119865	CNRIP1	-1.2	5.8E-02	9.1E-02	2.800	0.457	2.284	3.157	3.416	0.436	2.913	3.690
ENSG00000119866	BCL11A	-1.2	2.5E-03	5.3E-03	4.935	0.403	4.477	5.234	5.950	0.268	5.640	6.106
ENSG00000119969	HELLS	-1.2	3.1E-05	1.0E-04	41.716	1.126	40.762	42.959	48.138	1.584	47.215	49.967
ENSG00000119977	TCTN3	-1.2	1.2E-04	3.5E-04	39.586	1.282	38.628	41.042	46.093	0.608	45.725	46.794
ENSG00000119986	AVPI1	-1.2	2.2E-02	3.8E-02	10.478	0.430	10.052	10.911	12.830	0.514	12.368	13.384
ENSG00000120162	MOB3B	-1.2	6.8E-05	2.1E-04	16.852	0.265	16.552	17.054	20.094	1.053	18.953	21.028
ENSG00000120438	TCP1	-1.2	3.5E-08	2.0E-07	190.162	3.716	187.216	194.337	225.340	2.707	222.512	227.907
ENSG00000120662	MTRF1	-1.2	1.9E-02	3.4E-02	7.886	0.564	7.438	8.519	9.352	1.228	7.952	10.249
ENSG00000120705	ETF1	-1.2	2.5E-10	2.2E-09	163.596	1.318	162.108	164.614	203.140	4.797	197.608	206.162
ENSG00000120709	FAM53C	-1.2	1.6E-07	8.3E-07	30.974	0.788	30.077	31.559	38.460	0.085	38.374	38.543
ENSG00000120802	TMPO	-1.2	1.8E-06	7.5E-06	97.416	4.913	91.751	100.514	113.705	4.368	110.083	118.555
ENSG00000121022	COPS5	-1.2	1.1E-05	3.9E-05	18.999	0.347	18.607	19.266	22.755	0.842	22.176	23.721
ENSG00000121236	TRIM6	-1.2	6.4E-04	1.6E-03	13.715	0.402	13.374	14.157	16.537	0.253	16.272	16.777
ENSG00000121316	PLBD1	-1.2	1.7E-03	3.9E-03	15.670	0.998	14.995	16.816	18.971	1.800	16.914	20.255
ENSG00000121542	SEC22A	-1.2	1.0E-01	1.5E-01	3.598	0.678	3.006	4.337	4.198	0.461	3.890	4.728
ENSG00000121570	DPPA4	-1.2	8.5E-10	6.8E-09	409.613	7.777	403.119	418.231	499.674	15.439	483.818	514.660
ENSG00000121578	B4GALT4	-1.2	5.4E-06	2.1E-05	14.750	0.272	14.471	15.014	18.161	0.556	17.730	18.788
ENSG00000121753	ADGRB2	-1.2	7.7E-03	1.5E-02	6.566	0.611	6.050	7.241	7.681	0.568	7.150	8.281
ENSG00000121892	PDS5A	-1.2	1.1E-06	4.6E-06	38.214	0.144	38.079	38.366	45.118	1.506	43.387	46.128
ENSG00000122085	MTERF4	-1.2	2.6E-03	5.7E-03	8.145	0.672	7.424	8.755	9.441	0.419	9.094	9.907
ENSG00000122126	OCRL	-1.2	6.7E-05	2.0E-04	24.705	0.545	24.118	25.195	29.031	1.084	28.281	30.274
ENSG00000122257	RBBP6	-1.2	5.5E-06	2.1E-05	30.078	1.915	28.613	32.245	35.289	0.302	34.995	35.599
ENSG00000122390	NAA60	-1.2	1.9E-03	4.3E-03	14.843	0.469	14.436	15.356	17.212	1.164	16.027	18.354
ENSG00000122482	ZNF644	-1.2	3.0E-06	1.2E-05	35.510	2.727	33.594	38.632	43.668	3.182	41.712	47.340
ENSG00000122490	PQLC1	-1.2	3.2E-03	6.8E-03	3.913	0.244	3.755	4.195	4.872	0.171	4.679	5.005
ENSG00000122512	PMS2	-1.2	1.2E-02	2.2E-02	11.926	0.423	11.506	12.351	13.854	1.375	12.895	15.428
ENSG00000122550	KLHL7	-1.2	1.1E-07	5.6E-07	50.469	0.830	49.803	51.399	62.362	2.640	59.323	64.091
ENSG00000122574	WIPF3	-1.2	4.1E-03	8.4E-03	5.608	0.212	5.421	5.838	6.739	0.472	6.219	7.140
ENSG00000122642	FKBP9	-1.2	1.6E-02	2.9E-02	4.819	0.136	4.675	4.945	5.728	0.492	5.161	6.023
ENSG00000122643	NT5C3A	-1.2	5.7E-04	1.4E-03	18.359	0.700	17.728	19.111	22.362	1.087	21.354	23.515
ENSG00000122986	HVCN1	-1.2	7.1E-02	1.1E-01	4.558	0.369	4.202	4.938	5.289	0.618	4.807	5.985
ENSG00000123104	ITPR2	-1.2	3.1E-04	8.3E-04	27.077	1.213	25.676	27.802	32.205	0.947	31.422	33.257
ENSG00000123154	WDR83	-1.2	1.5E-02	2.7E-02	8.561	0.938	7.561	9.423	10.403	0.654	9.648	10.813
ENSG00000123297	TSFM	-1.2	4.3E-04	1.1E-03	20.193	1.211	18.880	21.401	24.425	1.086	23.182	25.193
ENSG00000123374	CDK2	-1.2	1.9E-04	5.2E-04	34.281	0.663	33.598	34.921	40.442	0.673	40.004	41.217
ENSG00000123395	ATG101	-1.2	1.5E-04	4.2E-04	29.340	1.667	28.161	31.246	35.054	1.669	33.841	36.957
ENSG00000123444	KBTBD4	-1.2	2.8E-03	6.0E-03	12.239	0.684	11.601	12.961	14.642	0.972	13.580	15.486
ENSG00000123472	ATPAF1	-1.2	4.7E-05	1.5E-04	29.191	1.078	27.992	30.080	34.339	1.386	33.012	35.777
ENSG00000123473	STIL	-1.2	2.5E-05	8.4E-05	18.372	0.722	17.632	19.074	22.433	0.547	22.069	23.063
ENSG00000123545	NDUFAF4	-1.2	1.2E-03	2.8E-03	39.478	1.912	37.960	41.626	45.792	1.824	44.558	47.888
ENSG00000123600	METTL8	-1.2	1.1E-04	3.2E-04	13.044	0.575	12.391	13.472	16.092	0.199	15.878	16.272
ENSG00000123636	BAZ2B	-1.2	5.6E-04	1.4E-03	5.351	0.372	5.069	5.773	6.251	0.357	5.839	6.480
ENSG00000123737	EXOSC9	-1.2	1.6E-05	5.4E-05	41.915	1.140	40.692	42.949	49.148	0.785	48.362	49.931
ENSG00000124097	HMGB1P1	-1.2	1.2E-02	2.2E-02	29.527	1.816	27.763	31.392	36.215	1.015	35.045	36.855
ENSG00000124226	RNF114	-1.2	6.8E-06	2.5E-05	52.001	0.674	51.487	52.764	61.076	0.729	60.522	61.901
ENSG00000124422	USP22	-1.2	1.2E-06	5.3E-06	124.581	1.376	123.718	126.168	145.233	4.476	140.694	149.642
ENSG00000124493	GRM4	-1.2	3.9E-04	1.0E-03	4.755	0.205	4.615	4.990	5.627	0.264	5.330	5.839
ENSG00000124541	RRP36	-1.2	7.0E-06	2.6E-05	68.292	2.238	65.731	69.872	82.819	3.754	79.253	86.737
ENSG00000124688	MAD2L1BP	-1.2	4.1E-04	1.1E-03	29.463	0.326	29.097	29.720	36.178	0.988	35.033	36.868
ENSG00000125037	EMC3	-1.2	1.2E-03	2.9E-03	16.582	0.943	15.510	17.282	19.878	1.629	18.554	21.698
ENSG00000125149	C16orf70	-1.2	2.3E-04	6.2E-04	8.944	0.320	8.700	9.306	10.883	0.341	10.493	11.125
ENSG00000125166	GOT2	-1.2	1.3E-09	1.0E-08	148.537	4.496	144.044	153.037	183.885	4.733	178.468	187.220
ENSG00000125351	UPF3B	-1.2	2.5E-05	8.4E-05	34.276	1.301	32.964	35.566	41.177	1.861	39.472	43.162
ENSG00000125447	GGA3	-1.2	1.3E-04	3.6E-04	17.828	0.910	16.916	18.737	20.882	0.827	20.305	21.830
ENSG00000125482	TTF1	-1.2	5.3E-04	1.3E-03	25.596	1.301	24.335	26.933	30.270	0.760	29.589	31.090
ENSG00000125503	PPP1R12C	-1.2	7.4E-04	1.8E-03	12.245	0.310	11.944	12.564	14.145	0.559	13.813	14.791
ENSG00000125531	FNDC11	-1.2	1.2E-01	1.7E-01	2.002	0.297	1.661	2.197	2.412	0.127	2.327	2.558
ENSG00000125630	POLR1B	-1.2	2.2E-05	7.3E-05	37.004	1.489	35.444	38.410	43.337	0.985	42.399	44.362
ENSG00000125818	PSMF1	-1.2	2.2E-06	8.9E-06	26.112	1.798	24.064	27.309	32.462	2.625	30.122	35.301
ENSG00000125844	RRBP1	-1.2	1.4E-07	7.2E-07	62.313	2.118	59.882	63.765	75.254	4.320	72.005	80.157
ENSG00000125863	MKKS	-1.2	5.4E-05	1.7E-04	12.617	0.271	12.396	12.920	15.266	0.656	14.566	15.866
ENSG00000125898	FAM110A	-1.2	7.0E-02	1.1E-01	5.621	0.834	4.890	6.530	6.631	0.124	6.517	6.763
ENSG00000125901	MRPS26	-1.2	1.5E-05	5.1E-05	115.054	4.448	111.781	120.119	143.319	13.603	133.615	158.867
ENSG00000126107	HECTD3	-1.2	3.3E-04	8.6E-04	17.462	0.916	16.552	18.384	20.686	0.766	20.132	21.568
ENSG00000126215	XRCC3	-1.2	1.0E-03	2.4E-03	11.121	0.256	10.967	11.417	12.953	0.179	12.746	13.060
ENSG00000126461	SCAF1	-1.2	3.0E-03	6.4E-03	29.771	0.209	29.552	29.967	34.536	2.688	31.513	36.699
ENSG00000126746	ZNF384	-1.2	7.9E-04	1.9E-03	21.561	1.069	20.364	22.422	24.938	0.961	24.319	26.045
ENSG00000126767	ELK1	-1.2	2.8E-04	7.4E-04	25.346	0.274	25.031	25.525	29.961	1.863	28.753	32.107
ENSG00000126821	SGPP1	-1.2	8.5E-03	1.6E-02	10.138	0.813	9.321	10.947	12.020	0.815	11.419	12.948
ENSG00000127054	INTS11	-1.2	3.5E-07	1.6E-06	17.942	0.982	16.881	18.819	22.461	1.073	21.705	23.689
ENSG00000127080	IPPK	-1.2	1.3E-02	2.4E-02	7.734	0.583	7.081	8.202	8.999	0.530	8.545	9.582
ENSG00000127586	CHTF18	-1.2	1.9E-04	5.3E-04	14.310	1.322	12.888	15.502	17.240	1.101	16.398	18.486
ENSG00000127838	PNKD	-1.2	6.1E-04	1.5E-03	8.072	0.197	7.860	8.251	9.992	0.358	9.620	10.333
ENSG00000127946	HIP1	-1.2	2.3E-09	1.7E-08	49.245	0.587	48.825	49.916	61.622	2.341	59.055	63.640
ENSG00000127948	POR	-1.2	1.4E-04	4.0E-04	32.882	2.259	31.220	35.454	38.063	1.675	36.385	39.735
ENSG00000127989	MTERF1	-1.2	6.2E-03	1.2E-02	7.363	0.173	7.236	7.560	8.721	0.603	8.256	9.402
ENSG00000128052	KDR	-1.2	1.2E-03	2.8E-03	24.636	1.376	23.501	26.167	30.578	1.648	28.889	32.183
ENSG00000128274	A4GALT	-1.2	5.9E-02	9.2E-02	5.080	0.392	4.633	5.363	6.099	0.312	5.828	6.440
ENSG00000128463	EMC4	-1.2	2.1E-05	7.1E-05	33.575	0.100	33.482	33.681	39.679	1.290	38.624	41.117
ENSG00000128607	KLHDC10	-1.2	1.5E-03	3.4E-03	15.802	0.449	15.430	16.301	18.289	1.055	17.112	19.150
ENSG00000128708	HAT1	-1.2	3.0E-06	1.2E-05	42.028	2.798	40.233	45.253	49.863	0.514	49.303	50.315
ENSG00000128872	TMOD2	-1.2	2.4E-02	4.2E-02	2.233	0.098	2.120	2.295	2.660	0.282	2.356	2.912
ENSG00000129003	VPS13C	-1.2	9.5E-04	2.3E-03	4.916	0.397	4.462	5.196	5.695	0.067	5.619	5.744
ENSG00000129351	ILF3	-1.2	1.2E-08	7.7E-08	170.908	2.320	168.311	172.773	202.437	4.166	198.494	206.795
ENSG00000129460	NGDN	-1.2	2.6E-05	8.6E-05	20.997	0.450	20.662	21.508	25.354	1.277	23.880	26.113
ENSG00000129667	RHBDF2	-1.2	3.2E-03	6.7E-03	5.870	0.101	5.774	5.976	7.017	0.385	6.577	7.287
ENSG00000129925	TMEM8A	-1.2	1.8E-06	7.6E-06	41.631	1.057	40.917	42.845	51.249	2.258	49.568	53.815
ENSG00000129932	DOHH	-1.2	1.8E-03	4.1E-03	13.655	0.885	12.716	14.475	16.863	0.643	16.386	17.595
ENSG00000129933	MAU2	-1.2	2.0E-03	4.5E-03	7.646	0.505	7.064	7.963	8.931	0.327	8.561	9.176
ENSG00000129991	TNNI3	-1.2	2.0E-02	3.5E-02	8.331	1.328	6.851	9.417	10.036	0.122	9.922	10.164
ENSG00000130032	PRRG3	-1.2	2.0E-02	3.4E-02	3.066	0.211	2.831	3.240	3.663	0.264	3.468	3.964
ENSG00000130165	ELOF1	-1.2	1.1E-03	2.5E-03	24.592	1.244	23.650	26.002	28.470	1.283	27.717	29.951
ENSG00000130177	CDC16	-1.2	4.8E-09	3.3E-08	59.028	1.335	57.727	60.396	73.928	2.005	72.009	76.009
ENSG00000130226	DPP6	-1.2	5.4E-03	1.1E-02	1.388	0.189	1.247	1.603	1.736	0.085	1.677	1.833
ENSG00000130254	SAFB2	-1.2	6.8E-05	2.1E-04	27.461	0.931	26.506	28.367	32.535	1.683	31.024	34.365
ENSG00000130307	USHBP1	-1.2	3.6E-02	6.0E-02	3.519	0.293	3.242	3.827	4.143	0.211	3.900	4.265
ENSG00000130312	MRPL34	-1.2	3.5E-05	1.1E-04	55.530	2.158	53.464	57.769	67.158	1.867	66.043	69.313
ENSG00000130382	MLLT1	-1.2	3.1E-04	8.2E-04	27.187	1.105	26.344	28.437	31.747	2.138	29.676	33.947
ENSG00000130479	MAP1S	-1.2	3.2E-04	8.4E-04	20.396	1.423	18.958	21.804	24.352	2.011	22.456	26.461
ENSG00000130635	COL5A1	-1.2	2.3E-02	3.9E-02	3.712	0.190	3.504	3.876	4.293	0.142	4.170	4.448
ENSG00000130669	PAK4	-1.2	3.8E-03	8.0E-03	15.463	0.448	14.947	15.747	18.132	1.812	16.217	19.819
ENSG00000130706	ADRM1	-1.2	3.9E-07	1.8E-06	126.302	1.775	124.517	128.067	155.529	11.336	143.145	165.392
ENSG00000130713	EXOSC2	-1.2	9.1E-07	4.0E-06	37.701	0.417	37.326	38.150	46.518	2.299	44.575	49.056
ENSG00000130940	CASZ1	-1.2	3.6E-02	6.0E-02	1.536	0.157	1.375	1.690	1.822	0.112	1.732	1.947
ENSG00000131148	EMC8	-1.2	6.3E-05	1.9E-04	24.956	1.425	23.979	26.592	30.737	2.655	28.000	33.302
ENSG00000131381	RBSN	-1.2	1.4E-05	4.9E-05	15.723	0.674	14.946	16.138	18.802	0.288	18.482	19.040
ENSG00000131408	NR1H2	-1.2	3.6E-03	7.5E-03	16.043	0.721	15.397	16.821	18.818	1.687	16.888	20.117
ENSG00000131446	MGAT1	-1.2	3.5E-07	1.7E-06	16.064	0.319	15.698	16.280	19.723	1.023	18.752	20.791
ENSG00000131748	STARD3	-1.2	1.4E-03	3.3E-03	18.029	1.570	16.243	19.189	21.090	1.571	19.972	22.886
ENSG00000132024	CC2D1A	-1.2	3.1E-04	8.3E-04	11.085	1.292	9.632	12.105	13.793	0.684	13.341	14.580
ENSG00000132297	HHLA1	-1.2	3.9E-02	6.3E-02	3.651	0.293	3.468	3.989	4.240	0.197	4.015	4.383
ENSG00000132313	MRPL35	-1.2	8.4E-05	2.5E-04	36.814	0.385	36.436	37.207	42.922	1.372	42.027	44.502
ENSG00000132323	ILKAP	-1.2	6.4E-04	1.6E-03	9.676	0.634	8.961	10.173	11.865	0.029	11.833	11.887
ENSG00000132383	RPA1	-1.2	6.1E-06	2.3E-05	89.223	2.680	86.601	91.957	103.010	4.100	99.298	107.411
ENSG00000132405	TBC1D14	-1.2	2.3E-07	1.1E-06	37.512	0.649	36.906	38.198	45.401	1.411	44.138	46.924
ENSG00000132466	ANKRD17	-1.2	1.3E-09	9.8E-09	43.354	2.351	41.260	45.896	54.279	0.507	53.974	54.864
ENSG00000132475	H3F3B	-1.2	2.5E-08	1.5E-07	207.954	6.397	201.206	213.929	247.815	6.781	240.249	253.345
ENSG00000132549	VPS13B	-1.2	6.2E-05	1.9E-04	6.007	0.185	5.861	6.215	7.215	0.464	6.737	7.663
ENSG00000132661	NXT1	-1.2	3.9E-03	8.1E-03	25.949	1.986	23.930	27.901	31.459	1.041	30.357	32.425
ENSG00000132780	NASP	-1.2	3.0E-09	2.2E-08	321.197	8.777	313.094	330.520	389.382	7.323	383.314	397.516
ENSG00000132952	USPL1	-1.2	1.5E-04	4.2E-04	17.942	0.638	17.347	18.616	21.418	0.984	20.633	22.522
ENSG00000132953	XPO4	-1.2	2.4E-09	1.7E-08	32.100	0.887	31.325	33.069	40.153	1.147	38.831	40.879
ENSG00000132967	HMGB1P5	-1.2	2.2E-09	1.6E-08	849.351	10.827	838.391	860.040	1049.122	13.999	1038.787	1065.054
ENSG00000133056	PIK3C2B	-1.2	4.5E-04	1.2E-03	7.960	0.153	7.804	8.111	9.496	0.282	9.229	9.792
ENSG00000133131	MORC4	-1.2	4.5E-02	7.2E-02	3.083	0.052	3.033	3.136	3.631	0.110	3.507	3.715
ENSG00000133169	BEX1	-1.2	3.3E-07	1.6E-06	181.927	6.942	176.590	189.775	224.850	0.629	224.367	225.561
ENSG00000133275	CSNK1G2	-1.2	1.3E-06	5.4E-06	64.761	1.834	63.501	66.865	78.131	5.296	73.112	83.667
ENSG00000133313	CNDP2	-1.2	5.3E-08	2.9E-07	40.000	1.033	39.056	41.103	48.899	3.070	46.818	52.425
ENSG00000133597	ADCK2	-1.2	3.2E-02	5.4E-02	7.292	0.363	6.980	7.690	8.682	0.915	8.104	9.737
ENSG00000133619	KRBA1	-1.2	1.6E-02	2.8E-02	4.334	0.128	4.258	4.483	5.240	0.604	4.794	5.928
ENSG00000133665	DYDC2	-1.2	7.8E-02	1.2E-01	3.727	0.385	3.288	4.007	4.542	0.674	3.821	5.157
ENSG00000133739	LRRCC1	-1.2	4.8E-03	9.8E-03	10.904	1.485	9.234	12.071	13.045	0.906	12.019	13.734
ENSG00000133884	DPF2	-1.2	1.1E-04	3.2E-04	22.931	0.135	22.807	23.074	26.630	0.936	25.557	27.272
ENSG00000133895	MEN1	-1.2	1.1E-06	3.1E-06	25.015	0.469	24.683	25.551	29.640	0.985	28.940	30.767
ENSG00000133985	TTC9	-1.2	1.4E-06	5.8E-06	27.252	0.647	26.509	27.692	33.255	0.792	32.506	34.084
ENSG00000134057	CCNB1	-1.2	2.6E-05	8.6E-05	286.354	8.800	276.666	293.854	333.801	8.653	325.766	342.962
ENSG00000134278	SPIRE1	-1.2	4.4E-03	9.1E-03	9.227	0.572	8.747	9.861	10.699	0.588	10.083	11.278
ENSG00000134313	KIDINS220	-1.2	4.3E-08	2.4E-07	12.985	0.208	12.796	13.208	16.212	0.189	16.018	16.396
ENSG00000134375	TIMM17A	-1.2	1.3E-04	3.8E-04	46.562	1.142	45.255	47.360	54.666	1.088	53.748	55.867
ENSG00000134569	LRP4	-1.2	4.6E-04	1.2E-03	9.925	0.600	9.245	10.385	11.786	0.540	11.309	12.372
ENSG00000134697	GNL2	-1.2	2.9E-06	1.1E-05	59.639	1.529	57.892	60.736	69.625	1.109	68.397	70.553
ENSG00000134815	DHX34	-1.2	6.5E-03	1.3E-02	12.815	0.131	12.736	12.966	15.005	0.462	14.530	15.453
ENSG00000134954	ETS1	-1.2	1.3E-03	3.0E-03	27.344	0.674	26.578	27.845	33.541	1.564	31.835	34.907
ENSG00000135119	RNFT2	-1.2	9.1E-02	1.3E-01	3.416	0.254	3.156	3.664	3.938	0.030	3.919	3.973
ENSG00000135148	TRAFD1	-1.2	2.8E-04	7.6E-04	29.159	0.259	28.872	29.374	33.714	0.306	33.362	33.918
ENSG00000135249	RINT1	-1.2	1.1E-03	2.7E-03	23.741	1.375	22.602	25.269	27.881	0.875	26.937	28.664
ENSG00000135387	CAPRIN1	-1.2	1.7E-09	1.3E-08	190.782	4.693	186.058	195.445	229.500	4.391	225.834	234.367
ENSG00000135392	DNAJC14	-1.2	1.5E-03	3.5E-03	17.047	1.043	16.088	18.121	20.065	0.877	19.336	21.039
ENSG00000135447	PPP1R1A	-1.2	7.8E-02	1.2E-01	3.313	0.460	2.785	3.628	3.924	0.577	3.333	4.486
ENSG00000135457	TFCP2	-1.2	2.2E-06	8.8E-06	42.478	3.512	38.597	45.439	52.052	0.413	51.579	52.342
ENSG00000135829	DHX9	-1.2	4.8E-09	3.3E-08	249.835	8.320	241.231	257.838	299.981	6.613	294.805	307.431
ENSG00000135835	KIAA1614	-1.2	1.2E-01	1.7E-01	0.588	0.082	0.525	0.681	0.718	0.084	0.655	0.814
ENSG00000135845	PIGC	-1.2	1.5E-03	3.5E-03	9.249	0.369	8.823	9.470	11.049	0.219	10.805	11.229
ENSG00000135912	TTLL4	-1.2	2.2E-05	7.5E-05	20.069	0.431	19.622	20.482	24.211	1.994	22.466	26.385
ENSG00000135930	EIF4E2	-1.2	1.6E-05	5.6E-05	44.134	1.134	42.972	45.236	51.381	1.791	50.254	53.447
ENSG00000135945	REV1	-1.2	6.6E-04	1.6E-03	14.444	0.312	14.166	14.781	16.817	0.706	16.002	17.264
ENSG00000135953	MFSD9	-1.2	8.2E-03	1.6E-02	10.165	0.908	9.247	11.063	11.816	0.259	11.526	12.022
ENSG00000136014	USP44	-1.2	8.3E-10	6.7E-09	132.602	1.630	131.538	134.478	164.392	4.699	159.756	169.151
ENSG00000136021	SCYL2	-1.2	1.0E-05	3.7E-05	29.037	0.465	28.514	29.403	34.136	1.680	32.382	35.730
ENSG00000136045	PWP1	-1.2	5.7E-06	2.1E-05	86.486	1.188	85.171	87.483	101.236	2.791	98.455	104.038
ENSG00000136110	CNMD	-1.2	6.6E-06	2.5E-05	93.219	3.145	90.133	96.421	112.657	1.400	111.402	114.167
ENSG00000136144	RCBTB1	-1.2	4.3E-06	1.6E-05	36.689	1.778	34.689	38.092	43.778	1.232	42.978	45.197
ENSG00000136279	DBNL	-1.2	1.7E-05	6.0E-05	6.884	0.368	6.597	7.299	8.582	0.148	8.411	8.678
ENSG00000136295	TTYH3	-1.2	7.4E-04	1.8E-03	79.657	1.842	78.522	81.782	94.682	8.284	85.422	101.387
ENSG00000136379	ABHD17C	-1.2	1.3E-04	3.6E-04	17.394	0.439	17.131	17.901	21.686	0.941	20.622	22.407
ENSG00000136527	TRA2B	-1.2	1.0E-10	9.8E-10	71.286	1.321	70.138	72.730	88.695	1.301	87.215	89.657
ENSG00000136643	RPS6KC1	-1.2	6.7E-04	1.7E-03	18.534	0.481	18.128	19.065	21.490	0.048	21.461	21.545
ENSG00000136699	SMPD4	-1.2	9.6E-08	5.1E-07	37.171	1.153	36.479	38.501	46.471	2.654	44.094	49.334
ENSG00000136732	GYPC	-1.2	1.4E-04	4.0E-04	13.941	0.348	13.711	14.341	17.336	0.372	16.951	17.693
ENSG00000136816	TOR1B	-1.2	5.3E-04	1.3E-03	16.461	1.888	14.330	17.926	20.256	0.160	20.106	20.424
ENSG00000136827	TOR1A	-1.2	1.9E-05	6.5E-05	25.104	1.473	23.648	26.593	30.797	0.742	30.015	31.491
ENSG00000136848	DAB2IP	-1.2	1.7E-05	5.7E-05	17.468	0.192	17.249	17.606	21.231	1.599	19.421	22.457
ENSG00000136877	FPGS	-1.2	2.7E-04	7.3E-04	27.806	1.398	26.585	29.331	32.646	1.100	31.875	33.905
ENSG00000136891	TEX10	-1.2	1.3E-08	8.0E-08	50.117	2.031	47.795	51.560	61.929	1.478	60.359	63.294
ENSG00000136937	NCBP1	-1.2	1.0E-07	5.3E-07	59.440	1.506	58.529	61.178	71.839	1.205	70.448	72.581
ENSG00000137106	GRHPR	-1.2	1.6E-04	4.5E-04	19.739	0.414	19.421	20.207	23.208	1.335	21.670	24.079
ENSG00000137135	ARHGEF39	-1.2	6.0E-04	1.5E-03	10.144	0.127	10.045	10.288	12.006	0.710	11.591	12.826
ENSG00000137145	DENND4C	-1.2	1.2E-04	3.4E-04	10.442	0.193	10.227	10.599	12.377	0.308	12.054	12.667
ENSG00000137177	KIF13A	-1.2	6.3E-05	1.9E-04	12.275	0.163	12.153	12.460	14.393	0.504	13.976	14.953
ENSG00000137200	CMTR1	-1.2	7.5E-05	2.3E-04	27.411	0.375	26.992	27.715	31.834	0.254	31.544	32.016
ENSG00000137266	SLC22A23	-1.2	1.4E-04	4.0E-04	11.753	0.289	11.420	11.934	14.132	0.573	13.731	14.789
ENSG00000137274	BPHL	-1.2	5.6E-03	1.1E-02	9.986	0.707	9.380	10.762	11.665	0.897	10.634	12.259
ENSG00000137364	TPMT	-1.2	7.3E-06	2.7E-05	42.706	1.287	41.503	44.062	51.359	1.847	49.226	52.463
ENSG00000137492	THAP12	-1.2	1.6E-07	8.2E-07	19.727	0.469	19.193	20.073	24.697	0.933	23.627	25.342
ENSG00000137815	RTF1	-1.2	2.2E-08	1.3E-07	61.081	0.567	60.429	61.458	74.256	0.732	73.537	75.001
ENSG00000137821	LRRC49	-1.2	1.6E-02	2.9E-02	2.875	0.228	2.664	3.117	3.385	0.206	3.160	3.562
ENSG00000138035	PNPT1	-1.2	5.4E-07	2.5E-06	35.921	1.051	34.844	36.944	43.912	0.936	43.370	44.993
ENSG00000138050	THUMPD2	-1.2	2.2E-03	4.8E-03	18.730	0.701	18.130	19.501	22.023	1.153	20.831	23.132
ENSG00000138073	PREB	-1.2	1.2E-04	3.5E-04	38.342	0.631	37.617	38.758	45.156	1.628	43.325	46.442
ENSG00000138160	KIF11	-1.2	6.1E-05	1.9E-04	81.327	0.819	80.382	81.828	94.144	2.035	91.935	95.942
ENSG00000138246	DNAJC13	-1.2	9.6E-09	6.2E-08	24.081	0.956	23.018	24.869	30.147	0.574	29.548	30.693
ENSG00000138346	DNA2	-1.2	2.8E-06	1.1E-05	36.377	1.220	35.329	37.716	43.886	0.785	43.287	44.775
ENSG00000138442	WDR12	-1.2	3.4E-08	2.0E-07	31.270	0.791	30.526	32.101	38.963	0.855	38.130	39.838
ENSG00000138463	DIRC2	-1.2	6.7E-03	1.3E-02	8.923	0.684	8.370	9.687	10.607	0.172	10.466	10.799
ENSG00000138592	USP8	-1.2	5.7E-07	2.6E-06	10.554	0.329	10.253	10.906	12.711	0.024	12.691	12.738
ENSG00000138600	SPPL2A	-1.2	2.4E-04	6.5E-04	9.013	0.226	8.760	9.192	11.005	0.894	10.194	11.964
ENSG00000138646	HERC5	-1.2	1.1E-04	3.3E-04	12.536	0.561	11.904	12.976	15.419	0.571	14.764	15.812
ENSG00000138660	AP1AR	-1.2	7.8E-04	1.9E-03	28.830	2.540	25.904	30.469	33.935	2.547	31.161	36.169
ENSG00000138663	COPS4	-1.2	1.6E-03	3.5E-03	36.665	1.058	35.459	37.441	42.342	2.808	40.190	45.518
ENSG00000138798	EGF	-1.2	6.5E-02	1.0E-01	2.912	0.232	2.762	3.179	3.392	0.310	3.144	3.739
ENSG00000138867	GUCD1	-1.2	2.3E-04	6.3E-04	38.901	0.730	38.145	39.601	45.122	2.687	42.224	47.557
ENSG00000138942	RNF185	-1.2	1.8E-05	6.3E-05	25.789	0.425	25.499	26.276	31.645	0.715	30.839	32.202
ENSG00000139083	ETV6	-1.2	1.5E-03	3.4E-03	7.862	0.109	7.778	7.985	9.351	0.454	9.039	9.872
ENSG00000139182	CLSTN3	-1.2	9.8E-03	1.9E-02	8.514	0.312	8.223	8.843	9.927	0.577	9.319	10.468
ENSG00000139200	PIANP	-1.2	1.1E-01	1.6E-01	3.450	0.346	3.091	3.781	4.012	0.267	3.841	4.320
ENSG00000139324	TMTC3	-1.2	6.7E-03	1.3E-02	23.887	2.156	21.740	26.053	28.684	2.688	26.744	31.753
ENSG00000139372	TDG	-1.2	7.4E-07	3.3E-06	53.618	2.607	51.563	56.550	65.561	1.718	64.064	67.437
ENSG00000139517	LNX2	-1.2	2.3E-04	6.2E-04	16.970	1.382	15.471	18.192	20.335	0.228	20.115	20.571
ENSG00000139629	GALNT6	-1.2	5.9E-03	1.2E-02	3.450	0.273	3.232	3.756	4.190	0.117	4.083	4.316
ENSG00000139645	ANKRD52	-1.2	4.7E-06	1.8E-05	20.620	0.232	20.352	20.759	24.752	1.384	23.471	26.221
ENSG00000139800	ZIC5	-1.2	3.5E-04	9.2E-04	35.985	0.220	35.734	36.145	41.572	1.511	39.830	42.520
ENSG00000139990	DCAF5	-1.2	5.3E-05	1.7E-04	11.567	0.779	10.960	12.445	14.002	0.207	13.809	14.220
ENSG00000140022	STON2	-1.2	9.7E-03	1.8E-02	5.560	0.186	5.347	5.690	6.506	0.163	6.353	6.677
ENSG00000140265	ZSCAN29	-1.2	5.6E-07	2.5E-06	19.190	0.103	19.085	19.290	23.953	0.793	23.083	24.636
ENSG00000140332	TLE3	-1.2	9.1E-05	2.7E-04	6.835	0.218	6.604	7.037	8.297	0.371	8.052	8.724
ENSG00000140350	ANP32A	-1.2	4.2E-08	2.4E-07	98.928	3.268	95.926	102.409	119.837	2.094	117.419	121.076
ENSG00000140391	TSPAN3	-1.2	6.3E-06	2.3E-05	29.603	0.552	28.968	29.972	34.979	1.349	33.863	36.479
ENSG00000140455	USP3	-1.2	1.8E-03	4.0E-03	4.835	0.314	4.505	5.129	5.859	0.188	5.631	5.989
ENSG00000140471	LINS1	-1.2	5.3E-03	1.1E-02	3.212	0.115	3.084	3.304	3.950	0.404	3.483	4.196
ENSG00000140525	FANCI	-1.2	1.6E-07	8.0E-07	50.747	0.837	50.111	51.695	60.716	1.533	58.963	61.804
ENSG00000140564	FURIN	-1.2	7.5E-04	1.8E-03	21.919	0.659	21.172	22.418	25.851	2.573	23.518	28.611
ENSG00000140598	EFL1	-1.2	8.7E-06	3.2E-05	34.735	1.268	33.358	35.855	41.381	0.406	40.912	41.621
ENSG00000140694	PARN	-1.2	8.7E-08	4.6E-07	34.011	0.536	33.486	34.557	42.452	0.662	41.776	43.099
ENSG00000140848	CPNE2	-1.2	2.7E-04	7.1E-04	7.023	0.157	6.905	7.201	8.633	0.607	8.007	9.218
ENSG00000140937	CDH11	-1.2	1.4E-02	2.5E-02	1.314	0.172	1.116	1.430	1.610	0.135	1.457	1.713
ENSG00000140950	TLDC1	-1.2	8.2E-06	3.0E-05	9.873	0.241	9.672	10.140	12.363	0.495	11.848	12.836
ENSG00000140983	RHOT2	-1.2	4.6E-05	1.4E-04	35.333	0.506	35.038	35.917	41.108	2.155	38.850	43.144
ENSG00000140987	ZSCAN32	-1.2	1.4E-03	3.3E-03	16.047	0.060	15.977	16.086	18.787	0.475	18.240	19.097
ENSG00000140992	PDPK1	-1.2	7.4E-05	2.2E-04	9.008	0.480	8.475	9.409	10.859	0.416	10.407	11.226
ENSG00000141040	ZNF287	-1.2	8.4E-02	1.3E-01	3.908	0.904	3.141	4.904	4.807	0.425	4.359	5.204
ENSG00000141084	RANBP10	-1.2	1.8E-03	4.0E-03	9.313	0.497	8.744	9.663	11.065	0.285	10.829	11.382
ENSG00000141179	PCTP	-1.2	3.0E-03	6.5E-03	8.002	0.356	7.691	8.391	9.569	0.539	8.975	10.025
ENSG00000141424	SLC39A6	-1.2	2.0E-06	8.1E-06	55.731	1.524	54.004	56.885	66.474	1.411	65.441	68.082
ENSG00000141452	C18orf8	-1.2	3.8E-03	7.9E-03	9.936	0.813	9.041	10.630	11.952	0.156	11.784	12.092
ENSG00000141543	EIF4A3	-1.2	4.7E-06	1.8E-05	54.968	1.021	53.791	55.592	64.063	1.104	63.036	65.230
ENSG00000141551	CSNK1D	-1.2	1.5E-06	6.2E-06	21.010	0.479	20.498	21.446	24.886	0.483	24.405	25.370
ENSG00000141556	TBCD	-1.2	1.1E-06	4.6E-06	21.679	0.370	21.361	22.084	26.202	0.974	25.079	26.819
ENSG00000141564	RPTOR	-1.2	3.5E-05	1.1E-04	7.531	0.245	7.316	7.797	9.262	0.195	9.046	9.426
ENSG00000141696	P3H4	-1.2	5.4E-03	1.1E-02	14.202	0.628	13.606	14.857	16.440	0.965	15.463	17.392
ENSG00000141965	FEM1A	-1.2	1.4E-05	4.9E-05	10.553	0.208	10.346	10.762	12.892	0.547	12.271	13.300
ENSG00000142065	ZFP14	-1.2	2.6E-04	7.1E-04	6.777	0.240	6.530	7.010	8.287	0.364	7.885	8.595
ENSG00000142208	AKT1	-1.2	3.6E-06	1.4E-05	9.789	0.111	9.694	9.910	12.246	1.204	10.913	13.255
ENSG00000142273	CBLC	-1.2	6.5E-03	1.3E-02	14.545	1.584	13.055	16.209	17.934	1.070	16.720	18.740
ENSG00000142552	RCN3	-1.2	1.8E-03	4.0E-03	14.487	0.932	13.422	15.149	17.957	1.944	15.818	19.614
ENSG00000143061	IGSF3	-1.2	1.4E-07	7.2E-07	28.962	1.359	28.080	30.528	35.661	1.743	34.245	37.608
ENSG00000143093	STRIP1	-1.2	1.9E-05	6.6E-05	13.646	0.711	12.827	14.108	16.648	0.420	16.302	17.115
ENSG00000143106	PSMA5	-1.2	5.2E-07	2.4E-06	69.094	4.807	64.854	74.316	84.073	3.148	80.855	87.146
ENSG00000143157	POGK	-1.2	4.3E-05	1.4E-04	21.747	0.876	20.956	22.688	25.484	1.096	24.753	26.744
ENSG00000143183	TMCO1	-1.2	3.8E-06	1.5E-05	22.721	0.561	22.296	23.357	28.175	1.056	27.106	29.218
ENSG00000143294	PRCC	-1.2	3.1E-05	1.0E-04	32.206	0.268	31.899	32.397	38.491	0.307	38.288	38.844
ENSG00000143314	MRPL24	-1.2	1.1E-03	2.5E-03	26.578	1.026	25.473	27.501	31.973	0.786	31.105	32.635
ENSG00000143315	PIGM	-1.2	3.2E-03	6.8E-03	6.730	0.727	6.183	7.554	8.237	0.623	7.520	8.636
ENSG00000143363	PRUNE1	-1.2	2.3E-06	9.3E-06	34.961	0.649	34.212	35.347	43.194	1.800	41.136	44.475
ENSG00000143373	ZNF687	-1.2	1.1E-05	3.8E-05	18.366	0.605	17.674	18.791	22.781	0.466	22.244	23.074
ENSG00000143514	TP53BP2	-1.2	2.2E-06	8.9E-06	22.269	1.151	20.971	23.164	27.224	1.040	26.278	28.338
ENSG00000143515	ATP8B2	-1.2	1.7E-05	5.8E-05	26.620	0.921	26.026	27.682	31.517	0.643	30.870	32.155
ENSG00000143549	TPM3	-1.2	8.1E-11	8.0E-10	181.004	3.451	178.655	184.967	223.781	1.416	222.586	225.345
ENSG00000143793	C1orf35	-1.2	1.7E-02	3.1E-02	9.452	0.847	8.515	10.166	11.305	2.101	9.436	13.579
ENSG00000143799	PARP1	-1.2	6.4E-11	6.5E-10	275.666	3.466	271.677	277.947	342.985	9.625	333.503	352.746
ENSG00000143942	CHAC2	-1.2	2.3E-02	4.0E-02	23.549	3.315	19.746	25.833	27.699	1.917	25.543	29.213
ENSG00000143952	VPS54	-1.2	1.1E-04	3.2E-04	19.076	0.222	18.888	19.321	22.772	0.993	21.883	23.844
ENSG00000144040	SFXN5	-1.2	1.8E-05	6.2E-05	6.544	0.255	6.320	6.822	8.165	0.560	7.723	8.794
ENSG00000144161	ZC3H8	-1.2	3.1E-02	5.3E-02	4.343	0.198	4.132	4.524	5.055	0.558	4.516	5.630
ENSG00000144362	PHOSPHO2	-1.2	1.0E-01	1.5E-01	8.080	0.473	7.676	8.600	9.536	1.597	7.688	10.582
ENSG00000144401	METTL21A	-1.2	3.7E-05	1.2E-04	14.062	0.350	13.737	14.432	17.055	0.212	16.829	17.251
ENSG00000144535	DIS3L2	-1.2	6.4E-04	1.6E-03	3.807	0.268	3.499	3.987	4.644	0.252	4.353	4.793
ENSG00000144580	CNOT9	-1.2	2.4E-05	7.9E-05	42.520	1.224	41.352	43.793	49.473	0.749	48.755	50.250
ENSG00000144591	GMPPA	-1.2	7.9E-04	1.9E-03	10.371	0.650	9.920	11.116	12.580	0.524	12.103	13.145
ENSG00000144824	PHLDB2	-1.2	1.1E-03	2.6E-03	11.714	0.748	11.173	12.567	13.587	0.703	12.829	14.209
ENSG00000144827	ABHD10	-1.2	3.4E-05	1.1E-04	30.326	0.473	29.838	30.781	36.702	1.325	35.659	38.193
ENSG00000145022	TCTA	-1.2	8.2E-02	1.2E-01	5.811	0.588	5.141	6.237	6.720	0.398	6.306	7.099
ENSG00000145041	DCAF1	-1.2	7.5E-06	2.7E-05	27.716	1.035	26.795	28.836	32.635	0.157	32.533	32.816
ENSG00000145088	EAF2	-1.2	7.0E-02	1.1E-01	4.691	0.883	3.750	5.502	5.748	0.545	5.163	6.240
ENSG00000145191	EIF2B5	-1.2	1.5E-05	5.3E-05	23.557	0.236	23.365	23.821	27.969	0.474	27.487	28.435
ENSG00000145220	LYAR	-1.2	1.7E-04	4.7E-04	69.091	2.517	66.286	71.151	79.938	2.267	77.359	81.617
ENSG00000145375	SPATA5	-1.2	2.1E-04	5.7E-04	7.294	0.467	6.889	7.804	8.985	0.638	8.317	9.587
ENSG00000145414	NAF1	-1.2	1.1E-05	4.0E-05	16.695	0.749	15.923	17.418	20.562	0.372	20.177	20.918
ENSG00000145545	SRD5A1	-1.2	6.3E-04	1.6E-03	13.029	0.582	12.414	13.570	15.454	0.985	14.414	16.383
ENSG00000145569	FAM105A	-1.2	1.9E-03	4.3E-03	8.698	0.313	8.341	8.925	10.670	0.446	10.343	11.178
ENSG00000145725	PPIP5K2	-1.2	4.2E-08	2.4E-07	13.256	0.528	12.678	13.712	16.344	0.414	15.919	16.747
ENSG00000145736	GTF2H2	-1.2	8.5E-06	3.1E-05	9.984	0.070	9.905	10.039	12.508	0.455	12.041	12.950
ENSG00000146085	MUT	-1.2	8.1E-04	2.0E-03	20.326	0.646	19.597	20.826	23.850	0.882	22.988	24.741
ENSG00000146143	PRIM2	-1.2	8.4E-05	2.5E-04	28.310	0.987	27.222	29.146	33.338	0.289	33.070	33.644
ENSG00000146223	RPL7L1	-1.2	2.0E-06	8.2E-06	52.578	0.236	52.383	52.840	61.333	1.135	60.333	62.567
ENSG00000146232	NFKBIE	-1.2	5.6E-03	1.1E-02	10.491	0.943	9.434	11.243	12.869	0.779	12.232	13.737
ENSG00000146243	IRAK1BP1	-1.2	9.7E-04	2.3E-03	5.498	0.189	5.280	5.618	6.855	0.574	6.256	7.401
ENSG00000146535	GNA12	-1.2	3.6E-07	1.7E-06	50.536	0.690	49.742	50.996	61.271	3.621	58.249	65.285
ENSG00000146540	C7orf50	-1.2	3.4E-06	1.3E-05	21.021	0.900	20.292	22.028	26.190	0.840	25.651	27.157
ENSG00000146574	CCZ1B	-1.2	6.9E-05	2.1E-04	30.435	0.271	30.222	30.740	35.251	1.174	33.895	35.947
ENSG00000146670	CDCA5	-1.2	1.7E-07	8.8E-07	61.570	1.324	60.560	63.069	76.511	4.711	71.568	80.949
ENSG00000146676	PURB	-1.2	4.0E-04	1.0E-03	27.430	1.199	26.108	28.446	31.731	2.666	29.095	34.425
ENSG00000146802	TMEM168	-1.2	4.2E-05	1.3E-04	7.103	0.225	6.854	7.289	8.856	0.547	8.306	9.400
ENSG00000146872	TLK2	-1.2	5.5E-06	2.1E-05	10.309	0.594	9.658	10.821	12.857	0.475	12.310	13.157
ENSG00000146918	NCAPG2	-1.2	1.1E-06	4.6E-06	59.751	0.611	59.217	60.417	69.801	1.415	68.259	71.041
ENSG00000147065	MSN	-1.2	1.2E-07	6.5E-07	149.702	5.449	144.035	154.903	176.489	5.382	170.545	181.033
ENSG00000147099	HDAC8	-1.2	9.1E-03	1.7E-02	6.980	0.306	6.628	7.190	8.214	0.730	7.580	9.011
ENSG00000147526	TACC1	-1.2	7.6E-04	1.9E-03	3.278	0.386	2.840	3.566	4.058	0.202	3.836	4.229
ENSG00000147596	PRDM14	-1.2	3.2E-05	1.0E-04	30.336	1.217	29.126	31.560	37.754	0.524	37.349	38.347
ENSG00000147649	MTDH	-1.2	3.9E-07	1.8E-06	35.022	0.655	34.439	35.730	42.561	0.831	41.905	43.495
ENSG00000147813	NAPRT	-1.2	2.3E-03	5.0E-03	9.385	1.478	8.312	11.071	11.591	0.612	10.934	12.146
ENSG00000147912	FBXO10	-1.2	5.4E-02	8.6E-02	2.256	0.177	2.057	2.395	2.707	0.394	2.259	3.001
ENSG00000148019	CEP78	-1.2	1.2E-05	4.3E-05	12.673	0.820	11.731	13.223	15.211	0.996	14.231	16.222
ENSG00000148110	MFSD14B	-1.2	1.1E-06	4.6E-06	61.031	1.322	59.686	62.328	73.335	0.846	72.758	74.307
ENSG00000148296	SURF6	-1.2	2.3E-05	7.8E-05	31.190	0.433	30.696	31.505	37.170	1.872	35.042	38.561
ENSG00000148308	GTF3C5	-1.2	9.7E-06	3.5E-05	42.124	1.460	40.908	43.743	51.029	3.419	47.366	54.135
ENSG00000148337	CIZ1	-1.2	6.5E-07	2.9E-06	17.642	0.601	17.080	18.276	22.010	1.006	21.331	23.166
ENSG00000148358	GPR107	-1.2	2.8E-08	1.7E-07	27.515	0.417	27.073	27.903	33.612	0.746	32.751	34.075
ENSG00000148362	C9orf142	-1.2	7.0E-04	1.7E-03	26.965	2.216	24.817	29.244	33.283	1.069	32.086	34.145
ENSG00000148399	DPH7	-1.2	3.0E-04	8.0E-04	15.248	0.201	15.017	15.378	18.401	1.703	16.912	20.259
ENSG00000148450	MSRB2	-1.2	2.3E-02	4.0E-02	10.599	1.336	9.571	12.110	12.803	0.849	12.127	13.756
ENSG00000148680	HTR7	-1.2	4.3E-03	8.9E-03	33.576	2.289	31.261	35.839	41.303	2.210	39.863	43.848
ENSG00000148824	MTG1	-1.2	8.6E-02	1.3E-01	4.570	0.651	3.819	4.975	5.327	0.706	4.742	6.111
ENSG00000148840	PPRC1	-1.2	2.5E-05	8.2E-05	36.416	1.640	34.728	38.002	43.169	1.125	42.170	44.388
ENSG00000148950	IMMP1L	-1.2	1.8E-03	4.1E-03	10.871	0.465	10.384	11.309	13.326	0.985	12.757	14.463
ENSG00000148985	PGAP2	-1.2	1.8E-05	6.2E-05	20.962	0.485	20.435	21.391	26.046	1.587	25.099	27.878
ENSG00000149115	TNKS1BP1	-1.2	3.7E-08	2.2E-07	45.746	0.702	44.973	46.341	57.159	1.240	55.727	57.897
ENSG00000149150	SLC43A1	-1.2	1.3E-02	2.4E-02	13.405	0.469	12.882	13.787	15.452	1.202	14.475	16.794
ENSG00000149212	SESN3	-1.2	2.7E-05	9.0E-05	10.760	0.442	10.342	11.222	13.068	1.004	12.188	14.162
ENSG00000149257	SERPINH1	-1.2	1.0E-09	8.1E-09	249.206	4.354	246.419	254.223	309.846	11.109	298.815	321.786
ENSG00000149260	CAPN5	-1.2	2.6E-05	8.5E-05	11.874	0.463	11.365	12.267	14.801	0.168	14.684	14.994
ENSG00000149262	INTS4	-1.2	1.2E-04	3.5E-04	11.995	0.945	10.975	12.839	14.690	0.960	13.666	15.570
ENSG00000149313	AASDHPPT	-1.2	4.3E-05	1.4E-04	30.474	0.059	30.427	30.539	35.608	2.194	33.280	37.639
ENSG00000149346	SLX4IP	-1.2	2.3E-03	5.1E-03	3.975	0.283	3.658	4.201	4.898	0.135	4.755	5.024
ENSG00000149483	TMEM138	-1.2	4.9E-04	1.2E-03	6.341	0.366	6.048	6.751	7.800	0.543	7.174	8.128
ENSG00000149554	CHEK1	-1.2	2.8E-07	1.4E-06	46.076	1.352	45.072	47.612	55.376	1.842	54.187	57.498
ENSG00000149657	LSM14B	-1.2	1.5E-05	5.2E-05	53.674	1.691	51.724	54.713	62.940	2.791	59.990	65.539
ENSG00000149792	MRPL49	-1.2	1.0E-06	4.4E-06	43.352	1.995	41.265	45.241	53.636	1.272	52.403	54.943
ENSG00000149948	HMGA2	-1.2	1.6E-08	9.9E-08	46.220	1.646	45.124	48.113	56.073	2.534	53.295	58.259
ENSG00000150401	DCUN1D2	-1.2	2.5E-03	5.4E-03	12.003	0.729	11.354	12.792	14.280	1.059	13.275	15.385
ENSG00000150779	TIMM8B	-1.2	6.9E-06	2.6E-05	111.087	2.931	108.415	114.222	134.046	5.232	128.507	138.904
ENSG00000151131	C12orf45	-1.2	3.4E-05	1.1E-04	4.192	0.166	4.043	4.370	5.078	0.165	4.899	5.225
ENSG00000151233	GXYLT1	-1.2	1.5E-05	5.1E-05	14.181	1.187	12.888	15.221	17.357	0.309	17.116	17.478
ENSG00000151332	MBIP	-1.2	2.7E-04	7.2E-04	19.794	0.366	19.405	20.131	23.882	0.587	23.278	24.449
ENSG00000151500	THYN1	-1.2	3.2E-04	8.5E-04	70.413	3.133	68.252	74.006	82.312	5.606	76.469	87.646
ENSG00000151503	NCAPD3	-1.2	1.2E-07	6.1E-07	29.472	0.692	28.862	30.223	35.798	0.676	35.022	36.253
ENSG00000151552	QDPR	-1.2	1.6E-04	4.5E-04	23.922	1.367	22.358	24.886	29.402	3.458	25.938	32.854
ENSG00000151835	SACS	-1.2	2.0E-06	8.3E-06	21.011	0.974	19.991	21.931	26.308	1.127	25.030	27.157
ENSG00000151849	CENPJ	-1.2	4.0E-05	1.3E-04	19.900	0.328	19.546	20.192	24.105	0.155	24.010	24.283
ENSG00000152154	TMEM178A	-1.2	4.6E-02	7.4E-02	7.581	0.353	7.249	7.952	8.917	0.379	8.481	9.164
ENSG00000152219	ARL14EP	-1.2	5.3E-06	2.0E-05	16.923	0.658	16.212	17.510	20.984	0.506	20.535	21.533
ENSG00000152229	PSTPIP2	-1.2	7.5E-02	1.1E-01	3.221	0.522	2.660	3.693	3.797	0.215	3.617	4.035
ENSG00000152240	HAUS1	-1.2	2.1E-05	7.1E-05	88.999	1.693	87.045	90.043	105.262	6.735	100.461	112.961
ENSG00000152291	TGOLN2	-1.2	1.4E-06	6.0E-06	46.344	1.589	44.511	47.314	54.755	1.704	52.829	56.070
ENSG00000152457	DCLRE1C	-1.2	1.4E-01	1.9E-01	1.362	0.265	1.074	1.595	1.615	0.264	1.311	1.789
ENSG00000152642	GPD1L	-1.2	8.7E-04	2.1E-03	6.299	0.169	6.186	6.493	7.846	0.453	7.360	8.256
ENSG00000152767	FARP1	-1.2	2.3E-05	7.6E-05	6.147	0.137	6.050	6.304	7.240	0.370	6.888	7.633
ENSG00000152990	ADGRA3	-1.2	8.0E-08	4.3E-07	27.911	1.589	26.481	29.621	34.181	0.071	34.129	34.263
ENSG00000153048	CARHSP1	-1.2	5.7E-06	2.1E-05	72.005	2.567	69.041	73.549	84.694	2.090	82.334	86.311
ENSG00000153066	TXNDC11	-1.2	6.1E-02	9.5E-02	5.718	0.481	5.332	6.257	6.655	0.243	6.481	6.932
ENSG00000153094	BCL2L11	-1.2	4.9E-04	1.2E-03	7.887	0.907	7.090	8.874	9.581	0.201	9.401	9.798
ENSG00000153165	RGPD3	-1.2	1.6E-02	2.9E-02	3.902	0.193	3.702	4.086	4.628	0.121	4.550	4.767
ENSG00000153406	NMRAL1	-1.2	8.3E-04	2.0E-03	29.014	1.417	28.028	30.638	33.912	1.088	33.019	35.138
ENSG00000153560	UBP1	-1.2	2.8E-05	9.1E-05	22.305	0.749	21.460	22.887	26.081	0.517	25.511	26.521
ENSG00000154122	ANKH	-1.2	3.4E-02	5.7E-02	1.969	0.146	1.848	2.131	2.349	0.156	2.184	2.494
ENSG00000154174	TOMM70	-1.2	2.9E-07	1.4E-06	65.525	1.525	63.765	66.461	78.353	1.888	76.366	80.122
ENSG00000154642	C21orf91	-1.2	1.7E-04	4.6E-04	19.893	1.841	17.898	21.528	23.763	1.062	22.865	24.936
ENSG00000154814	OXNAD1	-1.2	8.1E-04	2.0E-03	8.800	0.397	8.352	9.108	10.563	0.080	10.474	10.627
ENSG00000154832	CXXC1	-1.2	9.0E-05	2.7E-04	19.474	0.627	18.831	20.084	24.121	0.452	23.610	24.469
ENSG00000154889	MPPE1	-1.2	1.8E-03	4.0E-03	3.216	0.143	3.055	3.326	4.020	0.159	3.878	4.192
ENSG00000154945	ANKRD40	-1.2	2.4E-06	9.6E-06	38.392	1.679	36.454	39.410	46.599	0.417	46.301	47.076
ENSG00000155093	PTPRN2	-1.2	1.3E-01	1.8E-01	2.127	0.317	1.883	2.485	2.565	0.226	2.341	2.793
ENSG00000155380	SLC16A1	-1.2	4.1E-10	3.5E-09	326.769	8.557	321.797	336.650	401.305	13.527	385.752	410.334
ENSG00000155506	LARP1	-1.2	2.5E-08	1.5E-07	148.557	3.637	146.170	152.743	178.843	6.275	171.847	183.973
ENSG00000155542	SETD9	-1.2	8.1E-02	1.2E-01	8.090	0.193	7.867	8.203	9.358	1.150	8.505	10.666
ENSG00000155660	PDIA4	-1.2	5.2E-10	4.4E-09	238.737	3.467	234.935	241.722	298.789	5.257	293.841	304.308
ENSG00000155755	TMEM237	-1.2	1.1E-05	3.8E-05	15.353	0.305	15.007	15.582	18.574	0.393	18.189	18.974
ENSG00000155850	SLC26A2	-1.2	3.1E-05	1.0E-04	11.985	0.155	11.807	12.088	14.599	1.089	13.809	15.842
ENSG00000156170	NDUFAF6	-1.2	9.9E-04	2.4E-03	5.190	0.383	4.764	5.505	6.470	0.461	6.171	7.001
ENSG00000156381	ANKRD9	-1.2	2.4E-02	4.2E-02	8.811	1.220	7.573	10.011	10.743	0.920	9.680	11.302
ENSG00000156642	NPTN	-1.2	4.7E-06	1.8E-05	50.691	3.399	46.941	53.567	61.208	0.999	60.064	61.910
ENSG00000156687	UNC5D	-1.2	5.8E-05	1.8E-04	33.125	0.708	32.322	33.661	38.662	1.664	36.889	40.191
ENSG00000156876	SASS6	-1.2	1.3E-03	3.0E-03	16.176	0.727	15.466	16.918	19.121	1.621	17.592	20.820
ENSG00000156983	BRPF1	-1.2	1.0E-04	3.0E-04	9.659	0.888	8.921	10.644	12.111	0.331	11.859	12.486
ENSG00000157064	NMNAT2	-1.2	8.5E-05	2.5E-04	9.021	0.240	8.746	9.188	11.187	0.992	10.352	12.283
ENSG00000157349	DDX19B	-1.2	2.6E-03	5.5E-03	14.502	0.698	13.712	15.036	17.002	1.338	15.740	18.405
ENSG00000157368	IL34	-1.2	8.1E-02	1.2E-01	4.061	0.569	3.456	4.585	4.947	0.644	4.470	5.679
ENSG00000157916	RER1	-1.2	1.0E-04	3.0E-04	24.210	0.883	23.236	24.957	28.314	1.286	27.298	29.760
ENSG00000157954	WIPI2	-1.2	2.0E-05	6.8E-05	22.305	0.540	21.716	22.777	26.068	0.762	25.317	26.841
ENSG00000157992	KRTCAP3	-1.2	1.4E-02	2.5E-02	29.524	0.710	28.782	30.196	34.525	3.953	29.969	37.031
ENSG00000158158	CNNM4	-1.2	1.5E-02	2.7E-02	7.879	0.596	7.345	8.523	9.115	0.382	8.893	9.556
ENSG00000158169	FANCC	-1.2	6.5E-04	1.6E-03	5.515	0.300	5.192	5.785	6.751	0.392	6.323	7.093
ENSG00000158292	GPR153	-1.2	3.2E-02	5.4E-02	5.035	0.261	4.842	5.332	5.968	0.704	5.544	6.781
ENSG00000158301	GPRASP2	-1.2	7.2E-04	1.8E-03	12.801	0.864	12.115	13.772	15.419	0.309	15.216	15.775
ENSG00000158435	CNOT11	-1.2	4.3E-07	2.0E-06	59.115	2.582	56.340	61.448	73.290	2.140	72.012	75.761
ENSG00000158470	B4GALT5	-1.2	7.2E-06	2.7E-05	79.354	1.306	77.926	80.488	91.994	0.992	90.850	92.627
ENSG00000158526	TSR2	-1.2	1.8E-04	4.9E-04	65.933	2.030	63.591	67.192	78.655	2.717	75.887	81.318
ENSG00000158636	EMSY	-1.2	6.5E-05	2.0E-04	10.152	0.454	9.772	10.655	12.027	0.242	11.748	12.182
ENSG00000158793	NIT1	-1.2	3.7E-03	7.8E-03	16.998	0.436	16.494	17.254	19.609	1.155	18.303	20.498
ENSG00000158941	CCAR2	-1.2	2.8E-07	1.3E-06	58.544	0.464	58.211	59.075	70.143	1.935	68.155	72.021
ENSG00000158985	CDC42SE2	-1.2	3.8E-05	1.2E-04	20.997	1.079	20.299	22.240	24.962	0.569	24.357	25.486
ENSG00000159023	EPB41	-1.2	5.0E-08	2.8E-07	35.381	1.159	34.178	36.490	42.743	0.470	42.223	43.137
ENSG00000159147	DONSON	-1.2	3.3E-05	1.1E-04	22.671	0.900	22.017	23.698	27.749	1.262	26.423	28.935
ENSG00000159199	ATP5G1	-1.2	2.0E-04	5.5E-04	59.978	1.526	58.847	61.714	69.538	4.507	64.661	73.549
ENSG00000159307	SCUBE1	-1.2	2.9E-02	4.9E-02	1.527	0.152	1.383	1.685	1.857	0.094	1.749	1.922
ENSG00000159374	M1AP	-1.2	1.3E-01	1.8E-01	3.832	0.163	3.697	4.013	4.426	0.316	4.098	4.727
ENSG00000159459	UBR1	-1.2	4.3E-04	1.1E-03	12.757	0.158	12.577	12.875	14.786	0.330	14.591	15.167
ENSG00000159461	AMFR	-1.2	8.3E-04	2.0E-03	12.121	0.602	11.530	12.733	14.248	0.600	13.610	14.801
ENSG00000160049	DFFA	-1.2	5.1E-09	3.5E-08	50.506	1.672	49.294	52.413	62.796	1.707	60.830	63.906
ENSG00000160075	SSU72	-1.2	2.1E-05	7.2E-05	33.356	0.802	32.452	33.981	38.955	2.508	36.349	41.352
ENSG00000160087	UBE2J2	-1.2	6.8E-04	1.7E-03	19.829	0.973	18.802	20.738	23.302	0.741	22.646	24.106
ENSG00000160191	PDE9A	-1.2	2.3E-05	7.6E-05	13.574	0.606	13.100	14.256	16.761	0.471	16.234	17.140
ENSG00000160208	RRP1B	-1.2	1.3E-07	6.8E-07	75.365	2.147	73.130	77.411	90.659	0.162	90.475	90.782
ENSG00000160214	RRP1	-1.2	2.9E-05	9.5E-05	27.517	1.926	25.387	29.134	33.129	0.390	32.742	33.521
ENSG00000160221	C21orf33	-1.2	2.1E-03	4.7E-03	28.347	1.121	27.403	29.586	32.720	0.549	32.098	33.135
ENSG00000160293	VAV2	-1.2	3.7E-07	1.8E-06	30.780	0.844	29.935	31.624	37.583	0.965	36.596	38.524
ENSG00000160410	SHKBP1	-1.2	4.4E-04	1.1E-03	25.226	1.132	24.131	26.391	31.128	3.112	27.860	34.056
ENSG00000160447	PKN3	-1.2	3.7E-03	7.8E-03	11.499	0.593	10.821	11.923	13.867	1.448	12.417	15.313
ENSG00000160710	ADAR	-1.2	2.9E-09	2.1E-08	140.759	3.869	136.542	144.144	173.449	0.987	172.672	174.560
ENSG00000160796	NBEAL2	-1.2	4.1E-04	1.1E-03	11.254	0.625	10.701	11.932	13.389	0.801	12.818	14.304
ENSG00000160877	NACC1	-1.2	2.9E-08	1.7E-07	123.125	1.409	121.577	124.335	150.934	7.653	142.124	155.930
ENSG00000160917	CPSF4	-1.2	2.6E-04	7.1E-04	31.704	1.048	30.528	32.540	36.896	2.314	35.399	39.561
ENSG00000160959	LRRC14	-1.2	3.3E-04	8.6E-04	7.329	0.412	7.068	7.804	9.154	0.774	8.264	9.667
ENSG00000161013	MGAT4B	-1.2	2.1E-05	7.1E-05	20.493	1.892	18.932	22.598	25.561	2.666	22.777	28.091
ENSG00000161036	LRWD1	-1.2	4.4E-05	1.4E-04	12.270	0.258	11.981	12.477	15.216	0.728	14.591	16.016
ENSG00000161179	YDJC	-1.2	1.2E-02	2.3E-02	21.034	2.443	18.402	23.228	25.523	2.947	22.379	28.221
ENSG00000161671	EMC10	-1.2	3.7E-06	1.4E-05	13.292	0.211	13.094	13.513	16.080	0.328	15.775	16.428
ENSG00000161813	LARP4	-1.2	1.6E-07	8.0E-07	41.121	2.938	38.235	44.109	50.958	2.560	48.261	53.356
ENSG00000161847	RAVER1	-1.2	4.8E-05	1.5E-04	16.607	0.834	15.805	17.470	20.459	1.239	19.042	21.339
ENSG00000161956	SENP3	-1.2	2.0E-04	5.6E-04	21.102	0.461	20.787	21.630	25.196	0.829	24.239	25.681
ENSG00000161980	POLR3K	-1.2	1.7E-05	6.0E-05	36.562	0.345	36.197	36.883	45.694	0.408	45.252	46.056
ENSG00000161999	JMJD8	-1.2	6.1E-04	1.5E-03	32.135	1.429	30.527	33.255	37.887	1.715	36.052	39.450
ENSG00000162076	FLYWCH2	-1.2	6.4E-03	1.3E-02	21.113	1.597	19.675	22.832	25.691	1.313	24.458	27.073
ENSG00000162139	NEU3	-1.2	8.5E-05	2.5E-04	12.496	0.317	12.154	12.782	14.922	0.680	14.383	15.685
ENSG00000162231	NXF1	-1.2	4.9E-04	1.2E-03	12.941	0.603	12.553	13.636	15.324	0.417	14.845	15.606
ENSG00000162298	SYVN1	-1.2	1.3E-03	2.9E-03	8.360	0.567	7.863	8.977	10.137	0.447	9.658	10.545
ENSG00000162430	SELENON	-1.2	1.3E-06	5.4E-06	69.008	1.574	67.269	70.336	83.167	5.153	77.391	87.292
ENSG00000162458	FBLIM1	-1.2	1.6E-04	4.5E-04	37.962	1.403	36.843	39.536	43.785	2.239	42.223	46.350
ENSG00000162521	RBBP4	-1.2	9.9E-09	6.4E-08	80.558	0.883	79.846	81.546	96.816	2.063	94.434	98.011
ENSG00000162627	SNX7	-1.2	8.1E-04	2.0E-03	33.817	1.109	32.778	34.984	39.929	1.838	38.164	41.832
ENSG00000162885	B3GALNT2	-1.2	7.4E-05	2.2E-04	12.388	0.171	12.262	12.582	15.320	1.135	14.062	16.267
ENSG00000162889	MAPKAPK2	-1.2	7.2E-06	2.6E-05	23.835	0.415	23.364	24.145	29.865	2.798	26.672	31.888
ENSG00000162928	PEX13	-1.2	1.6E-04	4.6E-04	14.206	0.446	13.760	14.652	16.965	0.503	16.402	17.372
ENSG00000162949	CAPN13	-1.2	8.8E-02	1.3E-01	1.877	0.165	1.753	2.064	2.228	0.230	1.964	2.390
ENSG00000162961	DPY30	-1.2	2.3E-05	7.8E-05	63.022	4.067	58.990	67.644	75.464	4.559	70.292	78.901
ENSG00000162972	MAIP1	-1.2	2.9E-04	7.7E-04	37.172	1.725	35.682	39.062	45.478	1.952	43.327	47.136
ENSG00000163026	WDCP	-1.2	3.3E-04	8.6E-04	34.293	2.398	31.688	36.406	40.684	2.463	38.118	43.028
ENSG00000163029	SMC6	-1.2	2.4E-07	1.2E-06	31.785	2.024	29.569	33.535	39.114	2.157	37.195	41.449
ENSG00000163104	SMARCAD1	-1.2	6.5E-08	3.6E-07	51.601	1.724	50.439	53.582	62.924	2.224	60.740	65.186
ENSG00000163156	SCNM1	-1.2	1.3E-03	2.9E-03	9.959	0.675	9.189	10.448	12.291	0.494	11.765	12.746
ENSG00000163159	VPS72	-1.2	2.2E-04	5.9E-04	35.757	1.937	33.824	37.698	41.385	1.959	39.678	43.523
ENSG00000163281	GNPDA2	-1.2	5.8E-03	1.2E-02	4.433	0.400	4.154	4.892	5.367	0.283	5.099	5.663
ENSG00000163312	HELQ	-1.2	4.2E-02	6.8E-02	5.946	0.256	5.695	6.208	7.004	0.846	6.172	7.863
ENSG00000163348	PYGO2	-1.2	1.9E-04	5.4E-04	23.015	1.547	21.983	24.795	27.638	1.235	26.745	29.048
ENSG00000163449	TMEM169	-1.2	6.3E-02	9.8E-02	2.447	0.112	2.327	2.547	3.007	0.358	2.790	3.420
ENSG00000163517	HDAC11	-1.2	2.6E-03	5.7E-03	5.714	0.443	5.202	5.977	6.990	0.556	6.428	7.540
ENSG00000163655	GMPS	-1.2	4.5E-07	2.1E-06	66.048	0.803	65.444	66.960	77.107	0.550	76.777	77.743
ENSG00000163735	CXCL5	-1.2	1.4E-02	2.6E-02	19.226	0.325	18.853	19.443	23.456	2.278	20.829	24.877
ENSG00000163754	GYG1	-1.2	9.2E-04	2.2E-03	27.159	1.067	26.491	28.389	31.463	0.229	31.265	31.714
ENSG00000163788	SNRK	-1.2	8.9E-03	1.7E-02	6.940	0.478	6.557	7.475	8.125	0.881	7.110	8.700
ENSG00000163812	ZDHHC3	-1.2	5.2E-05	1.6E-04	21.783	1.339	20.711	23.284	25.609	0.939	24.582	26.422
ENSG00000163827	LRRC2	-1.2	2.7E-02	4.6E-02	4.650	0.177	4.446	4.755	5.424	0.115	5.302	5.530
ENSG00000163832	ELP6	-1.2	1.1E-04	3.1E-04	22.942	0.810	22.223	23.819	27.546	1.212	26.754	28.942
ENSG00000163870	TPRA1	-1.2	1.1E-03	2.5E-03	9.463	0.846	8.937	10.439	11.893	1.327	10.449	13.058
ENSG00000163913	IFT122	-1.2	1.2E-02	2.3E-02	6.519	0.060	6.452	6.567	7.521	0.523	6.922	7.885
ENSG00000163930	BAP1	-1.2	4.8E-07	2.2E-06	40.084	1.252	38.725	41.190	49.043	2.153	47.309	51.453
ENSG00000164048	ZNF589	-1.2	3.5E-06	1.4E-05	68.862	4.963	63.671	73.560	81.956	1.972	80.317	84.144
ENSG00000164077	MON1A	-1.2	3.8E-02	6.2E-02	3.431	0.723	2.622	4.016	4.225	0.697	3.421	4.664
ENSG00000164081	TEX264	-1.2	1.8E-02	3.2E-02	3.376	0.207	3.150	3.555	4.097	0.474	3.609	4.555
ENSG00000164087	POC1A	-1.2	1.4E-03	3.3E-03	33.524	0.637	32.796	33.981	39.680	3.308	35.870	41.813
ENSG00000164091	WDR82	-1.2	3.6E-10	3.1E-09	74.477	1.013	73.307	75.091	93.263	2.052	91.079	95.150
ENSG00000164190	NIPBL	-1.2	4.1E-06	1.6E-05	26.997	1.319	25.809	28.417	31.653	0.901	30.714	32.511
ENSG00000164237	CMBL	-1.2	2.4E-03	5.2E-03	21.024	0.741	20.532	21.877	24.973	2.029	22.686	26.559
ENSG00000164251	F2RL1	-1.2	2.6E-04	7.0E-04	20.925	0.650	20.333	21.621	25.121	0.131	25.005	25.263
ENSG00000164323	CFAP97	-1.2	8.6E-08	4.6E-07	27.285	1.052	26.091	28.075	34.116	1.684	32.219	35.480
ENSG00000164647	STEAP1	-1.2	7.2E-02	1.1E-01	10.367	0.589	9.688	10.738	12.046	0.643	11.304	12.419
ENSG00000164684	ZNF704	-1.2	1.2E-03	2.8E-03	2.019	0.181	1.839	2.201	2.525	0.168	2.333	2.643
ENSG00000164818	DNAAF5	-1.2	8.0E-05	2.4E-04	23.737	0.521	23.389	24.335	27.938	2.726	25.111	30.551
ENSG00000164841	TMEM74	-1.2	8.7E-02	1.3E-01	3.228	0.594	2.790	3.904	3.928	0.365	3.687	4.349
ENSG00000164934	DCAF13	-1.2	1.9E-05	6.4E-05	49.172	1.291	47.996	50.554	57.523	1.059	56.564	58.660
ENSG00000164961	WASHC5	-1.2	1.5E-07	7.8E-07	40.504	1.197	39.143	41.394	50.470	1.357	49.200	51.899
ENSG00000164985	PSIP1	-1.2	2.5E-06	1.0E-05	240.022	14.837	223.929	253.158	278.124	5.517	272.140	283.009
ENSG00000165072	MAMDC2	-1.2	1.4E-04	3.9E-04	16.739	0.547	16.300	17.352	20.531	0.785	19.626	21.022
ENSG00000165102	HGSNAT	-1.2	8.2E-02	1.2E-01	1.661	0.140	1.536	1.813	1.966	0.107	1.902	2.089
ENSG00000165219	GAPVD1	-1.2	4.4E-05	1.4E-04	10.942	0.414	10.477	11.270	13.065	0.496	12.630	13.605
ENSG00000165246	NLGN4Y	-1.2	1.0E-01	1.5E-01	2.010	0.276	1.796	2.322	2.328	0.241	2.054	2.506
ENSG00000165392	WRN	-1.2	1.4E-04	4.0E-04	41.038	1.119	39.779	41.920	47.502	1.087	46.422	48.596
ENSG00000165416	SUGT1	-1.2	9.2E-06	3.3E-05	14.487	0.636	13.767	14.970	17.140	0.425	16.759	17.597
ENSG00000165434	PGM2L1	-1.2	1.4E-05	4.8E-05	20.814	1.157	19.521	21.753	25.205	0.829	24.536	26.132
ENSG00000165494	PCF11	-1.2	8.9E-07	3.9E-06	17.502	1.573	15.821	18.938	21.874	0.601	21.432	22.558
ENSG00000165512	ZNF22	-1.2	2.1E-07	1.0E-06	76.174	1.697	74.421	77.810	93.279	4.374	89.503	98.072
ENSG00000165630	PRPF18	-1.2	7.1E-02	1.1E-01	9.341	0.506	8.996	9.922	10.823	0.191	10.602	10.935
ENSG00000165644	COMTD1	-1.2	1.5E-02	2.7E-02	14.556	0.506	13.972	14.849	17.447	1.191	16.373	18.728
ENSG00000165716	FAM69B	-1.2	5.6E-04	1.4E-03	24.342	0.787	23.775	25.240	29.656	2.824	26.398	31.405
ENSG00000165804	ZNF219	-1.2	4.6E-04	1.2E-03	31.234	1.701	29.465	32.858	38.491	3.420	34.948	41.772
ENSG00000165916	PSMC3	-1.2	9.8E-07	4.3E-06	166.998	3.905	163.877	171.377	195.654	2.569	192.830	197.855
ENSG00000166004	CEP295	-1.2	9.6E-05	2.8E-04	6.344	0.150	6.171	6.439	7.752	0.289	7.525	8.091
ENSG00000166033	HTRA1	-1.2	3.2E-02	5.4E-02	9.098	0.829	8.227	9.877	10.971	1.333	9.620	12.285
ENSG00000166086	JAM3	-1.2	8.4E-07	3.7E-06	24.951	0.544	24.323	25.281	30.631	0.629	30.144	31.341

ENSG00000166133	RPUSD2	-1.2	1.2E-03	2.8E-03	18.367	1.350	17.288	19.881	22.280	0.451	22.006	22.800
ENSG00000166313	APBB1	-1.2	1.0E-03	2.5E-03	13.991	0.262	13.716	14.239	16.450	0.434	15.956	16.772
ENSG00000166377	ATP9B	-1.2	9.4E-03	1.8E-02	2.959	0.196	2.835	3.185	3.439	0.179	3.272	3.627
ENSG00000166411	IDH3A	-1.2	1.5E-05	5.2E-05	11.445	0.510	10.957	11.974	14.287	0.633	13.892	15.018
ENSG00000166415	WDR72	-1.2	2.0E-02	3.6E-02	1.897	0.075	1.837	1.981	2.360	0.365	2.034	2.754
ENSG00000166444	ST5	-1.2	5.5E-03	1.1E-02	3.879	0.061	3.835	3.948	4.517	0.406	4.231	4.981
ENSG00000166454	ATMIN	-1.2	4.1E-07	1.9E-06	36.937	1.596	35.264	38.442	44.804	1.409	43.287	46.071
ENSG00000166508	MCM7	-1.2	5.0E-06	1.9E-05	238.255	2.725	235.390	240.814	275.062	5.578	271.729	281.502
ENSG00000166529	ZSCAN21	-1.2	5.7E-04	1.4E-03	19.001	0.597	18.356	19.534	23.288	0.768	22.403	23.768
ENSG00000166562	SEC11C	-1.2	5.7E-04	1.4E-03	18.459	1.431	16.903	19.719	21.887	1.085	20.663	22.732
ENSG00000166681	BEX3	-1.2	1.1E-06	4.6E-06	519.848	26.030	503.340	549.855	608.535	11.518	600.499	621.732
ENSG00000166685	COG1	-1.2	1.7E-03	3.8E-03	7.081	0.493	6.655	7.621	8.595	0.574	8.118	9.233
ENSG00000166689	PLEKHA7	-1.2	1.3E-02	2.3E-02	3.318	0.286	2.992	3.531	3.865	0.312	3.560	4.183
ENSG00000166747	AP1G1	-1.2	7.6E-06	2.8E-05	32.867	2.044	31.376	35.197	38.298	0.960	37.233	39.095
ENSG00000166831	RBPMS2	-1.2	8.8E-06	3.2E-05	142.722	5.973	136.747	148.692	168.612	8.568	161.994	178.290
ENSG00000166860	ZBTB39	-1.2	6.4E-05	1.9E-04	19.923	0.412	19.563	20.372	24.166	1.085	23.333	25.394
ENSG00000166887	VPS39	-1.2	5.2E-04	1.3E-03	20.848	0.707	20.194	21.599	24.113	0.263	23.861	24.386
ENSG00000166888	STAT6	-1.2	1.8E-05	6.1E-05	16.454	0.633	16.074	17.184	19.719	1.000	18.777	20.768
ENSG00000166902	MRPL16	-1.2	7.2E-07	3.2E-06	56.478	2.826	54.106	59.605	70.143	0.824	69.629	71.093
ENSG00000166908	PIP4K2C	-1.2	2.6E-04	7.0E-04	22.335	1.090	21.403	23.534	26.924	0.973	26.081	27.988
ENSG00000166938	DIS3L	-1.2	8.3E-05	2.5E-04	18.300	1.006	17.692	19.460	21.833	0.277	21.648	22.151
ENSG00000167081	PBX3	-1.2	3.3E-05	1.1E-04	17.239	0.724	16.701	18.062	21.295	1.174	20.022	22.335
ENSG00000167105	TMEM92	-1.2	4.9E-02	7.8E-02	8.595	1.167	7.433	9.766	10.065	1.552	8.379	11.433
ENSG00000167118	URM1	-1.2	5.3E-06	2.0E-05	19.599	0.336	19.277	19.885	24.526	0.215	24.357	24.768
ENSG00000167272	POP5	-1.2	8.3E-05	2.5E-04	29.343	2.002	27.773	31.597	36.252	2.313	33.685	38.173
ENSG00000167384	ZNF180	-1.2	3.4E-02	5.7E-02	7.323	0.251	7.045	7.534	8.479	0.786	7.907	9.375
ENSG00000167395	ZNF646	-1.2	2.1E-04	5.8E-04	8.071	0.327	7.704	8.332	9.732	0.411	9.365	10.175
ENSG00000167461	RAB8A	-1.2	3.7E-06	1.5E-05	27.037	1.149	25.839	28.131	33.185	1.559	31.511	34.596
ENSG00000167548	KMT2D	-1.2	2.1E-06	8.7E-06	15.503	1.115	14.277	16.457	19.186	0.808	18.624	20.112
ENSG00000167565	SERTAD3	-1.2	2.7E-02	4.6E-02	12.352	1.244	10.962	13.361	14.411	1.228	12.994	15.186
ENSG00000167595	PROSER3	-1.2	8.6E-03	1.6E-02	3.382	0.524	2.832	3.874	4.158	0.436	3.778	4.634
ENSG00000167632	TRAPPC9	-1.2	4.1E-03	8.4E-03	3.739	0.100	3.658	3.851	4.539	0.262	4.237	4.697
ENSG00000167637	ZNF283	-1.2	1.3E-02	2.4E-02	2.950	0.373	2.519	3.186	3.641	0.376	3.207	3.865
ENSG00000167670	CHAF1A	-1.2	1.3E-05	4.6E-05	80.314	1.424	78.687	81.330	93.491	2.290	91.078	95.634
ENSG00000167685	ZNF444	-1.2	6.8E-03	1.3E-02	8.471	0.155	8.296	8.593	9.796	0.040	9.769	9.841
ENSG00000167861	HID1	-1.2	2.7E-02	4.6E-02	2.417	0.218	2.167	2.570	2.937	0.270	2.751	3.246
ENSG00000167900	TK1	-1.2	8.8E-05	2.6E-04	82.354	3.762	79.001	86.422	96.716	3.374	94.126	100.531
ENSG00000167962	ZNF598	-1.2	1.5E-04	4.3E-04	17.840	0.038	17.800	17.875	21.696	2.226	20.307	24.264
ENSG00000168005	C11orf84	-1.2	1.8E-04	4.9E-04	59.280	1.384	57.681	60.082	69.263	4.060	64.793	72.723
ENSG00000168159	RNF187	-1.2	2.9E-05	9.4E-05	38.276	1.032	37.448	39.431	46.161	2.800	43.161	48.705
ENSG00000168243	GNG4	-1.2	2.3E-05	7.7E-05	24.590	0.916	23.930	25.636	29.881	1.767	28.049	31.575
ENSG00000168286	THAP11	-1.2	7.2E-05	2.2E-04	48.831	2.345	47.222	51.521	59.091	3.074	56.034	62.182
ENSG00000168434	COG7	-1.2	7.2E-03	1.4E-02	8.023	0.639	7.334	8.596	9.496	0.731	8.716	10.165
ENSG00000168496	FEN1	-1.2	2.7E-08	1.6E-07	103.140	1.606	101.996	104.976	127.264	1.957	125.295	129.208
ENSG00000168564	CDKN2AIP	-1.2	5.0E-05	1.6E-04	17.003	0.348	16.674	17.367	21.329	1.345	20.499	22.880
ENSG00000168591	TMUB2	-1.2	1.3E-03	3.1E-03	9.145	0.796	8.309	9.894	11.203	0.254	11.052	11.496
ENSG00000168765	GSTM4	-1.2	3.3E-02	5.5E-02	4.907	0.478	4.356	5.215	5.655	0.356	5.308	6.019
ENSG00000168872	DDX19A	-1.2	2.3E-04	6.2E-04	6.919	0.603	6.249	7.417	8.501	0.056	8.463	8.565
ENSG00000168884	TNIP2	-1.2	2.1E-04	5.7E-04	27.442	0.918	26.409	28.162	33.156	1.658	31.880	35.030
ENSG00000168913	ENHO	-1.2	4.0E-03	8.2E-03	23.111	1.142	21.814	23.965	28.719	1.916	27.474	30.925
ENSG00000168924	LETM1	-1.2	2.1E-06	8.6E-06	15.401	0.456	15.020	15.907	19.100	1.101	18.127	20.294
ENSG00000169071	ROR2	-1.2	4.6E-02	7.5E-02	3.824	0.146	3.715	3.990	4.448	0.340	4.106	4.785
ENSG00000169116	PARM1	-1.2	1.8E-02	3.1E-02	5.227	0.477	4.752	5.706	6.300	0.968	5.300	7.234
ENSG00000169118	CSNK1G1	-1.2	9.0E-03	1.7E-02	5.019	0.361	4.713	5.417	5.826	0.175	5.658	6.008
ENSG00000169129	AFAP1L2	-1.2	1.2E-01	1.7E-01	2.094	0.318	1.733	2.333	2.444	0.269	2.137	2.638
ENSG00000169169	CPT1C	-1.2	8.8E-04	2.1E-03	12.796	0.908	11.766	13.480	15.137	0.865	14.602	16.136
ENSG00000169213	RAB3B	-1.2	3.0E-06	1.2E-05	31.547	1.106	30.333	32.499	37.362	0.465	36.842	37.741
ENSG00000169604	ANTXR1	-1.2	6.0E-07	2.7E-06	20.294	0.594	19.693	20.881	25.121	0.710	24.307	25.611
ENSG00000169641	LUZP1	-1.2	1.5E-07	7.6E-07	34.419	1.233	33.401	35.790	41.551	0.796	40.832	42.407
ENSG00000169684	CHRNA5	-1.2	4.3E-03	8.9E-03	15.748	0.582	15.294	16.405	18.434	0.599	17.851	19.047
ENSG00000169696	ASPSCR1	-1.2	6.8E-04	1.7E-03	4.452	0.112	4.325	4.537	5.385	0.168	5.287	5.579
ENSG00000169758	TMEM266	-1.2	5.7E-02	9.0E-02	1.636	0.151	1.485	1.788	2.030	0.219	1.778	2.176
ENSG00000169862	CTNND2	-1.2	3.9E-02	6.5E-02	3.994	0.373	3.666	4.399	4.614	0.066	4.550	4.681
ENSG00000169926	KLF13	-1.2	3.3E-07	1.6E-06	16.851	0.742	16.019	17.441	21.069	0.395	20.695	21.482
ENSG00000169957	ZNF768	-1.2	7.1E-04	1.7E-03	31.192	0.664	30.498	31.821	37.902	3.596	34.811	41.791
ENSG00000170035	UBE2E3	-1.2	4.6E-06	1.7E-05	58.966	0.898	57.928	59.497	70.224	1.907	68.106	71.805
ENSG00000170185	USP38	-1.2	8.5E-05	2.5E-04	7.445	0.256	7.158	7.651	9.220	0.500	8.739	9.736
ENSG00000170234	PWWP2A	-1.2	3.2E-07	1.5E-06	26.504	0.334	26.270	26.887	32.816	0.872	32.216	33.817
ENSG00000170242	USP47	-1.2	3.7E-06	1.4E-05	14.030	0.481	13.543	14.504	16.983	0.680	16.388	17.740
ENSG00000170265	ZNF282	-1.2	9.4E-04	2.2E-03	23.134	1.020	22.435	24.304	26.907	0.959	25.809	27.578
ENSG00000170270	GON7	-1.2	4.1E-02	6.7E-02	16.943	1.081	16.050	18.145	19.742	0.901	18.788	20.578
ENSG00000170296	GABARAP	-1.2	8.7E-04	2.1E-03	24.313	0.682	23.530	24.785	29.292	1.290	27.922	30.484
ENSG00000170502	NUDT9	-1.2	3.6E-04	9.4E-04	24.434	0.588	23.757	24.820	29.141	2.187	26.639	30.688
ENSG00000170545	SMAGP	-1.2	6.9E-02	1.1E-01	3.077	0.201	2.874	3.277	3.706	0.287	3.388	3.965
ENSG00000170779	CDCA4	-1.2	7.3E-05	2.2E-04	58.627	2.587	56.920	61.604	68.770	1.215	67.371	69.565
ENSG00000170791	CHCHD7	-1.2	8.9E-06	3.2E-05	17.606	0.784	16.787	18.349	21.688	1.166	20.759	23.004
ENSG00000170836	PPM1D	-1.2	3.3E-03	7.0E-03	12.373	0.539	12.057	12.995	14.758	0.770	13.909	15.410
ENSG00000170915	PAQR8	-1.2	7.1E-04	1.8E-03	10.414	0.410	9.981	10.797	12.570	0.752	11.933	13.400
ENSG00000171103	TRMT61B	-1.2	7.5E-06	2.7E-05	37.883	1.994	35.584	39.143	47.349	1.304	46.493	48.850
ENSG00000171109	MFN1	-1.2	1.6E-07	7.9E-07	26.906	0.849	26.256	27.867	33.518	0.416	33.160	33.974
ENSG00000171241	SHCBP1	-1.2	8.9E-04	2.1E-03	13.351	0.582	12.859	14.019	15.939	0.751	15.071	16.392
ENSG00000171307	ZDHHC16	-1.2	1.1E-04	3.1E-04	30.862	1.201	30.073	32.244	37.219	2.956	34.945	40.561
ENSG00000171451	DSEL	-1.2	6.5E-04	1.6E-03	9.660	0.123	9.520	9.749	11.983	0.679	11.203	12.435
ENSG00000171492	LRRC8D	-1.2	5.4E-07	2.5E-06	34.591	1.538	32.925	35.957	42.922	1.650	41.018	43.957
ENSG00000171612	SLC25A33	-1.2	3.8E-03	8.0E-03	10.256	0.288	10.087	10.588	12.259	0.563	11.617	12.667
ENSG00000171621	SPSB1	-1.2	7.9E-02	1.2E-01	2.901	0.636	2.528	3.635	3.591	0.243	3.325	3.801
ENSG00000171700	RGS19	-1.2	6.4E-02	9.9E-02	7.021	0.853	6.469	8.004	8.424	0.718	7.715	9.151
ENSG00000171723	GPHN	-1.2	3.2E-03	6.8E-03	6.332	0.226	6.134	6.578	7.555	0.523	6.951	7.877
ENSG00000171724	VAT1L	-1.2	7.2E-05	2.2E-04	23.600	1.260	22.871	25.055	28.319	2.210	26.438	30.753
ENSG00000171763	SPATA5L1	-1.2	2.0E-03	4.5E-03	9.126	0.978	8.006	9.810	11.356	0.553	10.827	11.931
ENSG00000171793	CTPS1	-1.2	2.9E-08	1.7E-07	65.264	0.529	64.712	65.766	79.509	2.677	77.452	82.536
ENSG00000171865	RNASEH1	-1.2	7.5E-06	2.8E-05	34.195	0.929	33.644	35.268	42.214	1.786	40.176	43.503
ENSG00000171984	C20orf196	-1.2	9.9E-02	1.5E-01	3.314	0.200	3.083	3.433	4.102	0.385	3.717	4.487
ENSG00000172006	ZNF554	-1.2	7.3E-02	1.1E-01	1.989	0.223	1.843	2.246	2.404	0.193	2.183	2.539
ENSG00000172009	THOP1	-1.2	2.7E-07	1.3E-06	24.064	0.765	23.317	24.846	29.729	1.155	28.971	31.058
ENSG00000172046	USP19	-1.2	4.1E-06	1.6E-05	27.708	0.717	27.043	28.467	33.537	0.533	32.922	33.859
ENSG00000172197	MBOAT1	-1.2	8.7E-04	2.1E-03	18.496	1.644	17.293	20.369	23.005	1.602	21.283	24.451
ENSG00000172262	ZNF131	-1.2	1.7E-05	5.7E-05	13.054	0.555	12.675	13.691	15.955	0.270	15.660	16.190
ENSG00000172345	STARD5	-1.2	1.0E-01	1.5E-01	1.825	0.435	1.364	2.228	2.154	0.149	1.983	2.262
ENSG00000172575	RASGRP1	-1.2	4.3E-02	7.0E-02	2.036	0.195	1.810	2.152	2.458	0.178	2.329	2.661
ENSG00000173040	EVC2	-1.2	6.9E-03	1.4E-02	4.745	0.471	4.310	5.245	5.765	0.231	5.578	6.023
ENSG00000173418	NAA20	-1.2	1.4E-03	3.3E-03	35.925	1.406	34.560	37.369	41.640	2.057	39.349	43.329
ENSG00000173436	MINOS1	-1.2	1.8E-02	3.2E-02	5.688	0.408	5.217	5.935	6.652	0.288	6.321	6.835
ENSG00000173457	PPP1R14B	-1.2	9.1E-04	2.2E-03	115.489	4.423	112.505	120.570	133.206	15.262	117.399	147.856
ENSG00000173548	SNX33	-1.2	4.4E-05	1.4E-04	13.211	0.123	13.135	13.352	16.074	0.836	15.193	16.858
ENSG00000173598	NUDT4	-1.2	2.0E-07	1.0E-06	18.766	0.668	18.145	19.472	23.424	1.219	22.123	24.539
ENSG00000173653	RCE1	-1.2	1.2E-02	2.3E-02	13.177	0.613	12.682	13.863	15.240	0.301	15.025	15.585
ENSG00000173692	PSMD1	-1.2	4.1E-08	2.3E-07	150.423	1.800	148.425	151.918	178.115	3.252	175.263	181.656
ENSG00000173786	CNP	-1.2	2.1E-07	1.1E-06	21.635	0.248	21.349	21.793	26.815	0.537	26.485	27.434
ENSG00000173801	JUP	-1.2	7.9E-08	4.2E-07	46.506	1.040	45.746	47.691	58.238	3.478	54.666	61.614
ENSG00000173852	DPY19L1	-1.2	1.8E-03	4.0E-03	14.914	2.035	13.239	17.179	17.515	0.147	17.377	17.669
ENSG00000173914	RBM4B	-1.2	1.6E-03	3.7E-03	11.018	0.880	10.082	11.828	13.328	0.403	12.974	13.767
ENSG00000173950	XXYLT1	-1.2	2.8E-03	5.9E-03	12.542	0.449	12.071	12.964	14.562	0.918	13.507	15.180
ENSG00000173960	UBXN2A	-1.2	1.0E-03	2.4E-03	11.670	0.614	11.169	12.355	13.855	0.606	13.320	14.513
ENSG00000174080	CTSF	-1.2	4.6E-04	1.2E-03	15.784	1.109	14.741	16.949	19.555	0.688	18.933	20.294
ENSG00000174197	MGA	-1.2	2.9E-06	1.1E-05	16.102	0.482	15.548	16.425	19.120	0.659	18.642	19.872
ENSG00000174238	PITPNA	-1.2	3.2E-05	1.0E-04	36.958	1.073	35.814	37.942	43.246	0.322	42.893	43.525
ENSG00000174373	RALGAPA1	-1.2	4.5E-04	1.1E-03	3.266	0.119	3.129	3.336	4.000	0.153	3.830	4.126
ENSG00000174574	AKIRIN1	-1.2	1.5E-07	7.5E-07	286.881	7.061	280.148	294.230	339.413	6.231	333.518	345.933
ENSG00000174628	IQCK	-1.2	6.3E-02	9.8E-02	3.282	0.395	2.861	3.645	3.956	0.839	2.994	4.533
ENSG00000174839	DENND6A	-1.2	5.5E-03	1.1E-02	10.704	0.387	10.422	11.145	12.357	0.243	12.077	12.512
ENSG00000174953	DHX36	-1.2	8.3E-06	3.0E-05	41.165	0.629	40.655	41.867	47.932	0.870	47.388	48.935
ENSG00000174989	FBXW8	-1.2	2.3E-04	6.2E-04	13.217	0.432	12.819	13.676	15.920	0.244	15.647	16.119
ENSG00000175093	SPSB4	-1.2	7.9E-03	1.5E-02	7.675	0.105	7.594	7.794	9.564	1.709	7.619	10.826
ENSG00000175137	SH3BP5L	-1.2	5.4E-05	1.7E-04	15.113	0.546	14.656	15.718	18.160	1.061	16.935	18.776
ENSG00000175221	MED16	-1.2	2.9E-04	7.7E-04	24.396	0.448	24.128	24.913	28.911	1.992	27.395	31.168
ENSG00000175305	CCNE2	-1.2	1.1E-02	2.1E-02	4.531	0.030	4.504	4.563	5.523	0.444	5.020	5.861
ENSG00000175324	LSM1	-1.2	4.8E-05	1.5E-04	47.901	2.613	46.122	50.901	59.029	3.350	55.432	62.060
ENSG00000175595	ERCC4	-1.2	1.9E-02	3.4E-02	4.707	0.393	4.342	5.123	5.454	0.425	4.964	5.730
ENSG00000175707	KDF1	-1.2	7.5E-03	1.5E-02	14.876	2.461	12.946	17.647	18.190	1.128	17.062	19.317
ENSG00000175741	RWDD4P2	-1.2	1.5E-02	2.7E-02	49.108	3.828	46.007	53.387	58.611	2.442	56.322	61.181
ENSG00000175768	TOMM5	-1.2	2.7E-04	7.3E-04	20.566	0.938	19.510	21.305	24.683	0.234	24.457	24.924
ENSG00000175854	SWI5	-1.2	7.6E-04	1.8E-03	26.416	1.168	25.101	27.332	32.465	1.326	30.949	33.408
ENSG00000176101	SSNA1	-1.2	2.3E-05	7.7E-05	83.289	3.657	79.066	85.456	101.630	7.357	93.165	106.484
ENSG00000176204	LRRTM4	-1.2	3.6E-02	6.0E-02	3.019	0.292	2.682	3.195	3.664	0.094	3.579	3.765
ENSG00000176244	ACBD7	-1.2	1.2E-02	2.2E-02	17.811	2.100	15.387	19.062	21.355	0.892	20.755	22.380
ENSG00000176273	SLC35G1	-1.2	2.3E-02	3.9E-02	3.988	0.254	3.779	4.272	4.727	0.396	4.373	5.155
ENSG00000176463	SLCO3A1	-1.2	1.7E-02	3.0E-02	3.354	0.294	3.059	3.648	3.958	0.164	3.770	4.076
ENSG00000176619	LMNB2	-1.2	4.8E-10	4.1E-09	151.729	2.422	148.950	153.390	188.457	7.729	182.582	197.213
ENSG00000176783	RUFY1	-1.2	3.5E-05	1.1E-04	10.331	0.742	9.529	10.993	12.895	0.443	12.383	13.153
ENSG00000176788	BASP1	-1.2	2.2E-05	7.4E-05	126.751	2.285	125.108	129.360	146.400	5.676	140.129	151.186
ENSG00000176915	ANKLE2	-1.2	1.5E-04	4.1E-04	9.642	0.471	9.253	10.165	11.175	0.361	10.953	11.591
ENSG00000176974	SHMT1	-1.2	2.2E-04	6.0E-04	14.227	0.281	13.904	14.424	16.853	1.359	16.032	18.421
ENSG00000176986	SEC24C	-1.2	1.8E-07	8.9E-07	41.085	1.765	39.812	43.101	50.430	1.028	49.250	51.131
ENSG00000177302	TOP3A	-1.2	3.5E-05	1.1E-04	14.695	0.595	14.229	15.365	18.161	0.800	17.259	18.782
ENSG00000177479	ARIH2	-1.2	6.6E-07	2.9E-06	46.675	0.405	46.370	47.135	55.356	1.434	53.779	56.582
ENSG00000177542	SLC25A22	-1.2	1.8E-04	5.0E-04	12.757	0.671	12.090	13.432	15.882	1.409	15.067	17.518
ENSG00000177548	RABEP2	-1.2	2.0E-03	4.5E-03	5.640	0.434	5.263	6.115	7.004	0.269	6.743	7.281
ENSG00000177576	C18orf32	-1.2	1.0E-01	1.5E-01	1.995	0.096	1.907	2.097	2.322	0.070	2.263	2.399
ENSG00000177666	PNPLA2	-1.2	1.0E-03	2.4E-03	15.017	1.199	13.875	16.266	18.302	1.464	16.611	19.161
ENSG00000177830	CHID1	-1.2	4.3E-06	1.6E-05	21.317	0.505	20.764	21.755	26.297	0.266	26.005	26.525
ENSG00000177855	CACYBPP2	-1.2	9.6E-06	3.4E-05	173.200	6.058	167.742	179.717	211.168	1.393	209.706	212.479
ENSG00000177873	ZNF619	-1.2	2.1E-03	4.6E-03	6.664	0.339	6.331	7.010	8.320	0.510	8.005	8.908
ENSG00000177889	UBE2N	-1.2	1.2E-06	5.3E-06	84.130	0.882	83.115	84.708	97.943	1.756	96.056	99.529
ENSG00000177963	RIC8A	-1.2	1.7E-06	7.2E-06	20.147	0.630	19.449	20.674	24.982	1.228	23.828	26.273
ENSG00000177971	IMP3	-1.2	3.7E-05	1.4E-05	68.142	2.198	66.794	70.678	82.748	3.885	78.476	86.070
ENSG00000178162	FAR2P2	-1.2	1.3E-05	4.6E-05	14.577	0.474	14.078	15.022	18.160	0.563	17.604	18.730
ENSG00000178229	ZNF543	-1.2	2.8E-02	4.7E-02	3.762	0.775	2.868	4.259	4.685	0.201	4.453	4.818
ENSG00000178409	BEND3	-1.2	1.3E-03	2.9E-03	12.552	1.033	11.654	13.680	14.838	0.361	14.472	15.193
ENSG00000178531	CTXN1	-1.2	1.2E-01	1.7E-01	35.450	1.487	34.529	37.165	41.294	6.165	35.196	47.523
ENSG00000178537	SLC25A20	-1.2	5.1E-02	8.0E-02	6.078	1.262	4.668	7.102	7.450	0.131	7.330	7.590
ENSG00000178605	GTPBP6	-1.2	1.1E-04	3.2E-04	29.166	1.008	28.111	30.120	35.039	2.325	32.754	37.403
ENSG00000178691	SUZ12	-1.2	4.9E-07	2.3E-06	29.113	1.265	27.725	30.199	35.716	1.134	34.408	36.431
ENSG00000178814	OPLAH	-1.2	2.9E-03	6.2E-03	7.870	0.845	6.930	8.568	9.612	0.471	9.095	10.016
ENSG00000178888	MRFAP1L1	-1.2	9.0E-08	4.8E-07	81.225	4.308	76.254	83.877	100.961	2.049	99.447	103.292
ENSG00000179010	MRFAP1	-1.2	2.4E-07	1.2E-06	329.956	4.045	325.739	333.805	385.098	10.362	375.761	396.247
ENSG00000179091	CYC1	-1.2	3.6E-05	1.2E-04	116.910	5.765	110.897	122.389	136.101	4.289	132.120	140.643
ENSG00000179115	FARSA	-1.2	2.6E-06	1.0E-05	69.456	5.684	63.528	74.860	85.265	1.515	83.526	86.297
ENSG00000179151	EDC3	-1.2	5.2E-06	2.0E-05	23.688	0.434	23.220	24.076	29.018	0.937	27.937	29.591
ENSG00000179195	ZNF664	-1.2	2.0E-07	1.0E-06	44.931	1.959	42.726	46.469	54.321	1.208	53.190	55.594
ENSG00000179314	WSCD1	-1.2	3.0E-04	8.0E-04	12.281	0.165	12.117	12.446	14.912	0.775	14.058	15.570
ENSG00000179562	GCC1	-1.2	3.6E-04	9.3E-04	14.347	0.169	14.159	14.485	17.047	0.294	16.718	17.281
ENSG00000179918	SEPHS2	-1.2	1.7E-06	7.0E-06	62.931	2.322	60.257	64.438	76.879	0.203	76.707	77.103
ENSG00000180182	MED14	-1.2	3.2E-08	1.9E-07	33.186	0.877	32.257	34.001	41.242	1.218	39.838	42.021
ENSG00000180229	HERC2P3	-1.2	1.3E-03	3.0E-03	13.073	1.286	11.677	14.210	15.166	0.740	14.618	16.007
ENSG00000180304	OAZ2	-1.2	7.1E-08	3.9E-07	87.594	1.639	86.158	89.379	104.130	0.969	103.067	104.963
ENSG00000180537	RNF182	-1.2	4.5E-03	9.2E-03	7.373	0.833	6.415	7.926	9.136	1.446	7.468	10.041
ENSG00000180855	ZNF443	-1.2	6.5E-02	1.0E-01	7.232	0.859	6.260	7.887	8.446	0.953	7.792	9.539
ENSG00000181026	AEN	-1.2	2.2E-05	7.2E-05	66.097	1.933	64.880	68.326	76.685	3.288	73.651	80.178
ENSG00000181220	ZNF746	-1.2	5.2E-03	1.1E-02	5.133	0.388	4.834	5.572	6.178	0.645	5.699	6.912
ENSG00000181227	DLSTP1	-1.2	7.0E-02	1.1E-01	7.901	0.975	6.783	8.572	9.548	1.030	8.359	10.162
ENSG00000181449	SOX2	-1.2	7.4E-07	3.3E-06	211.573	10.443	199.523	218.005	251.296	10.582	242.229	262.923
ENSG00000181481	RNF135	-1.2	9.1E-03	1.7E-02	6.015	0.539	5.532	6.596	7.370	0.123	7.258	7.501
ENSG00000181523	SGSH	-1.2	7.3E-03	1.4E-02	3.415	0.554	2.782	3.813	4.230	0.436	3.766	4.631
ENSG00000181817	LSM10	-1.2	2.0E-04	5.6E-04	51.090	1.339	49.730	52.407	61.438	3.708	59.293	65.720
ENSG00000181904	C5orf24	-1.2	5.9E-04	1.5E-03	12.683	0.700	11.910	13.275	15.243	1.026	14.441	16.400
ENSG00000181929	PRKAG1	-1.2	1.4E-04	3.9E-04	20.062	0.528	19.537	20.593	24.072	1.183	23.046	25.366
ENSG00000181982	CCDC149	-1.2	2.1E-02	3.8E-02	1.730	0.146	1.595	1.885	2.111	0.060	2.041	2.149
ENSG00000182013	PNMA8A	-1.2	3.1E-06	1.2E-05	23.928	0.869	22.925	24.430	29.799	1.246	28.384	30.733
ENSG00000182108	DEXI	-1.2	1.3E-03	3.1E-03	31.743	2.194	29.464	33.840	37.901	1.343	36.666	39.330
ENSG00000182180	MRPS16	-1.2	6.6E-08	3.6E-07	106.392	1.838	104.340	107.887	129.547	1.416	127.889	130.755
ENSG00000182575	NXPH3	-1.2	6.3E-02	9.8E-02	1.871	0.177	1.667	1.983	2.280	0.213	2.052	2.473
ENSG00000182858	ALG12	-1.2	1.3E-03	2.9E-03	11.029	0.557	10.589	11.656	13.099	0.810	12.220	13.815
ENSG00000182871	COL18A1	-1.2	1.3E-06	5.5E-06	81.375	3.791	78.342	85.625	100.732	8.023	95.906	109.994
ENSG00000183060	LYSMD4	-1.2	1.1E-04	3.1E-04	8.833	0.215	8.586	8.968	10.903	0.255	10.617	11.105
ENSG00000183087	GAS6	-1.2	2.5E-03	5.5E-03	8.417	1.407	7.054	9.865	10.100	0.419	9.675	10.512
ENSG00000183166	CALN1	-1.2	8.1E-03	1.6E-02	6.715	0.612	6.009	7.103	7.811	0.323	7.554	8.174
ENSG00000183196	CHST6	-1.2	9.1E-04	2.2E-03	8.509	0.897	7.723	9.486	10.203	0.257	9.999	10.492
ENSG00000183307	CECR6	-1.2	1.1E-02	2.0E-02	3.514	0.160	3.366	3.684	4.363	0.193	4.161	4.546
ENSG00000183309	ZNF623	-1.2	9.1E-05	2.7E-04	12.874	0.805	11.988	13.561	15.479	0.287	15.169	15.736
ENSG00000183431	SF3A3	-1.2	1.2E-08	7.7E-08	117.464	2.835	114.634	120.304	142.134	4.307	138.244	146.762
ENSG00000183474	GTF2H2C	-1.2	6.3E-05	1.9E-04	13.741	0.686	12.986	14.326	16.581	0.278	16.384	16.899
ENSG00000183495	EP400	-1.2	1.2E-06	5.1E-06	17.040	0.324	16.669	17.269	20.330	1.235	18.904	21.049
ENSG00000183530	PRR14L	-1.2	1.1E-06	4.6E-06	24.745	1.760	22.860	26.345	29.881	0.273	29.573	30.093
ENSG00000183570	PCBP3	-1.2	9.9E-02	1.4E-01	1.152	0.311	0.895	1.498	1.396	0.093	1.289	1.458
ENSG00000183597	TANGO2	-1.2	4.1E-03	8.4E-03	3.460	0.196	3.243	3.635	4.294	0.296	4.106	4.635
ENSG00000183666	GUSBP1	-1.2	9.7E-02	1.4E-01	1.389	0.308	1.147	1.737	1.716	0.183	1.600	1.927
ENSG00000183684	ALYREF	-1.2	7.5E-06	2.7E-05	138.540	2.294	136.229	140.816	166.502	9.665	157.526	176.734
ENSG00000183763	TRAIP	-1.2	5.1E-03	1.0E-02	10.321	0.869	9.412	11.143	12.354	0.975	11.430	13.373
ENSG00000183765	CHEK2	-1.2	4.5E-06	1.7E-05	39.066	1.251	37.621	39.798	46.874	1.063	45.647	47.501
ENSG00000183783	KCTD8	-1.2	7.4E-02	1.1E-01	4.034	0.603	3.422	4.627	4.891	0.447	4.546	5.396
ENSG00000184182	UBE2F	-1.2	1.5E-02	2.7E-02	8.091	0.463	7.633	8.559	9.422	0.676	8.990	10.201
ENSG00000184208	C22orf46	-1.2	6.8E-03	1.3E-02	7.328	1.057	6.473	8.510	8.766	0.570	8.108	9.115
ENSG00000184517	ZFP1	-1.2	1.9E-03	4.1E-03	6.473	0.397	6.156	6.918	8.113	0.791	7.614	9.025
ENSG00000185009	AP3M1	-1.2	2.4E-05	7.9E-05	34.667	1.099	33.653	35.835	40.233	0.546	39.822	40.853
ENSG00000185100	ADSSL1	-1.2	5.4E-03	1.1E-02	3.466	0.309	3.181	3.794	4.325	0.649	3.580	4.770
ENSG00000185122	HSF1	-1.2	1.2E-03	2.8E-03	19.073	0.329	18.862	19.453	22.006	1.436	20.504	23.366
ENSG00000185158	LRRC37B	-1.2	1.4E-02	2.6E-02	4.878	0.561	4.392	5.492	5.752	0.576	5.276	6.392
ENSG00000185215	TNFAIP2	-1.2	4.0E-03	8.4E-03	5.993	0.237	5.810	6.261	7.175	0.392	6.877	7.619
ENSG00000185252	ZNF74	-1.2	6.9E-05	2.1E-04	26.919	0.533	26.389	27.455	31.435	1.488	29.922	32.896
ENSG00000185344	ATP6V0A2	-1.2	1.1E-04	3.2E-04	11.156	0.471	10.647	11.575	13.083	0.236	12.811	13.230
ENSG00000185480	PARPBP	-1.2	3.1E-04	8.1E-04	31.514	2.246	29.048	33.442	36.621	1.547	35.089	38.182
ENSG00000185504	FAAP100	-1.2	1.3E-05	4.4E-05	14.093	0.444	13.673	14.558	17.626	0.961	16.832	18.694
ENSG00000185585	OLFML2A	-1.2	9.1E-04	2.2E-03	5.739	0.533	5.396	6.352	7.097	0.828	6.150	7.681
ENSG00000185722	ANKFY1	-1.2	7.5E-08	4.0E-07	12.758	0.906	11.762	13.534	15.944	0.197	15.765	16.155
ENSG00000185728	YTHDF3	-1.2	3.5E-07	1.7E-06	50.583	2.800	47.534	53.040	60.788	1.700	58.966	62.330
ENSG00000185869	ZNF829	-1.2	1.3E-02	2.4E-02	3.904	0.350	3.543	4.242	4.603	0.213	4.376	4.799
ENSG00000185883	ATP6V0C	-1.2	1.2E-01	1.7E-01	11.602	0.351	11.200	11.849	13.541	0.729	13.093	14.383
ENSG00000185963	BICD2	-1.2	3.0E-06	1.2E-05	22.021	0.712	21.349	22.767	26.675	0.312	26.403	27.016
ENSG00000186019		-1.2	6.0E-03	1.2E-02	6.614	0.877	5.747	7.501	8.257	0.285	7.975	8.545
ENSG00000186073	C15orf41	-1.2	5.1E-02	8.1E-02	4.585	0.353	4.180	4.830	5.302	0.382	4.884	5.634
ENSG00000186174	BCL9L	-1.2	2.8E-04	7.5E-04	14.727	0.332	14.527	15.111	17.513	1.338	16.157	18.833
ENSG00000186185	KIF18B	-1.2	1.3E-06	5.4E-06	26.280	1.472	24.636	27.477	32.698	0.764	31.864	33.364
ENSG00000186318	BACE1	-1.2	7.6E-03	1.5E-02	3.741	0.306	3.544	4.094	4.541	0.248	4.267	4.752
ENSG00000186395	KRT10	-1.2	1.2E-02	2.3E-02	13.999	0.236	13.728	14.158	16.839	1.519	15.181	18.164
ENSG00000186469	GNG2	-1.2	7.8E-03	1.5E-02	9.882	0.847	8.904	10.409	11.455	0.397	10.998	11.716
ENSG00000186501	TMEM222	-1.2	1.4E-02	2.6E-02	9.454	0.758	8.759	10.263	10.989	0.949	9.893	11.546
ENSG00000186648	CARMIL3	-1.2	4.5E-02	7.2E-02	2.229	0.336	2.021	2.617	2.685	0.192	2.552	2.905
ENSG00000186834	HEXIM1	-1.2	1.8E-03	4.0E-03	30.247	2.844	27.097	32.624	35.312	3.026	31.818	37.095
ENSG00000186854	TRABD2A	-1.2	1.4E-02	2.5E-02	1.822	0.044	1.783	1.869	2.275	0.140	2.192	2.437
ENSG00000186871	ERCC6L	-1.2	4.9E-06	1.9E-05	30.168	1.344	28.789	31.474	36.919	1.342	36.074	38.466
ENSG00000187514	PTMA	-1.2	5.7E-05	1.8E-04	1099.735	28.336	1071.677	1128.342	1284.763	22.102	1260.459	1303.659
ENSG00000187522	HSPA14	-1.2	1.9E-04	5.3E-04	25.792	1.170	24.444	26.542	30.613	1.364	29.041	31.483
ENSG00000187531	SIRT7	-1.2	4.0E-03	8.3E-03	3.731	0.342	3.337	3.952	4.597	0.032	4.571	4.633
ENSG00000187627	RGPD1	-1.2	1.3E-01	1.8E-01	1.111	0.108	0.987	1.181	1.318	0.011	1.307	1.329
ENSG00000187630	DHRS4L2	-1.2	7.6E-02	1.2E-01	6.461	0.635	5.914	7.157	7.663	0.783	6.760	8.167
ENSG00000187764	SEMA4D	-1.2	5.8E-05	1.8E-04	9.711	0.478	9.254	10.208	11.505	0.423	11.044	11.874
ENSG00000188171	ZNF626	-1.2	1.3E-01	1.8E-01	7.877	1.559	6.254	9.362	9.132	1.130	8.186	10.383
ENSG00000188352	FOCAD	-1.2	1.8E-06	7.6E-06	21.376	0.750	20.515	21.883	25.437	0.439	24.946	25.790
ENSG00000188368	PRR19	-1.2	1.3E-01	1.9E-01	2.713	0.221	2.540	2.963	3.267	0.393	2.948	3.707
ENSG00000188529	SRSF10	-1.2	9.6E-08	5.1E-07	58.267	3.142	54.886	61.097	70.358	2.003	68.592	72.535
ENSG00000188559	RALGAPA2	-1.2	5.8E-06	2.2E-05	11.922	0.383	11.482	12.185	14.441	0.139	14.281	14.533
ENSG00000188677	PARVB	-1.2	3.2E-03	6.7E-03	4.311	0.440	3.857	4.736	5.306	0.559	4.661	5.655
ENSG00000188766	SPRED3	-1.2	1.3E-01	1.8E-01	1.172	0.251	0.895	1.384	1.428	0.096	1.319	1.501
ENSG00000188786	MTF1	-1.2	1.3E-03	3.1E-03	5.168	0.097	5.057	5.237	6.274	0.386	5.829	6.500
ENSG00000188803	SHISA6	-1.2	5.8E-02	9.1E-02	1.044	0.307	0.760	1.371	1.298	0.080	1.215	1.376
ENSG00000188883	KLRG2	-1.2	7.6E-04	1.9E-03	19.021	1.557	17.362	20.451	23.490	1.290	22.063	24.573
ENSG00000189077	TMEM120A	-1.2	9.4E-03	1.8E-02	11.402	1.739	9.916	13.314	14.031	0.985	13.247	15.136
ENSG00000189184	PCDH18	-1.2	1.0E-04	3.0E-04	25.697	0.529	25.186	26.243	30.044	0.806	29.553	30.974
ENSG00000189241	TSPYL1	-1.2	1.7E-05	5.8E-05	62.648	0.211	62.437	62.858	72.945	1.852	71.057	74.758
ENSG00000189298	ZKSCAN3	-1.2	4.3E-02	7.0E-02	6.342	0.140	6.181	6.436	7.353	1.383	5.900	8.653
ENSG00000189339	SLC35E2B	-1.2	1.2E-06	5.0E-06	15.221	0.368	14.930	15.634	18.907	1.087	17.803	19.975
ENSG00000189403	HMGB1	-1.2	2.6E-08	1.6E-07	213.569	5.542	209.575	219.896	256.101	12.882	242.686	268.376
ENSG00000196141	SPATS2L	-1.2	6.7E-07	3.0E-06	37.542	0.527	37.170	38.145	45.469	0.589	44.890	46.068
ENSG00000196199	MPHOSPH8	-1.2	2.0E-04	5.4E-04	24.152	1.657	22.311	25.524	28.764	0.571	28.251	29.380
ENSG00000196227	FAM217B	-1.2	1.1E-03	2.5E-03	16.862	0.367	16.454	17.168	19.553	0.586	18.880	19.944
ENSG00000196268	ZNF493	-1.2	1.5E-04	4.3E-04	7.915	1.140	6.992	9.188	9.944	0.784	9.368	10.837
ENSG00000196367	TRRAP	-1.2	7.6E-06	2.8E-05	31.450	0.523	31.026	32.035	36.532	0.785	35.792	37.356
ENSG00000196378	ZNF34	-1.2	1.3E-01	1.8E-01	4.153	0.549	3.543	4.607	4.898	0.608	4.480	5.595
ENSG00000196422	PPP1R26	-1.2	4.9E-06	1.9E-05	22.851	0.822	22.087	23.720	28.064	1.097	26.994	29.186
ENSG00000196453	ZNF777	-1.2	3.0E-02	5.0E-02	6.963	1.292	5.523	8.021	8.469	0.716	7.820	9.237
ENSG00000196458	ZNF605	-1.2	1.4E-03	3.3E-03	6.542	0.302	6.292	6.877	7.657	0.064	7.604	7.728
ENSG00000196498	NCOR2	-1.2	3.9E-06	1.5E-05	23.057	0.929	22.078	23.927	28.770	2.181	26.608	30.971
ENSG00000196510	ANAPC7	-1.2	1.2E-05	4.3E-05	29.547	0.317	29.257	29.886	34.696	0.962	33.586	35.283
ENSG00000196547	MAN2A2	-1.2	1.3E-02	2.3E-02	3.329	0.120	3.194	3.424	3.873	0.246	3.589	4.034
ENSG00000196557	CACNA1H	-1.2	1.4E-04	4.0E-04	11.427	0.839	10.869	12.392	14.044	1.424	13.148	15.687
ENSG00000196584	XRCC2	-1.2	8.4E-05	2.5E-04	33.049	1.174	32.250	34.397	39.364	2.665	36.445	41.665
ENSG00000196591	HDAC2	-1.2	2.7E-07	1.3E-06	90.796	1.480	89.291	92.250	105.089	1.651	103.465	106.765
ENSG00000196605	ZNF846	-1.2	1.1E-01	1.6E-01	3.139	0.367	2.722	3.417	3.656	0.024	3.630	3.678
ENSG00000196656		-1.2	6.8E-05	2.1E-04	405.250	18.023	384.443	415.979	469.191	4.707	464.459	473.873
ENSG00000196705	ZNF431	-1.2	1.1E-05	3.9E-05	9.373	0.623	8.690	9.910	11.360	0.687	10.764	12.112
ENSG00000196715	VKORC1L1	-1.2	9.2E-05	2.7E-04	36.903	1.046	36.111	38.088	43.449	1.639	41.586	44.668
ENSG00000196810	CTBP1-AS2	-1.2	1.0E-02	1.9E-02	2.663	0.204	2.449	2.855	3.256	0.072	3.174	3.305
ENSG00000196862	RGPD4	-1.2	2.6E-02	4.4E-02	2.916	0.291	2.635	3.217	3.517	0.065	3.452	3.581
ENSG00000196876	SCN8A	-1.2	1.1E-04	3.3E-04	4.941	0.146	4.826	5.105	5.971	0.085	5.884	6.053
ENSG00000196935	SRGAP1	-1.2	5.3E-03	1.1E-02	1.337	0.170	1.191	1.523	1.596	0.099	1.536	1.710
ENSG00000196976	LAGE3	-1.2	1.8E-02	3.3E-02	45.953	5.173	40.010	49.443	57.151	7.760	49.163	64.661
ENSG00000197037	ZSCAN25	-1.2	1.2E-03	2.8E-03	5.642	0.366	5.309	6.033	6.908	0.380	6.481	7.207
ENSG00000197045	GMFB	-1.2	1.8E-06	7.6E-06	59.126	4.410	54.873	63.677	73.258	5.402	69.670	79.471
ENSG00000197181	PIWIL2	-1.2	8.3E-02	1.2E-01	2.168	0.252	1.879	2.343	2.607	0.422	2.225	3.060
ENSG00000197345	MRPL21	-1.2	2.6E-05	8.5E-05	35.116	1.864	33.219	36.946	42.828	3.107	40.660	46.387
ENSG00000197417	SHPK	-1.2	7.2E-02	1.1E-01	4.981	0.670	4.525	5.751	5.789	0.204	5.569	5.971
ENSG00000197579	TOPORS	-1.2	1.1E-06	4.9E-06	43.596	1.314	42.184	44.783	52.704	0.905	51.948	53.706
ENSG00000197603	C5orf42	-1.2	3.9E-04	1.0E-03	3.362	0.211	3.139	3.558	4.064	0.067	3.991	4.123
ENSG00000197712	FAM114A1	-1.2	1.1E-03	2.7E-03	7.303	0.495	6.781	7.765	8.913	0.115	8.804	9.034
ENSG00000197728	RPS26	-1.2	8.3E-05	2.5E-04	42.173	2.341	39.494	43.824	51.164	1.125	49.985	52.225
ENSG00000197774	EME2	-1.2	1.9E-02	3.4E-02	2.238	0.443	1.955	2.749	2.787	0.103	2.686	2.891
ENSG00000197841	ZNF181	-1.2	7.5E-04	1.8E-03	5.271	0.239	4.995	5.413	6.541	0.434	6.235	7.037
ENSG00000197858	GPAA1	-1.2	3.0E-04	7.9E-04	32.433	1.694	31.326	34.384	39.885	4.262	34.965	42.462
ENSG00000197892	KIF13B	-1.2	4.4E-03	9.0E-03	5.097	0.101	4.981	5.167	5.942	0.542	5.318	6.295
ENSG00000197969	VPS13A	-1.2	1.2E-04	3.5E-04	11.352	0.700	10.921	12.160	13.181	0.503	12.795	13.750
ENSG00000198026	ZNF335	-1.2	2.0E-02	3.5E-02	4.963	0.131	4.813	5.049	5.784	0.184	5.571	5.893
ENSG00000198121	LPAR1	-1.2	1.4E-01	2.0E-01	1.149	0.095	1.039	1.210	1.435	0.112	1.318	1.541
ENSG00000198130	HIBCH	-1.2	3.1E-04	8.2E-04	14.522	0.531	13.936	14.971	16.966	0.203	16.757	17.163
ENSG00000198131	ZNF544	-1.2	2.3E-05	7.7E-05	15.677	0.023	15.651	15.694	18.939	0.652	18.200	19.435
ENSG00000198162	MAN1A2	-1.2	8.0E-06	2.9E-05	12.660	0.555	12.023	13.030	15.318	0.756	14.557	16.068
ENSG00000198182	ZNF607	-1.2	2.1E-04	5.7E-04	10.761	0.775	9.867	11.219	13.406	0.555	12.960	14.028
ENSG00000198198	SZT2	-1.2	1.2E-02	2.1E-02	1.660	0.090	1.607	1.763	1.955	0.236	1.757	2.216
ENSG00000198286	CARD11	-1.2	6.0E-05	1.8E-04	26.252	0.948	25.254	27.139	31.113	0.911	30.478	32.157
ENSG00000198373	WWP2	-1.2	4.9E-06	1.9E-05	11.109	0.633	10.444	11.703	13.768	0.733	13.295	14.612
ENSG00000198478	SH3BGRL2	-1.2	6.2E-04	1.6E-03	23.495	1.119	22.557	24.733	27.265	0.779	26.376	27.828
ENSG00000198554	WDHD1	-1.2	9.5E-07	4.2E-06	49.119	1.643	47.844	50.974	59.239	2.596	56.318	61.279
ENSG00000198720	ANKRD13B	-1.2	5.6E-03	1.1E-02	10.325	0.271	10.106	10.628	12.283	0.797	11.362	12.746
ENSG00000198746	GPATCH3	-1.2	2.5E-02	4.4E-02	13.111	0.129	12.972	13.228	15.153	0.964	14.402	16.239
ENSG00000198795	ZNF521	-1.2	2.6E-03	5.5E-03	4.029	0.266	3.724	4.217	4.992	0.193	4.799	5.185
ENSG00000198805	PNP	-1.2	1.3E-07	6.8E-07	48.359	1.560	47.374	50.157	59.410	1.095	58.656	60.666
ENSG00000198874	TYW1	-1.2	1.7E-04	4.7E-04	17.741	0.510	17.201	18.214	21.261	0.425	20.928	21.739
ENSG00000198900	TOP1	-1.2	5.0E-07	2.3E-06	162.135	2.455	159.345	163.969	188.484	2.130	186.030	189.845
ENSG00000198908	BHLHB9	-1.2	3.7E-04	9.6E-04	12.701	0.553	12.111	13.208	15.210	0.188	15.000	15.364
ENSG00000198960	ARMCX6	-1.2	5.1E-04	1.3E-03	11.650	0.477	11.100	11.949	14.568	0.310	14.255	14.874
ENSG00000198963	RORB	-1.2	4.0E-02	6.6E-02	2.414	0.067	2.357	2.488	2.840	0.233	2.573	3.005
ENSG00000203668	CHML	-1.2	3.2E-06	1.3E-05	48.393	2.620	45.372	50.037	58.883	5.561	54.315	65.076
ENSG00000203761	MSTO2P	-1.2	2.1E-02	3.7E-02	11.774	1.167	10.430	12.516	14.134	0.842	13.165	14.693
ENSG00000203993	ARRDC1-AS1	-1.2	1.1E-02	2.1E-02	18.334	1.468	17.200	19.992	21.305	1.076	20.072	22.051
ENSG00000204104	TRAF3IP1	-1.2	3.7E-04	9.7E-04	10.858	0.528	10.263	11.271	13.441	0.687	12.789	14.177
ENSG00000204138	PHACTR4	-1.2	8.9E-06	3.2E-05	23.460	0.622	22.745	23.884	27.753	0.319	27.386	27.957
ENSG00000204147	ASAH2B	-1.2	1.5E-02	2.8E-02	4.422	0.244	4.141	4.577	5.325	0.258	5.041	5.543
ENSG00000204271	SPIN3	-1.2	2.5E-03	5.5E-03	2.485	0.312	2.134	2.729	2.999	0.222	2.753	3.185
ENSG00000204304	PBX2	-1.2	1.3E-05	4.7E-05	59.678	0.772	58.858	60.390	70.623	1.969	69.442	72.896
ENSG00000204316	MRPL38	-1.2	6.3E-02	9.8E-02	3.403	0.393	3.028	3.811	4.029	0.147	3.877	4.168
ENSG00000204394	VARS	-1.2	9.0E-08	4.8E-07	58.179	1.629	56.805	59.979	72.299	4.561	69.626	77.566
ENSG00000204519	ZNF551	-1.2	3.3E-05	1.1E-04	24.654	0.159	24.474	24.775	29.774	2.477	27.249	32.200
ENSG00000204576	PRR3	-1.2	3.5E-02	5.8E-02	6.607	0.431	6.180	7.041	7.639	0.466	7.192	8.122
ENSG00000204713	TRIM27	-1.2	7.0E-06	2.6E-05	32.239	0.879	31.342	33.099	37.773	1.110	36.802	38.983
ENSG00000204791	SMIM25	-1.2	1.2E-01	1.7E-01	3.128	0.515	2.601	3.631	3.854	0.629	3.375	4.566
ENSG00000204843	DCTN1	-1.2	1.7E-06	7.0E-06	40.346	0.307	40.143	40.699	47.857	1.033	46.755	48.803
ENSG00000204859	ZBTB48	-1.2	2.3E-03	5.0E-03	6.364	0.564	5.793	6.921	7.836	0.636	7.445	8.570
ENSG00000205181	LINC00654	-1.2	5.0E-02	8.0E-02	2.518	0.174	2.317	2.632	2.968	0.354	2.591	3.293
ENSG00000205208	C4orf46	-1.2	2.0E-04	5.5E-04	42.007	1.630	40.253	43.476	48.714	1.902	47.398	50.894
ENSG00000205250	E2F4	-1.2	2.7E-05	9.0E-05	47.122	1.774	46.006	49.167	55.488	1.258	54.753	56.951
ENSG00000205476	CCDC85C	-1.2	4.3E-04	1.1E-03	5.099	0.227	4.837	5.237	6.066	0.095	5.956	6.127
ENSG00000206149	HERC2P9	-1.2	5.1E-05	1.6E-04	11.873	0.942	10.786	12.446	14.335	0.642	13.622	14.866
ENSG00000206557	TRIM71	-1.2	1.5E-07	7.6E-07	207.863	8.312	200.556	216.907	245.615	7.133	237.382	249.927
ENSG00000206560	ANKRD28	-1.2	3.8E-07	1.8E-06	19.046	0.804	18.511	19.971	23.327	0.436	23.025	23.827
ENSG00000212719	C17orf51	-1.2	1.3E-02	2.5E-02	3.375	0.046	3.335	3.425	3.968	0.108	3.843	4.032
ENSG00000212916	MAP10	-1.2	6.4E-02	9.9E-02	3.675	0.702	2.912	4.293	4.411	0.273	4.132	4.678
ENSG00000212994	RPS26P6	-1.2	1.8E-03	4.0E-03	153.271	10.379	142.570	163.294	181.698	7.109	174.873	189.061
ENSG00000213070	HMGB3P6	-1.2	6.1E-02	9.4E-02	36.130	2.896	32.909	38.519	41.860	0.906	41.026	42.824
ENSG00000213246	SUPT4H1	-1.2	1.7E-07	8.4E-07	76.378	3.989	73.391	80.908	95.210	2.662	92.450	97.762
ENSG00000213337	ANKRD39	-1.2	3.2E-02	5.3E-02	2.918	0.349	2.519	3.167	3.551	0.282	3.244	3.800
ENSG00000213362	FTH1P12	-1.2	1.3E-01	1.9E-01	11.935	2.210	10.532	14.482	14.483	0.721	13.779	15.220
ENSG00000213383		-1.2	1.4E-01	2.0E-01	12.977	0.944	12.096	13.974	15.062	2.217	13.114	17.474
ENSG00000213463	SYNJ2BP	-1.2	1.1E-03	2.7E-03	18.463	1.430	17.386	20.085	21.358	0.954	20.448	22.351
ENSG00000213516	RBMXL1	-1.2	4.1E-07	1.9E-06	31.433	0.193	31.217	31.587	38.546	0.325	38.172	38.753
ENSG00000213563	C8orf82	-1.2	3.0E-02	5.1E-02	8.009	0.534	7.561	8.600	9.604	2.077	7.866	11.905
ENSG00000213707	HMGB1P10	-1.2	5.1E-04	1.3E-03	105.725	1.826	103.751	107.353	126.637	4.560	123.986	131.902
ENSG00000213930	GALT	-1.2	2.2E-02	3.9E-02	4.572	0.170	4.383	4.711	5.459	0.330	5.103	5.755
ENSG00000214182	PTMAP5	-1.2	1.1E-02	2.0E-02	481.834	31.545	455.585	516.829	563.829	25.977	535.142	585.762
ENSG00000214706	IFRD2	-1.2	2.0E-04	5.5E-04	35.758	0.386	35.394	36.163	41.686	2.785	39.212	44.707
ENSG00000214820	MPRIPP1	-1.2	6.1E-02	9.6E-02	9.642	0.541	9.213	10.249	11.702	0.727	10.945	12.394
ENSG00000215021	PHB2	-1.2	1.9E-06	7.9E-06	277.312	7.743	272.156	286.215	323.401	12.771	311.847	337.113
ENSG00000215146		-1.2	1.0E-04	2.9E-04	26.040	3.569	22.009	28.798	32.490	2.255	30.227	34.737
ENSG00000215154		-1.2	8.4E-02	1.3E-01	4.477	0.583	3.875	5.039	5.340	0.570	4.832	5.957
ENSG00000215199	YWHAZP6	-1.2	1.2E-01	1.7E-01	16.874	0.675	16.184	17.533	19.802	0.854	19.055	20.733
ENSG00000215271	HOMEZ	-1.2	1.5E-03	3.5E-03	4.671	0.352	4.267	4.907	5.855	0.488	5.351	6.326
ENSG00000215788	TNFRSF25	-1.2	1.3E-01	1.9E-01	3.187	0.194	2.976	3.356	3.670	0.439	3.230	4.107
ENSG00000215866	LINC01356	-1.2	3.3E-02	5.6E-02	20.285	2.173	18.017	22.349	23.408	2.333	21.094	25.759
ENSG00000219682		-1.2	4.2E-02	6.8E-02	18.878	0.234	18.673	19.133	21.821	1.726	19.924	23.299
ENSG00000221829	FANCG	-1.2	5.5E-06	2.1E-05	31.970	1.132	31.199	33.270	39.284	0.963	38.459	40.342
ENSG00000221886	ZBED8	-1.2	3.6E-02	5.9E-02	8.628	0.514	8.102	9.129	10.027	1.162	8.811	11.127
ENSG00000221909	FAM200A	-1.2	2.9E-02	4.9E-02	11.197	0.924	10.510	12.247	12.952	0.888	12.339	13.971
ENSG00000223546	LINC00630	-1.2	2.9E-02	4.9E-02	3.053	0.467	2.548	3.470	3.800	0.454	3.276	4.077
ENSG00000223612		-1.2	1.1E-02	2.1E-02	47.366	6.492	39.876	51.368	57.764	4.546	52.530	60.726
ENSG00000223703		-1.2	1.4E-01	2.0E-01	2.197	0.616	1.514	2.711	2.734	0.668	1.964	3.155
ENSG00000223756	TSSC2	-1.2	1.5E-05	5.1E-05	74.474	1.865	72.559	76.284	88.124	5.212	82.113	91.373
ENSG00000224470	ATXN1L	-1.2	2.0E-05	6.8E-05	18.697	0.912	17.654	19.345	22.073	0.272	21.883	22.384
ENSG00000224531	SMIM13	-1.2	2.5E-03	5.4E-03	10.415	1.026	9.252	11.192	12.378	0.350	11.996	12.682
ENSG00000225022	UBE2D3P1	-1.2	5.6E-03	1.1E-02	127.269	1.964	125.054	128.798	146.852	8.682	138.272	155.633
ENSG00000225614	ZNF469	-1.2	1.1E-01	1.6E-01	0.597	0.160	0.430	0.749	0.735	0.074	0.688	0.820
ENSG00000226259	GTF2H2B	-1.2	2.6E-04	7.1E-04	13.747	0.221	13.493	13.895	16.889	0.572	16.341	17.483
ENSG00000226478	UPF3AP1	-1.2	3.3E-02	5.6E-02	15.686	1.116	14.584	16.815	18.835	2.979	15.412	20.830
ENSG00000226544	RPL7P22	-1.2	2.3E-02	4.0E-02	18.597	0.691	17.943	19.320	23.055	2.524	20.524	25.573
ENSG00000226887	ERVMER34-1	-1.2	8.9E-05	2.6E-04	29.283	1.057	28.179	30.287	34.952	1.283	34.023	36.415
ENSG00000227344	HAUS6P1	-1.2	3.6E-03	7.5E-03	28.937	1.497	28.063	30.665	34.222	2.191	31.695	35.575
ENSG00000228283	KATNBL1P6	-1.2	7.9E-02	1.2E-01	8.824	0.797	8.083	9.650	10.569	1.172	9.488	11.815
ENSG00000228624	HDAC2-AS2	-1.2	7.9E-02	1.2E-01	2.496	0.242	2.259	2.743	2.878	0.294	2.542	3.092
ENSG00000228709		-1.2	1.2E-01	1.7E-01	11.783	0.589	11.381	12.459	13.644	0.916	12.866	14.653
ENSG00000229419	RALGAPA1P1	-1.2	3.0E-04	8.0E-04	8.463	0.266	8.157	8.631	10.337	0.342	9.978	10.659
ENSG00000230030	HMGN2P3	-1.2	3.7E-03	7.7E-03	331.755	19.340	309.863	346.522	389.218	23.426	366.889	413.605
ENSG00000232368	FTLP2	-1.2	1.3E-01	1.8E-01	12.643	1.053	11.534	13.628	15.377	3.827	11.362	18.982
ENSG00000232380	ZDHHC20P4	-1.2	4.0E-02	6.5E-02	10.447	1.923	8.226	11.606	13.053	1.839	11.398	15.032
ENSG00000232677	LINC00665	-1.2	1.4E-06	6.0E-06	32.640	1.116	31.749	33.891	39.422	1.415	38.545	41.054
ENSG00000233016	SNHG7	-1.2	4.9E-03	1.0E-02	15.424	0.692	14.628	15.879	18.194	0.543	17.651	18.736
ENSG00000233170		-1.2	1.0E-01	1.5E-01	8.766	1.523	7.521	10.464	10.874	1.581	9.609	12.647
ENSG00000233225		-1.2	6.5E-04	1.6E-03	56.453	4.262	53.511	61.340	67.994	5.940	61.144	71.725
ENSG00000233393		-1.2	7.8E-04	1.9E-03	20.965	0.809	20.081	21.669	25.445	0.768	24.816	26.301
ENSG00000233723	LINC01122	-1.2	1.3E-01	1.8E-01	0.992	0.222	0.745	1.173	1.228	0.120	1.117	1.355
ENSG00000234297		-1.2	2.6E-04	6.9E-04	52.872	2.997	49.667	55.604	64.844	1.986	62.551	66.059
ENSG00000234444	ZNF736	-1.2	7.5E-03	1.5E-02	5.611	0.453	5.217	6.105	6.497	0.214	6.253	6.651
ENSG00000236675	MTX1P1	-1.2	5.2E-02	8.2E-02	22.142	2.443	20.290	24.911	25.928	1.933	23.809	27.595
ENSG00000237172	B3GNT9	-1.2	1.1E-01	1.6E-01	1.923	0.167	1.804	2.115	2.347	0.185	2.155	2.524
ENSG00000237943	PRKCQ-AS1	-1.2	2.6E-02	4.4E-02	6.210	0.259	5.922	6.425	7.266	0.077	7.178	7.323
ENSG00000237945	LINC00649	-1.2	2.8E-02	4.8E-02	1.028	0.041	0.998	1.074	1.266	0.096	1.157	1.336
ENSG00000237975	FLG-AS1	-1.2	1.3E-02	2.4E-02	1.655	0.043	1.606	1.682	2.070	0.057	2.011	2.125
ENSG00000238105	GOLGA2P5	-1.2	2.1E-02	3.7E-02	2.382	0.213	2.155	2.578	2.824	0.145	2.707	2.987
ENSG00000239389	PCDHA13	-1.2	1.1E-01	1.6E-01	1.531	0.208	1.328	1.743	1.869	0.302	1.694	2.217
ENSG00000239713	APOBEC3G	-1.2	1.2E-01	1.7E-01	1.106	0.205	0.881	1.279	1.370	0.324	1.147	1.742
ENSG00000239900	ADSL	-1.2	5.6E-03	1.1E-02	9.525	0.563	8.878	9.907	10.988	0.648	10.518	11.727
ENSG00000240184	PCDHGC3	-1.2	4.7E-02	7.5E-02	3.045	0.138	2.906	3.182	3.670	0.363	3.266	3.966
ENSG00000240225	ZNF542P	-1.2	3.1E-05	1.0E-04	18.772	2.061	16.850	20.949	23.334	0.521	22.853	23.887
ENSG00000241472	PTPRG-AS1	-1.2	4.6E-02	7.5E-02	1.705	0.116	1.589	1.820	2.131	0.065	2.093	2.206
ENSG00000242457	RBBP4P2	-1.2	1.2E-02	2.2E-02	15.793	0.335	15.448	16.116	19.303	2.552	17.276	22.170
ENSG00000242970		-1.2	3.0E-02	5.1E-02	73.629	6.635	66.273	79.163	87.622	12.859	77.889	102.199
ENSG00000244171	PRX2P1	-1.2	6.4E-04	1.6E-03	36.432	2.234	33.867	37.960	44.326	0.583	43.782	44.941
ENSG00000245680	ZNF585B	-1.2	1.9E-02	3.3E-02	4.545	0.087	4.470	4.640	5.303	0.447	5.026	5.819
ENSG00000246089		-1.2	4.1E-02	6.7E-02	5.919	0.290	5.718	6.252	7.030	0.868	6.094	7.808
ENSG00000247077	PGAM5	-1.2	5.5E-06	2.1E-05	45.076	0.965	43.968	45.729	53.920	1.589	52.930	55.753
ENSG00000247315	ZCCHC3	-1.2	3.7E-07	1.7E-06	67.783	3.680	63.931	71.263	82.960	3.422	79.548	86.393
ENSG00000247516	MIR4458HG	-1.2	1.4E-02	2.5E-02	4.176	0.175	4.055	4.377	5.166	0.690	4.683	5.956
ENSG00000249853	HS3ST5	-1.2	5.8E-02	9.1E-02	3.487	0.291	3.222	3.799	4.189	0.633	3.778	4.918
ENSG00000249992	TMEM158	-1.2	7.4E-02	1.1E-01	11.551	0.855	10.565	12.089	13.456	0.396	13.098	13.881
ENSG00000251247	ZNF345	-1.2	3.6E-02	5.9E-02	4.419	0.456	3.901	4.762	5.227	0.357	4.955	5.631
ENSG00000251333	RTN3P1	-1.2	4.1E-03	8.5E-03	48.415	3.127	44.937	50.995	58.110	3.862	53.686	60.807
ENSG00000251593	MSNP1	-1.2	8.0E-02	1.2E-01	9.847	0.390	9.400	10.115	11.406	0.907	10.877	12.453
ENSG00000251669	FAM86EP	-1.2	1.4E-01	1.9E-01	3.837	0.380	3.538	4.265	4.415	0.349	4.119	4.799
ENSG00000253230	LINC00599	-1.2	4.0E-02	6.5E-02	11.047	0.548	10.524	11.617	12.804	0.832	12.169	13.746
ENSG00000253797	UTP14C	-1.2	7.6E-06	2.8E-05	18.905	1.059	17.717	19.750	23.347	1.361	22.283	24.882
ENSG00000254004	ZNF260	-1.2	2.6E-05	8.6E-05	27.237	1.376	26.088	28.762	32.931	3.295	30.837	36.729
ENSG00000254093	PINX1	-1.2	2.1E-03	4.7E-03	15.976	0.394	15.534	16.289	19.257	0.606	18.565	19.692
ENSG00000254454	RCC2P6	-1.2	1.2E-01	1.7E-01	5.682	1.132	4.566	6.828	6.778	1.355	5.779	8.320
ENSG00000254470	AP5B1	-1.2	5.9E-03	1.2E-02	5.263	0.258	4.973	5.466	6.449	0.654	5.894	7.171
ENSG00000254719		-1.2	5.9E-02	9.2E-02	88.446	5.725	82.373	93.745	103.000	7.953	95.060	110.967
ENSG00000255725	TDGP1	-1.2	6.6E-02	1.0E-01	19.426	1.404	17.830	20.471	22.496	2.125	20.610	24.798
ENSG00000256229	ZNF486	-1.2	1.9E-03	4.3E-03	13.907	1.240	12.648	15.127	16.576	0.939	15.681	17.517
ENSG00000256294	ZNF225	-1.2	3.1E-02	5.3E-02	3.211	0.071	3.129	3.257	3.790	0.439	3.378	4.251
ENSG00000256304	CCDC150P1	-1.2	2.5E-02	4.4E-02	10.099	0.643	9.372	10.592	12.116	1.428	10.469	13.012
ENSG00000257093	KIAA1147	-1.2	2.1E-08	1.3E-07	29.442	1.554	27.739	30.782	36.830	0.762	35.963	37.391
ENSG00000257267	ZNF271P	-1.2	2.5E-05	8.2E-05	11.809	0.277	11.635	12.129	14.316	0.256	14.057	14.569
ENSG00000257727	CNPY2	-1.2	1.4E-02	2.6E-02	13.785	0.374	13.524	14.214	16.038	1.852	13.931	17.408
ENSG00000259431	THTPA	-1.2	9.7E-03	1.8E-02	6.452	0.539	5.867	6.928	7.899	0.631	7.239	8.495
ENSG00000259781	HMGB1P6	-1.2	7.1E-07	3.2E-06	645.195	7.914	639.100	654.139	765.771	18.177	754.055	786.711
ENSG00000259790	ANP32BP1	-1.2	2.0E-02	3.6E-02	10.926	0.947	10.294	12.014	13.386	0.672	12.870	14.146
ENSG00000260007		-1.2	1.1E-01	1.6E-01	1.108	0.140	0.976	1.255	1.330	0.124	1.202	1.449
ENSG00000261115	TMEM178B	-1.2	2.1E-04	5.7E-04	7.448	0.071	7.388	7.526	9.163	0.420	8.822	9.631
ENSG00000261490		-1.2	1.3E-01	1.9E-01	1.421	0.217	1.295	1.671	1.767	0.107	1.671	1.883
ENSG00000261512		-1.2	1.1E-02	2.0E-02	11.085	0.443	10.590	11.444	13.615	0.959	12.946	14.714
ENSG00000261801	LOXL1-AS1	-1.2	6.3E-02	9.8E-02	4.441	0.208	4.211	4.615	5.155	0.151	5.063	5.329
ENSG00000262919	FAM58A	-1.2	9.8E-03	1.9E-02	18.397	0.565	18.022	19.047	21.538	0.953	20.509	22.389
ENSG00000263002	ZNF234	-1.2	3.2E-03	6.8E-03	5.332	0.167	5.141	5.451	6.552	0.532	6.085	7.132
ENSG00000263606		-1.2	6.8E-02	1.0E-01	12.476	1.164	11.378	13.697	14.681	1.197	13.367	15.711
ENSG00000264364	DYNLL2	-1.2	8.5E-09	5.6E-08	64.899	1.294	63.707	66.276	81.006	2.461	78.611	83.529
ENSG00000265808	SEC22B	-1.2	1.1E-05	3.8E-05	9.876	0.180	9.699	10.059	12.233	0.581	11.567	12.640
ENSG00000266028	SRGAP2	-1.2	1.6E-06	6.8E-06	7.872	0.247	7.657	8.142	9.713	0.148	9.597	9.880
ENSG00000267325	LINC01415	-1.2	1.1E-01	1.6E-01	1.446	0.107	1.335	1.548	1.771	0.191	1.550	1.892
ENSG00000267383		-1.2	1.4E-03	3.3E-03	9.281	0.146	9.187	9.450	11.419	0.346	11.205	11.818
ENSG00000267422		-1.2	2.4E-02	4.1E-02	20.793	0.420	20.347	21.180	24.544	1.624	22.902	26.148
ENSG00000268034		-1.2	1.6E-04	4.6E-04	103.865	6.389	97.494	110.272	126.955	7.228	120.801	134.914
ENSG00000268858		-1.2	6.2E-02	9.7E-02	2.712	0.321	2.404	3.045	3.364	0.171	3.193	3.535
ENSG00000269067	ZNF728	-1.2	2.5E-02	4.3E-02	8.432	1.095	7.288	9.470	10.221	0.780	9.421	10.979
ENSG00000269153	LYPLA2P2	-1.2	6.8E-02	1.0E-01	18.148	1.770	17.125	20.192	21.854	1.962	19.734	23.605
ENSG00000269343	ZNF587B	-1.2	1.1E-04	3.1E-04	17.915	0.713	17.226	18.650	21.712	1.136	20.683	22.931
ENSG00000269825		-1.2	1.1E-01	1.6E-01	3.355	0.421	2.996	3.819	4.108	0.392	3.801	4.549
ENSG00000270885	RASL10B	-1.2	3.9E-04	1.0E-03	16.732	0.773	16.223	17.621	20.545	1.977	19.226	22.818
ENSG00000271976		-1.2	4.4E-02	7.2E-02	3.375	0.427	2.988	3.833	4.208	0.498	3.647	4.597
ENSG00000274180	NATD1	-1.2	1.1E-03	2.7E-03	8.545	1.247	7.584	9.954	10.567	0.245	10.368	10.840
ENSG00000274211	SOCS7	-1.2	1.7E-04	4.9E-04	18.754	0.818	17.886	19.511	21.992	0.201	21.793	22.195
ENSG00000275066	SYNRG	-1.2	1.8E-05	6.1E-05	9.965	0.193	9.820	10.184	11.821	0.332	11.443	12.063
ENSG00000275591	XKR5	-1.2	1.0E-01	1.5E-01	2.708	0.270	2.402	2.915	3.118	0.137	2.962	3.221
ENSG00000277013		-1.2	1.2E-01	1.7E-01	4.844	0.284	4.670	5.172	5.738	0.406	5.273	6.016
ENSG00000277103		-1.2	1.4E-02	2.5E-02	18.504	2.072	16.957	20.858	22.885	0.282	22.642	23.194
ENSG00000277161	PIGW	-1.2	3.3E-03	6.9E-03	27.531	1.060	26.635	28.702	31.902	1.633	30.037	33.080
ENSG00000277258	PCGF2	-1.2	3.5E-05	1.1E-04	24.792	0.767	24.056	25.586	30.047	1.335	28.725	31.394
ENSG00000279392		-1.2	9.6E-04	2.3E-03	141.304	7.710	133.172	148.507	169.146	6.519	165.274	176.672
ENSG00000280071		-1.2	1.1E-02	2.0E-02	5.706	0.601	5.160	6.350	7.090	0.731	6.420	7.870
ENSG00000280789	PAGR1	-1.2	5.3E-02	8.4E-02	10.298	1.223	8.887	11.063	12.094	1.037	10.998	13.059
ENSG00000281103	TRG-AS1	-1.2	1.1E-01	1.6E-01	1.758	0.242	1.607	2.037	2.156	0.081	2.106	2.249
ENSG00000283544		-1.2	1.1E-01	1.6E-01	6.243	0.359	5.833	6.504	7.752	0.362	7.418	8.137
ENSG00000283674		-1.2	1.1E-01	1.6E-01	2.112	0.378	1.863	2.546	2.479	0.341	2.115	2.792
ENSG00000000003	TSPAN6	-1.1	4.7E-03	9.6E-03	57.855	2.343	55.228	59.728	63.873	2.616	62.062	66.872
ENSG00000000460	C1orf112	-1.1	3.1E-03	6.6E-03	18.507	0.290	18.226	18.805	20.943	0.627	20.270	21.512
ENSG00000001629	ANKIB1	-1.1	2.1E-03	4.6E-03	31.732	0.216	31.485	31.887	35.421	2.002	33.201	37.090
ENSG00000001631	KRIT1	-1.1	3.7E-03	7.8E-03	16.759	0.779	15.972	17.530	18.961	0.686	18.175	19.499
ENSG00000004142	POLDIP2	-1.1	1.7E-04	4.7E-04	102.070	0.863	101.402	103.045	116.793	4.646	112.817	121.900
ENSG00000004766	VPS50	-1.1	1.1E-01	1.6E-01	4.640	0.717	3.820	5.151	5.137	0.442	4.774	5.629
ENSG00000004864	SLC25A13	-1.1	3.5E-03	7.4E-03	88.266	1.869	86.279	89.988	96.954	3.024	93.791	99.817
ENSG00000005206	SPPL2B	-1.1	1.3E-01	1.8E-01	8.058	0.225	7.877	8.309	8.774	0.936	8.150	9.849
ENSG00000005339	CREBBP	-1.1	5.7E-04	1.4E-03	13.942	0.469	13.649	14.483	15.729	0.181	15.537	15.897
ENSG00000005486	RHBDD2	-1.1	7.7E-02	1.2E-01	23.292	1.234	22.005	24.465	25.870	1.880	24.149	27.877
ENSG00000005810	MYCBP2	-1.1	3.9E-02	6.3E-02	16.544	0.859	15.827	17.496	17.827	0.591	17.190	18.358
ENSG00000005893	LAMP2	-1.1	1.0E-01	1.5E-01	29.082	0.778	28.199	29.663	31.082	2.213	28.526	32.362
ENSG00000006607	FARP2	-1.1	9.4E-04	2.2E-03	7.338	0.161	7.239	7.523	8.395	0.038	8.364	8.438
ENSG00000006740	ARHGAP44	-1.1	1.1E-01	1.6E-01	7.379	0.182	7.170	7.500	7.977	0.587	7.300	8.316
ENSG00000007202	KIAA0100	-1.1	3.5E-05	1.1E-04	67.012	1.321	66.035	68.515	76.753	0.223	76.530	76.976
ENSG00000009335	UBE3C	-1.1	2.0E-04	5.4E-04	31.398	0.269	31.108	31.640	35.427	0.444	35.148	35.939
ENSG00000009844	VTA1	-1.1	2.8E-02	4.7E-02	36.979	1.577	35.686	38.736	39.906	1.855	37.780	41.198
ENSG00000010270	STARD3NL	-1.1	4.8E-03	9.8E-03	30.400	0.481	30.118	30.955	33.966	0.897	32.930	34.498
ENSG00000011258	MBTD1	-1.1	6.2E-04	1.6E-03	57.672	2.090	56.037	60.026	64.120	2.337	61.468	65.877
ENSG00000011304	PTBP1	-1.1	5.4E-05	1.7E-04	191.859	3.752	187.605	194.698	217.812	10.277	209.542	229.318
ENSG00000013275	PSMC4	-1.1	9.8E-04	2.3E-03	120.646	1.902	119.002	122.729	133.974	2.853	130.969	136.646
ENSG00000013306	SLC25A39	-1.1	1.4E-02	2.5E-02	108.490	0.472	107.951	108.827	117.871	8.174	108.748	124.528
ENSG00000013374	NUB1	-1.1	3.0E-03	6.4E-03	24.738	1.035	24.058	25.929	27.820	0.478	27.416	28.347
ENSG00000013375	PGM3	-1.1	6.4E-04	1.6E-03	28.365	0.692	27.640	29.018	32.131	0.794	31.260	32.815
ENSG00000013523	ANGEL1	-1.1	7.9E-02	1.2E-01	19.431	0.575	18.828	19.974	20.851	0.428	20.407	21.260
ENSG00000017797	RALBP1	-1.1	7.5E-04	1.8E-03	39.968	0.883	39.340	40.978	44.389	0.903	43.437	45.234
ENSG00000018189	RUFY3	-1.1	2.0E-02	3.5E-02	13.032	0.422	12.595	13.436	14.336	0.738	13.619	15.094
ENSG00000018236	CNTN1	-1.1	1.2E-01	1.7E-01	5.314	0.590	4.853	5.978	5.931	0.307	5.593	6.193
ENSG00000018699	TTC27	-1.1	1.1E-02	2.1E-02	38.060	1.747	37.051	40.077	42.497	0.395	42.043	42.762
ENSG00000019582	CD74	-1.1	1.5E-02	2.7E-02	19.808	0.598	19.117	20.166	22.370	0.363	21.980	22.698
ENSG00000021776	AQR	-1.1	3.8E-05	1.2E-04	31.963	0.577	31.305	32.380	36.511	1.681	35.220	38.411
ENSG00000022277	RTFDC1	-1.1	4.4E-03	9.0E-03	31.264	0.865	30.562	32.231	35.002	0.631	34.502	35.710
ENSG00000022840	RNF10	-1.1	5.9E-02	9.3E-02	58.886	0.640	58.260	59.539	63.193	3.689	59.080	66.209
ENSG00000023734	STRAP	-1.1	4.7E-05	1.5E-04	352.208	10.634	339.982	359.310	397.434	9.932	386.668	406.239
ENSG00000025156	HSF2	-1.1	2.6E-02	4.5E-02	27.778	1.937	25.892	29.762	31.211	1.053	30.154	32.260
ENSG00000025800	KPNA6	-1.1	2.3E-02	3.9E-02	49.688	0.812	49.049	50.602	53.357	1.724	51.375	54.508
ENSG00000028116	VRK2	-1.1	8.7E-03	1.7E-02	22.252	2.371	19.530	23.867	25.475	1.079	24.240	26.231
ENSG00000028310	BRD9	-1.1	1.1E-01	1.5E-01	13.801	0.483	13.475	14.356	14.686	0.477	14.379	15.236
ENSG00000028839	TBPL1	-1.1	1.1E-02	2.1E-02	23.108	1.114	21.840	23.928	26.085	1.037	25.140	27.195
ENSG00000030066	NUP160	-1.1	1.9E-04	5.2E-04	86.351	0.923	85.766	87.415	96.825	2.035	95.612	99.174
ENSG00000035928	RFC1	-1.1	2.1E-04	5.8E-04	68.815	1.385	67.216	69.648	76.783	0.577	76.396	77.446
ENSG00000036257	CUL3	-1.1	6.3E-05	1.9E-04	32.651	0.508	32.131	33.146	36.948	0.981	36.292	38.076
ENSG00000039123	SKIV2L2	-1.1	1.1E-02	2.1E-02	81.359	1.759	79.358	82.665	87.456	2.366	85.491	90.082
ENSG00000039319	ZFYVE16	-1.1	7.4E-02	1.1E-01	11.133	0.311	10.792	11.401	12.025	0.606	11.672	12.724
ENSG00000039560	RAI14	-1.1	3.5E-02	5.8E-02	69.434	3.317	65.639	71.778	73.850	0.235	73.592	74.051
ENSG00000047249	ATP6V1H	-1.1	6.7E-02	1.0E-01	14.695	0.380	14.442	15.132	15.980	0.319	15.619	16.219
ENSG00000047578	KIAA0556	-1.1	4.5E-02	7.3E-02	5.493	0.272	5.232	5.774	6.121	0.167	5.963	6.296
ENSG00000048707	VPS13D	-1.1	1.0E-02	1.9E-02	9.602	0.519	9.181	10.182	10.555	0.479	10.144	11.080

ENSG00000048828	FAM120A	-1.1	2.1E-04	5.7E-04	34.940	1.139	33.724	35.981	39.511	0.392	39.075	39.832
ENSG00000048991	R3HDM1	-1.1	1.1E-03	2.6E-03	28.610	0.859	28.033	29.597	31.965	0.616	31.270	32.442
ENSG00000049618	ARID1B	-1.1	2.8E-02	4.7E-02	4.744	0.151	4.603	4.904	5.104	0.036	5.073	5.143
ENSG00000050165	DKK3	-1.1	1.2E-01	1.7E-01	12.772	0.463	12.449	13.302	13.804	0.677	13.100	14.451
ENSG00000050344	NFE2L3	-1.1	1.4E-03	3.2E-03	147.804	2.447	146.014	150.592	162.409	2.967	159.090	164.807
ENSG00000050555	LAMC3	-1.1	9.9E-02	1.4E-01	9.819	0.311	9.492	10.111	10.640	0.268	10.338	10.846
ENSG00000050748	MAPK9	-1.1	8.1E-02	1.2E-01	19.464	0.080	19.373	19.522	20.652	0.438	20.253	21.121
ENSG00000051523	CYBA	-1.1	6.7E-03	1.3E-02	43.088	1.808	41.105	44.643	48.108	2.927	44.732	49.922
ENSG00000052344	PRSS8	-1.1	1.7E-04	4.6E-04	89.282	1.993	88.116	91.584	101.595	5.035	96.111	106.009
ENSG00000053254	FOXN3	-1.1	3.9E-04	1.0E-03	48.485	1.498	46.765	49.507	54.170	1.389	52.590	55.194
ENSG00000053770	AP5M1	-1.1	1.0E-03	2.4E-03	11.919	0.724	11.457	12.753	13.748	0.259	13.451	13.922
ENSG00000054267	ARID4B	-1.1	7.0E-02	1.1E-01	11.698	0.790	10.789	12.212	12.580	0.683	11.822	13.183
ENSG00000056050	HPF1	-1.1	3.5E-02	5.8E-02	77.190	2.534	74.595	79.658	84.212	2.064	81.855	85.700
ENSG00000056586	RC3H2	-1.1	4.0E-02	6.6E-02	15.527	0.543	14.987	16.072	16.836	0.745	16.233	17.668
ENSG00000056972	TRAF3IP2	-1.1	3.2E-02	5.3E-02	7.482	0.198	7.282	7.679	8.390	0.146	8.228	8.511
ENSG00000058799	YIPF1	-1.1	1.1E-02	2.1E-02	31.155	3.192	27.497	33.373	35.718	1.962	33.630	37.523
ENSG00000059769	DNAJC25	-1.1	1.3E-01	1.8E-01	12.571	1.001	11.579	13.581	13.859	0.649	13.183	14.477
ENSG00000060688	SNRNP40	-1.1	1.4E-02	2.5E-02	65.500	0.746	64.846	66.312	70.983	2.791	68.476	73.991
ENSG00000062598	ELMO2	-1.1	3.2E-03	6.9E-03	11.316	0.405	10.901	11.710	12.951	0.222	12.752	13.190
ENSG00000062822	POLD1	-1.1	1.8E-03	4.0E-03	39.472	0.675	38.722	40.033	44.705	2.359	41.981	46.118
ENSG00000063244	U2AF2	-1.1	2.0E-05	6.6E-05	118.035	3.584	113.914	120.428	135.548	6.997	130.149	143.454
ENSG00000063587	ZNF275	-1.1	6.5E-03	1.3E-02	7.538	0.108	7.459	7.661	8.621	0.111	8.496	8.706
ENSG00000064313	TAF2	-1.1	6.3E-03	1.2E-02	24.690	0.561	24.099	25.214	27.185	0.744	26.393	27.868
ENSG00000064601	CTSA	-1.1	1.4E-02	2.6E-02	8.409	0.297	8.066	8.595	9.571	0.098	9.459	9.640
ENSG00000064763	FAR2	-1.1	1.0E-02	1.9E-02	22.428	1.315	21.536	23.938	25.065	1.632	23.774	26.900
ENSG00000064933	PMS1	-1.1	6.6E-02	1.0E-01	38.276	1.201	36.903	39.131	40.937	2.346	38.928	43.515
ENSG00000064995	TAF11	-1.1	1.4E-03	3.1E-03	82.428	2.032	80.441	84.501	93.742	2.829	92.008	97.007
ENSG00000065060	UHRF1BP1	-1.1	2.1E-03	4.6E-03	19.000	0.501	18.455	19.442	21.433	0.219	21.204	21.639
ENSG00000065526	SPEN	-1.1	7.4E-02	1.1E-01	18.159	0.570	17.799	18.816	19.381	1.118	18.497	20.638
ENSG00000065559	MAP2K4	-1.1	3.4E-03	7.2E-03	28.156	1.003	27.030	28.952	31.544	1.298	30.326	32.909
ENSG00000065600	TMEM206	-1.1	1.9E-02	3.4E-02	15.275	0.266	14.990	15.517	17.613	0.941	16.538	18.286
ENSG00000065883	CDK13	-1.1	5.3E-03	1.1E-02	21.283	0.432	20.831	21.692	23.394	0.156	23.261	23.566
ENSG00000066117	SMARCD1	-1.1	1.5E-02	2.7E-02	92.013	1.224	90.608	92.853	98.399	1.882	96.634	100.380
ENSG00000066583	ISOC1	-1.1	4.2E-02	6.8E-02	56.942	2.657	54.603	59.830	61.854	0.621	61.132	62.247
ENSG00000066654	THUMPD1	-1.1	3.6E-03	7.5E-03	51.035	1.999	49.621	53.323	55.978	0.970	54.872	56.683
ENSG00000066697	MSANTD3	-1.1	1.7E-02	3.0E-02	23.521	0.715	22.816	24.246	26.201	0.566	25.571	26.666
ENSG00000067900	ROCK1	-1.1	1.1E-02	2.1E-02	34.122	1.709	32.396	35.815	36.935	1.201	35.588	37.894
ENSG00000067955	CBFB	-1.1	3.6E-02	5.9E-02	27.430	0.802	26.732	28.307	29.892	1.178	28.996	31.226
ENSG00000068024	HDAC4	-1.1	4.2E-02	6.8E-02	4.722	0.011	4.711	4.733	5.215	0.183	5.058	5.416
ENSG00000068120	COASY	-1.1	4.7E-03	9.6E-03	36.126	0.433	35.688	36.554	40.393	1.845	38.283	41.704
ENSG00000068438	FTSJ1	-1.1	2.9E-02	4.9E-02	34.031	0.583	33.515	34.664	37.362	0.992	36.576	38.477
ENSG00000068697	LAPTM4A	-1.1	2.5E-04	6.7E-04	337.466	6.390	331.145	343.923	374.663	7.039	367.533	381.608
ENSG00000068796	KIF2A	-1.1	1.8E-02	3.2E-02	61.874	1.931	60.419	64.065	67.010	1.416	65.553	68.382
ENSG00000068878	PSME4	-1.1	1.0E-02	1.9E-02	63.635	1.150	62.451	64.747	69.010	2.140	67.573	71.470
ENSG00000069248	NUP133	-1.1	3.4E-05	1.1E-04	60.434	0.912	59.855	61.486	68.818	0.634	68.226	69.487
ENSG00000070087	PFN2	-1.1	4.8E-05	1.5E-04	186.623	1.685	184.815	188.150	213.160	1.463	211.725	214.649
ENSG00000070214	SLC44A1	-1.1	2.1E-04	5.9E-04	32.504	0.887	31.841	33.512	36.441	0.415	36.040	36.869
ENSG00000070610	GBA2	-1.1	2.0E-04	5.4E-04	47.987	1.076	46.863	49.007	54.752	1.832	52.641	55.938
ENSG00000070761	CFAP20	-1.1	2.2E-03	4.9E-03	33.975	1.243	32.874	35.322	39.080	1.388	37.649	40.421
ENSG00000070785	EIF2B3	-1.1	1.3E-02	2.3E-02	24.985	2.081	23.594	27.376	28.106	1.103	27.069	29.264
ENSG00000070814	TCOF1	-1.1	2.3E-02	4.0E-02	59.684	0.948	58.995	60.765	64.016	2.485	61.199	65.896
ENSG00000071054	MAP4K4	-1.1	1.2E-03	2.9E-03	63.167	0.347	62.831	63.525	68.997	2.397	66.435	71.185
ENSG00000071242	RPS6KA2	-1.1	1.2E-01	1.8E-01	9.353	0.788	8.469	9.984	10.149	1.231	8.829	11.266
ENSG00000072062	PRKACA	-1.1	5.1E-03	1.0E-02	12.817	0.763	11.988	13.490	14.605	0.288	14.279	14.826
ENSG00000072415	MPP5	-1.1	3.2E-03	6.8E-03	14.827	0.173	14.630	14.953	16.574	0.492	16.281	17.142
ENSG00000072506	HSD17B10	-1.1	1.6E-03	3.7E-03	128.200	3.012	126.070	131.646	144.375	4.112	141.475	149.081
ENSG00000073584	SMARCE1	-1.1	1.6E-02	2.9E-02	20.296	0.439	19.859	20.737	21.934	0.465	21.422	22.329
ENSG00000073614	KDM5A	-1.1	1.5E-03	3.5E-03	16.192	0.342	15.882	16.558	18.040	0.206	17.831	18.242
ENSG00000073905	VDAC1P1	-1.1	1.2E-01	1.8E-01	94.668	2.178	92.207	96.345	101.793	5.227	97.464	107.600
ENSG00000074211	PPP2R2C	-1.1	1.2E-01	1.7E-01	2.081	0.246	1.930	2.365	2.358	0.279	2.046	2.583
ENSG00000074755	ZZEF1	-1.1	3.1E-03	6.5E-03	5.492	0.187	5.292	5.663	6.278	0.279	5.956	6.446
ENSG00000074842	MYDGF	-1.1	1.1E-02	2.1E-02	49.617	0.511	49.027	49.915	56.314	5.084	51.114	61.274
ENSG00000075043	KCNQ2	-1.1	2.4E-02	4.2E-02	7.605	0.438	7.301	8.106	8.650	1.253	7.370	9.874
ENSG00000075218	GTSE1	-1.1	1.2E-01	1.7E-01	31.455	0.589	30.892	32.066	33.514	2.037	31.448	35.521
ENSG00000075292	ZNF638	-1.1	6.7E-05	2.1E-04	36.964	2.569	34.176	39.234	42.191	1.556	41.245	43.987
ENSG00000075539	FRYL	-1.1	3.1E-02	5.1E-02	10.019	0.894	9.090	10.872	10.872	0.071	10.820	10.953
ENSG00000075618	FSCN1	-1.1	6.2E-03	1.2E-02	256.219	4.320	251.235	258.879	284.168	17.319	266.266	300.838
ENSG00000075856	SART3	-1.1	6.4E-04	1.6E-03	47.366	1.194	45.988	48.081	52.664	1.463	51.314	54.219
ENSG00000076201	PTPN23	-1.1	6.4E-02	1.0E-01	19.746	0.799	18.942	20.540	21.469	1.128	20.202	22.366
ENSG00000076555	ACACB	-1.1	1.0E-01	1.5E-01	2.364	0.139	2.205	2.460	2.636	0.065	2.566	2.695
ENSG00000076604	TRAF4	-1.1	1.7E-04	4.8E-04	56.035	1.014	55.009	57.038	64.073	2.538	61.395	66.442
ENSG00000076924	XAB2	-1.1	7.6E-03	1.5E-02	32.874	1.251	31.644	34.145	36.704	2.474	34.586	39.423
ENSG00000077721	UBE2A	-1.1	5.8E-03	1.2E-02	28.305	0.253	28.120	28.594	31.523	0.901	30.485	32.100
ENSG00000078070	MCCC1	-1.1	3.6E-02	5.9E-02	15.503	0.759	14.643	16.078	17.169	0.691	16.464	17.845
ENSG00000078124	ACER3	-1.1	6.2E-02	9.6E-02	9.246	0.189	9.028	9.365	10.057	0.192	9.837	10.193
ENSG00000078140	UBE2K	-1.1	1.3E-04	3.9E-04	130.060	5.787	124.097	135.654	146.588	7.916	138.832	154.656
ENSG00000078177	N4BP2	-1.1	3.8E-02	6.3E-02	30.344	1.575	28.531	31.375	32.479	1.481	31.557	34.187
ENSG00000078674	PCM1	-1.1	1.6E-03	3.7E-03	38.576	1.205	37.349	39.600	42.176	0.594	41.574	42.762
ENSG00000078902	TOLLIP	-1.1	2.5E-02	4.3E-02	8.748	0.492	8.223	9.197	9.830	0.821	9.292	10.856
ENSG00000079616	KIF22	-1.1	1.5E-03	3.4E-03	74.495	2.616	71.555	76.566	82.323	2.930	79.109	84.847
ENSG00000079785	DDX1	-1.1	2.4E-06	9.7E-06	175.223	3.715	172.268	179.394	201.713	2.544	198.940	203.938
ENSG00000079805	DNM2	-1.1	1.8E-03	4.0E-03	23.633	0.908	22.906	24.651	26.283	1.477	25.261	27.976
ENSG00000079974	RABL2B	-1.1	3.4E-02	5.6E-02	6.330	0.076	6.271	6.415	7.177	0.482	6.621	7.472
ENSG00000080189	SLC35C2	-1.1	7.0E-02	1.1E-01	12.491	0.185	12.277	12.599	13.511	0.594	13.078	14.188
ENSG00000080345	RIF1	-1.1	7.2E-05	2.2E-04	32.127	1.425	30.498	33.146	36.414	1.859	35.055	38.532
ENSG00000080815	PSEN1	-1.1	6.5E-02	1.0E-01	17.992	0.840	17.069	18.711	19.208	0.316	18.860	19.477
ENSG00000080854	IGSF9B	-1.1	1.1E-01	1.6E-01	12.088	1.172	11.270	13.430	13.153	0.983	12.286	14.222
ENSG00000080986	NDC80	-1.1	9.8E-02	1.4E-01	47.486	1.555	46.147	49.192	51.188	3.172	48.543	54.711
ENSG00000081386	ZNF510	-1.1	2.3E-02	3.9E-02	11.223	0.279	11.022	11.541	12.680	0.762	11.838	13.321
ENSG00000082213	C5orf22	-1.1	2.8E-03	5.9E-03	30.116	0.740	29.523	30.946	34.222	1.661	32.326	35.418
ENSG00000082458	DLG3	-1.1	4.3E-02	7.0E-02	23.803	0.424	23.474	24.282	25.478	0.301	25.147	25.733
ENSG00000082515	MRPL22	-1.1	4.2E-02	6.8E-02	19.285	0.590	18.614	19.722	20.985	0.762	20.116	21.541
ENSG00000083312	TNPO1	-1.1	5.6E-02	8.8E-02	116.508	0.981	115.632	117.569	123.184	5.901	116.499	127.666
ENSG00000083642	PDS5B	-1.1	1.1E-01	1.6E-01	15.939	0.943	14.860	16.606	16.886	0.554	16.557	17.526
ENSG00000083720	OXCT1	-1.1	1.5E-02	2.7E-02	34.930	0.276	34.678	35.225	38.623	2.175	37.344	41.134
ENSG00000083817	ZNF416	-1.1	1.2E-01	1.7E-01	9.351	1.807	7.416	10.993	10.716	1.536	8.945	11.693
ENSG00000083896	YTHDC1	-1.1	9.8E-04	2.3E-03	47.525	1.341	45.984	48.428	52.763	1.463	51.194	54.091
ENSG00000084463	WBP11	-1.1	2.6E-02	4.5E-02	79.416	2.684	76.376	81.460	84.783	2.482	81.921	86.339
ENSG00000085063	CD59	-1.1	7.8E-04	1.9E-03	37.014	1.533	35.292	38.231	42.002	2.373	39.512	44.237
ENSG00000085449	WDFY1	-1.1	2.1E-03	4.6E-03	80.534	3.409	77.398	84.162	88.317	1.733	86.401	89.774
ENSG00000085978	ATG16L1	-1.1	4.8E-03	9.8E-03	15.909	0.298	15.566	16.106	17.882	0.823	17.071	18.716
ENSG00000085982	USP40	-1.1	4.6E-02	7.5E-02	4.860	0.152	4.686	4.968	5.415	0.037	5.392	5.457
ENSG00000086015	MAST2	-1.1	2.2E-03	4.9E-03	16.259	0.740	15.688	17.095	18.341	0.640	17.673	18.949
ENSG00000086200	IPO11	-1.1	2.2E-02	3.8E-02	39.498	0.780	38.793	40.337	42.460	1.149	41.216	43.481
ENSG00000086598	TMED2	-1.1	5.5E-03	1.1E-02	186.493	8.533	177.322	194.198	202.379	2.795	200.170	205.520
ENSG00000087008	ACOX3	-1.1	1.2E-01	1.7E-01	5.663	0.475	5.191	6.140	6.404	0.323	6.138	6.763
ENSG00000087095	NLK	-1.1	5.6E-02	8.8E-02	39.545	1.221	38.815	40.954	42.330	0.704	41.527	42.842
ENSG00000087274	ADD1	-1.1	2.3E-04	6.3E-04	26.824	1.423	25.633	28.399	30.202	1.078	28.975	30.995
ENSG00000087299	L2HGDH	-1.1	1.1E-01	1.6E-01	18.453	0.452	18.140	18.971	19.651	0.262	19.423	19.938
ENSG00000087470	DNM1L	-1.1	1.9E-04	5.3E-04	53.194	1.236	52.121	54.545	59.441	0.837	58.558	60.223
ENSG00000088179	PTPN4	-1.1	3.6E-02	6.0E-02	12.829	0.265	12.532	13.042	13.850	0.360	13.515	14.230
ENSG00000088247	KHSRP	-1.1	3.8E-02	6.3E-02	173.095	3.425	169.317	175.997	183.653	10.159	173.286	193.591
ENSG00000088899	LZTS3	-1.1	5.4E-02	8.5E-02	19.971	2.141	18.138	22.325	21.700	0.546	21.283	22.318
ENSG00000089022	MAPKAPK5	-1.1	4.5E-04	1.2E-03	11.835	0.297	11.494	12.041	13.470	0.570	12.856	13.983
ENSG00000089053	ANAPC5	-1.1	2.0E-03	4.5E-03	26.799	0.688	26.048	27.397	29.525	0.570	28.869	29.904
ENSG00000089737	DDX24	-1.1	8.0E-06	2.9E-05	58.168	1.154	57.270	59.470	67.049	2.138	65.444	69.476
ENSG00000089818	NECAP1	-1.1	5.3E-02	8.4E-02	6.550	0.350	6.150	6.799	7.322	0.383	6.915	7.675
ENSG00000090097	PCBP4	-1.1	6.0E-02	9.3E-02	8.798	0.478	8.424	9.336	9.852	0.443	9.553	10.360
ENSG00000090487	SPG21	-1.1	3.4E-02	5.7E-02	48.309	2.091	45.932	49.869	52.170	3.364	48.351	54.695
ENSG00000090889	KIF4A	-1.1	2.1E-03	4.6E-03	44.410	0.711	43.641	45.043	49.549	1.560	47.755	50.586
ENSG00000090971	NAT14	-1.1	1.5E-02	2.8E-02	34.946	0.844	33.995	35.608	39.471	0.960	38.674	40.537
ENSG00000090989	EXOC1	-1.1	2.1E-02	3.6E-02	18.386	1.481	16.753	19.641	20.359	1.053	19.382	21.474
ENSG00000091009	RBM27	-1.1	1.4E-01	1.9E-01	23.471	1.514	22.268	25.171	25.008	2.053	22.637	26.226
ENSG00000091127	PUS7	-1.1	6.0E-02	9.4E-02	46.844	1.877	44.688	48.114	50.028	1.088	49.320	51.281
ENSG00000091140	DLD	-1.1	1.1E-04	3.2E-04	51.026	2.960	48.818	54.390	58.339	0.759	57.654	59.155
ENSG00000091317	CMTM6	-1.1	1.7E-04	4.8E-04	82.667	3.165	80.689	86.317	94.103	5.661	87.911	99.013
ENSG00000091409	ITGA6	-1.1	2.0E-05	6.8E-05	106.833	4.635	102.772	111.882	122.239	2.408	118.789	124.603
ENSG00000091436	MAP3K20	-1.1	8.8E-03	1.7E-02	7.584	0.223	7.371	7.817	8.483	0.069	8.416	8.554
ENSG00000091592	NLRP1	-1.1	1.4E-01	2.0E-01	1.255	0.092	1.166	1.350	1.418	0.059	1.367	1.483
ENSG00000092010	PSME1	-1.1	1.6E-03	3.6E-03	52.787	0.983	51.654	53.408	60.635	2.496	57.863	62.707
ENSG00000092421	SEMA6A	-1.1	3.2E-02	5.3E-02	94.553	2.697	91.967	97.348	100.434	3.215	96.810	102.942
ENSG00000092847	AGO1	-1.1	7.1E-03	1.4E-02	29.285	0.617	28.600	29.796	31.773	1.317	30.722	33.251
ENSG00000092978	GPATCH2	-1.1	3.5E-02	5.8E-02	6.865	0.470	6.326	7.185	7.676	0.679	6.924	8.244
ENSG00000093144	ECHDC1	-1.1	7.5E-03	1.5E-02	35.180	0.329	34.883	35.534	38.657	2.143	36.504	40.790
ENSG00000094804	CDC6	-1.1	2.5E-03	5.5E-03	72.025	1.210	71.025	73.371	79.124	1.074	77.893	79.873
ENSG00000094880	CDC23	-1.1	4.6E-03	9.4E-03	43.758	2.210	41.230	45.322	48.462	2.120	46.885	50.873
ENSG00000095203	EPB41L4B	-1.1	6.3E-04	1.6E-03	15.586	0.406	15.123	15.884	17.793	0.390	17.380	18.155
ENSG00000095574	IKZF5	-1.1	9.9E-02	1.4E-01	10.783	0.266	10.480	10.980	11.896	0.893	11.227	12.910
ENSG00000096063	SRPK1	-1.1	5.6E-04	1.4E-03	95.016	2.593	92.172	97.249	105.228	4.122	101.273	109.499
ENSG00000096696	DSP	-1.1	4.2E-04	1.1E-03	42.396	1.147	41.627	43.714	47.088	1.249	45.659	47.890
ENSG00000097007	ABL1	-1.1	3.6E-04	9.5E-04	55.158	1.195	54.450	56.538	62.751	3.587	58.893	65.987
ENSG00000099246	RAB18	-1.1	1.0E-02	2.0E-02	30.817	0.441	30.309	31.092	34.057	3.045	30.678	36.589
ENSG00000099282	TSPAN15	-1.1	1.4E-01	1.9E-01	7.543	0.952	6.878	8.633	8.541	0.574	7.988	9.133
ENSG00000099331	MYO9B	-1.1	2.1E-02	3.6E-02	23.156	0.884	22.191	23.927	25.238	1.161	24.266	26.524
ENSG00000099381	SETD1A	-1.1	7.0E-03	1.4E-02	13.854	0.411	13.397	14.194	15.701	1.343	14.292	16.965
ENSG00000099385	BCL7C	-1.1	1.1E-01	1.6E-01	9.735	0.410	9.279	10.072	10.675	1.074	9.529	11.658
ENSG00000099624	ATP5D	-1.1	4.6E-02	7.4E-02	45.735	1.842	43.857	47.539	51.376	4.105	47.618	55.757
ENSG00000099800	TIMM13	-1.1	2.0E-02	3.5E-02	127.544	5.262	124.170	133.607	137.605	6.196	131.971	144.241
ENSG00000099954	CECR2	-1.1	3.5E-04	9.3E-04	43.778	0.293	43.454	43.978	49.110	1.083	48.259	50.329
ENSG00000100028	SNRPD3	-1.1	7.0E-02	1.1E-01	190.276	7.388	184.867	198.693	200.793	3.407	197.183	203.954
ENSG00000100077	GRK3	-1.1	1.9E-02	3.3E-02	17.677	0.580	17.256	18.338	19.397	1.005	18.242	20.070
ENSG00000100092	SH3BP1	-1.1	2.8E-02	4.7E-02	12.575	0.736	11.944	13.384	14.284	1.390	13.034	15.780
ENSG00000100104	SRRD	-1.1	8.9E-03	1.7E-02	20.470	1.584	18.641	21.399	23.583	0.418	23.275	24.058
ENSG00000100209	HSCB	-1.1	1.1E-01	1.6E-01	15.879	1.455	14.211	16.892	17.857	0.205	17.676	18.079
ENSG00000100219	XBP1	-1.1	6.5E-04	1.6E-03	85.661	1.804	83.699	87.247	96.340	2.976	93.233	99.164
ENSG00000100220	RTCB	-1.1	9.6E-04	2.3E-03	132.597	2.661	129.743	135.009	147.032	4.111	142.342	150.008
ENSG00000100227	POLDIP3	-1.1	2.2E-02	3.9E-02	77.800	1.998	75.957	79.923	84.321	0.844	83.648	85.267
ENSG00000100239	PPP6R2	-1.1	8.5E-02	1.3E-01	18.864	0.361	18.602	19.276	20.310	0.737	19.713	21.134
ENSG00000100241	SBF1	-1.1	7.7E-02	1.2E-01	23.454	1.131	22.410	24.655	24.988	1.455	23.318	25.854
ENSG00000100266	PACSIN2	-1.1	7.0E-03	1.4E-02	47.914	1.093	46.705	48.831	52.457	0.821	51.839	53.389
ENSG00000100280	AP1B1	-1.1	6.5E-03	1.3E-02	37.639	1.527	36.643	39.397	41.860	3.758	38.762	46.041
ENSG00000100294	MCAT	-1.1	6.2E-02	9.6E-02	10.659	0.530	10.122	11.182	12.250	0.899	11.290	13.070
ENSG00000100325	ASCC2	-1.1	6.5E-03	1.3E-02	14.371	0.462	13.993	14.886	16.401	0.760	15.627	17.146
ENSG00000100364	KIAA0930	-1.1	1.2E-02	2.3E-02	21.310	0.483	20.876	21.830	23.340	1.379	21.750	24.206
ENSG00000100379	KCTD17	-1.1	9.1E-02	1.4E-01	19.654	0.477	19.110	20.000	21.566	0.849	20.708	22.406
ENSG00000100393	EP300	-1.1	1.5E-03	3.4E-03	35.683	0.580	35.019	36.087	39.640	0.682	39.046	40.385
ENSG00000100401	RANGAP1	-1.1	3.3E-04	8.7E-04	90.149	0.943	89.199	91.085	101.071	3.105	97.975	104.184
ENSG00000100412	ACO2	-1.1	2.7E-04	7.3E-04	47.952	2.637	44.928	49.771	55.323	0.940	54.257	56.034
ENSG00000100462	PRMT5	-1.1	2.1E-04	5.7E-04	116.202	1.615	114.340	117.220	129.791	1.780	128.044	131.603
ENSG00000100519	PSMC6	-1.1	2.2E-02	3.8E-02	36.594	0.783	35.844	37.406	39.703	1.802	37.623	40.788
ENSG00000100532	CGRRF1	-1.1	1.1E-01	1.6E-01	8.482	1.193	7.203	9.564	9.544	0.987	8.455	10.412
ENSG00000100596	SPTLC2	-1.1	3.9E-03	8.0E-03	27.014	0.852	26.111	27.804	29.906	1.164	29.151	31.246
ENSG00000100632	ERH	-1.1	2.1E-03	4.6E-03	483.485	6.545	476.575	489.591	531.424	15.396	514.895	545.355
ENSG00000100731	PCNX1	-1.1	2.6E-04	7.0E-04	14.815	0.060	14.747	14.861	16.900	0.578	16.259	17.383
ENSG00000100744	GSKIP	-1.1	1.1E-01	1.6E-01	34.519	1.177	33.492	35.804	36.935	3.177	33.471	39.712
ENSG00000100749	VRK1	-1.1	1.5E-02	2.8E-02	87.557	1.758	86.224	89.549	94.756	2.523	91.885	96.621
ENSG00000100796	PPP4R3A	-1.1	3.1E-03	6.6E-03	58.032	4.068	53.651	61.690	65.565	1.651	64.075	67.340
ENSG00000100802	C14orf93	-1.1	8.0E-02	1.2E-01	4.017	0.255	3.783	4.289	4.627	0.357	4.379	5.037
ENSG00000100813	ACIN1	-1.1	1.5E-04	4.2E-04	75.130	2.764	73.447	78.320	84.311	1.578	82.494	85.343
ENSG00000100823	APEX1	-1.1	3.1E-03	6.5E-03	425.716	4.131	420.982	428.592	462.690	2.781	459.481	464.418
ENSG00000100883	SRP54	-1.1	1.2E-01	1.7E-01	56.670	0.878	56.116	57.683	59.693	2.224	57.403	61.845
ENSG00000100997	ABHD12	-1.1	8.3E-03	1.6E-02	32.350	1.109	31.337	33.535	36.361	1.801	34.301	37.639
ENSG00000101057	MYBL2	-1.1	3.0E-03	6.3E-03	150.473	2.787	147.256	152.149	170.998	12.005	160.413	184.042
ENSG00000101126	ADNP	-1.1	2.3E-04	6.3E-04	66.905	0.570	66.264	67.355	74.699	2.661	73.129	77.772
ENSG00000101150	TPD52L2	-1.1	6.1E-04	1.5E-03	62.588	1.369	61.424	64.096	69.639	0.554	69.219	70.266
ENSG00000101166	PRELID3B	-1.1	4.6E-02	7.5E-02	55.747	2.646	54.034	58.795	60.614	5.945	55.333	67.052
ENSG00000101181	MTG2	-1.1	1.7E-02	3.1E-02	8.263	0.537	7.666	8.708	9.502	0.591	9.144	10.183
ENSG00000101182	PSMA7	-1.1	1.1E-02	2.1E-02	328.864	6.350	322.236	334.893	354.451	10.258	343.394	363.659
ENSG00000101193	GID8	-1.1	9.8E-05	2.9E-04	65.833	0.915	64.829	66.622	74.766	1.013	73.610	75.496
ENSG00000101220	C20orf27	-1.1	3.5E-02	5.9E-02	50.476	2.014	48.637	52.628	55.141	2.784	52.116	57.594
ENSG00000101391	CDK5RAP1	-1.1	6.5E-02	1.0E-01	21.631	0.527	21.157	22.198	23.411	1.229	22.206	24.663
ENSG00000101442	ACTR5	-1.1	6.4E-02	9.9E-02	19.028	0.506	18.459	19.428	21.083	0.681	20.468	21.815
ENSG00000101457	DNTTIP1	-1.1	4.6E-02	7.4E-02	41.505	0.918	40.601	42.437	46.133	0.520	45.697	46.709
ENSG00000101464	PIGU	-1.1	6.0E-03	1.2E-02	30.077	2.660	27.153	32.353	34.416	0.623	33.902	35.109
ENSG00000101473	ACOT8	-1.1	7.3E-02	1.1E-01	10.420	0.305	10.148	10.749	11.478	0.557	10.842	11.879
ENSG00000101624	CEP76	-1.1	9.6E-02	1.4E-01	9.228	0.563	8.778	9.859	10.105	0.489	9.709	10.651
ENSG00000101966	XIAP	-1.1	1.6E-02	3.0E-02	17.350	0.717	16.529	17.858	19.175	1.234	18.229	20.570
ENSG00000101974	ATP11C	-1.1	3.2E-02	5.4E-02	50.130	2.213	47.865	52.288	53.528	1.645	52.523	55.426
ENSG00000102030	NAA10	-1.1	8.9E-02	1.3E-01	10.750	1.223	9.342	11.564	11.846	0.805	11.029	12.638
ENSG00000102158	MAGT1	-1.1	2.6E-02	4.4E-02	46.106	0.686	45.333	46.645	49.854	1.485	48.100	50.710
ENSG00000102241	HTATSF1	-1.1	1.4E-05	5.0E-05	118.408	3.871	114.183	121.784	136.602	3.066	133.285	139.332
ENSG00000102580	DNAJC3	-1.1	3.2E-03	6.9E-03	15.749	0.215	15.515	15.939	18.044	1.034	16.974	19.038
ENSG00000102710	SUPT20H	-1.1	4.7E-04	1.2E-03	21.536	0.849	20.767	22.447	24.224	0.338	23.835	24.446
ENSG00000102804	TSC22D1	-1.1	8.1E-03	1.6E-02	34.333	0.949	33.689	35.423	37.617	1.261	36.508	38.988
ENSG00000102893	PHKB	-1.1	1.5E-02	2.7E-02	15.508	0.373	15.144	15.889	17.074	1.095	16.436	18.338
ENSG00000103037	SETD6	-1.1	2.2E-02	3.9E-02	17.728	0.693	17.108	18.476	19.591	0.315	19.254	19.879
ENSG00000103043	VAC14	-1.1	1.0E-02	1.9E-02	18.730	0.974	17.610	19.378	20.684	0.942	19.596	21.239
ENSG00000103061	SLC7A6OS	-1.1	2.2E-02	3.9E-02	13.717	0.573	13.194	14.330	15.265	0.476	14.734	15.654
ENSG00000103067	ESRP2	-1.1	5.7E-04	1.4E-03	22.912	1.154	21.777	24.083	26.376	0.860	25.702	27.345
ENSG00000103091	WDR59	-1.1	6.0E-02	9.4E-02	18.045	0.249	17.762	18.233	19.286	0.740	18.473	19.921
ENSG00000103111	MON1B	-1.1	3.4E-02	5.6E-02	17.312	0.229	17.076	17.533	18.994	0.964	18.211	20.070
ENSG00000103226	NOMO3	-1.1	2.1E-03	4.6E-03	27.250	0.719	26.426	27.749	30.365	0.467	29.901	30.836
ENSG00000103342	GSPT1	-1.1	6.7E-06	2.5E-05	101.174	2.720	98.124	103.351	115.873	2.150	113.930	118.183
ENSG00000103353	UBFD1	-1.1	7.1E-05	2.1E-04	49.015	1.324	47.835	50.448	56.041	0.869	55.493	57.043
ENSG00000103356	EARS2	-1.1	1.0E-02	1.9E-02	37.248	1.544	35.467	38.214	40.721	1.645	38.835	41.861
ENSG00000103415	HMOX2	-1.1	1.2E-01	1.7E-01	21.398	0.902	20.543	22.340	22.944	0.354	22.723	23.352
ENSG00000103429	BFAR	-1.1	2.8E-04	7.5E-04	59.255	1.634	58.127	61.129	66.873	1.995	65.501	69.162
ENSG00000103512	NOMO1	-1.1	5.7E-02	8.9E-02	55.705	0.910	54.728	56.528	59.067	1.111	57.784	59.730
ENSG00000103591	AAGAB	-1.1	4.9E-04	1.2E-03	51.125	1.490	49.589	52.564	57.884	1.380	57.028	59.476
ENSG00000103707	MTFMT	-1.1	3.2E-02	5.4E-02	13.297	0.598	12.727	13.920	14.964	0.564	14.612	15.615
ENSG00000103723	AP3B2	-1.1	8.0E-02	1.2E-01	4.297	0.086	4.230	4.394	4.785	0.185	4.664	4.999
ENSG00000103769	RAB11A	-1.1	5.9E-02	9.2E-02	86.556	1.761	84.559	87.885	91.604	4.026	88.100	96.002
ENSG00000103852	TTC23	-1.1	3.5E-02	5.9E-02	10.076	0.171	9.901	10.243	11.168	0.454	10.862	11.689
ENSG00000104147	OIP5	-1.1	3.5E-02	5.8E-02	77.294	3.302	74.462	80.921	84.915	2.729	81.785	86.797
ENSG00000104413	ESRP1	-1.1	5.4E-04	1.4E-03	115.115	0.188	114.899	115.238	127.049	2.109	124.614	128.284
ENSG00000104517	UBR5	-1.1	3.7E-02	6.0E-02	84.463	1.398	82.948	85.703	89.382	2.272	87.153	91.694
ENSG00000104660	LEPROTL1	-1.1	5.9E-03	1.2E-02	31.841	1.375	30.392	33.127	35.732	0.790	34.914	36.490
ENSG00000104687	GSR	-1.1	2.0E-03	4.5E-03	79.399	2.399	77.549	82.110	88.181	1.096	87.213	89.371
ENSG00000104731	KLHDC4	-1.1	3.9E-03	8.2E-03	8.705	0.604	8.176	9.363	10.004	0.904	9.157	10.956
ENSG00000104805	NUCB1	-1.1	2.0E-03	4.5E-03	53.377	0.699	52.584	53.906	60.035	3.300	56.415	62.483
ENSG00000104852	SNRNP70	-1.1	6.8E-02	1.0E-01	133.936	4.006	129.437	137.117	141.290	7.377	136.467	149.783
ENSG00000104866	PPP1R37	-1.1	7.3E-02	1.1E-01	6.080	0.109	5.998	6.204	6.693	0.441	6.392	7.199
ENSG00000104884	ERCC2	-1.1	1.9E-02	3.4E-02	34.564	0.641	33.837	35.051	37.533	1.246	36.227	38.710
ENSG00000104889	RNASEH2A	-1.1	4.0E-02	6.5E-02	66.709	2.654	64.168	69.463	72.115	2.579	69.146	73.801
ENSG00000104897	SF3A2	-1.1	1.4E-01	2.0E-01	44.150	1.489	42.556	45.507	47.102	4.809	42.432	52.039
ENSG00000104907	TRMT1	-1.1	6.1E-03	1.2E-02	42.048	1.154	40.869	43.175	46.581	0.815	45.864	47.467
ENSG00000104967	NOVA2	-1.1	1.1E-01	1.6E-01	6.712	0.488	6.173	7.122	7.312	0.310	6.966	7.561
ENSG00000104980	TIMM44	-1.1	2.0E-02	3.6E-02	35.346	1.055	34.198	36.274	38.747	3.599	35.179	42.376
ENSG00000104998	IL27RA	-1.1	3.2E-02	5.4E-02	13.506	1.428	12.166	15.008	15.389	0.311	15.047	15.653
ENSG00000105088	OLFM2	-1.1	3.2E-03	6.8E-03	68.276	1.769	67.173	70.317	76.570	2.424	74.538	79.254
ENSG00000105127	AKAP8	-1.1	1.6E-02	3.0E-02	26.043	0.938	24.970	26.710	28.743	0.622	28.029	29.171
ENSG00000105176	URI1	-1.1	9.3E-02	1.4E-01	56.176	1.862	54.027	57.256	59.287	1.535	57.939	60.957
ENSG00000105197	TIMM50	-1.1	8.0E-04	1.9E-03	33.837	1.869	32.548	35.980	38.195	1.573	36.994	39.975
ENSG00000105223	PLD3	-1.1	1.9E-03	4.3E-03	65.449	1.255	64.157	66.664	73.242	4.115	70.812	77.993
ENSG00000105245	NUMBL	-1.1	5.2E-02	8.3E-02	6.366	0.598	5.687	6.813	7.250	0.749	6.621	8.079
ENSG00000105255	FSD1	-1.1	4.2E-03	8.6E-03	30.171	1.385	29.010	31.703	34.197	0.810	33.307	34.891
ENSG00000105364	MRPL4	-1.1	8.6E-04	2.1E-03	34.184	0.885	33.541	35.193	39.176	2.887	35.866	41.177
ENSG00000105379	ETFB	-1.1	6.8E-02	1.0E-01	4.085	0.171	3.889	4.203	4.637	0.144	4.470	4.722
ENSG00000105401	CDC37	-1.1	4.2E-04	1.1E-03	133.896	3.355	130.026	135.975	150.242	5.543	144.787	155.868
ENSG00000105404	RABAC1	-1.1	1.0E-02	1.9E-02	49.265	4.166	44.740	52.943	55.360	3.352	51.850	58.529
ENSG00000105429	MEGF8	-1.1	2.0E-02	3.6E-02	22.596	0.583	22.015	23.180	24.987	1.657	23.412	26.716
ENSG00000105576	TNPO2	-1.1	2.5E-04	6.9E-04	33.412	1.034	32.236	34.177	37.866	0.427	37.563	38.354
ENSG00000105583	WDR83OS	-1.1	1.5E-02	2.8E-02	30.577	2.775	28.694	33.764	34.454	2.189	32.187	36.555
ENSG00000105605	CACNG7	-1.1	5.5E-02	8.6E-02	33.898	0.405	33.461	34.261	36.856	3.298	33.376	39.935
ENSG00000105677	TMEM147	-1.1	1.1E-02	2.1E-02	93.159	1.336	91.914	94.570	103.034	6.560	95.511	107.565
ENSG00000105726	ATP13A1	-1.1	3.6E-03	7.5E-03	12.456	0.319	12.107	12.732	14.153	0.621	13.525	14.766
ENSG00000105821	DNAJC2	-1.1	1.6E-02	2.9E-02	28.515	2.292	26.289	30.868	32.041	2.894	29.515	35.199
ENSG00000106028	SSBP1	-1.1	8.5E-03	1.6E-02	103.032	2.909	100.506	106.213	111.530	2.511	109.106	114.120
ENSG00000106049	HIBADH	-1.1	1.3E-03	3.0E-03	50.776	1.401	49.301	52.088	57.894	3.267	54.885	61.369
ENSG00000106144	CASP2	-1.1	6.4E-03	1.3E-02	10.796	0.715	10.142	11.559	12.339	0.253	12.080	12.587
ENSG00000106266	SNX8	-1.1	2.3E-02	4.0E-02	16.402	0.455	16.122	16.926	18.099	1.074	16.865	18.826
ENSG00000106290	TAF6	-1.1	3.2E-03	6.8E-03	42.644	0.348	42.427	43.046	47.771	2.684	45.093	50.460
ENSG00000106344	RBM28	-1.1	1.3E-03	3.0E-03	11.327	0.441	10.880	11.761	12.853	0.486	12.344	13.312
ENSG00000106605	BLVRA	-1.1	7.9E-02	1.2E-01	34.066	0.965	33.419	35.176	37.232	2.301	34.596	38.835
ENSG00000106683	LIMK1	-1.1	3.5E-04	9.2E-04	39.499	1.003	38.730	40.633	45.051	2.702	41.975	47.041
ENSG00000106803	SEC61B	-1.1	1.0E-02	1.9E-02	174.744	4.890	171.049	180.290	191.717	2.650	188.873	194.116
ENSG00000106853	PTGR1	-1.1	1.9E-02	3.3E-02	28.811	1.274	27.608	30.146	31.815	1.850	29.682	32.981
ENSG00000106993	CDC37L1	-1.1	2.1E-02	3.6E-02	23.334	1.354	22.540	24.897	25.983	1.375	24.672	27.414
ENSG00000107186	MPDZ	-1.1	2.3E-04	6.3E-04	25.699	0.532	25.097	26.109	29.009	1.162	28.221	30.343
ENSG00000107372	ZFAND5	-1.1	1.0E-02	2.0E-02	67.299	2.213	65.821	69.844	73.080	2.715	70.086	75.379
ENSG00000107404	DVL1	-1.1	1.3E-01	1.8E-01	26.281	0.631	25.791	26.993	28.230	1.106	27.249	29.425
ENSG00000107562	CXCL12	-1.1	1.0E-02	1.9E-02	20.949	1.118	19.659	21.640	23.824	1.363	22.260	24.755
ENSG00000107566	ERLIN1	-1.1	1.7E-04	4.8E-04	24.452	0.780	23.653	25.212	28.171	0.329	27.928	28.546
ENSG00000107669	ATE1	-1.1	2.3E-02	4.0E-02	19.606	0.184	19.456	19.812	21.483	0.953	20.597	22.492
ENSG00000107731	UNC5B	-1.1	8.1E-02	1.2E-01	17.298	0.875	16.461	18.208	18.695	1.197	17.674	20.013
ENSG00000107815	TWNK	-1.1	3.3E-02	5.5E-02	34.460	2.553	32.163	37.209	37.777	0.205	37.650	38.014
ENSG00000107882	SUFU	-1.1	4.9E-02	7.8E-02	5.505	0.238	5.239	5.696	6.166	0.296	5.921	6.495
ENSG00000107937	GTPBP4	-1.1	2.0E-02	3.5E-02	64.485	0.815	63.604	65.213	68.875	0.655	68.348	69.608
ENSG00000108021	FAM208B	-1.1	6.4E-02	9.8E-02	34.828	1.219	33.855	36.195	37.120	2.061	35.322	39.368
ENSG00000108091	CCDC6	-1.1	1.1E-03	2.5E-03	42.096	1.363	41.116	43.653	46.764	0.232	46.515	46.975
ENSG00000108094	CUL2	-1.1	8.5E-02	1.3E-01	35.632	0.273	35.321	35.830	37.847	1.388	36.349	39.116
ENSG00000108344	PSMD3	-1.1	7.3E-06	2.7E-05	95.955	1.185	94.599	96.795	110.408	4.141	105.667	113.315
ENSG00000108384	RAD51C	-1.1	2.4E-02	4.1E-02	6.954	0.715	6.181	7.592	8.023	0.280	7.715	8.264
ENSG00000108395	TRIM37	-1.1	1.7E-02	3.0E-02	58.584	0.550	58.183	59.211	62.983	2.762	59.855	65.090
ENSG00000108424	KPNB1	-1.1	2.2E-02	3.9E-02	269.524	6.209	264.345	276.408	285.188	5.937	278.683	291.479
ENSG00000108439	PNPO	-1.1	1.3E-01	1.8E-01	9.127	1.078	7.943	10.051	10.236	0.473	9.877	10.772
ENSG00000108443	RPS6KB1	-1.1	1.0E-02	2.0E-02	14.317	0.769	13.842	15.205	15.984	0.719	15.495	16.809
ENSG00000108582	CPD	-1.1	3.3E-03	6.9E-03	23.203	1.051	21.993	23.881	25.508	0.457	24.994	25.870
ENSG00000108587	GOSR1	-1.1	3.4E-03	7.1E-03	12.706	0.311	12.346	12.887	14.311	0.373	14.017	14.730
ENSG00000108604	SMARCD2	-1.1	5.7E-03	1.1E-02	73.765	1.204	72.807	75.117	80.741	1.412	79.683	82.345
ENSG00000108654	DDX5	-1.1	7.4E-04	1.8E-03	235.800	12.914	220.905	243.849	259.142	6.272	251.913	263.134
ENSG00000108666	C17orf75	-1.1	1.1E-01	1.6E-01	19.780	0.687	19.088	20.461	21.228	0.512	20.811	21.799
ENSG00000108774	RAB5C	-1.1	9.3E-03	1.8E-02	22.949	1.243	21.929	24.333	26.279	1.859	24.133	27.411
ENSG00000108852	MPP2	-1.1	1.7E-02	3.1E-02	18.019	1.769	16.779	20.045	20.142	0.398	19.684	20.408
ENSG00000108883	EFTUD2	-1.1	1.4E-02	2.5E-02	50.995	2.415	48.405	53.185	54.869	1.240	53.546	56.004
ENSG00000108960	MMD	-1.1	1.2E-03	2.8E-03	46.088	0.972	45.012	46.903	52.290	3.047	49.317	55.405
ENSG00000109113	RAB34	-1.1	9.4E-04	2.2E-03	103.485	1.492	101.770	104.474	114.785	2.407	113.203	117.555
ENSG00000109332	UBE2D3	-1.1	1.5E-02	2.7E-02	104.416	1.814	102.898	106.425	111.226	1.495	109.588	112.518
ENSG00000109536	FRG1	-1.1	1.1E-01	1.6E-01	47.021	1.352	45.491	48.055	50.817	3.404	46.928	53.250
ENSG00000109586	GALNT7	-1.1	7.3E-02	1.1E-01	60.529	1.437	58.649	62.187	64.296	2.668	62.172	67.290
ENSG00000109606	DHX15	-1.1	8.5E-06	3.1E-05	138.418	2.331	135.730	139.870	157.038	2.745	153.871	158.737
ENSG00000109790	KLHL5	-1.1	7.0E-02	1.1E-01	18.818	0.910	17.793	19.533	20.496	1.986	18.265	22.070
ENSG00000109805	NCAPG	-1.1	8.1E-02	1.2E-01	68.850	1.931	66.861	70.718	72.968	1.982	71.451	75.210
ENSG00000109814	UGDH	-1.1	4.7E-03	9.5E-03	63.036	0.713	62.388	63.800	69.583	3.169	65.939	71.704
ENSG00000109832	DDX25	-1.1	1.6E-02	2.8E-02	4.984	0.160	4.838	5.156	5.678	0.077	5.615	5.764
ENSG00000109861	CTSC	-1.1	7.8E-03	1.5E-02	90.774	2.047	88.520	93.135	97.404	2.608	94.717	99.926
ENSG00000109881	CCDC34	-1.1	8.2E-02	1.2E-01	20.092	0.764	19.309	20.835	21.928	0.869	20.918	22.648
ENSG00000110042	DTX4	-1.1	6.9E-03	1.4E-02	12.868	0.535	12.458	13.473	14.534	0.287	14.275	14.842
ENSG00000110060	PUS3	-1.1	2.5E-02	4.2E-02	25.982	1.419	24.759	27.538	29.416	0.916	28.358	29.953
ENSG00000110075	PPP6R3	-1.1	7.0E-03	1.4E-02	68.705	3.340	65.996	72.437	74.979	3.319	71.161	77.178
ENSG00000110172	CHORDC1	-1.1	2.6E-03	5.7E-03	27.800	1.489	26.135	29.005	31.023	1.235	29.742	32.207
ENSG00000110274	CEP164	-1.1	9.1E-03	1.7E-02	3.368	0.173	3.171	3.494	3.886	0.082	3.815	3.975
ENSG00000110321	EIF4G2	-1.1	9.8E-03	1.9E-02	382.938	9.040	373.700	391.766	409.621	15.349	394.923	425.368
ENSG00000110344	UBE4A	-1.1	3.9E-04	1.0E-03	27.216	0.486	26.781	27.741	31.270	1.098	30.249	32.432
ENSG00000110651	CD81	-1.1	1.3E-01	1.8E-01	50.007	1.379	48.633	51.392	52.662	4.227	49.157	57.356
ENSG00000110696	C11orf58	-1.1	8.6E-04	2.1E-03	79.569	2.127	77.193	81.296	87.896	3.176	85.676	91.534
ENSG00000110717	NDUFS8	-1.1	2.7E-02	4.6E-02	33.679	0.825	33.017	34.603	36.513	1.791	34.559	38.078
ENSG00000110768	GTF2H1	-1.1	3.4E-03	7.2E-03	13.295	0.399	12.842	13.596	15.109	0.573	14.776	15.770
ENSG00000111145	ELK3	-1.1	3.9E-02	6.5E-02	13.838	0.819	12.971	14.598	15.541	0.689	14.962	16.304
ENSG00000111247	RAD51AP1	-1.1	1.0E-01	1.5E-01	66.723	0.737	65.980	67.453	70.812	2.443	68.153	72.957
ENSG00000111266	DUSP16	-1.1	1.1E-02	2.0E-02	57.428	1.780	55.792	59.323	62.469	1.314	61.498	63.964
ENSG00000111300	NAA25	-1.1	6.3E-02	9.7E-02	28.205	0.877	27.194	28.742	29.953	1.118	28.799	31.033
ENSG00000111445	RFC5	-1.1	3.7E-04	9.7E-04	40.334	0.513	39.953	40.917	46.382	1.185	45.370	47.686
ENSG00000111605	CPSF6	-1.1	2.6E-03	5.6E-03	79.493	3.817	75.661	83.295	86.909	2.675	84.786	89.913
ENSG00000111647	UHRF1BP1L	-1.1	7.9E-02	1.2E-01	17.221	0.661	16.564	17.886	18.563	0.285	18.261	18.828
ENSG00000111665	CDCA3	-1.1	6.2E-02	9.6E-02	17.641	0.040	17.602	17.682	19.050	0.524	18.526	19.573
ENSG00000111666	CHPT1	-1.1	6.7E-02	1.0E-01	12.372	0.693	11.671	13.058	13.486	0.518	13.145	14.081
ENSG00000111667	USP5	-1.1	6.3E-02	9.8E-02	91.417	2.899	88.672	94.449	97.202	2.634	94.957	100.101
ENSG00000111696	NT5DC3	-1.1	7.2E-02	1.1E-01	4.319	0.391	3.929	4.711	4.848	0.361	4.452	5.159
ENSG00000111802	TDP2	-1.1	7.6E-04	1.9E-03	75.532	4.398	70.521	78.752	85.619	3.176	82.030	88.064
ENSG00000111817	DSE	-1.1	1.7E-03	3.9E-03	14.669	0.029	14.636	14.691	16.343	0.375	16.065	16.769
ENSG00000111961	SASH1	-1.1	8.8E-02	1.3E-01	8.852	0.343	8.460	9.101	9.509	0.319	9.201	9.839
ENSG00000112031	MTRF1L	-1.1	9.7E-02	1.4E-01	12.699	0.788	11.794	13.232	14.112	0.778	13.499	14.987
ENSG00000112130	RNF8	-1.1	1.8E-02	3.2E-02	19.360	1.237	18.540	20.782	21.297	0.170	21.113	21.447
ENSG00000112200	ZNF451	-1.1	1.0E-01	1.5E-01	12.545	0.727	11.706	12.972	13.321	0.303	13.097	13.666
ENSG00000112210	RAB23	-1.1	7.0E-03	1.4E-02	16.938	1.261	15.949	18.358	19.399	1.352	17.938	20.607
ENSG00000112237	CCNC	-1.1	2.2E-03	4.9E-03	80.576	3.319	76.867	83.265	88.918	3.534	85.129	92.124
ENSG00000112245	PTP4A1	-1.1	1.4E-02	2.5E-02	60.390	2.873	58.717	63.707	66.982	5.770	60.608	71.848
ENSG00000112249	ASCC3	-1.1	3.0E-02	5.1E-02	50.710	0.316	50.349	50.932	54.306	2.356	52.215	56.748
ENSG00000112640	PPP2R5D	-1.1	5.9E-03	1.2E-02	53.025	0.704	52.387	53.780	58.195	0.434	57.831	58.676
ENSG00000112655	PTK7	-1.1	4.1E-03	8.5E-03	46.289	0.943	45.269	47.127	50.732	1.520	49.148	52.179
ENSG00000112699	GMDS	-1.1	8.0E-02	1.2E-01	17.391	0.378	16.963	17.674	18.968	1.421	17.753	20.530
ENSG00000112996	MRPS30	-1.1	1.1E-01	1.6E-01	33.200	1.353	31.868	34.572	35.148	1.090	34.387	36.397
ENSG00000113194	FAF2	-1.1	4.3E-03	8.8E-03	31.113	0.324	30.806	31.451	34.733	1.364	33.667	36.270
ENSG00000113282	CLINT1	-1.1	6.1E-04	1.5E-03	58.430	1.325	57.531	59.952	65.388	2.613	62.678	67.892
ENSG00000113300	CNOT6	-1.1	1.1E-02	2.1E-02	37.532	0.701	36.723	37.954	41.057	1.999	38.760	42.396
ENSG00000113456	RAD1	-1.1	8.5E-03	1.6E-02	22.708	1.284	21.232	23.565	25.352	0.876	24.558	26.291
ENSG00000113569	NUP155	-1.1	1.0E-02	2.0E-02	48.737	1.058	47.520	49.449	52.519	1.343	50.968	53.315
ENSG00000113594	LIFR	-1.1	8.5E-02	1.3E-01	8.307	0.557	7.806	8.907	9.031	0.684	8.517	9.808
ENSG00000113595	TRIM23	-1.1	1.3E-02	2.3E-02	9.979	0.082	9.894	10.057	11.504	0.719	10.723	12.138
ENSG00000113716	HMGXB3	-1.1	1.0E-01	1.5E-01	20.188	0.110	20.092	20.307	21.516	0.526	21.077	22.099
ENSG00000113719	ERGIC1	-1.1	5.3E-05	1.7E-04	23.506	0.719	22.742	24.169	26.902	0.417	26.438	27.245
ENSG00000113810	SMC4	-1.1	1.2E-02	2.2E-02	66.749	4.883	61.232	70.513	73.047	3.009	70.382	76.311
ENSG00000113845	TIMMDC1	-1.1	4.6E-04	1.2E-03	61.644	1.096	60.409	62.500	70.291	2.192	68.207	72.577
ENSG00000114098	ARMC8	-1.1	2.8E-03	6.0E-03	15.079	0.590	14.422	15.564	16.936	0.314	16.575	17.142
ENSG00000114353	GNAI2	-1.1	5.3E-04	1.3E-03	69.035	1.124	67.841	70.072	77.710	3.631	73.563	80.320
ENSG00000114354	TFG	-1.1	7.7E-02	1.2E-01	55.635	1.444	54.605	57.285	58.789	1.366	57.656	60.306
ENSG00000114520	SNX4	-1.1	8.2E-02	1.2E-01	36.406	1.181	35.429	37.718	39.340	2.977	36.242	42.180
ENSG00000114529	C3orf52	-1.1	4.8E-02	7.6E-02	7.942	0.245	7.683	8.171	9.008	0.616	8.493	9.690
ENSG00000114573	ATP6V1A	-1.1	8.6E-02	1.3E-01	60.996	2.819	58.069	63.694	64.656	3.401	60.924	67.583
ENSG00000114744	COMMD2	-1.1	2.4E-02	4.2E-02	37.836	0.890	37.170	38.847	40.947	1.721	39.064	42.439
ENSG00000114982	KANSL3	-1.1	2.3E-03	5.0E-03	15.122	0.115	15.031	15.251	17.034	0.581	16.363	17.373
ENSG00000114988	LMAN2L	-1.1	2.1E-02	3.6E-02	18.685	0.325	18.318	18.934	21.145	1.340	19.618	22.121
ENSG00000115053	NCL	-1.1	7.3E-03	1.4E-02	730.062	15.280	712.925	742.267	777.609	7.750	770.733	786.007
ENSG00000115211	EIF2B4	-1.1	5.7E-02	8.9E-02	23.358	0.898	22.389	24.161	25.210	1.287	23.800	26.323
ENSG00000115216	NRBP1	-1.1	1.7E-02	3.0E-02	80.850	1.512	79.530	82.500	86.632	1.930	85.113	88.804
ENSG00000115286	NDUFS7	-1.1	2.7E-02	4.6E-02	10.441	0.730	9.891	11.269	11.552	0.558	11.123	12.183
ENSG00000115306	SPTBN1	-1.1	1.2E-05	4.3E-05	64.888	0.614	64.179	65.247	73.180	1.414	72.143	74.791
ENSG00000115307	AUP1	-1.1	3.4E-02	5.6E-02	48.719	2.530	47.018	51.626	53.003	1.663	51.389	54.710
ENSG00000115364	MRPL19	-1.1	9.7E-05	2.9E-04	54.506	0.230	54.259	54.715	62.238	0.915	61.320	63.150
ENSG00000115368	WDR75	-1.1	1.2E-04	3.6E-04	76.272	2.062	74.047	78.119	86.070	1.566	84.977	87.863
ENSG00000115392	FANCL	-1.1	4.8E-02	7.7E-02	65.713	4.621	62.712	71.035	71.577	3.588	67.498	74.246
ENSG00000115464	USP34	-1.1	1.6E-02	3.0E-02	30.861	1.033	29.899	31.953	33.325	0.973	32.223	34.064
ENSG00000115514	TXNDC9	-1.1	5.6E-02	8.9E-02	39.574	1.051	38.368	40.293	43.220	1.466	41.938	44.818
ENSG00000115540	MOB4	-1.1	7.5E-03	1.5E-02	39.985	2.080	38.228	42.282	44.425	3.438	41.693	48.286
ENSG00000115541	HSPE1	-1.1	4.1E-04	1.1E-03	201.732	6.591	195.493	208.626	225.897	6.748	218.130	230.329
ENSG00000115677	HDLBP	-1.1	1.5E-02	2.8E-02	98.284	0.919	97.269	99.059	104.767	3.052	102.307	108.182
ENSG00000115687	PASK	-1.1	5.8E-02	9.1E-02	15.717	0.269	15.412	15.919	16.801	0.488	16.392	17.342
ENSG00000115762	PLEKHB2	-1.1	9.3E-03	1.8E-02	54.453	2.030	52.528	56.573	59.002	1.088	57.947	60.120
ENSG00000115970	THADA	-1.1	2.3E-03	5.1E-03	10.033	0.664	9.394	10.719	11.414	0.360	11.172	11.827
ENSG00000115977	AAK1	-1.1	1.0E-01	1.5E-01	3.714	0.238	3.439	3.859	4.041	0.328	3.707	4.362
ENSG00000116017	ARID3A	-1.1	4.1E-04	1.1E-03	27.362	1.116	26.074	28.043	31.268	1.053	30.069	32.038
ENSG00000116161	CACYBP	-1.1	9.2E-05	2.7E-04	82.721	0.734	81.927	83.376	93.643	1.136	92.336	94.399
ENSG00000116191	RALGPS2	-1.1	6.3E-04	1.6E-03	27.225	1.169	25.949	28.244	30.491	0.102	30.413	30.607
ENSG00000116199	FAM20B	-1.1	2.5E-02	4.4E-02	35.148	0.731	34.493	35.937	38.132	2.260	35.609	39.973
ENSG00000116212	LRRC42	-1.1	2.0E-02	3.5E-02	63.972	2.085	61.569	65.305	69.813	0.982	68.718	70.617
ENSG00000116221	MRPL37	-1.1	5.0E-02	7.9E-02	110.824	2.266	108.268	112.586	117.863	4.202	114.206	122.453
ENSG00000116273	PHF13	-1.1	6.2E-02	9.6E-02	38.857	0.517	38.395	39.415	41.730	1.806	40.225	43.732
ENSG00000116489	CAPZA1	-1.1	5.6E-02	8.8E-02	110.704	0.900	110.027	111.725	116.963	2.051	114.604	118.329
ENSG00000116580	GON4L	-1.1	1.2E-03	2.9E-03	15.878	0.540	15.443	16.483	17.806	0.378	17.549	18.240
ENSG00000116586	LAMTOR2	-1.1	3.0E-02	5.0E-02	42.823	3.747	39.650	46.957	47.890	0.528	47.320	48.362
ENSG00000116649	SRM	-1.1	1.4E-01	1.9E-01	103.113	4.269	100.085	107.995	109.622	9.844	99.062	118.545
ENSG00000116871	MAP7D1	-1.1	2.7E-02	4.6E-02	21.342	0.238	21.068	21.495	23.933	2.510	21.572	26.569
ENSG00000116906	GNPAT	-1.1	8.8E-03	1.7E-02	63.546	2.794	60.951	66.503	69.586	1.124	68.325	70.480
ENSG00000116957	TBCE	-1.1	9.6E-02	1.4E-01	41.052	1.281	39.729	42.286	44.083	2.266	42.265	46.622
ENSG00000117016	RIMS3	-1.1	2.9E-02	4.9E-02	24.488	0.563	23.839	24.836	26.792	1.493	25.783	28.507
ENSG00000117118	SDHB	-1.1	8.2E-03	1.6E-02	67.043	4.067	64.336	71.720	74.725	2.156	73.305	77.206
ENSG00000117174	ZNHIT6	-1.1	1.4E-02	2.5E-02	16.021	0.347	15.690	16.382	17.819	0.871	17.076	18.777
ENSG00000117322	CR2	-1.1	1.2E-01	1.7E-01	4.251	0.127	4.104	4.329	4.772	0.291	4.604	5.108
ENSG00000117411	B4GALT2	-1.1	6.8E-02	1.0E-01	39.605	1.264	38.562	41.010	42.878	3.149	39.440	45.623
ENSG00000117523	PRRC2C	-1.1	1.2E-03	2.8E-03	87.321	4.906	82.036	91.731	95.566	1.836	94.463	97.685
ENSG00000117593	DARS2	-1.1	1.0E-01	1.5E-01	52.976	0.229	52.727	53.179	55.974	1.285	54.499	56.851
ENSG00000117650	NEK2	-1.1	4.7E-02	7.5E-02	33.406	1.910	31.979	35.575	36.612	2.947	33.298	38.939
ENSG00000117697	NSL1	-1.1	9.0E-03	1.7E-02	27.483	0.086	27.388	27.556	30.688	1.358	29.132	31.548
ENSG00000117724	CENPF	-1.1	4.4E-02	7.2E-02	112.638	9.472	101.733	118.809	124.735	8.070	119.077	133.976
ENSG00000117906	RCN2	-1.1	4.3E-05	1.4E-04	59.405	2.944	56.200	61.989	67.648	3.179	63.996	69.795
ENSG00000117984	CTSD	-1.1	3.5E-02	5.9E-02	33.456	2.059	31.363	35.479	36.966	3.194	34.073	40.394
ENSG00000118046	STK11	-1.1	5.6E-02	8.8E-02	18.081	0.795	17.259	18.846	19.521	0.831	18.870	20.457
ENSG00000118260	CREB1	-1.1	1.2E-02	2.2E-02	28.843	1.708	27.130	30.546	31.622	1.225	30.220	32.489
ENSG00000118363	SPCS2	-1.1	8.7E-03	1.7E-02	41.031	1.526	39.283	42.090	45.903	2.494	43.556	48.522
ENSG00000118412	CASP8AP2	-1.1	7.0E-03	1.4E-02	16.624	2.226	14.054	17.958	18.940	0.659	18.474	19.694
ENSG00000118418	HMGN3	-1.1	1.5E-03	3.5E-03	295.041	20.473	275.514	316.345	331.623	11.447	318.482	339.426
ENSG00000118454	ANKRD13C	-1.1	2.2E-02	3.8E-02	19.097	1.296	17.870	20.453	21.415	0.413	20.946	21.724
ENSG00000118762	PKD2	-1.1	1.8E-02	3.2E-02	32.209	2.435	29.421	33.922	35.299	1.875	33.284	36.993
ENSG00000119139	TJP2	-1.1	6.3E-02	9.8E-02	56.173	0.505	55.593	56.515	59.187	1.150	58.208	60.454
ENSG00000119318	RAD23B	-1.1	3.4E-02	5.7E-02	215.431	4.549	212.025	220.598	228.183	9.639	217.116	234.746
ENSG00000119326	CTNNAL1	-1.1	3.5E-02	5.8E-02	100.749	0.572	100.369	101.406	107.246	1.027	106.107	108.101
ENSG00000119335	SET	-1.1	2.3E-02	4.0E-02	993.395	19.297	971.874	1009.156	1050.656	35.783	1009.507	1074.476
ENSG00000119383	PTPA	-1.1	9.5E-03	1.8E-02	74.332	3.817	72.126	78.739	82.065	0.836	81.121	82.710
ENSG00000119421	NDUFA8	-1.1	8.5E-02	1.3E-01	129.537	6.746	124.329	137.158	139.268	4.822	135.716	144.757
ENSG00000119487	MAPKAP1	-1.1	5.3E-03	1.1E-02	54.480	0.562	53.991	55.094	59.273	0.906	58.229	59.857
ENSG00000119523	ALG2	-1.1	1.9E-02	3.4E-02	27.235	1.068	26.228	28.355	30.382	1.469	28.844	31.771
ENSG00000119541	VPS4B	-1.1	2.8E-03	6.0E-03	29.973	0.771	29.116	30.612	33.778	0.596	33.117	34.277
ENSG00000119616	FCF1	-1.1	3.2E-04	8.3E-04	50.147	1.917	48.539	52.268	57.816	4.928	53.028	62.872
ENSG00000119638	NEK9	-1.1	8.9E-04	2.1E-03	18.604	0.366	18.186	18.870	20.792	0.541	20.331	21.388
ENSG00000119661	DNAL1	-1.1	1.5E-02	2.7E-02	6.918	0.173	6.788	7.115	7.886	0.639	7.229	8.506
ENSG00000119689	DLST	-1.1	3.7E-02	6.0E-02	70.997	1.264	69.547	71.866	75.786	0.410	75.324	76.105
ENSG00000119705	SLIRP	-1.1	2.6E-04	7.0E-04	111.415	2.818	109.508	114.652	125.911	5.980	119.380	131.119
ENSG00000119778	ATAD2B	-1.1	2.9E-02	5.0E-02	9.045	0.250	8.849	9.327	9.943	0.504	9.501	10.491
ENSG00000119979	FAM45A	-1.1	5.1E-02	8.1E-02	16.450	0.304	16.272	16.801	17.970	0.373	17.689	18.393
ENSG00000120008	WDR11	-1.1	3.9E-03	8.1E-03	18.983	0.536	18.438	19.510	20.940	0.566	20.381	21.513
ENSG00000120509	PDZD11	-1.1	1.9E-02	3.4E-02	40.669	1.828	38.745	42.382	46.147	0.611	45.577	46.792
ENSG00000120594	PLXDC2	-1.1	2.5E-03	5.5E-03	9.568	0.085	9.494	9.661	10.912	0.588	10.243	11.349
ENSG00000120685	PROSER1	-1.1	3.0E-03	6.4E-03	56.305	3.708	53.827	60.568	62.129	0.819	61.196	62.728
ENSG00000120733	KDM3B	-1.1	2.7E-02	4.7E-02	64.189	1.007	63.263	65.262	68.934	2.810	65.855	71.359
ENSG00000120742	SERP1	-1.1	6.6E-06	2.5E-05	80.249	0.098	80.142	80.334	92.481	3.171	90.638	96.142
ENSG00000120948	TARDBP	-1.1	4.6E-04	1.2E-03	95.196	1.702	93.499	96.903	106.271	6.828	98.522	111.406
ENSG00000121067	SPOP	-1.1	5.7E-02	8.9E-02	11.176	0.404	10.813	11.611	12.291	0.739	11.560	13.038
ENSG00000121406	ZNF549	-1.1	5.1E-02	8.1E-02	5.268	0.438	4.815	5.689	5.912	0.238	5.643	6.094
ENSG00000121766	ZCCHC17	-1.1	2.1E-04	5.7E-04	97.609	1.603	95.855	98.998	111.061	0.895	110.098	111.867
ENSG00000122376	FAM35A	-1.1	1.3E-02	2.5E-02	38.128	0.854	37.471	39.094	41.839	1.598	40.595	43.641
ENSG00000122435	TRMT10C	-1.1	4.2E-02	6.8E-02	13.635	0.881	12.847	14.587	15.317	1.036	14.286	16.358
ENSG00000122483	CCDC18	-1.1	5.0E-02	8.0E-02	11.530	1.215	10.412	12.823	13.018	1.325	11.706	14.355
ENSG00000122484	RPAP2	-1.1	2.9E-03	6.3E-03	4.922	0.121	4.786	5.019	5.667	0.307	5.347	5.960
ENSG00000122565	CBX3	-1.1	2.7E-04	7.3E-04	176.453	1.442	174.808	177.496	196.895	10.394	186.498	207.285
ENSG00000122566	HNRNPA2B1	-1.1	3.0E-02	5.1E-02	829.487	38.688	786.379	861.192	877.183	29.161	853.418	909.724
ENSG00000122674	CCZ1	-1.1	5.5E-04	1.4E-03	36.892	0.859	35.942	37.616	41.524	0.646	40.998	42.246
ENSG00000122692	SMU1	-1.1	4.8E-03	9.7E-03	37.962	2.273	36.500	40.581	41.848	1.546	40.221	43.297
ENSG00000122741	DCAF10	-1.1	3.9E-02	6.4E-02	9.161	0.561	8.623	9.742	10.154	0.200	9.967	10.341
ENSG00000122882	ECD	-1.1	7.6E-04	1.9E-03	49.087	0.319	48.735	49.356	55.971	1.886	54.254	57.922
ENSG00000122958	VPS26A	-1.1	1.4E-03	3.3E-03	45.430	0.421	45.027	45.867	51.318	2.926	48.616	54.426
ENSG00000123064	DDX54	-1.1	2.8E-02	4.8E-02	59.713	2.385	57.667	62.333	64.309	4.759	59.500	69.016
ENSG00000123124	WWP1	-1.1	2.7E-03	5.8E-03	40.123	0.933	39.478	41.194	44.479	1.802	42.463	45.932
ENSG00000123144	C19orf43	-1.1	4.0E-02	6.5E-02	142.996	8.623	134.422	151.668	155.351	11.763	145.467	168.362
ENSG00000123191	ATP7B	-1.1	5.2E-02	8.3E-02	3.604	0.273	3.308	3.846	4.049	0.148	3.885	4.172
ENSG00000123200	ZC3H13	-1.1	2.3E-02	4.0E-02	53.609	3.914	49.620	57.443	57.502	1.606	55.648	58.447
ENSG00000123213	NLN	-1.1	2.3E-05	7.8E-05	83.864	1.020	82.828	84.866	94.314	2.851	91.956	97.482
ENSG00000123219	CENPK	-1.1	8.3E-02	1.2E-01	30.274	2.109	27.992	32.150	32.670	2.379	30.232	34.987
ENSG00000123453	SARDH	-1.1	4.9E-02	7.8E-02	5.118	0.268	4.854	5.390	5.855	0.314	5.602	6.206
ENSG00000123684	LPGAT1	-1.1	2.2E-03	4.8E-03	11.409	0.380	11.077	11.823	13.046	0.123	12.915	13.158
ENSG00000123815	COQ8B	-1.1	6.7E-02	1.0E-01	13.164	0.749	12.307	13.697	14.390	0.621	14.013	15.107
ENSG00000124104	SNX21	-1.1	1.3E-01	1.9E-01	7.087	0.264	6.784	7.266	7.904	0.770	7.088	8.619
ENSG00000124151	NCOA3	-1.1	8.1E-02	1.2E-01	16.140	0.248	15.908	16.402	17.211	0.602	16.516	17.573
ENSG00000124177	CHD6	-1.1	7.6E-03	1.5E-02	22.278	1.506	20.542	23.242	24.415	0.563	23.817	24.934
ENSG00000124207	CSE1L	-1.1	1.2E-04	3.5E-04	399.630	15.419	382.262	411.706	444.460	7.113	436.503	450.200
ENSG00000124212	PTGIS	-1.1	7.7E-02	1.2E-01	25.264	0.360	24.887	25.603	27.124	1.482	25.789	28.719
ENSG00000124214	STAU1	-1.1	3.9E-04	1.0E-03	118.855	0.578	118.369	119.494	131.732	2.865	128.429	133.535
ENSG00000124228	DDX27	-1.1	2.2E-04	6.1E-04	42.287	1.255	41.211	43.666	48.049	2.579	45.129	50.014
ENSG00000124440	HIF3A	-1.1	7.5E-02	1.1E-01	3.982	0.191	3.761	4.094	4.456	0.108	4.372	4.577
ENSG00000124486	USP9X	-1.1	2.7E-04	7.2E-04	94.660	1.636	92.816	95.936	109.074	2.937	107.048	112.443
ENSG00000124562	SNRPC	-1.1	1.7E-02	3.1E-02	279.929	8.339	270.490	286.297	300.570	4.607	295.456	304.397
ENSG00000124571	XPO5	-1.1	2.3E-03	5.0E-03	54.982	1.155	53.658	55.784	60.232	1.749	58.518	62.014
ENSG00000124574	ABCC10	-1.1	9.3E-02	1.4E-01	5.765	0.649	5.022	6.227	6.358	0.324	6.140	6.731
ENSG00000124782	RREB1	-1.1	6.1E-03	1.2E-02	5.912	0.175	5.711	6.030	6.732	0.503	6.153	7.057
ENSG00000124786	SLC35B3	-1.1	4.8E-02	7.8E-02	18.322	1.551	16.540	19.362	20.799	0.613	20.100	21.244
ENSG00000124789	NUP153	-1.1	1.2E-05	4.2E-05	84.767	2.990	81.429	87.199	97.123	1.064	96.021	98.145
ENSG00000124802	EEF1E1	-1.1	1.7E-02	3.1E-02	50.112	1.853	47.982	51.355	55.986	3.590	53.846	60.132
ENSG00000125247	TMTC4	-1.1	4.3E-02	7.0E-02	25.610	1.253	24.212	26.631	27.573	0.653	26.971	28.266
ENSG00000125257	ABCC4	-1.1	1.8E-02	3.3E-02	8.458	0.740	7.765	9.238	9.618	0.103	9.530	9.731
ENSG00000125304	TM9SF2	-1.1	6.5E-02	1.0E-01	129.241	1.756	127.265	130.622	137.122	2.644	134.151	139.217
ENSG00000125534	PPDPF	-1.1	1.6E-02	2.8E-02	138.002	6.377	130.750	142.734	157.187	19.301	138.156	176.747
ENSG00000125676	THOC2	-1.1	2.2E-03	4.9E-03	29.123	1.546	27.379	30.327	32.373	0.878	31.487	33.243
ENSG00000125686	MED1	-1.1	8.7E-04	2.1E-03	40.103	1.457	38.450	41.201	44.648	1.689	42.726	45.894
ENSG00000125730	C3	-1.1	1.1E-01	1.6E-01	4.217	0.472	3.688	4.596	4.693	0.374	4.328	5.076
ENSG00000125733	TRIP10	-1.1	1.0E-02	1.9E-02	44.347	1.548	43.413	46.134	48.954	3.363	45.674	52.394
ENSG00000125779	PANK2	-1.1	8.7E-03	1.7E-02	17.075	0.659	16.455	17.767	18.983	0.520	18.513	19.541
ENSG00000125827	TMX4	-1.1	1.3E-01	1.8E-01	7.488	0.563	7.032	8.117	8.146	0.464	7.622	8.505
ENSG00000125834	STK35	-1.1	4.6E-02	7.4E-02	28.108	0.429	27.697	28.553	30.132	0.887	29.324	31.080
ENSG00000125835	SNRPB	-1.1	8.3E-04	2.0E-03	547.353	18.796	528.918	566.489	604.067	6.511	599.085	611.435
ENSG00000125846	ZNF133	-1.1	1.5E-02	2.8E-02	9.203	0.503	8.726	9.729	10.493	0.485	10.131	11.044
ENSG00000125871	MGME1	-1.1	2.4E-02	4.2E-02	81.503	3.805	78.093	85.607	88.436	3.477	85.168	92.089
ENSG00000125885	MCM8	-1.1	4.0E-02	6.5E-02	22.373	0.402	21.995	22.795	24.099	0.407	23.847	24.569
ENSG00000126456	IRF3	-1.1	1.8E-03	4.1E-03	39.542	1.814	38.486	41.637	45.617	2.373	43.040	47.712
ENSG00000126581	BECN1	-1.1	3.3E-03	6.9E-03	20.657	0.484	20.358	21.215	23.066	0.480	22.780	23.620
ENSG00000126602	TRAP1	-1.1	5.6E-02	8.8E-02	101.923	2.118	99.602	103.750	107.636	3.406	104.893	111.448
ENSG00000126705	AHDC1	-1.1	4.8E-02	7.7E-02	10.053	0.693	9.489	10.827	11.117	0.645	10.510	11.794
ENSG00000126749	EMG1	-1.1	1.1E-01	1.6E-01	9.008	0.723	8.176	9.478	9.826	0.523	9.429	10.419
ENSG00000126775	ATG14	-1.1	1.2E-01	1.7E-01	18.214	0.949	17.253	19.149	19.604	1.055	18.705	20.765
ENSG00000126878	AIF1L	-1.1	2.5E-02	4.2E-02	137.552	0.876	136.606	138.334	146.453	4.199	141.613	149.109
ENSG00000127314	RAP1B	-1.1	3.8E-02	6.2E-02	10.760	0.658	10.179	11.474	11.691	0.708	10.926	12.323
ENSG00000127334	DYRK2	-1.1	7.1E-03	1.4E-02	18.477	0.346	18.241	18.875	20.565	1.535	18.816	21.687
ENSG00000127423	AUNIP	-1.1	9.9E-03	1.9E-02	26.464	0.554	25.832	26.867	30.467	0.774	29.643	31.178
ENSG00000127445	PIN1	-1.1	7.6E-02	1.2E-01	22.780	0.251	22.557	23.051	24.538	0.606	23.842	24.945
ENSG00000127540	UQCR11	-1.1	4.2E-03	8.7E-03	38.403	2.603	36.753	41.404	43.948	1.268	43.008	45.391
ENSG00000127554	GFER	-1.1	3.1E-02	5.2E-02	26.890	1.472	25.849	28.574	29.958	2.101	27.709	31.870
ENSG00000127589	TUBBP1	-1.1	6.7E-03	1.3E-02	232.742	13.644	224.525	248.492	255.217	16.386	243.446	273.932
ENSG00000127603	MACF1	-1.1	3.7E-02	6.1E-02	34.989	1.656	33.167	36.405	37.368	0.830	36.672	38.287
ENSG00000127616	SMARCA4	-1.1	2.5E-03	5.4E-03	70.139	0.650	69.404	70.640	76.935	5.555	72.375	83.121
ENSG00000127884	ECHS1	-1.1	1.6E-04	4.6E-04	127.855	4.557	123.281	132.394	147.039	2.251	144.458	148.592
ENSG00000127955	GNAI1	-1.1	1.5E-02	2.7E-02	15.194	0.326	14.947	15.564	17.092	1.121	16.171	18.340
ENSG00000128000	ZNF780B	-1.1	2.3E-02	3.9E-02	5.123	0.467	4.689	5.618	5.903	0.153	5.748	6.055
ENSG00000128045	RASL11B	-1.1	5.3E-02	8.4E-02	71.048	0.603	70.654	71.742	79.449	4.039	74.856	82.445
ENSG00000128059	PPAT	-1.1	4.8E-02	7.7E-02	79.863	2.308	78.131	82.483	84.895	4.297	80.712	89.299
ENSG00000128159	TUBGCP6	-1.1	2.7E-02	4.6E-02	9.476	0.949	8.531	10.429	10.601	0.529	10.130	11.173
ENSG00000128595	CALU	-1.1	3.8E-04	9.9E-04	189.812	4.460	186.576	194.900	209.836	2.634	207.414	212.640
ENSG00000128833	MYO5C	-1.1	1.5E-03	3.5E-03	11.186	0.219	10.956	11.392	12.761	0.694	12.345	13.562
ENSG00000129187	DCTD	-1.1	1.1E-01	1.6E-01	51.216	1.691	49.392	52.733	54.265	1.708	52.796	56.140
ENSG00000129219	PLD2	-1.1	8.5E-02	1.3E-01	18.830	1.077	17.686	19.825	20.414	1.048	19.587	21.593
ENSG00000129244	ATP1B2	-1.1	9.2E-02	1.4E-01	8.814	0.471	8.275	9.140	10.070	1.152	8.741	10.772
ENSG00000129292	PHF20L1	-1.1	4.8E-02	7.7E-02	5.795	0.272	5.587	6.103	6.288	0.084	6.191	6.349
ENSG00000129317	PUS7L	-1.1	6.4E-02	9.9E-02	11.784	0.763	10.996	12.520	12.686	0.591	12.143	13.315
ENSG00000129347	KRI1	-1.1	3.3E-03	6.9E-03	22.228	1.068	21.233	23.357	25.549	1.746	24.020	27.451
ENSG00000129534	MIS18BP1	-1.1	7.1E-02	1.1E-01	62.496	4.088	58.060	66.111	66.688	3.492	64.232	70.693
ENSG00000129595	EPB41L4A	-1.1	1.4E-01	1.9E-01	2.072	0.228	1.872	2.320	2.352	0.153	2.228	2.523
ENSG00000129911	KLF16	-1.1	4.8E-02	7.8E-02	28.647	1.416	27.057	29.772	31.504	1.173	30.443	32.764
ENSG00000130202	NECTIN2	-1.1	7.2E-05	2.2E-04	68.618	1.698	67.474	70.568	79.058	2.748	75.921	81.034

ENSG00000130204	TOMM40	-1.1	6.4E-02	9.9E-02	77.458	2.253	76.041	80.055	82.755	5.479	79.134	89.059
ENSG00000130227	XPO7	-1.1	8.0E-06	2.9E-05	80.114	0.654	79.418	80.715	91.936	1.815	90.652	94.013
ENSG00000130396	AFDN	-1.1	5.1E-06	1.9E-05	52.085	1.289	50.636	53.102	59.508	1.506	58.024	61.035
ENSG00000130559	CAMSAP1	-1.1	3.4E-02	5.7E-02	22.802	0.183	22.593	22.935	24.516	1.036	23.470	25.542
ENSG00000130560	UBAC1	-1.1	2.1E-02	3.7E-02	40.484	3.241	37.978	44.144	45.009	3.107	42.475	48.475
ENSG00000130726	TRIM28	-1.1	8.2E-03	1.6E-02	395.488	5.251	389.939	400.373	432.599	25.783	402.997	450.158
ENSG00000130731	METTL26	-1.1	9.8E-02	1.4E-01	45.213	0.980	44.083	45.842	49.192	5.088	43.433	53.129
ENSG00000130733	YIPF2	-1.1	3.5E-02	5.9E-02	27.150	0.538	26.559	27.613	30.078	2.157	27.960	32.273
ENSG00000130764	LRRC47	-1.1	7.1E-02	1.1E-01	60.380	1.127	59.234	61.487	64.387	3.210	60.688	66.436
ENSG00000130803	ZNF317	-1.1	2.5E-03	5.4E-03	19.617	0.485	19.068	19.991	22.406	0.955	21.762	23.503
ENSG00000130816	DNMT1	-1.1	1.2E-05	4.3E-05	55.809	1.358	55.021	57.377	63.660	1.565	62.201	65.314
ENSG00000130818	ZNF426	-1.1	1.2E-02	2.2E-02	12.840	0.202	12.650	13.052	14.419	1.350	13.266	15.904
ENSG00000130826	DKC1	-1.1	4.0E-02	6.5E-02	124.083	2.832	121.266	126.930	131.800	2.646	128.965	134.204
ENSG00000131115	ZNF227	-1.1	1.7E-02	3.0E-02	12.657	0.779	11.944	13.489	14.280	0.315	13.916	14.473
ENSG00000131116	ZNF428	-1.1	9.5E-02	1.4E-01	16.896	1.308	16.115	18.405	18.790	0.679	18.091	19.446
ENSG00000131171	SH3BGRL	-1.1	9.8E-02	1.4E-01	91.743	4.572	86.553	95.179	97.436	5.957	90.908	102.576
ENSG00000131236	CAP1	-1.1	1.9E-03	4.2E-03	205.160	2.552	202.357	207.350	223.699	4.437	221.115	228.822
ENSG00000131242	RAB11FIP4	-1.1	2.2E-04	6.1E-04	17.856	0.576	17.294	18.445	20.308	0.649	19.560	20.719
ENSG00000131351	HAUS8	-1.1	6.8E-02	1.0E-01	5.770	0.633	5.398	6.502	6.510	0.241	6.337	6.784
ENSG00000131368	MRPS25	-1.1	2.4E-02	4.1E-02	28.599	0.554	27.983	29.055	31.032	0.397	30.662	31.451
ENSG00000131470	PSMC3IP	-1.1	9.5E-02	1.4E-01	17.760	0.579	17.277	18.402	19.537	1.318	18.469	21.010
ENSG00000131475	VPS25	-1.1	7.6E-03	1.5E-02	78.340	3.727	74.228	81.497	86.107	1.864	84.196	87.921
ENSG00000131508	UBE2D2	-1.1	6.1E-02	9.4E-02	86.126	1.068	85.088	87.223	91.300	2.868	88.275	93.979
ENSG00000131788	PIAS3	-1.1	3.3E-03	6.9E-03	17.902	0.298	17.603	18.199	20.534	0.642	19.798	20.972
ENSG00000131797	CLUHP3	-1.1	3.6E-02	5.9E-02	4.390	0.309	4.036	4.604	5.042	0.134	4.921	5.186
ENSG00000131845	ZNF304	-1.1	1.7E-02	3.0E-02	9.949	0.085	9.896	10.047	11.315	0.235	11.103	11.568
ENSG00000131899	LLGL1	-1.1	6.1E-04	1.5E-03	44.954	0.694	44.505	45.753	50.451	1.318	49.684	51.973
ENSG00000131914	LIN28A	-1.1	3.9E-06	1.5E-05	989.258	4.142	984.655	992.683	1131.345	19.996	1110.657	1150.569
ENSG00000132017	DCAF15	-1.1	1.0E-02	1.9E-02	38.443	2.854	35.199	40.566	42.984	1.273	41.515	43.778
ENSG00000132128	LRRC41	-1.1	5.8E-02	9.1E-02	17.711	0.373	17.282	17.953	19.008	0.485	18.520	19.490
ENSG00000132155	RAF1	-1.1	1.0E-04	3.0E-04	42.640	1.102	41.578	43.778	48.701	2.971	45.554	51.457
ENSG00000132254	ARFIP2	-1.1	1.1E-03	2.7E-03	23.029	1.719	21.124	24.465	26.482	0.658	25.876	27.182
ENSG00000132256	TRIM5	-1.1	6.3E-02	9.7E-02	16.619	1.439	15.743	18.280	18.213	0.533	17.797	18.813
ENSG00000132376	INPP5K	-1.1	1.2E-01	1.8E-01	7.184	1.040	6.029	8.047	8.027	0.334	7.660	8.314
ENSG00000132561	MATN2	-1.1	1.1E-01	1.6E-01	7.760	0.141	7.636	7.913	8.486	0.141	8.362	8.639
ENSG00000132581	SDF2	-1.1	6.6E-03	1.3E-02	20.707	0.803	20.217	21.633	23.668	1.610	22.713	25.528
ENSG00000132640	BTBD3	-1.1	1.3E-02	2.3E-02	34.878	1.089	33.725	35.889	37.914	1.093	36.652	38.557
ENSG00000132694	ARHGEF11	-1.1	1.6E-03	3.7E-03	15.137	0.201	14.962	15.357	17.241	0.078	17.168	17.323
ENSG00000132763	MMACHC	-1.1	6.4E-02	9.9E-02	4.021	0.150	3.847	4.108	4.608	0.296	4.351	4.932
ENSG00000132846	ZBED3	-1.1	8.1E-02	1.2E-01	4.752	0.760	4.028	5.544	5.406	0.141	5.318	5.568
ENSG00000132950	ZMYM5	-1.1	9.8E-02	1.4E-01	8.056	0.738	7.276	8.743	9.083	1.107	8.080	10.271
ENSG00000133030	MPRIP	-1.1	5.6E-03	1.1E-02	22.460	0.595	21.847	23.035	24.377	0.775	23.849	25.267
ENSG00000133059	DSTYK	-1.1	8.5E-03	1.6E-02	8.411	0.393	7.997	8.779	9.611	0.302	9.272	9.850
ENSG00000133104	SPG20	-1.1	1.5E-04	4.3E-04	28.289	1.064	27.090	29.124	32.531	0.172	32.347	32.688
ENSG00000133195	SLC39A11	-1.1	8.9E-02	1.3E-01	4.583	0.133	4.506	4.737	5.159	0.216	4.934	5.364
ENSG00000133265	HSPBP1	-1.1	1.2E-03	2.8E-03	51.552	1.820	50.147	53.608	59.330	1.235	58.295	60.697
ENSG00000133316	WDR74	-1.1	1.6E-03	3.6E-03	29.522	0.765	28.926	30.385	33.618	0.988	33.039	34.759
ENSG00000133318	RTN3	-1.1	3.0E-03	6.5E-03	97.345	2.113	95.096	99.288	106.649	2.412	104.558	109.288
ENSG00000133477	FAM83F	-1.1	3.8E-03	7.9E-03	5.155	0.084	5.086	5.249	5.889	0.061	5.833	5.953
ENSG00000133657	ATP13A3	-1.1	4.3E-04	1.1E-03	42.775	0.436	42.334	43.207	47.849	2.778	44.791	50.217
ENSG00000133704	IPO8	-1.1	1.0E-01	1.5E-01	45.437	2.080	43.193	47.300	48.001	2.382	45.618	50.383
ENSG00000133835	HSD17B4	-1.1	8.7E-03	1.7E-02	110.539	2.052	108.762	112.786	118.260	1.845	117.083	120.386
ENSG00000134001	EIF2S1	-1.1	2.1E-03	4.6E-03	54.988	0.552	54.491	55.581	60.465	0.845	59.506	61.103
ENSG00000134049	IER3IP1	-1.1	4.8E-02	7.6E-02	13.613	0.977	12.514	14.384	15.287	0.653	14.719	16.000
ENSG00000134086	VHL	-1.1	1.1E-01	1.6E-01	35.825	1.447	34.182	36.910	37.937	0.191	37.765	38.143
ENSG00000134215	VAV3	-1.1	1.4E-01	1.9E-01	6.723	0.866	5.749	7.409	7.346	0.094	7.243	7.425
ENSG00000134243	SORT1	-1.1	8.9E-02	1.3E-01	33.641	0.117	33.527	33.762	35.569	1.724	33.717	37.128
ENSG00000134283	PPHLN1	-1.1	2.2E-02	3.8E-02	17.339	0.544	16.927	17.956	18.769	0.468	18.368	19.284
ENSG00000134287	ARF3	-1.1	1.8E-05	6.2E-05	105.009	1.303	103.681	106.285	120.301	1.460	118.615	121.145
ENSG00000134461	ANKRD16	-1.1	8.1E-02	1.2E-01	6.793	0.249	6.509	6.972	7.753	0.538	7.132	8.083
ENSG00000134744	ZCCHC11	-1.1	5.8E-03	1.2E-02	10.444	0.550	9.890	10.989	11.580	0.644	10.926	12.211
ENSG00000134748	PRPF38A	-1.1	2.3E-02	3.9E-02	36.994	1.549	35.270	38.270	40.199	1.065	39.144	41.273
ENSG00000134755	DSC2	-1.1	1.1E-02	2.1E-02	13.011	0.821	12.389	13.942	14.540	1.263	13.282	15.808
ENSG00000134871	COL4A2	-1.1	5.9E-03	1.2E-02	46.719	1.998	44.500	48.376	51.608	3.689	48.071	55.431
ENSG00000134900	TPP2	-1.1	1.7E-04	4.7E-04	65.926	2.536	63.161	68.145	73.887	1.509	72.180	75.044
ENSG00000134910	STT3A	-1.1	1.3E-03	2.9E-03	56.866	2.074	55.225	59.198	62.742	1.418	61.790	64.372
ENSG00000134987	WDR36	-1.1	3.8E-02	6.2E-02	35.917	0.275	35.661	36.208	38.547	1.737	37.012	40.432
ENSG00000135049	AGTPBP1	-1.1	9.5E-04	2.3E-03	55.407	2.158	53.033	57.251	61.871	2.180	60.188	64.333
ENSG00000135052	GOLM1	-1.1	2.7E-05	8.9E-05	95.558	0.774	94.993	96.441	109.533	2.296	107.080	111.630
ENSG00000135083	CCNJL	-1.1	1.6E-02	2.8E-02	19.750	0.719	18.888	20.415	21.952	0.221	21.796	22.205
ENSG00000135185	TMEM243	-1.1	3.9E-02	6.5E-02	12.665	1.158	11.891	13.997	14.328	0.762	13.453	14.841
ENSG00000135297	MTO1	-1.1	5.2E-02	8.3E-02	7.105	0.553	6.560	7.665	7.784	0.412	7.309	8.043
ENSG00000135316	SYNCRIP	-1.1	1.5E-03	3.4E-03	151.710	4.156	148.026	156.215	166.730	5.403	160.992	171.720
ENSG00000135336	ORC3	-1.1	1.4E-03	3.2E-03	66.670	2.371	64.329	69.070	74.558	2.906	72.229	77.815
ENSG00000135404	CD63	-1.1	4.4E-03	9.0E-03	184.048	4.011	181.245	188.642	200.076	4.637	194.863	203.741
ENSG00000135446	CDK4	-1.1	2.1E-05	7.2E-05	130.290	2.174	127.786	131.684	149.178	1.209	147.782	149.878
ENSG00000135596	MICAL1	-1.1	5.1E-03	1.0E-02	16.574	0.735	15.969	17.391	18.699	0.855	18.072	19.674
ENSG00000135597	REPS1	-1.1	6.8E-04	1.7E-03	18.687	0.460	18.156	18.954	21.145	0.261	20.963	21.444
ENSG00000135632	SMYD5	-1.1	1.2E-02	2.3E-02	52.882	3.124	49.368	55.346	58.130	0.165	57.942	58.257
ENSG00000135720	DYNC1LI2	-1.1	1.1E-04	3.2E-04	44.088	2.448	41.319	45.964	50.184	1.660	48.397	51.679
ENSG00000135842	FAM129A	-1.1	4.6E-02	7.5E-02	13.135	0.886	12.113	13.688	14.364	0.369	14.089	14.783
ENSG00000135932	CAB39	-1.1	9.1E-02	1.3E-01	54.495	2.528	52.006	57.060	57.641	2.703	55.283	60.591
ENSG00000135972	MRPS9	-1.1	2.2E-04	6.1E-04	128.178	7.299	121.379	135.891	147.175	2.643	144.124	148.742
ENSG00000136100	VPS36	-1.1	2.8E-02	4.8E-02	17.446	1.309	15.946	18.360	19.397	1.248	18.156	20.652
ENSG00000136108	CKAP2	-1.1	1.6E-03	3.7E-03	74.582	4.013	70.470	78.489	83.316	2.769	81.597	86.510
ENSG00000136169	SETDB2	-1.1	8.3E-02	1.2E-01	5.472	0.700	4.669	5.955	6.206	0.574	5.585	6.718
ENSG00000136247	ZDHHC4	-1.1	2.4E-02	4.2E-02	24.277	1.041	23.091	25.038	26.886	1.291	25.929	28.354
ENSG00000136261	BZW2	-1.1	7.2E-02	1.1E-01	100.405	4.063	95.788	103.433	106.094	3.779	102.497	110.032
ENSG00000136270	TBRG4	-1.1	9.5E-05	2.8E-04	47.405	1.440	45.746	48.329	54.059	1.276	52.872	55.408
ENSG00000136425	CIB2	-1.1	7.6E-02	1.2E-01	45.687	2.594	43.514	48.560	49.699	5.337	44.002	54.583
ENSG00000136518	ACTL6A	-1.1	1.7E-03	3.8E-03	82.109	2.074	80.166	84.293	90.967	0.547	90.362	91.428
ENSG00000136522	MRPL47	-1.1	3.9E-04	1.0E-03	136.733	6.189	129.788	141.668	156.970	10.150	146.415	166.659
ENSG00000136536	MARCH7	-1.1	3.8E-02	6.3E-02	63.964	2.493	62.457	66.842	68.493	3.468	66.251	72.488
ENSG00000136813	KIAA0368	-1.1	2.5E-05	8.4E-05	74.183	1.725	72.288	75.662	84.910	2.541	82.566	87.610
ENSG00000136828	RALGPS1	-1.1	1.4E-01	2.0E-01	7.550	0.380	7.301	7.987	8.049	0.087	7.990	8.149
ENSG00000136897	MRPL50	-1.1	1.4E-03	3.2E-03	59.006	1.372	57.441	59.998	66.219	3.210	62.513	68.123
ENSG00000136940	PDCL	-1.1	5.1E-02	8.1E-02	25.906	1.984	23.689	27.516	28.350	0.828	27.428	29.030
ENSG00000137040	RANBP6	-1.1	3.0E-03	6.5E-03	36.611	1.198	35.430	37.826	41.087	2.939	38.234	44.104
ENSG00000137073	UBAP2	-1.1	5.7E-02	9.0E-02	17.295	0.739	16.467	17.887	18.486	0.461	17.967	18.849
ENSG00000137269	LRRC1	-1.1	3.6E-02	6.0E-02	11.175	0.104	11.104	11.295	12.448	0.320	12.095	12.719
ENSG00000137337	MDC1	-1.1	5.8E-05	1.8E-04	35.789	0.471	35.282	36.214	41.225	1.977	39.218	43.171
ENSG00000137404	NRM	-1.1	7.0E-02	1.1E-01	21.578	0.916	20.907	22.622	24.049	2.158	21.585	25.605
ENSG00000137497	NUMA1	-1.1	2.2E-04	6.1E-04	24.950	0.173	24.779	25.124	28.067	0.716	27.566	28.887
ENSG00000137522	RNF121	-1.1	2.6E-02	4.5E-02	16.640	1.641	14.842	18.057	18.785	1.115	18.006	20.062
ENSG00000137571	SLCO5A1	-1.1	9.1E-02	1.3E-01	1.710	0.059	1.665	1.776	1.960	0.079	1.870	2.012
ENSG00000137642	SORL1	-1.1	2.5E-02	4.2E-02	29.408	0.545	28.935	30.004	31.333	0.174	31.150	31.496
ENSG00000137710	RDX	-1.1	1.6E-03	3.6E-03	46.056	1.931	43.961	47.766	51.172	2.539	48.933	53.930
ENSG00000137776	SLTM	-1.1	1.3E-02	2.3E-02	70.201	4.280	66.360	74.815	78.269	1.197	77.200	79.563
ENSG00000137812	KNL1	-1.1	1.4E-01	2.0E-01	16.921	0.704	16.278	17.673	17.958	0.357	17.547	18.194
ENSG00000137814	HAUS2	-1.1	6.0E-03	1.2E-02	24.173	1.574	22.979	25.957	27.783	2.490	25.266	30.245
ENSG00000137824	RMDN3	-1.1	6.0E-02	9.4E-02	12.762	0.416	12.317	13.140	14.003	0.760	13.384	14.852
ENSG00000137947	GTF2B	-1.1	3.5E-02	5.8E-02	18.835	1.205	17.727	20.117	21.381	0.383	21.105	21.818
ENSG00000137996	RTCA	-1.1	8.8E-02	1.3E-01	38.196	1.253	37.202	39.604	40.911	1.393	39.303	41.757
ENSG00000138107	ACTR1A	-1.1	3.3E-04	8.8E-04	75.107	1.044	74.463	76.312	84.339	4.432	80.079	88.926
ENSG00000138111	MFSD13A	-1.1	4.7E-02	7.5E-02	8.481	0.410	8.065	8.885	9.630	0.502	9.330	10.210
ENSG00000138162	TACC2	-1.1	4.8E-02	7.7E-02	4.891	0.194	4.746	5.111	5.342	0.118	5.211	5.439
ENSG00000138279	ANXA7	-1.1	2.6E-03	5.6E-03	83.591	2.274	81.216	85.749	92.428	1.806	90.384	93.809
ENSG00000138363	ATIC	-1.1	2.2E-03	4.9E-03	158.565	4.876	153.799	163.544	172.474	1.471	171.161	174.063
ENSG00000138381	ASNSD1	-1.1	3.0E-03	6.3E-03	66.950	4.539	61.712	69.731	75.299	1.377	73.954	76.706
ENSG00000138385	SSB	-1.1	1.5E-03	3.4E-03	212.242	9.622	202.020	221.122	233.169	8.473	226.136	242.576
ENSG00000138434	SSFA2	-1.1	2.3E-02	4.0E-02	4.971	0.147	4.862	5.139	5.691	0.606	5.009	6.167
ENSG00000138459	SLC35A5	-1.1	2.8E-02	4.7E-02	21.554	1.014	20.451	22.443	23.543	0.400	23.097	23.871
ENSG00000138688	KIAA1109	-1.1	2.5E-02	4.3E-02	15.079	0.936	14.032	15.837	16.274	0.713	15.655	17.053
ENSG00000138698	RAP1GDS1	-1.1	7.4E-02	1.1E-01	19.763	1.545	18.053	21.058	21.586	1.759	20.024	23.490
ENSG00000138750	NUP54	-1.1	1.0E-01	1.5E-01	86.368	3.285	82.878	89.401	91.087	3.496	88.323	95.017
ENSG00000138757	G3BP2	-1.1	1.8E-02	3.2E-02	226.937	9.547	219.178	237.598	242.429	10.252	230.998	250.808
ENSG00000138767	CNOT6L	-1.1	5.8E-02	9.1E-02	8.826	0.531	8.280	9.340	9.709	0.748	9.200	10.568
ENSG00000138785	INTS12	-1.1	8.4E-02	1.3E-01	10.300	0.188	10.100	10.472	11.447	0.916	10.393	12.044
ENSG00000139112	GABARAPL1	-1.1	1.0E-03	2.5E-03	62.413	0.049	62.367	62.463	69.738	4.275	66.312	74.529
ENSG00000139178	C1RL	-1.1	7.1E-02	1.1E-01	8.819	0.881	7.855	9.582	9.746	0.354	9.337	9.954
ENSG00000139197	PEX5	-1.1	2.3E-02	4.0E-02	28.650	0.316	28.462	29.015	31.103	0.930	30.562	32.176
ENSG00000139209	SLC38A4	-1.1	8.2E-02	1.2E-01	24.297	1.521	22.566	25.419	26.460	3.116	23.785	29.882
ENSG00000139218	SCAF11	-1.1	4.3E-05	1.4E-04	25.795	0.914	24.876	26.704	29.340	0.720	28.545	29.948
ENSG00000139505	MTMR6	-1.1	2.3E-02	4.0E-02	11.956	0.384	11.572	12.339	13.416	0.769	12.577	14.086
ENSG00000139546	TARBP2	-1.1	6.9E-03	1.4E-02	21.530	1.161	20.379	22.701	24.348	1.139	23.395	25.609
ENSG00000139579	NABP2	-1.1	3.9E-03	8.0E-03	35.601	0.667	34.934	36.225	40.816	0.715	39.992	41.265
ENSG00000139613	SMARCC2	-1.1	1.4E-01	1.9E-01	19.593	0.705	18.800	20.150	20.688	0.679	20.017	21.375
ENSG00000139637	C12orf10	-1.1	3.9E-02	6.5E-02	36.389	2.850	34.683	39.679	39.882	0.334	39.505	40.142
ENSG00000139651	ZNF740	-1.1	6.0E-02	9.4E-02	14.009	0.447	13.744	14.525	15.142	0.920	14.188	16.025
ENSG00000139842	CUL4A	-1.1	2.9E-05	9.4E-05	48.217	2.621	45.644	50.883	55.481	1.678	54.107	57.352
ENSG00000139977	NAA30	-1.1	1.1E-03	2.7E-03	17.356	0.449	16.921	17.817	19.813	0.855	19.083	20.753
ENSG00000140307	GTF2A2	-1.1	2.2E-02	3.8E-02	46.021	2.446	43.417	48.270	50.641	2.077	48.263	52.100
ENSG00000140464	PML	-1.1	1.2E-02	2.2E-02	5.127	0.512	4.651	5.668	5.885	0.279	5.607	6.165
ENSG00000140553	UNC45A	-1.1	2.2E-04	5.9E-04	31.068	0.828	30.459	32.010	35.705	2.068	33.404	37.410
ENSG00000140612	SEC11A	-1.1	6.0E-03	1.2E-02	92.227	0.883	91.418	93.168	99.418	1.309	97.958	100.489
ENSG00000140632	GLYR1	-1.1	1.3E-04	3.7E-04	74.296	3.920	69.838	77.202	83.655	0.308	83.388	83.992
ENSG00000140829	DHX38	-1.1	1.3E-01	1.9E-01	26.094	0.541	25.639	26.691	27.518	1.112	26.440	28.662
ENSG00000140995	DEF8	-1.1	1.8E-02	3.2E-02	18.750	0.846	18.052	19.690	20.654	0.806	20.044	21.565
ENSG00000141002	TCF25	-1.1	5.8E-02	9.1E-02	12.996	0.163	12.883	13.182	14.203	1.520	12.494	15.404
ENSG00000141027	NCOR1	-1.1	1.5E-03	3.5E-03	29.468	1.020	28.295	30.148	32.295	0.825	31.367	32.949
ENSG00000141198	TOM1L1	-1.1	3.0E-02	5.0E-02	18.793	0.881	18.095	19.782	20.512	0.749	19.699	21.174
ENSG00000141279	NPEPPS	-1.1	2.5E-03	5.5E-03	36.595	0.360	36.271	36.983	39.921	0.758	39.053	40.448
ENSG00000141380	SS18	-1.1	1.5E-02	2.7E-02	66.792	3.065	64.237	70.191	72.617	3.444	68.869	75.642
ENSG00000141441	GAREM1	-1.1	1.4E-01	1.9E-01	3.491	0.186	3.324	3.691	3.907	0.201	3.692	4.090
ENSG00000141456	PELP1	-1.1	1.1E-03	2.6E-03	46.624	0.341	46.232	46.848	52.490	2.184	50.272	54.638
ENSG00000141503	MINK1	-1.1	5.1E-03	1.0E-02	17.759	0.308	17.475	18.086	20.262	1.938	18.513	22.346
ENSG00000141524	TMC6	-1.1	1.3E-01	1.8E-01	13.504	0.515	13.069	14.073	14.484	0.611	13.793	14.953
ENSG00000141560	FN3KRP	-1.1	2.0E-02	3.6E-02	19.843	1.445	18.183	20.816	22.346	0.482	21.847	22.809
ENSG00000141646	SMAD4	-1.1	2.2E-03	4.8E-03	16.751	0.458	16.313	17.228	18.861	1.801	16.782	19.927
ENSG00000141698	NT5C3B	-1.1	8.5E-02	1.3E-01	51.467	0.576	51.074	52.128	55.246	2.336	52.549	56.643
ENSG00000141699	RETREG3	-1.1	1.0E-01	1.5E-01	16.281	0.331	15.900	16.500	17.589	0.758	16.888	18.394
ENSG00000141956	PRDM15	-1.1	3.0E-02	5.0E-02	3.294	0.219	3.044	3.458	3.729	0.117	3.633	3.859
ENSG00000141971	MVB12A	-1.1	9.3E-02	1.4E-01	8.983	1.252	7.858	10.333	9.966	0.573	9.340	10.464
ENSG00000142039	CCDC97	-1.1	3.8E-02	6.3E-02	13.068	0.103	12.950	13.128	14.681	1.069	13.454	15.417
ENSG00000142166	IFNAR1	-1.1	5.8E-02	9.1E-02	8.653	0.103	8.549	8.756	9.543	0.478	9.193	10.087
ENSG00000142186	SCYL1	-1.1	1.0E-03	2.4E-03	30.360	0.279	30.062	30.616	34.691	1.706	33.568	36.654
ENSG00000142197	DOPEY2	-1.1	6.6E-02	1.0E-01	3.854	0.167	3.703	4.034	4.318	0.088	4.233	4.409
ENSG00000142449	FBN3	-1.1	6.6E-02	1.0E-01	11.688	0.277	11.371	11.881	12.711	0.744	12.137	13.551
ENSG00000142546	NOSIP	-1.1	1.1E-02	2.1E-02	29.911	1.315	29.117	31.429	33.189	1.078	32.132	34.288
ENSG00000142684	ZNF593	-1.1	3.5E-02	5.8E-02	53.739	1.531	52.559	55.470	60.711	1.939	59.581	62.950
ENSG00000142751	GPN2	-1.1	1.0E-01	1.5E-01	13.059	0.123	12.927	13.171	14.265	1.506	12.906	15.885
ENSG00000142784	WDTC1	-1.1	2.0E-02	3.5E-02	11.450	0.557	10.905	12.018	12.836	0.700	12.035	13.333
ENSG00000142798	HSPG2	-1.1	3.5E-03	7.4E-03	22.578	1.036	21.803	23.755	25.976	1.673	24.961	27.906
ENSG00000142892	PIGK	-1.1	2.3E-03	5.0E-03	18.204	0.542	17.666	18.750	20.887	1.518	19.145	21.917
ENSG00000143149	ALDH9A1	-1.1	7.2E-03	1.4E-02	78.521	0.848	77.548	79.099	85.725	1.055	84.577	86.651
ENSG00000143162	CREG1	-1.1	3.7E-03	7.7E-03	33.390	0.648	32.849	34.108	37.863	0.659	37.105	38.301
ENSG00000143179	UCK2	-1.1	8.7E-02	1.3E-01	39.960	0.465	39.427	40.278	42.202	1.233	40.815	43.173
ENSG00000143258	USP21	-1.1	8.8E-02	1.3E-01	12.614	0.679	11.974	13.325	13.920	0.761	13.100	14.605
ENSG00000143321	HDGF	-1.1	4.5E-02	7.3E-02	292.650	3.076	289.471	295.612	308.884	10.120	297.914	317.857
ENSG00000143322	ABL2	-1.1	1.4E-01	2.0E-01	10.097	0.313	9.835	10.444	10.669	0.176	10.547	10.870
ENSG00000143374	TARS2	-1.1	5.6E-03	1.1E-02	23.893	0.821	22.948	24.426	26.915	1.523	25.628	28.597
ENSG00000143376	SNX27	-1.1	4.6E-02	7.4E-02	25.978	0.435	25.487	26.316	27.770	0.785	27.283	28.676
ENSG00000143401	ANP32E	-1.1	6.1E-03	1.2E-02	213.838	14.189	198.577	226.631	234.174	16.268	220.525	252.175
ENSG00000143436	MRPL9	-1.1	1.2E-01	1.7E-01	82.033	1.373	80.745	83.478	86.923	3.197	83.952	90.306
ENSG00000143457	GOLPH3L	-1.1	1.4E-03	3.2E-03	49.618	0.180	49.442	49.802	56.148	2.749	53.701	59.123
ENSG00000143570	SLC39A1	-1.1	3.1E-04	8.2E-04	150.830	1.513	149.432	152.435	167.157	3.841	163.643	171.258
ENSG00000143621	ILF2	-1.1	1.2E-03	2.8E-03	522.069	5.621	516.490	527.731	568.407	10.662	558.324	579.567
ENSG00000143740	SNAP47	-1.1	7.3E-02	1.1E-01	14.125	0.593	13.462	14.604	15.232	0.627	14.508	15.603
ENSG00000143756	FBXO28	-1.1	5.1E-03	1.0E-02	27.653	1.142	26.749	28.936	30.666	1.120	29.443	31.640
ENSG00000143842	SOX13	-1.1	8.2E-04	2.0E-03	28.286	0.524	27.786	28.831	32.077	0.798	31.176	32.694
ENSG00000143889	HNRNPLL	-1.1	3.0E-03	6.3E-03	38.907	1.016	37.916	39.946	43.145	1.671	41.279	44.503
ENSG00000143970	ASXL2	-1.1	3.5E-02	5.9E-02	16.296	0.288	16.116	16.628	17.600	0.461	17.183	18.095
ENSG00000144034	TPRKB	-1.1	1.7E-02	3.0E-02	50.176	5.316	46.448	56.264	56.415	1.613	54.946	58.141
ENSG00000144231	POLR2D	-1.1	1.2E-01	1.7E-01	35.544	1.132	34.418	36.682	37.650	0.417	37.260	38.089
ENSG00000144283	PKP4	-1.1	1.1E-05	3.9E-05	36.346	0.961	35.237	36.916	41.855	0.833	40.909	42.480
ENSG00000144524	COPS7B	-1.1	1.0E-03	2.5E-03	23.414	0.743	22.745	24.213	26.587	0.316	26.256	26.885
ENSG00000144566	RAB5A	-1.1	4.2E-02	6.8E-02	78.620	4.314	73.769	82.025	84.008	1.030	82.888	84.916
ENSG00000144597	EAF1	-1.1	3.1E-02	5.3E-02	28.829	0.832	27.914	29.540	31.260	1.365	29.909	32.638
ENSG00000144635	DYNC1LI1	-1.1	7.8E-03	1.5E-02	34.534	2.396	32.911	37.286	38.055	1.464	36.470	39.355
ENSG00000144647	POMGNT2	-1.1	1.5E-02	2.8E-02	17.365	0.249	17.096	17.587	19.914	1.213	18.534	20.815
ENSG00000144677	CTDSPL	-1.1	2.0E-02	3.6E-02	25.996	0.654	25.245	26.445	28.606	2.544	26.945	31.534
ENSG00000144746	ARL6IP5	-1.1	1.9E-03	4.2E-03	48.540	1.686	46.598	49.634	55.167	2.033	53.637	57.475
ENSG00000144840	RABL3	-1.1	7.1E-03	1.4E-02	14.856	0.221	14.601	15.005	17.034	0.696	16.245	17.559
ENSG00000144909	OSBPL11	-1.1	1.3E-01	1.8E-01	26.942	0.496	26.627	27.515	28.742	1.873	26.770	30.497
ENSG00000145216	FIP1L1	-1.1	1.9E-02	3.4E-02	13.294	0.863	12.355	14.052	14.968	0.731	14.144	15.540
ENSG00000145332	KLHL8	-1.1	9.3E-02	1.4E-01	5.533	0.570	4.974	6.114	6.177	0.885	5.303	7.074
ENSG00000145388	METTL14	-1.1	1.0E-02	1.9E-02	17.477	1.043	16.275	18.130	19.497	1.008	18.541	20.550
ENSG00000145423	SFRP2	-1.1	1.7E-04	4.9E-04	457.080	2.051	455.884	459.448	517.295	37.341	479.697	554.374
ENSG00000145703	IQGAP2	-1.1	3.5E-02	5.8E-02	41.449	1.225	40.188	42.634	44.894	0.978	43.918	45.874
ENSG00000145715	RASA1	-1.1	2.3E-02	3.9E-02	37.086	1.008	35.962	37.911	40.126	0.752	39.329	40.822
ENSG00000145734	BDP1	-1.1	3.1E-02	5.3E-02	18.841	1.256	17.401	19.709	20.336	0.432	19.931	20.791
ENSG00000145860	RNF145	-1.1	8.6E-03	1.6E-02	67.901	0.724	67.083	68.460	73.683	1.882	72.387	75.842
ENSG00000145907	G3BP1	-1.1	4.2E-05	1.3E-04	153.443	4.657	149.018	158.301	171.807	7.137	163.567	175.984
ENSG00000145919	BOD1	-1.1	2.4E-02	4.2E-02	169.121	5.024	163.677	173.580	181.863	10.652	171.668	192.921
ENSG00000145996	CDKAL1	-1.1	1.2E-01	1.8E-01	14.178	1.308	12.678	15.078	15.355	0.649	14.608	15.784
ENSG00000146007	ZMAT2	-1.1	4.8E-02	7.8E-02	128.534	3.925	124.606	132.457	138.465	4.202	135.835	143.311
ENSG00000146063	TRIM41	-1.1	5.1E-02	8.1E-02	3.810	0.071	3.765	3.892	4.226	0.051	4.168	4.260
ENSG00000146215	CRIP3	-1.1	7.2E-02	1.1E-01	9.535	1.657	8.180	11.382	10.908	0.581	10.364	11.520
ENSG00000146282	RARS2	-1.1	3.6E-02	6.0E-02	50.977	2.905	48.943	54.305	55.406	0.863	54.438	56.095
ENSG00000146414	SHPRH	-1.1	8.1E-02	1.2E-01	10.086	0.228	9.915	10.345	10.701	0.190	10.530	10.906
ENSG00000146433	TMEM181	-1.1	8.5E-04	2.1E-03	24.272	0.393	23.821	24.541	27.773	0.918	27.101	28.819
ENSG00000146476	ARMT1	-1.1	3.5E-02	5.8E-02	91.587	2.187	89.941	94.068	98.315	1.946	96.405	100.294
ENSG00000146731	CCT6A	-1.1	2.0E-03	4.4E-03	360.051	7.143	352.087	365.891	391.876	11.492	379.339	401.912
ENSG00000146904	EPHA1	-1.1	9.2E-03	1.8E-02	95.876	2.287	94.116	98.462	103.462	3.565	100.346	107.350
ENSG00000146909	NOM1	-1.1	5.1E-02	8.1E-02	16.764	0.248	16.486	16.965	18.058	0.251	17.772	18.241
ENSG00000146938	NLGN4X	-1.1	1.5E-04	4.2E-04	99.619	6.716	95.076	107.333	112.884	2.265	110.446	114.924
ENSG00000147130	ZMYM3	-1.1	7.5E-03	1.5E-02	39.477	1.537	37.740	40.663	43.157	1.690	41.810	45.053
ENSG00000147140	NONO	-1.1	1.2E-05	4.2E-05	215.457	3.209	213.250	219.138	243.231	5.578	237.727	248.889
ENSG00000147164	SNX12	-1.1	1.2E-03	2.8E-03	69.857	1.337	68.368	70.956	78.579	1.160	77.403	79.722
ENSG00000147224	PRPS1	-1.1	2.8E-02	4.7E-02	209.906	3.080	207.242	213.279	223.176	5.076	217.620	227.569
ENSG00000147416	ATP6V1B2	-1.1	4.5E-02	7.3E-02	31.465	0.196	31.344	31.691	33.645	2.019	32.433	35.976
ENSG00000147439	BIN3	-1.1	9.4E-02	1.4E-01	8.488	0.320	8.204	8.835	9.359	0.696	8.829	10.147
ENSG00000147459	DOCK5	-1.1	9.0E-02	1.3E-01	4.032	0.310	3.675	4.232	4.415	0.226	4.158	4.580
ENSG00000147533	GOLGA7	-1.1	2.5E-03	5.5E-03	57.522	2.561	54.850	59.955	64.708	2.055	62.945	66.965
ENSG00000147548	NSD3	-1.1	1.5E-02	2.7E-02	19.652	0.536	19.051	20.078	21.244	0.187	21.028	21.358
ENSG00000147669	POLR2K	-1.1	1.3E-02	2.4E-02	88.664	3.656	85.096	92.402	97.308	4.152	93.485	101.725
ENSG00000147872	PLIN2	-1.1	2.3E-02	3.9E-02	14.746	1.003	14.106	15.902	16.720	1.276	15.738	18.161
ENSG00000147874	HAUS6	-1.1	7.3E-05	2.2E-04	46.839	2.826	43.598	48.794	53.674	1.582	52.746	55.500
ENSG00000148143	ZNF462	-1.1	6.0E-03	1.2E-02	16.120	1.009	14.958	16.772	17.890	0.485	17.354	18.298
ENSG00000148300	REXO4	-1.1	6.5E-03	1.3E-02	42.517	1.121	41.611	43.770	48.013	3.141	45.425	51.508
ENSG00000148331	ASB6	-1.1	2.7E-02	4.6E-02	10.626	0.554	10.079	11.187	12.071	0.257	11.787	12.288
ENSG00000148334	PTGES2	-1.1	5.7E-03	1.1E-02	45.039	1.979	42.765	46.362	50.788	2.681	48.080	53.441
ENSG00000148396	SEC16A	-1.1	8.1E-03	1.6E-02	24.582	1.663	22.777	26.052	27.015	0.671	26.371	27.710
ENSG00000148429	USP6NL	-1.1	4.5E-03	9.2E-03	8.425	0.499	7.945	8.940	9.521	0.276	9.207	9.726
ENSG00000148606	POLR3A	-1.1	1.0E-03	2.4E-03	23.499	0.668	23.074	24.269	26.390	0.513	25.798	26.709
ENSG00000148660	CAMK2G	-1.1	1.7E-02	3.0E-02	14.935	0.696	14.303	15.681	16.477	0.674	15.699	16.894
ENSG00000148688	RPP30	-1.1	1.0E-03	2.4E-03	31.738	0.635	31.016	32.208	36.174	1.910	34.347	38.157
ENSG00000148773	MKI67	-1.1	3.2E-02	5.4E-02	82.103	4.042	77.470	84.910	87.156	2.157	85.240	89.492
ENSG00000148835	TAF5	-1.1	7.4E-02	1.1E-01	26.023	1.466	25.133	27.715	28.300	1.631	26.587	29.834
ENSG00000149182	ARFGAP2	-1.1	3.9E-03	8.0E-03	25.703	0.550	25.083	26.133	28.625	1.416	27.050	29.793
ENSG00000149308	NPAT	-1.1	4.2E-02	6.8E-02	14.008	0.946	12.927	14.683	15.306	0.512	14.932	15.890
ENSG00000149418	ST14	-1.1	1.4E-01	2.0E-01	17.348	1.745	15.657	19.142	18.846	2.244	16.763	21.222
ENSG00000149474	KAT14	-1.1	1.7E-02	3.0E-02	18.184	1.707	16.877	20.115	20.630	1.782	18.573	21.696
ENSG00000149532	CPSF7	-1.1	3.2E-02	5.4E-02	38.924	1.274	37.998	40.377	41.821	1.081	40.823	42.969
ENSG00000149582	TMEM25	-1.1	7.3E-02	1.1E-01	3.881	0.245	3.600	4.051	4.444	0.371	4.017	4.686
ENSG00000149923	PPP4C	-1.1	3.7E-04	9.7E-04	79.576	2.451	76.892	81.694	91.187	6.451	86.143	98.456
ENSG00000149930	TAOK2	-1.1	2.7E-03	5.9E-03	11.752	0.615	11.042	12.118	13.528	0.594	12.862	14.001
ENSG00000150316	CWC15	-1.1	2.8E-03	6.1E-03	49.049	2.082	47.789	51.451	55.280	2.145	54.041	57.756
ENSG00000150459	SAP18	-1.1	5.4E-02	8.5E-02	92.528	1.197	91.274	93.659	98.482	3.315	94.730	101.015
ENSG00000150764	DIXDC1	-1.1	4.8E-02	7.7E-02	10.972	0.306	10.648	11.255	11.941	0.642	11.202	12.362
ENSG00000150776	C11orf57	-1.1	7.0E-02	1.1E-01	17.336	0.446	16.838	17.701	18.710	0.600	18.204	19.373
ENSG00000150907	FOXO1	-1.1	1.1E-01	1.6E-01	28.661	0.849	27.887	29.614	30.346	0.608	29.736	30.952
ENSG00000151148	UBE3B	-1.1	1.0E-01	1.5E-01	5.267	0.530	4.685	5.720	5.746	0.468	5.272	6.209
ENSG00000151208	DLG5	-1.1	2.8E-04	7.6E-04	33.165	1.002	32.380	34.294	37.573	1.828	36.315	39.670
ENSG00000151304	SRFBP1	-1.1	1.1E-02	2.0E-02	24.364	1.925	22.227	25.960	28.040	2.743	26.387	31.206
ENSG00000151458	ANKRD50	-1.1	4.2E-02	6.8E-02	19.884	0.819	19.201	20.793	21.388	1.025	20.794	22.572
ENSG00000151465	CDC123	-1.1	3.3E-02	5.5E-02	207.360	3.525	203.304	209.694	220.179	4.021	215.567	222.955
ENSG00000151532	VTI1A	-1.1	3.8E-02	6.3E-02	6.065	0.675	5.342	6.680	6.801	0.188	6.601	6.973
ENSG00000151553	FAM160B1	-1.1	4.3E-02	7.0E-02	12.337	0.293	12.055	12.639	13.525	0.219	13.272	13.663
ENSG00000151692	RNF144A	-1.1	1.9E-02	3.4E-02	13.760	0.280	13.449	13.993	15.271	0.400	14.810	15.530
ENSG00000151729	SLC25A4	-1.1	7.3E-02	1.1E-01	24.563	0.723	24.099	25.396	26.446	0.775	25.814	27.312
ENSG00000151806	GUF1	-1.1	1.2E-02	2.2E-02	44.721	0.920	43.959	45.743	48.831	0.964	47.852	49.779
ENSG00000151893	CACUL1	-1.1	1.7E-02	3.1E-02	18.598	0.236	18.338	18.799	20.090	0.409	19.686	20.504
ENSG00000152127	MGAT5	-1.1	1.2E-01	1.7E-01	23.710	0.393	23.266	24.013	25.138	0.561	24.640	25.746
ENSG00000152147	GEMIN6	-1.1	3.8E-02	6.2E-02	14.585	0.448	14.256	15.096	16.274	1.035	15.082	16.945
ENSG00000152270	PDE3B	-1.1	5.8E-02	9.1E-02	6.718	0.504	6.249	7.251	7.378	0.168	7.207	7.542
ENSG00000152455	SUV39H2	-1.1	5.0E-02	7.9E-02	39.118	1.973	37.451	41.297	42.617	2.450	39.819	44.375
ENSG00000152484	USP12	-1.1	6.4E-04	1.6E-03	30.335	1.900	28.209	31.868	34.655	0.306	34.386	34.988
ENSG00000152578	GRIA4	-1.1	3.9E-02	6.5E-02	7.551	0.374	7.309	7.982	8.359	0.310	8.021	8.631
ENSG00000152620	NADK2	-1.1	9.3E-02	1.4E-01	13.458	0.672	13.059	14.233	14.636	0.964	13.818	15.699
ENSG00000152683	SLC30A6	-1.1	3.9E-03	8.1E-03	27.049	0.484	26.523	27.475	30.207	0.713	29.444	30.855
ENSG00000152795	HNRNPDL	-1.1	2.5E-04	6.7E-04	211.787	12.733	197.305	221.226	236.106	3.505	232.222	239.034
ENSG00000152894	PTPRK	-1.1	1.8E-03	3.9E-03	25.872	0.912	25.087	26.873	28.782	0.718	27.953	29.227
ENSG00000152926	ZNF117	-1.1	1.4E-01	1.9E-01	10.114	1.116	8.828	10.839	10.922	0.841	10.025	11.694
ENSG00000152942	RAD17	-1.1	1.0E-02	1.9E-02	33.167	1.322	32.065	34.632	36.915	1.430	35.359	38.172
ENSG00000153147	SMARCA5	-1.1	1.7E-02	3.0E-02	157.902	2.727	155.904	161.010	167.862	1.873	165.895	169.625
ENSG00000153179	RASSF3	-1.1	1.7E-03	3.9E-03	44.140	2.551	41.673	46.768	49.474	1.382	48.401	51.034
ENSG00000153187	HNRNPU	-1.1	6.5E-05	2.0E-04	137.902	4.233	133.365	141.745	152.627	2.292	150.536	155.077
ENSG00000153208	MERTK	-1.1	1.1E-01	1.6E-01	5.113	0.313	4.835	5.451	5.726	0.471	5.251	6.192
ENSG00000153707	PTPRD	-1.1	3.0E-02	5.0E-02	18.139	0.479	17.778	18.682	19.565	0.568	18.967	20.097
ENSG00000153936	HS2ST1	-1.1	4.4E-02	7.1E-02	19.992	0.556	19.426	20.537	21.640	1.395	20.521	23.203
ENSG00000154059	IMPACT	-1.1	4.7E-02	7.6E-02	37.010	0.833	36.075	37.672	39.923	1.488	38.856	41.623
ENSG00000154118	JPH3	-1.1	3.4E-03	7.2E-03	14.056	0.338	13.666	14.262	16.182	1.314	14.719	17.260
ENSG00000154124	OTULIN	-1.1	1.9E-02	3.3E-02	12.744	0.582	12.131	13.288	14.082	0.626	13.687	14.804
ENSG00000154222	CC2D1B	-1.1	1.9E-02	3.3E-02	5.523	0.385	5.217	5.956	6.299	0.035	6.262	6.331
ENSG00000154229	PRKCA	-1.1	1.1E-01	1.6E-01	5.037	0.376	4.733	5.457	5.526	0.253	5.320	5.808
ENSG00000154305	MIA3	-1.1	2.4E-03	5.3E-03	22.432	1.272	20.972	23.300	24.869	0.486	24.414	25.380
ENSG00000154447	SH3RF1	-1.1	8.9E-02	1.3E-01	9.272	0.333	8.889	9.496	10.148	0.088	10.087	10.249
ENSG00000154767	XPC	-1.1	1.4E-02	2.6E-02	28.880	0.854	28.094	29.789	31.892	2.804	29.156	34.758
ENSG00000154978	VOPP1	-1.1	3.1E-02	5.2E-02	31.846	1.854	30.674	33.983	34.553	0.939	33.479	35.222
ENSG00000155115	GTF3C6	-1.1	3.0E-02	5.0E-02	122.165	5.527	116.909	127.928	134.352	9.320	127.328	144.925
ENSG00000155275	TRMT44	-1.1	1.1E-01	1.6E-01	4.044	0.663	3.278	4.436	4.563	0.299	4.237	4.824
ENSG00000155329	ZCCHC10	-1.1	8.9E-03	1.7E-02	32.472	2.803	29.253	34.379	37.190	2.869	34.530	40.231
ENSG00000155393	HEATR3	-1.1	4.2E-02	6.8E-02	25.116	0.874	24.447	26.106	27.351	0.317	26.989	27.579
ENSG00000155545	MIER3	-1.1	4.3E-03	8.8E-03	23.922	1.325	22.980	25.437	26.789	1.178	25.602	27.958
ENSG00000155636	RBM45	-1.1	3.6E-02	5.9E-02	7.512	0.203	7.363	7.743	8.598	0.448	8.082	8.877
ENSG00000155827	RNF20	-1.1	1.6E-04	4.6E-04	46.358	0.959	45.255	46.994	52.928	1.610	51.071	54.786
ENSG00000155974	GRIP1	-1.1	2.1E-02	3.6E-02	5.866	0.541	5.497	6.487	6.766	0.270	6.585	7.076
ENSG00000155980	KIF5A	-1.1	9.0E-02	1.3E-01	10.054	1.044	8.850	10.707	11.089	0.507	10.531	11.520
ENSG00000156011	PSD3	-1.1	4.0E-03	8.3E-03	7.346	0.885	6.361	8.077	8.450	0.378	8.022	8.741
ENSG00000156136	DCK	-1.1	7.2E-03	1.4E-02	27.704	0.316	27.494	28.068	31.367	2.649	28.354	33.331
ENSG00000156304	SCAF4	-1.1	4.5E-02	7.3E-02	25.113	1.197	23.758	26.026	27.037	0.688	26.276	27.613
ENSG00000156374	PCGF6	-1.1	3.6E-02	6.0E-02	20.490	0.355	20.231	20.895	23.069	0.973	22.399	24.185
ENSG00000156502	SUPV3L1	-1.1	9.6E-03	1.8E-02	28.573	1.718	27.135	30.476	31.925	1.419	30.429	33.252
ENSG00000156521	TYSND1	-1.1	2.0E-02	3.5E-02	9.832	0.353	9.619	10.240	11.200	0.694	10.460	11.836
ENSG00000156599	ZDHHC5	-1.1	5.0E-04	1.3E-03	37.235	0.744	36.391	37.793	42.232	1.946	40.658	44.408
ENSG00000156711	MAPK13	-1.1	5.7E-02	8.9E-02	8.387	0.216	8.140	8.540	9.208	0.478	8.657	9.492
ENSG00000156735	BAG4	-1.1	8.3E-02	1.2E-01	30.248	1.222	28.966	31.400	32.348	1.707	30.411	33.635
ENSG00000156860	FBRS	-1.1	9.6E-02	1.4E-01	3.051	0.189	2.837	3.193	3.443	0.097	3.367	3.552
ENSG00000156931	VPS8	-1.1	2.6E-03	4.5E-03	13.430	0.433	13.012	13.877	14.647	0.679	14.102	15.407
ENSG00000157020	SEC13	-1.1	6.2E-02	9.7E-02	43.678	0.527	43.105	44.142	46.425	1.018	45.272	47.198
ENSG00000157077	ZFYVE9	-1.1	1.2E-03	2.8E-03	29.331	0.810	28.736	30.254	33.448	2.110	31.819	35.832
ENSG00000157106	SMG1	-1.1	1.8E-02	3.2E-02	26.497	1.555	24.950	28.060	28.414	0.914	27.470	29.294
ENSG00000157184	CPT2	-1.1	1.1E-01	1.6E-01	8.208	0.439	7.709	8.530	8.802	0.223	8.555	8.989
ENSG00000157741	UBN2	-1.1	3.0E-02	5.0E-02	10.616	0.367	10.220	10.946	11.477	0.395	11.025	11.756
ENSG00000157837	SPPL3	-1.1	7.3E-03	1.4E-02	15.601	0.242	15.384	15.862	17.406	0.773	16.645	18.190
ENSG00000158156	XKR8	-1.1	4.9E-02	7.9E-02	15.520	1.587	13.906	17.078	17.487	0.984	16.754	18.606
ENSG00000158352	SHROOM4	-1.1	4.1E-02	6.7E-02	3.499	0.144	3.346	3.633	3.981	0.197	3.758	4.131
ENSG00000158483	FAM86C1	-1.1	1.3E-01	1.9E-01	6.767	1.317	5.534	8.154	7.649	0.368	7.356	8.062
ENSG00000158528	PPP1R9A	-1.1	4.6E-02	7.4E-02	8.620	0.057	8.554	8.658	9.377	0.369	9.095	9.795
ENSG00000158545	ZC3H18	-1.1	5.6E-03	1.1E-02	20.350	1.258	19.579	21.802	22.691	1.169	21.801	24.015
ENSG00000158769	F11R	-1.1	3.2E-04	8.5E-04	98.180	0.783	97.527	99.048	108.688	1.003	107.841	109.795
ENSG00000158796	DEDD	-1.1	8.8E-03	1.7E-02	31.680	1.575	30.496	33.468	35.706	0.385	35.384	36.132
ENSG00000159055	MIS18A	-1.1	3.3E-02	5.6E-02	101.090	7.597	94.961	109.590	109.600	2.731	107.546	112.699
ENSG00000159140	SON	-1.1	5.1E-02	8.2E-02	101.101	1.465	99.634	102.565	106.565	1.020	105.434	107.417
ENSG00000159202	UBE2Z	-1.1	6.8E-04	1.7E-03	41.741	0.399	41.306	42.090	46.807	0.725	46.007	47.421
ENSG00000159314	ARHGAP27	-1.1	1.3E-01	1.8E-01	3.444	0.231	3.242	3.696	3.789	0.253	3.573	4.068
ENSG00000159322	ADPGK	-1.1	9.2E-03	1.7E-02	18.992	0.554	18.583	19.622	21.272	0.214	21.026	21.409
ENSG00000159335	PTMS	-1.1	1.3E-01	1.9E-01	205.428	12.566	195.055	219.402	224.518	23.208	197.742	238.864
ENSG00000159352	PSMD4	-1.1	2.7E-04	7.4E-04	122.974	4.132	118.938	127.196	138.500	4.534	133.435	142.180
ENSG00000159377	PSMB4	-1.1	5.8E-02	9.1E-02	291.357	4.585	286.548	295.680	307.349	11.161	299.443	320.116
ENSG00000159593	NAE1	-1.1	2.0E-02	3.5E-02	121.672	2.843	118.719	124.391	129.795	2.302	128.027	132.399
ENSG00000160194	NDUFV3	-1.1	8.1E-03	1.6E-02	17.229	0.397	16.782	17.539	19.345	1.099	18.081	20.073
ENSG00000160200	CBS	-1.1	5.9E-03	1.2E-02	73.575	0.940	72.750	74.598	79.853	2.831	77.105	82.760
ENSG00000160201	U2AF1	-1.1	1.7E-02	3.1E-02	26.604	0.752	26.165	27.473	28.640	0.544	28.111	29.198
ENSG00000160218	TRAPPC10	-1.1	1.7E-02	3.0E-02	12.063	0.126	11.935	12.187	13.293	0.276	13.123	13.611
ENSG00000160336	ZNF761	-1.1	2.5E-02	4.3E-02	22.619	1.524	21.437	24.339	24.792	0.662	24.240	25.526
ENSG00000160352	ZNF714	-1.1	5.1E-02	8.2E-02	25.013	1.286	24.064	26.477	26.834	1.372	25.251	27.663
ENSG00000160408	ST6GALNAC6	-1.1	6.2E-02	9.7E-02	14.168	0.613	13.580	14.804	15.632	0.984	14.529	16.419
ENSG00000160570	DEDD2	-1.1	1.3E-01	1.9E-01	28.025	0.605	27.338	28.481	30.014	0.400	29.764	30.475
ENSG00000160691	SHC1	-1.1	2.0E-04	5.5E-04	156.861	0.911	155.877	157.676	175.536	4.723	172.760	180.989
ENSG00000160714	UBE2Q1	-1.1	1.8E-02	3.2E-02	40.201	0.347	39.946	40.596	43.658	0.447	43.147	43.978
ENSG00000160753	RUSC1	-1.1	1.4E-02	2.6E-02	16.873	1.349	15.982	18.424	18.858	0.565	18.275	19.404
ENSG00000160957	RECQL4	-1.1	3.4E-02	5.7E-02	27.787	1.252	26.344	28.586	30.616	1.671	28.821	32.127
ENSG00000161011	SQSTM1	-1.1	1.9E-02	3.3E-02	27.823	0.537	27.315	28.385	30.377	2.147	29.018	32.852
ENSG00000161395	PGAP3	-1.1	5.2E-02	8.3E-02	8.183	0.421	7.786	8.624	9.267	0.560	8.795	9.886
ENSG00000161526	SAP30BP	-1.1	2.6E-03	5.7E-03	17.178	0.601	16.681	17.846	19.256	0.509	18.669	19.577
ENSG00000161542	PRPSAP1	-1.1	9.7E-02	1.4E-01	13.763	0.566	13.124	14.200	14.807	0.122	14.675	14.916
ENSG00000161888	SPC24	-1.1	2.3E-02	4.0E-02	22.391	1.979	20.823	24.614	25.050	0.220	24.832	25.272
ENSG00000161940	BCL6B	-1.1	1.2E-01	1.7E-01	4.532	0.608	4.162	5.234	5.134	0.146	5.048	5.303
ENSG00000161996	WDR90	-1.1	5.1E-02	8.1E-02	4.195	0.414	3.780	4.607	4.693	0.223	4.442	4.871
ENSG00000162065	TBC1D24	-1.1	7.2E-02	1.1E-01	2.189	0.121	2.098	2.326	2.477	0.193	2.325	2.694
ENSG00000162073	PAQR4	-1.1	1.5E-02	2.7E-02	22.141	1.133	20.859	23.007	25.157	1.114	24.206	26.382
ENSG00000162104	ADCY9	-1.1	9.8E-02	1.4E-01	4.865	0.186	4.664	5.030	5.355	0.594	4.788	5.972
ENSG00000162385	MAGOH	-1.1	1.2E-02	2.2E-02	98.209	4.493	94.846	103.311	108.139	4.433	103.926	112.763
ENSG00000162413	KLHL21	-1.1	1.1E-02	2.0E-02	11.051	0.453	10.755	11.572	12.587	0.220	12.388	12.824
ENSG00000162517	PEF1	-1.1	8.2E-02	1.2E-01	48.872	0.707	48.251	49.641	52.422	1.848	50.619	54.312
ENSG00000162591	MEGF6	-1.1	7.3E-02	1.1E-01	5.524	0.344	5.232	5.903	6.187	0.103	6.092	6.296
ENSG00000162613	FUBP1	-1.1	2.6E-04	7.0E-04	149.551	9.119	139.257	156.616	167.206	4.848	161.632	170.443
ENSG00000162636	FAM102B	-1.1	7.7E-02	1.2E-01	12.237	0.573	11.599	12.706	13.205	0.495	12.759	13.737
ENSG00000162714	ZNF496	-1.1	5.8E-04	1.5E-03	22.440	0.582	21.994	23.099	25.494	1.581	23.836	26.985
ENSG00000162736	NCSTN	-1.1	1.0E-01	1.5E-01	39.988	0.475	39.472	40.408	42.639	2.374	40.006	44.616
ENSG00000162738	VANGL2	-1.1	4.0E-02	6.5E-02	64.457	1.861	62.891	66.514	68.910	1.532	67.150	69.943
ENSG00000162813	BPNT1	-1.1	2.2E-02	3.8E-02	14.606	0.553	14.068	15.173	16.583	0.966	15.975	17.697
ENSG00000162929	KIAA1841	-1.1	6.8E-02	1.0E-01	5.434	0.275	5.234	5.748	6.067	0.544	5.631	6.677
ENSG00000163125	RPRD2	-1.1	1.4E-02	2.5E-02	14.970	0.733	14.255	15.719	16.620	1.032	15.430	17.275
ENSG00000163161	ERCC3	-1.1	3.5E-03	7.3E-03	22.260	0.161	22.129	22.439	24.714	0.820	23.898	25.537
ENSG00000163166	IWS1	-1.1	2.4E-02	4.2E-02	80.423	0.877	79.419	81.042	86.242	1.337	85.092	87.709
ENSG00000163249	CCNYL1	-1.1	5.0E-04	1.3E-03	33.305	0.935	32.226	33.857	38.208	1.443	36.546	39.144
ENSG00000163378	EOGT	-1.1	1.0E-02	1.9E-02	17.584	0.716	16.925	18.345	19.661	0.941	19.083	20.746
ENSG00000163479	SSR2	-1.1	6.1E-02	9.4E-02	52.523	2.105	51.031	54.931	55.812	2.161	54.124	58.248
ENSG00000163527	STT3B	-1.1	3.7E-03	7.8E-03	141.955	3.871	137.512	144.598	154.723	4.857	149.340	158.780
ENSG00000163584	RPL22L1	-1.1	7.1E-04	1.8E-03	180.796	4.006	176.778	184.790	202.879	9.688	191.940	210.377
ENSG00000163590	PPM1L	-1.1	3.1E-03	6.6E-03	6.950	0.236	6.783	7.220	7.936	0.321	7.715	8.305
ENSG00000163624	CDS1	-1.1	3.4E-03	7.2E-03	19.765	0.813	19.283	20.704	22.682	0.628	22.013	23.259
ENSG00000163629	PTPN13	-1.1	1.2E-03	2.8E-03	56.787	2.133	54.825	59.058	62.498	1.937	60.412	64.241
ENSG00000163636	PSMD6	-1.1	2.0E-03	4.4E-03	34.704	1.472	33.110	36.012	38.220	0.925	37.541	39.274
ENSG00000163694	RBM47	-1.1	5.0E-03	1.0E-02	32.747	1.528	31.415	34.415	36.295	1.546	35.313	38.077
ENSG00000163719	MTMR14	-1.1	8.5E-02	1.3E-01	18.815	0.803	17.951	19.536	20.114	0.454	19.597	20.446
ENSG00000163755	HPS3	-1.1	5.1E-03	1.0E-02	45.512	1.067	44.459	46.593	49.727	0.659	49.302	50.486
ENSG00000163866	SMIM12	-1.1	3.0E-02	5.0E-02	8.610	0.677	8.165	9.389	9.731	0.673	9.034	10.376
ENSG00000163902	RPN1	-1.1	8.0E-05	2.4E-04	145.627	4.787	140.159	149.061	164.106	1.936	161.947	165.687
ENSG00000163918	RFC4	-1.1	1.5E-04	4.3E-04	67.214	2.378	64.505	68.960	77.242	2.467	74.619	79.516
ENSG00000163923	RPL39L	-1.1	1.5E-02	2.7E-02	116.203	4.720	111.354	120.782	128.040	1.640	126.438	129.715
ENSG00000163933	RFT1	-1.1	7.6E-02	1.1E-01	15.031	1.013	13.960	15.975	16.452	1.263	15.166	17.690
ENSG00000163939	PBRM1	-1.1	5.5E-04	1.4E-03	34.459	1.711	32.837	36.246	38.783	0.950	38.024	39.849
ENSG00000164039	BDH2	-1.1	2.3E-02	4.1E-02	27.053	1.450	25.915	28.685	30.235	3.064	26.704	32.207
ENSG00000164050	PLXNB1	-1.1	3.4E-03	7.1E-03	24.082	0.916	23.031	24.716	27.023	1.526	25.274	28.077
ENSG00000164062	APEH	-1.1	3.5E-04	9.2E-04	102.059	3.884	98.334	106.084	114.112	4.436	109.620	118.490
ENSG00000164066	INTU	-1.1	1.2E-01	1.7E-01	6.878	0.144	6.735	7.022	7.350	0.251	7.095	7.596
ENSG00000164151	ICE1	-1.1	6.0E-04	1.5E-03	56.608	2.378	54.141	58.887	63.268	1.271	62.006	64.548
ENSG00000164163	ABCE1	-1.1	1.3E-03	3.0E-03	111.741	2.570	109.017	114.124	122.898	3.030	119.635	125.623
ENSG00000164168	TMEM184C	-1.1	1.1E-02	2.1E-02	26.277	2.744	23.388	28.848	29.408	1.355	28.303	30.920
ENSG00000164199	ADGRV1	-1.1	1.4E-02	2.6E-02	7.054	0.460	6.558	7.466	7.670	0.097	7.574	7.767
ENSG00000164209	SLC25A46	-1.1	4.1E-03	8.5E-03	9.703	0.620	9.094	10.334	11.144	0.171	10.981	11.321
ENSG00000164244	PRRC1	-1.1	4.5E-02	7.2E-02	33.177	1.201	32.287	34.543	35.557	2.060	34.202	37.927
ENSG00000164329	PAPD4	-1.1	7.9E-03	1.5E-02	17.607	1.270	16.141	18.380	19.989	1.721	18.221	21.658
ENSG00000164332	UBLCP1	-1.1	4.6E-04	1.2E-03	72.370	4.003	67.830	75.397	83.453	3.720	79.596	87.018
ENSG00000164347	GFM2	-1.1	6.1E-03	1.2E-02	37.273	0.908	36.224	37.808	41.246	1.645	40.272	43.146
ENSG00000164494	PDSS2	-1.1	4.1E-02	6.7E-02	15.428	1.005	14.388	16.394	17.221	1.567	16.047	19.000
ENSG00000164609	SLU7	-1.1	9.8E-02	1.4E-01	22.735	1.005	21.627	23.588	24.307	0.982	23.460	25.383
ENSG00000164638	SLC29A4	-1.1	1.2E-01	1.7E-01	2.702	0.339	2.325	2.980	3.094	0.435	2.748	3.583
ENSG00000164902	PHAX	-1.1	5.1E-03	1.0E-02	37.183	2.500	34.379	39.180	41.428	0.499	40.853	41.754
ENSG00000164944	KIAA1429	-1.1	3.1E-05	1.0E-04	24.832	0.323	24.533	25.174	28.608	0.512	28.017	28.912
ENSG00000164967	RPP25L	-1.1	6.9E-02	1.1E-01	22.172	1.885	21.060	24.349	25.287	1.627	23.947	27.098
ENSG00000165055	METTL2B	-1.1	1.5E-02	2.7E-02	12.889	0.406	12.423	13.169	14.374	0.638	13.962	15.109
ENSG00000165264	NDUFB6	-1.1	1.4E-03	3.2E-03	98.321	3.293	95.577	101.973	112.269	2.123	109.836	113.750
ENSG00000165280	VCP	-1.1	5.3E-07	2.4E-06	298.826	4.758	293.447	302.482	343.537	7.560	335.760	350.861
ENSG00000165283	STOML2	-1.1	8.7E-02	1.3E-01	192.333	12.400	178.718	202.978	203.688	7.338	195.301	208.923
ENSG00000165304	MELK	-1.1	8.2E-02	1.2E-01	71.595	1.585	70.449	73.404	76.475	1.312	74.964	77.333
ENSG00000165338	HECTD2	-1.1	4.1E-02	6.6E-02	4.520	0.572	3.982	5.121	5.139	0.389	4.906	5.588
ENSG00000165525	NEMF	-1.1	4.6E-04	1.2E-03	17.263	0.841	16.326	17.951	19.747	0.825	18.853	20.480
ENSG00000165527	ARF6	-1.1	2.1E-04	5.7E-04	68.661	1.621	66.791	69.673	77.495	0.737	76.644	77.936
ENSG00000165801	ARHGEF40	-1.1	3.3E-04	8.6E-04	20.290	0.096	20.211	20.396	23.221	0.441	22.714	23.512
ENSG00000165802	NSMF	-1.1	7.7E-03	1.5E-02	34.959	0.268	34.701	35.236	38.885	2.771	35.839	41.255
ENSG00000165934	CPSF2	-1.1	1.7E-05	5.8E-05	36.806	1.630	35.810	38.687	42.227	0.773	41.606	43.093
ENSG00000165983	PTER	-1.1	2.1E-02	3.6E-02	12.690	0.669	11.953	13.260	14.335	0.437	13.838	14.656
ENSG00000166135	HIF1AN	-1.1	7.2E-04	1.8E-03	19.721	0.615	19.031	20.212	22.022	0.221	21.829	22.263
ENSG00000166165	CKB	-1.1	8.7E-02	1.3E-01	161.850	4.888	157.587	167.185	172.233	17.065	155.913	189.957
ENSG00000166197	NOLC1	-1.1	1.4E-02	2.6E-02	241.596	1.353	240.285	242.988	257.288	4.402	253.137	261.905
ENSG00000166228	PCBD1	-1.1	2.1E-02	3.6E-02	33.378	3.023	31.177	36.824	37.904	2.623	34.879	39.547
ENSG00000166233	ARIH1	-1.1	2.4E-02	4.1E-02	15.908	0.173	15.721	16.062	17.004	0.517	16.497	17.531
ENSG00000166265	CYYR1	-1.1	6.5E-04	1.6E-03	41.757	1.440	40.096	42.642	47.770	0.726	46.965	48.373
ENSG00000166582	CENPV	-1.1	7.1E-03	1.4E-02	94.965	0.970	93.853	95.644	104.478	5.518	98.267	108.813
ENSG00000166716	ZNF592	-1.1	4.6E-03	9.4E-03	15.964	0.362	15.545	16.177	17.964	1.152	17.191	19.288
ENSG00000166734	CASC4	-1.1	1.8E-02	3.2E-02	25.124	0.877	24.125	25.768	27.569	0.285	27.241	27.755
ENSG00000166788	SAAL1	-1.1	1.4E-02	2.5E-02	48.680	2.958	45.312	50.854	53.975	1.688	52.635	55.871
ENSG00000166794	PPIB	-1.1	1.1E-04	3.2E-04	418.440	2.516	416.293	421.208	465.597	12.736	452.178	477.518
ENSG00000166974	MAPRE2	-1.1	2.6E-04	7.0E-04	27.195	0.624	26.475	27.586	31.222	0.969	30.234	32.171
ENSG00000167004	PDIA3	-1.1	6.8E-05	2.1E-04	341.736	10.557	330.139	350.789	381.066	7.546	372.916	387.812
ENSG00000167005	NUDT21	-1.1	1.5E-04	4.1E-04	185.310	9.441	176.799	195.465	207.760	8.878	200.833	217.767
ENSG00000167088	SNRPD1	-1.1	5.0E-05	1.6E-04	87.165	2.862	84.863	90.369	99.124	3.457	95.640	102.554
ENSG00000167193	CRK	-1.1	4.7E-02	7.6E-02	62.311	1.426	61.249	63.932	66.752	1.066	65.553	67.590
ENSG00000167258	CDK12	-1.1	1.0E-03	2.4E-03	21.656	1.142	20.337	22.353	24.171	0.392	23.743	24.512
ENSG00000167280	ENGASE	-1.1	3.5E-02	5.8E-02	6.587	1.032	5.804	7.757	7.502	0.423	7.015	7.776
ENSG00000167325	RRM1	-1.1	8.1E-04	2.0E-03	115.576	3.769	111.551	119.023	127.577	2.239	125.001	129.054
ENSG00000167470	MIDN	-1.1	9.8E-04	2.3E-03	53.926	1.428	52.541	55.393	61.734	3.590	58.089	65.267
ENSG00000167513	CDT1	-1.1	4.1E-03	8.4E-03	53.628	1.552	52.127	55.226	60.172	3.891	55.691	62.704
ENSG00000167614	TTYH1	-1.1	6.0E-02	9.3E-02	4.710	0.257	4.466	4.979	5.392	0.343	5.178	5.788
ENSG00000167625	ZNF526	-1.1	1.2E-01	1.7E-01	8.924	0.726	8.167	9.615	9.775	0.170	9.583	9.908
ENSG00000167674		-1.1	4.8E-04	1.2E-03	70.224	3.192	67.139	73.514	79.468	5.802	74.474	85.833
ENSG00000167699	GLOD4	-1.1	1.5E-02	2.8E-02	34.397	1.300	32.968	35.509	37.570	0.341	37.197	37.864
ENSG00000167700	MFSD3	-1.1	7.7E-02	1.2E-01	55.209	4.047	50.541	57.743	60.415	4.470	56.166	65.077
ENSG00000167770	OTUB1	-1.1	5.2E-02	8.3E-02	45.748	1.217	44.980	47.150	48.663	1.264	47.278	49.756
ENSG00000167775	CD320	-1.1	1.1E-01	1.6E-01	44.389	3.519	40.339	46.705	47.704	2.514	45.765	50.544
ENSG00000167792	NDUFV1	-1.1	1.3E-01	1.9E-01	46.774	2.377	44.087	48.601	49.293	1.355	48.020	50.717
ENSG00000167797	CDK2AP2	-1.1	2.0E-02	3.5E-02	23.821	1.578	22.028	24.999	27.335	2.810	24.146	29.447
ENSG00000167840	ZNF232	-1.1	8.5E-03	1.6E-02	22.382	0.911	21.425	23.238	25.706	1.035	24.549	26.548
ENSG00000167842	MIS12	-1.1	5.4E-03	1.1E-02	30.690	0.599	30.027	31.192	34.534	1.374	33.561	36.106
ENSG00000167965	MLST8	-1.1	7.7E-02	1.2E-01	24.826	1.854	22.695	26.064	26.906	2.197	25.139	29.367
ENSG00000167967	E4F1	-1.1	4.5E-02	7.3E-02	7.635	0.750	7.107	8.494	8.566	0.129	8.466	8.712
ENSG00000168002	POLR2G	-1.1	1.1E-04	3.2E-04	113.711	1.912	111.945	115.742	130.339	0.228	130.122	130.576
ENSG00000168036	CTNNB1	-1.1	5.9E-06	2.2E-05	185.465	4.192	180.628	188.047	212.233	8.584	202.956	219.923
ENSG00000168078	PBK	-1.1	1.1E-01	1.6E-01	16.744	0.892	15.828	17.609	18.595	0.948	17.533	19.356
ENSG00000168092	PAFAH1B2	-1.1	1.2E-02	2.3E-02	50.059	1.302	48.737	51.341	54.201	0.481	53.823	54.743
ENSG00000168246	UBTD2	-1.1	8.3E-03	1.6E-02	50.404	3.113	46.945	52.981	55.715	2.362	53.785	58.350
ENSG00000168256	NKIRAS2	-1.1	3.2E-02	5.3E-02	17.809	0.440	17.526	18.316	19.678	1.400	18.622	21.265
ENSG00000168275	COA6	-1.1	5.6E-03	1.1E-02	61.502	3.756	58.239	65.607	70.043	1.706	68.995	72.011
ENSG00000168310	IRF2	-1.1	8.4E-02	1.3E-01	7.796	0.427	7.449	8.272	8.853	0.062	8.788	8.921
ENSG00000168385	SEPT2	-1.1	3.3E-06	1.3E-05	177.866	2.397	175.426	180.217	201.909	2.547	200.348	204.848
ENSG00000168393	DTYMK	-1.1	4.5E-02	7.3E-02	43.285	1.810	41.590	45.191	47.044	1.603	45.373	48.570
ENSG00000168394	TAP1	-1.1	5.6E-02	8.8E-02	5.970	0.685	5.187	6.453	6.834	0.320	6.469	7.063
ENSG00000168528	SERINC2	-1.1	8.6E-02	1.3E-01	16.766	0.889	16.008	17.744	18.325	1.126	17.043	19.154
ENSG00000168556	ING2	-1.1	8.4E-02	1.3E-01	27.335	2.550	24.815	29.913	30.522	2.083	28.996	32.895
ENSG00000168610	STAT3	-1.1	4.2E-03	8.7E-03	29.964	1.107	29.030	31.187	32.987	1.047	31.783	33.688
ENSG00000168701	TMEM208	-1.1	2.6E-02	4.4E-02	33.860	1.043	32.973	35.010	38.093	3.629	34.145	41.283
ENSG00000168710	AHCYL1	-1.1	7.0E-04	1.7E-03	98.869	2.275	96.247	100.319	109.200	2.435	106.521	111.281
ENSG00000168763	CNNM3	-1.1	4.6E-02	7.4E-02	16.144	1.735	14.299	17.741	18.058	0.490	17.493	18.370
ENSG00000168883	USP39	-1.1	6.0E-05	1.8E-04	67.826	1.966	66.209	70.014	77.301	1.165	76.171	78.498
ENSG00000168904	LRRC28	-1.1	7.6E-02	1.2E-01	3.103	0.333	2.758	3.423	3.477	0.042	3.432	3.515
ENSG00000169021	UQCRFS1	-1.1	7.2E-04	1.8E-03	79.005	1.367	77.441	79.972	89.377	3.998	85.682	93.620
ENSG00000169057	MECP2	-1.1	1.8E-02	3.3E-02	5.548	0.099	5.443	5.639	6.138	0.128	6.051	6.285
ENSG00000169155	ZBTB43	-1.1	8.9E-02	1.3E-01	9.484	0.271	9.255	9.784	10.289	0.414	9.939	10.746
ENSG00000169184	MN1	-1.1	1.2E-01	1.7E-01	3.080	0.209	2.846	3.250	3.465	0.161	3.287	3.598
ENSG00000169188	APEX2	-1.1	1.2E-01	1.7E-01	21.273	1.479	20.287	22.974	22.968	1.012	22.226	24.121
ENSG00000169220	RGS14	-1.1	9.4E-02	1.4E-01	9.986	0.633	9.533	10.710	11.100	0.530	10.627	11.672
ENSG00000169223	LMAN2	-1.1	1.7E-02	3.0E-02	99.305	0.833	98.359	99.929	108.111	5.822	103.759	114.725
ENSG00000169375	SIN3A	-1.1	6.8E-05	2.1E-04	27.303	0.973	26.227	28.120	31.155	0.543	30.817	31.781
ENSG00000169410	PTPN9	-1.1	1.3E-01	1.9E-01	18.631	0.244	18.353	18.811	19.786	1.114	18.917	21.042
ENSG00000169490	TM2D2	-1.1	8.9E-03	1.7E-02	24.001	1.735	22.788	25.988	27.227	1.478	25.964	28.852
ENSG00000169627	BOLA2B	-1.1	5.2E-02	8.2E-02	50.276	1.819	48.208	51.629	55.366	3.605	52.959	59.510
ENSG00000169629	RGPD8	-1.1	6.7E-02	1.0E-01	9.470	0.740	8.616	9.932	10.364	0.120	10.270	10.499
ENSG00000169679	BUB1	-1.1	1.7E-03	3.8E-03	119.229	1.391	117.622	120.035	131.311	0.702	130.733	132.092
ENSG00000169692	AGPAT2	-1.1	3.6E-02	5.9E-02	27.899	1.398	26.303	28.897	31.646	1.691	30.610	33.598
ENSG00000169714	CNBP	-1.1	2.9E-06	1.2E-05	575.631	12.794	566.938	590.322	659.658	3.834	656.977	664.049
ENSG00000169727	GPS1	-1.1	4.8E-04	1.2E-03	40.521	1.248	39.083	41.328	45.660	1.291	44.278	46.835
ENSG00000169738	DCXR	-1.1	4.6E-02	7.4E-02	26.017	2.085	23.698	27.736	29.124	1.250	27.688	29.968
ENSG00000169750	RAC3	-1.1	9.4E-02	1.4E-01	116.936	3.748	112.805	120.118	124.092	6.373	118.471	131.361
ENSG00000169902	TPST1	-1.1	9.4E-02	1.4E-01	11.906	0.814	11.166	12.778	13.237	0.629	12.602	13.858
ENSG00000169905	TOR1AIP2	-1.1	2.7E-02	4.7E-02	6.758	0.022	6.734	6.776	7.405	0.178	7.275	7.608
ENSG00000169925	BRD3	-1.1	3.9E-04	1.0E-03	36.075	0.623	35.363	36.519	40.838	1.958	39.249	43.025
ENSG00000169976	SF3B5	-1.1	9.5E-03	1.8E-02	249.595	7.240	243.749	257.694	275.992	12.906	264.662	290.040
ENSG00000170260	ZNF212	-1.1	1.6E-02	2.9E-02	16.203	0.797	15.712	17.123	18.586	0.965	17.955	19.706
ENSG00000170430	MGMT	-1.1	5.3E-02	8.4E-02	9.133	0.477	8.592	9.492	10.264	0.400	9.857	10.657
ENSG00000170471	RALGAPB	-1.1	2.6E-02	4.4E-02	21.708	0.422	21.296	22.139	23.476	0.479	22.959	23.905
ENSG00000170515	PA2G4	-1.1	5.9E-03	1.2E-02	150.312	6.432	145.286	157.561	162.837	4.565	158.252	167.976
ENSG00000170540	ARL6IP1	-1.1	2.4E-03	5.1E-03	136.485	4.968	130.750	139.472	150.024	2.893	147.266	153.034
ENSG00000170638	TRABD	-1.1	2.2E-02	3.8E-02	34.449	0.643	33.777	35.059	38.427	3.515	35.664	42.383
ENSG00000170776	AKAP13	-1.1	1.5E-02	2.7E-02	10.805	0.467	10.282	11.183	11.758	0.408	11.342	12.157
ENSG00000170846		-1.1	9.1E-02	1.3E-01	21.204	0.988	20.258	22.228	23.123	0.945	22.211	24.097
ENSG00000170860	LSM3	-1.1	2.2E-02	3.9E-02	68.317	1.704	66.372	69.547	73.724	1.523	71.973	74.740
ENSG00000170962	PDGFD	-1.1	7.5E-02	1.1E-01	6.169	0.986	5.536	7.306	7.080	0.568	6.425	7.433
ENSG00000171105	INSR	-1.1	1.0E-01	1.5E-01	9.702	0.398	9.245	9.971	10.477	0.596	10.038	11.156
ENSG00000171202	TMEM126A	-1.1	7.2E-02	1.1E-01	57.957	3.186	54.831	61.199	63.780	2.039	61.572	65.591
ENSG00000171222	SCAND1	-1.1	2.3E-02	4.0E-02	37.878	1.247	36.886	39.279	43.241	5.526	38.701	49.394
ENSG00000171302	CANT1	-1.1	6.2E-03	1.2E-02	16.272	0.462	15.898	16.788	18.506	0.600	17.852	19.031
ENSG00000171316	CHD7	-1.1	1.3E-05	4.5E-05	31.825	0.786	30.921	32.344	36.377	1.446	35.061	37.925
ENSG00000171365	CLCN5	-1.1	9.9E-03	1.9E-02	7.762	0.253	7.475	7.951	8.681	0.227	8.420	8.835
ENSG00000171421	MRPL36	-1.1	2.0E-02	3.6E-02	37.634	3.406	35.573	41.565	43.002	3.523	39.632	46.661
ENSG00000171453	POLR1C	-1.1	1.8E-02	3.2E-02	21.430	2.184	19.491	23.796	24.247	0.373	23.833	24.556
ENSG00000171497	PPID	-1.1	9.6E-03	1.8E-02	70.515	3.748	67.606	74.744	77.854	2.056	75.480	79.058
ENSG00000171617	ENC1	-1.1	6.9E-02	1.1E-01	10.415	0.299	10.091	10.680	11.501	0.643	10.778	12.011
ENSG00000171649	ZIK1	-1.1	2.4E-03	5.3E-03	24.506	1.356	23.400	26.019	27.848	0.470	27.386	28.326
ENSG00000171729	TMEM51	-1.1	1.4E-01	1.9E-01	8.352	1.073	7.363	9.493	9.400	0.364	8.980	9.618
ENSG00000171792	RHNO1	-1.1	4.0E-02	6.5E-02	54.286	0.939	53.636	55.362	58.609	2.448	56.499	61.293
ENSG00000171824	EXOSC10	-1.1	4.8E-03	9.9E-03	41.002	0.908	40.085	41.900	44.989	1.620	43.660	46.794

ENSG00000171827	ZNF570	-1.1	1.2E-01	1.7E-01	3.590	0.190	3.435	3.802	4.017	0.263	3.780	4.299
ENSG00000171861	MRM3	-1.1	3.3E-02	5.6E-02	27.291	1.108	26.161	28.376	30.456	0.800	29.561	31.102
ENSG00000171953	ATPAF2	-1.1	7.6E-02	1.1E-01	5.123	0.314	4.855	5.469	5.772	0.118	5.685	5.906
ENSG00000172037	LAMB2	-1.1	4.9E-02	7.8E-02	29.620	0.535	29.059	30.123	31.654	1.522	30.318	33.311
ENSG00000172172	MRPL13	-1.1	2.9E-02	4.9E-02	47.279	2.247	44.851	49.286	51.288	2.186	49.795	53.798
ENSG00000172244	C5orf34	-1.1	8.8E-02	1.3E-01	12.487	1.019	11.739	13.647	13.948	0.983	12.981	14.945
ENSG00000172586	CHCHD1	-1.1	7.7E-02	1.2E-01	81.448	0.942	80.537	82.418	87.302	0.522	86.709	87.693
ENSG00000172590	MRPL52	-1.1	2.1E-03	4.7E-03	53.381	0.935	52.839	54.460	58.820	2.617	56.841	61.751
ENSG00000172725	CORO1B	-1.1	3.4E-04	9.0E-04	32.169	1.663	30.380	33.669	37.058	1.693	36.056	39.012
ENSG00000172731	LRRC20	-1.1	8.5E-03	1.6E-02	23.845	1.493	22.613	25.505	26.902	0.320	26.567	27.205
ENSG00000172845	SP3	-1.1	1.6E-02	2.9E-02	30.974	2.625	28.032	33.076	33.785	1.688	32.536	35.718
ENSG00000172869	DMXL1	-1.1	1.1E-01	1.6E-01	16.255	0.063	16.213	16.327	17.151	0.344	16.766	17.430
ENSG00000172915	NBEA	-1.1	2.3E-03	5.1E-03	16.188	0.774	15.468	17.008	18.071	0.606	17.386	18.529
ENSG00000173011	TADA2B	-1.1	3.5E-02	5.8E-02	6.276	0.343	5.932	6.618	7.110	0.217	6.894	7.328
ENSG00000173113	TRMT112	-1.1	8.6E-02	1.3E-01	196.785	8.667	187.030	203.598	207.884	6.410	200.521	212.222
ENSG00000173156	RHOD	-1.1	9.7E-02	1.4E-01	14.502	1.693	12.934	16.296	16.533	0.866	15.570	17.247
ENSG00000173171	MTX1	-1.1	5.4E-03	1.1E-02	22.515	0.869	21.767	23.469	25.768	1.406	24.166	26.793
ENSG00000173175	ADCY5	-1.1	1.4E-02	2.6E-02	5.903	0.206	5.769	6.141	6.745	0.053	6.685	6.782
ENSG00000173226	IQCB1	-1.1	5.0E-03	1.0E-02	47.816	0.383	47.381	48.103	52.887	1.058	52.091	54.088
ENSG00000173230	GOLGB1	-1.1	4.3E-04	1.1E-03	28.468	2.744	25.393	30.666	32.441	1.296	31.375	33.883
ENSG00000173258	ZNF483	-1.1	5.7E-02	8.9E-02	8.291	1.023	7.424	9.420	9.293	0.311	9.041	9.640
ENSG00000173456	RNF26	-1.1	1.9E-02	3.4E-02	84.144	0.153	83.975	84.276	91.388	5.795	85.407	96.975
ENSG00000173473	SMARCC1	-1.1	2.5E-03	5.3E-03	207.028	3.181	203.658	209.979	223.978	3.121	222.021	227.578
ENSG00000173744	AGFG1	-1.1	7.5E-02	1.1E-01	35.237	0.641	34.707	35.950	37.356	2.235	35.009	39.458
ENSG00000173821	RNF213	-1.1	1.7E-03	3.9E-03	9.820	0.628	9.103	10.277	10.970	0.739	10.526	11.823
ENSG00000173905	GOLIM4	-1.1	9.3E-02	1.4E-01	28.654	1.216	27.402	29.830	30.601	0.258	30.313	30.811
ENSG00000173933	RBM4	-1.1	6.5E-02	1.0E-01	5.171	0.649	4.425	5.604	5.970	0.558	5.363	6.460
ENSG00000173992	CCS	-1.1	1.2E-01	1.8E-01	17.682	0.483	17.190	18.155	19.370	1.173	18.689	20.724
ENSG00000174010	KLHL15	-1.1	9.7E-03	1.9E-02	12.535	0.643	12.132	13.276	14.157	0.946	13.110	14.951
ENSG00000174106	LEMD3	-1.1	7.9E-04	1.9E-03	30.067	0.518	29.665	30.651	33.937	0.431	33.644	34.432
ENSG00000174437	ATP2A2	-1.1	4.4E-06	1.7E-05	66.187	1.756	64.873	68.181	75.528	0.302	75.260	75.856
ENSG00000174547	MRPL11	-1.1	1.2E-03	2.7E-03	84.596	0.261	84.439	84.898	94.568	3.211	91.438	97.854
ENSG00000174606	ANGEL2	-1.1	1.1E-02	2.0E-02	14.291	0.456	13.921	14.793	15.928	0.505	15.349	16.280
ENSG00000174652	ZNF266	-1.1	5.6E-02	8.9E-02	15.003	1.626	13.406	16.656	16.509	0.547	15.896	16.947
ENSG00000174695	TMEM167A	-1.1	1.4E-04	3.9E-04	185.585	8.836	177.495	195.015	208.955	9.041	200.654	218.588
ENSG00000174738	NR1D2	-1.1	1.3E-01	1.8E-01	17.120	0.512	16.784	17.710	18.321	1.231	16.940	19.301
[illegible]	[illegible]	[illegible]	[illegible]	[illegible]	[illegible]	[illegible]	[illegible]	[illegible]	[illegible]	[illegible]	[illegible]	[illegible]

ENSG00000079102	RUNX1T1	1.1	2.3E-02	4.0E-02	5.132	0.188	4.915	5.253	4.554	0.259	4.302	4.820
ENSG00000079950	STX7	1.1	1.7E-04	4.6E-04	11.698	0.426	11.388	12.184	10.239	0.241	9.961	10.391
ENSG00000080200	CRYBG3	1.1	5.5E-02	8.6E-02	8.476	0.550	7.881	8.964	7.809	0.180	7.603	7.937
ENSG00000080371	RAB21	1.1	5.1E-03	1.0E-02	8.742	0.381	8.308	9.020	7.814	0.530	7.306	8.364
ENSG00000080608	PUM3	1.1	8.3E-03	1.6E-02	109.006	2.837	105.834	111.300	100.358	4.083	95.771	103.594
ENSG00000080802	CNOT4	1.1	7.5E-02	1.1E-01	10.660	0.167	10.563	10.854	9.771	0.973	8.718	10.638
ENSG00000080819	CPOX	1.1	4.2E-02	6.8E-02	39.441	1.089	38.692	40.691	36.317	1.188	35.448	37.685
ENSG00000080822	CLDND1	1.1	6.0E-04	1.5E-03	35.428	1.966	34.150	37.692	31.181	0.453	30.660	31.476
ENSG00000081014	AP4E1	1.1	1.1E-01	1.6E-01	12.027	0.631	11.410	12.672	11.217	0.212	11.025	11.444
ENSG00000081181	ARG2	1.1	1.2E-02	2.2E-02	72.137	2.165	70.153	74.447	66.093	3.568	62.045	68.785
ENSG00000081307	UBA5	1.1	1.0E-03	2.4E-03	27.944	1.010	27.130	29.075	24.720	1.387	23.669	26.293
ENSG00000081791	KIAA0141	1.1	2.4E-02	4.2E-02	24.228	0.488	23.874	24.785	22.384	0.318	22.122	22.738
ENSG00000082258	CCNT2	1.1	2.7E-02	4.6E-02	26.404	0.513	25.818	26.776	24.601	1.489	23.470	26.289
ENSG00000083223	ZCCHC6	1.1	7.1E-02	1.1E-01	18.527	1.286	17.490	19.966	17.312	0.282	17.084	17.627
ENSG00000083444	PLOD1	1.1	1.3E-02	2.4E-02	31.211	1.007	30.335	32.311	28.626	0.782	27.938	29.477
ENSG00000084070	SMAP2	1.1	1.1E-01	1.6E-01	6.264	0.510	5.722	6.735	5.527	0.678	4.769	6.079
ENSG00000084092	NOA1	1.1	1.8E-03	4.0E-03	46.069	1.249	45.217	47.502	41.107	1.445	39.970	42.734
ENSG00000084207	GSTP1	1.1	1.0E-02	2.0E-02	770.837	20.840	747.539	787.703	725.774	13.927	716.325	741.768
ENSG00000084234	APLP2	1.1	3.6E-03	7.6E-03	208.520	8.153	202.398	217.774	194.500	1.264	193.539	195.931
ENSG00000084623	EIF3I	1.1	9.8E-03	1.9E-02	341.495	8.372	334.077	350.573	319.650	6.647	312.011	324.107
ENSG00000084652	TXLNA	1.1	2.1E-04	5.7E-04	45.806	0.311	45.501	46.122	40.287	1.436	38.640	41.271
ENSG00000084731	KIF3C	1.1	1.3E-01	1.8E-01	33.028	0.429	32.532	33.285	31.527	1.290	30.477	32.967
ENSG00000084733	RAB10	1.1	1.3E-02	2.5E-02	131.130	3.999	127.462	135.393	122.351	6.805	115.106	128.609
ENSG00000085224	ATRX	1.1	7.4E-04	1.8E-03	29.224	2.554	26.309	31.064	25.978	1.436	24.903	27.609
ENSG00000085365	SCAMP1	1.1	5.3E-02	8.4E-02	45.827	1.309	44.346	46.827	42.975	2.785	41.137	46.180
ENSG00000085377	PREP	1.1	1.8E-04	5.1E-04	84.633	3.289	82.205	88.375	74.196	3.965	69.696	77.173
ENSG00000085382	HACE1	1.1	3.9E-03	8.2E-03	9.981	0.415	9.681	10.455	8.826	0.312	8.488	9.103
ENSG00000085662	AKR1B1	1.1	9.4E-03	1.8E-02	110.013	3.158	106.465	112.515	101.794	4.277	97.451	106.001
ENSG00000086065	CHMP5	1.1	7.3E-02	1.1E-01	74.754	1.741	72.751	75.896	70.092	6.027	63.674	75.630
ENSG00000086504	MRPL28	1.1	7.2E-02	1.1E-01	80.515	3.922	77.446	84.934	76.006	2.166	73.505	77.257
ENSG00000087086	FTL	1.1	2.4E-05	8.0E-05	3260.744	53.015	3204.966	3310.477	2883.583	193.719	2698.613	3085.002
ENSG00000087088	BAX	1.1	1.2E-04	3.5E-04	107.540	3.124	103.988	109.865	94.677	2.403	92.116	96.882
ENSG00000087338	GMCL1	1.1	5.9E-02	9.2E-02	28.338	0.929	27.345	29.186	26.235	1.838	24.508	28.168
ENSG00000087460	GNAS	1.1	5.1E-05	1.6E-04	138.472	2.528	136.034	141.081	123.044	4.450	117.906	125.695
ENSG00000088298	EDEM2	1.1	1.3E-01	1.8E-01	22.348	0.647	21.710	23.004	20.520	1.447	18.853	21.460
ENSG00000088367	EPB41L1	1.1	1.1E-01	1.5E-01	11.439	1.050	10.818	12.651	10.662	0.239	10.522	10.938
ENSG00000088812	ATRN	1.1	4.5E-04	1.2E-03	20.029	0.149	19.898	20.191	17.473	0.871	16.833	18.464
ENSG00000089289	IGBP1	1.1	2.6E-02	4.5E-02	77.355	7.238	71.323	85.381	70.343	4.328	65.362	73.183
ENSG00000089693	MLF2	1.1	6.3E-02	9.8E-02	121.429	2.138	119.097	123.296	115.675	4.788	110.169	118.871
ENSG00000089916	GPATCH2L	1.1	9.4E-03	1.8E-02	15.061	0.154	14.951	15.238	13.908	0.705	13.105	14.426
ENSG00000090054	SPTLC1	1.1	8.2E-02	1.2E-01	59.827	0.538	59.361	60.415	56.800	1.216	55.492	57.897
ENSG00000091136	LAMB1	1.1	2.1E-02	3.7E-02	24.253	1.581	23.145	26.063	22.434	0.538	21.846	22.900
ENSG00000091527	CDV3	1.1	5.3E-04	1.3E-03	156.822	5.213	152.209	162.477	142.575	2.265	139.960	143.925
ENSG00000092036	HAUS4	1.1	5.2E-03	1.0E-02	30.016	0.877	29.024	30.689	26.503	0.624	25.967	27.188
ENSG00000092108	SCFD1	1.1	1.1E-01	1.6E-01	35.395	0.731	34.946	36.238	33.727	1.448	32.207	35.089
ENSG00000092964	DPYSL2	1.1	1.2E-02	2.3E-02	67.257	3.194	64.888	70.889	62.666	3.179	59.088	65.166
ENSG00000094975	SUCO	1.1	1.3E-04	3.6E-04	37.174	0.615	36.603	37.825	32.675	1.821	31.284	34.736
ENSG00000095209	TMEM38B	1.1	9.8E-02	1.4E-01	9.107	0.329	8.731	9.341	8.295	0.591	7.920	8.976
ENSG00000095637	SORBS1	1.1	3.3E-02	5.6E-02	13.915	0.655	13.296	14.601	12.952	0.179	12.806	13.151
ENSG00000095794	CREM	1.1	3.8E-02	6.2E-02	9.727	0.601	9.073	10.254	8.658	0.515	8.264	9.241
ENSG00000095951	HIVEP1	1.1	8.6E-02	1.3E-01	6.658	0.701	5.848	7.067	6.093	0.549	5.481	6.540
ENSG00000096717	SIRT1	1.1	6.4E-02	9.9E-02	51.178	0.766	50.301	51.717	48.369	0.893	47.513	49.294
ENSG00000096746	HNRNPH3	1.1	5.7E-03	1.1E-02	186.147	6.303	179.916	192.520	173.224	5.700	168.787	179.803
ENSG00000097033	SH3GLB1	1.1	1.0E-04	3.0E-04	29.915	0.417	29.446	30.242	26.180	0.689	25.425	26.776
ENSG00000099797	TECR	1.1	5.8E-03	1.2E-02	21.844	0.896	21.035	22.807	19.430	0.456	18.904	19.711
ENSG00000099999	RNF215	1.1	5.3E-02	8.4E-02	3.073	0.199	2.864	3.259	2.692	0.397	2.375	3.136
ENSG00000100029	PES1	1.1	5.8E-05	1.8E-04	77.010	2.594	74.018	78.629	67.863	0.784	67.223	68.738
ENSG00000100099	HPS4	1.1	1.6E-02	2.9E-02	13.608	0.378	13.184	13.911	12.320	0.748	11.505	12.976
ENSG00000100129	EIF3L	1.1	5.4E-05	1.7E-04	185.234	8.262	176.625	193.098	164.049	6.493	158.580	171.224
ENSG00000100150	DEPDC5	1.1	1.2E-01	1.7E-01	5.997	0.330	5.777	6.377	5.501	0.200	5.283	5.678
ENSG00000100201	DDX17	1.1	3.6E-03	7.5E-03	216.638	9.649	205.649	223.723	200.963	4.699	197.074	206.184
ENSG00000100207	TCF20	1.1	7.7E-02	1.2E-01	40.038	2.069	37.667	41.481	38.089	0.199	37.939	38.315
ENSG00000100221	JOSD1	1.1	8.1E-02	1.2E-01	27.523	0.326	27.188	27.839	25.780	0.519	25.219	26.243
ENSG00000100226	GTPBP1	1.1	3.8E-03	7.8E-03	45.042	0.706	44.437	45.818	41.149	0.924	40.165	41.997
ENSG00000100243	CYB5R3	1.1	5.8E-03	1.2E-02	42.370	0.651	41.714	43.016	38.463	2.823	36.179	41.619
ENSG00000100302	RASD2	1.1	1.2E-01	1.7E-01	4.868	0.081	4.805	4.959	4.260	0.386	3.848	4.615
ENSG00000100319	ZMAT5	1.1	5.6E-02	8.8E-02	8.067	0.470	7.598	8.537	7.061	0.247	6.861	7.337
ENSG00000100354	TNRC6B	1.1	5.8E-02	9.0E-02	10.235	0.349	10.003	10.637	9.644	0.245	9.410	9.898
ENSG00000100387	RBX1	1.1	2.8E-04	7.5E-04	69.182	2.932	66.219	72.083	60.905	1.362	59.714	62.390
ENSG00000100416	TRMU	1.1	7.6E-03	1.5E-02	14.465	0.929	13.466	15.303	12.649	0.490	12.087	12.987
ENSG00000100439	ABHD4	1.1	3.6E-03	7.5E-03	56.608	1.724	55.476	58.592	51.437	1.855	49.389	53.004
ENSG00000100528	CNIH1	1.1	3.0E-03	6.4E-03	60.694	2.781	57.485	62.411	54.886	2.321	52.443	57.062
ENSG00000100567	PSMA3	1.1	2.6E-02	4.5E-02	188.147	4.996	182.564	192.196	177.340	4.174	173.218	181.563
ENSG00000100568	VTI1B	1.1	4.2E-03	8.6E-03	42.886	1.297	41.779	44.313	39.252	0.987	38.275	40.249
ENSG00000100575	TIMM9	1.1	7.7E-02	1.2E-01	63.587	1.253	62.331	64.837	58.728	1.184	57.818	60.066
ENSG00000100580	TMED8	1.1	1.4E-01	2.0E-01	20.963	0.469	20.436	21.335	19.989	0.783	19.417	20.882
ENSG00000100600	LGMN	1.1	1.2E-02	2.2E-02	44.993	0.622	44.614	45.711	41.288	1.587	39.624	42.808
ENSG00000100614	PPM1A	1.1	1.9E-03	4.3E-03	22.005	0.834	21.472	22.966	19.815	0.881	18.799	20.354
ENSG00000100934	SEC23A	1.1	7.3E-02	1.1E-01	32.901	0.612	32.371	33.571	31.339	0.557	31.007	31.982
ENSG00000100941	PNN	1.1	4.2E-04	1.1E-03	199.471	14.531	182.823	209.608	179.903	2.569	176.937	181.418
ENSG00000101000	PROCR	1.1	9.7E-02	1.4E-01	24.990	1.406	23.893	26.574	22.714	1.834	21.056	24.683
ENSG00000101017	CD40	1.1	6.4E-02	9.9E-02	14.441	0.202	14.260	14.659	12.910	1.179	11.553	13.686
ENSG00000101158	NELFCD	1.1	3.4E-03	7.2E-03	27.434	0.589	26.762	27.859	24.789	0.944	23.799	25.679
ENSG00000101216	GMEB2	1.1	8.2E-02	1.2E-01	16.255	1.076	15.013	16.897	14.915	0.606	14.215	15.267
ENSG00000101365	IDH3B	1.1	1.2E-03	2.9E-03	70.806	2.064	68.518	72.528	63.385	0.792	62.906	64.299
ENSG00000101574	METTL4	1.1	6.7E-02	1.0E-01	8.769	0.131	8.621	8.868	7.965	0.050	7.909	8.004
ENSG00000101695	RNF125	1.1	6.2E-03	1.2E-02	13.708	0.649	13.021	14.310	11.980	1.002	11.105	13.073
ENSG00000101871	MID1	1.1	1.1E-02	2.1E-02	33.214	1.389	32.148	34.786	30.300	0.332	29.942	30.599
ENSG00000101888	NXT2	1.1	4.2E-02	6.8E-02	27.230	0.651	26.850	27.983	24.984	1.245	23.676	26.155
ENSG00000101955	SRPX	1.1	6.5E-02	1.0E-01	12.325	0.524	11.736	12.739	10.735	1.063	9.623	11.740
ENSG00000102024	PLS3	1.1	8.3E-06	3.0E-05	289.629	3.366	286.004	292.657	255.379	9.773	244.680	263.837
ENSG00000102531	FNDC3A	1.1	3.6E-02	5.9E-02	25.673	0.566	25.023	26.062	24.000	1.114	22.886	25.114
ENSG00000102547	CAB39L	1.1	5.2E-02	8.3E-02	5.609	0.656	5.027	6.321	4.897	0.291	4.690	5.230
ENSG00000102572	STK24	1.1	3.8E-04	1.0E-03	43.227	0.062	43.161	43.285	39.072	1.103	37.828	39.930
ENSG00000102897	LYRM1	1.1	1.0E-01	1.5E-01	14.441	1.533	12.980	16.038	13.141	0.197	12.942	13.335
ENSG00000102898	NUTF2	1.1	1.6E-04	4.6E-04	120.071	1.385	118.645	121.412	107.507	4.288	103.287	111.860
ENSG00000103021	CCDC113	1.1	9.6E-03	1.8E-02	10.465	0.691	9.766	11.146	9.200	0.261	8.979	9.488
ENSG00000103126	AXIN1	1.1	7.8E-04	1.9E-03	21.531	0.864	20.795	22.482	18.846	0.882	18.027	19.780
ENSG00000103148	NPRL3	1.1	4.7E-02	7.5E-02	8.010	0.431	7.594	8.454	7.198	0.487	6.637	7.510
ENSG00000103160	HSDL1	1.1	3.6E-03	7.6E-03	49.816	1.671	47.936	51.130	45.011	3.363	42.796	48.881
ENSG00000103202	NME4	1.1	1.2E-01	1.8E-01	116.084	1.351	114.524	116.876	109.075	11.468	99.038	121.574
ENSG00000103257	SLC7A5	1.1	4.2E-02	6.8E-02	284.720	3.721	280.598	287.830	256.770	10.938	245.507	267.350
ENSG00000103264	FBXO31	1.1	1.2E-01	1.7E-01	6.764	0.163	6.613	6.938	6.217	0.546	5.732	6.808
ENSG00000103363	ELOB	1.1	5.9E-02	9.2E-02	226.500	12.887	213.911	239.665	212.633	4.219	209.780	217.478
ENSG00000103485	QPRT	1.1	2.4E-03	5.3E-03	81.852	4.165	77.145	85.057	74.092	1.173	72.797	75.083
ENSG00000103489	XYLT1	1.1	7.5E-02	1.1E-01	14.344	0.498	13.851	14.847	13.400	0.500	12.904	13.904
ENSG00000103544	C16orf62	1.1	2.4E-02	4.1E-02	19.264	0.589	18.600	19.727	17.705	0.519	17.201	18.238
ENSG00000103671	TRIP4	1.1	9.4E-02	1.4E-01	17.015	0.165	16.827	17.133	15.443	1.056	14.485	16.575
ENSG00000104412	EMC2	1.1	1.1E-01	1.6E-01	33.743	1.877	31.801	35.546	31.211	2.802	29.338	34.433
ENSG00000104427	ZC2HC1A	1.1	1.0E-01	1.5E-01	12.521	1.258	11.075	13.365	11.422	1.254	10.339	12.796
ENSG00000104472	CHRAC1	1.1	2.5E-03	5.4E-03	34.229	1.350	32.965	35.650	29.945	2.113	27.553	31.560
ENSG00000104529	EEF1D	1.1	5.1E-03	1.0E-02	75.700	3.512	71.881	78.791	69.855	3.004	68.013	73.322
ENSG00000104723	TUSC3	1.1	1.1E-02	2.1E-02	46.894	1.093	45.642	47.664	43.289	0.835	42.459	44.128
ENSG00000104774	MAN2B1	1.1	2.2E-02	3.8E-02	20.124	0.555	19.512	20.593	18.395	0.852	17.416	18.967
ENSG00000104824	HNRNPL	1.1	8.1E-04	2.0E-03	133.197	1.957	131.976	135.454	121.813	3.977	118.756	126.309
ENSG00000104904	OAZ1	1.1	1.0E-02	1.9E-02	277.460	9.088	267.076	283.964	258.508	16.118	244.739	276.237
ENSG00000104964	AES	1.1	4.3E-03	8.9E-03	55.595	1.397	54.474	57.160	50.430	1.264	49.366	51.827
ENSG00000104976	SNAPC2	1.1	1.1E-01	1.6E-01	18.339	1.449	16.778	19.641	16.585	1.472	14.947	17.795
ENSG00000104979	C19orf53	1.1	8.2E-03	1.6E-02	116.219	2.006	114.331	118.325	104.992	3.486	102.031	108.834
ENSG00000105186	ANKRD27	1.1	3.3E-04	8.6E-04	32.256	0.665	31.782	33.017	28.546	1.682	27.244	30.445
ENSG00000105248	CCDC94	1.1	5.4E-02	8.6E-02	34.870	1.710	33.345	36.719	31.682	0.393	31.255	32.017
ENSG00000105254	TBCB	1.1	8.6E-02	1.3E-01	48.225	0.697	47.474	48.852	45.546	2.188	43.217	47.559
ENSG00000105325	FZR1	1.1	1.2E-01	1.7E-01	14.655	0.907	13.913	15.667	13.564	0.605	13.025	14.219
ENSG00000105443	CYTH2	1.1	1.8E-03	4.0E-03	12.252	0.485	11.719	12.669	10.766	0.424	10.468	11.252
ENSG00000105499	PLA2G4C	1.1	5.5E-02	8.6E-02	7.019	0.262	6.745	7.267	6.329	0.192	6.124	6.506
ENSG00000105568	PPP2R1A	1.1	1.2E-03	2.7E-03	133.664	5.404	130.524	139.904	121.615	4.502	116.416	124.240
ENSG00000105612	DNASE2	1.1	7.2E-02	1.1E-01	30.902	3.078	28.808	34.436	28.257	1.786	26.254	29.681
ENSG00000105655	ISYNA1	1.1	1.3E-01	1.8E-01	82.565	4.178	77.934	86.053	78.735	4.049	75.331	83.213
ENSG00000105669	COPE	1.1	1.9E-02	3.3E-02	67.258	2.793	65.322	70.460	61.673	1.304	60.672	63.147
ENSG00000105722	ERF	1.1	1.7E-02	3.0E-02	41.113	1.801	39.255	42.850	37.148	2.196	35.272	39.564
ENSG00000105819	PMPCB	1.1	7.0E-05	2.1E-04	54.891	1.761	53.081	56.600	47.988	1.038	47.352	49.186
ENSG00000105894	PTN	1.1	8.8E-02	1.3E-01	61.797	5.804	57.487	68.396	57.507	1.313	56.593	59.011
ENSG00000105993	DNAJB6	1.1	2.0E-05	6.7E-05	38.304	0.967	37.675	39.417	33.852	0.845	32.899	34.510
ENSG00000106105	GARS	1.1	1.5E-05	5.2E-05	285.566	5.138	279.776	289.579	253.137	5.088	249.568	258.963
ENSG00000106236	NPTX2	1.1	5.6E-02	8.8E-02	18.392	1.011	17.255	19.191	16.345	0.853	15.423	17.108
ENSG00000106244	PDAP1	1.1	7.2E-04	1.8E-03	172.711	3.171	169.050	174.547	156.319	3.316	152.599	158.967
ENSG00000106397	PLOD3	1.1	1.2E-01	1.7E-01	13.526	0.886	12.901	14.540	12.457	1.338	11.181	13.850
ENSG00000106399	RPA3	1.1	8.8E-02	1.3E-01	59.281	3.625	55.747	62.991	55.727	4.599	50.461	58.953
ENSG00000106610	STAG3L4	1.1	1.2E-01	1.7E-01	11.183	1.184	10.337	12.536	10.082	0.646	9.623	10.821
ENSG00000106624	AEBP1	1.1	3.8E-02	6.3E-02	18.166	1.375	17.332	19.753	16.525	1.313	15.111	17.705
ENSG00000106665	CLIP2	1.1	2.0E-03	4.4E-03	29.649	2.975	26.396	32.231	25.924	1.502	24.195	26.900
ENSG00000107223	EDF1	1.1	6.3E-02	9.7E-02	299.483	2.359	296.774	301.081	284.505	10.761	278.096	296.929
ENSG00000107554	DNMBP	1.1	1.1E-02	2.0E-02	17.188	0.362	16.825	17.549	15.628	0.466	15.090	15.919
ENSG00000107614	TRDMT1	1.1	1.3E-01	1.8E-01	7.167	0.506	6.593	7.545	6.695	0.131	6.566	6.829
ENSG00000107902	LHPP	1.1	8.6E-02	1.3E-01	16.274	0.477	15.913	16.815	14.917	0.521	14.333	15.334
ENSG00000108010	GLRX3	1.1	1.2E-01	1.7E-01	74.131	5.561	68.106	79.069	70.611	2.206	69.055	73.136
ENSG00000108100	CCNY	1.1	3.5E-03	7.3E-03	34.761	0.520	34.171	35.154	31.627	0.354	31.390	32.034
ENSG00000108370	RGS9	1.1	2.1E-02	3.6E-02	6.306	0.085	6.245	6.404	5.547	0.205	5.376	5.774
ENSG00000108465	CDK5RAP3	1.1	4.4E-02	7.2E-02	17.412	0.468	16.877	17.749	16.095	0.349	15.695	16.332
ENSG00000108506	INTS2	1.1	2.7E-03	5.8E-03	21.918	0.070	21.840	21.971	19.460	1.948	17.556	21.449
ENSG00000108518	PFN1	1.1	2.3E-05	7.6E-05	1251.111	18.110	1234.791	1270.594	1107.565	62.736	1040.561	1164.912
ENSG00000108523	RNF167	1.1	1.4E-03	3.2E-03	93.817	3.552	90.388	97.481	84.730	2.360	82.214	86.897
ENSG00000108561	C1QBP	1.1	3.6E-03	7.5E-03	385.864	5.497	379.703	390.268	355.454	20.929	331.303	368.310
ENSG00000108854	SMURF2	1.1	8.2E-02	1.2E-01	18.835	0.481	18.404	19.354	17.713	0.503	17.309	18.277
ENSG00000108946	PRKAR1A	1.1	5.2E-05	1.6E-04	65.003	2.840	62.194	67.873	57.408	1.113	56.416	58.611
ENSG00000109171	SLAIN2	1.1	3.3E-02	5.5E-02	22.422	0.736	21.621	23.067	20.582	0.438	20.337	21.098
ENSG00000109180	OCIAD1	1.1	9.2E-02	1.4E-01	97.003	1.480	95.312	98.059	92.641	5.337	86.781	97.225
ENSG00000109270	LAMTOR3	1.1	1.0E-01	1.5E-01	20.750	0.514	20.357	21.331	19.284	0.719	18.457	19.761
ENSG00000109381	ELF2	1.1	6.4E-02	9.9E-02	15.639	0.296	15.444	15.979	14.567	0.773	13.748	15.283
ENSG00000109475	RPL34	1.1	2.8E-05	9.3E-05	351.074	21.917	335.101	376.061	307.197	12.450	293.556	317.946
ENSG00000109670	FBXW7	1.1	3.0E-02	5.1E-02	9.553	0.102	9.453	9.657	8.686	0.703	8.033	9.430
ENSG00000109674	NEIL3	1.1	1.1E-01	1.6E-01	18.119	1.583	16.588	19.748	16.650	0.809	15.807	17.420
ENSG00000109736	MFSD10	1.1	8.2E-02	1.2E-01	46.850	1.703	45.010	48.371	43.673	3.688	39.488	46.447
ENSG00000109911	ELP4	1.1	7.3E-03	1.4E-02	3.087	0.272	2.838	3.376	2.701	0.162	2.523	2.839
ENSG00000109920	FNBP4	1.1	4.1E-04	1.1E-03	34.111	1.449	32.451	35.116	30.344	0.281	30.030	30.572
ENSG00000110048	OSBP	1.1	1.7E-02	3.0E-02	44.684	0.476	44.156	45.079	41.350	1.782	39.297	42.495
ENSG00000110063	DCPS	1.1	1.2E-01	1.7E-01	18.962	0.751	18.281	19.767	17.626	1.134	16.378	18.591
ENSG00000110066	KMT5B	1.1	1.2E-03	2.8E-03	18.687	0.577	18.103	19.256	16.538	0.699	15.797	17.186
ENSG00000110660	SLC35F2	1.1	1.2E-01	1.7E-01	26.281	1.974	24.193	28.116	24.680	0.587	24.018	25.104
ENSG00000110700	RPS13	1.1	3.2E-06	1.3E-05	677.280	14.194	668.992	693.670	591.446	22.427	565.566	605.193
ENSG00000110906	KCTD10	1.1	2.2E-02	3.9E-02	19.634	0.415	19.235	20.063	18.134	0.104	18.057	18.252
ENSG00000110958	PTGES3	1.1	2.3E-03	5.0E-03	502.533	15.050	486.178	515.798	464.532	12.880	449.934	474.295
ENSG00000111203	ITFG2	1.1	6.3E-02	9.8E-02	8.115	0.405	7.791	8.569	7.325	0.315	7.134	7.688
ENSG00000111229	ARPC3	1.1	2.9E-04	7.7E-04	104.149	0.304	103.860	104.466	92.858	0.239	92.696	93.133
ENSG00000111231	GPN3	1.1	4.5E-03	9.1E-03	68.015	2.045	66.628	70.364	61.484	0.929	60.842	62.550
ENSG00000111269	CREBL2	1.1	5.9E-02	9.2E-02	29.747	1.142	28.433	30.500	27.645	0.802	27.154	28.570
ENSG00000111358	GTF2H3	1.1	2.3E-02	4.0E-02	37.129	0.459	36.599	37.397	34.257	0.724	33.510	34.957
ENSG00000111450	STX2	1.1	1.5E-02	2.8E-02	30.515	1.512	28.772	31.470	27.064	1.680	25.176	28.436
ENSG00000111481	COPZ1	1.1	4.3E-02	7.0E-02	74.240	0.774	73.566	75.085	70.150	1.520	68.430	71.310
ENSG00000111596	CNOT2	1.1	1.4E-04	3.9E-04	22.015	1.088	20.834	22.975	19.436	0.316	19.081	19.686
ENSG00000111615	KRR1	1.1	1.4E-02	2.6E-02	28.805	1.360	27.240	29.703	26.688	1.356	25.832	28.252
ENSG00000111653	ING4	1.1	8.3E-02	1.2E-01	18.271	1.446	17.027	19.858	16.431	0.428	15.999	16.855
ENSG00000111716	LDHB	1.1	1.7E-02	3.0E-02	1520.968	19.956	1499.898	1539.582	1443.918	23.049	1427.735	1470.308
ENSG00000111752	PHC1	1.1	4.7E-04	1.2E-03	230.219	4.517	225.048	233.390	208.723	6.567	204.132	216.245
ENSG00000111775	COX6A1	1.1	1.2E-02	2.2E-02	100.617	5.767	95.390	106.804	91.034	4.358	87.115	95.728
ENSG00000111786	SRSF9	1.1	4.9E-02	7.8E-02	147.099	1.276	146.231	148.564	138.918	6.402	131.611	143.537
ENSG00000111846	GCNT2	1.1	1.1E-04	3.3E-04	32.689	0.641	31.974	33.211	28.657	0.892	27.717	29.492
ENSG00000111880	RNGTT	1.1	2.5E-04	6.8E-04	45.742	0.614	45.037	46.162	40.335	0.967	39.278	41.173
ENSG00000111885	MAN1A1	1.1	4.1E-02	6.7E-02	6.155	0.428	5.818	6.637	5.389	0.236	5.215	5.657
ENSG00000111911	HINT3	1.1	6.1E-02	9.5E-02	17.154	1.099	15.975	18.149	15.589	1.527	13.826	16.481
ENSG00000112033	PPARD	1.1	4.2E-02	6.8E-02	14.500	1.498	12.886	15.883	13.055	0.799	12.223	13.816
ENSG00000112159	MDN1	1.1	3.5E-02	5.8E-02	104.641	3.540	100.615	107.268	99.114	1.328	97.951	100.560
ENSG00000112282	MED23	1.1	1.0E-01	1.5E-01	14.166	0.478	13.665	14.619	13.287	0.348	12.898	13.569
ENSG00000112304	ACOT13	1.1	9.7E-03	1.8E-02	43.524	0.646	42.785	43.976	39.856	1.864	37.926	41.647
ENSG00000112306	RPS12	1.1	5.1E-06	1.9E-05	3061.161	104.634	2988.809	3181.961	2699.768	27.444	2680.398	2731.174
ENSG00000112339	HBS1L	1.1	9.2E-03	1.8E-02	29.683	0.455	29.375	30.205	27.489	1.211	26.145	28.494
ENSG00000112367	FIG4	1.1	2.7E-03	5.9E-03	33.772	0.492	33.259	34.240	30.192	0.732	29.400	30.843
ENSG00000112514	CUTA	1.1	1.1E-04	3.2E-04	147.187	4.478	142.339	151.158	129.497	5.124	123.903	133.964
ENSG00000112531	QKI	1.1	6.0E-02	9.3E-02	37.698	1.664	35.939	39.247	35.846	1.873	33.698	37.139
ENSG00000112695	COX7A2	1.1	7.1E-02	1.1E-01	182.077	3.574	179.176	186.070	173.335	6.217	166.160	177.118
ENSG00000112697	TMEM30A	1.1	3.0E-02	5.0E-02	103.816	4.941	98.129	107.050	91.120	4.495	86.401	95.352
ENSG00000112701	SENP6	1.1	5.2E-04	1.3E-03	34.252	0.759	33.618	35.092	30.935	1.003	30.082	32.040
ENSG00000112742	TTK	1.1	9.2E-02	1.4E-01	80.621	4.619	77.810	85.952	75.484	6.368	70.013	82.474
ENSG00000112893	MAN2A1	1.1	5.0E-05	1.6E-04	49.687	0.564	49.251	50.324	43.610	1.394	42.542	45.187
ENSG00000112977	DAP	1.1	5.5E-02	8.6E-02	39.617	1.927	37.398	40.858	36.833	0.665	36.159	37.488
ENSG00000112984	KIF20A	1.1	1.6E-03	3.7E-03	101.229	3.609	97.318	104.431	91.913	2.190	90.093	94.344
ENSG00000113048	MRPS27	1.1	1.4E-04	3.9E-04	75.558	2.021	73.796	77.765	67.604	1.577	66.208	69.314
ENSG00000113163	COL4A3BP	1.1	1.9E-02	3.4E-02	30.357	0.492	29.851	30.834	28.199	1.675	27.013	30.116
ENSG00000113269	RNF130	1.1	8.4E-04	2.0E-03	18.483	0.034	18.454	18.521	16.468	1.100	15.274	17.441
ENSG00000113387	SUB1	1.1	4.5E-04	1.1E-03	89.009	2.906	85.725	91.246	80.289	3.764	76.678	84.189
ENSG00000113615	SEC24A	1.1	3.8E-04	9.9E-04	33.339	1.512	31.594	34.263	29.412	1.459	28.564	31.096
ENSG00000113649	TCERG1	1.1	1.3E-02	2.4E-02	69.184	3.152	65.927	72.219	64.706	0.545	64.253	65.311
ENSG00000113658	SMAD5	1.1	2.4E-05	8.1E-05	40.324	0.579	39.859	40.973	35.247	0.637	34.529	35.745
ENSG00000113712	CSNK1A1	1.1	3.5E-03	7.4E-03	58.779	0.595	58.376	59.463	54.433	0.811	53.936	55.369
ENSG00000113851	CRBN	1.1	8.9E-02	1.3E-01	13.044	0.884	12.125	13.889	12.163	0.606	11.476	12.622
ENSG00000114062	UBE3A	1.1	9.6E-06	3.4E-05	40.694	0.648	40.036	41.330	35.553	1.147	34.604	36.827
ENSG00000114125	RNF7	1.1	2.4E-03	5.3E-03	76.925	4.462	73.014	81.785	68.976	3.552	65.092	72.059
ENSG00000114346	ECT2	1.1	7.8E-04	1.9E-03	108.254	5.343	102.113	111.839	95.538	4.390	90.660	99.171
ENSG00000114388	NPRL2	1.1	1.1E-01	1.6E-01	12.862	0.492	12.490	13.420	11.773	0.982	10.865	12.831
ENSG00000114439	BBX	1.1	7.1E-04	1.7E-03	24.799	0.710	24.029	25.428	22.324	0.719	21.883	23.154
ENSG00000114446	IFT57	1.1	3.1E-03	6.7E-03	30.129	0.297	29.919	30.469	26.722	1.378	25.201	27.887
ENSG00000114544	SLC41A3	1.1	5.9E-02	9.2E-02	10.957	0.586	10.289	11.379	9.993	0.670	9.324	10.664
ENSG00000114738	MAPKAPK3	1.1	9.9E-03	1.9E-02	46.305	0.313	46.034	46.647	42.448	1.232	41.557	43.854
ENSG00000114742	WDR48	1.1	3.4E-02	5.7E-02	37.233	0.666	36.570	37.901	35.028	1.036	33.890	35.918
ENSG00000114850	SSR3	1.1	1.7E-03	3.8E-03	127.431	1.251	126.133	128.630	116.469	2.336	114.082	118.752
ENSG00000114902	SPCS1	1.1	1.3E-01	1.9E-01	38.505	0.844	37.550	39.147	36.696	1.521	34.963	37.808
ENSG00000115042	FAHD2A	1.1	1.2E-01	1.7E-01	16.635	0.493	16.072	16.986	15.529	1.453	14.072	16.978
ENSG00000115084	SLC35F5	1.1	1.1E-01	1.6E-01	10.635	0.357	10.342	11.032	9.892	0.842	9.048	10.732
ENSG00000115091	ACTR3	1.1	9.7E-03	1.8E-02	53.962	1.390	52.854	55.522	50.549	1.480	49.295	52.181
ENSG00000115128	SF3B6	1.1	2.8E-03	6.0E-03	264.272	4.490	259.955	268.918	238.974	5.003	235.026	244.600
ENSG00000115159	GPD2	1.1	5.3E-04	1.3E-03	39.855	0.596	39.327	40.502	35.575	0.153	35.401	35.688
ENSG00000115204	MPV17	1.1	5.4E-02	8.5E-02	14.591	1.468	13.303	16.189	13.354	0.381	12.913	13.576
ENSG00000115268	RPS15	1.1	1.6E-03	3.6E-03	311.351	20.652	287.572	324.804	279.474	17.955	263.013	298.621
ENSG00000115414	FN1	1.1	1.8E-02	3.3E-02	30.716	1.488	29.096	32.022	28.559	0.954	27.587	29.493
ENSG00000115685	PPP1R7	1.1	1.4E-03	3.3E-03	44.855	1.411	43.585	46.374	39.584	1.296	38.090	40.393
ENSG00000115944	COX7A2L	1.1	7.8E-03	1.5E-02	51.997	1.936	49.782	53.368	47.892	1.421	46.395	49.221
ENSG00000116001	TIA1	1.1	2.1E-03	4.7E-03	78.636	4.875	73.378	83.005	70.970	3.745	68.605	75.288
ENSG00000116005	PCYOX1	1.1	2.6E-04	6.9E-04	48.381	0.999	47.682	49.525	42.939	1.748	41.883	44.956
ENSG00000116266	STXBP3	1.1	1.5E-03	3.5E-03	26.409	1.002	25.293	27.232	23.168	1.647	22.183	25.070
ENSG00000116288	PARK7	1.1	1.4E-03	3.3E-03	235.303	6.041	229.741	241.729	215.627	3.835	211.211	218.128
ENSG00000116350	SRSF4	1.1	1.9E-04	5.3E-04	39.143	1.144	37.829	39.919	34.695	1.772	32.703	36.095
ENSG00000116747	TROVE2	1.1	3.9E-02	6.4E-02	18.295	0.931	17.551	19.339	16.914	0.578	16.270	17.388
ENSG00000116962	NID1	1.1	9.7E-03	1.8E-02	35.944	1.474	34.244	36.856	32.838	2.247	30.896	35.300
ENSG00000117036	ETV3	1.1	2.6E-02	4.4E-02	15.922	0.915	14.932	16.737	14.511	0.528	13.907	14.881
ENSG00000117143	UAP1	1.1	3.8E-03	7.9E-03	44.472	1.069	43.562	45.649	39.865	0.679	39.301	40.619
ENSG00000117385	P3H1	1.1	4.2E-03	8.6E-03	19.105	0.625	18.409	19.619	16.856	0.694	16.083	17.425
ENSG00000117448	AKR1A1	1.1	1.4E-04	4.0E-04	131.089	2.760	128.109	133.559	117.483	1.182	116.772	118.847
ENSG00000117450	PRDX1	1.1	3.9E-04	1.0E-03	1800.434	26.625	1783.807	1831.143	1657.250	24.012	1629.583	1672.654
ENSG00000117505	DR1	1.1	3.2E-04	8.4E-04	24.205	0.633	23.824	24.936	21.212	1.472	19.753	22.697
ENSG00000117543	DPH5	1.1	1.3E-02	2.3E-02	58.145	1.515	56.444	59.349	53.463	1.101	52.254	54.407
ENSG00000118181	RPS25	1.1	2.0E-06	8.3E-06	1139.984	34.769	1119.188	1180.123	996.680	34.194	959.216	1026.209
ENSG00000118482	PHF3	1.1	2.6E-02	4.5E-02	48.832	3.388	44.930	51.036	45.699	1.995	43.742	47.729
ENSG00000118515	SGK1	1.1	3.5E-02	5.8E-02	5.807	0.721	5.036	6.465	5.134	0.275	4.925	5.445
ENSG00000118961	LDAH	1.1	2.2E-03	4.9E-03	36.858	1.219	35.892	38.227	32.485	1.405	31.284	34.030
ENSG00000119041	GTF3C3	1.1	5.2E-02	8.2E-02	35.563	0.662	34.801	35.997	33.586	0.367	33.164	33.836
ENSG00000119185	ITGB1BP1	1.1	4.0E-02	6.5E-02	9.001	0.656	8.459	9.731	8.126	0.348	7.902	8.527
ENSG00000119471	HSDL2	1.1	8.3E-03	1.6E-02	23.627	1.715	21.647	24.618	20.852	0.517	20.428	21.428
ENSG00000119514	GALNT12	1.1	7.9E-03	1.5E-02	21.357	1.098	20.261	22.457	18.624	1.275	17.411	19.952
ENSG00000119640	ACYP1	1.1	5.8E-02	9.1E-02	16.816	1.375	15.253	17.841	14.891	0.932	13.999	15.858
ENSG00000119707	RBM25	1.1	5.9E-03	1.2E-02	72.517	5.097	66.717	76.279	67.082	1.171	65.991	68.319
ENSG00000119760	SUPT7L	1.1	1.2E-01	1.7E-01	20.445	0.813	19.624	21.249	19.219	0.385	18.776	19.471
ENSG00000119787	ATL2	1.1	1.5E-02	2.7E-02	63.337	1.375	62.050	64.786	58.699	1.623	57.516	60.549
ENSG00000119862	LGALSL	1.1	9.5E-02	1.4E-01	28.764	0.257	28.506	29.020	26.891	1.426	25.821	28.510
ENSG00000119878	CRIPT	1.1	5.4E-02	8.5E-02	17.449	0.863	16.543	18.261	15.657	0.799	15.163	16.579
ENSG00000119953	SMNDC1	1.1	2.3E-03	5.0E-03	34.925	1.844	32.796	36.018	31.209	2.043	28.924	32.858
ENSG00000120053	GOT1	1.1	2.4E-02	4.2E-02	67.026	1.548	65.814	68.769	62.135	0.552	61.546	62.640
ENSG00000120727	PAIP2	1.1	6.8E-04	1.7E-03	198.166	8.061	193.351	207.472	178.908	3.102	175.915	182.108
ENSG00000120784	ZFP30	1.1	8.6E-02	1.3E-01	3.807	0.286	3.526	4.097	3.414	0.204	3.186	3.576
ENSG00000120798	NR2C1	1.1	2.5E-02	4.3E-02	19.066	1.340	17.730	20.411	17.453	0.575	16.792	17.843
ENSG00000120885	CLU	1.1	2.6E-02	4.5E-02	41.321	0.709	40.825	42.133	38.538	0.847	37.561	39.078
ENSG00000120896	SORBS3	1.1	7.4E-02	1.1E-01	14.668	0.437	14.249	15.122	13.055	0.488	12.624	13.576
ENSG00000120963	ZNF706	1.1	6.8E-02	1.0E-01	14.904	0.275	14.590	15.101	13.857	0.448	13.407	14.304
ENSG00000120992	LYPLA1	1.1	8.1E-04	2.0E-03	111.960	6.513	105.102	118.062	98.983	7.775	90.555	105.877
ENSG00000121057	AKAP1	1.1	3.1E-03	6.6E-03	42.296	0.771	41.579	43.112	38.052	1.076	36.925	39.068
ENSG00000121058	COIL	1.1	2.4E-03	5.1E-03	39.349	0.784	38.517	40.075	34.704	0.951	33.608	35.311
ENSG00000121289	CEP89	1.1	7.0E-03	1.4E-02	8.641	0.279	8.375	8.932	7.630	0.450	7.277	8.138
ENSG00000121440	PDZRN3	1.1	2.0E-02	3.5E-02	8.460	0.329	8.226	8.836	7.401	0.162	7.267	7.581
ENSG00000121481	RNF2	1.1	2.2E-03	4.9E-03	48.331	0.557	47.748	48.859	43.080	2.514	41.431	45.974
ENSG00000122026	RPL21	1.1	7.2E-04	1.8E-03	80.655	3.478	77.717	84.495	71.140	2.031	68.888	72.834
ENSG00000122591	FAM126A	1.1	6.2E-02	9.7E-02	43.039	2.308	40.751	45.366	40.387	2.202	38.541	42.825
ENSG00000122644	ARL4A	1.1	6.6E-02	1.0E-01	25.980	0.224	25.781	26.223	23.872	1.891	22.248	25.949
ENSG00000122729	ACO1	1.1	1.3E-04	3.7E-04	48.971	1.898	46.859	50.532	42.839	1.965	40.603	44.290
ENSG00000122824	NUDT10	1.1	1.4E-01	1.9E-01	45.442	3.783	41.173	48.376	42.795	0.918	41.757	43.499
ENSG00000123066	MED13L	1.1	1.2E-04	3.5E-04	12.295	0.296	12.056	12.627	10.727	0.522	10.332	11.319
ENSG00000123179	EBPL	1.1	8.7E-03	1.7E-02	139.335	2.573	136.998	142.092	127.072	5.588	121.808	132.954
ENSG00000123349	PFDN5	1.1	1.1E-04	3.1E-04	114.795	3.332	112.361	118.593	100.955	2.815	97.713	102.782
ENSG00000123384	LRP1	1.1	7.3E-03	1.4E-02	49.577	1.151	48.588	50.840	45.064	2.748	42.874	48.148
ENSG00000123485	HJURP	1.1	1.3E-03	3.1E-03	70.066	5.879	64.005	75.743	61.354	1.579	60.394	63.177
ENSG00000123836	PFKFB2	1.1	7.0E-02	1.1E-01	8.663	0.416	8.408	9.143	8.004	0.239	7.823	8.274
ENSG00000123975	CKS2	1.1	3.9E-03	8.1E-03	956.981	14.260	942.771	971.290	875.645	36.921	849.409	917.865
ENSG00000123992	DNPEP	1.1	7.6E-03	1.5E-02	29.465	0.291	29.254	29.796	26.567	0.173	26.385	26.729
ENSG00000124155	PIGT	1.1	9.6E-03	1.8E-02	7.793	0.251	7.493	7.930	7.056	0.377	6.626	7.326
ENSG00000124198	ARFGEF2	1.1	9.2E-02	1.4E-01	22.951	0.221	22.756	23.191	21.652	0.917	20.733	22.566
ENSG00000124222	STX16	1.1	8.4E-05	2.5E-04	44.662	1.036	43.891	45.840	38.979	0.834	38.105	39.767
ENSG00000124333	VAMP7	1.1	3.7E-02	6.0E-02	40.637	2.028	38.370	42.278	37.511	1.323	36.558	39.021
ENSG00000124374	PAIP2B	1.1	1.7E-02	3.0E-02	35.019	1.419	33.826	36.588	32.475	0.773	31.989	33.366
ENSG00000124532	MRS2	1.1	1.9E-05	6.4E-05	75.197	1.823	73.792	77.257	65.932	1.211	64.986	67.297
ENSG00000125107	CNOT1	1.1	2.9E-02	4.9E-02	153.811	1.436	152.301	155.158	146.519	3.602	144.103	150.659
ENSG00000125356	NDUFA1	1.1	5.1E-03	1.0E-02	229.050	4.246	225.467	233.740	207.225	3.251	204.973	210.952
ENSG00000125633	CCDC93	1.1	3.4E-02	5.6E-02	18.474	0.767	17.588	18.935	17.105	0.569	16.688	17.753
ENSG00000125755	SYMPK	1.1	1.1E-01	1.6E-01	23.755	0.582	23.306	24.413	22.656	0.727	22.173	23.493
ENSG00000125952	MAX	1.1	4.2E-02	6.9E-02	18.699	0.671	17.954	19.258	17.106	0.711	16.460	17.868
ENSG00000125971	DYNLRB1	1.1	1.3E-02	2.4E-02	52.862	1.784	51.005	54.563	47.989	1.886	45.825	49.279
ENSG00000126267	COX6B1	1.1	7.0E-04	1.7E-03	376.617	8.303	367.300	383.234	337.657	10.102	326.046	344.422
ENSG00000126653	NSRP1	1.1	2.8E-02	4.7E-02	15.107	1.999	12.798	16.279	13.477	0.369	13.052	13.705
ENSG00000126698	DNAJC8	1.1	2.0E-02	3.5E-02	158.132	4.922	154.840	163.790	147.738	2.394	144.985	149.331
ENSG00000126768	TIMM17B	1.1	1.0E-01	1.5E-01	39.757	0.350	39.404	40.105	37.277	1.203	36.367	38.641
ENSG00000126934	MAP2K2	1.1	9.0E-02	1.3E-01	44.945	2.149	42.702	46.987	42.329	3.236	39.191	45.654
ENSG00000127184	COX7C	1.1	1.7E-05	6.0E-05	539.653	12.126	527.535	551.786	479.934	14.343	464.416	492.705
ENSG00000127328	RAB3IP	1.1	6.4E-03	1.3E-02	7.732	0.221	7.478	7.880	6.918	0.308	6.574	7.168
ENSG00000127481	UBR4	1.1	2.9E-03	6.3E-03	51.440	1.747	49.561	53.014	47.621	1.229	46.898	49.041
ENSG00000127511	SIN3B	1.1	6.4E-04	1.6E-03	23.710	0.759	22.874	24.356	20.840	0.985	19.703	21.417
ENSG00000127824	TUBA4A	1.1	1.1E-01	1.6E-01	30.869	0.582	30.223	31.355	29.177	0.796	28.612	30.087
ENSG00000127914	AKAP9	1.1	1.1E-01	1.6E-01	10.359	1.532	8.595	11.356	9.249	0.612	8.554	9.711
ENSG00000127947	PTPN12	1.1	1.9E-02	3.4E-02	97.361	1.375	95.776	98.234	90.546	3.115	87.294	93.503
ENSG00000127993	RBM48	1.1	1.1E-01	1.6E-01	5.596	0.063	5.553	5.668	4.977	0.196	4.824	5.199
ENSG00000128165	ADM2	1.1	7.2E-02	1.1E-01	9.079	0.361	8.663	9.311	8.149	0.755	7.285	8.680
ENSG00000128829	EIF2AK4	1.1	9.6E-04	2.3E-03	71.374	2.075	69.221	73.361	65.430	1.629	63.588	66.681
ENSG00000128944	KNSTRN	1.1	1.5E-02	2.8E-02	58.650	0.687	58.149	59.433	54.035	0.350	53.646	54.323
ENSG00000128989	ARPP19	1.1	6.8E-06	2.5E-05	159.494	4.111	155.598	163.791	139.395	6.328	132.183	144.057
ENSG00000129071	MBD4	1.1	6.9E-02	1.1E-01	39.064	1.966	36.818	40.473	36.539	0.995	35.671	37.626
ENSG00000129083	COPB1	1.1	8.5E-04	2.1E-03	94.334	1.402	93.304	95.930	85.466	1.747	83.874	87.336
ENSG00000129197	RPAIN	1.1	9.6E-04	2.3E-03	24.311	0.260	24.014	24.495	21.621	0.911	20.720	22.542
ENSG00000129810	SGO1	1.1	8.8E-02	1.3E-01	23.554	0.328	23.215	23.870	21.770	1.346	20.875	23.318
ENSG00000130021	PUDP	1.1	7.6E-02	1.1E-01	45.211	1.392	43.654	46.334	42.419	1.933	40.566	44.423
ENSG00000130052	STARD8	1.1	1.2E-01	1.7E-01	4.447	0.258	4.166	4.673	3.957	0.328	3.695	4.326
ENSG00000130147	SH3BP4	1.1	4.7E-03	9.6E-03	32.089	1.442	30.549	33.408	29.142	1.059	27.922	29.818
ENSG00000130175	PRKCSH	1.1	1.4E-03	3.3E-03	95.027	2.086	92.920	97.090	86.054	5.667	79.633	90.354
ENSG00000130203	APOE	1.1	1.5E-04	4.3E-04	556.115	10.637	548.305	568.229	495.600	27.054	466.690	520.305
ENSG00000130294	KIF1A	1.1	7.5E-02	1.1E-01	93.697	2.548	90.858	95.786	89.443	5.424	85.455	95.620
ENSG00000130340	SNX9	1.1	1.3E-03	3.1E-03	36.252	1.417	35.263	37.876	32.265	1.511	30.787	33.806
ENSG00000130638	ATXN10	1.1	1.1E-04	3.3E-04	102.860	3.974	99.348	107.174	91.880	1.678	90.775	93.811
ENSG00000130640	TUBGCP2	1.1	1.1E-02	2.1E-02	24.987	1.396	23.767	26.509	22.857	0.627	22.485	23.580
ENSG00000130684	ZNF337	1.1	5.6E-02	8.8E-02	11.859	0.591	11.307	12.483	10.680	0.466	10.380	11.217
ENSG00000130703	OSBPL2	1.1	2.4E-02	4.1E-02	9.025	0.377	8.639	9.392	8.226	0.240	7.949	8.368
ENSG00000130707	ASS1	1.1	8.9E-04	2.1E-03	44.299	0.870	43.309	44.938	38.687	0.813	38.197	39.634
ENSG00000130741	EIF2S3	1.1	1.8E-04	5.1E-04	367.235	7.044	361.244	374.995	333.796	11.824	322.793	346.298
ENSG00000130770	ATPIF1	1.1	5.2E-02	8.2E-02	112.547	2.623	110.746	115.557	106.171	1.154	104.845	106.945
ENSG00000130779	CLIP1	1.1	6.7E-03	1.3E-02	7.747	0.863	6.764	8.378	6.893	0.071	6.813	6.946
ENSG00000130962	PRRG1	1.1	2.7E-02	4.5E-02	7.847	0.732	7.118	8.582	6.948	0.548	6.355	7.436
ENSG00000131013	PPIL4	1.1	1.4E-01	1.9E-01	45.178	1.625	43.341	46.428	42.707	1.137	41.445	43.650
ENSG00000131037	EPS8L1	1.1	1.0E-01	1.5E-01	9.677	0.855	8.785	10.489	8.843	0.365	8.483	9.213
ENSG00000131051	RBM39	1.1	1.8E-05	6.0E-05	69.811	1.639	68.497	71.647	60.936	2.072	58.752	62.875
ENSG00000131127	ZNF141	1.1	5.6E-02	8.9E-02	17.409	0.174	17.238	17.586	16.097	0.807	15.443	16.999
ENSG00000131174	COX7B	1.1	8.1E-04	2.0E-03	97.665	4.436	93.076	101.929	87.152	2.776	83.964	89.033
ENSG00000131263	RLIM	1.1	7.0E-03	1.4E-02	31.133	1.166	30.307	32.468	28.577	0.674	27.821	29.112
ENSG00000131269	ABCB7	1.1	5.4E-03	1.1E-02	55.510	2.201	53.287	57.689	50.186	1.833	48.071	51.314
ENSG00000131437	KIF3A	1.1	1.2E-02	2.3E-02	10.347	0.502	9.772	10.695	9.116	0.371	8.813	9.530
ENSG00000131459	GFPT2	1.1	8.9E-04	2.1E-03	97.484	0.702	96.675	97.935	88.384	3.990	83.930	91.631
ENSG00000131732	ZCCHC9	1.1	1.0E-02	1.9E-02	23.298	1.124	22.630	24.596	20.660	1.249	19.309	21.773
ENSG00000131747	TOP2A	1.1	3.0E-02	5.1E-02	351.648	2.656	349.749	354.683	332.586	13.205	317.359	340.888
ENSG00000131773	KHDRBS3	1.1	2.1E-02	3.6E-02	8.249	0.279	8.055	8.569	7.406	0.386	7.061	7.822
ENSG00000131778	CHD1L	1.1	1.5E-03	3.4E-03	101.489	1.910	99.623	103.439	92.540	2.497	90.316	95.241
ENSG00000131791	PRKAB2	1.1	1.3E-02	2.4E-02	40.669	0.764	40.078	41.531	37.033	0.890	36.363	38.043
ENSG00000131828	PDHA1	1.1	2.7E-02	4.6E-02	58.172	1.736	56.339	59.790	54.595	0.535	53.978	54.905
ENSG00000131876	SNRPA1	1.1	4.8E-04	1.2E-03	56.949	1.443	55.993	58.608	50.477	1.852	48.811	52.471
ENSG00000132305	IMMT	1.1	8.9E-05	2.6E-04	115.252	0.302	114.968	115.570	102.728	0.922	102.015	103.769
ENSG00000132356	PRKAA1	1.1	8.9E-02	1.3E-01	18.623	1.241	17.286	19.737	17.423	1.337	16.123	18.794
ENSG00000132359	RAP1GAP2	1.1	1.6E-03	3.6E-03	28.655	1.304	27.549	30.093	25.784	0.885	24.814	26.548
ENSG00000132478	UNK	1.1	1.2E-01	1.7E-01	14.508	0.431	14.063	14.923	13.571	0.364	13.355	13.991
ENSG00000132485	ZRANB2	1.1	3.0E-03	6.4E-03	118.609	7.890	109.556	124.018	106.360	7.376	98.974	113.726
ENSG00000132589	FLOT2	1.1	4.3E-03	8.8E-03	96.396	2.341	94.638	99.053	88.527	2.181	86.185	90.499
ENSG00000132670	PTPRA	1.1	6.4E-04	1.6E-03	51.522	0.845	51.001	52.497	45.954	2.012	43.666	47.448
ENSG00000132716	DCAF8	1.1	1.2E-01	1.7E-01	20.621	0.337	20.409	21.009	19.557	1.070	18.620	20.722
ENSG00000132781	MUTYH	1.1	1.3E-01	1.9E-01	23.161	0.918	22.297	24.126	21.758	0.747	20.976	22.465
ENSG00000132849	PATJ	1.1	4.1E-02	6.7E-02	20.666	0.689	19.887	21.196	19.280	0.352	19.039	19.684
ENSG00000132872	SYT4	1.1	8.3E-02	1.2E-01	8.289	0.631	7.792	8.999	7.406	0.205	7.178	7.573
ENSG00000133134	BEX2	1.1	1.3E-03	3.0E-03	120.680	4.285	116.090	124.569	106.187	4.524	101.359	110.329
ENSG00000133393	FOPNL	1.1	7.3E-04	1.8E-03	78.068	3.339	74.676	81.352	69.174	0.997	68.588	70.326
ENSG00000133422	MORC2	1.1	3.6E-03	7.5E-03	48.230	0.759	47.479	48.997	43.634	1.931	41.987	45.759
ENSG00000133424	LARGE1	1.1	1.4E-03	3.2E-03	18.208	0.383	17.909	18.640	16.173	0.956	15.162	17.063
ENSG00000133678	TMEM254	1.1	7.8E-02	1.2E-01	25.198	1.412	23.784	26.609	23.310	0.847	22.333	23.839
ENSG00000133706	LARS	1.1	2.2E-03	4.9E-03	192.578	3.253	190.231	196.292	178.996	5.259	174.224	184.634
ENSG00000133773	CCDC59	1.1	1.0E-01	1.5E-01	27.895	2.141	26.513	30.361	25.999	1.346	25.045	27.539
ENSG00000133961	NUMB	1.1	4.9E-02	7.9E-02	16.553	0.662	16.161	17.318	15.458	0.541	14.981	16.045
ENSG00000134030	CTIF	1.1	5.5E-02	8.7E-02	5.143	0.140	5.012	5.290	4.593	0.209	4.380	4.798
ENSG00000134058	CDK7	1.1	4.6E-04	1.2E-03	77.700	1.513	76.037	78.995	68.213	1.477	67.306	69.918
ENSG00000134146	DPH6	1.1	7.7E-02	1.2E-01	7.336	0.081	7.242	7.383	6.675	0.954	5.747	7.653
ENSG00000134207	SYT6	1.1	1.1E-01	1.6E-01	21.345	1.525	20.163	23.066	19.192	0.421	18.782	19.623
ENSG00000134297	PLEKHA8P1	1.1	3.3E-02	5.6E-02	25.299	0.725	24.505	25.925	22.501	1.880	20.983	24.604
ENSG00000134352	IL6ST	1.1	1.8E-02	3.3E-02	5.850	0.273	5.546	6.075	5.124	0.455	4.659	5.570
ENSG00000134453	RBM17	1.1	2.3E-02	4.0E-02	55.849	2.246	54.223	58.412	51.991	1.612	50.354	53.576
ENSG00000134597	RBMX2	1.1	9.5E-02	1.4E-01	24.200	1.636	22.318	25.281	22.084	0.990	20.966	22.851
ENSG00000134602	STK26	1.1	2.4E-04	6.4E-04	257.609	6.043	251.035	262.923	230.674	14.568	218.692	246.891
ENSG00000134690	CDCA8	1.1	1.2E-01	1.7E-01	74.147	1.227	72.735	74.955	70.604	2.271	68.119	72.572
ENSG00000134709	HOOK1	1.1	3.8E-02	6.2E-02	44.429	3.429	40.746	47.529	41.404	2.813	39.246	44.585
ENSG00000134717	BTF3L4	1.1	1.7E-02	3.1E-02	65.127	2.211	62.867	67.284	60.837	1.527	59.339	62.390
ENSG00000134901	KDELC1	1.1	1.4E-01	1.9E-01	22.796	0.762	21.979	23.488	21.044	1.988	18.737	22.214
ENSG00000135018	UBQLN1	1.1	4.8E-04	1.2E-03	100.692	0.680	100.215	101.471	91.429	2.828	88.232	93.603
ENSG00000135127	BICDL1	1.1	5.7E-02	9.0E-02	12.951	0.674	12.243	13.586	11.848	0.621	11.131	12.209
ENSG00000135272	MDFIC	1.1	6.7E-03	1.3E-02	15.578	0.392	15.206	15.987	13.733	0.331	13.449	14.096
ENSG00000135341	MAP3K7	1.1	6.1E-02	9.4E-02	51.018	1.584	50.041	52.845	48.349	1.811	46.624	50.235
ENSG00000135372	NAT10	1.1	6.5E-02	1.0E-01	79.554	2.868	76.821	82.540	75.540	3.135	73.220	79.107
ENSG00000135452	TSPAN31	1.1	1.2E-01	1.8E-01	9.350	0.357	8.943	9.611	8.667	0.291	8.407	8.981
ENSG00000135506	OS9	1.1	1.3E-01	1.9E-01	43.292	0.345	43.077	43.681	41.160	2.985	37.726	43.133
ENSG00000135525	MAP7	1.1	8.9E-02	1.3E-01	51.418	1.775	50.242	53.460	48.935	0.996	48.091	50.033
ENSG00000135535	CD164	1.1	4.4E-03	9.1E-03	73.694	2.084	71.602	75.769	67.572	4.337	62.601	70.579
ENSG00000135537	AFG1L	1.1	1.3E-01	1.8E-01	2.525	0.210	2.356	2.760	2.241	0.122	2.146	2.379
ENSG00000135778	NTPCR	1.1	1.3E-02	2.4E-02	7.969	0.519	7.372	8.314	7.214	0.261	6.970	7.489
ENSG00000135821	GLUL	1.1	2.5E-03	5.5E-03	110.772	2.648	108.567	113.709	102.768	0.372	102.425	103.162
ENSG00000135837	CEP350	1.1	3.4E-02	5.7E-02	18.230	0.909	17.474	19.239	17.055	0.799	16.133	17.543
ENSG00000135870	RC3H1	1.1	4.8E-02	7.7E-02	8.123	0.480	7.739	8.660	7.440	0.476	6.991	7.938
ENSG00000135926	TMBIM1	1.1	9.5E-02	1.4E-01	14.343	0.559	13.776	14.894	13.312	1.102	12.193	14.396
ENSG00000135940	COX5B	1.1	4.2E-03	8.6E-03	218.026	7.524	211.657	226.328	199.229	10.540	187.368	207.523
ENSG00000135968	GCC2	1.1	1.1E-02	2.1E-02	21.180	2.230	18.605	22.473	18.928	0.737	18.308	19.743
ENSG00000136044	APPL2	1.1	2.5E-02	4.3E-02	17.958	0.387	17.531	18.287	16.373	1.053	15.157	16.984
ENSG00000136141	LRCH1	1.1	1.6E-02	2.9E-02	6.755	0.212	6.569	6.986	5.903	0.480	5.353	6.234
ENSG00000136146	MED4	1.1	1.8E-03	4.1E-03	83.256	2.409	81.435	85.988	73.909	4.134	69.164	76.736
ENSG00000136436	CALCOCO2	1.1	6.2E-02	9.6E-02	10.148	0.197	9.934	10.323	9.362	0.314	9.136	9.720
ENSG00000136485	DCAF7	1.1	8.3E-03	1.6E-02	73.110	1.532	71.623	74.683	68.112	1.672	66.201	69.302
ENSG00000136521	NDUFB5	1.1	3.4E-02	5.7E-02	33.595	0.207	33.362	33.758	31.383	0.556	30.772	31.859
ENSG00000136560	TANK	1.1	2.4E-02	4.1E-02	10.891	0.231	10.651	11.112	9.704	0.624	9.068	10.316
ENSG00000136628	EPRS	1.1	2.7E-03	5.9E-03	243.644	4.953	238.013	247.326	226.331	3.116	223.834	229.824
ENSG00000136738	STAM	1.1	1.4E-01	1.9E-01	31.653	1.643	29.957	33.247	29.819	0.786	29.365	30.726
ENSG00000136754	ABI1	1.1	2.9E-02	4.8E-02	35.338	1.320	34.181	36.776	32.614	0.722	31.859	33.298
ENSG00000136810	TXN	1.1	9.4E-06	3.4E-05	587.408	16.133	573.803	605.231	512.867	10.549	503.955	524.514
ENSG00000136986	DERL1	1.1	2.2E-02	3.8E-02	25.954	1.010	25.024	27.028	24.055	0.681	23.290	24.592
ENSG00000137038	TMEM261	1.1	1.1E-03	2.5E-03	65.582	1.963	63.344	67.013	57.753	5.184	53.547	63.545
ENSG00000137054	POLR1E	1.1	1.0E-02	2.0E-02	43.777	0.716	42.962	44.302	39.548	1.681	37.758	41.093
ENSG00000137154	RPS6	1.1	1.0E-06	4.5E-06	1653.847	27.286	1626.296	1680.859	1443.857	37.757	1406.534	1482.039
ENSG00000137161	CNPY3	1.1	5.0E-02	7.9E-02	80.655	1.101	79.897	81.918	74.674	4.983	69.028	78.456
ENSG00000137185	ZSCAN9	1.1	2.6E-02	4.4E-02	13.287	0.473	12.741	13.581	11.706	0.083	11.650	11.801
ENSG00000137216	TMEM63B	1.1	1.0E-01	1.5E-01	20.484	0.847	19.515	21.079	19.116	1.500	17.741	20.716
ENSG00000137288	UQCC2	1.1	1.9E-03	4.3E-03	29.919	0.709	29.362	30.671	26.560	0.713	25.739	27.017
ENSG00000137309	HMGA1	1.1	6.1E-02	9.6E-02	2097.577	30.781	2063.166	2122.490	1999.659	130.292	1854.286	2105.899
ENSG00000137343	ATAT1	1.1	1.2E-01	1.7E-01	6.766	0.643	6.237	7.482	6.104	0.168	5.914	6.231
ENSG00000137409	MTCH1	1.1	9.0E-02	1.3E-01	156.312	1.837	154.219	157.657	148.977	9.062	140.761	158.697
ENSG00000137411	VARS2	1.1	6.5E-02	1.0E-01	10.832	0.571	10.469	11.490	10.040	0.489	9.706	10.601
ENSG00000137413	TAF8	1.1	2.9E-02	4.8E-02	8.586	0.361	8.200	8.915	7.780	0.355	7.404	8.108
ENSG00000137414	FAM8A1	1.1	3.0E-02	5.1E-02	18.176	1.492	16.468	19.227	16.400	0.386	16.173	16.845
ENSG00000137504	CREB3L1	1.1	1.7E-02	3.1E-02	55.098	4.349	50.170	58.397	50.918	2.349	48.668	53.355
ENSG00000137509	PRCP	1.1	2.6E-03	5.6E-03	21.580	0.434	21.143	22.010	19.580	0.937	19.016	20.662
ENSG00000137692	DCUN1D5	1.1	5.3E-03	1.1E-02	205.536	1.263	204.080	206.337	189.926	4.147	186.443	194.514
ENSG00000137764	MAP2K5	1.1	8.4E-02	1.3E-01	7.084	0.648	6.387	7.670	6.333	0.585	5.813	6.966
ENSG00000137817	PARP6	1.1	3.5E-02	5.7E-02	7.372	0.705	6.618	8.015	6.619	0.230	6.430	6.875
ENSG00000137831	UACA	1.1	7.1E-02	1.1E-01	15.318	0.344	14.930	15.583	14.385	1.055	13.623	15.588
ENSG00000137944	KYAT3	1.1	3.1E-02	5.3E-02	81.921	1.052	80.722	82.685	76.111	2.026	74.390	78.344
ENSG00000138036	DYNC2LI1	1.1	4.1E-02	6.7E-02	7.136	0.227	6.952	7.390	6.305	0.094	6.241	6.413
ENSG00000138101	DTNB	1.1	8.9E-02	1.3E-01	10.901	0.460	10.566	11.426	10.036	0.761	9.168	10.591
ENSG00000138138	ATAD1	1.1	1.1E-01	1.7E-01	45.522	1.600	43.791	46.947	43.257	2.150	41.108	45.407
ENSG00000138382	METTL5	1.1	9.0E-02	1.3E-01	59.881	1.591	58.165	61.309	56.366	2.230	53.926	58.298
ENSG00000138433	CIR1	1.1	1.2E-01	1.7E-01	22.684	1.565	21.010	24.111	20.879	2.089	18.475	22.248
ENSG00000138468	SENP7	1.1	4.1E-02	6.7E-02	7.652	0.825	7.136	8.603	6.747	0.850	5.781	7.382
ENSG00000138594	TMOD3	1.1	4.2E-04	1.1E-03	25.998	1.186	24.641	26.841	22.940	1.270	21.474	23.723
ENSG00000138617	PARP16	1.1	7.6E-02	1.2E-01	6.939	0.046	6.889	6.980	6.154	0.514	5.565	6.509
ENSG00000138623	SEMA7A	1.1	7.3E-02	1.1E-01	13.078	0.409	12.609	13.364	11.968	0.170	11.772	12.072
ENSG00000138758	SEPT11	1.1	7.6E-03	1.5E-02	59.458	0.448	58.941	59.740	55.287	2.253	52.981	57.482
ENSG00000138768	USO1	1.1	2.4E-04	6.5E-04	251.449	10.120	241.969	262.107	227.703	8.181	219.553	235.914
ENSG00000138796	HADH	1.1	1.2E-02	2.2E-02	15.216	0.900	14.400	16.181	13.822	0.453	13.347	14.250
ENSG00000138802	SEC24B	1.1	6.8E-03	1.3E-02	32.917	1.725	31.733	34.896	29.638	0.841	28.950	30.575
ENSG00000138814	PPP3CA	1.1	8.9E-05	2.6E-04	54.670	2.001	52.496	56.433	47.914	0.399	47.477	48.260
ENSG00000139132	FGD4	1.1	7.8E-03	1.5E-02	14.144	0.391	13.694	14.386	12.844	0.702	12.111	13.510
ENSG00000139146	FAM60A	1.1	2.2E-03	4.8E-03	303.879	2.939	300.937	306.814	280.525	7.320	272.430	286.679
ENSG00000139163	ETNK1	1.1	2.9E-02	4.9E-02	33.923	2.900	30.583	35.811	30.970	2.927	28.784	34.295
ENSG00000139436	GIT2	1.1	7.8E-03	1.5E-02	5.317	0.107	5.202	5.414	4.699	0.160	4.518	4.823
ENSG00000139644	TMBIM6	1.1	6.2E-04	1.6E-03	303.093	9.469	295.249	313.611	279.329	3.771	274.996	281.867
ENSG00000139684	ESD	1.1	6.6E-02	1.0E-01	82.973	1.415	81.399	84.141	78.566	3.688	74.496	81.685
ENSG00000139726	DENR	1.1	7.5E-04	1.8E-03	139.812	1.693	137.947	141.253	127.628	2.586	124.732	129.708
ENSG00000139746	RBM26	1.1	1.8E-02	3.1E-02	41.115	0.856	40.196	41.889	38.207	2.426	36.627	41.001
ENSG00000140299	BNIP2	1.1	1.4E-01	2.0E-01	28.610	1.102	27.503	29.708	27.128	2.063	24.778	28.642
ENSG00000140319	SRP14	1.1	6.4E-04	1.6E-03	347.845	5.702	341.262	351.192	315.198	8.819	305.302	322.223
ENSG00000140367	UBE2Q2	1.1	2.0E-02	3.5E-02	28.027	1.016	27.368	29.198	25.531	0.844	24.568	26.146
ENSG00000140374	ETFA	1.1	1.2E-01	1.7E-01	40.999	0.746	40.302	41.786	39.086	1.770	37.317	40.856
ENSG00000140474	ULK3	1.1	7.6E-02	1.1E-01	19.144	0.991	18.466	20.281	17.626	0.401	17.163	17.874
ENSG00000140691	ARMC5	1.1	1.0E-01	1.5E-01	3.752	0.391	3.513	4.203	3.289	0.213	3.073	3.499
ENSG00000140743	CDR2	1.1	7.1E-02	1.1E-01	31.270	1.246	30.338	32.686	29.189	1.653	27.327	30.483
ENSG00000140948	ZCCHC14	1.1	6.1E-03	1.2E-02	19.432	0.635	18.747	20.000	17.369	1.141	16.199	18.480
ENSG00000141068	KSR1	1.1	3.3E-02	5.5E-02	9.805	0.229	9.548	9.988	8.966	0.074	8.902	9.047
ENSG00000141295	SCRN2	1.1	1.3E-01	1.9E-01	24.078	1.295	22.802	25.391	22.491	0.550	21.988	23.078
ENSG00000141385	AFG3L2	1.1	1.1E-02	2.0E-02	62.353	0.874	61.348	62.926	57.783	1.341	56.262	58.794
ENSG00000141447	OSBPL1A	1.1	4.2E-03	8.6E-03	33.611	0.279	33.353	33.907	30.753	0.818	30.023	31.638
ENSG00000141506	PIK3R5	1.1	1.3E-01	1.8E-01	2.974	0.401	2.703	3.434	2.640	0.173	2.489	2.829
ENSG00000141568	FOXK2	1.1	1.2E-02	2.3E-02	22.176	0.715	21.589	22.972	20.466	0.733	19.630	20.995
ENSG00000141682	PMAIP1	1.1	1.1E-03	2.6E-03	188.060	7.859	179.003	193.082	168.534	10.798	159.852	180.626
ENSG00000141994	DUS3L	1.1	6.3E-03	1.2E-02	20.721	1.402	19.364	22.165	18.110	0.632	17.413	18.645
ENSG00000142082	SIRT3	1.1	1.4E-01	1.9E-01	7.773	0.117	7.645	7.872	7.139	0.569	6.713	7.785
ENSG00000142089	IFITM3	1.1	7.5E-02	1.1E-01	138.538	4.833	135.246	144.086	130.230	8.884	120.339	137.534
ENSG00000142252	GEMIN7	1.1	6.5E-02	1.0E-01	36.647	4.497	31.853	40.771	33.172	1.720	31.759	35.086
ENSG00000142599	RERE	1.1	4.0E-02	6.5E-02	27.692	1.004	26.534	28.321	25.932	1.388	24.731	27.467
ENSG00000142856	ITGB3BP	1.1	2.2E-03	4.9E-03	24.369	0.843	23.397	24.897	21.629	1.125	20.346	22.446
ENSG00000143155	TIPRL	1.1	1.2E-03	2.8E-03	48.999	1.559	47.551	50.649	43.416	1.977	41.163	44.856
ENSG00000143158	MPC2	1.1	1.2E-01	1.7E-01	36.064	1.146	34.822	37.082	33.823	1.472	32.274	35.203
ENSG00000143190	POU2F1	1.1	9.6E-04	2.3E-03	31.558	1.804	29.727	33.333	28.615	0.331	28.332	28.979
ENSG00000143198	MGST3	1.1	1.1E-02	2.1E-02	19.759	0.297	19.495	20.081	18.021	0.825	17.070	18.553
ENSG00000143324	XPR1	1.1	7.7E-03	1.5E-02	37.103	1.047	36.199	38.251	34.194	0.341	33.936	34.581
ENSG00000143353	LYPLAL1	1.1	6.2E-02	9.7E-02	15.594	0.619	14.886	16.028	13.930	0.619	13.423	14.620
ENSG00000143390	RFX5	1.1	1.8E-02	3.3E-02	43.765	2.001	41.869	45.856	40.299	2.258	38.744	42.889
ENSG00000143418	CERS2	1.1	5.5E-02	8.7E-02	90.179	4.430	87.412	95.288	85.516	0.844	84.884	86.474
ENSG00000143437	ARNT	1.1	3.0E-02	5.1E-02	20.199	0.263	20.032	20.502	18.660	0.740	17.973	19.444
ENSG00000143553	SNAPIN	1.1	1.3E-01	1.8E-01	30.815	1.101	29.990	32.064	28.336	1.751	26.780	30.231
ENSG00000143575	HAX1	1.1	8.0E-04	1.9E-03	88.627	4.749	83.191	91.968	77.883	2.556	74.933	79.430
ENSG00000143702	CEP170	1.1	4.5E-02	7.3E-02	36.261	0.777	35.370	36.796	34.269	1.656	32.363	35.353
ENSG00000143742	SRP9	1.1	9.8E-04	2.3E-03	787.069	30.641	766.749	822.313	714.118	46.092	670.185	762.101
ENSG00000143772	ITPKB	1.1	2.2E-02	3.9E-02	5.248	0.121	5.133	5.375	4.579	0.087	4.507	4.676
ENSG00000143811	PYCR2	1.1	3.9E-03	8.1E-03	42.684	1.388	41.549	44.231	38.193	2.017	36.531	40.437
ENSG00000143870	PDIA6	1.1	1.9E-02	3.4E-02	422.431	6.155	415.595	427.532	400.879	5.615	396.443	407.191
ENSG00000143933	CALM2	1.1	9.4E-05	2.8E-04	260.738	2.337	258.605	263.236	228.759	17.432	209.942	244.357
ENSG00000143977	SNRPG	1.1	3.7E-02	6.0E-02	151.984	2.362	149.593	154.316	142.470	6.925	134.501	147.029
ENSG00000144026	ZNF514	1.1	2.5E-02	4.3E-02	7.303	0.343	6.908	7.521	6.515	0.045	6.479	6.565
ENSG00000144029	MRPS5	1.1	2.0E-02	3.6E-02	23.361	0.330	22.982	23.578	21.572	0.853	20.811	22.494
ENSG00000144036	EXOC6B	1.1	2.6E-04	7.1E-04	24.290	1.439	22.629	25.183	21.283	0.494	20.713	21.591
ENSG00000144158		1.1	2.3E-02	4.0E-02	168.864	14.920	151.636	177.501	153.718	6.039	149.165	160.568
ENSG00000144224	UBXN4	1.1	4.1E-05	1.3E-04	108.559	3.121	105.405	111.647	96.333	1.732	94.669	98.126
ENSG00000144567	RETREG2	1.1	7.1E-02	1.1E-01	19.928	0.731	19.370	20.755	18.500	1.388	16.967	19.703
ENSG00000144815	NXPE3	1.1	6.1E-02	9.5E-02	19.276	0.672	18.772	20.038	18.075	0.522	17.513	18.545
ENSG00000144895	EIF2A	1.1	4.3E-02	7.0E-02	83.240	1.585	81.612	84.778	78.412	3.501	74.370	80.513
ENSG00000145012	LPP	1.1	3.2E-03	6.8E-03	10.965	0.587	10.620	11.643	9.743	0.159	9.579	9.896
ENSG00000145354	CISD2	1.1	9.5E-02	1.4E-01	48.644	4.139	44.799	53.024	45.518	1.828	43.855	47.475
ENSG00000145386	CCNA2	1.1	3.1E-03	6.5E-03	221.304	7.826	216.097	230.304	201.778	12.047	189.192	213.200
ENSG00000145687	SSBP2	1.1	3.4E-02	5.7E-02	5.993	0.398	5.683	6.443	5.398	0.035	5.358	5.422
ENSG00000145741	BTF3	1.1	2.5E-04	6.7E-04	637.310	6.139	632.078	644.068	579.387	9.455	572.082	590.067
ENSG00000145782	ATG12	1.1	2.9E-03	6.2E-03	20.444	1.393	19.328	22.005	18.181	0.873	17.237	18.958
ENSG00000145868	FBXO38	1.1	2.0E-03	4.5E-03	20.873	1.040	19.889	21.961	18.625	0.431	18.261	19.101
ENSG00000146066	HIGD2A	1.1	7.5E-03	1.5E-02	161.601	10.864	154.464	174.105	143.420	10.603	132.369	153.510
ENSG00000146067	FAM193B	1.1	9.1E-02	1.3E-01	13.324	0.291	13.004	13.572	12.410	0.425	12.147	12.900
ENSG00000146242	TPBG	1.1	7.1E-03	1.4E-02	14.061	0.703	13.257	14.561	12.578	0.548	12.039	13.135
ENSG00000146247	PHIP	1.1	1.7E-02	3.0E-02	47.312	2.271	44.690	48.669	43.833	2.857	41.510	47.023
ENSG00000146416	AIG1	1.1	1.8E-02	3.3E-02	16.648	0.696	16.095	17.430	15.074	0.531	14.721	15.685
ENSG00000146556	WASH2P	1.1	8.9E-02	1.3E-01	6.149	0.225	5.906	6.350	5.378	0.447	4.980	5.862
ENSG00000146701	MDH2	1.1	1.7E-02	3.1E-02	155.822	4.612	150.817	159.902	145.525	6.710	139.074	152.467
ENSG00000146733	PSPH	1.1	8.1E-03	1.6E-02	66.823	1.676	65.380	68.662	60.889	2.612	58.702	63.781
ENSG00000146833	TRIM4	1.1	7.1E-02	1.1E-01	10.097	0.357	9.724	10.437	9.154	0.477	8.754	9.682
ENSG00000147044	CASK	1.1	5.8E-04	1.5E-03	26.526	0.449	26.023	26.885	23.879	0.246	23.615	24.102
ENSG00000147123	NDUFB11	1.1	1.0E-01	1.5E-01	135.215	5.233	129.553	139.872	128.303	4.148	125.897	133.093
ENSG00000147231	CXorf57	1.1	5.7E-02	8.9E-02	6.029	0.475	5.485	6.361	5.307	0.452	4.861	5.765
ENSG00000147257	GPC3	1.1	1.7E-03	3.8E-03	71.968	2.181	70.472	74.470	63.386	2.541	61.768	66.315

ENSG00000147316	MCPH1	1.1	3.6E-02	5.9E-02	10.167	0.158	10.011	10.328	9.361	0.107	9.246	9.458
ENSG00000147471	PLPBP	1.1	7.4E-02	1.1E-01	35.558	1.184	34.424	36.786	33.274	0.885	32.400	34.170
ENSG00000147475	ERLIN2	1.1	5.5E-02	8.7E-02	26.304	0.852	25.435	27.138	24.714	1.066	23.486	25.403
ENSG00000147854	UHRF2	1.1	4.4E-03	9.1E-03	19.646	0.133	19.498	19.753	17.877	0.678	17.095	18.299
ENSG00000147894	C9orf72	1.1	1.8E-02	3.2E-02	19.580	0.469	19.045	19.922	17.649	1.166	16.376	18.665
ENSG00000147905	ZCCHC7	1.1	3.6E-02	6.0E-02	27.697	0.523	27.141	28.180	25.414	0.833	24.486	26.098
ENSG00000148468	FAM171A1	1.1	8.6E-02	1.3E-01	44.571	0.245	44.328	44.817	42.160	0.695	41.571	42.926
ENSG00000148484	RSU1	1.1	1.9E-04	5.2E-04	45.586	1.131	44.286	46.344	39.843	1.456	38.163	40.759
ENSG00000148498	PARD3	1.1	9.9E-03	1.9E-02	42.269	1.167	40.923	42.966	38.904	1.481	37.405	40.366
ENSG00000148672	GLUD1	1.1	3.6E-02	6.0E-02	108.108	1.956	106.575	110.312	101.910	2.487	99.123	103.903
ENSG00000148690	FRA10AC1	1.1	2.4E-02	4.1E-02	9.597	0.067	9.535	9.669	8.574	0.509	8.271	9.162
ENSG00000149131	SERPING1	1.1	8.0E-02	1.2E-01	26.538	1.671	24.941	28.274	24.645	0.758	24.192	25.520
ENSG00000149136	SSRP1	1.1	2.8E-03	5.9E-03	190.766	1.960	188.758	192.674	175.944	7.754	169.026	184.325
ENSG00000149231	CCDC82	1.1	1.1E-02	2.1E-02	11.261	0.880	10.275	11.967	9.892	0.543	9.380	10.461
ENSG00000149547	EI24	1.1	4.4E-02	7.2E-02	156.306	5.790	149.910	161.189	147.967	7.032	139.865	152.478
ENSG00000149806	FAU	1.1	1.5E-02	2.7E-02	302.761	9.026	294.117	312.126	283.719	4.715	279.624	288.873
ENSG00000149823	VPS51	1.1	3.9E-02	6.3E-02	41.425	1.667	40.429	43.349	38.396	2.275	35.881	40.310
ENSG00000149929	HIRIP3	1.1	3.8E-02	6.3E-02	14.351	0.302	14.174	14.700	12.940	0.332	12.579	13.233
ENSG00000150403	TMCO3	1.1	4.0E-04	1.0E-03	17.479	0.167	17.344	17.666	15.439	0.272	15.226	15.745
ENSG00000150593	PDCD4	1.1	1.8E-02	3.2E-02	24.269	0.537	23.811	24.860	21.848	0.360	21.622	22.262
ENSG00000150760	DOCK1	1.1	1.1E-02	2.0E-02	27.743	1.295	26.247	28.493	25.417	1.163	24.465	26.713
ENSG00000151176	PLBD2	1.1	3.0E-02	5.0E-02	23.625	0.961	22.565	24.440	21.647	1.678	20.449	23.565
ENSG00000151239	TWF1	1.1	4.9E-03	1.0E-02	38.246	0.893	37.331	39.115	33.424	2.723	31.403	36.521
ENSG00000151292	CSNK1G3	1.1	4.2E-04	1.1E-03	42.706	1.127	41.890	43.993	37.493	2.448	35.921	40.314
ENSG00000151327	FAM177A1	1.1	3.8E-03	7.9E-03	11.246	0.581	10.778	11.896	9.836	0.340	9.446	10.069
ENSG00000151353	TMEM18	1.1	2.7E-02	4.6E-02	13.843	0.369	13.419	14.095	12.418	0.329	12.185	12.794
ENSG00000151718	WWC2	1.1	5.8E-04	1.5E-03	71.626	1.691	69.673	72.609	64.943	1.679	63.005	65.951
ENSG00000151883	PARP8	1.1	5.6E-02	8.8E-02	9.684	0.174	9.524	9.869	8.926	0.184	8.733	9.099
ENSG00000151923	TIAL1	1.1	1.5E-04	4.3E-04	44.070	1.766	42.118	45.556	38.785	1.542	37.692	40.549
ENSG00000151929	BAG3	1.1	4.0E-02	6.5E-02	20.369	0.537	19.795	20.860	18.339	1.314	16.984	19.608
ENSG00000152082	MZT2B	1.1	9.6E-02	1.4E-01	55.694	5.522	52.292	62.066	51.021	5.040	45.216	54.282
ENSG00000152223	EPG5	1.1	3.1E-02	5.3E-02	9.093	0.153	8.938	9.243	8.361	0.367	7.957	8.675
ENSG00000152234	ATP5A1	1.1	3.9E-02	6.4E-02	195.868	7.242	190.958	204.185	186.624	3.241	184.173	190.299
ENSG00000152348	ATG10	1.1	9.5E-02	1.4E-01	10.414	1.142	9.107	11.217	9.446	0.471	9.037	9.960
ENSG00000152492	CCDC50	1.1	6.0E-02	9.4E-02	17.513	1.080	16.549	18.680	16.349	0.620	15.636	16.755
ENSG00000152503	TRIM36	1.1	2.6E-02	4.5E-02	8.371	0.067	8.311	8.443	7.384	0.454	7.084	7.906
ENSG00000152661	GJA1	1.1	7.0E-05	2.1E-04	931.498	35.840	897.629	969.028	828.611	53.483	766.920	861.932
ENSG00000152700	SAR1B	1.1	7.2E-02	1.1E-01	8.264	0.366	7.846	8.521	7.607	0.203	7.387	7.785
ENSG00000152818	UTRN	1.1	6.5E-04	1.6E-03	49.623	3.296	46.091	52.617	45.029	0.205	44.908	45.266
ENSG00000152939	MARVELD2	1.1	3.6E-02	5.9E-02	18.037	0.611	17.332	18.403	16.457	0.948	15.372	17.122
ENSG00000153015	CWC27	1.1	2.1E-02	3.7E-02	70.859	1.792	68.962	72.523	66.176	1.844	64.094	67.603
ENSG00000153207	AHCTF1	1.1	2.7E-02	4.6E-02	33.633	1.419	32.030	34.724	31.395	0.548	30.801	31.880
ENSG00000153391	INO80C	1.1	1.1E-01	1.5E-01	9.554	0.671	9.032	10.311	8.723	0.782	8.021	9.566
ENSG00000153443	UBALD1	1.1	3.1E-02	5.3E-02	15.247	0.284	15.013	15.564	13.510	1.248	12.159	14.620
ENSG00000153815	CMIP	1.1	1.1E-03	2.6E-03	13.829	0.405	13.417	14.227	12.098	0.712	11.404	12.826
ENSG00000154134	ROBO3	1.1	1.4E-01	2.0E-01	2.330	0.388	2.044	2.772	2.042	0.101	1.929	2.121
ENSG00000154144	TBRG1	1.1	2.1E-02	3.6E-02	14.485	1.294	13.020	15.470	13.099	0.367	12.819	13.514
ENSG00000154380	ENAH	1.1	4.1E-03	8.5E-03	91.396	4.396	86.654	95.336	84.492	4.474	80.196	89.125
ENSG00000154781	CCDC174	1.1	2.9E-02	5.0E-02	14.508	0.730	13.716	15.154	12.925	0.964	12.098	13.985
ENSG00000154920	EME1	1.1	3.8E-02	6.2E-02	13.868	1.169	12.558	14.806	12.326	0.784	11.840	13.231
ENSG00000155158	TTC39B	1.1	1.3E-02	2.4E-02	6.166	0.189	5.952	6.309	5.417	0.547	5.026	6.042
ENSG00000155252	PI4K2A	1.1	1.3E-02	2.4E-02	19.427	1.369	17.922	20.597	17.232	1.109	16.406	18.493
ENSG00000155561	NUP205	1.1	3.4E-05	1.1E-04	172.560	4.381	167.530	175.539	154.880	3.016	153.114	158.363
ENSG00000155621	C9orf85	1.1	4.2E-02	6.8E-02	6.705	0.014	6.691	6.719	5.872	0.292	5.569	6.152
ENSG00000155959	VBP1	1.1	3.6E-02	5.9E-02	117.424	3.508	114.701	121.383	109.850	5.011	104.400	114.258
ENSG00000156017	CARNMT1	1.1	8.9E-02	1.3E-01	37.160	3.107	33.682	39.664	34.944	1.438	33.366	36.180
ENSG00000156026	MCU	1.1	5.5E-04	1.4E-03	51.466	0.964	50.382	52.226	45.889	1.928	44.591	48.105
ENSG00000156273	BACH1	1.1	1.4E-02	2.5E-02	27.103	0.687	26.692	27.896	24.886	0.166	24.708	25.021
ENSG00000156298	TSPAN7	1.1	1.2E-01	1.8E-01	6.463	0.500	5.950	6.948	5.763	0.379	5.481	6.193
ENSG00000156639	ZFAND3	1.1	3.2E-03	6.8E-03	40.824	0.807	39.915	41.455	36.642	1.563	34.922	37.976
ENSG00000156875	MFSD14A	1.1	1.1E-01	1.6E-01	8.623	1.039	7.923	9.817	7.658	0.524	7.118	8.164
ENSG00000156990	RPUSD3	1.1	3.6E-02	6.0E-02	29.133	1.146	27.810	29.805	27.006	0.985	25.911	27.818
ENSG00000157110	RBPMS	1.1	9.0E-04	2.2E-03	57.124	0.917	56.136	57.948	51.552	2.631	48.580	53.585
ENSG00000157259	GATAD1	1.1	1.2E-01	1.7E-01	35.841	0.185	35.631	35.981	34.032	1.452	32.364	35.009
ENSG00000157426	AASDH	1.1	8.3E-03	1.6E-02	15.483	0.307	15.172	15.786	13.722	0.720	12.921	14.317
ENSG00000157450	RNF111	1.1	1.0E-02	2.0E-02	13.530	0.440	13.065	13.940	12.192	0.392	11.830	12.608
ENSG00000157456	CCNB2	1.1	7.7E-04	1.9E-03	125.529	5.938	119.204	130.984	112.747	4.869	108.028	117.754
ENSG00000157500	APPL1	1.1	2.2E-04	5.9E-04	45.675	1.608	44.059	47.274	40.398	1.270	39.263	41.769
ENSG00000157538	DSCR3	1.1	3.1E-03	6.6E-03	25.686	0.635	25.316	26.419	23.384	0.508	22.916	23.925
ENSG00000157540	DYRK1A	1.1	3.9E-02	6.4E-02	18.784	0.498	18.211	19.115	17.605	0.490	17.251	18.164
ENSG00000157796	WDR19	1.1	1.3E-01	1.9E-01	9.908	0.671	9.202	10.537	9.321	0.277	9.005	9.523
ENSG00000157827	FMNL2	1.1	1.0E-03	2.5E-03	45.485	0.630	44.939	46.174	40.417	2.821	37.338	42.879
ENSG00000157869	RAB28	1.1	1.7E-02	3.0E-02	40.068	1.783	38.954	42.125	36.006	2.660	32.957	37.854
ENSG00000157881	PANK4	1.1	9.5E-02	1.4E-01	13.007	1.202	11.749	14.144	11.840	1.235	10.489	12.910
ENSG00000158079	PTPDC1	1.1	2.7E-02	4.6E-02	6.556	0.564	5.914	6.973	5.742	0.167	5.631	5.934
ENSG00000158092	NCK1	1.1	1.1E-01	1.6E-01	22.183	0.514	21.866	22.776	20.673	0.568	20.031	21.111
ENSG00000158161	EYA3	1.1	2.0E-02	3.6E-02	22.494	0.780	21.932	23.385	20.433	1.292	18.973	21.426
ENSG00000158615	PPP1R15B	1.1	1.1E-01	1.6E-01	54.129	2.243	51.587	55.833	51.422	1.549	49.750	52.807
ENSG00000158623	COPG2	1.1	7.2E-05	2.2E-04	84.353	2.792	81.159	86.329	74.116	2.777	71.937	77.243
ENSG00000158691	ZSCAN12	1.1	2.7E-02	4.5E-02	10.213	0.681	9.775	10.997	9.031	1.010	8.239	10.168
ENSG00000158864	NDUFS2	1.1	1.8E-03	4.0E-03	99.528	5.214	96.114	105.529	90.222	1.140	89.052	91.331
ENSG00000159111	MRPL10	1.1	3.1E-02	5.2E-02	58.054	2.370	55.320	59.528	54.025	1.112	52.914	55.138
ENSG00000159164	SV2A	1.1	4.6E-04	1.2E-03	51.995	1.872	49.946	53.615	45.888	1.783	44.097	47.663
ENSG00000159256	MORC3	1.1	1.4E-02	2.6E-02	20.214	1.342	18.764	21.412	18.288	1.260	16.832	19.022
ENSG00000159267	HLCS	1.1	9.2E-02	1.4E-01	7.601	0.474	7.318	8.148	6.923	0.139	6.772	7.047
ENSG00000159346	ADIPOR1	1.1	5.3E-02	8.4E-02	77.617	3.974	73.186	80.868	73.242	1.554	71.470	74.372
ENSG00000159479	MED8	1.1	3.9E-02	6.4E-02	21.396	2.671	18.429	23.610	19.135	0.689	18.339	19.550
ENSG00000159592	GPBP1L1	1.1	1.9E-03	4.3E-03	56.592	0.319	56.233	56.843	51.935	0.153	51.826	52.110
ENSG00000160209	PDXK	1.1	1.3E-02	2.4E-02	21.324	0.509	20.853	21.864	19.681	1.091	18.423	20.359
ENSG00000160213	CSTB	1.1	9.7E-03	1.8E-02	55.489	1.236	54.248	56.719	50.970	1.287	49.484	51.738
ENSG00000160298	C21orf58	1.1	9.3E-02	1.4E-01	8.182	0.210	7.957	8.373	7.465	0.730	6.983	8.305
ENSG00000160445	ZER1	1.1	9.6E-02	1.4E-01	23.724	1.177	22.509	24.858	22.156	1.319	21.065	23.622
ENSG00000160679	CHTOP	1.1	4.4E-03	9.0E-03	62.889	0.991	61.898	63.880	58.000	0.899	57.291	59.011
ENSG00000161021	MAML1	1.1	2.4E-02	4.1E-02	23.116	0.480	22.691	23.636	21.436	1.101	20.398	22.591
ENSG00000161203	AP2M1	1.1	7.2E-04	1.8E-03	222.674	3.181	219.042	224.968	204.031	3.158	201.455	207.554
ENSG00000161243	FBXO27	1.1	7.2E-02	1.1E-01	11.352	0.873	10.371	12.041	10.073	0.814	9.242	10.868
ENSG00000161249	DMKN	1.1	1.2E-02	2.2E-02	37.284	1.251	35.892	38.312	34.601	0.707	34.096	35.409
ENSG00000162144	CYB561A3	1.1	6.6E-02	1.0E-01	4.694	0.393	4.253	5.005	4.268	0.114	4.165	4.391
ENSG00000162522	KIAA1522	1.1	1.1E-01	1.5E-01	45.121	2.015	42.954	46.939	42.923	1.966	41.286	45.104
ENSG00000162585	FAAP20	1.1	2.8E-02	4.8E-02	9.957	0.550	9.365	10.454	9.061	0.430	8.791	9.556
ENSG00000162601	MYSM1	1.1	5.8E-04	1.5E-03	47.949	3.719	43.656	50.158	41.933	1.748	40.277	43.760
ENSG00000162623	TYW3	1.1	7.5E-02	1.1E-01	46.772	1.147	45.714	47.991	44.205	1.739	42.362	45.816
ENSG00000162769	FLVCR1	1.1	1.4E-03	3.3E-03	37.720	1.146	36.563	38.854	33.912	0.621	33.551	34.629
ENSG00000162923	WDR26	1.1	3.7E-04	9.6E-04	42.765	1.153	41.852	44.061	38.156	0.976	37.197	39.148
ENSG00000162976	PQLC3	1.1	1.1E-01	1.5E-01	22.191	1.296	20.864	23.545	20.326	0.558	19.771	20.888
ENSG00000163041	H3F3A	1.1	2.3E-04	6.2E-04	310.880	4.521	305.713	314.113	283.001	4.135	280.562	287.775
ENSG00000163110	PDLIM5	1.1	4.7E-02	7.5E-02	16.575	0.339	16.228	16.905	15.398	0.501	14.829	15.771
ENSG00000163191	S100A11	1.1	1.7E-02	3.1E-02	387.089	27.835	359.490	415.153	364.629	28.251	338.098	387.250
ENSG00000163374	YY1AP1	1.1	1.3E-01	1.9E-01	19.055	1.433	17.766	20.598	17.890	0.639	17.159	18.345
ENSG00000163466	ARPC2	1.1	2.2E-02	3.9E-02	102.116	0.516	101.590	102.621	96.442	0.949	95.368	97.164
ENSG00000163528	CHCHD4	1.1	1.3E-01	1.8E-01	38.694	1.377	37.603	40.241	36.151	1.265	34.870	37.400
ENSG00000163539	CLASP2	1.1	9.8E-02	1.4E-01	17.964	0.305	17.656	18.265	17.153	0.597	16.522	17.709
ENSG00000163608	NEPRO	1.1	8.9E-04	2.1E-03	29.626	1.133	28.345	30.494	26.084	1.792	24.022	27.257
ENSG00000163625	WDFY3	1.1	5.6E-04	1.4E-03	17.764	0.300	17.453	18.052	15.943	0.298	15.737	16.285
ENSG00000163635	ATXN7	1.1	7.5E-02	1.1E-01	8.146	0.544	7.822	8.774	7.471	0.364	7.051	7.701
ENSG00000163681	SLMAP	1.1	1.4E-02	2.5E-02	16.568	0.074	16.503	16.648	15.300	0.475	14.873	15.811
ENSG00000163781	TOPBP1	1.1	3.8E-05	1.2E-04	50.072	1.574	48.307	51.331	43.986	1.159	43.201	45.318
ENSG00000163795	ZNF513	1.1	8.1E-02	1.2E-01	19.833	1.339	18.796	21.345	18.007	0.507	17.542	18.548
ENSG00000163811	WDR43	1.1	2.3E-02	3.9E-02	123.140	4.885	117.672	127.073	115.617	2.897	112.491	118.211
ENSG00000163875	MEAF6	1.1	7.4E-02	1.1E-01	32.825	1.734	31.051	34.517	30.496	1.466	28.938	31.847
ENSG00000163900	TMEM41A	1.1	5.6E-02	8.8E-02	6.196	0.127	6.054	6.299	5.624	0.102	5.522	5.726
ENSG00000163938	GNL3	1.1	4.1E-03	8.6E-03	220.814	3.191	217.893	224.220	203.941	0.827	202.986	204.441
ENSG00000164024	METAP1	1.1	1.1E-03	2.6E-03	129.079	6.102	124.262	135.941	116.836	3.048	114.601	120.308
ENSG00000164109	MAD2L1	1.1	1.8E-03	4.1E-03	88.594	0.879	87.838	89.559	80.719	3.887	77.030	84.778
ENSG00000164114	MAP9	1.1	1.3E-01	1.8E-01	9.327	1.003	8.173	9.985	8.687	0.513	8.096	9.022
ENSG00000164252	AGGF1	1.1	6.4E-03	1.3E-02	32.116	0.638	31.398	32.618	29.142	1.156	28.252	30.449
ENSG00000164253	WDR41	1.1	2.7E-04	7.2E-04	23.385	0.521	22.810	23.825	20.441	0.604	19.747	20.847
ENSG00000164258	NDUFS4	1.1	5.3E-04	1.3E-03	128.724	5.662	122.842	134.136	112.567	2.044	110.944	114.863
ENSG00000164294	GPX8	1.1	9.6E-03	1.8E-02	76.919	2.500	74.034	78.432	70.300	3.227	66.806	73.168
ENSG00000164346	NSA2	1.1	1.3E-03	3.0E-03	60.859	3.090	57.367	63.237	54.920	0.558	54.367	55.482
ENSG00000164484	TMEM200A	1.1	3.2E-03	6.7E-03	14.051	0.138	13.933	14.203	12.353	0.165	12.165	12.473
ENSG00000164506	STXBP5	1.1	5.7E-02	9.0E-02	6.035	0.395	5.780	6.490	5.479	0.323	5.253	5.849
ENSG00000164597	COG5	1.1	1.7E-03	3.8E-03	15.705	0.257	15.440	15.953	13.819	0.455	13.303	14.164
ENSG00000164611	PTTG1	1.1	1.4E-04	4.0E-04	231.536	11.247	224.167	244.481	206.728	7.419	199.056	213.124
ENSG00000164687	FABP5	1.1	8.3E-02	1.2E-01	58.009	2.964	55.067	60.995	54.047	2.281	51.580	56.079
ENSG00000164715	LMTK2	1.1	4.5E-04	1.2E-03	19.900	0.365	19.479	20.123	17.490	0.527	16.891	17.883
ENSG00000164751	PEX2	1.1	7.8E-02	1.2E-01	23.368	2.133	21.886	25.813	21.633	1.243	20.198	22.381
ENSG00000164823	OSGIN2	1.1	1.6E-02	2.9E-02	15.409	1.062	14.409	16.524	13.604	0.337	13.215	13.817
ENSG00000164830	OXR1	1.1	1.1E-01	1.6E-01	13.003	0.434	12.526	13.377	12.130	0.487	11.577	12.491
ENSG00000164953	TMEM67	1.1	1.2E-01	1.7E-01	5.299	0.757	4.567	6.078	4.802	0.311	4.611	5.162
ENSG00000165322	ARHGAP12	1.1	4.2E-03	8.8E-03	44.919	1.894	43.305	47.005	40.765	1.578	39.545	42.547
ENSG00000165480	SKA3	1.1	8.1E-02	1.2E-01	64.830	2.323	62.414	67.047	60.826	1.145	59.578	61.828
ENSG00000165487	MICU2	1.1	3.9E-03	8.2E-03	51.398	1.460	49.713	52.311	46.168	2.355	44.072	48.716
ENSG00000165609	NUDT5	1.1	1.8E-03	4.1E-03	76.684	2.121	74.255	78.171	69.679	0.346	69.375	70.056
ENSG00000165660	FAM175B	1.1	7.7E-02	1.2E-01	26.824	0.785	26.000	27.563	24.868	0.664	24.341	25.613
ENSG00000165689	SDCCAG3	1.1	6.9E-02	1.1E-01	64.317	2.331	61.715	66.215	60.246	3.751	57.427	64.503
ENSG00000165699	TSC1	1.1	8.4E-03	1.6E-02	16.325	0.354	16.041	16.722	14.924	0.344	14.639	15.306
ENSG00000165732	DDX21	1.1	2.0E-02	3.5E-02	363.277	4.714	359.922	368.666	341.282	5.280	337.021	347.189
ENSG00000165895	ARHGAP42	1.1	3.3E-03	6.9E-03	14.839	0.542	14.220	15.226	12.971	0.550	12.358	13.424
ENSG00000165914	TTC7B	1.1	4.5E-02	7.3E-02	3.230	0.119	3.135	3.364	2.942	0.126	2.808	3.057
ENSG00000165929	TC2N	1.1	1.4E-02	2.5E-02	28.300	0.233	28.081	28.545	25.999	0.588	25.311	26.394
ENSG00000166024	R3HCC1L	1.1	4.4E-02	7.2E-02	21.457	1.109	20.307	22.519	19.465	0.384	19.089	19.857
ENSG00000166128	RAB8B	1.1	2.5E-03	5.5E-03	43.992	1.658	42.268	45.574	39.476	2.427	36.765	41.448
ENSG00000166140	ZFYVE19	1.1	7.3E-02	1.1E-01	6.018	0.541	5.394	6.351	5.317	0.254	5.027	5.501
ENSG00000166169	POLL	1.1	2.3E-02	3.9E-02	14.637	0.331	14.255	14.848	13.228	0.133	13.093	13.359
ENSG00000166260	COX11	1.1	2.0E-02	3.6E-02	19.494	0.813	18.735	20.352	17.841	0.684	17.108	18.462
ENSG00000166348	USP54	1.1	4.8E-04	1.2E-03	19.148	0.146	18.997	19.290	16.781	0.954	15.840	17.748
ENSG00000166426	CRABP1	1.1	2.3E-02	3.9E-02	157.080	12.049	143.762	167.223	142.191	9.239	132.514	150.919
ENSG00000166441	RPL27A	1.1	4.8E-06	1.8E-05	199.875	8.119	191.016	206.961	174.939	3.377	172.345	178.758
ENSG00000166526	ZNF3	1.1	7.3E-02	1.1E-01	31.285	1.649	29.627	32.925	29.404	0.588	28.736	29.886
ENSG00000166579	NDEL1	1.1	1.4E-01	1.9E-01	10.196	0.478	9.794	10.724	9.585	0.416	9.201	10.028
ENSG00000166710	B2M	1.1	2.0E-03	4.5E-03	25.684	0.603	25.058	26.261	22.946	1.214	21.723	24.152
ENSG00000166855	CLPX	1.1	7.5E-02	1.1E-01	42.577	1.047	41.672	43.724	40.237	2.644	37.256	42.299
ENSG00000166912	MTMR10	1.1	5.5E-02	8.7E-02	8.762	0.166	8.579	8.902	8.066	0.226	7.890	8.321
ENSG00000166997	CNPY4	1.1	1.4E-01	2.0E-01	10.975	0.364	10.664	11.375	9.868	1.313	8.407	10.949
ENSG00000167196	FBXO22	1.1	1.1E-02	2.0E-02	26.633	0.832	25.701	27.302	23.915	1.148	22.591	24.605
ENSG00000167232	ZNF91	1.1	2.0E-02	3.5E-02	13.957	1.630	12.353	15.611	12.594	0.888	11.863	13.582
ENSG00000167283	ATP5L	1.1	1.2E-05	4.3E-05	129.502	3.766	125.391	132.785	113.464	0.152	113.340	113.634
ENSG00000167378	IRGQ	1.1	1.9E-02	3.4E-02	11.677	0.627	11.292	12.401	10.550	0.719	10.128	11.380
ENSG00000167468	GPX4	1.1	4.1E-02	6.7E-02	189.391	2.794	187.081	192.496	179.231	6.182	175.131	186.341
ENSG00000167515	TRAPPC2L	1.1	1.4E-01	1.9E-01	30.110	0.808	29.396	30.987	28.601	1.025	27.590	29.640
ENSG00000167526	RPL13	1.1	1.7E-02	3.0E-02	296.633	10.385	285.743	306.426	270.238	24.332	248.966	296.770
ENSG00000167555	ZNF528	1.1	1.1E-02	2.1E-02	6.870	0.239	6.601	7.060	5.997	0.338	5.785	6.387
ENSG00000167562	ZNF701	1.1	9.2E-02	1.4E-01	3.792	0.190	3.630	4.001	3.392	0.236	3.175	3.644
ENSG00000167635	ZNF146	1.1	5.3E-03	1.1E-02	181.819	7.488	173.596	188.245	168.837	5.408	163.003	173.684
ENSG00000167645	YIF1B	1.1	6.9E-02	1.1E-01	36.155	1.301	34.690	37.173	33.624	2.068	32.242	36.002
ENSG00000167771	RCOR2	1.1	1.1E-02	2.1E-02	60.020	2.890	56.883	62.574	54.620	2.501	51.886	56.792
ENSG00000167969	ECI1	1.1	5.0E-03	1.0E-02	28.749	0.385	28.308	29.018	25.234	2.008	23.022	26.944
ENSG00000167987	VPS37C	1.1	1.0E-01	1.5E-01	12.069	0.446	11.810	12.584	11.064	0.866	10.089	11.742
ENSG00000168038	ULK4	1.1	6.3E-02	9.7E-02	4.304	0.323	4.035	4.663	3.813	0.085	3.740	3.907
ENSG00000168040	FADD	1.1	6.1E-02	9.4E-02	69.929	3.556	67.714	74.030	64.866	1.677	63.053	66.363
ENSG00000168172	HOOK3	1.1	4.6E-03	9.3E-03	9.383	0.496	8.866	9.856	8.348	0.428	7.861	8.665
ENSG00000168268	NT5DC2	1.1	3.3E-03	6.9E-03	130.461	2.650	127.935	133.219	120.581	2.651	118.079	123.359
ENSG00000168374	ARF4	1.1	7.6E-04	1.9E-03	238.901	10.340	230.375	250.402	217.639	5.667	211.138	221.535
ENSG00000168461	RAB31	1.1	4.1E-02	6.7E-02	35.954	0.705	35.147	36.450	33.591	1.122	32.884	34.884
ENSG00000168502	MTCL1	1.1	5.8E-03	1.2E-02	11.414	0.460	11.139	11.945	10.257	0.422	9.772	10.541
ENSG00000168522	FNTA	1.1	5.8E-02	9.1E-02	19.361	0.712	18.890	20.179	18.163	0.352	17.804	18.509
ENSG00000168575	SLC20A2	1.1	8.5E-03	1.6E-02	17.972	0.740	17.364	18.796	16.138	0.451	15.645	16.529
ENSG00000168653	NDUFS5	1.1	3.9E-05	1.3E-04	1378.601	13.158	1364.008	1389.562	1211.989	27.992	1184.212	1240.191
ENSG00000168672	FAM84B	1.1	5.9E-02	9.2E-02	54.619	0.934	53.736	55.596	49.990	1.989	47.717	51.408
ENSG00000168724	DNAJC21	1.1	4.9E-03	9.9E-03	23.340	1.662	21.454	24.587	21.124	0.745	20.450	21.923
ENSG00000168887	C2orf68	1.1	5.1E-03	1.0E-02	24.532	0.385	24.092	24.809	21.934	1.068	20.828	22.959
ENSG00000168952	STXBP6	1.1	9.2E-02	1.4E-01	3.775	0.565	3.272	4.386	3.343	0.220	3.102	3.533
ENSG00000169019	COMMD8	1.1	1.0E-01	1.5E-01	11.244	1.179	10.051	12.408	9.833	1.126	8.859	11.066
ENSG00000169020	ATP5I	1.1	1.3E-03	3.0E-03	339.053	10.508	330.811	350.886	304.058	14.387	287.521	313.704
ENSG00000169047	IRS1	1.1	1.4E-02	2.6E-02	13.415	0.322	13.043	13.616	12.092	0.897	11.059	12.673
ENSG00000169203	NPIPB12	1.1	8.8E-02	1.3E-01	7.626	0.552	7.266	8.262	6.849	0.486	6.345	7.337
ENSG00000169249	ZRSR2	1.1	4.4E-02	7.2E-02	20.656	0.711	19.853	21.374	18.375	1.727	16.990	20.311
ENSG00000169439	SDC2	1.1	3.2E-02	5.3E-02	68.646	3.405	65.537	72.285	63.644	1.179	62.319	64.578
ENSG00000169740	ZNF32	1.1	5.7E-02	8.9E-02	40.973	0.388	40.597	41.371	37.394	0.429	36.939	37.792
ENSG00000169756	LIMS1	1.1	6.7E-04	1.7E-03	21.866	0.634	21.136	22.279	19.140	0.545	18.571	19.657
ENSG00000169992	NLGN2	1.1	2.2E-03	4.8E-03	28.650	1.485	27.669	30.358	25.483	1.671	23.709	27.027
ENSG00000170037	CNTROB	1.1	3.9E-03	8.2E-03	14.068	0.351	13.708	14.410	12.303	0.581	11.687	12.855
ENSG00000170043	TRAPPC1	1.1	3.4E-02	5.6E-02	84.150	4.854	79.047	88.708	76.625	3.646	74.079	80.802
ENSG00000170089		1.1	6.9E-03	1.3E-02	53.363	1.530	51.627	54.520	48.752	1.576	47.421	50.493
ENSG00000170142	UBE2E1	1.1	1.0E-03	2.4E-03	151.566	4.682	148.379	156.942	136.420	7.476	127.801	141.142
ENSG00000170144	HNRNPA3	1.1	2.6E-02	4.5E-02	264.864	5.685	260.505	271.294	250.370	13.644	235.603	262.509
ENSG00000170190	SLC16A5	1.1	1.4E-01	2.0E-01	11.719	0.431	11.277	12.138	10.725	0.722	9.903	11.257
ENSG00000170348	TMED10	1.1	7.1E-05	2.2E-04	153.344	3.362	149.462	155.336	137.196	4.706	134.352	142.628
ENSG00000170448	NFXL1	1.1	4.5E-03	9.1E-03	28.117	0.919	27.193	29.032	25.103	1.584	23.899	26.885
ENSG00000170542	SERPINB9	1.1	4.5E-02	7.2E-02	14.684	1.089	13.431	15.405	13.161	0.294	12.822	13.349
ENSG00000170759	KIF5B	1.1	4.6E-04	1.2E-03	110.994	4.321	107.994	115.946	100.412	2.559	97.498	102.293
ENSG00000170906	NDUFA3	1.1	2.1E-02	3.6E-02	43.884	3.598	39.850	46.761	39.856	0.427	39.504	40.332
ENSG00000171045	TSNARE1	1.1	1.3E-01	1.8E-01	10.264	0.161	10.083	10.391	9.380	1.302	8.480	10.872
ENSG00000171150	SOCS5	1.1	4.1E-02	6.7E-02	16.362	0.258	16.074	16.572	14.987	0.416	14.746	15.467
ENSG00000171219	CDC42BPG	1.1	9.6E-03	1.8E-02	9.194	0.350	8.956	9.596	8.029	0.133	7.876	8.120
ENSG00000171466	ZNF562	1.1	2.9E-02	4.9E-02	12.696	0.788	11.812	13.326	11.754	0.219	11.609	12.006
ENSG00000171490	RSL1D1	1.1	2.3E-03	5.0E-03	119.047	1.961	116.864	120.661	109.264	2.074	107.832	111.641
ENSG00000171530	TBCA	1.1	8.8E-02	1.3E-01	126.254	3.875	121.878	129.250	120.372	3.441	116.731	123.571
ENSG00000171603	CLSTN1	1.1	3.0E-02	5.0E-02	57.836	0.221	57.669	58.087	54.101	3.638	51.287	58.210
ENSG00000171604	CXXC5	1.1	2.6E-03	5.6E-03	55.042	2.630	52.463	57.700	49.200	2.166	47.034	51.366
ENSG00000171608	PIK3CD	1.1	2.1E-02	3.7E-02	12.932	1.604	11.138	14.227	11.408	0.377	11.008	11.758
ENSG00000171863	RPS7	1.1	2.1E-04	5.8E-04	129.653	2.163	127.288	131.529	116.890	3.350	113.138	119.580
ENSG00000171877	FRMD5	1.1	1.8E-02	3.1E-02	8.196	0.539	7.586	8.514	7.237	0.255	7.030	7.522
ENSG00000171928	TVP23B	1.1	3.6E-02	6.0E-02	12.198	0.426	11.897	12.686	10.938	0.882	10.274	11.939
ENSG00000171943	SRGAP2C	1.1	8.4E-02	1.3E-01	8.314	0.418	7.945	8.767	7.626	0.414	7.375	8.104
ENSG00000172053	QARS	1.1	4.6E-06	1.8E-05	137.677	3.115	134.516	140.743	120.191	3.164	117.465	123.661
ENSG00000172164	SNTB1	1.1	3.0E-03	6.3E-03	14.713	0.902	13.680	15.349	12.873	0.367	12.470	13.188
ENSG00000172270	BSG	1.1	1.8E-02	3.3E-02	329.972	6.640	323.540	336.802	310.707	19.583	292.360	331.328
ENSG00000172340	SUCLG2	1.1	1.4E-03	3.2E-03	88.001	1.984	85.730	89.396	79.133	2.580	76.257	81.244
ENSG00000172354	GNB2	1.1	2.1E-02	3.6E-02	87.530	3.258	83.909	90.221	80.828	5.587	75.345	86.533
ENSG00000172380	GNG12	1.1	5.4E-04	1.4E-03	60.484	2.292	58.964	63.120	53.030	2.448	50.919	55.713
ENSG00000172493	AFF1	1.1	7.2E-02	1.1E-01	29.537	0.559	29.153	30.178	28.128	0.961	27.338	29.198
ENSG00000172531	PPP1CA	1.1	1.1E-02	2.1E-02	161.077	4.007	156.820	164.774	149.650	6.114	143.501	155.728
ENSG00000172757	CFL1	1.1	3.6E-03	7.6E-03	310.138	5.455	303.921	314.121	285.769	13.176	270.990	296.285
ENSG00000172775	FAM192A	1.1	3.4E-02	5.7E-02	43.075	0.826	42.146	43.727	40.309	0.845	39.717	41.277
ENSG00000172795	DCP2	1.1	2.4E-02	4.2E-02	74.733	2.515	71.829	76.225	70.084	4.264	65.447	73.838
ENSG00000173065	FAM222B	1.1	6.2E-02	9.6E-02	40.319	1.207	39.608	41.713	37.936	2.407	35.239	39.865
ENSG00000173272	MZT2A	1.1	1.9E-02	3.4E-02	20.684	1.323	19.157	21.495	18.293	1.470	17.389	19.990
ENSG00000173542	MOB1B	1.1	1.8E-03	4.1E-03	22.229	1.659	20.431	23.701	19.747	1.282	18.500	21.062
ENSG00000173660	UQCRH	1.1	1.2E-02	2.2E-02	372.510	8.390	365.161	381.652	347.555	11.959	334.126	357.058
ENSG00000174021	GNG5	1.1	2.0E-04	5.6E-04	222.887	4.441	219.102	227.776	197.511	3.494	195.224	201.533
ENSG00000174028	FAM3C2	1.1	8.3E-02	1.2E-01	153.869	8.102	146.983	162.796	142.410	10.594	134.903	154.528
ENSG00000174136	RGMB	1.1	8.0E-03	1.6E-02	10.235	0.030	10.209	10.268	9.026	0.188	8.816	9.178
ENSG00000174231	PRPF8	1.1	5.4E-02	8.5E-02	117.463	0.736	116.767	118.234	111.708	4.544	106.953	116.007
ENSG00000174579	MSL2	1.1	7.1E-02	1.1E-01	27.399	1.644	26.060	29.234	25.704	0.516	25.138	26.148
ENSG00000174672	BRSK2	1.1	1.5E-02	2.7E-02	7.788	0.260	7.490	7.971	6.812	0.088	6.718	6.894
ENSG00000174851	YIF1A	1.1	2.0E-02	3.5E-02	39.481	0.887	38.456	39.998	35.722	1.788	34.303	37.744
ENSG00000174891	RSRC1	1.1	7.6E-02	1.2E-01	14.077	0.963	13.272	15.144	12.970	0.881	12.019	13.759
ENSG00000175104	TRAF6	1.1	3.6E-03	6.0E-03	8.021	0.652	7.399	8.699	7.037	0.558	6.393	7.363
ENSG00000175198	PCCA	1.1	6.8E-03	1.3E-02	16.266	0.229	16.034	16.491	14.607	0.394	14.317	15.055
ENSG00000175203	DCTN2	1.1	2.3E-03	5.1E-03	65.979	1.528	64.502	67.553	59.973	1.802	58.764	62.045
ENSG00000175309	PHYKPL	1.1	4.0E-02	6.6E-02	4.107	0.398	3.757	4.540	3.614	0.081	3.564	3.708
ENSG00000175354	PTPN2	1.1	6.0E-03	1.2E-02	22.271	0.672	21.495	22.681	19.956	0.603	19.259	20.325
ENSG00000175416	CLTB	1.1	5.7E-02	8.9E-02	22.544	1.399	21.196	23.988	20.267	1.965	18.018	21.661
ENSG00000175445	LPL	1.1	1.3E-02	2.4E-02	13.723	0.648	12.997	14.245	12.015	0.548	11.483	12.577
ENSG00000175550	DRAP1	1.1	3.0E-02	5.1E-02	73.327	2.135	71.953	75.786	67.323	5.453	61.601	72.460
ENSG00000175893	ZDHHC21	1.1	3.5E-02	5.8E-02	9.716	0.804	8.859	10.453	8.637	1.115	7.667	9.855
ENSG00000176018	LYSMD3	1.1	1.3E-02	2.4E-02	22.132	0.385	21.704	22.449	19.743	1.805	18.318	21.772
ENSG00000176135	ZNF135	1.1	1.1E-01	1.6E-01	3.710	0.332	3.331	3.943	3.304	0.128	3.166	3.417
ENSG00000176393	RNPEP	1.1	5.3E-03	8.4E-03	49.392	2.009	47.777	51.642	46.330	0.722	45.853	47.161
ENSG00000176407	KCMF1	1.1	1.6E-03	3.6E-03	45.548	0.885	44.683	46.452	41.374	1.302	39.953	42.511
ENSG00000176623	RMDN1	1.1	3.4E-02	5.7E-02	18.589	0.570	17.931	18.918	17.040	0.724	16.236	17.640
ENSG00000176853	FAM91A1	1.1	3.5E-02	5.8E-02	17.922	1.038	16.864	18.939	16.452	0.674	15.698	16.997
ENSG00000177119	ANO6	1.1	2.4E-02	4.2E-02	46.004	1.216	45.096	47.385	43.067	1.429	41.861	44.645
ENSG00000177426	TGIF1	1.1	3.3E-03	6.9E-03	51.731	0.809	50.818	52.356	47.418	0.204	47.214	47.622
ENSG00000177463	NR2C2	1.1	3.9E-03	8.0E-03	17.546	0.483	17.201	18.098	15.909	0.392	15.527	16.311
ENSG00000177595	PIDD1	1.1	3.2E-02	5.4E-02	7.252	0.270	7.026	7.550	6.355	0.171	6.252	6.552
ENSG00000177600	RPLP2	1.1	2.4E-02	4.2E-02	697.238	21.629	672.315	711.092	658.207	27.141	628.783	682.265
ENSG00000177728	TMEM94	1.1	4.8E-02	7.7E-02	11.894	0.989	10.808	12.741	10.906	0.379	10.618	11.335
ENSG00000177731	FLII	1.1	8.2E-02	1.2E-01	89.451	3.977	85.862	93.726	85.300	4.718	79.859	88.251
ENSG00000177868	SVBP	1.1	3.9E-02	6.4E-02	51.500	3.631	48.553	55.556	46.339	4.245	42.438	50.860
ENSG00000177888	ZBTB41	1.1	2.5E-02	4.2E-02	6.242	0.562	5.769	6.863	5.496	0.485	5.017	5.988
ENSG00000177932	ZNF354C	1.1	8.9E-02	1.3E-01	10.229	0.710	9.413	10.709	9.362	0.758	8.795	10.223
ENSG00000178053	MLF1	1.1	1.3E-01	1.8E-01	43.599	0.635	42.933	44.199	41.338	1.953	39.124	42.818
ENSG00000178057	NDUFAF3	1.1	1.5E-01	2.0E-01	20.975	1.289	19.511	21.941	19.234	0.863	18.376	20.101
ENSG00000178397	FAM220A	1.1	8.7E-03	1.7E-02	31.784	0.755	31.109	32.599	27.939	2.622	25.838	30.878
ENSG00000178982	EIF3K	1.1	5.2E-05	1.6E-04	162.507	5.172	156.619	166.319	143.628	5.612	140.241	150.106
ENSG00000179523	EIF3J-AS1	1.1	3.4E-02	5.6E-02	10.370	0.429	9.919	10.773	9.118	0.036	9.077	9.145
ENSG00000180346	TIGD2	1.1	8.5E-02	1.3E-01	10.232	0.314	9.890	10.507	9.199	0.516	8.605	9.527
ENSG00000180357	ZNF609	1.1	3.7E-02	6.1E-02	29.087	0.713	28.317	29.724	27.249	1.181	25.903	28.114
ENSG00000180448	ARHGAP45	1.1	5.4E-02	8.6E-02	11.814	0.267	11.506	11.970	10.684	0.793	10.097	11.586
ENSG00000180574		1.1	1.6E-03	3.7E-03	136.869	3.915	132.754	140.547	123.735	0.814	123.156	124.666
ENSG00000180917	CMTR2	1.1	1.6E-02	2.9E-02	11.384	0.898	10.783	12.417	10.164	0.572	9.514	10.594
ENSG00000181007	ZFP82	1.1	5.0E-03	1.0E-02	11.259	0.552	10.921	11.896	9.942	0.184	9.731	10.068
ENSG00000181690	PLAG1	1.1	8.9E-02	1.3E-01	11.773	0.439	11.271	12.089	10.944	0.805	10.184	11.788
ENSG00000181885	CLDN7	1.1	3.6E-03	7.5E-03	85.704	2.570	82.847	87.828	77.737	0.886	77.187	78.759
ENSG00000181924	COA4	1.1	1.7E-02	3.0E-02	98.933	4.367	95.469	103.838	91.334	4.795	86.885	96.413
ENSG00000182220	ATP6AP2	1.1	3.2E-03	6.8E-03	29.874	1.063	28.658	30.624	27.292	0.899	26.260	27.908
ENSG00000182628	SKA2	1.1	2.3E-02	4.0E-02	57.864	2.457	55.160	59.960	51.521	3.545	47.454	53.950
ENSG00000182636	NDN	1.1	1.5E-02	2.7E-02	70.736	0.979	69.701	71.646	64.190	1.190	62.863	65.163
ENSG00000182774	RPS17	1.1	3.1E-05	9.7E-05	525.182	17.970	505.636	540.986	464.262	19.704	442.099	479.803
ENSG00000182827	ACBD3	1.1	3.1E-03	6.6E-03	49.386	1.284	48.043	50.601	44.295	2.668	41.236	46.146
ENSG00000182899	RPL35A	1.1	3.0E-06	9.9E-06	541.538	19.358	524.043	562.334	482.596	9.783	472.367	491.861
ENSG00000182903	ZNF721	1.1	1.0E-01	1.5E-01	30.439	2.261	27.923	32.299	28.643	1.677	26.908	30.255
ENSG00000182963	GJC1	1.1	2.8E-03	6.1E-03	61.603	1.077	60.412	62.506	56.495	2.035	54.230	58.169
ENSG00000183020	AP2A2	1.1	6.1E-02	9.5E-02	15.371	0.663	14.619	15.870	14.414	0.345	14.117	14.792
ENSG00000183255	PTTG1IP	1.1	1.1E-03	2.7E-03	135.415	3.793	131.875	139.419	123.471	4.425	118.441	126.763
ENSG00000183291	SELENOF	1.1	8.9E-03	1.7E-02	182.393	4.945	176.753	185.982	168.972	4.767	163.885	173.484
ENSG00000183513	COA5	1.1	9.6E-03	1.8E-02	29.336	1.113	28.465	30.590	25.969	1.300	25.247	27.354
ENSG00000183723	CMTM4	1.1	1.3E-04	3.8E-04	46.477	1.200	45.238	47.634	41.271	0.392	40.872	41.656
ENSG00000183735	TBK1	1.1	2.2E-02	3.9E-02	28.713	2.489	25.995	30.882	26.172	0.968	25.314	27.222
ENSG00000183856	IQGAP3	1.1	9.1E-03	1.7E-02	17.108	0.817	16.483	18.033	15.420	0.606	15.056	16.120
ENSG00000184117	NIPSNAP1	1.1	5.9E-04	1.5E-03	220.658	5.394	215.065	225.829	199.851	1.715	198.533	201.790
ENSG00000184207	PGP	1.1	1.2E-01	1.8E-01	37.371	1.593	35.658	38.809	34.816	3.403	30.888	36.853
ENSG00000184220	CMSS1	1.1	7.3E-02	1.1E-01	23.551	0.847	22.639	24.314	21.944	0.248	21.788	22.230
ENSG00000184635	ZNF93	1.1	3.0E-04	7.8E-04	38.884	0.609	38.221	39.418	33.955	1.383	32.449	35.167
ENSG00000184677	ZBTB40	1.1	1.0E-01	1.5E-01	12.215	0.480	11.775	12.726	11.371	0.096	11.263	11.445
ENSG00000184697	CLDN6	1.1	9.9E-03	1.9E-02	722.274	10.705	711.303	732.691	675.726	19.302	654.904	692.736
ENSG00000184719	RNLS	1.1	1.3E-01	1.9E-01	5.595	0.588	4.917	5.943	4.961	0.616	4.254	5.374
ENSG00000184787	UBE2G2	1.1	2.6E-03	5.6E-03	46.112	3.272	42.783	49.324	41.731	1.639	40.046	43.319
ENSG00000184983	NDUFA6	1.1	1.6E-03	3.7E-03	159.843	5.044	154.066	163.374	141.873	5.241	137.614	147.726
ENSG00000185222	TCEAL9	1.1	1.9E-02	3.4E-02	206.293	10.072	196.719	216.799	190.518	10.005	183.125	201.902
ENSG00000185236	RAB11B	1.1	5.4E-03	1.1E-02	57.251	1.757	55.241	58.488	51.545	2.407	50.107	54.324
ENSG00000185262	UBALD2	1.1	4.4E-02	7.2E-02	18.538	0.663	17.795	19.068	16.300	0.687	15.547	16.922
ENSG00000185475	TMEM179B	1.1	8.6E-02	1.3E-01	16.422	0.472	15.992	16.926	14.391	2.028	12.969	16.714
ENSG00000185515	BRCC3	1.1	3.6E-03	7.6E-03	33.209	0.874	32.209	33.826	29.370	1.428	27.814	30.620
ENSG00000185591	SP1	1.1	4.1E-04	1.1E-03	57.730	1.952	56.131	59.906	52.251	1.029	51.128	53.150
ENSG00000185658	BRWD1	1.1	8.2E-02	1.2E-01	19.469	0.218	19.287	19.711	18.598	0.655	17.863	19.121
ENSG00000185716	C16orf52	1.1	5.6E-02	8.9E-02	10.333	0.677	9.581	10.893	9.358	0.626	8.665	9.880
ENSG00000186001	LRCH3	1.1	1.4E-02	2.5E-02	12.888	0.446	12.481	13.365	11.857	0.313	11.627	12.213
ENSG00000186184	POLR1D	1.1	1.5E-05	5.3E-05	30.162	0.154	30.071	30.340	26.332	0.719	25.535	26.932
ENSG00000186222	BLOC1S4	1.1	6.4E-02	9.9E-02	30.714	3.581	26.632	33.327	27.537	0.799	26.866	28.421
ENSG00000186230	ZNF749	1.1	2.7E-02	4.5E-02	17.108	1.561	15.929	18.878	15.132	0.734	14.353	15.812
ENSG00000186298	PPP1CC	1.1	3.2E-03	6.8E-03	265.356	0.966	264.650	266.456	247.904	6.232	242.020	254.435
ENSG00000186446	ZNF501	1.1	8.6E-02	1.3E-01	6.300	0.496	5.729	6.618	5.589	0.197	5.371	5.752
ENSG00000186468	RPS23	1.1	1.4E-06	6.0E-06	622.628	10.753	610.224	629.334	545.371	18.590	531.788	566.558
ENSG00000186523	FAM86B1	1.1	8.5E-02	1.3E-01	7.372	0.295	7.088	7.677	6.602	0.196	6.470	6.827
ENSG00000186591	UBE2H	1.1	7.7E-05	2.3E-04	73.690	2.112	72.056	76.076	65.116	1.871	64.000	67.276
ENSG00000186716	BCR	1.1	3.5E-03	7.3E-03	32.356	0.983	31.221	32.939	29.384	1.890	27.272	30.916
ENSG00000186814	ZSCAN30	1.1	9.3E-02	1.4E-01	9.952	1.202	8.771	11.174	9.216	0.126	9.077	9.323
ENSG00000186908	ZDHHC17	1.1	7.1E-02	1.1E-01	18.976	0.834	18.013	19.478	17.754	1.602	16.627	19.588
ENSG00000187446	CHP1	1.1	6.0E-04	1.5E-03	57.096	0.705	56.515	57.880	50.917	0.548	50.345	51.438
ENSG00000187653	TMSB4XP8	1.1	7.6E-06	2.8E-05	7588.210	173.931	7457.030	7785.504	6655.324	302.410	6388.128	6983.618
ENSG00000187720	THSD4	1.1	1.0E-01	1.5E-01	2.052	0.115	1.966	2.183	1.844	0.077	1.755	1.891
ENSG00000187801	ZFP69B	1.1	1.3E-01	1.8E-01	10.781	0.212	10.544	10.953	9.679	1.376	8.138	10.783
ENSG00000188243	COMMD6	1.1	7.5E-04	1.8E-03	54.700	4.359	51.535	59.672	47.775	1.802	45.752	49.210
ENSG00000188283	ZNF383	1.1	1.3E-01	1.9E-01	2.673	0.228	2.411	2.806	2.370	0.045	2.323	2.414
ENSG00000188313	PLSCR1	1.1	8.4E-02	1.3E-01	24.370	1.464	22.740	25.571	22.669	0.782	21.900	23.464
ENSG00000188612	SUMO2	1.1	1.5E-03	3.4E-03	227.274	4.217	224.194	232.080	206.614	13.067	193.154	219.248
ENSG00000188643	S100A16	1.1	8.7E-02	1.3E-01	33.701	1.865	32.433	35.842	30.624	1.610	29.661	32.483
ENSG00000188811	NHLRC3	1.1	2.0E-02	3.5E-02	11.022	0.359	10.713	11.415	9.873	0.446	9.521	10.375
ENSG00000189067	LITAF	1.1	4.0E-03	8.4E-03	53.764	1.920	52.323	55.944	48.361	2.058	46.864	50.708
ENSG00000196172	ZNF681	1.1	9.7E-03	1.8E-02	13.604	1.395	12.617	15.199	11.957	0.588	11.437	12.610
ENSG00000196182	STK40	1.1	9.3E-03	1.8E-02	19.978	1.232	18.568	20.849	17.607	1.102	16.343	18.369
ENSG00000196204	RNF216P1	1.1	2.2E-02	3.8E-02	13.244	0.258	13.012	13.522	11.845	0.830	11.205	12.782
ENSG00000196233	LCOR	1.1	8.4E-03	1.6E-02	5.327	0.154	5.231	5.504	4.821	0.101	4.718	4.919
ENSG00000196312	MFSD14C	1.1	3.5E-02	5.8E-02	11.522	0.909	10.857	12.557	10.154	0.342	9.891	10.540
ENSG00000196419	XRCC6	1.1	4.0E-03	8.3E-03	695.532	7.341	691.067	704.005	652.284	10.512	642.426	663.346
ENSG00000196428	TSC22D2	1.1	5.9E-02	9.3E-02	7.605	0.427	7.146	7.989	6.964	0.576	6.484	7.603
ENSG00000196465	MYL6B	1.1	1.7E-03	3.8E-03	66.566	0.853	65.589	67.164	59.284	0.579	58.829	59.935
ENSG00000196470	SIAH1	1.1	2.0E-03	4.5E-03	15.342	0.776	14.892	16.238	13.408	0.454	13.045	13.917
ENSG00000196531	NACA	1.1	5.8E-07	2.6E-06	212.729	1.742	210.718	213.780	185.545	2.970	183.192	188.882
ENSG00000196586	MYO6	1.1	1.0E-02	1.9E-02	18.116	0.489	17.816	18.680	16.532	0.179	16.330	16.671
ENSG00000196632	WNK3	1.1	3.6E-02	5.9E-02	11.613	0.141	11.503	11.773	10.732	0.339	10.341	10.947
ENSG00000196792	STRN3	1.1	6.1E-02	9.4E-02	25.548	1.061	24.323	26.176	23.829	1.015	22.741	24.749
ENSG00000196924	FLNA	1.1	5.9E-02	9.2E-02	397.594	11.141	387.804	409.716	376.498	19.202	358.402	397.273
ENSG00000197013	ZNF429	1.1	4.5E-02	7.3E-02	6.538	0.292	6.327	6.871	5.780	0.416	5.423	6.237
ENSG00000197217	ENTPD4	1.1	3.4E-04	8.9E-04	28.185	1.384	26.843	29.608	24.773	0.735	24.046	25.515
ENSG00000197302	ZNF720	1.1	8.6E-03	1.7E-02	9.085	0.660	8.688	9.847	7.961	0.581	7.488	8.610
ENSG00000197343	ZNF655	1.1	1.2E-02	2.3E-02	19.831	0.622	19.225	20.467	18.010	1.508	16.280	19.045
ENSG00000197535	MYO5A	1.1	1.5E-03	3.4E-03	12.316	0.522	11.741	12.760	10.977	0.442	10.468	11.236
ENSG00000197555	SIPA1L1	1.1	2.8E-02	4.7E-02	29.359	1.252	28.514	30.798	27.578	0.536	26.965	27.960
ENSG00000197601	FAR1	1.1	8.3E-04	2.0E-03	58.002	4.263	53.891	62.402	50.788	3.149	47.884	54.136
ENSG00000197746	PSAP	1.1	1.7E-03	3.9E-03	478.157	7.872	471.820	486.968	444.087	3.099	441.016	447.214
ENSG00000197971	MBP	1.1	5.2E-02	8.2E-02	5.701	0.137	5.546	5.804	5.269	0.241	5.069	5.536
ENSG00000198015	MRPL42	1.1	1.0E-03	2.5E-03	30.634	0.823	30.092	31.581	27.461	1.799	25.521	29.074
ENSG00000198146	ZNF770	1.1	2.5E-04	6.7E-04	146.006	6.653	138.429	150.892	130.376	5.683	125.055	136.362
ENSG00000198160	MIER1	1.1	3.7E-03	7.8E-03	18.223	1.150	16.903	19.009	16.193	1.089	15.230	17.374
ENSG00000198189	HSD17B11	1.1	1.0E-01	1.5E-01	14.657	1.844	12.532	15.847	13.303	0.371	12.876	13.539
ENSG00000198315	ZKSCAN8	1.1	3.4E-03	7.1E-03	28.960	1.193	27.687	30.054	26.326	1.243	25.362	27.728
ENSG00000198399	ITSN2	1.1	5.5E-02	8.7E-02	4.858	0.280	4.553	5.104	4.388	0.304	4.038	4.584
ENSG00000198521	ZNF43	1.1	3.4E-02	5.7E-02	17.546	1.930	15.321	18.777	15.889	0.954	15.009	16.903
ENSG00000198556	ZNF789	1.1	5.5E-02	8.7E-02	6.676	0.413	6.339	7.137	5.947	0.626	5.280	6.521
ENSG00000198618	PPIAP22	1.1	8.7E-06	3.1E-05	5603.159	76.979	5535.268	5686.794	4968.637	160.997	4800.756	5121.379
ENSG00000198730	CTR9	1.1	2.0E-03	4.5E-03	55.040	1.455	53.401	56.181	50.092	1.635	48.331	51.561
ENSG00000198791	CNOT7	1.1	4.4E-04	1.1E-03	50.854	1.003	49.698	51.493	45.955	0.888	45.228	46.958
ENSG00000198815	FOXJ3	1.1	3.2E-04	8.5E-04	36.497	0.174	36.387	36.697	32.347	1.145	31.431	33.631
ENSG00000198836	OPA1	1.1	4.6E-02	7.4E-02	68.458	1.963	66.358	70.246	64.811	2.108	62.403	66.319
ENSG00000198898	CAPZA2	1.1	8.8E-03	1.7E-02	46.933	2.257	44.812	49.306	43.396	2.212	41.062	45.463
ENSG00000198901	PRC1	1.1	1.8E-02	3.3E-02	55.073	0.987	54.113	56.085	50.849	3.094	48.265	54.278
ENSG00000198919	DZIP3	1.1	1.8E-03	4.0E-03	33.723	1.295	32.477	35.062	30.193	1.185	29.179	31.496
ENSG00000203879	GDI1	1.1	2.7E-03	5.9E-03	33.716	0.166	33.569	33.897	30.174	1.876	28.012	31.377
ENSG00000204256	BRD2	1.1	9.1E-04	2.2E-03	68.128	0.162	67.971	68.295	62.222	1.084	61.510	63.470
ENSG00000204308	RNF5	1.1	2.7E-02	4.6E-02	129.426	6.731	124.021	136.966	119.064	0.660	118.651	119.826
ENSG00000204463	BAG6	1.1	1.8E-03	4.0E-03	102.350	2.638	100.495	105.370	92.919	4.672	89.022	98.099
ENSG00000204531	POU5F1	1.1	4.6E-03	9.4E-03	1134.736	13.107	1125.718	1149.771	1063.055	16.356	1045.366	1077.631
ENSG00000204564	C6orf136	1.1	9.5E-02	1.4E-01	10.170	1.363	8.599	11.036	8.960	0.406	8.508	9.293
ENSG00000204580	DDR1	1.1	1.2E-04	3.3E-04	59.935	0.710	59.154	60.542	53.069	3.354	50.634	56.895
ENSG00000204604	ZNF468	1.1	8.6E-02	1.3E-01	12.576	0.608	12.167	13.275	11.445	0.283	11.144	11.706
ENSG00000204650	LINC02210	1.1	2.7E-02	4.7E-02	7.419	0.296	7.129	7.721	6.802	0.265	6.528	7.058
ENSG00000204681	GABBR1	1.1	1.1E-01	1.5E-01	16.057	0.168	15.872	16.200	15.128	0.399	14.843	15.584
ENSG00000205423	CNEP1R1	1.1	3.2E-02	5.4E-02	10.375	0.076	10.296	10.448	9.223	0.628	8.551	9.796
ENSG00000205581	HMGN1	1.1	2.2E-04	5.9E-04	263.352	2.089	261.599	265.663	239.404	4.409	234.380	242.632
ENSG00000205758	CRYZL1	1.1	6.2E-02	9.6E-02	7.748	0.633	7.146	8.409	6.970	0.266	6.739	7.261
ENSG00000206503	HLA-A	1.1	1.5E-05	5.2E-05	154.478	3.750	150.214	157.263	134.755	6.675	129.772	142.339
ENSG00000212789	ST13P5	1.1	1.0E-01	1.5E-01	58.033	2.947	54.633	59.858	53.717	1.855	51.711	55.372
ENSG00000213189	BTF3L4P2	1.1	7.4E-03	1.4E-02	352.886	7.886	344.162	359.509	316.108	8.765	307.406	324.935
ENSG00000213190	MLLT11	1.1	3.8E-03	8.0E-03	52.653	0.861	52.025	53.634	47.638	2.531	44.874	49.842
ENSG00000213236	YWHAZP2	1.1	5.1E-02	8.1E-02	116.267	6.747	109.236	122.688	106.650	6.738	101.648	114.313
ENSG00000213300	HNRNPA3P6	1.1	4.5E-02	7.3E-02	920.122	28.006	888.431	941.543	875.311	18.550	858.745	895.353
ENSG00000213420	GPC2	1.1	7.2E-04	1.8E-03	34.314	1.219	33.058	35.491	30.041	0.546	29.704	30.671
ENSG00000213465	ARL2	1.1	1.3E-01	1.8E-01	50.899	2.194	49.056	53.326	47.243	6.813	41.491	54.767
ENSG00000213468	FIRRE	1.1	6.5E-02	1.0E-01	22.856	0.613	22.244	23.469	21.191	1.039	20.493	22.386
ENSG00000213514		1.1	1.2E-01	1.7E-01	4.879	0.090	4.789	4.970	4.302	0.345	4.067	4.698
ENSG00000213683		1.1	1.5E-02	2.7E-02	97.784	4.013	93.724	101.748	87.113	3.245	83.402	89.419
ENSG00000213763	ACTBP2	1.1	5.2E-02	8.3E-02	36.908	2.298	35.254	39.532	32.621	2.226	30.973	35.153
ENSG00000213995	NAXD	1.1	8.6E-02	1.3E-01	32.426	0.922	31.364	33.017	30.297	0.686	29.832	31.085
ENSG00000214013	GANC	1.1	9.0E-02	1.3E-01	3.451	0.406	3.024	3.833	3.106	0.006	3.100	3.112
ENSG00000214029	ZNF891	1.1	8.0E-02	1.2E-01	4.358	0.407	3.955	4.768	3.883	0.272	3.670	4.166
ENSG00000214063	TSPAN4	1.1	6.1E-02	9.5E-02	17.477	0.184	17.282	17.646	16.156	1.533	14.469	17.463
ENSG00000214113	LYRM4	1.1	2.4E-03	5.3E-03	14.222	0.799	13.322	14.847	12.647	0.536	12.029	12.983
ENSG00000214253	FIS1	1.1	3.6E-03	7.6E-03	84.487	2.336	82.619	87.106	75.894	2.060	73.703	77.791
ENSG00000214753	HNRNPUL2	1.1	1.6E-02	2.9E-02	42.887	0.691	42.256	43.625	39.611	0.966	38.953	40.720
ENSG00000214941	ZSWIM7	1.1	9.0E-02	1.3E-01	23.924	1.023	22.799	24.801	21.925	0.427	21.635	22.416
ENSG00000218283	MORF4L1P1	1.1	3.0E-03	6.4E-03	1128.002	29.864	1098.346	1158.069	1048.199	13.931	1032.282	1058.176
ENSG00000218426		1.1	2.0E-06	8.1E-06	3736.226	113.196	3648.351	3863.959	3265.446	75.395	3180.002	3322.623
ENSG00000218510	LINC00339	1.1	9.3E-02	1.4E-01	22.050	1.064	21.362	23.275	20.177	1.662	18.306	21.482
ENSG00000219481	NBPF1	1.1	9.0E-02	1.3E-01	5.213	0.294	4.892	5.469	4.824	0.143	4.659	4.919
ENSG00000221914	PPP2R2A	1.1	1.6E-03	3.7E-03	46.429	0.944	45.487	47.374	42.174	1.312	41.221	43.670
ENSG00000221983	UBA52	1.1	3.8E-06	1.5E-05	245.552	6.564	237.980	249.623	215.923	5.022	211.803	221.517
ENSG00000223361	FTH1P10	1.1	1.0E-02	2.0E-02	150.993	12.769	143.148	165.727	134.537	8.180	125.183	140.349
ENSG00000223959	AFG3L1P	1.1	5.7E-02	8.9E-02	3.120	0.351	2.759	3.461	2.732	0.162	2.566	2.890
ENSG00000224078	SNHG14	1.1	4.7E-03	9.5E-03	73.189	1.616	71.335	74.297	67.914	1.271	66.678	69.217
ENSG00000224631	RPS27AP16	1.1	8.8E-04	2.1E-03	3351.417	78.944	3263.574	3416.432	3084.269	111.367	2973.938	3196.644
ENSG00000224831		1.1	4.3E-02	7.0E-02	154.369	6.366	147.018	158.091	143.877	8.209	134.899	151.000
ENSG00000224837	GCSHP5	1.1	7.6E-04	1.9E-03	477.897	8.321	471.376	487.269	428.396	9.436	420.342	438.779
ENSG00000225137	DYNC1I2P1	1.1	1.4E-03	3.3E-03	79.240	2.636	76.214	81.037	69.561	2.702	67.402	72.591
ENSG00000225151	GOLGA2P7	1.1	4.3E-03	8.9E-03	13.467	0.416	13.227	13.947	11.767	0.402	11.312	12.075
ENSG00000225159	NPM1P39	1.1	2.8E-03	5.9E-03	269.445	3.209	266.978	273.073	243.067	10.934	233.172	254.806
ENSG00000225259	ST13P6	1.1	1.2E-02	2.3E-02	107.953	0.192	107.794	108.167	97.372	7.906	88.615	103.984
ENSG00000225663	MCRIP1	1.1	3.1E-03	6.6E-03	42.186	2.513	39.458	44.407	37.367	1.774	35.466	38.978
ENSG00000226121		1.1	1.0E-01	1.5E-01	22.726	0.642	22.006	23.239	21.456	0.819	20.581	22.206
ENSG00000226864	RHEBP2	1.1	4.8E-03	9.7E-03	135.819	6.717	129.975	143.158	119.590	3.515	115.534	121.742
ENSG00000227946		1.1	9.8E-02	1.4E-01	18.336	2.388	15.698	20.352	16.164	0.462	15.694	16.617
ENSG00000228158	TLE1P1	1.1	1.4E-01	1.9E-01	31.439	1.291	30.177	32.757	27.812	4.196	24.309	32.463
ENSG00000229212		1.1	1.2E-01	1.7E-01	14.021	0.358	13.673	14.388	12.516	0.857	11.715	13.419
ENSG00000229927	RHEBP1	1.1	2.3E-02	4.0E-02	105.483	5.655	101.270	111.910	94.133	0.985	93.041	94.956
ENSG00000230043	TMSB4XP6	1.1	5.7E-02	8.9E-02	942.918	68.992	888.173	1020.412	866.507	84.792	817.072	964.414
ENSG00000230204	FTH1P5	1.1	7.3E-02	1.1E-01	45.199	1.685	43.694	47.019	40.032	1.126	38.764	40.917
ENSG00000231259		1.1	6.5E-02	1.0E-01	54.195	2.920	51.039	56.803	49.888	3.308	46.123	52.325
ENSG00000232112	TMA7	1.1	2.1E-03	4.7E-03	226.607	4.504	223.164	231.703	204.928	5.434	200.891	211.106
ENSG00000232150	ST13P4	1.1	6.7E-02	1.0E-01	61.207	1.981	58.632	63.431	56.416	1.062	55.269	57.364
ENSG00000232387	SKA2P1	1.1	1.1E-01	1.6E-01	52.643	2.009	50.361	54.140	46.713	2.673	44.930	49.786
ENSG00000232472	EEF1B2P3	1.1	2.7E-02	4.6E-02	899.409	12.287	887.370	911.930	847.739	5.756	842.073	853.581
ENSG00000233270	SNRPEP4	1.1	3.2E-02	5.4E-02	370.373	16.437	351.900	383.383	333.550	15.796	319.645	350.726
ENSG00000233276	GPX1	1.1	5.0E-03	1.0E-02	288.743	6.716	281.048	293.425	263.854	22.167	249.086	289.343
ENSG00000233836		1.1	4.3E-02	6.9E-02	34.044	2.443	31.263	35.848	31.350	1.232	30.536	32.768
ENSG00000233954	UQCRHL	1.1	3.6E-02	6.0E-02	161.268	2.965	157.964	163.695	151.673	3.827	149.428	156.092
ENSG00000234518	PTGES3P1	1.1	3.2E-02	5.4E-02	469.554	6.864	464.385	477.342	437.711	11.047	426.439	448.519
ENSG00000234743	EIF5AP4	1.1	7.0E-02	1.1E-01	164.398	11.917	151.308	174.617	151.026	6.414	145.796	158.183
ENSG00000234825	XRCC6P2	1.1	9.1E-02	1.3E-01	61.076	2.093	58.750	62.807	57.109	2.687	54.012	58.824
ENSG00000235602	POU5F1P3	1.1	2.6E-04	7.0E-04	612.669	13.605	601.044	627.633	549.100	28.456	517.633	573.025
ENSG00000235776		1.1	3.1E-02	5.2E-02	58.420	4.235	53.904	62.302	51.830	2.958	49.050	54.938
ENSG00000236349	SUCLG2P2	1.1	9.9E-02	1.4E-01	34.340	0.329	34.041	34.693	31.349	2.050	29.784	33.670
ENSG00000236534	H3F3BP1	1.1	6.0E-02	9.4E-02	139.440	3.771	136.217	143.587	126.428	13.635	111.813	138.806
ENSG00000236565	HNRNPA3P5	1.1	6.0E-02	9.4E-02	283.421	12.737	270.009	295.353	266.468	15.188	253.216	283.057
ENSG00000236603	RANP1	1.1	3.3E-02	5.6E-02	493.643	21.227	478.908	517.974	463.361	16.777	444.635	477.023
ENSG00000236972	FABP5P1	1.1	1.1E-01	1.5E-01	93.597	8.050	84.340	98.953	85.202	2.612	82.617	87.839
ENSG00000237039		1.1	1.2E-01	1.7E-01	427.199	7.937	418.828	434.617	400.473	13.229	388.136	414.442
ENSG00000237264	FTH1P11	1.1	3.4E-02	5.6E-02	224.899	8.884	215.569	233.257	207.326	8.452	197.972	214.413
ENSG00000237350	CDC42P6	1.1	2.3E-02	4.0E-02	295.322	7.962	287.089	302.982	271.739	13.491	256.389	281.715
ENSG00000239246		1.1	3.9E-02	6.5E-02	120.218	9.865	108.832	126.214	107.102	9.157	97.057	114.984
ENSG00000240445	FOXO3B	1.1	1.0E-01	1.5E-01	13.432	0.421	12.969	13.793	11.857	1.535	10.542	13.544
ENSG00000240509	RPL34P18	1.1	4.8E-02	7.6E-02	133.373	4.542	128.129	136.036	119.986	2.765	116.999	122.455
ENSG00000241468	ATP5J2	1.1	3.3E-02	5.5E-02	82.595	3.975	79.305	87.012	76.660	2.548	75.044	79.597
ENSG00000242086	LINC00969	1.1	3.5E-02	5.8E-02	2.721	0.145	2.602	2.882	2.428	0.091	2.341	2.522
ENSG00000242125	SNHG3	1.1	5.0E-04	1.3E-03	76.079	1.370	74.834	77.546	68.005	0.621	67.555	68.714
ENSG00000242247	ARFGAP3	1.1	1.4E-01	2.0E-01	21.063	0.473	20.726	21.604	19.717	1.577	18.138	21.291
ENSG00000243156	MICAL3	1.1	3.0E-03	6.5E-03	4.380	0.149	4.293	4.553	3.854	0.102	3.755	3.959
ENSG00000243364	EFNA4	1.1	8.2E-02	1.2E-01	23.975	1.524	22.403	25.447	21.282	1.991	19.023	22.781
ENSG00000243759	ST13P15	1.1	4.0E-02	6.6E-02	40.050	0.642	39.380	40.661	35.742	3.553	33.364	39.827
ENSG00000243927	MRPS6	1.1	2.0E-02	3.5E-02	36.631	0.684	36.134	37.411	33.531	2.114	31.388	35.614
ENSG00000244313		1.1	4.4E-04	1.1E-03	2495.306	120.891	2355.715	2565.529	2246.528	106.068	2151.630	2361.030
ENSG00000244363	RPL7P23	1.1	1.2E-04	3.6E-04	465.823	5.020	462.875	471.619	412.170	21.629	395.045	436.476
ENSG00000244754	N4BP2L2	1.1	9.0E-03	1.7E-02	14.097	0.331	13.797	14.452	12.916	0.852	12.294	13.886
ENSG00000245910	SNHG6	1.1	3.3E-02	5.6E-02	132.036	3.256	129.758	135.765	122.920	4.938	119.637	128.598
ENSG00000245958		1.1	3.7E-02	6.2E-02	6.380	0.386	6.024	6.790	5.606	0.471	5.092	6.019
ENSG00000246067	RAB30-AS1	1.1	1.2E-01	1.7E-01	8.194	0.600	7.625	8.821	7.244	0.294	6.905	7.419
ENSG00000248578	NPM1P21	1.1	5.8E-02	9.0E-02	39.652	2.401	36.959	41.570	35.088	0.165	34.898	35.202
ENSG00000248873	SERBP1P6	1.1	4.4E-02	7.1E-02	66.848	2.362	65.041	69.520	58.301	6.277	52.308	64.450
ENSG00000251369	ZNF550	1.1	1.1E-01	1.5E-01	9.429	0.330	9.062	9.699	8.655	0.409	8.337	9.117
ENSG00000251562	MALAT1	1.1	1.3E-01	1.8E-01	18.902	1.879	16.889	20.609	16.553	2.460	14.115	19.035
ENSG00000253250	C8orf88	1.1	6.7E-02	1.0E-01	42.234	3.493	39.364	46.123	37.963	1.175	36.607	38.687
ENSG00000253341		1.1	9.2E-03	1.8E-02	154.191	10.419	146.590	166.068	138.479	3.620	134.316	140.881
ENSG00000253570		1.1	6.0E-02	9.4E-02	79.122	5.797	74.096	85.464	70.134	5.132	66.919	76.052
ENSG00000253738	OTUD6B-AS1	1.1	1.1E-01	1.6E-01	14.353	1.774	12.304	15.397	13.232	1.151	12.143	14.436
ENSG00000254598	CSNK2A3	1.1	5.1E-03	1.0E-02	206.486	5.438	201.065	211.942	189.402	5.338	183.358	193.469
ENSG00000254934	LINC00678	1.1	1.1E-01	1.7E-01	578.111	32.604	542.267	606.004	542.374	17.058	531.728	562.048
ENSG00000254986	DPP3	1.1	9.7E-02	1.4E-01	49.811	2.245	47.229	51.307	47.095	2.257	45.538	49.683
ENSG00000255529	POLR2M	1.1	5.5E-02	8.7E-02	16.254	0.460	15.954	16.784	14.925	1.772	13.389	16.864
ENSG00000255717	SNHG1	1.1	1.5E-02	2.7E-02	128.975	5.817	122.419	133.518	120.476	5.297	114.398	124.105
ENSG00000256235	SMIM3	1.1	8.5E-02	1.3E-01	14.653	1.102	13.414	15.522	12.870	2.260	10.303	14.560
ENSG00000256968	SNRPEP2	1.1	5.0E-02	8.0E-02	365.205	7.008	358.028	372.030	337.677	12.906	323.838	349.386
ENSG00000257103	LSM14A	1.1	3.3E-05	1.1E-04	155.520	1.413	154.576	157.145	137.914	5.540	132.370	143.450
ENSG00000258102	MAP1LC3B2	1.1	3.0E-02	5.1E-02	19.804	1.462	18.125	20.801	17.326	0.490	16.997	17.889
ENSG00000258289	CHURC1	1.1	1.2E-02	2.3E-02	13.424	1.798	12.024	15.452	11.742	0.081	11.684	11.835
ENSG00000258427	RBM8B	1.1	2.4E-02	4.2E-02	206.604	20.235	186.983	227.402	186.871	9.016	177.147	194.954
ENSG00000258741		1.1	1.1E-01	1.5E-01	30.498	0.752	29.877	31.334	26.813	5.517	21.926	32.796
ENSG00000259086		1.1	1.3E-01	1.9E-01	45.603	2.872	42.489	48.149	40.511	4.083	36.807	44.889
ENSG00000259623		1.1	9.9E-02	1.4E-01	8.034	0.745	7.479	8.881	7.086	0.799	6.190	7.725
ENSG00000259917	HNRNPLP2	1.1	1.1E-03	2.6E-03	128.961	4.170	125.410	133.553	114.606	4.936	110.045	119.847
ENSG00000260075		1.1	1.3E-01	1.8E-01	15.915	0.929	14.945	16.798	14.283	0.496	13.784	14.776
ENSG00000260747		1.1	6.1E-02	9.5E-02	99.999	2.402	97.952	102.643	89.024	7.336	81.588	96.256
ENSG00000263266	RPS7P1	1.1	1.6E-06	6.5E-06	3001.951	52.561	2956.895	3059.695	2619.312	92.858	2514.864	2692.527
ENSG00000264522	OTUD7B	1.1	3.5E-02	5.8E-02	8.294	0.308	7.948	8.536	7.580	0.279	7.290	7.846
ENSG00000265763	ZNF488	1.1	7.9E-02	1.2E-01	10.707	0.509	10.127	11.082	9.611	0.727	8.790	10.174
ENSG00000267041	ZNF850	1.1	3.4E-03	7.2E-03	18.045	0.283	17.755	18.320	15.920	0.622	15.313	16.513
ENSG00000267374		1.1	4.5E-02	7.2E-02	14.414	0.928	13.870	15.486	12.809	1.368	11.274	13.898
ENSG00000267742	FAM60CP	1.1	1.7E-02	3.0E-02	156.160	7.255	149.039	163.542	141.341	8.308	131.799	146.973
ENSG00000268471		1.1	1.4E-01	1.9E-01	7.663	0.860	6.778	8.495	6.775	1.088	5.952	8.009
ENSG00000272163		1.1	9.2E-02	1.4E-01	11.447	1.179	10.096	12.271	10.348	0.417	9.774	10.560
ENSG00000272325	NUDT3	1.1	3.5E-03	7.4E-03	23.285	0.126	23.141	23.377	21.275	0.739	20.449	21.873
ENSG00000273749	CYFIP1	1.1	8.0E-04	1.9E-03	40.483	0.866	39.882	41.476	36.716	1.390	35.787	38.313
ENSG00000275004	ZNF280B	1.1	6.3E-02	9.8E-02	7.886	0.456	7.363	8.197	7.128	0.455	6.731	7.625
ENSG00000277072	STAG3L2	1.1	8.3E-02	1.2E-01	7.664	0.290	7.343	7.910	6.759	0.393	6.420	7.190
ENSG00000278318	ZNF229	1.1	1.0E-01	1.5E-01	7.128	0.851	6.147	7.666	6.390	0.572	5.925	7.029
ENSG00000279377		1.1	5.0E-03	1.0E-02	76.701	3.903	72.197	79.105	67.478	6.262	62.624	74.547
ENSG00000280798	LINC00294	1.1	3.3E-02	5.6E-02	11.474	0.071	11.397	11.538	10.149	0.745	9.296	10.674
ENSG00000281649	EBLN3P	1.1	5.9E-04	1.5E-03	33.058	0.411	32.613	33.423	29.113	1.405	28.077	30.711
ENSG00000002016	RAD52	1.2	1.9E-02	3.3E-02	5.177	0.592	4.494	5.540	4.330	0.086	4.234	4.426
ENSG00000003509	NDUFAF7	1.2	4.3E-03	8.9E-03	24.912	1.694	22.956	25.920	21.694	1.015	20.841	22.816
ENSG00000004399	PLXND1	1.2	1.4E-05	4.9E-05	25.331	0.314	25.091	25.686	21.135	1.333	19.609	22.069
ENSG00000004975	DVL2	1.2	4.6E-06	1.8E-05	26.827	0.793	26.152	27.700	22.036	0.551	21.436	22.521
ENSG00000005238	FAM214B	1.2	7.2E-03	1.4E-02	3.908	0.342	3.622	4.287	3.166	0.304	2.830	3.422
ENSG00000006652	IFRD1	1.2	8.9E-05	2.6E-04	28.074	0.937	27.396	29.143	24.051	0.931	23.467	25.125

ENSG00000007237	GAS7	1.2	1.7E-04	4.8E-04	5.333	0.370	4.997	5.730	4.323	0.186	4.104	4.481
ENSG00000007264	MATK	1.2	7.0E-02	1.1E-01	8.082	0.874	7.142	8.871	7.026	0.980	5.903	7.706
ENSG00000008083	JARID2	1.2	4.2E-10	3.6E-09	255.254	7.579	247.009	261.917	208.819	3.167	205.166	210.800
ENSG00000008513	ST3GAL1	1.2	5.6E-02	8.8E-02	4.795	0.675	4.227	5.541	4.093	0.064	4.027	4.155
ENSG00000008869	HEATR5B	1.2	1.0E-04	2.9E-04	14.101	0.448	13.825	14.618	11.833	0.363	11.479	12.205
ENSG00000008988	RPS20	1.2	8.7E-09	5.7E-08	923.735	27.201	893.202	945.384	775.873	24.667	755.351	803.239
ENSG00000009780	FAM76A	1.2	3.1E-02	5.3E-02	8.630	0.535	8.131	9.194	7.470	0.718	6.710	8.138
ENSG00000010292	NCAPD2	1.2	9.8E-11	9.4E-10	153.528	1.796	151.722	155.312	124.018	2.832	121.600	127.134
ENSG00000011243	AKAP8L	1.2	2.6E-06	1.0E-05	47.573	0.563	46.933	47.993	40.007	1.883	37.884	41.478
ENSG00000013583	HEBP1	1.2	1.0E-03	2.4E-03	21.097	0.628	20.372	21.480	18.035	0.658	17.451	18.748
ENSG00000017260	ATP2C1	1.2	8.7E-10	7.0E-09	58.724	0.237	58.498	58.971	47.475	0.166	47.303	47.633
ENSG00000018869	ZNF582	1.2	1.1E-01	1.6E-01	3.269	0.487	2.716	3.634	2.736	0.281	2.460	3.022
ENSG00000020426	MNAT1	1.2	7.6E-06	2.8E-05	36.542	1.634	35.427	38.417	29.854	1.636	28.337	31.588
ENSG00000021300	PLEKHB1	1.2	6.2E-02	9.6E-02	5.049	0.214	4.814	5.232	4.365	0.348	4.091	4.757
ENSG00000026103	FAS	1.2	1.2E-01	1.7E-01	2.272	0.265	1.985	2.507	1.895	0.144	1.731	2.004
ENSG00000026297	RNASET2	1.2	1.0E-02	1.9E-02	3.032	0.170	2.928	3.228	2.513	0.042	2.465	2.540
ENSG00000031698	SARS	1.2	1.5E-09	1.1E-08	123.044	5.072	119.095	128.764	99.245	2.357	97.337	101.881
ENSG00000034510	TMSB10	1.2	1.1E-07	5.8E-07	2614.114	77.246	2525.538	2667.496	2234.306	31.309	2216.142	2270.459
ENSG00000037042	TUBG2	1.2	1.4E-02	2.6E-02	13.850	0.786	13.183	14.717	11.958	0.095	11.851	12.029
ENSG00000039068	CDH1	1.2	4.9E-09	3.4E-08	149.314	2.094	147.352	151.519	121.879	2.264	119.357	123.735
ENSG00000042317	SPATA7	1.2	9.0E-02	1.3E-01	3.933	0.913	3.378	4.986	3.409	0.082	3.314	3.458
ENSG00000047457	CP	1.2	1.4E-01	1.9E-01	1.001	0.116	0.926	1.135	0.828	0.085	0.737	0.906
ENSG00000047849	MAP4	1.2	3.1E-07	1.5E-06	70.254	1.164	69.422	71.584	60.561	1.153	59.276	61.504
ENSG00000048028	USP28	1.2	9.2E-10	7.3E-09	102.967	0.619	102.581	103.681	83.371	2.618	80.902	86.116
ENSG00000049769	PPP1R3F	1.2	2.1E-02	3.6E-02	4.180	0.364	3.773	4.473	3.362	0.128	3.214	3.441
ENSG00000049860	HEXB	1.2	1.9E-05	6.4E-05	38.833	1.130	37.993	40.118	32.357	1.045	31.290	33.379
ENSG00000050438	SLC4A8	1.2	2.4E-03	5.3E-03	1.898	0.115	1.787	2.017	1.553	0.086	1.479	1.647
ENSG00000051108	HERPUD1	1.2	2.7E-04	7.2E-04	11.496	0.473	11.115	12.026	9.329	0.688	8.700	10.080
ENSG00000056097	ZFR	1.2	3.7E-07	1.7E-06	83.426	1.383	82.304	84.972	70.633	3.331	66.977	73.494
ENSG00000058063	ATP11B	1.2	9.1E-06	3.3E-05	17.747	0.294	17.422	17.993	14.870	0.711	14.057	15.372
ENSG00000058085	LAMC2	1.2	3.9E-05	1.2E-04	13.918	0.315	13.588	14.215	11.431	0.198	11.208	11.588
ENSG00000058091	CDK14	1.2	7.3E-03	1.4E-02	36.771	0.922	35.734	37.499	29.551	1.649	27.787	31.053
ENSG00000059588	TARBP1	1.2	4.7E-04	1.2E-03	18.209	0.202	17.987	18.382	15.740	0.633	15.022	16.218
ENSG00000059915	PSD	1.2	7.0E-02	1.1E-01	1.602	0.125	1.458	1.690	1.307	0.273	0.992	1.486
ENSG00000060491	OGFR	1.2	9.1E-05	2.7E-04	17.825	1.050	17.055	19.021	14.460	0.988	13.755	15.602
ENSG00000063177	RPL18	1.2	1.6E-08	1.0E-07	389.069	17.270	372.358	406.848	325.600	9.172	315.468	333.338
ENSG00000063761	ADCK1	1.2	3.0E-02	5.1E-02	4.792	0.340	4.412	5.081	4.049	0.415	3.573	4.341
ENSG00000064547	LPAR2	1.2	2.7E-03	5.9E-03	22.575	1.056	21.407	23.463	19.374	1.276	18.047	20.592
ENSG00000065978	YBX1	1.2	1.1E-10	1.0E-09	1199.505	7.898	1190.385	1204.111	971.288	46.151	918.166	1001.521
ENSG00000066135	KDM4A	1.2	1.3E-07	6.8E-07	93.378	1.247	91.960	94.304	77.767	1.969	76.434	80.028
ENSG00000066322	ELOVL1	1.2	1.8E-04	4.9E-04	26.500	0.985	25.445	27.397	21.520	1.071	20.288	22.232
ENSG00000066422	ZBTB11	1.2	1.8E-03	4.0E-03	14.309	0.524	13.820	14.862	12.365	0.149	12.209	12.507
ENSG00000066427	ATXN3	1.2	6.6E-04	1.6E-03	4.177	0.241	3.913	4.386	3.621	0.084	3.533	3.699
ENSG00000066651	TRMT11	1.2	4.7E-05	1.5E-04	42.782	2.684	41.153	45.880	35.127	2.950	32.747	38.427
ENSG00000066855	MTFR1	1.2	3.9E-04	1.0E-03	28.176	1.547	26.650	29.743	24.364	0.687	23.743	25.118
ENSG00000066933	MYO9A	1.2	2.2E-04	6.0E-04	7.047	0.289	6.714	7.238	6.051	0.162	5.864	6.157
ENSG00000067365	METTL22	1.2	1.0E-03	2.5E-03	10.876	0.347	10.491	11.163	9.354	0.115	9.240	9.470
ENSG00000067715	SYT1	1.2	3.6E-05	1.2E-04	29.796	0.267	29.537	30.069	24.997	0.823	24.064	25.619
ENSG00000068784	SRBD1	1.2	3.2E-05	1.0E-04	27.967	1.175	26.770	29.118	23.283	0.638	22.569	23.799
ENSG00000068885	IFT80	1.2	2.8E-03	6.0E-03	10.159	0.850	9.401	11.078	8.196	1.420	6.937	9.734
ENSG00000069956	MAPK6	1.2	3.3E-07	1.6E-06	142.108	3.153	139.292	145.515	120.661	6.204	113.617	125.314
ENSG00000070371	CLTCL1	1.2	4.4E-03	9.0E-03	6.188	0.345	5.852	6.542	5.323	0.197	5.129	5.523
ENSG00000070831	CDC42	1.2	1.4E-07	7.2E-07	196.903	4.261	192.117	200.286	167.011	5.551	161.092	172.100
ENSG00000071082	RPL31	1.2	4.9E-07	2.2E-06	374.725	17.229	359.498	393.426	320.992	14.276	304.525	329.895
ENSG00000071553	ATP6AP1	1.2	1.6E-04	4.5E-04	23.999	1.235	22.944	25.357	20.532	0.830	19.592	21.164
ENSG00000072110	ACTN1	1.2	4.6E-07	2.1E-06	89.085	2.915	86.532	92.261	75.509	2.513	73.525	78.335
ENSG00000072778	ACADVL	1.2	1.1E-04	3.3E-04	45.082	1.099	44.015	46.210	39.289	2.357	37.327	41.904
ENSG00000072832	CRMP1	1.2	4.0E-05	1.3E-04	64.048	2.304	61.416	65.703	54.994	1.825	53.431	56.999
ENSG00000072840	EVC	1.2	1.6E-02	2.8E-02	6.930	1.003	6.262	8.084	5.962	0.228	5.785	6.219
ENSG00000073008	PVR	1.2	1.2E-02	2.2E-02	11.347	0.679	10.627	11.974	9.594	0.851	8.892	10.540
ENSG00000073169	SELENOO	1.2	8.5E-04	2.1E-03	17.957	0.994	17.269	19.096	14.568	1.016	13.419	15.350
ENSG00000073417	PDE8A	1.2	2.5E-02	4.3E-02	2.399	0.170	2.202	2.505	2.007	0.158	1.824	2.110
ENSG00000073605	GSDMB	1.2	4.0E-03	8.4E-03	7.330	0.136	7.174	7.423	6.117	0.449	5.786	6.628
ENSG00000074855	ANO8	1.2	1.0E-03	2.4E-03	10.770	0.252	10.497	10.995	8.912	0.482	8.389	9.339
ENSG00000074935	TUBE1	1.2	1.2E-04	3.5E-04	10.481	0.461	10.094	10.991	8.431	0.536	7.904	8.976
ENSG00000075239	ACAT1	1.2	3.7E-05	1.2E-04	25.026	0.808	24.098	25.568	20.885	1.345	19.634	22.307
ENSG00000075624	ACTB	1.2	4.7E-06	1.8E-05	3627.980	37.415	3597.403	3669.700	3080.410	177.283	2919.484	3270.446
ENSG00000075945	KIFAP3	1.2	8.6E-02	1.3E-01	3.150	0.389	2.885	3.597	2.678	0.059	2.610	2.712
ENSG00000077009	NMRK2	1.2	4.0E-04	1.0E-03	63.535	2.146	61.436	65.724	53.398	5.000	47.746	57.246
ENSG00000077044	DGKD	1.2	1.5E-03	3.3E-03	9.034	0.629	8.451	9.702	7.754	0.564	7.122	8.206
ENSG00000077312	SNRPA	1.2	6.1E-09	4.1E-08	152.376	4.999	147.246	157.232	122.258	3.965	118.389	126.313
ENSG00000077380	DYNC1I2	1.2	5.7E-07	2.6E-06	66.817	1.051	66.069	68.019	56.071	0.428	55.605	56.446
ENSG00000077458	FAM76B	1.2	2.2E-03	4.9E-03	14.330	1.416	12.722	15.394	12.218	1.177	11.239	13.525
ENSG00000077549	CAPZB	1.2	1.7E-09	1.2E-08	240.500	3.777	236.554	244.081	195.245	5.635	188.911	199.700
ENSG00000077713	SLC25A43	1.2	3.8E-02	6.3E-02	5.426	0.324	5.153	5.784	4.531	0.236	4.260	4.694
ENSG00000078237	TIGAR	1.2	1.0E-03	2.4E-03	21.567	1.617	20.211	23.356	18.199	1.227	17.414	19.613
ENSG00000078304	PPP2R5C	1.2	1.4E-07	7.1E-07	26.030	0.772	25.146	26.567	21.499	0.688	21.092	22.306
ENSG00000078403	MLLT10	1.2	2.0E-07	1.0E-06	31.004	1.826	29.314	32.942	25.004	0.985	24.237	26.114
ENSG00000078618	NRDC	1.2	1.3E-06	5.4E-06	157.776	1.942	155.560	159.180	137.164	0.789	136.342	137.915
ENSG00000081051	AFP	1.2	4.2E-02	6.9E-02	4.211	0.887	3.221	4.930	3.422	0.129	3.300	3.557
ENSG00000081154	PCNP	1.2	6.0E-05	1.9E-04	237.471	10.758	228.563	249.423	200.227	17.940	184.564	219.800
ENSG00000081177	EXD2	1.2	4.3E-06	1.6E-05	18.375	0.361	17.991	18.707	14.957	0.340	14.564	15.154
ENSG00000081913	PHLPP1	1.2	3.3E-05	1.1E-04	38.845	0.250	38.603	39.102	33.770	2.106	31.476	35.617
ENSG00000082153	BZW1	1.2	2.0E-10	1.8E-09	139.527	0.938	138.910	140.607	112.595	2.546	110.937	115.526
ENSG00000082996	RNF13	1.2	8.2E-05	2.5E-04	35.580	1.055	34.362	36.221	30.092	1.072	29.223	31.290
ENSG00000083099	LYRM2	1.2	6.8E-05	2.1E-04	10.135	0.378	9.699	10.368	8.412	0.206	8.199	8.611
ENSG00000083123	BCKDHB	1.2	1.2E-04	3.6E-04	21.804	0.415	21.340	22.142	18.028	1.678	16.112	19.236
ENSG00000083290	ULK2	1.2	1.7E-03	3.9E-03	4.358	0.080	4.265	4.406	3.623	0.247	3.371	3.865
ENSG00000083544	TDRD3	1.2	6.9E-04	1.7E-03	9.879	0.543	9.307	10.386	8.175	0.342	7.865	8.542
ENSG00000083844	ZNF264	1.2	7.0E-04	1.7E-03	5.863	0.143	5.751	6.025	4.991	0.047	4.944	5.037
ENSG00000083845	RPS5	1.2	4.1E-10	3.5E-09	509.897	30.983	477.390	539.090	409.512	13.568	396.800	423.799
ENSG00000084072	PPIE	1.2	8.0E-05	2.4E-04	14.760	0.555	14.138	15.203	12.271	0.925	11.337	13.187
ENSG00000084693	AGBL5	1.2	2.1E-05	7.0E-05	29.022	2.172	27.614	31.524	23.842	0.250	23.618	24.111
ENSG00000087206	UIMC1	1.2	1.7E-04	4.8E-04	30.434	0.137	30.323	30.587	26.111	1.281	24.660	27.086
ENSG00000087245	MMP2	1.2	1.1E-05	4.0E-05	31.233	0.699	30.495	31.885	25.318	1.618	24.070	27.145
ENSG00000087301	TXNDC16	1.2	1.2E-03	2.7E-03	18.113	0.338	17.777	18.452	15.653	0.674	15.107	16.406
ENSG00000087586	AURKA	1.2	4.8E-05	1.5E-04	114.432	2.218	112.814	116.959	98.824	0.644	98.314	99.547
ENSG00000087903	RFX2	1.2	1.1E-01	1.6E-01	1.222	0.086	1.124	1.290	1.019	0.206	0.860	1.252
ENSG00000088876	ZNF343	1.2	8.1E-02	1.2E-01	3.206	0.458	2.756	3.671	2.703	0.369	2.483	3.129
ENSG00000089009	RPL6	1.2	3.3E-10	2.9E-09	329.151	4.393	324.782	333.569	268.985	2.545	266.510	271.595
ENSG00000089063	TMEM230	1.2	2.1E-05	7.0E-05	85.972	1.852	84.574	88.072	74.722	1.486	73.064	75.933
ENSG00000089123	TASP1	1.2	3.4E-02	5.7E-02	11.883	0.829	10.964	12.572	10.331	1.136	9.040	11.180
ENSG00000089199	CHGB	1.2	4.0E-02	6.6E-02	3.972	0.204	3.812	4.201	3.324	0.387	2.957	3.729
ENSG00000089248	ERP29	1.2	3.9E-06	1.5E-05	159.999	3.968	155.425	162.530	137.956	3.055	134.550	140.454
ENSG00000089351	GRAMD1A	1.2	5.3E-05	1.7E-04	46.196	0.750	45.370	46.835	39.333	3.352	35.592	42.065
ENSG00000089597	GANAB	1.2	1.7E-06	7.0E-06	322.509	3.618	320.154	326.675	280.933	9.799	270.693	290.221
ENSG00000089847	ANKRD24	1.2	3.7E-02	6.0E-02	7.541	0.947	6.451	8.163	6.439	0.601	5.876	7.072
ENSG00000090263	MRPS33	1.2	1.2E-05	4.2E-05	21.894	0.705	21.296	22.671	17.855	0.719	17.026	18.302
ENSG00000090861	AARS	1.2	2.6E-07	1.2E-06	262.225	2.982	260.096	265.632	226.237	6.992	218.166	230.464
ENSG00000091039	OSBPL8	1.2	4.8E-05	1.5E-04	52.250	3.094	48.698	54.352	45.105	2.034	43.245	47.277
ENSG00000091129	NRCAM	1.2	4.9E-02	7.8E-02	2.577	0.139	2.449	2.725	2.216	0.167	2.031	2.353
ENSG00000091640	SPAG7	1.2	2.3E-05	7.5E-05	69.997	3.207	66.297	71.968	59.536	2.001	57.237	60.884
ENSG00000092068	SLC7A8	1.2	1.9E-07	9.4E-07	116.328	2.523	113.540	118.453	97.856	4.199	93.102	101.060
ENSG00000092820	EZR	1.2	7.9E-09	5.2E-08	309.026	3.331	305.234	311.476	258.330	1.006	257.600	259.478
ENSG00000092841	MYL6	1.2	2.0E-09	1.5E-08	344.785	4.317	341.271	349.604	286.587	6.664	279.367	292.500
ENSG00000092871	RFFL	1.2	1.5E-04	4.2E-04	8.569	0.166	8.377	8.677	7.074	0.037	7.040	7.113
ENSG00000092931	MFSD11	1.2	2.3E-02	4.0E-02	4.928	0.119	4.792	5.015	4.277	0.242	4.029	4.513
ENSG00000094916	CBX5	1.2	6.6E-10	5.5E-09	117.597	1.752	115.579	118.736	96.982	1.481	95.880	98.673
ENSG00000095059	DHPS	1.2	7.4E-05	2.2E-04	40.015	1.638	38.933	41.899	33.106	1.597	31.692	34.839
ENSG00000096433	ITPR3	1.2	1.5E-03	3.3E-03	30.783	0.345	30.441	31.131	26.071	1.279	24.612	26.993
ENSG00000099365	STX18	1.2	1.1E-02	2.1E-02	5.085	0.479	4.723	5.629	4.214	0.148	4.061	4.356
ENSG00000099622	CIRBP	1.2	1.5E-07	7.8E-07	56.936	1.877	54.948	58.678	47.100	2.083	45.149	49.294
ENSG00000099849	RASSF7	1.2	6.5E-03	1.3E-02	17.398	1.092	16.154	18.201	14.816	1.051	13.621	15.595
ENSG00000099968	BCL2L13	1.2	1.9E-05	6.5E-05	28.754	0.780	28.263	29.653	24.749	0.205	24.533	24.940
ENSG00000100116	GCAT	1.2	8.9E-03	1.7E-02	21.875	1.418	20.599	23.402	18.831	1.634	17.063	20.286
ENSG00000100196	KDELR3	1.2	2.3E-03	5.1E-03	15.052	0.155	14.963	15.231	12.186	0.869	11.593	13.192
ENSG00000100242	SUN2	1.2	3.9E-03	8.1E-03	8.263	0.750	7.494	8.991	6.931	0.490	6.451	7.432
ENSG00000100271	TTLL1	1.2	6.3E-02	9.8E-02	11.085	0.612	10.394	11.561	9.526	0.335	9.164	9.824
ENSG00000100284	TOM1	1.2	9.0E-03	1.7E-02	8.697	0.514	8.259	9.262	7.402	0.832	6.876	8.362
ENSG00000100321	SYNGR1	1.2	2.0E-03	4.4E-03	11.072	1.077	9.836	11.813	9.405	0.149	9.281	9.571
ENSG00000100330	MTMR3	1.2	1.7E-06	7.0E-06	13.465	0.573	12.965	14.090	10.860	0.331	10.495	11.140
ENSG00000100441	KHNYN	1.2	4.9E-06	1.9E-05	20.666	0.722	20.135	21.487	17.188	0.155	17.027	17.331
ENSG00000100445	SDR39U1	1.2	8.7E-04	2.1E-03	24.198	1.501	22.523	25.418	20.308	1.717	19.112	22.275
ENSG00000100522	GNPNAT1	1.2	2.5E-08	1.5E-07	114.980	0.856	114.000	115.587	92.995	5.374	86.889	97.005
ENSG00000100852	ARHGAP5	1.2	5.7E-05	1.8E-04	30.247	1.289	28.780	31.195	26.237	0.888	25.455	27.218
ENSG00000100889	PCK2	1.2	9.3E-05	2.7E-04	83.797	1.777	81.938	85.480	72.866	5.461	67.793	78.647
ENSG00000100982	PCIF1	1.2	3.6E-05	1.1E-04	25.557	0.181	25.354	25.701	20.930	0.974	19.831	21.689
ENSG00000101004	NINL	1.2	2.0E-04	5.5E-04	25.784	1.705	23.815	26.790	22.281	0.962	21.274	23.190
ENSG00000101134	DOK5	1.2	2.4E-02	4.1E-02	11.481	0.966	10.375	12.155	9.662	0.979	8.536	10.312
ENSG00000101189	MRGBP	1.2	6.7E-05	2.0E-04	29.077	2.022	26.752	30.421	23.833	0.125	23.710	23.959
ENSG00000101224	CDC25B	1.2	2.2E-04	5.9E-04	18.333	1.068	17.403	19.499	15.299	0.120	15.186	15.425
ENSG00000101337	TM9SF4	1.2	2.1E-05	7.2E-05	41.685	1.194	40.348	42.642	35.436	1.629	33.587	36.661
ENSG00000101444	AHCY	1.2	7.4E-08	4.0E-07	344.226	14.047	328.739	356.144	291.627	8.332	284.327	300.704
ENSG00000101546	RBFA	1.2	2.5E-03	5.5E-03	16.297	0.681	15.644	17.002	13.796	0.381	13.372	14.111
ENSG00000101844	ATG4A	1.2	1.3E-01	1.9E-01	4.001	0.143	3.838	4.104	3.446	0.446	2.978	3.866
ENSG00000101856	PGRMC1	1.2	4.4E-09	3.1E-08	627.054	13.051	612.314	637.137	520.274	11.433	510.062	532.625
ENSG00000101882	NKAP	1.2	2.7E-03	5.8E-03	9.313	0.765	8.701	10.171	8.042	0.301	7.704	8.282
ENSG00000101997	CCDC22	1.2	2.1E-02	3.7E-02	13.224	1.177	11.935	14.241	11.202	1.561	9.438	12.405
ENSG00000102309	PIN4	1.2	2.3E-04	6.3E-04	27.505	0.667	26.790	28.111	23.247	1.174	21.894	23.995
ENSG00000102401	ARMCX3	1.2	2.6E-04	7.1E-04	25.871	0.785	25.306	26.767	21.932	1.326	20.530	23.166
ENSG00000102781	KATNAL1	1.2	7.6E-04	1.9E-03	7.760	0.667	6.989	8.158	6.377	0.360	5.973	6.661
ENSG00000102796	DHRS12	1.2	2.1E-02	3.7E-02	6.314	0.961	5.572	7.400	5.235	0.277	4.952	5.504
ENSG00000102882	MAPK3	1.2	5.1E-04	1.3E-03	30.632	1.579	29.322	32.384	26.158	2.158	24.138	28.432
ENSG00000102984	ZNF821	1.2	6.6E-02	1.0E-01	7.271	1.448	6.107	8.893	6.235	0.508	5.892	6.819
ENSG00000103222	ABCC1	1.2	4.2E-08	2.4E-07	49.694	2.407	47.527	52.285	40.129	1.808	38.412	42.016
ENSG00000103381	CPPED1	1.2	2.0E-03	4.5E-03	7.484	0.635	6.842	8.111	6.182	0.055	6.140	6.245
ENSG00000103510	KAT8	1.2	1.3E-04	3.8E-04	28.980	1.259	27.624	30.112	24.651	0.375	24.366	25.076
ENSG00000103740	ACSBG1	1.2	6.7E-02	1.0E-01	1.827	0.184	1.707	2.039	1.513	0.243	1.332	1.789
ENSG00000103995	CEP152	1.2	7.8E-04	1.9E-03	10.400	0.806	9.486	11.009	8.857	0.016	8.847	8.876
ENSG00000104343	UBE2W	1.2	9.6E-04	2.3E-03	11.840	0.160	11.678	11.998	10.245	0.645	9.826	10.988
ENSG00000104368	PLAT	1.2	2.9E-02	4.9E-02	3.154	0.500	2.578	3.475	2.539	0.210	2.322	2.740
ENSG00000104408	EIF3E	1.2	4.7E-08	2.6E-07	297.834	1.466	296.180	298.971	250.991	13.683	242.364	266.780
ENSG00000105048	TNNT1	1.2	3.4E-05	1.1E-04	60.263	4.384	55.241	63.328	49.275	3.496	45.619	52.585
ENSG00000105135	ILVBL	1.2	3.0E-05	9.9E-05	21.858	0.931	20.993	22.843	17.953	0.876	17.277	18.943
ENSG00000105143	SLC1A6	1.2	2.7E-03	5.7E-03	5.265	0.412	4.845	5.668	4.369	0.201	4.137	4.494
ENSG00000105227	PRX	1.2	1.8E-02	3.2E-02	7.253	0.355	6.980	7.655	6.303	0.534	5.886	6.904
ENSG00000105270	CLIP3	1.2	2.7E-03	5.9E-03	11.708	0.716	10.885	12.178	9.455	0.681	8.777	10.158
ENSG00000105372	RPS19	1.2	1.4E-07	7.1E-07	704.197	38.037	661.122	733.167	597.242	6.994	591.702	605.101
ENSG00000105613	MAST1	1.2	4.5E-03	9.2E-03	6.559	0.702	5.768	7.106	5.400	0.170	5.281	5.595
ENSG00000105617	LENG1	1.2	1.2E-01	1.8E-01	8.158	0.578	7.607	8.760	7.065	0.735	6.473	7.881
ENSG00000105639	JAK3	1.2	1.1E-02	2.1E-02	4.055	0.452	3.777	4.576	3.372	0.219	3.182	3.611
ENSG00000105640	RPL18A	1.2	8.3E-08	4.5E-07	131.043	6.693	123.528	136.364	107.626	5.653	103.307	114.025
ENSG00000105656	ELL	1.2	2.4E-03	5.3E-03	9.999	0.673	9.231	10.490	8.302	0.468	7.850	8.785
ENSG00000105701	FKBP8	1.2	5.3E-07	2.4E-06	230.123	10.220	218.327	236.337	193.337	7.278	185.561	199.985
ENSG00000105750	ZNF85	1.2	6.8E-03	1.3E-02	14.481	2.609	11.573	16.616	12.240	0.414	11.782	12.588
ENSG00000105792	CFAP69	1.2	5.5E-02	8.7E-02	2.071	0.095	1.976	2.167	1.772	0.190	1.559	1.923
ENSG00000105829	BET1	1.2	4.6E-05	1.5E-04	24.354	0.890	23.604	25.337	20.360	0.945	19.517	21.382
ENSG00000105856	HBP1	1.2	9.9E-06	3.5E-05	26.492	0.660	26.082	27.244	22.137	1.378	20.805	23.556
ENSG00000106025	TSPAN12	1.2	1.5E-04	4.2E-04	18.219	0.500	17.919	18.796	14.746	0.247	14.527	15.014
ENSG00000106052	TAX1BP1	1.2	1.3E-05	4.7E-05	49.266	1.588	47.559	50.700	42.785	1.944	40.976	44.840
ENSG00000106086	PLEKHA8	1.2	3.2E-07	1.5E-06	31.240	1.042	30.557	32.440	26.103	0.945	25.500	27.192
ENSG00000106348	IMPDH1	1.2	1.4E-03	3.2E-03	26.702	1.015	25.568	27.523	23.066	1.368	22.105	24.632
ENSG00000106400	ZNHIT1	1.2	9.7E-06	3.5E-05	21.988	0.878	21.004	22.690	17.996	0.283	17.670	18.167
ENSG00000106538	RARRES2	1.2	2.6E-07	1.3E-06	94.818	2.681	92.521	97.763	76.584	3.023	73.880	79.847
ENSG00000106554	CHCHD3	1.2	2.1E-05	7.1E-05	99.958	2.565	98.143	102.892	86.888	2.727	83.750	88.536
ENSG00000106615	RHEB	1.2	1.5E-05	5.2E-05	58.438	0.873	57.458	59.134	49.905	0.459	49.446	50.364
ENSG00000106628	POLD2	1.2	5.5E-07	2.5E-06	92.675	0.540	92.233	93.278	77.688	3.246	74.022	80.170
ENSG00000106733	NMRK1	1.2	5.4E-03	1.1E-02	7.254	0.441	6.972	7.762	5.931	0.441	5.638	6.439
ENSG00000107020	PLGRKT	1.2	8.3E-03	1.6E-02	29.667	2.865	26.380	31.635	25.315	1.614	23.564	26.744
ENSG00000107105	ELAVL2	1.2	6.0E-04	1.5E-03	18.804	0.138	18.672	18.948	15.875	1.230	14.830	17.230
ENSG00000107185	RGP1	1.2	2.1E-04	5.9E-04	23.225	0.895	22.254	24.018	20.233	0.540	19.611	20.581
ENSG00000107929	LARP4B	1.2	3.9E-06	1.5E-05	20.379	0.930	19.528	21.371	17.363	0.193	17.162	17.547
ENSG00000108175	ZMIZ1	1.2	3.2E-06	1.3E-05	17.206	0.590	16.628	17.807	13.974	0.630	13.579	14.700
ENSG00000108298	RPL19	1.2	8.1E-09	5.4E-08	1233.010	34.983	1192.637	1254.330	1038.155	37.061	1002.436	1076.425
ENSG00000108406	DHX40	1.2	1.4E-07	7.1E-07	41.111	0.511	40.587	41.608	33.218	1.265	31.789	34.195
ENSG00000108474	PIGL	1.2	7.4E-04	1.8E-03	7.729	0.403	7.269	8.016	6.300	0.708	5.588	7.005
ENSG00000108515	ENO3	1.2	8.9E-02	1.3E-01	4.766	0.459	4.472	5.295	4.108	0.542	3.792	4.734
ENSG00000108588	CCDC47	1.2	2.6E-06	1.1E-05	89.927	1.902	87.760	91.323	76.558	1.682	74.774	78.114
ENSG00000108641	BRD1	1.2	4.8E-04	1.2E-03	12.733	0.761	12.031	13.542	10.260	0.392	9.915	10.686
ENSG00000108953	YWHAE	1.2	1.8E-08	1.1E-07	833.788	22.709	814.368	858.758	711.799	19.025	697.188	733.311
ENSG00000109062	SLC9A3R1	1.2	1.5E-05	5.0E-05	104.584	4.224	100.610	109.020	86.852	5.778	80.675	92.126
ENSG00000109065	NAT9	1.2	3.4E-03	7.2E-03	18.893	0.574	18.401	19.523	16.432	1.336	14.931	17.491
ENSG00000109103	UNC119	1.2	4.1E-06	1.6E-05	28.846	0.511	28.263	29.216	24.053	0.178	23.907	24.252
ENSG00000109220	CHIC2	1.2	5.0E-02	8.0E-02	11.124	1.415	9.606	12.406	9.274	0.478	8.903	9.814
ENSG00000109323	MANBA	1.2	1.6E-05	5.6E-05	13.084	0.302	12.769	13.371	10.910	0.210	10.679	11.087
ENSG00000109390	NDUFC1	1.2	9.8E-05	2.9E-04	77.016	1.634	75.232	78.442	65.416	4.103	61.423	69.621
ENSG00000109445	ZNF330	1.2	2.2E-07	1.1E-06	80.273	2.420	78.438	83.016	64.997	2.147	62.535	66.484
ENSG00000109466	KLHL2	1.2	5.0E-03	1.0E-02	13.013	0.226	12.786	13.238	11.142	0.675	10.389	11.692
ENSG00000109572	CLCN3	1.2	1.8E-06	7.5E-06	31.208	1.456	29.976	32.816	26.081	0.720	25.533	26.897
ENSG00000109618	SEPSECS	1.2	1.8E-02	3.2E-02	5.817	0.213	5.574	5.969	5.001	0.264	4.726	5.199
ENSG00000109756	RAPGEF2	1.2	3.1E-08	1.8E-07	30.671	1.117	29.635	31.855	24.808	0.270	24.592	25.110
ENSG00000109787	KLF3	1.2	4.5E-04	1.1E-03	18.772	0.074	18.696	18.843	16.347	0.216	16.105	16.522
ENSG00000110315	RNF141	1.2	2.0E-04	5.6E-04	19.976	0.966	19.303	21.083	17.281	0.760	16.731	18.149
ENSG00000110330	BIRC2	1.2	9.0E-05	2.7E-04	36.439	0.819	35.816	37.366	30.954	2.220	28.644	33.073
ENSG00000110619	CARS	1.2	3.1E-07	1.5E-06	38.662	0.281	38.337	38.830	32.348	0.951	31.291	33.135
ENSG00000110711	AIP	1.2	4.1E-03	8.5E-03	26.195	4.230	21.762	30.187	21.392	1.700	19.505	22.803
ENSG00000111271	ACAD10	1.2	1.0E-03	2.5E-03	6.418	0.466	5.893	6.782	5.377	0.212	5.163	5.588
ENSG00000111678	C12orf57	1.2	2.5E-04	6.8E-04	76.444	5.437	70.779	81.619	64.771	2.001	62.589	66.520
ENSG00000111790	FGFR1OP2	1.2	2.7E-05	8.9E-05	21.158	2.212	18.612	22.598	17.257	0.977	16.169	18.061
ENSG00000111860	CEP85L	1.2	1.0E-02	1.9E-02	5.188	0.511	4.649	5.665	4.349	0.328	4.116	4.724
ENSG00000111897	SERINC1	1.2	2.5E-07	1.2E-06	93.596	1.839	92.102	95.650	77.256	4.180	73.419	81.711
ENSG00000111962	UST	1.2	7.9E-05	2.4E-04	41.344	1.937	39.107	42.486	33.681	1.389	32.096	34.685
ENSG00000112186	CAP2	1.2	1.3E-04	3.6E-04	19.454	0.089	19.352	19.508	16.086	0.725	15.286	16.699
ENSG00000112234	FBXL4	1.2	1.1E-04	3.1E-04	13.442	0.363	13.219	13.862	11.140	0.800	10.222	11.688
ENSG00000112511	PHF1	1.2	4.7E-04	1.2E-03	23.270	1.655	21.391	24.510	19.502	1.519	17.908	20.932
ENSG00000112624	BICRAL	1.2	4.1E-05	1.3E-04	10.818	0.854	10.263	11.802	8.699	0.162	8.567	8.880
ENSG00000112659	CUL9	1.2	7.2E-02	1.1E-01	1.863	0.133	1.775	2.016	1.618	0.240	1.380	1.859
ENSG00000112763	BTN2A1	1.2	3.2E-03	6.7E-03	11.641	0.665	11.249	12.409	9.750	0.392	9.300	10.010
ENSG00000112874	NUDT12	1.2	3.0E-05	9.8E-05	26.384	1.139	25.318	27.585	21.264	1.463	19.675	22.557
ENSG00000113318	MSH3	1.2	5.7E-05	1.8E-04	25.240	0.212	25.107	25.484	21.127	0.742	20.379	21.863
ENSG00000113319	RASGRF2	1.2	1.8E-02	3.1E-02	10.841	0.346	10.442	11.057	9.139	0.339	8.855	9.515
ENSG00000113384	GOLPH3	1.2	9.5E-06	3.4E-05	131.063	2.129	129.423	133.469	112.919	2.252	110.697	115.200
ENSG00000113391	FAM172A	1.2	3.1E-03	6.6E-03	12.384	0.524	11.810	12.836	10.700	0.314	10.353	10.963
ENSG00000113558	SKP1	1.2	2.8E-06	1.1E-05	61.985	1.022	61.070	63.088	53.924	0.867	52.923	54.446
ENSG00000113580	NR3C1	1.2	1.7E-02	3.0E-02	4.525	0.165	4.403	4.712	3.890	0.536	3.360	4.432
ENSG00000113638	TTC33	1.2	8.3E-03	1.6E-02	6.759	0.312	6.426	7.046	5.721	0.512	5.359	6.308
ENSG00000113734	BNIP1	1.2	1.8E-02	3.2E-02	12.805	0.331	12.529	13.172	10.984	0.676	10.429	11.736
ENSG00000113742	CPEB4	1.2	8.5E-03	1.6E-02	5.060	0.331	4.703	5.357	4.343	0.388	3.903	4.678
ENSG00000114391	RPL24	1.2	7.4E-10	6.0E-09	558.717	14.891	541.657	569.101	455.511	11.679	442.126	463.625
ENSG00000114416	FXR1	1.2	1.4E-10	1.3E-09	106.601	2.168	104.551	108.870	86.619	2.094	84.302	88.375
ENSG00000114450	GNB4	1.2	2.3E-08	1.4E-07	44.854	0.300	44.577	45.172	36.631	1.013	35.491	37.429
ENSG00000114735	HEMK1	1.2	1.9E-02	3.4E-02	1.726	0.257	1.573	2.022	1.459	0.150	1.288	1.565
ENSG00000114779	ABHD14B	1.2	4.2E-02	6.8E-02	5.082	0.719	4.365	5.803	4.277	0.139	4.141	4.419
ENSG00000114784	EIF1B	1.2	8.5E-06	3.1E-05	125.265	3.359	121.570	128.132	105.401	0.840	104.636	106.300
ENSG00000114854	TNNC1	1.2	1.0E-01	1.5E-01	8.161	1.020	7.088	9.118	6.803	1.303	5.860	8.289
ENSG00000115163	CENPA	1.2	8.3E-03	1.6E-02	39.427	3.189	35.860	42.237	33.484	1.038	32.685	34.658
ENSG00000115271	GCA	1.2	2.8E-06	1.1E-05	45.255	2.525	42.868	47.965	36.667	2.195	34.314	38.658
ENSG00000115339	GALNT3	1.2	3.4E-03	7.2E-03	17.057	1.062	16.140	18.220	14.421	0.941	13.357	15.143
ENSG00000115520	COQ10B	1.2	1.7E-03	3.7E-03	26.796	0.404	26.484	27.252	22.914	1.930	21.297	25.050
ENSG00000115539	PDCL3	1.2	2.0E-03	4.5E-03	42.269	2.386	40.469	44.976	36.203	2.078	34.399	38.476
ENSG00000115561	CHMP3	1.2	4.0E-03	8.3E-03	6.883	0.520	6.341	7.378	5.825	0.180	5.693	6.030
ENSG00000115652	UXS1	1.2	1.8E-04	5.1E-04	27.772	1.144	26.615	28.902	24.128	0.423	23.669	24.503
ENSG00000115750	TAF1B	1.2	1.9E-04	5.3E-04	28.092	1.918	25.882	29.320	23.256	2.783	21.541	26.467
ENSG00000115758	ODC1	1.2	2.4E-06	9.5E-06	173.432	4.947	167.734	176.632	149.254	3.123	145.661	151.313
ENSG00000115825	PRKD3	1.2	6.2E-09	4.2E-08	42.435	0.454	41.911	42.709	34.143	1.075	33.449	35.381
ENSG00000115966	ATF2	1.2	1.2E-06	5.2E-06	32.672	1.252	31.374	33.872	26.907	0.611	26.305	27.527
ENSG00000116044	NFE2L2	1.2	1.9E-04	5.2E-04	23.551	1.166	22.278	24.569	20.194	0.992	19.500	21.331
ENSG00000116251	RPL22	1.2	1.4E-07	7.2E-07	260.804	4.068	256.834	264.963	218.538	11.639	210.452	231.878
ENSG00000116329	OPRD1	1.2	1.0E-01	1.5E-01	1.843	0.109	1.718	1.911	1.601	0.177	1.495	1.805
ENSG00000116337	AMPD2	1.2	1.1E-05	3.9E-05	16.388	0.628	15.755	17.010	13.239	0.758	12.366	13.730
ENSG00000116396	KCNC4	1.2	1.4E-02	2.5E-02	1.907	0.142	1.769	2.053	1.659	0.130	1.551	1.802
ENSG00000116521	SCAMP3	1.2	4.6E-05	1.4E-04	43.329	0.209	43.139	43.553	35.709	2.050	33.463	37.480
ENSG00000116641	DOCK7	1.2	1.1E-06	4.8E-06	9.108	0.220	8.855	9.259	7.596	0.207	7.467	7.835
ENSG00000116754	SRSF11	1.2	1.3E-08	8.0E-08	112.897	4.415	107.806	115.673	94.196	2.232	92.346	96.676
ENSG00000116793	PHTF1	1.2	2.3E-05	7.6E-05	18.659	0.347	18.428	19.057	15.403	0.707	14.606	15.956
ENSG00000116885	OSCP1	1.2	1.1E-01	1.6E-01	2.608	0.332	2.387	2.990	2.126	0.134	2.004	2.269
ENSG00000116990	MYCL	1.2	5.0E-05	1.6E-04	24.653	0.382	24.377	25.090	20.661	1.351	19.175	21.816
ENSG00000117020	AKT3	1.2	1.1E-06	4.6E-06	15.324	0.624	14.743	15.984	12.352	0.743	11.557	13.028
ENSG00000117114	ADGRL2	1.2	1.9E-09	1.4E-08	86.005	0.813	85.066	86.498	69.123	2.701	67.144	72.200
ENSG00000117533	VAMP4	1.2	8.9E-03	1.7E-02	11.772	0.783	11.070	12.617	10.216	0.587	9.542	10.614
ENSG00000117614	SYF2	1.2	2.9E-04	7.7E-04	39.496	1.580	37.858	41.011	33.604	1.382	32.124	34.860
ENSG00000117859	OSBPL9	1.2	8.3E-08	4.5E-07	40.960	0.464	40.439	41.329	33.967	0.151	33.826	34.126
ENSG00000118058	KMT2A	1.2	5.3E-06	2.0E-05	19.089	1.152	17.873	20.163	16.467	0.224	16.258	16.703
ENSG00000118620	ZNF430	1.2	1.3E-03	2.9E-03	6.386	0.532	5.773	6.717	5.177	0.188	4.975	5.347
ENSG00000118922	KLF12	1.2	4.3E-03	8.9E-03	6.812	0.288	6.514	7.088	5.937	0.186	5.818	6.151
ENSG00000118960	HS1BP3	1.2	1.4E-02	2.5E-02	3.925	0.307	3.655	4.260	3.357	0.214	3.115	3.523
ENSG00000119401	TRIM32	1.2	4.0E-04	1.0E-03	20.438	0.794	19.804	21.328	17.296	0.696	16.739	18.076
ENSG00000119522	DENND1A	1.2	1.6E-03	3.6E-03	10.923	0.754	10.065	11.483	9.245	1.046	8.581	10.451
ENSG00000119630	PGF	1.2	6.9E-02	1.1E-01	1.604	0.121	1.476	1.715	1.298	0.167	1.121	1.453
ENSG00000119888	EPCAM	1.2	1.1E-10	1.0E-09	323.921	6.567	316.520	329.051	261.512	3.011	259.025	264.860
ENSG00000119900	OSGEPL1	1.2	5.1E-04	1.3E-03	11.986	0.781	11.085	12.464	10.100	0.236	9.941	10.371
ENSG00000120156	TEK	1.2	5.4E-04	1.4E-03	34.965	1.107	33.729	35.865	30.242	2.094	28.897	32.654
ENSG00000120159	CAAP1	1.2	4.1E-04	1.1E-03	52.841	4.010	49.726	57.365	44.874	3.290	41.171	47.459
ENSG00000120254	MTHFD1L	1.2	2.5E-07	1.2E-06	86.769	2.715	83.653	88.625	72.008	2.035	70.609	74.342
ENSG00000120318	ARAP3	1.2	5.4E-05	1.7E-04	40.782	1.114	39.512	41.590	34.811	2.412	32.066	36.591
ENSG00000120526	NUDCD1	1.2	1.4E-07	7.2E-07	91.324	1.694	89.369	92.352	75.082	3.179	72.620	78.671
ENSG00000120616	EPC1	1.2	1.7E-04	4.7E-04	7.730	0.150	7.563	7.853	6.429	0.283	6.138	6.702
ENSG00000120686	UFM1	1.2	1.3E-05	4.4E-05	42.510	1.127	41.564	43.756	34.822	1.325	33.359	35.942
ENSG00000120690	ELF1	1.2	3.4E-04	8.9E-04	13.643	0.568	13.032	14.154	11.127	0.640	10.670	11.858
ENSG00000120756	PLS1	1.2	1.7E-04	4.6E-04	33.399	0.792	32.562	34.137	28.488	1.503	26.984	30.001
ENSG00000120868	APAF1	1.2	1.6E-04	4.6E-04	21.939	1.162	20.623	22.825	18.525	1.307	17.088	19.643
ENSG00000120915	EPHX2	1.2	1.7E-03	3.9E-03	7.688	0.467	7.149	7.979	6.275	0.375	5.884	6.632
ENSG00000120925	RNF170	1.2	3.9E-03	8.1E-03	8.709	1.196	7.877	10.080	7.249	0.236	7.004	7.475
ENSG00000121089	NACA3P	1.2	9.5E-03	1.8E-02	57.447	2.993	54.674	60.619	48.393	5.333	42.575	53.048
ENSG00000121775	TMEM39B	1.2	3.0E-03	6.4E-03	16.031	0.266	15.782	16.311	13.177	1.867	11.987	15.329
ENSG00000121988	ZRANB3	1.2	6.9E-04	1.7E-03	7.819	0.018	7.800	7.835	6.619	0.285	6.293	6.822
ENSG00000122008	POLK	1.2	1.3E-03	3.1E-03	4.784	0.497	4.255	5.241	3.892	0.167	3.706	4.029
ENSG00000122203	KIAA1191	1.2	6.7E-07	3.0E-06	102.556	2.566	99.933	105.060	86.337	2.224	84.444	88.786
ENSG00000122335	SERAC1	1.2	4.3E-03	8.8E-03	6.214	0.298	6.000	6.554	5.349	0.188	5.133	5.479
ENSG00000122417	ODF2L	1.2	4.9E-03	1.0E-02	9.073	1.035	7.903	9.867	7.878	0.506	7.560	8.461
ENSG00000122507	BBS9	1.2	1.1E-04	3.3E-04	24.632	0.986	23.824	25.730	20.881	1.285	19.656	22.218
ENSG00000122545	SEPT7	1.2	5.8E-07	2.6E-06	67.031	4.351	63.372	71.842	56.700	2.029	55.073	58.974
ENSG00000122557	HERPUD2	1.2	2.4E-04	6.6E-04	22.515	0.984	21.595	23.553	18.634	0.427	18.160	18.988
ENSG00000123094	RASSF8	1.2	6.2E-05	1.9E-04	21.034	1.023	19.853	21.667	17.956	0.445	17.486	18.371
ENSG00000123106	CCDC91	1.2	7.8E-03	1.5E-02	11.062	1.183	9.710	11.907	9.519	0.546	8.994	10.085
ENSG00000123130	ACOT9	1.2	5.9E-04	1.5E-03	27.216	0.770	26.603	28.080	23.548	0.587	23.153	24.223
ENSG00000123352	SPATS2	1.2	8.0E-04	2.0E-03	17.155	0.669	16.548	17.872	14.906	0.514	14.314	15.235
ENSG00000123562	MORF4L2	1.2	4.1E-10	3.5E-09	441.495	3.350	438.374	445.035	360.402	5.948	354.140	365.976
ENSG00000123595	RAB9A	1.2	9.0E-05	2.7E-04	65.407	2.198	63.884	67.928	54.833	3.409	52.042	58.632
ENSG00000123870	ZNF137P	1.2	9.9E-02	1.5E-01	5.969	1.112	4.695	6.746	4.929	0.291	4.613	5.186
ENSG00000124145	SDC4	1.2	6.8E-06	2.5E-05	108.110	4.070	103.552	111.381	91.684	1.043	90.506	92.491
ENSG00000124171	PARD6B	1.2	5.9E-03	1.2E-02	7.650	0.068	7.610	7.728	6.361	0.527	5.973	6.961
ENSG00000124613	ZNF391	1.2	5.7E-04	1.4E-03	11.012	0.934	10.006	11.854	9.019	0.276	8.754	9.305
ENSG00000124614	RPS10	1.2	5.6E-03	1.1E-02	23.910	1.110	22.673	24.820	20.213	0.401	19.751	20.457
ENSG00000124635	HIST1H2BJ	1.2	9.8E-02	1.4E-01	11.595	2.404	8.885	13.470	9.676	1.235	8.602	11.025
ENSG00000124767	GLO1	1.2	1.1E-10	1.0E-09	534.504	6.702	526.793	538.925	434.669	10.194	423.813	444.128
ENSG00000125246	CLYBL	1.2	7.8E-03	1.5E-02	4.078	0.400	3.802	4.537	3.396	0.169	3.245	3.579
ENSG00000125458	NT5C	1.2	2.2E-04	5.9E-04	52.443	2.545	49.517	54.141	43.386	4.091	40.635	48.087
ENSG00000125741	OPA3	1.2	6.6E-04	1.6E-03	8.494	0.412	8.219	8.969	7.099	0.448	6.692	7.579
ENSG00000125743	SNRPD2	1.2	1.8E-06	7.6E-06	434.293	12.908	423.908	448.744	375.010	11.714	363.378	386.804
ENSG00000125814	NAPB	1.2	4.6E-03	9.3E-03	11.058	0.399	10.705	11.491	9.403	1.094	8.155	10.195
ENSG00000125868	DSTN	1.2	7.7E-07	3.4E-06	189.414	3.616	187.125	193.582	163.006	5.965	156.386	167.963
ENSG00000125875	TBC1D20	1.2	3.2E-04	8.5E-04	14.277	0.309	14.002	14.612	12.116	0.351	11.740	12.433
ENSG00000125977	EIF2S2	1.2	2.8E-09	2.0E-08	287.576	2.384	284.823	288.978	235.410	5.688	229.157	240.308
ENSG00000125991	ERGIC3	1.2	5.6E-05	1.7E-04	47.639	1.858	45.906	49.601	41.237	0.852	40.336	42.029
ENSG00000125995	ROMO1	1.2	5.4E-04	1.4E-03	188.954	7.421	180.468	194.228	162.167	9.537	155.408	173.075
ENSG00000126012	KDM5C	1.2	9.4E-10	7.5E-09	62.553	0.558	61.974	63.087	50.234	1.564	48.451	51.376
ENSG00000126016	AMOT	1.2	3.1E-05	1.0E-04	8.829	0.223	8.646	9.077	7.164	0.068	7.094	7.229
ENSG00000126091	ST3GAL3	1.2	1.9E-02	3.3E-02	4.787	0.351	4.416	5.115	3.932	0.458	3.530	4.430
ENSG00000126214	KLC1	1.2	9.2E-06	3.3E-05	5.916	0.212	5.717	6.138	4.771	0.216	4.586	5.008
ENSG00000126247	CAPNS1	1.2	3.0E-06	1.2E-05	120.409	0.764	119.645	121.174	103.910	3.567	100.167	107.271
ENSG00000126261	UBA2	1.2	1.9E-08	1.1E-07	205.364	2.407	202.989	207.802	172.485	2.154	170.049	174.139
ENSG00000126432	PRDX5	1.2	3.6E-06	1.4E-05	524.586	3.228	522.505	528.305	449.501	25.948	422.193	473.832
ENSG00000126457	PRMT1	1.2	4.5E-09	3.1E-08	276.420	0.574	275.775	276.873	231.289	4.134	227.276	235.535
ENSG00000126777	KTN1	1.2	3.1E-04	8.2E-04	47.841	4.084	43.139	50.492	41.404	2.490	39.710	44.263
ENSG00000126804	ZBTB1	1.2	8.5E-04	2.1E-03	22.065	0.720	21.503	22.877	19.210	1.315	17.707	20.145
ENSG00000126858	RHOT1	1.2	1.6E-05	5.5E-05	31.991	0.093	31.902	32.088	27.124	1.480	25.687	28.643
ENSG00000127311	HELB	1.2	6.0E-03	1.2E-02	4.048	0.369	3.812	4.473	3.427	0.408	3.190	3.898
ENSG00000127337	YEATS4	1.2	1.8E-04	5.1E-04	79.186	0.444	78.887	79.696	67.691	4.141	62.930	70.460
ENSG00000127483	HP1BP3	1.2	1.8E-05	6.2E-05	53.216	2.503	50.337	54.877	45.953	0.974	45.340	47.076
ENSG00000127922	SEM1	1.2	5.0E-06	1.9E-05	32.163	1.905	29.970	33.403	27.490	1.151	26.715	28.813
ENSG00000127995	CASD1	1.2	4.2E-04	1.1E-03	25.914	1.018	24.832	26.853	22.078	1.980	19.843	23.613
ENSG00000128050	PAICS	1.2	4.5E-12	5.7E-11	438.088	4.754	433.096	442.563	351.661	6.435	344.679	357.354
ENSG00000128272	ATF4	1.2	8.6E-10	6.8E-09	555.288	0.362	555.043	555.703	456.604	12.480	442.022	464.381
ENSG00000128563	PRKRIP1	1.2	7.4E-05	2.2E-04	19.511	1.145	18.197	20.290	15.803	0.245	15.534	16.012
ENSG00000128590	DNAJB9	1.2	1.0E-03	2.4E-03	17.571	0.550	17.090	18.171	14.479	0.929	13.555	15.411
ENSG00000128692	EIF2S2P4	1.2	2.5E-08	1.5E-07	512.180	23.235	487.203	533.153	418.611	11.252	411.189	431.557
ENSG00000128923	MINDY2	1.2	4.8E-07	2.2E-06	21.063	1.240	19.918	22.380	17.038	0.660	16.287	17.526
ENSG00000129235	TXNDC17	1.2	2.7E-08	1.6E-07	78.116	2.146	75.819	80.070	63.117	1.730	61.119	64.122
ENSG00000129472	RAB28	1.2	2.6E-03	5.7E-03	19.498	0.337	19.112	19.733	16.653	1.061	15.563	17.681
ENSG00000129473	BCL2L2	1.2	7.0E-04	1.7E-03	17.889	0.942	16.802	18.460	14.935	0.782	14.063	15.573
ENSG00000129484	PARP2	1.2	2.0E-07	9.9E-07	39.615	1.474	38.399	41.254	31.769	0.941	30.686	32.393
ENSG00000129657	SEC14L1	1.2	2.8E-06	1.1E-05	21.683	1.203	20.556	22.949	18.264	0.472	17.985	18.810
ENSG00000129757	CDKN1C	1.2	1.3E-02	2.4E-02	9.291	1.610	7.643	10.859	7.511	0.928	6.536	8.384
ENSG00000130024	PHF10	1.2	8.0E-06	2.9E-05	34.977	2.187	32.451	36.240	29.370	1.102	28.098	30.013
ENSG00000130255	RPL36	1.2	2.3E-07	1.1E-06	496.740	8.938	486.824	504.176	424.918	17.766	406.772	442.277
ENSG00000130304	SLC27A1	1.2	2.4E-02	4.1E-02	3.461	0.212	3.266	3.686	2.950	0.260	2.659	3.157
ENSG00000130313	PGLS	1.2	1.8E-03	4.0E-03	31.714	1.314	30.215	32.669	26.755	4.011	23.257	31.133
ENSG00000130429	ARPC1B	1.2	7.3E-02	1.1E-01	4.831	0.684	4.228	5.575	4.175	0.211	3.932	4.311
ENSG00000130520	LSM4	1.2	9.1E-08	4.9E-07	191.105	7.402	183.637	198.438	159.017	7.800	150.756	166.255
ENSG00000130545	CRB3	1.2	6.3E-04	1.6E-03	26.328	0.985	25.208	27.062	21.448	0.794	20.883	22.356
ENSG00000130766	SESN2	1.2	6.8E-04	1.7E-03	34.130	1.492	32.407	35.021	29.262	1.281	28.053	30.605
ENSG00000130787	HIP1R	1.2	6.1E-04	1.5E-03	5.892	0.351	5.672	6.297	4.747	0.093	4.642	4.819
ENSG00000130827	PLXNA3	1.2	3.8E-02	6.3E-02	2.420	0.200	2.188	2.562	2.110	0.062	2.041	2.158
ENSG00000131016	AKAP12	1.2	7.4E-09	4.9E-08	81.329	3.039	79.020	84.772	67.068	1.594	65.617	68.774
ENSG00000131143	COX4I1	1.2	1.7E-06	6.9E-06	126.687	3.002	123.286	128.935	109.844	0.331	109.500	110.159
ENSG00000131469	RPL27	1.2	5.4E-09	3.7E-08	919.084	11.112	906.284	926.245	776.797	11.512	767.940	789.810
ENSG00000131558	EXOC4	1.2	3.2E-07	1.5E-06	25.706	0.995	24.769	26.750	21.046	1.038	20.099	22.156
ENSG00000131697	NPHP4	1.2	3.5E-02	5.8E-02	2.397	0.281	2.073	2.579	2.047	0.222	1.879	2.299
ENSG00000131941	RHPN2	1.2	3.0E-05	9.8E-05	28.242	1.160	26.968	29.238	23.795	0.896	22.831	24.603
ENSG00000131979	GCH1	1.2	2.4E-03	5.3E-03	14.080	1.233	12.658	14.840	11.630	0.750	10.767	12.118
ENSG00000132003	ZSWIM4	1.2	3.5E-04	9.2E-04	20.552	0.816	19.650	21.239	17.129	0.811	16.539	18.054
ENSG00000132109	TRIM21	1.2	1.9E-03	4.3E-03	22.584	2.003	20.964	24.823	18.554	0.730	18.026	19.387
ENSG00000132326	PER2	1.2	4.6E-02	7.4E-02	2.429	0.182	2.222	2.564	2.048	0.142	1.884	2.133
ENSG00000132535	DLG4	1.2	2.3E-03	5.0E-03	12.895	0.257	12.652	13.164	11.197	0.012	11.183	11.206
ENSG00000132680	KIAA0907	1.2	8.2E-07	3.6E-06	35.850	1.659	34.814	37.764	29.199	1.266	28.265	30.640
ENSG00000132823	OSER1	1.2	4.5E-05	1.4E-04	66.968	0.648	66.359	67.649	54.671	2.275	53.036	57.269
ENSG00000133065	SLC41A1	1.2	1.6E-06	6.8E-06	28.415	0.578	27.776	28.904	23.443	0.994	22.470	24.457
ENSG00000133069	TMCC2	1.2	1.7E-02	3.0E-02	4.422	0.315	4.167	4.774	3.671	0.337	3.377	4.038
ENSG00000133460	SLC2A11	1.2	6.2E-02	9.6E-02	2.017	0.176	1.855	2.205	1.709	0.151	1.574	1.871
ENSG00000133612	AGAP3	1.2	6.1E-04	1.5E-03	5.323	0.272	5.014	5.526	4.427	0.225	4.242	4.677
ENSG00000133710	SPINK5	1.2	1.6E-02	2.8E-02	5.143	0.276	4.966	5.461	4.209	0.615	3.594	4.825
ENSG00000133731	IMPA1	1.2	2.7E-03	5.9E-03	27.284	1.000	26.355	28.342	23.619	2.054	21.664	25.760
ENSG00000133805	AMPD3	1.2	6.7E-03	1.3E-02	3.923	0.226	3.663	4.062	3.158	0.444	2.710	3.599
ENSG00000133818	RRAS2	1.2	2.4E-09	1.7E-08	152.817	2.707	149.745	154.849	123.927	2.435	121.415	126.278
ENSG00000133858	ZFC3H1	1.2	1.8E-06	7.4E-06	30.234	1.739	29.040	32.229	25.210	0.816	24.579	26.132
ENSG00000133872	SARAF	1.2	3.0E-07	1.4E-06	135.710	1.943	134.334	137.933	115.146	1.592	114.167	116.983
ENSG00000134046	MBD2	1.2	1.3E-02	2.3E-02	8.790	0.679	8.326	9.569	7.667	0.693	7.117	8.445
ENSG00000134201	GSTM5	1.2	9.0E-02	1.3E-01	2.858	0.456	2.341	3.203	2.446	0.242	2.181	2.654
ENSG00000134285	FKBP11	1.2	5.8E-04	1.4E-03	14.945	0.901	13.917	15.600	12.552	0.645	12.082	13.287
ENSG00000134419	RPS15A	1.2	3.1E-09	2.2E-08	95.118	2.800	92.785	98.223	77.897	1.086	77.059	79.124
ENSG00000134440	NARS	1.2	3.4E-07	1.6E-06	308.315	0.106	308.228	308.433	265.014	7.256	258.685	272.933
ENSG00000134874	DZIP1	1.2	2.4E-04	6.5E-04	14.227	0.633	13.836	14.957	12.012	0.568	11.366	12.435
ENSG00000134996	OSTF1	1.2	2.7E-03	5.9E-03	30.879	1.941	28.782	32.612	25.460	1.557	23.983	27.086
ENSG00000135002	RFK	1.2	6.6E-04	1.6E-03	17.904	1.060	16.681	18.547	14.662	1.401	13.599	16.250
ENSG00000135164	DMTF1	1.2	1.7E-08	1.0E-07	41.330	0.351	40.943	41.628	33.208	2.014	30.887	34.488
ENSG00000135334	AKIRIN2	1.2	5.1E-05	1.6E-04	97.821	0.588	97.162	98.292	83.886	5.332	79.103	89.635
ENSG00000135390	ATP5G2	1.2	4.3E-08	2.4E-07	157.725	5.475	152.691	163.553	129.657	1.873	127.513	130.975
ENSG00000135441	BLOC1S1	1.2	5.1E-02	8.1E-02	18.566	0.878	17.889	19.576	16.209	2.442	13.504	18.253
ENSG00000135486	HNRNPA1	1.2	9.7E-11	9.3E-10	485.647	10.172	473.902	491.643	391.128	20.657	368.408	410.527
ENSG00000135503	ACVR1B	1.2	7.8E-03	1.5E-02	15.589	1.479	14.296	17.202	13.321	0.850	12.668	14.282
ENSG00000135540	NHSL1	1.2	7.0E-04	1.7E-03	7.308	0.281	7.134	7.632	5.982	0.236	5.753	6.225
ENSG00000135622	SEMA4F	1.2	5.1E-02	8.1E-02	3.855	0.201	3.623	3.991	3.308	0.049	3.275	3.364
ENSG00000135823	STX6	1.2	1.2E-06	5.1E-06	49.527	0.628	48.976	50.210	40.774	1.154	39.454	41.590
ENSG00000135974	C2orf69	1.2	4.1E-05	1.3E-04	20.126	0.495	19.617	20.606	16.584	0.448	16.159	17.051
ENSG00000135976	ANKRD36	1.2	1.8E-02	3.2E-02	3.764	0.268	3.577	4.071	3.252	0.339	2.897	3.572
ENSG00000136238	RAC1	1.2	4.9E-08	2.7E-07	242.808	1.356	241.316	243.964	204.223	7.080	196.135	209.295
ENSG00000136504	KAT7	1.2	4.3E-07	2.0E-06	50.838	1.447	49.494	52.370	43.346	0.773	42.462	43.890
ENSG00000136603	SKIL	1.2	3.5E-04	9.1E-04	79.367	5.962	72.672	84.103	65.246	6.262	60.419	72.321
ENSG00000136682	CBWD2	1.2	6.1E-06	2.3E-05	13.244	0.413	12.769	13.520	10.806	0.421	10.495	11.285
ENSG00000136717	BIN1	1.2	2.7E-04	7.3E-04	17.224	0.828	16.707	18.179	14.802	0.688	14.163	15.547
ENSG00000136770	DNAJC1	1.2	6.4E-04	1.6E-03	23.749	0.356	23.452	24.143	19.361	1.532	17.876	20.937
ENSG00000136802	LRRC8A	1.2	3.1E-09	2.2E-08	94.904	2.935	92.924	98.276	76.249	3.832	71.901	79.132
ENSG00000136807	CDK9	1.2	2.8E-04	7.5E-04	60.798	0.859	60.050	61.735	52.803	0.957	51.917	53.818
ENSG00000136895	GARNL3	1.2	1.6E-02	2.9E-02	2.694	0.264	2.436	2.963	2.249	0.075	2.194	2.335
ENSG00000136925	TSTD2	1.2	1.8E-06	7.3E-06	20.490	1.182	19.441	21.772	16.443	0.623	15.986	17.153
ENSG00000136935	GOLGA1	1.2	9.5E-04	2.3E-03	6.240	0.523	5.642	6.607	5.066	0.126	4.975	5.211
ENSG00000136942	RPL35	1.2	4.5E-09	3.1E-08	901.544	8.216	892.705	908.949	740.958	37.134	711.181	782.566
ENSG00000137074	APTX	1.2	8.7E-05	2.6E-04	32.129	2.002	30.621	34.400	27.043	1.238	25.840	28.314
ENSG00000137171	KLC4	1.2	3.2E-03	6.8E-03	9.756	0.089	9.699	9.859	8.432	0.203	8.289	8.665
ENSG00000137210	TMEM14B	1.2	1.3E-05	4.7E-05	32.525	0.224	32.392	32.784	27.375	0.769	26.775	28.242
ENSG00000137500	CCDC90B	1.2	6.8E-06	2.5E-05	30.957	1.565	29.830	32.743	25.406	0.255	25.112	25.564
ENSG00000137513	NARS2	1.2	2.2E-04	6.0E-04	63.596	3.101	61.262	67.115	55.221	2.381	52.806	57.566
ENSG00000137818	RPLP1	1.2	2.3E-11	2.6E-10	944.476	8.904	934.677	952.068	764.326	24.157	738.022	785.518
ENSG00000137876	RSL24D1	1.2	6.6E-10	5.4E-09	244.798	4.923	239.892	249.737	197.392	7.084	190.011	204.137
ENSG00000137970	RPL7P9	1.2	3.2E-10	2.8E-09	3074.784	36.077	3044.520	3114.708	2525.031	84.361	2427.646	2575.684
ENSG00000138069	RAB1A	1.2	1.4E-06	6.1E-06	153.892	1.898	152.361	156.015	131.684	4.974	126.843	136.781
ENSG00000138078	PREPL	1.2	1.7E-08	1.0E-07	44.184	1.524	42.426	45.114	35.863	1.712	33.902	37.059
ENSG00000138180	CEP55	1.2	7.1E-03	1.4E-02	43.022	0.729	42.232	43.669	34.804	2.128	32.350	36.147
ENSG00000138326	RPS24	1.2	1.0E-10	9.9E-10	709.281	12.821	695.571	720.974	573.796	18.705	557.832	594.376
ENSG00000138443	ABI2	1.2	6.7E-09	4.5E-08	27.397	0.534	26.780	27.718	22.606	0.486	22.046	22.919
ENSG00000138448	ITGAV	1.2	5.9E-05	1.8E-04	34.409	2.087	32.013	35.821	29.826	1.098	28.564	30.571
ENSG00000138622	HCN4	1.2	3.5E-03	7.3E-03	5.983	0.343	5.606	6.278	4.839	0.267	4.561	5.094
ENSG00000138685	FGF2	1.2	6.1E-02	9.5E-02	68.030	6.573	60.646	73.245	58.699	3.880	55.189	62.865
ENSG00000138759	FRAS1	1.2	2.2E-07	1.1E-06	58.193	1.700	56.407	59.792	50.113	0.553	49.495	50.560
ENSG00000138780	GSTCD	1.2	5.9E-04	1.5E-03	24.075	1.369	22.614	25.328	21.006	0.099	20.948	21.120
ENSG00000138829	FBN2	1.2	2.5E-06	9.9E-06	15.318	0.797	14.831	16.238	12.594	0.270	12.400	12.902
ENSG00000139133	ALG10	1.2	9.7E-03	1.8E-02	6.150	0.296	5.936	6.488	5.074	0.485	4.773	5.633
ENSG00000139154	AEBP2	1.2	4.7E-05	1.5E-04	27.879	1.155	26.996	29.186	23.677	0.727	22.928	24.380
ENSG00000139168	ZCRB1	1.2	3.4E-04	9.0E-04	71.226	0.836	70.542	72.158	61.607	2.522	58.973	64.000
ENSG00000139187	KLRG1	1.2	9.5E-02	1.4E-01	3.443	0.160	3.261	3.565	2.796	0.126	2.652	2.887
ENSG00000139350	NEDD1	1.2	1.2E-04	3.4E-04	38.444	1.729	37.109	40.397	32.860	1.371	31.348	34.022
ENSG00000139370	SLC15A4	1.2	1.8E-04	5.1E-04	18.022	0.126	17.917	18.161	15.274	0.335	14.888	15.482
ENSG00000139631	CSAD	1.2	6.9E-02	1.1E-01	2.937	0.151	2.778	3.077	2.478	0.144	2.370	2.641
ENSG00000139636	LMBR1L	1.2	1.1E-03	2.6E-03	13.078	0.314	12.815	13.426	11.185	1.043	10.328	12.346
ENSG00000139675	HNRNPA1L2	1.2	3.7E-09	2.6E-08	203.110	3.655	199.164	206.380	166.008	5.917	159.703	171.439
ENSG00000139687	RB1	1.2	3.3E-04	8.6E-04	13.996	0.667	13.288	14.613	11.574	1.148	10.256	12.352
ENSG00000139722	VPS37B	1.2	8.5E-03	1.6E-02	4.912	0.205	4.693	5.099	4.023	0.501	3.567	4.558
ENSG00000140006	WDR89	1.2	3.6E-06	1.4E-05	52.113	2.095	49.759	53.769	41.862	3.135	38.244	43.768
ENSG00000140105	WARS	1.2	4.0E-09	2.8E-08	78.170	2.479	75.475	80.352	64.215	0.608	63.527	64.680
ENSG00000140264	SERF2	1.2	1.1E-07	5.6E-07	96.728	3.528	92.798	99.622	81.020	1.771	79.698	83.031
ENSG00000140451	PIF1	1.2	1.5E-04	4.2E-04	60.302	1.034	59.240	61.306	51.747	2.497	49.219	54.213
ENSG00000140521	POLG	1.2	8.5E-05	2.5E-04	36.399	0.666	35.761	37.090	31.537	0.522	31.208	32.139
ENSG00000140941	MAP1LC3B	1.2	7.1E-06	2.6E-05	37.429	1.086	36.561	38.647	31.787	0.932	30.945	32.788
ENSG00000140988	RPS2	1.2	9.9E-07	4.3E-06	1452.258	33.275	1425.498	1489.516	1258.572	45.372	1229.060	1310.817
ENSG00000141034	GID4	1.2	1.5E-03	3.5E-03	13.023	0.641	12.524	13.746	11.137	0.511	10.548	11.449
ENSG00000141101	NOB1	1.2	1.7E-07	8.6E-07	108.887	0.641	108.468	109.625	89.358	1.109	88.259	90.478
ENSG00000141127	PRPSAP2	1.2	2.1E-06	8.7E-06	37.348	1.035	36.680	38.540	30.962	1.284	29.639	32.204
ENSG00000141298	SSH2	1.2	3.8E-03	8.0E-03	6.859	0.522	6.411	7.432	5.979	0.270	5.688	6.238
ENSG00000141378	PTRH2	1.2	1.5E-04	4.1E-04	21.105	0.955	20.254	22.138	17.717	0.523	17.232	18.271
ENSG00000141384	TAF4B	1.2	2.9E-06	1.1E-05	24.974	0.490	24.603	25.529	20.231	0.711	19.441	20.817
ENSG00000141425	RPRD1A	1.2	2.0E-07	9.8E-07	91.090	2.923	89.177	94.454	76.574	3.335	72.752	78.895
ENSG00000141552	ANAPC11	1.2	3.5E-04	9.2E-04	39.980	2.305	38.380	42.621	34.613	0.851	33.751	35.454
ENSG00000141627	DYM	1.2	2.1E-06	8.5E-06	23.973	0.183	23.848	24.182	20.008	0.533	19.526	20.580
ENSG00000142327	RNPEPL1	1.2	8.6E-02	1.3E-01	3.569	0.355	3.163	3.817	3.053	0.587	2.646	3.727
ENSG00000142687	KIAA0319L	1.2	3.7E-05	1.2E-04	26.432	1.086	25.627	27.667	22.801	0.521	22.225	23.239
ENSG00000142733	MAP3K6	1.2	1.7E-02	3.1E-02	5.111	0.284	4.825	5.393	4.292	0.170	4.176	4.487
ENSG00000142794	NBPF3	1.2	4.2E-04	1.1E-03	8.865	0.540	8.292	9.365	7.362	0.127	7.215	7.440
ENSG00000142937	RPS8	1.2	5.5E-09	3.7E-08	1066.254	25.549	1038.222	1088.231	903.105	18.924	886.856	923.882
ENSG00000142945	KIF2C	1.2	1.6E-05	5.6E-05	93.687	3.458	90.436	97.320	81.217	0.675	80.495	81.832
ENSG00000143153	ATP1B1	1.2	7.8E-07	3.4E-06	57.414	1.573	55.842	58.988	46.430	0.672	45.887	47.181
ENSG00000143156	NME7	1.2	6.2E-03	1.2E-02	11.798	0.899	10.905	12.704	10.096	0.301	9.817	10.415
ENSG00000143164	DCAF6	1.2	1.0E-03	2.4E-03	11.001	0.076	10.924	11.076	9.338	0.277	9.049	9.601
ENSG00000143222	UFC1	1.2	3.4E-05	1.1E-04	71.335	3.058	68.530	74.595	60.400	2.705	57.866	63.248
ENSG00000143228	NUF2	1.2	8.2E-04	2.0E-03	44.404	2.560	41.548	46.494	38.535	0.680	37.810	39.184
ENSG00000143256	PFDN2	1.2	1.2E-05	4.1E-05	212.018	7.810	203.319	218.426	173.640	7.286	165.274	178.596
ENSG00000143337	TOR1AIP1	1.2	3.3E-04	8.6E-04	12.344	0.565	11.911	12.984	10.600	0.281	10.338	10.896
ENSG00000143486	EIF2D	1.2	5.0E-05	1.6E-04	16.654	1.357	15.675	18.203	13.905	0.495	13.477	14.447
ENSG00000143494	VASH2	1.2	1.1E-08	7.3E-08	61.966	0.535	61.359	62.366	50.895	1.488	49.256	52.195
ENSG00000143612	C1orf43	1.2	3.9E-06	1.5E-05	143.250	3.492	139.277	145.837	121.910	6.441	114.825	127.412
ENSG00000143776	CDC42BPA	1.2	5.8E-08	3.2E-07	22.559	1.147	21.272	23.470	18.115	0.533	17.502	18.476
ENSG00000144713	RPL32	1.2	6.2E-10	5.1E-09	497.955	12.130	484.130	506.813	411.406	1.474	410.243	413.064
ENSG00000144741	SLC25A26	1.2	4.3E-05	1.4E-04	13.820	0.756	13.222	14.670	11.305	0.186	11.002	11.366
ENSG00000144791	LIMD1	1.2	3.1E-06	1.2E-05	12.003	0.457	11.476	12.270	9.888	0.064	9.824	9.951
ENSG00000144834	TAGLN3	1.2	7.8E-02	1.2E-01	7.029	0.851	6.254	7.939	5.950	0.595	5.330	6.515
ENSG00000145241	CENPC	1.2	5.2E-03	1.0E-02	10.650	0.710	9.856	11.223	9.238	0.428	8.749	9.544
ENSG00000145331	TRMT10A	1.2	8.1E-02	1.2E-01	4.830	0.543	4.476	5.455	3.991	0.680	3.239	4.562
ENSG00000145425	RPS3A	1.2	3.4E-09	2.4E-08	544.979	16.235	533.941	563.620	442.401	20.002	421.521	461.391
ENSG00000145723	GIN1	1.2	1.2E-02	2.2E-02	7.376	0.597	6.948	8.058	6.092	0.977	5.445	7.215
ENSG00000145743	FBXL17	1.2	9.9E-02	1.5E-01	2.024	0.111	1.898	2.109	1.757	0.197	1.561	1.955
ENSG00000145882	PCYOX1L	1.2	9.1E-04	2.2E-03	6.907	0.271	6.713	7.216	5.643	0.437	5.156	6.000
ENSG00000146250	PRSS35	1.2	1.4E-01	2.0E-01	5.395	1.908	4.137	7.591	4.319	0.711	3.515	4.862
ENSG00000146350	TBC1D32	1.2	1.2E-02	2.2E-02	3.721	0.167	3.528	3.828	3.082	0.284	2.841	3.395
ENSG00000146425	DYNLT1	1.2	1.1E-04	3.3E-04	69.444	1.731	68.418	71.443	60.257	2.669	57.731	63.050
ENSG00000147118	ZNF182	1.2	2.8E-04	7.6E-04	13.556	0.826	12.645	14.254	10.874	0.824	9.922	11.371
ENSG00000147202	DIAPH2	1.2	6.7E-07	3.0E-06	38.337	1.274	36.866	39.093	31.794	1.010	31.113	32.955
ENSG00000147457	CHMP7	1.2	4.0E-04	1.0E-03	29.162	0.700	28.504	29.897	25.233	1.142	24.054	26.335
ENSG00000147592	LACTB2	1.2	1.3E-04	3.8E-04	33.024	1.554	31.663	34.717	26.510	0.280	26.211	26.766
ENSG00000147604	RPL7	1.2	2.8E-07	1.3E-06	205.805	9.242	196.536	215.020	172.105	7.555	163.387	176.735
ENSG00000147650	LRP12	1.2	6.1E-03	1.2E-02	5.735	0.308	5.383	5.960	4.938	0.074	4.854	4.986
ENSG00000147677	EIF3H	1.2	1.1E-09	8.6E-09	129.013	4.036	126.520	133.670	105.563	1.687	104.082	107.400
ENSG00000148154	UGCG	1.2	4.0E-07	1.9E-06	42.694	1.088	41.706	43.860	34.810	1.680	33.034	36.374
ENSG00000148158	SNX30	1.2	6.0E-05	1.8E-04	12.455	0.093	12.391	12.562	10.578	0.252	10.370	10.858
ENSG00000148187	MRRF	1.2	5.9E-05	1.8E-04	11.242	0.711	10.470	11.869	9.266	0.753	8.411	9.830
ENSG00000148290	SURF1	1.2	5.4E-03	1.1E-02	37.387	1.630	35.527	38.565	32.412	1.195	31.695	33.792
ENSG00000148291	SURF2	1.2	6.0E-03	1.2E-02	49.817	3.323	46.090	52.474	42.506	1.747	40.742	44.237
ENSG00000148303	RPL7A	1.2	1.6E-08	9.7E-08	761.433	32.247	732.275	796.067	638.488	4.686	635.408	643.880
ENSG00000148400	NOTCH1	1.2	9.3E-06	3.3E-05	35.325	1.349	34.025	36.719	28.529	1.936	26.538	30.405
ENSG00000149016	TUT1	1.2	2.4E-02	4.1E-02	7.227	0.233	7.036	7.487	6.176	0.451	5.749	6.648
ENSG00000149089	APIP	1.2	5.6E-04	1.4E-03	15.125	0.533	14.599	15.665	12.781	0.906	11.736	13.322
ENSG00000149177	PTPRJ	1.2	4.1E-06	1.6E-05	15.864	0.474	15.339	16.258	13.110	0.582	12.759	13.784
ENSG00000149187	CELF1	1.2	3.6E-06	1.4E-05	84.190	4.300	79.370	87.633	72.673	3.098	69.246	75.272
ENSG00000149273	RPS3	1.2	1.3E-08	8.3E-08	662.384	11.492	649.189	670.202	562.038	11.738	549.895	573.324
ENSG00000149600	COMMD7	1.2	7.3E-06	2.7E-05	43.245	1.442	41.585	44.186	34.849	2.512	31.949	36.307
ENSG00000149761	NUDT22	1.2	4.9E-03	1.0E-02	15.148	0.668	14.614	15.896	12.621	2.169	10.399	14.732
ENSG00000149809	TM7SF2	1.2	3.6E-04	9.5E-04	23.852	0.860	22.862	24.411	19.482	2.015	18.240	21.807
ENSG00000150457	LATS2	1.2	6.5E-05	2.0E-04	19.251	0.511	18.940	19.840	16.136	0.364	15.753	16.477
ENSG00000150477	KIAA1328	1.2	6.4E-02	9.9E-02	1.721	0.233	1.451	1.859	1.480	0.144	1.331	1.617
ENSG00000150551	LYPD1	1.2	1.3E-01	1.9E-01	3.142	0.529	2.535	3.499	2.510	0.402	2.143	2.940
ENSG00000150967	ABCB9	1.2	1.0E-01	1.5E-01	0.986	0.106	0.879	1.091	0.839	0.118	0.704	0.920
ENSG00000151065	DCP1B	1.2	4.4E-03	9.0E-03	14.457	0.402	14.122	14.902	12.555	0.302	12.366	12.903
ENSG00000151150	ANK3	1.2	5.6E-07	2.6E-06	12.900	0.190	12.734	13.106	10.894	0.391	10.662	11.346
ENSG00000151276	MAGI1	1.2	2.5E-07	1.2E-06	39.208	0.607	38.518	39.657	33.086	0.400	32.743	33.526
ENSG00000151413	NUBPL	1.2	4.7E-03	9.5E-03	10.556	0.796	9.666	11.203	8.993	0.448	8.534	9.428
ENSG00000151575	TEX9	1.2	1.2E-02	2.3E-02	4.179	0.200	3.994	4.391	3.547	0.259	3.356	3.842
ENSG00000151665	PIGF	1.2	6.2E-03	1.2E-02	14.107	0.978	13.182	15.130	12.300	0.295	12.026	12.540

ENSG00000151694	ADAM17	1.2	2.9E-04	7.6E-04	29.310	0.218	29.058	29.442	25.356	1.011	24.312	26.330
ENSG00000151779	NBAS	1.2	9.0E-08	4.8E-07	31.467	0.293	31.129	31.653	25.969	0.622	25.252	26.365
ENSG00000152117		1.2	4.0E-03	8.3E-03	20.180	1.988	18.140	22.111	17.387	1.175	16.274	18.616
ENSG00000152332	UHMK1	1.2	1.2E-04	3.3E-04	25.675	0.682	25.128	26.439	22.320	1.185	21.001	23.295
ENSG00000152422	XRCC4	1.2	1.6E-04	4.4E-04	22.362	0.335	22.019	22.688	18.267	0.302	18.009	18.599
ENSG00000152454	ZNF256	1.2	8.7E-03	1.7E-02	26.538	0.968	25.451	27.309	23.026	1.340	21.688	24.377
ENSG00000152465	NMT2	1.2	8.9E-04	2.1E-03	15.962	0.958	14.945	16.847	13.357	0.665	12.683	14.013
ENSG00000152749	GPR180	1.2	2.7E-04	7.2E-04	9.511	0.298	9.203	9.798	7.970	0.525	7.373	8.360
ENSG00000152779	SLC16A12	1.2	5.7E-02	9.0E-02	2.183	0.289	1.852	2.382	1.759	0.237	1.589	2.031
ENSG00000152782	PANK1	1.2	3.4E-05	1.1E-04	23.674	0.347	23.313	24.005	20.260	0.946	19.549	21.334
ENSG00000152904	GGPS1	1.2	1.8E-05	6.0E-05	45.769	0.448	45.342	46.236	38.851	1.451	37.370	40.269
ENSG00000152944	MED21	1.2	3.4E-04	8.9E-04	52.653	1.552	51.370	54.379	45.758	3.437	42.000	48.744
ENSG00000153046	CDYL	1.2	2.8E-06	1.1E-05	89.766	2.620	86.866	91.961	77.214	1.762	75.600	79.093
ENSG00000153071	DAB2	1.2	3.9E-02	6.4E-02	3.557	0.178	3.429	3.760	3.073	0.182	2.880	3.243
ENSG00000153130	SCOC	1.2	6.5E-05	2.0E-04	37.901	1.122	36.750	38.992	32.649	2.158	30.917	35.066
ENSG00000153140	CETN3	1.2	6.8E-06	2.5E-05	48.546	1.672	46.662	49.852	40.104	1.691	38.152	41.100
ENSG00000153832	FBXO36	1.2	3.2E-03	6.8E-03	7.464	0.587	6.789	7.857	6.047	0.537	5.461	6.515
ENSG00000153902	LGI4	1.2	1.3E-01	1.8E-01	1.714	0.077	1.635	1.788	1.438	0.227	1.202	1.655
ENSG00000153956	CACNA2D1	1.2	4.6E-04	1.2E-03	12.339	0.940	11.426	13.303	10.412	0.917	9.581	11.395
ENSG00000154114	TBCEL	1.2	2.9E-04	7.8E-04	11.403	0.375	11.120	11.829	9.433	0.386	9.013	9.773
ENSG00000154127	UBASH3B	1.2	1.2E-02	2.2E-02	4.787	0.342	4.419	5.097	4.098	0.234	3.829	4.252
ENSG00000154217	PITPNC1	1.2	6.6E-04	1.6E-03	17.413	0.300	17.135	17.730	15.090	0.495	14.775	15.661
ENSG00000154370	TRIM11	1.2	3.4E-03	7.2E-03	9.233	0.399	8.772	9.476	7.961	0.679	7.188	8.459
ENSG00000154548	SRSF12	1.2	9.3E-03	1.8E-02	6.158	0.299	5.818	6.378	5.066	0.664	4.300	5.487
ENSG00000154719	MRPL39	1.2	1.2E-05	4.1E-05	128.100	5.333	122.112	132.336	107.148	2.755	104.008	109.164
ENSG00000155008	APOOL	1.2	1.6E-03	3.7E-03	9.995	0.841	9.321	10.937	8.211	0.403	7.768	8.555
ENSG00000155097	ATP6V1C1	1.2	7.2E-05	2.2E-04	47.204	1.489	45.848	48.797	40.619	0.305	40.347	40.948
ENSG00000155099	TMEM55A	1.2	1.8E-04	4.9E-04	46.773	2.750	44.855	49.924	39.164	3.130	35.580	41.362
ENSG00000155100	OTUD6B	1.2	5.7E-05	1.8E-04	44.981	3.915	40.634	48.230	36.541	3.939	33.636	41.025
ENSG00000155229	MMS19	1.2	2.6E-07	1.3E-06	71.356	1.533	69.879	72.939	60.244	1.873	59.030	62.400
ENSG00000155366	RHOC	1.2	3.0E-04	8.0E-04	19.374	1.787	17.544	21.114	15.933	0.983	14.877	16.824
ENSG00000155367	PPM1J	1.2	2.2E-03	4.8E-03	14.694	0.838	13.856	15.532	11.916	0.203	11.762	12.146
ENSG00000155511	GRIA1	1.2	1.1E-01	1.6E-01	0.941	0.136	0.809	1.080	0.769	0.077	0.681	0.821
ENSG00000155970	MICU3	1.2	2.1E-02	3.6E-02	4.386	0.547	3.887	4.970	3.573	0.637	2.839	3.973
ENSG00000155975	VPS37A	1.2	1.1E-04	3.2E-04	19.539	0.886	19.009	20.561	16.648	0.511	16.061	16.998
ENSG00000156110	ADK	1.2	4.3E-04	1.1E-03	37.100	0.480	36.745	37.646	31.952	1.559	30.579	33.647
ENSG00000156239	N6AMT1	1.2	6.9E-04	1.7E-03	12.983	1.333	11.544	14.175	10.828	0.094	10.735	10.924
ENSG00000156261	CCT8	1.2	3.9E-10	3.3E-09	489.555	10.008	478.148	496.864	399.774	14.387	383.496	410.787
ENSG00000156467	UQCRB	1.2	9.7E-07	4.2E-06	68.894	3.039	65.714	71.770	58.548	2.359	56.306	61.008
ENSG00000156482	RPL30	1.2	1.9E-07	9.4E-07	486.791	7.036	479.100	492.904	422.048	1.018	420.927	422.915
ENSG00000156675	RAB11FIP1	1.2	5.8E-06	2.2E-05	28.668	1.335	27.276	29.938	24.267	0.108	24.142	24.335
ENSG00000156795	WDYHV1	1.2	9.2E-05	2.7E-04	32.134	1.321	30.745	33.375	25.967	1.886	24.795	28.142
ENSG00000156976	EIF4A2	1.2	5.4E-08	3.0E-07	364.184	4.323	359.478	367.978	304.995	13.422	293.102	319.548
ENSG00000157107	FCHO2	1.2	1.4E-03	3.3E-03	10.554	0.780	9.850	11.392	8.741	0.821	8.100	9.667
ENSG00000157224	CLDN12	1.2	8.2E-08	4.4E-07	41.001	1.258	39.587	41.996	32.955	1.711	31.011	34.233
ENSG00000157326	DHRS4	1.2	2.8E-02	4.8E-02	12.166	0.656	11.742	12.921	10.466	0.456	10.012	10.924
ENSG00000157514	TSC22D3	1.2	1.5E-04	4.2E-04	13.144	0.497	12.727	13.694	10.614	0.360	10.200	10.856
ENSG00000157557	ETS2	1.2	5.8E-04	1.5E-03	33.001	0.616	32.393	33.624	28.665	0.266	28.454	28.964
ENSG00000157570	TSPAN18	1.2	8.4E-03	1.6E-02	7.034	0.286	6.709	7.244	5.957	0.342	5.687	6.341
ENSG00000157764	BRAF	1.2	2.1E-03	4.6E-03	10.969	0.902	10.013	11.807	9.529	0.535	9.176	10.145
ENSG00000158122	AAED1	1.2	7.5E-02	1.1E-01	6.182	0.662	5.533	6.857	5.304	0.598	4.687	5.881
ENSG00000158195	WASF2	1.2	1.3E-06	5.7E-06	94.063	1.861	92.224	95.945	79.582	1.164	78.824	80.922
ENSG00000158402	CDC25C	1.2	2.2E-03	4.9E-03	40.031	2.821	36.912	42.404	34.688	0.903	34.012	35.713
ENSG00000158427	TMSB15B	1.2	2.7E-02	4.7E-02	3.409	0.079	3.335	3.492	2.873	0.139	2.723	2.996
ENSG00000158604	TMED4	1.2	1.2E-04	3.5E-04	41.695	0.896	40.914	42.672	35.822	1.841	34.579	37.936
ENSG00000158805	ZNF276	1.2	1.3E-02	2.4E-02	2.705	0.223	2.451	2.868	2.172	0.285	1.891	2.461
ENSG00000158813	EDA	1.2	8.5E-03	1.6E-02	6.776	0.507	6.395	7.352	5.790	0.354	5.548	6.196
ENSG00000159086	PAXBP1	1.2	4.2E-06	1.6E-05	49.987	3.221	46.270	51.970	42.659	1.116	41.640	43.851
ENSG00000160058	BSDC1	1.2	1.2E-05	4.3E-05	27.164	1.284	25.780	28.317	22.770	0.740	22.106	23.567
ENSG00000160199	PKNOX1	1.2	3.1E-05	1.0E-04	13.675	0.396	13.227	13.975	11.335	0.316	11.108	11.696
ENSG00000160323	ADAMTS13	1.2	6.2E-02	9.6E-02	1.684	0.275	1.507	2.001	1.361	0.113	1.256	1.481
[illegible]												

ENSG00000238165		1.2	1.2E-01	1.7E-01	32.961	5.579	28.197	39.098	27.412	1.769	25.739	29.263
ENSG00000239374		1.2	1.4E-01	2.0E-01	11.048	0.660	10.332	11.634	8.951	1.326	7.420	9.761
ENSG00000239559		1.2	1.6E-02	2.9E-02	63.510	6.828	58.376	71.259	51.196	5.263	46.399	56.825
ENSG00000240024	LINC00888	1.2	1.4E-03	3.2E-03	17.336	1.466	15.813	18.738	14.013	0.687	13.459	14.782
ENSG00000240344	PPIL3	1.2	4.9E-05	1.5E-04	33.444	2.981	30.139	35.929	27.174	2.090	25.311	29.434
ENSG00000240356	RPL23AP7	1.2	1.5E-02	2.7E-02	9.532	1.091	8.348	10.497	7.754	0.956	6.889	8.780
ENSG00000240376		1.2	4.2E-07	2.0E-06	427.806	3.748	423.509	430.399	349.444	2.609	347.409	352.385
ENSG00000240776		1.2	1.2E-01	1.7E-01	30.748	2.751	28.279	33.714	26.705	2.778	23.516	28.593
ENSG00000241556		1.2	1.5E-02	2.7E-02	73.831	3.445	69.876	76.178	63.154	6.101	58.885	70.142
ENSG00000241837	ATP5O	1.2	4.3E-04	1.1E-03	58.976	1.587	57.390	60.564	51.168	2.703	49.435	54.283
ENSG00000242071	RPL7AP6	1.2	2.0E-09	1.5E-08	2073.482	59.205	2005.501	2113.735	1682.793	90.162	1579.080	1742.517
ENSG00000242294	STAG3L5P	1.2	3.0E-02	5.1E-02	8.931	1.146	7.665	9.899	7.607	0.409	7.208	8.024
ENSG00000242299		1.2	3.9E-07	1.8E-06	1643.124	114.196	1532.329	1760.441	1374.305	6.880	1367.310	1381.062
ENSG00000242445	RPL7AP11	1.2	6.0E-04	1.5E-03	92.323	3.402	89.402	96.058	76.343	8.785	70.875	86.477
ENSG00000242571	RPL21P11	1.2	3.6E-02	6.0E-02	29.142	3.028	26.503	32.448	24.001	0.963	22.940	24.820
ENSG00000243199		1.2	1.4E-08	8.6E-08	602.616	24.509	578.580	627.572	496.517	12.555	483.206	508.145
ENSG00000243335	KCTD7	1.2	5.4E-05	1.7E-04	8.501	0.511	8.100	9.076	6.855	0.215	6.633	7.062
ENSG00000243547	HNRNPKP4	1.2	1.0E-06	4.5E-06	133.617	1.864	131.575	135.227	108.471	7.912	99.828	115.357
ENSG00000243716	NPIPB5	1.2	3.0E-04	8.0E-04	8.346	0.225	8.123	8.573	7.027	0.327	6.794	7.401
ENSG00000243943	ZNF512	1.2	5.8E-04	1.5E-03	28.427	1.655	26.537	29.614	24.346	1.294	22.851	25.094
ENSG00000243964	RPL23AP65	1.2	1.6E-03	3.6E-03	191.912	19.228	172.873	211.323	161.247	4.942	156.815	166.575
ENSG00000243989	ACY1	1.2	7.8E-02	1.2E-01	1.701	0.112	1.572	1.771	1.380	0.095	1.290	1.479
ENSG00000244021		1.2	4.8E-07	2.2E-06	510.308	8.207	500.851	515.568	414.091	24.196	399.453	442.019
ENSG00000244119	PDCL3P4	1.2	7.2E-02	1.1E-01	12.267	1.359	10.789	13.462	9.964	1.158	8.689	10.953
ENSG00000244245		1.2	3.8E-02	6.2E-02	60.161	10.633	52.173	72.230	49.754	3.212	47.366	53.406
ENSG00000247400	DNAJC3-AS1	1.2	7.8E-02	1.2E-01	5.137	1.026	4.154	6.202	4.297	0.641	3.670	4.951
ENSG00000248569		1.2	2.0E-02	3.6E-02	30.872	1.686	29.800	32.816	26.139	0.868	25.445	27.112
ENSG00000249353	NPM1P27	1.2	2.8E-10	2.5E-09	4194.281	149.988	4021.101	4282.565	3452.030	52.072	3396.179	3499.243
ENSG00000249936	RAC1P2	1.2	7.8E-07	3.4E-06	465.810	11.853	452.250	474.202	389.846	12.825	376.403	401.947
ENSG00000250251	PKD1P6	1.2	5.0E-04	1.3E-03	9.365	1.248	8.361	10.762	7.505	0.499	7.049	8.038
ENSG00000250461		1.2	8.8E-03	1.7E-02	45.772	0.793	44.943	46.522	38.249	3.178	36.138	41.904
ENSG00000250903	GMDS-AS1	1.2	1.4E-01	1.9E-01	0.494	0.039	0.464	0.539	0.405	0.073	0.321	0.456
ENSG00000250966		1.2	1.3E-01	1.8E-01	26.255	1.895	24.422	28.206	22.536	2.122	20.131	24.144
ENSG00000251022	THAP9-AS1	1.2	8.8E-05	2.6E-04	37.799	1.802	35.840	39.387	32.037	1.510	30.330	33.199
ENSG00000251495	PPIAP11	1.2	4.5E-03	9.2E-03	83.516	8.352	75.864	92.425	69.311	5.406	63.648	74.417
ENSG00000253506	NACA2	1.2	5.3E-03	1.1E-02	65.177	4.796	62.341	70.714	55.764	1.183	54.976	57.125
ENSG00000254244	PAICSP4	1.2	1.1E-04	3.2E-04	73.186	3.318	69.639	76.215	60.921	1.950	59.736	63.171
ENSG00000254331	CKS1BP7	1.2	2.4E-02	4.2E-02	188.580	25.489	159.328	205.960	161.766	8.459	154.397	171.002
ENSG00000255135		1.2	1.4E-02	2.5E-02	53.286	3.628	49.219	56.187	45.459	4.862	40.017	49.373
ENSG00000255192	NANOGP8	1.2	3.3E-05	1.1E-04	101.174	5.032	96.917	106.728	81.881	1.484	80.191	82.974
ENSG00000255213	NPM1P35	1.2	1.2E-02	2.3E-02	18.114	1.154	16.979	19.286	14.523	0.382	14.129	14.893
ENSG00000255642	PABPC1P4	1.2	1.2E-01	1.8E-01	6.509	1.753	5.119	8.479	5.406	1.018	4.532	6.523
ENSG00000255883	FUNDC2P1	1.2	2.8E-02	4.7E-02	31.765	2.380	29.189	33.883	26.222	3.584	23.117	30.143
ENSG00000256167	ATF4P4	1.2	3.7E-02	6.2E-02	7.890	0.213	7.644	8.017	6.522	0.832	5.591	7.196
ENSG00000256211		1.2	5.6E-02	8.9E-02	34.984	3.796	31.228	38.819	29.775	1.740	28.086	31.563
ENSG00000257621	PSMA3-AS1	1.2	2.2E-04	6.1E-04	9.621	0.790	8.729	10.231	7.857	0.537	7.287	8.352
ENSG00000257815		1.2	3.6E-02	6.0E-02	10.383	0.713	9.829	11.188	8.566	0.754	8.020	9.426
ENSG00000257923	CUX1	1.2	4.4E-07	2.0E-06	12.614	0.394	12.217	13.005	10.385	0.292	10.075	10.654
ENSG00000258445		1.2	8.8E-02	1.3E-01	23.359	1.987	22.194	25.653	20.144	0.333	19.891	20.521
ENSG00000258925	NPM1P5	1.2	9.8E-03	1.9E-02	40.580	2.553	38.000	43.106	34.111	1.358	32.561	35.092
ENSG00000259330	INAFM2	1.2	5.8E-04	1.5E-03	31.405	1.712	29.585	32.984	26.699	2.054	24.807	28.883
ENSG00000259429	UBE2Q2P2	1.2	6.6E-03	1.3E-02	11.637	0.916	11.021	12.689	9.370	0.743	8.512	9.814
ENSG00000259843		1.2	4.7E-02	7.6E-02	13.556	1.532	12.120	15.168	11.397	1.707	9.734	13.144
ENSG00000259959		1.2	2.5E-02	4.2E-02	6.466	0.416	6.003	6.809	5.463	0.196	5.266	5.658
ENSG00000260261		1.2	9.4E-02	1.4E-01	3.031	0.149	2.867	3.158	2.526	0.243	2.346	2.681
ENSG00000260708		1.2	5.7E-02	9.0E-02	35.827	3.050	33.433	39.261	30.715	4.538	25.644	34.396
ENSG00000260920		1.2	4.4E-02	7.1E-02	7.364	0.756	6.492	7.821	6.150	0.814	5.234	6.788
ENSG00000260942	CAPN10-AS1	1.2	1.1E-01	1.6E-01	3.778	0.234	3.550	4.017	3.284	0.294	2.952	3.511
ENSG00000261008	LINC01572	1.2	3.1E-02	5.2E-02	2.716	0.136	2.573	2.843	2.190	0.210	1.953	2.352
ENSG00000261094		1.2	4.4E-02	7.1E-02	5.387	0.418	4.917	5.714	4.438	0.630	3.723	4.911
ENSG00000261116		1.2	1.0E-01	1.5E-01	5.402	0.240	5.149	5.626	4.502	0.766	3.855	5.348
ENSG00000261824	LINC00662	1.2	1.2E-04	3.6E-04	9.881	0.219	9.723	10.131	8.118	0.350	7.915	8.522
ENSG00000262160		1.2	1.1E-01	1.5E-01	6.361	0.199	6.131	6.489	5.543	0.911	4.979	6.594
ENSG00000263006	ROCK1P1	1.2	1.1E-01	1.6E-01	6.445	0.452	5.923	6.720	5.580	0.969	5.018	6.698
ENSG00000263072	ZNF213-AS1	1.2	1.9E-02	3.4E-02	4.612	0.650	4.007	5.300	3.710	0.393	3.313	4.099
ENSG00000263535		1.2	2.0E-03	4.4E-03	147.966	9.718	136.778	154.317	127.319	6.488	123.335	134.806
ENSG00000264112		1.2	4.5E-02	7.3E-02	3.809	0.493	3.351	4.330	3.281	0.202	3.052	3.435
ENSG00000266412	NCOA4	1.2	2.7E-06	1.1E-05	91.735	1.134	90.816	93.002	79.087	1.389	77.558	80.272
ENSG00000267312		1.2	1.4E-01	1.9E-01	7.391	1.023	6.751	8.571	6.169	1.069	5.287	7.358
ENSG00000267508	ZNF285	1.2	2.4E-02	4.1E-02	2.765	0.377	2.364	3.114	2.272	0.136	2.146	2.416
ENSG00000268089	GABRQ	1.2	8.1E-05	2.4E-04	11.734	0.796	10.941	12.532	9.438	0.565	8.827	9.942
ENSG00000268658	LINC00664	1.2	3.9E-03	8.2E-03	9.606	0.767	8.728	10.143	8.210	0.072	8.151	8.290
ENSG00000269226		1.2	1.5E-02	2.7E-02	5.485	0.348	5.100	5.775	4.465	0.070	4.386	4.522
ENSG00000269609	RPARP-AS1	1.2	1.2E-03	2.8E-03	13.123	0.509	12.633	13.649	10.985	0.676	10.209	11.444
ENSG00000269713	NBPF9	1.2	2.7E-02	4.6E-02	2.090	0.204	1.935	2.321	1.732	0.150	1.606	1.898
ENSG00000269893	SNHG8	1.2	4.5E-05	1.4E-04	174.405	7.624	165.602	178.850	147.285	5.542	142.272	153.235
ENSG00000269929		1.2	1.2E-01	1.7E-01	7.755	1.754	5.882	9.358	6.449	0.961	5.354	7.152
ENSG00000269973		1.2	1.2E-01	1.7E-01	2.692	0.234	2.552	2.962	2.226	0.503	1.646	2.551
ENSG00000270883		1.2	1.3E-01	1.8E-01	18.422	1.136	17.510	19.694	14.836	0.976	14.184	15.958
ENSG00000271383	NBPF19	1.2	6.5E-03	1.3E-02	2.011	0.209	1.842	2.245	1.688	0.126	1.562	1.815
ENSG00000272086		1.2	6.1E-02	9.5E-02	13.328	0.840	12.588	14.242	10.795	0.912	9.928	11.745
ENSG00000272143	FGF14-AS2	1.2	6.6E-02	1.0E-01	8.965	1.106	8.076	10.204	7.236	0.871	6.243	7.872
ENSG00000272150	NBPF25P	1.2	1.0E-01	1.5E-01	2.231	0.119	2.146	2.367	1.844	0.187	1.662	2.036
ENSG00000272335		1.2	6.3E-03	1.2E-02	10.022	1.394	8.769	11.524	8.036	0.879	7.261	8.991
ENSG00000272391	POM121C	1.2	1.1E-06	4.7E-06	22.316	0.199	22.197	22.546	18.223	1.077	17.007	19.056
ENSG00000272419		1.2	4.5E-02	7.2E-02	1.777	0.062	1.708	1.829	1.435	0.098	1.362	1.546
ENSG00000272645	GTF2IP20	1.2	3.2E-02	5.3E-02	3.929	0.594	3.243	4.280	3.181	0.183	3.068	3.392
ENSG00000272686		1.2	3.1E-02	5.2E-02	6.883	1.169	5.956	8.197	5.553	0.492	5.058	6.042
ENSG00000272831		1.2	9.1E-02	1.3E-01	16.085	2.675	13.301	18.635	13.048	1.684	11.285	14.640
ENSG00000272886	DCP1A	1.2	1.2E-03	2.8E-03	19.716	0.560	19.070	20.051	17.166	0.955	16.069	17.806
ENSG00000273136	NBPF26	1.2	2.3E-02	4.0E-02	3.816	0.597	3.249	4.438	3.192	0.419	2.869	3.666
ENSG00000273329		1.2	8.1E-02	1.2E-01	1.234	0.084	1.169	1.329	0.994	0.087	0.927	1.092
ENSG00000273590	SMIM11B	1.2	1.6E-02	2.9E-02	4.263	0.150	4.094	4.379	3.470	0.151	3.296	3.570
ENSG00000273899	NOL12	1.2	5.8E-02	9.1E-02	3.519	0.512	2.932	3.871	2.877	0.279	2.660	3.192
ENSG00000274333		1.2	1.0E-06	4.5E-06	14.399	0.575	13.741	14.801	11.611	0.268	11.423	11.918
ENSG00000274349	ZNF658	1.2	7.9E-02	1.2E-01	3.139	0.138	2.990	3.262	2.691	0.291	2.356	2.869
ENSG00000274499		1.2	1.1E-01	1.6E-01	4.491	0.695	3.708	5.035	3.625	0.260	3.390	3.904
ENSG00000274602	PI4KAP1	1.2	7.1E-04	1.7E-03	11.388	0.534	10.799	11.840	9.366	0.540	8.907	9.961
ENSG00000275496		1.2	3.3E-06	1.3E-05	42.513	1.026	41.374	43.365	34.744	0.958	33.720	35.617
ENSG00000275764		1.2	6.1E-03	1.2E-02	16.253	1.876	15.024	18.413	13.442	0.408	13.033	13.849
ENSG00000275993		1.2	1.1E-02	2.0E-02	5.074	0.258	4.778	5.252	4.134	0.262	3.901	4.417
ENSG00000276045	ORAI1	1.2	2.6E-04	6.9E-04	27.437	1.885	25.521	29.290	22.019	1.817	20.873	24.114
ENSG00000276077		1.2	2.5E-06	9.9E-06	35.237	1.377	33.692	36.335	28.596	0.723	28.089	29.424
ENSG00000277067		1.2	3.4E-06	1.3E-05	38.613	1.404	37.035	39.725	31.446	0.845	30.795	32.400
ENSG00000277147	LINC00869	1.2	5.4E-03	1.1E-02	3.015	0.253	2.730	3.213	2.451	0.040	2.407	2.485
ENSG00000277200		1.2	4.8E-02	7.8E-02	8.099	0.226	7.943	8.359	6.869	0.430	6.478	7.280
ENSG00000277203	F8A1	1.2	1.1E-01	1.5E-01	11.409	0.419	10.944	11.759	9.852	1.228	8.821	11.258
ENSG00000277443	MARCKS	1.2	2.6E-06	1.0E-05	189.806	8.938	183.359	200.010	164.886	1.217	163.877	166.237
ENSG00000277991		1.2	1.3E-06	5.4E-06	15.722	0.426	15.231	15.992	12.713	0.267	12.426	12.955
ENSG00000278530	CHMP1B2P	1.2	1.5E-02	2.8E-02	3.627	0.538	3.023	4.056	3.011	0.233	2.787	3.252
ENSG00000278730		1.2	2.7E-02	4.6E-02	9.434	0.360	9.036	9.735	8.125	0.464	7.605	8.496
ENSG00000278817		1.2	8.6E-02	1.3E-01	5.695	0.663	5.060	6.383	4.571	0.373	4.181	4.923
ENSG00000278803		1.2	1.1E-01	1.6E-01	3.293	0.101	3.177	3.362	2.812	0.204	2.606	3.015
ENSG00000279483		1.2	3.0E-06	1.2E-05	15354.425	461.963	14891.226	15815.143	12684.479	955.109	11641.739	13516.901
ENSG00000280145		1.2	6.7E-02	1.0E-01	1.630	0.110	1.556	1.756	1.361	0.133	1.210	1.459
ENSG00000280206		1.2	4.3E-02	7.0E-02	12.322	1.127	11.025	13.058	9.957	1.602	8.394	11.595
ENSG00000283041		1.2	7.1E-10	5.8E-09	1750.780	51.629	1693.753	1794.344	1444.537	26.967	1420.102	1473.470
ENSG00000001460	STPG1	1.3	3.3E-03	7.0E-03	3.528	0.093	3.425	3.605	2.828	0.492	2.288	3.271
ENSG00000002919	SNX11	1.3	8.7E-07	3.8E-06	22.412	0.994	21.427	23.413	17.188	0.898	16.231	18.013
ENSG00000005471	ABCB4	1.3	6.6E-02	1.0E-01	0.824	0.170	0.702	1.018	0.630	0.084	0.582	0.727
ENSG00000008294	SPAG9	1.3	2.0E-11	2.2E-10	33.295	1.294	32.531	34.790	24.766	0.748	24.147	25.596
ENSG00000010318	PHF7	1.3	1.7E-03	3.7E-03	6.527	0.792	5.743	7.328	4.901	0.669	4.315	5.629
ENSG00000010704	HFE	1.3	2.7E-02	4.6E-02	1.270	0.093	1.170	1.353	0.954	0.059	0.892	1.009
ENSG00000011422	PLAUR	1.3	1.2E-04	3.4E-04	8.157	0.507	7.827	8.741	6.246	0.462	5.844	6.750
ENSG00000012174	MBTPS2	1.3	2.8E-05	9.1E-05	7.918	0.435	7.661	8.420	6.310	0.259	6.111	6.603
ENSG00000012822	CALCOCO1	1.3	9.9E-05	2.9E-04	5.258	0.347	5.018	5.655	4.041	0.266	3.736	4.230
ENSG00000013364	MVP	1.3	1.5E-03	3.5E-03	3.342	0.438	2.836	3.595	2.478	0.170	2.289	2.620
ENSG00000013563	DNASE1L1	1.3	3.3E-04	8.6E-04	7.138	0.808	6.266	7.861	5.381	0.146	5.283	5.549
ENSG00000022567	SLC45A4	1.3	1.0E-06	4.4E-06	13.760	0.015	13.743	13.771	10.953	0.014	10.937	10.962
ENSG00000023171	GRAMD1B	1.3	7.7E-06	2.8E-05	5.087	0.335	4.826	5.464	3.873	0.386	3.596	4.314
ENSG00000023330	ALAS1	1.3	9.8E-09	6.4E-08	59.757	2.202	57.455	61.842	45.921	1.679	44.850	47.856
ENSG00000024422	EHD2	1.3	4.3E-04	1.1E-03	8.078	0.997	7.068	9.061	6.130	0.580	5.626	6.778
ENSG00000024526	DEPDC1	1.3	4.4E-04	1.1E-03	55.356	1.792	53.789	57.309	43.819	2.133	42.494	46.279
ENSG00000029639	TFB1M	1.3	9.4E-08	5.0E-07	41.894	0.796	41.003	42.538	31.470	1.799	29.393	32.522
ENSG00000041988	THAP3	1.3	6.1E-04	1.5E-03	7.051	0.528	6.458	7.467	5.514	0.225	5.325	5.763
ENSG00000047644	WWC3	1.3	1.1E-07	5.6E-07	24.461	0.791	23.736	25.304	18.808	0.423	18.320	19.072
ENSG00000048392	RRM2B	1.3	1.1E-04	3.1E-04	99.913	3.341	97.619	103.746	75.734	3.434	72.654	79.437
ENSG00000049239	H6PD	1.3	1.5E-06	6.4E-06	8.756	0.327	8.436	9.090	6.642	0.398	6.189	6.934
ENSG00000050426	LETMD1	1.3	5.0E-06	1.9E-05	14.866	0.941	13.931	15.814	11.708	0.243	11.440	11.914
ENSG00000050820	BCAR1	1.3	4.6E-06	1.8E-05	35.525	1.749	33.917	37.388	27.261	1.862	25.113	28.418
ENSG00000052841	TTC17	1.3	1.7E-09	1.3E-08	21.699	0.287	21.421	21.995	17.032	0.593	16.359	17.480
ENSG00000053108	FSTL4	1.3	1.1E-02	2.1E-02	2.986	0.269	2.796	3.294	2.391	0.291	2.187	2.725
ENSG00000054611	TBC1D22A	1.3	2.0E-05	6.9E-05	11.291	1.104	10.019	12.002	8.585	0.838	7.622	9.152
ENSG00000058272	PPP1R12A	1.3	1.1E-11	1.3E-10	46.502	0.702	45.736	47.115	35.235	1.315	33.793	36.367
ENSG00000059758	CDK17	1.3	8.1E-07	3.6E-06	19.197	0.610	18.590	19.810	15.115	0.749	14.309	15.789
ENSG00000063046	EIF4B	1.3	1.7E-13	2.9E-12	367.496	2.070	365.438	369.578	286.463	5.579	281.159	292.281
ENSG00000064687	ABCA7	1.3	1.0E-04	3.0E-04	4.369	0.391	3.929	4.675	3.249	0.356	2.890	3.602
ENSG00000066027	PPP2R5A	1.3	2.3E-08	1.4E-07	57.530	1.486	56.101	59.068	46.122	1.459	45.205	47.804
ENSG00000067704	IARS2	1.3	3.7E-11	4.0E-10	142.112	3.799	138.507	146.079	111.481	0.617	110.792	111.985
ENSG00000068323	TFE3	1.3	4.1E-08	2.3E-07	56.951	2.844	54.013	59.690	43.430	1.652	41.779	45.083
ENSG00000068354	TBC1D25	1.3	6.9E-03	1.4E-02	4.864	0.285	4.642	5.185	3.881	0.126	3.746	3.995
ENSG00000068745	IP6K2	1.3	8.9E-09	5.8E-08	41.528	1.718	39.822	43.258	33.119	1.123	32.296	34.398
ENSG00000068903	SIRT2	1.3	8.2E-04	2.0E-03	3.684	0.402	3.221	3.938	2.726	0.515	2.155	3.155
ENSG00000069424	KCNAB2	1.3	2.0E-03	4.5E-03	3.206	0.395	2.789	3.575	2.497	0.216	2.260	2.681
ENSG00000069966	GNB5	1.3	2.9E-06	1.1E-05	9.228	0.155	9.054	9.352	7.371	0.227	7.209	7.631
ENSG00000072134	EPN2	1.3	1.2E-06	5.2E-06	20.058	0.124	19.942	20.189	16.050	0.916	15.253	17.051
ENSG00000072657	TRHDE	1.3	5.7E-03	1.1E-02	1.438	0.224	1.182	1.594	1.115	0.013	1.106	1.130
ENSG00000073060	SCARB1	1.3	2.7E-10	2.4E-09	26.925	0.525	26.320	27.258	20.495	0.173	20.337	20.681
ENSG00000073150	PANX2	1.3	1.1E-02	2.1E-02	5.179	0.446	4.675	5.523	3.899	1.057	3.107	5.099
ENSG00000073712	FERMT2	1.3	1.9E-13	3.2E-12	77.796	1.643	76.122	79.406	58.235	1.474	56.580	59.409
ENSG00000075391	RASAL2	1.3	1.3E-09	1.0E-08	16.332	0.375	16.005	16.741	12.592	0.482	12.215	13.136
ENSG00000076716	GPC4	1.3	9.2E-13	1.3E-11	346.468	5.584	340.345	351.282	259.225	3.495	255.986	262.929
ENSG00000076944	STXBP2	1.3	3.2E-05	1.0E-04	7.923	0.263	7.621	8.094	6.092	0.450	5.683	6.574
ENSG00000077585	GPR137B	1.3	6.9E-04	1.7E-03	11.310	1.297	9.976	12.567	8.591	1.018	7.441	9.377
ENSG00000079819	EPB41L2	1.3	2.0E-11	2.2E-10	74.785	0.563	74.239	75.365	59.220	0.584	58.813	59.902
ENSG00000080947	CROCCP3	1.3	3.2E-03	6.8E-03	3.571	0.304	3.251	3.856	2.842	0.220	2.590	2.997
ENSG00000082701	GSK3B	1.3	1.7E-09	1.3E-08	37.514	1.236	36.091	38.319	29.509	0.973	28.598	30.534
ENSG00000083937	CHMP2B	1.3	4.6E-09	3.2E-08	100.967	5.116	96.456	106.526	79.949	2.502	77.086	81.717
ENSG00000084444	FAM234B	1.3	9.7E-07	4.2E-06	17.606	0.995	16.627	18.616	13.815	0.543	13.362	14.417
ENSG00000084754	HADHA	1.3	9.4E-11	9.1E-10	200.287	6.703	194.943	207.808	160.112	3.361	157.667	163.945
ENSG00000085832	EPS15	1.3	2.0E-08	1.2E-07	39.079	1.125	37.936	40.185	31.060	2.056	28.757	32.710
ENSG00000086666	ZFAND6	1.3	9.5E-12	1.2E-10	89.290	1.969	87.554	91.430	68.478	1.594	66.739	69.871
ENSG00000086848	ALG9	1.3	1.5E-02	2.7E-02	2.005	0.272	1.714	2.254	1.581	0.270	1.273	1.770
ENSG00000087842	PIR	1.3	4.2E-06	1.6E-05	35.732	1.309	34.377	36.990	27.382	2.955	24.482	30.391
ENSG00000088451	TGDS	1.3	6.5E-07	2.9E-06	31.022	1.951	28.778	32.313	23.169	2.200	21.015	25.413
ENSG00000088538	DOCK3	1.3	2.3E-04	6.2E-04	4.776	0.142	4.654	4.932	3.740	0.182	3.536	3.886
ENSG00000089157	RPLP0	1.3	7.1E-14	1.3E-12	1087.726	51.800	1043.829	1144.861	819.557	21.946	795.148	837.661
ENSG00000089902	RCOR1	1.3	1.4E-07	7.4E-07	27.833	0.443	27.351	28.222	22.206	0.330	21.899	22.556
ENSG00000090581	GNPTG	1.3	1.1E-04	3.1E-04	8.221	0.175	8.112	8.423	6.258	0.433	5.929	6.748
ENSG00000091986	CCDC80	1.3	6.5E-03	1.3E-02	1.878	0.124	1.736	1.962	1.484	0.026	1.453	1.502
ENSG00000092330	TINF2	1.3	2.9E-06	1.1E-05	26.982	1.149	26.009	28.250	20.550	0.118	20.464	20.685
ENSG00000093072	ADA2	1.3	7.3E-06	2.7E-05	12.903	0.574	12.258	13.359	10.050	0.645	9.369	10.650
ENSG00000095066	HOOK2	1.3	4.9E-07	2.2E-06	19.676	0.955	18.574	20.241	15.501	0.759	14.626	15.973
ENSG00000095397	WHRN	1.3	1.3E-03	2.9E-03	5.313	0.214	5.067	5.459	4.152	0.244	3.932	4.414
ENSG00000097096	SYDE2	1.3	2.6E-04	6.9E-04	6.981	0.334	6.618	7.274	5.545	0.417	5.122	5.955
ENSG00000099204	ABLIM1	1.3	1.2E-11	1.5E-10	42.776	1.566	40.968	43.718	32.434	0.884	31.691	33.411
ENSG00000099822	HCN2	1.3	5.6E-02	8.9E-02	1.999	0.113	1.921	2.128	1.512	0.440	1.025	1.881
ENSG00000100106	TRIOBP	1.3	9.3E-09	6.1E-08	11.987	0.376	11.560	12.269	9.153	0.051	9.099	9.200
ENSG00000100359	SGSM3	1.3	2.7E-02	4.6E-02	1.673	0.048	1.618	1.701	1.272	0.120	1.172	1.406
ENSG00000100523	DDHD1	1.3	9.4E-09	6.2E-08	8.352	0.125	8.258	8.494	6.363	0.232	6.148	6.609
ENSG00000100949	RABGGTA	1.3	1.3E-03	3.0E-03	7.138	0.071	7.097	7.220	5.573	0.430	5.083	5.885
ENSG00000100985	MMP9	1.3	2.0E-02	3.5E-02	6.377	0.846	5.418	7.018	4.860	1.182	4.032	6.304
ENSG00000101109	STK4	1.3	4.8E-11	5.0E-10	30.603	1.494	29.501	32.303	22.936	0.665	22.186	23.455
ENSG00000101349	PAK5	1.3	1.1E-01	1.6E-01	0.825	0.019	0.808	0.845	0.630	0.108	0.526	0.742
ENSG00000101445	PPP1R16B	1.3	2.6E-06	1.0E-05	13.283	0.445	12.903	13.772	10.291	0.576	9.776	10.913
ENSG00000101558	VAPA	1.3	9.0E-09	5.9E-08	36.152	0.283	35.942	36.474	28.977	1.492	27.542	30.521
ENSG00000101577	LPIN2	1.3	3.3E-08	1.9E-07	16.419	0.757	15.731	17.230	12.395	0.890	11.463	13.235
ENSG00000101665	SMAD7	1.3	3.4E-04	9.0E-04	13.637	1.593	11.915	15.058	10.751	0.673	9.977	11.191
ENSG00000101751	POLI	1.3	9.5E-09	6.2E-08	14.261	0.774	13.412	14.928	10.612	0.333	10.294	10.959
ENSG00000101843	PSMD10	1.3	1.1E-10	1.0E-09	106.377	2.197	104.197	108.590	79.514	4.907	74.115	83.704
ENSG00000101901	ALG13	1.3	7.1E-10	5.8E-09	31.483	0.842	30.519	32.074	24.449	0.988	23.592	25.530
ENSG00000102048	ASB9	1.3	6.5E-02	1.0E-01	2.325	0.657	1.698	3.009	1.745	0.175	1.558	1.904
ENSG00000102053	ZC3H12B	1.3	1.0E-02	1.9E-02	1.354	0.075	1.285	1.435	1.017	0.029	0.988	1.045
ENSG00000102225	CDK16	1.3	7.0E-09	4.7E-08	60.791	2.105	58.886	63.051	47.704	3.439	43.765	50.104
ENSG00000102226	USP11	1.3	5.5E-07	2.5E-06	61.690	2.219	59.206	63.480	48.977	1.511	47.336	50.311
ENSG00000102471	NDFIP2	1.3	2.0E-08	1.2E-07	28.445	0.732	27.787	29.234	21.171	1.652	20.097	23.074
ENSG00000102802	MEDAG	1.3	2.9E-03	6.2E-03	8.561	0.117	8.452	8.685	6.709	0.285	6.433	7.002
ENSG00000102890	ELMO3	1.3	1.2E-03	2.8E-03	10.439	1.005	9.645	11.569	8.068	0.305	7.757	8.367
ENSG00000103152	MPG	1.3	6.7E-06	2.5E-05	47.867	1.502	46.140	48.862	37.251	3.217	34.365	40.720
ENSG00000103187	COTL1	1.3	6.8E-06	2.5E-05	60.696	2.978	58.057	63.924	47.390	5.060	41.811	51.685
ENSG00000103449	SALL1	1.3	1.3E-06	5.5E-06	37.971	0.601	37.280	38.372	29.531	2.339	27.691	32.163
ENSG00000103479	RBL2	1.3	2.1E-07	1.0E-06	22.610	0.923	21.929	23.660	17.886	1.101	16.806	19.007
ENSG00000104177	MYEF2	1.3	1.0E-09	8.1E-09	30.744	1.470	29.099	31.931	23.653	1.283	22.887	25.133
ENSG00000104313	EYA1	1.3	5.3E-02	8.4E-02	1.317	0.407	0.955	1.758	0.994	0.098	0.892	1.088
ENSG00000104497	SNX16	1.3	1.3E-03	3.0E-03	9.452	1.270	8.705	10.918	7.451	0.480	6.969	7.929
ENSG00000104872	PIH1D1	1.3	2.4E-08	1.4E-07	75.342	3.637	72.438	79.422	58.746	0.688	57.951	59.255
ENSG00000104883	PEX11G	1.3	4.3E-02	7.0E-02	4.852	0.372	4.540	5.264	3.619	1.063	2.877	4.837
ENSG00000104936	DMPK	1.3	2.4E-03	5.2E-03	6.364	0.392	5.925	6.681	5.061	0.487	4.503	5.401
ENSG00000104983	CCDC61	1.3	3.2E-03	6.8E-03	10.750	0.856	10.139	11.729	8.472	1.174	7.125	9.273
ENSG00000105193	RPS16	1.3	3.0E-11	3.3E-10	602.984	15.118	592.482	620.311	476.713	17.713	457.488	492.371
ENSG00000105202	FBL	1.3	2.0E-11	2.3E-10	597.829	13.052	587.854	612.601	474.079	18.822	454.081	491.448
ENSG00000105278	ZFR2	1.3	7.7E-03	1.5E-02	3.166	0.366	2.799	3.532	2.509	0.230	2.327	2.767
ENSG00000105373	NOP53	1.3	1.4E-10	1.3E-09	102.288	2.265	99.725	104.021	77.541	6.685	70.393	83.640
ENSG00000105397	TYK2	1.3	9.9E-06	3.6E-05	7.943	0.485	7.634	8.502	6.119	0.253	5.839	6.332
ENSG00000105427	CNFN	1.3	5.9E-02	9.2E-02	20.085	2.379	17.449	22.073	15.490	1.495	14.363	17.187
ENSG00000105479	CCDC114	1.3	1.2E-01	1.7E-01	1.324	0.257	1.133	1.616	1.020	0.430	0.523	1.269
ENSG00000105519	CAPS	1.3	1.1E-01	1.6E-01	0.946	0.133	0.833	1.093	0.717	0.063	0.648	0.772
ENSG00000105649	RAB3A	1.3	9.3E-03	1.8E-02	10.559	1.368	9.018	11.621	8.224	0.374	7.837	8.584
ENSG00000105662	CRTC1	1.3	1.0E-03	2.5E-03	3.818	0.339	3.470	4.148	2.895	0.390	2.467	3.230
ENSG00000105767	CADM4	1.3	2.3E-07	1.1E-06	35.905	1.170	34.979	37.219	26.723	0.535	26.291	27.321
ENSG00000105810	CDK6	1.3	1.2E-07	6.4E-07	11.960	0.294	11.783	12.300	9.314	0.396	8.876	9.644
ENSG00000106003	LFNG	1.3	1.7E-04	4.7E-04	15.139	1.396	13.936	16.669	12.001	0.988	11.270	13.126
ENSG00000106080	FKBP14	1.3	1.3E-06	5.4E-06	19.387	0.290	19.139	19.707	14.857	0.568	14.287	15.422
ENSG00000106153	CHCHD2	1.3	2.9E-08	1.7E-07	209.981	9.717	201.872	220.752	159.613	3.349	157.152	163.426
ENSG00000106261	ZKSCAN1	1.3	3.7E-06	1.4E-05	20.861	1.922	19.625	23.076	16.579	0.531	15.968	16.921
ENSG00000106460	TMEM106B	1.3	2.6E-08	1.5E-07	13.764	0.845	12.811	14.421	10.334	0.732	9.618	11.082
ENSG00000106682	EIF4H	1.3	4.0E-12	5.2E-11	429.056	5.312	422.929	432.368	340.215	2.289	338.131	342.665
ENSG00000107036	RIC1	1.3	4.7E-09	3.2E-08	28.458	1.231	27.435	29.824	22.483	0.633	21.788	23.026
ENSG00000107281	NPDC1	1.3	2.8E-06	1.1E-05	19.120	1.043	18.079	20.164	14.234	0.866	13.270	14.947
ENSG00000107295	SH3GL2	1.3	6.4E-03	1.3E-02	7.981	0.911	6.945	8.655	6.081	0.575	5.440	6.553
ENSG00000107438	PDLIM1	1.3	3.4E-12	4.5E-11	184.044	2.146	181.623	185.715	137.304	8.608	128.333	145.498
ENSG00000107537	PHYH	1.3	2.2E-06	9.1E-06	26.037	0.892	25.337	27.041	20.822	0.777	19.955	21.457
ENSG00000107625	DDX50	1.3	3.4E-11	3.6E-10	103.534	3.092	100.166	106.244	78.041	2.727	75.023	80.330
ENSG00000107679	PLEKHA1	1.3	1.0E-07	5.4E-07	7.086	0.395	6.667	7.451	5.528	0.473	5.071	6.016
ENSG00000107954	NEURL1	1.3	1.0E-01	1.5E-01	1.081	0.170	0.964	1.276	0.849	0.119	0.765	0.986
ENSG00000107968	MAP3K8	1.3	6.4E-03	1.3E-02	4.859	0.744	4.185	5.658	3.741	0.092	3.635	3.802
ENSG00000108107	RPL28	1.3	2.0E-11	2.2E-10	162.365	4.591	157.496	166.616	128.242	5.507	121.917	131.974
ENSG00000108239	TBC1D12	1.3	4.9E-03	1.0E-02	2.995	0.265	2.753	3.278	2.333	0.228	2.079	2.522
ENSG00000108306	FBXL20	1.3	3.1E-05	1.0E-04	5.125	0.598	4.554	5.747	3.914	0.249	3.681	4.176
ENSG00000108733	PEX12	1.3	3.2E-03	6.9E-03	6.210	0.994	5.169	7.149	4.661	0.366	4.345	5.062
ENSG00000108799	EZH1	1.3	4.6E-06	1.7E-05	10.426	0.553	9.807	10.871	8.230	0.365	7.856	8.586
ENSG00000109083	IFT20	1.3	2.2E-04	6.0E-04	12.258	0.688	11.588	12.962	9.624	0.522	9.047	10.064
ENSG00000109654	TRIM2	1.3	8.5E-12	1.0E-10	35.099	1.098	34.047	36.237	26.282	0.333	25.898	26.485
ENSG00000109680	TBC1D19	1.3	2.2E-02	3.8E-02	3.422	0.477	3.105	3.971	2.582	0.647	2.034	3.296
ENSG00000110002	VWA5A	1.3	3.8E-09	2.7E-08	23.325	0.653	22.584	23.816	17.656	0.484	17.126	18.102
ENSG00000110880	CORO1C	1.3	3.3E-13	5.3E-12	98.994	2.611	97.476	102.009	74.060	1.163	72.899	75.225
ENSG00000111144	LTA4H	1.3	7.8E-11	7.7E-10	101.257	2.371	99.337	103.907	80.670	0.954	79.584	81.409
ENSG00000111199	TRPV4	1.3	4.0E-02	6.5E-02	2.415	0.287	2.094	2.648	1.884	0.280	1.646	2.192
ENSG00000111540	RAB5B	1.3	1.2E-09	9.4E-09	44.458	2.304	42.523	47.007	34.748	1.307	33.650	36.194
ENSG00000112406	HECA	1.3	7.2E-07	3.2E-06	12.906	0.270	12.620	13.156	9.738	0.117	9.619	9.853
ENSG00000112679	DUSP22	1.3	4.2E-07	1.9E-06	16.733	0.644	16.279	17.471	12.641	1.267	11.227	13.674
ENSG00000113140	SPARC	1.3	5.7E-11	5.9E-10	249.214	3.575	245.170	251.955	199.962	7.286	192.111	206.505
ENSG00000113248	PCDHB15	1.3	1.4E-03	3.3E-03	6.282	0.658	5.810	7.034	4.893	0.387	4.473	5.234
ENSG00000113583	C5orf15	1.3	5.2E-08	2.9E-07	87.207	5.603	80.743	90.672	65.588	6.667	61.087	73.247
ENSG00000113971	NPHP3	1.3	3.0E-03	6.5E-03	2.313	0.312	2.125	2.673	1.803	0.031	1.768	1.827
ENSG00000114054	PCCB	1.3	1.2E-11	1.4E-10	43.052	0.886	42.041	43.690	32.577	0.480	32.068	33.022
ENSG00000114127	XRN1	1.3	4.2E-06	1.6E-05	9.164	0.525	8.566	9.551	7.326	0.454	6.972	7.838
ENSG00000114200	BCHE	1.3	3.0E-03	6.3E-03	6.407	0.556	5.905	7.005	4.791	0.470	4.379	5.303
ENSG00000114209	PDCD10	1.3	1.4E-09	1.1E-08	80.728	2.228	78.470	82.924	62.924	1.635	61.045	64.022
ENSG00000114626	ABTB1	1.3	1.5E-02	2.8E-02	2.749	0.323	2.382	2.987	2.165	0.257	1.999	2.462
ENSG00000114670	NEK11	1.3	4.2E-02	6.8E-02	1.291	0.156	1.182	1.470	0.885	0.029	0.965	1.019
ENSG00000115657	ABCB6	1.3	2.8E-03	6.0E-03	2.872	0.240	2.595	3.025	2.139	0.263	1.972	2.442
ENSG00000115661	STK16	1.3	4.9E-05	1.5E-04	13.866	1.115	12.793	15.018	10.839	0.441	10.330	11.104
ENSG00000116151	MORN1	1.3	3.4E-02	5.7E-02	0.946	0.013	0.931	0.953	0.750	0.130	0.632	0.890
ENSG00000116574	RHOU	1.3	3.5E-04	9.1E-04	8.775	0.200	8.586	8.985	6.872	0.324	6.614	7.235
ENSG00000116761	CTH	1.3	1.3E-07	6.6E-07	41.670	0.798	40.936	42.519	31.798	1.068	30.926	32.989
ENSG00000116874	WARS2	1.3	1.3E-04	3.7E-04	14.479	1.070	13.762	15.708	11.538	1.060	10.315	12.206
ENSG00000116918	TSNAX	1.3	4.9E-06	1.9E-05	18.834	1.635	16.966	20.003	14.445	1.880	12.760	16.473
ENSG00000117479	SLC19A2	1.3	3.4E-09	2.4E-08	53.595	1.454	52.250	55.138	41.388	1.806	39.337	42.741
ENSG00000117758	STX12	1.3	5.7E-04	1.4E-03	12.175	0.784	11.438	12.999	9.595	0.638	9.080	10.309
ENSG00000118197	DDX59	1.3	8.4E-06	3.0E-05	15.916	0.736	15.070	16.414	12.058	0.526	11.480	12.507
ENSG00000118257	NRP2	1.3	1.7E-05	5.8E-05	8.583	0.518	8.008	9.015	6.526	0.278	6.243	6.799
ENSG00000118707	TGIF2	1.3	1.2E-08	7.6E-08	69.213	2.965	65.846	71.432	53.313	1.174	51.979	54.192
ENSG00000119048	UBE2B	1.3	1.7E-07	8.6E-07	28.126	1.007	27.089	29.100	21.134	0.946	20.054	21.817
ENSG00000119314	PTBP3	1.3	1.2E-12	1.7E-11	95.688	2.361	93.126	97.778	71.519	3.366	67.825	74.413
ENSG00000119408	NEK6	1.3	1.3E-07	6.7E-07	19.734	0.183	19.628	19.946	14.812	0.794	14.190	15.707
ENSG00000119655	NPC2	1.3	1.1E-09	8.6E-09	89.151	1.598	87.448	90.619	69.204	3.604	65.676	72.879
ENSG00000119685	TTLL5	1.3	2.4E-07	1.2E-06	11.875	0.218	11.640	12.069	9.325	0.505	8.791	9.794
ENSG00000120051	CFAP58	1.3	1.8E-02	3.3E-02	2.098	0.196	1.876	2.244	1.567	0.141	1.422	1.704
ENSG00000120057	SFRP5	1.3	8.5E-02	1.3E-01	3.517	0.137	3.363	3.625	2.748	0.433	2.282	3.140
ENSG00000120063	GNA13	1.3	8.9E-09	5.8E-08	77.383	0.531	76.963	77.979	61.866	1.769	59.899	63.327
ENSG00000120327	PCDHB14	1.3	6.9E-03	1.4E-02	3.036	0.120	2.904	3.139	2.351	0.103	2.262	2.464
ENSG00000120539	MASTL	1.3	1.3E-11	1.6E-10	74.994	0.796	74.164	75.750	55.999	1.382	54.744	57.497
ENSG00000120699	EXOSC8	1.3	2.0E-09	1.5E-08	77.725	1.989	75.637	79.598	60.884	2.363	58.452	63.170
ENSG00000120820	GLT8D2	1.3	4.1E-03	8.5E-03	8.047	0.094	7.940	8.116	6.371	0.475	6.086	6.920
ENSG00000121211	MND1	1.3	1.1E-06	4.9E-06	53.423	2.326	50.769	55.109	40.197	1.627	39.077	42.064
ENSG00000121644	DESI2	1.3	1.5E-10	1.4E-09	132.759	1.980	130.556	134.390	100.054	3.486	96.260	103.117
ENSG00000121691	CAT	1.3	4.4E-07	2.0E-06	26.908	0.784	26.091	27.656	20.635	0.843	19.824	21.507
ENSG00000121879	PIK3CA	1.3	1.9E-06	7.7E-06	10.380	0.720	9.554	10.875	8.028	0.537	7.417	8.423
ENSG00000122406	RPL5	1.3	4.6E-12	5.9E-11	1493.889	27.807	1472.135	1525.219	1183.006	36.563	1141.126	1208.565
ENSG00000122678	POLM	1.3	1.8E-03	4.0E-03	8.297	0.844	7.561	9.219	6.621	0.264	6.320	6.807
ENSG00000122970	IFT81	1.3	8.2E-06	3.0E-05	15.365	1.340	13.837	16.340	12.285	0.638	11.902	13.022
ENSG00000123178	SPRYD7	1.3	1.5E-03	3.4E-03	7.479	0.956	6.377	8.088	5.809	0.591	5.270	6.440
ENSG00000124019	FAM124B	1.3	1.5E-02	2.7E-02	2.923	0.308	2.610	3.226	2.182	0.102	2.065	2.249
ENSG00000124523	SIRT5	1.3	1.9E-05	6.6E-05	10.852	0.329	10.652	11.232	8.258	0.459	7.750	8.643
ENSG00000124535	WRNIP1	1.3	3.6E-07	1.7E-06	37.645	0.842	36.759	38.435	29.453	2.645	26.410	31.198
ENSG00000124788	ATXN1	1.3	5.7E-03	1.1E-02	0.988	0.143	0.828	1.102	0.748	0.061	0.709	0.819
ENSG00000125378	BMP4	1.3	5.3E-02	8.3E-02	2.402	0.158	2.304	2.584	1.877	0.184	1.733	2.085
ENSG00000125457	MIF4GD	1.3	7.9E-04	1.9E-03	15.803	1.647	13.951	17.105	12.570	0.588	11.931	13.117
ENSG00000125648	SLC25A23	1.3	1.9E-07	9.6E-07	35.025	0.971	33.930	35.784	27.427	2.105	24.997	28.653
ENSG00000125691	RPL23	1.3	1.0E-10	9.7E-10	550.199	23.674	532.621	577.119	431.611	26.257	401.439	449.270
ENSG00000125744	RTN2	1.3	4.2E-05	1.3E-04	16.514	0.454	15.991	16.818	12.801	1.188	11.475	13.771
ENSG00000125966	MMP24	1.3	3.2E-03	6.8E-03	9.624	1.139	8.497	10.775	7.196	0.362	6.889	7.595
ENSG00000126603	GLIS2	1.3	6.3E-04	1.6E-03	10.037	0.405	9.736	10.497	7.894	1.274	6.501	9.001
ENSG00000126756	UXT	1.3	2.7E-06	1.1E-05	66.499	6.858	60.856	74.132	50.951	1.178	49.696	52.032
ENSG00000126803	HSPA2	1.3	1.1E-06	4.8E-06	22.326	1.428	20.706	23.405	17.512	0.187	17.385	17.727
ENSG00000127870	RNF6	1.3	6.3E-07	2.8E-06	14.565	0.762	13.695	15.108	10.901	0.683	10.446	11.699
ENSG00000127920	GNG11	1.3	2.4E-02	4.1E-02	5.367	0.711	4.749	6.144	4.266	0.664	3.704	4.999
ENSG00000128245	YWHAH	1.3	2.6E-12	3.5E-11	350.642	6.112	345.020	357.148	270.135	11.290	257.623	279.562
ENSG00000128294	TPST2	1.3	1.0E-08	6.6E-08	33.521	1.673	31.945	35.276	26.155	0.216	25.942	26.373
ENSG00000128394	APOBEC3F	1.3	7.9E-03	1.5E-02	4.803	0.892	4.185	5.825	3.716	0.099	3.636	3.827
ENSG00000128585	MKLN1	1.3	6.4E-09	4.3E-08	28.360	0.198	28.221	28.587	22.533	1.000	21.781	23.669
ENSG00000128739	SNRPN	1.3	6.8E-11	6.8E-10	231.182	9.896	223.256	242.273	176.523	6.541	168.971	180.358
ENSG00000128791	TWSG1	1.3	4.1E-08	2.3E-07	31.318	2.661	29.199	34.304	23.566	1.840	22.032	25.607
ENSG00000128881	TTBK2	1.3	1.1E-07	5.8E-07	11.087	0.776	10.193	11.586	8.596	0.341	8.213	8.868
ENSG00000129682	FGF13	1.3	1.4E-10	1.3E-09	16.666	0.280	16.345	16.858	12.609	0.443	12.119	12.982
ENSG00000129993	CBFA2T3	1.3	1.1E-01	1.6E-01	0.730	0.142	0.569	0.833	0.558	0.055	0.494	0.594
ENSG00000130224	LRCH2	1.3	8.0E-05	2.4E-04	9.936	0.899	8.924	10.645	7.705	0.781	7.027	8.559
ENSG00000130363	RSPH3	1.3	4.6E-03	9.3E-03	3.515	0.616	3.023	4.206	2.755	0.373	2.452	3.171
ENSG00000130402	ACTN4	1.3	4.8E-12	6.2E-11	204.987	6.101	201.297	212.029	158.264	7.347	150.810	165.500
ENSG00000130540	SULT4A1	1.3	9.2E-04	2.2E-03	9.928	0.955	9.156	10.996	7.728	0.465	7.323	8.241
ENSG00000130844	ZNF331	1.3	2.5E-07	1.2E-06	13.431	1.301	12.103	14.703	10.263	0.289	9.996	10.569
ENSG00000131374	TBC1D5	1.3	2.5E-09	1.8E-08	18.764	0.794	17.998	19.582	14.385	0.120	14.305	14.522
ENSG00000131389	SLC6A6	1.3	3.5E-12	4.6E-11	117.701	4.182	112.872	120.196	89.184	2.491	87.106	91.946
ENSG00000131435	PDLIM4	1.3	1.5E-03	3.4E-03	7.174	0.382	6.818	7.577	5.358	0.827	4.449	6.066
ENSG00000131471	AOC3	1.3	5.6E-02	8.9E-02	1.589	0.200	1.393	1.794	1.208	0.424	0.911	1.694
ENSG00000131480	AOC2	1.3	2.7E-03	5.8E-03	5.545	0.489	5.071	6.047	4.180	0.399	3.733	4.498
ENSG00000131725	WDR44	1.3	2.7E-07	1.3E-06	28.951	1.310	27.439	29.710	22.789	0.513	22.250	23.270
ENSG00000132406	TMEM128	1.3	2.9E-07	1.4E-06	47.613	1.131	46.833	48.910	36.315	1.853	34.450	38.155
ENSG00000132471	WBP2	1.3	7.8E-09	5.2E-08	27.334	0.532	26.845	27.900	20.806	1.225	19.456	21.848
ENSG00000132964	CDK8	1.3	2.7E-07	1.3E-06	40.172	1.961	38.079	41.968	31.907	1.290	30.487	33.007
ENSG00000132970	WASF3	1.3	7.7E-08	4.2E-07	38.100	0.905	37.296	39.081	30.365	1.229	28.979	31.321
ENSG00000133067	LGR6	1.3	8.6E-02	1.3E-01	1.024	0.085	0.931	1.098	0.758	0.175	0.610	0.951
ENSG00000133112	TPT1	1.3	1.5E-14	3.1E-13	680.374	2.401	677.706	682.364	523.919	9.187	517.046	534.353
ENSG00000133136	GNG5P2	1.3	9.9E-02	1.4E-01	18.045	0.154	17.896	18.203	13.504	2.488	10.867	15.810
ENSG00000133247	KMT5C	1.3	4.4E-06	1.7E-05	10.418	0.113	10.313	10.538	7.808	0.559	7.284	8.396
ENSG00000133874	RNF122	1.3	8.0E-05	2.4E-04	21.361	0.616	20.768	21.998	15.999	1.513	14.300	17.199
ENSG00000134825	TMEM258	1.3	1.0E-08	6.8E-08	82.799	3.510	80.455	86.834	65.218	2.736	63.014	68.280
ENSG00000134884	ARGLU1	1.3	2.4E-09	1.7E-08	151.203	10.537	140.293	161.323	119.000	4.589	114.171	123.303
ENSG00000135069	PSAT1	1.3	9.1E-11	8.9E-10	880.308	10.632	869.628	890.892	704.297	2.219	702.191	706.613
ENSG00000135317	SNX14	1.3	1.6E-05	5.4E-05	13.724	1.148	12.882	15.033	10.974	0.438	10.468	11.228
ENSG00000135333	EPHA7	1.3	1.1E-05	3.9E-05	7.625	0.378	7.209	7.946	5.842	0.217	5.639	6.072
ENSG00000135338	LCA5	1.3	3.9E-04	1.0E-03	5.292	0.399	4.983	5.743	4.113	0.165	3.937	4.263
ENSG00000135631	RAB11FIP5	1.3	3.2E-05	1.0E-04	12.497	1.076	11.584	13.684	9.862	1.025	8.827	10.876
ENSG00000136273	HUS1	1.3	4.2E-05	1.3E-04	14.950	0.957	14.300	16.049	11.670	0.526	11.309	12.273
ENSG00000136854	STXBP1	1.3	8.1E-08	4.4E-07	17.031	0.604	16.362	17.538	13.163	0.368	12.785	13.519
ENSG00000136868	SLC31A1	1.3	7.7E-11	7.6E-10	67.334	1.119	66.562	68.617	52.579	0.434	52.088	52.915
ENSG00000137094	DNAJB6	1.3	3.5E-07	1.7E-06	33.786	2.499	30.919	35.502	26.972	1.277	25.941	28.400
ENSG00000137133	HINT2	1.3	1.2E-04	3.4E-04	18.387	1.489	17.157	20.043	14.251	1.080	13.580	15.497
ENSG00000137393	RNF144B	1.3	7.0E-03	1.4E-02	5.389	0.254	5.100	5.578	4.315	0.416	3.926	4.754
ENSG00000137460	FHDC1	1.3	2.3E-07	1.1E-06	31.146	0.383	30.861	31.581	24.973	0.609	24.535	25.667
ENSG00000137573	SULF1	1.3	2.3E-05	7.6E-05	9.607	0.625	9.178	10.324	7.628	0.505	7.149	8.155
ENSG00000137575	SDCBP	1.3	8.6E-09	5.6E-08	96.076	1.064	94.850	96.767	75.403	7.071	68.424	82.562
ENSG00000137720	C11orf1	1.3	3.5E-04	9.2E-04	9.550	0.386	9.105	9.798	7.458	0.792	6.899	8.365
ENSG00000137770	CTDSPL2	1.3	6.6E-08	3.6E-07	31.021	1.157	29.928	32.233	24.563	2.332	22.179	26.839
ENSG00000138032	PPM1B	1.3	2.3E-11	2.6E-10	88.348	5.189	82.857	93.169	67.386	2.330	65.503	69.992
ENSG00000138100	TRIM54	1.3	4.4E-02	7.1E-02	3.865	0.864	3.127	4.816	3.002	0.425	2.516	3.306
ENSG00000138286	FAM149B1	1.3	2.3E-06	9.2E-06	11.161	0.248	10.997	11.447	8.760	0.173	8.561	8.877
ENSG00000138439	FAM117B	1.3	2.9E-07	1.4E-06	27.712	1.602	25.862	28.662	21.795	0.495	21.299	22.289
ENSG00000138738	PRDM5	1.3	1.9E-07	9.5E-07	13.490	0.582	12.819	13.849	10.103	0.768	9.356	10.891
ENSG00000138834	MAPKBIP3	1.3	4.6E-09	3.2E-08	13.294	0.452	12.912	13.794	9.973	0.585	9.344	10.501
ENSG00000139160	ETFBKMT	1.3	1.1E-01	1.6E-01	0.652	0.029	0.629	0.685	0.484	0.080	0.427	0.576
ENSG00000139239		1.3	3.1E-11	3.3E-10	1929.483	15.861	1911.329	1940.660	1542.978	30.409	1516.602	1576.240
ENSG00000139318	DUSP6	1.3	2.0E-09	1.5E-08	328.590	6.291	323.858	335.729	246.279	4.469	243.668	251.440
ENSG00000139354	GAS2L3	1.3	6.6E-05	2.0E-04	9.097	0.408	8.627	9.359	7.267	0.433	6.839	7.705
ENSG00000139641	ESYT1	1.3	2.0E-10	1.8E-09	90.177	1.576	88.541	91.685	69.474	3.260	66.169	72.687
ENSG00000139668	WDFY2	1.3	4.0E-09	2.8E-08	14.182	0.242	13.923	14.401	11.111	0.655	10.401	11.692
ENSG00000139921	TMX1	1.3	2.8E-08	1.7E-07	99.549	4.352	94.620	102.861	78.367	7.527	71.348	86.315
ENSG00000140199	SLC12A6	1.3	7.0E-07	3.1E-06	8.086	0.232	7.833	8.288	6.146	0.122	6.007	6.236
ENSG00000140400	MAN2C1	1.3	3.1E-07	1.5E-06	11.027	1.014	9.877	11.790	8.354	0.004	8.351	8.358
ENSG00000140416	TPM1	1.3	2.0E-12	2.7E-11	91.616	3.862	87.331	94.829	70.316	1.114	69.207	71.434
ENSG00000140459	CYP11A1	1.3	1.4E-02	2.6E-02	3.238	0.570	2.607	3.714	2.480	0.122	2.383	2.618
ENSG00000140543	DET1	1.3	4.6E-04	1.2E-03	4.376	0.546	3.984	5.000	3.284	0.440	3.016	3.791
ENSG00000140905	GCSH	1.3	7.2E-06	2.7E-05	30.581	1.594	28.985	32.173	24.338	1.739	22.616	26.092
ENSG00000141446	ESCO1	1.3	7.1E-10	5.8E-09	52.778	3.323	49.095	55.552	39.395	2.433	37.340	42.081
ENSG00000141858	SAMD1	1.3	1.1E-05	4.0E-05	50.883	0.885	50.353	51.904	40.772	4.086	36.604	44.771
ENSG00000142156	COL6A1	1.3	6.6E-11	6.6E-10	61.843	1.947	59.596	63.017	47.594	1.581	46.391	49.384
ENSG00000142178	SIK1	1.3	3.1E-03	6.6E-03	4.661	0.145	4.496	4.770	3.668	0.170	3.503	3.841
ENSG00000142235	LMTK3	1.3	1.5E-03	3.4E-03	6.466	0.843	5.680	7.357	5.046	0.507	4.464	5.392
ENSG00000142534	RPS11	1.3	3.4E-13	5.4E-12	1911.577	39.138	1886.792	1956.696	1488.328	34.761	1453.631	1523.153
ENSG00000142657	PGD	1.3	1.2E-11	1.4E-10	397.077	9.439	387.043	405.779	315.261	3.922	311.096	318.884
ENSG00000142669	SH3BGRL3	1.3	1.8E-07	8.9E-07	79.445	2.208	77.701	81.928	62.069	3.479	58.425	65.356
ENSG00000142676	RPL11	1.3	4.7E-12	6.1E-11	1551.241	11.744	1540.227	1563.600	1237.781	24.300	1218.492	1265.074
ENSG00000142694	EVA1B	1.3	1.7E-03	3.8E-03	25.318	1.662	24.089	27.210	19.687	3.332	17.763	23.534
ENSG00000142698	C1orf94	1.3	6.8E-04	1.7E-03	11.910	0.462	11.504	12.413	9.084	0.724	8.405	9.846
ENSG00000142949	PTPRF	1.3	3.9E-11	4.1E-10	128.669	1.267	127.418	129.952	99.988	5.643	93.773	104.779
ENSG00000143178	TBX19	1.3	1.0E-01	1.5E-01	1.093	0.255	0.801	1.270	0.824	0.096	0.766	0.935
ENSG00000143303	RRNAD1	1.3	6.2E-05	1.9E-04	14.268	1.639	12.653	15.930	10.706	1.395	9.169	11.890
ENSG00000143382	ADAMTSL4	1.3	1.3E-02	2.3E-02	1.612	0.185	1.459	1.817	1.207	0.197	0.984	1.357
ENSG00000143614	GATAD2B	1.3	3.9E-07	1.8E-06	12.095	0.738	11.563	12.937	9.473	0.346	9.077	9.713
ENSG00000143622	RIT1	1.3	3.0E-07	1.4E-06	22.179	0.622	21.703	22.883	16.601	1.357	15.588	18.143
ENSG00000143630	HCN3	1.3	1.3E-03	2.9E-03	5.446	0.264	5.210	5.730	4.133	0.478	3.593	4.503
ENSG00000143674	MAP3K21	1.3	7.3E-09	4.8E-08	30.225	1.343	28.702	31.236	22.680	0.543	22.067	23.099
ENSG00000144136	SLC20A1	1.3	1.9E-10	1.7E-09	64.161	1.408	63.101	65.758	49.950	0.774	49.083	50.570
ENSG00000144468	RHBDD1	1.3	4.1E-08	2.3E-07	15.180	0.270	14.952	15.479	11.294	0.675	10.840	12.069
ENSG00000144560	VGLL4	1.3	9.0E-09	5.9E-08	24.796	0.704	24.316	25.604	19.435	0.170	19.241	19.556
ENSG00000144596	GRIP2	1.3	2.7E-02	4.5E-02	0.935	0.066	0.896	1.011	0.732	0.050	0.674	0.764
ENSG00000144730	IL17RD	1.3	4.0E-09	2.8E-08	95.703	1.793	93.765	97.302	71.864	3.129	69.987	75.476
ENSG00000144747	TMF1	1.3	2.4E-08	1.5E-07	19.868	1.764	18.458	21.847	15.015	0.348	14.671	15.367
ENSG00000144893	MED12L	1.3	6.8E-06	2.5E-05	6.158	0.064	6.085	6.200	4.682	0.277	4.516	5.002
ENSG00000144935	TRPC1	1.3	4.5E-06	1.7E-05	15.147	1.146	13.952	16.238	11.576	0.397	11.154	11.942
ENSG00000145391	SETD7	1.3	1.5E-05	5.1E-05	2.910	0.100	2.820	3.017	2.167	0.061	2.099	2.218
ENSG00000145439	CBR4	1.3	7.8E-09	5.2E-08	36.304	0.958	35.219	37.035	28.363	0.425	27.966	28.812
ENSG00000145592	RPL37	1.3	5.1E-11	5.3E-10	333.939	10.356	324.820	345.198	267.092	6.891	259.277	272.296
ENSG00000145675	PIK3R1	1.3	9.1E-05	2.7E-04	4.805	0.065	4.731	4.847	3.822	0.208	3.620	4.035
ENSG00000145819	ARHGAP26	1.3	2.0E-08	1.2E-07	8.128	0.338	7.844	8.501	6.047	0.225	5.788	6.183
ENSG00000146587	RBAK	1.3	9.7E-09	6.3E-08	18.591	0.726	17.786	19.194	14.088	0.060	14.029	14.140
ENSG00000146700	SSC4D	1.3	1.2E-03	2.9E-03	13.766	0.990	12.761	14.741	10.977	0.724	10.324	11.755
ENSG00000147144	CCDC120	1.3	1.5E-04	4.2E-04	7.809	0.225	7.620	8.058	6.149	0.416	5.816	6.616
ENSG00000147162	OGT	1.3	8.3E-09	5.5E-08	87.692	6.065	80.903	92.574	69.284	4.626	63.942	72.008
ENSG00000147403	RPL10	1.3	2.5E-10	2.2E-09	271.379	13.072	262.426	286.380	214.536	8.958	207.116	224.488
ENSG00000147862	NFIB	1.3	1.1E-04	3.2E-04	2.335	0.072	2.285	2.417	1.777	0.042	1.729	1.806
ENSG00000148843	LIN7C	1.3	1.5E-04	4.3E-04	28.726	2.580	27.024	31.695	22.056	1.989	20.070	24.046
ENSG00000149476	TKFC	1.3	2.5E-06	1.0E-05	11.049	0.267	10.793	11.326	8.672	0.331	8.481	9.055
ENSG00000149577	SIDT2	1.3	1.7E-05	6.0E-05	4.764	0.360	4.532	5.178	3.567	0.116	3.446	3.677
ENSG00000149596	JPH2	1.3	2.6E-02	4.5E-02	1.715	0.124	1.636	1.857	1.316	0.118	1.183	1.410
ENSG00000149743	TRPT1	1.3	3.1E-05	1.0E-04	22.221	0.114	22.132	22.349	17.259	1.140	16.055	18.322
ENSG00000150093	ITGB1	1.3	1.8E-11	2.0E-10	340.961	1.618	339.109	342.101	266.534	6.877	259.128	272.718
ENSG00000150977	RILPL2	1.3	1.1E-02	2.1E-02	2.472	0.115	2.339	2.539	1.937	0.202	1.706	2.083
ENSG00000151067	CACNA1C	1.3	9.7E-02	1.4E-01	0.413	0.127	0.298	0.549	0.327	0.061	0.256	0.364
ENSG00000151135	TMEM263	1.3	1.2E-04	3.4E-04	13.495	0.969	12.376	14.060	10.751	0.686	10.124	11.484
ENSG00000151151	IPMK	1.3	3.2E-06	1.3E-05	14.855	1.084	13.741	15.906	11.453	1.053	10.750	12.664
ENSG00000151687	ANKAR	1.3	8.2E-02	1.2E-01	1.173	0.350	0.831	1.531	0.925	0.040	0.900	0.970
ENSG00000151789	ZNF385D	1.3	4.0E-03	8.2E-03	1.463	0.043	1.416	1.502	1.116	0.256	0.891	1.395
ENSG00000151846	PABPC3	1.3	1.0E-06	4.4E-06	27.147	2.000	25.040	29.021	21.033	0.196	20.807	21.156
ENSG00000152242	C18orf25	1.3	1.2E-08	7.5E-08	15.817	0.540	15.391	16.425	11.884	0.806	10.965	12.399
ENSG00000152409	JMY	1.3	1.8E-08	1.1E-07	15.719	0.165	15.528	15.824	11.952	0.834	11.024	12.641
ENSG00000152684	PELO	1.3	1.5E-08	9.2E-08	29.363	0.390	29.112	29.812	22.381	0.331	22.047	22.709
ENSG00000153044	CENPH	1.3	4.6E-10	3.9E-09	140.578	4.218	135.709	143.078	108.837	0.689	108.186	109.559
ENSG00000153157	SYCP2L	1.3	8.1E-03	1.6E-02	4.154	0.644	3.506	4.793	3.139	0.606	2.468	3.648
ENSG00000153317	ASAP1	1.3	7.7E-08	4.2E-07	21.219	0.426	20.915	21.706	16.733	0.715	16.008	17.438
ENSG00000153774	CFDP1	1.3	6.7E-10	5.5E-09	80.488	2.873	78.786	83.805	61.186	1.961	59.232	63.154
ENSG00000153879	CEBPG	1.3	2.9E-09	2.1E-08	65.737	1.294	64.280	66.753	52.591	1.016	51.472	53.456
ENSG00000153933	DGKE	1.3	1.8E-04	5.0E-04	4.997	0.208	4.786	5.202	4.009	0.053	3.948	4.041
ENSG00000154240	CEP112	1.3	1.4E-02	2.5E-02	2.053	0.184	1.841	2.174	1.630	0.254	1.372	1.881
ENSG00000154310	TNIK	1.3	6.3E-11	6.4E-10	71.471	3.406	69.088	75.373	55.672	0.317	55.326	55.950
ENSG00000154710	RABGEF1	1.3	9.3E-02	1.4E-01	0.819	0.238	0.658	1.093	0.613	0.128	0.472	0.723
ENSG00000154721	JAM2	1.3	4.2E-03	8.6E-03	4.175	0.252	3.906	4.405	3.133	0.447	2.620	3.437
ENSG00000154727	GABPA	1.3	1.6E-07	8.2E-07	36.386	2.414	33.619	38.061	27.940	1.830	25.938	29.525
ENSG00000154845	PPP4R1	1.3	1.1E-09	8.6E-09	52.420	0.783	51.899	53.321	41.312	0.775	40.602	42.138
ENSG00000155189	AGPAT5	1.3	1.9E-10	1.7E-09	124.417	5.965	119.749	131.137	95.102	4.922	91.005	100.561
ENSG00000155313	USP25	1.3	6.7E-11	6.8E-10	42.490	2.126	40.269	44.507	32.390	0.415	32.052	32.852
ENSG00000155792	DEPTOR	1.3	2.9E-02	4.9E-02	4.493	1.305	3.414	5.943	3.477	0.153	3.303	3.588
ENSG00000155849	ELMO1	1.3	1.5E-03	3.5E-03	3.366	0.189	3.226	3.581	2.634	0.156	2.468	2.779
ENSG00000156076	WIF1	1.3	6.1E-02	9.5E-02	4.346	1.472	3.194	6.005	3.405	0.608	2.879	4.070
ENSG00000156171	DRAM2	1.3	1.2E-08	7.6E-08	38.798	1.234	37.382	39.647	29.526	0.394	29.157	29.941
ENSG00000156172	C8orf37	1.3	2.0E-02	3.6E-02	3.700	0.460	3.262	4.179	2.903	0.534	2.572	3.519
ENSG00000156574	NODAL	1.3	6.1E-03	1.2E-02	12.419	0.521	11.842	12.855	9.549	2.114	7.225	11.357
ENSG00000156671	SAMD8	1.3	1.2E-06	5.3E-06	19.982	0.565	19.629	20.634	16.012	0.779	15.526	16.911
ENSG00000156869	FRRS1	1.3	3.6E-03	7.6E-03	5.794	0.513	5.330	6.345	4.546	0.371	4.117	4.762
ENSG00000156958	GALK2	1.3	3.1E-05	1.0E-04	5.504	0.244	5.247	5.733	4.126	0.413	3.678	4.490
ENSG00000157045	NTAN1	1.3	1.0E-05	3.6E-05	25.806	1.975	23.577	27.338	20.324	0.495	19.761	20.692
ENSG00000158019	BABAM2	1.3	1.2E-04	3.3E-04	15.288	0.238	15.133	15.562	11.861	1.202	10.707	13.106
ENSG00000158863	FAM160B2	1.3	1.7E-03	3.8E-03	2.494	0.300	2.237	2.824	1.889	0.223	1.637	2.059
ENSG00000158887	MPZ	1.3	3.0E-02	5.0E-02	5.309	0.192	5.088	5.426	4.237	0.576	3.826	4.895
ENSG00000159200	RCAN1	1.3	3.3E-04	8.6E-04	4.868	0.437	4.416	5.289	3.705	0.335	3.355	4.023
ENSG00000159648	TEPP	1.3	9.9E-02	1.5E-01	1.903	0.061	1.833	1.940	1.507	0.163	1.351	1.676
ENSG00000159685	CHCHD6	1.3	1.5E-06	6.2E-06	28.245	1.800	26.554	30.137	21.308	1.555	19.947	23.003
ENSG00000159884	CCDC107	1.3	6.1E-03	1.2E-02	13.956	0.539	13.351	14.388	10.947	1.770	9.096	12.623
ENSG00000159905	ZNF221	1.3	2.1E-02	3.6E-02	3.961	0.268	3.664	4.187	3.039	0.221	2.820	3.262
ENSG00000160111	CPAMD8	1.3	5.4E-03	1.1E-02	2.454	0.042	2.406	2.482	1.963	0.215	1.726	2.144
ENSG00000160117	ANKLE1	1.3	8.1E-04	2.0E-03	8.000	0.600	7.395	8.596	6.184	0.476	5.673	6.615
ENSG00000160284	SPATC1L	1.3	4.1E-04	1.1E-03	14.965	0.367	14.753	15.389	11.734	1.126	10.777	12.976
ENSG00000160469	BRSK1	1.3	1.2E-04	3.4E-04	10.136	0.544	9.662	10.729	7.804	0.469	7.492	8.343
ENSG00000160471	COX6B2	1.3	9.8E-02	1.4E-01	1.179	0.175	0.979	1.304	0.910	0.072	0.848	0.989
ENSG00000160539	PLPP7	1.3	1.5E-02	2.8E-02	4.220	0.597	3.674	4.857	3.185	0.391	2.806	3.587
ENSG00000160767	FAM189B	1.3	7.5E-06	2.8E-05	25.757	1.774	24.536	27.792	20.471	1.543	18.696	21.492
ENSG00000160783	PMF1	1.3	3.8E-06	1.5E-05	26.170	0.281	25.898	26.460	20.084	0.213	19.845	20.252
ENSG00000160867	FGFR4	1.3	1.4E-08	8.5E-08	48.103	0.957	47.261	49.143	37.415	0.687	36.650	37.980
ENSG00000160948	VPS28	1.3	3.1E-06	1.2E-05	16.357	0.559	15.807	16.925	12.370	0.742	11.786	13.205
ENSG00000161558	TMEM143	1.3	1.8E-03	4.1E-03	6.154	0.317	5.797	6.403	4.766	0.265	4.529	5.052
ENSG00000161654	LSM12	1.3	8.7E-08	4.7E-07	69.736	2.015	68.008	71.949	55.634	2.360	52.923	57.234
ENSG00000161912	ADCY10P1	1.3	4.5E-03	9.3E-03	3.740	0.090	3.641	3.818	2.981	0.196	2.792	3.183
ENSG00000162433	AK4	1.3	1.1E-03	2.6E-03	122.665	3.603	118.724	125.790	96.491	2.362	94.630	99.149
ENSG00000162775	RBM15	1.3	8.1E-07	3.6E-06	21.359	1.353	20.260	22.871	16.405	0.851	15.805	17.379
ENSG00000163017	ACTG2	1.3	1.1E-01	1.6E-01	2.097	0.435	1.620	2.470	1.663	0.161	1.503	1.825
ENSG00000163170	BOLA3	1.3	3.5E-07	1.7E-06	37.714	2.478	36.105	40.568	28.842	0.876	27.869	29.566
ENSG00000163293	NIPAL1	1.3	1.7E-03	3.8E-03	5.830	0.326	5.469	6.104	4.666	0.545	4.068	5.136
ENSG00000163346	PBXIP1	1.3	5.5E-08	3.1E-07	33.491	0.484	33.193	34.050	25.776	0.827	25.228	26.728
ENSG00000163428	LRRC58	1.3	4.9E-08	2.8E-07	26.648	0.856	25.660	27.151	20.583	1.298	19.308	21.903
ENSG00000163513	TGFBR2	1.3	5.1E-06	1.9E-05	15.175	0.191	15.023	15.389	11.919	0.316	11.566	12.175
ENSG00000163597	SNHG16	1.3	2.3E-10	2.1E-09	59.838	2.203	57.941	62.255	45.872	2.314	43.716	48.317
ENSG00000163637	PRICKLE2	1.3	2.4E-04	6.5E-04	2.405	0.155	2.227	2.512	1.852	0.086	1.752	1.907
ENSG00000163882	POLR2H	1.3	8.8E-09	5.8E-08	69.122	2.286	66.483	70.479	54.389	1.095	53.270	55.458
ENSG00000164008	C1orf50	1.3	1.3E-01	1.8E-01	2.195	0.467	1.706	2.637	1.642	0.483	1.101	2.032
ENSG00000164104	HMGB2	1.3	2.1E-10	1.9E-09	354.140	7.424	346.353	361.139	281.957	5.273	275.869	285.137
ENSG00000164142	FAM160A1	1.3	1.5E-07	7.8E-07	28.565	1.288	27.274	29.851	22.425	0.724	22.007	23.261
ENSG00000164187	LMBRD2	1.3	6.5E-05	2.0E-04	6.785	0.892	5.957	7.729	5.190	0.233	5.043	5.459
ENSG00000164414	SLC35A1	1.3	3.5E-03	7.4E-03	7.999	0.445	7.687	8.303	6.010	0.185	5.885	6.223
ENSG00000164615	CAMLG	1.3	7.7E-08	4.2E-07	76.152	4.729	72.982	81.587	58.796	2.304	57.288	61.448
ENSG00000164938	TP53INP1	1.3	5.6E-05	1.7E-04	18.757	0.774	17.893	19.389	14.192	1.077	12.949	14.840
ENSG00000165185	KIAA1958	1.3	4.2E-07	2.0E-06	15.497	0.932	14.794	16.555	12.208	0.860	11.517	13.171
ENSG00000165288	BRWD3	1.3	7.2E-09	4.8E-08	25.488	1.455	23.869	26.687	19.947	0.613	19.593	20.655
ENSG00000165704	HPRT1	1.3	2.6E-07	1.3E-06	127.803	4.191	124.638	132.556	101.266	3.368	98.519	105.024
ENSG00000165775	FUNDC2	1.3	1.4E-07	7.2E-07	14.074	0.494	13.511	14.431	11.134	0.282	10.834	11.392
ENSG00000165995	CACNB2	1.3	8.6E-02	1.3E-01	1.141	0.314	0.783	1.366	0.884	0.121	0.748	0.980
ENSG00000165997	ARL5B	1.3	3.7E-07	1.7E-06	80.266	3.531	76.402	83.325	63.512	5.008	60.279	69.281
ENSG00000166025	AMOTL1	1.3	4.7E-11	4.9E-10	35.485	0.990	34.631	36.570	27.215	0.705	26.437	27.812
ENSG00000166192	SENP8	1.3	1.2E-02	2.2E-02	4.252	0.682	3.469	4.716	3.420	0.174	3.229	3.571
ENSG00000166333	ILK	1.3	1.6E-11	1.9E-10	95.377	3.555	91.377	98.174	70.860	2.957	68.802	74.248
ENSG00000166436	TRIM66	1.3	4.6E-04	1.2E-03	2.234	0.183	2.126	2.445	1.702	0.088	1.640	1.803
ENSG00000166482	MFAP4	1.3	9.6E-03	1.8E-02	5.912	1.211	4.757	7.172	4.410	0.308	4.089	4.704
ENSG00000166750	SLFN5	1.3	2.0E-03	4.5E-03	3.078	0.250	2.811	3.304	2.437	0.119	2.322	2.559
ENSG00000166881	NEMP1	1.3	1.2E-09	9.5E-09	59.298	2.316	56.813	61.396	47.105	0.118	47.036	47.242
ENSG00000166965	RCCD1	1.3	6.7E-06	2.5E-05	9.959	0.330	9.629	10.290	7.531	0.501	7.140	8.096
ENSG00000167377	ZNF23	1.3	6.3E-02	9.7E-02	1.047	0.012	1.034	1.059	0.798	0.237	0.617	1.067
ENSG00000167460	TPM4	1.3	2.1E-13	3.6E-12	278.102	6.989	271.804	285.621	216.130	1.420	214.527	217.228
ENSG00000167654	ATCAY	1.3	6.8E-07	3.0E-06	27.370	1.644	25.474	28.396	21.588	0.814	20.812	22.437
ENSG00000167766	ZNF83	1.3	4.5E-07	2.1E-06	10.431	1.114	9.218	11.407	7.814	0.348	7.547	8.207
ENSG00000167992	VWCE	1.3	1.1E-02	2.1E-02	3.932	0.248	3.647	4.096	3.032	0.678	2.452	3.777
ENSG00000168003	SLC3A2	1.3	3.6E-12	4.7E-11	221.862	4.800	216.905	226.488	172.215	5.850	167.232	178.656
ENSG00000168028	RPSA	1.3	1.0E-10	9.8E-10	323.334	14.795	313.502	340.349	257.213	7.128	252.933	265.442
ENSG00000168090	COPS6	1.3	1.6E-08	9.7E-08	102.930	6.449	96.345	109.235	81.516	3.354	77.931	84.577
ENSG00000168264	IRF2BP2	1.3	1.7E-08	1.1E-07	34.049	1.618	32.687	35.838	25.818	0.609	25.164	26.369
ENSG00000168566	SNRNP48	1.3	1.0E-07	5.3E-07	25.705	1.017	24.705	26.739	19.777	1.224	18.365	20.549
ENSG00000168769	TET2	1.3	5.3E-03	1.1E-02	1.154	0.084	1.062	1.227	0.896	0.030	0.873	0.930

ENSG00000168772	CXXC4	1.3	3.6E-02	5.9E-02	4.264	0.487	3.714	4.642	3.382	0.735	2.875	4.225
ENSG00000168917	SLC35G2	1.3	2.6E-03	5.7E-03	4.627	0.443	4.140	5.008	3.554	0.303	3.356	3.903
ENSG00000168944	CEP120	1.3	1.4E-06	6.0E-06	19.538	0.918	18.551	20.367	15.606	0.180	15.399	15.728
ENSG00000168970	JMJD7-PLA2G	1.3	1.1E-02	2.1E-02	1.296	0.126	1.178	1.429	0.977	0.175	0.805	1.155
ENSG00000169100	SLC25A6	1.3	6.1E-11	6.2E-10	701.964	28.100	670.986	725.810	549.019	31.046	517.828	579.918
ENSG00000169398	PTK2	1.3	1.1E-11	1.3E-10	56.482	0.455	55.957	56.768	44.337	0.451	43.870	44.770
ENSG00000169499	PLEKHA2	1.3	4.4E-03	9.0E-03	5.249	0.842	4.327	5.979	3.966	0.190	3.801	4.174
ENSG00000169599	NFU1	1.3	5.3E-07	2.4E-06	38.879	0.573	38.218	39.231	29.977	0.742	29.120	30.413
ENSG00000169621	APLF	1.3	3.3E-03	7.0E-03	3.443	0.280	3.137	3.685	2.732	0.353	2.369	3.074
ENSG00000169660	HEXDC	1.3	4.5E-04	1.2E-03	6.870	0.460	6.449	7.361	5.348	0.267	5.147	5.651
ENSG00000169760	NLGN1	1.3	5.4E-04	1.4E-03	7.955	0.710	7.240	8.659	6.068	0.362	5.765	6.470
ENSG00000169964	TMEM42	1.3	1.1E-04	3.3E-04	11.183	0.736	10.634	12.020	8.642	0.395	8.185	8.872
ENSG00000170248	PDCD6IP	1.3	1.0E-10	9.8E-10	54.966	1.098	53.835	56.027	43.352	1.756	41.416	44.840
ENSG00000170275	CRTAP	1.3	2.5E-10	2.2E-09	66.819	0.460	66.432	67.328	52.376	2.449	49.553	53.927
ENSG00000170417	TMEM182	1.3	6.8E-03	1.3E-02	1.913	0.029	1.881	1.938	1.424	0.136	1.281	1.553
ENSG00000170456	DENND5B	1.3	3.3E-07	1.6E-06	9.268	0.140	9.185	9.430	7.110	0.287	6.867	7.427
ENSG00000170647	TMEM133	1.3	3.0E-03	6.3E-03	11.861	0.201	11.697	12.086	9.190	1.797	7.122	10.371
ENSG00000170852	KBTBD2	1.3	9.1E-10	7.3E-09	35.398	0.448	34.881	35.673	26.580	0.929	25.648	27.506
ENSG00000170889	RPS9	1.3	2.5E-11	2.7E-10	463.431	21.284	439.139	478.807	364.829	6.169	357.721	368.792
ENSG00000170955	CAVIN3	1.3	3.7E-04	9.6E-04	18.701	0.895	17.688	19.386	14.445	0.564	14.036	15.088
ENSG00000171055	FEZ2	1.3	8.3E-09	5.5E-08	17.349	0.388	17.006	17.770	13.357	0.205	13.166	13.575
ENSG00000171148	TADA3	1.3	2.2E-09	1.6E-08	49.609	0.800	49.068	50.528	37.497	2.029	36.324	39.839
ENSG00000171160	MORN4	1.3	6.9E-06	2.6E-05	24.204	1.404	22.612	25.265	18.321	2.019	16.404	20.428
ENSG00000171174	RBKS	1.3	6.1E-02	9.5E-02	1.677	0.509	1.177	2.194	1.239	0.236	1.047	1.503
ENSG00000171435	KSR2	1.3	4.5E-02	7.3E-02	0.698	0.041	0.674	0.746	0.559	0.070	0.496	0.634
ENSG00000171444	MCC	1.3	1.4E-05	5.0E-05	8.673	0.253	8.474	8.957	6.824	0.199	6.637	7.033
ENSG00000171467	ZNF318	1.3	8.6E-10	6.9E-09	19.663	0.795	18.747	20.155	14.826	0.677	14.116	15.464
ENSG00000171502	COL24A1	1.3	9.3E-03	1.8E-02	1.020	0.136	0.907	1.171	0.754	0.035	0.715	0.784
ENSG00000171735	CAMTA1	1.3	2.7E-06	1.1E-05	14.896	0.552	14.307	15.402	11.899	0.601	11.376	12.556
ENSG00000171885	AQP4	1.3	8.7E-02	1.3E-01	1.415	0.346	1.137	1.802	1.127	0.186	0.962	1.328
ENSG00000172007	RAB33B	1.3	3.2E-06	1.3E-05	12.599	0.309	12.279	12.895	9.445	0.861	8.462	10.066
ENSG00000172062	SMN1	1.3	1.0E-07	5.5E-07	41.713	1.464	40.030	42.686	32.254	1.457	31.262	33.926
ENSG00000172115	CYCS	1.3	1.3E-08	8.1E-08	251.144	5.223	247.952	257.172	200.709	15.181	185.580	215.942
ENSG00000172348	RCAN2	1.3	2.1E-05	7.0E-05	15.312	0.451	14.844	15.743	11.790	0.995	10.910	12.870
ENSG00000172663	TMEM134	1.3	4.1E-04	1.1E-03	7.086	0.443	6.588	7.438	5.467	0.901	4.694	6.457
ENSG00000172732	MUS81	1.3	2.2E-06	9.0E-06	11.432	0.464	10.936	11.857	8.531	0.545	8.087	9.139
ENSG00000172889	EGFL7	1.3	4.3E-06	1.6E-05	29.379	0.675	28.675	30.019	22.648	1.836	20.792	24.463
ENSG00000173207	CKS1B	1.3	6.0E-10	4.9E-09	139.421	3.279	136.747	143.080	108.253	5.324	104.753	114.380
ENSG00000173210	ABLIM3	1.3	1.3E-01	1.9E-01	0.652	0.122	0.512	0.726	0.498	0.141	0.406	0.661
ENSG00000173273	TNKS	1.3	3.9E-13	6.2E-12	29.243	0.445	28.735	29.567	21.851	0.415	21.407	22.230
ENSG00000173349	SFT2D3	1.3	3.2E-05	1.0E-04	12.727	0.254	12.473	12.981	9.859	0.551	9.425	10.478
ENSG00000173848	NET1	1.3	5.9E-11	6.0E-10	122.747	4.116	118.008	125.431	93.150	4.950	87.729	97.430
ENSG00000174032	SLC25A30	1.3	2.7E-05	9.0E-05	8.588	0.556	8.110	9.198	6.694	0.488	6.148	7.085
ENSG00000174206	C12orf66	1.3	1.1E-04	3.2E-04	6.210	0.290	5.980	6.535	4.714	0.495	4.168	5.132
ENSG00000174748	RPL15	1.3	3.2E-14	6.3E-13	439.001	8.440	430.806	447.666	333.671	13.285	321.049	347.532
ENSG00000175279	CENPS	1.3	7.2E-02	1.1E-01	3.176	0.232	2.987	3.434	2.454	0.504	1.882	2.830
ENSG00000175536	LIPT2	1.3	3.1E-02	5.1E-02	8.986	1.138	7.795	10.062	7.135	0.688	6.355	7.699
ENSG00000176102	CSTF3	1.3	1.3E-08	8.4E-08	67.506	3.479	63.694	70.511	53.704	1.186	52.387	54.687
ENSG00000176209	SMIM19	1.3	2.4E-07	1.2E-06	23.915	1.444	22.931	25.573	17.911	1.447	16.703	19.515
ENSG00000176720	BOK	1.3	2.2E-08	1.3E-07	59.289	1.070	58.091	60.150	45.392	3.260	41.691	47.834
ENSG00000177410	ZFAS1	1.3	6.2E-08	3.4E-07	64.976	3.208	61.367	67.506	50.322	1.999	48.139	52.064
ENSG00000177565	TBL1XR1	1.3	2.1E-11	2.3E-10	39.352	1.921	37.180	40.831	29.933	0.708	29.140	30.501
ENSG00000177707	NECTIN3	1.3	2.3E-04	6.2E-04	35.131	2.393	32.522	37.225	27.101	1.824	26.042	29.207
ENSG00000177854	TMEM187	1.3	7.7E-03	1.5E-02	7.922	1.511	6.749	9.627	6.009	0.542	5.518	6.591
ENSG00000178035	IMPDH2	1.3	6.8E-15	1.5E-13	476.964	5.107	471.170	480.812	354.908	8.504	349.195	364.682
ENSG00000178429	RPS3AP5	1.3	3.3E-10	2.9E-09	414.675	27.369	386.097	440.648	309.982	18.565	290.558	327.548
ENSG00000178467	P4HTM	1.3	1.8E-04	5.0E-04	4.657	0.198	4.540	4.886	3.583	0.442	3.073	3.857
ENSG00000178567	EPM2AIP1	1.3	4.6E-09	3.2E-08	21.364	1.853	19.393	23.071	16.274	0.095	16.177	16.367
ENSG00000178715		1.3	2.7E-03	5.8E-03	70.853	7.603	62.137	76.125	55.024	7.696	48.256	63.395
ENSG00000178947	SMIM10L2A	1.3	6.8E-03	1.3E-02	4.250	0.531	3.660	4.686	3.238	0.409	2.998	3.710
ENSG00000179085	DPM3	1.3	1.1E-03	2.7E-03	36.541	4.265	33.664	41.441	27.465	0.670	26.746	28.072
ENSG00000179454	KLHL28	1.3	1.1E-05	3.8E-05	8.546	0.044	8.505	8.592	6.715	0.407	6.264	7.057
ENSG00000179456	ZBTB18	1.3	1.5E-06	6.1E-06	18.437	0.083	18.368	18.529	14.240	0.743	13.406	14.830
ENSG00000179743		1.3	6.2E-03	1.2E-02	5.072	0.197	4.857	5.243	3.840	0.472	3.419	4.349
ENSG00000179941	BBS10	1.3	1.4E-05	4.8E-05	30.512	1.508	28.852	31.799	24.436	1.798	22.839	26.383
ENSG00000180211		1.3	3.7E-03	7.7E-03	69.643	4.154	66.860	74.418	54.922	7.909	45.898	60.646
ENSG00000180354	MTURN	1.3	3.6E-04	9.3E-04	7.298	0.724	6.782	8.126	5.748	0.793	5.166	6.651
ENSG00000180667	YOD1	1.3	6.0E-07	2.7E-06	24.711	1.897	22.829	26.623	19.430	0.542	18.951	20.018
ENSG00000181038	METTL23	1.3	5.6E-06	2.1E-05	36.524	3.124	33.430	39.676	27.804	0.581	27.252	28.427
ENSG00000181450	ZNF678	1.3	6.4E-08	3.5E-07	18.856	1.869	16.707	20.101	14.433	0.760	13.682	15.203
ENSG00000182165	TP53TG1	1.3	2.1E-02	3.7E-02	15.040	1.347	13.760	16.446	11.803	3.182	8.658	15.020
ENSG00000182472	CAPN12	1.3	1.7E-02	3.0E-02	1.583	0.165	1.454	1.768	1.188	0.078	1.120	1.273
ENSG00000182923	CEP63	1.3	5.2E-08	2.9E-07	9.929	0.391	9.521	10.301	7.558	0.220	7.310	7.733
ENSG00000183077	AFMID	1.3	1.6E-05	5.5E-05	18.072	1.359	17.177	19.635	14.339	0.464	13.803	14.620
ENSG00000183298	RPSAP19	1.3	2.2E-10	2.0E-09	695.248	12.438	683.350	708.163	543.159	21.432	518.717	558.737
ENSG00000183615	FAM167B	1.3	9.7E-02	1.4E-01	5.464	0.533	5.112	6.076	4.248	1.097	2.985	4.953
ENSG00000183718	TRIM52	1.3	1.0E-03	2.4E-03	5.030	0.811	4.116	5.665	3.887	0.174	3.704	4.049
ENSG00000183762	KREMEN1	1.3	8.8E-04	2.1E-03	3.274	0.120	3.137	3.357	2.484	0.431	2.031	2.890
ENSG00000184154	LRTOMT	1.3	1.7E-02	3.1E-02	2.402	0.346	2.010	2.661	1.892	0.213	1.762	2.137
ENSG00000184203	PPP1R2	1.3	4.5E-07	2.1E-06	29.668	0.825	28.751	30.352	23.552	1.092	22.293	24.240
ENSG00000184349	EFNA5	1.3	1.1E-03	2.6E-03	5.432	0.276	5.142	5.692	4.302	0.382	3.919	4.684
ENSG00000184557	SOCS3	1.3	8.0E-08	4.3E-07	43.304	0.913	42.490	44.291	32.329	2.340	29.634	33.837
ENSG00000184743	ATL3	1.3	1.6E-06	6.6E-06	15.462	0.655	14.758	16.052	12.110	0.873	11.543	13.116
ENSG00000185088	RPS27L	1.3	2.0E-08	1.2E-07	28.341	0.165	28.206	28.525	22.204	1.501	20.513	23.378
ENSG00000185532	PRKG1	1.3	3.6E-08	2.1E-07	12.590	0.752	12.049	13.449	9.406	0.381	9.101	9.832
ENSG00000185624	P4HB	1.3	5.4E-11	5.6E-10	212.195	3.543	208.119	214.540	162.411	9.375	156.924	173.236
ENSG00000185641		1.3	5.7E-10	4.7E-09	1726.171	56.605	1665.710	1777.908	1374.725	59.769	1320.608	1438.876
ENSG00000185885	IFITM1	1.3	7.5E-08	4.1E-07	238.329	7.002	230.253	242.705	189.356	15.188	172.368	201.624
ENSG00000185989	RASA3	1.3	9.6E-06	3.4E-05	27.945	0.216	27.706	28.128	22.224	1.853	21.116	24.364
ENSG00000186364	NUDT17	1.3	9.4E-02	1.4E-01	3.578	0.346	3.260	3.947	2.796	0.864	2.291	3.794
ENSG00000186715	MST1L	1.3	2.6E-02	4.4E-02	2.114	0.058	2.050	2.162	1.683	0.199	1.468	1.861
ENSG00000186994	KANK3	1.3	2.6E-02	4.5E-02	2.596	0.521	2.136	3.161	1.959	0.237	1.788	2.230
ENSG00000187098	MITF	1.3	1.3E-01	1.8E-01	0.688	0.193	0.494	0.880	0.534	0.109	0.427	0.645
ENSG00000187118	CMC1	1.3	1.8E-05	6.2E-05	6.848	0.504	6.383	7.383	5.362	0.230	5.112	5.564
ENSG00000187742	SECISBP2	1.3	3.4E-09	2.4E-08	36.647	0.772	36.035	37.514	29.342	1.265	28.445	30.789
ENSG00000188042	ARL4C	1.3	2.8E-04	7.5E-04	18.378	1.114	17.113	19.213	14.564	0.411	14.132	14.951
ENSG00000188321	ZNF559	1.3	7.9E-06	2.9E-05	11.182	0.110	11.059	11.271	8.789	0.058	8.745	8.855
ENSG00000188459	WASF4P	1.3	1.3E-02	2.4E-02	13.903	1.171	12.603	14.877	11.114	0.952	10.191	12.094
ENSG00000188599	NPIPP1	1.3	2.0E-02	3.6E-02	7.532	0.571	6.943	8.083	5.928	0.547	5.490	6.540
ENSG00000188610	FAM72B	1.3	3.9E-08	2.2E-07	33.472	0.758	32.720	34.235	25.070	1.144	24.301	26.385
ENSG00000188613	NANOS1	1.3	6.1E-03	1.2E-02	4.319	0.254	4.111	4.602	3.296	0.191	3.150	3.512
ENSG00000188763	FZD9	1.3	5.3E-02	8.4E-02	3.998	0.103	3.888	4.093	3.145	0.266	2.928	3.441
ENSG00000188846	RPL14	1.3	3.6E-13	5.7E-12	159.451	4.195	154.943	163.239	122.202	2.828	120.049	125.405
ENSG00000189007	ADAT2	1.3	1.3E-07	6.8E-07	12.143	0.613	11.767	12.850	9.069	0.560	8.628	9.699
ENSG00000189050	RNFT1	1.3	2.9E-02	5.0E-02	4.128	0.510	3.556	4.537	3.208	0.141	3.123	3.371
ENSG00000189114	BLOC1S3	1.3	1.7E-02	3.1E-02	5.173	0.605	4.516	5.707	4.060	0.301	3.825	4.399
ENSG00000189171	S100A13	1.3	1.6E-04	4.6E-04	22.204	1.697	20.826	24.099	17.224	1.459	15.692	18.596
ENSG00000196118	CCDC189	1.3	9.2E-02	1.4E-01	1.763	0.267	1.528	2.054	1.339	0.164	1.151	1.451
ENSG00000196507	TCEAL3	1.3	1.7E-04	4.6E-04	20.525	0.446	20.156	21.021	15.990	0.376	15.706	16.417
ENSG00000196544	BORCS6	1.3	7.1E-03	1.4E-02	5.597	0.721	4.917	6.354	4.183	0.469	3.822	4.713
ENSG00000196550	FAM72A	1.3	2.1E-07	1.1E-06	25.298	0.764	24.655	26.142	19.122	0.449	18.604	19.400
ENSG00000196636	SDHAF3	1.3	1.7E-06	7.0E-06	38.206	1.562	37.214	40.007	28.492	2.917	26.526	31.844
ENSG00000196670	ZFP62	1.3	4.5E-10	3.8E-09	43.378	1.508	41.704	44.631	32.785	0.276	32.620	33.113
ENSG00000196839	ADA	1.3	1.8E-04	5.0E-04	20.099	1.460	18.845	21.702	15.796	0.568	15.300	16.416
ENSG00000196912	ANKRD36B	1.3	1.1E-03	2.6E-03	3.244	0.107	3.122	3.320	2.469	0.286	2.142	2.672
ENSG00000196937	FAM3C	1.3	3.9E-07	1.8E-06	51.077	3.556	46.984	53.407	40.558	2.838	37.690	43.365
ENSG00000197077	KIAA1671	1.3	1.6E-09	1.2E-08	12.982	0.179	12.775	13.089	9.730	0.488	9.305	10.263
ENSG00000197258	EIF4BP6	1.3	1.0E-10	9.9E-10	267.643	1.813	266.168	269.667	210.253	5.524	204.768	215.816
ENSG00000197415	VEPH1	1.3	4.5E-05	1.4E-04	7.257	0.306	6.994	7.593	5.617	0.400	5.213	6.013
ENSG00000197808	ZNF461	1.3	1.9E-03	4.3E-03	4.266	0.169	4.075	4.397	3.205	0.217	3.077	3.456
ENSG00000197863	ZNF790	1.3	8.5E-03	1.6E-02	4.706	0.504	4.128	5.058	3.703	0.220	3.541	3.954
ENSG00000197903	HIST1H2BK	1.3	2.4E-03	5.3E-03	49.819	1.100	48.614	50.771	37.555	3.799	34.119	41.635
ENSG00000197927	C2orf27A	1.3	2.5E-02	4.3E-02	3.512	1.017	2.847	4.682	2.640	0.271	2.329	2.817
ENSG00000197958	RPL12	1.3	2.9E-13	4.7E-12	462.847	18.979	441.289	477.038	349.521	5.471	344.239	355.164
ENSG00000197961	ZNF121	1.3	6.0E-04	1.5E-03	79.092	2.311	76.568	81.104	62.348	4.336	57.358	65.207
ENSG00000198034	RPS4X	1.3	5.7E-13	8.7E-12	1354.622	39.986	1323.141	1399.612	1060.537	21.552	1039.095	1082.188
ENSG00000198046	ZNF667	1.3	6.5E-08	3.6E-07	11.974	0.087	11.896	12.068	9.091	0.210	8.884	9.304
ENSG00000198075	SULT1C4	1.3	5.5E-02	8.7E-02	3.201	0.357	2.823	3.533	2.568	0.445	2.121	3.011
ENSG00000198089	SFI1	1.3	8.3E-06	3.0E-05	4.360	0.147	4.197	4.480	3.240	0.231	3.029	3.488
ENSG00000198221	AFDN-AS1	1.3	7.3E-02	1.1E-01	3.045	0.244	2.769	3.230	2.411	0.218	2.262	2.661
ENSG00000198252	STYX	1.3	3.7E-08	2.2E-07	42.370	1.821	41.083	44.454	33.086	2.384	30.831	35.581
ENSG00000198420	TCAF1	1.3	8.7E-08	4.7E-07	30.487	1.164	29.656	31.817	24.288	0.576	23.820	24.931
ENSG00000198625	MDM4	1.3	1.3E-07	6.6E-07	12.938	0.302	12.633	13.236	9.842	0.966	9.142	11.015
ENSG00000198682	PAPSS2	1.3	1.2E-08	7.7E-08	39.043	1.096	38.125	40.257	29.606	0.452	29.096	29.958
ENSG00000198825	INPP5F	1.3	2.3E-08	1.4E-07	31.204	0.167	31.023	31.353	24.513	1.406	23.164	25.970
ENSG00000198839	ZNF277	1.3	2.3E-06	9.1E-06	24.391	1.731	22.423	25.678	18.816	1.155	17.665	19.975
ENSG00000198861	PJA2	1.3	1.0E-11	1.2E-10	91.104	0.480	90.789	91.657	69.081	3.666	64.849	71.253
ENSG00000203279		1.3	5.2E-02	8.2E-02	10.268	0.277	10.069	10.585	7.830	2.123	5.921	10.117
ENSG00000204091	TDRG1	1.3	1.0E-01	1.5E-01	2.630	0.350	2.291	2.990	1.973	0.360	1.565	2.246
ENSG00000204172	AGAP9	1.3	9.2E-03	1.8E-02	7.603	1.028	6.694	8.718	5.945	0.420	5.625	6.421
ENSG00000204177	BMS1P1	1.3	8.4E-03	1.6E-02	3.467	0.166	3.293	3.624	2.695	0.222	2.458	2.897
ENSG00000204387	C6orf48	1.3	4.4E-07	2.0E-06	90.641	4.711	85.344	94.363	72.057	2.298	69.711	74.304
ENSG00000204592	HLA-E	1.3	3.7E-08	2.1E-07	44.993	1.872	43.068	46.806	33.977	1.462	32.359	35.203
ENSG00000204623	ZNRD1ASP	1.3	2.2E-03	4.8E-03	2.021	0.056	1.957	2.064	1.611	0.248	1.339	1.824
ENSG00000204778	CBWD4P	1.3	2.4E-03	5.2E-03	25.299	2.837	23.213	28.530	18.975	1.085	17.725	19.680
ENSG00000204899	MZT1	1.3	2.1E-04	5.7E-04	80.531	6.463	73.918	86.834	63.646	7.088	55.874	69.753
ENSG00000205517	RGL3	1.3	7.1E-04	1.8E-03	9.368	1.123	8.358	10.578	7.451	0.412	7.130	7.916
ENSG00000205571	SMN2	1.3	3.0E-06	1.2E-05	31.750	1.994	29.448	32.936	24.938	0.800	24.071	25.647
ENSG00000205832	C16orf96	1.3	4.1E-02	6.6E-02	2.032	0.234	1.785	2.250	1.550	0.153	1.388	1.692
ENSG00000205871		1.3	7.5E-05	2.3E-04	53.377	5.586	50.132	59.828	40.631	2.208	38.302	42.695
ENSG00000205940	HSP90AB2P	1.3	1.1E-01	1.6E-01	2.501	0.428	2.185	2.988	1.947	0.512	1.631	2.538
ENSG00000206573	THUMPD3-AS	1.3	4.3E-08	2.4E-07	16.941	0.381	16.504	17.198	12.853	0.349	12.452	13.090
ENSG00000207442	SNORD116-6	1.3	7.6E-02	1.1E-01	84.165	8.462	74.396	89.233	66.719	11.883	53.454	76.392
ENSG00000212664		1.3	2.0E-08	1.2E-07	779.099	9.724	772.160	790.213	622.785	10.680	613.621	634.514
ENSG00000213015	ZNF580	1.3	3.2E-03	6.8E-03	9.638	0.254	9.467	9.930	7.558	1.116	6.331	8.510
ENSG00000213178		1.3	5.9E-11	6.0E-10	1695.246	12.756	1683.243	1708.641	1307.411	25.973	1279.450	1330.786
ENSG00000213239	NPM1P32	1.3	2.6E-02	4.4E-02	14.580	0.871	13.880	15.555	11.217	0.787	10.451	12.023
ENSG00000213315		1.3	1.8E-02	3.2E-02	17.849	0.226	17.597	18.035	13.318	2.417	10.942	15.775
ENSG00000213432	RPL17P34	1.3	1.2E-02	2.2E-02	17.604	1.250	16.453	18.933	13.184	0.250	12.912	13.403
ENSG00000213598		1.3	8.2E-06	3.0E-05	130.050	3.571	126.127	133.111	98.003	1.075	97.194	99.223
ENSG00000213626	LBH	1.3	1.7E-07	8.8E-07	21.858	0.386	21.578	22.299	16.578	0.370	16.237	16.972
ENSG00000213719	CLIC1	1.3	4.7E-11	4.9E-10	406.431	7.348	398.043	411.733	318.974	4.228	315.126	323.500
ENSG00000213860	RPL21P75	1.3	8.2E-10	6.6E-09	1788.558	7.738	1780.210	1795.491	1434.170	68.223	1370.585	1500.971
ENSG00000213866	YBX1P10	1.3	2.5E-09	1.8E-08	392.668	13.658	383.933	408.407	313.459	12.760	299.087	323.460
ENSG00000213881	NPM1P6	1.3	1.6E-09	1.2E-08	586.123	17.599	565.805	596.606	468.921	18.391	448.096	482.939
ENSG00000213904	LIPE-AS1	1.3	1.4E-01	2.0E-01	0.881	0.296	0.656	1.216	0.681	0.145	0.564	0.843
ENSG00000213918	DNASE1	1.3	9.1E-05	2.7E-04	3.759	0.206	3.534	3.940	2.971	0.110	2.854	3.073
ENSG00000214019		1.3	4.4E-03	9.0E-03	15.617	2.059	13.271	17.125	12.517	0.506	12.121	13.087
ENSG00000214076	CPSF1P1	1.3	6.3E-02	9.7E-02	1.734	0.091	1.639	1.821	1.353	0.159	1.244	1.535
ENSG00000214078	CPNE1	1.3	3.2E-10	2.8E-09	76.707	2.127	74.325	78.415	58.484	2.438	55.751	60.438
ENSG00000214401	KANSL1-AS1	1.3	1.2E-01	1.7E-01	5.104	0.314	4.744	5.322	4.031	0.500	3.601	4.580
ENSG00000214535	RPS15AP1	1.3	1.9E-06	7.9E-06	321.819	16.223	309.727	340.256	254.921	11.865	241.301	263.019
ENSG00000214541	RPS4XP3	1.3	1.4E-01	1.9E-01	14.939	4.393	12.050	19.994	11.551	2.816	9.872	14.802
ENSG00000214558		1.3	4.5E-03	9.2E-03	9.556	0.305	9.346	9.906	7.652	0.323	7.388	8.013
ENSG00000214784		1.3	6.8E-08	3.7E-07	313.418	16.919	294.003	325.009	248.959	14.972	239.444	266.217
ENSG00000214875	PPIAP29	1.3	9.3E-09	6.1E-08	577.621	15.082	565.037	594.339	457.078	22.389	433.957	478.654
ENSG00000215012	C22orf29	1.3	1.4E-06	5.8E-06	14.862	0.847	14.221	15.822	11.554	0.540	11.150	12.167
ENSG00000215126	CBWD6	1.3	2.0E-06	8.2E-06	5.354	0.177	5.157	5.497	4.026	0.222	3.770	4.166
ENSG00000215158		1.3	1.1E-03	2.6E-03	5.940	0.586	5.287	6.420	4.699	0.571	4.095	5.230
ENSG00000215256	DHRS4-AS1	1.3	2.8E-04	7.5E-04	11.295	0.425	10.842	11.685	8.944	0.057	8.882	8.995
ENSG00000215784	FAM72D	1.3	1.5E-06	6.4E-06	29.711	0.442	29.244	30.123	22.291	0.816	21.403	23.007
ENSG00000216866	RPS2P55	1.3	1.2E-09	9.2E-09	561.205	4.298	556.894	565.490	439.032	36.678	404.461	477.506
ENSG00000217027	TPT1P4	1.3	1.0E-02	1.9E-02	23.906	2.643	20.868	25.674	17.691	3.476	14.085	21.019
ENSG00000217716	RPS10P3	1.3	2.3E-06	9.1E-06	226.325	7.763	219.828	234.923	180.661	9.454	171.466	190.355
ENSG00000218208	RPS27AP11	1.3	6.1E-05	1.9E-04	110.909	8.748	103.364	120.498	86.405	2.143	83.932	87.714
ENSG00000218739	CEBPZOS	1.3	1.2E-11	1.4E-10	103.658	4.012	99.039	106.278	77.104	4.983	73.028	82.659
ENSG00000219451	RPL23P8	1.3	4.9E-03	9.9E-03	42.299	5.478	37.925	48.443	32.773	2.617	30.134	35.368
ENSG00000220842	RPL21P16	1.3	2.4E-10	2.1E-09	2115.723	31.534	2082.226	2144.835	1695.300	62.569	1638.399	1762.307
ENSG00000221923	ZNF880	1.3	5.2E-05	1.6E-04	21.181	2.269	19.422	23.742	16.869	0.326	16.520	17.165
ENSG00000221968	FADS3	1.3	9.6E-06	3.4E-05	10.702	0.765	9.822	11.202	8.121	0.686	7.359	8.725
ENSG00000223591	CENPVL1	1.3	1.3E-01	1.8E-01	2.923	0.163	2.750	3.073	2.311	0.367	1.894	2.587
ENSG00000224043	CCNT2-AS1	1.3	6.7E-02	1.0E-01	8.692	0.933	7.670	9.498	6.921	0.661	6.443	7.675
ENSG00000224174		1.3	1.2E-01	1.7E-01	10.016	0.987	8.935	10.871	7.569	0.665	6.835	8.132
ENSG00000224546	EIF4BP3	1.3	1.5E-08	9.4E-08	107.584	6.133	101.063	113.236	81.584	6.138	74.969	87.096
ENSG00000225031	EIF4BP7	1.3	4.8E-10	4.0E-09	176.680	3.296	173.219	179.781	136.994	7.399	128.617	142.640
ENSG00000225486		1.3	1.4E-01	2.0E-01	12.699	1.956	10.576	14.429	9.802	1.921	7.914	11.749
ENSG00000225573	RPL35P5	1.3	2.5E-07	1.2E-06	408.412	16.370	389.627	419.617	316.444	17.956	295.988	329.602
ENSG00000225739	NPM1P18	1.3	2.9E-03	6.2E-03	24.062	0.662	23.503	24.793	17.985	3.310	14.331	20.768
ENSG00000226328	NUP50-AS1	1.3	5.7E-02	8.9E-02	4.388	0.192	4.210	4.591	3.496	0.242	3.231	3.704
ENSG00000226360		1.3	1.5E-09	1.2E-08	703.846	43.150	670.459	752.569	550.314	23.921	526.044	573.870
ENSG00000226711	FAM66C	1.3	1.2E-01	1.7E-01	1.068	0.074	1.007	1.151	0.833	0.075	0.756	0.905
ENSG00000226855	RPSAP17	1.3	1.1E-01	1.5E-01	6.038	0.685	5.574	6.824	4.780	0.630	4.250	5.476
ENSG00000227627		1.3	6.9E-02	1.1E-01	3.724	0.622	3.237	4.425	2.939	0.383	2.522	3.276
ENSG00000227671		1.3	4.1E-06	1.6E-05	55.932	1.041	54.880	56.962	42.370	3.654	38.888	46.175
ENSG00000227694	RPL23AP74	1.3	6.3E-04	1.6E-03	73.490	9.629	62.556	80.701	56.882	3.111	53.334	59.141
ENSG00000227973	PIN4P1	1.3	2.6E-03	5.6E-03	48.696	3.358	45.909	52.424	37.581	0.466	37.190	38.097
ENSG00000228065	LINC01515	1.3	2.8E-04	7.5E-04	2.590	0.138	2.456	2.732	1.950	0.146	1.833	2.113
ENSG00000228218	ATF4P3	1.3	1.7E-02	3.1E-02	16.523	1.154	15.305	17.600	13.166	1.638	12.193	15.057
ENSG00000228594	FNDC10	1.3	4.1E-04	1.1E-03	15.186	0.597	14.636	15.821	11.720	1.000	10.585	12.475
ENSG00000228613		1.3	7.5E-02	1.1E-01	8.330	0.614	7.659	8.864	6.301	0.390	5.865	6.618
ENSG00000228797	FAM207BP	1.3	2.2E-03	4.8E-03	47.597	0.488	47.043	47.961	37.258	2.145	34.969	39.221
ENSG00000228801		1.3	6.3E-02	9.8E-02	3.850	0.629	3.252	4.507	2.899	0.597	2.326	3.517
ENSG00000229097	CALM2P2	1.3	4.7E-05	1.5E-04	161.501	10.698	149.776	170.731	124.601	10.904	113.338	135.106
ENSG00000229117	RPL41	1.3	2.1E-12	2.8E-11	756.566	19.287	740.140	777.803	577.856	23.610	559.734	604.556
ENSG00000229180		1.3	1.2E-04	3.6E-04	4.228	0.173	4.121	4.427	3.218	0.109	3.103	3.320
ENSG00000229251	HNRNPA1P8	1.3	4.8E-05	1.5E-04	40.545	1.936	38.672	42.538	31.119	1.529	29.415	32.372
ENSG00000229503		1.3	6.9E-02	1.1E-01	15.991	2.278	13.572	18.095	12.667	1.614	11.345	14.466
ENSG00000229605	RPL21P93	1.3	2.2E-06	8.9E-06	120.203	4.465	115.186	123.738	90.656	6.369	84.150	96.879
ENSG00000229638	RPL4P4	1.3	7.6E-12	9.4E-11	1372.230	41.755	1336.879	1418.300	1084.335	35.542	1045.803	1115.835
ENSG00000229692	SOS1-IT1	1.3	2.1E-02	3.7E-02	6.958	0.817	6.400	7.896	5.276	0.506	4.709	5.681
ENSG00000230207	RPL4P5	1.3	2.7E-09	2.0E-08	276.815	8.245	268.044	284.409	221.909	7.662	213.873	229.131
ENSG00000230383		1.3	1.6E-09	1.2E-08	288.576	9.335	278.105	296.028	222.284	8.228	213.210	229.259
ENSG00000230623		1.3	3.2E-07	1.5E-06	65.756	2.017	63.506	67.402	52.066	1.793	50.050	53.485
ENSG00000230629	RPS23P8	1.3	8.7E-12	1.1E-10	1846.603	26.259	1816.403	1864.051	1424.642	64.227	1381.075	1498.402
ENSG00000230673	PABPC1P3	1.3	1.1E-02	2.0E-02	23.677	2.013	21.737	25.755	18.017	1.843	16.488	20.063
ENSG00000230724	LINC01001	1.3	5.8E-03	1.2E-02	3.340	0.085	3.274	3.436	2.477	0.465	2.146	3.008
ENSG00000231369		1.3	2.4E-03	5.2E-03	27.608	3.548	23.904	30.977	20.471	2.069	19.136	22.855
ENSG00000231549	USMG5P1	1.3	1.1E-01	1.6E-01	27.565	6.261	20.493	32.398	21.369	5.272	15.408	25.418
ENSG00000231747		1.3	5.0E-04	1.3E-03	93.971	6.151	86.879	97.855	70.670	12.647	59.509	84.406
ENSG00000231889	PPP1R2P3	1.3	7.9E-03	1.5E-02	5.445	0.550	4.810	5.775	4.064	0.425	3.813	4.555
ENSG00000232445		1.3	1.5E-08	9.5E-08	249.471	3.893	246.127	253.745	191.601	7.888	186.759	200.715
ENSG00000232531		1.3	9.4E-02	1.4E-01	4.486	0.459	4.017	4.935	3.398	0.643	2.954	4.136
ENSG00000232888		1.3	3.0E-08	1.8E-07	981.606	43.263	944.412	1029.085	775.456	40.785	730.975	811.095
ENSG00000233223		1.3	1.3E-03	3.0E-03	21.145	1.186	19.890	22.246	15.717	3.103	12.757	18.946
ENSG00000233381	AK6P3	1.3	1.3E-04	3.7E-04	54.459	2.022	52.214	56.137	41.461	2.088	39.781	43.813
ENSG00000233406		1.3	3.4E-02	5.7E-02	8.899	0.678	8.494	9.681	6.844	0.814	5.910	7.402
ENSG00000233461		1.3	7.0E-02	1.1E-01	5.795	0.673	5.246	6.545	4.434	0.970	3.557	5.476
ENSG00000233830	EIF4HP1	1.3	1.3E-08	8.0E-08	392.477	5.884	387.444	398.946	314.579	2.063	313.052	316.925
ENSG00000233913	RPL10P9	1.3	1.6E-09	1.2E-08	758.139	33.551	726.368	793.224	604.504	12.457	590.253	613.326
ENSG00000233927	RPS28	1.3	7.1E-06	2.6E-05	56.703	4.036	52.792	60.852	45.139	3.550	42.370	49.141
ENSG00000233937		1.3	3.7E-06	1.4E-05	14.662	0.871	13.953	15.634	10.926	0.369	10.544	11.281
ENSG00000233966	UBE2SP1	1.3	3.7E-07	1.8E-06	244.278	12.244	230.214	252.563	192.959	15.217	175.581	203.897
ENSG00000234004		1.3	1.8E-02	3.2E-02	30.842	1.799	29.588	32.904	24.112	2.256	21.609	25.990
ENSG00000234742		1.3	1.5E-04	4.3E-04	76.193	4.924	71.890	81.563	58.645	4.237	53.869	61.954
ENSG00000234745	HLA-B	1.3	3.7E-07	1.7E-06	29.605	2.269	27.644	32.090	22.537	1.016	21.569	23.594
ENSG00000234797	RPS3AP6	1.3	6.7E-14	1.2E-12	3590.302	121.885	3518.953	3731.050	2692.422	108.565	2567.989	2767.818
ENSG00000234851	RPL23AP42	1.3	1.4E-13	2.5E-12	4130.156	80.449	4051.460	4212.250	3168.665	69.862	3112.888	3247.685
ENSG00000234881		1.3	1.3E-02	2.4E-02	18.454	1.877	16.615	20.367	14.460	1.622	12.602	15.593
ENSG00000235084	CHCHD2P6	1.3	3.2E-03	6.7E-03	44.660	1.221	43.912	46.069	34.258	4.767	31.349	39.759
ENSG00000235174		1.3	5.6E-12	7.0E-11	10352.211	319.936	10069.866	10699.709	7862.605	358.807	7473.988	8181.314
ENSG00000235453	SMIM27	1.3	1.1E-02	2.0E-02	7.411	0.579	6.767	7.888	5.601	1.051	4.719	6.763
ENSG00000235486		1.3	4.8E-02	7.7E-02	7.333	1.373	6.022	8.761	5.553	0.168	5.387	5.724
ENSG00000235884	LINC00941	1.3	5.0E-02	8.0E-02	3.978	0.507	3.549	4.537	3.019	0.617	2.472	3.689
ENSG00000235954	TTC28-AS1	1.3	2.6E-04	6.9E-04	4.085	0.345	3.706	4.381	3.236	0.114	3.113	3.340
ENSG00000236060	HSPB1P1	1.3	6.1E-02	9.5E-02	27.885	3.956	24.344	32.154	21.271	4.494	16.277	24.988
ENSG00000236088	COX10-AS1	1.3	2.3E-03	5.1E-03	2.595	0.245	2.351	2.841	1.963	0.185	1.774	2.144
ENSG00000236094	LINC00545	1.3	2.2E-03	4.8E-03	60.617	3.365	56.736	62.716	46.041	3.464	42.144	48.772
ENSG00000236493	EIF2S2P3	1.3	5.1E-03	1.0E-02	14.672	0.840	13.920	15.579	11.134	1.556	9.554	12.666
ENSG00000236552	RPL13AP5	1.3	5.9E-12	7.5E-11	5084.924	173.745	4896.341	5238.499	4008.188	126.173	3882.798	4135.129
ENSG00000236686	BZW1P1	1.3	8.7E-02	1.3E-01	3.701	0.484	3.249	4.211	2.765	0.214	2.603	3.008
ENSG00000236753	MKLN1-AS	1.3	3.8E-02	6.2E-02	3.389	0.587	3.013	4.065	2.611	0.393	2.260	3.036
ENSG00000236876	TMSB4XP1	1.3	3.7E-05	1.2E-04	440.352	21.507	423.001	464.414	350.690	13.028	336.037	360.966
ENSG00000236924		1.3	3.4E-03	7.1E-03	35.507	1.680	34.039	37.340	27.392	2.395	24.628	28.867
ENSG00000237094		1.3	2.6E-02	4.4E-02	1.633	0.088	1.531	1.691	1.245	0.179	1.038	1.360
ENSG00000237161		1.3	1.1E-01	1.5E-01	3.232	0.492	2.777	3.754	2.543	0.185	2.360	2.729
ENSG00000237491		1.3	3.3E-02	5.6E-02	1.266	0.080	1.189	1.349	0.962	0.061	0.924	1.032
ENSG00000237506	RPSAP15	1.3	3.3E-11	3.6E-10	439.371	6.657	435.409	447.056	337.732	4.455	333.867	342.605
ENSG00000237550	RPL8P9	1.3	4.5E-12	5.7E-11	3374.099	90.049	3270.376	3432.285	2671.417	77.279	2625.682	2760.642
ENSG00000237682		1.3	2.7E-04	7.3E-04	23.034	0.898	22.230	24.004	18.159	0.878	17.578	19.169
ENSG00000237931	CLIC4P3	1.3	1.5E-02	2.7E-02	18.177	2.471	15.325	19.669	14.119	2.191	12.663	16.639
ENSG00000237991	RPL35P1	1.3	8.5E-04	2.0E-03	118.308	5.565	112.662	123.788	92.987	14.420	76.893	104.730
ENSG00000238035		1.3	6.0E-02	9.3E-02	3.146	0.165	2.956	3.254	2.417	0.446	1.932	2.810
ENSG00000238041		1.3	1.1E-01	1.6E-01	25.427	2.315	22.769	26.994	20.303	3.727	17.099	24.393
ENSG00000238103	RPL9P7	1.3	3.4E-12	4.4E-11	2685.640	82.434	2590.685	2738.867	2104.224	49.013	2049.673	2144.554
ENSG00000238142		1.3	4.8E-03	9.7E-03	61.013	10.105	49.402	67.816	48.471	1.377	47.064	49.816
ENSG00000240087	RPSAP12	1.3	7.3E-08	4.0E-07	230.401	8.644	220.732	237.381	183.514	10.157	171.846	190.384
ENSG00000240230	COX19	1.3	3.4E-04	8.9E-04	7.182	0.666	6.414	7.603	5.645	0.169	5.470	5.807
ENSG00000240342		1.3	1.5E-12	2.2E-11	2416.063	9.949	2404.601	2422.478	1831.949	145.309	1672.567	1957.056
ENSG00000240395	RPL5P23	1.3	6.7E-03	1.3E-02	15.929	1.662	14.830	17.841	11.853	1.982	10.353	14.100
ENSG00000240771	ARHGEF25	1.3	2.9E-02	4.9E-02	2.217	0.312	1.867	2.467	1.704	0.042	1.675	1.752
ENSG00000240849	TMEM189	1.3	3.5E-06	1.4E-05	10.771	0.633	10.042	11.175	8.460	0.421	7.996	8.820
ENSG00000241061	RPL5P1	1.3	1.6E-04	4.6E-04	59.960	6.555	53.307	66.413	47.397	2.426	45.730	50.179
ENSG00000241081		1.3	1.2E-04	3.4E-04	191.018	17.839	174.881	210.174	152.849	18.554	136.203	172.852
ENSG00000241484	ARHGAP8	1.3	4.8E-03	9.7E-03	3.049	0.080	2.963	3.123	2.318	0.141	2.156	2.405
ENSG00000241511		1.3	2.3E-03	5.1E-03	48.188	3.444	46.024	52.159	36.440	5.135	32.901	42.330
ENSG00000241549	GUSBP2	1.3	4.7E-02	7.5E-02	3.739	0.300	3.394	3.939	2.911	0.114	2.788	3.012
ENSG00000241612		1.3	9.5E-03	1.8E-02	25.584	1.190	24.485	26.848	19.630	0.284	19.325	19.886
ENSG00000241741	RPL7AP30	1.3	5.6E-12	7.1E-11	500.397	14.434	490.043	516.885	373.001	9.467	366.094	383.793
ENSG00000242083		1.3	2.8E-03	6.0E-03	36.481	2.446	33.940	38.818	28.747	2.582	25.867	30.893
ENSG00000242259	C22orf39	1.3	1.5E-06	6.3E-06	12.085	0.080	12.005	12.165	9.252	0.688	8.748	10.036
ENSG00000242715	CCDC169	1.3	4.1E-04	1.1E-03	5.603	0.688	4.812	6.061	4.354	0.136	4.197	4.442
ENSG00000242951		1.3	2.0E-05	6.7E-05	175.667	11.621	162.412	184.100	136.617	5.943	129.926	141.281
ENSG00000242952	RPSAP3	1.3	1.4E-02	2.5E-02	11.618	0.732	10.896	12.358	8.748	0.862	7.786	9.450
ENSG00000243004		1.3	2.6E-04	6.9E-04	28.007	1.209	26.890	29.292	21.683	0.868	20.719	22.402
ENSG00000243071		1.3	1.5E-04	4.2E-04	56.916	5.516	50.547	60.196	42.222	1.548	40.985	43.959
ENSG00000243302		1.3	7.5E-02	1.1E-01	9.065	0.160	8.922	9.237	7.193	1.283	5.828	8.373
ENSG00000243517		1.3	5.0E-02	8.0E-02	19.589	4.612	14.331	22.954	15.582	1.195	14.252	16.566
ENSG00000243824	RPL12P6	1.3	6.2E-05	1.9E-04	78.059	9.359	72.187	88.852	58.802	2.464	55.959	60.322
ENSG00000243829		1.3	1.7E-03	3.8E-03	32.381	4.894	27.227	36.967	24.049	0.880	23.350	25.037
ENSG00000244398		1.3	1.2E-11	1.4E-10	4472.503	55.870	4408.047	4507.083	3556.453	9.143	3545.920	3562.334
ENSG00000244687	UBE2V1	1.3	7.5E-03	1.5E-02	4.255	0.480	3.704	4.581	3.400	0.250	3.117	3.589
ENSG00000244716		1.3	6.9E-11	6.9E-10	1544.049	36.789	1507.786	1581.341	1203.466	38.436	1175.823	1247.357
ENSG00000245149	RNF139-AS1	1.3	3.7E-02	6.1E-02	1.165	0.187	0.983	1.356	0.876	0.276	0.570	1.107
ENSG00000245571		1.3	5.0E-03	1.0E-02	6.560	0.289	6.302	6.873	4.970	0.664	4.412	5.704
ENSG00000246082	NUDT16P1	1.3	1.2E-05	4.3E-05	23.839	1.698	22.565	25.766	18.027	1.766	16.615	20.006
ENSG00000246339	EXTL3-AS1	1.3	1.3E-01	1.8E-01	0.722	0.136	0.571	0.834	0.543	0.048	0.495	0.590
ENSG00000246560		1.3	1.2E-01	1.7E-01	6.524	0.436	6.119	6.985	5.081	1.026	3.981	6.011
ENSG00000247950	SEC24B-AS1	1.3	1.0E-01	1.5E-01	1.886	0.295	1.633	2.210	1.432	0.212	1.189	1.583
ENSG00000248278	SUMO2P17	1.3	4.8E-04	1.2E-03	29.990	0.945	29.284	31.064	22.684	3.218	19.004	24.965
ENSG00000249741		1.3	6.3E-02	9.8E-02	12.252	2.285	10.619	14.863	9.416	1.140	8.722	10.732
ENSG00000249855	EEF1A1P19	1.3	4.8E-07	2.2E-06	237.348	23.113	218.189	263.017	181.681	10.488	175.371	193.788
ENSG00000250337	LINC01021	1.3	4.1E-05	1.3E-04	52.760	3.561	48.739	55.516	42.298	3.753	38.510	46.016
ENSG00000250571	GLI4	1.3	3.3E-02	5.5E-02	3.126	0.358	2.867	3.534	2.433	0.491	2.056	2.989
ENSG00000250859	HNRNPKP1	1.3	5.0E-03	1.0E-02	16.126	0.446	15.675	16.566	12.876	1.280	11.834	14.306
ENSG00000251192	ZNF674	1.3	3.9E-04	1.0E-03	3.874	0.087	3.782	3.954	2.940	0.033	2.902	2.961
ENSG00000251259		1.3	3.4E-03	7.1E-03	30.390	0.494	29.821	30.704	23.355	2.045	21.205	25.276
ENSG00000251400	ALDH7A1P1	1.3	4.0E-05	1.3E-04	60.364	4.704	57.442	65.791	48.496	4.667	43.230	52.121
ENSG00000253276	CCDC71L	1.3	5.4E-05	1.7E-04	13.904	0.389	13.539	14.313	11.030	0.699	10.331	11.729
ENSG00000253910	PCDHGB2	1.3	1.1E-01	1.6E-01	1.199	0.272	0.914	1.456	0.936	0.234	0.791	1.206
ENSG00000254332		1.3	2.1E-07	1.0E-06	169.559	1.141	168.504	170.770	134.048	2.489	131.415	136.362
ENSG00000254510		1.3	7.6E-02	1.2E-01	3.931	0.298	3.598	4.169	3.002	0.943	2.153	4.017
ENSG00000254535	PABPC4L	1.3	2.1E-04	5.7E-04	7.996	1.002	7.118	9.088	6.036	0.792	5.185	6.753
ENSG00000254759	NAP1L1P1	1.3	1.5E-03	3.5E-03	17.030	0.556	16.408	17.478	12.847	0.658	12.221	13.534
ENSG00000254999	BRK1	1.3	3.4E-12	4.5E-11	406.851	6.564	399.282	410.971	304.308	15.601	293.074	322.120
ENSG00000255356		1.3	8.0E-03	1.5E-02	30.064	2.599	27.106	31.982	22.437	1.618	20.745	23.969
ENSG00000255769	GOLGA2P10	1.3	2.0E-07	1.0E-06	46.434	1.681	45.201	48.349	35.378	0.627	34.658	35.810
ENSG00000255823	MTRNR2L8	1.3	1.0E-01	1.5E-01	2.936	0.427	2.479	3.325	2.230	0.265	2.066	2.536
ENSG00000255857	PXN-AS1	1.3	2.5E-03	5.4E-03	9.961	1.251	8.730	11.232	7.703	0.806	7.148	8.628
ENSG00000255867	HADHAP2	1.3	4.6E-02	7.4E-02	2.822	0.142	2.664	2.938	2.103	0.363	1.803	2.506
ENSG00000256043	CTSO	1.3	3.3E-02	5.6E-02	2.818	0.316	2.456	3.032	2.162	0.112	2.047	2.270
ENSG00000256393		1.3	2.5E-09	1.8E-08	3389.949	172.368	3216.129	3560.828	2576.523	167.027	2386.846	2701.612
ENSG00000256546		1.3	4.8E-03	9.7E-03	7.502	0.890	6.915	8.526	5.612	0.526	5.035	6.066
ENSG00000256745		1.3	5.8E-05	1.8E-04	135.456	5.806	131.180	142.066	108.486	7.789	100.588	116.161
ENSG00000256806	C17orf100	1.3	5.1E-02	8.1E-02	3.135	0.272	2.821	3.307	2.449	0.320	2.089	2.701
ENSG00000257002		1.3	8.7E-02	1.3E-01	6.578	1.237	5.152	7.368	5.004	0.624	4.289	5.444
ENSG00000258477	PPIAP6	1.3	1.7E-03	3.8E-03	53.160	5.407	47.645	58.452	41.561	4.813	36.676	46.299
ENSG00000258890	CEP95	1.3	1.8E-06	7.3E-06	14.839	1.028	13.655	15.512	11.877	0.179	11.742	12.080
ENSG00000259291	ZNF710-AS1	1.3	2.0E-02	3.5E-02	2.913	0.193	2.766	3.132	2.215	0.162	2.083	2.396
ENSG00000259366		1.3	6.0E-02	9.4E-02	14.298	0.566	13.652	14.703	11.321	1.414	10.188	12.906
ENSG00000260083	MIR762HG	1.3	5.8E-02	9.0E-02	3.366	0.303	3.061	3.667	2.601	0.110	2.479	2.694
ENSG00000260526		1.3	7.4E-02	1.1E-01	4.327	1.365	3.143	5.820	3.281	0.689	2.545	3.936
ENSG00000260917		1.3	1.4E-02	2.5E-02	3.141	0.622	2.769	3.859	2.426	0.299	2.223	2.769
ENSG00000261609	GAN	1.3	2.6E-07	1.3E-06	8.515	0.335	8.176	8.847	6.611	0.172	6.461	6.799
ENSG00000261716		1.3	4.0E-03	8.3E-03	4.383	0.351	4.083	4.769	3.403	0.131	3.323	3.554
ENSG00000265273		1.3	1.4E-01	2.0E-01	2.277	0.440	1.825	2.703	1.703	0.411	1.431	2.176
ENSG00000266145		1.3	1.2E-01	1.7E-01	12.469	3.119	9.141	15.325	9.271	3.074	6.394	12.510
ENSG00000266338	NBPF15	1.3	1.2E-04	3.5E-04	6.289	0.519	5.691	6.615	4.722	0.206	4.485	4.854
ENSG00000266916	ZNF793-AS1	1.3	4.8E-02	7.7E-02	5.454	0.590	4.791	5.920	4.246	0.654	3.510	4.758
ENSG00000266967	AARSD1	1.3	9.2E-02	1.4E-01	1.394	0.203	1.228	1.621	1.075	0.091	0.987	1.169
ENSG00000266992	DHX40P1	1.3	1.8E-02	3.2E-02	6.732	0.228	6.470	6.887	5.149	0.428	4.798	5.626
ENSG00000267278	MAP3K14-AS	1.3	5.4E-02	8.5E-02	2.074	0.151	1.978	2.249	1.632	0.213	1.443	1.862
ENSG00000268205		1.3	3.5E-03	7.4E-03	6.023	0.166	5.911	6.214	4.580	0.653	4.067	5.315
ENSG00000268403		1.3	1.2E-01	1.7E-01	6.416	0.764	5.578	7.076	5.081	1.350	3.532	6.006
ENSG00000269293	ZSCAN16-AS1	1.3	3.0E-02	5.1E-02	3.561	0.360	3.353	3.977	2.738	0.089	2.665	2.837
ENSG00000269378		1.3	3.2E-11	3.4E-10	552.683	15.280	538.319	568.739	420.585	3.854	418.071	425.023
ENSG00000269906		1.3	1.4E-01	1.9E-01	7.085	1.312	5.641	8.203	5.454	1.435	4.083	6.945
ENSG00000269958		1.3	1.7E-02	3.1E-02	15.651	4.333	10.658	18.414	11.752	0.469	11.368	12.274
ENSG00000270231	NBPF8	1.3	1.4E-02	2.6E-02	3.075	0.442	2.565	3.331	2.450	0.231	2.208	2.668
ENSG00000270277		1.3	1.4E-01	1.9E-01	2.293	0.070	2.214	2.347	1.775	0.134	1.632	1.896
ENSG00000270580		1.3	4.9E-02	7.8E-02	2.663	0.156	2.490	2.794	2.111	0.143	1.954	2.233
ENSG00000270629	NBPF14	1.3	3.7E-03	7.7E-03	1.968	0.250	1.682	2.143	1.566	0.078	1.505	1.654
ENSG00000270804		1.3	3.6E-02	5.9E-02	11.455	1.982	9.570	13.522	8.740	1.938	7.139	10.894
ENSG00000271425	NBPF10	1.3	1.2E-02	2.3E-02	1.005	0.032	0.971	1.035	0.785	0.089	0.726	0.888
ENSG00000271576		1.3	8.4E-03	1.6E-02	7.359	0.608	6.793	8.002	5.580	0.469	5.061	5.971
ENSG00000272079		1.3	6.5E-02	1.0E-01	3.803	0.140	3.649	3.922	2.849	0.712	2.312	3.656
ENSG00000272606		1.3	4.5E-02	7.3E-02	13.571	0.691	12.945	14.312	10.264	1.070	9.091	11.187
ENSG00000273015		1.3	2.4E-06	9.8E-06	6.897	0.458	6.377	7.240	5.161	0.511	4.625	5.642
ENSG00000273045	C2orf15	1.3	1.3E-02	2.4E-02	5.599	0.939	4.856	6.654	4.199	0.711	3.380	4.661
ENSG00000273142		1.3	4.7E-02	7.5E-02	2.768	0.544	2.217	3.306	2.175	0.430	1.757	2.615
ENSG00000273148		1.3	9.6E-02	1.4E-01	2.576	0.235	2.307	2.742	1.954	0.121	1.815	2.038
ENSG00000273373		1.3	1.5E-04	4.2E-04	10.581	0.652	10.182	11.334	8.013	0.643	7.273	8.430
ENSG00000274925		1.3	8.7E-03	1.7E-02	4.614	0.476	4.154	5.104	3.546	0.530	2.979	4.027
ENSG00000276256		1.3	2.8E-06	1.1E-05	52.499	2.411	50.107	54.928	42.091	1.542	40.979	43.851
ENSG00000276476		1.3	7.0E-03	1.4E-02	6.629	0.857	5.644	7.204	5.025	0.713	4.310	5.735
ENSG00000277666		1.3	8.8E-02	1.3E-01	9.495	1.772	7.467	10.748	7.355	0.878	6.549	8.291
ENSG00000277998		1.3	3.1E-09	2.2E-08	635.345	17.812	614.938	647.766	485.568	10.546	478.354	497.671
ENSG00000278129	ZNF8	1.3	1.5E-05	5.4E-05	9.043	0.522	8.518	9.562	7.095	0.524	6.513	7.532
ENSG00000278611		1.3	1.1E-01	1.6E-01	5.826	0.528	5.424	6.424	4.505	1.059	3.311	5.329
ENSG00000278662	GOLGA6L10	1.3	3.5E-02	5.9E-02	2.420	0.053	2.370	2.475	1.827	0.266	1.620	2.091
ENSG00000279039		1.3	6.3E-03	1.2E-02	5.836	0.623	5.169	6.403	4.495	0.485	3.937	4.816
ENSG00000279041		1.3	5.5E-04	1.4E-03	23.124	1.433	21.817	24.656	18.097	0.122	17.977	18.220
ENSG00000279089		1.3	5.0E-02	8.0E-02	2.283	0.274	1.970	2.478	1.787	0.072	1.740	1.869
ENSG00000280077		1.3	2.4E-02	4.1E-02	5.465	0.577	4.921	6.071	4.172	0.455	3.905	4.697
ENSG00000282416		1.3	7.5E-02	1.1E-01	6.260	0.843	5.287	6.765	4.752	0.656	4.130	5.438
ENSG00000282458		1.3	1.0E-04	3.0E-04	4.677	0.359	4.297	5.010	3.540	0.103	3.429	3.633
ENSG00000283093	CENPVL2	1.3	1.3E-01	1.9E-01	2.965	0.266	2.662	3.160	2.346	0.364	1.936	2.630
ENSG00000283341		1.3	1.1E-02	2.1E-02	3.140	0.261	2.841	3.325	2.334	0.298	2.082	2.662
ENSG00000003756	RBM5	1.4	1.3E-12	1.9E-11	27.683	0.285	27.460	28.004	20.332	0.278	20.156	20.652
ENSG00000005483	KMT2E	1.4	1.1E-12	1.5E-11	23.005	0.613	22.322	23.510	16.158	0.660	15.732	16.918
ENSG00000007314	SCN4A	1.4	4.7E-05	1.5E-04	6.139	0.202	5.927	6.331	4.264	0.347	3.906	4.599
ENSG00000008394	MGST1	1.4	1.5E-15	3.7E-14	156.373	4.421	152.938	161.361	109.870	3.475	106.542	113.476
ENSG00000008405	CRY1	1.4	3.4E-09	2.4E-08	30.521	1.267	29.059	31.297	22.492	0.983	21.551	23.511
ENSG00000008710	PKD1	1.4	4.8E-08	2.7E-07	7.025	0.893	6.140	7.926	4.880	0.170	4.775	5.076
ENSG00000011405	PIK3C2A	1.4	3.4E-13	5.4E-12	87.491	4.184	82.759	90.701	64.050	2.152	61.855	66.156
ENSG00000012061	ERCC1	1.4	9.7E-10	7.7E-09	20.468	1.542	19.074	22.124	14.404	0.195	14.208	14.599
ENSG00000025039	RRAGD	1.4	1.0E-07	5.4E-07	17.413	0.798	16.578	18.168	12.939	0.469	12.474	13.412
ENSG00000034053	APBA2	1.4	4.5E-10	3.8E-09	15.948	0.768	15.069	16.493	11.167	0.550	10.582	11.673
ENSG00000034713	GABARAPL2	1.4	3.3E-11	3.6E-10	57.417	3.404	54.222	60.997	40.313	1.913	38.230	41.989
ENSG00000038382	TRIO	1.4	1.0E-12	1.4E-11	19.233	0.657	18.795	19.988	14.057	0.303	13.802	14.392
ENSG00000043514	TRIT1	1.4	2.6E-09	1.9E-08	50.634	1.346	49.122	51.701	36.524	1.401	35.093	37.893
ENSG00000046647	GEMIN8	1.4	4.4E-08	2.5E-07	28.766	1.379	27.656	30.309	20.228	1.701	18.441	21.827
ENSG00000048471	SNX29	1.4	1.2E-05	4.4E-05	3.558	0.086	3.461	3.625	2.626	0.155	2.489	2.794
ENSG00000061273	HDAC7	1.4	1.9E-12	2.6E-11	31.454	1.964	29.188	32.650	22.188	1.182	21.398	23.556
ENSG00000063660	GPC1	1.4	5.0E-09	3.4E-08	21.596	1.653	19.705	22.764	15.073	0.962	14.106	16.030
ENSG00000065308	TRAM2	1.4	2.6E-12	3.5E-11	27.031	0.691	26.455	27.797	18.822	0.520	18.358	19.384
ENSG00000065357	DGKA	1.4	9.8E-07	4.3E-06	4.145	0.468	3.605	4.433	2.866	0.243	2.670	3.137
ENSG00000066923	STAG3	1.4	3.5E-04	9.2E-04	2.322	0.080	2.254	2.410	1.674	0.115	1.549	1.774
ENSG00000067191	CACNB1	1.4	3.9E-04	1.0E-03	2.688	0.367	2.265	2.906	1.866	0.083	1.784	1.949
ENSG00000068615	REEP1	1.4	2.5E-02	4.3E-02	1.194	0.097	1.084	1.262	0.878	0.077	0.810	0.961
ENSG00000070444	MNT	1.4	9.1E-08	4.8E-07	7.251	0.458	6.862	7.755	5.061	0.353	4.849	5.468
ENSG00000070756	PABPC1	1.4	6.1E-16	1.6E-14	1069.471	8.562	1059.960	1076.565	794.347	18.366	779.754	814.970
ENSG00000073792	IGF2BP2	1.4	1.3E-15	3.2E-14	103.538	1.506	102.624	105.276	72.780	1.050	72.018	73.926
ENSG00000074410	CA12	1.4	8.2E-05	2.5E-04	3.061	0.132	2.955	3.208	2.163	0.162	2.063	2.349
ENSG00000074590	NUAK1	1.4	6.1E-10	5.0E-09	19.391	0.138	19.250	19.526	13.721	0.354	13.361	14.068
ENSG00000077274	CAPN6	1.4	1.3E-08	8.0E-08	28.767	1.287	28.015	30.253	21.029	0.765	20.202	21.711
ENSG00000078043	PIAS2	1.4	3.7E-10	3.2E-09	10.644	0.154	10.471	10.765	7.835	0.575	7.204	8.329
ENSG00000078142	PIK3C3	1.4	1.7E-10	1.6E-09	13.501	0.372	13.101	13.837	10.007	0.369	9.586	10.278
ENSG00000078246	TULP3	1.4	2.9E-07	1.4E-06	28.408	1.254	27.172	29.680	21.012	1.308	20.068	22.506
ENSG00000078487	ZCWPW1	1.4	6.1E-02	9.5E-02	1.383	0.226	1.163	1.614	1.019	0.103	0.945	1.137
ENSG00000079432	CIC	1.4	2.4E-09	1.7E-08	14.797	0.104	14.686	14.892	10.442	0.771	9.637	11.174
ENSG00000080298	RFX3	1.4	4.8E-08	2.7E-07	10.126	0.406	9.777	10.572	7.293	0.380	6.861	7.579
ENSG00000081818	PCDHB4	1.4	8.8E-02	1.3E-01	0.814	0.152	0.642	0.933	0.590	0.085	0.504	0.674
ENSG00000082641	NFE2L1	1.4	5.5E-15	1.2E-13	203.091	3.177	199.488	205.488	145.502	2.052	143.923	147.821
ENSG00000083454	P2RX5	1.4	1.6E-02	2.9E-02	2.163	0.645	1.550	2.836	1.579	0.126	1.434	1.660
ENSG00000086300	SNX10	1.4	1.1E-07	5.8E-07	26.907	1.693	25.220	28.607	19.668	1.376	18.257	21.005
ENSG00000086991	NOX4	1.4	2.6E-02	4.4E-02	1.323	0.042	1.283	1.367	0.974	0.244	0.693	1.130
ENSG00000087510	TFAP2C	1.4	7.2E-04	1.8E-03	5.168	0.586	4.495	5.564	3.697	0.432	3.254	4.117
ENSG00000088002	SULT2B1	1.4	8.6E-02	1.3E-01	1.286	0.166	1.108	1.438	0.920	0.159	0.773	1.089
ENSG00000088448	ANKRD10	1.4	9.4E-13	1.4E-11	91.833	2.726	90.214	94.980	65.265	4.129	62.516	70.012
ENSG00000088543	C3orf18	1.4	3.6E-05	1.1E-04	9.223	0.436	8.746	9.599	6.689	0.768	6.214	7.575
ENSG00000090238	YPEL3	1.4	5.3E-05	1.6E-04	10.441	0.636	10.064	11.175	7.694	0.549	7.070	8.102
ENSG00000090932	DLL3	1.4	3.3E-03	6.9E-03	8.698	1.299	7.223	9.672	6.414	0.491	5.854	6.771
ENSG00000096093	EFHC1	1.4	2.0E-08	1.2E-07	3.459	0.317	3.225	3.820	2.417	0.215	2.230	2.653
ENSG00000096968	JAK2	1.4	5.1E-04	1.3E-03	3.291	0.546	2.950	3.920	2.327	0.321	2.100	2.694
ENSG00000099219	ERMP1	1.4	8.9E-12	1.1E-10	31.912	0.537	31.474	32.511	22.725	0.261	22.549	23.025
ENSG00000099942	CRKL	1.4	1.8E-13	3.0E-12	126.103	0.585	125.527	126.696	91.800	1.882	89.628	92.910
ENSG00000099977	DDT	1.4	1.6E-08	9.8E-08	71.722	7.547	63.915	78.979	50.457	5.165	45.474	55.787
ENSG00000100027	YPEL1	1.4	1.9E-04	5.3E-04	13.092	0.586	12.641	13.755	9.656	2.080	7.617	11.776
ENSG00000100122	CRYBB1	1.4	2.1E-02	3.7E-02	6.483	0.465	6.039	6.967	4.652	1.008	4.056	5.816
ENSG00000100316	RPL3	1.4	9.0E-17	2.6E-15	1366.070	38.597	1325.048	1401.669	985.403	22.800	968.606	1011.359
ENSG00000100320	RBFOX2	1.4	3.6E-15	8.3E-14	85.432	1.475	83.881	86.817	61.660	1.794	59.759	63.322
ENSG00000100353	EIF3D	1.4	1.8E-15	4.3E-14	222.020	9.007	213.176	231.182	157.143	3.413	153.451	160.184
ENSG00000100376	FAM118A	1.4	1.2E-09	9.5E-09	15.542	1.061	14.751	16.748	10.866	0.428	10.575	11.358
ENSG00000100814	CCNB1IP1	1.4	1.8E-13	3.1E-12	155.500	6.594	148.292	161.227	113.019	2.349	110.536	115.206
ENSG00000101104	PABPC1L	1.4	7.7E-08	4.2E-07	19.743	2.213	17.559	21.983	14.424	0.325	14.227	14.798
ENSG00000101236	RNF24	1.4	3.4E-10	3.0E-09	12.191	0.155	12.024	12.331	8.489	0.440	8.143	8.984
ENSG00000101493	ZNF516	1.4	3.5E-06	1.4E-05	4.980	0.356	4.586	5.278	3.505	0.130	3.369	3.627
ENSG00000102218	RP2	1.4	9.3E-07	4.1E-06	18.764	1.321	17.249	19.676	13.788	0.552	13.161	14.141
ENSG00000102265	TIMP1	1.4	1.8E-07	9.1E-07	59.236	2.830	57.484	62.501	43.458	3.622	40.338	47.430
ENSG00000102271	KLHL4	1.4	4.1E-09	2.9E-08	51.878	2.220	49.318	53.277	36.901	1.019	35.837	37.867
ENSG00000102409	BEX4	1.4	6.3E-12	7.8E-11	148.500	4.499	145.283	153.641	106.414	0.890	105.660	107.396
ENSG00000102780	DGKH	1.4	8.5E-07	3.7E-06	3.910	0.238	3.635	4.052	2.886	0.126	2.743	2.981
ENSG00000103196	CRISPLD2	1.4	2.8E-05	9.2E-05	3.675	0.506	3.374	4.259	2.578	0.201	2.436	2.808
ENSG00000103227	LMF1	1.4	2.2E-03	4.9E-03	1.316	0.236	1.050	1.498	0.923	0.060	0.878	0.990
ENSG00000104067	TJP1	1.4	1.1E-15	2.7E-14	78.022	2.971	74.592	79.755	55.940	0.745	55.156	56.639
ENSG00000104231	ZFAND1	1.4	3.0E-09	2.1E-08	36.092	1.415	34.503	37.217	26.539	0.732	26.057	27.381
ENSG00000104671	DCTN6	1.4	9.7E-07	4.2E-06	32.459	1.235	31.041	33.298	24.060	1.612	22.956	25.910
ENSG00000104695	PPP2CB	1.4	4.2E-14	8.0E-13	88.432	1.616	86.695	89.890	62.288	1.003	61.281	63.287
ENSG00000104728	ARHGEF10	1.4	2.0E-05	6.9E-05	3.120	0.169	2.982	3.309	2.209	0.287	1.916	2.488
ENSG00000104808	DHDH	1.4	7.5E-02	1.1E-01	2.796	0.491	2.236	3.153	1.929	0.655	1.292	2.601
ENSG00000104812	GYS1	1.4	1.8E-11	2.1E-10	39.977	0.577	39.415	40.568	28.516	1.461	27.043	29.964
ENSG00000104823	ECH1	1.4	4.7E-10	4.0E-09	54.021	3.760	49.682	56.322	38.248	1.891	36.131	39.770
ENSG00000104885	DOT1L	1.4	3.5E-09	2.5E-08	18.854	1.661	17.760	20.765	13.680	0.861	12.987	14.650
ENSG00000104946	TBC1D17	1.4	4.1E-09	2.9E-08	19.150	0.797	18.233	19.680	13.689	0.436	13.421	14.193
ENSG00000105641	SLC5A5	1.4	5.1E-02	8.1E-02	1.254	0.360	0.934	1.645	0.886	0.208	0.650	1.045
ENSG00000105771	SMG9	1.4	1.3E-07	6.9E-07	7.289	0.287	7.085	7.617	5.235	0.265	5.057	5.539
ENSG00000106211	HSPB1	1.4	5.5E-09	3.8E-08	121.363	7.075	115.464	129.206	88.209	9.234	77.695	94.997
ENSG00000106367	AP1S1	1.4	6.4E-10	5.3E-09	84.724	2.988	82.941	88.173	60.529	3.444	57.818	64.404
ENSG00000106404	CLDN15	1.4	5.6E-03	1.1E-02	3.015	0.286	2.721	3.293	2.123	0.448	1.835	2.640
ENSG00000106415	GLCCI1	1.4	1.6E-08	9.9E-08	15.422	0.541	14.807	15.827	11.447	0.445	10.933	11.707
ENSG00000107331	ABCA2	1.4	1.6E-06	6.6E-06	4.241	0.512	3.853	4.821	2.946	0.088	2.833	3.009
ENSG00000108187	PBLD	1.4	2.5E-02	4.3E-02	1.363	0.088	1.283	1.457	0.993	0.090	0.891	1.060
ENSG00000108599	AKAP10	1.4	6.0E-08	3.3E-07	22.069	1.621	20.881	23.932	16.344	0.405	15.876	16.595
ENSG00000108691	CCL2	1.4	1.7E-02	3.1E-02	3.318	0.849	2.370	4.009	2.334	0.449	1.923	2.813
ENSG00000108773	KAT2A	1.4	8.5E-10	6.8E-09	31.437	1.918	29.649	33.463	22.696	1.096	21.793	23.915
ENSG00000108840	HDAC5	1.4	2.5E-12	3.4E-11	33.366	0.238	33.092	33.512	23.472	1.259	22.261	24.774
ENSG00000108848	LUC7L3	1.4	7.2E-13	1.1E-11	100.476	8.640	90.534	106.165	73.353	1.866	71.468	75.200
ENSG00000109775	UFSP2	1.4	1.6E-10	1.4E-09	46.547	0.239	46.271	46.694	32.615	0.631	32.247	33.343
ENSG00000110031	LPXN	1.4	4.5E-02	7.2E-02	2.373	0.396	1.974	2.767	1.764	0.548	1.189	2.280
ENSG00000110427	KIAA1549L	1.4	1.5E-03	3.3E-03	1.890	0.145	1.742	2.032	1.395	0.290	1.130	1.705
ENSG00000110492	MDK	1.4	1.1E-13	1.9E-12	250.757	11.512	240.798	263.362	176.534	12.878	164.623	190.199
ENSG00000110841	PPFIBP1	1.4	2.6E-10	2.3E-09	15.333	0.535	14.809	15.878	10.983	0.286	10.664	11.214
ENSG00000111011	RSRC2	1.4	2.1E-12	2.9E-11	53.006	3.002	49.548	54.949	37.941	1.079	36.879	39.037
ENSG00000111057	KRT18	1.4	4.9E-15	1.1E-13	419.924	19.691	398.172	436.534	297.603	9.772	290.320	308.708
ENSG00000111325	OGFOD2	1.4	6.7E-02	1.0E-01	0.970	0.018	0.951	0.987	0.705	0.236	0.546	0.976
ENSG00000111704	NANOG	1.4	2.7E-12	3.6E-11	60.617	0.562	60.011	61.121	45.021	1.470	44.095	46.716
ENSG00000112139	MDGA1	1.4	5.5E-02	8.7E-02	0.398	0.033	0.371	0.435	0.290	0.086	0.217	0.384
ENSG00000112232	KHDRBS2	1.4	3.7E-04	9.6E-04	11.963	1.866	9.999	13.712	8.554	0.157	8.380	8.686
ENSG00000112658	SRF	1.4	1.1E-06	4.7E-06	74.475	1.362	73.530	76.036	53.199	3.839	49.034	56.596
ENSG00000113272	THG1L	1.4	2.0E-08	1.2E-07	20.439	0.878	19.458	21.151	14.214	0.721	13.448	14.880
ENSG00000113328	CCNG1	1.4	1.1E-13	2.0E-12	565.255	10.163	554.978	575.300	412.598	20.991	389.487	430.480
ENSG00000113966	ARL6	1.4	1.2E-04	3.4E-04	9.344	0.822	8.801	10.289	6.744	0.501	6.380	7.315
ENSG00000115020	PIKFYVE	1.4	6.9E-15	1.5E-13	40.013	1.657	38.573	41.824	28.151	0.490	27.777	28.706
ENSG00000115073	ACTR1B	1.4	8.6E-09	5.7E-08	37.034	2.729	34.023	39.345	26.572	0.905	25.793	27.564
ENSG00000115137	DNAJC27	1.4	6.1E-04	1.5E-03	3.294	0.363	2.882	3.565	2.395	0.207	2.250	2.633
ENSG00000115289	PCGF1	1.4	4.3E-03	8.9E-03	4.827	0.291	4.543	5.125	3.574	0.171	3.401	3.743
ENSG00000115310	RTN4	1.4	2.4E-16	6.8E-15	190.289	3.455	186.422	193.075	133.852	3.684	130.353	137.715
ENSG00000115808	STRN	1.4	9.8E-13	1.4E-11	49.898	2.195	47.868	52.226	37.022	1.113	36.031	38.227
ENSG00000116106	EPHA4	1.4	2.3E-05	7.5E-05	3.325	0.141	3.167	3.438	2.335	0.055	2.275	2.382
ENSG00000116260	QSOX1	1.4	3.8E-13	6.0E-12	49.665	1.603	48.220	51.389	36.186	1.562	34.401	37.296
ENSG00000116473	RAP1A	1.4	3.3E-10	2.9E-09	31.673	0.223	31.440	31.884	23.050	1.293	21.667	24.228
ENSG00000116604	MEF2D	1.4	5.7E-08	3.2E-07	12.045	0.135	11.908	12.179	8.608	0.830	7.727	9.375
ENSG00000116661	FBXO2	1.4	1.1E-08	6.8E-08	36.927	1.296	35.990	38.406	26.543	1.436	24.908	27.600
ENSG00000116863	ADPRHL2	1.4	1.0E-08	6.4E-08	53.478	5.306	47.586	57.881	37.297	0.672	36.681	38.014
ENSG00000116984	MTR	1.4	9.0E-11	8.7E-10	23.154	1.459	21.672	24.589	17.093	0.758	16.230	17.648
ENSG00000116991	SIPA1L2	1.4	6.4E-08	3.5E-07	11.088	0.780	10.544	11.981	7.901	0.526	7.459	8.482
ENSG00000117481	NSUN4	1.4	9.5E-09	6.2E-08	8.742	0.155	8.585	8.894	6.274	0.221	6.021	6.425
ENSG00000117525	F3	1.4	1.0E-03	2.4E-03	5.695	0.669	4.939	6.210	4.148	0.623	3.547	4.790
ENSG00000118276	B4GALT6	1.4	3.4E-08	2.0E-07	32.169	2.190	29.671	33.765	23.778	1.643	22.693	25.668
ENSG00000118680	MYL12B	1.4	6.0E-14	1.1E-12	406.089	8.363	396.753	412.896	297.252	1.012	296.355	298.350
ENSG00000118816	CCNI	1.4	1.5E-15	3.7E-14	225.431	2.710	223.108	228.408	159.893	3.042	157.425	163.292
ENSG00000119321	FKBP15	1.4	8.6E-09	5.6E-08	9.574	0.497	9.143	10.118	7.058	0.250	6.830	7.325
ENSG00000119537	KDSR	1.4	7.8E-11	7.6E-10	19.121	1.051	17.928	19.911	13.445	0.770	12.785	14.291
ENSG00000119684	MLH3	1.4	2.4E-08	1.5E-07	10.041	0.443	9.689	10.539	7.269	0.526	6.715	7.761
ENSG00000119723	COQ6	1.4	5.3E-05	1.6E-04	3.130	0.135	2.977	3.232	2.192	0.226	2.019	2.447
ENSG00000120725	SIL1	1.4	2.3E-04	6.1E-04	4.554	0.190	4.371	4.750	3.199	0.240	2.924	3.365
ENSG00000121903	ZSCAN20	1.4	9.1E-03	1.7E-02	2.934	0.160	2.752	3.054	2.156	0.689	1.430	2.800
ENSG00000121931	LRIF1	1.4	2.5E-08	1.5E-07	32.512	2.760	30.222	35.576	23.858	1.292	23.009	25.345
ENSG00000122971	ACADS	1.4	3.6E-04	9.3E-04	6.993	0.215	6.820	7.233	5.049	0.356	4.685	5.407
ENSG00000123119	NECAB1	1.4	1.6E-02	2.9E-02	1.544	0.378	1.108	1.775	1.144	0.288	0.851	1.426
ENSG00000123268	ATF1	1.4	2.5E-07	1.2E-06	35.059	3.690	30.980	38.165	25.900	1.883	24.490	28.038
ENSG00000123572	NRK	1.4	5.7E-07	2.6E-06	5.158	0.295	4.985	5.498	3.628	0.121	3.490	3.716
ENSG00000124225	PMEPA1	1.4	7.7E-08	4.2E-07	12.910	0.578	12.334	13.491	9.212	0.688	8.552	9.926
ENSG00000124343	XG	1.4	9.2E-02	1.4E-01	1.214	0.328	0.948	1.580	0.867	0.145	0.705	0.983
ENSG00000124602	UNC5CL	1.4	4.3E-03	8.9E-03	3.107	0.139	3.002	3.264	2.256	0.259	2.004	2.522
ENSG00000124733	MEA1	1.4	1.6E-12	2.2E-11	281.387	10.906	271.563	293.122	206.152	1.879	204.481	208.185

ENSG00000124831	LRRFIP1	1.4	7.8E-12	9.5E-11	41.149	0.751	40.679	42.015	30.053	0.651	29.324	30.576
ENSG00000124875	CXCL6	1.4	2.4E-04	6.5E-04	10.489	1.197	9.333	11.724	7.656	0.804	7.084	8.575
ENSG00000125249	RAP2A	1.4	6.6E-12	8.2E-11	55.723	1.179	54.396	56.651	40.688	1.765	39.048	42.559
ENSG00000125354	SEPT6	1.4	5.8E-07	2.6E-06	10.731	0.677	10.215	11.498	7.889	0.292	7.555	8.097
ENSG00000125821	DTD1	1.4	1.8E-12	2.5E-11	69.710	0.191	69.547	69.921	49.876	1.794	48.212	51.777
ENSG00000126368	NR1D1	1.4	9.2E-03	1.7E-02	4.190	0.711	3.385	4.733	3.005	0.117	2.872	3.094
ENSG00000126583	PRKCG	1.4	1.5E-04	4.2E-04	8.445	0.248	8.159	8.594	6.250	0.721	5.536	6.977
ENSG00000126790	L3HYPDH	1.4	1.8E-05	6.2E-05	9.681	0.629	9.074	10.330	6.955	0.679	6.266	7.623
ENSG00000127241	MASP1	1.4	3.7E-03	7.7E-03	0.975	0.182	0.853	1.185	0.707	0.052	0.647	0.746
ENSG00000128536	CDHR3	1.4	4.3E-04	1.1E-03	2.628	0.056	2.568	2.680	1.929	0.146	1.764	2.040
ENSG00000129566	TEP1	1.4	9.0E-08	4.8E-07	4.917	0.438	4.415	5.216	3.456	0.059	3.388	3.491
ENSG00000130066	SAT1	1.4	1.4E-10	1.3E-09	62.872	0.778	61.974	63.352	46.151	1.372	44.817	47.558
ENSG00000130208	APOC1	1.4	5.3E-08	2.9E-07	73.189	5.166	67.310	77.006	52.732	3.884	48.341	55.719
ENSG00000130433	CACNG6	1.4	1.2E-02	2.3E-02	4.538	0.495	4.082	5.064	3.190	0.682	2.731	3.974
ENSG00000130449	ZSWIM6	1.4	2.2E-09	1.6E-08	22.261	1.206	20.960	23.340	15.978	1.066	15.022	17.073
ENSG00000130985	UBA1	1.4	1.7E-14	3.4E-13	256.188	2.741	253.301	258.751	187.359	5.789	181.202	192.691
ENSG00000131018	SYNE1	1.4	1.1E-03	2.6E-03	0.391	0.070	0.348	0.472	0.282	0.026	0.263	0.312
ENSG00000131378	RFTN1	1.4	4.8E-05	1.5E-04	5.839	0.159	5.657	5.946	4.303	0.079	4.212	4.357
ENSG00000131626	PPFIA1	1.4	1.2E-10	1.1E-09	31.081	0.536	30.462	31.409	22.929	0.225	22.707	23.157
ENSG00000131931	THAP1	1.4	7.1E-06	2.6E-05	22.615	2.021	20.563	24.603	16.454	2.315	13.847	18.270
ENSG00000131944	FAAP24	1.4	5.6E-03	1.1E-02	4.512	0.610	4.080	5.210	3.122	0.662	2.358	3.510
ENSG00000132164	SLC6A11	1.4	1.3E-02	2.5E-02	1.183	0.108	1.060	1.260	0.851	0.157	0.712	1.022
ENSG00000132749	TESMIN	1.4	1.2E-08	7.9E-08	19.637	1.090	18.380	20.314	14.517	0.674	13.739	14.920
ENSG00000133138	TBC1D8B	1.4	5.0E-07	2.3E-06	5.448	0.352	5.127	5.825	3.877	0.316	3.578	4.209
ENSG00000133488	SEC14L4	1.4	7.0E-04	1.7E-03	7.665	0.942	7.117	8.753	5.519	0.358	5.117	5.803
ENSG00000133624	ZNF767P	1.4	1.9E-05	6.4E-05	8.105	0.667	7.335	8.523	5.803	0.539	5.180	6.124
ENSG00000133636	NTS	1.4	4.3E-10	3.7E-09	94.897	3.695	92.627	99.160	65.684	5.755	61.122	72.150
ENSG00000133789	SWAP70	1.4	2.8E-14	5.6E-13	41.433	0.457	40.916	41.786	28.824	0.848	28.316	29.803
ENSG00000134245	WNT2B	1.4	6.9E-02	1.1E-01	0.364	0.113	0.238	0.455	0.262	0.049	0.206	0.300
ENSG00000135074	ADAM19	1.4	8.2E-09	5.4E-08	18.319	0.402	17.958	18.753	13.519	0.466	13.200	14.054
ENSG00000135124	P2RX4	1.4	4.9E-05	1.5E-04	2.724	0.361	2.318	3.008	1.883	0.049	1.827	1.918
ENSG00000135365	PHF21A	1.4	2.4E-09	1.8E-08	10.201	0.062	10.153	10.270	7.524	0.045	7.478	7.568
ENSG00000135679	MDM2	1.4	4.1E-03	8.6E-03	25.848	1.151	24.531	26.661	18.325	0.573	17.676	18.761
ENSG00000135749	PCNX2	1.4	7.2E-12	8.9E-11	23.441	1.283	22.434	24.885	17.405	0.325	17.038	17.657
ENSG00000135929	CYP27A1	1.4	4.7E-03	9.7E-03	3.156	0.443	2.742	3.623	2.226	0.623	1.509	2.639
ENSG00000136010	ALDH1L2	1.4	3.2E-07	1.5E-06	9.987	0.602	9.308	10.456	7.365	0.300	7.069	7.668
ENSG00000136048	DRAM1	1.4	3.0E-04	8.1E-04	5.481	0.547	4.864	5.908	4.008	0.239	3.767	4.244
ENSG00000136143	SUCLA2	1.4	2.5E-05	8.2E-05	89.247	1.953	87.905	91.488	62.385	2.330	59.782	64.274
ENSG00000136149	RPL13AP25	1.4	1.4E-12	1.9E-11	542.308	20.549	518.706	556.217	383.344	31.063	354.105	415.956
ENSG00000136156	ITM2B	1.4	8.1E-13	1.2E-11	48.891	1.284	47.408	49.647	35.970	1.055	35.244	37.179
ENSG00000136158	SPRY2	1.4	5.9E-11	6.0E-10	52.620	0.802	52.045	53.537	38.231	1.177	37.106	39.454
ENSG00000136237	RAPGEF5	1.4	2.5E-08	1.5E-07	7.698	0.404	7.232	7.938	5.482	0.195	5.271	5.654
ENSG00000136267	DGKB	1.4	2.9E-03	6.3E-03	1.264	0.150	1.169	1.437	0.898	0.083	0.839	0.992
ENSG00000136451	VEZF1	1.4	2.0E-11	2.3E-10	52.733	1.530	51.755	54.496	37.909	1.663	36.096	39.362
ENSG00000136811	ODF2	1.4	4.1E-10	3.5E-09	20.862	1.033	19.703	21.685	14.755	0.213	14.530	14.954
ENSG00000137193	PIM1	1.4	1.6E-11	1.9E-10	117.267	2.994	114.294	120.281	85.959	5.229	81.833	91.840
ENSG00000138380	CARF	1.4	7.4E-05	2.2E-04	2.243	0.031	2.223	2.279	1.564	0.151	1.400	1.697
ENSG00000138413	IDH1	1.4	3.8E-17	1.2E-15	548.209	3.408	545.174	551.897	393.753	7.370	385.416	399.403
ENSG00000138696	BMPR1B	1.4	2.3E-02	4.0E-02	1.374	0.079	1.320	1.464	1.021	0.181	0.821	1.173
ENSG00000138771	SHROOM3	1.4	7.4E-14	1.3E-12	28.762	0.958	27.916	29.802	19.890	1.225	18.545	20.943
ENSG00000138944	KIAA1644	1.4	2.6E-06	1.0E-05	7.993	0.695	7.329	8.716	5.804	0.402	5.488	6.259
ENSG00000139219	COL2A1	1.4	1.1E-04	3.2E-04	9.789	0.875	8.962	10.705	6.962	0.683	6.311	7.691
ENSG00000139496	NUP58	1.4	2.0E-14	4.0E-13	44.090	0.737	43.652	44.941	31.203	0.451	30.703	31.578
ENSG00000139714	MORN3	1.4	7.0E-02	1.1E-01	1.005	0.231	0.761	1.221	0.720	0.156	0.543	0.839
ENSG00000140534	TICRR	1.4	1.5E-10	1.4E-09	28.772	0.646	28.327	29.514	21.379	0.519	21.001	21.971
ENSG00000140795	MYLK3	1.4	3.3E-02	5.5E-02	0.529	0.022	0.508	0.551	0.379	0.049	0.323	0.415
ENSG00000140859	KIFC3	1.4	1.0E-04	3.1E-04	3.755	0.276	3.436	3.923	2.614	0.389	2.314	3.054
ENSG00000141570	CBX8	1.4	1.1E-01	1.6E-01	0.863	0.244	0.624	1.112	0.633	0.184	0.439	0.805
ENSG00000142541	RPL13A	1.4	3.0E-17	9.8E-16	757.834	28.498	727.771	784.453	525.064	17.508	505.012	537.319
ENSG00000142549	IGLON5	1.4	6.4E-08	3.5E-07	32.725	0.612	32.028	33.168	22.960	2.913	19.645	25.114
ENSG00000142867	BCL10	1.4	1.3E-07	6.8E-07	25.478	1.236	24.414	26.833	18.478	0.895	17.805	19.494
ENSG00000143248	RGS5	1.4	3.6E-12	4.7E-11	37.823	1.544	36.930	39.606	26.512	1.249	25.589	27.934
ENSG00000143344	RGL1	1.4	1.1E-03	2.6E-03	2.975	0.311	2.653	3.274	2.147	0.219	1.988	2.396
ENSG00000143420	ENSA	1.4	1.9E-13	3.2E-12	141.050	1.956	139.344	143.186	103.177	6.027	96.270	107.370
ENSG00000143768	LEFTY2	1.4	5.7E-05	1.8E-04	13.892	0.724	13.174	14.623	10.284	0.175	10.113	10.462
ENSG00000145247	OCIAD2	1.4	4.4E-12	5.7E-11	114.132	5.422	108.000	118.295	80.053	1.570	78.817	81.819
ENSG00000146021	KLHL3	1.4	2.0E-07	9.9E-07	6.028	0.508	5.521	6.537	4.385	0.300	4.054	4.637
ENSG00000146038	DCDC2	1.4	2.5E-02	4.3E-02	0.997	0.022	0.975	1.020	0.694	0.088	0.619	0.792
ENSG00000146205	ANO7	1.4	7.6E-04	1.9E-03	2.687	0.027	2.656	2.708	1.923	0.284	1.742	2.251
ENSG00000146267	FAXC	1.4	4.0E-08	2.3E-07	5.623	0.079	5.532	5.679	4.042	0.217	3.905	4.292
ENSG00000146373	RNF217	1.4	7.4E-06	2.7E-05	2.806	0.055	2.744	2.848	2.024	0.076	1.936	2.070
ENSG00000146677		1.4	7.9E-13	1.2E-11	1150.808	60.620	1095.787	1215.792	812.321	24.447	785.147	832.528
ENSG00000146729	NIPSNAP2	1.4	1.2E-12	1.8E-11	36.401	1.566	34.622	37.571	25.802	0.582	25.214	26.397
ENSG00000147364	FBXO25	1.4	1.8E-06	7.6E-06	3.155	0.077	3.094	3.242	2.247	0.141	2.162	2.409
ENSG00000147394	ZNF185	1.4	1.5E-07	7.8E-07	15.543	1.027	14.479	16.529	11.367	0.915	10.740	12.417
ENSG00000147400	CETN2	1.4	9.3E-11	9.0E-10	82.711	2.451	81.177	85.538	57.202	2.193	55.733	59.723
ENSG00000147421	HMBOX1	1.4	2.1E-06	8.5E-06	9.075	0.870	8.097	9.763	6.559	0.079	6.475	6.633
ENSG00000147676	MAL2	1.4	1.3E-13	2.3E-12	212.017	3.450	208.033	214.068	156.349	8.088	147.017	161.313
ENSG00000147853	AK3	1.4	1.7E-11	2.0E-10	47.802	2.289	45.792	50.293	34.880	1.506	33.672	36.567
ENSG00000147996	CBWD5	1.4	3.8E-08	2.2E-07	5.005	0.214	4.809	5.234	3.552	0.253	3.264	3.734
ENSG00000148225	WDR31	1.4	5.2E-02	8.3E-02	0.793	0.013	0.782	0.807	0.563	0.116	0.453	0.684
ENSG00000148925	BTBD10	1.4	7.7E-11	7.6E-10	39.262	1.095	37.998	39.901	27.815	1.019	26.668	28.617
ENSG00000149485	FADS1	1.4	3.2E-15	7.5E-14	85.648	0.870	85.079	86.650	61.728	0.850	61.033	62.676
ENSG00000149573	MPZL2	1.4	1.2E-03	2.9E-03	2.869	0.441	2.391	3.261	2.017	0.316	1.705	2.337
ENSG00000150672	DLG2	1.4	3.6E-06	1.4E-05	3.140	0.062	3.075	3.198	2.297	0.138	2.150	2.424
ENSG00000150768	DLAT	1.4	2.3E-12	3.2E-11	88.996	4.272	86.008	93.889	65.695	1.303	64.610	67.141
ENSG00000151414	NEK7	1.4	3.1E-07	1.5E-06	14.639	0.324	14.267	14.854	10.838	0.870	10.304	11.842
ENSG00000151748	SAV1	1.4	1.0E-12	1.5E-11	59.111	2.562	56.517	61.640	42.199	0.708	41.541	42.949
ENSG00000151881	TMEM267	1.4	7.7E-05	2.3E-04	9.062	0.232	8.871	9.320	6.686	0.218	6.434	6.812
ENSG00000151952	TMEM132D	1.4	1.7E-06	7.1E-06	8.498	0.250	8.280	8.770	6.301	0.056	6.245	6.358
ENSG00000152208	GRID2	1.4	6.9E-14	1.3E-12	48.108	0.855	47.550	49.092	33.514	0.489	32.956	33.867
ENSG00000152217	SETBP1	1.4	2.1E-05	7.2E-05	2.834	0.152	2.741	3.009	1.970	0.179	1.866	2.177
ENSG00000152558	TMEM123	1.4	8.5E-10	6.8E-09	98.387	6.509	92.503	105.378	72.355	3.822	68.372	75.991
ENSG00000152580	IGSF10	1.4	2.4E-04	6.4E-04	2.157	0.138	2.007	2.279	1.543	0.112	1.415	1.620
ENSG00000154079	SDHAF4	1.4	1.4E-07	7.2E-07	42.026	0.416	41.570	42.383	29.726	2.961	27.367	33.049
ENSG00000154359	LONRF1	1.4	1.9E-12	2.6E-11	59.772	0.801	58.926	60.518	44.085	1.012	43.319	45.232
ENSG00000154639	CXADR	1.4	1.9E-14	3.9E-13	127.389	3.705	123.193	130.210	90.020	5.074	84.807	94.942
ENSG00000154640	BTG3	1.4	6.8E-05	2.1E-04	37.439	2.709	35.146	40.428	26.927	0.911	26.290	27.970
ENSG00000155096	AZIN1	1.4	1.3E-15	3.2E-14	271.082	4.428	266.495	275.332	195.413	4.968	189.774	199.142
ENSG00000155368	DBI	1.4	2.4E-14	4.8E-13	246.245	9.430	235.553	253.377	173.797	0.936	172.981	174.819
ENSG00000155508	CNOT8	1.4	3.9E-12	5.1E-11	77.014	3.534	72.939	79.217	56.534	0.597	55.982	57.167
ENSG00000155729	KCTD18	1.4	2.0E-04	5.4E-04	4.027	0.463	3.643	4.542	2.880	0.184	2.717	3.080
ENSG00000155858	LSM11	1.4	3.1E-07	1.5E-06	9.139	0.342	8.744	9.344	6.581	0.490	6.089	7.068
ENSG00000155926	SLA	1.4	9.8E-03	1.9E-02	1.221	0.071	1.154	1.297	0.853	0.217	0.622	1.053
ENSG00000156006	NAT2	1.4	1.0E-01	1.5E-01	3.071	0.713	2.456	3.853	2.275	0.741	1.595	3.064
ENSG00000156030	ELMSAN1	1.4	7.1E-07	3.2E-06	4.920	0.177	4.724	5.068	3.496	0.465	3.118	4.015
ENSG00000156049	GNA14	1.4	1.7E-04	4.6E-04	19.761	0.925	18.910	20.745	14.545	1.115	13.348	15.553
ENSG00000156508	EEF1A1	1.4	9.0E-18	3.2E-16	2869.466	96.669	2776.362	2969.343	2017.959	54.962	1970.476	2078.168
ENSG00000156515	HK1	1.4	1.2E-16	3.4E-15	245.501	4.477	240.428	248.895	170.972	3.485	167.290	174.219
ENSG00000157214	STEAP2	1.4	2.2E-08	1.3E-07	13.052	0.218	12.825	13.261	9.662	0.234	9.466	9.922
ENSG00000157734	SNX22	1.4	7.8E-05	2.4E-04	4.415	0.201	4.265	4.644	3.164	0.155	3.050	3.340
ENSG00000157985	AGAP1	1.4	1.6E-11	1.9E-10	17.099	0.591	16.478	17.656	12.021	1.116	10.775	12.927
ENSG00000158286	RNF207	1.4	6.3E-02	9.8E-02	0.550	0.079	0.492	0.641	0.385	0.121	0.256	0.497
ENSG00000158373	HIST1H2BD	1.4	4.9E-03	1.0E-02	15.653	1.638	14.692	17.544	11.409	1.659	9.685	12.994
ENSG00000158710	TAGLN2	1.4	8.5E-12	1.0E-10	91.915	4.772	86.416	94.964	64.004	3.918	59.509	66.701
ENSG00000159176	CSRP1	1.4	1.6E-11	1.9E-10	18.359	0.402	17.942	18.744	12.802	0.509	12.469	13.388
ENSG00000159214	CCDC24	1.4	3.1E-05	1.0E-04	9.145	0.830	8.404	10.042	6.757	0.275	6.525	7.060
ENSG00000160602	NEK8	1.4	1.7E-05	5.8E-05	7.350	0.413	7.054	7.822	5.379	0.328	5.167	5.757
ENSG00000160888	IER2	1.4	4.1E-11	4.4E-10	139.224	3.244	136.980	142.944	97.489	5.486	91.352	101.917
ENSG00000161914	ZNF653	1.4	1.1E-02	2.0E-02	2.770	0.170	2.606	2.946	2.006	0.241	1.777	2.257
ENSG00000162004	CCDC78	1.4	7.2E-02	1.1E-01	1.139	0.260	0.857	1.370	0.836	0.074	0.780	0.921
ENSG00000162604	TM2D1	1.4	4.8E-05	1.5E-04	7.663	0.755	6.828	8.298	5.677	0.539	5.093	6.154
ENSG00000162616	DNAJB4	1.4	3.7E-07	1.7E-06	17.296	0.443	17.026	17.807	12.303	1.020	11.391	13.404
ENSG00000162620	LRRIQ3	1.4	7.6E-02	1.2E-01	0.335	0.067	0.259	0.388	0.238	0.004	0.234	0.241
ENSG00000162687	KCNT2	1.4	2.0E-06	8.2E-06	8.960	0.501	8.413	9.396	6.611	0.758	5.762	7.221
ENSG00000163013	FBXO41	1.4	1.9E-08	1.2E-07	8.233	0.525	7.861	8.834	5.756	0.418	5.440	6.230
ENSG00000163322	FAM175A	1.4	3.1E-11	3.4E-10	24.406	0.783	23.567	25.116	17.119	1.006	16.035	18.024
ENSG00000163463	KRTCAP2	1.4	7.2E-04	1.8E-03	4.978	0.709	4.236	5.649	3.677	0.028	3.651	3.707
ENSG00000163874	ZC3H12A	1.4	8.5E-06	3.1E-05	9.620	0.371	9.248	9.990	6.660	0.358	6.423	7.072
ENSG00000163961	RNF168	1.4	1.2E-08	7.7E-08	50.768	1.577	49.434	52.508	37.246	1.570	36.265	39.056
ENSG00000164040	PGRMC2	1.4	1.9E-14	3.9E-13	79.658	2.286	77.341	81.913	55.577	2.172	53.531	57.856
ENSG00000164088	PPM1M	1.4	9.5E-04	2.3E-03	3.615	0.289	3.285	3.818	2.541	0.103	2.480	2.660
ENSG00000164118	CEP44	1.4	2.9E-08	1.7E-07	12.443	0.571	12.054	13.098	9.048	0.493	8.755	9.617
ENSG00000164291	ARSK	1.4	6.4E-07	2.9E-06	15.106	1.248	14.133	16.513	11.062	0.129	10.950	11.204
ENSG00000164588	HCN1	1.4	2.9E-04	7.6E-04	1.358	0.188	1.149	1.515	0.960	0.042	0.915	0.999
ENSG00000164603	BMT2	1.4	6.7E-06	2.5E-05	8.604	0.426	8.238	9.071	6.059	0.735	5.273	6.727
ENSG00000164631	ZNF12	1.4	2.3E-10	2.1E-09	32.274	1.075	31.120	33.248	23.852	0.682	23.143	24.525
ENSG00000164675	IQUB	1.4	8.6E-02	1.3E-01	0.603	0.176	0.434	0.785	0.420	0.123	0.284	0.525
ENSG00000164741	DLC1	1.4	1.5E-03	3.5E-03	1.296	0.090	1.193	1.351	0.951	0.060	0.884	1.000
ENSG00000165113	GKAP1	1.4	1.1E-04	3.2E-04	7.737	0.377	7.360	8.114	5.571	0.792	4.875	6.433
ENSG00000165115	KIF27	1.4	2.1E-05	6.9E-05	6.462	0.277	6.237	6.772	4.672	0.540	4.308	5.293
ENSG00000165152	TMEM246	1.4	1.4E-08	8.9E-08	22.223	1.017	21.068	22.980	16.496	0.646	15.981	17.221
ENSG00000165169	DYNLT3	1.4	6.5E-05	2.0E-04	10.642	0.565	10.240	11.288	7.801	0.977	6.928	8.857
ENSG00000165240	ATP7A	1.4	3.7E-05	1.2E-04	3.206	0.066	3.131	3.254	2.287	0.168	2.094	2.407
ENSG00000165591	FAAH2	1.4	3.9E-04	1.0E-03	8.471	0.802	7.645	9.246	6.055	0.299	5.741	6.337
ENSG00000166016	ABTB2	1.4	6.3E-05	1.9E-04	8.977	1.202	7.748	10.151	6.456	0.459	5.955	6.855
ENSG00000166136	NDUFB8	1.4	1.1E-06	4.6E-06	29.734	2.724	26.709	31.993	21.426	1.474	19.974	22.920
ENSG00000166439	RNF169	1.4	1.6E-08	9.7E-08	17.473	0.610	17.086	18.176	12.321	0.878	11.435	13.191
ENSG00000166478	ZNF143	1.4	4.0E-11	4.2E-10	35.844	1.547	34.281	37.375	25.210	0.810	24.418	26.037
ENSG00000166821	PEX11A	1.4	2.6E-03	5.5E-03	5.069	0.548	4.478	5.560	3.635	0.300	3.348	3.946
ENSG00000166949	SMAD3	1.4	1.1E-08	7.1E-08	11.488	0.463	11.015	11.941	8.391	0.493	7.971	8.933
ENSG00000167549	CORO6	1.4	1.8E-02	3.2E-02	1.223	0.025	1.202	1.250	0.846	0.100	0.744	0.944
ENSG00000167615	LENG8	1.4	3.2E-12	4.2E-11	40.636	2.228	38.295	42.731	28.804	2.219	27.467	31.366
ENSG00000167658	EEF2	1.4	9.0E-16	2.3E-14	2412.169	85.871	2316.019	2481.225	1737.198	86.628	1653.954	1826.855
ENSG00000167695	FAM57A	1.4	3.9E-07	1.8E-06	31.513	1.171	30.805	32.865	23.237	1.266	21.807	24.216
ENSG00000167740	CYB5D2	1.4	6.5E-04	1.6E-03	6.891	0.613	6.437	7.588	4.857	0.920	3.995	5.826
ENSG00000168214	RBPJ	1.4	8.1E-15	1.7E-13	106.750	2.333	104.824	109.344	77.264	3.556	73.261	80.058
ENSG00000168237	GLYCTK	1.4	2.0E-03	4.5E-03	4.676	0.655	4.256	5.430	3.265	0.708	2.566	3.983
ENSG00000168291	PDHB	1.4	5.9E-16	1.5E-14	137.531	2.748	134.470	139.788	95.641	3.488	91.669	98.209
ENSG00000168491	CCDC110	1.4	8.4E-03	1.6E-02	2.936	0.164	2.779	3.106	2.118	0.272	1.950	2.433
ENSG00000168748	CA7	1.4	4.2E-02	6.8E-02	2.723	0.247	2.441	2.901	1.986	0.307	1.661	2.270
ENSG00000168795	ZBTB5	1.4	5.5E-11	5.6E-10	27.057	1.156	25.995	28.288	19.007	0.214	18.778	19.203
ENSG00000168894	RNF181	1.4	7.3E-11	7.3E-10	118.772	3.344	115.032	121.473	82.714	4.681	77.630	86.847
ENSG00000169239	CA5B	1.4	1.7E-05	6.0E-05	4.342	0.094	4.240	4.426	3.220	0.224	3.056	3.475
ENSG00000169372	CRADD	1.4	5.6E-04	1.4E-03	4.283	0.404	3.824	4.584	3.087	0.323	2.842	3.452
ENSG00000169967	MAP3K2	1.4	4.4E-09	3.0E-08	12.616	0.747	11.789	13.245	9.332	0.676	8.709	10.051
ENSG00000169989	TIGD4	1.4	2.4E-03	5.3E-03	3.893	0.423	3.566	4.371	2.698	0.320	2.427	3.051
ENSG00000170004	CHD3	1.4	4.9E-09	3.4E-08	10.381	0.682	9.617	10.928	7.359	0.416	6.887	7.673
ENSG00000170175	CHRNB1	1.4	9.0E-06	3.3E-05	7.644	0.554	7.160	8.249	5.485	0.342	5.131	5.813
ENSG00000171126	KCNG3	1.4	3.6E-03	7.5E-03	3.706	0.492	3.141	4.036	2.749	0.262	2.594	3.052
ENSG00000171298	GAA	1.4	4.9E-08	2.7E-07	17.058	0.932	16.495	18.133	12.184	0.846	11.379	13.065
ENSG00000171720	HDAC3	1.4	6.0E-14	1.1E-12	79.484	0.429	79.109	79.951	55.791	1.300	54.533	57.130
ENSG00000171823	FBXL14	1.4	1.3E-04	3.7E-04	9.850	1.079	8.642	10.717	6.956	0.703	6.291	7.692
ENSG00000171988	JMJD1C	1.4	8.3E-15	1.8E-13	120.183	4.723	114.848	123.830	87.366	2.951	84.484	90.382
ENSG00000172020	GAP43	1.4	6.9E-08	3.8E-07	31.772	1.592	30.618	33.588	22.216	1.613	20.386	23.426
ENSG00000172175	MALT1	1.4	1.4E-09	1.1E-08	10.842	0.538	10.271	11.340	7.761	0.305	7.553	8.111
ENSG00000172375	C2CD2L	1.4	1.1E-02	2.0E-02	0.901	0.077	0.837	0.986	0.657	0.075	0.576	0.725
ENSG00000172456	FGGY	1.4	7.9E-05	2.4E-04	2.207	0.142	2.117	2.371	1.598	0.094	1.512	1.698
ENSG00000173039	RELA	1.4	1.1E-07	5.5E-07	11.242	0.946	10.357	12.239	7.830	0.740	7.107	8.585
ENSG00000173281	PPP1R3B	1.4	5.3E-10	4.4E-09	29.055	0.613	28.652	29.760	21.482	1.415	19.928	22.697
ENSG00000173531	MST1	1.4	1.6E-02	2.9E-02	1.542	0.380	1.120	1.857	1.116	0.253	0.968	1.408
ENSG00000173726	TOMM20	1.4	6.3E-15	1.4E-13	343.583	9.013	338.226	353.988	248.732	10.058	237.447	256.752
ENSG00000173812	EIF1	1.4	3.7E-15	8.4E-14	254.978	6.939	247.398	261.018	180.593	6.424	176.465	187.994
ENSG00000174292	TNK1	1.4	1.3E-04	3.7E-04	7.397	0.658	6.718	8.032	5.203	1.008	4.063	5.973
ENSG00000174720	LARP7	1.4	9.4E-12	1.1E-10	46.239	2.608	43.643	48.858	32.309	1.629	30.594	33.836
ENSG00000174799	CEP135	1.4	5.9E-09	4.0E-08	12.121	0.528	11.665	12.700	8.810	0.226	8.554	8.982
ENSG00000175061	LRRC75A-AS1	1.4	7.5E-14	1.4E-12	501.723	16.295	491.881	520.532	371.063	10.490	359.497	379.963
ENSG00000175182	FAM131A	1.4	8.6E-07	3.8E-06	6.978	0.672	6.213	7.475	4.958	0.284	4.694	5.259
ENSG00000175548	ALG10B	1.4	2.9E-07	1.4E-06	4.240	0.405	3.828	4.639	2.993	0.161	2.818	3.136
ENSG00000175573	C11orf68	1.4	3.3E-07	1.6E-06	36.462	2.901	33.912	39.617	25.608	1.957	24.035	27.799
ENSG00000175575	PAAF1	1.4	8.2E-08	4.4E-07	10.949	0.928	10.348	12.018	8.021	0.238	7.766	8.237
ENSG00000176049	JAKMIP2	1.4	1.8E-12	2.5E-11	20.107	0.505	19.753	20.686	14.136	0.287	13.805	14.323
ENSG00000176428	VPS37D	1.4	3.1E-03	6.5E-03	6.578	1.306	5.790	8.086	4.518	0.930	3.459	5.200
ENSG00000176912	TYMSOS	1.4	2.5E-03	5.5E-03	18.619	1.781	16.899	20.456	13.477	0.858	12.714	14.405
ENSG00000177000	MTHFR	1.4	2.1E-07	1.1E-06	6.709	0.118	6.598	6.833	4.964	0.283	4.652	5.207
ENSG00000177030	DEAF1	1.4	2.2E-08	1.3E-07	23.412	1.684	21.494	24.647	16.920	1.424	15.280	17.857
ENSG00000177042	TMEM80	1.4	3.8E-04	9.9E-04	4.925	0.612	4.474	5.622	3.591	0.228	3.363	3.819
ENSG00000177197	PCNPP5	1.4	1.6E-04	4.6E-04	44.801	2.180	42.352	46.529	31.508	3.976	26.932	34.123
ENSG00000177425	PAWR	1.4	6.2E-13	9.4E-12	108.045	4.915	103.267	113.087	77.681	2.978	74.340	80.056
ENSG00000178573	MAF	1.4	1.1E-04	3.1E-04	3.152	0.302	2.898	3.485	2.235	0.135	2.096	2.365
ENSG00000178860	MSC	1.4	3.3E-02	5.5E-02	3.244	0.255	2.996	3.506	2.382	0.509	2.049	2.969
ENSG00000178922	HYI	1.4	8.9E-04	2.1E-03	8.578	1.460	7.154	10.071	6.282	0.540	5.659	6.619
ENSG00000179104	TMTC2	1.4	5.8E-05	1.8E-04	3.425	0.077	3.338	3.485	2.520	0.270	2.299	2.821
ENSG00000179241	LDLRAD3	1.4	9.5E-10	7.5E-09	22.817	1.135	21.524	23.651	16.242	0.487	15.827	16.779
ENSG00000179262	RAD23A	1.4	3.9E-11	4.2E-10	66.281	4.371	61.310	69.528	45.981	3.286	42.323	48.682
ENSG00000179833	SERTAD2	1.4	9.2E-06	3.3E-05	6.530	0.401	6.235	6.987	4.625	0.209	4.386	4.773
ENSG00000180155	LYNX1	1.4	2.5E-04	6.7E-04	6.301	0.492	5.778	6.755	4.581	0.244	4.311	4.787
ENSG00000180190	TDRP	1.4	2.0E-12	2.8E-11	96.902	1.942	94.668	98.189	70.615	3.849	66.200	73.266
ENSG00000180739	S1PR5	1.4	5.5E-03	1.1E-02	4.101	0.880	3.135	4.859	2.882	0.504	2.489	3.450
ENSG00000180758	GPR157	1.4	1.6E-03	3.7E-03	3.815	0.500	3.362	4.351	2.802	0.185	2.615	2.984
ENSG00000180769	WDFY3-AS2	1.4	2.3E-02	4.0E-02	1.353	0.449	0.846	1.701	0.962	0.053	0.907	1.011
ENSG00000180773	SLC36A4	1.4	6.8E-11	6.8E-10	15.682	0.375	15.252	15.939	11.012	0.646	10.363	11.654
ENSG00000181264	TMEM136	1.4	6.8E-07	3.0E-06	14.806	0.158	14.713	14.989	10.983	0.434	10.599	11.454
ENSG00000181722	ZBTB20	1.4	1.4E-01	1.9E-01	0.101	0.047	0.053	0.147	0.074	0.018	0.062	0.094
ENSG00000181826	RELL1	1.4	3.0E-03	6.3E-03	5.886	0.274	5.623	6.169	4.131	0.706	3.322	4.622
ENSG00000181827	RFX7	1.4	4.6E-11	4.8E-10	25.159	1.198	23.776	25.878	18.562	0.888	17.541	19.227
ENSG00000182134	TDRKH	1.4	2.0E-09	1.5E-08	26.206	0.784	25.401	26.968	19.279	0.792	18.371	19.824
ENSG00000182287	AP1S2	1.4	1.9E-12	2.7E-11	195.931	9.091	187.177	205.325	141.081	10.754	130.645	152.127
ENSG00000182919	C11orf54	1.4	3.3E-07	1.6E-06	10.328	0.929	9.381	11.237	7.517	0.131	7.417	7.665
ENSG00000183011	NAA38	1.4	1.7E-06	7.2E-06	20.395	1.336	19.214	21.846	14.354	0.157	14.212	14.522
ENSG00000183067	IGSF5	1.4	6.7E-02	1.0E-01	2.496	0.716	2.021	3.320	1.850	0.576	1.273	2.425
ENSG00000183230	CTNNA3	1.4	2.8E-02	4.8E-02	0.487	0.022	0.466	0.511	0.350	0.075	0.269	0.417
ENSG00000183340	JRKL	1.4	7.9E-07	3.5E-06	15.897	1.004	15.210	17.049	11.396	0.561	10.758	11.813
ENSG00000183778	B3GALT5	1.4	8.4E-12	1.0E-10	23.814	0.769	22.928	24.304	17.557	0.446	17.228	18.065
ENSG00000183850	ZNF730	1.4	1.1E-06	4.8E-06	15.938	0.767	15.149	16.681	11.795	0.475	11.284	12.224
ENSG00000184009	ACTG1	1.4	6.3E-08	3.5E-07	3341.732	197.849	3122.713	3507.520	2411.208	121.616	2285.479	2528.246
ENSG00000184261	KCNK12	1.4	2.1E-03	4.7E-03	2.631	0.323	2.404	3.002	1.951	0.225	1.734	2.183
ENSG00000184619	KRBA2	1.4	9.0E-02	1.3E-01	1.520	0.235	1.326	1.781	1.129	0.286	0.830	1.400
ENSG00000184678	HIST2H2BE	1.4	3.9E-03	8.1E-03	5.631	0.731	5.171	6.474	4.124	0.208	3.884	4.257
ENSG00000184786	TCTE3	1.4	8.0E-05	2.4E-04	7.853	0.834	7.053	8.717	5.584	0.410	5.213	6.024
ENSG00000184809	B3GALT5-AS1	1.4	4.2E-07	1.9E-06	23.358	0.972	22.379	24.322	16.268	1.308	14.864	17.452
ENSG00000184903	IMMP2L	1.4	1.6E-05	5.6E-05	9.202	0.557	8.661	9.774	6.484	0.462	5.977	6.880
ENSG00000184916	JAG2	1.4	1.9E-03	4.3E-03	2.443	0.262	2.147	2.645	1.787	0.006	1.780	1.791
ENSG00000185065		1.4	7.1E-02	1.1E-01	1.923	0.770	1.104	2.632	1.345	0.236	1.073	1.496
ENSG00000185070	FLRT2	1.4	5.5E-04	1.4E-03	0.576	0.032	0.540	0.599	0.414	0.029	0.392	0.447
ENSG00000185420	SMYD3	1.4	2.9E-06	1.2E-05	10.582	0.522	10.228	11.183	7.849	0.447	7.334	8.136
ENSG00000185567	AHNAK2	1.4	1.6E-03	3.6E-03	0.741	0.021	0.718	0.757	0.545	0.034	0.521	0.584
ENSG00000185650	ZFP36L1	1.4	3.1E-07	1.5E-06	138.655	1.533	137.762	140.425	97.009	5.879	90.283	101.167
ENSG00000185834	RPL12P4	1.4	1.2E-15	2.9E-14	1797.321	40.706	1751.005	1827.415	1277.126	20.511	1261.415	1300.331
ENSG00000185842	DNAH14	1.4	9.8E-09	6.4E-08	4.268	0.326	3.940	4.592	2.976	0.218	2.849	3.227
ENSG00000186409	CCDC30	1.4	4.7E-02	7.6E-02	0.309	0.030	0.284	0.343	0.215	0.074	0.145	0.293
ENSG00000186665	C17orf58	1.4	6.3E-08	3.5E-07	37.831	2.499	34.957	39.489	28.120	0.636	27.751	28.855
ENSG00000186666	BCDIN3D	1.4	1.6E-03	9.4E-03	2.581	0.370	2.157	2.835	1.803	0.072	1.727	1.869
ENSG00000186868	MAPT	1.4	1.1E-02	2.1E-02	0.564	0.095	0.455	0.629	0.392	0.091	0.292	0.471
ENSG00000187323	DCC	1.4	1.2E-01	1.7E-01	0.386	0.111	0.258	0.456	0.285	0.034	0.246	0.308
ENSG00000187391	MAGI2	1.4	4.9E-08	2.7E-07	2.516	0.021	2.492	2.531	1.757	0.224	1.547	1.994
ENSG00000187953	PMS2CL	1.4	2.7E-05	8.7E-05	8.134	0.420	7.716	8.555	5.945	0.334	5.582	6.240
ENSG00000188026	RILPL1	1.4	2.4E-05	8.1E-05	2.818	0.101	2.706	2.902	1.950	0.150	1.809	2.108
ENSG00000188185	LINC00265	1.4	2.9E-05	9.4E-05	8.728	0.786	7.822	9.230	6.092	0.460	5.650	6.569
ENSG00000188219	POTEE	1.4	4.2E-03	8.7E-03	3.847	0.363	3.529	4.243	2.831	0.730	2.275	3.658
ENSG00000188647	PTAR1	1.4	5.9E-08	3.2E-07	40.962	2.115	38.650	42.802	29.627	1.686	28.424	31.567
ENSG00000188706	ZDHHC9	1.4	3.5E-11	3.7E-10	30.626	1.056	29.560	31.671	21.365	1.365	19.902	22.605
ENSG00000189190	ZNF600	1.4	2.2E-04	6.1E-04	5.071	0.600	4.398	5.548	3.640	0.284	3.390	3.949
ENSG00000196126	HLA-DRB1	1.4	3.7E-05	1.2E-04	20.214	1.493	18.943	21.858	14.174	1.244	12.760	15.100
ENSG00000196183	RPS2P4	1.4	5.2E-04	1.3E-03	29.415	2.349	27.738	32.100	21.677	2.579	18.980	24.118
ENSG00000196205	EEF1A1P5	1.4	5.4E-19	2.4E-17	17276.599	304.995	16926.823	17487.046	12062.109	227.543	11867.302	12312.201
ENSG00000196284	SUPT3H	1.4	1.7E-08	1.0E-07	23.180	0.183	23.015	23.377	16.579	0.256	16.357	16.859
ENSG00000196371	FUT4	1.4	1.0E-06	4.3E-06	8.512	0.257	8.305	8.800	6.257	0.298	5.971	6.567
ENSG00000196511	TPK1	1.4	2.0E-03	4.4E-03	1.346	0.060	1.307	1.415	0.970	0.011	0.960	0.983
ENSG00000196873	CBWD3	1.4	2.3E-07	1.1E-06	5.027	0.184	4.918	5.240	3.703	0.067	3.626	3.751
ENSG00000196878	LAMB3	1.4	1.2E-01	1.7E-01	0.727	0.256	0.554	1.021	0.521	0.188	0.360	0.727
ENSG00000196911	KPNA5	1.4	3.7E-07	1.7E-06	7.256	0.408	6.926	7.713	5.356	0.254	5.069	5.551
ENSG00000197381	ADARB1	1.4	1.2E-08	7.7E-08	5.931	0.299	5.660	6.251	4.123	0.057	4.057	4.156
ENSG00000197557	TTC30A	1.4	3.0E-05	9.7E-05	6.053	0.440	5.717	6.552	4.466	0.388	4.062	4.835
ENSG00000197566	ZNF624	1.4	3.9E-03	8.1E-03	2.336	0.237	2.137	2.598	1.702	0.299	1.447	2.031
ENSG00000197568	HHLA3	1.4	1.7E-04	4.8E-04	15.136	1.041	14.525	16.338	10.753	0.551	10.174	11.271
ENSG00000197694	SPTAN1	1.4	1.9E-13	3.3E-12	83.164	4.242	78.607	86.998	61.455	1.578	59.675	62.680
ENSG00000197847	SLC22A20	1.4	6.4E-04	1.6E-03	4.527	0.361	4.185	4.905	3.312	0.183	3.165	3.517
ENSG00000197978	GOLGA6L9	1.4	1.7E-06	7.0E-06	7.737	0.267	7.525	8.037	5.627	0.587	4.949	5.971
ENSG00000198001	IRAK4	1.4	2.0E-06	8.3E-06	7.995	0.161	7.856	8.171	5.655	0.623	4.966	6.179
ENSG00000198242	RPL23A	1.4	4.6E-14	8.7E-13	242.396	2.777	240.245	245.532	178.683	3.794	174.480	181.854
ENSG00000198270	TMEM116	1.4	2.9E-05	9.6E-05	3.791	0.277	3.577	4.104	2.781	0.076	2.728	2.868
ENSG00000198363	ASPH	1.4	5.9E-15	1.3E-13	24.771	0.679	23.999	25.271	17.515	0.444	17.060	17.947
ENSG00000198879	SFMBT2	1.4	3.8E-05	1.2E-04	4.658	0.609	4.001	5.202	3.440	0.415	2.961	3.699
ENSG00000198925	ATG9A	1.4	1.6E-10	1.5E-09	20.300	0.604	19.635	20.814	14.747	0.265	14.559	15.051
ENSG00000200534	SNORA33	1.4	4.5E-02	7.2E-02	42.178	8.768	32.057	47.447	30.322	7.266	23.077	37.608
ENSG00000203739		1.4	6.1E-02	9.6E-02	1.790	0.382	1.351	2.056	1.299	0.206	1.135	1.530
ENSG00000204060	FOXO6	1.4	1.4E-02	2.5E-02	2.776	0.459	2.253	3.114	1.933	0.310	1.706	2.287
ENSG00000204217	BMPR2	1.4	1.2E-09	9.1E-09	12.762	0.168	12.568	12.869	9.401	0.576	8.869	10.013
ENSG00000204815	TTC25	1.4	2.4E-02	4.1E-02	2.322	0.280	2.037	2.597	1.630	0.089	1.575	1.732
ENSG00000204860	FAM201A	1.4	1.2E-03	2.9E-03	3.246	0.156	3.089	3.400	2.306	0.337	2.083	2.694
ENSG00000205133	TRIQK	1.4	1.7E-09	1.3E-08	15.986	0.619	15.305	16.515	11.288	0.927	10.525	12.319
ENSG00000205189	ZBTB10	1.4	8.3E-09	5.5E-08	10.807	0.811	10.105	11.694	7.910	0.365	7.502	8.204
ENSG00000205707	ETFRF1	1.4	1.3E-04	3.8E-04	9.870	1.501	8.820	11.589	6.892	0.324	6.525	7.137
ENSG00000205710	C17orf107	1.4	2.3E-03	5.0E-03	4.618	0.490	4.214	5.163	3.378	0.305	3.135	3.721
ENSG00000205838	TTC23L	1.4	1.7E-02	3.0E-02	1.517	0.399	1.060	1.799	1.068	0.177	0.885	1.238
ENSG00000211751		1.4	3.5E-02	5.8E-02	6.242	0.275	5.944	6.486	4.481	0.575	4.131	5.144
ENSG00000213370	RANP6	1.4	3.7E-02	6.0E-02	12.217	4.473	7.057	15.014	9.112	0.937	8.258	10.114
ENSG00000213553	RPLP0P6	1.4	6.0E-15	1.3E-13	3344.590	148.869	3214.296	3506.843	2463.549	34.663	2431.679	2500.455
ENSG00000213801	ZNF321P	1.4	8.0E-03	1.5E-02	4.573	0.707	3.945	5.339	3.363	0.171	3.204	3.543
ENSG00000213846		1.4	6.5E-11	6.5E-10	51.744	0.214	51.580	51.986	36.229	0.919	35.654	37.288
ENSG00000213862		1.4	6.2E-10	5.1E-09	393.604	9.771	387.552	404.877	292.034	18.853	277.170	313.241
ENSG00000213958	KRT18P29	1.4	5.6E-03	1.1E-02	7.551	1.189	6.234	8.545	5.275	0.980	4.652	6.404
ENSG00000214012	KRT18P38	1.4	3.2E-02	5.4E-02	4.619	0.322	4.431	4.991	3.230	0.813	2.467	4.086
ENSG00000214016	RPSAP61	1.4	1.0E-05	3.7E-05	36.309	2.054	34.055	38.076	26.285	0.490	25.917	26.841
ENSG00000214413	BBIP1	1.4	6.9E-09	4.6E-08	8.247	0.298	7.920	8.505	5.732	0.259	5.519	6.020
ENSG00000214810	CYCSP55	1.4	5.4E-05	1.7E-04	82.671	6.428	75.588	88.133	58.180	4.560	53.421	62.509
ENSG00000215089	KRT18P11	1.4	3.9E-03	8.0E-03	10.175	0.648	9.772	10.922	7.485	0.636	6.840	8.113
ENSG00000215482	CALM2P3	1.4	7.0E-03	1.4E-02	27.993	1.951	26.752	30.242	20.769	2.171	19.444	23.274
ENSG00000218069	RSL24D1P1	1.4	3.2E-03	6.8E-03	27.577	5.223	21.603	31.279	20.074	1.225	18.701	21.055
ENSG00000219023		1.4	2.3E-07	1.1E-06	231.260	4.791	226.095	235.559	169.872	8.296	160.796	177.062
ENSG00000219133		1.4	6.3E-05	1.9E-04	40.716	0.554	40.242	41.326	28.192	2.442	25.815	30.695
ENSG00000219200	RNASEK	1.4	4.4E-02	7.1E-02	2.585	0.160	2.450	2.761	1.834	0.432	1.336	2.108
ENSG00000219545	UMAD1	1.4	1.9E-05	6.6E-05	10.353	0.666	9.832	11.104	7.557	0.827	6.724	8.377
ENSG00000219747		1.4	6.5E-05	2.0E-04	55.351	3.096	52.704	58.755	38.842	3.488	35.153	42.087
ENSG00000219932	RPL12P8	1.4	1.4E-04	4.0E-04	29.103	2.962	25.928	31.792	20.155	1.701	18.196	21.258
ENSG00000220472		1.4	3.4E-06	1.3E-05	89.180	4.691	84.161	93.455	65.726	7.354	57.565	71.837
ENSG00000220583	RPL35P2	1.4	1.4E-03	3.2E-03	54.221	4.321	49.264	57.190	40.252	3.488	36.300	42.948
ENSG00000220793		1.4	1.7E-11	1.9E-10	831.099	22.113	810.274	854.306	617.418	4.723	611.991	620.602
ENSG00000223802	CERS1	1.4	8.3E-03	1.6E-02	4.697	0.888	4.092	5.717	3.458	0.883	2.643	4.396
ENSG00000223804		1.4	9.2E-04	2.2E-03	12.098	1.578	10.779	13.845	8.359	1.057	7.496	9.538
ENSG00000225067	RPL23AP2	1.4	4.1E-12	5.3E-11	385.909	18.995	369.911	406.902	269.931	5.758	263.924	275.402
ENSG00000225138	SLC9A3-AS1	1.4	1.3E-01	1.8E-01	0.592	0.109	0.528	0.718	0.431	0.024	0.412	0.458
ENSG00000225356		1.4	2.5E-04	6.7E-04	72.692	6.696	65.001	77.230	52.881	4.806	47.483	56.697
ENSG00000225439	BOLA3-AS1	1.4	2.8E-05	9.1E-05	20.770	1.164	19.537	21.797	15.276	1.801	13.938	17.324
ENSG00000225675	LINC01771	1.4	1.2E-01	1.7E-01	5.801	1.128	4.580	6.806	4.251	0.574	3.607	4.711
ENSG00000225912		1.4	8.1E-04	2.0E-03	55.679	6.520	48.591	61.421	40.983	8.349	31.343	45.872
ENSG00000226067	LINC00623	1.4	8.1E-05	2.4E-04	13.870	0.844	12.910	14.494	10.054	1.089	8.877	11.025
ENSG00000226396		1.4	1.4E-01	2.0E-01	5.477	2.330	2.969	7.575	3.794	1.452	2.317	5.219
ENSG00000226882	CENPCP1	1.4	2.3E-02	4.0E-02	2.330	0.085	2.234	2.395	1.659	0.235	1.436	1.904
ENSG00000227001	NBPF2P	1.4	4.5E-02	7.2E-02	6.751	0.503	6.238	7.244	4.864	0.783	4.041	5.600
ENSG00000227008		1.4	6.7E-11	6.7E-10	767.309	41.361	719.551	791.631	558.933	33.781	522.404	589.046
ENSG00000227110	LMCD1-AS1	1.4	5.0E-02	8.0E-02	0.666	0.165	0.550	0.855	0.465	0.059	0.411	0.528
ENSG00000227347	HNRNPKP2	1.4	9.7E-07	4.2E-06	44.797	2.509	42.293	47.310	32.865	2.836	29.729	35.251
ENSG00000227486		1.4	1.1E-01	1.6E-01	3.972	1.190	3.066	5.319	2.907	1.004	1.760	3.630
ENSG00000227939	RPL3P2	1.4	1.8E-05	6.1E-05	26.311	3.694	22.191	29.328	18.235	2.369	15.870	20.607
ENSG00000228050	TOP3BP1	1.4	4.4E-02	7.2E-02	3.982	1.010	3.097	5.083	2.960	0.365	2.717	3.379
ENSG00000228399		1.4	9.6E-03	1.8E-02	34.866	8.359	27.360	43.875	25.208	2.453	23.569	28.028
ENSG00000228598	MACC1-AS1	1.4	5.4E-02	8.6E-02	6.090	0.783	5.334	6.898	4.343	0.305	4.153	4.695
ENSG00000228834		1.4	4.3E-02	7.0E-02	18.258	4.852	13.999	23.540	12.725	3.498	8.687	14.832
ENSG00000228903	RASA4CP	1.4	8.2E-02	1.2E-01	0.930	0.367	0.703	1.353	0.660	0.071	0.617	0.742
ENSG00000229119		1.4	2.2E-12	3.0E-11	1282.340	67.295	1236.466	1359.593	907.197	54.900	848.031	956.491
ENSG00000229133	RPS7P4	1.4	2.8E-02	4.7E-02	10.723	2.515	9.112	13.620	7.520	2.267	5.037	9.482
ENSG00000229546	LINC00428	1.4	6.0E-02	9.4E-02	17.747	3.891	13.586	21.297	12.475	3.530	8.410	14.769
ENSG00000229994	RPL5P4	1.4	1.4E-08	8.8E-08	98.235	4.448	93.289	101.906	70.509	2.800	67.597	73.183
ENSG00000230002	ALMS1-IT1	1.4	1.7E-02	3.0E-02	4.142	0.534	3.621	4.688	2.986	0.722	2.269	3.713
ENSG00000230076	RPL10P6	1.4	3.6E-04	9.5E-04	55.135	4.505	50.186	58.999	39.420	0.446	38.919	39.772
ENSG00000230882		1.4	7.3E-02	1.1E-01	1.095	0.174	0.936	1.281	0.769	0.305	0.563	1.119
ENSG00000231742	LINC01273	1.4	9.8E-02	1.4E-01	3.714	0.737	3.022	4.489	2.689	0.313	2.500	3.051
ENSG00000232134	RPS15AP12	1.4	1.0E-05	3.7E-05	99.032	5.203	93.919	104.320	71.775	2.920	68.404	73.528
ENSG00000232202		1.4	9.1E-02	1.3E-01	3.745	0.388	3.313	4.062	2.742	0.712	2.296	3.563
ENSG00000232486		1.4	4.9E-02	7.8E-02	2.555	0.457	2.034	2.885	1.888	0.528	1.289	2.292
ENSG00000232573	RPL3P4	1.4	3.7E-15	8.4E-14	4170.123	252.193	3948.544	4444.553	2936.046	110.410	2827.613	3048.333
ENSG00000233045		1.4	3.0E-03	6.4E-03	19.831	1.234	18.420	20.708	14.034	3.271	11.002	17.500
ENSG00000233476	EEF1A1P6	1.4	1.7E-17	5.9E-16	7821.810	197.996	7595.529	7963.232	5588.366	89.360	5486.071	5651.215
ENSG00000233503	HNRNPLP1	1.4	4.8E-02	7.7E-02	3.497	0.696	2.847	4.232	2.605	0.434	2.125	2.966
ENSG00000233558		1.4	1.1E-04	3.1E-04	51.669	1.976	50.293	53.933	37.675	7.019	31.727	45.418
ENSG00000233837	EIF3LP2	1.4	1.0E-02	1.9E-02	9.489	0.498	9.074	10.041	6.542	2.100	4.327	8.504
ENSG00000233967		1.4	6.7E-02	1.0E-01	3.777	0.574	3.194	4.340	2.672	0.108	2.556	2.771
ENSG00000234268		1.4	5.6E-06	2.1E-05	147.436	10.562	137.141	158.245	104.267	11.633	91.110	113.185
ENSG00000234648		1.4	1.5E-09	1.1E-08	3656.151	239.918	3456.285	3922.219	2705.103	236.938	2530.598	2974.841
ENSG00000234779		1.4	9.7E-02	1.4E-01	7.356	0.555	6.827	7.934	5.200	0.928	4.142	5.875
ENSG00000234782	TPT1P9	1.4	7.4E-04	1.8E-03	35.680	0.745	34.933	36.424	25.592	1.173	24.731	26.928
ENSG00000234911	TEX21P	1.4	1.4E-01	1.9E-01	1.957	0.141	1.870	2.119	1.405	0.624	1.041	2.125
ENSG00000235034	C19orf81	1.4	1.0E-01	1.5E-01	3.259	0.366	2.925	3.650	2.289	0.443	1.888	2.764
ENSG00000235508	RPS2P7	1.4	1.6E-06	6.6E-06	70.284	4.009	67.112	74.790	51.866	4.611	47.088	56.289
ENSG00000236204	LINC01376	1.4	1.1E-01	1.6E-01	1.045	0.057	0.990	1.104	0.751	0.149	0.598	0.896
ENSG00000236432		1.4	3.6E-02	6.0E-02	1.429	0.165	1.245	1.564	1.012	0.137	0.885	1.158
ENSG00000236438	FAM157A	1.4	9.6E-05	2.8E-04	2.762	0.342	2.526	3.154	1.909	0.064	1.835	1.955
ENSG00000236682		1.4	6.0E-02	9.4E-02	1.708	0.314	1.434	2.052	1.205	0.189	1.039	1.411
ENSG00000237214		1.4	8.9E-07	3.9E-06	76.883	3.149	73.414	79.561	54.728	4.335	49.724	57.331
ENSG00000237804	HNRNPA1P39	1.4	3.9E-02	6.4E-02	4.687	0.824	3.737	5.211	3.362	0.332	3.029	3.693
ENSG00000239797	RPL21P39	1.4	5.1E-05	1.6E-04	64.726	2.461	63.213	67.566	47.749	1.039	46.612	48.650
ENSG00000240036		1.4	2.5E-08	1.5E-07	219.605	3.669	215.738	223.038	159.697	9.030	152.568	169.852
ENSG00000240048	DDX50P2	1.4	3.9E-02	6.4E-02	2.076	0.392	1.653	2.428	1.433	0.216	1.274	1.679
ENSG00000241978	AKAP2	1.4	4.7E-02	7.5E-02	0.705	0.122	0.569	0.805	0.511	0.126	0.376	0.624
ENSG00000242622		1.4	4.1E-03	8.4E-03	5.882	0.934	5.055	6.894	4.114	0.754	3.311	4.806
ENSG00000242756	RHOT1P3	1.4	1.7E-02	3.1E-02	19.700	3.132	17.816	23.316	13.895	1.435	12.589	15.431
ENSG00000243181		1.4	2.7E-04	7.1E-04	33.485	1.031	32.573	34.603	23.322	1.157	22.450	24.635
ENSG00000243802		1.4	1.0E-01	1.5E-01	9.047	2.280	7.206	11.597	6.569	1.901	4.870	8.622
ENSG00000244627		1.4	4.6E-05	1.4E-04	9.470	0.701	8.726	10.117	6.809	1.261	5.799	8.222
ENSG00000245213		1.4	6.3E-03	1.2E-02	2.470	0.540	2.063	3.083	1.744	0.138	1.659	1.903
ENSG00000246090		1.4	1.2E-04	3.3E-04	3.580	0.267	3.382	3.884	2.637	0.153	2.467	2.763
ENSG00000246792		1.4	2.9E-02	5.0E-02	1.867	0.269	1.568	2.089	1.337	0.052	1.292	1.394
ENSG00000247556	OIP5-AS1	1.4	4.0E-07	1.9E-06	88.214	1.639	86.749	89.985	62.640	1.574	60.832	63.706
ENSG00000248485	PCP4L1	1.4	8.0E-02	1.2E-01	2.738	0.245	2.508	2.996	2.014	0.387	1.570	2.271
ENSG00000249152	LNCPRESS2	1.4	5.7E-10	4.7E-09	272.120	19.243	251.057	288.781	193.816	11.760	182.807	206.206
ENSG00000249863		1.4	2.1E-02	3.7E-02	6.471	0.736	5.644	7.057	4.673	0.762	4.119	5.542
ENSG00000250317	SMIM20	1.4	4.0E-10	3.4E-09	65.607	5.010	62.714	71.392	45.416	0.744	44.916	46.272
ENSG00000250366	TUNAR	1.4	4.7E-13	7.2E-12	70.412	3.603	68.027	74.556	48.727	0.526	48.186	49.236
ENSG00000250568		1.4	1.9E-06	7.9E-06	145.891	6.925	137.934	150.555	104.756	11.501	92.682	115.583
ENSG00000250575		1.4	4.3E-03	8.8E-03	7.332	0.646	6.586	7.710	5.218	0.501	4.639	5.515
ENSG00000251141		1.4	2.5E-02	4.3E-02	2.949	0.520	2.349	3.281	2.176	0.234	1.908	2.338
ENSG00000253144		1.4	2.2E-05	7.3E-05	47.645	1.735	46.174	49.558	33.045	4.529	28.073	36.937
ENSG00000254373		1.4	9.3E-04	2.2E-03	48.051	8.133	40.702	56.789	33.472	5.754	29.893	40.109
ENSG00000254860	TMEM9B-AS1	1.4	2.1E-02	3.7E-02	5.083	0.190	4.864	5.203	3.599	0.714	2.783	4.107
ENSG00000256008		1.4	3.7E-02	6.1E-02	4.634	0.409	4.352	5.104	3.313	0.426	2.925	3.769
ENSG00000256124	LINC01152	1.4	8.6E-02	1.3E-01	0.936	0.214	0.691	1.090	0.680	0.136	0.577	0.834
ENSG00000256338	RPL41P2	1.4	3.0E-06	1.2E-05	151.810	9.959	145.730	163.304	112.209	9.355	102.008	120.387
ENSG00000257261		1.4	1.0E-02	1.9E-02	4.100	0.580	3.604	4.738	2.861	0.685	2.273	3.613
ENSG00000258199		1.4	1.4E-02	2.5E-02	18.230	2.868	15.703	21.347	12.893	3.031	9.474	15.251
ENSG00000259022	DNAJC8P1	1.4	5.0E-02	8.0E-02	10.474	1.468	9.128	12.040	7.265	2.572	4.346	9.200
ENSG00000259948		1.4	1.6E-03	3.6E-03	23.910	2.837	22.020	27.173	17.187	1.899	15.587	19.285
ENSG00000260257		1.4	4.9E-02	7.9E-02	2.775	1.232	1.600	4.056	1.956	0.252	1.770	2.243
ENSG00000260267		1.4	8.4E-03	1.6E-02	3.209	0.138	3.111	3.366	2.311	0.399	2.044	2.770
ENSG00000260521		1.4	4.7E-02	7.6E-02	4.198	0.573	3.642	4.786	3.111	0.526	2.666	3.691
ENSG00000260528	FAM157C	1.4	7.4E-03	1.4E-02	1.935	0.069	1.895	2.015	1.413	0.132	1.277	1.541
ENSG00000260641		1.4	1.3E-01	1.9E-01	0.560	0.152	0.402	0.705	0.418	0.027	0.396	0.448
ENSG00000260645		1.4	7.0E-02	1.1E-01	2.348	0.677	1.745	3.081	1.744	0.086	1.645	1.798
ENSG00000260751		1.4	8.9E-02	1.3E-01	3.424	1.101	2.412	4.597	2.436	0.583	1.910	3.063
ENSG00000260766		1.4	3.6E-02	6.0E-02	3.409	0.326	3.184	3.782	2.512	0.210	2.275	2.671
ENSG00000260806		1.4	1.3E-01	1.8E-01	2.297	0.691	1.503	2.756	1.712	0.255	1.478	1.984
ENSG00000261136		1.4	1.3E-02	2.3E-02	2.462	0.348	2.077	2.753	1.717	0.447	1.238	2.123
ENSG00000261326	LINC01355	1.4	8.6E-04	2.1E-03	4.238	0.436	3.735	4.508	3.123	0.477	2.705	3.644
ENSG00000261559	FSCN1P1	1.4	1.2E-02	2.2E-02	7.295	1.170	5.955	8.113	5.393	0.658	4.763	6.076
ENSG00000262227		1.4	1.0E-01	1.5E-01	4.724	1.040	3.871	5.883	3.283	0.710	2.475	3.803
ENSG00000263513	FAM72C	1.4	1.8E-06	7.4E-06	22.795	0.881	21.835	23.566	16.584	0.823	15.655	17.189
ENSG00000264281		1.4	1.9E-11	2.2E-10	3282.852	114.092	3156.820	3379.092	2387.123	144.367	2266.871	2547.231
ENSG00000264575	LINC00526	1.4	6.9E-03	1.4E-02	5.502	0.647	5.005	6.233	3.970	0.507	3.502	4.509
ENSG00000267058		1.4	5.4E-02	8.6E-02	2.800	0.189	2.604	2.982	2.053	0.720	1.368	2.803
ENSG00000267317		1.4	8.4E-02	1.3E-01	3.656	0.658	2.965	4.276	2.640	0.196	2.453	2.844
ENSG00000268573		1.4	2.8E-03	6.1E-03	2.750	0.490	2.408	3.311	1.935	0.276	1.649	2.200
ENSG00000268575		1.4	2.4E-02	4.1E-02	1.819	0.313	1.582	2.173	1.338	0.092	1.246	1.430
ENSG00000268942	CKS1BP3	1.4	4.3E-10	3.6E-09	487.003	3.602	483.424	490.627	346.643	19.767	328.639	367.795
ENSG00000269044		1.4	8.7E-03	1.7E-02	5.842	0.340	5.502	6.182	4.218	0.787	3.333	4.836
ENSG00000269190	FBXO17	1.4	4.5E-06	1.7E-05	7.732	0.084	7.655	7.821	5.432	0.400	5.013	5.811
ENSG00000269940		1.4	5.3E-03	1.1E-02	19.605	3.229	16.755	23.113	14.056	1.155	13.116	15.318
ENSG00000269981		1.4	1.4E-02	2.6E-02	26.980	4.404	22.968	31.692	19.276	2.025	17.082	21.074
ENSG00000270012		1.4	1.3E-02	2.4E-02	2.416	0.032	2.388	2.451	1.704	0.384	1.346	2.109
ENSG00000270605		1.4	1.4E-02	2.6E-02	5.125	1.509	3.412	6.259	3.737	0.517	3.232	4.265
ENSG00000270714		1.4	1.3E-01	1.8E-01	14.452	6.767	8.693	21.905	10.411	0.646	9.760	11.051
ENSG00000271918		1.4	1.1E-01	1.6E-01	2.053	0.634	1.613	2.780	1.494	0.476	1.048	1.996
ENSG00000272316		1.4	3.7E-03	7.8E-03	2.751	0.202	2.597	2.980	2.017	0.358	1.620	2.317
ENSG00000272525		1.4	1.4E-01	2.0E-01	3.590	0.710	3.054	4.396	2.667	0.912	1.878	3.666
ENSG00000273014		1.4	4.6E-02	7.3E-02	12.340	4.273	8.134	16.678	8.846	2.056	6.934	11.021
ENSG00000273117		1.4	2.7E-04	7.1E-04	14.226	0.347	13.833	14.493	10.450	1.484	8.744	11.440
ENSG00000273173	SNURF	1.4	6.4E-02	9.9E-02	3.762	0.959	2.662	4.421	2.719	0.153	2.553	2.853
ENSG00000273759		1.4	3.6E-02	5.9E-02	9.155	1.416	7.524	10.070	6.766	0.913	5.815	7.634
ENSG00000274220		1.4	7.3E-02	1.1E-01	1.598	0.180	1.390	1.714	1.177	0.124	1.067	1.311
ENSG00000274712		1.4	2.1E-03	4.6E-03	6.458	1.118	5.638	7.731	4.541	0.692	4.111	5.339
ENSG00000275183	LENG9	1.4	2.6E-02	4.4E-02	3.682	0.711	3.050	4.453	2.704	0.637	1.968	3.082
ENSG00000275234		1.4	6.2E-04	1.5E-03	25.828	0.987	24.902	26.867	18.636	1.293	17.258	19.822
ENSG00000276023	DUSP14	1.4	1.3E-07	6.8E-07	29.248	2.059	26.891	30.697	21.195	0.587	20.517	21.547
ENSG00000277383		1.4	8.1E-02	1.2E-01	5.835	0.465	5.299	6.108	4.211	0.432	3.840	4.686
ENSG00000278238		1.4	9.6E-02	1.4E-01	7.046	2.513	4.595	9.617	4.970	1.466	3.786	6.610
ENSG00000278540	ACACA	1.4	1.1E-14	2.4E-13	123.970	2.266	121.422	125.758	91.265	3.103	87.767	93.685
ENSG00000278600		1.4	2.9E-03	6.2E-03	6.844	1.117	5.598	7.756	4.922	0.471	4.392	5.296
ENSG00000279050	PWAR1	1.4	8.0E-02	1.2E-01	1.234	0.206	1.009	1.414	0.881	0.212	0.637	1.017
ENSG00000279095		1.4	2.0E-02	3.6E-02	2.968	0.377	2.594	3.349	2.101	0.105	2.017	2.218
ENSG00000279145		1.4	8.0E-03	1.5E-02	1.546	0.319	1.311	1.910	1.073	0.183	0.870	1.224
ENSG00000279673		1.4	1.0E-03	2.4E-03	12.433	1.019	11.755	13.605	8.704	0.862	7.709	9.230
ENSG00000280056		1.4	1.0E-02	2.0E-02	80.218	17.802	59.735	91.960	58.245	9.875	50.295	69.300
ENSG00000280142		1.4	7.9E-04	1.9E-03	28.398	4.093	23.824	31.718	20.353	2.873	17.083	22.479
ENSG00000280279		1.4	5.5E-02	8.6E-02	2.204	0.148	2.083	2.370	1.624	0.244	1.437	1.900
ENSG00000280852		1.4	8.2E-12	1.0E-10	175.114	4.608	170.036	179.029	122.137	2.688	120.016	125.173
ENSG00000281571		1.4	7.4E-02	1.1E-01	3.204	0.776	2.346	3.857	2.192	0.570	1.592	2.726
ENSG00000282048		1.4	2.1E-02	3.7E-02	27.940	2.874	25.567	31.135	19.849	3.537	15.766	21.961
ENSG00000282826	FRG1CP	1.4	1.1E-04	3.1E-04	34.643	3.812	30.242	36.872	25.084	2.394	22.656	27.441
ENSG00000002746	HECW1	1.5	1.1E-05	3.9E-05	2.374	0.281	2.089	2.650	1.625	0.218	1.440	1.865
ENSG00000004455	AK2	1.5	3.1E-17	9.9E-16	74.352	1.678	72.957	76.214	48.790	1.554	47.051	50.043
ENSG00000008277	ADAM22	1.5	3.2E-13	5.1E-12	12.477	0.315	12.121	12.720	8.299	0.460	7.780	8.655
ENSG00000010404	IDS	1.5	1.0E-10	9.7E-10	13.735	1.165	12.599	14.927	9.322	0.447	8.806	9.591
ENSG00000012660	ELOVL5	1.5	2.3E-18	9.0E-17	283.384	6.178	277.124	289.476	191.924	2.450	189.621	194.499
ENSG00000013297	CLDN11	1.5	1.2E-05	4.3E-05	3.255	0.151	3.095	3.394	2.133	0.044	2.084	2.170
ENSG00000013441	CLK1	1.5	2.4E-13	3.9E-12	40.412	0.920	39.856	41.475	26.359	1.627	24.925	28.127
ENSG00000014123	UFL1	1.5	3.2E-12	4.2E-11	33.571	0.944	32.917	34.652	22.926	0.910	22.333	23.974
ENSG00000018408	WWTR1	1.5	4.6E-10	3.9E-09	13.700	0.394	13.351	14.127	9.370	0.125	9.265	9.509
ENSG00000021645	NRXN3	1.5	2.7E-03	5.8E-03	0.597	0.055	0.533	0.632	0.406	0.076	0.325	0.477
ENSG00000022355	GABRA1	1.5	1.0E-02	1.9E-02	0.844	0.198	0.623	1.006	0.546	0.178	0.342	0.669
ENSG00000033178	UBA6	1.5	3.7E-15	8.4E-14	39.749	1.374	38.250	40.949	26.393	0.804	25.526	27.114
ENSG00000034693	PEX3	1.5	1.6E-09	1.2E-08	38.613	2.856	36.027	41.678	25.916	0.287	25.591	26.132
ENSG00000044090	CUL7	1.5	4.2E-10	3.5E-09	19.093	1.216	17.888	20.362	13.076	0.324	12.790	13.428
ENSG00000048740	CELF2	1.5	2.8E-09	2.0E-08	13.562	0.983	12.682	14.623	9.362	0.637	8.670	9.925
ENSG00000054148	PHPT1	1.5	2.8E-11	3.1E-10	83.038	4.971	77.635	87.420	57.047	1.900	54.853	58.173
ENSG00000054690	PLEKHH1	1.5	2.6E-13	4.2E-12	15.974	0.661	15.222	16.462	10.771	0.290	10.583	11.106
ENSG00000063180	CA11	1.5	4.9E-11	5.1E-10	67.061	3.919	63.153	70.991	44.150	0.681	43.636	44.922
ENSG00000067057	PFKP	1.5	2.7E-16	7.6E-15	101.628	1.765	99.613	102.899	68.007	3.553	65.078	71.960
ENSG00000068976	PYGM	1.5	2.2E-05	7.4E-05	5.962	1.029	5.110	7.104	4.038	0.418	3.585	4.408
ENSG00000069869	NEDD4	1.5	1.0E-07	5.4E-07	7.819	1.050	6.889	8.957	5.336	0.221	5.083	5.486
ENSG00000070669	ASNS	1.5	4.4E-17	1.4E-15	237.676	3.640	233.930	241.201	156.601	2.604	154.376	159.464
ENSG00000072571	HMMR	1.5	1.3E-08	8.1E-08	144.547	9.467	134.114	152.591	97.863	3.798	95.550	102.246
ENSG00000075420	FNDC3B	1.5	6.8E-13	1.0E-11	16.855	0.673	16.133	17.465	11.519	0.498	11.074	12.057
ENSG00000077684	JADE1	1.5	4.2E-18	1.6E-16	127.524	4.840	122.368	131.970	83.925	1.494	82.300	84.811
ENSG00000081760	AACS	1.5	2.9E-15	6.9E-14	20.036	0.599	19.459	20.656	13.115	0.579	12.620	13.751
ENSG00000085117	CD82	1.5	5.7E-05	1.8E-04	3.899	0.318	3.604	4.236	2.605	0.219	2.388	2.825
ENSG00000090339	ICAM1	1.5	3.6E-04	9.4E-04	3.598	0.408	3.154	3.957	2.449	0.259	2.224	2.731
ENSG00000095713	CRTAC1	1.5	3.4E-02	5.6E-02	1.092	0.467	0.594	1.520	0.716	0.050	0.668	0.768
ENSG00000095739	BAMBI	1.5	8.7E-11	8.5E-10	39.937	0.648	39.554	40.685	26.803	0.870	25.837	27.523
ENSG00000099991	CABIN1	1.5	2.2E-13	3.7E-12	38.420	0.952	37.589	39.459	26.215	1.191	25.012	27.394
ENSG00000100097	LGALS1	1.5	4.1E-08	2.3E-07	53.667	6.509	46.317	58.706	36.285	1.781	35.180	38.340
ENSG00000100504	PYGL	1.5	3.1E-15	7.1E-14	58.650	1.900	56.599	60.350	39.179	1.228	37.771	40.032
ENSG00000100577	GSTZ1	1.5	1.4E-08	8.8E-08	9.945	0.773	9.427	10.834	6.879	0.570	6.467	7.530
ENSG00000100918	REC8	1.5	5.1E-08	2.8E-07	17.624	0.561	16.978	17.989	11.763	1.226	10.570	13.019
ENSG00000100968	NFATC4	1.5	2.0E-10	1.8E-09	6.801	0.193	6.623	7.007	4.465	0.024	4.449	4.493
ENSG00000101928	MOSPD1	1.5	2.3E-11	2.5E-10	38.027	1.348	37.064	39.563	24.958	0.689	24.163	25.379
ENSG00000101977	MCF2	1.5	1.8E-03	4.0E-03	2.021	0.231	1.793	2.255	1.317	0.300	1.035	1.632
ENSG00000102007	PLP2	1.5	2.5E-03	5.4E-03	7.139	0.599	6.648	7.488	4.813	1.302	3.386	5.936
ENSG00000102606	ARHGEF7	1.5	1.1E-15	2.8E-14	21.089	0.361	20.789	21.490	13.773	0.168	13.579	13.882
ENSG00000102908	NFAT5	1.5	8.2E-10	6.6E-09	14.637	0.604	14.173	15.320	9.970	0.275	9.661	10.186
ENSG00000103018	CYB5B	1.5	2.5E-17	8.3E-16	77.461	2.154	76.045	79.940	50.900	1.233	50.124	52.323
ENSG00000103365	GGA2	1.5	7.1E-17	2.1E-15	67.732	0.770	66.991	68.528	46.188	0.676	45.488	46.835
ENSG00000103507	BCKDK	1.5	5.1E-13	7.8E-12	44.850	1.021	43.758	45.780	29.561	2.085	27.519	31.688
ENSG00000103599	IQCH	1.5	1.7E-02	3.1E-02	0.626	0.097	0.540	0.731	0.411	0.076	0.326	0.472
ENSG00000104814	MAP4K1	1.5	1.5E-04	4.4E-04	4.567	0.155	4.412	4.722	3.094	0.670	2.544	3.841
ENSG00000105122	RASAL3	1.5	2.3E-03	5.0E-03	1.792	0.212	1.550	1.942	1.197	0.259	0.925	1.442
ENSG00000106341	PPP1R17	1.5	7.6E-06	2.8E-05	10.768	0.075	10.683	10.826	7.200	0.363	6.782	7.436
ENSG00000106392	C1GALT1	1.5	6.9E-15	1.5E-13	63.919	2.815	61.047	66.675	43.178	2.861	39.887	45.065
ENSG00000106479	ZNF862	1.5	8.9E-04	2.1E-03	1.525	0.112	1.400	1.617	1.033	0.160	0.928	1.217
ENSG00000106638	TBL2	1.5	1.1E-10	1.0E-09	16.627	0.506	16.332	17.211	10.993	0.821	10.311	11.903
ENSG00000107771	CCSER2	1.5	1.4E-13	2.5E-12	19.120	0.187	18.960	19.326	12.407	0.232	12.188	12.651
ENSG00000108039	XPNPEP1	1.5	3.9E-15	8.9E-14	40.285	0.798	39.400	40.950	27.127	1.003	26.180	28.177
ENSG00000108379	WNT3	1.5	5.7E-05	1.8E-04	3.383	0.117	3.248	3.455	2.187	0.204	2.063	2.423
ENSG00000108684	ASIC2	1.5	1.1E-02	2.1E-02	1.118	0.071	1.048	1.189	0.744	0.126	0.599	0.829
ENSG00000109919	MTCH2	1.5	4.6E-16	1.2E-14	144.593	6.604	137.083	149.488	95.987	1.647	94.941	97.886
ENSG00000111254	AKAP3	1.5	9.2E-04	2.2E-03	2.368	0.296	2.132	2.699	1.526	0.102	1.409	1.599
ENSG00000111912	NCOA7	1.5	2.0E-08	1.2E-07	7.005	0.392	6.618	7.402	4.639	0.281	4.315	4.823
ENSG00000112182	BACH2	1.5	1.7E-06	6.9E-06	4.084	0.139	3.938	4.213	2.793	0.212	2.669	3.038
ENSG00000113231	PDE8B	1.5	1.6E-02	3.0E-02	0.798	0.062	0.748	0.867	0.547	0.053	0.511	0.607
ENSG00000113240	CLK4	1.5	9.5E-09	6.2E-08	9.730	0.859	8.921	10.631	6.565	0.706	5.868	7.281
ENSG00000115257	PCSK4	1.5	4.8E-03	9.7E-03	2.068	0.410	1.628	2.440	1.389	0.242	1.179	1.654
ENSG00000115828	QPCT	1.5	1.0E-04	3.0E-04	6.355	0.501	5.799	6.770	4.220	0.082	4.139	4.302
ENSG00000116133	DHCR24	1.5	5.6E-19	2.4E-17	681.086	8.477	674.958	690.761	449.891	8.437	441.711	458.562
ENSG00000117151	CTBS	1.5	9.0E-05	2.7E-04	2.858	0.235	2.625	3.094	1.951	0.316	1.592	2.188
ENSG00000117335	CD46	1.5	6.2E-17	1.9E-15	107.296	6.150	100.326	111.963	70.080	3.027	66.887	72.908
ENSG00000117598	PLPPR5	1.5	8.7E-06	3.1E-05	5.658	0.824	5.007	6.585	3.695	0.315	3.353	3.973
ENSG00000117616	RSRP1	1.5	3.6E-11	3.8E-10	23.679	1.705	21.710	24.668	15.832	1.424	14.440	17.287
ENSG00000118322	ATP10B	1.5	1.0E-02	1.9E-02	0.565	0.069	0.491	0.629	0.385	0.062	0.348	0.457
ENSG00000119686	FLVCR2	1.5	6.6E-05	2.0E-04	4.398	0.941	3.357	5.187	2.884	0.310	2.587	3.205
ENSG00000119899	SLC17A5	1.5	1.9E-08	1.1E-07	24.954	0.742	24.303	25.762	16.902	0.666	16.156	17.438
ENSG00000120049	KCNIP2	1.5	2.1E-03	4.7E-03	2.896	0.325	2.530	3.151	1.954	0.121	1.866	2.091
ENSG00000120860	WASHC3	1.5	6.9E-05	2.1E-04	6.866	0.107	6.745	6.948	4.735	0.468	4.207	5.097
ENSG00000120910	PPP3CC	1.5	3.3E-04	8.7E-04	5.010	0.122	4.927	5.150	3.420	0.341	3.057	3.734
ENSG00000121851	POLR3GL	1.5	1.1E-03	2.6E-03	6.649	0.499	6.244	7.207	4.428	0.323	4.093	4.737
ENSG00000122367	LDB3	1.5	1.0E-01	1.5E-01	0.387	0.156	0.256	0.559	0.250	0.153	0.126	0.421
ENSG00000122477	LRRC39	1.5	1.4E-01	1.9E-01	0.964	0.296	0.652	1.240	0.621	0.354	0.274	0.982
ENSG00000123096	SSPN	1.5	3.9E-04	1.0E-03	1.708	0.197	1.493	1.880	1.119	0.113	0.989	1.199

ENSG00000123983	ACSL3	1.5	1.0E-17	3.6E-16	131.126	3.084	128.233	134.371	86.637	4.169	83.324	91.318
ENSG00000124257	NEURL2	1.5	6.4E-03	1.3E-02	5.223	1.203	4.153	6.525	3.454	0.276	3.144	3.674
ENSG00000125352	RNF113A	1.5	1.1E-06	4.8E-06	28.015	1.441	26.352	28.900	19.210	1.724	17.655	21.064
ENSG00000126106	TMEM53	1.5	7.3E-05	2.2E-04	5.633	0.817	4.798	6.430	3.744	0.282	3.459	4.022
ENSG00000126773	PCNX4	1.5	4.2E-14	8.0E-13	11.272	0.059	11.223	11.337	7.675	0.192	7.520	7.890
ENSG00000127252	HRASLS	1.5	2.5E-03	5.4E-03	8.300	0.872	7.298	8.887	5.498	0.702	4.780	6.182
ENSG00000127507	ADGRE2	1.5	2.7E-05	8.9E-05	2.524	0.134	2.391	2.658	1.641	0.257	1.377	1.890
ENSG00000127526	SLC35E1	1.5	5.1E-11	5.3E-10	17.554	0.686	16.825	18.185	11.848	0.258	11.645	12.139
ENSG00000127561	SYNGR3	1.5	1.6E-08	1.0E-07	18.707	0.305	18.368	18.958	12.837	0.900	11.886	13.676
ENSG00000127957	PMS2P3	1.5	1.2E-03	2.8E-03	6.963	0.505	6.437	7.444	4.804	0.993	3.878	5.854
ENSG00000130517	PGPEP1	1.5	6.5E-12	8.0E-11	15.264	0.852	14.351	16.038	9.883	0.535	9.399	10.451
ENSG00000130720	FIBCD1	1.5	1.5E-03	3.4E-03	2.431	0.376	2.004	2.712	1.603	0.343	1.349	1.993
ENSG00000130724	CHMP2A	1.5	6.4E-15	1.4E-13	86.706	0.975	85.619	87.505	56.130	1.803	54.064	57.386
ENSG00000131095	GFAP	1.5	4.0E-02	6.5E-02	0.300	0.075	0.235	0.382	0.196	0.082	0.146	0.290
ENSG00000131188	PRR7	1.5	9.9E-05	2.9E-04	11.112	1.237	10.191	12.518	7.547	0.806	6.622	8.093
ENSG00000132004	FBXW9	1.5	4.0E-07	1.9E-06	19.264	1.813	17.891	21.320	12.671	1.703	10.705	13.675
ENSG00000132199	ENOSF1	1.5	1.7E-09	1.2E-08	7.647	0.148	7.477	7.749	5.282	0.273	4.993	5.536
ENSG00000132205	EMILIN2	1.5	1.2E-05	4.1E-05	3.960	0.245	3.694	4.176	2.679	0.398	2.301	3.094
ENSG00000132424	PNISR	1.5	3.1E-11	3.4E-10	90.850	8.545	81.732	98.676	60.509	1.888	59.074	62.648
ENSG00000132470	ITGB4	1.5	4.0E-05	1.3E-04	2.651	0.280	2.329	2.839	1.778	0.225	1.632	2.037
ENSG00000133019	CHRM3	1.5	1.7E-08	1.1E-07	3.875	0.260	3.574	4.031	2.566	0.056	2.502	2.605
ENSG00000134013	LOXL2	1.5	3.6E-14	6.9E-13	35.366	0.796	34.754	36.264	23.892	0.833	22.959	24.561
ENSG00000134668	SPOCD1	1.5	7.5E-03	1.5E-02	0.958	0.118	0.850	1.084	0.640	0.179	0.434	0.754
ENSG00000135423	GLS2	1.5	3.1E-08	1.8E-07	10.995	0.473	10.541	11.485	7.239	0.742	6.586	8.046
ENSG00000135473	PAN2	1.5	2.3E-11	2.6E-10	15.897	0.033	15.861	15.926	10.640	0.859	9.655	11.232
ENSG00000135951	TSGA10	1.5	2.7E-04	7.2E-04	2.410	0.151	2.239	2.526	1.648	0.333	1.277	1.923
ENSG00000136147	PHF11	1.5	3.4E-05	1.1E-04	5.129	0.208	4.895	5.293	3.501	0.178	3.296	3.607
ENSG00000136193	SCRN1	1.5	6.1E-19	2.6E-17	126.477	1.537	125.527	128.250	82.619	1.997	81.398	84.923
ENSG00000136274	NACAD	1.5	1.3E-04	3.7E-04	3.436	0.348	3.036	3.673	2.316	0.317	2.059	2.670
ENSG00000138134	STAMBPL1	1.5	1.2E-05	4.2E-05	4.554	0.461	4.159	5.060	3.123	0.483	2.686	3.647
ENSG00000138166	DUSP5	1.5	5.7E-13	8.7E-12	65.872	2.979	63.019	68.962	42.951	1.284	41.506	43.959
ENSG00000138303	ASCC1	1.5	1.3E-12	1.8E-11	24.004	1.132	23.334	25.310	15.846	0.271	15.550	16.083
ENSG00000139547	RDH16	1.5	1.1E-01	1.6E-01	0.643	0.122	0.515	0.757	0.439	0.166	0.323	0.630
ENSG00000139734	DIAPH3	1.5	3.4E-13	5.4E-12	32.321	1.327	30.870	33.475	21.795	0.591	21.131	22.261
ENSG00000140104	C14orf79	1.5	2.9E-03	6.2E-03	1.806	0.364	1.447	2.175	1.239	0.075	1.157	1.305
ENSG00000140398	NEIL1	1.5	1.5E-04	4.2E-04	2.023	0.254	1.869	2.316	1.321	0.078	1.236	1.389
ENSG00000140443	IGF1R	1.5	2.1E-17	7.1E-16	50.244	1.892	48.433	52.208	33.368	0.183	33.162	33.512
ENSG00000140876	NUDT7	1.5	2.4E-05	8.0E-05	13.726	1.243	12.387	14.844	9.375	1.126	8.102	10.240
ENSG00000140945	CDH13	1.5	6.8E-02	1.0E-01	0.235	0.064	0.166	0.292	0.161	0.013	0.150	0.176
ENSG00000141391	PRELID3A	1.5	1.2E-03	2.9E-03	5.152	0.265	4.847	5.326	3.467	0.711	2.819	4.228
ENSG00000141562	NARF	1.5	1.1E-12	1.6E-11	26.757	1.018	25.993	27.912	18.309	0.400	18.071	18.770
ENSG00000141738	GRB7	1.5	5.7E-11	5.8E-10	26.830	1.120	25.694	27.933	17.994	1.277	16.582	19.069
ENSG00000142405	NLRP12	1.5	9.6E-03	1.8E-02	1.378	0.064	1.304	1.425	0.940	0.260	0.670	1.188
ENSG00000143367	TUFT1	1.5	1.3E-09	1.0E-08	21.418	2.299	18.919	23.443	13.978	0.560	13.631	14.624
ENSG00000143429		1.5	2.8E-03	5.9E-03	3.457	0.842	2.874	4.422	2.349	0.320	2.012	2.649
ENSG00000143545	RAB13	1.5	2.8E-14	5.5E-13	112.937	3.472	109.127	115.923	74.993	2.549	72.246	77.283
ENSG00000143641	GALNT2	1.5	8.4E-17	2.5E-15	74.210	0.320	73.949	74.567	50.492	1.417	48.890	51.583
ENSG00000143774	GUK1	1.5	1.2E-11	1.4E-10	32.393	0.278	32.169	32.704	22.088	2.033	20.888	24.444
ENSG00000143815	LBR	1.5	8.3E-17	2.5E-15	133.792	1.934	132.477	136.012	88.787	4.071	84.432	92.497
ENSG00000143882	ATP6V1C2	1.5	4.5E-02	7.2E-02	1.022	0.282	0.751	1.314	0.680	0.034	0.641	0.702
ENSG00000144366	GULP1	1.5	9.9E-04	2.4E-03	71.681	6.427	64.438	76.702	49.062	3.884	45.727	53.326
ENSG00000145194	ECE2	1.5	4.3E-07	2.0E-06	6.457	0.695	5.995	7.256	4.330	0.018	4.313	4.350
ENSG00000145642	FAM159B	1.5	4.1E-05	1.3E-04	18.166	0.964	17.193	19.122	12.507	1.025	11.431	13.471
ENSG00000145780	FEM1C	1.5	3.8E-13	6.0E-12	27.773	0.656	27.016	28.188	18.249	0.988	17.186	19.166
ENSG00000145920	CPLX2	1.5	9.2E-02	1.4E-01	0.420	0.144	0.261	0.541	0.281	0.098	0.211	0.394
ENSG00000146858	ZC3HAV1L	1.5	1.6E-03	3.6E-03	9.493	0.471	9.069	10.000	6.182	1.223	5.020	7.459
[illegible]	[illegible]	[illegible]	[illegible]	[illegible]	[illegible]	[illegible]	[illegible]	[illegible]	[illegible]	[illegible]	[illegible]	[illegible]

ENSG00000229007		1.6	1.8E-06	7.5E-06	62.489	6.474	58.580	69.962	37.946	3.410	35.223	41.771
ENSG00000228506		1.6	2.5E-07	1.2E-06	14.632	0.384	14.323	15.063	9.048	0.483	8.491	9.356
ENSG00000229358	DPY19L1P1	1.6	3.1E-03	6.6E-03	2.124	0.238	1.909	2.380	1.322	0.290	1.078	1.642
ENSG00000230551		1.6	1.2E-14	2.6E-13	17.795	0.955	16.693	18.382	10.948	0.384	10.516	11.249
ENSG00000231160	KLF3-AS1	1.6	1.3E-02	2.3E-02	0.814	0.071	0.736	0.874	0.518	0.083	0.423	0.578
ENSG00000231365		1.6	7.1E-05	2.2E-04	3.316	0.669	2.585	3.900	2.141	0.245	1.969	2.422
ENSG00000231544	RSL24D1P11	1.6	4.3E-03	8.9E-03	13.719	1.228	12.307	14.546	8.799	2.424	6.117	10.831
ENSG00000231721	LINC-PINT	1.6	2.1E-03	4.6E-03	1.395	0.105	1.294	1.504	0.861	0.215	0.619	1.028
ENSG00000232093	DCST1-AS1	1.6	2.6E-02	4.5E-02	3.939	1.512	2.422	5.445	2.420	0.479	1.869	2.737
ENSG00000232320		1.6	1.1E-04	3.2E-04	34.937	5.228	29.356	39.720	22.488	3.505	18.452	24.624
ENSG00000232593	KANTR	1.6	7.9E-08	4.3E-07	4.592	0.563	4.120	5.216	2.925	0.137	2.802	3.073
ENSG00000233527	ZNF529-AS1	1.6	1.7E-04	4.9E-04	5.708	0.636	5.024	6.280	3.487	0.702	2.842	4.234
ENSG00000234129		1.6	1.6E-02	2.9E-02	1.160	0.127	1.016	1.253	0.707	0.139	0.620	0.867
ENSG00000234498	RPL13AP20	1.6	1.6E-06	6.6E-06	39.185	0.940	38.106	39.824	24.884	3.410	21.052	27.586
ENSG00000235703	LINC00894	1.6	4.2E-03	8.6E-03	0.824	0.100	0.708	0.886	0.527	0.012	0.518	0.541
ENSG00000235706	DICER1-AS1	1.6	1.8E-05	6.1E-05	3.872	0.575	3.515	4.536	2.413	0.092	2.308	2.477
ENSG00000236892	RPL12P12	1.6	1.1E-06	4.9E-06	49.558	2.927	46.621	52.476	30.885	4.830	25.308	33.697
ENSG00000237668	RPS15AP38	1.6	3.6E-08	2.1E-07	94.502	3.326	91.270	97.914	58.114	2.181	56.544	60.604
ENSG00000237921		1.6	5.4E-02	8.5E-02	4.981	0.639	4.601	5.719	3.012	0.393	2.650	3.429
ENSG00000240231	RPS27P29	1.6	7.5E-07	3.3E-06	104.557	14.826	90.429	119.994	65.182	5.604	60.405	71.351
ENSG00000240972	MIF	1.6	4.2E-19	1.9E-17	823.127	19.209	801.132	836.601	525.600	19.481	511.014	547.724
ENSG00000242327		1.6	6.8E-03	1.3E-02	12.886	1.319	11.560	14.198	8.092	2.938	5.276	11.138
ENSG00000243753	HLA-L	1.6	6.9E-04	1.7E-03	1.654	0.374	1.339	2.066	1.013	0.080	0.928	1.087
ENSG00000243859	RPL5P17	1.6	2.2E-02	3.8E-02	4.320	0.400	3.887	4.674	2.739	1.156	1.406	3.472
ENSG00000244509	APOBEC3C	1.6	9.4E-15	2.0E-13	57.337	0.951	56.276	58.114	35.671	1.563	34.623	37.468
ENSG00000245317		1.6	8.4E-02	1.3E-01	1.478	0.580	0.893	2.054	0.957	0.354	0.673	1.354
ENSG00000246596		1.6	4.8E-04	1.2E-03	6.354	1.010	5.550	7.487	4.051	0.296	3.717	4.292
ENSG00000247373		1.6	2.2E-02	3.9E-02	0.388	0.125	0.314	0.532	0.246	0.022	0.231	0.272
ENSG00000247675	LRP4-AS1	1.6	5.4E-02	8.6E-02	1.650	0.391	1.233	2.008	1.033	0.351	0.682	1.394
ENSG00000248383	PCDHAC1	1.6	4.8E-06	1.8E-05	2.426	0.353	2.023	2.683	1.486	0.068	1.408	1.532
ENSG00000249264	EEF1A1P9	1.6	2.5E-11	2.8E-10	92.203	3.487	88.178	94.310	58.446	8.027	50.860	66.852
ENSG00000249898	MCPH1-AS1	1.6	9.0E-03	1.7E-02	1.786	0.408	1.447	2.238	1.141	0.136	1.030	1.292
ENSG00000250067	YJEFN3	1.6	1.5E-02	2.8E-02	1.483	0.125	1.345	1.591	0.936	0.360	0.523	1.180
ENSG00000250328		1.6	3.8E-03	7.9E-03	2.512	0.345	2.166	2.857	1.561	0.152	1.474	1.737
ENSG00000251095		1.6	9.3E-07	4.1E-06	18.871	1.183	17.523	19.738	11.768	1.440	10.156	12.926
ENSG00000251595	ABCA11P	1.6	7.4E-03	1.4E-02	1.821	0.664	1.272	2.560	1.112	0.105	1.001	1.209
ENSG00000254277		1.6	1.7E-13	2.9E-12	150.043	6.833	142.743	156.284	95.006	3.904	90.517	97.603
ENSG00000254413	CHKB-CPT1B	1.6	8.6E-02	1.3E-01	0.301	0.091	0.215	0.396	0.185	0.071	0.109	0.250
ENSG00000254577		1.6	5.3E-02	8.4E-02	11.665	0.139	11.531	11.808	7.312	2.188	5.378	9.687
ENSG00000256633		1.6	1.7E-02	3.1E-02	6.970	0.607	6.600	7.671	4.379	1.308	2.964	5.545
ENSG00000256667	KLRA1P	1.6	1.1E-06	4.9E-06	9.303	0.165	9.183	9.491	5.675	0.455	5.161	6.026
ENSG00000257557	PPP1R12A-AS	1.6	2.1E-03	4.6E-03	2.031	0.375	1.623	2.359	1.290	0.133	1.138	1.391
ENSG00000257698		1.6	1.4E-02	2.5E-02	4.115	1.123	3.464	5.412	2.642	0.337	2.357	3.014
ENSG00000258472		1.6	1.5E-04	4.2E-04	3.764	0.610	3.119	4.332	2.305	0.438	1.879	2.754
ENSG00000258655	ARHGAP5-AS	1.6	2.4E-03	5.3E-03	3.503	0.540	3.007	4.078	2.175	0.462	1.674	2.583
ENSG00000259470	KRT8P9	1.6	1.0E-02	1.9E-02	2.869	0.115	2.792	3.001	1.785	0.458	1.278	2.169
ENSG00000259865		1.6	3.0E-04	7.9E-04	11.048	2.237	8.485	12.610	7.149	1.160	6.037	8.352
ENSG00000260948		1.6	1.4E-03	3.2E-03	3.936	0.764	3.290	4.779	2.540	0.047	2.486	2.571
ENSG00000261366	MANEA-AS1	1.6	3.4E-02	5.7E-02	1.495	0.670	1.002	2.257	0.961	0.211	0.721	1.116
ENSG00000261409		1.6	1.3E-09	1.0E-08	14.171	0.970	13.386	15.255	8.813	0.941	8.217	9.898
ENSG00000262333	HNRNPA1P16	1.6	5.4E-03	1.1E-02	5.986	0.994	5.010	6.998	3.874	0.875	2.944	4.681
ENSG00000263786		1.6	4.6E-03	9.5E-03	7.702	1.150	6.458	8.726	4.972	0.505	4.481	5.490
ENSG00000264456		1.6	2.0E-02	3.5E-02	2.412	0.135	2.323	2.568	1.476	0.674	0.846	2.187
ENSG00000265254		1.6	3.3E-02	5.6E-02	5.549	1.305	4.280	6.888	3.385	0.601	2.766	3.967
ENSG00000265800		1.6	1.2E-02	2.1E-02	10.605	1.275	9.242	11.768	6.474	1.413	4.921	7.683
ENSG00000266680		1.6	2.3E-02	4.0E-02	2.291	0.230	2.025	2.428	1.417	0.348	1.102	1.791
ENSG00000267076	CCDC58P3	1.6	2.7E-04	7.3E-04	22.886	1.532	21.178	24.139	14.171	3.239	10.465	16.456
ENSG00000267277		1.6	3.7E-02	6.1E-02	3.950	0.484	3.395	4.284	2.509	0.404	2.048	2.799
ENSG00000267309		1.6	1.2E-02	2.3E-02	3.260	0.737	2.495	3.965	2.094	0.175	1.907	2.253
ENSG00000268201		1.6	4.7E-02	7.6E-02	5.612	2.614	3.246	8.419	3.618	0.088	3.556	3.719
ENSG00000268225		1.6	2.3E-05	7.7E-05	11.850	0.769	11.080	12.617	7.279	1.254	6.167	8.637
ENSG00000268903		1.6	5.6E-05	1.7E-04	15.418	1.770	13.811	17.316	9.584	0.726	9.112	10.420
ENSG00000270069	MIR222HG	1.6	6.8E-06	2.5E-05	8.790	1.385	7.759	10.364	5.571	0.346	5.288	5.957
ENSG00000271966		1.6	3.1E-03	6.6E-03	5.775	0.574	5.250	6.388	3.614	1.133	2.400	4.644
ENSG00000272047	GTF2H5	1.6	9.5E-12	1.2E-10	10.187	0.158	10.048	10.358	6.292	0.337	5.976	6.647
ENSG00000272168	CASC15	1.6	2.5E-08	1.5E-07	2.489	0.087	2.439	2.589	1.524	0.138	1.428	1.683
ENSG00000272196	HIST2H2AA4	1.6	5.8E-03	1.2E-02	7.459	0.243	7.206	7.689	4.612	0.360	4.393	5.028
ENSG00000272758		1.6	3.7E-03	7.6E-03	1.946	0.341	1.574	2.242	1.200	0.072	1.119	1.256
ENSG00000273102		1.6	2.5E-02	4.3E-02	7.272	1.090	6.015	7.965	4.409	2.642	2.307	7.375
ENSG00000275765		1.6	2.3E-02	4.0E-02	4.193	0.342	3.827	4.505	2.610	0.353	2.324	3.005
ENSG00000276524		1.6	9.2E-02	1.4E-01	2.914	0.327	2.624	3.268	1.863	0.465	1.415	2.344
ENSG00000278023	RDM1	1.6	2.7E-05	8.9E-05	9.987	0.765	9.452	10.863	6.238	1.023	5.305	7.332
ENSG00000278811	LINC00624	1.6	1.5E-02	2.7E-02	0.551	0.079	0.499	0.642	0.333	0.009	0.326	0.343
ENSG00000279088		1.6	1.8E-06	7.6E-06	19.696	1.587	18.107	21.282	12.754	1.871	10.591	14.127
ENSG00000279355	AGPAT4-IT1	1.6	8.5E-03	1.6E-02	3.222	0.525	2.630	3.630	2.066	0.607	1.413	2.614
ENSG00000279400		1.6	1.5E-02	2.7E-02	1.793	0.458	1.376	2.283	1.137	0.314	0.912	1.495
ENSG00000280099		1.6	1.5E-02	2.7E-02	2.350	0.440	1.978	2.836	1.425	0.596	0.737	1.790
ENSG00000280123		1.6	8.7E-08	4.7E-07	16.812	2.380	14.180	18.810	10.658	0.732	9.897	11.357
ENSG00000280322		1.6	8.6E-04	2.1E-03	9.226	2.177	7.357	11.616	5.756	0.778	4.875	6.348
ENSG00000280594		1.6	3.5E-02	5.8E-02	1.061	0.084	1.007	1.158	0.683	0.122	0.582	0.818
ENSG00000282508	LINC01002	1.6	1.4E-04	4.1E-04	1.386	0.051	1.339	1.441	0.891	0.044	0.856	0.941
ENSG00000001617	SEMA3F	1.7	1.6E-17	5.4E-16	44.829	1.339	44.017	46.375	26.189	1.495	24.806	27.775
ENSG00000006837	CDKL3	1.7	8.0E-03	1.5E-02	1.028	0.279	0.707	1.209	0.591	0.086	0.535	0.690
ENSG00000007384	RHBDF1	1.7	1.0E-11	1.2E-10	15.374	0.450	15.096	15.893	9.193	0.546	8.585	9.642
ENSG00000029534	ANK1	1.7	1.8E-11	2.1E-10	6.651	0.240	6.389	6.860	3.813	0.243	3.544	4.015
ENSG00000033867	SLC4A7	1.7	1.4E-19	6.9E-18	87.332	1.929	86.035	89.549	51.675	2.516	48.810	53.526
ENSG00000047346	FAM214A	1.7	1.9E-10	1.7E-09	5.162	0.223	4.917	5.353	3.067	0.235	2.802	3.246
ENSG00000050405	LIMA1	1.7	5.0E-18	1.9E-16	72.790	2.703	69.822	75.110	42.806	1.402	41.772	44.401
ENSG00000054179	ENTPD2	1.7	2.0E-03	4.5E-03	2.296	0.364	1.876	2.528	1.369	0.207	1.220	1.605
ENSG00000057294	PKP2	1.7	1.1E-17	3.8E-16	37.413	0.966	36.653	38.500	21.925	0.598	21.321	22.517
ENSG00000060642	PIGV	1.7	3.5E-10	3.1E-09	13.137	0.848	12.322	14.015	7.830	0.771	7.063	8.605
ENSG00000060656	PTPRU	1.7	8.8E-18	3.2E-16	36.925	1.368	35.600	38.333	22.000	0.084	21.930	22.093
ENSG00000060982	BCAT1	1.7	1.0E-21	7.0E-20	295.804	4.068	291.106	298.188	177.611	8.484	168.962	185.919
ENSG00000064102	INTS13	1.7	9.3E-20	4.6E-18	130.400	3.123	126.847	132.708	77.384	1.238	76.518	78.802
ENSG00000065618	COL17A1	1.7	3.5E-03	7.4E-03	0.847	0.199	0.620	0.985	0.498	0.137	0.413	0.656
ENSG00000065911	MTHFD2	1.7	1.2E-21	7.9E-20	316.416	9.714	309.340	327.492	183.416	6.592	176.459	189.569
ENSG00000067798	NAV3	1.7	5.5E-07	2.5E-06	1.594	0.062	1.535	1.659	0.915	0.090	0.811	0.967
ENSG00000070404	FSTL3	1.7	6.8E-05	2.1E-04	3.849	0.609	3.472	4.552	2.293	0.496	1.788	2.778
ENSG00000071575	TRIB2	1.7	2.5E-15	5.8E-14	90.900	6.127	87.031	97.965	52.849	2.338	51.267	55.534
ENSG00000072042	RDH11	1.7	2.7E-21	1.7E-19	141.109	2.087	138.963	143.132	82.323	2.268	80.080	84.615
ENSG00000075826	SEC31B	1.7	1.4E-04	4.0E-04	1.251	0.121	1.128	1.370	0.758	0.046	0.721	0.809
ENSG00000078687	TNRC6C	1.7	6.2E-16	1.6E-14	17.387	0.453	17.122	17.910	10.091	0.371	9.756	10.490
ENSG00000079841	RIMS1	1.7	5.3E-05	1.6E-04	1.010	0.115	0.941	1.142	0.608	0.095	0.513	0.704
ENSG00000080561	MID2	1.7	2.0E-04	5.4E-04	2.967	0.097	2.891	3.076	1.738	0.488	1.189	2.120
ENSG00000088826	SMOX	1.7	1.0E-09	8.2E-09	15.092	0.296	14.865	15.428	9.034	0.331	8.733	9.388
ENSG00000089094	KDM2B	1.7	8.0E-20	3.9E-18	45.077	0.990	44.077	46.057	26.371	0.238	26.145	26.619
ENSG00000089163	SIRT4	1.7	2.8E-05	9.1E-05	9.501	0.822	8.715	10.356	5.678	0.538	5.062	6.058
ENSG00000095015	MAP3K1	1.7	1.5E-16	4.3E-15	22.804	0.368	22.446	23.182	13.083	1.041	12.311	14.267
ENSG00000095380	NANS	1.7	5.3E-16	1.4E-14	33.748	1.135	32.441	34.495	20.169	0.584	19.538	20.689
ENSG00000100075	SLC25A1	1.7	4.3E-11	4.6E-10	256.084	6.366	250.320	262.917	150.444	15.640	136.023	167.070
ENSG00000101608	MYL12A	1.7	2.3E-21	1.4E-19	284.153	4.704	278.802	287.634	168.868	0.309	168.551	169.168
ENSG00000103316	CRYM	1.7	5.5E-08	3.0E-07	7.467	0.746	6.824	8.285	4.477	0.492	3.915	4.831
ENSG00000105700	KXD1	1.7	3.3E-19	1.5E-17	82.352	1.230	80.960	83.291	49.900	1.075	48.748	50.876
ENSG00000105707	HPN	1.7	1.1E-09	8.7E-09	12.113	1.058	11.478	13.334	6.995	0.303	6.687	7.302
ENSG00000106089	STX1A	1.7	8.0E-05	2.4E-04	3.887	0.180	3.696	4.054	2.359	0.261	2.068	2.572
ENSG00000108786	HSD17B1	1.7	2.7E-02	4.6E-02	0.550	0.073	0.465	0.596	0.331	0.087	0.241	0.414
ENSG00000109099	PMP22	1.7	3.5E-03	7.3E-03	2.068	0.818	1.319	2.941	1.187	0.236	0.961	1.433
ENSG00000109519	GRPEL1	1.7	1.6E-15	3.9E-14	53.047	0.967	52.306	54.141	32.102	1.793	30.901	34.164
ENSG00000110011	DNAJC4	1.7	3.8E-07	1.8E-06	9.455	1.057	8.732	10.668	5.727	0.525	5.295	6.311
ENSG00000110888	CAPRIN2	1.7	4.9E-16	1.3E-14	25.360	1.689	24.161	27.292	15.266	0.096	15.164	15.355
ENSG00000112379	ARFGEF3	1.7	7.4E-18	2.7E-16	17.908	0.431	17.544	18.385	10.349	0.179	10.197	10.547
ENSG00000113070	HBEGF	1.7	9.1E-06	3.3E-05	5.009	0.647	4.617	5.755	2.889	0.522	2.304	3.308
ENSG00000114656	KIAA1257	1.7	1.2E-03	2.8E-03	0.869	0.131	0.771	1.018	0.523	0.123	0.402	0.648
ENSG00000117154	IGSF21	1.7	7.1E-10	5.8E-09	18.618	1.976	16.911	20.783	10.969	1.658	9.282	12.597
ENSG00000117595	IRF6	1.7	1.5E-14	3.0E-13	22.716	0.426	22.226	22.999	13.495	0.896	12.701	14.466
ENSG00000118432	CNR1	1.7	5.8E-03	1.2E-02	0.660	0.143	0.514	0.801	0.387	0.094	0.279	0.442
ENSG00000118804	STBD1	1.7	1.2E-02	2.2E-02	1.074	0.356	0.664	1.309	0.638	0.217	0.399	0.822
ENSG00000119411	BSPRY	1.7	3.4E-06	1.3E-05	9.837	1.151	8.645	10.942	5.963	1.369	4.778	7.462
ENSG00000120137	PANK3	1.7	4.1E-19	1.9E-17	103.028	4.277	99.116	107.594	59.297	2.160	56.833	60.859
ENSG00000120708	TGFBI	1.7	1.1E-09	8.7E-09	5.957	0.255	5.757	6.245	3.575	0.162	3.405	3.726
ENSG00000121297	TSHZ3	1.7	1.3E-12	1.8E-11	14.736	0.702	14.153	15.515	8.809	0.099	8.697	8.885
ENSG00000124006	OBSL1	1.7	5.9E-11	6.0E-10	41.433	2.180	39.412	43.742	24.310	2.949	21.467	27.355
ENSG00000125124	BBS2	1.7	1.8E-13	3.1E-12	10.231	0.134	10.114	10.377	6.184	0.103	6.065	6.248
ENSG00000125731	SH2D3A	1.7	4.7E-14	8.9E-13	19.562	1.160	18.855	20.900	11.255	0.903	10.606	12.286
ENSG00000129203	ASPHD2	1.7	1.5E-09	1.2E-08	14.198	1.410	12.813	15.633	8.563	0.534	7.989	9.046
ENSG00000130244	FAM98C	1.7	8.6E-06	3.1E-05	3.075	0.345	2.690	3.357	1.765	0.304	1.424	2.005
ENSG00000130997	POLN	1.7	4.4E-04	1.1E-03	1.069	0.110	0.946	1.160	0.641	0.127	0.528	0.778
ENSG00000131080	EDA2R	1.7	7.2E-12	8.9E-11	18.131	0.530	17.520	18.459	10.809	0.948	9.754	11.588
ENSG00000131089	ARHGEF9	1.7	1.5E-16	4.2E-15	13.518	0.548	12.923	14.003	7.804	0.368	7.432	8.169
ENSG00000131094	C1QL1	1.7	3.0E-06	8.0E-06	7.221	0.856	6.258	7.897	4.319	1.060	3.128	5.160
ENSG00000132334	PTPRE	1.7	1.6E-03	3.6E-03	0.440	0.031	0.405	0.462	0.251	0.066	0.175	0.298
ENSG00000132692	BCAN	1.7	9.3E-05	2.8E-04	1.940	0.220	1.784	2.191	1.152	0.248	0.988	1.438
ENSG00000132824	SERINC3	1.7	2.3E-18	9.0E-17	75.063	0.679	74.327	75.666	45.266	0.490	44.733	45.698
ENSG00000133454	MYO18B	1.7	1.1E-02	2.1E-02	0.353	0.108	0.234	0.445	0.205	0.068	0.173	0.261
ENSG00000134595	SOX3	1.7	1.6E-11	1.8E-10	48.359	1.844	46.484	50.171	28.051	3.568	24.046	30.893
ENSG00000135605	TEC	1.7	2.1E-07	1.0E-06	7.867	0.379	7.430	8.115	4.748	0.678	4.229	5.515
ENSG00000135740	SLC9A5	1.7	5.3E-07	2.4E-06	3.990	0.548	3.359	4.329	2.377	0.259	2.189	2.672
ENSG00000137449	CPEB2	1.7	4.2E-06	1.6E-05	2.015	0.248	1.787	2.278	1.200	0.101	1.102	1.304
ENSG00000138028	CGREF1	1.7	2.4E-06	9.5E-06	5.567	0.595	4.964	6.153	3.312	0.459	2.801	3.690
ENSG00000138769	CDKL2	1.7	1.2E-03	2.8E-03	1.510	0.105	1.391	1.592	0.880	0.160	0.702	1.010
ENSG00000138835	RGS3	1.7	3.1E-12	4.1E-11	6.538	0.226	6.320	6.770	3.886	0.332	3.629	4.261
ENSG00000139926	FRMD6	1.7	2.8E-14	5.6E-13	14.833	0.915	13.802	15.549	8.962	0.553	8.371	9.466
ENSG00000141837	CACNA1A	1.7	4.3E-09	3.0E-08	0.976	0.058	0.921	1.037	0.569	0.082	0.476	0.628
ENSG00000142192	APP	1.7	2.3E-23	2.1E-21	620.466	0.887	619.468	621.162	362.583	2.494	358.704	364.080
ENSG00000143067	ZNF697	1.7	1.6E-15	3.8E-14	45.780	1.908	44.537	47.978	26.314	0.857	25.401	27.101
ENSG00000147454	SLC25A37	1.7	2.4E-16	6.8E-15	30.541	0.917	29.972	31.599	18.245	0.259	17.960	18.466
ENSG00000147606	SLC26A7	1.7	9.6E-02	1.4E-01	0.231	0.102	0.113	0.295	0.133	0.040	0.087	0.157
ENSG00000149091	DGKZ	1.7	1.5E-15	3.6E-14	19.064	1.066	17.960	20.086	10.986	0.948	10.267	12.060
ENSG00000151474	FRMD4A	1.7	2.0E-16	5.6E-15	15.548	0.421	15.113	15.954	8.951	0.664	8.301	9.628
ENSG00000154328	NEIL2	1.7	1.9E-12	2.7E-11	30.284	1.535	28.801	31.866	18.154	0.555	17.788	18.792
ENSG00000154917	RAB6B	1.7	2.0E-09	1.5E-08	10.210	0.662	9.448	10.641	5.858	0.112	5.746	5.970
ENSG00000155111	CDK19	1.7	6.6E-11	6.6E-10	8.413	0.764	7.540	8.958	5.061	0.118	4.932	5.165
ENSG00000156218	ADAMTSL3	1.7	9.4E-04	2.3E-03	0.652	0.159	0.475	0.782	0.376	0.090	0.293	0.473
ENSG00000156564	LRFN2	1.7	8.8E-05	2.6E-04	2.778	0.209	2.549	2.957	1.615	0.105	1.501	1.709
ENSG00000157193	LRP8	1.7	2.0E-20	1.1E-18	50.012	2.034	47.758	51.712	29.379	0.840	28.864	30.349
ENSG00000158711	ELK4	1.7	3.8E-14	7.3E-13	19.700	0.659	18.966	20.241	11.841	0.307	11.649	12.195
ENSG00000160712	IL6R	1.7	2.9E-06	1.1E-05	3.652	0.638	2.953	4.201	2.088	0.202	1.883	2.288
ENSG00000161638	ITGA5	1.7	3.1E-16	8.5E-15	26.338	1.177	24.983	27.114	15.321	1.073	14.189	16.323
ENSG00000162409	PRKAA2	1.7	1.5E-13	2.5E-12	11.244	0.475	10.808	11.750	6.547	0.532	5.933	6.884
ENSG00000164105	SAP30	1.7	5.3E-11	5.5E-10	60.555	2.611	58.060	63.268	35.371	2.593	32.438	37.356
ENSG00000164124	TMEM144	1.7	4.1E-07	1.9E-06	2.176	0.064	2.121	2.246	1.296	0.054	1.254	1.357
ENSG00000164305	CASP3	1.7	2.9E-20	1.5E-18	233.580	5.349	230.471	239.756	134.768	4.732	129.848	139.287
ENSG00000164975	SNAPC3	1.7	3.2E-16	8.9E-15	34.233	1.136	33.241	35.472	19.971	1.097	18.705	20.605
ENSG00000166130	IKBIP	1.7	1.2E-11	1.4E-10	18.714	0.749	18.068	19.534	11.194	0.460	10.672	11.543
ENSG00000166575	TMEM135	1.7	2.7E-19	1.3E-17	56.738	0.748	56.112	57.566	32.581	0.736	31.827	33.297
ENSG00000167578	RAB4B	1.7	5.0E-06	1.9E-05	3.885	0.581	3.509	4.555	2.264	0.394	2.012	2.718
ENSG00000168615	ADAM9	1.7	6.3E-16	1.6E-14	34.048	3.304	30.432	36.911	19.664	1.176	18.305	20.348
ENSG00000168961	LGALS9	1.7	6.0E-02	9.3E-02	0.312	0.067	0.236	0.360	0.186	0.061	0.131	0.253
ENSG00000169570	DTWD2	1.7	4.3E-09	3.0E-08	9.827	0.323	9.461	10.070	5.709	0.580	5.055	6.202
ENSG00000171097	KYAT1	1.7	9.3E-14	1.7E-12	23.879	1.080	22.678	24.769	13.873	0.287	13.659	14.199
ENSG00000171206	TRIM8	1.7	1.2E-11	1.4E-10	28.384	1.316	26.867	29.224	16.966	2.001	14.707	18.514
ENSG00000171223	JUNB	1.7	7.3E-06	2.7E-05	7.421	0.853	6.511	8.203	4.405	0.459	3.896	4.789
ENSG00000171291	ZNF439	1.7	3.3E-08	1.9E-07	5.340	0.338	5.003	5.680	3.130	0.234	2.868	3.316
ENSG00000172794	RAB37	1.7	2.0E-07	1.0E-06	4.449	0.423	4.031	4.876	2.676	0.273	2.392	2.937
ENSG00000172840	PDP2	1.7	4.2E-17	1.3E-15	31.269	0.559	30.744	31.858	18.429	0.236	18.227	18.689
ENSG00000172890	NADSYN1	1.7	2.0E-15	4.8E-14	7.897	0.486	7.335	8.181	4.524	0.039	4.484	4.561
ENSG00000173805	HAP1	1.7	3.7E-05	1.2E-04	2.152	0.486	1.736	2.687	1.279	0.158	1.100	1.401
ENSG00000173890	GPR160	1.7	6.9E-17	2.1E-15	46.709	1.455	45.222	48.129	27.160	1.638	25.816	28.984
ENSG00000174669	SLC29A2	1.7	8.8E-14	1.6E-12	47.774	4.795	42.332	51.381	28.387	0.921	27.527	29.359
ENSG00000175183	CSRP2	1.7	3.7E-20	1.9E-18	96.092	2.806	93.185	98.786	58.012	1.057	57.080	59.161
ENSG00000175471	MCTP1	1.7	8.5E-05	2.5E-04	1.118	0.113	1.047	1.249	0.662	0.044	0.616	0.705
ENSG00000176845	METRNL	1.7	3.5E-10	3.1E-09	18.209	1.859	16.893	20.336	10.870	0.823	9.947	11.528
ENSG00000179431	FJX1	1.7	4.2E-09	2.9E-08	12.797	1.005	11.992	13.923	7.345	0.392	7.058	7.792
ENSG00000181016	LSMEM1	1.7	2.2E-03	4.8E-03	1.895	0.180	1.691	2.030	1.107	0.031	1.078	1.139
ENSG00000181798	LINC00471	1.7	1.4E-03	3.3E-03	4.686	0.793	3.798	5.324	2.809	0.775	2.070	3.616
ENSG00000182050	MGAT4C	1.7	2.4E-07	1.2E-06	0.929	0.076	0.845	0.994	0.559	0.053	0.521	0.619
ENSG00000182197	EXT1	1.7	4.2E-15	9.4E-14	21.498	1.797	19.436	22.734	12.745	0.402	12.450	13.202
ENSG00000182310	SPACA6	1.7	4.3E-03	8.8E-03	1.706	0.711	0.889	2.183	1.028	0.105	0.963	1.149
ENSG00000182534	MXRA7	1.7	3.9E-14	7.4E-13	11.710	0.182	11.501	11.832	7.077	0.301	6.796	7.395
ENSG00000182795	C1orf116	1.7	7.6E-09	5.0E-08	6.354	0.161	6.176	6.490	3.725	0.209	3.501	3.916
ENSG00000182901	RGS7	1.7	1.8E-04	5.0E-04	3.207	0.615	2.518	3.700	1.870	0.085	1.801	1.966
ENSG00000182957	SPATA13	1.7	1.6E-15	3.8E-14	20.545	1.438	19.534	22.191	12.448	0.326	12.202	12.818
ENSG00000183117	CSMD1	1.7	8.4E-04	2.0E-03	0.326	0.026	0.297	0.347	0.188	0.026	0.160	0.210
ENSG00000183780	SLC35F3	1.7	4.7E-05	1.5E-04	3.699	0.872	2.754	4.471	2.137	0.060	2.070	2.186
ENSG00000184465	WDR27	1.7	2.6E-13	4.3E-12	11.767	0.752	10.954	12.439	7.080	0.150	6.945	7.241
ENSG00000185015	CA13	1.7	2.5E-04	6.9E-04	1.369	0.122	1.229	1.440	0.820	0.082	0.758	0.913
ENSG00000185522	LMNTD2	1.7	3.4E-02	5.6E-02	0.880	0.145	0.719	1.000	0.521	0.125	0.381	0.623
ENSG00000186020	ZNF529	1.7	3.1E-12	4.2E-11	13.050	1.031	12.030	14.092	7.905	0.677	7.291	8.631
ENSG00000186166	CCDC84	1.7	5.2E-11	5.4E-10	12.476	0.907	11.433	13.084	7.519	0.722	6.690	8.014
ENSG00000186439	TRDN	1.7	1.7E-09	1.3E-08	49.022	2.852	45.729	50.678	29.768	1.670	27.842	30.804
ENSG00000188994	ZNF292	1.7	3.2E-15	7.5E-14	21.142	0.617	20.698	21.846	12.456	0.916	11.405	13.087
ENSG00000189014	FAM35DP	1.7	2.0E-08	1.2E-07	12.592	0.361	12.179	12.849	7.637	1.138	6.830	8.938
ENSG00000196110	ZNF699	1.7	8.4E-06	3.0E-05	4.051	0.546	3.454	4.525	2.426	0.288	2.096	2.622
ENSG00000196549	MME	1.7	4.1E-17	1.3E-15	23.188	0.556	22.799	23.825	13.975	0.542	13.580	14.593
ENSG00000196741	LINC01560	1.7	3.2E-04	8.4E-04	6.239	0.612	5.543	6.695	3.687	0.796	2.805	4.353
ENSG00000197467	COL13A1	1.7	6.7E-07	3.0E-06	3.477	0.211	3.233	3.606	2.107	0.182	1.917	2.279
ENSG00000203288	TDRKH-AS1	1.7	5.2E-04	1.3E-03	6.369	0.117	6.259	6.493	3.854	0.188	3.673	4.066
ENSG00000204186	ZDBF2	1.7	6.0E-15	1.3E-13	14.182	1.237	13.091	15.526	8.315	0.342	7.992	8.673
ENSG00000204194	RPL12P1	1.7	7.9E-03	1.5E-02	7.580	0.797	6.812	8.403	4.560	1.088	3.680	5.776
ENSG00000204311	DFNB59	1.7	1.6E-03	3.6E-03	2.236	0.033	2.200	2.262	1.278	0.513	0.687	1.610
ENSG00000204711	C9orf135	1.7	7.3E-08	4.0E-07	20.064	2.327	18.223	22.679	11.941	0.509	11.432	12.451
ENSG00000204789	ZNF204P	1.7	2.5E-12	3.3E-11	40.965	2.096	39.479	43.362	24.357	2.172	23.097	26.865
ENSG00000205485		1.7	5.4E-04	1.4E-03	2.458	0.324	2.084	2.653	1.481	0.339	1.221	1.864
ENSG00000211459	MT-RNR1	1.7	9.5E-21	5.4E-19	3614.608	87.868	3518.975	3691.776	2182.345	113.965	2070.498	2298.316
ENSG00000211772	TRBC2	1.7	1.5E-13	2.6E-12	127.333	6.089	121.022	133.172	73.500	3.385	69.708	76.217
ENSG00000213406	ANXA2P1	1.7	1.9E-03	4.2E-03	6.791	1.078	5.655	7.800	4.077	0.293	3.755	4.327
ENSG00000213493	ACTN4P1	1.7	7.0E-03	1.4E-02	3.059	0.645	2.677	3.803	1.833	0.302	1.492	2.068
ENSG00000213700	RPL17P50	1.7	3.2E-06	1.3E-05	24.146	1.972	22.836	26.415	14.023	0.781	13.212	14.770
ENSG00000214946	TBC1D26	1.7	9.2E-02	1.4E-01	0.238	0.056	0.187	0.298	0.138	0.062	0.074	0.197
ENSG00000215424	MCM3AP-AS1	1.7	8.8E-10	7.0E-09	4.616	0.133	4.482	4.749	2.731	0.191	2.580	2.949
ENSG00000216285		1.7	9.8E-20	4.8E-18	847.326	21.000	830.736	870.937	487.766	4.065	484.640	492.361
ENSG00000218582	GAPDHP63	1.7	1.4E-02	2.5E-02	10.506	1.748	8.787	12.283	6.289	3.023	4.162	9.750
ENSG00000223478		1.7	2.1E-04	5.7E-04	15.313	2.335	13.129	17.773	9.187	1.533	7.447	10.338
ENSG00000225603		1.7	8.7E-03	1.7E-02	2.024	0.274	1.854	2.340	1.179	0.293	0.844	1.392
ENSG00000226696	LENG8-AS1	1.7	1.2E-04	3.6E-04	7.071	0.599	6.380	7.421	4.039	0.290	3.830	4.371
ENSG00000226833		1.7	1.4E-01	2.0E-01	0.994	0.245	0.751	1.242	0.573	0.573	0.194	1.233
ENSG00000227953	LINC01341	1.7	2.2E-02	3.9E-02	0.680	0.160	0.535	0.852	0.407	0.078	0.342	0.494
ENSG00000228169	PPIAP19	1.7	1.4E-03	3.2E-03	13.405	0.482	12.918	13.883	8.087	1.471	7.020	9.764
ENSG00000228331		1.7	1.8E-03	4.0E-03	10.927	2.361	8.340	12.964	6.521	1.613	4.801	7.999
ENSG00000230732		1.7	4.0E-02	6.6E-02	1.435	0.210	1.202	1.610	0.819	0.152	0.686	0.989
ENSG00000231185		1.7	7.7E-03	1.5E-02	2.363	0.145	2.198	2.472	1.404	0.351	1.003	1.657
ENSG00000231793	DOC2GP	1.7	6.5E-03	1.3E-02	1.254	0.192	1.032	1.374	0.751	0.161	0.626	0.932
ENSG00000232385	RPS3AP25	1.7	3.3E-05	1.1E-04	15.909	1.196	14.703	17.095	9.650	2.060	7.544	11.660
ENSG00000233396	LINC01719	1.7	2.1E-06	8.5E-06	7.419	0.789	6.694	8.259	4.359	0.271	4.051	4.563
ENSG00000233971	RPS20P10	1.7	1.9E-03	4.2E-03	25.378	5.151	19.951	30.199	15.289	1.922	14.079	17.505
ENSG00000237886	NALT1	1.7	3.4E-03	7.2E-03	5.018	1.856	3.921	7.161	2.886	0.679	2.311	3.635
ENSG00000240303	ACAD11	1.7	1.7E-04	4.9E-04	2.090	0.488	1.529	2.403	1.249	0.211	1.083	1.487
ENSG00000241163	LINC00877	1.7	5.8E-04	1.5E-03	1.327	0.164	1.171	1.497	0.797	0.048	0.745	0.839
ENSG00000241404	EGFL8	1.7	3.6E-03	7.6E-03	1.647	0.152	1.472	1.736	0.969	0.203	0.805	1.197
ENSG00000242028	HYPK	1.7	3.4E-04	8.9E-04	1.215	0.027	1.193	1.245	0.711	0.261	0.546	1.012
ENSG00000243056	EIF4EBP3	1.7	5.7E-03	1.1E-02	7.654	2.555	5.808	10.571	4.497	1.392	3.639	6.104
ENSG00000243422	RPL23AP49	1.7	1.3E-02	2.5E-02	2.993	1.001	1.997	3.998	1.746	0.663	0.982	2.157
ENSG00000246130		1.7	7.1E-03	1.4E-02	1.422	0.229	1.171	1.618	0.825	0.372	0.480	1.219
ENSG00000246582		1.7	1.9E-05	6.3E-05	4.361	0.311	4.050	4.671	2.533	0.358	2.185	2.900
ENSG00000247363		1.7	2.9E-02	4.9E-02	0.965	0.085	0.868	1.027	0.568	0.235	0.297	0.719
ENSG00000247746	USP51	1.7	1.4E-09	1.1E-08	9.047	0.162	8.913	9.227	5.388	0.488	4.977	5.927
ENSG00000248049	UBA6-AS1	1.7	5.2E-12	6.7E-11	10.519	0.263	10.307	10.814	6.143	0.348	5.825	6.515
ENSG00000248605		1.7	9.3E-15	2.0E-13	150.504	5.514	145.046	156.073	88.863	6.208	83.382	95.604
ENSG00000248746	ACTN3	1.7	4.0E-07	1.9E-06	5.256	0.555	4.771	5.861	3.055	0.145	2.908	3.198
ENSG00000248932		1.7	2.7E-03	5.9E-03	2.661	0.161	2.527	2.839	1.601	0.630	0.901	2.122
ENSG00000250144		1.7	2.4E-03	5.3E-03	3.552	0.653	3.044	4.288	2.055	0.287	1.728	2.268
ENSG00000250539	KRT8P33	1.7	2.6E-07	1.2E-06	15.911	1.433	14.264	16.873	9.623	0.466	9.114	10.029
ENSG00000250994		1.7	6.2E-03	1.2E-02	7.278	0.400	6.970	7.729	4.282	0.388	4.011	4.726
ENSG00000253541	SEPT10P1	1.7	6.4E-06	2.4E-05	14.807	2.324	12.154	16.483	8.927	1.275	8.156	10.398
ENSG00000253731	PCDHGA6	1.7	9.2E-04	2.2E-03	0.970	0.165	0.809	1.139	0.580	0.132	0.431	0.684
ENSG00000254285	KRT8P3	1.7	2.7E-15	6.4E-14	134.521	7.390	127.345	142.108	79.966	5.305	75.733	85.918
ENSG00000254502		1.7	7.5E-03	1.5E-02	3.171	0.348	2.789	3.468	1.875	0.069	1.796	1.922
ENSG00000254585	MAGEL2	1.7	4.0E-10	3.5E-09	10.041	0.664	9.301	10.586	6.063	0.248	5.855	6.338
ENSG00000254851		1.7	5.8E-03	1.2E-02	1.439	0.072	1.367	1.512	0.838	0.182	0.628	0.947
ENSG00000255310		1.7	1.4E-08	8.9E-08	14.842	1.260	13.493	15.889	8.571	0.303	8.357	8.918
ENSG00000255495		1.7	2.7E-05	8.8E-05	7.083	0.786	6.337	7.904	4.049	0.389	3.654	4.432
ENSG00000256232	LINC02387	1.7	6.5E-04	1.6E-03	7.753	0.920	6.880	8.713	4.536	0.674	4.085	5.311
ENSG00000257315	ZBED6	1.7	1.0E-02	1.9E-02	0.241	0.076	0.161	0.312	0.137	0.004	0.134	0.142
ENSG00000257497		1.7	6.9E-03	1.4E-02	2.966	0.410	2.495	3.229	1.717	0.093	1.613	1.791
ENSG00000259153		1.7	1.2E-08	7.5E-08	48.505	4.338	44.408	53.049	28.110	2.234	25.557	29.710
ENSG00000259363		1.7	1.3E-02	2.3E-02	2.363	0.076	2.313	2.451	1.416	0.296	1.143	1.731
ENSG00000259658		1.7	9.5E-03	1.8E-02	2.635	0.287	2.304	2.826	1.587	0.368	1.303	2.002
ENSG00000260852	FBXL19-AS1	1.7	3.5E-02	5.8E-02	0.679	0.187	0.469	0.828	0.411	0.128	0.276	0.530
ENSG00000260941	LINC00622	1.7	1.4E-02	2.6E-02	2.242	0.674	1.706	2.998	1.283	0.600	0.638	1.824
ENSG00000261757		1.7	7.9E-03	1.5E-02	2.707	0.243	2.428	2.870	1.586	0.594	0.915	2.044
ENSG00000261786		1.7	3.2E-07	1.5E-06	3.815	0.450	3.473	4.325	2.191	0.144	2.026	2.288
ENSG00000268129		1.7	5.0E-04	1.3E-03	14.134	2.985	11.280	17.235	8.495	1.222	7.268	9.711
ENSG00000269918		1.7	2.5E-05	8.2E-05	6.329	0.571	5.888	6.974	3.729	0.634	3.047	4.300
ENSG00000269974		1.7	1.0E-02	1.9E-02	4.827	0.939	3.880	5.758	2.848	0.939	1.765	3.430
ENSG00000270055		1.7	1.3E-03	3.1E-03	2.472	0.382	2.037	2.752	1.456	0.156	1.327	1.630
ENSG00000271855		1.7	1.4E-02	2.5E-02	3.124	0.146	2.961	3.243	1.799	0.424	1.399	2.244
ENSG00000271871		1.7	5.1E-03	1.0E-02	6.447	1.384	4.852	7.340	3.713	1.059	2.657	4.776
ENSG00000272288		1.7	1.2E-02	2.2E-02	4.622	0.424	4.204	5.053	2.755	0.772	1.993	3.537
ENSG00000272631		1.7	4.4E-06	1.7E-05	2.693	0.314	2.433	3.042	1.583	0.318	1.217	1.792
ENSG00000272644		1.7	1.8E-03	4.0E-03	10.991	1.632	9.666	12.815	6.394	0.507	5.820	6.783
ENSG00000272716		1.7	2.7E-02	4.6E-02	3.851	1.086	2.601	4.560	2.280	0.765	1.766	3.159
ENSG00000272720		1.7	3.8E-02	6.2E-02	1.472	0.445	1.185	1.984	0.874	0.207	0.666	1.079
ENSG00000273456		1.7	5.4E-02	8.5E-02	3.677	0.545	3.135	4.225	2.195	0.556	1.868	2.837
ENSG00000274020	LINC01138	1.7	6.5E-06	2.4E-05	2.180	0.271	1.867	2.349	1.276	0.261	1.026	1.548
ENSG00000274265		1.7	8.5E-06	3.1E-05	12.491	1.242	11.534	13.895	7.342	0.603	6.551	7.664
ENSG00000277767		1.7	1.1E-01	1.6E-01	0.680	0.260	0.390	0.890	0.381	0.335	0.154	0.766
ENSG00000278384		1.7	2.0E-03	4.5E-03	2.137	0.104	2.031	2.239	1.267	0.435	0.925	1.757
ENSG00000278709	NKILA	1.7	5.1E-05	1.6E-04	4.284	0.205	4.124	4.515	2.583	0.147	2.420	2.706
ENSG00000279425		1.7	3.5E-02	5.8E-02	1.305	0.123	1.169	1.410	0.762	0.617	0.321	1.468
ENSG00000279668		1.7	1.9E-03	4.3E-03	0.843	0.056	0.793	0.904	0.495	0.139	0.361	0.638
ENSG00000279765		1.7	5.0E-02	8.0E-02	0.445	0.131	0.309	0.571	0.265	0.138	0.115	0.386
ENSG00000279865		1.7	3.0E-02	5.0E-02	1.530	0.307	1.183	1.770	0.915	0.253	0.627	1.104
ENSG00000280046		1.7	4.9E-04	1.2E-03	0.993	0.125	0.865	1.115	0.570	0.175	0.394	0.743
ENSG00000280058		1.7	5.5E-06	2.1E-05	15.040	0.443	14.661	15.527	8.796	1.072	8.097	10.030
ENSG00000280780	JAKMIP2-AS1	1.7	8.4E-08	4.5E-07	14.347	1.405	13.257	15.934	8.531	0.588	8.082	9.213
ENSG00000283213		1.7	3.4E-02	5.6E-02	0.444	0.072	0.370	0.514	0.269	0.101	0.167	0.370
ENSG00000283491		1.7	5.1E-03	1.0E-02	6.838	0.586	6.351	7.488	4.121	0.712	3.520	4.908
ENSG00000283945	LINC00032	1.7	8.6E-02	1.3E-01	0.425	0.279	0.200	0.738	0.252	0.072	0.182	0.326
ENSG00000005001	PRSS22	1.8	1.2E-02	2.3E-02	1.232	0.077	1.156	1.310	0.680	0.374	0.457	1.112
ENSG00000006432	MAP3K9	1.8	5.6E-20	2.8E-18	21.807	0.291	21.546	22.121	11.822	0.265	11.549	12.079
ENSG00000009694	TENM1	1.8	3.0E-10	2.6E-09	2.862	0.148	2.705	2.999	1.623	0.084	1.544	1.712
ENSG00000062716	VMP1	1.8	1.2E-19	5.7E-18	60.744	0.689	60.302	61.539	33.285	1.825	32.208	35.392
ENSG00000065609	SNAP91	1.8	2.8E-02	4.8E-02	0.381	0.062	0.317	0.441	0.208	0.155	0.042	0.349
ENSG00000065717	TLE2	1.8	2.6E-11	2.8E-10	10.139	0.730	9.296	10.588	5.511	0.361	5.154	5.876
ENSG00000067992	PDK3	1.8	3.6E-14	7.0E-13	40.882	1.592	39.346	42.524	22.505	2.532	19.593	24.186
ENSG00000070182	SPTB	1.8	7.9E-04	1.9E-03	0.701	0.155	0.606	0.879	0.392	0.117	0.265	0.495
ENSG00000079931	MOXD1	1.8	4.5E-10	3.8E-09	5.940	0.340	5.695	6.328	3.316	0.173	3.117	3.431
ENSG00000081019	RSBN1	1.8	2.3E-16	6.5E-15	23.772	0.971	22.661	24.459	13.389	0.324	13.018	13.623
ENSG00000091542	ALKBH5	1.8	1.2E-18	4.9E-17	91.274	2.466	89.513	94.093	51.380	2.154	49.132	53.328
ENSG00000095564	BTAF1	1.8	3.8E-22	2.8E-20	90.478	3.114	87.190	93.384	51.761	1.154	50.430	52.471
ENSG00000099337	KCNK6	1.8	7.6E-14	1.4E-12	12.978	0.233	12.780	13.236	7.387	0.313	7.133	7.737
ENSG00000100564	PIGH	1.8	7.5E-08	4.1E-07	7.901	0.937	7.044	8.902	4.448	0.377	4.100	4.848
ENSG00000101654	RNMT	1.8	2.8E-23	2.5E-21	88.335	1.530	86.570	89.279	48.013	1.085	47.361	49.277
ENSG00000103145	HCFC1R1	1.8	3.5E-14	6.7E-13	64.600	1.573	63.106	66.242	36.302	3.240	32.656	38.852
ENSG00000105963	ADAP1	1.8	1.3E-04	3.8E-04	1.911	0.252	1.647	2.148	1.061	0.066	1.000	1.131
ENSG00000106617	PRKAG2	1.8	4.0E-14	7.6E-13	12.413	0.406	12.000	12.812	6.821	0.226	6.586	7.047
ENSG00000107077	KDM4C	1.8	7.4E-18	2.7E-16	15.377	0.195	15.151	15.490	8.385	0.276	8.084	8.626
ENSG00000107798	LIPA	1.8	6.3E-19	2.7E-17	44.806	1.081	44.133	46.053	24.533	0.932	23.912	25.605
ENSG00000107819	SFXN3	1.8	4.9E-06	1.8E-05	2.653	0.177	2.487	2.839	1.498	0.201	1.267	1.620
ENSG00000107960	STN1	1.8	4.4E-12	5.7E-11	36.812	3.105	33.239	38.850	20.111	1.340	18.832	21.504
ENSG00000108176	DNAJC12	1.8	2.1E-05	7.1E-05	5.254	0.255	5.028	5.531	2.861	0.719	2.386	3.688
ENSG00000108590	MED31	1.8	1.7E-08	1.1E-07	14.499	2.105	12.890	16.881	7.971	0.454	7.651	8.490
ENSG00000114646	CSPG5	1.8	2.2E-08	1.3E-07	5.110	0.356	4.702	5.354	2.784	0.376	2.544	3.217
ENSG00000116183	PAPPA2	1.8	2.6E-05	8.5E-05	0.888	0.131	0.805	1.040	0.487	0.035	0.447	0.515
ENSG00000116678	LEPR	1.8	2.5E-09	1.8E-08	2.417	0.207	2.213	2.626	1.347	0.078	1.261	1.413
ENSG00000118508	RAB32	1.8	1.9E-06	8.0E-06	17.889	2.165	15.422	19.473	9.838	1.294	8.433	10.981
ENSG00000118898	PPL	1.8	3.1E-18	1.2E-16	38.906	1.388	38.016	40.506	22.005	1.415	20.628	23.454
ENSG00000123358	NR4A1	1.8	3.8E-11	4.1E-10	9.490	1.559	7.972	11.087	5.144	0.199	4.979	5.365
ENSG00000128342	LIF	1.8	1.4E-09	1.1E-08	6.761	0.232	6.494	6.912	3.676	0.243	3.401	3.861
ENSG00000129535	NRL	1.8	1.1E-02	2.1E-02	1.122	0.433	0.730	1.587	0.640	0.228	0.404	0.858
ENSG00000133135	RNF128	1.8	7.1E-06	2.6E-05	3.580	0.621	2.968	4.209	1.941	0.168	1.750	2.066
ENSG00000134070	IRAK2	1.8	6.3E-04	1.6E-03	2.219	0.082	2.127	2.284	1.263	0.268	1.049	1.564
ENSG00000134109	EDEM1	1.8	9.0E-16	2.3E-14	20.885	0.862	19.893	21.449	11.809	0.142	11.663	11.947
ENSG00000134574	DDB2	1.8	1.6E-17	5.4E-16	36.931	1.430	35.365	38.170	20.075	0.819	19.214	20.843
ENSG00000134686	PHC2	1.8	5.3E-10	4.4E-09	9.153	1.098	7.917	10.016	5.049	0.492	4.492	5.427
ENSG00000134824	FADS2	1.8	2.8E-24	2.8E-22	265.583	3.542	263.103	269.647	147.062	2.908	145.068	150.399
ENSG00000135318	NT5E	1.8	7.4E-03	1.4E-02	1.522	0.296	1.253	1.839	0.841	0.336	0.481	1.148
ENSG00000137502	RAB30	1.8	1.3E-12	1.8E-11	5.879	0.241	5.600	6.027	3.292	0.291	3.008	3.589
ENSG00000139973	SYT16	1.8	3.2E-08	1.9E-07	1.709	0.184	1.540	1.905	0.942	0.131	0.821	1.082
ENSG00000140043	PTGR2	1.8	8.2E-12	1.0E-10	7.225	0.319	6.887	7.521	4.097	0.336	3.835	4.476
ENSG00000141337	ARSG	1.8	1.7E-06	7.0E-06	2.158	0.052	2.109	2.213	1.214	0.133	1.067	1.325
ENSG00000143195	ILDR2	1.8	8.7E-05	2.6E-04	0.575	0.065	0.500	0.620	0.326	0.084	0.231	0.393
ENSG00000146281	PM20D2	1.8	3.4E-13	5.5E-12	24.152	0.802	23.342	24.946	13.536	1.028	12.512	14.568
ENSG00000149926	FAM57B	1.8	1.3E-09	1.0E-08	8.561	0.937	7.485	9.192	4.830	0.766	4.003	5.515
ENSG00000154553	PDLIM3	1.8	1.3E-08	8.0E-08	3.582	0.231	3.335	3.792	1.992	0.257	1.795	2.282
ENSG00000154556	SORBS2	1.8	5.8E-10	4.8E-09	2.801	0.095	2.740	2.911	1.554	0.073	1.471	1.603
ENSG00000157483	MYO1E	1.8	3.9E-18	1.5E-16	31.882	1.290	30.417	32.852	18.120	0.179	17.917	18.255
ENSG00000159733	ZFYVE28	1.8	5.7E-06	2.1E-05	1.415	0.136	1.271	1.540	0.763	0.113	0.687	0.893
ENSG00000163001	CFAP36	1.8	1.2E-15	3.0E-14	31.447	1.555	29.732	32.763	17.056	1.267	15.644	18.094
ENSG00000163673	DCLK3	1.8	6.9E-05	2.1E-04	1.390	0.128	1.281	1.531	0.780	0.069	0.702	0.832
ENSG00000164266	SPINK1	1.8	4.2E-02	6.8E-02	1.317	0.383	1.043	1.755	0.722	0.336	0.369	1.038
ENSG00000164300	SERINC5	1.8	4.0E-19	1.8E-17	73.902	1.852	72.191	75.868	40.615	1.606	38.814	41.899
ENSG00000164442	CITED2	1.8	4.6E-16	1.2E-14	62.422	0.847	61.642	63.323	34.788	1.452	33.503	36.363
ENSG00000164951	PDP1	1.8	1.2E-11	1.4E-10	22.519	1.254	21.114	23.524	12.871	0.268	12.590	13.123
ENSG00000164889	CCDC171	1.8	8.2E-09	5.4E-08	2.071	0.095	1.963	2.141	1.185	0.055	1.122	1.218
ENSG00000167535	CACNB2	1.8	2.7E-07	1.3E-06	2.231	0.247	1.952	2.417	1.214	0.214	1.002	1.430
ENSG00000168280	KIF5C	1.8	5.4E-22	3.9E-20	64.058	1.485	62.499	65.456	35.476	1.349	34.035	36.708
ENSG00000168939	SPRY3	1.8	3.0E-11	3.2E-10	5.792	0.284	5.613	6.119	3.300	0.214	3.164	3.547
ENSG00000169241	SLC50A1	1.8	2.8E-16	7.8E-15	62.146	1.589	60.338	63.323	35.213	1.551	34.056	36.975
ENSG00000169291	SHE	1.8	2.0E-03	4.5E-03	0.572	0.165	0.384	0.692	0.319	0.052	0.258	0.350
ENSG00000169299	PGM2	1.8	1.4E-18	5.7E-17	59.624	1.456	58.460	61.256	34.009	0.633	33.281	34.427
ENSG00000169894	MUC3A	1.8	3.4E-13	5.4E-12	6.924	0.272	6.636	7.176	3.803	0.381	3.365	4.052
ENSG00000170017	ALCAM	1.8	1.7E-16	4.9E-15	36.556	2.271	34.443	38.958	20.072	1.086	19.109	21.248
ENSG00000170222	ADPRM	1.8	1.6E-08	1.0E-07	14.682	0.422	14.328	15.149	8.060	0.395	7.660	8.450
ENSG00000170485	NPAS2	1.8	4.8E-03	9.7E-03	0.441	0.095	0.346	0.535	0.241	0.034	0.218	0.280
ENSG00000171004	HS6ST2	1.8	1.6E-13	2.8E-12	17.534	1.350	16.015	18.594	9.821	0.406	9.542	10.287
ENSG00000172071	EIF2AK3	1.8	1.3E-18	5.3E-17	32.637	0.478	32.315	33.187	17.790	0.494	17.340	18.318
ENSG00000172461	FUT9	1.8	3.8E-16	1.0E-14	9.328	0.231	9.136	9.584	5.086	0.243	4.851	5.335
ENSG00000173846	PLK3	1.8	4.0E-08	2.3E-07	6.343	0.280	6.034	6.579	3.578	0.109	3.453	3.645
ENSG00000174514	MFSD4A	1.8	2.5E-02	4.3E-02	0.471	0.174	0.368	0.673	0.265	0.113	0.141	0.362
ENSG00000175105	ZNF654	1.8	1.7E-11	2.0E-10	7.176	0.576	6.778	7.836	3.980	0.340	3.587	4.247
ENSG00000175155	YPEL2	1.8	5.4E-10	4.5E-09	3.898	0.306	3.576	4.185	2.175	0.155	1.998	2.289
ENSG00000175395	ZNF25	1.8	5.2E-05	1.6E-04	1.765	0.276	1.491	2.044	0.971	0.063	0.900	1.020
ENSG00000176170	SPHK1	1.8	4.0E-04	1.0E-03	1.566	0.363	1.153	1.838	0.852	0.285	0.525	1.044
ENSG00000177106	EPS8L2	1.8	2.1E-13	3.5E-12	13.487	0.520	13.037	14.056	7.620	0.746	6.767	8.144
ENSG00000177337	DLGAP1-AS1	1.8	1.7E-05	5.7E-05	5.809	0.959	4.704	6.417	3.227	0.516	2.672	3.692
ENSG00000179075	GRAMD1C	1.8	7.1E-06	2.6E-05	1.823	0.059	1.778	1.890	1.029	0.174	0.882	1.222
ENSG00000179029	TMEM107	1.8	2.8E-07	1.4E-06	5.662	0.819	4.977	6.569	3.118	0.366	2.845	3.533
ENSG00000179046	TRIML2	1.8	1.1E-04	3.2E-04	56.069	4.666	51.568	60.883	30.974	1.401	29.380	32.012
ENSG00000182718	ANXA2	1.8	2.0E-20	1.1E-18	43.270	0.211	43.026	43.397	23.878	0.761	23.041	24.530
ENSG00000182732	RGS6	1.8	1.4E-03	3.3E-03	0.527	0.098	0.445	0.636	0.295	0.053	0.234	0.336
ENSG00000183258	DDX41	1.8	3.8E-20	1.9E-18	77.133	2.500	74.452	79.400	43.339	2.302	40.681	44.703
ENSG00000183421	RIPK4	1.8	1.9E-08	1.2E-07	7.709	0.518	7.298	8.291	4.312	0.741	3.464	4.835
ENSG00000187902	SHISA7	1.8	1.4E-03	3.2E-03	0.683	0.060	0.614	0.725	0.372	0.063	0.300	0.413
ENSG00000188910	GJB3	1.8	7.2E-03	1.4E-02	1.343	0.028	1.310	1.360	0.754	0.240	0.585	1.028
ENSG00000196187	TMEM63A	1.8	5.4E-18	2.0E-16	23.832	1.050	23.050	25.025	13.599	0.532	13.068	14.132
ENSG00000196353	CPNE4	1.8	6.4E-06	2.4E-05	1.940	0.116	1.836	2.065	1.102	0.069	1.022	1.146
ENSG00000196684	HSH2D	1.8	1.6E-04	4.6E-04	2.246	0.392	1.799	2.532	1.227	0.329	0.993	1.603
ENSG00000197191	CYSRT1	1.8	1.9E-02	3.4E-02	2.013	0.324	1.644	2.253	1.104	0.399	0.650	1.400
ENSG00000197226	TBC1D9B	1.8	2.5E-19	1.1E-17	31.069	0.523	30.715	31.669	17.360	0.952	16.412	18.316
ENSG00000198142	SOWAHC	1.8	3.1E-18	1.2E-16	39.546	1.692	37.618	40.780	22.115	0.162	21.957	22.280
ENSG00000198265	HELZ	1.8	2.2E-20	1.2E-18	23.946	0.488	23.383	24.249	13.414	0.355	13.004	13.633
ENSG00000198626	RYR2	1.8	8.0E-14	1.4E-12	4.301	0.296	3.959	4.474	2.361	0.090	2.262	2.436
ENSG00000201785	SNORD117	1.8	5.0E-02	8.0E-02	20.837	2.436	18.709	23.494	11.529	4.000	9.180	16.148
ENSG00000204228	HSD17B8	1.8	9.1E-05	2.7E-04	7.047	1.492	5.950	8.733	3.839	0.634	3.377	4.562
ENSG00000204385	SLC44A4	1.8	4.4E-05	1.4E-04	3.413	0.520	2.840	3.854	1.934	0.323	1.741	2.307
ENSG00000205269	TMEM170B	1.8	6.8E-15	1.5E-13	12.071	0.320	11.740	12.379	6.773	0.333	6.409	7.062
ENSG00000205560	CPT1B	1.8	9.1E-03	1.7E-02	0.783	0.052	0.728	0.831	0.441	0.193	0.272	0.651
ENSG00000206262	FOXL2NB	1.8	3.3E-10	2.9E-09	10.283	1.437	8.821	11.693	5.612	0.342	5.291	5.972
ENSG00000211451	GNRHR2	1.8	6.0E-05	1.8E-04	3.207	0.462	2.935	3.741	1.769	0.209	1.627	2.009
ENSG00000213025	COX20P1	1.8	2.1E-09	1.5E-08	79.191	5.137	74.877	84.874	43.342	4.316	38.770	47.345
ENSG00000214021	TTLL3	1.8	1.5E-06	6.4E-06	1.202	0.017	1.191	1.222	0.681	0.045	0.631	0.719
ENSG00000214575	CPEB1	1.8	6.8E-07	3.0E-06	1.953	0.231	1.714	2.173	1.094	0.129	0.946	1.181
ENSG00000224934		1.8	5.9E-05	1.8E-04	2.847	0.490	2.523	3.411	1.572	0.264	1.351	1.865
ENSG00000225285	LINC01770	1.8	5.1E-03	1.0E-02	4.325	1.473	3.175	5.985	2.334	0.485	1.970	2.884
ENSG00000226287	TMEM191A	1.8	1.3E-06	5.4E-06	5.176	0.226	4.917	5.331	2.803	0.472	2.276	3.190
ENSG00000226318	RPS3AP38	1.8	7.8E-04	1.9E-03	6.362	0.781	5.531	7.081	3.466	0.875	2.502	4.211
ENSG00000227354	RBM26-AS1	1.8	4.0E-04	1.0E-03	1.885	0.460	1.359	2.209	1.060	0.249	0.895	1.347
ENSG00000228232	GAPDHP1	1.8	2.9E-18	1.1E-16	528.438	32.601	501.333	564.614	290.210	5.810	285.051	296.503
ENSG00000228393	LINC01004	1.8	4.3E-04	1.1E-03	4.893	0.147	4.736	5.029	2.655	0.798	1.740	3.211
ENSG00000231203	KRT8P10	1.8	4.3E-08	2.5E-07	16.622	1.613	15.352	18.436	9.273	1.041	8.375	10.413
ENSG00000232936		1.8	1.7E-02	3.0E-02	3.499	0.659	2.951	4.230	1.915	0.369	1.578	2.309
ENSG00000233056	ERVH48-1	1.8	2.5E-11	2.8E-10	22.182	1.916	20.913	24.385	12.473	0.178	12.318	12.667
ENSG00000233057	EEF1A1P14	1.8	5.3E-03	1.1E-02	3.180	1.431	2.338	4.832	1.760	0.357	1.357	2.035
ENSG00000234028		1.8	8.3E-05	2.5E-04	2.173	0.267	1.874	2.388	1.244	0.272	1.011	1.543
ENSG00000234678	ELF3-AS1	1.8	1.5E-02	2.8E-02	3.203	0.926	2.278	4.131	1.796	0.867	1.157	2.783
ENSG00000236735	RPL31P63	1.8	7.4E-04	1.8E-03	19.877	1.475	18.592	21.487	11.149	3.584	8.425	15.209
ENSG00000239198	RPL5P22	1.8	1.7E-02	3.1E-02	3.240	1.047	2.356	4.396	1.852	0.350	1.607	2.254
ENSG00000239467		1.8	5.1E-08	2.8E-07	23.453	0.352	23.144	23.836	12.909	2.077	10.690	14.807
ENSG00000241860		1.8	6.8E-05	2.1E-04	1.158	0.121	1.069	1.296	0.646	0.052	0.605	0.705
ENSG00000243709	LEFTY1	1.8	1.6E-10	1.4E-09	28.492	1.361	27.468	30.037	16.122	1.279	14.647	16.916
ENSG00000243978	RTL9	1.8	1.8E-03	4.1E-03	0.923	0.087	0.823	0.977	0.510	0.214	0.305	0.733
ENSG00000245532	NEAT1	1.8	6.8E-03	1.3E-02	1.474	0.135	1.383	1.630	0.811	0.071	0.729	0.859
ENSG00000246882		1.8	1.2E-01	1.7E-01	0.691	0.253	0.444	0.950	0.376	0.320	0.191	0.746
ENSG00000248626	GAPDHP40	1.8	1.0E-03	2.5E-03	6.759	0.452	6.360	7.249	3.755	0.888	2.777	4.541
ENSG00000249859	PVT1	1.8	1.5E-11	1.8E-10	8.494	0.406	8.024	8.739	4.609	0.141	4.454	4.730
ENSG00000250221	KRT8P32	1.8	4.9E-05	1.5E-04	6.615	1.278	5.742	8.082	3.767	0.182	3.612	3.968
ENSG00000251017		1.8	3.0E-02	5.1E-02	3.398	1.224	2.035	4.404	1.944	0.352	1.545	2.212
ENSG00000254087	LYN	1.8	2.1E-17	7.1E-16	37.778	1.785	35.902	39.454	21.337	0.457	20.914	21.822
ENSG00000255471		1.8	3.9E-03	8.0E-03	2.792	0.336	2.408	3.035	1.524	0.350	1.120	1.731
ENSG00000256813		1.8	3.9E-03	8.1E-03	1.940	0.436	1.482	2.352	1.089	0.524	0.784	1.695
ENSG00000257150	PGAM1P5	1.8	1.5E-04	4.2E-04	3.071	0.480	2.674	3.604	1.723	0.308	1.534	2.079
ENSG00000258315	C17orf49	1.8	1.8E-02	3.1E-02	1.014	0.240	0.757	1.233	0.565	0.295	0.363	0.904
ENSG00000258649		1.8	1.5E-13	2.6E-12	139.838	2.194	137.312	141.274	79.916	4.194	75.082	82.580
ENSG00000259577	CERNA1	1.8	9.5E-03	1.8E-02	1.614	0.416	1.326	2.091	0.882	0.475	0.333	1.171
ENSG00000260822		1.8	3.5E-16	9.6E-15	27.623	0.820	26.766	28.401	15.583	1.010	14.570	16.590
ENSG00000261120		1.8	8.1E-03	1.6E-02	7.529	2.715	5.763	10.655	4.197	0.821	3.328	4.959
ENSG00000261135		1.8	4.6E-02	7.4E-02	1.467	0.276	1.280	1.784	0.810	0.245	0.667	1.093
ENSG00000261324		1.8	6.1E-05	1.9E-04	2.259	0.422	1.941	2.737	1.278	0.096	1.191	1.382
ENSG00000261596		1.8	6.8E-02	1.0E-01	1.852	0.269	1.546	2.051	1.005	0.155	0.844	1.153
ENSG00000266904	LINC00662	1.8	8.5E-04	2.0E-03	1.334	0.234	1.068	1.509	0.759	0.104	0.677	0.876
ENSG00000267573	KRT8P5	1.8	9.2E-04	2.2E-03	3.378	0.211	3.158	3.579	1.857	0.360	1.473	2.187
ENSG00000268723		1.8	1.0E-04	3.1E-04	19.348	2.192	16.957	21.262	10.690	0.561	10.099	11.214
ENSG00000269397		1.8	6.1E-05	1.9E-04	4.847	0.787	3.981	5.519	2.624	0.147	2.455	2.718
ENSG00000270362	HMGN3-AS1	1.8	3.0E-04	7.9E-04	2.382	0.555	1.834	2.944	1.368	0.180	1.250	1.575
ENSG00000271601	LIX1L	1.8	8.6E-05	2.6E-04	2.595	0.545	2.254	3.223	1.479	0.235	1.230	1.698
ENSG00000272677		1.8	2.3E-04	6.2E-04	6.539	0.686	5.807	7.166	3.574	0.176	3.414	3.763
ENSG00000272973		1.8	1.6E-02	2.8E-02	3.814	0.854	3.178	4.784	2.115	0.350	1.835	2.507
ENSG00000273230		1.8	4.8E-03	9.8E-03	1.135	0.212	0.893	1.287	0.627	0.025	0.600	0.650
ENSG00000273284		1.8	4.0E-05	1.3E-04	8.969	0.506	8.577	9.541	4.943	1.631	3.925	6.824
ENSG00000276386	CNTNAP3P2	1.8	1.4E-03	3.1E-03	1.043	0.045	1.008	1.094	0.565	0.164	0.464	0.753
ENSG00000278390		1.8	2.8E-03	6.1E-03	1.535	0.258	1.250	1.752	0.863	0.170	0.716	1.049
ENSG00000278558	TMEM191B	1.8	2.5E-04	6.8E-04	3.191	0.196	3.062	3.416	1.819	0.281	1.521	2.078
ENSG00000279022		1.8	3.8E-02	6.3E-02	4.406	1.043	3.210	5.130	2.407	0.677	1.892	3.174
ENSG00000279048		1.8	3.9E-02	6.4E-02	0.696	0.123	0.588	0.829	0.382	0.144	0.232	0.520
ENSG00000279267		1.8	1.0E-01	1.5E-01	0.553	0.078	0.502	0.643	0.313	0.095	0.245	0.421
ENSG00000279312		1.8	1.7E-03	3.9E-03	2.207	0.410	1.865	2.661	1.239	0.301	0.894	1.449
ENSG00000280164		1.8	7.2E-04	1.8E-03	5.216	0.803	4.602	6.125	2.958	0.521	2.621	3.557
ENSG00000280511		1.8	1.6E-14	3.2E-13	247.680	4.372	242.734	251.030	135.137	13.862	119.157	143.925
ENSG00000281026	N4BP2L2-IT2	1.8	1.0E-03	2.5E-03	1.240	0.484	0.960	1.799	0.683	0.121	0.558	0.800
ENSG00000000005	TNMD	1.9	1.6E-05	5.6E-05	5.023	0.220	4.857	5.273	2.586	0.289	2.256	2.795
ENSG00000010818	HIVEP2	1.9	5.2E-17	1.6E-15	9.728	0.451	9.264	10.166	5.046	0.164	4.930	5.234
ENSG00000026508	CD44	1.9	6.5E-08	3.6E-07	4.453	0.495	4.043	5.003	2.345	0.221	2.111	2.549
ENSG00000054967	RELT	1.9	4.8E-11	5.0E-10	5.605	0.521	5.238	6.201	2.935	0.223	2.713	3.160
ENSG00000070540	WIPI1	1.9	4.5E-16	1.2E-14	30.625	0.878	29.633	31.300	15.739	1.134	14.494	16.715
ENSG00000072274	TFRC	1.9	1.4E-17	4.7E-16	189.673	3.466	185.923	192.758	102.137	3.404	98.637	105.435
ENSG00000086475	SEPHS1	1.9	5.2E-26	7.3E-24	1269.691	20.795	1245.687	1282.224	675.166	8.172	665.999	681.687
ENSG00000095539	SEMA4G	1.9	9.9E-12	1.2E-10	7.597	0.219	7.463	7.850	4.027	0.440	3.537	4.389
ENSG00000100078	PLA2G3	1.9	2.1E-18	8.4E-17	65.354	2.630	62.552	67.768	34.408	0.594	33.780	34.961
ENSG00000100234	TIMP3	1.9	2.8E-10	2.5E-09	8.615	0.771	7.753	9.239	4.503	0.224	4.246	4.651
ENSG00000104689	TNFRSF10A	1.9	1.8E-12	2.5E-11	17.707	0.115	17.574	17.779	9.465	0.774	8.641	10.177
ENSG00000104892	KLC3	1.9	4.8E-08	2.7E-07	4.629	0.090	4.531	4.708	2.393	0.148	2.232	2.523
ENSG00000105246	EBI3	1.9	2.1E-02	3.7E-02	1.298	0.248	1.011	1.443	0.683	0.217	0.438	0.850
ENSG00000106714	CNTNAP3	1.9	1.1E-10	1.0E-09	3.105	0.344	2.765	3.453	1.638	0.101	1.577	1.754
ENSG00000108375	RNF43	1.9	8.4E-06	3.0E-05	1.427	0.166	1.276	1.604	0.741	0.153	0.651	0.918
ENSG00000108861	DUSP3	1.9	1.5E-20	8.3E-19	46.297	0.487	45.891	46.838	23.880	0.971	22.770	24.573
ENSG00000109084	TMEM97	1.9	4.3E-23	3.6E-21	200.971	7.169	195.810	209.156	103.923	1.877	101.935	105.664
ENSG00000113916	BCL6	1.9	4.2E-09	2.9E-08	3.486	0.335	3.280	3.873	1.798	0.293	1.623	2.136
ENSG00000114378	HYAL1	1.9	3.0E-08	1.8E-07	6.829	0.829	5.947	7.593	3.666	0.439	3.256	4.130
ENSG00000115138	POMC	1.9	7.1E-03	1.4E-02	2.578	0.767	1.849	3.377	1.357	0.286	1.064	1.635
ENSG00000117868	ESYT2	1.9	1.2E-18	5.1E-17	45.520	3.018	42.447	48.481	23.964	0.372	23.537	24.224
ENSG00000118971	CCND2	1.9	1.8E-21	1.2E-19	87.063	1.480	85.661	88.610	47.175	1.110	46.242	48.403
ENSG00000120889	TNFRSF10B	1.9	3.9E-16	1.0E-14	109.053	5.422	103.208	113.919	57.861	0.836	56.972	58.630
ENSG00000124191	TOX2	1.9	2.0E-05	6.8E-05	3.374	0.184	3.161	3.485	1.789	0.621	1.134	2.369
ENSG00000124839	RAB17	1.9	2.3E-04	6.2E-04	145.721	5.036	141.150	151.120	78.849	1.338	78.036	80.393
ENSG00000125384	PTGER2	1.9	1.4E-03	3.3E-03	2.290	0.827	1.380	2.996	1.176	0.351	0.796	1.489
ENSG00000125746	EML2	1.9	3.6E-13	5.7E-12	8.323	0.901	7.383	9.178	4.311	0.148	4.223	4.482
ENSG00000126001	CEP250	1.9	2.5E-19	1.1E-17	17.469	0.343	17.135	17.821	9.054	0.854	8.247	9.875
ENSG00000126822	PLEKHG3	1.9	2.2E-20	1.2E-18	26.340	0.455	25.971	26.849	14.058	0.364	13.687	14.415
ENSG00000130045	NXNL2	1.9	2.6E-03	5.7E-03	1.498	0.453	0.990	1.861	0.777	0.121	0.691	0.915
ENSG00000130876	SLC7A10	1.9	6.8E-04	1.7E-03	1.765	0.155	1.588	1.878	0.923	0.296	0.629	1.220
ENSG00000130956	HABP4	1.9	5.6E-15	1.3E-13	18.771	0.666	18.054	19.370	9.860	0.838	8.954	10.607
ENSG00000132432	SEC61G	1.9	2.9E-21	1.8E-19	209.425	2.054	207.173	211.196	107.845	8.214	99.273	115.648
ENSG00000132718	SYT11	1.9	2.4E-17	7.9E-16	28.878	2.240	26.294	30.265	15.142	0.706	14.502	15.900
ENSG00000135549	PKIB	1.9	4.4E-15	9.9E-14	30.988	0.648	30.600	31.735	16.506	1.254	15.098	17.502
ENSG00000135643	KCNMB4	1.9	1.0E-17	3.6E-16	27.619	1.077	26.664	28.786	14.330	0.844	13.373	14.971
ENSG00000136826	KLF4	1.9	2.9E-05	9.5E-05	2.151	0.270	1.904	2.440	1.127	0.113	1.006	1.230
ENSG00000137962	ARHGAP29	1.9	1.4E-11	1.6E-10	3.569	0.216	3.356	3.788	1.855	0.118	1.766	1.989
ENSG00000139433	GLTP	1.9	1.6E-17	5.4E-16	64.573	0.369	64.250	64.976	34.027	2.756	31.710	37.075
ENSG00000139890	REM2	1.9	4.5E-06	1.7E-05	4.657	0.668	4.195	5.422	2.446	0.383	2.074	2.840
ENSG00000140563	MCTP2	1.9	7.2E-09	4.8E-08	1.685	0.137	1.534	1.801	0.893	0.119	0.757	0.979
ENSG00000143320	CRABP2	1.9	1.1E-12	1.6E-11	158.151	9.641	148.004	167.189	84.547	2.720	82.183	87.520
ENSG00000143384	MCL1	1.9	2.2E-25	2.7E-23	499.312	10.908	486.871	507.234	260.801	6.111	254.599	266.817
ENSG00000147437	GNRH1	1.9	2.1E-05	7.2E-05	4.673	0.206	4.514	4.906	2.447	0.645	2.061	3.192
ENSG00000148798	INA	1.9	6.0E-17	1.8E-15	34.474	0.790	33.577	35.066	18.136	0.642	17.423	18.667
ENSG00000151229	SLC2A13	1.9	4.1E-13	6.4E-12	9.781	0.336	9.405	10.053	5.224	0.286	4.896	5.419
ENSG00000151490	PTPRO	1.9	1.5E-06	6.4E-06	1.806	0.173	1.607	1.921	0.967	0.113	0.889	1.096
ENSG00000151914	DST	1.9	4.8E-06	1.8E-05	37.577	3.905	33.202	40.711	20.266	1.375	19.144	21.801
ENSG00000151967	SCHIP1	1.9	8.9E-06	3.2E-05	1.446	0.217	1.246	1.677	0.753	0.193	0.535	0.901
ENSG00000152104	PTPN14	1.9	7.3E-20	3.6E-18	40.235	2.033	37.897	41.579	20.997	0.624	20.310	21.527
ENSG00000153558	FBXL2	1.9	3.7E-08	2.1E-07	2.549	0.220	2.375	2.796	1.352	0.146	1.203	1.495
ENSG00000154511	FAM69A	1.9	2.0E-12	2.7E-11	14.311	0.442	13.832	14.704	7.670	0.450	7.367	8.187
ENSG00000154760	SLFN13	1.9	7.7E-15	1.7E-13	12.686	1.211	11.294	13.502	6.758	0.296	6.490	7.076
ENSG00000157542	KCNJ6	1.9	1.7E-05	5.8E-05	0.412	0.031	0.391	0.448	0.215	0.024	0.194	0.242
ENSG00000160961	ZNF333	1.9	1.6E-10	1.5E-09	3.419	0.341	3.061	3.740	1.806	0.060	1.744	1.865
ENSG00000161905	ALOX15	1.9	2.4E-03	5.2E-03	1.053	0.028	1.020	1.073	0.550	0.065	0.492	0.621

ENSG00000162873	KLHDC8A	1.9	4.4E-06	1.7E-05	3.517	0.303	3.201	3.805	1.860	0.387	1.425	2.166
ENSG00000164695	CHMP4C	1.9	7.0E-11	6.9E-10	16.088	1.540	14.310	16.992	8.406	0.280	8.137	8.696
ENSG00000166171	DPCD	1.9	3.2E-14	6.2E-13	37.945	2.097	35.525	39.230	19.781	1.196	18.891	21.140
ENSG00000167601	AXL	1.9	3.4E-17	1.1E-15	32.131	2.626	29.188	34.234	16.874	0.976	16.340	17.885
ENSG00000168490	PHYHIP	1.9	1.5E-08	9.3E-08	6.819	0.413	6.479	7.279	3.578	0.772	2.981	4.450
[illegible]	[illegible]	[illegible]	[illegible]	[illegible]	[illegible]	[illegible]	[illegible]	[illegible]	[illegible]	[illegible]	[illegible]	[illegible]

ENSG00000162618	ADGRL4	2.7	2.8E-03	6.0E-03	4.956	0.876	4.198	5.915	1.814	0.093	1.706	1.874
ENSG00000182685	BRICD5	2.7	1.4E-07	7.1E-07	3.994	0.767	3.112	4.505	1.485	0.380	1.080	1.833
ENSG00000110680	CALCA	2.7	2.1E-04	5.9E-04	1.457	0.321	1.088	1.678	0.543	0.029	0.525	0.577
ENSG00000102384	CENPI	2.7	6.2E-26	8.5E-24	55.751	0.552	55.260	56.348	20.464	0.668	19.914	21.207
ENSG00000144810	COL8A1	2.7	5.0E-09	3.4E-08	1.299	0.225	1.148	1.557	0.482	0.030	0.448	0.507
ENSG00000074800	ENO1	2.7	7.0E-31	2.3E-28	3529.629	67.382	3469.354	3602.376	1310.332	34.862	1275.246	1344.967
ENSG00000169710	FASN	2.7	7.2E-09	4.8E-08	734.558	30.855	700.981	761.665	272.096	18.232	255.799	291.787
ENSG00000163285	GABRG1	2.7	2.1E-08	1.3E-07	1.512	0.226	1.251	1.651	0.553	0.024	0.535	0.580
ENSG00000216624	GAPDHP72	2.7	3.3E-07	1.6E-06	2.990	0.330	2.759	3.368	1.119	0.092	1.063	1.226
ENSG00000158555	GDPD5	2.7	3.0E-17	9.7E-16	5.970	0.133	5.856	6.116	2.186	0.086	2.109	2.280
ENSG00000147246	HTR2C	2.7	3.9E-14	7.5E-13	6.295	0.639	5.770	7.006	2.330	0.229	2.164	2.591
ENSG00000105376	ICAM5	2.7	9.6E-08	5.1E-07	2.147	0.289	1.820	2.369	0.803	0.236	0.576	1.047
ENSG00000169991	IFFO2	2.7	5.8E-21	3.4E-19	20.053	0.811	19.118	20.539	7.466	0.636	6.840	8.111
ENSG00000135070	ISCA1	2.7	6.3E-28	1.2E-25	150.019	0.836	149.197	150.867	55.277	3.029	51.941	57.856
ENSG00000242759	LINC00882	2.7	1.2E-11	1.5E-10	5.494	0.552	5.091	6.123	2.036	0.278	1.835	2.353
ENSG00000185847	LINC01405	2.7	2.5E-17	8.3E-16	56.399	2.894	53.444	59.229	21.302	2.210	18.752	22.667
ENSG00000139793	MBNL2	2.7	4.8E-12	6.2E-11	4.376	0.165	4.201	4.529	1.652	0.337	1.395	2.034
ENSG00000228526	MIR34AHG	2.7	9.9E-10	7.9E-09	13.254	2.099	10.907	14.950	4.873	0.333	4.564	5.226
ENSG00000136997	MYC	2.7	3.4E-26	4.9E-24	195.930	8.844	188.903	205.861	72.274	3.866	67.819	74.747
ENSG00000116774	OLFML3	2.7	3.8E-26	5.5E-24	111.196	2.740	108.064	113.149	41.758	0.810	40.901	42.512
ENSG00000102230	PCYT1B	2.7	1.5E-27	2.6E-25	91.548	0.531	90.970	92.015	33.530	0.786	32.953	34.425
ENSG00000184381	PLA2G6	2.7	3.0E-15	7.0E-14	4.931	0.562	4.408	5.524	1.830	0.100	1.715	1.902
ENSG00000146278	PNRC1	2.7	7.8E-21	4.5E-19	36.179	1.340	35.046	37.659	13.314	0.948	12.260	14.097
ENSG00000087074	PPP1R15A	2.7	4.3E-21	2.6E-19	32.468	0.709	31.674	33.036	12.218	1.081	11.110	13.270
ENSG00000112183	RBM24	2.7	8.3E-03	1.6E-02	0.496	0.189	0.289	0.660	0.176	0.143	0.040	0.324
ENSG00000160326	SLC2A6	2.7	5.6E-20	2.8E-18	31.683	2.265	30.347	34.298	11.846	0.244	11.637	12.114
ENSG00000147003	TMEM27	2.7	3.1E-10	2.7E-09	11.388	2.432	8.846	13.694	4.240	0.187	4.116	4.455
ENSG00000173530	TNFRSF10D	2.7	2.0E-21	1.3E-19	41.674	1.356	40.121	42.623	15.447	0.894	14.415	15.990
ENSG00000235501		2.7	2.5E-17	8.2E-16	43.935	1.217	42.712	45.146	16.022	1.398	14.418	16.978
ENSG00000238178		2.7	6.1E-04	1.5E-03	2.277	0.600	1.750	2.929	0.834	0.326	0.458	1.038
ENSG00000245904		2.7	7.6E-05	2.3E-04	0.434	0.051	0.386	0.487	0.159	0.072	0.108	0.241
ENSG00000248161		2.7	3.5E-05	1.1E-04	4.587	1.248	3.273	5.757	1.688	0.762	0.825	2.268
ENSG00000259820		2.7	5.1E-21	3.0E-19	14.045	0.605	13.602	14.735	5.145	0.317	4.879	5.495
ENSG00000260196		2.7	7.1E-06	2.6E-05	1.578	0.272	1.338	1.874	0.577	0.089	0.485	0.662
ENSG00000267519		2.7	4.8E-07	2.2E-06	4.030	1.130	2.725	4.698	1.462	0.108	1.384	1.585
ENSG00000102001	CACNA1F	2.8	6.2E-07	2.8E-06	0.775	0.103	0.656	0.847	0.274	0.029	0.244	0.302
ENSG00000004948	CALCR	2.8	5.3E-11	5.4E-10	4.529	0.146	4.432	4.697	1.605	0.409	1.316	2.074
ENSG00000148600	CDHR1	2.8	1.1E-20	5.9E-19	11.793	0.463	11.425	12.313	4.202	0.069	4.136	4.273
ENSG00000159208	CIART	2.8	4.6E-15	1.0E-13	11.896	1.171	11.018	13.225	4.272	0.295	3.963	4.552
ENSG00000111799	COL12A1	2.8	4.9E-19	2.2E-17	4.801	0.347	4.403	5.033	1.728	0.014	1.712	1.737
ENSG00000135678	CPM	2.8	1.6E-06	6.8E-06	0.683	0.132	0.574	0.830	0.246	0.019	0.224	0.260
ENSG00000241095	CYP51A1P1	2.8	7.5E-19	3.2E-17	43.190	1.522	41.451	44.280	15.394	1.368	14.198	16.886
ENSG00000105339	DENND3	2.8	2.7E-16	7.6E-15	3.093	0.038	3.054	3.131	1.102	0.080	1.032	1.188
ENSG00000069696	DRD4	2.8	2.5E-03	5.5E-03	1.181	0.626	0.674	1.881	0.410	0.242	0.185	0.666
ENSG00000142627	EPHA2	2.8	9.6E-14	1.7E-12	61.207	3.607	57.059	63.610	21.892	1.230	20.690	23.148
ENSG00000225828	FAM229A	2.8	1.0E-06	4.4E-06	3.247	0.694	2.549	3.937	1.141	0.486	0.736	1.680
ENSG00000174721	FGFBP3	2.8	4.6E-31	1.6E-28	420.039	7.720	411.314	425.985	149.094	3.537	145.149	151.983
ENSG00000121743	GJA3	2.8	5.4E-06	2.0E-05	0.733	0.020	0.709	0.748	0.257	0.040	0.216	0.295
ENSG00000013588	GPRC5A	2.8	1.2E-05	4.1E-05	0.546	0.093	0.442	0.622	0.197	0.044	0.149	0.233
ENSG00000127124	HIVEP3	2.8	4.0E-05	1.3E-04	0.304	0.088	0.206	0.377	0.108	0.046	0.067	0.158
ENSG00000149084	HSD17B12	2.8	2.1E-30	6.2E-28	153.490	1.693	152.065	155.361	54.782	3.003	51.506	57.404
ENSG00000105419	MEIS3	2.8	2.5E-18	9.9E-17	12.283	1.704	10.865	14.208	4.362	0.275	4.117	4.659
ENSG00000123342	MMP19	2.8	1.3E-08	8.0E-08	2.043	0.060	1.980	2.100	0.743	0.019	0.722	0.759
ENSG00000117115	PADI2	2.8	4.1E-13	6.3E-12	5.126	0.400	4.666	5.391	1.809	0.187	1.611	1.983
ENSG00000166473	PKD1L2	2.8	1.7E-13	2.8E-12	1.551	0.151	1.449	1.724	0.560	0.108	0.450	0.666
ENSG00000106772	PRUNE2	2.8	2.0E-25	2.6E-23	22.400	0.966	21.474	23.402	8.056	0.168	7.863	8.169
ENSG00000123892	RAB38	2.8	1.3E-21	8.3E-20	78.359	2.032	77.116	80.704	27.936	0.663	27.272	28.598
ENSG00000105329	TGFB1	2.8	1.9E-14	3.8E-13	12.216	1.889	10.495	14.237	4.387	0.680	3.734	5.091
ENSG00000111669	TPI1	2.8	1.3E-30	4.2E-28	1224.068	34.077	1191.201	1259.239	439.592	6.589	432.792	445.946
ENSG00000007255	TRAPPC6A	2.8	1.1E-21	7.7E-20	103.373	5.866	96.620	107.200	37.370	1.324	35.954	38.576
ENSG00000204644	ZFP57	2.8	1.8E-05	6.1E-05	2.174	0.470	1.654	2.568	0.773	0.210	0.650	1.015
ENSG00000177822		2.8	3.6E-20	1.9E-18	12.324	0.071	12.257	12.398	4.335	0.159	4.152	4.432
ENSG00000272341		2.8	8.0E-07	3.6E-06	2.440	0.419	1.961	2.737	0.875	0.042	0.831	0.913
ENSG00000272812		2.8	8.0E-05	2.4E-04	5.444	0.425	5.090	5.916	1.934	0.218	1.704	2.138
ENSG00000272851		2.8	2.7E-05	8.9E-05	6.593	0.303	6.303	6.907	2.296	0.501	1.745	2.724
ENSG00000276564		2.8	4.4E-05	1.4E-04	1.546	0.347	1.156	1.824	0.538	0.213	0.293	0.681
ENSG00000143632	ACTA1	2.9	6.1E-08	3.4E-07	5.708	0.683	5.041	6.406	1.974	0.741	1.495	2.828
ENSG00000164236	ANKRD33B	2.9	1.2E-25	1.6E-23	20.208	0.527	19.611	20.607	7.019	0.318	6.654	7.234
ENSG00000180347	CCDC129	2.9	7.5E-05	2.3E-04	0.833	0.062	0.762	0.873	0.288	0.120	0.212	0.427
ENSG00000129521	EGLN3	2.9	1.2E-24	1.3E-22	22.370	0.364	22.007	22.736	7.789	0.293	7.560	8.120
ENSG00000175318	GRAMD2	2.9	9.3E-11	9.0E-10	2.867	0.199	2.662	3.060	0.995	0.238	0.840	1.269
ENSG00000147050	KDM6A	2.9	3.0E-24	3.0E-22	197.095	3.809	194.867	201.492	68.515	2.786	66.452	71.685
ENSG00000234787	LINC00458	2.9	2.9E-05	9.6E-05	72.701	0.893	71.901	73.665	24.780	0.889	23.769	25.434
ENSG00000167011	NAT16	2.9	2.5E-03	5.5E-03	0.401	0.157	0.220	0.503	0.132	0.075	0.073	0.217
ENSG00000196408	NOXO1	2.9	1.9E-05	6.4E-05	1.610	0.325	1.353	1.976	0.558	0.139	0.453	0.716
ENSG00000163545	NUAK2	2.9	1.1E-11	1.4E-10	22.332	2.071	20.000	23.953	7.769	0.725	6.935	8.253
ENSG00000170909	OSCAR	2.9	1.9E-07	9.7E-07	3.416	0.667	2.661	3.925	1.151	0.384	0.742	1.505
ENSG00000137709	POU2F3	2.9	1.4E-06	5.8E-06	0.703	0.146	0.535	0.806	0.237	0.008	0.231	0.246
ENSG00000054356	PTPRN	2.9	2.2E-12	3.0E-11	3.353	0.535	3.008	3.969	1.167	0.110	1.040	1.234
ENSG00000105784	RUNDC3B	2.9	3.4E-07	1.6E-06	1.596	0.265	1.344	1.872	0.560	0.158	0.448	0.741
ENSG00000109929	SC5D	2.9	7.1E-13	1.1E-11	174.728	4.205	169.906	177.630	60.537	3.321	58.259	64.348
ENSG00000226415	TPI1P1	2.9	4.8E-30	1.3E-27	1473.685	28.973	1453.622	1506.901	515.205	15.076	499.246	529.207
ENSG00000184470	TXNRD2	2.9	5.8E-23	4.8E-21	15.699	0.710	15.245	16.517	5.412	0.285	5.122	5.692
ENSG00000147852	VLDLR	2.9	7.8E-28	1.4E-25	71.193	1.207	70.035	72.443	24.341	1.622	22.486	25.488
ENSG00000186918	ZNF395	2.9	1.3E-23	1.2E-21	29.494	0.477	29.053	30.000	10.093	0.587	9.434	10.558
ENSG00000230454		2.9	1.9E-04	5.3E-04	1.087	0.148	0.941	1.237	0.369	0.133	0.268	0.519
ENSG00000233264		2.9	4.9E-04	1.3E-03	1.927	0.724	1.471	2.762	0.666	0.441	0.186	1.053
ENSG00000248015		2.9	2.8E-03	6.1E-03	0.401	0.178	0.279	0.605	0.132	0.047	0.078	0.160
ENSG00000254339		2.9	7.1E-22	4.9E-20	411.044	26.285	384.120	436.640	141.469	4.989	136.930	146.810
ENSG00000151726	ACSL1	3	2.3E-22	1.7E-20	20.165	0.969	19.414	21.259	6.627	0.689	5.989	7.358
ENSG00000154736	ADAMTS5	3	4.4E-12	5.7E-11	2.175	0.422	1.687	2.420	0.721	0.081	0.648	0.807
ENSG00000140057	AK7	3	2.2E-18	8.7E-17	9.465	0.719	8.666	10.062	3.156	0.055	3.092	3.190
ENSG00000011021	CLCN6	3	1.6E-26	2.5E-24	30.148	0.896	29.232	31.023	10.185	0.149	10.024	10.294
ENSG00000233588	CYP51A1P2	3	3.4E-23	2.9E-21	74.890	2.329	72.930	77.465	25.012	0.467	24.478	25.345
ENSG00000184731	FAM110C	3	2.1E-22	1.6E-20	34.494	3.331	30.817	37.307	11.322	0.561	10.737	11.856
ENSG00000230316	FEZF1-AS1	3	1.4E-03	3.2E-03	0.592	0.217	0.343	0.745	0.194	0.117	0.126	0.329
ENSG00000138641	HERC3	3	2.0E-20	1.1E-18	10.685	0.965	9.964	11.781	3.548	0.088	3.485	3.648
ENSG00000006062	MAP3K14	3	2.2E-19	1.0E-17	9.977	0.255	9.767	10.260	3.379	0.293	3.127	3.700
ENSG00000157168	NRG1	3	1.8E-10	1.6E-09	1.261	0.128	1.117	1.363	0.422	0.106	0.301	0.496
ENSG00000162631	NTNG1	3	1.1E-13	1.9E-12	2.909	0.361	2.652	3.322	0.978	0.171	0.841	1.169
ENSG00000229288	PCAT6	3	2.3E-13	3.8E-12	22.411	1.997	20.291	24.257	7.541	0.292	7.267	7.848
ENSG00000197747	S100A10	3	2.1E-28	4.3E-26	300.544	8.625	294.602	310.437	100.496	2.143	98.406	102.688
ENSG00000214193	SH3D21	3	8.7E-18	3.2E-16	10.090	0.495	9.660	10.630	3.331	0.193	3.142	3.528
ENSG00000137501	SYTL2	3	3.8E-21	2.3E-19	8.033	0.451	7.513	8.310	2.689	0.049	2.643	2.740
ENSG00000166046	TCP11L2	3	2.1E-13	3.5E-12	2.927	0.074	2.846	2.992	0.981	0.033	0.943	1.001
ENSG00000166483	WEE1	3	3.2E-30	8.8E-28	123.528	2.058	121.241	125.231	41.734	1.410	40.588	43.308
ENSG00000226800		3	2.6E-05	8.5E-05	5.310	0.570	4.710	5.843	1.751	0.373	1.322	1.996
ENSG00000267102		3	9.6E-11	9.2E-10	22.871	0.183	22.677	23.041	7.741	1.121	6.684	8.917
ENSG00000275055		3	8.8E-07	3.8E-06	6.563	0.913	5.513	7.161	2.162	0.352	1.867	2.552
ENSG00000120437	ACAT2	3.1	2.0E-33	1.1E-30	548.021	10.162	536.368	555.035	177.246	6.188	173.026	184.350
ENSG00000100346	CACNA1I	3.1	4.7E-14	8.8E-13	2.589	0.041	2.542	2.617	0.841	0.082	0.751	0.912
ENSG00000105971	CAV2	3.1	6.0E-16	1.6E-14	4.807	0.393	4.524	5.256	1.565	0.140	1.437	1.714
ENSG00000133101	CCNA1	3.1	1.4E-07	7.4E-07	2.871	0.398	2.435	3.216	0.928	0.278	0.682	1.230
ENSG00000124762	CDKN1A	3.1	1.4E-04	4.0E-04	47.223	2.018	44.887	48.909	15.140	1.730	13.831	17.102
ENSG00000130176	CNN1	3.1	3.2E-11	3.4E-10	47.464	5.139	41.568	50.995	15.244	0.260	15.048	15.540
ENSG00000268869	ESPNP	3.1	1.5E-06	6.4E-06	1.351	0.135	1.251	1.506	0.429	0.086	0.332	0.493
ENSG00000204856	FAM216A	3.1	1.7E-24	1.8E-22	57.943	0.609	57.546	58.645	18.908	1.496	17.962	20.632
ENSG00000135245	HILPDA	3.1	2.5E-25	3.1E-23	91.778	3.242	88.678	95.144	30.004	0.741	29.152	30.500
ENSG00000213859	KCTD11	3.1	1.9E-21	1.2E-19	25.164	0.448	24.667	25.442	8.034	0.731	7.273	8.731
ENSG00000006459	KDM7A	3.1	6.8E-29	1.5E-26	39.390	0.911	38.344	40.003	12.879	0.508	12.436	13.433
ENSG00000067082	KLF6	3.1	2.6E-26	3.9E-24	41.298	0.727	40.717	42.113	13.487	0.152	13.379	13.661
ENSG00000250889	LINC01336	3.1	2.2E-07	1.1E-06	8.144	2.857	5.645	11.258	2.569	0.274	2.290	2.837
ENSG00000131711	MAP1B	3.1	4.4E-32	1.9E-29	150.691	7.333	144.059	158.567	49.034	0.435	48.769	49.536
ENSG00000078814	MYH7B	3.1	1.4E-03	3.2E-03	0.240	0.077	0.179	0.327	0.076	0.027	0.053	0.106
ENSG00000151014	NOCT	3.1	9.7E-25	1.1E-22	63.462	1.904	61.497	65.298	20.322	1.154	19.414	21.620
ENSG00000133121	STARD13	3.1	1.1E-17	4.0E-16	5.284	0.501	4.729	5.704	1.692	0.174	1.509	1.853
ENSG00000041982	TNC	3.1	1.5E-19	6.9E-18	9.505	0.634	9.031	10.225	3.058	0.232	2.856	3.311
ENSG00000226869	LHFPL3-AS1	3.1	4.2E-02	6.9E-02	0.162	0.069	0.098	0.235	0.048	0.013	0.032	0.056
ENSG00000109046	WSB1	3.1	1.2E-29	2.9E-27	124.491	7.427	116.506	131.193	39.636	1.972	38.474	41.913
ENSG00000170949	ZNF160	3.1	7.8E-28	1.4E-25	34.399	1.005	33.802	35.559	11.252	0.420	10.803	11.637
ENSG00000254554		3.1	2.2E-04	6.0E-04	4.329	1.449	3.187	5.959	1.381	0.450	0.875	1.740
ENSG00000272983		3.1	6.2E-04	1.5E-03	0.313	0.107	0.201	0.415	0.098	0.068	0.020	0.138
ENSG00000279531		3.1	3.6E-13	5.7E-12	286.380	32.030	251.096	313.624	93.628	8.481	84.864	101.794
ENSG00000279602		3.1	1.6E-06	6.6E-06	3.529	0.721	3.072	4.359	1.152	0.509	0.634	1.652
ENSG00000184144	CNTN2	3.2	1.2E-13	2.2E-12	2.160	0.118	2.063	2.291	0.680	0.114	0.560	0.785
ENSG00000163354	DCST2	3.2	2.5E-03	5.3E-03	0.503	0.208	0.352	0.740	0.149	0.142	0.046	0.311
ENSG00000182580	EPHB3	3.2	1.8E-08	1.1E-07	1.560	0.360	1.325	1.975	0.491	0.037	0.451	0.524
ENSG00000099251	HSD17B7P2	3.2	1.0E-12	1.5E-11	12.389	1.671	11.317	14.315	3.934	0.629	3.246	4.481
ENSG00000270164	LINC01480	3.2	7.7E-03	1.5E-02	0.803	0.141	0.661	0.943	0.241	0.077	0.162	0.317
ENSG00000187193	MT1X	3.2	5.6E-16	1.5E-14	53.529	7.833	44.631	59.385	16.603	0.992	15.740	17.688
ENSG00000188211	NCR3LG1	3.2	2.4E-14	4.9E-13	4.300	0.262	4.059	4.579	1.367	0.177	1.186	1.541
ENSG00000089041	P2RX7	3.2	4.9E-08	2.8E-07	1.135	0.073	1.054	1.197	0.352	0.129	0.221	0.479
ENSG00000104881	PPP1R13L	3.2	3.6E-19	1.6E-17	9.795	0.656	9.381	10.552	3.109	0.272	2.804	3.325
ENSG00000185008	ROBO2	3.2	1.3E-18	5.5E-17	3.331	0.244	3.098	3.585	1.055	0.080	0.979	1.138
ENSG00000118160	SLC8A2	3.2	2.1E-14	4.3E-13	4.686	0.361	4.449	5.102	1.468	0.222	1.217	1.637
ENSG00000145777	TSLP	3.2	8.8E-10	7.0E-09	2.466	0.186	2.258	2.616	0.767	0.079	0.690	0.848
ENSG00000094841	UPRT	3.2	1.6E-24	1.7E-22	61.139	2.848	58.665	64.253	19.042	1.303	17.717	20.322
ENSG00000189068	VSTM1	3.2	2.3E-05	7.8E-05	2.783	0.285	2.501	3.072	0.877	0.227	0.736	1.139
ENSG00000197462		3.2	1.1E-04	3.2E-04	48.837	5.093	43.020	52.495	15.296	2.464	13.522	18.109
ENSG00000228274		3.2	7.1E-11	7.1E-10	3.388	0.230	3.126	3.557	1.048	0.074	0.975	1.123
ENSG00000279191		3.2	8.5E-06	3.1E-05	1.007	0.158	0.888	1.187	0.309	0.111	0.222	0.434
ENSG00000149925	ALDOA	3.3	2.0E-24	2.1E-22	716.190	28.647	683.112	732.772	217.015	11.896	204.685	228.423
ENSG00000154188	ANGPT1	3.3	1.5E-14	3.2E-13	3.607	0.219	3.421	3.848	1.076	0.152	0.947	1.243
ENSG00000163516	ANKZF1	3.3	2.4E-30	6.9E-28	57.827	1.310	56.326	58.743	17.774	0.331	17.418	18.073
ENSG00000165985	C1QL3	3.3	2.6E-07	1.3E-06	2.605	0.913	1.939	3.646	0.779	0.223	0.564	1.009
ENSG00000138764	CCNG2	3.3	1.6E-27	2.8E-25	26.530	0.036	26.506	26.572	8.023	0.181	7.847	8.208
ENSG00000129355	CDKN2D	3.3	7.3E-12	8.9E-11	8.972	0.391	8.626	9.396	2.706	0.426	2.245	3.085
ENSG00000172893	DHCR7	3.3	9.1E-25	1.0E-22	223.227	4.773	217.795	226.747	67.702	3.444	64.213	71.098
ENSG00000197930	ERO1A	3.3	7.9E-32	3.2E-29	82.733	2.421	80.066	84.794	25.100	0.127	25.003	25.243
ENSG00000283632	EXOC3L2	3.3	4.2E-11	4.4E-10	5.694	0.304	5.476	6.041	1.731	0.207	1.518	1.932
ENSG00000125848	FLRT3	3.3	4.8E-09	3.3E-08	2.098	0.255	1.836	2.346	0.632	0.179	0.433	0.781
ENSG00000170345	FOS	3.3	1.1E-15	2.8E-14	10.292	0.626	9.725	10.964	3.161	0.157	2.990	3.300
ENSG00000164604	GPR85	3.3	1.6E-07	8.4E-07	0.692	0.113	0.571	0.793	0.206	0.048	0.152	0.246
ENSG00000132196	HSD17B7	3.3	7.6E-28	1.4E-25	58.621	1.484	57.227	60.181	17.601	0.750	16.814	18.307
ENSG00000133816	MICAL2	3.3	7.4E-13	1.1E-11	1.230	0.128	1.142	1.377	0.369	0.059	0.309	0.427
ENSG00000218537	MIF-AS1	3.3	5.2E-07	2.4E-06	2.949	0.478	2.419	3.346	0.878	0.238	0.667	1.136
ENSG00000078114	NEBL	3.3	1.7E-25	2.2E-23	11.460	0.656	10.705	11.888	3.519	0.217	3.304	3.738
ENSG00000122861	PLAU	3.3	3.2E-23	2.8E-21	69.616	4.534	66.515	74.820	21.030	1.459	19.688	22.590
ENSG00000034677	RNF19A	3.3	3.5E-25	4.3E-23	27.626	1.473	25.957	28.744	8.384	0.316	8.027	8.625
ENSG00000113739	STC2	3.3	3.6E-30	9.7E-28	110.505	2.124	108.054	111.814	33.635	0.791	32.733	34.215
ENSG00000101255	TRIB3	3.3	1.6E-22	1.2E-20	31.336	1.749	29.688	33.171	9.601	1.033	8.624	10.682
ENSG00000005448	WDR54	3.3	3.1E-18	1.2E-16	61.702	2.402	60.228	64.474	18.549	0.726	17.876	19.319
ENSG00000231551		3.3	7.2E-04	1.8E-03	0.516	0.236	0.244	0.655	0.158	0.071	0.084	0.225
ENSG00000251432		3.3	4.6E-09	3.2E-08	2.424	0.198	2.257	2.642	0.735	0.171	0.540	0.856
ENSG00000104765	BNIP3L	3.4	2.7E-31	1.0E-28	161.058	3.252	157.323	163.262	47.362	0.581	46.698	47.776
ENSG00000164970	FAM219A	3.4	9.3E-23	7.6E-21	32.069	3.065	28.573	34.292	9.333	0.649	8.587	9.771
ENSG00000204882	GPR20	3.4	7.1E-11	7.0E-10	7.537	0.301	7.335	7.884	2.225	0.469	1.763	2.700
ENSG00000172965	MIR4435-2HG	3.4	3.7E-21	2.2E-19	12.437	1.189	11.132	13.460	3.675	0.221	3.451	3.892
ENSG00000076067	RBMS2	3.4	2.1E-26	3.1E-24	27.867	0.881	27.056	28.804	8.339	0.720	7.516	8.851
ENSG00000173262	SLC2A14	3.4	2.6E-27	4.3E-25	45.182	1.348	43.808	46.503	13.291	0.981	12.513	14.393
ENSG00000118785	SPP1	3.4	5.5E-18	2.1E-16	113.822	3.843	111.589	118.260	33.319	2.210	31.315	35.689
ENSG00000149591	TAGLN	3.4	1.2E-14	2.5E-13	286.224	21.592	263.029	305.742	84.473	7.097	78.031	92.080
ENSG00000105825	TFPI2	3.4	1.6E-22	1.2E-20	37.570	1.100	36.788	38.827	11.054	0.654	10.453	11.750
ENSG00000229124	VIM-AS1	3.4	3.6E-09	2.5E-08	3.763	0.523	3.178	4.184	1.092	0.179	0.912	1.270
ENSG00000275216		3.4	5.7E-06	2.1E-05	0.805	0.030	0.771	0.826	0.231	0.126	0.116	0.366
ENSG00000203876	ADD3-AS1	3.5	7.2E-06	2.6E-05	0.411	0.154	0.288	0.584	0.118	0.022	0.094	0.136
ENSG00000150594	ADRA2A	3.5	1.1E-06	4.6E-06	0.914	0.147	0.824	1.084	0.260	0.076	0.186	0.338
ENSG00000163347	CLDN1	3.5	1.1E-10	1.0E-09	7.976	0.612	7.374	8.597	2.264	0.210	2.085	2.495
ENSG00000179532	DNHD1	3.5	2.3E-20	1.2E-18	3.373	0.246	3.169	3.647	0.955	0.021	0.932	0.974
ENSG00000122877	EGR2	3.5	1.5E-10	1.4E-09	2.812	0.454	2.301	3.168	0.801	0.204	0.572	0.961
ENSG00000088340	FER1L4	3.5	4.6E-12	5.9E-11	1.811	0.285	1.584	2.130	0.515	0.064	0.462	0.587
ENSG00000164849	GPR146	3.5	2.3E-08	1.4E-07	2.102	0.576	1.438	2.475	0.600	0.062	0.530	0.647
ENSG00000196083	IL1RAP	3.5	2.1E-21	1.3E-19	5.862	0.441	5.529	6.362	1.655	0.135	1.510	1.776
ENSG00000185261	KIAA0825	3.5	1.6E-15	3.8E-14	2.931	0.292	2.596	3.136	0.834	0.160	0.655	0.967
ENSG00000128606	LRRC17	3.5	3.7E-19	1.7E-17	21.575	1.652	19.668	22.546	6.214	0.245	5.994	6.478
ENSG00000106631	MYL7	3.5	3.8E-06	1.5E-05	2.882	0.635	2.227	3.493	0.823	0.480	0.287	1.216
ENSG00000130751	NPAS1	3.5	2.8E-16	7.7E-15	11.273	0.235	11.002	11.418	3.263	0.616	2.597	3.812
ENSG00000181649	PHLDA2	3.5	4.6E-12	5.9E-11	16.294	2.472	13.995	18.910	4.652	0.078	4.564	4.712
ENSG00000153233	PTPRR	3.5	2.0E-06	8.0E-06	0.799	0.072	0.726	0.871	0.225	0.128	0.081	0.328
ENSG00000242242	PVRL3-AS1	3.5	4.5E-07	2.1E-06	3.640	1.490	2.220	5.191	1.026	0.249	0.844	1.310
ENSG00000260630	SNAI3-AS1	3.5	3.0E-12	3.9E-11	3.095	0.116	2.965	3.187	0.881	0.165	0.693	1.002
ENSG00000188897		3.5	1.3E-18	5.5E-17	5.076	0.384	4.809	5.517	1.435	0.052	1.374	1.466
ENSG00000144476	ACKR3	3.6	2.5E-10	2.3E-09	4.272	0.390	3.841	4.601	1.165	0.305	0.928	1.510
ENSG00000213199	ASIC3	3.6	8.0E-04	1.9E-03	0.552	0.237	0.281	0.724	0.151	0.048	0.102	0.198
ENSG00000130829	DUSP9	3.6	1.3E-09	1.0E-08	3.793	0.375	3.567	4.226	1.048	0.241	0.906	1.326
ENSG00000010310	GIPR	3.6	7.9E-05	2.4E-04	0.603	0.043	0.570	0.651	0.167	0.121	0.058	0.297
ENSG00000159399	HK2	3.6	3.8E-34	2.2E-31	186.441	1.426	185.152	187.973	51.551	2.047	49.504	53.598
ENSG00000067064	IDI1	3.6	2.2E-34	1.4E-31	385.231	9.923	374.906	394.695	106.178	4.076	103.221	110.828
ENSG00000119950	MXI1	3.6	2.3E-26	3.3E-24	38.393	2.241	35.903	40.246	10.623	0.451	10.300	11.139
ENSG00000118946	PCDH17	3.6	1.3E-08	8.0E-08	0.898	0.141	0.751	1.033	0.245	0.086	0.168	0.338
ENSG00000128564	VGF	3.6	7.3E-07	3.2E-06	29.568	1.442	27.913	30.555	8.207	0.929	7.646	9.279
ENSG00000270168		3.6	2.8E-07	1.4E-06	2.105	0.218	1.862	2.283	0.584	0.085	0.529	0.682
ENSG00000272583		3.6	8.5E-06	3.1E-05	7.699	1.471	6.435	9.314	2.100	1.104	1.204	3.333
ENSG00000273796		3.6	3.7E-05	1.2E-04	5.441	2.451	3.761	8.254	1.481	0.475	0.996	1.945
ENSG00000144550	CPNE9	3.7	4.8E-19	2.1E-17	18.657	0.986	17.537	19.395	5.098	0.319	4.730	5.299
ENSG00000095596	CYP26A1	3.7	4.3E-10	3.7E-09	3.598	0.682	2.840	4.162	0.967	0.274	0.734	1.269
ENSG00000188820	FAM26F	3.7	1.6E-09	1.2E-08	6.197	0.759	5.688	7.069	1.668	0.088	1.599	1.766
ENSG00000162981	FAM84A	3.7	2.6E-20	1.4E-18	7.652	0.668	7.225	8.422	2.062	0.247	1.805	2.297
ENSG00000166573	GALR1	3.7	9.2E-15	1.9E-13	2.187	0.251	1.963	2.459	0.597	0.056	0.532	0.636
ENSG00000228612	HK2P1	3.7	7.1E-18	2.6E-16	13.373	1.132	12.660	14.678	3.659	0.325	3.292	3.911
ENSG00000137331	IER3	3.7	6.0E-25	7.1E-23	132.683	4.726	129.919	138.139	36.432	1.494	35.498	38.155
ENSG00000163453	IGFBP7	3.7	2.4E-07	1.2E-06	2.314	0.113	2.187	2.399	0.625	0.119	0.495	0.728
ENSG00000277586	NEFL	3.7	9.5E-25	1.1E-22	30.285	0.511	29.734	30.743	8.150	0.775	7.502	9.009
ENSG00000182752	PAPPA	3.7	9.1E-13	1.3E-11	1.177	0.104	1.069	1.276	0.320	0.009	0.314	0.330
ENSG00000105650	PDE4C	3.7	2.1E-19	9.9E-18	4.739	0.165	4.559	4.883	1.275	0.074	1.219	1.359
ENSG00000203867	RBM20	3.7	2.8E-07	1.4E-06	0.524	0.101	0.416	0.617	0.142	0.043	0.103	0.188
ENSG00000164023	SGMS2	3.7	4.8E-06	1.8E-05	0.630	0.172	0.466	0.809	0.170	0.047	0.128	0.221
ENSG00000111981	ULBP1	3.7	2.5E-07	1.2E-06	2.723	0.323	2.351	2.923	0.743	0.377	0.380	1.134
ENSG00000228528		3.7	8.3E-04	2.0E-03	2.499	0.669	1.742	3.008	0.651	0.407	0.198	0.985
ENSG00000260103		3.7	3.2E-06	1.2E-05	1.358	0.255	1.140	1.638	0.370	0.182	0.242	0.578
ENSG00000261655		3.7	1.4E-13	2.5E-12	8.195	0.945	7.461	9.261	2.213	0.325	1.934	2.570
ENSG00000173157	ADAMTS20	3.8	6.3E-26	8.6E-24	20.793	1.009	19.791	21.808	5.449	0.290	5.164	5.743
ENSG00000227295	ELL2P1	3.8	3.9E-15	8.7E-14	11.429	1.060	10.418	12.533	3.011	0.731	2.199	3.614
ENSG00000113161	HMGCR	3.8	1.7E-35	1.5E-32	660.167	18.367	640.219	676.378	174.456	3.578	170.656	177.760
ENSG00000203930	LINC00632	3.8	7.3E-11	7.2E-10	3.257	0.249	2.976	3.451	0.847	0.192	0.725	1.068
ENSG00000052802	MSMO1	3.8	1.2E-34	7.8E-32	679.258	11.382	670.422	692.102	178.195	5.647	172.386	183.665
ENSG00000125851	PCSK2	3.8	2.4E-08	1.5E-07	0.759	0.157	0.654	0.940	0.196	0.060	0.138	0.258
ENSG00000227533	SLC2A1-AS1	3.8	5.4E-12	6.9E-11	4.389	0.930	3.579	5.404	1.154	0.129	1.045	1.297
ENSG00000185518	SV2B	3.8	1.7E-08	1.1E-07	0.494	0.106	0.377	0.582	0.130	0.017	0.112	0.147
ENSG00000026025	VIM	3.8	2.4E-08	1.4E-07	158.226	8.142	152.164	167.480	41.201	2.283	39.756	43.833
ENSG00000279744		3.8	1.2E-07	6.3E-07	3.388	0.517	2.891	3.923	0.900	0.521	0.330	1.350
ENSG00000166535	A2ML1	3.9	1.2E-22	9.7E-21	11.289	0.078	11.202	11.351	2.922	0.038	2.894	2.965
ENSG00000138772	ANXA3	3.9	1.6E-21	1.0E-19	64.299	1.875	62.607	66.316	16.718	0.641	16.142	17.408
ENSG00000159388	BTG2	3.9	4.1E-21	2.4E-19	21.417	0.740	20.658	22.137	5.453	0.408	4.997	5.787
ENSG00000113361	CDH6	3.9	1.2E-07	6.2E-07	0.556	0.080	0.486	0.643	0.142	0.037	0.102	0.176
ENSG00000222041	CYTOR	3.9	8.2E-22	5.6E-20	22.252	0.493	21.965	22.822	5.756	0.571	5.214	6.353
ENSG00000175197	DDIT3	3.9	6.2E-17	1.9E-15	27.803	2.585	24.874	29.763	7.201	0.798	6.289	7.774
ENSG00000100867	DHRS2	3.9	1.1E-12	1.6E-11	1.657	0.281	1.450	1.976	0.423	0.018	0.409	0.444
ENSG00000142227	EMP3	3.9	1.0E-22	8.4E-21	42.506	0.976	41.383	43.147	10.918	0.590	10.389	11.554
ENSG00000203780	FANK1	3.9	2.7E-08	1.6E-07	1.447	0.219	1.247	1.681	0.369	0.043	0.330	0.415
ENSG00000143167	GPA33	3.9	1.8E-06	7.6E-06	1.035	0.126	0.916	1.167	0.261	0.046	0.213	0.306
ENSG00000188483	IER5L	3.9	5.8E-08	3.2E-07	1.831	0.273	1.521	2.036	0.461	0.051	0.415	0.515
ENSG00000213398	LCAT	3.9	3.9E-16	1.0E-14	8.906	0.895	7.879	9.523	2.272	0.237	2.129	2.545
ENSG00000203706	SERTAD4-AS1	3.9	3.6E-10	3.1E-09	8.666	0.775	7.840	9.377	2.211	0.203	2.087	2.445
ENSG00000170006	TMEM154	3.9	1.1E-18	4.8E-17	3.076	0.121	2.970	3.208	0.787	0.052	0.730	0.832
ENSG00000181634	TNFSF15	3.9	2.5E-09	1.8E-08	0.870	0.162	0.685	0.988	0.223	0.024	0.196	0.242
ENSG00000254258		3.9	5.5E-02	8.6E-02	0.310	0.060	0.263	0.377	0.066	0.036	0.045	0.108
ENSG00000265401		3.9	1.1E-03	2.6E-03	6.617	3.287	2.993	9.407	1.700	1.238	0.303	2.662
ENSG00000101670	LIPG	4	4.1E-24	4.0E-22	8.681	0.361	8.296	9.012	2.167	0.133	2.027	2.290
ENSG00000138119	MYOF	4	1.5E-05	5.3E-05	6.941	0.819	6.142	7.778	1.736	0.170	1.543	1.867
ENSG00000057657	PRDM1	4	5.5E-07	2.5E-06	0.682	0.145	0.557	0.841	0.168	0.085	0.107	0.265
ENSG00000111218	PRMT8	4	6.2E-11	6.3E-10	1.283	0.112	1.156	1.368	0.321	0.071	0.273	0.402
ENSG00000176641	RNF152	4	3.9E-14	7.5E-13	3.356	0.633	2.889	4.076	0.832	0.150	0.705	0.999
ENSG00000145901	TNIP1	4	2.7E-29	6.1E-27	44.229	0.904	43.248	45.028	11.138	0.798	10.272	11.843
ENSG00000281508	CDR1	4.1	1.5E-09	1.2E-08	4.267	0.423	3.846	4.692	1.029	0.099	0.941	1.136
ENSG00000237753		4.1	1.8E-09	1.3E-08	2.840	0.126	2.729	2.977	0.685	0.197	0.484	0.877
ENSG00000256742		4.1	3.0E-10	2.6E-09	16.702	3.774	13.052	20.588	4.087	1.133	3.360	5.392
ENSG00000267424		4.1	7.6E-13	1.1E-11	22.117	2.888	18.798	24.051	5.385	0.216	5.137	5.536
ENSG00000113369	ARRDC3	4.2	2.1E-06	8.5E-06	19.902	0.563	19.308	20.428	4.731	0.666	3.989	5.279
ENSG00000176383	B3GNT4	4.2	1.2E-12	1.7E-11	2.592	0.042	2.547	2.630	0.611	0.066	0.540	0.669
ENSG00000099860	GADD45B	4.2	2.0E-18	8.0E-17	18.514	1.195	17.760	19.891	4.382	1.234	3.593	5.804
ENSG00000119125	GDA	4.2	1.9E-23	1.7E-21	10.225	0.742	9.608	11.049	2.455	0.169	2.260	2.561
ENSG00000188290	HES4	4.2	6.1E-04	1.5E-03	1.450	0.125	1.307	1.526	0.333	0.193	0.122	0.500
ENSG00000162194	LBHD1	4.2	2.5E-07	1.2E-06	1.526	0.079	1.448	1.607	0.355	0.254	0.208	0.649
ENSG00000185022	MAFF	4.2	1.9E-24	2.1E-22	15.812	1.168	14.738	17.056	3.809	0.095	3.716	3.906
ENSG00000059728	MXD1	4.2	1.9E-15	4.5E-14	3.730	0.187	3.537	3.910	0.895	0.123	0.792	1.031
ENSG00000235823	OLMALINC	4.2	4.3E-31	1.5E-28	139.548	6.009	135.743	146.476	33.503	2.043	32.233	35.860
ENSG00000134594	RAB33A	4.2	2.4E-16	6.7E-15	18.253	0.673	17.632	18.967	4.306	1.023	3.220	5.253
ENSG00000166401	SERPINB8	4.2	4.0E-10	3.4E-09	1.316	0.092	1.210	1.381	0.315	0.032	0.286	0.349
ENSG00000003436	TFPI	4.2	7.1E-13	1.1E-11	0.979	0.072	0.934	1.061	0.234	0.010	0.227	0.245
ENSG00000163762	TM4SF18	4.2	8.3E-06	3.0E-05	14.105	0.734	13.499	14.921	3.356	0.027	3.325	3.377
ENSG00000198796	ALPK2	4.3	3.0E-09	2.2E-08	0.684	0.067	0.620	0.753	0.157	0.033	0.130	0.194
ENSG00000162373	BEND5	4.3	2.8E-21	1.8E-19	24.361	1.995	22.545	26.496	5.653	0.331	5.294	5.946
ENSG00000248019	FAM13A-AS1	4.3	4.3E-06	1.6E-05	1.031	0.500	0.694	1.609	0.237	0.067	0.181	0.312
ENSG00000175265	GOLGA8A	4.3	2.0E-29	4.7E-27	37.975	2.667	35.037	40.242	8.901	0.439	8.599	9.405
ENSG00000182379	NXPH4	4.3	5.5E-08	3.1E-07	2.165	0.252	1.887	2.381	0.497	0.168	0.317	0.650
ENSG00000068971	PPP2R5B	4.3	1.9E-18	7.4E-17	17.835	0.867	16.876	18.562	4.112	0.229	3.848	4.248
ENSG00000165105	RASEF	4.3	4.6E-25	5.5E-23	16.229	0.457	15.793	16.704	3.817	0.538	3.261	4.335
ENSG00000172426	RSPH9	4.3	2.0E-06	8.0E-06	1.216	0.038	1.172	1.243	0.281	0.058	0.254	0.319
ENSG00000172123	SLFN12	4.3	8.0E-25	9.3E-23	32.435	1.447	31.593	34.105	7.479	0.244	7.201	7.662
ENSG00000112715	VEGFA	4.3	3.9E-31	1.4E-28	34.107	0.674	33.362	34.675	7.940	0.208	7.706	8.102
ENSG00000158023	WDR66	4.3	5.8E-19	2.5E-17	2.725	0.248	2.444	2.912	0.638	0.089	0.584	0.742
ENSG00000204758		4.3	4.5E-05	1.4E-04	0.659	0.056	0.614	0.722	0.150	0.002	0.147	0.152
ENSG00000257298		4.3	3.6E-05	1.2E-04	0.645	0.187	0.453	0.827	0.147	0.051	0.089	0.179
ENSG00000267745		4.3	4.1E-12	5.3E-11	9.650	1.398	8.772	11.262	2.261	0.732	1.463	2.900
ENSG00000283024		4.3	1.4E-03	3.2E-03	2.032	0.394	1.731	2.478	0.458	0.281	0.137	0.662
ENSG00000118777	ABCG2	4.4	9.1E-24	8.5E-22	15.170	0.369	14.840	15.569	3.447	0.062	3.375	3.486
ENSG00000167772	ANGPTL4	4.4	7.2E-13	1.1E-11	5.282	0.728	4.447	5.783	1.210	0.264	0.957	1.483
ENSG00000060718	COL11A1	4.4	1.4E-30	4.4E-28	42.828	3.054	39.530	45.557	9.746	0.508	9.223	10.237
ENSG00000196361	ELAVL3	4.4	2.1E-06	8.5E-06	0.661	0.257	0.478	0.955	0.146	0.060	0.086	0.205
ENSG00000115008	IL1A	4.4	8.6E-10	6.8E-09	4.517	0.928	3.481	5.273	1.026	0.320	0.789	1.390
ENSG00000225783	MIAT	4.4	5.6E-17	1.7E-15	104.229	6.433	97.781	110.647	23.672	0.687	23.249	24.465
ENSG00000189430	NCR1	4.4	2.1E-08	1.3E-07	3.035	0.302	2.691	3.258	0.687	0.245	0.504	0.966
ENSG00000162772	ATF3	4.5	1.5E-29	3.6E-27	60.169	1.923	57.966	61.510	13.537	0.575	12.911	14.041
ENSG00000118985	ELL2	4.5	2.1E-25	2.6E-23	69.017	0.504	68.644	69.590	15.249	0.914	14.310	16.136
ENSG00000175592	FOSL1	4.5	1.2E-16	3.6E-15	14.242	0.848	13.742	15.221	3.135	0.809	2.221	3.758
ENSG00000130513	GDF15	4.5	8.3E-04	2.0E-03	13.478	0.991	12.869	14.622	2.973	0.144	2.850	3.079
ENSG00000115548	KDM3A	4.5	7.8E-33	3.8E-30	45.040	0.341	44.661	45.323	10.059	0.450	9.574	10.464
ENSG00000136167	LCP1	4.5	1.2E-25	1.6E-23	27.709	2.208	25.257	29.539	6.229	0.098	6.116	6.293
ENSG00000122420	PTGFR	4.5	5.6E-07	2.6E-06	0.798	0.048	0.758	0.851	0.177	0.080	0.116	0.268
ENSG00000267265		4.5	8.9E-14	1.6E-12	11.547	1.752	10.335	13.556	2.602	0.809	1.886	3.479
ENSG00000165617	DACT1	4.6	2.0E-24	2.1E-22	114.626	6.178	107.574	119.082	24.728	1.231	23.985	26.149
ENSG00000118407	FILIP1	4.6	9.9E-26	1.3E-23	11.301	0.518	10.761	11.792	2.445	0.241	2.174	2.635
ENSG00000118473	SGIP1	4.6	3.3E-10	2.9E-09	0.775	0.062	0.707	0.828	0.167	0.038	0.134	0.208
ENSG00000137801	THBS1	4.6	4.8E-07	2.2E-06	48.753	3.386	45.103	51.790	10.589	0.204	10.403	10.808
ENSG00000145390	USP53	4.6	2.6E-32	1.2E-29	37.721	2.315	35.675	40.233	8.173	0.406	7.714	8.483
ENSG00000254365		4.6	8.0E-06	2.9E-05	56.982	2.510	54.302	59.279	12.488	0.884	11.472	13.087
ENSG00000278875		4.6	1.1E-10	1.0E-09	4.177	0.714	3.551	4.954	0.899	0.175	0.688	1.014
ENSG00000124143	ARHGAP40	4.7	9.6E-07	4.2E-06	1.749	0.519	1.353	2.337	0.362	0.067	0.308	0.437
ENSG00000105643	ARRDC2	4.7	5.7E-22	4.0E-20	11.317	0.831	10.395	12.007	2.413	0.262	2.199	2.705
ENSG00000164096	C6orf3	4.7	2.8E-33	1.5E-30	242.904	1.835	240.787	244.046	51.749	1.964	49.886	53.800
ENSG00000118523	CTGF	4.7	6.7E-08	3.7E-07	618.412	40.223	572.334	646.502	130.572	4.996	124.818	133.800
ENSG00000163565	IFI16	4.7	1.3E-13	2.2E-12	2.947	0.404	2.498	3.281	0.622	0.180	0.464	0.818
ENSG00000162975	KCNF1	4.7	6.9E-15	1.5E-13	6.884	0.184	6.676	7.020	1.454	0.197	1.282	1.668
ENSG00000104450	SPAG1	4.7	1.8E-14	3.6E-13	3.745	0.333	3.460	4.110	0.786	0.163	0.663	0.971
ENSG00000144339	TMEFF2	4.7	3.7E-09	2.6E-08	0.956	0.141	0.793	1.047	0.202	0.030	0.169	0.226
ENSG00000181458	TMEM45A	4.7	1.5E-27	2.6E-25	31.194	0.909	30.407	32.189	6.682	0.463	6.181	7.095
ENSG00000224163		4.7	7.3E-08	4.0E-07	7.645	1.669	6.546	9.566	1.574	0.696	0.772	2.009
ENSG00000254297		4.7	4.2E-04	1.1E-03	52.049	2.808	49.590	55.108	11.071	0.080	10.994	11.153
ENSG00000267547		4.7	1.2E-07	6.1E-07	6.577	1.530	5.174	8.208	1.378	0.126	1.301	1.524
ENSG00000167508	MVD	4.8	3.2E-35	2.7E-32	173.594	7.036	167.658	181.366	36.570	0.205	36.333	36.691
ENSG00000076641	PAG1	4.8	8.5E-09	5.6E-08	0.453	0.025	0.426	0.476	0.092	0.032	0.064	0.127
ENSG00000095303	PTGS1	4.8	1.2E-12	1.7E-11	2.119	0.114	2.014	2.239	0.441	0.160	0.259	0.558
ENSG00000080031	PTPRH	4.8	2.1E-08	1.2E-07	1.350	0.445	0.851	1.706	0.276	0.140	0.129	0.408
ENSG00000175793	SFN	4.8	8.6E-21	4.9E-19	34.852	3.829	31.561	39.055	7.299	0.512	6.715	7.669
ENSG00000254815		4.8	1.2E-10	1.1E-09	3.322	0.745	2.745	4.162	0.688	0.148	0.523	0.809
ENSG00000148926	ADM	4.9	4.7E-28	9.0E-26	324.256	15.069	308.856	338.972	66.253	3.667	62.020	68.456
ENSG00000143507	DUSP10	4.9	3.3E-10	2.9E-09	2.574	0.170	2.395	2.732	0.527	0.246	0.281	0.772
ENSG00000228451	SDAD1P1	4.9	3.5E-17	1.1E-15	8.622	0.644	7.901	9.139	1.770	0.297	1.430	1.976
ENSG00000236404	VLDLR-AS1	4.9	2.3E-24	2.3E-22	17.476	0.812	16.561	18.109	3.586	0.163	3.403	3.719
ENSG00000280114		4.9	3.6E-07	1.7E-06	2.199	0.111	2.112	2.324	0.437	0.182	0.312	0.646
ENSG00000204128	C2orf72	5	7.0E-17	2.1E-15	6.081	0.952	5.206	7.094	1.225	0.054	1.168	1.275
ENSG00000135625	EGR4	5	5.0E-02	7.9E-02	0.126	0.130	0.000	0.260	0.020	0.034	0.000	0.059
ENSG00000137440	FGFBP1	5	4.0E-07	1.9E-06	2.576	0.125	2.499	2.720	0.513	0.183	0.301	0.621
ENSG00000095752	IL11	5	8.3E-14	1.5E-12	5.717	0.211	5.483	5.894	1.146	0.202	0.919	1.307
ENSG00000204054	LINC00963	5	2.1E-16	6.0E-15	1.361	0.060	1.324	1.430	0.274	0.037	0.235	0.308
ENSG00000223784	LINP1	5	7.3E-13	1.1E-11	17.645	1.209	16.746	19.020	3.529	0.996	2.453	4.418
ENSG00000039139	DNAH5	5.1	1.4E-17	4.9E-16	1.627	0.196	1.508	1.852	0.321	0.041	0.273	0.348
ENSG00000137463	MGARP	5.1	4.6E-11	4.8E-10	6.746	1.175	5.455	7.752	1.310	0.519	0.730	1.731
ENSG00000196660	SLC30A10	5.1	2.0E-08	1.2E-07	0.866	0.146	0.703	0.986	0.169	0.075	0.095	0.244
ENSG00000095383	TBC1D2	5.1	3.2E-24	3.2E-22	19.816	1.284	18.696	21.217	3.876	0.277	3.556	4.049
ENSG00000225205		5.1	1.5E-18	6.0E-17	16.182	1.443	14.697	17.579	3.182	0.454	2.786	3.677
ENSG00000283930		5.1	3.1E-08	1.8E-07	1.290	0.500	0.995	1.867	0.246	0.036	0.212	0.284
ENSG00000186480	INSIG1	5.2	3.8E-35	3.1E-32	292.182	6.537	284.958	297.689	56.416	3.185	53.388	59.738
ENSG00000059804	SLC2A3	5.2	3.6E-39	1.0E-35	1582.865	38.917	1538.498	1611.225	305.345	7.779	300.474	314.316
ENSG00000136160	EDNRB	5.3	1.3E-06	5.4E-06	76.029	2.162	73.617	77.790	14.430	0.986	13.326	15.226
ENSG00000112972	HMGCS1	5.3	9.4E-39	2.3E-35	2068.624	29.312	2046.353	2101.832	395.112	15.914	379.216	411.043
ENSG00000170525	PFKFB3	5.3	8.0E-35	5.9E-32	75.818	2.516	73.716	78.605	14.320	0.332	14.074	14.698
ENSG00000151006	PRSS53	5.3	1.6E-11	1.9E-10	1.363	0.123	1.291	1.505	0.252	0.021	0.230	0.273
ENSG00000163661	PTX3	5.3	3.3E-15	7.7E-14	8.527	1.470	7.196	10.104	1.624	0.283	1.406	1.944
ENSG00000173401	GLIPR1L1	5.4	3.4E-10	2.9E-09	4.175	0.279	3.870	4.418	0.770	0.256	0.505	1.016
ENSG00000145632	PLK2	5.4	3.2E-08	1.9E-07	52.073	1.376	50.577	53.284	9.718	0.432	9.258	10.114
ENSG00000164211	STARD4	5.4	3.6E-27	5.7E-25	236.527	9.218	227.016	245.421	44.047	4.320	41.336	49.028
ENSG00000166148	AVPR1A	5.5	5.9E-08	3.3E-07	0.655	0.077	0.572	0.725	0.116	0.038	0.094	0.160
ENSG00000275410	HNF1B	5.5	1.6E-05	5.5E-05	0.699	0.188	0.525	0.898	0.125	0.099	0.047	0.236
ENSG00000109107	ALDOC	5.6	2.8E-34	1.7E-31	237.666	4.961	232.219	241.927	42.899	2.873	41.172	46.216
ENSG00000166923	GREM1	5.6	2.7E-11	3.0E-10	0.506	0.064	0.454	0.578	0.088	0.032	0.058	0.121
ENSG00000250412	KLHL2P1	5.6	1.6E-09	1.2E-08	11.868	2.173	10.221	14.331	2.078	0.923	1.475	3.141
ENSG00000100505	TRIM9	5.6	2.1E-17	7.2E-16	9.959	0.331	9.577	10.157	1.791	0.330	1.437	2.090
ENSG00000167612	ANKRD33	5.7	9.9E-09	6.4E-08	1.231	0.388	0.917	1.665	0.213	0.060	0.147	0.264
ENSG00000173930	SLCO4C1	5.7	2.4E-28	4.8E-26	28.155	1.854	26.159	29.822	4.907	0.515	4.468	5.473
ENSG00000259203		5.7	3.7E-21	2.2E-19	75.498	1.712	73.666	77.059	13.270	0.826	12.535	14.164
ENSG00000114315	HES1	5.8	1.0E-27	1.8E-25	53.414	0.536	52.899	53.970	9.153	0.626	8.536	9.787
ENSG00000275074	NUDT18	5.8	2.0E-12	2.7E-11	5.853	1.493	4.130	6.748	1.004	0.256	0.777	1.281
ENSG00000139289	PHLDA1	5.8	3.6E-15	8.1E-14	98.763	4.294	94.821	103.338	17.032	0.101	16.916	17.104
ENSG00000069667	RORA	5.8	1.9E-26	3.0E-24	7.369	0.598	6.703	7.860	1.266	0.153	1.172	1.443
ENSG00000159167	STC1	5.8	3.3E-08	1.9E-07	114.452	1.897	112.321	115.957	19.721	2.090	17.878	21.992
ENSG00000284294		5.8	8.6E-09	5.7E-08	5.760	1.386	4.230	6.932	0.979	0.255	0.686	1.146
ENSG00000148848	ADAM12	5.9	7.2E-09	4.8E-08	0.509	0.044	0.461	0.548	0.084	0.013	0.071	0.097
ENSG00000135046	ANXA1	5.9	5.7E-11	5.8E-10	17.169	1.262	16.278	18.613	2.932	0.329	2.589	3.246
ENSG00000185933	CALHM1	5.9	1.2E-10	1.1E-09	2.169	0.400	1.909	2.629	0.365	0.095	0.268	0.458
ENSG00000131668	BARX1	6	9.5E-17	2.8E-15	10.864	0.683	10.076	11.282	1.797	0.119	1.661	1.887
ENSG00000196968	FUT11	6	4.9E-30	1.3E-27	31.187	0.157	31.058	31.362	5.223	0.017	5.206	5.240
ENSG00000100100	PIK3IP1	6	8.9E-21	5.1E-19	9.668	0.512	9.214	10.222	1.608	0.340	1.358	1.995
ENSG00000119938	PPP1R3C	6	1.6E-22	1.2E-20	16.225	0.469	15.756	16.695	2.695	0.115	2.562	2.767
ENSG00000171903	CYP4F11	6.1	1.3E-07	6.6E-07	1.796	0.688	1.165	2.530	0.287	0.053	0.244	0.347
ENSG00000079739	PGM1	6.1	1.3E-35	1.3E-32	177.911	3.715	175.283	182.162	29.094	2.285	26.537	30.937
ENSG00000272405		6.1	5.9E-10	4.9E-09	2.048	0.060	2.012	2.118	0.329	0.211	0.087	0.477
ENSG00000102554	KLF5	6.2	1.1E-20	5.9E-19	8.901	0.710	8.400	9.714	1.428	0.224	1.296	1.686
ENSG00000101746	NOL4	6.2	5.3E-19	2.3E-17	4.295	0.250	4.017	4.500	0.692	0.028	0.663	0.719
ENSG00000122884	P4HA1	6.2	1.7E-35	1.5E-32	214.846	5.065	210.942	220.570	34.678	0.963	33.962	35.773
ENSG00000020577	SAMD4A	6.2	1.5E-30	4.6E-28	15.859	1.133	15.153	17.166	2.577	0.197	2.433	2.801
ENSG00000276141	WHAMMP3	6.2	1.1E-09	8.4E-09	0.701	0.122	0.607	0.839	0.110	0.059	0.054	0.172
ENSG00000237595		6.2	6.2E-12	7.8E-11	7.329	0.688	6.560	7.884	1.173	0.112	1.057	1.282
ENSG00000003137	CYP26B1	6.3	1.1E-06	4.6E-06	0.665	0.159	0.482	0.769	0.102	0.081	0.054	0.195
ENSG00000072682	P4HA2	6.3	1.1E-26	1.7E-24	13.840	1.052	12.635	14.583	2.210	0.379	1.791	2.530
ENSG00000169174	PCSK9	6.4	1.1E-36	2.0E-33	143.649	5.796	138.297	149.806	22.619	1.086	21.510	23.681
ENSG00000152952	PLOD2	6.4	2.8E-36	4.4E-33	106.692	3.417	102.872	109.458	16.623	0.670	15.944	17.283
ENSG00000177181	RIMKLA	6.4	1.8E-29	4.3E-27	22.335	1.570	21.327	24.144	3.498	0.101	3.428	3.614
ENSG00000105877	DNAH11	6.5	4.2E-24	4.1E-22	4.400	0.231	4.251	4.666	0.674	0.113	0.571	0.795
ENSG00000268964	ERVV-2	6.5	2.9E-10	2.6E-09	2.206	0.226	1.959	2.404	0.333	0.148	0.172	0.463
ENSG00000111432	FZD10	6.6	2.2E-10	2.0E-09	2.276	0.713	1.458	2.770	0.339	0.188	0.170	0.557
ENSG00000139278	GLIPR1	6.6	1.8E-11	2.1E-10	1.190	0.372	0.837	1.578	0.177	0.019	0.157	0.196
ENSG00000139832	RAB20	6.6	5.2E-31	1.8E-28	138.861	7.470	133.289	147.349	20.997	1.993	19.224	23.154
ENSG00000250241		6.6	6.4E-12	8.0E-11	2.349	0.350	1.980	2.677	0.354	0.093	0.250	0.427
ENSG00000006606	CCL26	6.7	8.1E-21	4.6E-19	46.541	0.916	45.911	47.592	6.959	0.901	5.988	7.766
ENSG00000134769	DTNA	6.7	1.3E-35	1.3E-32	42.683	0.600	42.294	43.374	6.374	0.485	5.959	6.908
ENSG00000151812	SLC35F4	6.7	4.2E-08	2.4E-07	0.834	0.175	0.704	1.034	0.120	0.070	0.066	0.199
ENSG00000167874	TMEM88	6.7	1.2E-08	7.5E-08	4.013	0.754	3.175	4.638	0.580	0.235	0.321	0.779
ENSG00000232433	GXYLT1P3	6.8	5.8E-11	6.0E-10	4.740	0.481	4.253	5.215	0.686	0.271	0.491	0.995
ENSG00000260231	JHDM1D-AS1	6.8	2.1E-20	1.2E-18	15.879	1.488	14.595	17.510	2.330	0.364	2.113	2.750
ENSG00000140939	NOL3	6.8	8.8E-19	3.7E-17	7.302	0.475	6.944	7.841	1.067	0.291	0.747	1.316
ENSG00000152256	PDK1	6.8	6.7E-26	9.0E-24	44.421	0.920	43.669	45.447	6.571	0.054	6.509	6.606
ENSG00000095370	SH2D3C	6.8	5.2E-24	5.0E-22	10.597	0.548	10.071	11.165	1.569	0.118	1.439	1.668
ENSG00000265972	TXNIP	6.8	6.3E-11	6.4E-10	16.551	0.171	16.396	16.734	2.424	0.184	2.288	2.633
ENSG00000267696	ERVK-28	6.9	3.8E-06	1.5E-05	2.642	0.295	2.322	2.903	0.355	0.091	0.255	0.433
ENSG00000128591	FLNC	6.9	1.7E-09	1.3E-08	58.455	6.639	51.880	65.155	8.427	0.675	7.886	9.184
ENSG00000178878	APOLD1	7	2.4E-17	7.9E-16	2.500	0.074	2.429	2.577	0.357	0.072	0.289	0.433
ENSG00000122641	INHBA	7	6.1E-07	2.8E-06	0.362	0.043	0.325	0.409	0.050	0.017	0.034	0.067
ENSG00000119715	ESRRB	7.1	4.6E-12	5.9E-11	2.794	0.412	2.467	3.257	0.394	0.188	0.269	0.610
ENSG00000275026	GXYLT1P4	7.2	3.0E-11	3.3E-10	2.530	0.259	2.262	2.779	0.345	0.113	0.248	0.469
ENSG00000069812	HES2	7.2	2.5E-04	6.7E-04	0.235	0.055	0.171	0.270	0.030	0.033	0.000	0.065
ENSG00000279882		7.2	6.3E-04	1.6E-03	2.064	0.493	1.683	2.621	0.243	0.089	0.142	0.311
ENSG00000175513	TSGA10IP	7.3	6.6E-16	1.7E-14	7.390	0.200	7.186	7.585	1.009	0.172	0.818	1.152
ENSG00000211448	DIO2	7.4	1.6E-12	2.2E-11	1.289	0.195	1.112	1.499	0.173	0.038	0.131	0.205
ENSG00000240694	PNMA2	7.4	6.7E-31	2.3E-28	42.886	1.893	40.701	44.049	5.797	0.286	5.468	5.988
ENSG00000232907	DLGAP4-AS1	7.5	1.5E-18	6.3E-17	8.120	0.408	7.790	8.577	1.084	0.166	0.895	1.204
ENSG00000235674	LDHAP2	7.5	3.7E-20	1.9E-18	28.415	2.641	26.612	31.447	3.800	0.384	3.450	4.211
ENSG00000214110	LDHAP4	7.5	7.8E-33	3.8E-30	487.226	20.518	468.922	509.405	65.446	3.772	61.120	68.044
ENSG00000228328		7.6	6.5E-11	6.5E-10	4.784	0.864	3.836	5.528	0.613	0.197	0.492	0.839
ENSG00000274628		7.6	7.1E-11	7.1E-10	0.684	0.103	0.578	0.784	0.089	0.022	0.067	0.110
ENSG00000187017	ESPN	7.7	5.1E-30	1.3E-27	20.352	0.535	19.742	20.741	2.650	0.303	2.394	2.985
ENSG00000168875	SOX14	7.7	4.1E-11	4.4E-10	3.381	1.113	2.629	4.660	0.429	0.146	0.265	0.544
ENSG00000134333	LDHA	7.8	1.4E-09	1.1E-08	1773.267	12.703	1758.599	1780.651	228.992	1.287	227.695	230.268
ENSG00000267279		7.8	5.0E-20	2.5E-18	24.993	1.567	23.836	26.776	3.201	0.772	2.345	3.845
ENSG00000113083	LOX	7.9	8.0E-22	5.5E-20	6.609	0.322	6.261	6.895	0.835	0.045	0.806	0.887
ENSG00000006128	TAC1	7.9	4.1E-25	4.9E-23	33.480	2.681	30.494	35.679	4.242	0.608	3.625	4.840
ENSG00000224063		7.9	5.2E-10	4.4E-09	3.311	0.416	2.831	3.574	0.409	0.148	0.285	0.573
ENSG00000138135	CH25H	8	8.0E-10	6.5E-09	2.474	0.158	2.361	2.655	0.301	0.090	0.249	0.404
ENSG00000101438	SLC32A1	8.1	7.9E-11	7.8E-10	2.087	0.155	1.979	2.265	0.251	0.123	0.109	0.326
ENSG00000145824	CXCL14	8.2	6.8E-06	2.5E-05	4.087	0.562	3.456	4.533	0.491	0.204	0.283	0.690
ENSG00000090530	P3H2	8.3	2.2E-26	3.3E-24	63.096	0.869	62.206	63.943	7.657	0.442	7.302	8.152
ENSG00000117394	SLC2A1	8.3	2.2E-39	7.6E-36	1146.163	28.637	1114.305	1169.762	138.863	2.425	136.138	140.782
ENSG00000167995	BEST1	8.4	2.9E-11	3.2E-10	0.575	0.069	0.509	0.648	0.068	0.025	0.046	0.094
ENSG00000161958	FGF11	8.4	6.1E-22	4.3E-20	7.048	0.323	6.679	7.282	0.839	0.064	0.778	0.907
ENSG00000235847	LDHAP7	8.4	1.1E-21	7.4E-20	45.733	5.535	39.641	50.454	5.457	0.292	5.284	5.794
ENSG00000102144	PGK1	8.4	2.8E-14	5.5E-13	2177.012	70.272	2105.443	2245.911	259.225	1.578	257.477	260.545
ENSG00000115457	IGFBP2	8.5	5.7E-18	2.1E-16	856.873	34.846	816.702	878.949	101.364	8.297	92.246	108.472
ENSG00000142871	CYR61	8.6	1.6E-08	9.8E-08	618.491	48.829	564.230	658.890	71.782	5.941	66.003	77.874
ENSG00000113578	FGF1	8.6	3.0E-13	4.8E-12	1.910	0.478	1.492	2.431	0.220	0.109	0.103	0.318
ENSG00000251493	FOXD1	8.6	1.3E-08	8.0E-08	1.413	0.154	1.236	1.518	0.159	0.003	0.156	0.161
ENSG00000121769	FABP3	8.7	5.4E-35	4.2E-32	267.207	5.322	261.092	270.797	30.637	0.573	30.037	31.178
ENSG00000146151	HMGCLL1	8.7	1.9E-10	1.8E-09	1.028	0.021	1.007	1.048	0.116	0.077	0.059	0.204
ENSG00000169856	ONECUT1	8.7	3.2E-22	2.4E-20	8.683	0.476	8.137	9.011	0.998	0.054	0.956	1.059
ENSG00000172782	FADS6	8.8	4.0E-10	3.4E-09	1.759	0.068	1.702	1.834	0.193	0.124	0.098	0.334
ENSG00000257913		8.8	4.4E-12	5.7E-11	2.596	0.157	2.414	2.690	0.289	0.035	0.250	0.314
ENSG00000132510	KDM6B	8.9	3.3E-24	3.3E-22	6.781	0.339	6.406	7.067	0.759	0.126	0.616	0.852
ENSG00000183379	SYNDIG1L	8.9	2.0E-24	2.1E-22	35.740	1.414	34.241	37.051	4.029	1.125	3.316	5.325
ENSG00000213290	PGK1P2	9	5.0E-19	2.2E-17	53.327	4.945	48.684	58.527	5.923	2.453	4.156	8.724
ENSG00000217801		9	1.2E-16	3.5E-15	8.132	0.921	7.139	8.958	0.895	0.422	0.518	1.351
ENSG00000274893		9.1	3.5E-10	3.0E-09	2.401	0.875	1.856	3.410	0.253	0.048	0.203	0.298
ENSG00000100678	SLC8A3	9.2	9.8E-19	4.1E-17	2.627	0.073	2.576	2.710	0.285	0.085	0.216	0.380
ENSG00000206172	HBA1	9.3	4.5E-09	3.1E-08	3.750	0.252	3.576	4.040	0.384	0.074	0.321	0.465
ENSG00000166670	MMP10	9.3	1.3E-09	1.0E-08	2.734	0.941	1.847	3.720	0.288	0.181	0.080	0.397
ENSG00000143878	RHOB	9.3	2.3E-34	1.4E-31	143.426	6.201	136.372	148.017	15.489	1.073	14.251	16.154
ENSG00000114023	FAM162A	9.5	5.2E-40	3.1E-36	303.656	4.702	298.506	307.721	32.125	0.469	31.729	32.644
ENSG00000223839	FAM95B1	9.7	2.9E-12	3.9E-11	0.775	0.063	0.715	0.840	0.078	0.030	0.052	0.110
ENSG00000269968		9.7	4.5E-05	1.4E-04	42.077	12.713	27.502	50.877	4.325	1.158	2.998	5.131
ENSG00000236090	LDHAP3	9.8	7.8E-23	6.4E-21	41.011	2.733	38.714	44.034	4.197	0.636	3.534	4.802
ENSG00000126562	WNK4	9.9	1.9E-12	2.6E-11	1.658	0.612	1.244	2.360	0.163	0.079	0.082	0.241
ENSG00000122691	TWIST1	10	3.5E-10	3.0E-09	1.913	0.326	1.539	2.126	0.183	0.042	0.134	0.207
ENSG00000269526	ERVV-1	10.2	1.4E-10	1.3E-09	1.878	0.340	1.548	2.228	0.179	0.066	0.128	0.254
ENSG00000177606	JUN	10.2	8.5E-34	4.8E-31	83.280	4.252	79.448	87.854	8.151	0.748	7.685	9.014
ENSG00000125629	INSIG2	10.3	1.7E-35	1.5E-32	76.397	2.217	74.873	78.941	7.454	0.556	6.896	8.008
ENSG00000213574	LDHAP5	10.3	1.6E-12	2.2E-11	12.084	2.046	9.760	13.614	1.156	0.298	0.877	1.469
ENSG00000127329	PTPRB	10.6	1.7E-21	1.1E-19	2.258	0.076	2.210	2.346	0.214	0.039	0.169	0.242
ENSG00000019549	SNAI2	10.7	1.2E-15	3.1E-14	5.027	0.108	4.902	5.097	0.463	0.151	0.371	0.638
ENSG00000131069	ACSS2	10.8	1.2E-38	2.5E-35	174.280	5.401	170.413	180.450	16.148	1.083	15.383	17.388
ENSG00000121966	CXCR4	10.8	5.4E-22	3.9E-20	13.776	0.373	13.362	14.086	1.267	0.230	1.131	1.533
ENSG00000151834	GABRA2	10.8	2.0E-25	2.5E-23	4.608	0.455	4.207	5.102	0.427	0.044	0.377	0.460
ENSG00000231672	DIRC3	10.9	4.8E-12	6.1E-11	1.122	0.060	1.079	1.191	0.099	0.018	0.088	0.120
ENSG00000100311	PDGFB	10.9	3.3E-23	2.8E-21	84.447	5.873	77.728	88.604	7.729	0.434	7.243	8.049
ENSG00000279689		11	1.3E-13	2.2E-12	2.020	0.266	1.715	2.210	0.180	0.021	0.156	0.194
ENSG00000234456	MAGI2-AS3	11.2	2.8E-21	1.7E-19	2.591	0.099	2.485	2.682	0.231	0.091	0.176	0.336
ENSG00000280143		11.5	1.5E-23	1.4E-21	13.274	1.129	11.973	13.997	1.149	0.271	0.957	1.459
ENSG00000231890	DARS-AS1	11.6	9.8E-16	2.5E-14	1.654	0.164	1.483	1.810	0.141	0.041	0.107	0.187
ENSG00000269888		11.6	2.0E-03	4.5E-03	25.536	10.827	18.870	38.029	2.168	0.808	1.321	2.931
ENSG00000147869	CER1	11.8	5.3E-28	1.0E-25	78.111	5.013	73.844	83.631	6.628	0.794	5.748	7.290
ENSG00000227471	AKR1B15	12	2.3E-13	3.8E-12	2.063	0.265	1.817	2.344	0.167	0.040	0.126	0.205
ENSG00000104419	NDRG1	12	1.0E-31	4.1E-29	15.542	0.849	14.678	16.376	1.295	0.223	1.038	1.431
ENSG00000136928	GABBR2	12.1	3.2E-24	3.2E-22	5.156	0.393	4.712	5.459	0.424	0.052	0.384	0.482
ENSG00000114480	GBE1	12.1	2.7E-36	4.4E-33	87.288	2.970	83.976	89.714	7.257	0.334	6.879	7.510
ENSG00000138650	PCDH10	12.1	5.6E-22	4.0E-20	26.489	0.678	26.042	27.269	2.197	0.136	2.040	2.282
ENSG00000170379	TCAF2	12.1	8.0E-14	1.4E-12	1.213	0.088	1.137	1.309	0.098	0.037	0.074	0.141
ENSG00000078401	EDN1	12.3	5.6E-14	1.0E-12	4.312	0.248	4.056	4.551	0.342	0.277	0.069	0.622
ENSG00000155897	ADCY8	12.4	1.1E-19	5.3E-18	30.115	1.052	29.136	31.227	2.441	0.338	2.219	2.830
ENSG00000124942	AHNAK	12.4	5.0E-16	1.3E-14	20.783	0.680	20.249	21.548	1.673	0.104	1.553	1.737
ENSG00000206532		12.5	2.5E-20	1.3E-18	16.313	1.028	15.126	16.911	1.291	0.133	1.201	1.444
ENSG00000184545	DUSP8	12.7	6.0E-21	3.5E-19	5.147	0.469	4.651	5.584	0.401	0.063	0.333	0.457
ENSG00000188624	IGFL3	13	6.9E-11	6.9E-10	6.733	1.195	5.370	7.601	0.487	0.311	0.211	0.824
ENSG00000075426	FOSL2	13.1	1.5E-30	4.6E-28	20.725	1.015	19.708	21.739	1.585	0.128	1.483	1.729
ENSG00000272870		13.3	7.3E-22	5.1E-20	12.875	0.514	12.538	13.467	0.969	0.464	0.437	1.288
ENSG00000135766	EGLN1	13.4	1.0E-35	1.3E-32	52.634	1.652	50.777	53.939	3.927	0.497	3.367	4.317
ENSG00000114268	PFKFB4	13.4	5.7E-36	7.4E-33	65.635	1.857	63.492	66.740	4.912	0.682	4.437	5.694
ENSG00000147883	CDKN2B	13.6	1.2E-13	2.1E-12	1.754	0.208	1.521	1.920	0.126	0.072	0.067	0.207
ENSG00000173727		14.1	5.5E-12	6.9E-11	3.437	0.123	3.298	3.531	0.236	0.139	0.154	0.397
ENSG00000125462	C1orf61	14.8	9.1E-30	2.3E-27	8.698	0.626	8.025	9.263	0.587	0.039	0.543	0.617
ENSG00000181577	C6orf223	14.8	3.0E-30	8.2E-28	29.159	1.008	28.024	29.948	1.973	0.523	1.391	2.405
ENSG00000215244		15	6.8E-11	6.8E-10	1.435	0.047	1.382	1.473	0.090	0.046	0.044	0.136
ENSG00000235831	BHLHE40-AS1	15.4	1.1E-14	2.4E-13	3.503	0.268	3.297	3.806	0.221	0.104	0.103	0.301
ENSG00000115461	IGFBP5	15.5	1.1E-26	1.7E-24	9.492	0.382	9.247	9.932	0.611	0.123	0.477	0.720
ENSG00000230537		15.5	2.6E-11	2.9E-10	6.438	1.100	5.577	7.676	0.394	0.420	0.000	0.837
ENSG00000205129	C4orf47	15.9	1.2E-24	1.3E-22	36.123	2.326	34.237	38.721	2.267	0.320	1.956	2.595
ENSG00000261051		15.9	1.3E-12	1.8E-11	2.949	0.334	2.715	3.332	0.177	0.078	0.130	0.267
ENSG00000168209	DDIT4	16.1	7.3E-40	3.1E-36	771.138	17.798	754.392	789.828	48.050	2.505	45.816	50.758
ENSG00000223749	MIR503HG	16.5	2.0E-19	9.4E-18	10.591	1.352	9.198	11.898	0.633	0.161	0.503	0.813
ENSG00000143847	PPFIA4	17	5.1E-32	2.1E-29	17.316	1.162	16.235	18.545	1.019	0.140	0.927	1.180
ENSG00000186352	ANKRD37	17.4	9.4E-25	1.1E-22	16.013	0.870	15.321	16.990	0.917	0.255	0.687	1.191
ENSG00000226510	UPK1A-AS1	17.5	2.6E-20	1.4E-18	25.719	2.951	23.889	29.122	1.451	0.591	0.879	2.058
ENSG00000279092		17.9	3.6E-10	3.1E-09	0.922	0.071	0.850	0.992	0.048	0.036	0.015	0.087
ENSG00000224106	CYP4F25P	18.7	3.5E-10	3.1E-09	6.035	0.675	5.627	6.814	0.291	0.194	0.087	0.473
ENSG00000183691	NOG	19.3	9.7E-16	2.5E-14	5.844	0.962	4.735	6.434	0.293	0.070	0.221	0.360
ENSG00000105668	UPK1A	19.3	2.0E-19	9.4E-18	15.868	0.405	15.405	16.158	0.816	0.549	0.409	1.441
ENSG00000183775	KCTD16	19.6	2.5E-23	2.2E-21	2.494	0.259	2.198	2.680	0.127	0.040	0.086	0.166
ENSG00000123095	BHLHE41	20	1.6E-25	2.1E-23	15.270	0.054	15.208	15.306	0.763	0.148	0.637	0.926
ENSG00000179388	EGR3	20.4	1.5E-08	9.3E-08	0.846	0.067	0.769	0.890	0.038	0.016	0.029	0.056

ENSG00000103522	IL21R	20.6	2.4E-30	6.9E-28	18.648	1.313	17.399	20.017	0.807	0.150	0.818	1.080
ENSG00000146592	CREB5	21.1	7.2E-32	3.0E-29	11.325	0.712	10.694	12.097	0.535	0.055	0.494	0.598
ENSG00000148677	ANKRD1	22.9	5.8E-10	4.8E-09	14.642	2.096	12.286	16.301	0.630	0.321	0.284	0.918
ENSG00000044524	EPHA3	24.8	5.1E-17	1.6E-15	5.043	0.280	4.864	5.365	0.201	0.035	0.163	0.232
ENSG00000247095	MIR210HG	25.2	2.5E-27	4.3E-25	29.834	3.237	26.097	31.751	1.178	0.107	1.058	1.264
ENSG00000140279	DUOX2	25.6	8.7E-11	8.5E-10	0.406	0.110	0.338	0.534	0.014	0.014	0.000	0.028
ENSG00000197358	BNIP3P1	29.5	7.9E-23	6.4E-21	185.469	4.283	180.537	188.254	6.288	0.748	5.464	6.926
ENSG00000061656	SPAG4	30.4	3.0E-26	4.3E-24	18.383	0.596	17.709	18.841	0.597	0.224	0.437	0.852
ENSG00000176171	BNIP3	30.9	5.5E-15	1.2E-13	282.789	2.727	279.921	285.348	9.188	0.565	8.578	9.693
ENSG00000120738	EGR1	32.6	2.4E-09	1.8E-08	101.401	12.913	87.224	112.491	3.113	0.200	2.955	3.339
ENSG00000204335	SP5	37.8	5.2E-11	5.4E-10	2.017	0.173	1.873	2.209	0.045	0.039	0.000	0.068
ENSG00000152804	HHEX	38.2	8.0E-15	1.7E-13	3.086	0.587	2.421	3.536	0.073	0.030	0.055	0.108
ENSG00000167851	CD300A	41.2	8.3E-25	9.4E-23	16.535	0.560	16.199	17.182	0.396	0.163	0.208	0.508
ENSG00000106366	SERPINE1	43.7	6.0E-11	6.1E-10	259.424	24.642	230.976	274.162	5.944	0.607	5.245	6.327
ENSG00000276600	RAB7B	44.5	3.0E-09	2.2E-08	0.792	0.243	0.501	0.925	0.012	0.021	0.000	0.037
ENSG00000172478	C2orf54	49.1	1.2E-17	4.2E-16	4.408	0.481	4.052	4.956	0.083	0.075	0.000	0.148
ENSG00000171388	APLN	52.9	3.4E-36	4.8E-33	84.882	5.756	78.303	88.984	1.601	0.413	1.133	1.915
ENSG00000120937	NPPB	57.8	2.4E-23	2.1E-21	36.189	3.873	31.756	38.921	0.600	0.010	0.589	0.608
ENSG00000164879	CA3	63.5	8.5E-35	6.0E-32	68.998	1.944	67.085	70.971	1.082	0.337	0.791	1.451
ENSG00000015592	STMN4	68.3	2.2E-19	1.0E-17	5.169	0.940	4.446	6.232	0.070	0.024	0.042	0.085
ENSG00000204677	FAM153C	76.6	5.4E-13	8.3E-12	1.152	0.150	1.009	1.308	0.011	0.019	0.000	0.033
ENSG00000134107	BHLHE40	77.7	4.2E-40	3.1E-36	126.514	4.080	121.806	129.006	1.630	0.153	1.457	1.747
ENSG00000230432		78.9	2.1E-24	2.2E-22	86.525	4.353	81.889	90.524	1.038	0.508	0.452	1.347
ENSG00000182230	FAM153B	92.6	7.5E-24	7.1E-22	2.210	0.144	2.117	2.376	0.022	0.010	0.013	0.032
ENSG00000170074	FAM153A	99.3	2.4E-14	4.7E-13	0.658	0.016	0.644	0.676	0.005	0.004	0.000	0.008
ENSG00000141526	SLC16A3	199.7	5.4E-40	3.1E-36	41.401	1.102	40.483	42.623	0.206	0.056	0.155	0.266
ENSG00000124785	NRN1	213.4	2.3E-28	4.7E-26	27.020	2.217	24.892	29.317	0.119	0.061	0.060	0.181
ENSG00000157219	HTR5A	578.2	6.1E-17	1.8E-15	3.511	0.479	3.232	4.064	0.000	0.000	0.000	0.000
ENSG00000185633	NDUFA4L2	835	9.8E-20	4.8E-18	5.470	0.792	4.763	6.326	0.000	0.000	0.000	0.000
ENSG00000258667	HIF1A-AS2	1201.1	7.1E-23	5.9E-21	41.351	2.988	37.936	43.483	0.000	0.000	0.000	0.000
ENSG00000220575	HTR5A-AS1	1214.8	1.2E-21	8.3E-20	8.160	0.334	7.789	8.437	0.000	0.000	0.000	0.000

Overlap Results

Collection(s):	H
# overlaps shown:	10
# genesets in collections:	50
# genes in comparison (n):	1704
# genes in universe (N):	45956

Gene Set Name	# Genes in G	Description	# Genes in Ove	k/K
HALLMARK_HYPOXIA	200	Genes up-regul	102	0.51
HALLMARK_TNFA_SIGNALING_VIA_NFKB	200	Genes regulate	81	0.405
HALLMARK_MTORC1_SIGNALING	200	Genes up-regul	64	0.32
HALLMARK_CHOLESTEROL_HOMEOSTASIS	74	Genes involved	41	0.5541
HALLMARK_EPITHELIAL_MESENCHYMAL_TRANSITIC	200	Genes defining	56	0.28
HALLMARK_GLYCOLYSIS	200	Genes encoding	54	0.27
HALLMARK_P53_PATHWAY	200	Genes involved	53	0.265
HALLMARK_MYOGENESIS	200	Genes involved	43	0.215
HALLMARK_APOPTOSIS	161	Genes mediatir	38	0.236
HALLMARK_ESTROGEN_RESPONSE_EARLY	200	Genes defining	42	0.21

Gene/Gene Set Overlap Matrix

Entrez Gene Id	Gene Symbo	Gene Descriptic	HALLMARK_HY	HALLMARK_TN
4783	NFIL3	nuclear factor,	HALLMARK_HY	HALLMARK_TN
1026	CDKN1A	cyclin-depende	HALLMARK_HY	HALLMARK_TN
23645	PPP1R15A	protein phosph	HALLMARK_HY	HALLMARK_TN
8553	BHLHE40	basic helix-loop	HALLMARK_HY	HALLMARK_TN
6515	SLC2A3	solute carrier fa	HALLMARK_HY	HALLMARK_TN
467	ATF3	activating trans	HALLMARK_HY	HALLMARK_TNI
10957	PNRC1	proline-rich nuc	HALLMARK_HY	HALLMARK_TNI
7422	VEGFA	vascular endoth	HALLMARK_HY	HALLMARK_TNI
3725	JUN	jun proto-oncog	HALLMARK_HY	HALLMARK_TNI
5054	SERPINE1	serpin peptidas	HALLMARK_HY	HALLMARK_TNI
3491	CYR61	cysteine-rich, a	HALLMARK_HY	HALLMARK_TNI
7128	TNFAIP3	tumor necrosis	HALLMARK_HY	HALLMARK_TNI
8870	IER3	immediate earl	HALLMARK_HY	HALLMARK_TNI
2353	FOS	FBJ murine oste	HALLMARK_HY	HALLMARK_TNI
694	BTG1	B-cell transloca	HALLMARK_HY	HALLMARK_TNI
25976	TIPARP	TCDD-inducible	HALLMARK_HY	HALLMARK_TNI
1843	DUSP1	dual specificity	HALLMARK_HY	HALLMARK_TNI
1316	KLF6	Kruppel-like fac	HALLMARK_HY	HALLMARK_TNI
23764	MAFF	v-maf musculoa	HALLMARK_HY	HALLMARK_TNI
7538	ZFP36	zinc finger prot	HALLMARK_HY	HALLMARK_TNI
5209	PFKFB3	6-phosphofruct	HALLMARK_HY	HALLMARK_TNI
1942	EFNA1	ephrin-A1	HALLMARK_HY	HALLMARK_TNI
2355	FOSL2	FOS-like antiger	HALLMARK_HY	HALLMARK_TNI
57007	CXCR7	chemokine (C-X	HALLMARK_HY	HALLMARK_TNI
54541	DDIT4	DNA-damage-ir	HALLMARK_HYPOXIA	
7436	VLDLR	very low densit	HALLMARK_HYPOXIA	
3099	HK2	hexokinase 2	HALLMARK_HYPOXIA	
5033	P4HA1	prolyl 4-hydrox	HALLMARK_HYPOXIA	

30001	ERO1L	ERO1-like (S. ce	HALLMARK_HYPOXIA
7852	CXCR4	chemokine (C-X	HALLMARK_HYPOXIA
226	ALDOA	aldolase A, fruc	HALLMARK_HYPOXIA
3939	LDHA	lactate dehydro	HALLMARK_HYPOXIA
6781	STC1	stanniocalcin 1	HALLMARK_HYPOXIA
5230	PGK1	phosphoglycera	HALLMARK_HYPOXIA
2023	ENO1	enolase 1, (alph	HALLMARK_HYPOXIA
7167	TPI1	triosephosphat	HALLMARK_HYPOXIA
1649	DDIT3	DNA-damage-in	HALLMARK_HYPOXIA
6513	SLC2A1	solute carrier fa	HALLMARK_HYPOXIA
2632	GBE1	glucan (1,4-alph	HALLMARK_HYPOXIA
5211	PFKL	phosphofructok	HALLMARK_HYPOXIA
5236	PGM1	phosphoglucom	HALLMARK_HYPOXIA
5163	PDK1	pyruvate dehyd	HALLMARK_HYPOXIA
2821	GPI	glucose-6-phos	HALLMARK_HYPOXIA
2597	GAPDH	glyceraldehyde	HALLMARK_HYPOXIA
26136	TES	testis derived tr	HALLMARK_HYPOXIA
5165	PDK3	pyruvate dehyd	HALLMARK_HYPOXIA
230	ALDOC	aldolase C, fruc	HALLMARK_HYPOXIA
54206	ERRFI1	ERBB receptor f	HALLMARK_HYPOXIA
2026	ENO2	enolase 2 (gam	HALLMARK_HYPOXIA
7045	TGFBI	transforming gr	HALLMARK_HYPOXIA
1490	CTGF	connective tissu	HALLMARK_HYPOXIA
4015	LOX	lysyl oxidase	HALLMARK_HYPOXIA
26355	FAM162A	family with seq	HALLMARK_HYPOXIA
8614	STC2	stanniocalcin 2	HALLMARK_HYPOXIA
8497	PPFIA4	protein tyrosine	HALLMARK_HYPOXIA
3669	ISG20	interferon stimu	HALLMARK_HYPOXIA
136	ADORA2B	adenosine A2b	HALLMARK_HYPOXIA
10370	CITED2	Cbp/p300-inter	HALLMARK_HYPOXIA
51129	ANGPTL4	angiopoietin-lik	HALLMARK_HYPOXIA
4601	MXI1	MAX interactor	HALLMARK_HYPOXIA
8974	P4HA2	prolyl 4-hydrox	HALLMARK_HYPOXIA
4282	MIF	macrophage mi	HALLMARK_HYPOXIA
5214	PFKP	phosphofructok	HALLMARK_HYPOXIA
8819	SAP30	Sin3A-associate	HALLMARK_HYPOXIA
5066	PAM	peptidylglycine	HALLMARK_HYPOXIA
10570	DPYSL4	dihydropyrimid	HALLMARK_HYPOXIA
1944	EFNA3	ephrin-A3	HALLMARK_HYPOXIA
2131	EXT1	exostosin 1	HALLMARK_HYPOXIA
23036	ZNF292	zinc finger prot	HALLMARK_HYPOXIA
55139	ANKZF1	ankyrin repeat a	HALLMARK_HYPOXIA
55276	PGM2	phosphoglucom	HALLMARK_HYPOXIA
10397	NDRG1	N-myc downstr	HALLMARK_HYPOXIA
2309	FOXO3	forkhead box O	HALLMARK_HYPOXIA
4627	MYH9	myosin, heavy c	HALLMARK_HYPOXIA
1837	DTNA	dystrobrevin, al	HALLMARK_HYPOXIA
5507	PPP1R3C	protein phosph	HALLMARK_HYPOXIA
5837	PYGM	phosphorylase,	HALLMARK_HYPOXIA
857	CAV1	caveolin 1, cave	HALLMARK_HYPOXIA
665	BNIP3L	BCL2/adenoviru	HALLMARK_HYPOXIA
6478	SIAH2	seven in absent	HALLMARK_HYPOXIA

302	ANXA2	annexin A2	HALLMARK_HYPOXIA
6095	RORA	RAR-related or	HALLMARK_HYPOXIA
133	ADM	adrenomedullin	HALLMARK_HYPOXIA
7163	TPD52	tumor protein I	HALLMARK_HYPOXIA
4214	MAP3K1	mitogen-activat	HALLMARK_HYPOXIA
284119	PTRF	polymerase I ar	HALLMARK_HYPOXIA
6576	SLC25A1	solute carrier fa	HALLMARK_HYPOXIA
901	CCNG2	cyclin G2	HALLMARK_HYPOXIA
5155	PDGFB	platelet-derivec	HALLMARK_HYPOXIA
7511	XPNPEP1	X-prolyl aminop	HALLMARK_HYPOXIA
8609	KLF7	Kruppel-like fac	HALLMARK_HYPOXIA
1466	CSRP2	cysteine and gly	HALLMARK_HYPOXIA
23210	JMJD6	jumonji domair	HALLMARK_HYPOXIA
23327	NEDD4L	neural precursc	HALLMARK_HYPOXIA
25819	CCRN4L	CCR4 carbon ca	HALLMARK_HYPOXIA
26118	WSB1	WD repeat and	HALLMARK_HYPOXIA
3423	IDS	iduronate 2-sul	HALLMARK_HYPOXIA
54800	KLHL24	kelch-like 24 (D	HALLMARK_HYPOXIA
55076	TMEM45A	transmembrane	HALLMARK_HYPOXIA
55818	KDM3A	lysine (K)-specil	HALLMARK_HYPOXIA
63827	BCAN	brevican	HALLMARK_HYPOXIA
8987	STBD1	starch binding c	HALLMARK_HYPOXIA
3949	LDLR	low density lipoprotein receptor	HALLMARK_TN
7832	BTG2	BTG family, member 2	HALLMARK_TN
10135	NAMPT	nicotinamide phosphoribosyltra	HALLMARK_TN
182	JAG1	jagged 1	HALLMARK_TNI
960	CD44	CD44 molecule (Indian blood gr	HALLMARK_TNI
1647	GADD45A	growth arrest and DNA-damage	HALLMARK_TNI
4616	GADD45B	growth arrest and DNA-damage	HALLMARK_TNI
388	RHOB	ras homolog gene family, meml	HALLMARK_TNI
3624	INHBA	inhibin, beta A	HALLMARK_TNI
3398	ID2	inhibitor of DNA binding 2, dom	HALLMARK_TNI
2669	GEM	GTP binding protein overexpres	HALLMARK_TNI
3371	TNC	tenascin C	HALLMARK_TNI
5806	PTX3	pentraxin 3, long	HALLMARK_TNI
1839	HBEGF	heparin-binding EGF-like growtl	HALLMARK_TNI
8877	SPHK1	sphingosine kinase 1	HALLMARK_TNI
650	BMP2	bone morphogenetic protein 2	HALLMARK_TNI
3552	IL1A	interleukin 1, alpha	HALLMARK_TNI
9314	KLF4	Kruppel-like factor 4 (gut)	HALLMARK_TNI
3976	LIF	leukemia inhibitory factor (chol	HALLMARK_TNI
4084	MXD1	MAX dimerization protein 1	HALLMARK_TNI
5791	PTPRE	protein tyrosine phosphatase, r	HALLMARK_TNI
10769	PLK2	polo-like kinase 2	HALLMARK_TNI
51278	IER5	immediate early response 5	HALLMARK_TNI
1960	EGR3	early growth response 3	HALLMARK_TNI
4170	MCL1	myeloid cell leukemia sequence	HALLMARK_TNI
4609	MYC	v-myc myelocytomatosis viral o	HALLMARK_TNI
7071	KLF10	Kruppel-like factor 10	HALLMARK_TNI
3280	HES1	hairy and enhancer of split 1, (I	HALLMARK_TNI
22822	PHLDA1	pleckstrin homology-like domai	HALLMARK_TNI
23258	DENND5A	DENN/MADD domain containin	HALLMARK_TNI

3383	ICAM1	intercellular adhesion molecule	HALLMARK_TNI
1906	EDN1	endothelin 1	HALLMARK_TNI
5734	PTGER4	prostaglandin E receptor 4 (sub	HALLMARK_TNI
25816	TNFAIP8	tumor necrosis factor, alpha-inc	HALLMARK_TNI
5328	PLAU	plasminogen activator, urokina:	HALLMARK_TNI
604	BCL6	B-cell CLL/lymphoma 6	HALLMARK_TNI
3726	JUNB	jun B proto-oncogene	HALLMARK_TNI
2354	FOSB	FBJ murine osteosarcoma viral	HALLMARK_TNI
3164	NR4A1	nuclear receptor subfamily 4, g	HALLMARK_TNI
1847	DUSP5	dual specificity phosphatase 5	HALLMARK_TNI
7280	TUBB2A	tubulin, beta 2A class IIa	HALLMARK_TNI
602	BCL3	B-cell CLL/lymphoma 3	HALLMARK_TNI
4929	NR4A2	nuclear receptor subfamily 4, g	HALLMARK_TNI
10318	TNIP1	TNFAIP3 interacting protein 1	HALLMARK_TNI
10725	NFAT5	nuclear factor of activated T-ce	HALLMARK_TNI
11182	SLC2A6	solute carrier family 2 (facilitate	HALLMARK_TNI
1958	EGR1	early growth response 1	HALLMARK_TNI
1959	EGR2	early growth response 2	HALLMARK_TNI
23135	KDM6B	lysine (K)-specific demethylase	HALLMARK_TNI
23529	CLCF1	cardiotrophin-like cytokine fact	HALLMARK_TNI
24147	FJX1	four jointed box 1 (Drosophila)	HALLMARK_TNI
4791	NFKB2	nuclear factor of kappa light po	HALLMARK_TNI
5187	PER1	period homolog 1 (Drosophila)	HALLMARK_TNI
5271	SERPINB8	serpin peptidase inhibitor, clad	HALLMARK_TNI
687	KLF9	Kruppel-like factor 9	HALLMARK_TNI
7262	PHLDA2	pleckstrin homology-like domai	HALLMARK_TNI
8061	FOSL1	FOS-like antigen 1	HALLMARK_TNI
57761	TRIB3	tribbles homolog 3 (Drosophila)	
6319	SCD	stearoyl-CoA desaturase (delta-9-desaturase)	
10682	EBP	emopamil binding protein (sterol isomerase)	
6309	SC5DL	sterol-C5-desaturase (ERG3 delta-5-desaturase	
1717	DHCR7	7-dehydrocholesterol reductase	
928	CD9	CD9 molecule	
3422	IDI1	isopentenyl-diphosphate delta isomerase 1	
3157	HMGCS1	3-hydroxy-3-methylglutaryl-CoA synthase 1 (sol	
3156	HMGCR	3-hydroxy-3-methylglutaryl-CoA reductase	
9415	FADS2	fatty acid desaturase 2	
27346	TMEM97	transmembrane protein 97	
6713	SQLE	squalene epoxidase	
134429	STARD4	StAR-related lipid transfer (START) domain cont	
1595	CYP51A1	cytochrome P450, family 51, subfamily A, polyp	
5352	PLOD2	procollagen-lysine, 2-oxoglutarate 5-dioxygena:	
112399	EGLN3	egl nine homolog 3 (C. elegans)	
23657	SLC7A11	solute carrier family 7 (anionic amino acid trans	
120	ADD3	adducin 3 (gamma)	
60481	ELOVL5	ELOVL fatty acid elongase 5	
3638	INSIG1	insulin induced gene 1	
3488	IGFBP5	insulin-like growth factor binding protein 5	
1718	DHCR24	24-dehydrocholesterol reductase	
2181	ACSL3	acyl-CoA synthetetase long-chain family member	
440	ASNS	asparagine synthetase (glutamine-hydrolyzing)	
7037	TFRC	transferrin receptor (p90, CD71)	

51109	RDH11	retinol dehydrogenase 11 (all-trans/9-cis/11-cis
8935	SKAP2	src kinase associated phosphoprotein 2
47	ACLY	ATP citrate lyase
79071	ELOVL6	ELOVL fatty acid elongase 6
80777	CYB5B	cytochrome b5 type B (outer mitochondrial me
10797	MTHFD2	methylenetetrahydrofolate dehydrogenase (NA
9695	EDEM1	ER degradation enhancer, mannosidase alpha-li
586	BCAT1	branched chain amino-acid transaminase 1, cyt
23062	GGA2	golgi-associated, gamma adaptin ear containing
54843	SYTL2	synaptotagmin-like 2
51330	TNFRSF12A	tumor necrosis factor receptor superfamily, me
50814	NSDHL	NAD(P) dependent steroid dehydrogenase-like
2224	FDPS	farnesyl diphosphate synthase
3958	LGALS3	lectin, galactoside-binding, soluble, 3
552	AVPR1A	arginine vasopressin receptor 1A
7869	SEMA3B	sema domain, immunoglobulin domain (Ig), sho
2222	FDFT1	farnesyl-diphosphate farnesyltransferase 1
2194	FASN	fatty acid synthase
214	ALCAM	activated leukocyte cell adhesion molecule
39	ACAT2	acetyl-CoA acetyltransferase 2
51478	HSD17B7	hydroxysteroid (17-beta) dehydrogenase 7
4597	MVD	mevalonate (diphospho) decarboxylase
308	ANXA5	annexin A5
132864	CPEB2	cytoplasmic polyadenylation element binding p
4047	LSS	lanosterol synthase (2,3-oxidosqualene-lanoste
4598	MVK	mevalonate kinase
55902	ACSS2	acyl-CoA synthetase short-chain family member
5833	PCYT2	phosphate cytidylyltransferase 2, ethanolamine
6721	SREBF2	sterol regulatory element binding transcription
4907	NT5E	5'-nucleotidase, ecto (CD73)
7040	TGFB1	transforming growth factor, beta 1
1292	COL6A2	collagen, type VI, alpha 2
6876	TAGLN	transgelin
8038	ADAM12	ADAM metallopeptidase domain 12
7078	TIMP3	TIMP metallopeptidase inhibitor 3
1301	COL11A1	collagen, type XI, alpha 1
5376	PMP22	peripheral myelin protein 22
627	BDNF	brain-derived neurotrophic factor
6591	SNAI2	snail homolog 2 (Drosophila)
6696	SPP1	secreted phosphoprotein 1
2014	EMP3	epithelial membrane protein 3
3678	ITGA5	integrin, alpha 5 (fibronectin receptor, alpha po
7980	TFPI2	tissue factor pathway inhibitor
3956	LGALS1	lectin, galactoside-binding, soluble, 1
1004	CDH6	cadherin 6, type 2, K-cadherin (fetal kidney)
9353	SLIT2	slit homolog 2 (Drosophila)
3485	IGFBP2	insulin-like growth factor binding protein 2, 36k
7057	THBS1	thrombospondin 1
7857	SCG2	secretogranin II
23705	CADM1	cell adhesion molecule 1
667	DST	dystonin
1000	CDH2	cadherin 2, type 1, N-cadherin (neuronal)

10272	FSTL3	follistatin-like 3 (secreted glycoprotein)
11010	GLIPR1	GLI pathogenesis-related 1
1294	COL7A1	collagen, type VII, alpha 1
1303	COL12A1	collagen, type XII, alpha 1
26585	GREM1	gremlin 1
30008	EFEMP2	EGF containing fibulin-like extracellular matrix p
4017	LOXL2	lysyl oxidase-like 2
50863	NTM	neurotrimin
5270	SERPINE2	serpin peptidase inhibitor, clade E (nexin, plasm
59	ACTA2	actin, alpha 2, smooth muscle, aorta
7431	VIM	vimentin
800	CALD1	caldesmon 1
9235	IL32	interleukin 32
1999	ELF3	E74-like factor 3 (ets domain transcription facto
283	ANG	angiogenin, ribonuclease, RNase A family, 5
3161	HMMR	hyaluronan-mediated motility receptor (RHAMI
4351	MPI	mannose phosphate isomerase
5223	PGAM1	phosphoglycerate mutase 1 (brain)
5315	PKM2	pyruvate kinase, muscle
5318	PKP2	plakophilin 2
5836	PYGL	phosphorylase, glycogen, liver
6676	SPAG4	sperm associated antigen 4
8996	NOL3	nucleolar protein 3 (apoptosis repressor with C/
90161	HS6ST2	heparan sulfate 6-O-sulfotransferase 2
9048	ARTN	artemin
9123	SLC16A3	solute carrier family 16, member 3 (monocarbo:
351	APP	amyloid beta (A4) precursor protein
3691	ITGB4	integrin, beta 4
1012	CDH13	cadherin 13, H-cadherin (heart)
894	CCND2	cyclin D2
54884	RETSAT	retinol saturase (all-trans-retinol 13,14-reducta:
10628	TXNIP	thioredoxin interacting protein
2810	SFN	stratifin
6004	RGS16	regulator of G-protein signaling 16
3732	CD82	CD82 molecule
22824	HSPA4L	heat shock 70kDa protein 4-like
6281	S100A10	S100 calcium binding protein A10
1030	CDKN2B	cyclin-dependent kinase inhibitor 2B (p15, inhib
7378	UPP1	uridine phosphorylase 1
1969	EPHA2	EPH receptor A2
1643	DDB2	damage-specific DNA binding protein 2, 48kDa
5429	POLH	polymerase (DNA directed), eta
1263	PLK3	polo-like kinase 3
27165	GLS2	glutaminase 2 (liver, mitochondrial)
27244	SESN1	sestrin 1
4808	NHLH2	nescient helix loop helix 2
55270	NUDT15	nudix (nucleoside diphosphate linked moiety X)
57763	ANKRA2	ankyrin repeat, family A (RFXANK-like), 2
5784	PTPN14	protein tyrosine phosphatase, non-receptor typ
5900	RALGDS	ral guanine nucleotide dissociation stimulator
64393	ZMAT3	zinc finger, matrin-type 3
64787	EPS8L2	EPS8-like 2

26353	HSPB8	heat shock 22kDa protein 8
29970	SCHIP1	schwannomin interacting protein 1
5621	PRNP	prion protein
2180	ACSL1	acyl-CoA synthetase long-chain family member
88	ACTN2	actinin, alpha 2
58	ACTA1	actin, alpha 1, skeletal muscle
89	ACTN3	actinin, alpha 3
8082	SSPN	sarcospan (Kras oncogene-associated gene)
25937	WWTR1	WW domain containing transcription regulator
43	ACHE	acetylcholinesterase
23175	LPIN1	lipin 1
11155	LDB3	LIM domain binding 3
11156	PTP4A3	protein tyrosine phosphatase type IVA, member
1266	CNN3	calponin 3, acidic
1489	CTF1	cardiotrophin 1
2049	EPHB3	EPH receptor B3
2170	FABP3	fatty acid binding protein 3, muscle and heart (r
3490	IGFBP7	insulin-like growth factor binding protein 7
4620	MYH2	myosin, heavy chain 2, skeletal muscle, adult
58498	MYL7	myosin, light chain 7, regulatory
58529	MYOZ1	myozenin 1
688	KLF5	Kruppel-like factor 5 (intestinal)
8736	MYOM1	myomesin 1, 185kDa
2012	EMP1	epithelial membrane protein 1
836	CASP3	caspase 3, apoptosis-related cysteine peptidase
8900	CCNA1	cyclin A1
3005	H1F0	H1 histone family, member 0
5526	PPP2R5B	protein phosphatase 2, regulatory subunit B', be
301	ANXA1	annexin A1
7465	WEE1	WEE1 homolog (S. pombe)
4000	LMNA	lamin A/C
8682	PEA15	phosphoprotein enriched in astrocytes 15
9890	LPPR4	lipid phosphate phosphatase-related protein typ
3480	IGF1R	insulin-like growth factor 1 receptor
3880	KRT19	keratin 19
27295	PDLIM3	PDZ and LIM domain 3
11098	PRSS23	protease, serine, 23
2317	FLNB	filamin B, beta
10202	DHRS2	dehydrogenase/reductase (SDR family) member
11145	PLA2G16	phospholipase A2, group XVI
26509	MYOF	myoferlin
4277	MICB	MHC class I polypeptide-related sequence B
56603	CYP26B1	cytochrome P450, family 26, subfamily B, polyp
6658	SOX3	SRY (sex determining region Y)-box 3
799	CALCR	calcitonin receptor
3856	KRT8	keratin 8
23030	KDM4B	lysine (K)-specific demethylase 4B
2674	GFRA1	GDNF family receptor alpha 1
347733	TUBB2B	tubulin, beta 2B class IIb
375449	MAST4	microtubule associated serine/threonine kinase
55191	NADSYN1	NAD synthetase 1
56944	OLFML3	olfactomedin-like 3

64284	RAB17	RAB17, member RAS oncogene family
6505	SLC1A1	solute carrier family 1 (neuronal/epithelial high
8829	NRP1	neuropilin 1
3037	HAS2	hyaluronan synthase 2
11099	PTPN21	protein tyrosine phosphatase, non-receptor typ
7035	TFPI	tissue factor pathway inhibitor (lipoprotein-asso
5002	SLC22A18	solute carrier family 22, member 18
2650	GCNT1	glucosaminyl (N-acetyl) transferase 1, core 2
5737	PTGFR	prostaglandin F receptor (FP)
11044	PAPD7	PAP associated domain containing 7
10659	CELF2	CUGBP, Elav-like family member 2
23229	ARHGEF9	Cdc42 guanine nucleotide exchange factor (GEF
23576	DDAH1	dimethylarginine dimethylaminohydrolase 1
2869	GRK5	G protein-coupled receptor kinase 5
4131	MAP1B	microtubule-associated protein 1B
5797	PTPRM	protein tyrosine phosphatase, receptor type, M
773	CACNA1A	calcium channel, voltage-dependent, P/Q type,
5732	PTGER2	prostaglandin E receptor 2 (subtype EP2), 53kDa
3662	IRF4	interferon regulatory factor 4
9446	GSTO1	glutathione S-transferase omega 1
4643	MYO1E	myosin IE
126669	SHE	Src homology 2 domain containing E
3177	SLC29A2	solute carrier family 29 (nucleoside transporters
23208	SYT11	synaptotagmin XI
3664	IRF6	interferon regulatory factor 6
64116	SLC39A8	solute carrier family 39 (zinc transporter), mem
79026	AHNAK	AHNAK nucleoprotein
4067	LYN	v-yes-1 Yamaguchi sarcoma viral related oncoge
3249	HPN	hepsin
7855	FZD5	frizzled family receptor 5
10148	EBI3	Epstein-Barr virus induced 3
166929	SGMS2	sphingomyelin synthase 2
3656	IRAK2	interleukin-1 receptor-associated kinase 2
3696	ITGB8	integrin, beta 8
5027	P2RX7	purinergic receptor P2X, ligand-gated ion chann
558	AXL	AXL receptor tyrosine kinase
5724	PTAFR	platelet-activating factor receptor
60675	PROK2	prokineticin 2
7439	BEST1	bestrophin 1
9966	TNFSF15	tumor necrosis factor (ligand) superfamily, mem
2707	GJB3	gap junction protein, beta 3, 31kDa
4665	NAB2	NGFI-A binding protein 2 (EGR1 binding protein
56521	DNAJC12	DnaJ (Hsp40) homolog, subfamily C, member 12
57834	CYP4F11	cytochrome P450, family 4, subfamily F, polypep
9547	CXCL14	chemokine (C-X-C motif) ligand 14
22936	ELL2	elongation factor, RNA polymerase II, 2
6611	SMS	spermine synthase
84159	ARID5B	AT rich interactive domain 5B (MRF1-like)
6197	RPS6KA3	ribosomal protein S6 kinase, 90kDa, polypeptide
10627	MYL12A	myosin, light chain 12A, regulatory, non-sarcom
2005	ELK4	ELK4, ETS-domain protein (SRF accessory protei
55768	NGLY1	N-glycanase 1

8916	HERC3	hect domain and RLD 3
9510	ADAMTS1	ADAM metallopeptidase with thrombospondin
4319	MMP10	matrix metallopeptidase 10 (stromelysin 2)
11184	MAP4K1	mitogen-activated protein kinase kinase kinase
9734	HDAC9	histone deacetylase 9
9892	SNAP91	synaptosomal-associated protein, 91kDa homol
9935	MAFB	v-maf musculoaponeurotic fibrosarcoma oncog
28951	TRIB2	tribbles homolog 2 (Drosophila)
3800	KIF5C	kinesin family member 5C
3936	LCP1	lymphocyte cytosolic protein 1 (L-plastin)
54462	FAM190B	family with sequence similarity 190, member B
5593	PRKG2	protein kinase, cGMP-dependent, type II
5801	PTPRR	protein tyrosine phosphatase, receptor type, R
639	PRDM1	PR domain containing 1, with ZNF domain
64123	ELTD1	EGF, latrophilin and seven transmembrane dom
8780	RIOK3	RIO kinase 3 (yeast)
10133	OPTN	optineurin
54498	SMOX	spermine oxidase
81689	ISCA1	iron-sulfur cluster assembly 1 homolog (S. cerev
23002	DAAM1	dishevelled associated activator of morphogene
23762	OSBP2	oxysterol binding protein 2
25897	RNF19A	ring finger protein 19A
26502	NARF	nuclear prelamin A recognition factor
286	ANK1	ankyrin 1, erythrocytic
51312	SLC25A37	solute carrier family 25, member 37
55532	SLC30A10	solute carrier family 30, member 10
56180	MOSPD1	motile sperm domain containing 1
6622	SNCA	synuclein, alpha (non A4 component of amyloid
6710	SPTB	spectrin, beta, erythrocytic
80853	JHDM1D	jumonji C domain containing histone demethyla
8301	PICALM	phosphatidylinositol binding clathrin assembly p
831	CAST	calpastatin
9429	ABCG2	ATP-binding cassette, sub-family G (WHITE), me
2182	ACSL4	acyl-CoA synthetase long-chain family member
3155	HMGCL	3-hydroxymethyl-3-methylglutaryl-CoA lyase
387893	SETD8	SET domain containing (lysine methyltransferas
131076	CCDC58	coiled-coil domain containing 5
2954	GSTZ1	glutathione transferase zeta 1
5793	PTPRG	protein tyrosine phosphatase, receptor type, G
58476	TP53INP2	tumor protein p53 inducible nuclear protein 2
8608	RDH16	retinol dehydrogenase 16 (all-trans)
8754	ADAM9	ADAM metallopeptidase domain 9
7414	VCL	vinculin
11096	ADAMTS5	ADAM metallopeptidase with thrombospondin
1308	COL17A1	collagen, type XVII, alpha 1
2318	FLNC	filamin C, gamma
257194	NEGR1	neuronal growth regulator 1
2886	GRB7	growth factor receptor-bound protein 7
3675	ITGA3	integrin, alpha 3 (antigen CD49C, alpha 3 subun
5010	CLDN11	claudin 11
51474	LIMA1	LIM domain and actin binding 1
57863	CADM3	cell adhesion molecule 3

6237	RRAS	related RAS viral (r-ras) oncogene homolog
65266	WNK4	WNK lysine deficient protein kinase 4
7087	ICAM5	intercellular adhesion molecule 5, telencephalir
7791	ZYX	zyxin
81607	PVRL4	poliovirus receptor-related 4
80273	GRPEL1	GrpE-like 1, mitochondrial (E. coli)
64859	OBFC2A	oligonucleotide/oligosaccharide-binding fold co
204	AK2	adenylate kinase 2
593	BCKDHA	branched chain keto acid dehydrogenase E1, alp
23788	MTCH2	mitochondrial carrier 2
284	ANGPT1	angiopoietin 1
65124	ANKRD57	ankyrin repeat domain 57
7316	UBC	ubiquitin C
7423	VEGFB	vascular endothelial growth factor B
3175	ONECUT1	one cut homeobox 1
6782	HSPA13	heat shock protein 70kDa family, member 13
255738	PCSK9	proprotein convertase subtilisin/kexin type 9
54704	PDP1	pyruvate dehyrogenase phosphatase catalytic s
10019	SH2B3	SH2B adaptor protein 3
1368	CPM	carboxypeptidase M
23348	DOCK9	dedicator of cytokinesis 9
30819	KCNIP2	Kv channel interacting protein 2
3988	LIPA	lipase A, lysosomal acid, cholesterol esterase
4179	CD46	CD46 molecule, complement regulatory protein
51440	HPCAL4	hippocalcin like 4
9806	SPOCK2	sparc/osteonectin, cwcv and kazal-like domains
2161	F12	coagulation factor XII (Hageman factor)
5028	P2RY1	purinergic receptor P2Y, G-protein coupled, 1
9615	GDA	guanine deaminase
4864	NPC1	Niemann-Pick disease, type C1
10998	SLC27A5	solute carrier family 27 (fatty acid transporter),
1734	DIO2	deiodinase, iodothyronine, type II
23461	ABCA5	ATP-binding cassette, sub-family A (ABC1), mem
4734	NEDD4	neural precursor cell expressed, developmental
50640	PNPLA8	patatin-like phospholipase domain containing 8
51268	PIPOX	pipecolic acid oxidase
9023	CH25H	cholesterol 25-hydroxylase
9241	NOG	noggin
2081	ERN1	endoplasmic reticulum to nucleus signaling 1
55062	WIPI1	WD repeat domain, phosphoinositide interactin
79094	CHAC1	ChaC, cation transport regulator homolog 1 (E. c
9451	EIF2AK3	eukaryotic translation initiation factor 2-alpha k
3135	HLA-G	major histocompatibility complex, class I, G
7903	ST8SIA4	ST8 alpha-N-acetyl-neuraminide alpha-2,8-sialyl
1615	DARS	aspartyl-tRNA synthetase
3589	IL11	interleukin 11
9437	NCR1	natural cytotoxicity triggering receptor 1
51735	RAPGEF6	Rap guanine nucleotide exchange factor (GEF) 6
55024	BANK1	B-cell scaffold protein with ankyrin repeats 1
57162	PELI1	pellino homolog 1 (Drosophila)
3897	L1CAM	L1 cell adhesion molecule
1382	CRABP2	cellular retinoic acid binding protein 2

774	CACNA1B	calcium channel, voltage-dependent, N type, al
1592	CYP26A1	cytochrome P450, family 26, subfamily A, polyp
316	AOX1	aldehyde oxidase 1
3931	LCAT	lecithin-cholesterol acyltransferase
55502	HES6	hairy and enhancer of split 6 (Drosophila)
6560	SLC12A4	solute carrier family 12 (potassium/chloride tra
9361	LONP1	lon peptidase 1, mitochondrial
5071	PARK2	parkinson protein 2, E3 ubiquitin protein ligase
50832	TAS2R4	taste receptor, type 2, member 4
51168	MYO15A	myosin XVA
55220	KLHDC8A	kelch domain containing 8A
55890	GPRC5C	G protein-coupled receptor, family C, group 5, r
5625	PRODH	proline dehydrogenase (oxidase) 1
57451	ODZ2	odz, odd Oz/ten-m homolog 2 (Drosophila)
6262	RYR2	ryanodine receptor 2 (cardiac)
6620	SNCB	synuclein, beta
778	CACNA1F	calcium channel, voltage-dependent, L type, alp
1845	DUSP3	dual specificity phosphatase 3
5563	PRKAA2	protein kinase, AMP-activated, alpha 2 catalytic
84441	MAML2	mastermind-like 2 (Drosophila)
11104	KATNA1	katanin p60 (ATPase containing) subunit A 1
11133	KPTN	kaptin (actin binding protein)
11135	CDC42EP1	CDC42 effector protein (Rho GTPase binding) 1
11190	CEP250	centrosomal protein 250kDa
23022	PALLD	palladin, cytoskeletal associated protein
5829	PXN	paxillin
8470	SORBS2	sorbin and SH3 domain containing 2
8874	ARHGEF7	Rho guanine nucleotide exchange factor (GEF) 7
9411	ARHGAP29	Rho GTPase activating protein 29
1032	CDKN2D	cyclin-dependent kinase inhibitor 2D (p19, inhib
10587	TXNRD2	thioredoxin reductase 2
3087	HHEX	hematopoietically expressed homeobox
2581	GALC	galactosylceramidase
664	BNIP3	BCL2/adenovirus E1B 19kDa interacting protein
858	CAV2	caveolin 2
9805	SCRN1	secernin 1
3953	LEPR	leptin receptor
83729	INHBE	inhibin, beta E
246	ALOX15	arachidonate 15-lipoxygenase
3361	HTR5A	5-hydroxytryptamine (serotonin) receptor 5A
5443	POMC	proopiomelanocortin
54760	PCSK4	proprotein convertase subtilisin/kexin type 4
22854	NTNG1	netrin G1
3930	LBR	lamin B receptor
81624	DIAPH3	diaphanous homolog 3 (Drosophila)
9924	PAN2	PAN2 poly(A) specific ribonuclease subunit hom
5126	PCSK2	proprotein convertase subtilisin/kexin type 2
2987	GUK1	guanylate kinase 1
404672	GTF2H5	general transcription factor IIH, polypeptide 5
84265	POLR3GL	polymerase (RNA) III (DNA directed) polypeptid
8731	RNMT	RNA (guanine-7-) methyltransferase
10161	LPAR6	lysophosphatidic acid receptor

135112	NCOA7	nuclear receptor coactivator 7
100037417	DDTL	D-dopachrome tautomerase-like
100113407	TMEM170B	transmembrane protein 170B
100128071	LOC1001280	uncharacterized LOC100128071
100128264	LOC1001282	uncharacterized LOC100128264
100129550	LOC1001295	uncharacterized LOC100129550
100129654	TCF24	transcription factor 24
100129662	LOC1001296	uncharacterized LOC100129662
100129716	LOC1001297	uncharacterized LOC100129716
100129845	LOC1001298	uncharacterized LOC100129845
100129924	LOC1001299	uncharacterized LOC100129924
100129995	LOC1001299	uncharacterized LOC100129995
100130522	LOC1001305	uncharacterized LOC100130522
100130691	LOC1001306	uncharacterized LOC100130691
100130742	LRRC69	leucine rich repeat containing 6
100130950	LOC1001309	uncharacterized LOC100130950
100131089	LOC1001310	uncharacterized LOC100131089
100131138	LOC1001311	uncharacterized LOC100131138
100131347	LOC1001313	RAD52 motif 1 pseudogene
100132594	PGAM1P5	phosphoglycerate mutase 1 pseudogene 5
100132916	FAM159B	family with sequence similarity 159, member B
100133036	FAM95B1	family with sequence similarity 95, member B1
100133091	LOC1001330	uncharacterized LOC100133091
100134229	LOC1001342	uncharacterized LOC100134229
100268168	LOC1002681	uncharacterized LOC100268168
100271846	ERVV-2	endogenous retrovirus group V, member 2
100288175	LOC1002881	uncharacterized LOC100288175
100288198	LOC1002881	uncharacterized LOC100288198
100289098	LOC1002890	uncharacterized LOC100289098
100289211	LOC1002892	uncharacterized LOC100289211
100289279	ATP5A1P9	ATP synthase, H+ transporting, mitochondrial F1
1003	CDH5	cadherin 5, type 2 (vascular endothelium)
100302640	LOC1003026	uncharacterized LOC100302640
100381270	ZBED6	zinc finger, BED-type containing 6
10044	SH2D3C	SH2 domain containing 3C
10045	SH2D3A	SH2 domain containing 3A
100500802	MIR3685	microRNA 3685
100505538	RBM26-AS1	RBM26 antisense RNA 1 (non-protein coding)
100505783	LOC1005057	uncharacterized LOC100505783
100505817	LOC1005058	uncharacterized LOC100505817
100505881	MAGI2-AS3	MAGI2 antisense RNA 3 (non-protein coding)
100506100	LOC1005061	uncharacterized LOC100506100
100506658	OCLN	occludin
100506696	LOC1005066	uncharacterized LOC100506696
100507062	LOC1005070	uncharacterized LOC100507062
100507103	LOC1005071	uncharacterized LOC100507103
100507250	LOC1005072	uncharacterized LOC100507250
100507291	LOC1005072	uncharacterized LOC100507291
100507347	LOC1005073	uncharacterized LOC100507347
100507401	LOC1005074	uncharacterized LOC100507401
100507428	LINC00458	long intergenic non-protein coding RNA 458
100507436	MICA	MHC class I polypeptide-related sequence A

100507475	LOC1005074	uncharacterized LOC100507475
100507487	LOC1005074	uncharacterized LOC100507487
100507495	LOC1005074	uncharacterized LOC100507495
100507501	LOC1005075	uncharacterized LOC100507501
100507582	LOC1005075	uncharacterized LOC100507582
10062	NR1H3	nuclear receptor subfamily 1, group H, member
100642175	SPRY4-IT1	SPRY4 intronic transcript 1 (non-protein coding)
10076	PTPRU	protein tyrosine phosphatase, receptor type, U
10082	GPC6	glypican 6
100862728		
100874002		
100885848		
100996419		
10144	FAM13A	family with sequence similarity 13, member A
10150	MBNL2	muscleblind-like 2 (Drosophila)
10178	ODZ1	odz, odd Oz/ten-m homolog 1 (Drosophila)
101926941		
101926975		
101926987		
101927043		
101927221		
101927288		
101927359		
101927599		
101927931		
101928000		
101928069		
101928136		
101928550		
101928674		
101928837		
101929027		
101929140		
101929147		
101929243		
101929302		
101929333		
101929563		
101929705		
10223	GPA33	glycoprotein A33 (transmembrane)
10233	LRRC23	leucine rich repeat containing 2
10234	LRRC17	leucine rich repeat containing 1
10251	SPRY3	sprouty homolog 3 (Drosophila)
10252	SPRY1	sprouty homolog 1, antagonist of FGF signaling
102723465		
102723548		
102724571		
102724788		
102724826		
10295	BCKDK	branched chain ketoacid dehydrogenase kinase
10344	CCL26	chemokine (C-C motif) ligand 26
10345	TRDN	triadin

104326191		
104355426		
10485	C1orf61	chromosome 1 open reading frame 61
10529	NEBL	nebulette
105369758		
105370526		
105372482		
105379252		
105416157		
10566	AKAP3	A kinase (PRKA) anchor protein 3
10602	CDC42EP3	CDC42 effector protein (Rho GTPase binding) 3
10637	LEFTY1	left-right determination factor 1
10650	SLMO1	slowmo homolog 1 (Drosophila)
106614088		
10669	CGREF1	cell growth regulator with EF-hand domain 1
10670	RRAGA	Ras-related GTP binding A
10675	CSPG5	chondroitin sulfate proteoglycan 5 (neuroglycar
10687	PNMA2	paraneoplastic antigen MA2
10690	FUT9	fucosyltransferase 9 (alpha (1,3) fucosyltransfer
10718	NRG3	neuregulin 3
10763	NES	nestin
10765	KDM5B	lysine (K)-specific demethylase 5B
107985075		
107986266		
10814	CPLX2	complexin 2
10826	C5orf4	chromosome 5 open reading frame 4
10842	PPP1R17	protein phosphatase 1, regulatory subunit 17
10848	PPP1R13L	protein phosphatase 1, regulatory subunit 13 lil
108570035		
10882	C1QL1	complement component 1, q subcomponent-lik
10955	SERINC3	serine incorporator 3
109616976		
109729141		
10981	RAB32	RAB32, member RAS oncogene family
11033	ADAP1	ArfGAP with dual PH domains 1
11043	MID2	midline 2
11045	UPK1A	uroplakin 1A
1106	CHD2	chromodomain helicase DNA binding protein 2
11161	C14orf1	chromosome 14 open reading frame 1
11186	RASSF1	Ras association (RalGDS/AF-6) domain family m
11211	FZD10	frizzled family receptor 10
11221	DUSP10	dual specificity phosphatase 10
11240	PADI2	peptidyl arginine deiminase, type II
11247	NXPH4	neurexophilin 4
112597	LINC00152	long intergenic non-protein coding RNA 152
112942	CCDC104	coiled-coil domain containing 104
1131	CHRM3	cholinergic receptor, muscarinic 3
11314	CD300A	CD300a molecule
113230	LOC113230	uncharacterized LOC113230
113402	SFT2D1	SFT2 domain containing 1
113791	PIK3IP1	phosphoinositide-3-kinase interacting protein 1
114	ADCY8	adenylate cyclase 8 (brain)

114044	MCM3AP-AS	MCM3AP antisense RNA 1 (non-protein coding)
114088	TRIM9	tripartite motif containing 9
114134	SLC2A13	solute carrier family 2 (facilitated glucose transp
114757	CYGB	cytoglobin
114780	PKD1L2	polycystic kidney disease 1-like 2
114786	XKR4	XK, Kell blood group complex subunit-related fa
114805	GALNT13	UDP-N-acetyl-alpha-D-galactosamine:polypeptid
114814	GNRHR2	gonadotropin-releasing hormone (type 2) recep
115111	SLC26A7	solute carrier family 26, member 7
115207	KCTD12	potassium channel tetramerisation domain con
115265	DDIT4L	DNA-damage-inducible transcript 4-like
115330	GPR146	G protein-coupled receptor 146
115650	TNFRSF13C	tumor necrosis factor receptor superfamily, me
115701	ALPK2	alpha-kinase 2
115727	RASGRP4	RAS guanyl releasing protein 4
115948	CCDC151	coiled-coil domain containing 151
116228	FAM36A	family with sequence similarity 36, member A
116328	C8orf34	chromosome 8 open reading frame 34
116441	TM4SF18	transmembrane 4 L six family member 18
116442	RAB39B	RAB39B, member RAS oncogene family
116828	CG030	uncharacterized CG030
116966	WDR17	WD repeat domain 17
116984	ARAP2	ArfGAP with RhoGAP domain, ankyrin repeat ar
117531	TMC1	transmembrane channel-like 1
118472	ZNF511	zinc finger protein 511
1185	CLCN6	chloride channel 6
1195	CLK1	CDC-like kinase 1
119504	ANAPC16	anaphase promoting complex subunit 16
1198	CLK3	CDC-like kinase 3
120227	CYP2R1	cytochrome P450, family 2, subfamily R, polype
120863	DEPDC4	DEP domain containing 4
121457	IKBIP	IKBKB interacting protein
121504	HIST4H4	histone cluster 4, H4
122481	AK7	adenylate kinase 7
122525	C14orf28	chromosome 14 open reading frame 28
122616	C14orf79	chromosome 14 open reading frame 79
122786	FRMD6	FERM domain containing 6
123606	NIPA1	non imprinted in Prader-Willi/Angelman syndro
124056	NOXO1	NADPH oxidase organizer 1
124637	CYB5D1	cytochrome b5 domain containing 1
124923	SGK494	uncharacterized serine/threonine-protein kinas
124944	C17orf49	chromosome 17 open reading frame 49
124976	SPNS2	spinster homolog 2 (Drosophila)
125228	C18orf19	chromosome 18 open reading frame 19
126014	OSCAR	osteoclast associated, immunoglobulin-like rece
126068	ZNF441	zinc finger protein 441
126353	C19orf21	chromosome 19 open reading frame 21
1264	CNN1	calponin 1, basic, smooth muscle
126731	C1orf96	chromosome 1 open reading frame 96
1268	CNR1	cannabinoid receptor 1 (brain)
126917	IFFO2	intermediate filament family orphan 2
127495	LRRC39	leucine rich repeat containing 3

127579	DCST2	DC-STAMP domain containing 2
127707	KLHDC7A	kelch domain containing 7A
127845	GOLT1A	golgi transport 1A
128077	LIX1L	Lix1 homolog (mouse)-like
1295	COL8A1	collagen, type VIII, alpha 1
1299	COL9A3	collagen, type IX, alpha 3
130132	RFTN2	raftlin family member 2
130162	C2orf63	chromosome 2 open reading frame 63
1305	COL13A1	collagen, type XIII, alpha 1
131034	CPNE4	copine IV
131096	KCNH8	potassium voltage-gated channel, subfamily H (
132204	SYNPR	synaptoporin
134265	AFAP1L1	actin filament associated protein 1-like 1
134492	NUDCD2	NudC domain containing 2
135293	PM20D2	peptidase M20 domain containing 2
1375	CPT1B	carnitine palmitoyltransferase 1B (muscle)
137994	LETM2	leucine zipper-EF-hand containing transmembra
138255	C9orf135	chromosome 9 open reading frame 135
138429	PIP5KL1	phosphatidylinositol-4-phosphate 5-kinase-like
139221	MUM1L1	melanoma associated antigen (mutated) 1-like
139596	UPRT	uracil phosphoribosyltransferase (FUR1) homolo
140606	SELM	selenoprotein M
140612	ZFP28	zinc finger protein 28 homolog (mouse)
140679	SLC32A1	solute carrier family 32 (GABA vesicular transpo
140691	TRIM69	tripartite motif containing 69
140733	MACROD2	MACRO domain containing 2
140825	NEURL2	neuralized homolog 2 (Drosophila)
1416	CRYBB2P1	crystallin, beta B2 pseudogene
1428	CRYM	crystallin, mu
143282	FGFBP3	fibroblast growth factor binding protein 3
144132	DNHD1	dynein heavy chain domain 1
144195	SLC2A14	solute carrier family 2 (facilitated glucose transp
144406	WDR66	WD repeat domain 66
144568	A2ML1	alpha-2-macroglobulin-like 1
145482	PTGR2	prostaglandin reductase 2
146691	TOM1L2	target of myb1-like 2 (chicken)
146857	SLFN13	schlafen family member 13
146923	RUNDC1	RUN domain containing 1
147040	KCTD11	potassium channel tetramerisation domain con
147650	LINC00085	long intergenic non-protein coding RNA 85
147660	ZNF578	zinc finger protein 578
147664	ERVV-1	endogenous retrovirus group V, member 1
147700	KLC3	kinesin light chain 3
147837	ZNF563	zinc finger protein 563
147906	DACT3	dapper, antagonist of beta-catenin, homolog 3 (
147965	FAM98C	family with sequence similarity 98, member C
148229	ATP8B3	ATPase, aminophospholipid transporter, class I,
148523	C1orf51	chromosome 1 open reading frame 51
148534	TMEM56	transmembrane protein 56
1486	CTBS	chitobiase, di-N-acetyl-
148641	SLC35F3	solute carrier family 35, member F3
148808	MFSD4	major facilitator superfamily domain containing

149483	CCDC17	coiled-coil domain containing 1
150	ADRA2A	adrenergic, alpha-2A-, receptor
150082	LCA5L	Leber congenital amaurosis 5-like
150159	SLC9B1	solute carrier family 9, subfamily B (cation proto
150290	DUSP18	dual specificity phosphatase 18
150786	WTH3DI	RAB6C-like
151011	10-Sep	septin 10
151176	FAM132B	family with sequence similarity 132, member B
151354	FAM84A	family with sequence similarity 84, member A
151477	LINC00471	long intergenic non-protein coding RNA 471
151556	GPR155	G protein-coupled receptor 155
151835	CPNE9	copine family member IX
153163	MGC32805	uncharacterized protein MGC32805
153222	C5orf41	chromosome 5 open reading frame 41
153469	LOC153469	uncharacterized LOC153469
153768	PRELID2	PRELI domain containing 2
154	ADRB2	adrenergic, beta-2-, receptor, surface
154091	SLC2A12	solute carrier family 2 (facilitated glucose transp
1545	CYP1B1	cytochrome P450, family 1, subfamily B, polype
154661	RUNDC3B	RUN domain containing 3B
154860	LOC154860	uncharacterized LOC154860
157489	SDAD1P1	SDA1 domain containing 1 pseudogene 1
158035	LINC00032	long intergenic non-protein coding RNA 32
158038	LINGO2	leucine rich repeat and Ig domain containing 2
158046	NXNL2	nucleoredoxin-like 2
158056	MAMDC4	MAM domain containing 4
158158	RASEF	RAS and EF-hand domain containing
158160	HSD17B7P2	hydroxysteroid (17-beta) dehydrogenase 7 pseu
158471	PRUNE2	prune homolog 2 (Drosophila)
158747	MOSPD2	motile sperm domain containing 2
158787	RIBC1	RIB43A domain with coiled-coils 1
158880	USP51	ubiquitin specific peptidase 51
161253	REM2	RAS (RAD and GEM)-like GTP binding 2
1613	DAPK3	death-associated protein kinase 3
161357	MDGA2	MAM domain containing glycosylphosphatidylir
162387	MFSD6L	major facilitator superfamily domain containing
162417	NAGS	N-acetylglutamate synthase
162967	ZNF320	zinc finger protein 320
162979	ZNF296	zinc finger protein 296
163404	LPPR5	lipid phosphate phosphatase-related protein typ
164832	LONRF2	LON peptidase N-terminal domain and ring finge
169044	COL22A1	collagen, type XXII, alpha 1
170261	ZCCHC12	zinc finger, CCHC domain containing 12
170384	FUT11	fucosyltransferase 11 (alpha (1,3) fucosyltransfe
171546	SPTSSA	serine palmitoyltransferase, small subunit A
1767	DNAH5	dynein, axonemal, heavy chain 5
1815	DRD4	dopamine receptor D4
1850	DUSP8	dual specificity phosphatase 8
1852	DUSP9	dual specificity phosphatase 9
1910	EDNRB	endothelin receptor type B
1912	PHC2	polyhomeotic homolog 2 (Drosophila)
1948	EFNB2	ephrin-B2

1961	EGR4	early growth response 4
196394	AMN1	antagonist of mitotic exit network 1 homolog (S
196996	GRAMD2	GRAM domain containing 2
197187	MGC23284	uncharacterized LOC197187
1995	ELAVL3	ELAV (embryonic lethal, abnormal vision, Droso
200162	SPAG17	sperm associated antigen 17
200205	IBA57	IBA57, iron-sulfur cluster assembly homolog (S.
200407	CREG2	cellular repressor of E1A-stimulated genes 2
201232	SLC16A13	solute carrier family 16, member 13 (monocarb
201299	RDM1	RAD52 motif 1
201799	TMEM154	transmembrane protein 154
201895	C4orf34	chromosome 4 open reading frame 34
202051	SPATA24	spermatogenesis associated 24
2021	ENDOG	endonuclease G
202134	FAM153B	family with sequence similarity 153, member B
202333	CMYA5	cardiomyopathy associated 5
203111	C8orf47	chromosome 8 open reading frame 47
203238	C9orf93	chromosome 9 open reading frame 93
203259	C9orf25	chromosome 9 open reading frame 25
2034	EPAS1	endothelial PAS domain protein 1
2042	EPHA3	EPH receptor A3
2047	EPHB1	EPH receptor B1
205428	C3orf58	chromosome 3 open reading frame 58
205860	TRIML2	tripartite motif family-like 2
2103	ESRRB	estrogen-related receptor beta
2173	FABP7	fatty acid binding protein 7, brain
219749	ZNF25	zinc finger protein 25
220004	PPP1R32	protein phosphatase 1, regulatory subunit 32
220441	RNF152	ring finger protein 152
220965	FAM13C	family with sequence similarity 13, member C
221002	RASGEF1A	RasGEF domain family, member 1A
221178	SPATA13	spermatogenesis associated 13
221416	C6orf223	chromosome 6 open reading frame 223
221421	RSPH9	radial spoke head 9 homolog (Chlamydomonas)
221491	C6orf1	chromosome 6 open reading frame 1
221662	RBM24	RNA binding motif protein 24
222171	PRR15	proline rich 15
222950	C7orf51	chromosome 7 open reading frame 51
223075	CCDC129	coiled-coil domain containing 129
2246	FGF1	fibroblast growth factor 1 (acidic)
2256	FGF11	fibroblast growth factor 11
2275	FHL3	four and a half LIM domains 3
22898	DENND3	DENN/MADD domain containing 3
22901	ARSG	arylsulfatase G
22927	HABP4	hyaluronan binding protein 4
22929	SEPHS1	selenophosphate synthetase 1
2297	FOXD1	forkhead box D1
22986	SORCS3	sortilin-related VPS10 domain containing recept
22998	LIMCH1	LIM and calponin homology domains 1
22999	RIMS1	regulating synaptic membrane exocytosis 1
23015	GOLGA8A	golgin A8 family, member A
23023	TMCC1	transmembrane and coiled-coil domain family 1

23034	SAMD4A	sterile alpha motif domain containing 4A
23037	PDZD2	PDZ domain containing 2
23061	TBC1D9B	TBC1 domain family, member 9B (with GRAM d
23072	HECW1	HECT, C2 and WW domain containing E3 ubiqui
23081	KDM4C	lysine (K)-specific demethylase 4C
23089	PEG10	paternally expressed 10
23097	CDK19	cyclin-dependent kinase 19
23120	ATP10B	ATPase, class V, type 10B
23148	NACAD	NAC alpha domain containing
23237	ARC	activity-regulated cytoskeleton-associated prot
23253	ANKRD12	ankyrin repeat domain 12
23284	LPHN3	latrophilin 3
23336	SYNM	synemin, intermediate filament protein
23349	KIAA1045	KIAA1045
23353	SUN1	Sad1 and UNC84 domain containing 1
23363	OBSL1	obscurin-like 1
23376	UFL1	UFM1-specific ligase 1
23409	SIRT4	sirtuin 4
23433	RHOQ	ras homolog gene family, member Q
23480	SEC61G	Sec61 gamma subunit
23492	CBX7	chromobox homolog 7
23523	CABIN1	calcineurin binding protein 1
23671	TMEFF2	transmembrane protein with EGF-like and two f
23682	RAB38	RAB38, member RAS oncogene family
23767	FLRT3	fibronectin leucine rich transmembrane protein
24139	EML2	echinoderm microtubule associated protein like
24141	C20orf103	chromosome 20 open reading frame 103
24142	NAT6	N-acetyltransferase 6 (GCN5-related)
245973	ATP6V1C2	ATPase, H+ transporting, lysosomal 42kDa, V1 s
2491	CENPI	centromere protein I
252884	ZNF396	zinc finger protein 396
252969	NEIL2	nei endonuclease VIII-like 2 (E. coli)
253018	HCG27	HLA complex group 27 (non-protein coding)
253152	EPHX4	epoxide hydrolase 4
253769	WDR27	WD repeat domain 27
254122	SNX32	sorting nexin 32
254187	TSGA10IP	testis specific, 10 interacting protein
254528	C16orf73	chromosome 16 open reading frame 73
255022	CALHM1	calcium homeostasis modulator 1
255394	TCP11L2	t-complex 11 (mouse)-like 2
2554	GABRA1	gamma-aminobutyric acid (GABA) A receptor, a
2555	GABRA2	gamma-aminobutyric acid (GABA) A receptor, a
255783	PRR24	proline rich 24
256329	C11orf35	chromosome 11 open reading frame 35
256472	TMEM151A	transmembrane protein 151A
2565	GABRG1	gamma-aminobutyric acid (GABA) A receptor, g
256710	GLIPR1L1	GLI pathogenesis-related 1 like 1
256714	MAP7D2	MAP7 domain containing 2
256987	SERINC5	serine incorporator 5
257407	C2orf72	chromosome 2 open reading frame 72
25764	C15orf63	chromosome 15 open reading frame 63
25797	QPCT	glutaminyl-peptide cyclotransferase

25805	BAMBI	BMP and activin membrane-bound inhibitor hoı
25827	FBXL2	F-box and leucine-rich repeat protein 2
25833	POU2F3	POU class 2 homeobox 3
25834	MGAT4C	mannosyl (alpha-1,3-)-glycoprotein beta-1,4-N-a
2587	GALR1	galanin receptor 1
25870	SUMF2	sulfatase modifying factor 2
2590	GALNT2	UDP-N-acetyl-alpha-D-galactosamine:polypeptic
25911	DPCD	deleted in primary ciliary dyskinesia homolog (n
25956	SEC31B	SEC31 homolog B (S. cerevisiae
25957	PNISR	PNN-interacting serine/arginine-rich protein
25961	NUDT13	nudix (nucleoside diphosphate linked moiety X)-
26002	MOXD1	monooxygenase, DBH-like 1
26025	PCDHGA12	protocadherin gamma subfamily A, 12
26030	PLEKHG3	pleckstrin homology domain containing, family
26038	CHD5	chromodomain helicase DNA binding protein 5
260434	PYDC1	PYD (pyrin domain) containing :
26091	HERC4	hect domain and RLD 4
26140	TTLL3	tubulin tyrosine ligase-like family, member 3
26256	CABYR	calcium binding tyrosine-(Y)-phosphorylation re
2628	GATM	glycine amidinotransferase (L-arginine:glycine a
26297	SERGEF	secretion regulating guanine nucleotide exchan
2644	GCHFR	GTP cyclohydrolase I feedback regulator
26468	LHX6	LIM homeobox 6
26503	SLC17A5	solute carrier family 17 (anion/sugar transporte
26507	CNNM1	cyclin M1
26608	TBL2	transducin (beta)-like 2
2670	GFAP	glial fibrillary acidic protein
2686	GGT7	gamma-glutamyltransferase 7
2696	GIPR	gastric inhibitory polypeptide receptor
26996	GPR160	G protein-coupled receptor 160
2700	GJA3	gap junction protein, alpha 3, 46kDa
27063	ANKRD1	ankyrin repeat domain 1 (cardiac muscle)
27106	ARRDC2	arrestin domain containing 2
27113	BBC3	BCL2 binding component 3
27120	DKKL1	dickkopf-like 1
27145	FILIP1	filamin A interacting protein 1
27147	DENND2A	DENN/MADD domain containing 2A
27189	IL17C	interleukin 17C
27243	CHMP2A	charged multivesicular body protein 2A
27253	PCDH17	protocadherin 17
27254	CSDC2	cold shock domain containing C2, RNA binding
27314	RAB30	RAB30, member RAS oncogene family
27328	PCDH11X	protocadherin 11 X-linked
2734	GLG1	golgi glycoprotein 1
27345	KCNMB4	potassium large conductance calcium-activated
27350	APOBEC3C	apolipoprotein B mRNA editing enzyme, catalyt
27440	CECR5	cat eye syndrome chromosome region, candida
2796	GNRH1	gonadotropin-releasing hormone 1 (luteinizing-
282996	RBM20	RNA binding motif protein 20
283130	SLC25A45	solute carrier family 25, member 45
283131	NEAT1	nuclear paraspeckle assembly transcript 1 (non-
283299	LOC283299	uncharacterized LOC283299

283335	LOC283335	uncharacterized LOC283335
283417	DPY19L2	dpy-19-like 2 (C. elegans)
283870	C16orf79	chromosome 16 open reading frame 79
283927	NUDT7	nudix (nucleoside diphosphate linked moiety X)
283932	FBXL19-AS1	FBXL19 antisense RNA 1 (non-protein coding)
283985	FADS6	fatty acid desaturase domain family, member 6
284207	METRNL	meteorin, glial cell differentiation regulator-like
284254	C18orf26	chromosome 18 open reading frame 26
2843	GPR20	G protein-coupled receptor 20
284339	TMEM145	transmembrane protein 145
284371	ZNF841	zinc finger protein 841
284415	VSTM1	V-set and transmembrane domain containing 1
284422	C19orf77	chromosome 19 open reading frame 77
284439	SLC25A42	solute carrier family 25, member 42
284454	LOC284454	uncharacterized LOC284454
284656	EPHA10	EPH receptor A10
284716	RIMKLA	ribosomal modification protein rimK-like family
284729	ESPNP	espin pseudogene
285016	FAM150B	family with sequence similarity 150, member B
285141	LOC285141	uncharacterized LOC285141
285220	EPHA6	EPH receptor A6
285286	LOC285286	uncharacterized LOC285286
285512	FAM13A-AS1	FAM13A antisense RNA 1 (non-protein coding)
285550	FAM200B	family with sequence similarity 200, member B
285596	FAM153A	family with sequence similarity 153, member A
285600	KIAA0825	KIAA0825
285605	DTWD2	DTW domain containing 2
285613	RELL2	RELT-like 2
285761	DCBLD1	discoidin, CUB and LCCL domain containing 1
285830	HLA-F-AS1	HLA-F antisense RNA 1 (non-protein coding)
285966	FAM115C	family with sequence similarity 115, member C
285973	ATG9B	ATG9 autophagy related 9 homolog B (S. cerevis
286006	C7orf53	chromosome 7 open reading frame 53
286042	FLJ10661	family with sequence similarity 86, member A p
286046	XKR6	XK, Kell blood group complex subunit-related fa
286059	LOC286059	tumor necrosis factor receptor superfamily, me
2861	GPR37	G protein-coupled receptor 37 (endothelin rece
286411	RP1-177G6.2	uncharacterized LOC286411
28983	TMPRSS11E	transmembrane protease, serine 11E
2903	GRIN2A	glutamate receptor, ionotropic, N-methyl D-asp
29085	PHPT1	phosphohistidine phosphatase
2925	GRPR	gastrin-releasing peptide receptor
2978	GUCA1A	guanylate cyclase activator 1A (retina)
29902	C12orf24	chromosome 12 open reading frame 24
29923	C7orf68	chromosome 7 open reading frame 68
3039	HBA1	hemoglobin, alpha 1
306	ANXA3	annexin A3
30817	EMR2	egf-like module containing, mucin-like, hormone
3084	NRG1	neuregulin 1
3097	HIVEP2	human immunodeficiency virus type I enhancer
3139	HLA-L	major histocompatibility complex, class I, L, pse
317671	RFESD	Rieske (Fe-S) domain containing

3241	HPCAL1	hippocalcin-like 1
3263	HPX	hemopexin
326624	RAB37	RAB37, member RAS oncogene family
326625	MMAB	methylmalonic aciduria (cobalamin deficiency) c
3292	HSD17B1	hydroxysteroid (17-beta) dehydrogenase 1
3300	DNAJB2	DnaJ (Hsp40) homolog, subfamily B, member 2
3338	DNAJC4	DnaJ (Hsp40) homolog, subfamily C, member 4
3358	HTR2C	5-hydroxytryptamine (serotonin) receptor 2C
3373	HYAL1	hyaluronoglucosaminidase 1
338382	RAB7B	RAB7B, member RAS oncogene family
338596	ST8SIA6	ST8 alpha-N-acetyl-neuraminide alpha-2,8-sialyl
338657	CCDC84	coiled-coil domain containing 8
338739	LOC338739	uncharacterized LOC338739
339105	PRSS53	protease, serine, 53
339210	C17orf67	chromosome 17 open reading frame 67
3400	ID4	inhibitor of DNA binding 4, dominant negative h
340526	RGAG4	retrotransposon gag domain containing 4
340527	NHSL2	NHS-like 2
340533	KIAA2022	KIAA2022
341405	ANKRD33	ankyrin repeat domain 33
341880	SLC35F4	solute carrier family 35, member F4
342667	STAC2	SH3 and cysteine rich domain 2
3428	IFI16	interferon, gamma-inducible protein 16
342908	ZNF404	zinc finger protein 404
343578	ARHGAP40	Rho GTPase activating protein 40
343990	C2orf55	chromosome 2 open reading frame 55
345757	FAM174A	family with sequence similarity 174, member A
346171	ZFP57	zinc finger protein 57 homolog (mouse)
346389	MACC1	metastasis associated in colon cancer 1
353149	TBC1D26	TBC1 domain family, member 26
353189	SLCO4C1	solute carrier organic anion transporter family,
353322	ANKRD37	ankyrin repeat domain 37
353324	SPATA12	spermatogenesis associated 12
353497	POLN	polymerase (DNA directed) nu
3556	IL1RAP	interleukin 1 receptor accessory protein
3570	IL6R	interleukin 6 receptor
3579	CXCR2	chemokine (C-X-C motif) receptor 2
3621	ING1	inhibitor of growth family, member 1
3635	INPP5D	inositol polyphosphate-5-phosphatase, 145kDa
3727	JUND	jun D proto-oncogene
374378	GALNTL4	UDP-N-acetyl-alpha-D-galactosamine:polypeptid
374383	B7H6	B7 homolog 6
374875	HSD11B1L	hydroxysteroid (11-beta) dehydrogenase 1-like
374879	ZNF699	zinc finger protein 699
374887	YJEFN3	YjeF N-terminal domain containing 3
375033	PEAR1	platelet endothelial aggregation receptor 1
3754	KCNF1	potassium voltage-gated channel, subfamily F, r
375607	C7orf52	chromosome 7 open reading frame 52
375791	C9orf169	chromosome 9 open reading frame 169
3763	KCNJ6	potassium inwardly-rectifying channel, subfamil
376940	ZC3H6	zinc finger CCCH-type containing 6
3775	KCNK1	potassium channel, subfamily K, member 1

377677	CA13	carbonic anhydrase XIII
3787	KCNS1	potassium voltage-gated channel, delayed-recti
378805	FLJ43663	uncharacterized LOC378805
387597	ILDR2	immunoglobulin-like domain containing recepto
387841	RPL13AP20	ribosomal protein L13a pseudogene 20
387856	C12orf68	chromosome 12 open reading frame 68
387914	SHISA2	shisa homolog 2 (Xenopus laevis)
388284	C16orf86	chromosome 16 open reading frame 86
388403	YPEL2	yippee-like 2 (Drosophila)
388555	IGFL3	IGF-like family member 3
388558	ZNF808	zinc finger protein 808
388566	ZNF470	zinc finger protein 470
388611	CD164L2	CD164 sialomucin-like 2
388650	FAM69A	family with sequence similarity 69, member A
389058	SP5	Sp5 transcription factor
389337	ARHGEF37	Rho guanine nucleotide exchange factor (GEF) 3
389524	GTF2IRD2B	GTF2I repeat domain containing 2B
389549	FEZF1	FEZ family zinc finger 1
389641	LOC389641	uncharacterized LOC389641
389792	IER5L	immediate early response 5-like
389840	MAP3K15	mitogen-activated protein kinase kinase kinase
389941	C1QL3	complement component 1, q subcomponent-lik
3965	LGALS9	lectin, galactoside-binding, soluble, 9
397	ARHGDIB	Rho GDP dissociation inhibitor (GDI) beta
399715	LOC399715	uncharacterized LOC399715
399726	C10orf114	chromosome 10 open reading frame 114
399947	C11orf87	chromosome 11 open reading frame 87
399959	MIR100HG	mir-100-let-7a-2 cluster host gene (non-protein
40	ACCN1	amiloride-sensitive cation channel 1, neuronal
400073	C12orf76	chromosome 12 open reading frame 76
400242	DICER1-AS	DICER1 antisense RNA (non-protein coding)
400464	LOC400464	uncharacterized LOC400464
400499	LOC400499	uncharacterized LOC400499
400604	LOC400604	uncharacterized LOC400604
4007	PRICKLE3	prickle homolog 3 (Drosophila)
400745	SH2D5	SH2 domain containing 5
400960	PCBP1-AS1	PCBP1 antisense RNA 1 (non-protein coding)
401074	LOC401074	uncharacterized LOC401074
401089	C3orf72	chromosome 3 open reading frame 72
401152	C4orf3	chromosome 4 open reading frame 3
401232	DKFZP686I15	uncharacterized LOC401232
401237	LINC00340	long intergenic non-protein coding RNA 340
401251	C6orf26	chromosome 6 open reading frame 26
401265	KLHL31	kelch-like 31 (Drosophila)
401491	FLJ35024	uncharacterized LOC401491
4070	TACSTD2	tumor-associated calcium signal transducer 2
4093	SMAD9	SMAD family member 9
414241	FAM35B	family with sequence similarity 35, member A p
4162	MCAM	melanoma cell adhesion molecule
4168	MCF2	MCF.2 cell line derived transforming sequence
4293	MAP3K9	mitogen-activated protein kinase kinase kinase
4311	MME	membrane metallo-endopeptidase

432	ASGR1	asialoglycoprotein receptor 1
4327	MMP19	matrix metallopeptidase 19
439921	MXRA7	matrix-remodelling associated 7
439965	FAM35B2	family with sequence similarity 35, member A p
439990	LOC439990	uncharacterized LOC439990
440026	TMEM41B	transmembrane protein 41B
440104	LOC440104	1110012D08Rik pseudogene
440270	GOLGA8B	golgin A8 family, member B
440584	FLJ32224	uncharacterized LOC440584
440823	MIAT	myocardial infarction associated transcript (non
440952	FLJ33065	uncharacterized LOC440952
441054	C4orf47	chromosome 4 open reading frame 47
441150	C6orf226	chromosome 6 open reading frame 226
441168	FAM26F	family with sequence similarity 26, member F
441282	AKR1B15	aldo-keto reductase family 1, member B15
441531	PGAM4	phosphoglycerate mutase family member 4
4501	MT1X	metallothionein 1X
4549	RNR1	s-rRNA
4584	MUC3A	mucin 3A, cell surface associated
4741	NEFM	neurofilament, medium polypeptide
4747	NEFL	neurofilament, light polypeptid
4758	NEU1	sialidase 1 (lysosomal sialidase)
4776	NFATC4	nuclear factor of activated T-cells, cytoplasmic,
4804	NGFR	nerve growth factor receptor
4861	NPAS1	neuronal PAS domain protein 1
4862	NPAS2	neuronal PAS domain protein 2
4879	NPPB	natriuretic peptide B
4901	NRL	neural retina leucine zipper
491	ATP2B2	ATPase, Ca++ transporting, plasma membrane 2
494513	DFNB59	deafness, autosomal recessive 59
49854	ZNF295	zinc finger protein 295
49855	SCAPER	S-phase cyclin A-associated protein in the ER
5016	OVGP1	oviductal glycoprotein 1, 120kDa
5017	OVOL1	ovo-like 1(Drosophila)
5032	P2RY11	purinergic receptor P2Y, G-protein coupled, 11
50487	PLA2G3	phospholipase A2, group III
50506	DUOX2	dual oxidase 2
50615	IL21R	interleukin 21 receptor
5069	PAPPA	pregnancy-associated plasma protein A, pappaly
50840	TAS2R14	taste receptor, type 2, member 14
51003	MED31	mediator complex subunit 31
51008	ASCC1	activating signal cointegrator 1 complex subunit
51019	CCDC53	coiled-coil domain containing 5
51085	MLXIPL	MLX interacting protein-like
51131	PHF11	PHD finger protein 11
51141	INSIG2	insulin induced gene 2
51144	HSD17B12	hydroxysteroid (17-beta) dehydrogenase 12
51202	DDX47	DEAD (Asp-Glu-Ala-Asp) box polypeptide 47
51228	GLTP	glycolipid transfer protein
51232	CRIM1	cysteine rich transmembrane BMP regulator 1 (
51265	CDKL3	cyclin-dependent kinase-like 3
51276	ZNF571	zinc finger protein 571

51299	NRN1	neuritin 1
51301	GCNT4	glucosaminyl (N-acetyl) transferase 4, core 2
51339	DACT1	dapper, antagonist of beta-catenin, homolog 1 (
51400	PPME1	protein phosphatase methylesterase 1
51421	AMOTL2	angiomotin like 2
51422	PRKAG2	protein kinase, AMP-activated, gamma 2 non-ca
51428	DDX41	DEAD (Asp-Glu-Ala-Asp) box polypeptide 41
5143	PDE4C	phosphodiesterase 4C, cAMP-specific
5144	PDE4D	phosphodiesterase 4D, cAMP-specific
51454	GULP1	GULP, engulfment adaptor PTB domain containi
51517	NCKIPSD	NCK interacting protein with SH3 domain
51537	MTFP1	mitochondrial fission process 1
51545	ZNF581	zinc finger protein 581
51560	RAB6B	RAB6B, member RAS oncogene family
51655	RASD1	RAS, dexamethasone-induced 1
51760	SYT17	synaptotagmin XVII
5210	PFKFB4	6-phosphofructo-2-kinase/fructose-2,6-biphosp
5277	PIGA	phosphatidylinositol glycan anchor biosynthesis
5283	PIGH	phosphatidylinositol glycan anchor biosynthesis
5333	PLCD1	phospholipase C, delta 1
53345	TM6SF2	transmembrane 6 superfamily member 2
53358	SHC3	SHC (Src homology 2 domain containing) transfo
5336	PLCG2	phospholipase C, gamma 2 (phosphatidylinosito
5355	PLP2	proteolipid protein 2 (colonic epithelium-enrich
53616	ADAM22	ADAM metallopeptidase domain 22
5387	PMS2P3	postmeiotic segregation increased 2 pseudogen
53916	RAB4B	RAB4B, member RAS oncogene family
54101	RIPK4	receptor-interacting serine-threonine kinase 4
5413	5-Sep	septin 5
541565	C8orf58	chromosome 8 open reading frame 58
54187	NANS	N-acetylneuraminic acid synthase
54329	GPR85	G protein-coupled receptor 85
54361	WNT4	wingless-type MMTV integration site family, me
54429	TAS2R5	taste receptor, type 2, member 5
54438	GFOD1	glucose-fructose oxidoreductase domain contai
54455	FBXO42	F-box protein 42
54511	HMGCLL1	3-hydroxymethyl-3-methylglutaryl-CoA lyase-lik
5453	POU3F1	POU class 3 homeobox 1
54532	USP53	ubiquitin specific peptidase 53
54551	MAGEL2	MAGE-like 2
54583	EGLN1	egl nine homolog 1 (C. elegans)
54585	LZTFL1	leucine zipper transcription factor-like 1
54626	HES2	hairy and enhancer of split 2 (Drosophila)
54665	RSBN1	round spermatid basic protein 1
54715	RBFOX1	RNA binding protein, fox-1 homolog (C. elegans
54762	GRAMD1C	GRAM domain containing 1C
54798	DCHS2	dachsous 2 (Drosophila)
54805	CNNM2	cyclin M2
54814	QPCTL	glutaminyl-peptide cyclotransferase-like
54836	BSPRY	B-box and SPRY domain containing
54858	PGPEP1	pyroglutamyl-peptidase I
54890	ALKBH5	alkB, alkylation repair homolog 5 (E. coli)

54894	RNF43	ring finger protein 43
54928	IMPAD1	inositol monophosphatase domain containing 1
5493	PPL	periplakin
54932	EXD3	exonuclease 3'-5' domain containing 3
54985	HCFC1R1	host cell factor C1 regulator 1 (XPO1 dependent
550112	LOC550112	uncharacterized LOC550112
55106	SLFN12	schlafen family member 12
55112	WDR60	WD repeat domain 60
55118	CRTAC1	cartilage acidic protein 1
55150	C19orf73	chromosome 19 open reading frame 73
55186	SLC25A36	solute carrier family 25, member 36
55214	LEPREL1	leprecan-like 1
55236	UBA6	ubiquitin-like modifier activating enzyme 6
55279	ZNF654	zinc finger protein 654
55314	TMEM144	transmembrane protein 144
55321	C20orf46	chromosome 20 open reading frame 46
5533	PPP3CC	protein phosphatase 3, catalytic subunit, gamm
55351	STK32B	serine/threonine kinase 32B
55357	TBC1D2	TBC1 domain family, member 2
554251	FBXO48	F-box protein 48
554279	C1orf98	chromosome 1 open reading frame 98
55534	MAML3	mastermind-like 3 (Drosophila)
55556	ENOSF1	enolase superfamily member 1
55640	FLVCR2	feline leukemia virus subgroup C cellular recept
55647	RAB20	RAB20, member RAS oncogene family
55650	PIGV	phosphatidylinositol glycan anchor biosynthesis
55689	YEATS2	YEATS domain containing 2
55691	FRMD4A	FERM domain containing 4A
5570	PKIB	protein kinase (cAMP-dependent, catalytic) inhi
55726	C12orf11	chromosome 12 open reading frame 11
55760	DHX32	DEAH (Asp-Glu-Ala-His) box polypeptide 32
55784	MCTP2	multiple C2 domains, transmembrane 2
55799	CACNA2D3	calcium channel, voltage-dependent, alpha 2/de
55800	SCN3B	sodium channel, voltage-gated, type III, beta
55805	LRP2BP	LRP2 binding protein
55824	PAG1	phosphoprotein associated with glycosphingolip
55893	ZNF395	zinc finger protein 395
55917	CTTNBP2NL	CTTNBP2 N-terminal like
55974	SLC50A1	solute carrier family 50 (sugar transporter), mer
5598	MAPK7	mitogen-activated protein kinase 7
56033	BARX1	BARX homeobox 1
5604	MAP2K1	mitogen-activated protein kinase kinase 1
56109	PCDHGA6	protocadherin gamma subfamily A, 6
56135	PCDHAC1	protocadherin alpha subfamily C, 1
56204	KIAA1370	KIAA1370
56256	SERTAD4	SERTA domain containing 4
56270	WDR45L	WDR45-like
56301	SLC7A10	solute carrier family 7 (neutral amino acid trans
56341	PRMT8	protein arginine methyltransferase 8
56477	CCL28	chemokine (C-C motif) ligand 2[illegible]
56849	TCEAL7	transcription elongation factor A (SII)-like 7
56899	ANKS1B	ankyrin repeat and sterile alpha motif domain c

56901	NDUFA4L2	NADH dehydrogenase (ubiquinone) 1 alpha sub
56913	C1GALT1	core 1 synthase, glycoprotein-N-acetylgalactosa
56917	MEIS3	Meis homeobox 3
56929	FEM1C	fem-1 homolog c (C. elegans)
56950	SMYD2	SET and MYND domain containing 2
56985	C17orf48	chromosome 17 open reading frame 48
57035	C1orf63	chromosome 1 open reading frame 63
57110	HRASLS	HRAS-like suppressor
57111	RAB25	RAB25, member RAS oncogene family
57125	PLXDC1	plexin domain containing 1
57168	ASPHD2	aspartate beta-hydroxylase domain containing
57188	ADAMTSL3	ADAMTS-like 3
57221	KIAA1244	KIAA1244
57393	TMEM27	transmembrane protein 27
57396	CLK4	CDC-like kinase 4
5742	PTGS1	prostaglandin-endoperoxide synthase 1 (prosta
57469	PNMAL2	PNMA-like 2
57475	PLEKHH1	pleckstrin homology domain containing, family
57488	ESYT2	extended synaptotagmin-like protein 2
57493	HEG1	HEG homolog 1 (zebrafish)
57497	LRFN2	leucine rich repeat and fibronectin type III doma
57501	KIAA1257	KIAA1257
57519	STARD9	StAR-related lipid transfer (START) domain cont
57524	CASKIN1	CASK interacting protein 1
57526	PCDH19	protocadherin 19
57528	KCTD16	potassium channel tetramerisation domain con
57529	RGAG1	retrotransposon gag domain containing 1
57535	KIAA1324	KIAA1324
57546	PDP2	pyruvate dehyrogenase phosphatase catalytic s
57559	STAMBPL1	STAM binding protein-like 1
57561	ARRDC3	arrestin domain containing 3
57574	4-Mar	membrane-associated ring finger (C3HC4) 4
57575	PCDH10	protocadherin 10
57616	TSHZ3	teashirt zinc finger homeobox 3
57643	ZSWIM5	zinc finger, SWIM-type containing 5
57644	MYH7B	myosin, heavy chain 7B, cardiac muscle, beta
57683	ZDBF2	zinc finger, DBF-type containing 2
57690	TNRC6C	trinucleotide repeat containing 6C
577	BAI3	brain-specific angiogenesis inhibitor 3
57709	SLC7A14	solute carrier family 7 (orphan transporter), me
57711	ZNF529	zinc finger protein 529
57715	SEMA4G	sema domain, immunoglobulin domain (Ig), tra
57732	ZFYVE28	zinc finger, FYVE domain containing 28
57801	HES4	hairy and enhancer of split 4 (Drosophila)
5787	PTPRB	protein tyrosine phosphatase, receptor type, B
5794	PTPRH	protein tyrosine phosphatase, receptor type, H
5798	PTPRN	protein tyrosine phosphatase, receptor type, N
5800	PTPRO	protein tyrosine phosphatase, receptor type, O
583	BBS2	Bardet-Biedl syndrome 2
5870	RAB6A	RAB6A, member RAS oncogene family
5872	RAB13	RAB13, member RAS oncogene family
5923	RASGRF1	Ras protein-specific guanine nucleotide-releasi

59269	HIVEP3	human immunodeficiency virus type I enhancer
5939	RBMS2	RNA binding motif, single stranded interacting p
5997	RGS2	regulator of G-protein signaling 2, 24kDa
5998	RGS3	regulator of G-protein signaling 3
6000	RGS7	regulator of G-protein signaling 7
6001	RGS10	regulator of G-protein signaling 10
6018	RLF	rearranged L-myc fusion
6019	RLN2	relaxin 2
60401	EDA2R	ectodysplasin A2 receptor
60468	BACH2	BTB and CNC homology 1, basic leucine zipper t
60676	PAPPA2	pappalysin 2
60682	SMAP1	small ArfGAP 1
6092	ROBO2	roundabout, axon guidance receptor, homolog
6307	MSMO1	methylsterol monooxygenase 1
6322	SCML1	sex comb on midleg-like 1 (Drosophila)
6330	SCN4B	sodium channel, voltage-gated, type IV, beta
63875	MRPL17	mitochondrial ribosomal protein L17
63917	GALNT11	UDP-N-acetyl-alpha-D-galactosamine:polypepti
6405	SEMA3F	sema domain, immunoglobulin domain (Ig), sho
6406	SEMG1	semenogelin I
64063	PRSS22	protease, serine, 22
64102	TNMD	tenomodulin
641371	ACOT1	acyl-CoA thioesterase 1
641649	TMEM91	transmembrane protein 91
64218	SEMA4A	sema domain, immunoglobulin domain (Ig), tra
642273	FAM110C	family with sequence similarity 110, member C
64236	PDLIM2	PDZ and LIM domain 2 (mystique)
64285	RHBDF1	rhomboid 5 homolog 1 (Drosophila)
642946	FLVCR1-AS1	FLVCR1 antisense RNA 1 (non-protein coding)
642987	TMEM232	transmembrane protein 232
64343	AZI2	5-azacytidine induced 2
643641	ZNF862	zinc finger protein 862
644242	LOC644242	uncharacterized LOC644242
64430	C14orf135	chromosome 14 open reading frame 135
64478	CSMD1	CUB and Sushi multiple domains 1
64506	CPEB1	cytoplasmic polyadenylation element binding p
645166	LOC645166	lymphocyte-specific protein 1 pseudogene
645426	TMEM191C	transmembrane protein 191C
645591	LOC645591	uncharacterized LOC645591
6457	SH3GL3	SH3-domain GRB2-like 3
645784	ANKRD36BP	ankyrin repeat domain 36B pseudogene 2
6461	SHB	Src homology 2 domain containing adaptor prot
64651	CSRNP1	cysteine-serine-rich nuclear protein 1
646658	SYNDIG1L	synapse differentiation inducing 1-like
64778	FNDC3B	fibronectin type III domain containing 3B
64799	IQCH	IQ motif containing H
64900	LPIN3	lipin 3
64926	RASAL3	RAS protein activator like 3
65009	NDRG4	NDRG family member 4
65010	SLC26A6	solute carrier family 26, member 6
65084	TMEM135	transmembrane protein 135
651746	ANKRD33B	ankyrin repeat domain 33B

653319	KIAA0895L	KIAA0895-like
653583	PHLDB3	pleckstrin homology-like domain, family B, mem
6543	SLC8A2	solute carrier family 8 (sodium/calcium exchang
6546	SLC8A1	solute carrier family 8 (sodium/calcium exchang
6547	SLC8A3	solute carrier family 8 (sodium/calcium exchang
6553	SLC9A5	solute carrier family 9 (sodium/hydrogen excha
65981	CAPRIN2	caprin family member 2
65985	AACS	acetoacetyl-CoA synthetase
65996	MGC2752	CENPB DNA-binding domains containing 1 pseu
661	POLR3D	polymerase (RNA) III (DNA directed) polypeptid
6615	SNAI1	snail homolog 1 (Drosophila)
6617	SNAPC1	small nuclear RNA activating complex, polypept
6619	SNAPC3	small nuclear RNA activating complex, polypept
6640	SNTA1	syntrophin, alpha 1 (dystrophin-associated prot
6674	SPAG1	sperm associated antigen 1
6690	SPINK1	serine peptidase inhibitor, Kazal type 1
6751	SSTR1	somatostatin receptor 1
677768	SCARNA13	small Cajal body-specific RNA 1
6804	STX1A	syntaxin 1A (brain)
6855	SYP	synaptophysin
6863	TAC1	tachykinin, precursor 1
6900	CNTN2	contactin 2 (axonal)
6911	TBX6	T-box 6
692233	SNORD117	small nucleolar RNA, C/D box 117
6928	HNF1B	HNF1 homeobox B
7003	TEAD1	TEA domain family member 1 (SV40 transcriptio
7005	TEAD3	TEA domain family member 3
7006	TEC	tec protein tyrosine kinase
7089	TLE2	transducin-like enhancer of split 2 (E(sp1) homo
7126	TNFAIP1	tumor necrosis factor, alpha-induced protein 1 (
7188	TRAF5	TNF receptor-associated factor 5
723790	HIST2H2AA4	histone cluster 2, H2aa4
728229	TMEM191B	transmembrane protein 191B
728537	LOC728537	uncharacterized LOC728537
728577	CNTNAP3B	contactin associated protein-like 3B
7286	TUFT1	tuftelin 1
728752	LOC728752	uncharacterized LOC728752
7291	TWIST1	twist homolog 1 (Drosophila)
729603	LOC729603	calcium binding protein P22 pseudogene
729956	SHISA7	shisa homolog 7 (Xenopus laevis)
729970	LOC729970	hCG2028352-like
729993	SHISA9	shisa homolog 9 (Xenopus laevis)
7403	KDM6A	lysine (K)-specific demethylase 6A
7425	VGF	VGF nerve growth factor inducible
7434	VIPR2	vasoactive intestinal peptide receptor 2
7447	VSNL1	visinin-like 1
7464	CORO2A	coronin, actin binding protein, 2A
7473	WNT3	wingless-type MMTV integration site family, me
7525	YES1	v-yes-1 Yamaguchi sarcoma viral oncogene hom
7593	MZF1	myeloid zinc finger 1
7597	ZBTB25	zinc finger and BTB domain containing 25
761	CA3	carbonic anhydrase III, muscle specific

768	CA9	carbonic anhydrase IX
770	CA11	carbonic anhydrase XI
7718	ZNF165	zinc finger protein 165
7737	RNF113A	ring finger protein 113A
777	CACNA1E	calcium channel, voltage-dependent, R type, alp
7804	LRP8	low density lipoprotein receptor-related protein
784	CACNB3	calcium channel, voltage-dependent, beta 3 sub
7846	TUBA1A	tubulin, alpha 1a
785	CACNB4	calcium channel, voltage-dependent, beta 4 sub
79001	VKORC1	vitamin K epoxide reductase complex, subunit 1
79036	C19orf50	chromosome 19 open reading frame 50
79081	C11orf48	chromosome 11 open reading frame 48
79090	TRAPPC6A	trafficking protein particle complex 6A
790952	ESRG	embryonic stem cell related (non-protein codin
79098	C1orf116	chromosome 1 open reading frame 116
7919	DDX39B	DEAD (Asp-Glu-Ala-Asp) box polypeptide 39B
79228	THOC6	THO complex 6 homolog (Drosophila)
7923	HSD17B8	hydroxysteroid (17-beta) dehydrogenase 8
79365	BHLHE41	basic helix-loop-helix family, member e41
79369	B3GNT4	UDP-GlcNAc:betaGal beta-1,3-N-acetylglucosam
79567	FAM65A	family with sequence similarity 65, member A
79583	TMEM231	transmembrane protein 231
79589	RNF128	ring finger protein 128
796	CALCA	calcitonin-related polypeptide alpha
79628	SH3TC2	SH3 domain and tetratricopeptide repeats 2
79635	CCDC121	coiled-coil domain containing 121
79639	TMEM53	transmembrane protein 53
79646	PANK3	pantothenate kinase 3
79656	BEND5	BEN domain containing 5
79661	NEIL1	nei endonuclease VIII-like 1 (E. coli)
79667	FLJ13197	uncharacterized FLJ13197
79690	GAL3ST4	galactose-3-O-sulfotransferase 4
79729	SH3D21	SH3 domain containing 21
79739	TTLL7	tubulin tyrosine ligase-like family, member 7
79772	MCTP1	multiple C2 domains, transmembrane 1
79783	C7orf10	chromosome 7 open reading frame 10
79816	TLE6	transducin-like enhancer of split 6 (E(sp1) homo
79841	AGBL2	ATP/GTP binding protein-like 2
79854	LINC00115	long intergenic non-protein coding RNA 115
79873	NUDT18	nudix (nucleoside diphosphate linked moiety X)
79896	THNSL1	threonine synthase-like 1 (S. cerevisiae)
79899	PRR5L	proline rich 5 like
79919	C2orf54	chromosome 2 open reading frame 54
79930	DOK3	docking protein 3
79937	CNTNAP3	contactin associated protein-like 3
79939	SLC35E1	solute carrier family 35, member E1
79958	DENND1C	DENN/MADD domain containing 1C
79960	PHF17	PHD finger protein 17
79963	ABCA11P	ATP-binding cassette, sub-family A (ABC1), mem
79987	SVEP1	sushi, von Willebrand factor type A, EGF and pe
79991	OBFC1	oligonucleotide/oligosaccharide-binding fold co
79992	AGPAT4-IT1	AGPAT4 intronic transcript 1 (non-protein codir

80070	ADAMTS20	ADAM metallopeptidase with thrombospondin
80108	ZFP2	zinc finger protein 2 homolog (mouse)
80115	BAIAP2L2	BAI1-associated protein 2-like 2
80173	IFT74	intraflagellar transport 74 homolog (Chlamydon
80312	TET1	tet methylcytosine dioxygenase 1
80329	ULBP1	UL16 binding protein 1
80339	PNPLA3	patatin-like phospholipase domain containing 3
80705	TSGA10	testis specific, 10
80736	SLC44A4	solute carrier family 44, member 4
80758	PRR7	proline rich 7 (synaptic)
80856	KIAA1715	KIAA1715
80864	EGFL8	EGF-like-domain, multiple 8
81544	GDPD5	glycerophosphodiester phosphodiesterase dom
81551	STMN4	stathmin-like 4
81575	APOLD1	apolipoprotein L domain containing 1
81603	TRIM8	tripartite motif containing 8
81671	VMP1	vacuole membrane protein 1
817	CAMK2D	calcium/calmodulin-dependent protein kinase I
81788	NUAK2	NUAK family, SNF1-like kinase, 2
81855	SFXN3	sideroflexin 3
83259	PCDH11Y	protocadherin 11 Y-linked
8337	HIST2H2AA3	histone cluster 2, H2aa3
8353	HIST1H3E	histone cluster 1, H3e
83591	THAP2	THAP domain containing, apoptosis associated
83592	AKR1E2	aldo-keto reductase family 1, member E2
83715	ESPN	espin
83723	FAM57B	family with sequence similarity 57, member B
83851	SYT16	synaptotagmin XVI
83856	FSD1L	fibronectin type III and SPRY domain containing
83886	PRSS27	protease, serine 27
83891	SNX25	sorting nexin 25
8398	PLA2G6	phospholipase A2, group VI (cytosolic, calcium-i
84000	TMPRSS13	transmembrane protease, serine 13
8403	SOX14	SRY (sex determining region Y)-box 14
84033	OBSCN	obscurin, cytoskeletal calmodulin and titin-inter
84034	EMILIN2	elastin microfibril interfacer 2
84058	WDR54	WD repeat domain 54
84084	RAB6C	RAB6C, member RAS oncogene family
84129	ACAD11	acyl-CoA dehydrogenase family, member 11
84163	GTF2IRD2	GTF2I repeat domain containing 2
84222	TMEM191A	transmembrane protein 191A
84249	PSD2	pleckstrin and Sec7 domain containing 2
84251	SGIP1	SH3-domain GRB2-like (endophilin) interacting
84261	FBXW9	F-box and WD repeat domain containing 9
84293	C10orf58	chromosome 10 open reading frame 58
84314	TMEM107	transmembrane protein 107
84324	SARNP	SAP domain containing ribonucleoprotein
8433	UTF1	undifferentiated embryonic cell transcription fa
84336	TMEM101	transmembrane protein 101
84449	ZNF333	zinc finger protein 333
84460	ZMAT1	zinc finger, matrin-type 1
84557	MAP1LC3A	microtubule-associated protein 1 light chain 3 a

84617	TUBB6	tubulin, beta 6 class V
8462	KLF11	Kruppel-like factor 11
84634	KISS1R	KISS1 receptor
84678	KDM2B	lysine (K)-specific demethylase 2B
84695	LOXL3	lysyl oxidase-like 3
84698	CAPS2	calcyphosine 2
84700	MYO18B	myosin XVIIIB
84706	GPT2	glutamic pyruvate transaminase (alanine amino
84708	LNX1	ligand of numb-protein X 1
84709	C4orf49	chromosome 4 open reading frame 49
84848	MGC16121	uncharacterized protein MGC16121
84910	TMEM87B	transmembrane protein 87B
84929	FIBCD1	fibrinogen C domain containing 1
84941	HSH2D	hematopoietic SH2 domain containing
84951	TNS4	tensin 4
84957	RELT	RELT tumor necrosis factor receptor
84962	JUB	jub, ajuba homolog (Xenopus laevis)
84966	IGSF21	immunoglobin superfamily, member 21
84969	TOX2	TOX high mobility group box family member 2
8504	PEX3	peroxisomal biogenesis factor 3
8525	DGKZ	diacylglycerol kinase, zeta
85301	COL27A1	collagen, type XXVII, alpha 1
85360	SYDE1	synapse defective 1, Rho GTPase, homolog 1 (C.
85443	DCLK3	doublecortin-like kinase 3
85465	EPT1	ethanolaminephosphotransferase 1 (CDP-ethan
85480	TSLP	thymic stromal lymphopoietin
860	RUNX2	runt-related transcription factor 2
8622	PDE8B	phosphodiesterase 8B
8637	EIF4EBP3	eukaryotic translation initiation factor 4E bindin
8701	DNAH11	dynein, axonemal, heavy chain 11
8715	NOL4	nucleolar protein 4
8793	TNFRSF10D	tumor necrosis factor receptor superfamily, me
8795	TNFRSF10B	tumor necrosis factor receptor superfamily, me
8797	TNFRSF10A	tumor necrosis factor receptor superfamily, me
883	CCBL1	cysteine conjugate-beta lyase, cytoplasmic
8862	APLN	apelin
8911	CACNA1I	calcium channel, voltage-dependent, T type, alp
89782	LMLN	leishmanolysin-like (metallopeptidase M8 family
89795	NAV3	neuron navigator 3
8989	TRPA1	transient receptor potential cation channel, sub
8999	CDKL2	cyclin-dependent kinase-like 2 (CDC2-related kin
9001	HAP1	huntingtin-associated protein 1
9020	MAP3K14	mitogen-activated protein kinase kinase kinase
90332	EXOC3L2	exocyst complex component 3-like 2
90338	ZNF160	zinc finger protein 160
90423	ATP6V1E2	ATPase, H+ transporting, lysosomal 31kDa, V1 s
9044	BTAF1	BTAF1 RNA polymerase II, B-TFIID transcription
9052	GPRC5A	G protein-coupled receptor, family C, group 5, r
90557	CCDC74A	coiled-coil domain containing 74A
90594	ZNF439	zinc finger protein 439
90627	STARD13	StAR-related lipid transfer (START) domain cont
90673	PPP1R3E	protein phosphatase 1, regulatory subunit 3E

9076	CLDN1	claudin 1
90853	SPOCD1	SPOC domain containing 1
90874	ZNF697	zinc finger protein 697
9098	USP6	ubiquitin specific peptidase 6 (Tre-2 oncogene)
90993	CREB3L1	cAMP responsive element binding protein 3-like
9118	INA	internexin neuronal intermediate filament prot
91319	DERL3	Der1-like domain family, member 3
91373	UAP1L1	UDP-N-acteylglucosamine pyrophosphorylase 1
91409	CCDC74B	coiled-coil domain containing 74B
9143	SYNGR3	synaptogyrin 3
91452	ACBD5	acyl-CoA binding domain containing 5
91523	FAM113B	family with sequence similarity 113, member B
91607	SLFN11	schlafen family member 11
91662	NLRP12	NLR family, pyrin domain containing 12
91663	MYADM	myeloid-associated differentiation marker
91947	ARRDC4	arrestin domain containing 4
9196	KCNAB3	potassium voltage-gated channel, shaker-relate
9201	DCLK1	doublecortin-like kinase 1
92092	ZC3HAV1L	zinc finger CCCH-type, antiviral 1-like
92162	TMEM88	transmembrane protein 88
92211	CDHR1	cadherin-related family member 1
92270	ATP6AP1L	ATPase, H+ transporting, lysosomal accessory p
92421	CHMP4C	charged multivesicular body protein 4C
92565	FANK1	fibronectin type III and ankyrin repeat domains
9278	ZBTB22	zinc finger and BTB domain containing 22
92949	ADAMTSL1	ADAMTS-like 1
930	CD19	CD19 molecule
9311	ACCN3	amiloride-sensitive cation channel 3
93129	ORAI3	ORAI calcium release-activated calcium modula
9350	CER1	cerberus 1, cysteine knot superfamily, homolog
9363	RAB33A	RAB33A, member RAS oncogene family
93649	MYOCD	myocardin
9369	NRXN3	neurexin 3
9378	NRXN1	neurexin 1
9388	LIPG	lipase, endothelial
93953	ACRC	acidic repeat containing
94122	SYTL5	synaptotagmin-like 5
9413	FAM189A2	family with sequence similarity 189, member A
9424	KCNK6	potassium channel, subfamily K, member 6
9468	PCYT1B	phosphate cytidylyltransferase 1, choline, beta
9478	CABP1	calcium binding protein 1
9497	SLC4A7	solute carrier family 4, sodium bicarbonate cotr
9518	GDF15	growth differentiation factor 15
953	ENTPD1	ectonucleoside triphosphate diphosphohydrola
954	ENTPD2	ectonucleoside triphosphate diphosphohydrola
9568	GABBR2	gamma-aminobutyric acid (GABA) B receptor, 2
9586	CREB5	cAMP responsive element binding protein 5
9628	RGS6	regulator of G-protein signaling 6
9645	MICAL2	microtubule associated monoxygenase, calponi
9708	PCDHGA8	protocadherin gamma subfamily A, 8
9718	ECE2	endothelin converting enzyme
9725	TMEM63A	transmembrane protein 63A

9744	ACAP1	ArfGAP with coiled-coil, ankyrin repeat and PH (
9780	PIEZO1	piezo-type mechanosensitive ion channel comp
9796	PHYHIP	phytanoyl-CoA 2-hydroxylase interacting protei
98	ACYP2	acylphosphatase 2, muscle type
9820	CUL7	cullin 7
9829	DNAJC6	DnaJ (Hsp40) homolog, subfamily C, member 6
9853	RUSC2	RUN and SH3 domain containing 2
9899	SV2B	synaptic vesicle glycoprotein 2B
9931	HELZ	helicase with zinc finger
9956	HS3ST2	heparan sulfate (glucosamine) 3-O-sulfotransfer
9982	FGFBP1	fibroblast growth factor binding protein 1
9985	REC8	REC8 homolog (yeast)

p-value	FDR q-value
1.58E-90	7.92E-89
6.89E-62	1.72E-60
1.09E-41	1.81E-40
4.70E-39	5.87E-38
3.48E-33	3.48E-32
3.64E-31	3.04E-30
3.59E-30	2.56E-29
6.93E-21	4.33E-20
4.06E-20	2.25E-19
5.03E-20	2.52E-19

HALLMARK_MT	HALLMARK_CH	HALLMARK_EPI	HALLMARK_GL	HALLMARK_P5	HALLMARK_MY	HALLMARK_AP
HALLMARK_MT	HALLMARK_CHOLESTEROL_HOMEOSTASIS					
HALLMARK_MTORC1_SIGNALING				HALLMARK_P5	HALLMARK_MY	HALLMARK_AP
HALLMARK_MTORC1_SIGNALING				HALLMARK_P53_PATHWAY		
HALLMARK_MTORC1_SIGNALING					HALLMARK_MYOGENESIS	
HALLMARK_MTORC1_SIGNALING						
FA_SIGNALING_	HALLMARK_CHOLESTEROL_HOMEOSTASIS			HALLMARK_P53_PATHWAY		HALLMARK_AP
FA_SIGNALING_	HALLMARK_CHOLESTEROL_HOMEOSTASIS					
FA_SIGNALING_VIA_NFKB		HALLMARK_EPI	HALLMARK_GLYCOLYSIS			
FA_SIGNALING_VIA_NFKB		HALLMARK_EPITHELIAL_MESEN		HALLMARK_P53_PATHWAY		HALLMARK_AP
FA_SIGNALING_VIA_NFKB		HALLMARK_EPITHELIAL_MESENCHYMAL_TRANSITION				
FA_SIGNALING_VIA_NFKB		HALLMARK_EPITHELIAL_MESENCHYMAL_TRANSITION				
FA_SIGNALING_VIA_NFKB		HALLMARK_EPITHELIAL_MESENCHYMAL_TRANSITION				
FA_SIGNALING_VIA_NFKB			HALLMARK_GL	HALLMARK_P53_PATHWAY		HALLMARK_AP
FA_SIGNALING_VIA_NFKB				HALLMARK_P53_PATHWAY		
FA_SIGNALING_VIA_NFKB				HALLMARK_P53_PATHWAY		
FA_SIGNALING_VIA_NFKB						
FA_SIGNALING_VIA_NFKB						
FA_SIGNALING_VIA_NFKB						
FA_SIGNALING_VIA_NFKB						
FA_SIGNALING_VIA_NFKB						
FA_SIGNALING_VIA_NFKB						
FA_SIGNALING_VIA_NFKB						
FA_SIGNALING_VIA_NFKB						
FA_SIGNALING_VIA_NFKB						
HALLMARK_MTORC1_SIGNALING			HALLMARK_GL	HALLMARK_P53_PATHWAY		
HALLMARK_MTORC1_SIGNALING			HALLMARK_GLYCOLYSIS			
HALLMARK_MTORC1_SIGNALING			HALLMARK_GLYCOLYSIS			
HALLMARK_MTORC1_SIGNALING			HALLMARK_GLYCOLYSIS			

HALLMARK_MTORC1_SIGNALING			HALLMARK_GLYCOLYSIS			
HALLMARK_MTORC1_SIGNALING			HALLMARK_GLYCOLYSIS			
HALLMARK_MTORC1_SIGNALING			HALLMARK_GLYCOLYSIS			
HALLMARK_MTORC1_SIGNALING			HALLMARK_GLYCOLYSIS			
HALLMARK_MTORC1_SIGNALING			HALLMARK_GLYCOLYSIS			
HALLMARK_MTORC1_SIGNALING			HALLMARK_GLYCOLYSIS			
HALLMARK_MTORC1_SIGNALING			HALLMARK_GLYCOLYSIS			
HALLMARK_MTORC1_SIGNALING			HALLMARK_GLYCOLYSIS			
HALLMARK_MTORC1_SIGNALING				HALLMARK_P53_PATHWAY		HALLMARK_AP
HALLMARK_MTORC1_SIGNALING						
HALLMARK_MTORC1_SIGNALING						
HALLMARK_MTORC1_SIGNALING						
HALLMARK_MTORC1_SIGNALING						
HALLMARK_MTORC1_SIGNALING						
HALLMARK_MTORC1_SIGNALING						
HALLMARK_MTORC1_SIGNALING						
HALLMARK_MTORC1_SIGNALING						
	HALLMARK_CHOLESTEROL_HO		HALLMARK_GLYCOLYSIS			
	HALLMARK_CHOLESTEROL_HOMEOSTASIS					
	HALLMARK_CHOLESTEROL_HOMEOSTASIS					
		HALLMARK_EPI	HALLMARK_GLYCOLYSIS			HALLMARK_AP
		HALLMARK_EPI	HALLMARK_GLYCOLYSIS			
		HALLMARK_EPITHELIAL_MESENCHYMAL_TRANSITION				
		HALLMARK_EPITHELIAL_MESENCHYMAL_TRANSITION				
			HALLMARK_GL	HALLMARK_P53_PATHWAY		
			HALLMARK_GLYCOLYSIS		HALLMARK_MYOGENESIS	
			HALLMARK_GLYCOLYSIS		HALLMARK_MYOGENESIS	
			HALLMARK_GLYCOLYSIS			HALLMARK_AP
			HALLMARK_GLYCOLYSIS			
			HALLMARK_GLYCOLYSIS			
			HALLMARK_GLYCOLYSIS			
			HALLMARK_GLYCOLYSIS			
			HALLMARK_GLYCOLYSIS			
			HALLMARK_GLYCOLYSIS			
			HALLMARK_GLYCOLYSIS			
			HALLMARK_GLYCOLYSIS			
			HALLMARK_GLYCOLYSIS			
			HALLMARK_GLYCOLYSIS			
			HALLMARK_GLYCOLYSIS			
			HALLMARK_GLYCOLYSIS			
			HALLMARK_GLYCOLYSIS			
			HALLMARK_GLYCOLYSIS			
			HALLMARK_GLYCOLYSIS			
				HALLMARK_P53_PATHWAY		
				HALLMARK_P53_PATHWAY		
					HALLMARK_MYOGENESIS	
					HALLMARK_MYOGENESIS	
					HALLMARK_MYOGENESIS	
					HALLMARK_MYOGENESIS	
						HALLMARK_AP
						HALLMARK_AP

HALLMARK_MT	HALLMARK_CHOLESTEROL_HOMEOSTASIS					
HALLMARK_MTORC1_SIGNALING				HALLMARK_P53_PATHWAY		HALLMARK_AP
HALLMARK_MTORC1_SIGNALING						
FA_SIGNALING_	HALLMARK_CHOLESTEROL_HOMEOSTASIS					
FA_SIGNALING_VIA_NFKB		HALLMARK_EPI	HALLMARK_GLYCOLYSIS			HALLMARK_AP
FA_SIGNALING_VIA_NFKB		HALLMARK_EPITHELIAL_MESEN		HALLMARK_P53_PATHWAY		HALLMARK_AP
FA_SIGNALING_VIA_NFKB		HALLMARK_EPITHELIAL_MESENCHYMAL_TRAN			HALLMARK_MY	HALLMARK_AP
FA_SIGNALING_VIA_NFKB		HALLMARK_EPITHELIAL_MESENCHYMAL_TRANSITION				HALLMARK_AP
FA_SIGNALING_VIA_NFKB		HALLMARK_EPITHELIAL_MESENCHYMAL_TRANSITION				
FA_SIGNALING_VIA_NFKB		HALLMARK_EPITHELIAL_MESENCHYMAL_TRANSITION				
FA_SIGNALING_VIA_NFKB		HALLMARK_EPITHELIAL_MESENCHYMAL_TRANSITION				
FA_SIGNALING_VIA_NFKB		HALLMARK_EPITHELIAL_MESENCHYMAL_TRANSITION				
FA_SIGNALING_VIA_NFKB		HALLMARK_EPITHELIAL_MESENCHYMAL_TRANSITION				
FA_SIGNALING_VIA_NFKB				HALLMARK_P5	HALLMARK_MYOGENESIS	
FA_SIGNALING_VIA_NFKB				HALLMARK_P5	HALLMARK_MYOGENESIS	
FA_SIGNALING_VIA_NFKB				HALLMARK_P53_PATHWAY		HALLMARK_AP
FA_SIGNALING_VIA_NFKB				HALLMARK_P53_PATHWAY		HALLMARK_AP
FA_SIGNALING_VIA_NFKB				HALLMARK_P53_PATHWAY		
FA_SIGNALING_VIA_NFKB				HALLMARK_P53_PATHWAY		
FA_SIGNALING_VIA_NFKB				HALLMARK_P53_PATHWAY		
FA_SIGNALING_VIA_NFKB				HALLMARK_P53_PATHWAY		
FA_SIGNALING_VIA_NFKB				HALLMARK_P53_PATHWAY		
FA_SIGNALING_VIA_NFKB				HALLMARK_P53_PATHWAY		
FA_SIGNALING_VIA_NFKB						HALLMARK_AP
FA_SIGNALING_VIA_NFKB						HALLMARK_AP
FA_SIGNALING_VIA_NFKB						
FA_SIGNALING_VIA_NFKB						
FA_SIGNALING_VIA_NFKB						
FA_SIGNALING_VIA_NFKB						
FA_SIGNALING_VIA_NFKB						

FA_SIGNALING_VIA_NFKB						
FA_SIGNALING_VIA_NFKB						
FA_SIGNALING_VIA_NFKB						
FA_SIGNALING_VIA_NFKB						
FA_SIGNALING_VIA_NFKB						
FA_SIGNALING_VIA_NFKB						
FA_SIGNALING_VIA_NFKB						
FA_SIGNALING_VIA_NFKB						
FA_SIGNALING_VIA_NFKB						
FA_SIGNALING_VIA_NFKB						
FA_SIGNALING_VIA_NFKB						
FA_SIGNALING_VIA_NFKB						
FA_SIGNALING_VIA_NFKB						
FA_SIGNALING_VIA_NFKB						
FA_SIGNALING_VIA_NFKB						
FA_SIGNALING_VIA_NFKB						
FA_SIGNALING_VIA_NFKB						
FA_SIGNALING_VIA_NFKB						
FA_SIGNALING_VIA_NFKB						
FA_SIGNALING_VIA_NFKB						
FA_SIGNALING_VIA_NFKB						
FA_SIGNALING_VIA_NFKB						
FA_SIGNALING_VIA_NFKB						
FA_SIGNALING_VIA_NFKB						
FA_SIGNALING_VIA_NFKB						
FA_SIGNALING_VIA_NFKB						
FA_SIGNALING_VIA_NFKB						
HALLMARK_MT	HALLMARK_CHOLESTEROL_HOMEOSTASIS			HALLMARK_P53_PATHWAY		
HALLMARK_MT	HALLMARK_CHOLESTEROL_HOMEOSTASIS				HALLMARK_MYOGENESIS	
HALLMARK_MT	HALLMARK_CHOLESTEROL_HOMEOSTASIS					HALLMARK_AP
HALLMARK_MT	HALLMARK_CHOLESTEROL_HOMEOSTASIS					HALLMARK_AP
HALLMARK_MT	HALLMARK_CHOLESTEROL_HOMEOSTASIS					
HALLMARK_MT	HALLMARK_CHOLESTEROL_HOMEOSTASIS					
HALLMARK_MT	HALLMARK_CHOLESTEROL_HOMEOSTASIS					
HALLMARK_MT	HALLMARK_CHOLESTEROL_HOMEOSTASIS					
HALLMARK_MT	HALLMARK_CHOLESTEROL_HOMEOSTASIS					
HALLMARK_MT	HALLMARK_CHOLESTEROL_HOMEOSTASIS					
HALLMARK_MT	HALLMARK_CHOLESTEROL_HOMEOSTASIS					
HALLMARK_MT	HALLMARK_CHOLESTEROL_HOMEOSTASIS					
HALLMARK_MT	HALLMARK_CHOLESTEROL_HOMEOSTASIS					
HALLMARK_MT	HALLMARK_CHOLESTEROL_HOMEOSTASIS					
HALLMARK_MTORC1_SIGNALIN		HALLMARK_EPI	HALLMARK_GLYCOLYSIS			
HALLMARK_MTORC1_SIGNALING			HALLMARK_GLYCOLYSIS			
HALLMARK_MTORC1_SIGNALING				HALLMARK_P53_PATHWAY		
HALLMARK_MTORC1_SIGNALING						
HALLMARK_MTORC1_SIGNALING						
HALLMARK_MTORC1_SIGNALING						
HALLMARK_MTORC1_SIGNALING						
HALLMARK_MTORC1_SIGNALING						
HALLMARK_MTORC1_SIGNALING						
HALLMARK_MTORC1_SIGNALING						
HALLMARK_MTORC1_SIGNALING						

HALLMARK_MTORC1_SIGNALING						
HALLMARK_MTORC1_SIGNALING						
HALLMARK_MTORC1_SIGNALING						
HALLMARK_MTORC1_SIGNALING						
HALLMARK_MTORC1_SIGNALING						
HALLMARK_MTORC1_SIGNALING						
HALLMARK_MTORC1_SIGNALING						
HALLMARK_MTORC1_SIGNALING						
HALLMARK_MTORC1_SIGNALING						
HALLMARK_MTORC1_SIGNALING						
mber 12A	HALLMARK_CH	HALLMARK_EPITHELIAL_MESENCHYMAL_TRANSITION				HALLMARK_AP
	HALLMARK_CHOLESTEROL_HO		HALLMARK_GLYCOLYSIS			
	HALLMARK_CHOLESTEROL_HOMEOSTASIS				HALLMARK_MYOGENESIS	
	HALLMARK_CHOLESTEROL_HOMEOSTASIS					HALLMARK_AP
	HALLMARK_CHOLESTEROL_HOMEOSTASIS					HALLMARK_AP
rt basic domain	HALLMARK_CHOLESTEROL_HOMEOSTASIS					
	HALLMARK_CHOLESTEROL_HOMEOSTASIS					
	HALLMARK_CHOLESTEROL_HOMEOSTASIS					
	HALLMARK_CHOLESTEROL_HOMEOSTASIS					
	HALLMARK_CHOLESTEROL_HOMEOSTASIS					
	HALLMARK_CHOLESTEROL_HOMEOSTASIS					
	HALLMARK_CHOLESTEROL_HOMEOSTASIS					
	HALLMARK_CHOLESTEROL_HOMEOSTASIS					
rotein 2	HALLMARK_CHOLESTEROL_HOMEOSTASIS					
rol cyclase)	HALLMARK_CHOLESTEROL_HOMEOSTASIS					
	HALLMARK_CHOLESTEROL_HOMEOSTASIS					
r 2	HALLMARK_CHOLESTEROL_HOMEOSTASIS					
	HALLMARK_CHOLESTEROL_HOMEOSTASIS					
factor 2	HALLMARK_CHOLESTEROL_HOMEOSTASIS					
		HALLMARK_EPI	HALLMARK_GLYCOLYSIS			
		HALLMARK_EPITHELIAL_MESEN		HALLMARK_P5	HALLMARK_MYOGENESIS	
		HALLMARK_EPITHELIAL_MESENCHYMAL_TRAN			HALLMARK_MYOGENESIS	
		HALLMARK_EPITHELIAL_MESENCHYMAL_TRAN			HALLMARK_MYOGENESIS	
		HALLMARK_EPITHELIAL_MESENCHYMAL_TRAN			HALLMARK_MYOGENESIS	
		HALLMARK_EPITHELIAL_MESENCHYMAL_TRANSITION				HALLMARK_AP
		HALLMARK_EPITHELIAL_MESENCHYMAL_TRANSITION				
		HALLMARK_EPITHELIAL_MESENCHYMAL_TRANSITION				
		HALLMARK_EPITHELIAL_MESENCHYMAL_TRANSITION				
		HALLMARK_EPITHELIAL_MESENCHYMAL_TRANSITION				
		HALLMARK_EPITHELIAL_MESENCHYMAL_TRANSITION				
		HALLMARK_EPITHELIAL_MESENCHYMAL_TRANSITION				
lypeptide)		HALLMARK_EPITHELIAL_MESENCHYMAL_TRANSITION				
		HALLMARK_EPITHELIAL_MESENCHYMAL_TRANSITION				
		HALLMARK_EPITHELIAL_MESENCHYMAL_TRANSITION				
		HALLMARK_EPITHELIAL_MESENCHYMAL_TRANSITION				
		HALLMARK_EPITHELIAL_MESENCHYMAL_TRANSITION				
Da		HALLMARK_EPITHELIAL_MESENCHYMAL_TRANSITION				
		HALLMARK_EPITHELIAL_MESENCHYMAL_TRANSITION				
		HALLMARK_EPITHELIAL_MESENCHYMAL_TRANSITION				
		HALLMARK_EPITHELIAL_MESENCHYMAL_TRANSITION				
		HALLMARK_EPITHELIAL_MESENCHYMAL_TRANSITION				
		HALLMARK_EPITHELIAL_MESENCHYMAL_TRANSITION				

	HALLMARK_EPITHELIAL_MESENCHYMAL_TRANSITION				
	HALLMARK_EPITHELIAL_MESENCHYMAL_TRANSITION				
	HALLMARK_EPITHELIAL_MESENCHYMAL_TRANSITION				
	HALLMARK_EPITHELIAL_MESENCHYMAL_TRANSITION				
	HALLMARK_EPITHELIAL_MESENCHYMAL_TRANSITION				
rotein 2	HALLMARK_EPITHELIAL_MESENCHYMAL_TRANSITION				
	HALLMARK_EPITHELIAL_MESENCHYMAL_TRANSITION				
	HALLMARK_EPITHELIAL_MESENCHYMAL_TRANSITION				
inogen activator inhibitor type	HALLMARK_EPITHELIAL_MESENCHYMAL_TRANSITION				
	HALLMARK_EPITHELIAL_MESENCHYMAL_TRANSITION				
	HALLMARK_EPITHELIAL_MESENCHYMAL_TRANSITION				
	HALLMARK_EPITHELIAL_MESENCHYMAL_TRANSITION				
	HALLMARK_EPITHELIAL_MESENCHYMAL_TRANSITION				
r, epithelial-specific)		HALLMARK_GLYCOLYSIS			
		HALLMARK_GLYCOLYSIS			
M)		HALLMARK_GLYCOLYSIS			
		HALLMARK_GLYCOLYSIS			
		HALLMARK_GLYCOLYSIS			
		HALLMARK_GLYCOLYSIS			
		HALLMARK_GLYCOLYSIS			
		HALLMARK_GLYCOLYSIS			
		HALLMARK_GLYCOLYSIS			
ARD domain)		HALLMARK_GLYCOLYSIS			
		HALLMARK_GLYCOLYSIS			
		HALLMARK_GLYCOLYSIS			
xylic acid transporter 4)		HALLMARK_GLYCOLYSIS			
			HALLMARK_P5	HALLMARK_MY	HALLMARK_AP
			HALLMARK_P5	HALLMARK_MYOGENESIS	
			HALLMARK_P5	HALLMARK_MYOGENESIS	
			HALLMARK_P53_PATHWAY		HALLMARK_AP
se)			HALLMARK_P53_PATHWAY		HALLMARK_AP
			HALLMARK_P53_PATHWAY		HALLMARK_AP
			HALLMARK_P53_PATHWAY		
			HALLMARK_P53_PATHWAY		
			HALLMARK_P53_PATHWAY		
			HALLMARK_P53_PATHWAY		
			HALLMARK_P53_PATHWAY		
its CDK4)			HALLMARK_P53_PATHWAY		
			HALLMARK_P53_PATHWAY		
			HALLMARK_P53_PATHWAY		
			HALLMARK_P53_PATHWAY		
			HALLMARK_P53_PATHWAY		
			HALLMARK_P53_PATHWAY		
			HALLMARK_P53_PATHWAY		
			HALLMARK_P53_PATHWAY		
			HALLMARK_P53_PATHWAY		
-type motif 15			HALLMARK_P53_PATHWAY		
			HALLMARK_P53_PATHWAY		
e 14			HALLMARK_P53_PATHWAY		
			HALLMARK_P53_PATHWAY		
			HALLMARK_P53_PATHWAY		
			HALLMARK_P53_PATHWAY		

	HALLMARK_MYOGENESIS	
	HALLMARK_MYOGENESIS	
	HALLMARK_MYOGENESIS	
1	HALLMARK_MYOGENESIS	
	HALLMARK_MYOGENESIS	
	HALLMARK_MYOGENESIS	
	HALLMARK_MYOGENESIS	
	HALLMARK_MYOGENESIS	
	HALLMARK_MYOGENESIS	
	HALLMARK_MYOGENESIS	
	HALLMARK_MYOGENESIS	
	HALLMARK_MYOGENESIS	
r 3	HALLMARK_MYOGENESIS	
	HALLMARK_MYOGENESIS	
	HALLMARK_MYOGENESIS	
	HALLMARK_MYOGENESIS	
nammary-derived growth inhibitor)	HALLMARK_MYOGENESIS	
	HALLMARK_MYOGENESIS	
	HALLMARK_MYOGENESIS	
	HALLMARK_MYOGENESIS	
	HALLMARK_MYOGENESIS	
	HALLMARK_MYOGENESIS	
	HALLMARK_MYOGENESIS	
		HALLMARK_AP
		HALLMARK_AP
		HALLMARK_AP
		HALLMARK_AP
eta		HALLMARK_AP
		HALLMARK_AP
		HALLMARK_AP
		HALLMARK_AP
		HALLMARK_AP
pe 4		HALLMARK_AP
r 2		
eptide 1		
family member		

affinity glutamate transporter, system Xag), member 1

e 21
ciated coagulation inhibitor)

) 9

alpha 1A subunit

s), member 2

ber 8

ene homolog

el, 7

nber 15

2)

ptide 11

e 3
neric
n 1)

type 1 motif, 1

kinase 1

og (mouse)
ene homolog B (avian)

ain containing 1

visiae)
esis 1

precursor)

ase 1 homolog D (S. cerevisiae)
rotein

ember 2
4

e) 8

type 1 motif, 5

it of VLA-3 receptor)

ntaining 2A

pha polypeptide

ubunit 1

proteoglycan (testican) 2

member 5

nber 5
ly down-regulated 4

g 1
coli)
inase 3

ltransferase 4

ɔha 1B subunit
eptide 1

nsporters), member 4

(parkin)

nember C

ɔha 1F subunit

subunit

ɔits CDK4)

3

ıolog (S. cerevisiae)

e G (32kD)-like

1 complex, alpha subunit 1 pseudogene 9

(Drosophila)

ı C)

rase)

:e 1

ember 1

porter), member 13

mily, member 4
de N-acetylgalactosaminyltransferase 13 (GalNAc-T13)
otor 2

taining 12

mber 13C

nd PH domain 2

ptide 1

me 1

e SgK494

eptor

eag-related), member 8

ane protein 2

og (S. cerevisiae)

rter), member 1

porter), member 14

taining 11

(Xenopus laevis)

type 8B, member 3

4

ɔn antiporter 2), member 1

ɔorter), member 12
ptide 1

ıdogene 2

nositol anchor 2
: 6-like

pe 5
er 2

erase)

. cerevisiae)

phila)-like 3 (Hu antigen C)

cerevisiae)

oxylic acid transporter 13)

tor 3

omain)
tin protein ligase

ein

Follistatin-like domains 2

e 2

ubunit C2

lpha 1
lpha 2

amma 1

molog (Xenopus laevis)

acetylglucosaminyltransferase, isozyme C (putative)

de N-acetylgalactosaminyltransferase 2 (GalNAc-T2)
nouse)

-type motif 13

G (with RhoGef domain) member 3

gulated
midinotransferase)
ge factor

r), member 5

channel, subfamily M, beta member 4
ic polypeptide-like 3C
te 5
releasing hormone)

-protein coding)

-type motif 7

member A

siae)

seudogene
mily, member 6
mber 10d, decoy with truncated death domain pseudogene
ptor type B-like)

artate 2A

e receptor-like 2

binding protein
udogene

:blB type

ltransferase 6

ıelix-loop-helix protein

member 4C1

de N-acetylgalactosaminyltransferase-like 4

nember 1

ly J, member 6

fier, subfamily S, member 1

or 2

37

15
e 3

coding)

seudogene

9

seudogene

ı-protein coding)

calcineurin-dependent 4

ysin 1

: 1

chordin-like)

(Xenopus laevis)

ıtalytic subunit

ing 1

ɔhatase 4
;, class A
;, class H

ɔrming protein 3
ɔl-specific)
ed)

ıe 3

ember 4

ning 1

:e 1

) 1

a isozyme

or family, member 2

, class V

bitor beta

elta subunit 3

oid microdomains 1

mber 1

porter light chain, asc system), member 10

ontaining 1B

complex, 4-like 2
mine 3-beta-galactosyltransferase, 1

glandin G/H synthase and cyclooxygenase)

H (with MyTH4 domain) member 1

ain containing 2

aining 9

taining 16

ubunit 2

mber 14

nsmembrane domain (TM) and short cytoplasmic domain, (semaphorin) 4G

g factor 1

binding protein
protein 2

ranscription factor 2

2 (Drosophila)

de N-acetylgalactosaminyltransferase 11 (GalNAc-T11)
rt basic domain, secreted, (semaphorin) 3F

nsmembrane domain (TM) and short cytoplasmic domain, (semaphorin) 4A

rotein 1

tein B

ıber 3
ger), member 2
ger), member 1
ger), member 3
nger), member 5

dogene
e D, 44kDa

ide 1, 43kDa
ide 3, 50kDa
ein A1, 59kDa, acidic component)

onal enhancer factor)

olog, Drosophila)
(endothelial)

ember 3
ıolog 1

pha 1E subunit
n 8, apolipoprotein e receptor
bunit

bunit

minyltransferase

olog, Drosophila)

-type motif 18

mber 11, pseudogene
ntraxin domain containing 1
ntaining 1

type 1 motif, 20

nonas)

ain containing 5

l delta

protein 2

1-like

ndependent)

racting RhoGEF

protein 1

ctor 1

lpha

transferase) 2

. elegans)

iolamine-specific)

ig protein 3

mber 10d, decoy with truncated death domain
mber 10b
mber 10a

pha 1I subunit
y)

family A, member 1
nase)

14

ubunit E2
factor-associated, 170kDa (Mot1 homolog, S. cerevisiae)
nember A

aining 13

e 1
ein, alpha

-like 1

d subfamily, beta member 3

rotein 1-like

1

tor 3
(Xenopus laevis)

ansporter, member 7

se 1
se 2

n and LIM domain containing 2

domains 1
onent 1

rase 2

HALLMARK_ESTROGEN_RESPONSE_EARLY

OPTOSIS

HALLMARK_ESTROGEN_RESPONSE_EARLY

OPTOSIS

OPTOSIS

OPTOSIS
HALLMARK_ESTROGEN_RESPONSE_EARLY

HALLMARK_ESTROGEN_RESPONSE_EARLY

OPTOSIS
HALLMARK_ESTROGEN_RESPONSE_EARLY

OPTOSIS

HALLMARK_ESTROGEN_RESPONSE_EARLY

OPTOSIS

OPTOSIS
OPTOSIS
HALLMARK_ESTROGEN_RESPONSE_EARLY

ɔPTOSIS

HALLMARK_ESTROGEN_RESPONSE_EARLY
ɔPTOSIS
ɔPTOSIS
ɔPTOSIS

ɔPTOSIS
ɔPTOSIS
HALLMARK_ESTROGEN_RESPONSE_EARLY

HALLMARK_ESTROGEN_RESPONSE_EARLY
ɔPTOSIS
HALLMARK_ESTROGEN_RESPONSE_EARLY
HALLMARK_ESTROGEN_RESPONSE_EARLY
HALLMARK_ESTROGEN_RESPONSE_EARLY

OPTOSIS
OPTOSIS
HALLMARK_ESTROGEN_RESPONSE_EARLY

HALLMARK_ESTROGEN_RESPONSE_EARLY
HALLMARK_ESTROGEN_RESPONSE_EARLY

OPTOSIS

OPTOSIS
OPTOSIS
HALLMARK_ESTROGEN_RESPONSE_EARLY
HALLMARK_ESTROGEN_RESPONSE_EARLY
HALLMARK_ESTROGEN_RESPONSE_EARLY

OPTOSIS

HALLMARK_ESTROGEN_RESPONSE_EARLY

OPTOSIS

OPTOSIS
OPTOSIS
OPTOSIS
HALLMARK_ESTROGEN_RESPONSE_EARLY

HALLMARK_ESTROGEN_RESPONSE_EARLY

OPTOSIS
OPTOSIS
OPTOSIS
OPTOSIS
OPTOSIS
OPTOSIS
OPTOSIS
OPTOSIS
OPTOSIS
OPTOSIS
HALLMARK_ESTROGEN_RESPONSE_EARLY
HALLMARK_ESTROGEN_RESPONSE_EARLY
HALLMARK_ESTROGEN_RESPONSE_EARLY
HALLMARK_ESTROGEN_RESPONSE_EARLY
HALLMARK_ESTROGEN_RESPONSE_EARLY
HALLMARK_ESTROGEN_RESPONSE_EARLY
HALLMARK_ESTROGEN_RESPONSE_EARLY
HALLMARK_ESTROGEN_RESPONSE_EARLY
HALLMARK_ESTROGEN_RESPONSE_EARLY
HALLMARK_ESTROGEN_RESPONSE_EARLY
HALLMARK_ESTROGEN_RESPONSE_EARLY
HALLMARK_ESTROGEN_RESPONSE_EARLY
HALLMARK_ESTROGEN_RESPONSE_EARLY
HALLMARK_ESTROGEN_RESPONSE_EARLY
HALLMARK_ESTROGEN_RESPONSE_EARLY
HALLMARK_ESTROGEN_RESPONSE_EARLY
HALLMARK_ESTROGEN_RESPONSE_EARLY
HALLMARK_ESTROGEN_RESPONSE_EARLY
HALLMARK_ESTROGEN_RESPONSE_EARLY

HALLMARK_ESTROGEN_RESPONSE_EARLY
HALLMARK_ESTROGEN_RESPONSE_EARLY
